# Comparative Transcriptomics and Proteomics of Cancer Cell Lines Cultivated by Physiological and Commercial Media

**DOI:** 10.3390/biom12111575

**Published:** 2022-10-27

**Authors:** Junyao Wang, Wenjing Peng, Aiying Yu, Mohamed Fokar, Yehia Mechref

**Affiliations:** 1 Department of Chemistry and Biochemistry, Texas Tech University, Lubbock, TX 79409, USA; 2 Center of Biotechnology and Genomics, Texas Tech University, Lubbock, TX 79409, USA

**Keywords:** proteomics, transcriptomics, culture media, cancer cell line, differential expression analysis, LC-MS/MS

## Abstract

Aiming to reduce the gap between in vitro and in vivo environment, a complex culture medium, Plasmax, was introduced recently, which includes nutrients and metabolites with concentrations normally found in human plasma. Herein, to study the influence of this medium on cellular behaviors, we utilized Plasmax to cultivate two cancer cell lines, including one breast cancer cell line, MDA-MB-231BR, and one brain cancer cell line, CRL-1620. Cancer cells were harvested and prepared for transcriptomics and proteomics analyses to assess the discrepancies caused by the different nutritional environments of Plasmax and two commercial media: DMEM, and EMEM. Total RNAs of cells were extracted using mammalian total RNA extract kits and analyzed by next-generation RNA sequencing; proteomics analyses were performed using LC-MS/MS. Gene oncology and pathway analysis were employed to study the affected functions. The cellular invasion and cell death were inhibited in MDA-MB-231BR cell line when cultured in Plasmax compared to DMEM and EMEM, whereas the invasion, migration and protein synthesis of CRL-1620 cell line were activated in Plasmax in relative to both commercial media. The expression changes of some proteins were more significant compared to their corresponding transcripts, indicating that Plasmax has more influence upon regulatory processes of proteins after translation. This work provides complementary information to the original study of Plasmax, aiming to facilitate the selection of appropriate media for in vitro cancer cell studies.

## 1. Introduction

Due to the different nutrient compositions and concentrations, the culturing environment of media can greatly affect the cell metabolism [1,2,3], and cellular behaviors, such as cell growth and proliferation [4,5,6]. For most of the commercially available cell culturing media, the nutrient compositions are not as close as the in vivo environment for cells because they were designed to provide a minimal amount of nutrients for continuous cultivation of cells, instead of reproducing a physiological cellular environment like human plasma. For example, Eagle’s Minimal Essential Medium (EMEM) is a synthetic cell culture medium developed by Harry Eagle, which includes 6 salts, 8 vitamins, and essential amino acids [7]. Dulbecco’s Modified Eagle’s Medium (DMEM) was introduced with higher concentrations of amino acids and vitamins based on EMEM to avoid nutrient exhaustion during the cultivation process without attendance for a longer period of time [8]. Both of EMEM and DMEM are two common media that have been widely used in cell culturing [9,10,11]. However, with high concentrations of selected nutrients and the absence of important minor elements such as selenium, it might lead to metabolic alterations inside the cells for in vitro studies compared to the in vivo studies [12].

Recently, more attentions have been drawn to the optimization of the nutrient compositions and concentrations of culturing media for cancer cell line cultivation [3,13,14,15]. A complex physiological culture medium called Plasmax was introduced by Tardito and co-workers [16] to mimic human plasma. More than 60 types of nutrients and metabolites were included in Plasmax, while 35% of the Plasmax compositions are not included in commercial media, such as DMEM or EMEM. Therefore, Plasmax can provide more nutritional options during cell line cultivation, and better mimic the in vivo environment for cells. Compared to DMEM and EMEM, the concentrations of nutrients in Plasmax are different. For example, both EMEM and DMEM have high abundance of D-Glucose and L-glutamine, whereas Plasmax has relatively lower abundance for those nutrients. To minimize the gap between in vitro and in vivo environments and reproduce the in vivo environment for cancer cell line cultivation, the concentration of each nutrient in Plasmax was maintained based on the physiological concentration in human blood. According to the reported study [16], compared to the cells cultivated in DMEM-F12, Plasmax can help enhance the colony-forming capacity, and the micronutrient selenium in Plasmax played an important role in preventing ferroptosis of breast cancer cells. In addition, the transcriptional and metabolic phenotypes of cancer cells cultivated by Plasmax were found independent of the proliferation rate, which suggests that the high concentrations of some nutrients in commercial media is not required for cell growth, and it misdirects cell line metabolism. Our previous study also suggests that Plasmax can lead to alterations in glycosylation [17]. However, a comprehensive investigation of gene and protein expression changes in Plasmax medium is necessary to guide the medium selection when performing in vitro cell line experiments.

In this study, we made comparisons between Plasmax and two commercially available media, DMEM and EMEM by investigating the expression changes of transcriptomes and proteomes of cancer cell lines to obtain more comprehensive understandings about how this novel medium can affect the cellular functions. A breast cancer cell line (MDA-MB-231BR), and a brain cancer cell line (CRL-1620) were subject to cultivation in the abovementioned three different culturing media and cell harvest. Total RNAs and proteins were extracted and prepared to evaluate the gene and protein expressions, respectively. Unsupervised principal component analysis (PCA) was applied to assess the expression differences within two cancer cell lines cultured by three media. Gene ontology analysis and protein function annotation were performed utilizing Ingenuity pathway analysis software (QIAGEN IPA) to study the altered cellular functions. Significant up- or down-regulations of proteins that are associated with cell movement, cell death and survival, and protein synthesis were observed.

## 2. Materials and Methods

### 2.1. Materials and Reagents

Breast cancer cell line MDA-MB-231BR (231BR) was generously provided by Dr. Paul Lockman (West Virginia University, School of Pharmacy, Morgantown, WV, USA). Brain cancer cell line CRL-1620 (CRL), DMEM, EMEM, and Fetal Bovine Serum (FBS) were acquired from American Type Culture Collection (ATCC, Manassas, VA, USA). Corning Trypsin EDTA 1× (25% Trypsin/2.21 mM EDTA), MS-grade formic acid (FA), HPLC grade water, methanol, and acetonitrile (ACN) were obtained from Fisher Scientific (Fair Lawn, New Jersey, NJ, USA). Mammalian total RNA extraction kit, sodium hydroxide beads, Dithiothreitol (DTT), iodoacetamide (IAA), ammonium bicarbonate (ABC) and sodium deoxycholate (SDC) were purchased from Sigma-Aldrich (St. Louis, MO, USA). NEBNext rRNA depletion fit (human/mouse/rat), and NEBNext Ultra II Directional RNA library prep kit were obtained from New England Biolabs (Ipswich, MA, USA). Trypsin/Lys-C mix, mass-spectrometry-grade, was purchased from Promega (Madison, WI, USA).

### 2.2. Preparation of Plasmax Medium

Plasmax medium was prepared based on the formulation (Supporting Information Table S1) and following the preparation steps reported by Voorde et al. [16]. Overall, eight stock solutions were prepared first. For stock solution #1, each nutrient was weighed and dissolved in 10mL HPLC grade water. The pH value of stock solution #1 was adjusted to 1.00 by adding saturated HCl solution. According to the same procedure, stock solution #2 was prepared, however no pH adjustment was required. For solution #3, since the amount of some of the nutrients were too small to be weighed accurately, the nutrients were separated into two groups. The first group was prepared using the same method as aforementioned for solution #1 and #2, while the second group had the amount of the compositions multiplied by 10,000 times then dissolved in 1000 mL of HPLC grade water. Then, 9 mL from group 1 and 1mL from group 2 were mixed to produce stock solution #3. Stock solution #4 only contains one metabolite, urate. Since urate can only be dissolved under highly basic conditions so the pH value was adjusted to 13.3 using concentrated NaOH solution followed by sonication. Stock solution #5 contains BME vitamins and L-Glutamine, both of which are commercially available and in liquid phase, so a certain volume of each of them was added directly into the final Plasmax medium based on the formulation. Solution #6 and solution #8 were prepared using the same method as solution #1 and #2. Solution #7 was Earle’s Balanced Salt Solution (EBSS), which served as the base solution of Plasmax medium.

To keep the stock solutions from being contaminated, the mixing process was performed in a strictly aseptic environment. A bottle of 500 mL Plasmax medium was prepared according to the following steps: First, 5mL of stock solution #1 and #2 were added into 482 mL of EBSS, followed by 0.5 mL of solutions #3 and #8. Then, 1mL of solutions #4 and 0.05 mL of #6 were added. Next, 5 mL of BME-Vitamin, 1.625 mL of L-Glutamine and 12.5 mL of fetal bovine serum (FBS) were also added. After adding all stock solutions and other required compositions, the mixture was shaken for 10 min to help the medium mix evenly. Then, the mixture was pushed through a Nalgene rapid-flow filter (Thermo Scientific, Rochester, NY, USA) with a sterilizing-grade 0.2 μm SFCA membrane installed to remove any bacteria that potentially involved in the medium solution. Finally, the well-prepared 500 mL Plasmax medium was stored in 4 °C for future use.

### 2.3. Cell Culturing and Harvesting

The cancer cell lines were cultured and harvested according to the previously reported procedures [18,19]. Two cancer cell lines (231BR and CRL) were cultivated in three media (Plasmax, DMEM, and EMEM). Cells stored in liquid nitrogen were firstly thawed at 37 °C and inoculated in 75 cm^2^ flasks. Cell culture was incubated at 37 °C for 4–8 days and fed every 2–4 days. When the cell confluence reached 90%, cells were washed with a 10mL aliquot of PBS twice and detached by 2.2 mL trypsin-EDTA solution. After incubating at 37 °C for 5 min, 5 mL of fresh medium was added to the cell solution to quench trypsin. The cell suspension solution was then moved into three 175 cm^2^ flasks for biological triplicate and incubated at 37 °C for about 7 days, until it reached 80% of cell confluence. Cells were washed and detached from the flasks using the abovementioned method. After adding 10 mL of fresh medium, cells were harvested by centrifuging at 500× *g* for 5 min. Cell pellets were collected and washed twice with PBS to get rid of the extra medium. Part of the fresh cells were taken for RNA extraction. The rest of cell samples were stored in −20 °C for future proteomics analyses.

### 2.4. Transcriptomics Analysis

Cancer cells were first washed with PBS solution and total RNAs were extracted from the cells using a Mammalian Total RNA Extraction Kit following the procedure provided by the vendor. For each cell line, three biological replicates were prepared. After the extraction, a 5-μL aliquot of total RNA solution was taken to determine the concentration using a Nanodrop 1000 spectrophotometer (Thermo Scientific, Wilmington, DE, USA). Then, RNA quality was determined using RNA Screen Tape (Agilent technologies, Santa Clara, CA, USA). Ribosomal RNA depletion was achieved using a NEB Next rRNA Depletion Kit (Human/Mouse/Rat). RNA fragmentation, double stranded cDNA, and adaptor ligation was generated using NEBNext Ultra II Directional RNA Library Prep following the manufacturer’s procedures. PCR enriched libraries were quantified by Qubit and equimolar indexed libraries were pooled. Pooled libraries were quantitatively checked using software Tapestation 2200 (Agilent Technologies, Santa Clara, CA, USA) and quantified using Qubit. The libraries were then diluted to 200 pM and spiked with 2% phiX libraries (Illumina control). The transcriptome sequencing was performed on the barcoded stranded RNA-Seq libraries using Illumina NovaSeq 6000 S1 flow cell (Illumina, San Diego, CA, USA), paired-end reads (2 × 100 bp).

### 2.5. Proteomics Analysis

The protein extraction was accomplished by following previously reported procedures [20,21,22]. The cell pellets were first thawed at room temperature. Then, a 100-μL aliquot of ammonium bicarbonate (ABC) buffer and 100 μL of 5% sodium deoxycholate (SDC) were added to resuspend the cells. The cell solution was transferred into sample vials with zirconium beads (400 μm) for beads beating. Each sample was shaken by a BeadBug Microtube Homogenizer (Benchmark Scientific, Edison, NJ, USA) at 4 °C for 5 rounds, with 30 s during each round and 30 s intervals to prevent overheat. Then, the samples were sonicated in iced water for 1 h and centrifuged at 1000× *g* for 10 min. The supernatant, which contained the extracted cell proteins, was collected. Extracted protein from 231BR and CRL cell lines were then treated by tryptic digestion [23,24,25]. Briefly, the proteins were first denatured at 90 °C for 15 min, then 2 μL from each sample was taken for protein assay using a micro BCA protein assay kit (Thermo Scientific, Rockford, IL, USA), following the manufacturer’s instruction. The protein concentration was determined by a Multiskan plate reader (Thermo Scientific, Rockford, IL, USA).

Based on the protein concentration, the remaining samples were first diluted to make a 0.5% concentration of SDC, then reduced by adding 200 mM dithiothreitol (DTT) and incubated at 60 °C for 45 min. Then, the reduced proteins were alkylated by adding 200 mM iodoacetamide (IAA) followed by incubation at 37.5 °C for 45 min in dark. The alkylation reaction was quenched by adding 200 mM DTT again and incubated at 37.5 °C for 30 min. The pH value of each sample was checked before the addition of enzyme to make sure the high efficiency of enzymatic digestion. The protease trypsin/Lys C [26,27], was added into the sample solution with an enzyme/protein ratio of 1:25 and the samples were incubated at 37.5 °C for 18 h. After trypsin digestion, FA was added (final concentration equals 0.5%) to stop the digestion process and precipitate SDC detergent. The sample was then vortexed and centrifuged at 14,800 rpm for 10 min. After centrifuging, the supernatant, which contained digested peptides, was collected and analyzed using LC-MS/MS.

The raw data files generated by LC-MS/MS were processed using software MaxQuant v2.0.2.0 (MPIB, Munich, Germany) [28]. Proteins were identified by searching against the UniprotKB/Swiss-Prot human database. The group-specific parameters in MaxQuant were set as follows: Trypsin was selected as the digestion enzyme with 2 miscleavages allowed. The shortest identified peptides contain 7 amino acids. Acetylation of protein N-terminal and oxidation of methionine were selected as variable modifications, while cysteine carbamidomethylation was set as fixed modification. Protein quantification was achieved using the label-free quantification approach (LFQ). The match tolerance of peptide precursor and fragment ions was 20 ppm and 0.5 Da, respectively. The false discovery rate (FDR) was set at 0.01. After the raw data processing, the data files generated by MaxQuant were subjected to software Perseus v1.6.15.0 (MPIB, Munich, Germany) for quantitative analysis. Potential contaminants, reversed identifications, and only identified by site were excluded for further analysis. The MaxQuant software reported normalized LFQ intensities of identified proteins, which was based on algorithms created by Cox, J. et al. [29]. Unsupervised principal component analysis (PCA) was employed to assess the differences of protein expressions between Plasmax and commercial media using software MarkerView v1.3 (Sciex, Framingham, MA, USA). Then, Student’s *t* test was applied to search for significant changes of protein abundances (*p* < 0.05), followed by Benjamini-Hochberg (BH) procedure for the multiple testing correction. Data sets of significant proteins from both cell lines were uploaded to Ingenuity Pathway Analysis (IPA, QIAGEN, Redwood City, CA, USA) for functional annotation and pathway analysis to investigate the cellular function discrepancies correlated with the altered protein expressions.

### 2.6. LC-MS/MS Conditions

Proteomics analyses were performed using a Dionex UltiMate 3000 nano LC system (Dionex, Sunnyvale, CA, USA) coupled with an LTQ Orbitrap Velos mass spectrometer (Thermo Scientific, San Jose, CA, USA) through a nano-ESI source. A C18 Acclaim PepMap 100 trapping column (75 μm I.D. × 2 cm, 3 μm particle sizes, 100 Å pore sizes, Thermo Scientific, San Jose, CA, USA) was used to remove salt and impurities. Peptide was separated by a C18 Acclaim PepMap RSLC column (75 μm I.D. × 15cm, 2 μm particle sizes, 100 Å pore sizes, Thermo Scientific, San Jose, CA, USA). The column compartment temperature was set at 29.5 °C. Mobile phase A contained 2% of ACN and 0.1% of FA, while mobile phase B had 0.1% FA in ACN. A 120 min elution method was utilized for the separation of peptides. The gradient of mobile phase B started at 5% over the first 10 min, then increased to 20% in 55 min, 20% to 30% in 25 min, 30% to 50% in 20min, then ramped up to 80% in 1 min and stayed at 80% for 4 min, then dropped back to 5% in 1 min and stayed for 4 min. Two MS scan events were applied for data acquisition, including a full MS scan with the m/z range of 400–2000 at a resolution of 60,000, and a CID MS/MS scan using normalized collision energy (CE) of 35% and a 10 ms activation time. A data dependent acquisition (DDA) method was employed, which selected the top eight most intense ions from the full scan for the MS/MS analysis.

## 3. Results and Discussion

### 3.1. Culturing Media Specification

The concentrations of the nutrients and metabolites of the Plasmax media was based on the freely available resource (www.serummetabolome.ca, accessed on 11 January 2019) [30]. Supporting Information Figure S1 gives direct comparisons of the major nutrient compositions of Plasmax and the other two commercial media. The glucose and glutamine take approximately 60% in EMEM and more than 75% in DMEM, however, these two nutrients are only 43% in Plasmax. Moreover, 35% of the nutrients and metabolites found in Plasmax are absent in both commercial media, including urea, lactate and urate. In addition, some trace elements such as zinc and manganese are only present in Plasmax. The full formulation of Plasmax is provided by Supporting Information Table S1.

### 3.2. Expression Changes of Transcriptome in Cancer Cell Lines

**Figure 1:**
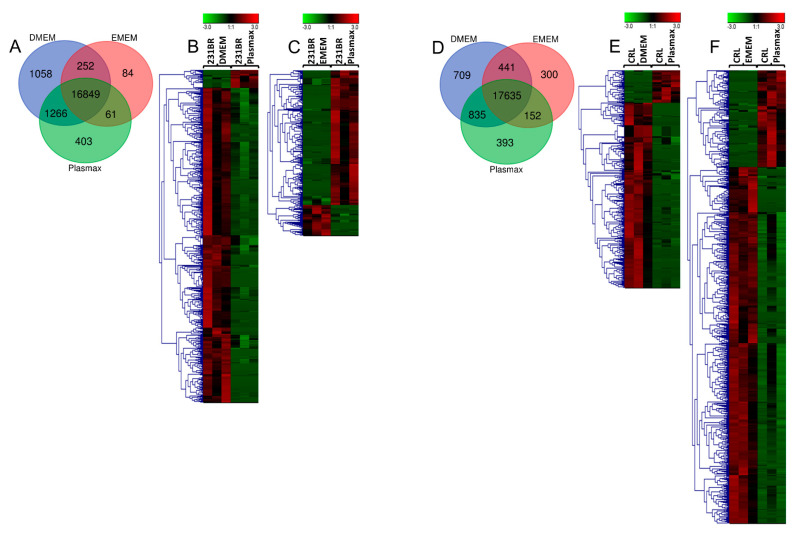
(**A**) Venn diagrams of transcriptome expressions in 231BR cell line cultivated by three media; (**B**) Heatmaps of significant transcriptomes from 231BR cell line cultivated in Plasmax and DMEM; (**C**) Significant transcriptomes from 231BR cell line cultivated in Plasmax vs. EMEM; (**D**) Venn diagrams of transcriptome expressions in CRL cell line cultivated by three media; (**E**) Significant transcriptomes from CRL cell line cultivated in Plasmax vs. DMEM; (**F**) Significant transcriptomes from CRL cell line cultivated in Plasmax vs. EMEM. The color codes denote expression level of genes. The green color denotes lower expression, while the red color denotes higher expression.

Next generation RNA sequencing was performed to investigate the alterations of gene expressions among the cells cultured in the different media. Overall, 19973 genes from 231BR, and 20465 from CRL were identified (Supporting Information Table S2). Based on the RPKM of genes, the *p*-values between Plasmax and commercial media were calculated using student’s *t* test. For 231BR cell lines cultivated in Plasmax, a total of 536 and 265 genes were found as statistically significant (*p* < 0.05) when compared to this cell line cultivated in DMEM and EMEM, respectively. On the other hand, the CRL cell lines cultivated by Plasmax included 349 and 712 significant genes in relative to DMEM and EMEM, respectively. The Venn diagrams in Figure 1 show the distribution of all identified genes in three media, while the heatmaps demonstrate the up- or down-regulation of significant genes.

### 3.3. Gene Ontology (GO) Analysis of Transcriptomes with Different Expressions

In gene ontology analysis, differently expressed transcriptomes were clustered according to their molecular and cellular functions [31,32]. Compared to the two commercial media, the top five significantly altered functions of both cell lines cultivated in Plasmax are provided by Figure S2. The cellular movement of 231BR cell line were affected by Plasmax compared to both DMEM and EMEM. In addition, cell-to-cell signaling and interaction, drug metabolism, lipid metabolism, and small molecule biochemistry of 231BR cell line were altered by Plasmax in relative to DMEM, while cell death and survival, cellular development, cellular function and maintenance, and cellular growth and proliferation were affected compared to EMEM. When it comes to the CRL cell line, cell morphology was found in both comparisons. The other significantly different functions of CRL cell lines between Plasmax and DMEM include cellular function and maintenance, cellular development, cellular growth and proliferation, and cell-to-cell signaling and interaction. In relative to EMEM, cell cycle, cellular assembly and organization, DNA replication, recombination, and repair, and cell death and survival are the mostly affected functions.

### 3.4. Pathway Analysis of Differently Expressed Transcriptomes

**Figure 2:**
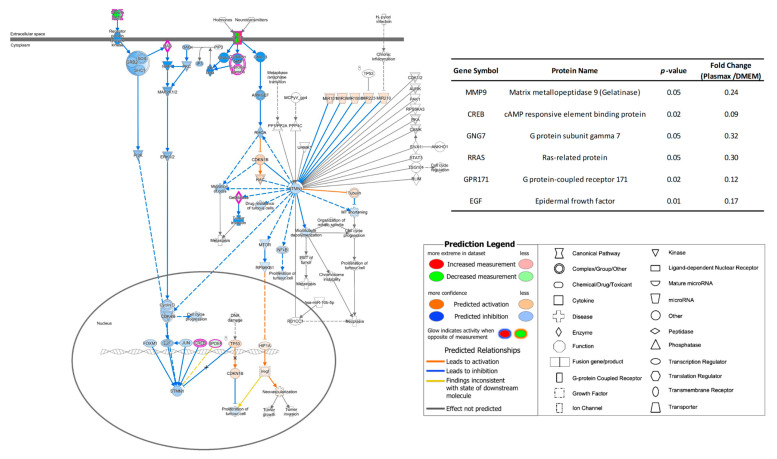
Inhibited pathway of breast cancer regulation by stathmin 1 of 231BR cell line cultivated by Plasmax compared to DMEM. Blue line denotes that the corresponding protein change cause an inhibition to the function in Plasmax. Orange line denotes that the corresponding protein change cause an activation to the function in Plasmax, and yellow line denotes the inconsistent changes of corresponding proteins and their downstream molecules. The inset table provides the *p*-values and fold-change numbers of transcriptomes that are involved in this pathway.

In order to gain more understandings about the impacts on cellular functions due to different transcriptome expressions in Plasmax, the pathway analysis was performed using QIAGEN IPA. The two commercial media were employed as baseline, and the transcriptomics results acquired from cell lines cultivated in Plasmax were compared with the counterparts in DMEM and EMEM, respectively. The logarithm of fold changes and *p*-values of statistically significant transcriptomes were uploaded to IPA. IPA will search against its database and predict the corresponding upstream regulators and the influence on downstream processes based on the given data. In breast cancer cell line 231BR, as shown by Figure 2, the pathway of breast cancer regulation by stathmin 1 was inhibited by Plasmax in relative to DMEM due to the down-regulation of correlated genes (see the inset table). Stathmin 1 (STMN1) is a cytosolic phosphoprotein [33], which has been reported as a regulator of the proliferation of breast cancer [34,35]. The predicted inhibition of this pathway will lead to reduced proliferation of 231BR cell line. In addition, among the significant genes within this pathway, gelatinase (MMP9) was down-regulated by 76% in Plasmax. MMP9 has been reported to enhance the invasiveness of tumor cells [36]. Thus, the down-regulation of may hamper the invasion of the 231BR cell line. On the contrary, this same pathway of stathmin 1 regulation was activated in Plasmax when compared to EMEM. In Figure S3, the gene of G1/S-specific cyclin-D2 (CCND2) exhibits significant up-regulation. It is a regulatory component of the cyclin D2-CDK4/6 complex, which can phosphorylate the retinoblastoma (RB) protein family and allow the dissociation of the transcription factor E2F from the RB/E2F complex [37,38]. Furthermore, the E2F regulates the expression of STMN1 [39,40]. Therefore, according to this pathway, the proliferation as well as the invasion of 231BR cell line was predicted to be more stimulated in Plasmax than in EMEM.

**Figure 3:**
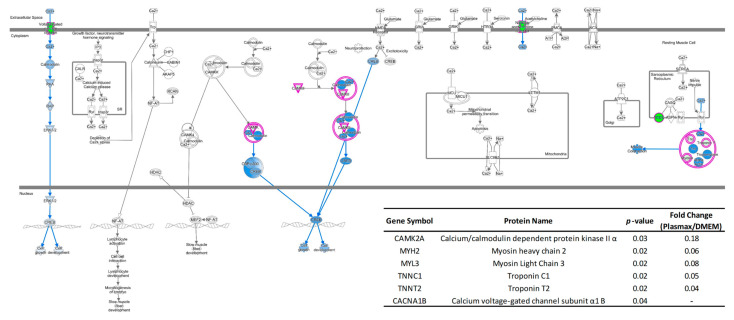
Inhibited calcium signaling pathway of CRL cell line cultivated by Plasmax compared to DMEM. Color codes and symbols are the same as Figure 2. The inset table provides the *p*-values and fold-change numbers of transcriptomes that are involved in this pathway.

For the brain cancer cell line CRL, the transcriptomics discrepancies between Plasmax and DMEM resulted in the inhibition of the calcium signaling pathway. As shown in Figure 3, Ca^2+^ and calmodulin can activate calcium/calmodulin dependent protein kinase (CAMK) and lead to phosphorylation [41], then activate the transcription factor of cyclic AMP-responsive element-binding protein (CREB) [42]. It has been reported that overexpression of CREB was found in different types of tumors [43], suggesting that CREB is involved in tumor growth or development. However, the down-regulated expression (-82%) of CAMK in Plasmax might hamper the abovementioned functions of CRL cell line compared to DMEM. The down-regulation of CAMK can also be explained by the lower abundance of voltage-gated calcium channel, which mediate the entry of calcium ions into cells [44]. On the other hand, in relative to EMEM, the mainly affected function is cell cycle. Figure S4 demonstrates the pathway of cyclins and cell cycle regulation. The correlated transcriptomes such as CCNA/B, CDK, and E2F were all down-regulated, leading to the inhibition of G1/2 phase, M phase, and S phase of cell cycle [45].

### 3.5. Comparative Proteomics Analysis of Cancer Cell Lines Cultivated in Three Media

Bottom-up proteomics analysis was performed using LC-MS/MS [24,46,47,48]. Protein identification and quantitation was accomplished using MaxQuant software. The proteins which were only identified in one of the three replicates of each group were excluded. The abundance of each protein is the sum of normalized intensities of all peptides of the corresponding protein. Overall, a total of 2296 and 2301 proteins were identified in 231BR and CRL cell line, respectively. Venn diagrams were generated for both cell lines to demonstrate the common and unique proteins in three media. As shown in Figure S5A, 1848 proteins were identified in 231BR cell lines cultivated by all three media, however, 35, 55, and 94 unique proteins were found in DMEM, EMEM, and Plasmax, respectively. For CRL cell lines (Figure S5D), 1992 proteins were observed in all three media, while 59, 45, and 40 proteins were only identified in DMEM, EMEM, and Plasmax, respectively. A list of all identified proteins is provided in Supporting Information Table S3. Student’s *t* test was then utilized to search for significant changes of protein abundances between Plasmax and commercial media (*p* < 0.05). In 231BR cell lines (Figure S5B,C), 179 proteins were observed with significant alterations between Plasmax and DMEM (71 up-regulations and 108 down-regulations), while 97 significant proteins were found between Plasmax and EMEM (37 up-regulations and 60 down-regulations). In CRL cell line, as shown by Figure S5E,F, 354 significant proteins (167 up-regulations and 187 down-regulations) were identified in Plasmax when compared to DMEM, and 192 significant proteins (107 up-regulations and 86 down-regulations) between Plasmax and EMEM.

### 3.6. Unsupervised Pricipal Component Analysis (PCA)

Unsupervised PCA was applied for overall comparison of cell line protein expressions among three media. As a mathematical method to simplify high dimensional data sets into lower dimensional sets, PCA transforms the possibly correlated variables into principal components. Different sets of observations are mapped to show the similarities and differences [49]. The normalized abundances of all proteins from 231BR and CRL cell lines were uploaded to MarkerView software (Sciex v1.3) and generated the PCA plots as shown in Figure S6. For both cell lines, the plots of triplicates are clustered closely, indicating a good reproducibility of sample preparation and proteomics analyses. Each circle of a different media can be clearly separated from the other two, showing that the protein expressions from the three media are distinct. Specifically, in Figure S6A, the three replicates of Plasmax are separated from DMEM by first principal component (PC1), while Plasmax and EMEM are separated by second principal component (PC2), suggesting that the protein expressions of 231BR cell line were more different between Plasmax and DMEM. For CRL cell line (Figure S6B), Plasmax and DMEM are also different in terms of PC1, however, EMEM was separated from Plasmax by both PC1 and PC2.

### 3.7. Gene Oncology (GO) Analysis of Proteins with Different Expressions

The GO analysis of significant proteins was also performed. As shown in Supporting Information Figure S7A,B, protein folding and cellular compromise of 231BR cell line are the mostly affected functions when compared to DMEM and EMEM, respectively. Post-translational modification is significantly changed compared to DMEM, while the alterations of protein synthesis, cellular function and maintenance are observed between Plasmax and EMEM. These two functions were also found different in CRL cell line when compared to both commercial media (Supporting Information Figure S7C,D). Moreover, the two functions of cellular movement, and cell death and survival were listed in both 231BR and CRL cell lines compared to the two commercial media, indicating that Plasmax tends to affect the expressions of the correlated proteins in different types of cancer cell lines.

### 3.8. Function Annotation of Significant Proteins

**Figure 4:**
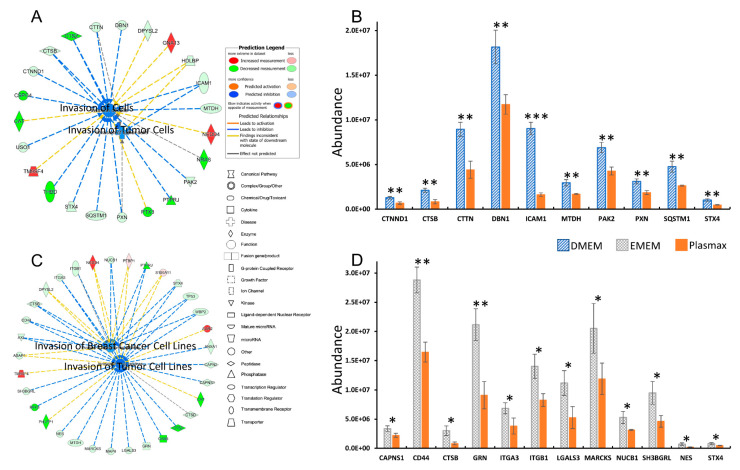
Functional annotation of significant proteins that associate with cell invasion of 231BR cell lines. (**A**) Inhibition of cell invasion of 231BR in Plasmax compared to DMEM; (**B**) Significant down-regulations of invasion-related proteins, Plasmax vs. DMEM. (**C**) Inhibition of cell invasion of 231BR in Plasmax compared to EMEM; (**D**) Significant down-regulations of invasion-related proteins, Plasmax vs. EMEM. * *p* < 0.05; ** *p* < 0.01; *** *p* < 0.001. Color codes and symbols are the same as Figure 2.

The Based on the GO analysis results, significant proteins that are associated with the altered cellular functions were grouped using software QIAGEN IPA to investigate the activations or inhibitions of such functions due to the up-or down-regulation of the protein expressions. For example, Figure 4A shows the differently expressed proteins, which are related to cell invasion of 231BR cell line cultivated in DMEM and Plasmax. The green color denotes the down-regulation of certain proteins in Plasmax, thus up-regulation in DMEM, while the red color indicates the up-regulation in Plasmax and down-regulation in DMEM. The blue dashed line means the alteration of protein expressions lead to the inhibition of the associated function. The bar graph in Figure 4B listed the cell invasion-related proteins with normalized LFQ intensities of 1.5-fold higher in DMEM compared to Plasmax. The error bars denote the standard deviations of the biological triplicates. Among these proteins, cathepsin B (CTSB), src substrate cortactin (CTTN), and syntaxin–4 (STX4) were observed with less than 50% of intensities in Plasmax compared to DMEM. Moreover, intercellular adhesion molecule 1 (ICAM 1) was found with less than 20% of intensities in Plasmax than in DMEM. Therefore, the invasive capability of 231BR cell line cultivated in Plasmax was predicted as inhibited by IPA, since the down-regulation of these correlated proteins.

Similar results were also observed between Plasmax and another commercial medium EMEM. Figure 4C suggests that the invasion of breast cancer cell lines and tumor cell lines were inhibited. The down-regulations of the related proteins were also listed as shown in Figure 4D. Again, the decrease in Cathepsin B (CTSB) was observed. Compared to the expression of CTSB in 231BR cell line cultivated in EMEM, the LFQ intensity was reduced by more than 70% in Plasmax. In addition to that, progranulin (GRN), galectin-3 (LGALS3) and nestin (NES) were identified with more than 2-fold higher intensities in EMEM than in Plasmax. In relative to both DMEM and EMEM, significant down-regulations of multiple proteins that related to cell invasion were observed, indicating that Plasmax might inhibit such function of 231BR breast cancer cell line.

Unlike 231BR, the invasion as well as the migration of brain cancer cell line CRL were predicted as activated in Plasmax than both DMEM and EMEM. As shown in Figure S8, multiple proteins that associated with cellular invasion and migration were observed with up-regulations when cultivated in Plasmax. For example, compared to DMEM (see Figure S8B), vimentin (VIM), annexin A1 (ANXA1), nucleophosmin (NPM1), and endoplasmic reticulum chaperone BiP (HSPA5) were over-expressed by 76%, 61%, 55%, and 41%, respectively. Compared to EMEM (Figure S8D), the abundances of VIM, ANXA1, and HSPA5 were also found with 73%, 30%, and 24% higher in Plasmax, respectively. Moreover, antiviral innate immune response receptor RIG-I (DDX58), dynamin-binding protein (DNMBP), O-GlcNAc transferase (OGT), and peptidyl-prolyl cis-trans isomerase NIMA-interacting 1 (PIN1) were only identified in Plasmax. In addition to invasion and migration of cells, the activation of protein translation, synthesis, and expression was also observed in CRL cell line cultivated by Plasmax. Supporting Information Figure S9 shows the correlated proteins with altered expressions, such as gelsolin (GSN) and protein niban 1 (NIBAN 1), as well as the aforementioned VIM and HSPA5.

Another affected cellular function is cell death, such as apoptosis and necrosis. Supporting Information Figure S10 depicts the inhibition of cell death for both 231BR (Figure S10A,B) and CRL (Figure S10D,E) cell lines cultivated in Plasmax compared to the two commercial media. Specifically, T-complex protein 1 subunit theta (CCT8) and glutaminase kidney isoform (GLS) extracted from 231BR cell line were found with more than 40% of abundance when cultivated in Plasmax than both commercial media. On the opposite, syntaxin-4 (STX4) and cathepsin B (CTSB) have significantly lower abundance in Plasmax. Moreover, glutathione peroxidase 1 (GPX1), e3-ubiquitin-protein ligase (NEDD4), nudC domain-containing protein 3 (NUDCD3), and transmembrane 9 superfamily member 4 (TM9SF4) were only expressed in Plasmax (see Figure S10C). The altered protein expressions discussed above contribute to the inhibition of cell death of 231BR cell line. The similar results of cell death were also confirmed in CRL cell line. Prothymosin α (PTMA) and thymosin β-10 (TMSB10) extracted from CRL cultivated in Plasmax have more than 2-fold of abundance compared to this cell line cultivated by the two commercial media. Furthermore, the expression of aldose reductase (AKR1B1) in Plasmax was 11.7 and 7.9 times higher than DMEM and EMEM, respectively. Along with the other significantly altered proteins in Figure S10F, the cell death of CRL cell line was also inhibited, suggesting that the physiological medium Plasmax can hinder the death of different types of cancer cells.

### 3.9. Pathway Analysis of Significant Proteins in MDA-MB-231BR Cell Line

**Figure 5:**
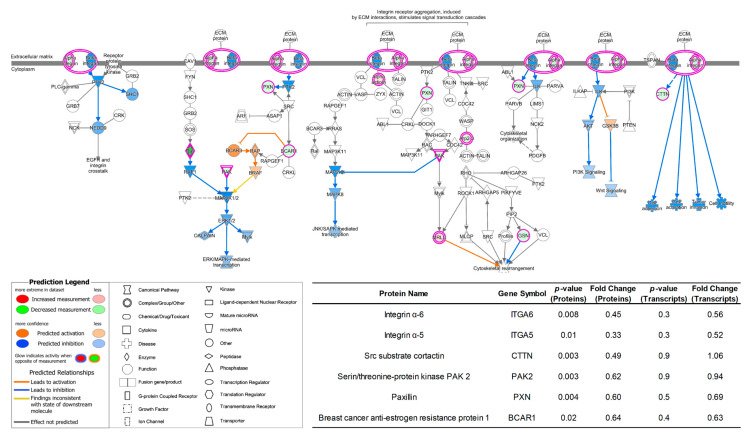
Inhibited integrin signaling pathway of protein expressions in 231BR cell line cultivated by Plasmax in comparison with DMEM. Color codes and symbols are the same as Figure 2. The inset table provides the *p*-values and fold-change numbers of proteins and their corresponding transcripts that are involved in this pathway.

Pathway analysis of proteins with significantly different expressions was also performed using QIAGEN IPA. According to the abovementioned cellular function alterations, the breast cancer cell line 231BR cultivated in Plasmax was less invasive compared to the ones cultivated using commercial media DMEM or EMEM. This result was also confirmed by studying the pathway which can regulate such functions. For example, Figure 5 depicts the integrin signaling pathway of 231BR cell line. In which, DMEM was utilized as baseline, while the proteomics data acquired from Plasmax-cultured 231BR was compared with DMEM. The integrin family includes α- and β-integrin, which are cell surface receptors that can facilitate the intercellular adhesion or attach cells to the extracellular matrix (ECM) for signal mediating [50,51]. It has been reported that integrins play important roles in cancer. For example, integrin signaling can affect the function and population of the stem cells and initiate tumor growth [52]. Additionally, it regulates various functions of tumor cells, such as invasion, migration, and proliferation [53]. For human breast cancer specifically, integrin was found associated with the dissemination of cancer cells via the bloodstream, thus leads to the metastasis of cancer to other parts of the body and becomes fatal [54]. Compared to DMEM, the integrin signaling pathway of 231BR cell line were inhibited in Plasmax due to the down-regulation of multiple proteins, which resulted in decreased of cancer cell invasion, migration, and adhesion.

To investigate the different expressions of these proteins from transcriptomics level and the correlation between the proteome and the transcriptome, the *p*-value and fold change information of the proteins and their corresponding transcripts are compared (see inset table in Figure 5). Most of the listed proteins had the same regulation trend as their transcripts. For example, the protein abundances of integrin α-5 and integrin α-6 were both down regulated in Plasmax in relative to DMEM, and the RPKM of their transcripts (ITGA5 and ITGA6) were also reduced. However, the *p*-values of all listed transcripts indicate that the differences of the gene expressions are not significant. Furthermore, using DMEM as the baseline, the fold change of some proteins and transcripts are different: the abundance of src substrate cortactin and serin/threonine-protein kinase PAK 2 derived from Plasmax-cultivated 231BR were decreased by 51% and 38%, respectively. However, the RPKM of CTTN and PAK2 are comparable between Plasmax and DMEM. The *p*-value and fold change discrepancies between the proteins and the transcripts could be related to the regulatory processes of protein expression after transcription, such as translation and post-translational modifications (PTM), which also suggests that Plasmax might have more impacts on the expressions of proteomes than transcriptomes during the cell line cultivation.

By using EMEM as the baseline, the inhibition of integrin signaling pathway of 231BR cell line in Plasmax were also observed. As shown by Supporting Information Figure S11, both of α- integrin (ITGA3) and β-integrin (ITGB1) were down-regulated. In addition to that, ARF GTPase-activating protein 1 (ASAP1), calpain-2 (CAPN2), and ras-related protein Rap-2b (RAP2B), which have been reported to promote the metastasis and invasiveness of different types of cancer cells [55,56,57,58,59], were also decreased in Plasmax.

The PAK2 that mentioned above in integrin signaling pathway is also involved in PAK signaling pathway (Supporting Information Figure S12), which is correlated with cell death or apoptosis. PAK familys include six isoforms (PAK1-6) of serin/threonine-protein kinase that can regulate cell survival and proliferation [60]. Among the 6 isoforms, PAK2 has been reported as the unique one because of its dual function for cell death and survival regulation. The activated full-length PKA2 can stimulate cell survival and growth [61], however the caspase-activated PAK-2p34 fragment promotes cell death and apoptosis [62]. In this study, PAK-2p34 was down-regulated in Plasmax, thus the stimulation of cell death can be more active due to the higher abundance of PAK-2p34 fragments when cultivated in the commercial media.

Based on the significant changes of protein expressions, IPA also predicted the upstream regulators and downstream functions. As depicted by Figure S13A, compared to DMEM, the three blue nodes on top are three upstream regulators: STIM1, PGR, and THPO. They were predicted as down-regulated by IPA, which would lead to the down-regulation of the intermediate proteins in the middle shown by the green nodes. Then, these proteins could further inhibit the downstream functions such as cell movement, invasion, and adhesion of tumor cell lines. Similar when compared to EMEM (Figure S13B), five upstream regulators were predicted by IPA, including MAP2K1, CD40, PGR, estrogen receptor, and MYOC, which would result in the down-regulations of the correlated proteins, followed by inhibited downstream functions of invasion of tumor cells.

### 3.10. Pathway Analysis of Significant Proteins in CRL-1620 Cell Line

**Figure 6:**
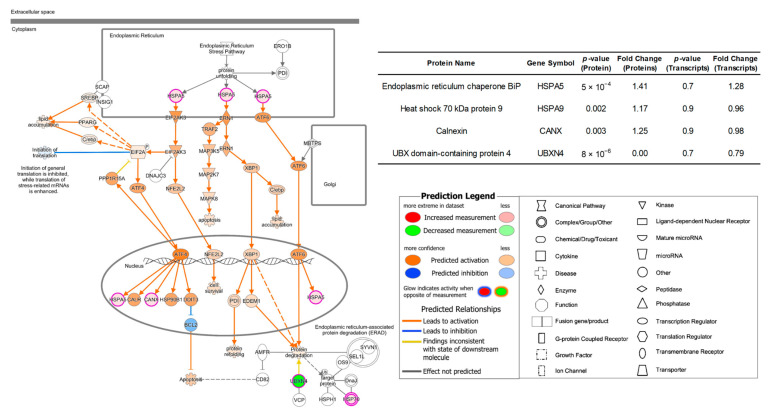
Activated unfolded protein response pathway of protein expressions in CRL cell line cultivated by Plasmax in comparison with DMEM. Color codes and symbols are the same as Figure 2. The inset table provides the *p*-values and fold-change numbers of proteins and their corresponding transcripts that are involved in this pathway.

In contrary to the 231BR cell line, the cell movement capability, such as cell invasion and migration of brain cancer cell line CRL were more activated when cultured in Plasmax than both DMEM and EMEM. Additionally, the functions related to protein expressions and synthesis were also stimulated by Plasmax. The abovementioned function alterations are associated with the activation of unfolded protein response (UPR) signaling pathway as shown in Figure 6 and Supporting Information Figure S14, using DMEM and EMEM as baseline, respectively.

UPR is an adaptive mechanism in eukaryotic cells to response to the endoplasmic reticulum (ER) stress of misfolded proteins [63]. The protein chaperone GRP78/HSPA5 regulates three major ER stress sensors, including PKR-like endoplasmic reticulum kinase (PERK/EIF2AK3) [64], inositol-requiring enzyme 1 (IRE1/ERN1) [65], and activating transcription factor 6 α (ATF6) [66]. Then, the three sensors activate the downstream pathways to reduce the protein misfolding. During cancer development, various cellular function alterations can be involved, such as proliferation, migration, and invasion. Therefore, an increase in protein synthesis and protein folding is expected, which leads to UPR activation [67,68,69]. The connection between the overexpression of GRP78/HSPA5 and cancer cell invasion and metastasis has also been reported by several studies [70,71,72,73].

In our work, the chaperon GRP78 of CRL cell line and its corresponding transcript HSPA5 were up-regulated by 41% and 28%, respectively, when cultivated in Plasmax than in DMEM. (inset table of Figure 6) Thus, the UPR signaling pathway was activated. The significant up-regulation (*p* < 0.01) of proteins involved in the downstream signaling pathway, including heat shock 70 kDa protein 9 (HSPA9) and calnexin (CANX) were also observed in Plasmax. However, similar as discussed in 231BR cell line, the transcripts of HSPA9 and CANX did not present any significant expression changes, (*p* > 0.9) suggesting that the expressions of these downstream proteins are not only regulated by their transcripts but also controlled by other regulatory processed after transcriptions.

Another important finding of CRL cell line is the overexpression of 14-3-3 family proteins. By binding to the phosphorylated serine or threonine residues of the target proteins [74], 14-3-3 proteins can increase their activity or stability and regulate different cellular processes such as cell cycle, cell proliferation and apoptosis [75]. The 14-3-3ε (YWHAE) isoform has been reported with promoting cell survival by targeting the pro-apoptotic proteins and blocking their activities [76]. The 14-3-3 proteins also play a key role in cancer progression. For example, both of the isoforms of 14-3-3β (YWHAB) and 14-3-3τ/θ (YWHAQ) have been reported of contributing cancer cell migration and invasion [77,78,79]. Supporting Information Figure S15 depicts the activated 14-3-3 mediated signaling pathway of CRL cell line cultivated by Plasmax compared to DMEM, where all three isoforms discussed above (ε, β, τ/θ) were up-regulated by Plasmax, leading to the activation of the correlated functions. Furthermore, the regulator effects analysis indicates that the upstream regulator OSM was up-regulated in CRL cell line when cultivated in Plasmax. The intermediated proteins shown in Figure S16 are related to the downstream function of cell proliferation of tumor cell lines, which was predicted as up-regulated.

## 4. Summary

In this study, we investigated and compared the physiological cell culturing media, Plasmax with two commercially available media DMEM and EMEM through transcriptomics and proteomics. Significant differences of gene and protein expressions were observed in two cancer cell lines cultured by these three media. The gene oncology and pathway analyses of significant transcriptomes suggest alterations in cellular functions. The cell proliferation and invasion of 231BR were inhibited by Plasmax compared to DMEM. However, these functions were more activated than in EMEM. For CRL, the calcium signaling pathway suggested inhibition of cell growth and development in Plasmax in relative to DMEM, while cell cycle was hindered compared to EMEM. On the other hand, the GO analyses of significant proteins indicate that cellular movement, and cell death and survival were the top affected cellular functions in both breast cancer and brain cancer cell lines. According to the expression changes of proteins in 231BR cell line, it also exhibits less cellular invasiveness in Plasmax compared to both commercial media. However, for CRL cell line, its invasion and migration were predicted to be enhanced in Plasmax. The protein synthesis in CRL was another promoted function by Plasmax in relative to DMEM and EMEM. Moreover, the cell death of both cancer cell lines was inhibited when cultivated in Plasmax than both commercial media. The pathway analysis of proteins was utilized to further assess the cellular function alterations introduced by different media. Compared to the commercial media, breast cancer cell line 231BR cultivated in Plasmax exhibited the inhibition of integrin signaling, and PAK signaling pathways, leading to the decrease in cell invasion and cell death, while the activation of unfolded protein response signaling and 14-3-3 mediated signaling pathways in brain cancer cell line CRL are associated with enhanced protein synthesis, cellular migration and invasion.

The comparisons between the protein expressions and their corresponding transcriptomes involved in the inhibited or activated pathways suggest that Plasmax has more impacts on these protein expressions, since the RPKM changes of the genes were not as significant as the proteins. In addition, both of transcriptomics and proteomics analyses of 231BR cell line indicated that the invasiveness was inhibited by Plasmax in relative to DMEM. However, the transcriptomics and proteomics analyses suggested opposite results when compared to EMEM. Additionally, the pathway analyses of significant genes and proteins in CRL cell line led to different cellular functions that were affected. The inconsistency between transcriptomics and proteomics indicates that Plasmax might affect the protein expressions after the translations, thus resulting in unique function alterations in proteomics analyses. It can be expected due to the complicated regulation between proteins and their transcripts, with different level of enzymes involved. Overall, these variances of gene and protein expressions indicate the potential influence from the nutrient environment of Plasmax and result in cell behavior changes in different types of cancer cell lines. Our investigation of transcriptomics and proteomics provided complementary information to the original study which focused on the cell proliferation and metabolomics. Researchers may find more clues about media selection for in vitro cell-based transcriptomics or proteomics in future studies.

## Data Availability

Transcriptomics data were submitted to NCBI BioProject PRJNA837884 with the BiosSample accession SAMN28233490. The data can be accessed via a reviewer’s link at https://dataview.ncbi.nlm.nih.gov/object/PRJNA837884?reviewer=tbilmnanuejqdhskqr4bl7c0fc, accessed on 1 September 2022. Proteomics data have been deposited to the ProteomeXchange Consortium via the PRIDE partner repository with the dataset identifier PXD033841. All the information necessary to access the data is provided using the following credentials: Username: reviewer_pxd033841@ebi.ac.uk; Password: RRbJMZC1.

## Supplementary Materials

The following supporting information can be downloaded at: https://www.mdpi.com/article/10.3390/biom12111575/s1, Table S1. Full list of the formulation of Plasmax. Table S2. RPKM of identified transcriptomes from 231 BR and CRL cell lines. Table S3. Normalized LFQ intensities of identified proteins from 231 BR and CRL cell lines. Figure S1. Major nutritional compositions of three media. Figure S2. GO enrichment of molecular and cellular functions of transcriptomes that exhibited significantly different expressions. Figure S3. Activated pathway of breast cancer regulation by stathmin 1 of 231BR cell line cultivated by Plasmax compared to EMEM. Figure S4. Inhibited cyclins and cell cycle regulation of CRL cell line in Plasmax vs. EMEM. Figure S5. Heatmaps of transcriptome expressions that exhibited significant differences among the two cell lines cultured in Plasmax and commercial media. Figure S6. Unsupervised principal component analysis of protein expressions of two cell lines. Figure S7. GO enrichment of molecular and cellular functions of proteins that exhibited significantly different expressions. Figure S8. Functional annotation of significant proteins that associate with cell invasion and migration of CRL cell lines. Figure S9. Functional annotation of significant proteins that associate with protein synthesis function in CRL cell line. Figure S10. Functional annotation of significant proteins that associate with cell death. Figure S11. Integrin signaling pathway of protein expressions from 231BR cell line cultivated in Plasmax vs. EMEM. Figure S12. PAK signaling pathway of protein expressions from 231BR cell line cultivated in Plasmax vs. DMEM. Figure S13. Regulator effects analysis of 231BR cell line. Figure S14. Unfolded protein response signaling pathway of protein expressions from CRL cell line cultivated by Plasmax vs. EMEM. Figure S15. 14-3-3 mediated signaling pathway of protein expressions from CRL cell line cultivated by Plasmax vs. DMEM. Figure S16. Regulator effects analysis of CRL cell line.
