# Supplementary material for: Comparative Transcriptomics and Proteomics of Cancer Cell Lines Cultivated by Physiological and Commercial Media
Source: Biomolecules. 2022 Oct 27;12(11):1575. doi: 10.3390/biom12111575 (PMC9687725; doi:10.3390/biom12111575)
Supplement: Supplementary file 1 [file biomolecules-12-01575-s001.zip › biomolecules-1924587-supplementary.pdf]

Supporting Information

Comparative Transcriptomics and Proteomics of Cancer Cell Lines Cultivated by Physiological and Commercial Media

Junyao Wang[1], Wenjing Peng[1], Aiying Yu[1], Mohamed Fokar[2] and Yehia Mechref[1,2*]

[1] Department of Chemistry and Biochemistry, Texas Tech University, Lubbock TX, 79409
[2] Center of Biotechnology and Genomics, Texas Tech University, Lubbock TX, 79409

*Correspondence: Yehia.Mechref@ttu.edu (Y.M.); Tel: 806-742-3059

Keywords: Proteomics, Transcriptomics, Culture media, Cancer cell line, Differential expression analysis, LC-MS/MS

Table of Contents

Table S1. Full list of the formulation of Plasmax.

Table S2. This table is provided by an attached Excel file which contains RPKM of identified transcriptomes from 231 BR and CRL cell lines.

Table S3. This table is provided by an attached Excel file which contains normalized LFQ intensities of identified proteins from 231 BR and CRL cell lines.

Figure S1. Major nutritional compositions of three media: Plasmax, EMEM, and DMEM.

Figure S2. GO enrichment of molecular and cellular functions of transcriptomes that exhibited significantly different expressions. **(A)** 231BR cell line Plasmax *vs.* DMEM; **(B)** 231BR cell line Plasmax *vs.* EMEM; **(C)** CRL cell line Plasmax *vs.* DMEM; **(D)** CRL cell line Plasmax *vs.* EMEM.

Figure S3. Activated pathway of breast cancer regulation by stathmin 1 of 231BR cell line cultivated by Plasmax compared to EMEM. Blue line denotes that the corresponding protein change cause an inhibition to the function in Plasmax. Orange line denotes that the corresponding protein change cause an activation to the function in Plasmax, and yellow line denotes the inconsistent changes of corresponding proteins and their downstream molecules.

Figure S4. Inhibited cyclins and cell cycle regulation of CRL cell line in Plasmax *vs.* EMEM. Color codes and symbols are same as **Figure S3**.

Figure S5. Heatmaps of transcriptome expressions that exhibited significant differences among the two cell lines cultured in Plasmax and commercial media **(A)** Significant proteins from 231BR cell line cultivated in Plasmax and DMEM; **(B)** Significant proteins from

231BR cell line cultivated in Plasmax *vs.* EMEM; **(C)** Significant proteins from CRL cell line cultivated in Plasmax *vs.* DMEM; **(D)** Significant proteins from CRL cell line cultivated in Plasmax *vs.* EMEM. The color codes denote expression level of genes. The green color denotes lower expression, while the red color denotes higher expression.

Figure S6. Unsupervised principal component analysis of protein expressions of **(A)** 231BR cell line and **(B)** CRL cell line in three media. Symbols, Plasmax, red square; DMEM, magenta diamond; EMEM, green diamond.

Figure S7. GO enrichment of molecular and cellular functions of proteins that exhibited significantly different expressions. **(A)** 231BR cell line Plasmax *vs.* DMEM; **(B)** 231BR cell line Plasmax *vs.* EMEM; **(C)** CRL cell line Plasmax *vs.* DMEM; **(D)** CRL cell line Plasmax *vs.* EMEM.

Figure S8. Functional annotation of significant proteins that associate with cell invasion and migration of CRL cell lines. **(A)** Activation of cell invasion and migration of CRL in Plasmax compared to DMEM; **(B)** Significant up-regulations of correlated proteins, Plasmax *vs.* DMEM. **(C)** Activation of cell invasion and migration of CRL in Plasmax compared to EMEM; **(D)** Significant up-regulations of correlated proteins, Plasmax *vs.* EMEM. Color codes and symbols are the same as **Figure 2**.

Figure S9. Functional annotation of significant proteins that associate with protein synthesis function in CRL cell line. **(A)** Activation of synthesis and translation of proteins in Plasmax compared to DMEM. **(C)** Activation of expression and synthesis of proteins in Plasmax compared to EMEM. **(B)** Bar graph of the associated protein abundances (Plasmax *vs.* DMEM). **(D)** Bar graph of the associated protein abundances (Plasmax *vs.* EMEM). *: $p<0.05$; **: $p<0.01$; ***: $p<0.001$. Color codes and symbols are same as **Figure S3**.

Figure S10. Functional annotation of significant proteins that associate with cell death of **(A)** 231BR cell line, Plasmax vs. DMEM; **(B)** 231BR cell line, Plasmax vs. EMEM; **(D)** CRL cell line, Plasmax vs. DMEM; **(E)** CRL cell line, Plasmax vs. EMEM. **(C)** Normalized abundances of associated proteins derived from 231BR cell line; **(F)** Normalized abundances of associated proteins derived from CRL cell line. *: $p<0.05$; **: $p<0.01$; ***: $p<0.001$. Color codes and symbols are same as **Figure S3**.

Figure S11. Integrin signaling pathway of protein expressions from 231BR cell line cultivated in Plasmax *vs*. EMEM. Color codes and symbols are same as **Figure S3**.

Figure S12. PAK signaling pathway of protein expressions from 231BR cell line cultivated in Plasmax *vs*. DMEM. Color codes are same as **Figure S3**.

Figure S13. Regulator effects analysis of 231BR cell line.

Figure S14. Unfolded protein response signaling pathway of protein expressions from CRL cell line cultivated by Plasmax *vs*. EMEM. Color codes are same as **Figure S3**.

Figure S15. 14-3-3 mediated signaling pathway of protein expressions from CRL cell line cultivated by Plasmax *vs*. DMEM. Color codes are same as **Figure S3**.

Figure S16. Regulator effects analysis of CRL cell line.

Table S1. Full list of the formulation of Plasmax.

No.	Component	Vendor	Product No.	(g/mol)	C (μM)	Weight for 1L(mg)
1	L-Alanine	Sigma	A7469-25G	89.09	510	45.44
	L-Arginine	Sigma	A8094-25G	174.20	64	11.15
	L-Asparagine	Sigma	A4159-25G	132.12	41	5.42
	L-Aspartic acid	Sigma	A4534-100G	133.10	6	0.80
	L-Glutamate	Sigma	RES5063G-A701X	187.13	98	18.34
	Glycine	Sigma	G-8898-1KG	75.07	330	24.77
	L-Histidine	Sigma	H6034-25G	155.15	120	18.62
	L-Isoleucine	Sigma	I2752-25G	131.17	140	18.36
	L-Leucine	Sigma	L8912-25G	131.17	170	22.30
	L-Lysine	Sigma	L5501-25G	146.19	220	32.16
	L-Methionine	Sigma	M5308-25G	149.21	30	4.48
	L-Phenylalanine	Sigma	P5482-25G	165.19	68	11.23
	L-Proline	Sigma	P5607-25G	115.13	360	41.45
	L-Serine	Sigma	S4311-25G	105.09	140	14.71
	L-Threonine	Sigma	T8441-25G	119.12	240	28.59
	L-Tryptophan	Sigma	T8941-25G	204.23	78	15.93
	L-Tyrosine	Sigma	T8566-25G	181.19	74	13.41
	L-Valine	Sigma	V0513-25G	117.15	230	26.94
	L-Citrulline	Sigma	C7629-5G	175.19	55	9.64
	L-Cystine	Sigma	C7602-25G	240.30	65	15.62
	L-Ornithine	Sigma	O6503-25G	168.62	80	13.49
2	L-Cysteine	Sigma	C7352-25G	121.16	33	4.00
	DL-2-Aminobutyric acid	Sigma	162663-25G	103.12	41	4.23
	L-Homocysteine	Sigma	69453-10MG	135.18	9	1.22
	4-Hydroxy-L-proline	Sigma	H54409-10G	131.13	13	1.70
	L-Pyroglutamate	Sigma	83160-25G	129.11	20	2.58
	L-Acetyl glycine	Sigma	A16300-100G	117.10	70	8.20
	L-Carnosine	Sigma	C9625-5G	226.23	6	1.36
	Glutathione (reduced)	Sigma	1040900005	307.32	37	11.37
	Taurine	Sigma	T8691-25G	125.15	130	16.27
	N-trimethylglycine (Betaine)	Sigma	B2629-50G	117.15	72	8.43
	Acetate	Sigma	241245-5G	82.03	42	3.45
	Acetone	MACRON	MK-2435-10	58.08	55	3.19
	O-Acetyl-L-carnitine hydrochloride	Sigma	A6706-1G	239.70	5	1.20

No.	Component	Vendor	Product No.	(g/mol)	C (μM)	Weight for 1L(mg)
2	Sodium citrate dihydrate	Sigma	W302600-1KG-K	294.10	114	33.53
	Carnitine	Sigma	C0283-5G	197.66	46	9.09
	Creatine	Sigma	C0780-50G	131.13	37	4.85
	Creatinine	Sigma	C4255-25G	113.12	74	8.37
	Sodium formate	Sigma	71539-500G	68.01	33	2.24
	Glycerol	Sigma	G5516-100ML	92.09	82	7.55
	2-hydroxybutyric acid sodium salt	Sigma	220116-5G	126.09	31	3.91
	(+-)-sodium 3-hydroxybutyrate	Sigma	54965-10G-F	126.09	77	9.71
	(+-)-sodium B-hydroxyisobutyrate	Sigma	36105-250MG	126.09	20	2.52
	Hypoxanthine	Sigma	H9636-5G	136.11	5	0.68
	Lactate	Sigma	71718-10G	112.06	500	56.03
	Methyl acetoacetate	Sigma	537365-100G	116.12	41	4.76
	di-sodium succinate	Sigma	8186010100	162.05	23	3.73
	Uracil	Sigma	U1128-25G	112.09	2	0.22
	Urea	Thermo Scientific	Prod#29700	60.06	3000	180.17
	Uridine	Sigma	U3003-5G	244.20	3	0.73
3	Ammonium Chloride	MACRON	MK-3384-12	53.49	50	26.74
	Ferric Sulfate	Sigma	307718-100G	399.88	1.0428	4.17
	Zinc Sulfate	Sigma	Z0251-100G	287.56	1.5	4.31
	Ammonium Metavanadate	Sigma	10028-25G	116.98	0.0026	3.04
	Copper(II) sulfate	Sigma	C1297-100G	159.61	0.0052	8.30
	Ferric Nitrate	Sigma	F8508-100G	404.00	0.1238	500.15
	Manganous Chloride	Sigma	244589-10G	125.84	0.0002	0.25
	Sodium Selenite	Sigma	S5261-10G	172.94	0.0289	49.98
4	Urate	Sigma	U2875-5G	190.09	270	256.62
5	L-Glutamine	Sigma	59202C-100ML	146.15	650	94.99
	BME vitamins	Sigma	B6891-100ML	-	-	-
6	Ascorbate	Sigma	A4034-100G	198.11	62	1228.28
	Vitamin B12	Sigma	V6629-100MG	1355.37	0.005	0.68
7	EBSS	ThermoFisher	24010043	-	-	-
8	sodium pyruvate	Sigma	P5280-25G	110.04	100.00	11.00

Figure S1

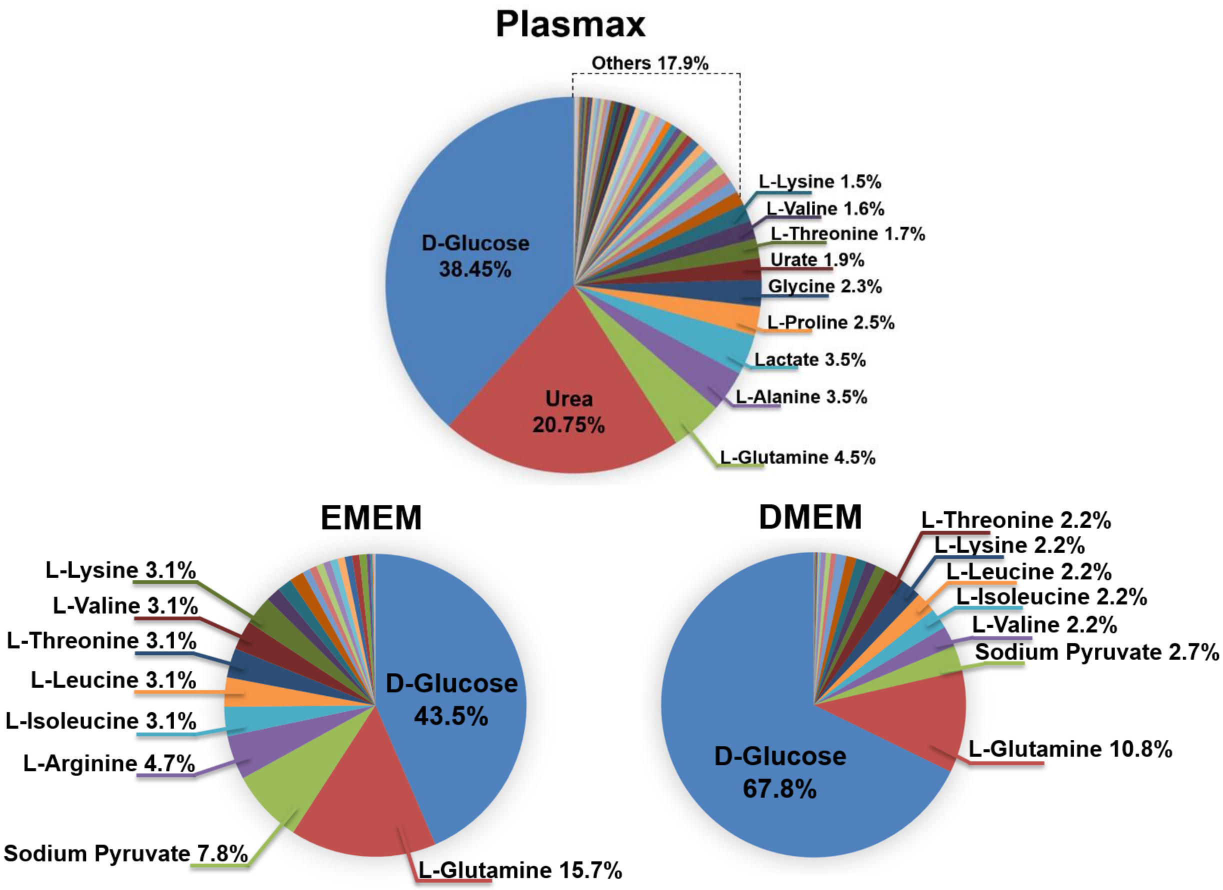

Figure S1. Major nutritional compositions of three media: Plasmax, EMEM, and DMEM.

Figure S2

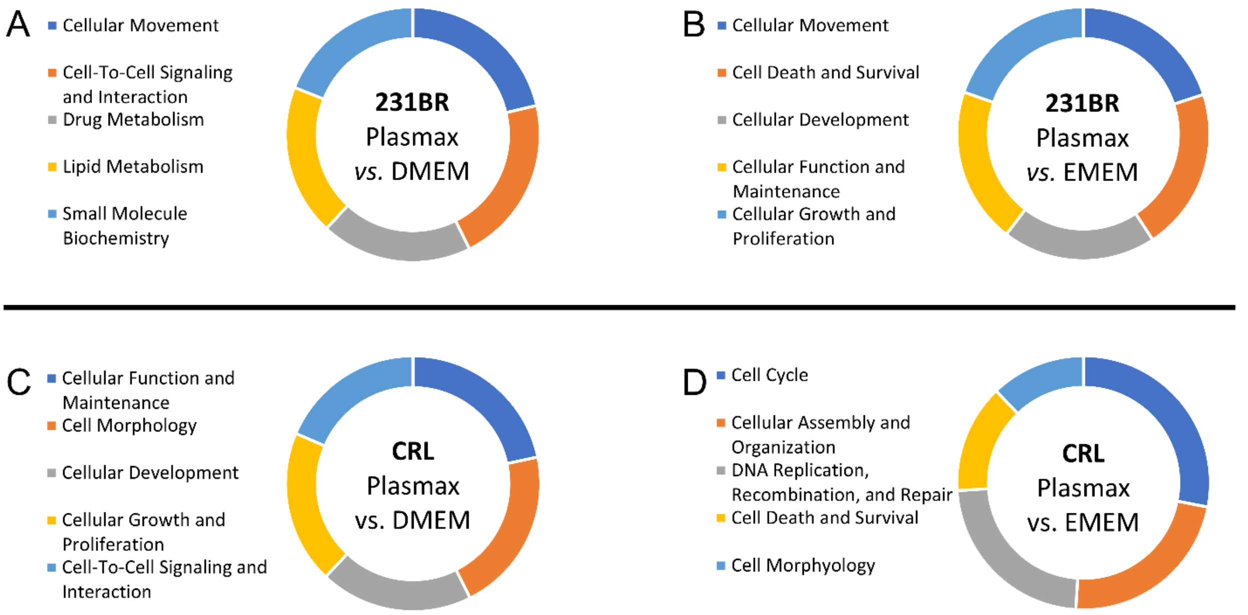

Figure S2. GO enrichment of molecular and cellular functions of transcriptomes that exhibited significantly different expressions. **(A)** 231BR cell line Plasmax *vs.* DMEM; **(B)** 231BR cell line Plasmax *vs.* EMEM; **(C)** CRL cell line Plasmax *vs.* DMEM; **(D)** CRL cell line Plasmax *vs.* EMEM.

Figure S3

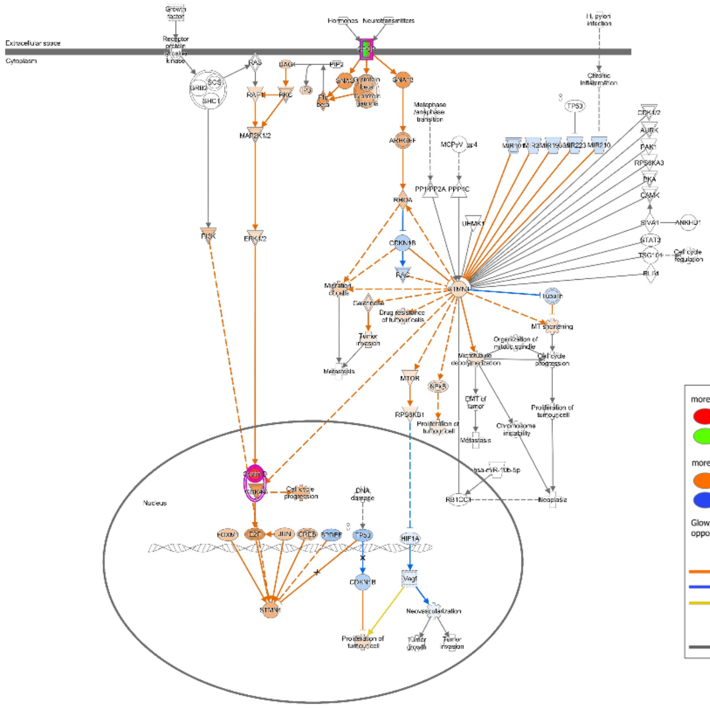

Gene Symbol	Protein Name	*p*-value	Fold Change (Plasmax/EMEM)
GPR17	G protein-coupled receptor 17	4.E-02	3.79
S1PR1	Sphingosine-1-phosphate receptor 1	5.E-02	1.90
CCND2	Cyclin D2	2.E-02	∞

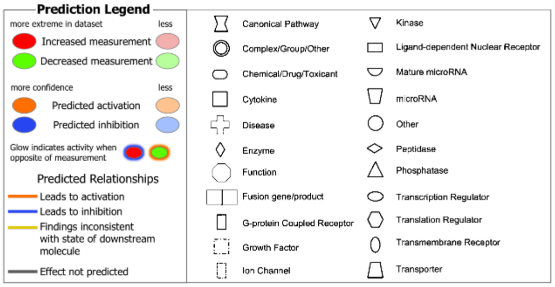

Figure S3. Activated pathway of breast cancer regulation by stathmin 1 of 231BR cell line cultivated by Plasmax compared to EMEM. Blue line denotes that the corresponding protein change cause an inhibition to the function in Plasmax. Orange line denotes that the corresponding protein change cause an activation to the function in Plasmax, and yellow line denotes the inconsistent changes of corresponding proteins and their downstream molecules.

Figure S4

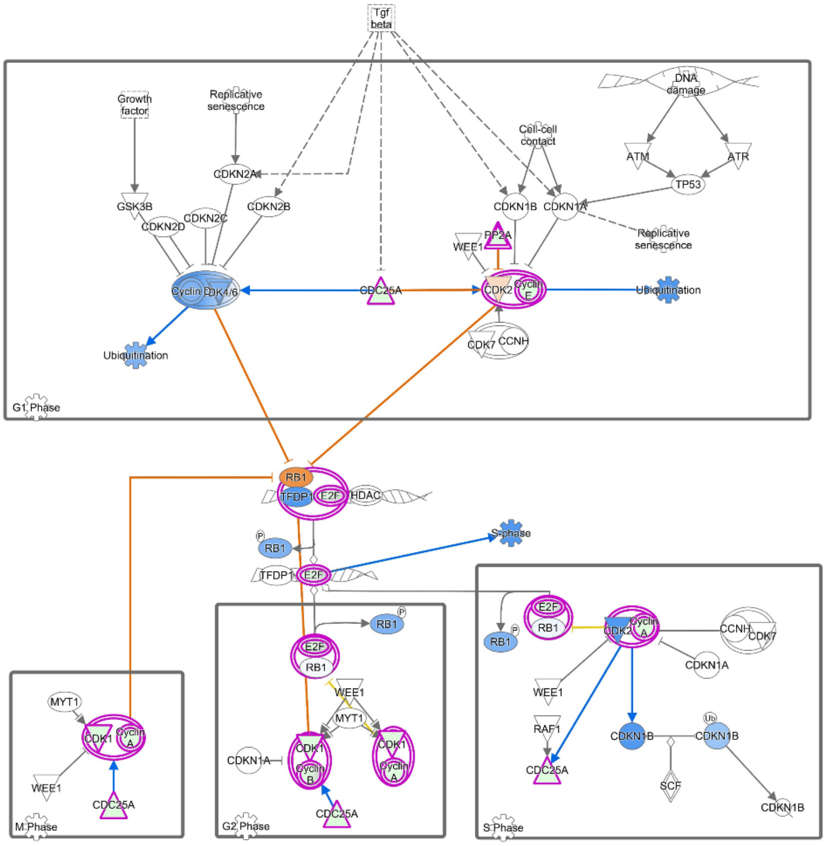

Gene Symbol	Protein Name	*p*-value	Fold Change (Plasmax/EMEM)
CCNA2	Cyclin A2	6.E-03	0.08
CCNB1	Cyclin B1	2.E-02	0.24
CCNB2	Cyclin B2	4.E-03	0.16
CDC25A	Cell division cycle 25A	7.E-03	0.24
CDK1	Cyclin dependent kinase 1	1.E-02	0.12
E2F1	E2F transcription factor 1	7.E-03	0.24

Figure S4. Inhibited cyclins and cell cycle regulation of CRL cell line in Plasmax *vs.* EMEM. Color codes and symbols are same as **Figure S3**.

Figure S5

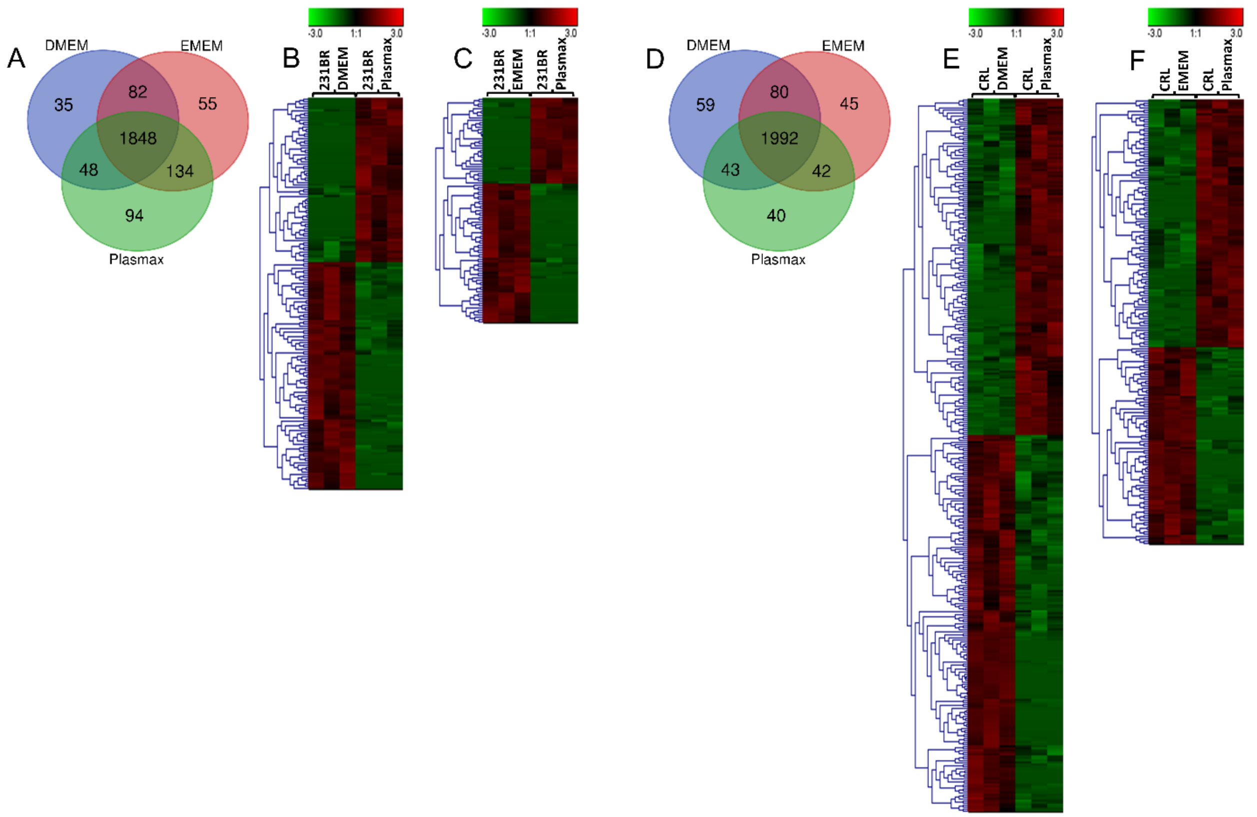

Figure S5. Heatmaps of transcriptome expressions that exhibited significant differences among the two cell lines cultured in Plasmax and commercial media **(A)** Significant proteins from 231BR cell line cultivated in Plasmax and DMEM; **(B)** Significant proteins from 231BR cell line cultivated in Plasmax *vs.* EMEM; **(C)** Significant proteins from CRL cell line cultivated in Plasmax *vs.* DMEM; **(D)** Significant proteins from CRL cell line cultivated in Plasmax *vs.* EMEM. The color codes denote expression level of genes. The green color denotes lower expression, while the red color denotes higher expression.

Figure S6

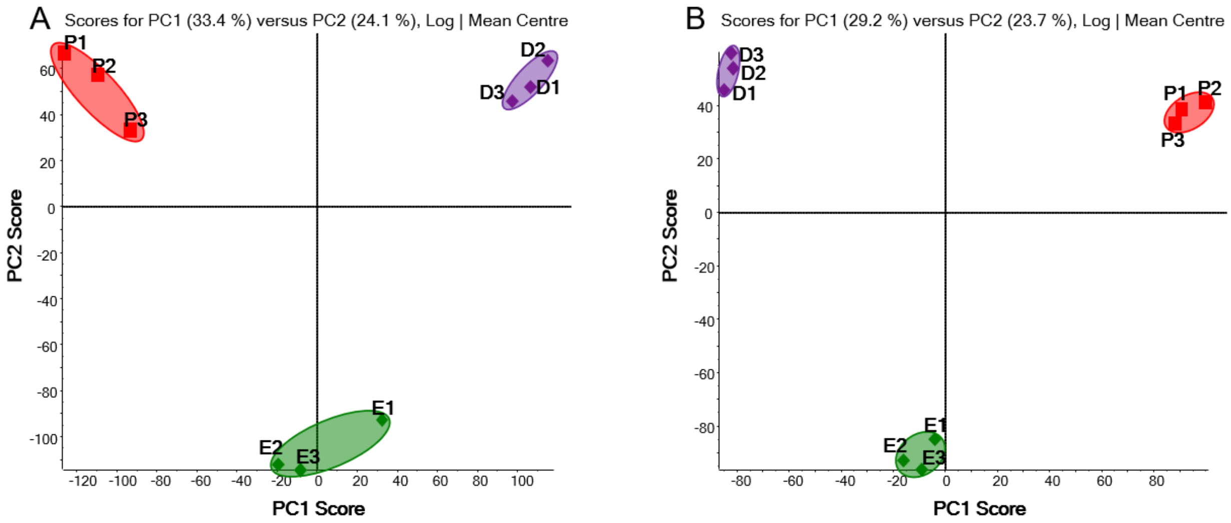

Figure S6. Unsupervised principal component analysis of protein expressions of **(A)** 231BR cell line and **(B)** CRL cell line in three media. Symbols, Plasmax, red square; DMEM, magenta diamond; EMEM, green diamond.

Figure S7

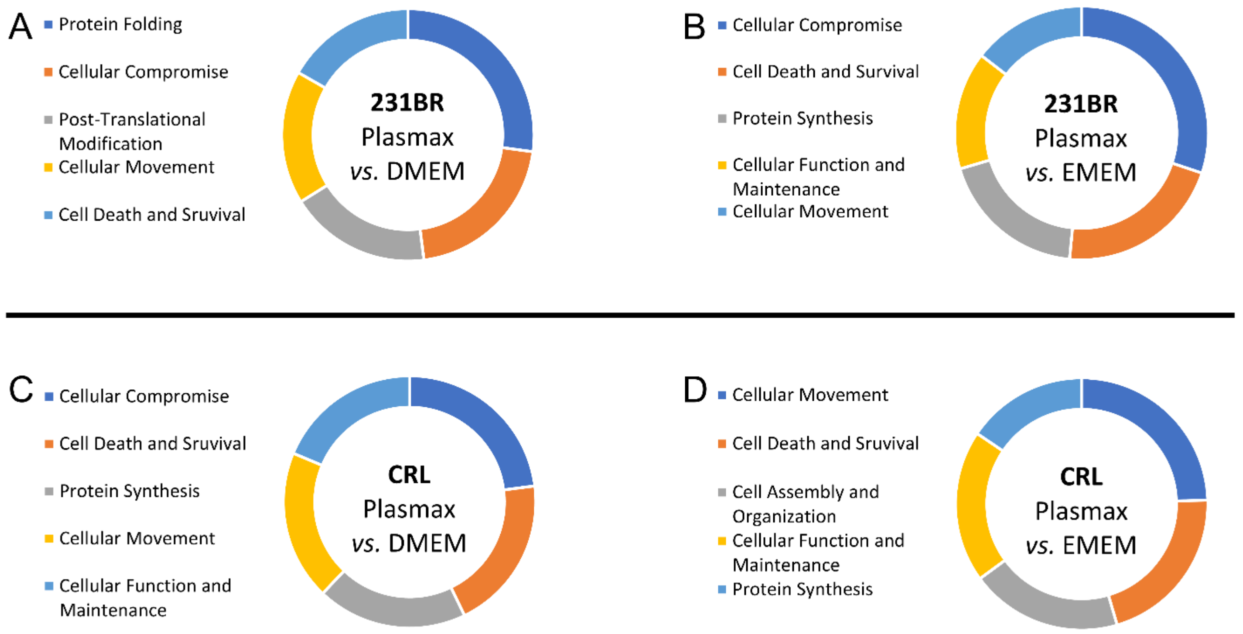

Figure S7. GO enrichment of molecular and cellular functions of proteins that exhibited significantly different expressions. **(A)** 231BR cell line Plasmax *vs.* DMEM; **(B)** 231BR cell line Plasmax *vs.* EMEM; **(C)** CRL cell line Plasmax *vs.* DMEM; **(D)** CRL cell line Plasmax *vs.* EMEM.

Figure S8

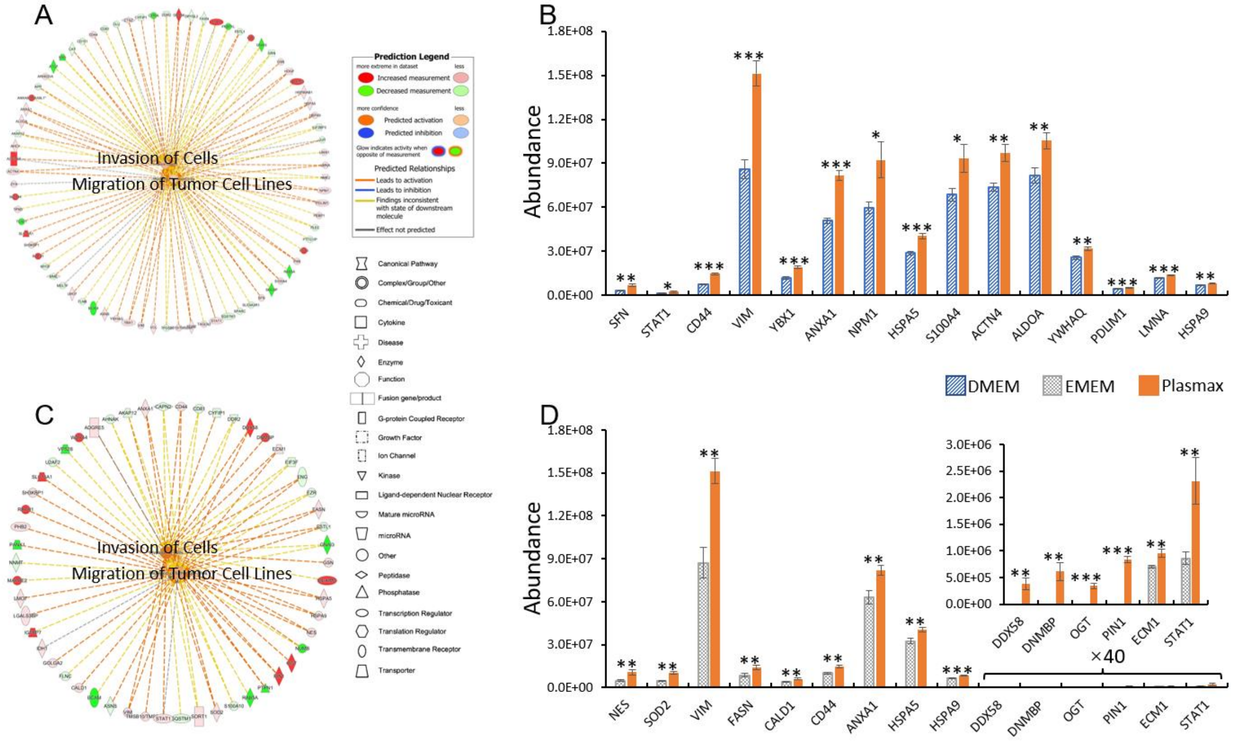

Figure S8. Functional annotation of significant proteins that associate with cell invasion and migration of CRL cell lines. **(A)** Activation of cell invasion and migration of CRL in Plasmax compared to DMEM; **(B)** Significant up-regulations of correlated proteins, Plasmax *vs*. DMEM. **(C)** Activation of cell invasion and migration of CRL in Plasmax compared to EMEM; **(D)** Significant up-regulations of correlated proteins, Plasmax *vs*. EMEM. Color codes and symbols are the same as **Figure S3**.

Figure S9

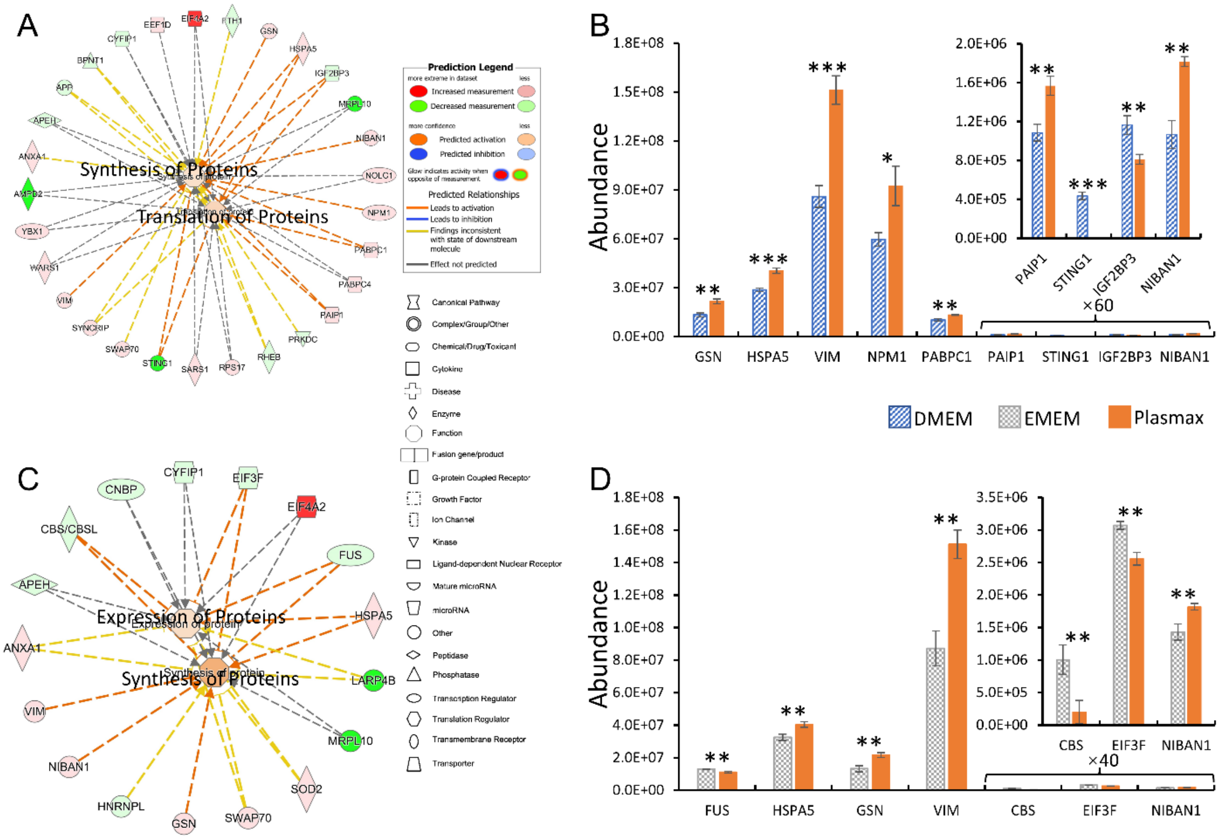

Figure S9. Functional annotation of significant proteins that associate with protein synthesis function in CRL cell line. **(A)** Activation of synthesis and translation of proteins in Plasmax compared to DMEM. **(C)** Activation of expression and synthesis of proteins in Plasmax compared to EMEM. **(B)** Bar graph of the associated protein abundances (Plasmax *vs.* DMEM). **(D)** Bar graph of the associated protein abundances (Plasmax *vs.* EMEM). *: p<0.05; **: p<0.01; ***: p<0.001. Color codes and symbols are same as **Figure S3**.

Figure S10

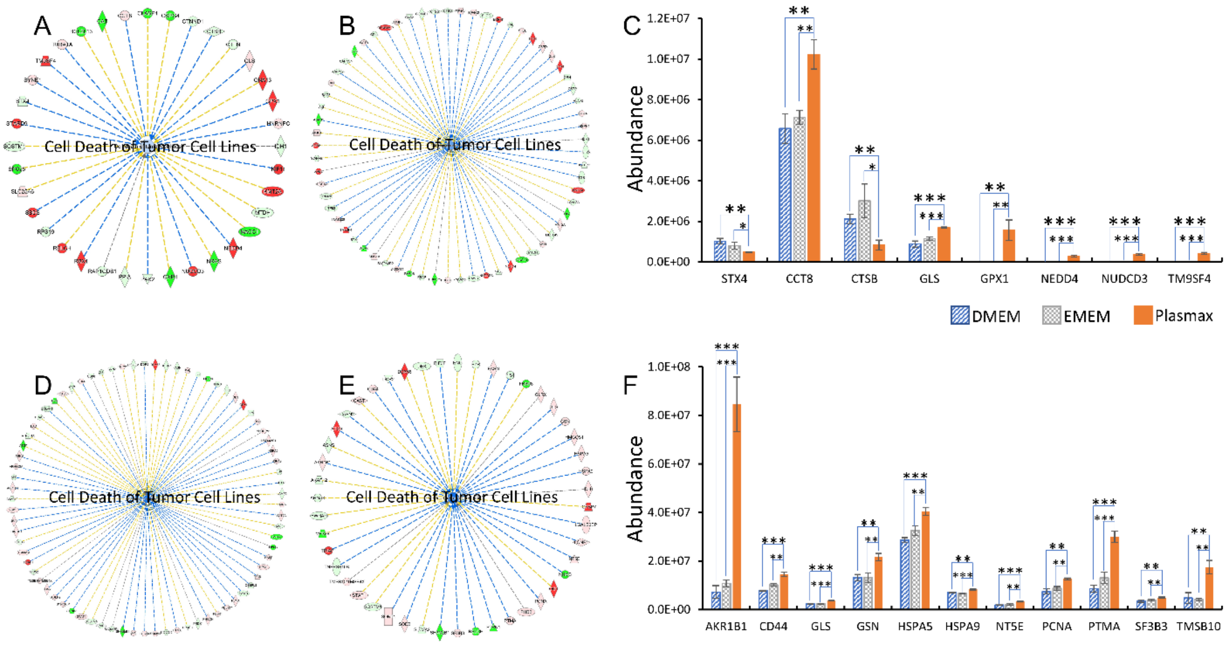

Figure S10. Functional annotation of significant proteins that associate with cell death of **(A)** 231BR cell line, Plasmax vs. DMEM; **(B)** 231BR cell line, Plasmax vs. EMEM; **(D)** CRL cell line, Plasmax vs. DMEM; **(E)** CRL cell line, Plasmax vs. EMEM. **(C)** Normalized abundances of associated proteins derived from 231BR cell line; **(F)** Normalized abundances of associated proteins derived from CRL cell line. *: $p<0.05$; **: $p<0.01$; ***: $p<0.001$. Color codes and symbols are same as **Figure S3**.

Figure S11

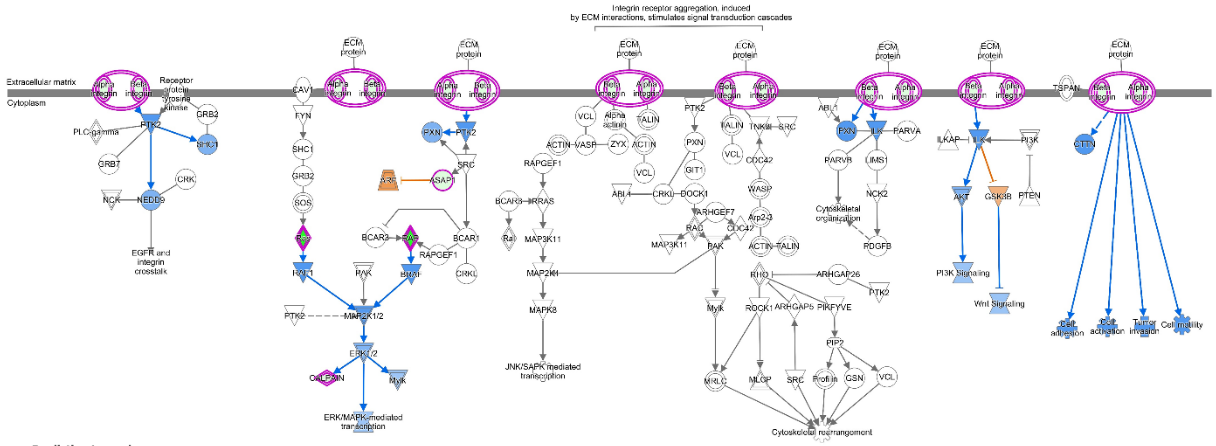

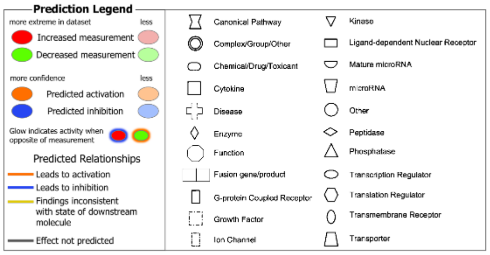

Portein Name	Gene Symbol	*p*-value	Fold Change (Plasmax/EMEM)	Log_2(Fold change) (Plasmax/EMEM)
Integrin α-3	ITGA3	3.E-02	0.56	-0.85
Integrin β-1	ITGB1	1.E-02	0.59	-0.76
ARF GTPase-activating protein 1	ASAP1	1.E-02	0.61	-0.72
Calpain-2	CAPN2	1.E-02	0.76	-0.40
Ras-related protein Rap-2b	RAP2B	1.E-04	0.00	-∞

Figure S11. Integrin signaling pathway of protein expressions from 231BR cell line cultivated in Plasmax *vs*. EMEM. Color codes and symbols are same as **Figure S3**.

Figure S12

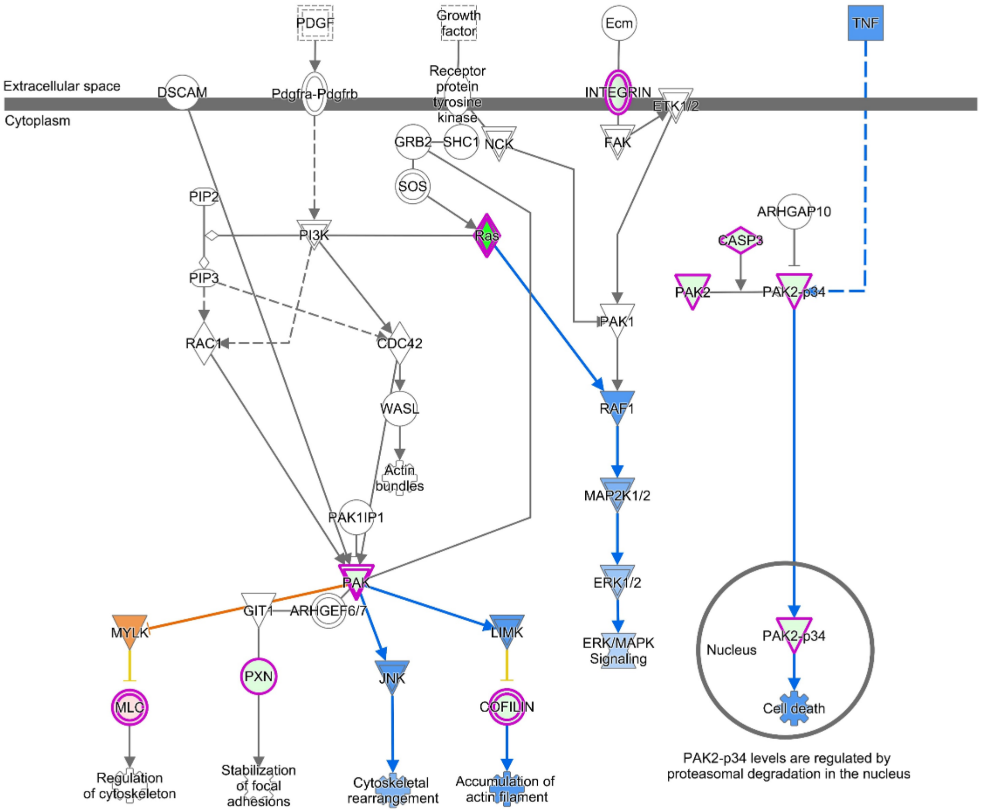

Portein Name	Gene Symbol	*p*-value	Fold Change (Plasmax/DMEM)	Log_2(Fold change) (Plasmax/DMEM)
Serine/threonine-protein kinase PAK 2	PAK2	3.E-03	0.62	-0.69
Paxillin	PXN	4.E-03	0.60	-0.74
Caspase-3	CASP3	2.E-02	0.62	-0.69
Cofilin-1	CFL1	2.E-02	0.78	-0.36

Figure S12. PAK signaling pathway of protein expressions from 231BR cell line cultivated in Plasmax *vs*. DMEM. Color codes are same as **Figure S3**.

Figure S13

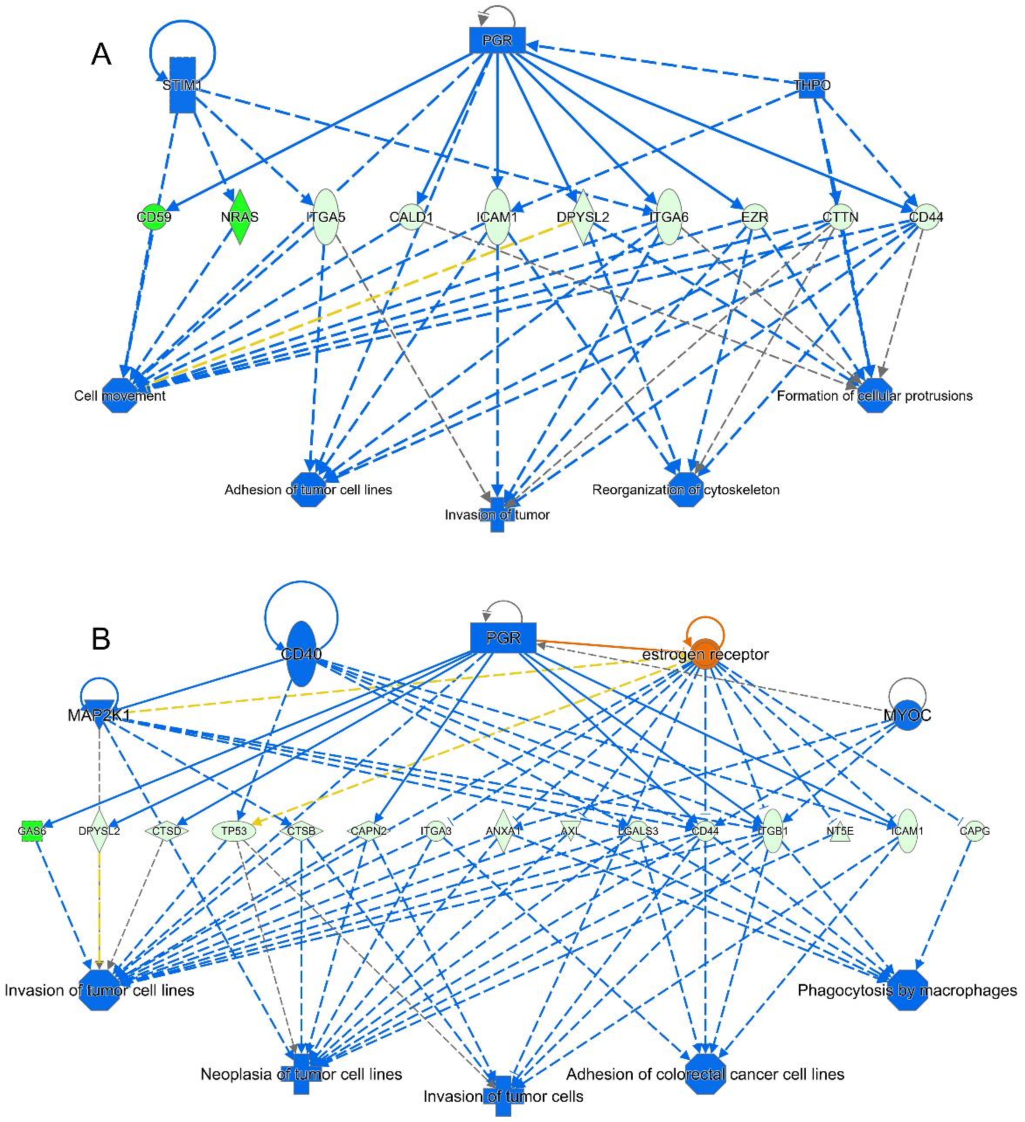

Figure S13. Regulator effects analysis of 231BR cell line. (A) Plasmax *vs.* DMEM; (B) Plasmax *vs.* EMEM.

Figure S14

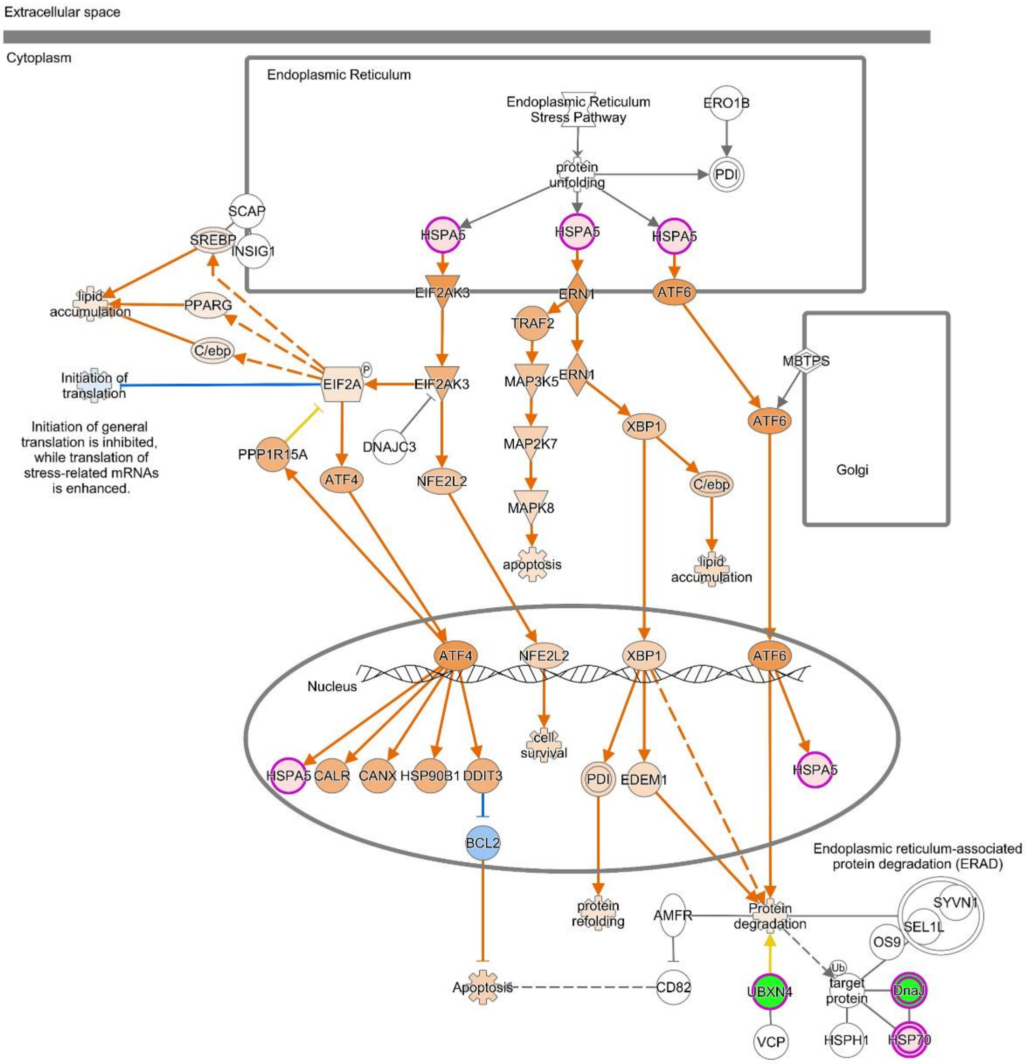

Portein Name	Gene Symbol	*p*-value	Fold Change (Plasmax/EMEM)	Log_2(Fold change) (Plasmax/EMEM)
Endoplasmic reticulum chaperone BiP	HSPA5	6.E-03	1.24	0.31
Heat shock 70 kDa protein 9	HSPA9	3.E-04	1.24	0.31
UBX domain-containing protein 4	UBXN4	3.E-03	0.00	-∞

Figure S14. Unfolded protein response signaling pathway of protein expressions from CRL cell line cultivated by Plasmax *vs*. EMEM. Color codes are same as **Figure S3**.

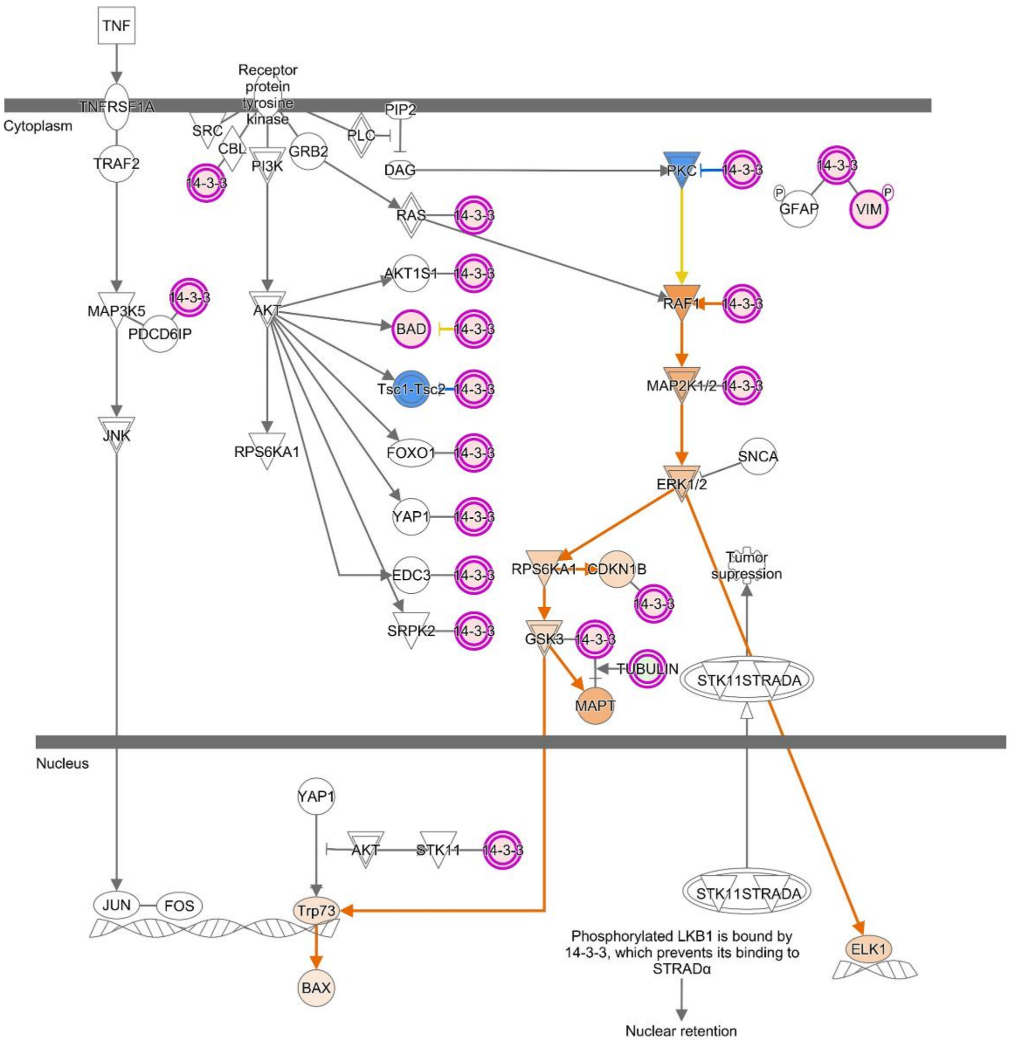

Portein Name	Gene Symbol	*p*-value	Fold Change (Plasmax/DMEM)	Log_2(Fold change) (Plasmax/DMEM)
14-3-3 protein β	YWHAB	3.E-02	1.25	0.32
14-3-3 protein ε	YWHAE	4.E-02	1.30	0.38
14-3-3 protein τ/θ	YWHAQ	3.E-03	1.24	0.30

Figure S15. 14-3-3 mediated signaling pathway of protein expressions from CRL cell line cultivated by Plasmax *vs*. DMEM. Color codes are same as **Figure S3**.

Figure S16

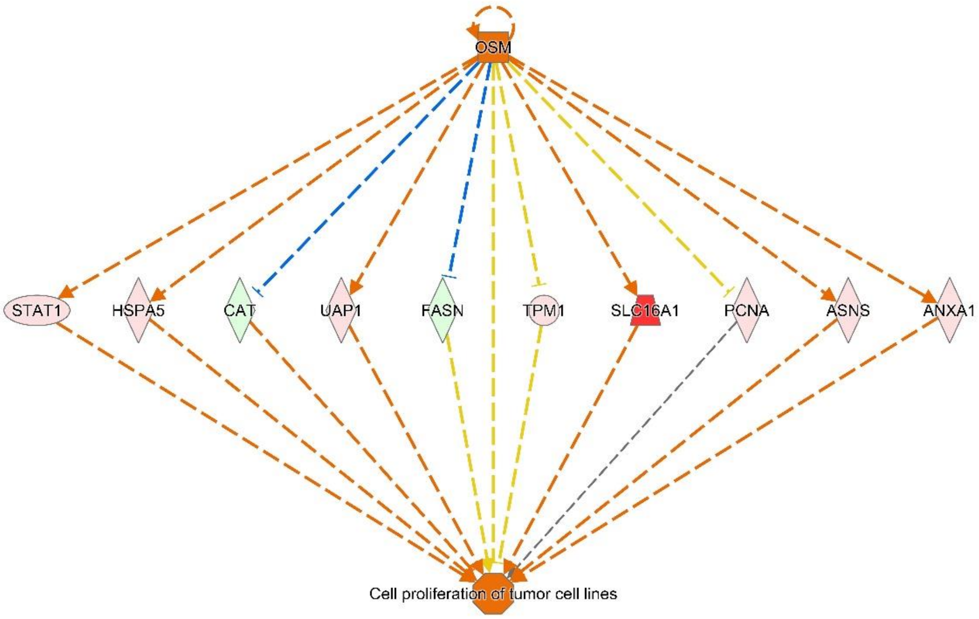

Figure S16. Regulator effects analysis of CRL cell line.

Table S2A. RPKM of identified transcriptomes from MDA-MB-231BR cell line.

Gene	231BR_D1	231BR_D2	231BR_D3	231BR_E1	231BR_E2	231BR_E3	231BR_P1	231BR_P2	231BR_P3
RN7SL1	162188.7	58813.6	88466.1	6549.6	18279.9	23458.1	123924.7	9405.7	61611.1
RNA45S5	29826.5	57311.0	50317.6	71049.3	66840.0	63797.2	43220.9	69823.4	51122.8
RN7SL2	107361.1	42996.2	61857.6	5344.3	13235.9	16755.9	71291.2	7448.8	41265.3
RN7SK	90185.3	45204.1	60268.4	12093.8	21811.0	31465.4	48333.1	9962.0	43052.3
RMRP	32879.5	15731.9	21735.5	3350.4	6487.5	8572.1	16127.4	3427.3	17051.5
RPPH1	18701.4	8259.2	12738.4	1537.3	4091.0	5380.7	15163.1	2180.1	13011.5
RNR1	2942.8	4955.1	9268.9	1444.9	2031.8	5929.3	2610.1	3149.5	2196.6
SNORD3A	9740.3	2303.8	3048.7	612.6	1288.7	1573.2	8541.3	533.3	3619.9
EEF1A1	6213.9	2402.0	3456.9	273.9	875.0	1362.0	7242.1	774.9	4667.1
RNR2	1855.8	2798.4	5325.1	653.2	1013.9	1907.7	1903.3	1734.8	1365.0
RNU105A	4670.1	1796.8	2333.1	420.2	845.1	1002.3	4181.8	586.5	2443.0
SNORA73A	3795.9	1341.5	1893.0	196.1	475.5	630.4	3517.9	368.0	1853.9
RNU4-1	4769.6	1634.2	1890.2	173.9	438.9	407.7	3183.2	219.7	1262.9
RNU2-2P	3747.6	1484.0	2234.1	65.5	403.8	384.4	3001.5	193.8	1252.8
RNU4-2	3987.8	1502.5	1809.5	167.2	462.3	395.4	2656.3	192.2	1041.0
COX1	2805.4	1350.5	1772.0	195.2	593.8	883.8	1783.6	369.4	2234.1
COX2	2451.4	1333.1	1674.9	229.4	567.9	920.1	1382.8	351.4	1915.0
MT2A	2476.0	869.1	1242.8	235.6	472.7	658.6	2179.5	304.4	1458.8
RNU1-28P	1645.1	1329.8	1456.0	185.9	548.4	573.4	2216.3	266.6	1322.4
RNU1-27P	1645.1	1329.8	1456.0	185.9	548.4	573.4	2216.3	266.6	1322.4
RNVU1-18	1645.1	1329.8	1456.0	185.9	548.4	573.4	2216.3	266.6	1322.4
RNU1-2	1645.1	1329.8	1456.0	185.9	548.4	573.4	2216.3	266.6	1322.4
RNU1-4	1645.1	1329.8	1456.0	185.9	548.4	573.4	2216.3	266.6	1322.4
RNU1-3	1645.1	1329.8	1456.0	185.9	548.4	573.4	2216.3	266.6	1322.4
RNU1-1	1645.1	1329.8	1456.0	185.9	548.4	573.4	2216.3	266.6	1322.4
RNVU1-9	1636.9	1328.2	1452.1	185.8	546.7	570.4	2204.2	266.5	1319.5
RNVU1-7	1636.9	1328.2	1452.1	185.8	546.7	570.4	2204.2	266.5	1319.5
RPL13A	1973.4	755.8	1189.7	145.5	360.8	476.0	2230.2	271.6	1356.3
ATP6	1942.3	1059.7	1346.5	148.8	443.4	684.7	1160.5	272.4	1627.2
RPL13	1985.5	779.4	1038.2	291.0	463.9	645.4	1818.2	287.3	1221.7
RPL37	1900.5	739.8	1077.3	164.6	351.0	485.6	1834.9	345.7	1564.6
RPL18A	2151.9	816.9	1209.1	255.1	427.7	609.7	1302.5	330.4	1328.9
ND1	1737.4	875.5	1165.2	130.7	397.0	606.0	1106.6	296.2	1650.1
RPL8	1856.2	742.1	1045.6	212.2	391.2	534.1	1536.4	278.4	1367.7
RPLP0	1633.7	574.7	871.6	107.7	246.4	356.9	1848.5	233.0	1210.7
RPS16	1654.3	615.4	1040.3	152.8	291.7	400.5	1517.4	221.4	1076.0
RPL28	1735.7	645.8	889.3	260.5	372.8	564.8	1136.0	297.8	1052.1
ND4	1524.4	785.0	1025.1	121.5	359.2	551.2	924.6	243.5	1404.4
ND4L	1470.7	729.2	893.4	112.5	343.8	542.8	969.1	219.6	1279.4
ND2	1390.1	752.9	948.5	87.6	285.6	436.8	910.3	234.2	1395.4
ACTB	1628.1	580.3	733.3	202.4	332.5	470.4	1110.2	194.4	829.5
SCARNA2	1609.0	423.0	774.4	113.9	220.4	266.3	1726.2	118.5	630.5
RPL37A	1417.0	579.8	807.9	109.6	245.6	367.1	1155.7	205.0	951.3
RPS11	1236.7	528.5	805.6	122.1	270.1	389.0	1034.2	221.0	1027.4
COX3	1217.6	652.1	862.5	121.1	326.9	528.6	647.9	186.0	1079.7
RPS18	1175.7	528.5	851.8	88.6	244.1	294.4	1121.3	180.6	1040.6
GNB2L1	1263.5	461.7	640.7	108.7	224.2	322.6	1198.2	194.2	842.1
RPS3	1263.7	487.5	730.9	71.9	188.9	261.8	1112.4	149.5	776.3
RPS27	1184.1	523.3	732.3	74.9	217.3	307.7	761.4	178.3	922.5
RPLP1	1019.6	370.4	546.4	84.3	178.0	235.6	1321.6	162.2	820.6
RPS28	1136.8	495.4	697.6	200.5	302.7	413.4	545.4	179.0	692.0
ATP8	980.8	606.7	729.6	58.0	217.8	330.6	573.0	132.3	885.9

RPS19	1078.2	413.6	588.4	78.1	183.5	252.3	1064.2	145.6	692.4
HMGA1	874.7	358.7	426.4	159.1	267.5	377.6	853.4	204.8	944.5
RPL23A	1195.0	451.0	621.3	100.4	209.8	313.1	726.2	148.9	675.3
RPS2	1022.0	410.8	513.0	122.0	215.2	302.9	955.2	172.1	711.9
RPL32	999.1	434.9	613.6	94.0	218.6	312.3	718.6	175.4	833.8
EEF1G	993.0	388.3	507.4	80.7	190.7	268.6	971.6	155.3	776.6
RPL19	953.5	398.1	574.2	88.1	206.4	282.3	934.5	159.1	724.0
RPS17L	902.4	428.5	632.6	133.7	220.0	306.9	630.0	206.3	803.0
RPL7A	940.6	355.9	533.3	60.9	163.9	216.7	883.0	117.8	640.4
CYTB	757.5	397.6	529.8	70.7	195.8	284.2	576.8	160.0	904.5
ACTG1	947.1	370.2	474.6	113.4	198.8	277.1	642.3	129.0	571.0
RPL26	793.7	310.7	491.3	40.9	142.2	195.6	824.0	131.4	756.4
RPL31	880.9	365.6	557.3	54.3	151.8	189.4	728.6	102.9	625.3
RNY1	867.5	545.2	457.8	161.3	192.2	241.7	445.2	160.7	569.3
RPS4X	840.2	339.4	467.3	46.4	149.0	205.8	736.0	124.3	609.9
RPL23	830.2	341.6	464.9	47.9	131.0	182.6	694.4	118.0	675.6
RPL27	819.8	359.8	550.8	87.1	162.9	241.6	502.1	146.7	604.1
TPT1	789.8	302.4	473.0	42.3	124.8	170.5	739.1	117.6	657.0
SNORA48	854.3	316.6	467.0	65.6	149.1	153.7	706.6	85.8	400.2
IGFBP4	1050.7	417.8	525.8	152.4	230.5	304.6	226.0	50.2	185.8
RPS23	741.1	283.7	399.7	31.0	122.1	165.0	773.2	83.7	515.7
RPL36A	770.8	313.3	432.8	48.6	126.1	170.5	580.5	104.3	562.8
ND3	724.3	401.2	489.7	25.6	135.0	200.8	428.5	83.5	583.1
CYR61	628.3	267.9	332.5	67.2	136.2	197.8	642.2	117.0	571.3
ND5	670.4	332.4	454.4	53.7	166.8	249.7	390.9	87.0	550.2
SNORA63	752.3	299.8	397.5	19.0	84.5	87.4	850.4	49.4	411.2
RPL36	696.3	292.5	379.2	95.1	161.1	209.4	521.6	112.0	436.0
RPS5	654.6	249.2	399.5	69.9	129.1	182.7	638.3	103.2	450.7
VIM	567.2	213.8	262.6	102.6	145.4	200.0	706.7	134.5	519.1
RPS29	730.2	307.8	444.1	70.2	120.9	179.6	504.3	92.2	399.5
RPS14	642.9	220.6	373.0	45.6	136.6	180.5	706.0	75.8	433.6
RPL18	668.0	273.4	391.2	75.5	136.7	191.8	527.9	101.2	425.5
RPS25	676.1	295.0	431.2	35.7	122.6	171.3	490.7	81.5	472.5
RPL35	636.5	232.6	343.7	62.7	129.3	171.0	605.3	103.8	473.7
TERC	752.0	331.1	527.9	43.3	105.5	135.0	481.8	61.7	316.7
UBA52	729.8	269.6	370.6	59.3	131.6	201.8	496.4	87.3	391.7
GAPDH	553.2	224.5	283.3	62.9	136.1	192.0	618.6	120.0	517.7
SNORA71A	725.1	244.4	397.3	33.1	69.3	58.9	863.0	51.9	262.9
RPL3	593.8	225.2	298.4	51.0	122.4	167.8	652.2	92.6	489.7
PTMA	626.7	208.2	310.9	55.8	112.6	140.8	685.5	92.5	432.1
RPL38	569.1	263.7	364.2	36.8	116.9	164.0	424.6	103.5	570.1
RPLP2	652.9	234.9	345.6	60.6	113.0	153.7	559.4	83.0	368.7
RPL27A	637.4	282.7	403.8	63.4	130.5	159.9	407.7	79.3	388.7
RPL6	529.4	208.0	289.7	25.6	103.6	142.9	636.8	83.7	529.0
S100A6	720.9	338.0	499.7	48.0	124.0	150.5	318.3	50.6	265.6
PKM	624.9	244.4	325.8	71.2	143.0	192.3	453.5	79.2	364.7
RPL15	724.8	230.6	346.7	45.3	95.6	147.1	521.8	67.7	311.5
RPL30	592.1	243.2	338.9	27.3	97.2	130.5	534.3	81.1	416.9
TMSB4X	821.0	268.4	408.7	20.3	76.7	95.8	502.4	35.0	217.2
RPS7	555.0	235.7	359.5	32.0	99.7	139.1	514.1	73.5	427.6
RPL4	624.2	229.9	298.4	35.1	100.4	138.3	532.3	76.4	380.8
RPS21	579.4	209.7	366.1	51.9	107.4	136.3	487.8	69.9	384.5
RPL12	589.1	221.9	316.9	46.6	96.6	145.7	468.1	87.1	413.7
RPS15	552.5	242.8	326.7	67.8	129.9	172.5	424.5	91.1	373.7
RPL10	504.3	183.6	259.9	34.2	97.8	131.6	601.2	83.9	463.7

RPL29	564.0	239.0	357.9	50.9	113.7	154.3	361.1	85.2	414.9
TMSB10	616.0	261.9	381.2	31.5	104.5	127.8	396.5	68.3	318.5
HSPA8	550.9	214.1	262.6	23.7	77.0	113.6	506.8	81.1	436.3
PFN1	534.1	211.1	258.6	58.9	111.0	156.3	480.1	81.0	362.9
RPS12	493.4	172.3	285.0	22.3	73.1	94.0	631.2	65.5	410.6
RPS8	483.8	194.8	286.5	38.1	90.6	128.6	474.7	74.7	385.7
ND6	491.7	238.6	347.6	35.2	105.3	151.2	311.4	60.5	408.3
RPS17	451.2	214.2	316.3	66.9	110.0	153.4	315.0	103.2	401.5
NPM1	472.0	181.8	253.1	13.1	67.7	91.2	588.1	61.9	401.1
RPL17	501.1	202.9	287.5	27.3	84.7	116.9	421.8	65.9	380.1
ENO1	508.0	184.0	251.9	30.1	87.0	106.7	507.5	61.6	335.2
UBB	509.4	205.3	291.8	45.4	99.3	131.4	430.5	64.0	294.1
RPS24	456.9	197.7	271.5	29.4	91.8	137.3	377.2	70.6	416.5
PABPC1	494.8	182.9	226.8	40.0	89.6	136.0	408.8	76.5	383.6
FTH1	616.8	235.3	289.1	47.0	103.6	156.8	279.2	51.9	233.0
RPS3A	459.1	170.6	276.7	11.4	57.9	82.7	549.1	49.1	355.8
EEF2	494.5	184.5	225.6	60.3	100.1	136.2	394.6	79.0	324.6
RPL5	445.8	179.0	265.6	15.2	62.7	94.2	479.7	53.4	338.2
RNY3	515.7	280.0	289.4	51.8	88.5	129.3	225.4	48.1	267.6
RPS6	407.3	164.7	252.8	21.6	70.3	95.0	471.9	58.9	340.7
AKR1B1	720.6	270.1	393.4	24.7	52.1	69.1	182.5	30.3	115.5
RPL24	387.5	161.6	245.4	21.0	70.9	96.3	462.8	55.9	337.1
RPL10A	409.4	154.1	228.4	33.5	87.8	114.7	436.6	52.8	292.9
RPS27A	400.6	155.6	243.4	12.7	56.1	69.9	505.7	41.3	282.1
FAU	416.3	160.9	245.2	33.0	79.5	103.6	369.5	55.5	289.1
RPS10	248.0	162.9	269.2	35.3	32.3	121.4	563.4	15.7	299.0
MYC	334.0	128.4	155.2	51.3	84.7	122.2	362.5	84.0	398.5
RPL14	441.0	193.2	261.8	26.4	73.0	108.3	263.1	54.8	282.7
CALR	348.3	131.6	174.9	26.6	61.3	83.7	435.1	65.4	299.6
CFL1	429.0	156.0	205.9	38.1	75.8	101.0	316.4	51.3	236.4
C17orf76-AS1	383.3	168.7	242.9	21.8	64.3	86.4	323.6	44.4	256.1
IFITM3	502.1	195.4	245.3	42.2	72.0	100.7	214.7	44.5	171.2
SNORA57	352.1	154.2	233.9	59.1	86.1	94.1	356.9	49.0	187.8
RTN4	388.3	123.9	170.3	46.6	68.3	90.4	375.4	52.5	211.8
TUBB	385.2	142.1	209.4	45.8	90.8	124.1	244.3	45.5	223.2
RPL11	368.2	163.9	207.5	26.9	78.4	96.6	247.2	51.8	260.9
SNORD13	547.6	99.4	132.3	46.6	63.8	64.4	303.2	37.2	198.5
FTL	393.7	167.1	204.5	36.3	92.0	133.3	225.9	43.7	192.3
RPL35A	353.8	142.7	198.1	15.9	56.6	83.5	306.9	48.2	279.3
APLP2	462.0	166.5	211.8	30.5	76.9	98.9	223.5	36.2	173.7
RPS15A	344.2	154.0	234.3	17.1	63.5	80.5	263.9	41.1	275.8
RPL7	331.8	125.8	181.6	10.5	52.3	67.9	383.9	39.7	273.0
PSAP	448.6	157.0	215.9	29.1	66.9	90.1	252.0	30.9	155.0
IER3	398.7	165.6	204.2	29.0	47.1	69.2	245.9	62.1	200.7
APP	426.1	136.1	194.4	26.5	58.9	81.8	258.6	38.9	187.4
RPSA	345.2	122.2	237.8	13.5	43.8	55.7	335.4	34.7	205.6
AXL	287.1	114.8	138.9	46.4	83.8	117.7	263.6	46.6	202.2
PPIA	305.8	125.3	177.7	10.1	43.0	61.6	313.8	34.0	222.4
RPL9	311.5	122.4	180.6	13.8	44.0	56.3	282.5	37.9	230.9
RPS9	299.7	123.3	169.3	25.7	54.4	74.5	267.2	43.1	205.9
RPS26	293.4	112.4	158.6	24.2	53.3	70.9	279.6	45.7	213.1
FLNA	337.5	115.6	147.3	41.9	71.5	93.1	199.9	35.3	155.7
COX6A1	270.5	116.9	173.4	28.9	59.8	77.0	237.9	45.3	168.4
GNAS	307.9	107.9	136.5	28.3	63.2	84.2	222.6	38.7	182.4
ANXA2	250.7	101.2	132.2	11.4	40.3	56.7	299.1	38.8	237.7

PTTG1IP	363.9	124.4	171.0	29.1	55.0	85.1	179.4	23.9	106.4
UBC	294.8	105.4	139.0	18.3	47.4	62.6	244.1	34.6	186.6
RPS13	283.8	110.3	154.0	13.8	43.8	57.9	256.1	36.4	175.2
HIST2H2BE	262.5	120.5	163.7	59.2	99.3	134.5	95.7	37.0	150.6
MIF	248.7	128.0	165.2	59.1	77.6	103.9	127.7	43.4	160.2
ZFP36L1	263.2	98.7	123.4	41.3	57.6	86.3	204.7	48.2	180.3
SNORD33	291.7	84.1	96.2	64.5	86.4	87.0	149.8	30.4	208.4
BSG	331.2	126.5	167.3	35.6	66.5	89.4	134.8	30.4	110.6
HPCAL1	256.5	108.5	135.1	72.9	116.7	161.4	86.6	24.8	101.5
MYH9	317.4	101.8	134.7	38.9	68.2	82.2	155.6	24.3	111.7
PLEC	293.8	92.3	129.8	45.1	63.9	75.5	186.1	27.0	120.7
RPS20	238.7	94.4	138.7	11.1	37.2	48.9	251.8	29.0	174.9
ANKRD1	223.4	89.5	119.3	13.7	44.1	60.9	242.4	31.6	186.0
ACTN4	290.2	110.1	147.2	42.6	67.7	84.5	134.7	27.6	105.9
CCL2	332.4	149.2	204.7	14.3	30.8	47.3	92.5	25.5	106.4
MSN	261.8	92.8	123.5	16.2	40.6	54.6	212.1	29.9	152.3
DUSP4	297.4	102.4	138.3	25.2	38.8	52.3	173.5	32.0	121.8
GRN	320.0	113.6	148.4	32.5	47.6	79.2	129.2	21.6	85.5
JUND	250.4	108.2	134.3	55.0	63.2	86.7	130.7	34.8	111.1
BTF3	251.8	98.1	131.3	13.6	43.9	60.1	187.6	28.5	155.9
RPL34	214.8	88.6	134.5	7.9	36.6	43.3	234.8	27.5	181.1
MALAT1	362.8	133.0	201.7	7.2	26.7	32.5	109.3	11.9	82.7
SNORD17	256.2	72.9	108.2	17.2	38.2	43.0	255.3	26.3	148.6
ACTN1	255.0	88.5	114.0	28.3	53.0	70.4	183.4	30.4	136.3
SCD	346.1	148.5	177.6	8.2	22.7	31.5	97.0	18.8	106.8
SQSTM1	291.6	116.1	143.3	25.0	50.3	67.2	140.3	21.0	89.3
PHLDA1	228.8	92.2	122.7	50.5	67.3	91.6	118.3	34.0	136.3
GAS5	214.1	73.0	120.1	7.5	25.1	34.1	261.2	24.9	181.2
SLC3A2	134.8	47.7	62.8	8.7	16.3	24.4	387.5	50.2	206.9
PCBP2	255.0	103.5	131.5	16.7	41.9	58.4	147.7	28.1	142.2
CHCHD2	234.0	91.3	131.5	20.6	41.0	57.8	201.1	26.2	120.9
ARHGDIA	238.3	90.3	111.9	23.8	47.3	62.4	179.6	28.5	139.6
RPL21	223.8	87.5	120.2	4.4	29.0	44.3	217.6	23.5	165.4
CANX	224.7	78.1	101.6	10.3	32.9	46.9	236.4	28.4	154.7
HSP90AB1	196.7	73.4	89.0	9.4	33.9	46.7	235.5	31.5	188.6
CTGF	125.5	49.0	63.2	17.1	30.6	41.3	308.2	48.5	218.2
RPL39	204.5	105.7	169.8	4.5	36.6	41.4	106.6	27.3	196.4
FKBP1A	244.1	88.8	116.4	13.3	43.4	60.3	162.8	25.1	133.7
TUBA1B	206.6	78.9	107.0	25.0	53.9	75.3	172.5	29.5	134.6
EEF1D	207.1	71.5	97.4	23.7	38.2	55.2	208.2	30.8	146.5
TACSTD2	302.6	129.1	161.9	29.4	43.0	55.3	77.1	18.3	56.3
OAZ1	225.1	89.9	127.2	14.7	33.0	46.5	171.9	23.0	138.6
SNORA71C	179.3	74.0	120.7	9.9	24.7	24.9	311.7	20.0	95.6
HSP90B1	191.8	73.4	88.7	8.1	27.2	39.5	229.5	29.6	171.4
EIF6	188.7	81.6	99.0	25.6	54.4	71.4	164.2	26.9	139.0
NCL	189.7	68.7	78.4	15.6	30.5	45.4	212.6	32.6	175.8
HIST1H2BK	179.1	68.7	96.4	40.8	66.2	92.8	145.2	28.6	128.6
SLC25A3	206.0	73.7	103.3	10.8	29.2	43.6	211.2	25.0	141.7
EEF1B2	186.7	79.2	110.9	11.5	36.0	49.4	177.1	27.0	160.9
CNBP	168.7	67.1	90.5	12.0	35.9	44.5	204.2	30.5	185.1
YBX1	182.6	81.3	95.4	23.3	48.6	66.7	137.3	36.1	166.4
PEBP1	214.5	89.2	113.7	20.6	41.3	58.3	148.8	27.0	117.1
EIF5A	192.9	77.4	97.3	13.5	42.0	53.1	175.5	24.1	154.0
SLC7A5	195.5	78.5	95.0	30.4	50.3	69.1	138.1	30.9	140.9
CAPN2	246.3	87.3	113.9	17.2	36.1	47.8	142.8	24.1	112.6

IGFBP7	257.5	92.7	131.3	31.1	43.6	61.1	119.4	16.4	64.5
TIMP3	223.4	83.1	105.9	20.9	43.2	57.1	155.9	25.9	100.5
ARF1	204.2	90.7	108.8	28.8	45.0	61.6	118.1	29.3	127.8
SNORD10	192.0	56.8	94.7	15.7	39.8	27.9	277.2	13.9	89.4
KRT18	209.9	79.5	99.6	12.3	29.2	37.9	176.8	27.4	131.6
RNU5A-1	250.3	145.9	129.6	9.7	30.5	32.2	130.9	12.3	61.3
ITGA3	177.0	61.9	81.3	32.0	56.9	78.8	160.1	25.7	127.0
EGFR	206.7	66.8	96.0	16.2	34.4	48.2	164.1	25.3	128.8
PCBP1	196.5	86.5	105.2	21.1	36.4	48.5	129.3	30.1	131.5
TIMP2	223.2	97.9	112.4	24.6	43.8	56.2	95.4	23.5	102.7
HLA-A	254.9	94.5	128.5	28.8	50.5	64.5	89.8	13.2	54.6
HIST1H1E	142.6	58.7	81.0	25.3	46.5	57.3	197.6	30.5	139.3
ATF4	202.0	73.3	90.9	7.0	25.6	33.7	191.0	24.0	129.7
GPX1	182.6	80.1	111.2	22.9	40.9	54.8	145.6	26.2	107.4
SCARNA12	192.5	73.3	120.1	11.8	33.6	31.6	172.7	17.8	110.6
ALDOA	197.7	76.9	102.2	24.3	42.2	58.8	130.6	23.3	103.6
PLAT	253.8	101.4	127.7	11.9	25.3	34.8	99.1	18.5	85.4
DAP	211.6	102.0	114.9	21.3	44.5	65.4	67.7	23.0	95.5
DYNLRB1	210.6	95.8	122.7	26.0	45.5	66.4	64.3	21.2	92.0
PTRF	202.0	72.1	90.9	26.7	40.8	54.5	138.2	24.9	93.4
P4HB	191.0	86.7	110.4	16.4	32.1	43.1	141.3	23.9	97.4
CCND1	180.2	61.8	78.1	31.8	49.3	65.3	138.3	26.5	110.7
EIF1	177.8	59.4	82.3	3.1	19.0	22.6	210.4	17.7	149.6
GPX4	206.6	79.8	111.4	22.4	42.1	58.1	117.3	19.1	80.8
RPL22	195.0	78.0	99.4	7.2	32.6	43.4	131.5	21.3	127.8
SNORA47	182.0	75.0	107.9	33.0	48.2	51.9	143.0	26.4	63.6
NPC2	214.1	88.0	121.3	13.6	36.0	55.6	98.7	20.5	80.4
MT1E	132.5	51.6	68.3	17.2	41.3	61.1	214.5	21.9	112.7
HSPA5	144.7	52.5	67.5	8.4	18.8	26.3	219.5	28.9	152.3
SOD2	295.6	108.6	151.3	11.6	16.9	25.4	51.5	12.3	41.0
TPM4	184.1	65.9	83.8	22.3	39.0	50.4	141.7	23.2	102.3
BCL2L1	195.8	70.6	90.6	13.8	30.8	36.9	139.3	23.0	110.5
HIST1H4K	152.1	64.2	90.3	30.9	54.7	76.8	114.7	20.2	98.3
HIST1H4J	152.1	64.2	90.3	30.9	54.7	76.8	114.7	20.2	98.3
FKBP8	212.6	80.2	103.9	19.8	36.3	47.2	107.1	16.1	75.8
SNRPD2	176.8	67.3	110.3	16.5	37.8	49.6	121.6	19.6	96.0
ATP5L	182.5	60.5	92.9	13.8	30.4	41.9	149.8	23.0	99.4
SNORD15B	210.0	50.4	79.7	8.8	29.0	27.2	192.1	8.2	85.5
YWHAE	180.0	71.8	96.5	10.7	29.7	43.3	122.5	19.4	107.7
HNRNPA1	171.1	67.2	83.5	8.3	26.1	35.1	153.9	20.1	110.3
HIST2H2AA4	127.5	50.1	77.8	32.6	54.5	70.7	129.6	23.2	101.9
HIST2H2AA3	127.5	50.1	77.8	32.6	54.5	70.7	129.6	23.2	101.9
B2M	207.9	91.1	122.7	11.1	39.4	49.3	70.2	12.8	60.1
ATP1A1	184.1	67.8	86.5	10.9	27.2	36.0	131.8	19.0	99.6
NME1-NME2	142.3	67.9	95.2	19.0	34.5	49.9	110.6	25.0	116.0
LGALS3BP	198.8	78.5	98.3	20.9	39.2	52.4	89.6	15.1	66.7
PRKCSH	179.9	66.4	85.2	22.7	37.9	51.2	102.2	23.1	90.9
EIF4G1	179.9	63.7	80.0	14.4	30.2	40.8	126.4	20.9	102.8
MT1X	173.4	71.3	98.9	19.7	37.1	58.1	103.8	16.7	77.3
EXT1	193.9	71.0	90.2	10.3	30.1	38.8	104.6	17.9	98.5
HIST2H2AC	142.8	65.4	93.4	28.9	52.7	68.9	83.4	21.4	93.2
SCARNA7	170.8	70.5	102.8	4.6	19.5	34.1	136.4	14.4	93.2
EIF4B	161.5	60.7	75.4	8.6	25.5	34.1	146.7	21.7	106.6
RNU12	206.0	80.1	63.6	21.0	38.7	42.1	88.1	17.1	81.7
EIF3G	166.5	64.3	85.2	19.8	32.9	48.5	97.4	24.1	97.8

NAP1L1	157.7	56.2	73.3	4.8	18.5	24.9	164.8	18.2	111.2
CST3	207.1	87.0	116.1	24.5	40.2	48.1	56.5	9.5	39.9
FOSL1	108.3	44.9	56.1	32.0	49.6	73.2	112.3	28.6	122.6
RPL36AL	162.6	61.7	80.0	7.7	22.6	35.6	141.7	18.8	91.9
CRIM1	153.1	50.0	63.8	10.8	22.8	29.7	164.6	22.3	105.2
SURF4	179.4	60.9	77.5	11.2	24.3	28.9	127.0	17.5	93.8
FASN	218.4	77.3	101.0	15.4	23.8	30.8	79.8	12.8	56.1
H3F3B	154.0	60.3	76.4	14.1	27.4	35.5	125.4	21.7	100.4
HIST1H1C	147.1	60.0	92.4	10.4	34.8	41.7	122.6	17.1	88.9
ZFAS1	119.8	50.8	73.8	4.6	17.7	24.9	155.5	24.9	141.0
QSOX1	166.7	63.5	83.5	20.3	35.8	50.3	106.7	15.0	70.8
PRMT1	141.4	53.5	73.8	18.5	32.4	43.8	118.9	22.9	99.9
HNRNPC	148.3	60.3	78.3	6.4	23.8	33.1	123.0	19.0	106.7
TPI1	153.2	64.1	78.7	11.4	27.4	36.7	123.4	17.1	86.1
EMP3	145.7	65.6	79.6	14.8	29.4	45.3	98.4	22.4	96.6
C19orf43	154.1	58.0	82.4	18.0	30.0	40.4	102.9	22.9	88.6
DUSP1	128.9	57.2	83.9	16.1	28.1	45.4	120.1	20.7	95.1
ERP29	147.6	66.4	82.4	10.9	26.8	35.7	92.2	22.5	108.1
SET	132.6	50.4	63.2	7.8	21.2	30.8	147.9	21.2	116.2
HDGF	165.2	62.4	75.4	12.8	25.8	36.5	114.0	18.0	81.0
EDF1	133.8	60.6	70.0	19.2	36.8	47.3	97.7	22.5	101.9
KIFC3	159.5	56.0	71.4	18.6	33.8	43.9	114.6	15.7	74.6
GLTSCR2	147.2	54.6	69.2	20.8	33.5	39.0	117.8	20.9	83.5
CDC37	139.2	54.1	67.9	21.3	34.4	48.3	108.4	20.6	91.8
PPP1R14B	128.7	48.0	66.3	19.2	37.6	49.7	119.2	22.7	91.9
TNFRSF12A	137.8	52.0	65.2	23.7	37.7	50.1	126.6	18.1	63.2
SLC1A5	114.7	45.2	56.1	19.0	31.0	46.0	135.2	21.2	104.0
NONO	150.0	54.1	70.0	11.7	28.8	36.8	116.1	16.5	86.1
PRNP	139.3	58.9	75.3	17.3	32.6	46.1	83.6	19.4	97.0
PTX3	160.5	57.2	82.3	6.8	13.7	18.1	132.0	14.5	83.0
HYOU1	112.9	40.8	52.9	9.6	22.3	30.4	157.7	22.7	113.2
CAV1	136.8	58.1	70.8	14.5	28.9	40.2	99.2	22.9	89.7
CEBPB	143.9	57.0	67.2	25.8	29.9	38.0	101.6	20.6	75.4
HIST2H4B	121.6	42.8	66.5	19.5	39.9	48.3	129.9	14.6	75.9
HIST2H4A	121.6	42.8	66.5	19.5	39.9	48.3	129.9	14.6	75.9
HLA-B	179.0	66.8	93.1	22.2	39.0	50.6	58.7	9.3	40.1
SCARNA21	141.4	65.3	96.9	9.6	20.7	29.6	84.2	18.0	90.5
NACA	137.2	60.8	84.6	7.5	24.5	33.0	86.4	17.5	104.3
RNU5B-1	138.7	105.6	66.3	7.2	23.2	27.4	113.5	10.4	62.7
LASP1	139.9	57.0	68.0	16.0	30.4	41.9	89.3	21.2	89.0
SNORA10	108.6	70.8	68.5	35.6	52.7	40.5	74.3	27.2	73.5
COLGALT1	154.9	60.1	75.8	16.9	27.6	39.0	86.2	14.9	72.5
EIF3H	134.1	57.1	73.0	10.4	25.4	36.7	88.3	18.7	103.2
RNA5S9	2.6	169.4	128.2	126.8	1.0	0.6	38.5	55.8	21.5
MRFAP1	154.9	52.7	70.0	17.4	28.3	33.0	101.3	13.0	70.4
SNHG5	109.3	38.7	64.6	4.7	15.7	21.0	165.4	15.4	105.3
PPIB	128.8	51.9	70.7	8.8	23.0	30.8	116.7	15.3	93.0
LOC101559451	122.6	48.5	70.6	19.0	26.0	31.1	118.5	23.4	76.0
TRMT112	142.9	59.9	80.3	11.2	24.7	28.6	100.1	14.3	72.8
PLAU	148.8	63.8	78.4	10.0	29.4	36.0	84.5	14.8	68.9
COX4I1	142.7	55.4	77.3	8.8	22.4	29.4	112.0	14.6	71.6
LDHB	123.6	44.6	60.8	3.8	18.3	25.1	151.6	12.7	91.8
HIST1H3C	97.0	44.3	63.5	22.2	47.3	63.7	92.6	20.9	76.2
PEG10	196.7	76.2	88.1	11.5	27.5	33.2	46.0	8.6	39.8
PRSS23	161.0	61.5	80.2	13.2	29.5	40.4	74.9	10.6	55.0

C6orf48	127.3	46.2	66.1	5.8	20.8	24.1	110.4	19.2	106.4
LDLR	177.5	67.3	88.4	11.3	21.8	30.0	64.3	10.3	53.2
MAF1	128.3	57.1	67.7	12.7	24.8	36.0	88.3	18.7	89.9
LGALS1	123.2	57.5	72.8	18.7	33.4	46.9	82.6	16.9	70.6
YWHAB	137.0	54.2	66.9	6.6	21.9	31.2	100.0	14.4	90.1
HSPB1	152.9	67.2	79.5	20.8	29.2	37.7	67.1	15.9	51.9
U2AF2	131.1	50.3	64.5	13.6	22.6	31.0	98.6	20.3	88.3
RNU5E-1	171.3	93.4	87.1	5.6	25.8	27.9	62.6	6.4	39.9
SENP3-EIF4A1	134.9	53.3	70.4	7.2	20.7	28.5	108.6	15.7	79.9
WDR83OS	153.2	52.7	75.4	12.1	23.0	32.7	97.4	12.8	59.8
RAB11B	137.6	54.7	68.6	20.6	33.8	42.7	70.5	18.6	70.2
HLA-C	159.4	64.1	86.3	21.8	35.2	46.1	52.4	10.0	42.2
RAN	128.8	48.1	66.2	5.4	19.7	29.7	122.8	14.1	82.5
POLR2L	129.8	51.7	77.6	22.3	28.6	43.8	81.8	16.7	63.1
TMED10	151.6	54.5	75.6	7.3	20.0	26.8	97.8	12.5	68.5
GAS6	134.2	45.6	62.9	25.5	42.9	54.2	82.4	13.3	53.2
CITED2	108.5	52.4	63.9	18.1	27.8	35.9	75.2	27.1	94.7
AHCY	129.8	51.8	73.6	11.9	23.0	31.5	95.4	14.0	71.1
CLIC1	125.6	48.3	65.1	8.6	24.6	34.6	103.1	14.7	77.5
EPHA2	140.5	52.6	69.3	13.1	22.9	30.4	92.3	15.1	65.6
PEA15	111.7	49.1	56.3	11.1	27.1	38.4	83.6	21.0	103.2
FADS2	167.1	69.8	82.0	7.5	16.4	22.7	62.7	13.4	59.2
FBL	102.5	41.5	51.9	9.9	21.7	33.0	121.3	21.0	97.3
HSF1	125.4	49.7	63.0	13.4	21.4	30.1	93.3	19.4	84.2
SEC61A1	121.0	48.6	58.3	7.8	20.2	27.4	101.2	19.0	96.3
HM13	124.1	53.0	63.4	12.7	23.9	35.3	84.0	19.1	82.3
SNORA67	124.0	66.7	76.0	6.6	22.0	17.7	130.6	7.8	43.7
TRIM28	116.5	42.0	53.1	12.7	23.7	32.2	115.7	18.6	77.6
TNIP1	155.6	63.4	77.4	13.0	21.2	30.0	65.8	12.9	52.1
UGDH-AS1	165.0	32.6	76.2	4.0	19.9	22.5	102.6	7.3	61.3
MTCH1	136.6	52.2	71.1	11.4	22.4	31.2	94.6	12.4	57.8
SPINT2	142.2	57.9	73.6	12.1	23.1	32.8	80.5	10.9	56.0
CTNNA1	143.3	50.0	64.2	9.7	22.2	30.2	88.0	13.7	66.4
TMED9	132.6	48.4	64.0	10.0	20.5	26.6	101.1	13.7	67.6
EIF4H	122.4	51.1	60.1	10.7	25.1	37.7	79.6	15.6	82.2
PKN1	136.0	51.8	66.0	13.9	24.6	33.9	86.0	13.5	57.8
ERH	123.6	58.2	71.6	5.8	23.8	34.7	64.9	14.8	81.3
S100A11	126.2	58.7	68.8	5.9	24.5	35.7	74.7	13.6	70.3
H1F0	119.7	47.9	58.6	10.9	21.5	27.3	74.6	21.5	96.3
SNRPB	108.6	42.7	58.2	5.9	15.7	21.1	130.2	14.7	80.4
TRIP10	132.8	50.1	65.4	11.0	24.5	31.3	82.1	13.9	65.0
MCL1	109.0	38.7	54.7	14.0	22.7	30.9	112.3	16.9	74.8
RPN2	129.9	45.9	60.3	9.4	20.6	30.3	91.5	13.0	73.1
ATP1B1	149.2	52.3	69.7	3.4	10.3	15.3	97.1	12.4	63.7
CD59	107.0	52.9	73.1	11.7	29.9	41.3	73.9	11.9	71.1
PTBP1	125.0	46.7	57.0	14.2	26.6	36.1	77.2	16.2	73.3
SARS	119.9	43.2	60.1	7.2	21.1	27.9	105.8	13.6	73.4
DDIT4	105.0	39.4	54.9	10.3	19.9	26.9	122.8	14.4	78.0
AHNAK	150.9	40.5	59.1	4.7	18.0	28.7	116.9	7.7	42.2
SPTBN1	138.4	46.1	61.1	9.8	21.9	26.6	85.2	12.9	65.7
EIF3F	118.6	44.1	58.4	13.6	21.8	31.5	90.4	17.6	71.9
PHLDA2	118.3	47.8	59.7	24.9	28.7	41.3	66.1	16.2	62.9
TRIM8	140.3	50.4	63.5	15.3	22.3	29.9	75.0	13.3	55.6
HIST2H2BF	85.2	36.7	53.3	25.9	37.8	54.4	66.0	21.0	85.0
GRINA	136.4	59.3	69.7	13.6	27.0	38.0	54.6	11.7	54.6

ERGIC3	125.2	51.5	71.6	8.3	21.5	28.6	81.9	13.0	62.3
MGAT4B	158.8	53.4	73.5	14.6	25.6	34.6	56.2	8.1	38.4
SHMT2	111.8	49.6	59.2	9.6	22.7	32.0	88.1	15.5	73.3
CAPG	135.5	54.6	71.6	12.9	29.5	37.1	62.9	9.0	45.6
RCN1	123.5	49.5	62.3	8.7	20.2	27.9	86.2	12.1	67.7
ANXA5	111.4	43.1	57.1	3.4	16.9	21.9	104.0	13.5	82.5
PGK1	116.7	47.8	66.2	4.9	17.6	22.8	96.3	12.1	68.0
SNORA7A	119.5	36.2	59.1	7.9	17.0	18.7	136.4	11.8	45.5
TMED2	121.3	48.0	68.9	6.8	19.2	25.9	80.7	12.9	67.4
PTMS	136.5	41.4	66.6	8.6	18.4	25.1	102.3	11.1	41.0
CYBA	121.8	48.7	71.0	20.0	34.7	45.0	60.3	8.8	39.6
TMBIM6	114.0	45.3	54.6	4.2	15.3	20.9	108.3	13.5	73.5
CSF1	191.2	75.3	94.1	6.7	17.8	24.2	18.2	3.2	17.5
PDIA6	108.2	40.5	52.6	4.6	14.1	18.6	116.4	13.3	79.5
SNORA71D	87.0	43.0	52.2	18.6	28.1	18.4	124.5	11.0	64.6
RAD23A	121.4	43.2	56.1	15.6	24.5	35.8	74.8	13.7	61.4
TUBB4B	110.3	46.2	60.7	14.7	27.5	39.4	70.7	14.9	61.9
CYC1	111.1	47.4	63.9	13.6	24.0	36.1	77.2	12.7	60.0
ERGIC1	106.9	47.0	59.5	16.5	27.4	38.6	54.7	17.2	77.5
PHB	111.6	42.9	59.5	9.5	20.1	27.2	89.5	13.9	68.7
EIF4EBP1	94.3	43.4	48.1	12.2	27.5	34.0	88.7	18.5	75.8
TUBA1C	116.3	42.5	58.0	10.1	25.3	34.0	82.6	11.3	62.3
FAM96B	114.8	52.8	67.1	14.9	24.4	31.9	58.7	13.3	64.4
MBOAT7	120.7	50.7	68.8	15.8	23.4	34.5	64.1	11.4	52.9
SSR3	94.3	41.4	56.8	7.0	21.3	28.9	81.0	15.6	94.4
SRSF9	112.3	38.5	51.6	11.7	19.0	23.5	105.6	15.5	62.5
OST4	122.8	52.2	69.6	15.8	26.5	32.1	59.2	12.6	48.5
CPNE1	127.6	47.6	63.7	8.7	21.3	29.2	71.8	10.7	55.1
PGAM1	114.7	40.7	56.6	8.0	17.7	24.1	101.1	12.1	59.8
ODC1	75.5	27.6	34.8	4.1	14.7	20.4	142.2	14.4	99.4
TM4SF1	135.2	52.6	71.0	5.3	18.6	24.0	74.2	7.6	44.6
CHMP4B	124.7	46.3	57.2	10.4	19.7	30.2	70.0	13.2	57.8
G0S2	100.6	38.0	52.6	9.9	14.8	20.7	114.5	15.0	62.9
ESRG	136.0	45.4	66.4	7.3	16.8	21.8	66.7	11.2	57.2
TECR	113.8	48.7	58.1	11.7	22.4	32.1	59.9	14.8	67.0
CCT7	82.3	34.4	49.2	6.5	18.0	23.9	112.2	16.0	85.7
MMP14	133.0	48.7	63.0	13.7	26.0	34.7	56.8	9.8	42.1
CCDC124	93.3	37.9	46.5	17.9	26.9	36.0	78.2	17.3	73.0
CDR1	110.7	39.3	50.7	4.0	16.0	23.3	87.9	13.2	81.7
IER2	103.1	36.3	50.6	18.0	20.9	31.2	86.3	17.1	61.9
HIST2H3D	78.0	28.9	48.2	22.6	38.1	48.8	91.1	12.5	56.6
TCEB2	112.8	43.8	61.6	14.3	26.1	35.2	70.8	11.3	48.5
TXNIP	230.3	81.1	99.7	0.6	2.1	2.7	3.6	0.4	2.9
MRPS24	104.1	42.3	55.3	16.7	24.9	31.8	80.1	15.8	50.9
FAM129B	115.8	42.7	54.0	12.5	20.7	29.0	77.8	12.8	56.5
RPL41	98.1	26.9	45.4	9.6	30.0	20.7	112.8	15.5	61.1
PXDN	116.8	43.1	55.7	15.1	25.6	33.1	61.4	12.5	55.6
YBX3	99.6	37.0	48.5	13.8	20.8	28.2	87.9	15.6	67.5
DNAJB6	118.8	43.5	61.5	9.8	18.8	24.6	73.1	11.3	57.5
SNHG7	105.6	38.7	53.9	7.4	14.8	17.6	108.2	13.0	59.0
BRI3	116.1	50.6	64.9	15.8	23.7	35.1	50.3	12.2	48.5
HIST1H4B	100.8	33.6	52.6	7.7	20.0	21.5	117.1	8.8	54.1
COTL1	98.4	38.4	46.7	14.0	28.1	36.2	72.8	16.3	63.5
HIST1H3B	68.0	25.8	41.7	12.2	25.7	38.9	116.6	13.2	70.6
CUTA	108.7	41.3	52.9	10.1	23.7	31.4	79.2	11.1	52.5

HIST1H2BD		92.1	41.5	53.1	14.6	34.3	42.3	49.1	14.8	67.6
NT5E		115.4	41.3	51.4	8.2	18.0	25.9	81.5	10.7	56.8
KHSRP		99.5	43.2	50.4	10.3	21.5	29.0	64.6	16.2	73.9
ATP5B		99.3	38.9	52.0	5.9	17.2	23.0	95.3	11.0	66.0
DKK1		64.5	25.9	34.9	13.9	25.0	36.7	118.0	16.0	72.6
HSP90AA1		97.7	40.6	46.6	3.6	15.5	22.7	92.5	12.1	73.0
EIF2S2		105.0	42.2	52.5	4.2	17.1	22.0	76.7	12.0	71.6
GANAB		106.6	37.3	49.8	6.2	17.9	23.2	87.4	11.2	61.9
TRNF		79.9	54.2	45.7	28.8	44.9	44.6	35.6	15.1	51.5
LARP1		96.3	33.9	42.8	11.4	20.9	26.6	80.2	15.7	72.5
CDH11		169.1	53.5	74.3	5.9	14.4	20.1	32.8	5.2	24.0
CD151		104.0	43.6	59.4	15.0	28.7	38.5	52.1	10.4	47.4
RNASEK		99.6	51.4	61.3	5.9	22.4	36.3	51.9	12.5	56.7
MAZ		100.8	39.4	50.1	12.8	19.6	28.2	71.3	14.5	60.8
SLC38A2		80.9	29.6	39.7	2.4	9.7	13.9	106.7	15.2	99.1
BRD4		105.8	35.5	46.4	14.8	20.8	26.8	64.0	16.6	65.2
NR2F2		99.5	35.3	45.1	18.8	28.1	37.7	61.6	14.5	55.1
ILF3		93.4	34.4	45.1	11.0	21.8	30.0	75.7	15.7	68.8
COX5B		102.3	39.3	58.8	9.9	22.2	31.2	64.6	10.5	56.4
PGLS		118.0	42.6	56.1	14.2	24.4	33.6	62.0	9.0	33.0
TMEM123		99.6	47.3	56.8	7.0	18.9	29.3	49.3	13.3	71.3
AMOTL2		93.1	36.1	45.0	15.1	24.7	32.3	75.1	14.4	56.7
HLA-E		102.3	40.9	50.9	10.6	21.6	27.3	75.6	11.0	51.5
WDR1		96.4	35.4	45.1	9.3	19.8	26.9	80.7	12.7	65.0
YWHAQ		104.1	39.8	54.7	9.5	19.5	23.9	69.4	10.4	58.6
ANKRD11		91.8	27.8	38.8	11.6	17.0	22.4	88.0	16.4	74.0
PDIA3		87.1	33.5	40.8	5.4	13.5	19.0	100.2	15.4	71.9
MDH2		99.2	40.0	52.0	12.9	19.8	30.2	64.7	12.8	54.2
BCAR3		94.7	32.8	43.9	9.2	21.5	29.5	76.7	12.6	63.8
PAWR		82.7	29.6	40.1	15.2	19.7	26.3	88.3	15.5	66.5
MMP1		24.0	10.5	14.8	7.3	26.4	36.7	139.6	16.6	107.9
CTSD		107.9	40.5	55.7	19.1	31.6	43.6	39.5	7.9	35.6
MYL6		102.7	42.6	64.3	7.8	20.8	26.5	57.6	11.3	47.7
ARPC3		92.2	41.6	53.1	5.0	18.0	20.9	69.5	12.8	65.7
LINC00152		105.9	43.3	63.9	5.4	16.3	21.3	62.2	9.2	51.2
SERP1		92.0	35.1	47.7	9.6	19.1	23.7	69.5	14.1	67.7
TKT		95.3	39.0	47.9	10.8	17.7	25.4	63.6	15.2	62.9
SNORA7B		104.5	40.0	61.0	6.3	14.8	17.0	84.7	10.7	38.8
HNRNPH1		101.8	38.6	54.5	4.9	14.8	16.7	75.4	10.9	59.7
RPN1		98.0	35.9	48.4	5.9	12.5	18.3	83.2	12.5	60.6
NDUFB7		104.5	43.0	60.9	14.3	23.3	25.6	52.6	11.0	38.2
DRAP1		87.8	34.9	44.8	14.6	25.8	34.7	60.7	13.0	57.1
	44813.0	108.8	42.3	52.8	12.2	20.7	26.2	46.8	11.7	51.3
TUFM		91.1	37.2	46.4	10.4	18.7	26.4	72.1	12.5	57.4
LMNA		103.4	37.4	48.3	14.7	23.5	31.7	60.8	10.1	42.0
MCAM		95.2	39.1	49.1	7.9	17.1	22.6	73.7	11.8	54.8
PRELID1		92.7	35.6	44.6	8.9	17.8	24.6	76.4	12.4	58.2
ANXA1		98.2	37.7	53.9	4.4	16.0	21.3	74.2	9.4	55.3
BCAR1		102.3	40.6	52.1	12.2	16.4	22.6	60.5	13.0	49.6
UQCRH		85.3	34.3	57.2	6.0	17.3	20.8	76.5	11.6	59.7
SLC9A3R2		120.7	43.3	55.0	15.3	23.6	31.7	42.1	7.4	29.1
LRP10		114.0	44.0	55.8	11.8	20.6	28.2	48.1	8.5	37.1
COX7C		102.0	38.1	57.3	2.8	13.7	18.9	78.0	7.2	49.8
AJUBA		93.6	37.4	49.4	10.4	19.8	27.4	72.4	11.3	45.6
ANP32B		78.5	26.8	35.4	4.7	13.2	15.2	111.3	13.8	68.1

KRT8	119.4	45.1	56.8	6.8	13.8	18.4	55.9	9.0	40.8
LMAN2	105.2	43.2	58.9	11.7	18.8	27.8	51.9	9.7	38.1
DDOST	102.3	41.4	54.7	7.5	15.8	22.2	60.3	10.4	50.6
CSDE1	97.4	34.5	43.6	3.6	14.2	19.6	78.0	10.3	63.7
HNRNPU	87.6	32.3	41.7	10.8	20.0	25.8	75.6	12.9	58.2
UQCR11	99.6	30.5	50.4	11.4	18.8	27.9	79.6	9.2	36.5
LOXL2	112.3	39.8	53.0	11.8	22.5	31.5	50.8	7.7	33.1
RHOA	99.6	36.4	49.8	4.9	14.8	18.4	79.7	8.9	49.0
CCDC85B	91.5	31.5	45.7	15.7	21.9	33.1	71.7	10.8	39.2
C1QBP	83.4	31.9	45.9	7.2	15.5	20.6	82.6	11.9	62.3
EZR	91.4	34.3	41.9	7.6	14.7	19.0	72.0	14.5	65.3
COX8A	90.9	49.3	62.9	11.0	20.3	31.8	47.7	7.8	39.1
PPP1R15A	65.8	25.2	33.4	9.7	20.1	26.6	86.5	15.9	76.7
HNRNPUL1	103.3	40.9	50.2	10.1	20.8	27.1	51.2	10.9	44.6
SNORA8	90.2	35.7	38.5	15.1	30.3	25.9	53.6	12.4	57.3
PSMA7	89.6	33.1	51.1	6.4	15.3	21.9	77.4	9.0	54.6
CCT3	87.9	34.0	46.2	4.4	14.9	20.4	80.7	10.2	59.2
SYNGR2	99.2	42.3	52.7	9.9	18.8	25.4	56.4	11.1	41.6
C12orf57	79.3	30.5	47.6	13.6	20.1	30.5	73.5	14.3	47.7
STIP1	83.8	33.1	42.9	6.4	14.4	19.3	68.6	14.2	73.4
AHSA1	87.1	35.1	41.8	6.2	13.9	16.8	73.5	13.1	66.5
TMEM259	95.1	40.2	51.8	11.7	18.7	23.4	54.6	11.6	46.0
CST1	161.6	80.9	98.5	0.9	2.8	2.2	2.9	0.9	2.0
YWHAZ	97.4	38.3	49.3	4.1	16.8	22.3	59.1	9.4	55.9
SLC25A5	87.1	32.6	47.4	5.8	14.2	19.6	76.9	10.9	57.5
KDELR1	112.3	41.0	50.8	6.8	15.9	23.5	47.9	9.1	44.6
PKIG	89.3	36.5	51.8	11.8	19.4	29.8	58.6	9.1	45.6
PSMD2	81.5	31.2	39.2	7.9	20.0	24.3	77.0	10.9	59.9
SUMO2	97.9	34.1	52.9	3.4	17.0	22.1	63.5	7.4	52.5
ITPK1	95.5	38.3	47.3	10.0	17.9	22.6	56.4	11.3	51.2
INF2	115.1	35.4	60.1	10.7	21.6	22.1	44.6	6.5	33.6
TSPO	117.5	48.4	60.9	10.7	21.9	28.2	36.8	5.5	19.8
NFE2L1	96.6	37.2	47.1	7.9	16.0	22.8	61.4	10.3	49.0
LOC101927338	117.7	21.3	49.1	5.2	17.0	20.3	67.8	5.6	43.6
TRNY	122.7	46.7	47.1	16.6	19.1	27.3	27.3	9.3	30.2
ATP5G2	90.1	39.8	51.7	7.8	19.0	26.7	53.0	10.6	47.0
CCT5	84.0	32.8	40.7	4.2	15.0	19.8	78.3	10.8	59.8
RAC1	96.1	35.5	45.2	6.1	18.0	21.1	61.2	11.3	50.7
HGS	83.2	34.9	43.5	13.3	22.9	29.7	55.0	11.2	51.2
CLTA	99.9	36.7	47.2	7.4	15.2	18.3	64.3	9.4	46.5
MARCKS	99.7	38.1	49.3	18.7	23.4	26.3	47.4	9.4	32.3
HIST2H3A	46.6	17.2	27.3	20.8	34.9	47.6	82.5	13.8	53.4
HIST2H3C	46.6	17.2	27.3	20.8	34.9	47.6	82.5	13.8	53.4
ACLY	102.6	41.8	50.1	6.4	14.5	19.4	53.5	9.1	46.8
NHP2	94.5	34.4	52.4	8.1	19.1	27.1	53.5	9.1	45.9
KXD1	84.8	30.6	50.0	10.4	18.0	26.6	62.9	11.7	48.8
HIST1H2BC	86.3	39.1	50.5	13.5	22.8	33.6	39.0	11.3	47.3
PRRC2A	91.3	33.4	41.4	9.7	17.5	23.5	58.9	12.4	55.2
TOMM40	81.6	31.3	41.2	8.4	16.2	20.8	75.1	11.2	55.5
CD63	96.4	39.3	55.4	4.7	12.4	18.8	61.1	7.2	45.7
AUP1	85.2	39.0	53.0	6.8	13.8	20.5	56.5	11.2	54.0
CALM3	98.7	41.2	51.9	9.4	20.3	28.6	41.5	8.2	39.0
BASP1	68.3	27.0	35.8	15.9	21.1	28.9	73.3	14.8	53.4
ERRFI1	79.9	31.0	38.1	3.5	11.5	15.8	71.7	11.6	75.2
HNRNPK	70.1	30.9	35.0	4.1	14.6	22.0	70.7	12.3	77.7

PFDN2	72.9	31.4	38.8	6.1	16.4	20.0	62.0	13.2	76.7
HEG1	125.3	41.5	54.4	6.7	16.8	21.8	33.9	5.6	30.1
COL8A1	72.3	30.1	36.3	6.9	16.4	21.5	73.7	14.0	64.3
ADI1	71.3	27.3	33.8	7.1	18.1	21.9	86.1	11.2	57.5
NUDC	82.0	32.9	38.7	8.6	18.3	24.9	64.8	11.3	52.7
MAT2A	55.1	22.8	28.9	3.5	11.3	14.7	103.0	14.1	80.8
MYL12A	102.2	39.5	58.4	4.5	15.8	20.2	43.5	6.9	43.0
CDK2AP1	84.7	35.0	48.8	7.7	13.6	19.6	59.9	11.3	52.8
HERPUD1	74.1	28.2	32.8	6.5	11.6	16.6	78.0	15.6	69.3
CALM2	90.4	37.1	50.1	2.7	12.9	19.0	62.3	8.8	49.0
IFITM2	97.4	46.1	53.8	11.3	17.9	25.2	33.9	10.0	36.6
TPBG	88.7	37.0	44.7	11.0	14.2	20.1	58.7	11.6	46.1
SCARNA10	79.9	42.5	57.0	1.6	11.0	18.6	66.1	6.5	48.9
NDUFS8	74.5	29.8	43.2	12.7	20.2	32.6	58.8	12.7	47.5
SERBP1	64.3	24.0	30.5	5.5	13.8	16.8	90.1	14.1	72.1
AES	100.7	38.0	51.9	8.0	15.8	21.3	45.7	7.7	41.0
EIF3D	82.5	29.1	36.8	6.6	15.7	21.9	69.6	10.9	55.8
RAB35	83.6	32.6	42.2	7.3	12.9	20.9	59.6	12.2	57.5
SERINC3	96.8	37.7	48.7	5.4	15.4	21.4	48.3	8.9	45.7
SAP18	93.9	35.2	46.1	8.4	17.4	20.1	53.0	10.7	43.0
ITGB1	91.5	33.7	42.8	3.4	12.9	19.3	64.5	7.7	50.9
PFDN5	84.0	38.6	54.8	4.7	16.8	20.6	52.2	7.8	47.2
ATP5I	88.7	35.8	54.6	7.7	17.2	23.2	49.6	9.8	39.8
CSNK1E	84.2	31.7	39.3	8.2	14.6	19.3	59.5	13.4	55.0
CD81	96.1	35.0	44.1	7.9	13.0	19.6	58.7	8.5	42.2
NFKBIA	111.9	46.0	62.1	4.9	9.6	12.7	39.1	8.0	30.6
PRDX6	74.7	31.4	40.4	5.3	15.9	21.5	73.1	9.9	52.3
SF1	89.3	35.1	44.1	11.0	18.5	24.4	45.4	10.9	45.3
MYOF	89.5	29.7	38.5	6.4	18.6	25.6	60.5	8.4	45.3
ZYX	83.9	31.6	38.3	11.1	18.7	23.6	56.3	11.5	46.7
C19orf10	93.5	39.7	47.8	8.5	15.7	21.1	52.9	7.8	34.0
SNRNP70	80.3	35.1	44.3	11.5	18.3	22.5	54.0	10.3	44.2
ATP6V0C	95.1	34.5	49.9	10.1	16.0	24.3	44.1	9.5	37.0
GADD45GIP1	74.7	31.0	40.8	9.6	16.0	21.9	64.8	13.0	48.5
DAD1	84.3	33.3	47.8	4.8	11.6	20.8	62.6	9.2	46.0
TSPAN3	98.0	37.8	46.9	4.9	14.8	20.6	50.5	7.3	39.5
PIGT	101.1	40.9	49.9	12.2	19.3	28.6	30.8	7.0	29.3
PLD3	96.0	35.8	47.0	8.7	16.7	21.6	49.1	8.7	35.7
SNORA84	74.1	38.5	46.0	7.5	15.2	12.2	86.9	8.2	30.3
PDLIM5	92.2	29.9	40.5	5.5	13.8	19.1	62.3	8.3	47.2
VAT1	92.9	37.5	45.5	9.7	17.4	22.7	48.4	7.6	36.9
EDIL3	102.0	38.5	52.2	3.4	13.8	18.5	46.6	6.2	37.3
MRPL4	81.9	32.7	45.2	11.8	21.2	26.9	49.0	8.5	41.0
BANF1	85.2	31.2	43.7	4.1	13.8	16.6	72.1	6.3	44.5
COMT	85.6	33.7	44.5	9.2	16.4	18.6	57.5	9.9	42.0
AMIGO2	55.0	19.9	26.5	3.4	10.3	13.9	105.9	11.1	71.5
MAPK1IP1L	84.1	31.2	37.4	9.1	14.6	20.8	48.5	13.7	57.9
SSR2	84.7	33.8	47.3	5.2	15.8	19.8	55.1	9.9	44.8
PLOD2	78.0	30.2	39.8	1.5	7.8	12.4	73.5	8.3	64.7
TP53	103.1	35.9	31.1	10.5	18.8	29.7	38.8	11.0	36.6
BCL9L	99.8	41.3	49.9	10.7	16.2	21.5	31.6	9.0	34.8
JUN	80.2	28.8	40.1	13.8	19.6	27.3	50.7	11.7	42.3
SF3B2	89.3	33.0	40.6	5.9	13.3	18.0	58.0	10.4	45.2
NDRG1	90.9	40.1	49.5	3.9	6.5	9.8	55.6	12.1	45.1
CDC42EP1	75.5	27.4	34.0	12.5	18.3	25.4	59.9	11.6	48.9

CORO1C	74.8	27.9	37.9	5.0	16.0	22.1	65.2	8.5	56.1
BAG6	81.9	32.0	39.9	8.4	15.5	22.0	52.4	11.2	47.7
UQCR10	74.7	40.6	50.8	13.4	19.9	32.9	33.7	10.7	34.2
FAM122C	108.5	18.9	46.6	6.1	9.8	11.5	75.9	5.5	27.4
USP22	86.6	31.4	39.8	9.2	18.5	25.0	49.5	9.0	40.3
PRDX2	85.9	35.3	45.1	7.3	15.5	23.0	54.0	7.2	35.9
AP2S1	79.2	31.8	41.4	7.5	15.0	20.9	60.3	8.6	44.6
PYCR1	66.1	27.2	34.5	9.9	17.1	24.9	66.4	11.4	51.4
PUF60	83.4	32.5	43.0	8.4	15.6	20.3	53.4	9.7	42.1
CDV3	65.7	29.9	36.7	4.9	15.4	15.8	53.4	16.0	70.3
SLC2A1	62.0	26.0	30.0	8.5	16.4	22.7	75.5	11.9	54.7
CD320	84.1	29.8	41.0	13.8	19.1	27.0	53.2	7.2	30.6
SSR4	77.3	30.4	42.1	7.0	11.5	18.2	67.3	9.2	43.0
ARHGEF18	82.6	28.4	36.6	9.1	14.2	19.2	63.5	9.5	41.9
CKAP4	72.4	28.0	34.0	10.1	17.1	23.6	62.4	10.6	46.3
AP2M1	78.3	30.2	39.0	8.2	20.0	26.6	51.2	7.5	43.1
MAP2K2	89.0	31.3	41.1	10.0	17.9	26.7	48.7	7.2	32.2
PNPLA2	81.1	30.8	37.4	11.7	18.5	23.8	52.6	9.9	37.5
BOP1	62.1	21.8	30.0	10.5	17.0	23.7	76.5	10.7	50.9
RUVBL2	65.1	25.3	32.3	11.3	19.6	25.7	58.1	11.7	53.9
YWHAG	70.9	29.0	34.9	9.9	18.3	26.0	53.7	10.8	49.6
ANXA6	87.5	34.3	42.5	6.7	16.6	21.8	47.8	7.7	37.3
OAF	68.6	25.0	34.5	11.4	17.1	23.2	64.4	11.1	46.7
EIF3C	79.0	27.7	34.8	8.8	18.1	24.3	55.0	9.0	45.1
EIF4A2	76.1	28.6	40.5	2.2	10.8	13.6	70.5	8.3	51.1
EMP1	87.1	33.9	39.3	5.7	17.2	25.5	38.8	7.8	44.9
RNF181	78.2	32.5	43.8	6.1	16.4	21.0	49.7	8.4	43.6
GNB2	84.7	33.3	43.3	8.8	14.7	22.5	46.9	8.2	37.1
SSRP1	73.6	27.2	34.4	5.3	12.6	18.2	64.5	10.0	53.8
GTF2F1	74.6	30.4	39.4	11.0	16.4	20.7	47.3	11.2	48.3
FRMD6	59.6	23.0	28.8	5.2	12.6	16.7	73.7	13.0	66.7
MORF4L1	78.3	30.4	42.2	2.2	14.1	15.3	58.7	8.2	49.6
SERPINH1	78.9	30.7	40.0	9.1	13.5	18.6	58.6	10.0	39.7
PSMB4	76.9	30.7	41.2	4.7	11.6	19.7	62.0	8.4	44.0
PTGES	91.4	37.6	46.9	20.3	30.2	42.9	14.4	3.2	11.7
NDUFB4	83.1	34.9	43.3	8.4	17.0	25.0	33.4	10.0	43.0
LINC00657	81.2	28.3	35.9	7.0	16.0	23.1	54.6	8.3	43.7
PNPLA6	93.5	33.7	47.4	8.5	14.9	19.3	43.0	7.3	29.9
LRRC59	82.5	29.1	37.1	7.3	12.5	18.0	58.8	10.0	42.0
RPL17-C18orf32	73.7	29.1	41.3	4.1	12.0	16.5	59.7	9.4	51.4
CSTB	74.6	36.0	45.1	8.8	16.6	24.3	36.6	9.4	45.1
STT3A	71.7	30.3	36.9	3.9	11.5	17.1	57.9	9.7	57.2
DNAJB1	89.2	34.2	40.3	6.0	11.7	16.4	50.9	9.2	38.2
CDCP1	95.0	30.5	38.9	6.6	11.5	18.8	48.3	8.4	37.7
CSNK2B	69.5	30.4	37.2	9.5	18.3	26.7	48.2	9.2	46.6
CYB5R3	85.6	35.2	43.0	9.5	16.8	22.5	38.3	8.7	35.9
H2AFZ	67.8	26.6	38.9	5.8	13.2	15.0	68.3	8.4	50.9
SLC25A6	64.5	24.8	33.8	9.9	16.2	22.4	62.3	11.8	47.9
SLC25A6	64.5	24.8	33.8	9.9	16.2	22.4	62.3	11.8	47.9
CD164	79.7	29.7	42.1	9.3	13.2	22.0	47.6	8.3	41.7
ECH1	82.9	33.7	45.6	9.7	18.5	24.4	42.0	6.7	29.4
PSMB3	78.0	30.9	46.9	5.5	14.2	17.4	52.9	7.0	39.5
LDHA	85.2	30.5	41.2	3.3	10.9	15.5	54.0	6.6	44.9
ROMO1	73.3	26.8	42.8	7.8	12.7	17.9	71.1	6.1	33.0
SDC4	96.7	37.9	51.6	7.1	14.1	19.9	36.4	5.2	22.2

DDX5	84.0	30.9	38.5	2.5	11.2	15.3	61.5	6.5	40.0
XRCC6	66.4	26.6	33.4	3.5	10.3	13.9	69.3	8.7	57.8
WBP2	80.7	34.8	44.5	9.6	16.8	24.6	33.4	8.5	37.2
MAPRE1	75.3	23.9	35.3	2.7	12.0	14.4	68.0	7.6	50.7
PRDX5	83.3	37.1	50.9	7.5	15.5	20.0	40.2	5.9	29.2
PVRL2	83.7	32.4	40.9	8.7	13.0	19.1	45.0	9.1	37.4
EIF3A	75.3	24.3	32.1	4.8	12.6	15.8	66.2	9.5	48.7
MYL12B	83.7	32.8	47.9	3.3	11.8	14.5	47.8	5.7	41.7
LOC100506634	87.6	23.0	44.5	7.2	14.4	16.6	55.6	6.8	33.4
LOC101928293	70.9	25.7	31.9	16.0	17.1	24.9	52.0	12.1	38.5
DBN1	63.6	27.3	33.9	11.9	19.0	26.6	51.7	11.5	43.3
RAB7A	77.8	33.5	40.8	4.4	14.2	18.3	43.3	8.4	48.1
COX6B1	77.1	32.1	44.2	8.1	15.7	20.0	45.9	8.0	37.5
TXN	63.1	24.1	34.0	3.2	11.9	16.6	73.8	8.9	52.0
GSTO1	67.6	26.5	38.0	7.0	16.0	20.9	63.2	6.5	41.7
RAB1B	82.9	32.5	43.7	8.3	17.0	25.8	35.9	6.3	34.3
RAB5C	82.3	30.2	42.0	6.8	13.3	19.1	40.7	9.6	42.6
LAMP1	82.6	30.1	38.1	7.7	15.4	20.9	44.1	8.2	37.9
CLPP	81.0	28.8	37.4	10.2	16.1	23.0	44.7	8.6	34.6
MRPL52	59.3	22.2	35.9	8.6	17.6	24.2	65.3	9.1	42.2
PABPC4	63.8	20.7	27.5	4.8	9.5	14.6	73.2	12.7	57.1
APEX1	66.9	27.5	32.2	4.4	11.1	16.6	64.1	9.9	50.7
ICAM1	111.2	45.5	56.8	5.1	9.7	14.6	20.2	4.3	15.8
HNRNPA2B1	63.8	25.0	31.9	3.4	11.1	15.8	68.9	10.0	53.1
RHOC	81.1	33.2	42.7	7.0	13.6	22.8	38.4	6.5	37.3
PAFAH1B2	75.2	25.2	35.5	4.6	12.6	16.2	59.9	8.1	45.2
EPN1	79.7	30.6	37.4	11.2	15.9	22.2	43.0	8.9	33.1
IMPDH2	61.3	24.7	29.9	4.4	11.2	17.1	69.2	10.3	53.5
HSPA9	64.5	24.9	29.3	2.9	12.2	14.9	66.2	9.4	57.1
DDX39A	77.0	29.4	39.7	4.9	10.5	13.4	54.6	9.3	42.7
HDLBP	77.8	28.3	36.4	5.4	14.2	19.0	49.5	8.2	42.1
NEAT1	87.3	35.9	52.8	5.6	14.0	18.3	34.8	4.3	27.1
DUSP6	73.8	30.5	37.7	8.2	14.6	20.3	46.5	9.6	38.5
ZFAND3	71.9	26.4	31.9	3.9	11.8	15.3	51.9	9.2	57.2
NUTF2	65.7	37.2	43.0	6.5	17.9	22.8	30.1	8.8	47.4
PTPRF	59.0	21.6	28.5	10.5	18.2	26.1	59.8	10.8	44.7
NOTCH2	93.1	25.5	40.4	4.5	10.1	15.2	45.5	5.9	38.8
PRKAR1A	80.8	28.6	35.9	3.4	11.8	18.8	50.6	7.3	40.5
TBCA	58.9	27.1	34.9	4.9	12.7	18.2	58.7	9.8	51.2
HNRNPA0	68.7	25.0	29.7	9.1	12.0	13.2	66.7	9.6	42.4
UBE2S	59.6	23.7	29.5	9.3	15.5	19.0	64.0	9.9	45.1
ADRM1	70.3	26.2	35.1	8.1	12.2	17.4	51.4	11.6	43.1
PCNA-AS1	63.9	25.3	31.9	8.9	16.3	20.1	60.8	8.9	39.0
KPNB1	63.4	21.9	28.4	3.6	10.8	14.2	68.7	9.0	54.7
IDS	75.6	26.7	35.0	6.6	15.1	22.0	48.8	7.3	37.5
RBM39	78.4	29.7	41.8	2.9	8.8	11.8	49.9	7.7	42.1
ECE1	86.0	32.8	40.4	9.5	19.3	24.5	26.9	5.9	27.7
NDUFA13	74.6	34.1	46.1	9.6	15.9	22.6	35.7	6.0	28.2
PRPF8	76.6	25.5	33.2	4.8	11.9	15.8	52.9	8.2	42.7
COPE	75.9	30.6	39.3	7.8	14.2	18.8	45.2	7.6	31.9
RBM8A	73.6	27.1	33.9	5.2	14.0	18.0	54.0	8.5	36.9
SLC52A2	70.1	27.3	34.3	7.3	11.9	15.7	58.7	8.4	37.5
MZT2B	77.2	25.7	37.0	9.5	14.9	21.8	44.0	7.0	33.8
MARS	72.4	26.1	33.8	5.0	11.3	16.0	57.4	7.9	41.0
PPP2R1A	81.2	31.0	39.1	6.9	15.3	18.9	42.7	5.8	29.6

LOC101929361	81.7	28.2	42.3	13.6	17.7	24.1	37.9	5.7	19.3
LAPTM4A	80.1	29.9	38.9	3.4	9.1	15.8	48.2	6.9	37.9
TRAPPC5	78.3	31.7	41.6	13.5	16.8	21.6	29.4	8.6	28.6
LRP5	90.2	33.3	44.8	12.1	19.4	24.8	23.4	4.7	17.2
C19orf53	63.6	28.8	39.4	8.7	15.0	19.6	40.4	12.6	41.6
AP2B1	72.7	24.1	35.6	3.1	11.9	13.9	58.3	6.1	43.7
FARSA	56.5	21.5	28.3	7.4	15.0	19.8	62.4	10.3	47.7
CNN2	67.4	26.6	33.7	8.4	16.8	24.4	44.0	10.2	36.8
CCT6A	65.2	24.1	29.7	4.4	10.4	14.8	62.0	9.1	48.5
CD44	63.0	25.6	34.1	7.5	18.0	26.9	36.3	9.0	47.1
EIF4G2	67.2	25.5	31.6	2.7	10.6	13.4	59.1	8.4	48.9
DYSF	79.3	27.7	35.8	7.2	16.4	20.9	43.4	6.2	30.3
TGFB1	79.7	30.7	40.3	9.3	14.4	21.9	33.4	7.3	29.9
IRF2BPL	79.3	31.5	40.9	10.4	14.3	18.6	36.8	7.2	27.1
CNN3	61.9	28.7	39.2	3.6	12.8	17.7	47.8	7.4	47.0
S100A10	76.4	35.4	39.2	1.7	10.0	13.3	43.0	6.6	39.9
RNH1	76.4	27.1	35.1	7.2	13.1	17.8	49.9	7.6	31.1
GIT1	81.2	29.4	36.2	8.5	12.0	18.4	38.9	7.8	32.8
ARCN1	73.8	29.1	35.7	2.9	9.7	14.0	48.9	6.9	43.2
HINT1	66.5	33.7	42.3	4.1	11.3	18.2	42.6	8.8	36.6
HNRNPF	71.1	28.3	35.3	5.2	12.5	15.2	43.9	8.6	43.2
EIF3L	64.0	26.2	33.4	4.4	11.4	16.9	49.9	9.3	46.9
NDUFA3	68.2	29.5	39.4	10.5	19.6	25.0	29.5	9.0	31.0
CDK2AP2	62.6	25.6	32.3	7.2	11.9	16.8	50.1	11.3	43.6
CCNI	89.9	33.2	38.9	4.5	12.9	17.2	33.3	4.9	26.6
VASP	72.2	29.1	35.8	6.3	13.1	15.8	46.0	8.3	34.4
MRPS34	64.9	26.4	32.9	10.5	14.2	18.9	47.3	8.4	36.2
PHB2	62.8	26.4	34.1	5.8	14.0	18.4	47.9	8.8	41.2
HSPH1	77.1	27.8	35.0	1.5	6.2	8.6	55.7	7.0	40.5
SRGN	54.5	21.4	35.7	2.2	9.6	13.9	67.6	7.0	47.3
MORF4L2	63.7	25.1	33.6	4.7	13.3	18.4	51.6	7.0	41.5
FUS	70.4	27.5	33.4	4.4	9.6	13.4	46.4	8.3	45.4
G3BP1	61.5	21.6	28.3	3.8	12.7	15.2	60.0	7.6	47.8
COX5A	63.1	27.2	36.5	5.2	12.5	19.0	43.6	8.5	42.8
LY6E	66.6	26.0	33.6	8.0	15.9	20.8	50.7	5.7	30.3
RALY	75.6	29.1	37.5	8.5	17.2	20.6	31.8	7.2	30.1
TMEM109	63.6	25.0	30.9	6.1	13.3	16.9	54.1	8.3	39.2
ATXN2L	61.7	24.5	30.0	7.3	13.0	16.6	44.4	11.9	47.9
MYADM	66.6	23.6	30.2	9.7	15.9	21.1	46.7	9.2	34.4
PRPF19	54.3	21.8	26.6	5.4	11.3	17.1	55.9	11.0	53.6
MRPL34	66.1	31.8	39.8	7.5	13.1	18.5	43.9	7.5	28.5
DSTN	71.1	29.2	39.8	0.9	8.6	12.8	47.8	6.1	40.6
CTSA	81.8	31.4	40.1	9.4	15.4	22.5	25.9	5.8	24.5
PODXL	69.4	28.2	34.9	6.3	12.1	17.1	35.3	10.3	43.0
AARS	71.8	26.1	33.9	4.1	11.9	15.5	49.8	6.8	36.6
DYNLL1	68.6	30.2	40.4	4.8	11.8	17.8	34.9	7.8	40.1
SND1	66.3	23.0	29.5	5.5	12.5	16.7	54.4	7.7	40.5
RUNX1	69.5	24.3	31.2	12.1	14.4	20.0	37.0	10.6	36.7
LOC101927337	59.8	27.8	48.1	2.8	8.2	9.7	33.5	9.7	56.3
NSMF	77.2	29.0	35.9	10.0	17.0	24.3	31.5	5.6	25.4
BUB3	55.0	20.0	30.3	6.3	15.9	21.2	53.5	7.9	44.6
SLC39A1	71.1	23.8	37.1	4.0	9.2	14.6	58.5	6.8	29.6
NOP10	67.2	31.5	42.9	6.1	15.0	20.7	29.5	6.8	35.0
MARCKSL1	92.0	36.4	46.8	5.6	9.6	13.2	25.9	5.1	20.2
NOLC1	53.5	19.8	25.6	4.2	10.5	15.5	57.6	10.2	57.1

RNF7	66.8	22.8	38.8	4.7	10.3	12.1	54.1	6.4	38.1
FAM32A	68.4	31.0	36.6	7.0	14.0	19.6	32.3	9.7	35.2
HNRNPH3	55.3	21.6	29.9	2.2	7.3	10.5	60.4	8.3	58.2
CAPNS1	72.2	32.2	37.4	9.4	17.4	24.5	26.1	5.8	28.7
GARS	62.9	21.2	25.7	5.0	11.8	15.8	59.2	9.0	43.0
TUBB6	63.1	24.1	32.7	9.4	14.4	19.8	45.4	9.4	35.1
PDIA4	59.4	22.0	27.3	3.5	9.5	13.0	64.8	9.6	44.2
C1orf43	59.0	26.4	33.9	3.6	9.2	13.2	57.0	7.9	43.0
DDA1	65.6	26.1	34.0	8.0	14.2	21.2	36.8	9.8	37.4
SRSF2	71.3	29.0	39.1	8.7	13.4	18.5	37.7	7.1	27.7
SRP14	67.2	29.2	40.9	4.7	11.3	15.9	40.3	7.5	35.6
RRBP1	68.4	27.2	33.5	7.3	15.4	19.3	41.0	7.6	32.6
SNORA49	60.0	25.1	37.4	3.6	7.2	12.5	53.6	7.7	45.2
SNORA70	65.7	21.0	34.1	6.3	10.9	10.6	64.5	4.3	34.4
EIF3M	61.4	23.9	32.2	1.7	9.0	11.3	63.3	7.1	41.6
KRTCAP2	60.9	32.1	36.9	7.8	15.4	18.9	32.8	6.9	39.4
SFPQ	68.7	27.7	34.9	7.6	12.4	15.8	39.3	8.6	35.9
LOC101927374	83.7	16.9	38.3	3.1	11.3	13.6	48.0	4.3	31.2
UBE2M	61.3	25.1	32.3	7.1	15.1	19.2	41.0	9.7	39.3
APRT	61.4	25.3	31.0	10.1	16.5	22.5	42.8	7.2	32.5
LOC100996482	81.6	17.6	32.9	3.8	9.2	9.3	63.4	5.0	26.4
DHCR24	77.5	29.8	40.0	4.0	8.0	11.4	39.8	6.9	31.3
PARK7	63.0	25.4	34.9	3.8	9.7	14.8	53.1	5.5	38.2
PYGB	80.2	29.3	37.6	6.8	13.4	18.5	32.2	5.4	24.5
CCT4	59.6	23.6	30.2	2.6	9.4	13.4	57.7	7.5	44.0
TPD52L2	65.1	24.3	33.3	4.7	11.6	16.3	48.6	6.8	36.7
DDX54	77.9	26.5	34.1	8.3	14.0	19.4	35.9	6.1	24.9
DCBLD2	58.8	23.5	29.1	6.3	13.4	18.4	43.8	8.2	45.2
FKBP2	54.4	23.2	29.8	5.8	11.0	20.5	49.7	11.6	40.3
PPP1CA	63.9	26.2	34.5	6.1	11.7	16.3	47.5	6.3	33.5
SLC25A39	63.0	27.2	36.4	8.1	14.9	21.8	38.5	6.9	28.8
MPRIP	66.5	22.0	29.6	5.5	11.5	14.1	49.3	8.1	38.7
PRMT5	51.6	22.9	27.2	3.2	8.9	12.9	62.5	8.9	46.8
XBP1	57.3	20.2	28.4	3.9	7.9	11.6	63.3	9.6	42.6
EIF3I	56.5	25.0	36.4	4.7	10.4	15.0	46.0	8.4	42.0
PHPT1	63.7	30.5	45.1	7.4	13.5	24.0	26.2	5.8	27.6
MBD3	67.8	25.7	32.3	9.6	12.1	18.0	40.0	7.8	30.2
NUDT4	77.9	26.4	34.8	5.5	10.6	15.0	40.4	5.8	26.3
SLC25A37	67.2	27.7	38.7	7.0	12.2	16.4	30.3	8.6	34.3
C11orf68	60.9	24.0	30.9	9.4	15.3	21.1	43.1	7.3	30.1
COX6C	65.5	26.9	38.9	2.5	8.6	10.4	51.1	5.2	33.1
EID1	67.9	26.0	35.7	7.0	11.6	19.4	37.0	6.4	31.1
SEC13	62.3	21.3	30.6	4.9	10.1	13.5	54.7	9.0	34.9
PTOV1	60.8	27.2	35.5	8.3	15.8	18.7	35.9	7.4	31.6
CTDNEP1	68.8	29.5	31.6	4.7	10.2	14.8	34.8	7.0	39.5
FLII	55.8	21.1	28.4	8.8	14.8	19.9	47.6	7.7	36.7
DUSP5	56.5	24.2	31.5	6.9	11.7	14.8	43.6	9.8	41.7
CTSB	72.8	28.9	37.2	6.2	14.0	19.3	31.9	5.1	24.7
PSMB6	63.0	25.6	31.5	5.4	12.4	16.8	40.2	6.9	38.4
SKP1	66.9	27.0	41.7	2.0	10.8	11.7	41.3	4.6	34.1
HSPD1	55.9	21.6	28.8	1.7	6.8	11.1	63.6	7.0	43.6
MCM7	50.7	20.6	29.1	6.2	12.4	15.9	53.5	9.1	42.5
TSC22D1	60.8	24.9	33.1	8.1	15.6	18.6	37.9	8.0	32.9
TMEM258	61.2	30.1	41.6	5.7	11.5	15.8	32.1	6.6	35.0
GNAI2	64.9	26.1	32.8	7.6	14.9	18.8	36.8	7.4	30.2

TRNA	67.6	27.6	32.4	13.5	16.7	30.2	19.1	6.2	26.0
RNPS1	62.8	25.9	31.1	6.8	10.8	17.7	37.1	9.2	37.0
TRAPPC1	73.0	30.1	35.9	5.4	14.2	17.9	31.0	5.7	25.2
EIF3E	57.5	23.9	33.5	1.7	9.3	13.2	49.1	5.9	44.1
ASNA1	67.1	28.5	38.4	5.9	12.5	16.3	33.7	5.9	29.9
CALU	63.4	24.1	37.1	1.7	8.2	10.0	49.8	6.3	36.8
STMN1	52.0	21.3	29.4	3.3	12.9	16.7	51.1	8.1	42.0
SNORA80B	45.5	22.8	34.8	7.3	16.4	18.2	54.1	8.8	28.3
VEGFA	61.0	21.6	28.9	6.1	12.1	17.2	45.7	8.9	34.4
ALKBH5	68.7	24.1	28.8	6.8	11.2	14.9	41.1	8.2	32.2
NUPR1	72.9	23.9	37.2	4.9	10.0	12.7	40.6	7.9	24.9
TAPBP	72.8	31.8	38.7	9.2	15.1	20.4	21.8	5.0	19.9
MGAT1	58.8	24.3	28.6	8.0	11.2	15.0	48.4	7.9	32.4
PABPN1	55.5	21.8	29.0	5.0	13.2	16.3	41.0	8.8	43.9
GATAD2A	55.8	22.4	27.1	8.4	12.0	16.9	37.4	10.4	44.0
EWSR1	68.1	27.1	34.9	5.2	13.7	18.9	31.2	5.4	30.0
PLP2	50.5	15.9	24.4	4.5	12.5	17.1	59.3	8.1	42.0
ETS1	53.5	20.6	25.1	5.0	10.7	15.4	48.6	9.6	45.7
HSPA1A	68.3	23.4	30.2	6.6	10.0	12.7	38.2	8.1	36.2
OGT	59.9	22.5	32.9	2.7	10.0	13.1	46.0	5.9	40.7
MDK	75.2	28.3	39.4	6.1	10.1	14.0	30.6	5.9	24.1
SYS1-DBNDD2	53.1	23.0	27.1	3.7	8.9	13.0	52.4	8.6	43.5
ISCU	63.8	25.1	36.2	3.0	11.4	12.7	43.8	5.4	31.7
SCAND1	65.1	25.2	36.6	8.1	12.3	15.3	39.0	5.8	24.8
SERF2	62.3	26.3	39.4	6.3	10.3	13.5	36.0	7.3	30.8
SRRM2	64.5	21.0	29.2	6.5	11.6	15.3	41.8	7.8	34.4
SYDE1	59.8	23.0	29.4	7.9	11.6	16.6	42.0	8.3	33.3
TIMM13	60.7	21.8	30.4	6.8	13.4	15.8	45.3	6.3	30.2
COX7B	54.9	21.9	31.0	3.7	12.6	14.8	42.2	6.9	42.2
FZD2	48.0	19.9	23.6	10.9	15.3	22.0	43.8	10.5	35.7
HNRNPD	54.5	21.4	24.1	6.7	10.6	15.1	48.5	8.1	40.7
SUMO3	59.5	23.7	34.4	9.5	12.5	18.5	30.1	8.8	32.4
HMGN1	65.9	25.9	36.2	4.1	10.4	14.4	35.6	5.9	30.8
SRSF6	60.3	24.7	30.7	6.6	12.4	17.7	38.6	6.6	31.5
NDUFS5	60.2	23.2	38.8	2.5	8.8	14.5	41.7	5.7	33.3
SNORA24	57.4	22.9	34.9	3.3	10.7	8.1	59.2	5.6	26.4
SEC14L1	59.8	20.4	26.3	3.7	9.0	11.3	51.3	7.6	38.9
TIMP1	69.7	24.6	31.5	5.7	11.1	16.3	37.5	5.5	25.9
SNHG8	52.3	19.3	27.0	2.4	7.0	10.8	63.6	7.2	38.2
NCOR2	62.1	22.8	29.5	10.9	13.7	17.9	32.7	8.0	30.0
PSMB5	59.7	23.3	31.4	3.5	9.8	13.3	48.0	5.9	32.6
UBXN1	57.5	22.3	31.2	7.7	14.1	16.5	40.2	7.0	30.8
OGFRL1	59.1	20.1	27.6	4.5	10.6	13.9	46.2	6.8	37.8
CAPRIN1	61.1	21.6	26.5	4.1	11.3	16.7	39.7	7.3	38.5
HIST1H2AG	34.9	17.1	22.2	13.4	21.9	31.1	41.3	9.5	35.2
CMIP	61.1	21.4	26.6	4.7	11.0	14.5	43.7	7.6	35.2
PRDX1	66.5	23.2	33.6	1.5	8.0	11.8	45.8	5.0	30.3
DCTN1	60.0	22.2	28.4	5.6	10.9	14.2	45.0	6.9	32.5
SCARNA17	60.0	21.8	34.4	6.2	10.8	12.3	45.0	5.8	29.4
SRSF3	48.4	16.1	22.3	1.8	7.2	10.5	74.0	6.4	38.8
BOD1	58.3	24.9	31.9	6.4	12.7	16.0	35.7	6.4	33.3
TOMM6	60.2	23.8	32.0	3.3	7.7	13.2	46.2	6.0	33.0
HIST1H4C	47.3	21.0	32.5	5.4	9.6	17.6	49.4	6.2	36.0
PPP6R1	60.5	24.2	30.6	5.9	9.6	13.5	38.3	8.4	34.2
ATP5E	50.3	23.5	36.2	2.3	12.6	19.8	31.6	7.3	41.1

RNF44	61.1	25.1	30.1	8.9	12.0	17.9	31.4	7.5	30.4
FAM168B	54.0	21.1	28.2	7.2	11.6	16.1	34.8	9.9	41.1
TRIB3	54.5	22.0	25.9	5.1	8.6	12.5	51.8	8.2	35.0
BABAM1	63.6	23.0	31.7	6.5	11.2	17.2	37.5	5.7	27.0
ARPC2	51.9	22.9	25.5	5.6	13.4	16.1	37.7	8.1	41.8
LSM4	55.3	19.2	29.9	5.8	10.5	13.6	52.2	7.0	29.6
RNU4ATAC	48.1	28.4	26.9	18.9	21.1	21.1	20.7	8.5	29.2
NACC1	65.0	20.8	28.8	6.8	9.9	13.7	39.8	7.5	30.5
SLC38A1	55.7	19.3	22.6	1.5	6.5	8.5	53.9	7.4	47.4
TARS	48.4	20.1	22.8	2.5	8.6	11.7	55.6	6.8	46.2
PSMF1	60.0	22.8	27.8	4.9	9.9	13.4	46.4	6.2	31.1
SLC35E1	58.5	21.5	27.2	8.1	11.4	16.0	37.4	8.1	34.2
HSBP1	52.5	25.9	38.9	3.8	14.4	19.3	28.5	6.5	32.3
CTBP1	65.1	25.0	30.6	8.4	12.8	17.1	32.2	5.9	24.9
MRPS26	58.8	20.6	28.4	9.1	10.6	16.9	44.4	6.5	26.2
NDUFA11	48.5	22.0	31.5	7.7	13.6	18.3	40.9	7.1	31.9
RAD21	61.1	22.2	29.9	2.1	8.6	10.5	46.7	5.7	34.6
PYURF	51.6	22.1	30.0	6.8	10.9	12.9	48.6	7.7	30.4
ARF6	47.6	19.9	24.2	7.1	11.4	16.4	46.7	8.6	38.6
RASD1	74.5	32.8	40.9	7.8	10.3	14.2	18.6	4.4	16.4
CPSF1	58.7	24.2	32.4	7.3	11.5	15.5	33.6	7.4	29.1
EHD2	69.4	23.1	28.5	7.4	11.3	15.5	37.1	5.1	22.1
KTN1	61.4	21.8	28.4	1.6	7.6	10.6	44.0	5.8	38.3
HIST1H4H	44.8	15.9	26.0	7.6	14.8	18.9	46.9	6.7	37.8
ATOX1	47.4	24.3	27.8	7.7	17.7	25.7	33.9	5.4	29.3
SNRPC	54.7	23.8	33.3	4.2	10.1	16.3	34.3	8.0	34.3
HMGB1	46.6	17.0	23.1	2.3	8.6	15.6	56.4	6.1	43.4
IGF2BP2	49.6	18.3	21.7	3.8	9.3	12.5	48.9	8.4	46.6
USF2	64.8	24.3	31.8	7.8	11.6	18.0	26.2	7.1	27.3
H1FX	52.9	25.7	34.7	6.7	11.5	13.3	31.3	9.6	32.5
LSM7	50.8	24.4	29.9	6.7	12.5	15.3	36.7	7.3	34.8
REEP5	60.0	22.1	29.7	3.0	8.9	11.7	44.2	6.2	32.6
SERTAD2	51.4	19.3	23.5	7.4	11.7	15.3	41.6	8.9	39.0
PSMD3	58.7	23.3	28.2	5.9	13.2	16.7	33.6	7.2	31.2
SZRD1	60.3	26.6	28.9	5.7	11.0	16.9	32.3	6.9	29.3
BCAP31	59.4	20.4	27.6	3.0	8.1	11.9	49.6	5.5	32.2
MCFD2	60.9	25.1	32.9	4.0	9.9	15.5	27.8	5.9	35.3
DAZAP2	61.4	29.5	34.8	4.8	10.2	15.5	22.9	6.0	32.1
TAGLN2	58.6	26.9	34.7	3.1	8.9	11.8	37.1	5.6	29.6
KDELR2	53.7	23.0	27.1	3.5	9.7	12.9	43.5	7.8	35.2
PIK3R2	59.9	22.9	29.8	9.5	16.2	20.7	28.2	5.7	23.3
RANGAP1	49.8	17.4	25.4	6.2	11.2	15.7	46.6	7.4	35.5
COPG1	58.4	22.9	26.9	4.5	10.3	14.6	39.0	6.3	31.7
IARS	59.6	21.0	26.0	2.3	9.1	12.2	48.1	5.2	31.3
GPRC5A	47.9	16.6	21.4	4.6	10.9	14.4	48.1	7.1	43.4
NNMT	82.5	31.9	42.5	2.8	7.5	10.0	22.3	2.7	11.9
FLNB	56.0	20.5	23.7	4.3	9.5	11.4	46.1	6.5	36.1
ZNF259	51.7	21.1	28.6	3.2	7.2	11.3	45.2	7.4	37.8
CAP1	58.0	22.5	29.0	3.5	8.8	14.3	36.2	6.0	35.2
NPDC1	63.9	24.4	33.6	8.2	11.9	16.3	29.9	4.9	20.3
IMP3	50.5	18.4	26.5	7.2	11.0	15.7	45.3	8.7	29.1
DHPS	58.6	23.6	34.1	4.7	9.7	12.5	33.9	6.3	28.8
VKORC1	62.4	18.3	30.2	3.6	8.4	12.1	46.8	4.9	25.6
VPS28	61.7	24.3	35.7	8.0	13.7	17.9	24.8	4.1	22.1
ATP5A1	60.0	21.7	31.2	2.7	9.7	12.4	44.9	4.5	24.8

UBE2V1	58.9	18.9	28.9	4.2	11.3	16.1	36.3	5.4	31.6
PCGF2	55.2	22.5	28.7	7.8	10.6	15.7	31.1	8.3	31.1
DHCR7	64.7	28.8	33.8	2.9	5.5	8.4	30.7	7.0	29.0
TLN1	58.8	19.0	24.9	5.1	10.6	13.8	43.3	5.9	29.1
CD55	46.8	19.0	25.7	4.3	10.8	15.4	45.7	7.0	35.7
COL13A1	45.9	17.8	23.2	9.2	17.5	21.8	41.3	5.7	27.7
XAB2	61.8	25.0	31.3	6.0	9.2	12.9	33.2	6.7	23.7
DPP7	56.3	24.5	32.0	7.6	12.6	18.1	27.9	5.8	24.7
ARL2	55.9	23.0	28.9	7.5	13.4	16.9	26.8	7.6	29.3
KARS	48.6	19.2	23.8	2.7	8.1	12.4	46.0	7.4	41.0
CLPTM1	62.4	24.9	31.6	7.0	11.6	15.2	26.1	5.8	24.4
NOB1	53.4	20.9	25.7	4.9	9.8	14.0	38.1	7.7	34.4
GNG5	49.8	23.1	27.0	3.7	7.3	14.1	34.4	7.0	41.8
CD9	52.9	24.4	30.0	3.1	8.8	12.2	36.5	6.5	33.8
FST	25.3	11.5	14.6	3.7	9.4	11.8	70.1	9.5	52.4
PLCG1	67.2	26.6	39.0	3.3	7.5	9.8	27.0	4.9	22.9
REXO2	51.0	21.1	28.5	4.2	9.8	13.0	43.8	5.7	30.7
HNRNPAB	44.0	17.7	20.9	4.5	9.6	13.4	49.9	8.6	39.2
ZNF358	62.7	23.2	28.6	8.3	12.2	17.4	30.8	5.4	18.9
CSNK1D	57.3	22.9	27.8	5.5	9.8	13.7	34.0	7.2	29.1
UBALD2	50.1	22.2	26.4	11.8	14.2	19.2	24.3	9.1	30.0
ARHGAP23	60.9	22.9	28.8	5.3	9.3	12.5	34.8	6.6	26.2
EHD1	51.2	20.5	23.8	7.0	10.7	15.3	35.8	8.1	34.9
DNM2	59.7	23.7	28.8	5.8	11.3	15.1	30.9	6.3	25.4
NOL7	46.4	19.3	24.8	6.9	10.8	13.3	44.8	6.6	33.9
JUNB	56.0	25.8	36.4	5.4	9.3	13.4	29.1	7.2	23.9
CCDC50	48.6	19.2	25.3	2.8	9.3	11.7	44.3	7.1	38.0
CHMP1A	62.5	23.1	30.4	4.8	9.3	12.1	33.4	5.9	24.5
ALKBH7	54.6	21.2	30.6	6.8	10.6	15.5	38.4	5.3	22.8
NAMPT	54.6	23.3	29.5	1.4	8.1	11.4	35.5	5.5	36.5
RNF10	54.3	19.6	25.0	3.6	8.6	11.7	42.1	6.4	33.8
CERS2	59.1	22.6	29.2	3.0	8.4	11.1	35.3	5.9	30.0
SMAD3	62.3	23.4	32.0	6.9	14.1	17.5	22.7	5.1	20.6
PPIF	59.8	20.8	30.0	4.2	7.3	10.8	39.1	5.8	26.6
HIST1H2AC	40.0	18.7	30.6	9.6	10.1	14.8	41.7	9.6	28.9
NGRN	52.1	20.6	26.6	3.0	7.8	10.8	44.5	6.1	32.3
DYNC1H1	51.6	13.3	19.9	4.9	10.6	12.2	52.0	6.1	33.2
SRM	44.2	18.0	19.7	8.6	11.8	18.0	41.2	7.6	34.6
SOD1	55.0	23.4	30.0	4.0	10.3	14.5	35.1	5.4	25.7
GNB1	58.8	19.8	26.1	4.9	10.1	13.6	36.3	5.9	28.0
CDK4	38.4	17.8	20.9	4.3	10.8	14.5	49.5	7.2	40.1
ECHS1	56.0	18.7	29.3	4.1	9.6	12.2	43.3	4.5	25.5
CCDC86	44.9	18.1	21.3	5.2	9.8	13.3	46.8	8.6	34.8
SF3B14	46.9	16.2	25.0	0.9	6.1	7.7	49.7	6.2	44.2
SNORA21	46.7	14.5	21.7	2.4	5.7	5.8	65.1	5.8	34.8
ATP6V1G1	48.2	23.1	32.2	5.3	10.1	13.4	31.5	7.3	30.9
MRPS6	57.3	23.0	32.9	1.9	5.1	6.4	40.0	6.5	29.1
LOC728752	60.7	16.0	31.1	8.0	11.7	13.1	32.7	5.0	23.5
HIST1H2BO	36.8	14.4	20.5	11.4	22.9	28.3	30.6	7.2	29.4
RELA	47.4	21.1	24.2	7.4	11.3	15.3	31.2	8.4	35.1
SSBP4	51.0	18.7	25.3	7.5	11.2	14.1	32.7	7.0	33.6
ARPC5	56.5	21.1	32.5	6.3	11.4	14.7	29.3	5.6	23.5
AP3D1	62.3	21.6	28.3	6.3	10.4	14.5	28.6	5.3	23.8
SF3B5	51.2	25.1	28.6	5.6	13.0	18.7	25.8	5.9	26.9
ILF2	44.3	18.1	22.9	2.6	7.2	11.9	48.7	7.0	38.1

PTPN11	57.0	18.3	25.5	2.4	8.4	11.3	40.7	4.6	32.6
ITGB5	62.9	24.2	29.5	4.0	8.4	10.6	32.5	4.9	23.8
HIGD2A	56.0	23.9	28.6	4.0	8.9	14.6	30.1	8.2	26.4
TRIM44	53.4	19.0	24.0	4.2	7.3	11.3	45.1	6.9	29.1
DPYSL2	66.6	23.4	29.9	4.0	8.9	11.7	30.2	4.6	21.1
CHRAC1	53.3	24.9	27.5	4.6	10.2	15.9	26.1	7.5	30.2
ATP5D	57.2	20.8	25.2	9.4	13.8	20.9	28.6	5.6	18.7
NGFRAP1	47.3	18.5	23.9	4.6	12.0	14.8	43.7	6.7	28.7
TGOLN2	56.4	20.6	29.2	5.5	12.1	17.2	27.3	5.1	26.6
CORO1B	51.1	19.8	26.6	7.3	12.8	17.4	32.9	6.2	25.6
WARS	49.6	20.6	24.1	4.5	10.5	15.1	33.9	6.6	34.9
NHP2L1	47.9	18.2	26.4	3.8	8.4	12.4	38.2	7.7	36.5
POLR2E	54.0	21.1	26.5	7.0	11.2	16.1	29.7	6.2	27.7
PXN	52.0	19.9	25.1	7.4	11.4	16.7	32.8	7.0	27.1
SRSF1	56.2	17.2	26.7	3.6	8.6	11.2	42.3	4.8	28.8
TFPI	70.2	27.2	35.1	1.6	8.7	11.7	20.1	3.5	21.2
GLUD1	52.6	19.4	25.4	3.4	8.8	10.3	44.7	5.5	29.1
ALYREF	48.1	17.4	25.3	6.1	10.6	13.5	37.9	8.5	31.7
SNORA52	49.0	22.4	33.7	5.9	7.9	6.5	52.0	4.6	16.9
TM9SF4	60.1	22.3	27.5	4.0	8.6	12.1	33.5	5.5	25.3
MLEC	53.4	18.8	23.6	3.4	9.1	12.3	41.5	5.9	30.5
TFRC	46.2	16.3	22.7	1.3	4.7	6.9	53.9	6.0	40.4
MTDH	44.3	15.9	20.9	2.0	6.6	8.6	54.4	7.2	38.4
SLC43A3	45.8	18.7	21.5	4.8	10.9	14.6	35.8	7.9	38.2
YAP1	51.6	17.6	24.6	4.6	9.6	13.9	35.6	7.0	33.6
PFKP	49.7	21.6	23.3	5.6	12.8	15.6	36.5	6.0	27.0
CLIC4	60.0	20.3	28.4	1.9	9.1	11.6	35.6	4.3	26.7
EPAS1	73.4	25.6	32.6	5.5	9.7	13.5	18.7	3.8	15.1
MRPL2	53.6	20.0	25.9	5.4	11.0	15.2	35.8	4.6	26.4
ECSIT	49.4	16.6	27.0	5.1	8.0	10.9	44.8	6.8	28.8
NEDD8	41.1	20.7	31.5	3.9	10.3	12.7	32.5	7.6	37.0
TMEM147	49.2	20.5	28.1	5.3	8.2	16.5	36.5	5.1	27.8
GABARAP	53.2	25.0	27.6	3.1	10.1	12.3	31.9	5.9	27.9
RAC2	62.2	23.7	32.5	7.0	13.1	17.4	19.3	4.1	17.5
MAX	45.0	19.0	21.7	6.1	10.4	14.8	33.5	7.6	38.4
ELK3	46.8	17.2	21.1	5.9	11.1	16.5	34.6	8.1	35.1
LAMB3	53.2	19.0	25.2	4.8	9.3	12.3	40.2	6.2	26.0
MZT2A	51.2	20.0	29.1	8.2	12.8	17.9	33.1	3.8	20.1
DNAJA1	54.0	18.4	22.6	1.3	6.2	7.6	45.7	5.6	34.4
CD99L2	66.8	27.3	36.6	5.8	11.5	17.2	14.5	2.9	13.3
VDAC1	50.8	21.3	26.4	3.3	8.9	12.3	34.6	6.0	32.0
RPL7L1	45.2	18.6	24.1	1.7	8.8	10.2	44.1	4.9	37.0
HNRNPL	43.8	17.5	22.5	5.5	13.1	15.2	39.1	6.3	31.5
RABAC1	52.4	21.2	31.5	9.8	14.4	19.9	26.4	3.4	15.4
TADA3	57.2	22.0	27.2	4.6	10.9	13.8	32.6	4.7	21.3
FSTL1	49.5	20.2	25.3	2.7	7.5	10.0	43.8	5.9	29.3
H2AFY	52.2	17.3	25.9	3.2	6.5	9.0	41.3	5.2	33.4
HNRNPM	55.4	19.7	26.0	4.4	9.0	11.6	38.7	5.3	23.8
LOC101928600	62.3	11.6	29.1	4.4	9.0	10.5	43.9	3.7	19.5
MRPL49	52.1	21.3	30.2	4.0	8.9	13.1	30.2	4.6	29.4
SLC5A3	66.6	20.6	32.5	1.2	4.5	5.2	36.7	3.9	22.4
SUPT5H	50.0	19.4	24.1	5.5	9.6	12.5	35.9	6.7	29.8
PHGDH	49.7	17.1	22.6	3.8	8.2	10.3	46.0	6.6	28.9
HMGN2	52.9	18.1	29.7	3.5	11.2	15.7	33.9	4.7	23.4
SLC39A7	51.1	18.7	23.9	3.0	6.7	10.2	41.3	6.8	31.6

DDB1		49.9	19.7	23.6	3.3	8.9	11.9	37.2	6.5	32.0
UQCRQ		48.4	20.1	27.3	3.3	9.0	12.5	42.6	4.4	25.3
MPST		43.7	16.5	20.4	8.9	11.5	17.0	40.8	8.1	25.6
HIST4H4		52.0	21.7	34.2	5.3	12.3	14.3	26.9	3.7	21.7
CTSZ		56.3	23.2	31.0	4.7	9.4	14.2	25.7	5.4	22.4
SLC4A2		54.9	21.2	28.9	4.7	7.8	10.8	34.2	5.5	24.1
PSMD8		49.6	20.3	27.7	5.8	9.7	15.1	27.1	6.5	30.0
CNPY3		48.4	19.5	24.2	4.1	7.5	9.8	42.3	6.0	30.3
HEXIM1		54.3	21.8	26.0	5.8	7.7	11.5	33.2	6.7	24.8
FADS1		50.1	20.4	24.6	3.3	8.2	12.0	33.4	6.9	32.7
RANBP1		43.5	16.0	21.2	4.6	8.2	14.9	38.3	8.3	36.5
CDC42EP3		41.3	16.5	18.7	5.6	11.0	15.5	40.3	7.0	35.3
PRDX3		53.0	19.7	32.4	2.7	8.4	11.0	33.0	4.6	26.3
C11orf58		46.3	18.1	24.6	2.0	9.5	10.1	43.4	6.0	31.2
CNIH		44.6	22.9	31.2	2.9	8.0	14.4	26.7	6.5	33.6
NOMO1		51.6	19.1	24.1	3.4	8.0	11.4	38.5	5.6	29.2
AKT1S1		52.1	21.6	25.8	7.7	11.0	14.4	28.6	7.0	22.5
TRNV		54.4	27.8	19.1	9.0	11.2	18.0	19.1	6.8	24.6
C6orf223		7.4	30.5	21.3	31.2	18.8	13.3	19.3	33.0	15.0
NDUFA2		50.3	21.4	35.1	4.6	9.1	12.1	30.6	5.0	21.3
GIPC1		53.7	22.5	30.0	5.9	9.7	12.2	28.1	4.9	22.2
SF3A2		47.0	19.1	22.0	6.4	9.0	14.1	31.8	8.3	31.4
NINJ1		56.9	27.5	33.3	7.2	9.6	14.2	17.6	4.8	18.0
SREBF1		63.5	24.1	29.9	7.0	11.4	15.7	18.9	3.6	15.0
DEK		45.2	16.5	19.7	2.4	7.6	9.0	40.9	5.9	41.8
GPI		49.1	18.2	24.2	4.6	9.3	12.7	34.1	5.9	30.9
SLMO2-ATP5E		44.9	18.3	24.5	2.6	7.3	9.9	40.7	5.8	34.9
TNFRSF1A		55.1	24.8	32.2	4.6	8.4	12.4	24.2	4.4	22.5
PGD		52.2	22.4	26.9	3.0	6.2	10.4	32.3	6.2	28.9
CLPTM1L		48.1	19.0	24.0	3.4	7.1	8.7	43.8	5.7	28.7
SPRY2		37.2	15.4	20.4	6.7	11.1	15.4	39.8	8.1	34.3
CIRBP		50.4	21.0	24.6	3.6	8.1	13.2	32.8	5.3	28.9
TGFBR2		53.7	19.0	24.0	3.4	8.0	10.1	36.9	4.9	27.9
AIP		48.4	19.6	26.4	5.1	8.7	13.5	34.1	6.0	25.9
HSPA1B		59.0	20.2	26.0	5.2	8.1	10.0	35.0	6.5	17.8
MRPL41		48.3	20.0	26.5	5.4	8.8	12.4	34.7	5.1	26.7
SNRNP200		49.1	17.1	21.3	3.4	7.8	10.7	40.9	5.8	31.5
BRD2		47.5	18.4	23.0	3.5	7.6	9.9	36.3	7.3	34.0
RGMB		39.0	12.9	17.6	5.6	9.3	13.8	44.6	7.3	37.2
MRPL12		41.9	17.2	23.1	4.6	9.9	14.2	44.9	5.6	26.0
	44819.0	45.5	18.8	26.2	1.1	4.3	7.7	52.3	3.1	28.3
CSNK1A1		44.6	16.7	21.9	2.5	8.2	10.1	43.7	5.7	33.8
C15orf40		58.8	12.3	26.4	3.0	8.4	8.9	45.4	3.4	20.5
CSK		46.0	18.2	23.1	4.1	7.6	10.9	39.1	5.7	32.3
NEK6		58.3	21.1	31.1	4.9	10.9	14.7	21.7	4.0	20.2
EXOSC5		42.7	17.1	21.0	4.0	8.1	11.1	49.2	5.6	28.1
RAB10		50.8	18.3	25.1	1.7	7.9	10.3	32.4	5.9	34.1
LEPREL1		47.8	18.8	23.3	3.8	8.9	12.6	39.9	5.9	25.1
GUK1		48.5	21.6	30.2	6.3	9.6	15.1	27.9	5.5	21.3
VCL		56.8	20.3	26.5	3.2	9.2	11.5	31.8	4.0	22.8
PTP4A2		49.5	18.9	24.6	1.9	7.6	10.4	36.4	4.2	32.5
MANBAL		48.1	22.6	24.6	5.9	11.4	15.9	23.4	6.9	27.0
HIST1H3H		36.9	14.7	22.2	9.2	14.9	19.9	36.5	5.4	25.7
TMA7		46.6	18.5	29.4	4.9	9.2	10.6	31.5	6.8	27.8
ARHGAP29		47.2	17.5	23.6	2.3	9.3	12.9	35.3	4.8	32.3

SNRPD3		49.9	19.2	27.1	4.4	7.8	11.8	31.6	6.2	27.2
AP1M1		48.4	21.5	26.2	5.4	10.1	13.8	26.5	5.2	27.8
SF3B4		45.0	18.8	20.7	6.2	10.4	14.9	30.6	6.8	31.5
MRPS2		43.4	17.9	21.7	4.9	9.1	11.8	36.7	8.2	30.8
ATP2A2		47.1	14.7	19.4	2.4	9.0	8.4	46.4	5.2	31.8
ADAM15		44.9	19.3	23.8	5.4	9.4	14.1	35.7	5.7	26.0
C19orf70		44.3	21.6	28.7	6.5	10.7	16.8	28.6	4.9	22.1
RAD23B		40.5	17.9	22.2	3.2	8.1	11.7	34.4	7.3	38.6
SLC7A1		44.4	15.4	18.3	3.6	6.9	9.9	45.8	6.1	33.8
SNORD32A		50.1	17.2	16.0	7.9	13.4	19.6	25.1	6.7	27.9
GSTP1		51.8	20.7	25.1	5.6	11.6	16.3	27.3	4.2	21.0
HSPE1		47.3	21.5	29.2	1.6	7.1	9.2	28.2	5.4	34.0
AP2A1		55.1	19.6	26.7	6.6	11.0	14.6	26.7	4.4	18.8
BAG3		52.5	18.8	23.7	7.6	12.2	16.1	24.8	6.0	21.7
STRN4		47.3	18.9	24.4	5.9	9.1	13.6	31.4	6.6	25.9
EIF4A3		46.0	16.5	20.9	2.8	8.0	10.8	40.3	5.2	32.7
H2AFX		39.7	16.3	19.8	8.9	13.3	18.6	31.4	7.5	27.5
INSIG1		55.4	23.4	26.8	4.0	7.0	10.1	30.8	4.4	21.0
SDHD		52.4	20.5	29.6	1.7	7.0	10.2	33.9	3.8	23.5
SART1		47.5	17.4	22.7	5.6	8.9	10.9	38.8	5.9	25.0
DBI		42.1	23.7	33.7	3.9	8.1	15.9	17.1	6.2	32.1
SF3B1		54.4	20.5	25.9	2.3	5.5	9.5	32.7	4.6	27.0
MYO1C		45.5	16.4	21.2	5.8	11.1	14.8	35.8	5.8	26.1
ZNF598		50.8	18.9	22.8	7.6	10.4	14.0	29.2	6.1	22.6
RDH11		52.4	17.6	27.9	2.1	7.0	9.5	31.8	4.6	29.1
MID1IP1		53.9	19.2	23.2	4.1	7.8	9.1	33.9	4.9	24.9
	44806.0	49.6	19.8	25.9	2.7	8.1	10.8	32.2	4.6	27.1
CTTN		41.4	14.4	19.3	4.3	9.7	12.1	42.2	5.8	31.6
ANXA11		52.0	20.6	26.3	4.0	9.1	13.6	27.9	4.8	22.5
PPP1R18		51.1	19.3	24.0	4.2	8.3	11.6	31.5	5.5	25.4
POLR2J3		50.3	18.5	26.2	6.0	10.8	15.1	23.4	5.2	25.1
SCRIB		48.5	16.7	23.2	5.6	8.7	11.3	36.6	5.7	24.1
VTI1B		46.8	19.7	26.7	3.3	10.2	11.5	29.4	4.4	28.3
PDLIM1		52.0	19.7	24.9	3.5	9.4	10.7	29.6	5.0	25.4
NDUFB11		43.3	16.3	24.1	4.7	9.0	13.1	37.3	5.1	26.9
TOMM34		43.9	15.5	20.6	5.2	12.1	16.2	33.8	5.4	27.3
TMUB1		45.2	20.1	24.2	6.3	10.7	13.9	28.4	7.0	23.9
CBX6		44.3	17.8	19.5	9.5	10.9	13.9	29.8	7.5	26.5
HNRNPA3		43.9	18.8	21.3	2.5	7.6	12.2	39.5	5.3	28.3
PSAT1		37.0	14.5	17.4	2.1	6.3	8.3	51.4	5.6	36.8
TOMM20		48.6	19.2	22.8	2.1	7.9	8.2	36.0	4.4	29.9
TFG		41.4	16.4	19.7	3.8	7.5	12.4	32.4	8.5	37.2
NDUFS6		47.3	19.8	24.4	4.4	9.3	13.2	30.5	5.5	24.8
NDUFB10		45.7	18.3	26.9	5.5	9.2	14.6	24.7	6.9	27.1
TCP1		38.9	14.7	19.6	1.0	5.5	7.0	53.0	4.6	34.2
UBAP2L		49.8	18.0	24.8	4.2	10.9	13.6	25.0	4.8	27.2
TMED3		48.4	17.9	23.5	4.0	8.8	11.0	33.0	4.6	26.9
HADHA		44.1	17.8	22.1	2.6	8.1	11.3	38.7	4.8	28.4
VHL		54.6	19.8	26.8	4.4	7.2	10.1	31.4	3.9	19.7
ZNF664		36.9	14.2	19.9	3.7	7.9	11.1	40.7	8.0	35.3
NOP16		38.1	14.4	18.4	2.2	7.1	9.3	51.0	6.0	31.0
PLXNB2		57.6	20.6	27.2	7.7	12.0	15.0	20.0	3.4	13.8
OSMR		52.8	18.2	24.5	1.2	4.4	8.9	35.1	3.5	29.0
F3		40.0	17.9	21.5	3.6	10.2	13.8	36.0	5.4	29.2
SCARNA6		44.4	17.1	28.5	2.6	6.1	8.2	39.9	4.3	26.4

SRPR	41.7	16.6	20.3	2.8	7.2	9.6	40.9	5.5	32.9
PTRHD1	45.1	19.9	30.4	7.3	10.9	16.3	23.0	5.7	19.0
PPP1R13L	42.5	15.4	19.2	9.6	12.3	15.7	32.6	6.1	23.6
SCAMP2	51.3	19.6	23.8	5.3	9.5	14.0	25.9	6.0	21.7
SCAF1	47.3	16.9	21.6	6.6	9.0	11.5	31.7	6.6	25.5
PPA1	42.2	16.7	22.5	1.3	6.4	8.5	41.6	5.8	31.6
EIF3K	37.0	13.0	19.7	4.6	11.5	16.6	39.0	5.4	29.7
CDK5RAP3	52.3	19.8	29.1	4.1	8.8	12.9	28.9	3.2	17.3
PSMC4	45.0	18.3	23.5	4.0	8.5	10.9	34.3	4.8	26.8
DLGAP4	46.3	18.8	23.6	5.4	9.4	12.8	24.0	7.0	28.6
PSME1	49.9	20.2	27.1	2.5	8.8	10.5	30.3	3.9	22.6
MAP7D1	44.8	16.7	21.4	7.5	9.9	12.0	29.8	7.5	26.3
RAB34	44.9	20.2	25.4	3.5	9.1	12.5	32.0	4.5	23.5
LTBR	52.3	21.1	26.0	4.4	8.4	11.9	24.4	5.5	21.8
PA2G4	39.1	15.3	18.4	2.6	8.1	11.9	38.2	6.7	35.3
INPPL1	52.1	21.0	28.3	4.4	8.1	11.3	25.5	4.7	20.1
VDAC2	39.4	17.8	21.6	3.7	6.1	9.6	41.0	6.4	29.8
FOSL2	45.1	20.5	23.8	6.9	11.7	15.1	23.1	6.1	23.1
JDP2	33.3	12.6	17.9	3.9	7.2	9.6	44.6	9.1	36.8
PVR	41.9	15.4	21.0	4.9	9.4	12.4	35.5	6.5	28.0
CIB1	50.8	19.1	25.8	3.3	6.9	10.1	31.9	5.3	21.6
POLD2	42.9	18.4	21.5	5.7	11.8	16.2	27.4	5.5	25.2
CLEC2D	41.0	16.1	22.1	2.9	6.7	9.6	37.9	5.8	32.7
ARL6IP4	48.4	21.6	27.0	5.4	11.8	14.5	23.2	3.5	19.2
NR1H2	49.6	20.9	23.4	4.4	8.5	12.9	28.8	5.1	21.1
MATR3	49.4	17.8	22.4	1.9	6.5	9.3	33.8	4.5	28.9
SHISA5	45.6	19.0	26.7	9.3	11.3	15.2	19.1	4.4	23.9
NELFB	53.1	20.4	25.8	4.4	8.4	11.8	27.4	4.3	18.7
FJX1	37.4	15.8	19.4	6.8	10.2	13.6	42.9	5.6	22.5
SNORA61	31.4	24.0	20.2	13.0	10.1	15.2	26.8	8.3	25.1
BRK1	52.7	22.6	32.0	3.2	8.2	11.5	18.2	4.8	20.8
EIF3B	43.8	15.8	19.3	4.7	9.6	12.2	38.6	4.5	25.4
SLC39A14	34.5	11.9	16.7	2.6	6.1	8.2	51.1	6.8	36.1
SNHG6	46.9	18.4	27.7	2.6	7.0	9.6	31.4	4.7	25.5
SNORA22	29.9	29.6	17.2	3.5	13.2	9.7	16.7	10.6	43.7
TPM3	46.0	17.9	23.8	3.8	9.5	12.9	31.0	4.5	24.6
CLSTN1	49.0	16.9	21.3	4.8	8.3	12.2	32.3	5.4	23.7
PDAP1	48.1	19.0	26.6	5.7	10.5	15.5	20.4	5.0	23.0
AGAP3	47.4	18.0	23.1	4.8	7.4	9.1	32.3	5.7	25.8
CHERP	46.1	18.2	21.3	6.7	9.4	12.4	25.7	7.3	26.1
YARS	43.6	14.4	19.2	2.7	7.6	10.8	41.3	5.0	28.5
IMMT	47.9	17.0	20.2	2.4	7.6	10.4	35.2	4.7	27.8
PYCR2	45.5	20.2	26.0	3.8	9.5	11.5	27.6	4.2	24.7
VPS37B	44.6	17.0	19.5	7.0	10.8	14.4	28.1	6.2	25.1
SUPT6H	51.1	16.1	21.6	3.6	7.5	10.0	32.8	5.3	25.0
SH2B3	44.5	14.4	19.0	6.4	9.8	13.8	35.9	5.6	23.2
S100A16	37.9	16.1	20.8	5.6	11.5	15.0	27.4	7.7	30.6
U2AF1	43.0	16.7	20.2	2.6	5.8	8.2	37.9	6.5	31.6
DLST	49.7	20.1	24.8	2.3	7.1	11.3	29.5	4.5	23.3
ERCC1	44.2	15.9	20.7	5.6	12.7	17.3	29.1	5.5	21.5
UBE2L6	48.4	19.3	26.2	6.5	10.3	15.5	24.0	4.8	17.6
FIBP	40.7	16.3	21.1	6.4	12.6	16.6	31.3	4.9	22.6
SDCBP	48.4	19.2	22.8	1.4	6.0	9.2	34.9	4.9	25.6
MEA1	40.7	18.6	27.3	4.4	8.0	11.6	31.1	6.4	24.2
SEMA7A	35.0	14.6	17.9	5.8	10.5	16.8	34.0	6.9	30.7

DPP9	49.5	19.3	24.5	4.8	9.1	12.7	26.0	5.0	21.3
DDAH1	45.5	16.2	19.8	3.4	6.7	9.0	37.9	5.9	27.7
PQBP1	43.9	15.8	24.2	4.2	7.2	10.0	31.6	6.6	28.5
SSH1	52.7	16.2	23.5	4.4	8.8	11.1	27.0	5.4	22.6
TMX2	42.7	17.7	21.6	3.8	8.3	12.8	32.9	4.8	27.1
DUSP14	39.2	16.0	20.6	2.6	6.5	10.9	37.9	7.1	30.5
ATP6V0D1	49.2	20.7	23.9	3.8	8.5	13.2	25.3	5.6	20.8
ATP6V1F	41.7	21.7	26.7	5.0	10.9	15.4	20.4	5.9	23.4
SSR1	40.7	15.4	19.5	1.9	6.3	9.3	40.2	5.9	31.6
SNX3	52.0	19.6	27.7	4.1	7.6	10.5	24.9	3.8	20.7
LMAN1	45.5	14.9	19.2	2.0	6.6	8.2	38.9	5.4	30.0
KRT19	46.7	20.9	24.6	4.3	6.8	9.3	30.9	5.1	21.8
FAT1	51.3	14.0	21.2	2.2	6.4	8.6	36.3	4.4	25.7
CEBPD	60.5	26.6	34.1	7.2	7.9	9.1	12.4	3.5	8.9
CRTAP	47.4	18.0	23.7	4.9	9.5	13.2	30.1	4.1	19.3
CHMP3	51.7	20.3	22.3	2.4	8.1	10.4	30.6	4.2	20.1
RPS19BP1	44.8	16.8	22.1	4.2	7.3	8.7	38.2	4.6	23.2
POLR1D	43.8	18.7	27.5	2.1	4.9	9.9	30.1	5.0	27.7
GPS1	43.8	17.3	23.3	6.2	10.3	15.7	25.4	4.7	23.0
RHOF	47.2	19.1	23.5	4.5	6.7	10.5	29.1	5.6	23.4
DDX3X	41.3	15.1	19.0	1.6	6.1	8.1	42.6	5.0	30.6
NPC1	45.1	15.9	19.7	2.1	5.9	7.7	39.4	5.3	28.2
LOC101926889	57.8	11.9	26.1	2.1	7.0	8.6	32.9	2.5	20.2
CSRP1	45.1	17.9	21.4	3.6	9.0	13.2	28.3	4.8	25.8
TRAM1	35.3	13.7	20.7	1.5	6.1	8.0	39.2	7.0	37.4
AAMP	38.7	15.6	19.6	2.8	7.9	9.1	43.6	5.0	26.8
DDX21	34.8	13.2	16.3	2.0	5.9	8.0	42.3	6.3	40.1
PEMT	41.0	18.8	24.3	5.7	10.0	14.8	27.3	4.3	22.5
STAU1	46.0	16.3	21.3	2.6	7.2	9.5	30.0	6.1	29.3
VEGFB	44.4	16.2	20.1	7.6	10.7	13.0	28.2	4.9	23.1
SPCS2	41.6	15.2	22.4	1.6	5.4	7.1	38.0	4.2	32.7
C8orf33	44.7	17.4	22.7	5.2	10.8	14.4	29.3	4.3	19.5
PSMD4	42.2	16.1	19.7	3.5	8.5	12.1	33.5	4.9	27.5
SNAPC1	40.5	17.2	20.5	2.8	10.1	15.3	29.6	4.8	26.8
CAV2	46.8	15.9	24.7	5.5	8.9	9.8	29.2	4.3	22.5
RSL1D1	39.4	15.6	18.9	2.1	7.2	9.9	34.7	6.0	33.7
RBMS2	39.4	14.0	19.6	2.5	7.3	11.0	30.3	6.8	36.3
SDF4	48.8	19.8	25.2	4.4	7.8	10.8	26.1	4.8	19.4
APH1A	44.9	16.4	22.0	4.0	8.8	11.9	30.9	4.4	23.7
RHOB	45.1	18.4	25.3	4.9	6.9	10.7	31.1	4.2	20.4
C9orf89	49.9	20.1	23.5	5.9	10.7	12.9	23.4	4.4	16.0
CD97	48.5	18.3	24.3	4.5	8.9	11.7	25.6	4.2	20.8
AKAP8L	39.9	15.8	20.1	3.6	6.1	8.0	35.7	6.6	30.9
YY1	37.9	14.6	18.5	7.3	8.6	8.6	34.6	7.4	29.3
CYB5A	43.4	20.2	27.7	2.7	7.6	9.1	29.9	4.7	21.3
ARHGEF28	40.3	13.5	18.8	2.6	6.4	9.2	39.8	5.7	30.4
PRKCA	46.9	13.5	21.6	4.6	8.2	9.9	34.2	4.4	23.1
KLHDC3	45.7	18.4	24.6	3.1	7.1	11.1	27.3	4.6	24.3
CDC34	41.8	17.9	19.5	5.2	7.5	11.1	29.4	6.9	27.0
ST13	46.5	17.6	22.1	2.1	7.7	10.1	30.6	4.4	25.1
NCBP2	40.4	16.4	19.0	2.6	6.8	9.4	34.9	5.2	31.0
TMEM184B	47.1	19.9	25.0	6.4	9.0	13.0	18.4	5.6	21.4
NCSTN	57.9	18.5	22.7	2.5	7.7	13.0	20.6	3.3	19.4
IRS1	36.0	13.4	18.5	6.3	10.2	13.3	32.2	6.6	29.0
TMBIM1	52.5	22.5	26.0	3.5	7.6	11.0	21.2	4.1	17.0

CHPF	52.7	19.0	23.6	7.4	10.4	14.8	19.7	3.6	14.2
PSMC3	41.1	16.7	21.5	3.4	9.1	11.0	30.1	4.6	27.5
PSMC5	40.5	15.1	19.0	3.2	8.9	11.5	33.4	5.6	27.7
GPSM3	40.6	16.9	20.5	7.0	10.4	14.4	26.0	6.5	22.7
TRA2B	38.7	16.7	19.5	3.0	7.2	10.4	32.5	6.0	30.7
YIPF3	44.5	18.4	23.1	4.2	7.8	10.0	28.4	5.1	23.3
ETFA	42.5	16.9	26.0	2.7	7.8	10.2	31.3	4.2	22.9
TOP1MT	42.1	17.1	19.3	4.0	8.0	10.2	31.1	6.1	26.4
UBE2I	42.3	18.1	22.6	2.8	7.7	11.7	25.1	6.1	27.8
ZDHHC7	51.2	18.2	22.5	2.7	7.1	9.0	28.2	4.1	20.8
ATP13A2	51.0	19.4	25.1	6.1	10.0	13.4	20.5	3.3	14.8
C20orf24	44.2	18.0	23.4	4.4	6.5	7.9	30.4	5.3	23.5
POLR2I	38.7	16.1	23.2	7.5	10.3	15.1	26.3	5.6	21.0
TNNT1	40.7	18.0	21.3	4.0	8.7	12.3	29.1	5.5	23.8
PPP4C	44.0	17.8	26.0	3.5	7.8	10.2	29.3	4.2	20.6
DERL1	45.7	18.3	25.1	3.4	6.2	8.4	29.1	4.1	22.8
GDF15	45.1	19.9	22.9	4.4	5.1	9.3	30.1	4.8	21.4
MRPS12	36.0	16.8	22.8	4.5	8.1	12.0	32.7	5.8	24.2
FOXK2	42.5	16.1	18.1	4.5	7.6	10.1	29.4	6.6	27.9
UBE2D3	44.0	17.3	21.4	0.8	5.6	9.0	26.9	5.6	32.1
APMAP	49.8	18.3	23.9	3.1	6.6	10.0	26.8	4.0	20.3
REPIN1	39.7	14.7	17.1	5.3	7.7	10.7	33.8	7.0	26.7
GLTP	42.7	16.9	19.7	5.3	9.5	14.0	26.4	5.8	22.3
ORAI1	41.4	18.0	21.9	9.1	12.6	19.3	16.9	5.7	17.4
LENG8	45.1	18.2	25.0	6.4	11.3	13.7	20.2	4.1	18.3
SNORA71B	32.8	18.9	21.2	6.6	9.0	6.8	31.5	7.0	28.5
TMEM66	51.3	19.4	26.4	2.5	7.4	9.3	25.6	3.5	16.7
HK1	45.8	17.8	22.5	3.5	7.2	10.7	27.7	4.9	21.8
FXR1	41.9	17.1	22.0	1.5	5.0	7.3	34.6	3.9	28.7
NUMA1	46.1	17.0	21.4	5.1	9.1	11.4	25.6	4.9	21.3
RTN3	44.6	16.3	23.2	1.9	4.3	6.3	28.7	5.9	30.5
LRPAP1	46.4	18.2	22.9	4.6	8.1	10.2	27.8	4.4	19.0
TOMM5	37.9	16.7	23.1	3.7	7.6	10.0	32.1	5.4	25.0
ATRAID	43.3	19.3	26.3	4.0	7.9	12.3	25.6	3.9	19.0
MAP1LC3B	39.4	21.1	25.3	3.7	6.6	9.9	25.7	4.3	25.4
OS9	46.4	18.4	26.4	3.2	8.0	9.8	24.1	4.2	21.0
TRIO	42.6	12.6	19.6	4.1	7.6	9.4	34.2	5.2	26.2
STUB1	43.1	17.1	22.2	5.7	10.8	13.4	24.3	4.1	20.4
ATN1	40.6	16.6	20.7	5.6	8.6	11.4	25.1	6.5	25.9
MRPL11	43.7	17.8	22.1	3.8	9.0	12.0	28.7	3.8	20.0
GCN1L1	44.7	14.5	21.1	3.8	8.1	10.1	33.1	4.1	21.4
JTB	41.0	14.9	20.9	1.8	6.5	8.7	38.3	4.7	24.1
CHD3	46.4	16.2	22.2	3.9	8.1	10.4	26.9	4.6	22.0
GHITM	42.9	17.8	26.7	1.3	7.1	8.0	30.4	3.6	22.6
SNORA68	36.2	22.3	26.2	4.5	9.5	9.0	26.9	4.5	21.1
USP39	44.2	16.2	22.1	2.6	6.3	9.0	30.9	4.9	24.0
C19orf33	34.8	16.2	22.3	7.3	14.4	16.9	23.8	4.6	19.7
FNDC3B	51.5	17.8	22.7	2.1	5.9	8.2	25.1	4.4	22.4
SNORA64	49.6	15.2	13.7	2.2	6.6	5.3	50.8	2.9	13.6
ARHGEF1	37.9	16.0	20.2	5.2	8.2	11.2	32.1	5.2	23.8
LOC101926898	52.9	9.9	25.3	1.4	6.2	7.3	33.2	2.4	21.1
PCNP	42.5	16.9	27.0	1.9	6.9	9.4	26.4	4.5	24.2
CASP4	41.9	18.8	23.0	2.0	7.8	11.7	24.8	4.2	25.5
NAA20	44.6	13.7	24.0	1.4	6.1	7.8	32.6	4.1	25.3
FAM50A	43.5	16.8	22.5	4.4	8.0	11.8	24.8	4.2	23.5

PRPF31	45.7	17.0	22.5	5.2	9.1	11.7	23.9	4.5	20.0
TNFRSF10B	47.0	17.3	22.9	2.9	6.6	8.2	29.8	3.6	21.2
C14orf166	42.7	18.3	23.4	1.6	7.5	9.1	27.1	4.2	25.6
KIAA1191	51.7	19.4	20.7	3.6	7.4	11.4	22.1	3.9	19.2
UBE2N	44.3	15.5	21.3	1.9	6.2	9.6	30.1	3.5	26.9
TBC1D9B	48.5	17.4	23.1	4.7	8.5	10.6	24.5	3.8	18.0
VCP	44.2	15.2	19.4	2.1	6.4	8.3	36.1	3.8	23.7
LRRC20	33.6	13.6	21.0	5.2	8.3	10.9	31.7	7.1	27.8
NUCKS1	39.4	16.5	17.9	1.9	7.4	10.6	32.7	4.9	27.8
RPP25	40.3	14.1	17.1	6.4	9.0	12.8	32.1	5.1	22.0
KEAP1	42.0	14.9	19.8	4.0	8.1	10.0	33.3	5.2	21.5
PAK4	44.3	18.4	24.8	4.7	7.2	10.0	26.0	4.9	18.3
ENC1	42.2	15.9	19.2	3.4	8.5	11.4	30.7	4.2	22.9
MAP2K7	45.4	16.0	21.6	4.7	8.9	10.9	24.2	5.3	21.5
HMGA2	39.4	13.7	19.5	7.2	11.7	12.7	26.3	5.6	22.3
CLDND1	43.4	18.3	26.7	2.3	8.5	11.4	24.2	2.8	20.5
FDFT1	48.0	20.8	28.9	1.3	4.1	6.0	26.6	3.6	18.8
SHARPIN	41.6	15.5	21.5	6.4	11.4	14.7	24.1	4.9	18.2
RTFDC1	41.4	16.0	21.3	3.0	6.9	9.5	31.7	4.0	24.2
HN1	42.3	16.5	21.4	3.2	7.7	10.6	30.3	3.8	22.0
TMEM230	43.9	15.7	24.6	2.9	8.6	13.8	21.8	4.3	21.9
GORASP2	40.7	15.0	17.8	3.2	5.3	9.2	26.8	6.8	32.6
RBM3	41.8	16.8	22.7	3.7	10.7	12.8	21.2	3.5	24.2
SNORA76	44.4	15.8	23.9	5.5	3.0	6.8	35.4	3.0	19.7
ALCAM	39.6	17.2	20.9	1.3	4.7	9.0	32.6	5.0	27.0
KISS1	19.4	7.9	9.1	2.7	3.3	4.8	63.6	7.2	39.2
PPM1G	37.9	14.6	19.7	2.9	6.8	9.1	32.0	5.6	28.6
GOLM1	53.9	22.1	27.3	2.6	6.5	8.5	18.3	2.7	15.1
SPRY4	38.7	14.3	20.1	6.3	8.1	12.3	28.2	5.9	23.2
HMGB2	36.7	15.0	17.3	2.0	8.9	9.9	33.6	4.4	29.3
NPM3	35.9	13.1	21.0	2.7	8.2	9.5	38.5	4.8	22.9
PIM3	37.5	13.4	16.3	5.0	6.9	11.2	39.6	5.5	21.4
YDJC	37.4	13.8	18.8	4.7	5.2	10.8	41.1	6.4	18.4
PLAC8	40.9	17.3	21.3	2.2	8.5	11.4	29.4	2.7	22.9
RND3	39.6	17.3	20.3	2.3	7.2	10.1	32.1	4.8	22.8
RBM42	42.9	16.6	19.3	4.8	8.0	10.4	29.2	5.2	19.9
CARM1	42.9	16.9	20.5	3.6	7.3	10.5	25.7	5.4	23.5
NFKBIB	40.9	12.6	18.8	4.2	7.0	8.9	34.8	5.5	23.2
TCF3	40.8	16.2	19.5	5.8	8.2	9.6	25.1	6.9	24.1
MGLL	41.4	16.0	20.7	2.4	6.9	8.6	26.2	5.5	28.2
PPP1R9B	46.5	17.2	21.5	4.3	6.8	8.7	27.3	4.1	19.3
CKS1B	35.2	14.6	17.6	2.1	6.9	9.9	38.1	4.5	26.7
LTBP4	44.2	17.1	23.1	6.5	9.8	12.9	22.0	3.8	16.1
CDC42	43.1	15.7	20.9	1.3	5.5	8.2	30.6	4.2	26.1
DDX17	36.5	13.0	17.4	3.1	7.1	9.8	36.6	5.5	26.3
PIEZO1	36.9	14.0	19.4	6.4	10.3	13.7	27.6	5.2	22.0
ARL2-SNX15	44.2	16.9	22.3	4.3	8.2	11.6	24.2	4.1	19.6
LONP1	45.1	17.2	20.5	5.1	7.8	10.9	25.9	4.5	18.3
FLOT1	37.6	15.8	19.4	4.8	9.8	13.4	30.7	4.0	19.6
PDCD4	43.3	15.2	20.0	1.5	5.9	8.0	33.5	3.9	23.8
STOML2	39.6	16.1	23.2	2.7	8.6	8.7	30.3	4.2	21.5
RAP2B	32.7	12.2	18.5	5.5	9.1	13.3	34.5	5.7	23.5
SH3BGRL3	30.8	17.7	20.1	5.7	9.1	14.4	26.2	5.6	25.4
RAB8A	43.4	17.0	20.2	2.1	7.9	9.7	23.7	5.4	25.3
WDR45B	41.9	16.5	20.6	3.1	7.8	10.5	25.1	4.9	24.2

SRSF5	38.2	15.0	20.8	2.3	6.7	10.0	32.1	4.4	25.2
NCEH1	35.7	15.0	20.2	3.2	7.7	10.5	31.9	5.5	24.9
CTNNB1	43.9	14.6	20.2	2.3	6.6	8.3	32.7	3.6	22.3
SIGMAR1	42.3	17.7	21.9	5.1	10.0	14.4	23.7	3.3	16.1
RBMX	42.1	16.4	16.6	2.5	6.2	10.9	27.7	5.0	26.9
PRRC2B	42.7	13.8	18.9	3.6	7.3	9.1	29.2	5.0	24.4
ASXL1	41.9	15.0	20.0	2.4	6.9	8.4	31.1	4.3	24.1
BLOC1S5-TXNDC5	35.7	12.2	16.7	1.7	4.9	7.2	41.4	4.8	29.2
NDUFV1	41.9	16.3	22.9	4.2	9.2	11.4	26.8	3.2	17.5
SAMD1	35.7	15.4	18.4	4.4	7.2	10.3	29.7	6.4	25.7
GDI1	42.3	18.4	26.1	2.8	6.8	9.4	23.4	3.8	19.9
CALM1	37.9	14.2	18.8	1.4	6.0	9.4	37.1	4.8	23.2
HIF1A	49.5	18.8	22.3	1.2	5.0	7.0	23.7	3.8	21.4
ITPRIPL2	38.5	12.1	17.3	5.1	8.3	11.0	36.3	4.6	19.5
WRNIP1	38.6	12.5	18.5	4.4	6.8	9.4	34.8	5.0	22.7
AKT1	46.5	18.7	23.0	5.1	9.9	12.5	17.6	3.7	15.4
RABL6	40.6	15.4	21.0	5.0	8.9	12.2	22.1	5.1	22.2
PPP2CA	42.6	14.8	19.9	1.8	5.5	7.8	32.8	3.7	23.7
IER5	35.3	11.0	15.2	4.0	6.3	7.0	43.6	5.2	24.9
UBQLN4	39.8	14.7	18.9	4.0	7.4	12.2	25.0	4.7	25.7
DNAJC5	42.0	13.5	19.1	4.7	8.4	9.8	28.2	4.6	21.9
SPRED2	45.2	13.5	15.8	3.9	6.8	8.7	31.1	4.9	22.3
IPO5	34.4	13.1	16.6	1.6	5.5	8.1	37.8	4.8	30.2
MESDC2	37.2	14.1	18.8	3.4	6.7	8.7	33.1	6.0	23.9
MLLT6	41.6	15.5	20.6	5.2	8.7	12.1	23.9	4.4	19.8
IRAK1	34.3	12.8	16.0	6.3	9.5	14.0	30.8	5.2	22.9
DENR	41.4	14.2	20.5	2.0	5.9	7.1	33.3	3.9	23.4
STX10	47.2	17.2	22.2	5.0	7.6	11.6	20.6	3.8	16.4
ELOF1	41.6	16.8	19.0	2.9	7.0	8.9	28.3	6.5	20.5
HIST1H2AM	30.7	12.6	17.0	6.6	12.1	16.2	27.0	6.1	23.0
ASPH	41.4	13.5	17.1	2.0	7.0	9.6	30.9	4.6	25.2
ESRRA	42.1	15.2	20.4	3.1	4.7	6.7	34.6	4.4	20.0
RAVER1	40.7	15.3	20.1	5.3	8.2	10.6	25.9	5.4	19.5
POLDIP2	38.0	15.4	19.8	3.7	7.5	11.0	28.3	4.5	22.8
KAT2A	42.3	18.0	22.6	3.8	7.6	10.4	23.8	4.3	18.1
SCAMP3	42.0	19.1	20.9	4.5	6.6	10.7	22.4	4.5	20.1
GSK3A	39.7	15.4	20.7	3.9	7.7	9.4	25.4	5.6	23.0
LMNB2	38.4	14.1	18.7	5.3	8.5	10.8	30.3	5.0	19.6
POFUT1	44.4	16.9	21.1	3.2	7.4	9.9	25.0	3.4	19.2
THAP11	38.4	13.5	18.7	5.0	7.1	11.3	28.8	5.9	21.5
KIAA0100	40.6	14.2	19.4	2.6	6.8	9.6	29.8	4.5	22.6
TBCB	37.9	15.9	21.8	5.1	7.6	11.5	23.8	5.3	21.0
PPP1CC	40.1	17.5	20.0	0.8	5.7	7.7	32.4	3.9	21.7
SPR	46.8	14.4	19.7	3.2	5.1	7.4	32.9	3.4	16.9
TIMM44	39.9	15.1	20.6	2.4	6.5	9.5	29.7	4.1	21.8
ASAP2	41.3	13.8	19.1	3.9	8.5	11.1	26.1	4.2	21.7
ATP5F1	39.1	16.3	24.0	2.2	6.8	8.8	24.2	4.3	24.0
TRAP1	35.1	13.7	16.1	4.7	8.6	11.9	31.0	5.0	23.5
SMURF2	31.7	11.5	15.9	2.2	7.8	10.0	33.7	5.6	31.3
ILK	39.5	17.0	20.9	2.5	8.2	10.2	25.2	4.1	21.4
LAMTOR1	39.4	18.4	21.3	5.9	10.4	13.5	18.0	4.2	17.9
RSL24D1	35.4	13.4	21.2	0.8	5.6	6.7	31.4	4.7	29.7
PSMB2	35.8	16.0	19.3	2.9	6.2	10.9	30.6	4.7	22.3
ALG3	37.3	15.0	19.4	4.3	9.6	13.4	23.2	4.6	21.9
ARPC4	41.3	15.5	18.9	3.0	7.1	12.3	25.6	3.5	21.3

SNORA23	41.9	19.6	18.1	1.6	5.2	5.8	34.6	2.8	18.8
MAP1S	39.7	15.5	18.4	5.8	8.2	10.7	25.0	5.4	19.9
AKAP2	31.4	11.4	14.4	3.3	7.6	10.7	35.0	5.7	29.0
THBS1	39.7	13.4	17.6	5.0	10.7	13.7	24.5	4.0	19.8
LOXL4	55.9	22.9	30.1	3.5	8.4	9.4	10.3	1.5	6.6
ATP5J2	32.3	17.0	24.3	2.7	8.6	12.5	20.2	5.2	25.6
NDUFA12	41.6	17.1	22.6	1.7	7.4	10.4	23.0	4.1	20.5
ANAPC11	46.8	15.0	21.3	3.4	7.5	11.4	27.5	3.0	12.4
IL11	14.9	7.5	9.8	3.5	6.8	8.9	52.7	8.4	35.7
EXOSC4	31.4	10.6	17.3	3.2	6.9	8.9	37.8	6.2	25.8
SF3A1	41.3	14.5	18.0	3.7	6.9	9.8	26.6	4.9	22.3
FAM120A	40.6	14.1	18.1	4.3	8.3	10.2	25.5	4.7	22.1
TRNL1	40.5	13.0	27.6	1.9	4.6	9.0	23.1	3.9	24.3
PHC2	39.8	16.0	20.8	3.7	6.5	10.1	25.3	3.7	21.7
AMFR	44.2	15.3	20.1	2.8	6.1	8.8	27.0	3.7	19.5
PNMA1	36.5	14.0	18.1	2.3	6.3	9.8	30.9	4.4	25.3
CLUH	40.3	14.8	18.4	5.3	8.4	11.1	24.7	4.7	19.6
PALLD	45.2	14.2	19.5	4.2	6.0	7.8	24.8	5.6	20.2
PRPF6	39.3	13.3	18.1	3.6	7.2	10.3	30.9	3.9	20.7
BNIP3	38.8	18.2	19.7	1.4	6.2	8.2	27.8	3.7	22.9
BTG1	41.6	16.6	22.0	3.5	8.5	10.4	18.8	3.9	21.6
E2F4	33.7	15.0	16.9	3.2	7.7	10.6	28.7	5.8	25.3
FDPS	42.9	20.0	25.5	1.6	5.8	6.8	23.7	2.8	17.8
PYGL	26.3	9.7	13.2	2.4	5.4	7.8	42.3	6.1	33.6
IL8	60.0	23.5	34.4	1.0	4.2	6.6	10.1	1.3	5.5
HAX1	31.0	14.8	21.9	2.2	6.5	9.7	30.4	4.2	25.9
TPRA1	34.7	14.0	18.5	4.6	9.4	11.1	28.0	4.8	21.6
ERF	37.6	15.4	19.1	4.5	7.3	10.1	24.2	6.3	22.1
LOX	46.1	18.5	24.0	3.6	6.2	7.4	20.1	4.5	16.1
IFITM1	54.8	19.6	24.6	2.1	4.9	7.0	19.8	3.0	10.5
SUB1	39.6	15.9	22.8	1.1	4.8	8.7	27.4	3.6	22.2
CSNK1G2	40.9	17.7	21.4	5.5	7.6	11.5	18.9	4.8	17.8
HSPBP1	40.0	14.5	19.4	3.8	7.7	10.1	27.4	4.2	18.9
SNORA3	31.3	14.2	15.1	5.9	11.0	7.1	38.8	3.9	18.6
FOXA2	36.9	15.9	19.1	5.3	7.5	9.5	24.4	5.9	21.3
CCT2	35.7	13.0	17.3	1.4	5.6	7.5	36.1	3.9	25.1
SGTA	37.6	15.5	18.6	4.0	7.0	9.8	23.5	5.7	23.6
ARHGEF12	42.5	13.2	18.4	1.8	6.1	8.0	27.9	3.7	23.5
RGS10	31.9	13.4	19.9	5.5	9.7	13.0	23.2	5.4	23.3
SYVN1	36.0	14.3	17.5	4.2	6.5	9.0	28.0	6.2	23.4
RBCK1	36.5	15.1	19.6	5.5	8.9	13.1	22.9	4.7	18.6
SYMPK	39.5	13.3	19.1	4.5	8.1	10.6	25.4	4.5	19.7
DVL3	39.1	13.5	18.9	4.8	8.6	11.9	22.5	5.0	20.5
NOTCH2NL	45.7	12.8	20.3	2.5	7.6	7.7	30.5	2.7	14.8
UQCRB	31.2	13.8	17.3	1.1	6.4	8.6	28.7	6.6	30.4
MBP	46.7	18.2	20.8	2.1	10.0	5.3	22.0	1.7	17.4
ARFGAP1	40.5	16.1	20.8	3.4	6.9	9.1	24.6	3.3	19.4
ADORA2B	29.9	12.3	15.4	6.8	10.6	15.2	27.4	4.8	21.7
ERCC2	33.5	12.9	16.1	6.5	12.5	18.5	22.9	3.8	17.3
AGO2	38.5	12.4	18.3	4.0	7.7	9.5	24.6	5.0	23.9
DDX27	38.6	15.5	19.2	2.7	7.0	10.0	25.2	4.6	21.2
PSMA5	34.6	14.9	21.4	1.4	5.9	8.7	30.9	3.6	22.6
FXYD5	32.3	13.5	14.8	4.8	8.1	11.4	23.5	6.8	28.6
ADM	36.2	12.4	20.4	4.8	11.0	14.4	24.9	3.5	16.0
NSA2	33.0	16.1	21.0	1.5	6.8	9.2	24.0	4.5	27.4

PSMB7	33.1	14.6	18.5	2.7	7.1	9.8	28.4	4.6	24.6
PDDC1	42.0	11.9	19.3	5.3	8.0	10.7	27.8	3.4	14.8
MLLT1	36.2	14.6	18.8	4.9	7.4	10.3	22.0	5.6	23.4
CMTM7	43.0	16.0	18.4	6.0	7.8	10.3	20.2	3.5	17.7
LTA4H	40.2	14.6	18.0	1.6	5.1	8.4	28.9	3.7	22.4
SNORA12	33.2	16.4	24.8	1.2	6.2	7.1	28.1	4.2	21.8
TAF7	41.6	15.6	21.3	1.8	5.5	8.2	23.0	4.0	22.0
FAM207A	38.3	13.8	18.4	4.8	7.8	10.3	26.0	5.3	18.1
RNA5S17	9.3	43.4	27.9	27.6	4.9	1.7	9.7	13.2	5.0
RNA5S16	9.3	43.4	27.9	27.6	4.9	1.7	9.7	13.2	5.0
RNA5S15	9.3	43.4	27.9	27.6	4.9	1.7	9.7	13.2	5.0
RNA5S14	9.3	43.4	27.9	27.6	4.9	1.7	9.7	13.2	5.0
RNA5S13	9.3	43.4	27.9	27.6	4.9	1.7	9.7	13.2	5.0
RNA5S12	9.3	43.4	27.9	27.6	4.9	1.7	9.7	13.2	5.0
RNA5S11	9.3	43.4	27.9	27.6	4.9	1.7	9.7	13.2	5.0
RNA5S10	9.3	43.4	27.9	27.6	4.9	1.7	9.7	13.2	5.0
RNA5S8	9.3	43.4	27.9	27.6	4.9	1.7	9.7	13.2	5.0
RNA5S7	9.3	43.4	27.9	27.6	4.9	1.7	9.7	13.2	5.0
RNA5S6	9.3	43.4	27.9	27.6	4.9	1.7	9.7	13.2	5.0
RNA5S5	9.3	43.4	27.9	27.6	4.9	1.7	9.7	13.2	5.0
RNA5S4	9.3	43.4	27.9	27.6	4.9	1.7	9.7	13.2	5.0
RNA5S3	9.3	43.4	27.9	27.6	4.9	1.7	9.7	13.2	5.0
RNA5S2	9.3	43.4	27.9	27.6	4.9	1.7	9.7	13.2	5.0
RNA5S1	9.3	43.4	27.9	27.6	4.9	1.7	9.7	13.2	5.0
GSS	43.2	16.8	21.7	3.0	8.1	10.8	19.1	2.8	16.8
SCARNA9	43.8	18.5	25.9	2.0	7.0	8.3	14.6	3.0	19.3
DCTD	39.7	14.1	19.6	1.9	7.0	9.6	28.0	3.7	18.9
CIZ1	33.7	13.9	16.0	5.9	9.4	12.9	22.4	5.5	22.6
KLF16	36.0	12.7	16.7	6.8	9.9	10.3	23.5	6.6	19.6
XPOT	41.8	15.9	19.2	0.9	5.0	7.6	25.4	3.5	22.9
MRPL43	34.8	16.3	22.6	5.9	8.2	11.6	18.4	5.6	18.7
RUVBL1	34.1	13.2	17.9	2.8	5.8	7.0	32.1	3.9	25.4
TRMT1	31.1	12.7	17.4	4.7	7.7	11.3	28.3	5.1	23.4
PET100	37.4	16.3	23.5	1.0	5.5	8.7	28.2	3.1	18.1
ARHGEF2	38.0	13.6	17.8	5.2	9.9	13.5	23.0	4.4	16.1
EBNA1BP2	25.8	10.3	14.0	2.2	7.1	8.5	37.0	5.3	31.0
ATP1B3	38.1	14.3	19.7	1.2	4.7	6.9	29.1	4.2	23.1
SRF	35.2	13.7	17.1	5.1	7.0	10.1	24.4	6.0	22.7
GM2A	39.3	16.7	20.3	1.5	4.0	5.8	31.2	4.0	18.5
NCLN	38.2	14.5	18.4	5.0	7.5	11.1	24.7	4.1	17.4
NARS	32.2	11.6	16.0	1.7	5.9	7.9	36.6	3.3	25.8
CPSF7	34.0	14.7	19.4	2.7	6.5	8.5	23.9	5.3	25.7
SEZ6L2	42.7	16.0	19.8	4.8	8.3	11.2	18.4	3.6	15.8
FGF5	27.1	9.7	14.6	2.9	6.6	8.5	33.7	6.5	30.9
ATP5G1	32.7	14.4	19.2	3.1	8.7	12.1	25.4	3.7	21.3
ELAVL1	34.9	14.0	17.8	3.3	7.9	10.2	22.9	5.5	24.1
FURIN	44.5	17.1	22.0	3.7	5.9	7.5	19.7	3.8	16.4
PLIN3	38.0	15.7	18.6	5.1	8.1	11.0	22.7	4.2	17.0
SEL1L	41.9	14.8	20.3	2.0	7.1	6.9	24.5	3.7	19.2
SMARCB1	35.5	14.2	18.1	3.2	7.3	9.7	26.7	4.4	21.3
SNORD68	30.3	14.6	10.3	10.7	7.5	19.2	16.4	5.6	25.7
NBR1	39.4	14.1	17.8	2.1	6.1	9.1	26.4	4.0	21.4
MFGE8	45.6	16.4	23.9	3.6	8.6	10.2	17.1	2.4	12.3
GDI2	34.8	13.3	18.4	1.0	5.0	8.1	31.3	4.1	24.1
MTHFD1	26.8	10.4	13.2	2.2	7.0	9.0	36.2	5.2	30.2

PIH1D1	32.1	13.6	18.9	3.7	8.3	10.3	26.6	4.4	22.1
ANXA3	33.2	13.3	18.6	1.1	5.3	7.5	32.4	3.6	24.9
COX7A2L	41.8	16.2	22.3	2.0	6.3	7.9	23.0	3.0	17.4
STARD3	38.8	17.0	21.6	3.2	5.4	8.2	23.0	4.0	18.6
SREBF2	42.8	15.6	20.3	3.0	6.1	8.0	23.3	3.3	17.2
FOXK1	36.6	13.1	16.5	6.7	9.5	11.1	19.0	6.0	21.0
GPR108	41.9	18.9	25.2	4.0	7.8	10.9	15.1	3.0	12.8
SUPT4H1	37.5	13.9	19.8	1.7	6.6	8.4	25.9	4.7	21.0
NRP1	45.9	15.5	20.5	2.9	8.5	10.9	18.6	2.5	14.2
MAPKAPK2	35.2	14.9	16.9	3.4	6.2	10.3	24.1	4.5	23.8
SSNA1	36.6	16.1	21.1	3.6	5.6	9.0	26.4	4.2	16.4
SLIRP	37.2	14.1	18.8	1.7	7.3	8.8	27.3	4.1	19.8
TRIM25	45.3	17.0	21.8	4.0	6.1	7.7	16.7	4.4	16.1
POLR2H	32.2	14.0	19.0	1.4	4.8	7.0	30.7	3.9	25.9
ARPC1B	37.7	13.5	19.6	4.6	6.7	8.5	26.2	4.2	17.9
AMPD2	40.7	15.7	21.5	3.2	5.5	8.7	22.1	4.4	16.9
C19orf48	23.7	11.5	12.9	3.5	5.8	11.3	33.9	6.6	29.0
ETF1	34.3	12.9	16.3	1.9	5.2	7.1	29.2	4.2	27.1
SELM	39.9	15.7	24.1	3.7	6.5	9.9	20.1	3.9	14.5
NPAS2	39.1	14.2	19.3	5.4	9.2	12.4	17.5	4.2	16.9
SMIM7	38.7	11.6	19.5	2.6	6.7	7.9	26.0	3.4	21.6
SDC1	33.0	15.0	16.3	5.3	8.3	10.5	24.3	5.3	20.1
NOG	41.6	17.5	21.6	4.8	7.0	10.3	18.0	3.3	13.8
MLF2	39.3	15.3	18.9	3.4	7.3	9.9	22.8	3.6	17.2
KIF1C	37.4	14.7	18.3	4.1	7.9	10.9	21.9	3.9	18.7
H2AFV	31.8	12.4	19.5	2.8	7.9	12.7	25.8	3.2	21.7
SLC25A1	43.3	17.2	22.2	4.7	7.0	9.0	17.6	3.5	13.4
LARS	39.0	13.1	17.5	1.2	5.7	8.0	29.6	3.1	20.6
LOC100128531	43.4	9.4	18.0	2.6	4.8	6.8	34.4	2.7	15.4
KLHL5	39.8	13.9	17.9	2.3	7.0	11.4	25.0	3.2	17.0
SNHG16	34.8	12.4	16.7	1.9	4.9	7.4	31.1	4.5	23.6
GSPT1	36.3	11.7	15.0	2.1	5.5	5.8	32.0	4.1	24.7
COPS3	32.4	13.5	17.2	0.8	4.9	6.7	31.5	4.0	26.2
CCT8	32.2	13.2	17.1	1.1	4.9	6.5	33.5	4.1	24.6
DCAF7	39.9	14.6	18.6	3.8	6.9	8.4	22.0	4.4	18.5
LOC284889	33.0	16.7	17.8	6.8	8.9	12.2	16.9	5.7	19.0
SAE1	30.6	11.1	14.0	1.9	4.9	7.4	35.7	5.1	26.0
PTK7	37.5	14.6	19.9	3.9	7.1	10.6	22.1	3.2	17.5
NTN4	41.5	15.9	20.8	3.4	8.7	11.0	18.6	2.5	14.1
MAP2K3	39.9	13.7	17.6	2.2	5.4	5.6	27.5	4.4	20.1
SMARCE1	33.0	12.6	19.1	3.1	6.4	10.0	22.9	4.9	24.4
DAZAP1	31.2	12.7	14.5	4.5	8.8	11.8	25.6	4.8	22.3
SHC1	36.3	14.2	17.9	3.3	7.3	9.6	25.0	4.4	18.4
SMG5	36.1	13.9	17.1	2.3	6.0	8.0	26.9	4.2	21.9
IGF2R	42.9	13.1	18.5	2.5	6.5	7.8	24.8	2.9	17.4
MFSD12	36.4	14.4	16.9	6.5	9.6	12.6	21.3	3.6	14.9
LCP1	46.0	16.5	20.9	1.4	5.0	7.2	20.8	2.4	16.0
IK	38.2	14.7	18.3	2.6	6.1	9.3	22.2	4.2	20.4
PMAIP1	38.0	17.4	21.6	1.4	5.4	6.7	20.6	4.5	20.4
UBL5	35.3	16.0	23.4	1.3	5.3	6.1	27.0	2.2	19.2
COASY	37.5	15.1	17.8	2.8	6.1	8.5	26.4	4.2	17.4
DHX30	38.1	12.4	18.1	3.1	5.7	7.9	28.4	4.0	18.2
VAPA	39.3	14.9	16.8	1.6	5.1	7.8	25.4	3.4	21.2
LOC101929046	33.8	12.2	15.0	4.1	6.9	8.1	28.2	5.7	21.8
SNX19	37.7	14.0	18.7	3.1	7.3	9.3	23.2	3.6	18.8

HSPA4	33.0	12.8	15.5	1.2	4.6	6.4	31.0	4.2	27.1
BTBD2	46.5	18.3	21.0	4.3	8.5	11.4	12.6	2.7	10.4
POLR2F	37.7	12.3	20.6	3.5	6.9	9.8	21.3	4.9	18.4
PRR13	30.5	14.9	17.1	3.4	8.6	11.0	21.8	4.7	23.2
LOC100505817	38.5	14.8	20.6	1.2	4.5	6.1	25.0	3.6	21.0
SDHB	37.8	13.3	19.5	2.4	7.1	7.8	26.9	3.2	17.3
TXNRD1	33.1	11.3	15.8	1.4	5.5	6.9	33.6	3.8	24.0
TM9SF3	37.0	12.5	15.8	2.1	4.8	6.6	31.2	3.7	21.7
NFIC	35.2	12.5	17.8	4.9	8.0	10.5	22.5	5.0	18.9
TOR4A	35.7	12.3	15.9	4.4	6.0	8.3	29.7	4.5	18.4
PICALM	33.5	14.0	17.3	2.4	7.1	8.5	22.8	4.9	24.7
VEGFC	36.9	13.4	17.1	1.4	4.2	6.5	27.5	4.5	23.6
FMNL1	42.6	16.1	20.5	5.1	9.2	11.9	14.5	2.8	12.2
SDCCAG3	32.7	15.3	17.9	3.2	5.4	9.5	26.5	4.5	20.0
ATP6V0E1	36.0	14.1	27.1	1.8	5.0	8.8	21.0	3.0	18.1
LAYN	31.8	13.1	16.4	2.5	6.6	9.5	28.4	4.6	22.1
DNASE2	40.0	16.1	21.2	3.7	7.1	9.9	16.1	3.5	17.3
ZNF580	35.4	17.3	19.1	9.6	10.3	15.7	13.5	3.5	10.5
KIRREL	35.0	12.8	16.5	4.0	7.1	9.7	27.0	4.3	18.5
MFSD10	38.7	15.1	20.6	3.9	6.8	10.7	21.2	3.4	14.2
UPF1	38.8	14.6	17.4	3.7	6.6	9.4	22.1	3.9	18.1
C20orf27	32.1	12.5	15.7	4.0	8.2	11.2	28.3	4.5	17.9
STAT3	42.7	15.1	19.6	2.6	5.4	7.3	21.5	3.5	16.6
NDUFA1	36.5	15.9	22.5	1.6	4.0	6.4	26.7	2.6	18.0
CYHR1	42.0	15.5	21.3	3.9	7.2	7.8	18.5	3.2	14.8
MIR100HG	36.6	12.6	19.0	0.9	4.9	7.2	27.7	3.0	22.0
UBE2E1	34.6	14.0	19.7	1.7	6.4	9.9	21.1	4.3	22.3
PLEKHB2	35.5	11.4	17.4	1.3	4.4	5.0	34.4	3.1	21.3
SNHG1	25.6	11.0	15.4	0.6	2.5	3.5	35.5	5.6	34.1
MYBBP1A	29.7	11.2	14.3	4.3	6.9	9.7	29.3	5.0	23.1
PSMB1	35.2	13.9	18.2	1.3	6.5	8.7	26.8	3.4	19.6
TGFB2	40.1	15.0	18.3	1.8	5.3	7.6	22.0	4.1	19.2
NCK2	38.4	12.3	16.2	3.6	5.6	7.0	24.8	5.0	20.6
FKBP10	38.0	16.2	21.8	5.1	10.2	13.2	14.8	2.6	11.5
SPANXB1	29.7	12.4	17.9	3.7	9.6	13.2	19.2	4.6	22.8
SPANXB2	29.7	12.4	17.9	3.7	9.6	13.2	19.2	4.6	22.8
ANAPC5	36.9	13.8	20.0	2.3	5.6	7.6	24.6	3.5	19.0
CLNS1A	34.1	11.0	16.1	1.5	5.5	7.1	32.2	3.8	22.1
MACROD1	37.6	16.3	21.7	5.0	9.4	12.7	16.0	2.6	11.9
SOX9	32.8	12.0	14.3	9.0	11.8	14.1	15.9	4.8	18.3
CLTB	33.4	12.9	16.4	4.4	8.2	11.0	23.3	4.8	18.7
ZC3H7B	36.9	14.5	18.1	5.4	9.1	12.3	18.8	3.8	14.2
PSMD7	34.8	14.7	18.2	2.3	7.6	9.8	22.2	4.1	19.2
SLC35F2	31.7	12.5	15.1	1.5	6.2	7.5	29.6	4.0	24.9
TEX264	35.7	14.3	19.2	2.4	5.3	7.9	25.8	3.9	18.4
ABL2	31.9	10.0	15.0	1.9	6.9	6.5	29.7	5.8	24.9
PLXNA1	41.5	14.3	18.9	4.9	8.1	10.8	17.2	2.9	13.6
GSG1L	12.4	21.6	15.1	39.1	6.8	0.0	14.2	16.0	7.2
SMARCA4	33.5	11.8	14.7	4.6	7.3	9.7	24.7	5.1	20.9
HIST1H2BJ	24.7	11.2	15.0	6.5	11.9	15.8	22.9	5.0	19.1
RPP21	33.1	13.6	16.5	4.6	7.9	9.1	23.1	4.5	19.9
SRSF7	31.6	12.4	18.6	1.4	4.9	6.7	31.6	4.1	21.0
IQGAP1	36.4	12.4	15.8	2.0	6.5	8.3	25.8	3.7	21.4
GLRX	33.5	12.5	17.8	2.2	4.7	6.3	28.0	4.1	23.0
IL6ST	38.0	13.6	18.2	1.3	5.3	7.5	24.4	3.1	20.5

NTMT1	31.9	14.8	18.6	2.7	4.9	7.0	24.8	4.6	22.7
PPP5C	33.0	13.0	15.5	3.2	7.2	9.6	25.3	4.1	21.1
C14orf119	33.6	14.0	20.5	1.9	4.2	7.9	24.7	3.4	21.7
PDLIM7	27.2	11.6	14.7	7.5	10.3	16.1	21.4	4.6	18.5
F2RL1	32.8	13.8	16.6	1.7	5.7	8.2	25.1	4.0	23.9
SUN1	36.6	12.6	17.2	2.7	5.5	7.5	25.5	4.0	20.1
ABR	39.4	14.0	18.2	3.5	6.7	8.5	20.5	3.5	17.4
MROH1	41.3	14.5	22.0	4.7	7.6	9.8	16.7	2.8	12.2
SAMD4B	40.4	14.7	17.7	4.8	7.9	11.6	17.6	3.2	13.6
ACADVL	33.7	14.4	17.6	4.4	9.7	13.8	18.8	3.4	15.5
TRAPPC4	35.6	15.6	19.4	2.3	6.7	8.9	23.6	3.0	16.2
ABHD17A	38.8	15.7	19.0	5.7	8.8	11.1	16.0	3.8	12.5
ELL2	34.2	12.9	17.6	1.9	4.3	6.3	25.0	4.9	24.2
CHMP1B	36.6	13.0	19.0	2.5	5.5	9.3	23.9	3.2	18.3
ITM2B	38.5	16.9	20.8	3.2	7.2	9.0	16.4	3.3	15.9
OLA1	34.3	13.3	18.3	1.0	5.0	6.5	25.7	3.4	23.6
SDF2	38.3	15.3	22.8	2.2	5.1	6.8	23.7	2.6	14.1
ITPRIP	31.6	11.4	16.2	3.5	5.2	7.5	28.4	5.0	22.0
ASNS	33.6	12.9	16.5	1.3	5.5	7.7	27.0	3.0	23.4
PPP1R12C	34.3	12.5	17.0	5.8	9.1	11.3	21.8	3.9	15.0
MAVS	33.4	11.1	15.9	4.1	7.3	10.1	25.4	4.4	19.1
TBC1D17	38.9	15.6	19.1	4.8	7.7	10.3	16.4	3.7	14.3
XRCC5	33.4	12.1	15.1	1.2	5.0	7.0	30.7	3.2	23.1
CPSF6	29.2	12.1	18.8	2.9	6.4	9.0	25.3	4.5	22.3
GPS2	33.2	14.8	19.0	3.6	7.2	9.2	18.0	4.6	20.7
MTA2	31.9	12.2	15.8	1.9	5.3	7.7	25.3	4.7	25.4
NDUFS4	33.6	15.0	22.1	3.0	7.1	8.7	20.7	2.8	17.2
NUCB1	37.4	15.5	17.8	5.3	8.3	12.1	16.8	3.2	13.9
SSSCA1	28.8	13.2	15.7	4.8	6.5	11.3	25.4	6.0	18.5
TRNM	32.1	17.7	10.2	12.6	13.8	11.4	18.3	3.5	10.6
ARMCX6	36.1	16.1	20.4	1.6	4.9	7.5	22.5	3.8	17.1
LAMTOR5	37.3	13.9	23.1	2.1	6.4	8.9	17.6	3.4	17.1
MYO18A	45.4	18.2	23.4	3.9	7.4	9.6	12.0	2.1	8.0
USP10	30.7	11.5	13.5	2.0	4.5	8.1	27.4	5.2	26.8
RNF114	36.0	13.3	16.4	2.3	5.7	6.5	26.7	3.6	19.0
C8orf82	32.5	15.4	18.9	6.2	9.4	12.1	17.8	3.7	13.5
FBXW5	37.7	13.6	18.0	5.2	7.7	10.6	19.8	3.5	13.6
NDUFB9	30.1	11.7	17.7	1.8	6.9	8.8	26.5	3.5	22.5
EIF2S1	29.5	12.6	15.2	0.9	4.3	6.0	31.1	3.3	26.4
ACIN1	33.7	13.5	18.1	3.0	7.1	8.8	20.8	4.0	20.3
MRPL51	36.7	12.1	19.9	1.8	4.7	6.9	27.1	2.9	17.2
AGR2	40.8	15.9	23.1	0.7	1.8	2.5	21.5	4.5	18.3
ANTXR2	37.0	13.2	17.1	1.3	4.8	6.5	27.0	2.9	19.4
FAM65A	35.4	13.6	17.4	3.4	6.6	9.0	21.2	4.1	18.3
KLF6	34.8	13.8	17.1	2.6	5.4	9.4	23.3	3.8	18.6
ATP5G3	34.1	11.9	21.2	0.8	4.5	6.7	27.6	3.2	19.0
GID8	36.3	14.5	17.8	2.2	5.3	7.7	24.6	2.9	17.6
LOC101928783	38.8	10.1	19.0	4.6	6.6	8.5	24.0	3.5	13.6
EFHD2	36.0	14.6	19.3	5.6	9.2	11.5	14.1	4.0	14.4
ZNF787	32.4	13.4	17.1	5.5	5.8	8.6	20.3	5.9	19.5
MTA1	31.3	11.1	15.0	5.4	7.8	11.1	22.6	4.3	20.0
PLBD2	45.3	15.6	20.7	4.1	8.0	10.6	12.8	2.1	9.5
LINC00987	28.4	13.8	15.9	3.8	7.3	10.1	17.9	6.5	24.8
GOT2	32.2	12.7	16.0	2.0	6.0	8.8	25.4	3.6	21.8
ACTR2	36.1	13.9	16.2	1.2	5.1	6.9	24.9	3.4	20.7

MXRA7	39.3	14.9	18.9	5.8	9.9	9.7	15.3	2.7	11.8
AGPAT2	37.3	13.7	17.5	3.6	6.9	9.3	19.7	3.6	16.8
EIF2B2	35.0	14.4	18.7	3.5	6.6	10.6	20.7	3.0	15.8
GABARAPL2	42.4	12.4	21.7	1.4	4.3	6.3	22.2	2.4	15.0
SLC37A2	39.3	17.6	22.4	5.2	11.0	14.2	9.2	1.8	7.4
ATPIF1	27.7	14.7	18.9	4.5	7.6	12.3	18.5	4.9	19.0
ARF3	37.9	13.0	15.5	2.3	5.9	7.9	26.2	2.4	16.9
SIN3B	37.8	14.5	19.7	3.9	6.3	8.2	19.3	3.5	14.7
UQCRFS1	33.7	14.6	17.2	2.1	6.3	9.7	22.6	3.7	18.2
TIE1	34.6	13.2	17.8	3.8	7.0	9.4	22.4	3.5	16.2
SAFB	31.8	11.4	14.7	3.4	5.6	6.9	28.5	4.8	20.7
PSME2	31.7	13.6	15.7	2.0	7.8	8.2	23.7	3.5	21.3
SLC35F6	39.5	15.9	19.6	3.5	6.5	9.6	17.0	3.1	12.7
DDX41	36.3	13.7	17.6	3.9	6.6	8.8	20.3	3.3	16.9
PTGES3	36.0	11.9	17.1	3.0	5.6	6.8	26.1	3.7	17.3
TTLL12	32.2	13.0	14.7	5.5	7.7	11.2	20.8	3.9	18.6
C11orf31	27.1	11.5	17.9	5.6	9.6	10.2	26.7	3.3	15.5
PLAUR	31.1	11.4	14.9	4.7	9.3	12.0	23.4	3.4	17.2
CHD4	35.7	12.4	15.4	2.0	5.3	6.9	25.5	3.9	20.4
IRF2BP2	34.0	13.3	18.1	3.4	6.5	8.5	20.7	4.6	18.2
DUSP3	30.2	11.1	14.0	5.1	10.4	11.0	24.1	4.5	17.0
SNORD76	25.7	13.4	11.2	6.6	8.4	9.0	22.3	6.6	24.0
SAR1A	32.9	13.1	17.3	0.9	4.1	5.6	26.4	3.9	23.0
FAM24B	34.9	12.4	20.8	1.1	5.1	5.0	24.1	3.0	20.8
LRRC8A	38.1	15.6	16.7	4.5	6.2	8.3	19.4	4.0	14.3
VIMP	34.5	11.8	18.1	1.4	4.7	6.3	28.4	3.4	18.6
PTK2	35.6	12.8	17.5	1.6	5.4	7.2	26.0	3.1	18.0
STARD7	31.9	11.3	15.7	1.8	4.7	6.3	30.1	3.9	21.4
CHCHD10	27.3	12.5	16.4	8.1	9.6	12.7	20.6	4.3	15.4
SRPRB	26.4	10.3	13.9	1.6	3.8	6.1	36.9	4.4	23.5
RNF26	35.6	13.2	17.1	3.8	7.1	9.6	22.2	3.1	15.2
SNRPF	26.6	12.2	13.4	1.2	5.5	5.6	32.7	3.9	25.7
CCNJL	33.6	12.6	15.7	4.3	8.3	11.3	18.0	5.4	17.7
IMP4	30.3	13.1	15.2	4.3	7.2	10.7	23.0	4.5	18.4
TSEN34	33.0	12.5	17.1	3.3	5.9	7.7	23.7	4.5	19.1
TPCN1	37.3	14.1	18.4	4.9	9.3	12.8	14.9	2.5	12.5
LINC00458	38.5	10.9	17.4	2.1	4.7	5.4	25.5	3.9	18.3
WWTR1	34.8	13.0	16.5	4.1	7.3	10.7	19.0	4.8	16.6
CNOT3	37.8	15.0	18.4	3.8	7.0	8.0	16.4	4.4	15.8
TNRC18	37.3	12.8	17.7	4.3	6.4	7.7	21.3	3.8	15.2
NEDD4	36.2	12.5	16.5	1.0	3.9	5.8	25.4	3.5	21.8
SHB	36.0	12.7	16.1	4.3	6.8	9.3	20.7	4.4	16.0
HK2	29.2	10.0	12.6	2.4	5.5	6.5	34.1	4.0	22.0
GRWD1	30.9	10.1	14.5	3.7	6.5	8.4	26.4	5.3	20.4
NME4	22.8	10.7	13.5	5.0	8.7	10.5	27.8	5.7	21.6
TMEM248	35.4	13.3	15.2	3.8	6.8	9.5	18.3	4.5	19.2
NOSIP	34.2	12.1	16.5	5.0	7.8	10.6	22.0	3.2	14.2
PITX1	33.2	14.4	16.3	4.6	8.1	10.1	15.3	5.7	17.8
GNG12	37.4	12.0	17.2	1.9	5.0	7.1	23.2	3.7	18.0
PIN1	36.3	12.9	17.8	5.4	9.8	12.5	15.3	3.5	11.9
CASC3	32.4	12.2	15.8	2.4	6.0	8.0	23.1	4.1	21.3
CRK	31.7	11.9	16.6	2.5	5.4	6.8	27.7	3.8	18.8
SNRPG	32.3	13.9	17.7	0.5	5.1	6.3	24.6	2.2	22.5
UBA1	33.3	12.0	13.6	3.6	7.2	9.7	24.1	3.8	17.9
HIST1H2AH	20.2	9.3	13.6	8.4	12.0	14.7	23.6	4.3	18.9

NOP56	25.6	11.7	15.0	2.7	7.1	9.5	27.2	4.3	21.9
TUBG1	31.3	12.2	16.5	4.6	7.6	10.6	19.3	4.6	18.3
RERE	36.1	11.9	15.9	4.8	6.7	9.1	17.6	4.7	18.1
RILPL1	31.0	11.3	15.8	4.0	5.6	8.0	24.7	5.2	19.3
TSC22D4	39.2	16.3	20.9	2.9	5.4	7.1	17.3	3.4	12.3
PUM2	31.6	11.6	14.9	1.5	5.1	6.4	26.7	3.8	23.3
PRCC	34.0	12.9	15.4	2.3	4.9	7.2	25.4	4.3	18.3
TOLLIP	34.3	13.0	15.6	4.7	6.0	8.8	23.1	4.6	14.6
PINK1	34.3	14.2	16.1	3.9	8.0	10.2	20.6	4.0	13.3
CYCS	25.7	11.6	15.6	1.2	5.4	5.1	31.3	4.6	23.8
BAX	34.3	13.9	17.3	3.5	7.6	10.2	18.8	3.5	15.3
SPCS1	34.0	14.6	17.2	3.7	6.4	9.7	18.9	3.0	16.8
FLJ20464	40.7	8.8	18.0	2.3	4.6	5.4	29.2	2.4	13.0
DGKZ	35.1	13.5	16.3	4.1	6.0	9.3	20.6	4.0	15.2
TNS3	42.8	14.5	19.1	3.3	6.7	8.4	15.0	2.7	11.7
TMEM205	38.2	13.9	17.7	5.0	8.0	10.9	15.9	2.1	12.3
ACP1	30.7	12.4	17.3	1.3	5.9	6.3	26.8	3.7	19.3
PERP	42.0	14.7	20.1	2.1	4.4	6.9	17.3	3.2	13.1
GLRX5	32.1	13.1	17.8	4.5	6.4	8.0	22.0	4.2	15.7
NELFCD	33.9	13.5	18.0	2.4	5.4	7.6	20.4	3.5	19.0
TMEM43	29.5	11.0	15.8	3.4	7.6	10.6	24.1	3.5	18.3
SEC62	32.3	13.6	18.5	1.5	4.6	6.7	20.9	3.8	21.6
YWHAH	29.9	11.5	14.7	2.4	5.9	7.7	26.0	4.5	20.8
UBE2R2	34.9	12.6	16.2	2.1	5.0	6.8	22.6	5.0	18.1
CFDP1	31.1	12.6	14.4	1.7	4.8	6.6	26.1	3.8	22.0
TRAPPC12	34.0	10.0	16.4	4.9	7.8	9.3	22.8	3.9	14.1
HMG20B	39.5	14.0	18.1	4.1	7.2	10.9	15.8	2.5	10.9
MINK1	36.4	15.3	20.2	3.3	5.9	7.8	16.6	3.1	14.4
IRF2BP1	27.3	11.2	14.4	3.6	6.3	8.1	27.8	5.0	18.9
BACH1	29.7	10.2	14.1	0.8	3.2	4.0	32.6	3.9	24.1
TMED7	31.4	11.1	16.2	2.1	4.7	6.8	25.1	4.9	20.2
HNRNPR	25.6	9.2	11.5	1.8	6.1	8.2	29.4	4.0	26.7
LGMN	42.2	15.3	18.9	1.9	4.0	5.2	19.2	2.5	13.3
CAST	34.7	12.5	16.0	1.4	5.0	6.9	22.3	3.5	20.1
LOC101928785	40.9	9.8	17.7	2.1	5.8	6.3	22.7	2.2	14.8
TRPC4AP	35.7	13.4	17.3	2.6	6.9	8.5	21.1	2.7	14.0
C12orf52	31.0	12.9	15.7	3.8	6.0	8.6	20.0	4.6	19.6
C4orf48	32.4	11.9	14.3	7.4	8.0	8.6	22.8	4.2	12.6
OXA1L	30.3	12.0	16.3	2.8	7.4	10.1	21.1	4.0	17.9
NPEPPS	37.6	13.0	17.2	2.3	5.2	6.5	21.7	2.9	15.4
FKBP1A-SDCBP2	30.6	12.0	15.3	6.2	8.3	11.6	17.3	5.3	15.3
SYNCRIP	29.2	10.8	13.1	1.2	4.4	6.6	28.6	3.7	24.2
PDGFC	36.7	13.2	17.3	1.4	4.1	6.4	22.5	3.0	17.0
WSB2	31.6	11.6	15.8	2.8	6.4	7.6	25.6	3.1	17.2
LRCH4	36.1	14.2	19.7	3.8	6.3	7.9	16.2	3.8	13.7
SCNM1	37.9	9.7	17.5	2.3	4.0	6.2	26.5	2.8	14.9
LOC101928247	34.1	12.5	18.8	5.2	6.3	8.0	18.4	4.8	13.6
CRTC1	34.9	13.8	16.3	5.4	6.7	9.2	14.6	4.2	16.4
SNW1	33.2	12.6	15.2	1.1	4.8	6.4	25.3	3.5	19.3
STRA13	35.0	13.5	20.3	5.1	7.1	7.1	17.7	3.7	12.1
MLPH	38.4	15.3	20.6	3.8	7.9	9.7	14.3	2.0	9.4
LOC101929402	41.8	8.1	16.5	2.0	4.4	4.6	29.3	2.5	12.3
MAP1B	30.3	8.9	12.5	1.8	4.6	5.5	28.9	4.6	24.4
C11orf84	31.6	12.4	16.1	3.3	4.8	7.6	20.5	4.4	20.5
ABCF1	29.3	10.8	13.6	2.6	6.2	7.3	25.6	4.3	21.5

VPS51	32.2	11.1	15.1	4.3	6.7	9.3	23.6	3.6	15.2
PGRMC2	32.4	11.9	16.0	5.5	6.5	8.3	17.5	4.2	18.8
STK10	35.8	13.4	17.3	4.1	7.7	8.9	16.1	3.2	14.4
TINAGL1	20.4	8.8	11.3	4.3	7.9	11.1	31.5	4.6	20.9
THRAP3	32.4	11.7	14.6	1.9	5.7	7.6	21.4	4.0	21.5
GLG1	37.4	12.9	17.1	2.3	5.5	8.2	20.6	2.6	14.2
SNORA16A	24.6	15.2	11.0	6.2	7.8	10.7	20.1	5.0	20.1
CCDC47	28.7	10.3	12.8	1.1	4.3	5.5	31.8	4.2	22.0
PCMT1	30.8	10.6	17.0	1.6	4.4	5.2	27.8	3.6	19.6
ADAM8	37.3	15.4	20.5	6.4	10.6	14.2	8.8	1.6	5.7
FSCN1	33.3	12.9	16.2	5.5	7.6	10.1	17.8	3.8	13.4
UGCG	31.9	13.4	16.0	1.5	5.4	7.3	21.4	3.4	20.2
SCARNA16	31.8	12.7	16.8	0.8	7.3	5.5	28.1	3.0	14.4
UROD	32.0	13.0	18.3	2.4	6.9	9.2	20.1	3.0	15.5
TACC1	29.1	9.1	11.4	1.7	4.9	5.7	32.1	4.5	22.0
BUD31	31.2	10.9	16.2	1.9	4.1	6.6	26.8	3.9	18.9
PNRC1	35.6	13.6	18.6	2.6	5.2	7.2	19.5	3.8	14.4
NCOA5	36.2	12.5	16.6	2.9	5.3	8.4	19.9	3.3	15.1
CLASRP	35.3	14.0	20.2	3.5	4.3	6.4	17.4	4.0	15.2
ZNF428	40.5	14.0	18.4	3.0	5.5	7.1	15.3	2.9	13.5
SEC11A	33.3	12.2	18.7	1.2	4.0	5.5	24.4	2.6	18.0
FOXP1	31.4	7.8	18.3	3.4	8.2	10.7	21.6	5.6	12.8
HIPK2	33.7	10.7	14.0	5.0	9.3	11.9	16.2	3.5	15.5
MCM2	25.2	8.5	11.7	3.8	6.4	8.9	28.9	4.9	21.5
LOC100506792	42.4	8.0	16.2	3.0	3.2	5.5	29.0	2.4	10.0
GRHPR	32.5	12.9	16.5	4.1	7.1	10.2	20.1	3.1	13.2
BICD2	31.0	11.6	15.4	3.5	5.9	7.5	23.7	4.2	16.7
QSOX2	31.5	12.6	16.2	2.5	4.7	7.3	24.4	3.2	16.9
MKNK2	35.4	13.9	16.9	3.5	5.9	7.8	18.2	3.7	13.8
LOC284454	34.1	8.2	15.6	2.0	4.6	6.2	30.7	2.7	15.0
HP1BP3	33.3	11.3	16.7	1.6	5.4	7.3	22.4	3.0	18.1
SF3B3	31.2	11.0	14.4	2.0	6.0	7.8	25.7	3.2	17.7
DVL1	29.0	11.6	14.7	4.3	7.0	10.4	19.2	3.8	19.0
TOR1AIP2	30.9	10.8	14.9	1.3	4.7	5.3	26.5	3.0	21.5
MYO9B	32.5	10.8	14.9	3.2	5.8	7.7	23.2	3.7	17.0
UBALD1	28.9	13.2	15.3	5.0	7.0	10.8	12.8	5.5	20.4
PES1	28.2	11.1	13.4	2.7	6.1	7.7	25.3	4.4	19.8
MIDN	33.3	14.7	15.3	6.7	7.0	9.9	13.0	4.3	14.7
FBLIM1	33.8	11.5	14.4	2.8	4.9	6.2	26.2	3.5	15.4
PAF1	28.7	13.3	16.3	3.2	5.0	6.8	20.7	4.9	19.8
CAPN1	34.9	13.4	16.4	3.3	5.8	8.6	18.7	3.4	14.3
SHKBP1	32.2	14.6	18.3	3.7	6.6	9.1	17.3	3.0	13.9
PPDPF	22.9	12.6	15.3	8.7	13.3	17.7	8.9	4.3	14.9
CAPZA1	32.2	14.0	18.7	0.8	4.9	7.7	18.6	3.4	18.5
MBNL1	34.6	11.5	14.7	2.3	4.7	7.4	19.5	3.6	20.3
PTPRM	32.8	11.8	15.6	2.3	6.0	9.0	19.5	3.8	17.8
LOC284023	38.1	7.7	16.7	2.6	4.7	5.4	28.6	2.4	12.4
C11orf48	24.3	10.0	14.3	5.0	8.3	11.9	21.7	4.2	19.0
THEM6	33.4	14.6	16.1	6.6	6.5	11.9	15.4	2.6	11.6
UNC45A	32.8	12.7	15.9	2.3	4.4	6.5	25.5	3.5	15.0
DDTL	15.7	6.9	22.2	6.6	4.3	14.6	28.0	2.5	17.8
HNF1A-AS1	37.3	7.6	17.2	2.2	4.2	5.0	29.1	2.4	13.3
MANF	24.8	9.8	12.5	0.9	4.7	4.8	31.1	6.7	22.8
METAP2	32.4	11.6	15.3	1.5	4.5	6.5	23.4	3.3	19.4
HCFC1	32.4	11.6	15.1	4.6	6.3	8.2	18.2	4.5	16.8

GADD45B	27.1	9.6	13.5	4.1	6.1	8.5	24.2	4.8	19.9
NPTN	39.2	13.7	16.9	1.7	4.8	6.8	17.0	3.2	14.4
TMEM158	23.8	12.3	14.9	12.3	14.4	18.0	9.6	3.0	9.5
TOMM22	25.0	9.7	13.6	2.8	5.8	6.6	28.4	4.8	21.0
STEAP3	29.9	13.0	16.1	4.4	7.1	10.1	17.6	4.2	15.1
ADO	27.0	10.8	12.6	5.5	6.9	9.2	22.6	4.8	18.2
RNF126	24.9	9.8	13.3	6.1	7.7	10.8	20.9	5.3	18.8
IDH3B	31.2	12.7	16.2	2.6	5.9	7.6	22.0	2.9	16.4
MDH1	31.9	11.1	18.1	0.9	4.1	5.9	26.8	2.5	16.1
SBDS	27.1	10.9	14.1	1.1	4.7	7.4	24.8	4.2	23.1
FLJ46363	34.8	9.4	17.7	4.2	5.8	6.9	24.0	3.0	11.6
OSER1	30.2	11.2	17.9	1.4	5.3	5.8	22.0	4.6	18.9
UCK2	22.1	8.5	11.6	2.6	5.8	7.3	28.9	5.3	25.3
TRAF7	31.5	12.1	15.5	3.6	7.1	9.5	18.3	3.3	16.1
CDC42EP2	24.4	10.4	14.5	4.6	6.1	10.4	20.4	5.4	21.1
RAB1A	28.8	12.8	17.3	0.8	4.4	6.2	21.3	3.1	22.5
RHEB	28.7	11.8	15.9	0.5	4.0	7.5	23.5	3.4	21.6
CHST11	28.3	10.1	12.7	4.5	7.4	11.6	19.4	4.4	18.6
AKT2	31.1	13.2	16.1	3.2	6.7	8.5	17.6	3.2	17.3
ATP6AP1	34.0	14.5	17.8	4.3	6.3	7.3	15.5	3.4	13.6
ANXA7	34.5	14.2	18.7	1.6	4.9	7.9	19.0	2.0	13.8
KHDRBS1	27.9	9.9	13.1	3.2	5.6	7.6	25.2	4.1	20.1
CLTC	33.6	10.9	13.9	1.1	4.5	6.3	26.3	2.8	17.2
BOLA2	27.3	10.8	16.5	3.3	6.2	7.9	24.1	3.4	17.1
C9orf69	38.6	15.4	18.6	5.4	7.3	9.6	11.2	2.2	8.2
RBM4	28.0	10.1	15.3	1.2	5.8	6.6	20.1	6.4	22.9
ACTR1A	32.0	11.8	14.5	2.0	5.0	8.0	21.7	3.6	17.7
WIZ	33.0	10.3	15.3	3.4	5.2	10.0	20.1	3.0	16.0
DNMT1	27.5	9.7	12.4	3.0	6.1	8.1	25.2	4.5	19.8
SCYL1	35.3	13.6	16.2	3.3	6.4	8.1	17.1	3.2	13.1
TIMM50	29.9	11.5	15.7	2.9	6.2	7.3	23.0	3.5	16.1
NDUFA4	29.4	15.3	18.6	0.8	4.2	7.6	21.5	3.0	15.7
MRP63	24.6	11.5	15.0	4.3	7.9	9.0	22.5	4.4	16.9
EPS8L2	33.1	14.4	19.1	4.4	7.6	9.6	14.1	3.0	10.7
RCN3	37.3	14.1	18.5	5.5	7.6	9.6	11.8	2.0	9.4
CENPB	28.3	12.7	14.1	3.4	6.3	8.4	22.6	3.5	16.5
PXDC1	29.0	10.0	12.4	3.0	5.6	7.0	25.8	3.9	19.4
SERPINE1	20.3	8.5	10.5	3.6	7.2	10.7	25.3	4.8	24.9
CCDC6	28.9	11.8	14.3	3.5	5.4	7.4	19.9	4.5	20.1
SNAPC2	32.5	10.5	13.8	2.8	4.6	7.4	23.5	4.3	16.4
FAM136A	26.9	10.8	11.9	2.0	5.8	5.9	23.5	4.1	25.0
CTDSP1	34.2	14.2	16.8	3.0	5.4	9.3	17.8	2.3	12.9
INO80E	26.0	11.1	15.7	3.8	6.8	8.8	23.7	3.3	16.5
LAMC1	34.9	11.5	15.0	2.7	6.7	8.6	19.7	2.9	13.6
SLAMF7	23.6	9.5	13.4	1.5	5.2	7.1	28.3	4.4	22.4
YIF1A	32.7	13.6	20.3	3.7	8.2	9.5	12.2	2.4	12.7
COL6A2	34.8	14.0	18.0	5.2	8.6	11.4	11.7	2.3	9.6
ATP5O	27.7	11.6	16.7	2.1	5.1	7.8	23.6	3.4	17.5
TRIP6	28.9	10.9	13.7	3.7	6.1	8.0	23.4	4.3	16.4
PLOD1	34.6	13.8	17.2	3.0	6.3	8.8	15.8	2.7	13.1
MAFF	26.2	6.6	12.0	3.4	3.7	6.3	35.4	4.7	17.0
TSC22D2	32.9	10.8	14.0	3.3	5.2	6.9	22.2	3.9	16.1
ABCD4	32.8	12.7	18.2	2.5	5.5	7.6	18.7	2.8	14.3
TMEM132A	42.6	15.9	21.9	3.5	5.2	7.7	10.2	1.6	6.6
CHCHD1	33.5	13.2	19.2	2.8	5.7	6.7	18.4	3.1	12.3

FAM213A	33.1	13.9	18.1	1.4	3.5	5.0	22.6	3.2	14.2
AATF	27.5	10.7	12.7	2.0	5.5	7.1	25.5	3.8	20.1
MRPL17	25.0	11.3	14.0	4.3	7.0	8.5	24.4	4.4	16.0
FUT8	34.8	13.0	18.2	2.5	5.4	7.8	17.7	2.4	12.9
KLC2	32.8	11.8	15.4	3.2	5.2	7.8	19.4	3.6	15.6
NEDD4L	27.5	10.3	13.3	3.2	6.6	10.6	19.3	4.6	19.4
DIAPH1	32.5	11.3	14.8	1.9	4.5	5.9	24.0	3.4	16.6
SNORA74A	30.3	16.8	20.9	0.9	2.5	3.2	19.4	2.0	18.6
SRP68	28.0	10.3	13.2	1.6	4.4	5.4	25.9	4.1	21.6
OGFR	30.5	12.8	15.2	4.7	6.5	9.3	18.5	3.6	13.4
SPIRE1	32.7	10.6	13.8	1.8	4.2	4.9	25.5	3.3	17.7
ATP5H	32.9	12.7	19.7	1.3	5.3	9.1	18.3	2.3	12.8
H3F3A	29.5	12.1	17.6	2.0	5.5	8.6	20.3	3.2	15.6
MRPL38	29.7	11.9	16.3	3.8	7.3	8.7	21.1	3.2	12.3
SAMD4A	30.6	9.5	14.0	3.0	6.1	6.6	20.9	4.9	18.5
SEC24C	28.9	11.3	15.5	2.4	5.6	7.9	20.9	3.9	17.7
ARL1	32.9	13.1	17.0	1.3	3.7	4.8	19.9	2.9	18.6
LOC100190986	38.1	6.7	16.6	2.1	5.3	5.5	25.3	2.0	12.7
LOC440157	31.6	7.1	15.0	5.8	9.6	11.1	20.7	2.1	11.0
CSE1L	28.8	10.5	13.7	1.0	4.5	6.5	25.3	2.8	20.9
PAK2	29.6	10.6	12.3	1.2	4.2	6.7	24.9	3.4	21.2
HCFC1R1	25.4	12.9	15.3	5.7	9.3	11.8	15.7	4.6	13.3
RNU6ATAC	36.8	12.3	18.0	6.1	7.1	6.0	13.8	3.0	10.7
MAEA	30.4	11.0	14.9	3.3	5.6	7.3	22.1	3.4	15.7
TRNN	39.7	19.2	11.8	7.2	7.4	7.3	8.1	2.2	10.6
CD68	33.8	14.7	18.1	4.8	8.5	12.1	8.4	2.6	10.5
UAP1	21.9	9.9	12.0	0.9	3.7	5.2	32.4	3.2	24.3
AP1S1	31.8	13.0	16.5	1.7	3.9	6.0	23.0	2.2	15.5
RNASEK-C17orf49	30.8	12.3	16.7	3.0	6.2	9.5	18.1	3.2	13.6
DYNLT1	30.6	13.1	18.8	1.2	4.8	6.7	18.8	2.6	16.8
EFTUD2	30.6	10.5	14.9	2.0	5.5	7.9	22.4	2.9	16.6
PPAN	28.5	12.1	15.8	3.7	4.6	7.4	21.7	3.4	16.1
MRPL10	30.3	11.9	16.2	3.0	5.5	7.9	17.7	3.6	17.3
HADHB	30.6	11.0	15.8	1.2	4.4	6.3	26.6	2.7	14.7
SNORA80	23.6	12.5	17.2	4.5	8.5	8.4	23.6	3.8	11.1
VPS4A	29.8	11.8	15.3	2.5	4.9	7.9	20.5	3.6	16.8
MVB12A	30.5	12.4	15.7	3.6	7.1	10.4	19.6	2.1	11.6
RBFOX2	29.6	11.4	13.0	2.9	5.8	7.4	20.5	4.0	18.5
HEXB	32.8	13.5	17.7	1.9	5.4	6.7	19.1	2.9	12.9
CBX3	26.3	7.9	15.2	0.4	2.7	4.6	29.9	2.0	23.9
ARL8B	32.6	13.1	17.5	3.0	5.8	6.6	17.0	2.5	14.8
NUDT21	26.2	11.9	13.8	2.0	5.7	8.7	21.8	3.3	19.4
WASF2	31.7	10.8	14.0	2.6	5.2	7.5	19.7	3.7	17.6
EI24	28.8	11.1	14.6	1.7	4.8	6.3	23.9	2.7	18.7
FAM89B	29.1	12.3	14.4	3.6	5.7	7.6	21.4	4.2	14.3
IFRD2	28.0	9.1	13.0	3.8	5.4	8.5	27.0	2.6	15.2
NUP62	27.1	10.8	13.3	3.6	6.0	8.7	18.2	4.5	20.3
PCIF1	27.8	10.2	13.0	3.2	4.8	7.8	22.5	4.3	19.0
TMX1	30.0	11.2	14.5	1.8	3.5	5.3	25.8	3.0	17.6
GART	23.4	6.7	11.1	0.9	4.2	5.4	36.0	2.8	22.2
SPTAN1	32.0	10.4	14.1	1.8	4.2	6.0	24.2	3.2	16.5
ANAPC16	35.4	9.3	16.6	1.8	5.4	6.0	22.6	2.3	12.9
BZW1	23.3	9.7	12.1	1.1	3.9	4.6	31.5	3.2	22.8
CHID1	31.2	12.1	17.1	2.9	5.4	8.2	19.2	3.0	12.9
IPO7	28.3	10.6	13.3	1.1	4.5	6.5	24.0	3.2	20.6

TCEA1	31.0	11.8	14.1	1.4	5.0	5.1	23.3	2.9	17.5
LSS	37.1	15.0	19.2	2.0	4.5	6.0	15.4	2.3	10.5
MRPL53	23.9	10.7	13.4	3.6	6.8	9.8	25.9	3.3	14.5
NELFE	25.8	11.2	15.0	3.0	6.5	10.3	19.9	3.4	16.9
WDR6	29.3	11.7	15.7	2.8	5.6	7.1	20.3	3.5	15.8
ARF4	28.3	11.3	14.3	0.7	3.8	4.6	25.0	2.5	21.2
UNG	26.2	9.7	12.4	1.8	5.0	6.3	28.1	3.8	18.7
DMPK	33.0	12.3	16.7	2.8	4.8	6.7	19.6	2.6	13.2
CSNK2A1	26.7	14.0	12.5	2.0	4.6	9.4	21.2	3.1	18.2
DPAGT1	27.5	11.2	14.9	1.9	5.9	8.7	20.7	3.3	17.6
TIAL1	30.7	11.6	16.7	1.1	4.3	6.6	21.1	2.5	17.0
IST1	31.4	11.8	15.6	1.8	4.1	5.0	22.1	2.8	16.9
GTF3A	30.5	12.1	17.8	3.1	4.3	8.0	17.9	3.5	14.4
TALDO1	28.4	11.3	14.2	4.5	8.4	11.7	16.2	3.1	13.7
SSU72	31.0	12.4	14.5	2.6	4.4	7.3	20.9	3.1	15.4
MBTPS1	31.9	11.7	16.2	2.3	6.6	8.7	17.8	2.8	13.7
ZNF581	22.9	9.8	11.1	2.4	4.8	6.5	24.3	5.5	24.2
MCCC1	29.0	10.8	13.8	3.0	7.4	9.7	19.8	2.9	15.0
TAF9	26.9	10.8	14.8	1.2	4.6	6.3	21.0	4.0	21.7
SPTLC1	32.0	10.4	15.2	1.5	4.6	5.5	22.4	3.3	16.4
GLS	32.6	9.4	16.1	2.3	3.4	5.7	22.6	3.9	15.4
PTPRS	34.4	12.8	16.5	4.9	8.0	11.4	11.6	2.1	9.5
GNA11	31.1	11.4	15.2	3.0	5.5	6.4	19.7	3.3	15.6
FOXP4	34.0	13.3	16.3	3.7	6.0	7.1	15.0	3.5	12.3
CHTOP	26.4	9.9	12.6	1.8	4.6	7.6	25.4	3.9	18.9
CTDSP2	32.9	12.0	15.6	1.7	3.9	5.5	21.6	3.1	14.8
LSMD1	29.2	12.3	16.7	3.6	7.5	9.9	16.7	2.7	12.4
AMOTL1	29.7	9.9	13.1	4.1	7.1	9.8	17.8	3.6	15.5
KLHL21	28.2	9.6	13.3	3.8	5.3	6.8	24.2	4.1	15.4
WBP5	26.2	9.9	15.7	0.7	5.2	4.8	22.4	3.7	21.8
DLAT	31.6	11.5	14.8	2.3	4.9	6.9	18.6	3.2	16.7
SRI	31.1	12.8	17.7	1.6	5.6	6.9	17.3	2.9	14.4
XRN2	30.1	11.2	15.1	1.3	4.1	6.0	21.3	2.9	18.4
KLF2	22.3	10.3	13.1	6.9	7.4	10.4	17.4	5.4	17.3
COL6A1	28.2	12.3	16.0	7.4	12.0	15.2	9.3	2.1	7.8
UBE2D2	28.2	10.0	14.3	1.1	4.7	6.9	22.5	3.8	18.8
AAR2	29.9	11.4	13.8	1.8	4.6	7.3	20.1	3.8	17.6
EIF2S3	28.8	12.3	14.7	2.0	5.4	6.4	19.5	3.1	18.0
VAC14	31.5	10.7	12.8	3.3	5.6	7.7	18.9	3.6	16.0
SNORA38	27.2	14.5	14.1	1.1	3.3	2.6	35.1	1.0	11.3
COPA	33.0	11.4	14.8	1.4	4.9	6.3	21.5	2.7	14.2
C16orf13	26.6	10.3	11.7	4.6	7.2	8.9	21.5	3.9	15.3
CHMP2A	33.1	12.1	15.7	3.9	6.5	9.4	15.2	2.5	11.6
NDST1	30.8	11.1	14.8	3.6	5.4	8.0	17.6	3.4	15.1
SUMF2	27.1	10.7	14.2	3.0	6.7	8.9	21.8	2.6	14.9
ZDHHC5	28.8	12.2	14.4	2.4	5.0	7.5	17.0	3.7	18.9
ACO2	32.5	13.4	16.3	3.1	5.7	7.8	15.2	2.7	13.0
FBRSL1	29.1	10.9	13.5	3.8	5.7	7.2	19.7	4.0	15.7
LGALS8	35.4	12.3	13.9	1.6	6.0	7.3	14.8	2.6	15.8
LSM12	27.8	10.6	13.0	1.6	4.7	7.3	19.0	4.0	21.6
ZNHIT1	31.1	10.8	16.4	1.7	3.7	6.4	24.5	2.4	12.7
CCNL2	34.9	14.9	20.9	2.4	4.7	5.7	13.7	2.0	10.6
BAD	28.6	12.6	16.8	4.8	5.9	7.9	16.5	3.3	13.1
MMS19	22.7	9.0	11.7	2.2	4.9	8.2	27.0	4.0	19.8
PDZD8	23.8	11.1	12.8	2.3	6.3	6.7	25.8	3.3	17.4

SNORA44	23.8	10.4	13.4	7.4	7.1	10.5	16.1	5.3	15.6
SLC16A3	29.6	11.5	15.0	3.4	6.3	8.3	19.2	3.0	13.3
DDRGK1	31.1	10.5	15.1	2.3	4.8	5.8	20.9	3.2	15.6
ETV5	26.9	10.8	12.1	2.3	4.5	7.2	20.7	4.3	20.6
PPP1R16A	33.5	12.6	15.7	5.0	6.8	9.1	13.7	2.8	10.1
PTPRA	32.6	12.9	15.2	1.6	3.9	5.6	18.7	2.6	16.3
RXRB	30.2	12.2	14.0	2.8	4.5	6.3	18.8	3.4	17.1
MTSS1L	27.5	11.9	14.1	4.8	6.0	7.8	16.2	4.2	16.7
ZNF593	25.3	9.1	15.9	2.8	4.8	6.5	22.8	3.8	18.2
WDR74	27.4	11.4	14.3	3.0	5.6	7.9	21.0	3.4	15.2
NOP14	24.9	9.2	12.3	2.5	5.6	7.6	24.5	3.7	19.0
PDCD2	19.9	8.7	10.7	3.2	6.2	9.3	26.2	3.9	20.9
COMMD5	27.0	10.4	16.0	2.8	5.3	6.7	24.1	2.7	13.9
CDIPT	27.6	11.6	14.9	2.4	5.2	8.6	18.8	3.8	16.0
SOX12	26.3	10.4	12.3	5.3	7.0	9.4	18.9	4.2	15.1
RCC1	29.9	11.6	15.9	1.8	3.6	5.3	21.0	3.2	16.7
EIF5	25.5	8.9	13.0	0.7	3.4	3.9	27.7	3.9	21.8
CYTH2	28.0	12.0	14.4	2.8	6.3	7.8	19.6	3.6	14.3
FAM127A	35.0	12.0	13.5	3.9	6.7	8.7	17.6	1.8	9.6
SLC9A3R1	33.5	12.6	15.1	2.8	3.8	6.0	18.1	3.5	13.2
ADAM9	32.7	11.7	14.3	1.0	4.4	5.6	21.3	2.2	15.5
COMMD7	30.8	11.2	18.2	1.3	3.5	5.3	21.5	2.6	14.3
C19orf60	34.0	12.0	18.7	6.2	8.1	11.4	10.8	1.8	5.6
NECAP2	29.8	12.7	15.7	2.5	5.0	6.7	18.1	3.1	15.1
NUFIP2	23.9	9.2	12.5	1.6	4.4	5.5	24.1	4.5	22.8
TNK2	35.5	12.9	14.1	3.8	5.8	7.4	16.3	2.6	10.1
ZC3H11A	28.5	9.0	12.9	1.5	4.9	5.9	23.6	3.0	19.0
CYBRD1	30.6	12.1	15.2	1.5	5.0	6.5	18.3	2.7	16.3
PATL1	30.5	11.5	13.9	1.6	4.7	6.3	18.9	3.5	17.3
ACSL4	28.8	10.2	13.3	0.9	4.0	5.3	24.0	2.7	19.0
HSD17B12	34.3	12.9	17.3	1.1	3.6	5.7	17.3	1.9	14.0
NDUFS7	27.6	12.6	15.6	4.5	6.2	9.4	16.5	3.0	12.5
DDX39B	21.7	9.5	12.8	2.2	6.3	3.3	26.2	5.2	20.6
GATC	28.7	9.9	14.1	1.8	5.1	5.8	22.5	3.6	16.5
TAF6	29.5	10.7	16.0	3.0	5.7	7.2	17.4	3.5	14.6
MAFG	26.7	10.0	12.9	3.8	5.5	6.6	22.9	3.8	15.7
TRIB2	39.6	15.8	20.3	1.6	4.8	5.9	10.2	1.5	8.1
REXO4	27.0	10.8	13.4	2.2	5.2	6.8	21.7	3.4	17.2
ENG	29.7	13.1	15.2	3.9	5.9	8.4	13.3	3.4	14.7
QARS	28.8	11.3	14.0	2.4	6.8	7.9	19.8	2.8	14.0
MXI1	28.4	12.5	14.3	2.4	5.2	7.7	17.2	3.3	16.7
USMG5	25.3	10.0	14.4	0.8	5.3	5.8	21.5	3.5	20.8
TCF25	33.3	12.6	15.5	3.7	6.9	9.7	13.2	2.4	10.1
MPG	27.8	11.5	17.7	4.7	6.9	9.2	17.0	2.5	10.0
YTHDF1	25.7	10.1	13.6	2.5	4.9	6.9	19.4	4.5	19.8
TMEM18	27.0	11.0	14.1	4.1	4.9	10.1	19.2	3.6	13.3
DAXX	28.3	11.8	13.3	2.3	4.7	6.9	19.8	3.9	16.4
ANTXR1	31.9	11.7	14.1	3.0	6.8	9.0	15.0	2.7	13.0
VMP1	27.0	10.0	16.5	1.4	4.2	5.2	24.5	3.0	15.5
LAPTM5	33.3	14.5	16.5	3.5	5.6	8.7	11.6	2.6	11.0
GLOD4	30.1	11.3	14.5	1.1	3.3	4.6	23.7	2.3	16.5
SIVA1	26.4	9.9	16.0	2.5	4.1	6.3	24.5	2.6	15.0
TM9SF1	29.4	12.2	15.8	2.1	4.3	7.5	18.7	2.9	14.1
CWC15	27.4	11.1	16.2	0.9	4.4	6.4	19.3	2.8	18.5
SHFM1	32.1	9.0	14.5	1.8	3.8	4.5	21.2	3.1	17.0

ITGB2	34.4	14.2	16.7	2.4	5.1	5.3	14.8	2.8	11.1
FADS3	22.9	10.5	12.8	3.0	5.9	8.6	21.3	4.3	17.4
IGBP1	29.0	11.1	14.8	1.1	3.8	5.4	22.6	3.0	16.1
HARS	28.2	11.9	15.3	2.0	5.6	6.1	21.2	2.8	13.7
TSPAN4	31.1	13.1	15.9	4.2	8.2	9.4	13.7	1.4	9.8
ADAR	29.5	11.1	14.2	1.9	5.2	7.2	19.2	3.0	15.5
TMEM189	30.8	12.0	14.8	2.9	5.2	7.0	15.9	3.3	14.6
POLG	32.3	12.3	17.3	2.3	4.6	6.3	17.6	2.9	11.0
STK40	25.8	9.8	11.5	2.8	5.4	8.2	22.8	3.4	16.9
CIAO1	26.1	9.7	11.9	2.3	4.9	6.7	26.2	3.0	15.7
SRSF4	27.6	8.8	13.5	2.3	3.9	5.1	22.4	4.6	18.1
CTSC	23.8	9.3	13.2	2.2	5.8	7.8	22.2	3.8	18.3
TMED4	25.3	10.0	13.8	2.5	5.8	6.9	22.2	3.7	16.1
MPV17L2	25.3	12.0	13.2	3.3	6.3	8.0	17.4	3.7	17.1
FAM189B	29.9	11.6	15.6	4.1	6.3	9.3	14.0	3.3	12.1
DAP3	23.8	9.2	12.1	0.9	4.5	6.6	24.0	3.0	22.1
DR1	24.9	9.1	12.1	0.9	4.0	5.8	25.3	4.1	20.1
RDX	30.8	11.4	14.2	0.9	3.7	5.6	19.9	2.8	16.9
PLCD3	27.0	11.3	14.7	4.6	7.4	10.6	15.3	2.7	12.6
TAF8	36.7	8.3	15.3	2.7	6.3	6.7	12.6	2.9	14.6
NAPA	27.7	10.9	13.7	3.0	6.2	8.5	18.8	3.1	14.2
ZDHHC8	30.1	12.9	15.4	4.8	6.0	9.1	13.7	2.9	11.1
SIPA1L3	28.2	9.8	12.4	5.1	8.0	10.5	15.4	3.3	13.3
ADSL	28.5	10.0	15.0	1.2	4.8	6.6	22.8	2.5	14.6
POLRMT	28.6	10.9	13.5	3.8	6.1	8.5	18.8	3.2	12.5
NUS1	26.8	10.8	13.0	2.3	4.1	7.0	22.0	3.6	16.2
LAPTM4B	34.2	11.4	14.8	2.0	4.5	5.3	20.5	1.7	11.2
CREB3L2	31.2	8.2	14.1	3.6	7.3	7.8	15.4	3.4	14.9
BNIP3L	29.0	12.0	14.9	1.5	5.0	6.1	19.0	2.7	15.7
SERPINE2	43.0	17.2	21.9	0.9	2.8	4.0	8.2	1.3	6.3
ATP5C1	28.6	11.1	15.1	1.4	4.7	6.1	19.0	3.1	16.5
SBF1	30.3	11.0	14.4	3.7	6.2	8.2	17.2	2.6	12.0
ZMIZ1	30.0	9.7	13.2	4.9	7.9	10.8	12.8	2.9	13.3
TMUB2	25.2	12.1	18.0	2.5	4.7	7.5	17.9	2.6	14.9
SLC44A2	31.0	11.0	13.9	2.5	5.9	7.6	18.3	2.5	12.7
TOMM7	27.7	9.7	15.4	0.6	3.9	4.9	26.0	2.7	14.6
TTYH3	32.1	11.5	15.3	5.0	7.4	9.1	13.5	2.4	9.0
FKBP4	25.3	9.5	12.0	1.9	4.2	6.2	24.5	2.6	19.1
REEP3	33.0	12.0	16.8	0.7	2.8	4.3	15.1	2.6	17.9
ATP5SL	27.1	11.1	14.4	2.2	4.9	6.0	20.8	3.3	15.5
DUS1L	27.5	10.5	14.3	3.3	6.5	8.4	18.4	3.3	12.9
MLXIP	27.2	9.4	13.3	4.0	5.5	8.1	16.0	3.9	17.6
C14orf178	36.5	5.2	13.9	2.1	3.8	4.6	27.2	1.5	10.3
BAP1	31.5	11.8	15.4	2.3	5.0	6.3	16.4	3.2	13.2
SNORA74B	27.8	14.3	19.2	2.0	6.1	3.7	21.4	1.7	8.9
CXXC1	29.0	11.4	14.5	2.5	4.6	6.4	19.6	3.0	13.9
LARP6	30.2	12.1	13.6	2.7	4.7	6.1	17.6	3.8	14.1
TNFRSF10D	26.9	8.5	12.6	1.4	3.5	4.6	23.4	3.7	20.1
CPSF3L	30.7	9.2	14.8	2.9	5.2	6.4	20.2	2.8	12.7
MRPS18B	27.4	13.3	14.7	2.3	6.4	8.4	13.3	2.8	15.9
NANS	23.1	8.8	11.5	1.4	3.8	5.7	26.1	4.4	20.0
CHAC1	19.8	9.2	10.2	3.5	4.2	7.4	23.2	4.8	22.3
OSTC	24.8	8.2	12.4	1.4	4.2	6.1	25.8	3.3	18.4
PAFAH1B3	28.6	11.3	15.2	2.2	4.0	6.3	19.7	2.9	14.5
MRPL24	24.2	11.1	14.4	3.1	8.3	10.5	15.3	2.7	14.8

ENAH	27.4	10.1	12.5	2.2	5.2	5.9	18.8	3.4	18.7
SRRT	25.9	9.6	12.8	2.7	4.9	6.6	20.5	4.2	17.1
ADAM17	31.6	10.9	15.3	1.1	3.2	4.7	19.4	2.4	15.6
GNA12	29.4	9.9	13.3	2.8	4.8	6.3	21.5	3.3	12.8
ARPP19	29.3	13.0	15.8	1.2	4.9	6.3	16.9	2.6	14.0
NDUFB5	31.1	10.0	19.3	1.9	3.6	6.3	20.1	2.0	9.8
ATP6V1E1	30.7	12.7	15.9	1.1	3.8	5.9	16.8	3.0	14.1
CYB5B	25.4	10.0	14.3	2.0	3.2	5.6	22.4	3.6	17.4
GOLGA3	30.1	9.6	14.4	2.8	5.3	7.0	18.9	2.5	13.3
WDR18	27.2	11.0	13.8	4.9	6.9	9.2	14.5	3.5	12.9
CCNB1IP1	28.3	12.3	18.8	1.2	5.5	6.7	15.3	2.4	13.5
NAA10	24.1	10.7	14.0	3.1	5.5	8.1	18.5	3.2	16.5
B4GALT1	37.1	12.8	17.5	3.0	4.2	7.2	12.0	2.2	7.8
TES	29.3	9.8	13.1	1.2	3.1	5.1	23.7	3.0	15.5
GNS	34.6	12.2	16.1	1.3	3.9	5.2	15.5	2.2	12.8
NFIX	27.5	10.4	13.6	3.6	5.8	7.6	16.8	3.6	14.8
ABHD8	30.7	11.0	15.7	3.0	5.1	5.8	16.5	3.4	12.2
C12orf75	20.9	9.5	13.5	1.9	5.3	6.8	19.2	5.0	21.2
LZTS2	28.5	12.2	15.5	3.2	5.1	6.7	16.5	3.1	12.5
ZFR	30.4	11.3	14.6	1.8	5.0	6.7	15.6	2.7	15.2
ZNF207	29.1	10.2	14.7	1.6	4.3	6.7	17.1	3.2	16.3
ACSL5	27.8	11.0	14.6	1.4	4.6	6.4	20.0	2.7	14.7
MANEAL	10.6	19.4	14.6	8.8	7.0	2.6	17.3	9.0	13.8
USP11	29.0	11.2	14.4	2.8	5.2	7.8	16.9	3.2	12.8
ARHGAP35	31.7	10.2	13.3	1.7	4.4	6.1	17.8	2.7	15.1
TOP1	28.2	10.6	12.6	1.4	4.5	5.9	20.0	2.8	17.0
LRRC47	29.9	11.3	14.1	3.1	4.7	6.7	18.6	3.2	11.4
LOC101927812	29.5	8.8	14.1	3.2	4.6	5.4	21.2	3.6	12.5
KRT80	28.5	11.2	14.0	2.4	4.2	6.0	19.7	3.5	13.4
SERINC1	29.6	10.9	14.6	0.8	3.7	5.3	19.4	2.5	16.1
TMEM38A	32.5	13.7	17.5	2.7	5.1	7.5	11.9	2.1	10.0
NDUFC1	24.8	13.4	18.1	3.6	5.7	9.1	9.5	3.0	15.7
RHOBTB3	27.2	10.1	13.8	1.1	3.3	4.7	21.9	3.2	17.6
ACOT2	29.5	11.7	15.9	2.1	4.8	6.1	18.9	2.2	11.7
TRIAP1	27.0	10.4	14.0	2.3	3.4	6.2	17.6	3.9	18.0
NUMB	28.7	10.4	12.3	2.3	5.4	7.6	17.0	3.4	15.7
EHD4	21.8	8.3	11.0	3.3	4.5	6.6	23.9	4.5	18.8
TBRG4	25.0	9.9	13.3	2.1	4.6	6.3	22.6	3.0	15.8
ZNRF1	27.0	9.0	14.1	2.2	4.5	5.3	22.9	3.7	13.9
COMMD3	29.4	12.4	19.0	2.6	4.7	7.6	14.1	1.6	11.1
PSMA6	26.1	13.1	17.7	1.2	3.9	5.9	18.1	2.3	14.3
LSM14A	27.1	11.5	12.8	2.3	5.0	6.8	15.8	3.3	18.1
PRKAR2A-AS1	32.5	6.9	14.3	1.8	3.9	4.3	25.5	2.0	11.3
MRPS23	28.9	9.4	18.1	1.5	4.4	4.5	20.9	2.1	12.6
TCP11L2	35.5	10.9	14.3	0.8	3.3	4.6	20.0	1.7	11.2
CREG1	25.2	10.2	12.5	3.2	5.0	8.6	21.9	3.4	12.1
LIMA1	23.9	9.5	12.3	1.6	5.9	7.6	19.1	3.6	18.6
UCKL1	28.8	10.5	15.1	3.3	5.3	7.7	15.4	2.9	13.2
ATP13A1	31.5	10.1	13.2	3.8	6.4	8.1	15.0	2.5	11.5
FZD8	26.2	11.4	14.0	5.5	6.8	8.5	16.7	2.9	10.1
UBE2Z	23.2	9.3	11.9	4.3	7.0	8.3	18.9	3.4	15.7
APBB2	27.4	8.8	9.9	2.1	5.1	7.8	19.3	3.1	18.5
TCIRG1	33.1	12.9	19.3	3.3	5.4	7.6	10.8	1.7	7.8
TMEM127	29.6	9.6	13.7	3.5	5.8	7.6	17.9	2.1	12.1
LOC101927279	32.8	6.4	14.6	1.9	4.0	4.0	26.0	1.8	10.2

CTNNBL1	24.8	9.9	12.3	1.3	4.3	5.6	22.6	3.1	17.8
POLR2C	28.1	9.9	12.6	1.5	3.8	5.0	21.9	3.1	15.8
MITF	24.6	10.2	11.4	1.6	4.8	5.4	19.7	4.3	19.6
PITPNM1	28.8	10.8	14.3	3.7	5.7	7.5	15.8	3.1	12.0
CXCL1	40.6	18.6	26.8	2.1	3.2	4.5	2.7	0.7	2.3
DMWD	27.8	10.5	14.2	3.8	5.3	7.6	16.6	2.8	12.7
LOC100506688	31.9	6.4	13.4	2.1	4.2	4.4	25.1	1.8	12.2
CYTH3	28.5	10.2	13.0	2.1	5.3	6.2	16.5	3.4	16.1
PSMD9	25.1	8.9	13.6	3.3	5.0	7.8	20.3	2.6	14.9
LPIN1	32.9	12.2	15.7	1.6	3.4	4.4	15.8	2.3	13.3
WASH1	29.7	12.1	16.5	2.9	4.8	6.9	14.5	2.9	11.1
ANP32A	20.5	7.2	8.9	1.7	5.4	5.8	27.6	3.6	20.8
EEF1A2	28.4	12.3	15.4	3.3	5.0	6.9	15.3	2.7	12.1
CGGBP1	27.4	12.3	16.6	1.1	4.4	6.4	15.9	2.6	14.6
MOGS	25.4	10.3	13.6	2.6	4.8	6.6	19.6	3.5	15.1
ZNF496	25.3	6.5	10.1	2.1	3.6	5.8	26.4	3.4	17.9
IL18	29.0	11.6	15.9	0.1	1.8	2.7	23.1	2.5	14.5
LAMTOR2	31.0	10.9	18.2	2.2	5.2	6.8	13.5	1.9	11.4
LEMD2	28.0	11.7	15.0	2.4	4.4	5.5	19.8	2.8	11.5
CDK6	23.5	6.9	11.7	2.2	5.1	7.0	24.0	3.6	16.9
CDH4	25.7	10.3	12.1	3.7	6.2	8.0	15.7	3.8	15.5
MRPL28	25.1	11.3	13.1	2.7	5.8	8.1	14.9	3.8	16.0
STX5	25.7	11.1	13.0	2.5	5.6	8.6	15.2	3.3	15.8
HSD17B10	25.9	10.9	15.7	2.1	4.5	6.2	19.7	2.3	13.6
PLIN2	28.6	10.8	14.9	1.2	3.3	5.0	19.7	2.5	15.0
ZNF579	25.9	9.8	11.9	3.5	4.9	6.5	20.7	3.7	13.9
TMEM255B	29.5	10.5	11.2	5.8	7.0	10.2	12.5	3.3	10.7
CDC25B	24.4	10.2	12.8	2.9	5.7	6.8	19.0	3.2	15.6
RAB11FIP5	27.2	10.1	12.7	3.2	4.8	6.5	18.2	4.1	13.9
SNX17	23.5	9.5	11.8	3.3	7.7	10.4	20.2	2.8	11.5
LOC100506248	23.1	10.1	12.1	2.0	5.3	5.3	19.2	4.7	19.0
MRPL9	27.2	9.1	14.3	2.2	4.7	7.3	21.0	2.6	12.2
MXD4	31.4	12.8	17.8	4.2	5.2	7.7	11.1	2.0	8.4
OAT	28.4	12.4	14.7	1.0	4.5	5.1	19.4	2.2	12.9
TGFB1I1	25.0	12.0	15.8	3.2	5.9	7.8	15.8	3.3	11.8
WLS	27.1	9.7	13.0	1.3	2.9	5.3	22.0	2.8	16.3
BLCAP	27.7	9.6	12.3	1.4	4.9	6.5	22.6	2.2	13.0
MRFAP1L1	25.0	11.2	12.6	2.1	5.9	6.3	20.3	2.7	14.2
SNORD94	27.5	7.0	12.7	1.6	5.5	2.4	32.9	1.1	9.7
CELF1	28.2	9.4	12.2	1.7	4.1	5.9	19.8	3.0	15.9
CDK10	28.4	11.6	16.0	2.9	5.8	7.9	15.1	2.5	10.1
PSME3	27.0	9.5	12.9	1.5	3.9	5.4	21.7	2.9	15.5
PRRC2C	25.8	8.0	11.5	2.0	4.8	7.1	17.7	3.6	19.7
PSMA3	25.8	9.3	13.6	1.1	3.7	4.7	22.9	2.4	16.7
PDXK	27.7	10.6	13.1	2.7	4.5	5.3	20.0	2.9	13.5
CRADD	26.6	8.7	12.8	1.4	4.9	4.4	23.3	2.8	15.0
LOC101928137	35.6	5.7	13.5	1.4	4.0	4.7	22.7	1.5	11.0
KCNAB2	28.4	11.6	15.8	3.0	5.6	8.6	13.7	2.5	10.9
CARS	30.2	11.4	13.6	1.7	4.4	6.7	16.3	2.7	13.1
KIAA2013	25.8	11.2	14.0	3.2	5.2	6.5	17.1	3.5	13.6
DNAJB11	22.7	10.2	11.3	1.0	3.9	5.0	23.8	3.0	18.9
TNPO2	25.7	9.8	13.4	2.9	5.3	7.9	17.7	3.0	14.1
ERAL1	28.1	11.0	15.0	1.7	4.8	5.0	16.5	2.4	15.2
PLOD3	26.6	10.2	13.8	3.5	5.8	7.4	17.2	2.7	12.7
FAM104A	24.5	9.8	13.5	4.0	5.8	7.5	15.7	4.8	14.2

EXOSC6	26.7	9.3	13.2	3.5	5.4	5.6	22.4	2.9	10.8
ARF5	27.5	12.0	15.3	2.3	6.0	7.8	15.4	2.0	11.4
PRCP	27.6	11.8	14.2	1.6	4.9	5.8	18.2	2.7	12.9
TMEM203	24.4	9.5	13.9	3.3	4.3	7.6	20.5	3.5	12.6
PAIP2	24.0	9.7	14.8	0.6	3.6	5.6	19.5	2.8	18.9
RILPL2	28.8	9.8	13.1	1.8	4.6	6.5	18.6	3.2	13.2
SH3GL1	23.4	9.1	13.4	2.9	4.9	6.9	19.3	3.9	15.8
ITPR3	25.6	8.7	12.2	3.0	5.4	7.3	17.8	3.3	16.0
CDCA7L	25.4	8.6	11.1	1.3	4.0	5.0	25.2	2.5	16.3
POLR1A	26.8	8.2	11.7	2.4	5.0	6.6	20.2	2.9	15.7
IGF1R	27.3	8.0	12.2	2.5	4.8	5.9	19.9	3.6	15.3
DAG1	29.1	12.6	15.6	2.7	5.2	7.1	12.1	3.1	12.0
INTS3	30.6	12.0	16.1	1.9	4.4	5.8	14.8	2.4	11.4
NUDT3	23.1	10.3	11.9	3.3	5.2	5.2	21.4	2.7	16.1
TCEB1	25.2	10.5	15.6	1.1	4.0	4.0	18.3	2.1	18.4
CNDP2	25.0	9.4	11.1	2.8	7.2	9.4	18.3	2.5	13.4
TSR3	26.3	9.3	12.9	3.9	6.7	7.2	19.4	2.8	10.6
TNKS1BP1	28.1	10.9	14.2	2.3	4.8	5.8	17.6	2.7	12.7
CIC	29.5	11.0	13.9	3.0	4.7	6.0	15.6	3.1	12.4
TXNDC5	22.4	10.1	10.6	4.7	6.0	6.3	18.5	3.3	17.2
YIPF2	26.9	9.4	12.8	2.9	5.3	7.1	18.6	2.6	13.3
MRPL14	21.7	10.3	14.4	1.7	5.8	6.5	19.5	3.0	16.0
DUT	27.5	11.0	16.1	2.9	5.3	7.2	16.4	1.8	10.6
FYN	25.3	8.6	11.4	2.0	4.8	5.6	21.6	3.4	16.1
BMP4	25.7	10.7	13.7	2.2	7.1	7.3	16.8	2.5	12.8
LOC100652901	33.6	5.6	14.8	2.1	6.1	5.7	20.2	1.6	9.0
EDEM1	33.0	10.3	14.6	1.4	3.1	4.7	17.7	1.9	11.9
CDC123	23.9	9.2	12.0	1.1	3.9	6.0	20.1	3.1	19.3
UBE2L3	30.3	13.0	15.1	0.7	3.2	8.0	8.1	1.8	18.4
PPT1	23.8	10.4	12.5	1.1	3.7	5.5	21.1	2.2	18.5
MRGBP	25.4	10.2	12.7	1.5	4.4	5.4	18.7	3.1	17.3
KRI1	21.9	7.9	10.2	3.0	6.0	8.8	21.0	3.5	16.4
LOC101928372	32.4	6.6	15.2	2.7	4.5	4.7	19.5	1.9	11.0
LPHN1	29.5	11.0	14.4	3.4	4.9	6.6	14.2	2.8	11.7
DCAF8	29.7	10.9	14.7	2.7	6.5	8.5	13.9	1.6	10.0
MYL6B	26.0	10.4	14.5	2.8	5.6	8.0	15.3	2.6	13.3
TWSG1	30.5	10.6	15.8	1.0	2.8	4.4	19.9	1.6	11.9
SLC25A23	28.7	11.9	14.7	2.8	5.8	7.5	14.4	2.1	10.5
DVL2	25.7	11.8	15.0	3.2	5.8	8.4	12.8	3.0	12.6
EIF3CL	25.8	9.1	11.4	2.9	5.9	7.9	17.7	2.9	14.6
SPPL3	28.0	10.6	13.1	1.2	3.9	5.4	18.1	3.0	14.9
STK11	27.0	10.4	12.1	3.1	5.3	7.4	17.6	2.6	12.6
TNFAIP2	36.0	15.1	19.6	2.5	4.1	4.9	7.9	1.7	6.6
RBM34	29.9	7.8	13.4	1.7	3.5	4.8	22.0	2.1	13.0
SRP72	24.1	8.9	13.5	1.4	4.3	6.1	17.4	3.5	19.0
HBEGF	19.9	7.9	11.7	1.5	4.9	6.3	24.9	3.5	17.5
GTPBP3	24.4	9.9	12.3	3.6	5.3	6.8	19.6	3.1	13.2
DNTTIP1	26.8	11.5	12.9	2.2	5.2	7.1	15.2	3.0	14.2
BCLAF1	27.6	8.8	12.5	0.7	3.3	5.4	16.5	2.1	21.2
EMC4	26.9	11.2	15.0	1.4	5.2	5.7	17.7	2.3	12.5
AURKAIP1	26.9	9.0	13.7	2.4	5.3	6.3	18.5	3.0	12.8
TIGD1	33.6	7.5	14.4	0.9	4.0	4.6	19.4	1.5	11.9
LINC00493	22.7	11.0	14.5	1.9	4.7	6.6	20.3	2.7	13.4
CUEDC2	26.3	10.6	14.7	2.4	4.7	7.1	17.5	2.1	12.3
BYSL	22.8	9.0	11.1	2.2	4.9	5.2	21.0	3.6	17.7

TNFRSF21	27.7	10.2	13.2	3.6	7.4	10.1	12.6	2.1	10.8
TMED1	27.5	11.8	14.5	2.2	4.8	5.8	16.7	2.3	12.0
UBXN6	26.7	9.9	12.9	3.5	5.6	9.3	14.6	3.0	12.0
NMT1	25.9	10.4	12.6	2.8	4.7	7.1	16.1	3.4	14.4
SRP9	30.4	9.9	15.2	0.6	3.3	5.3	17.4	2.8	12.6
SEC61B	25.4	10.6	16.5	1.5	3.2	4.6	16.9	2.9	15.7
LGALS3	31.1	15.2	19.8	1.4	4.1	5.7	9.9	1.6	8.5
DDX24	28.4	10.0	12.7	1.5	4.2	6.3	17.2	2.7	14.3
SPG21	28.5	11.3	13.4	0.6	3.9	6.2	17.9	2.2	13.3
CNPY2	25.8	10.2	13.7	1.6	4.7	6.6	17.8	2.9	14.0
PSMD11	23.9	10.0	12.3	1.6	4.2	5.4	20.5	3.1	16.1
HIPK2	26.4	8.5	11.9	6.5	8.5	12.2	8.5	2.7	11.9
ORC6	29.9	6.9	13.0	1.6	3.7	4.5	23.6	2.4	11.6
SNRPA	22.8	9.8	13.3	2.9	5.8	8.1	16.1	3.6	14.6
EHBP1L1	30.3	10.9	14.8	3.1	5.2	7.6	13.1	2.1	9.8
FAM127B	28.4	10.2	14.3	4.0	7.1	7.9	12.7	2.6	9.8
WDR26	25.8	9.5	13.5	2.6	5.0	6.4	16.4	3.4	14.2
COPS6	22.8	9.6	12.4	2.9	7.2	8.0	17.7	3.2	12.8
NXN	29.9	12.4	14.0	3.2	5.9	8.1	11.4	2.2	9.6
TRAPPC2L	25.6	11.0	16.0	2.4	4.9	5.4	15.9	2.6	13.0
PLXND1	33.3	13.2	17.4	3.2	5.0	6.8	8.3	2.0	7.5
B4GALT3	25.1	10.4	14.7	1.7	4.2	5.7	16.8	2.8	15.3
CSNK2A2	24.3	7.9	10.1	1.6	2.7	5.0	24.8	3.2	17.2
CAMLG	25.9	9.8	14.1	1.6	3.7	5.1	18.5	2.8	15.3
WAC	26.7	9.2	13.0	1.7	3.9	4.6	18.4	3.1	16.1
SCAMP4	27.6	9.8	15.3	1.8	4.0	5.5	17.0	2.7	12.7
HMGB3	37.8	14.7	16.8	0.9	5.6	5.9	7.6	1.1	6.0
LAMB2	28.0	11.5	15.2	2.8	5.8	7.8	13.6	2.2	9.7
THYN1	27.5	11.4	14.8	1.6	5.9	6.5	14.3	2.2	12.2
MRPL3	25.3	10.4	14.3	0.9	4.0	5.1	19.9	2.2	14.2
NUBP2	26.4	11.2	14.0	3.1	4.6	7.6	16.2	2.0	11.3
CBX1	26.5	8.5	10.9	0.8	3.9	4.5	21.6	2.5	16.9
OSCAR	24.6	10.4	12.7	3.6	5.6	6.3	17.3	3.3	12.5
FAM3C	22.8	10.1	12.7	0.6	4.0	5.4	21.4	2.4	16.6
LOC100144595	37.0	6.7	14.8	2.1	3.1	3.1	21.1	1.8	6.5
PTDSS1	21.6	8.2	10.9	1.5	4.3	5.9	22.7	2.6	18.2
MRPL18	27.2	10.2	16.5	0.9	3.8	5.3	17.8	2.2	12.1
POM121C	25.6	10.1	12.0	3.3	5.4	7.3	14.0	3.7	14.5
MEF2D	25.4	9.6	12.7	3.4	5.3	6.9	13.9	3.9	14.7
PSEN1	26.5	9.3	12.8	1.1	3.5	4.1	19.9	2.6	16.1
TFAP4	20.1	9.0	10.7	4.3	7.2	8.4	16.0	3.9	16.3
CPD	39.4	13.3	17.9	1.1	3.0	4.0	9.2	1.2	6.6
ILVBL	27.5	9.7	13.4	2.3	4.5	5.8	19.4	2.0	11.1
ADD1	29.4	11.6	14.3	1.8	4.4	6.6	14.0	2.3	11.4
ZFP36	25.0	11.8	16.2	4.1	4.7	7.7	13.0	3.1	10.2
SNX12	28.0	8.4	12.9	2.5	4.7	5.8	18.6	2.8	12.0
YLPM1	25.4	8.0	12.0	2.0	3.9	5.6	18.6	3.4	16.7
KPNA2	23.5	8.7	11.4	1.4	4.6	5.8	20.8	3.1	16.1
CC2D1A	27.8	11.7	14.0	3.3	5.6	7.5	13.4	2.3	9.9
TFDP1	23.7	8.3	12.1	1.7	4.2	5.5	20.5	3.0	16.5
DCAF5	29.5	9.7	11.2	2.2	4.3	5.8	16.7	3.1	13.1
KDSR	27.2	9.0	12.0	1.3	4.6	6.5	18.7	2.2	13.8
CHMP5	26.0	9.5	15.1	0.7	2.9	4.8	19.6	1.9	14.9
LPCAT4	24.6	10.4	13.5	2.4	4.8	6.9	16.2	2.9	13.8
SLC35B2	24.8	10.5	13.9	2.4	4.6	7.1	15.0	2.5	14.6

OGDH	24.9	11.4	15.3	2.2	5.3	5.6	18.4	2.1	10.1
TMEM8A	26.0	10.8	12.8	3.7	5.9	8.5	13.2	3.1	11.3
CS	26.8	10.3	11.6	1.3	4.4	4.9	18.2	2.4	15.3
C2CD2L	19.5	7.9	9.7	2.0	3.9	5.9	22.3	5.0	19.1
BECN1	25.5	11.3	12.6	1.7	4.0	6.1	16.5	2.8	14.8
LYRM4	29.1	7.1	11.7	1.4	4.4	4.7	20.2	3.3	13.3
GRPEL1	21.2	9.0	11.5	1.2	4.7	5.0	20.6	3.5	18.3
RCC2	24.4	7.9	11.5	2.1	4.2	4.5	23.8	2.9	13.7
KDM2A	27.2	9.1	12.8	1.7	3.8	4.9	18.3	2.9	14.3
COX17	24.0	9.8	13.9	1.9	5.4	5.5	16.2	3.5	14.7
PTPN1	26.7	9.9	12.2	2.3	5.7	6.9	14.5	2.9	13.8
MINPP1	29.9	12.1	16.5	3.2	4.6	6.3	11.3	2.1	9.0
ZNF511	27.0	9.9	14.2	2.3	4.6	5.8	16.0	2.7	12.5
MAGEF1	26.7	9.8	11.8	2.2	5.2	6.9	17.2	3.0	12.0
PNN	25.5	9.4	13.4	1.3	4.4	5.6	17.9	2.7	14.7
PDCD5	25.7	10.7	14.1	2.9	4.9	7.3	12.2	3.5	13.6
PTPMT1	22.6	8.9	12.9	3.3	5.2	5.8	17.7	3.4	15.0
CDC42BPB	28.1	8.8	11.9	2.3	4.6	6.3	17.8	2.5	12.2
NT5C2	26.7	10.0	13.9	1.2	3.9	5.1	17.3	2.9	13.7
PARL	27.9	11.1	14.4	1.5	4.1	4.7	16.6	2.6	11.6
LOC101927011	32.0	6.4	14.2	1.0	4.1	4.6	20.3	1.2	10.9
CST4	39.8	22.8	28.8	0.4	1.2	0.9	0.4	0.0	0.4
TNIP2	24.5	9.3	12.9	2.6	4.3	5.2	19.7	2.5	13.7
CBX4	26.6	10.1	14.1	3.3	3.8	4.8	15.0	3.9	12.9
SLC39A9	25.9	9.7	12.3	1.5	3.7	5.6	18.1	2.4	15.4
SUCLG1	26.4	10.7	15.7	1.8	4.7	6.2	14.4	2.1	12.4
TMEM59	27.5	10.1	16.2	1.2	4.5	5.1	17.6	2.2	10.3
CNOT1	27.2	9.2	11.2	1.2	3.7	5.6	18.0	2.5	15.9
HEBP1	29.2	11.6	14.0	2.1	5.8	7.1	13.6	2.0	9.0
WFS1	25.2	9.7	11.9	6.1	8.4	11.5	10.6	2.1	8.7
PHF1	26.0	11.0	15.2	2.3	4.9	6.8	14.3	2.5	11.4
RBM14	29.3	8.0	10.7	3.3	6.6	7.8	11.4	4.0	13.3
C12orf49	24.6	9.7	13.9	2.4	4.8	6.4	16.9	2.6	13.0
NRBP1	24.0	10.4	13.5	1.5	3.9	6.0	17.0	2.8	15.2
UBQLN1	22.3	7.9	10.6	1.4	4.3	6.5	19.3	3.4	18.7
CLK3	26.8	9.6	13.4	2.0	3.3	5.4	18.1	2.9	12.7
PSMA4	26.6	11.1	16.3	0.6	3.6	5.1	15.0	1.8	14.1
PHLDB1	27.3	11.5	11.3	2.3	4.3	5.3	19.4	2.2	10.6
WDR83	28.0	10.2	14.9	2.0	3.6	5.0	17.8	2.4	10.4
POGK	19.0	7.4	9.5	1.1	3.0	4.3	28.2	2.9	18.8
METTL9	25.9	9.3	13.1	2.5	4.5	5.7	18.8	2.8	11.5
LDB1	26.2	10.6	13.6	2.9	4.4	6.5	13.4	3.0	13.4
DAPK3	24.0	9.7	11.5	3.2	5.5	6.9	17.3	3.4	12.5
RAB11A	25.7	9.6	13.2	1.3	4.2	6.1	15.4	2.0	16.5
GLRX3	24.1	9.1	11.9	0.9	3.3	4.5	22.6	2.4	15.0
HN1L	24.2	8.1	12.1	1.9	4.2	6.4	19.6	2.7	14.6
G3BP2	25.2	9.5	11.3	1.1	3.3	4.2	21.2	2.4	15.5
CD8B	33.1	6.0	12.8	2.3	3.8	4.2	21.2	1.8	8.4
KCMF1	22.2	9.3	11.7	1.6	3.7	5.3	18.6	3.9	17.2
SETD8	21.8	9.4	12.1	1.9	3.7	6.7	16.9	3.2	17.8
SCAP	24.1	8.9	11.7	2.8	5.0	6.8	18.9	2.9	12.4
FGFR1	22.9	8.2	10.6	3.3	6.5	8.3	17.4	3.3	13.2
BTBD1	24.3	8.7	11.9	2.8	3.9	6.5	17.0	3.0	15.3
COA4	23.3	9.4	12.6	3.7	7.1	9.6	14.4	2.3	11.2
MTHFD2	19.6	7.3	11.1	1.2	2.9	5.3	23.5	3.0	19.6

PAIP1	24.7	9.4	11.9	1.5	3.7	4.4	19.4	2.9	15.7
CCNK	26.0	9.8	11.8	2.5	5.0	5.9	15.4	3.4	13.7
OTUB1	25.6	10.4	14.4	2.8	5.6	6.7	12.6	2.6	12.6
BCAT2	24.2	10.2	11.4	2.3	4.2	6.6	18.0	3.1	13.5
GOLIM4	24.7	10.0	12.4	1.7	4.8	5.7	16.0	2.8	15.2
DHX15	22.5	8.8	11.0	0.9	2.9	4.4	21.0	3.0	18.7
SNHG17	21.5	8.3	10.9	1.9	3.6	4.5	22.4	3.5	16.7
PML	29.1	9.7	12.8	2.9	4.4	5.9	14.8	2.8	10.7
NR2F6	27.3	9.8	13.2	3.4	3.3	4.8	18.1	2.6	10.5
OBSL1	30.9	10.2	14.4	3.0	4.9	6.6	13.2	2.0	7.9
PC	24.6	9.6	13.3	2.8	5.1	6.5	16.5	2.6	12.2
PDE4A	29.2	10.9	14.0	2.7	4.8	6.8	11.7	2.4	10.6
ABCF3	26.7	9.7	12.8	1.9	4.1	6.7	17.2	2.5	11.4
RIC8A	20.9	8.1	10.5	2.3	5.0	6.2	22.7	2.7	14.6
SCRN1	24.8	8.7	11.7	1.7	3.8	5.9	19.0	2.6	14.7
EMC8	24.3	10.4	11.9	2.8	4.1	6.0	17.4	3.5	12.3
HYI	16.2	7.4	8.8	3.9	7.9	10.4	20.8	3.1	14.2
HDAC3	23.4	10.9	13.1	1.9	4.1	4.8	16.9	2.9	15.0
NFKB2	29.4	11.3	15.0	2.6	4.4	5.6	13.4	2.1	9.0
M6PR	25.7	9.9	14.4	1.0	3.7	4.2	16.8	2.1	15.0
DIABLO	25.0	10.5	14.6	1.2	3.8	6.3	15.7	2.4	13.1
MAFK	21.9	7.3	11.2	2.5	3.8	5.2	21.8	4.1	14.7
LOC101929455	23.5	7.9	12.1	1.7	4.9	5.3	19.3	2.5	15.3
RCN2	22.1	8.6	10.7	2.0	4.0	5.7	20.3	2.8	16.2
RHOG	23.2	8.7	11.5	2.2	4.6	5.6	16.8	4.3	15.6
DOK1	22.3	8.5	12.0	2.7	4.6	6.8	20.9	2.5	12.1
LARP4B	24.1	8.1	11.8	1.6	3.5	4.9	20.8	2.9	14.7
EPB41L4A-AS1	22.0	8.7	11.6	1.3	3.2	4.3	23.7	3.2	14.4
TMCO1	24.9	10.2	12.7	1.1	3.0	4.5	19.9	2.5	13.5
UBTD1	24.8	8.8	12.8	3.3	4.5	7.2	15.4	3.4	12.1
HDAC1	25.3	9.6	12.8	1.0	3.5	5.1	18.6	2.4	13.9
NDUFV2	26.1	10.8	16.1	0.8	3.7	5.4	15.0	1.9	12.4
LOC643406	31.7	6.0	13.6	1.4	4.3	5.3	17.3	1.5	10.9
PTRH2	21.1	8.9	12.1	0.8	3.2	4.5	21.1	2.7	17.7
NOP2	24.5	9.6	12.9	0.9	2.4	3.5	19.3	3.2	15.7
CRTC2	23.1	8.9	11.9	2.4	3.9	5.6	16.4	4.6	15.2
AFF4	24.4	7.6	10.5	1.3	4.3	4.9	18.7	3.3	17.1
EXOC7	22.6	8.6	10.6	2.8	6.0	7.8	16.7	2.7	14.3
MRPL45	22.0	8.7	11.7	1.1	3.9	5.5	21.7	2.7	14.7
PTCD3	25.1	9.8	13.0	0.8	3.8	5.3	17.5	2.4	14.2
RNU11	21.6	16.5	17.6	2.5	4.4	4.7	11.2	4.1	9.4
KPNA4	20.6	8.2	10.7	0.6	3.7	5.3	20.8	2.9	19.0
DNAJC7	26.0	9.4	10.9	1.2	3.5	4.7	19.5	2.2	14.3
CALCOCO2	24.9	11.0	12.3	0.9	3.8	4.4	15.8	2.7	15.9
FTSJ1	20.7	8.9	11.1	2.3	5.7	7.2	18.6	3.0	14.1
RBBP4	24.4	8.1	11.7	0.9	3.7	4.0	21.1	1.9	15.9
UBE2Q1	25.8	9.4	11.8	2.2	4.3	6.6	16.2	2.7	12.7
SRCAP	26.5	8.4	12.3	2.2	4.2	5.8	15.6	2.9	13.7
ECI1	22.6	9.6	12.2	3.0	5.4	6.4	17.3	2.2	12.9
LPXN	20.4	8.9	10.3	2.4	6.7	9.7	17.7	2.3	13.1
POLR2G	22.9	9.6	12.6	1.1	4.2	5.2	19.9	2.5	13.6
RAB6A	27.5	8.4	11.1	1.2	4.7	4.9	16.9	2.6	14.3
C2orf68	21.6	8.4	10.5	3.9	6.9	9.1	15.3	2.8	13.0
ATIC	22.7	9.2	10.1	1.6	4.5	6.9	18.9	2.6	15.0
CRKL	21.0	7.2	10.2	1.7	4.0	4.8	21.2	3.8	17.5

SBNO1	23.2	7.6	10.5	0.9	3.2	4.1	22.2	2.4	17.3
GRB10	25.6	8.5	11.9	1.3	2.8	4.0	19.4	3.1	14.7
SNX22	23.0	7.8	11.4	2.0	3.8	5.5	21.5	2.7	13.7
PACS2	29.0	9.5	13.5	3.5	5.5	7.3	11.6	2.5	9.0
ISOC2	20.0	9.7	13.3	2.8	6.5	9.1	15.5	2.4	11.9
KCTD20	24.1	8.1	10.2	0.9	3.0	3.4	22.8	2.4	16.3
NFE2L2	26.8	9.1	11.3	0.8	3.4	5.0	16.9	2.5	15.5
SRRM1	24.0	9.9	12.6	2.3	4.9	6.9	13.1	3.0	14.6
CKLF	25.8	10.5	15.3	1.3	3.6	5.5	15.3	2.5	11.5
RPL26L1	23.6	9.6	16.1	2.7	5.1	8.1	11.0	2.6	12.5
NLGN2	27.5	10.6	14.2	4.1	6.1	7.3	10.9	2.1	8.4
GSE1	26.4	9.5	13.4	3.6	5.3	6.7	13.8	2.4	10.0
FXR2	22.0	8.8	10.6	1.5	4.0	5.4	19.8	3.6	15.3
BCAT1	21.5	7.8	10.4	0.7	3.3	4.5	24.3	2.0	16.5
SCARNA22	23.0	10.9	17.5	2.0	3.2	5.6	15.7	2.1	10.7
MTHFD1L	19.7	7.1	8.9	1.6	3.4	4.8	27.5	2.7	15.0
C5orf15	24.2	10.6	14.1	2.8	5.9	7.5	14.1	2.4	9.3
RNF130	29.3	10.4	13.3	1.5	4.1	5.1	15.2	2.1	9.7
MTPN	26.0	8.7	12.8	0.9	3.5	5.8	16.3	3.0	13.8
SNHG4	29.2	7.2	11.9	1.7	3.5	4.7	20.1	2.0	10.4
SNORD89	23.3	13.9	12.7	2.5	6.5	5.1	13.1	2.6	11.2
SNORA34	25.8	10.1	12.6	2.2	2.9	3.0	24.3	1.4	8.6
MED25	25.3	10.1	12.3	3.1	4.8	7.3	13.4	3.0	11.4
PELP1	22.1	8.1	11.0	2.6	4.7	6.1	18.6	3.1	14.4
SPCS3	19.5	7.9	9.7	3.6	4.2	8.5	20.5	2.9	13.9
NT5C3B	22.2	8.8	11.7	2.0	4.1	5.2	18.7	2.9	15.0
ITGB1BP1	25.1	9.7	15.8	0.6	2.8	4.8	15.4	1.9	14.3
MGAT2	17.0	7.8	8.9	1.9	3.5	4.7	28.0	3.4	15.3
RNF40	25.6	8.9	11.6	2.3	4.0	6.0	17.1	2.9	12.1
UQCRC1	24.3	9.5	13.7	2.9	5.2	7.5	14.4	2.2	10.8
RAB13	22.1	9.1	12.3	2.0	5.9	7.8	14.5	2.0	14.7
AASDHPPT	27.0	9.2	12.7	1.3	3.4	4.7	18.9	2.1	11.0
TSR1	20.3	8.3	9.8	1.6	3.8	5.7	21.2	2.9	16.8
RNF220	23.6	8.1	12.0	1.8	3.4	5.5	17.1	3.1	15.6
POLE4	26.1	8.9	14.0	3.0	4.3	5.6	18.3	2.0	8.1
C11orf83	27.2	11.9	14.2	1.8	3.8	5.1	14.1	1.9	10.4
VGLL4	27.1	10.3	13.5	4.6	4.9	7.1	9.9	2.8	9.9
CD46	25.6	10.1	13.9	0.8	2.7	4.3	18.0	2.0	12.9
SON	25.9	8.4	10.5	1.1	3.8	4.4	19.8	2.1	14.2
AKIP1	28.1	6.9	13.0	1.8	3.6	4.7	20.5	1.8	9.9
FAM192A	24.5	9.5	13.9	2.1	5.6	7.4	12.1	2.6	12.6
ETHE1	26.9	10.2	14.7	1.4	4.3	6.4	13.6	1.9	10.6
SLC7A6	22.9	8.8	11.8	1.3	3.8	4.5	18.8	2.8	15.4
DGUOK	25.3	10.3	14.8	1.6	4.1	4.9	14.9	1.9	12.1
AFAP1	22.7	8.8	10.7	2.2	5.3	6.8	17.8	2.4	13.4
DPF2	21.0	7.8	11.1	1.3	3.3	4.6	22.4	3.2	15.3
TCEA2	28.1	11.4	15.4	4.7	7.5	9.6	7.1	1.4	4.7
ANAPC13	21.9	11.0	14.2	1.4	4.9	6.1	16.8	1.9	11.9
CCNL1	24.9	8.1	14.2	1.9	3.3	4.2	14.6	3.1	15.6
LOC101929567	30.0	5.8	13.2	1.0	4.3	4.4	18.9	1.6	10.8
HNRNPH2	25.6	9.7	11.8	0.8	3.6	4.8	18.8	2.3	12.5
LOC101929616	21.9	7.1	12.7	4.5	8.7	11.2	11.9	2.0	9.8
SREK1	23.9	8.5	11.6	1.2	2.6	4.5	19.1	2.6	15.9
UNC13D	20.5	8.3	10.0	3.6	6.6	8.5	16.5	2.8	12.9
ARAP1	23.5	8.6	11.2	3.3	5.5	7.4	16.8	2.5	11.0

LOC100268168	28.2	6.0	12.7	1.3	3.4	4.3	22.3	1.8	9.7
ACTR3	21.3	8.6	11.7	1.0	3.4	4.6	20.2	2.6	16.3
TMEM55B	23.2	10.4	13.4	1.7	3.0	5.2	16.5	3.1	13.2
CAPZB	25.5	9.4	12.1	1.4	3.7	5.8	16.0	2.4	13.2
SETD7	19.7	7.4	8.4	1.3	3.6	4.8	21.9	3.6	18.8
C17orf62	27.9	9.2	13.2	2.2	4.9	5.4	15.3	2.2	9.1
EFR3A	26.7	9.4	12.9	0.8	3.1	4.2	16.1	1.7	14.3
WIPI2	24.8	8.8	12.1	2.4	4.4	6.1	15.7	3.3	11.6
LOC101929698	29.9	5.5	12.3	2.0	2.9	4.1	21.3	1.8	9.4
SEC61G	19.4	10.5	12.4	0.3	2.4	2.8	24.4	1.9	15.2
SBNO2	25.6	9.9	13.7	3.1	5.2	6.8	12.6	2.5	9.8
NPLOC4	24.1	9.6	11.5	2.0	4.8	5.9	15.2	2.9	13.1
NDFIP1	24.6	11.4	14.0	1.4	3.5	5.4	14.0	2.2	12.6
LOC101927740	27.9	5.7	12.6	1.4	3.5	4.6	21.4	1.8	10.3
NUDT22	25.4	9.8	14.8	3.0	5.5	7.4	12.0	2.3	8.9
HIST2H2AB	18.4	5.7	10.1	3.1	6.8	9.0	21.3	2.9	11.7
ZBTB7A	25.9	9.3	12.3	4.0	4.7	6.8	12.7	2.8	10.5
SEH1L	22.1	7.3	11.0	1.0	3.0	4.2	20.5	3.3	16.6
C6orf120	22.5	9.2	14.5	3.4	6.1	7.1	14.6	2.5	8.9
PEF1	26.6	10.3	13.7	1.8	3.4	6.9	13.3	1.7	11.1
CLCN7	24.2	9.4	12.5	3.2	5.9	8.0	13.3	2.3	10.2
NDUFAB1	20.6	8.8	14.2	1.5	4.3	5.8	16.6	2.9	14.1
ABHD4	29.8	9.6	12.4	2.3	4.2	6.5	12.3	2.2	9.4
COX7A2	21.7	8.5	13.0	1.1	3.4	5.4	20.5	2.4	12.5
NKIRAS2	20.3	9.8	10.7	2.1	4.1	5.9	18.4	3.2	14.2
POGZ	28.2	7.6	12.3	1.5	3.9	4.6	15.7	1.8	13.0
TTC9C	25.2	7.5	14.2	1.8	3.4	4.6	17.9	2.3	11.9
ATP13A3	22.1	7.8	9.7	0.7	3.4	4.6	21.3	2.3	16.8
MAGED2	23.6	10.1	12.5	1.3	4.1	5.1	17.5	2.3	12.1
SIX1	19.3	8.2	10.3	3.3	6.1	7.7	16.4	3.3	13.9
KLF4	20.9	8.4	10.6	2.2	3.6	5.3	18.2	4.1	15.1
ATXN10	24.9	8.9	11.9	1.2	3.7	5.3	18.2	2.2	12.3
TNFSF15	26.7	10.1	15.1	0.5	0.9	1.6	17.1	2.8	13.5
EREG	26.2	10.3	14.7	0.9	3.6	4.9	12.3	1.9	13.4
SUPT16H	21.3	7.5	9.5	1.0	3.6	4.8	21.2	2.7	16.9
FLCN	28.9	10.6	15.1	2.2	4.5	5.7	11.9	1.8	7.8
VIPR1	16.4	6.4	8.0	6.8	13.3	22.8	8.3	1.1	5.1
ALDH2	24.5	9.8	12.9	2.7	4.9	7.6	13.2	2.1	10.6
ZNF768	18.3	8.0	8.9	3.0	4.1	5.6	18.9	4.2	17.3
PARP6	24.0	9.0	11.8	1.3	3.5	4.9	17.3	2.7	13.7
TRNS1	35.7	5.3	11.2	1.2	1.6	3.7	15.9	1.9	11.9
THOC3	25.2	8.4	11.0	1.5	4.3	6.0	17.7	2.2	11.8
APOA1BP	20.9	8.3	12.2	2.1	4.3	5.1	18.6	2.5	14.2
UFC1	22.2	9.7	15.3	1.2	3.1	4.3	17.9	2.3	12.3
KHDRBS3	20.4	8.0	10.8	2.0	3.3	5.3	19.6	3.4	15.4
UBE2C	17.7	7.8	9.7	2.5	8.1	10.0	16.6	4.2	11.6
TXNL4A	19.2	7.1	10.6	2.5	4.6	6.0	19.2	2.6	16.4
DFFA	16.3	6.8	8.4	2.0	5.6	6.0	21.4	3.3	18.3
BBX	24.6	7.4	10.9	1.4	3.7	6.3	17.7	2.6	13.4
RNF11	22.1	9.6	14.8	1.3	4.5	5.2	14.3	3.2	13.1
YKT6	25.0	7.2	10.7	1.0	2.0	3.9	18.7	3.5	16.0
SOCS3	18.8	9.9	11.8	3.7	5.4	7.1	14.7	2.9	13.8
LOC101929147	31.0	6.7	13.2	2.0	4.7	5.2	14.3	1.8	9.1
SRSF10	24.7	7.9	11.2	1.2	3.1	5.5	16.5	2.4	15.4
EDC4	26.2	10.4	13.6	2.1	4.0	4.7	13.4	2.3	11.3

C16orf80	19.8	9.7	10.8	2.1	4.2	6.9	16.4	3.2	14.8
DEDD2	25.4	10.6	12.3	2.6	4.2	5.3	12.5	3.0	12.0
PLS3	21.5	7.8	9.9	0.6	3.8	5.0	22.1	2.1	15.1
PMP22	23.3	9.5	12.3	2.6	6.7	8.3	11.8	2.5	11.0
PLA2G16	25.3	9.5	13.0	1.9	6.2	6.3	14.3	2.1	9.3
PPRC1	20.3	7.0	9.2	1.9	3.6	5.4	20.3	3.6	16.5
LOC101929224	28.2	5.2	12.4	1.9	3.1	3.8	21.5	1.6	10.0
EIF3J	19.1	7.1	8.5	1.4	3.0	4.0	24.2	2.8	17.7
SNORD46	18.4	12.7	13.2	2.9	8.5	4.5	13.2	0.3	14.1
TMEM179B	17.9	8.0	11.0	2.8	4.6	7.1	20.5	3.7	12.1
MCMBP	22.1	8.7	11.2	1.1	3.5	4.8	19.5	2.5	14.3
MIR4435-1HG	27.7	10.6	15.5	1.5	4.3	6.0	11.3	1.1	9.6
CYB561	24.6	11.1	13.2	2.6	3.4	5.2	14.8	2.4	10.2
B4GALT5	27.3	8.5	12.2	1.5	3.8	5.0	15.5	2.3	11.6
ZNF622	20.2	6.5	9.7	1.6	2.7	4.0	23.5	3.0	16.2
EIF2B5	24.7	10.5	12.9	1.5	3.7	5.5	14.2	2.0	12.5
UBE2K	21.5	9.3	11.1	0.5	2.8	3.8	17.2	2.7	18.6
TUBA1A	28.1	12.1	15.7	1.4	3.8	5.1	9.8	2.0	9.4
CDT1	15.5	5.4	7.6	3.6	5.5	7.2	20.8	5.0	16.7
DCTN2	22.8	10.3	12.1	2.1	4.3	6.6	14.9	2.4	11.8
TWF2	21.0	8.5	10.3	2.5	4.7	6.5	17.4	2.9	13.2
TCEAL8	25.6	9.6	13.4	1.1	3.5	4.8	15.4	1.7	12.1
ZBTB12	18.5	6.9	9.3	2.6	4.4	6.3	18.5	4.1	16.5
HUWE1	23.3	6.4	9.6	1.9	4.6	5.7	18.6	2.7	14.3
LRPPRC	22.7	7.5	10.3	1.4	3.1	4.2	21.6	2.3	14.0
FMNL2	27.6	9.7	12.8	1.8	3.8	4.8	13.6	2.2	10.7
GPR137	25.6	10.5	11.6	2.9	5.4	8.2	12.4	1.9	8.5
TBL1XR1	23.8	7.9	11.4	0.5	2.7	3.5	17.8	2.6	16.8
JAG1	24.6	8.5	11.1	2.2	4.8	6.5	15.2	2.1	11.9
DCTPP1	20.7	7.8	11.8	2.3	3.6	5.5	15.9	3.2	16.2
COPRS	23.6	11.4	12.4	1.8	3.4	5.3	15.2	2.6	11.3
ST6GALNAC6	27.4	9.6	13.2	2.0	4.1	5.3	11.7	2.7	10.8
ARPC1A	23.1	8.1	12.6	1.8	4.2	6.2	15.9	2.5	12.4
UBE2E3	25.8	11.0	11.9	1.1	3.1	5.2	13.4	1.7	13.6
METRN	20.1	7.9	9.5	6.2	7.7	11.5	12.6	2.4	8.9
MLX	22.6	10.4	11.8	1.2	3.7	5.5	14.7	2.6	14.2
BRMS1	21.3	8.5	10.4	2.1	3.6	5.0	17.7	3.2	15.0
SELT	21.7	8.9	15.2	1.3	4.0	5.1	14.9	2.5	13.1
CASC4	25.6	9.7	12.5	1.3	3.3	4.7	15.1	2.2	12.3
PRMT2	22.8	8.2	11.3	2.1	3.7	5.6	18.6	2.4	12.0
OPN3	22.4	8.8	11.4	2.7	3.6	5.6	17.8	2.9	11.4
LETM1	22.2	7.5	10.8	2.2	4.1	4.9	18.8	2.7	13.5
PHF23	19.7	8.4	10.2	2.3	4.4	5.5	17.0	3.0	16.2
SNX5	23.0	10.0	12.0	1.2	3.5	5.3	16.7	2.1	12.7
MYO19	19.1	7.4	10.7	1.6	4.8	6.4	18.0	2.3	16.3
CYP51A1	27.7	9.2	12.7	0.5	1.8	2.3	20.1	1.4	10.9
STK24	23.8	8.8	11.0	1.1	3.3	4.4	16.6	3.0	14.5
TRABD	22.4	8.3	11.8	3.6	6.0	7.1	14.1	2.7	10.5
NT5DC2	20.5	8.6	11.1	3.3	4.9	6.6	15.5	3.2	12.7
AXIN1	25.5	9.1	9.5	1.9	3.2	4.0	17.0	2.4	13.8
DRG2	21.8	9.0	11.5	1.9	4.2	6.6	17.5	2.7	11.0
MEN1	23.7	9.0	12.3	2.5	4.3	6.3	14.6	2.2	11.4
SLC29A1	23.7	9.9	12.6	1.5	3.5	4.6	12.6	3.3	14.6
TMEM219	23.6	9.9	14.0	2.6	4.8	6.8	13.6	2.0	8.7
GPT2	22.5	7.6	9.6	2.2	4.4	5.2	18.1	2.7	13.9

TM9SF2	26.0	9.1	11.2	0.8	2.8	3.8	17.2	2.2	13.2
CACYBP	22.9	9.2	10.8	0.7	3.0	5.5	17.1	1.9	14.7
ELOVL1	22.8	10.1	11.6	1.6	4.4	6.6	13.6	2.3	13.1
GOLPH3	23.5	8.3	11.4	2.7	4.3	5.9	16.3	2.6	10.9
MGST1	23.7	11.1	12.8	1.2	4.6	6.3	10.1	1.9	14.3
NCOA4	21.6	9.6	11.4	1.0	3.5	5.2	17.5	2.3	13.7
CCDC92	28.2	10.1	13.1	2.6	4.3	6.0	11.2	1.9	8.5
AKAP1	19.5	7.9	10.1	1.9	3.6	5.4	17.6	3.4	16.6
ZC3H18	22.9	8.0	10.5	2.6	3.7	5.5	16.1	3.1	13.5
ZNHIT3	21.4	8.8	13.5	1.5	3.5	4.3	17.3	2.1	13.6
SH3BP4	19.0	7.8	9.4	2.3	4.7	6.8	17.3	3.0	15.6
CCDC85C	17.5	6.9	8.9	5.1	4.0	5.9	20.3	4.3	13.0
ARHGDIB	22.2	9.7	13.0	1.0	3.7	4.5	15.1	3.2	13.4
CEP250	23.9	8.1	11.4	2.4	4.9	5.7	15.6	2.4	11.3
NXF1	23.8	7.2	12.2	0.8	2.7	5.0	16.7	2.6	14.6
FOS	23.3	12.3	19.4	2.4	4.1	6.4	9.5	1.7	6.6
GBA	23.6	8.3	10.7	3.3	6.9	9.0	11.1	2.5	10.5
C6orf106	24.4	8.5	11.7	1.3	2.6	4.3	15.5	3.1	14.3
PGRMC1	19.8	9.2	10.6	2.1	5.3	4.7	15.3	3.5	15.2
TMEM14C	27.4	6.9	13.3	1.1	3.9	4.9	17.8	1.8	8.7
TRAM2	20.3	8.2	10.0	1.4	4.4	5.8	16.9	3.3	15.4
EIF2A	20.8	8.4	12.1	0.7	3.2	4.3	17.3	2.5	16.3
LOC90834	28.1	5.5	12.4	1.6	3.2	4.0	20.4	1.9	8.6
MED15	20.6	8.1	10.0	4.0	5.6	8.3	12.5	3.6	12.7
PSMA1	23.9	10.8	14.5	1.2	4.1	5.9	12.1	2.2	10.7
DCTN4	22.5	8.8	12.9	1.2	3.6	4.9	15.7	2.8	13.0
TTC17	22.6	7.3	11.1	1.0	3.6	4.8	17.5	2.6	14.9
DNAJC8	21.7	8.8	11.5	0.8	3.2	4.1	16.8	2.7	15.7
DYNC1LI2	25.2	8.8	11.7	1.2	3.6	4.4	16.4	2.2	11.7
TEAD4	19.5	7.7	9.4	2.1	4.4	5.4	16.7	3.6	16.5
NDUFS3	25.0	9.2	13.5	1.5	3.5	4.6	15.8	2.0	10.1
POR	26.3	9.9	13.9	2.2	2.8	4.3	13.3	2.6	9.8
TSC2	27.3	9.8	13.3	3.0	4.7	7.0	10.2	1.9	7.9
PCED1A	23.3	9.9	12.7	3.0	5.8	7.8	11.3	2.0	9.3
DEF8	23.7	9.7	12.8	2.1	3.9	4.9	14.9	2.6	10.4
FAM195B	29.4	8.3	12.7	3.4	5.1	7.5	9.3	2.6	6.6
CISD2	23.6	7.6	11.0	0.8	3.4	4.2	20.5	1.7	12.3
CIRH1A	19.8	7.1	10.7	1.3	4.0	5.4	19.7	2.7	14.3
PTPRG	24.1	7.9	10.3	1.9	4.4	5.6	15.4	2.5	12.9
VOPP1	22.5	8.1	10.8	1.9	5.3	6.2	14.6	3.1	12.2
AP1G2	23.3	10.0	12.7	2.0	3.7	5.8	14.1	2.3	10.9
IRF3	21.6	9.2	12.0	2.4	3.9	5.7	15.9	2.4	11.6
FLNC	18.5	6.6	8.4	2.2	4.1	4.6	20.7	3.6	15.9
LOC389641	27.4	5.9	12.1	1.6	3.1	3.4	19.9	1.7	9.5
PDCD6	23.5	7.9	12.8	2.7	4.8	6.0	14.2	2.2	10.4
NIP7	19.0	6.3	9.4	1.3	3.4	4.9	23.6	2.0	14.8
PAM	26.1	9.4	12.4	1.2	3.4	4.9	13.8	2.0	11.2
SGCE	24.7	11.9	15.0	1.0	3.7	5.5	10.1	1.8	10.8
CTXN1	21.1	9.2	10.9	5.4	5.0	7.7	15.2	2.3	7.6
ARL6IP1	23.4	9.4	12.1	1.0	3.0	4.4	17.6	1.9	11.5
C12orf10	22.8	9.9	12.0	2.2	3.8	6.1	13.4	3.0	11.0
GEM	23.8	8.5	12.6	1.1	2.6	3.2	16.2	3.7	12.3
KCTD10	21.1	8.5	10.6	2.0	4.8	5.3	15.5	2.8	13.6
ADRB2	21.3	6.9	11.1	2.3	4.4	7.2	14.5	2.2	14.4
PEPD	24.5	8.9	11.4	2.6	4.9	7.2	13.8	2.2	8.6

DLC1	18.1	6.8	9.0	2.3	4.4	6.3	17.7	3.2	16.1
DOCK9	19.8	6.2	8.3	1.4	4.1	5.3	20.3	2.8	15.8
CHCHD3	21.5	7.3	9.7	1.4	3.4	4.5	19.3	2.6	14.2
FOXN3	20.9	7.6	9.1	2.9	4.3	6.7	16.4	2.7	13.3
GAA	26.3	9.8	14.2	4.1	6.9	8.9	7.4	1.2	5.0
BCL6	28.8	10.2	13.6	1.8	2.8	4.4	10.7	2.3	9.1
TMEM104	24.0	7.9	10.5	2.7	4.7	6.0	13.5	2.7	11.6
DYNLL2	16.6	10.5	9.6	2.2	3.6	5.6	13.6	4.0	18.1
DLG1	23.6	8.4	10.6	1.0	3.6	5.2	15.7	2.3	13.5
RNF187	28.9	11.0	12.0	3.1	4.7	5.8	10.4	1.9	5.6
VARS	21.1	8.5	10.7	2.3	4.7	6.1	16.5	2.4	11.2
BAIAP2	16.1	6.8	8.8	2.2	3.4	4.8	22.0	4.0	15.5
MAP3K11	24.2	9.6	12.2	3.2	5.1	6.0	11.6	2.6	8.9
BCL7B	20.6	7.8	10.0	2.1	4.0	5.4	15.0	3.4	15.2
CSF2	30.4	14.1	19.5	1.9	3.6	5.0	4.5	0.4	4.0
SLBP	20.4	8.0	9.5	2.5	4.5	7.3	15.7	3.1	12.3
G6PD	21.4	9.3	11.0	3.4	6.0	8.2	11.7	2.3	10.2
SLC35C2	21.9	9.6	11.4	2.8	5.5	7.4	12.5	2.1	10.1
CBR1	20.0	8.8	12.6	3.2	4.7	6.3	14.1	2.9	10.7
CUX1	25.1	9.4	10.1	2.4	4.0	5.8	13.4	2.7	10.3
LPAR1	18.0	7.3	9.7	1.2	3.9	4.9	19.3	2.7	16.2
ENY2	18.2	8.3	10.8	1.5	4.3	4.4	17.7	3.3	14.6
SSFA2	20.3	7.0	10.0	1.6	3.5	4.9	18.6	3.0	14.3
ZFAND6	21.5	9.4	14.0	1.4	3.2	5.0	11.0	2.6	14.9
LOC101926982	26.2	5.6	13.0	2.0	3.2	3.1	20.7	1.4	7.9
R3HDM4	23.8	8.1	11.1	2.8	4.5	6.5	14.8	2.4	9.0
KIAA1967	21.5	8.2	10.0	1.7	3.4	4.8	17.0	2.9	13.5
SEC16A	25.1	8.1	10.9	2.2	4.2	5.3	14.5	2.3	10.5
MYL9	22.6	9.2	12.9	2.7	6.3	9.5	9.0	2.9	7.7
C5orf24	20.5	7.9	9.1	1.1	3.7	5.0	15.4	2.7	17.5
KLF5	22.6	8.2	10.3	1.2	3.2	3.6	13.9	4.1	15.7
C12orf44	22.5	7.0	10.4	1.5	3.5	4.9	18.3	2.9	12.0
FAM103A1	23.2	9.5	12.8	0.9	3.6	4.8	14.1	2.5	11.6
SPAG7	26.8	8.7	11.4	1.8	4.3	5.7	11.9	2.0	10.3
UQCC	22.8	9.5	12.1	1.1	4.0	5.7	13.8	2.2	11.7
ASAP1	19.5	6.8	9.0	2.0	4.5	5.9	15.3	2.9	16.9
ITM2C	26.8	10.7	13.7	1.9	5.1	7.1	7.6	1.9	7.9
LINC00649	26.7	5.1	12.2	2.2	4.0	4.4	18.2	1.6	8.3
SRGAP2C	22.1	8.6	11.0	1.2	3.9	5.4	13.9	2.5	14.2
ERLEC1	22.1	8.2	11.2	1.0	2.6	4.0	17.6	2.1	13.9
BCL2L12	20.8	7.6	11.3	2.3	4.6	5.2	16.6	2.4	11.9
RANBP3	24.2	8.9	10.3	1.6	3.7	4.4	15.4	2.3	11.9
LOC101928328	26.8	5.3	11.2	1.4	3.4	4.0	20.4	1.5	8.6
SDHA	22.6	7.6	10.9	2.4	4.7	6.1	15.0	2.3	11.1
GRB2	23.2	8.4	10.3	0.8	2.9	4.5	17.0	2.0	13.7
ISCA2	22.2	7.4	14.0	2.6	4.1	5.7	15.3	1.9	9.4
PCNXL4	19.2	6.9	9.1	0.6	3.0	4.1	21.6	2.4	15.7
MAT2B	22.4	8.5	13.3	0.8	4.4	5.8	13.1	2.3	11.9
RPUSD1	19.1	8.0	9.5	2.6	4.0	5.6	17.4	3.3	13.0
SOGA2	21.1	7.1	10.0	2.7	4.3	6.2	16.2	2.8	12.2
NBPF24	23.9	7.6	10.9	1.4	3.8	4.6	17.1	2.0	11.2
PCYT2	27.0	10.2	13.8	2.7	3.0	4.9	11.1	1.2	8.4
FUBP3	21.8	9.4	11.2	2.1	4.7	6.2	11.5	2.6	13.0
LDOC1	22.7	7.0	10.6	2.9	4.8	7.5	16.5	1.9	8.5
TMPO	18.6	7.5	8.8	1.9	4.2	5.7	17.5	3.0	15.3

ABTB1	22.6	10.3	12.9	2.2	4.4	6.0	13.6	1.9	8.5
FHL1	24.3	8.9	10.9	1.1	2.7	4.4	15.2	2.3	12.6
SLC27A1	25.9	9.4	13.0	2.9	5.2	6.6	10.1	1.7	7.4
QTRT1	22.8	8.8	12.0	2.9	5.3	7.4	11.5	2.2	9.2
ARAP3	24.2	9.3	13.0	1.5	3.3	4.3	13.6	2.5	10.6
VEZF1	22.7	9.1	11.5	1.3	3.8	5.2	11.3	3.0	14.2
GNL1	21.4	8.2	10.8	2.1	4.2	5.4	16.4	2.0	11.6
C21orf33	21.7	8.6	11.0	3.9	6.3	8.2	12.6	2.1	7.7
PPP2R5D	24.3	8.9	11.5	1.7	4.1	5.7	12.0	2.5	11.4
SLC35A4	22.7	8.2	9.9	2.4	5.1	6.5	14.8	2.4	10.1
TIMM17A	21.0	9.5	12.1	0.6	2.9	4.3	16.0	2.3	13.3
EMC3	24.2	10.2	15.2	1.1	2.5	4.2	11.6	1.8	11.1
RAC3	18.7	6.8	9.6	4.1	5.1	8.4	15.8	2.9	10.5
PLK2	22.9	8.9	12.4	1.9	5.2	7.2	11.3	2.2	9.9
PKP4	20.7	8.0	9.7	1.5	3.5	5.1	15.8	2.9	14.9
LOC101929415	28.0	5.0	12.8	1.2	4.2	4.2	15.1	1.3	10.2
ANKRD40	22.5	8.4	10.6	1.4	3.5	4.8	14.0	3.2	13.5
SPATS2L	24.7	9.3	12.6	1.7	4.2	5.8	11.3	2.3	10.0
SOX4	31.5	12.0	17.3	3.3	4.1	4.4	4.5	1.1	3.8
EXT2	23.9	8.9	11.8	1.1	3.9	5.0	14.7	1.7	11.0
MAP2K1	18.4	7.1	8.9	1.1	2.8	4.9	20.0	2.8	15.8
SEPN1	26.0	9.1	11.6	2.9	4.3	7.5	9.7	2.0	8.7
ZNRF3-AS1	25.7	5.6	11.3	1.1	3.0	3.3	20.9	1.4	9.4
TARDBP	23.2	6.8	8.9	0.8	4.0	3.9	16.5	2.5	15.3
MRPS11	22.8	8.9	10.9	1.6	4.2	7.0	13.0	2.7	10.6
LUC7L2	20.2	8.0	9.6	1.4	3.4	4.8	15.8	2.6	15.9
FAM43A	22.5	7.9	10.2	4.0	6.5	7.1	12.3	2.4	8.7
USB1	23.7	8.7	11.1	1.9	3.4	5.9	14.2	2.1	10.5
NAA50	20.3	7.0	8.6	0.4	2.0	3.8	22.0	2.0	15.5
RAF1	21.2	9.0	10.9	1.2	3.4	4.7	14.5	2.7	13.9
SPSB3	21.0	9.0	11.2	2.4	4.2	5.9	13.3	2.8	11.6
GFPT2	24.0	7.9	11.4	2.1	4.8	6.4	13.1	2.0	9.7
RNF135	23.5	9.9	12.1	3.2	3.8	6.1	11.5	2.1	9.1
INPP5K	22.6	9.3	10.9	1.8	4.4	5.3	15.2	1.8	10.2
SF3A3	19.8	7.6	9.2	1.0	3.3	4.6	17.1	2.8	16.0
SNORD97	21.4	8.0	9.3	0.1	3.4	1.8	25.6	1.3	10.3
PSMD13	21.3	8.1	11.0	1.0	2.9	4.7	18.4	1.9	11.9
WAC-AS1	20.7	6.8	9.3	1.0	3.2	4.1	20.7	2.1	13.3
ADIPOR1	23.5	8.8	11.2	1.3	3.3	4.7	14.1	2.6	11.8
HEATR2	22.0	8.6	11.1	3.4	4.4	6.4	13.0	2.3	10.0
DKC1	16.9	6.9	7.8	1.0	3.5	4.1	21.8	2.7	16.4
VAMP3	22.5	8.6	12.9	1.4	4.3	5.2	12.4	2.1	11.7
LOC101929259	26.5	5.3	11.2	1.6	2.9	3.5	20.3	1.4	8.4
RNASEH2A	17.4	6.1	9.4	1.7	4.6	5.2	20.7	3.0	12.9
SLC25A4	19.1	7.8	9.6	1.4	2.6	4.4	19.6	2.5	14.0
BLVRB	24.6	9.1	13.6	3.4	6.3	7.6	8.7	1.1	6.7
CD3EAP	17.7	7.0	7.9	2.1	4.4	6.2	17.2	2.9	15.5
CHIC2	17.8	6.4	9.0	1.1	3.9	4.1	21.1	2.7	14.8
AREL1	24.2	8.4	10.7	1.2	3.4	4.7	14.0	2.1	12.3
BDH1	12.3	4.4	6.3	3.1	5.6	7.9	22.2	3.1	16.0
SNRPB2	21.7	9.3	12.1	0.5	2.4	3.3	14.5	2.2	15.0
DEAF1	23.9	8.4	10.5	3.4	5.1	7.1	12.4	1.9	8.1
AMZ2	23.7	9.4	12.4	0.8	3.4	5.2	12.1	1.9	11.8
HMHA1	24.4	7.0	11.0	2.1	3.8	4.9	16.2	1.9	9.3
FBXW11	22.4	7.8	9.7	1.0	2.6	3.5	19.1	2.2	12.3

SRXN1	17.6	7.6	10.5	3.4	5.0	7.1	14.0	3.1	12.4
PARP1	16.7	6.7	7.7	1.3	3.5	4.7	19.1	3.3	17.6
STX3	21.3	9.2	11.2	0.7	3.4	3.8	13.8	2.5	14.6
BCYRN1	27.9	6.8	14.0	0.9	3.2	7.6	11.7	0.8	7.5
NDUFS2	23.6	9.4	11.7	1.6	4.8	5.7	13.5	1.6	8.4
PSMC1	22.8	9.0	11.4	0.9	3.8	4.9	14.4	2.1	11.0
ABCF2	19.2	7.0	9.0	1.3	3.8	4.7	18.0	2.7	14.7
SAV1	19.2	7.2	8.7	0.6	1.9	2.8	18.8	3.2	17.9
FNBP1	21.9	7.7	10.0	1.5	4.0	4.8	14.6	2.5	13.2
TXLNA	17.7	7.1	9.8	1.8	4.2	5.4	15.9	3.1	15.2
SNORD14C	16.9	7.1	7.5	2.1	8.0	5.4	13.4	2.3	17.3
KIAA0195	27.4	11.0	15.2	2.0	3.4	5.1	8.1	1.6	6.4
LSM14B	22.2	8.3	9.9	2.4	3.7	6.7	11.7	2.4	12.6
LOC101929511	26.4	5.3	10.6	1.4	3.3	3.5	19.4	1.4	8.7
GTPBP4	16.9	7.0	8.8	1.0	3.0	3.7	22.2	2.7	14.7
ZBTB4	21.7	8.6	10.9	2.7	4.6	6.9	11.8	2.8	10.0
TM2D3	24.2	8.4	10.2	1.4	3.2	4.6	15.8	2.0	10.1
TMC6	21.9	9.4	11.9	2.2	4.3	5.0	13.0	2.5	9.8
AP3S1	20.1	9.2	10.8	2.4	3.9	6.5	13.0	2.0	12.1
SMARCC2	21.1	7.8	9.8	2.3	4.6	6.2	12.4	2.8	12.8
NELFA	22.0	8.5	11.5	3.1	4.1	5.8	11.2	3.3	10.4
CHPF2	22.7	8.6	11.5	1.7	3.3	5.2	14.7	2.3	9.8
RRP12	19.2	6.7	8.7	1.9	3.6	5.2	18.3	2.5	13.7
DDX55	20.3	7.8	9.9	1.1	3.4	4.6	18.7	1.8	12.2
TSPAN14	22.7	8.0	9.6	1.4	4.0	4.3	16.2	1.8	11.8
TRIM27	19.5	7.6	9.0	2.1	4.1	5.3	16.5	3.2	12.5
ESYT2	20.2	7.1	9.9	1.7	3.5	5.1	16.9	2.6	12.8
LRFN4	20.8	9.4	11.0	2.5	3.6	5.4	13.1	3.0	11.0
TCEAL4	21.5	9.1	11.4	1.4	4.3	6.4	10.7	2.3	12.6
SYF2	23.1	8.9	12.1	1.0	3.0	4.5	14.2	2.3	10.7
MET	22.2	7.7	10.0	0.8	2.8	4.0	16.6	2.2	13.5
PDCD6IP	23.2	9.1	11.1	1.3	3.1	4.6	13.7	2.0	11.3
CARHSP1	20.2	10.6	14.8	2.0	3.8	4.1	11.3	2.3	10.5
PQLC1	22.7	9.2	9.5	2.6	3.7	5.3	14.5	2.0	10.0
WDR43	16.7	6.4	8.7	1.5	3.2	5.0	18.7	2.7	16.7
ZFPL1	20.3	8.2	10.0	3.8	4.8	8.0	11.5	2.5	10.5
CBFB	19.9	7.9	10.1	1.7	3.8	4.2	16.5	2.9	12.6
F2R	20.9	7.8	10.0	1.5	4.0	6.0	13.6	2.9	12.7
SART3	23.5	7.9	9.6	1.5	3.1	4.7	16.2	2.2	10.7
MAN1B1	22.7	9.3	11.2	2.7	6.0	7.2	10.7	1.7	7.9
DHX9	19.1	7.2	8.7	1.2	3.6	4.8	18.3	2.3	14.4
COX16	22.6	8.5	12.7	0.1	2.0	2.8	18.6	1.0	10.9
PFN2	18.4	8.1	11.4	2.2	3.6	6.8	15.2	2.4	11.3
LOC101928514	25.8	5.4	11.3	1.4	3.7	4.0	18.2	1.5	8.3
AGPAT1	21.0	8.5	10.8	2.3	5.4	6.6	12.0	2.5	10.3
PWP1	18.3	6.7	9.5	1.4	3.3	4.6	18.4	2.4	14.7
TSPYL1	20.1	8.1	10.9	2.7	4.3	5.7	13.1	2.6	11.8
ATAD1	22.3	7.5	9.9	0.5	2.9	3.4	17.5	1.8	13.5
SDC3	28.3	11.3	13.4	2.5	4.0	5.4	7.0	1.3	6.2
TSC22D3	24.3	8.4	10.6	0.9	1.2	1.8	18.9	2.3	10.8
AZIN1	15.9	6.5	9.0	0.5	2.1	3.4	22.3	1.9	17.6
KMT2A	21.9	6.1	9.3	1.1	3.1	4.1	16.9	2.4	14.2
TXNL1	19.4	9.1	11.5	1.3	3.2	4.9	13.2	2.4	14.1
ZNF384	18.3	8.1	9.1	2.2	3.8	6.1	11.9	3.4	16.1
PARVA	21.3	7.7	9.7	1.9	4.5	5.5	15.7	1.9	11.2

ENSA	21.9	7.8	10.9	2.6	4.9	7.1	11.9	2.0	10.0
ST6GALNAC4	18.9	7.5	9.6	2.5	3.6	5.1	17.2	2.0	12.7
MFN2	22.0	8.6	11.0	1.6	3.7	5.0	14.5	2.0	10.6
CREBBP	19.5	6.1	8.6	2.2	3.6	4.9	14.7	3.8	15.6
ARRB1	24.5	9.0	11.9	1.5	3.8	4.5	12.6	2.1	9.1
TPP1	25.8	9.2	13.1	2.2	4.6	7.1	8.8	1.5	6.7
SURF6	18.5	6.9	8.6	2.3	4.4	4.8	17.8	3.0	12.6
GTF3C5	24.2	10.0	11.9	2.5	4.5	6.1	10.1	1.9	7.9
HDAC2	21.6	7.1	9.5	1.5	3.2	4.3	17.0	1.9	12.8
CYFIP1	23.3	8.3	10.7	1.9	4.2	5.3	13.2	1.9	10.1
HSPA13	20.3	8.5	10.9	0.3	2.7	3.3	18.3	1.6	13.2
SCHIP1	21.3	7.7	11.9	1.6	3.0	3.5	16.2	2.2	11.4
TP53I13	22.1	9.5	11.6	3.1	4.1	5.7	12.3	1.9	8.4
RGL2	21.8	8.7	11.2	1.8	4.0	4.9	15.0	1.9	9.4
RNF166	20.7	8.7	11.6	3.2	5.7	7.9	9.2	2.6	9.0
HRAS	22.4	8.0	8.5	2.3	4.2	5.3	14.0	2.9	11.3
PTGES2	20.9	8.4	11.7	3.0	4.6	5.8	13.8	1.7	8.9
YY1AP1	22.4	7.8	8.8	1.3	2.9	3.9	15.9	2.3	13.4
DNAJA2	21.7	7.9	11.4	0.9	3.2	3.7	14.6	2.2	13.0
TMEM54	22.6	7.9	12.3	3.4	4.3	6.9	10.2	2.1	8.9
MEX3D	21.0	9.5	10.7	3.8	4.3	6.0	10.7	2.9	9.7
RPTOR	20.2	7.4	9.9	2.8	4.4	5.8	14.1	2.4	11.5
PVRL3	19.2	7.2	9.3	1.6	4.1	6.0	15.2	2.5	13.4
RNF167	20.8	8.6	10.7	1.7	4.4	6.6	12.2	2.4	11.0
C1orf86	24.6	6.1	10.9	2.1	3.5	5.1	16.1	1.6	8.5
CNP	21.1	8.6	10.5	2.1	4.4	5.5	13.5	2.5	10.2
STAMBP	21.6	8.5	10.4	1.4	3.5	4.9	14.2	2.3	11.8
ABLIM3	22.5	7.9	10.9	2.5	5.1	7.2	10.9	1.9	9.4
CHP1	23.3	8.4	10.0	1.1	3.5	4.5	13.1	2.2	12.2
TMEM214	20.9	7.8	9.0	1.9	4.0	5.0	15.0	2.6	11.8
TAF15	17.3	6.5	8.9	2.1	6.0	8.1	14.8	2.1	12.3
PALM2-AKAP2	16.7	6.0	7.7	1.8	4.1	5.6	17.8	3.0	15.5
NFKBIZ	27.8	10.6	16.3	1.2	2.9	3.9	7.9	1.2	6.3
WDR45	17.8	7.7	10.0	2.4	4.4	6.0	14.2	2.6	12.9
LSM2	19.3	8.0	11.3	1.5	3.2	5.6	13.1	2.2	13.8
ABHD2	24.8	8.1	11.1	1.6	3.2	4.3	12.9	2.4	9.6
LOC283335	22.7	7.4	10.2	0.8	2.8	3.6	17.5	1.6	11.3
DDX46	17.9	6.4	8.2	0.7	2.4	3.0	20.8	2.4	16.1
TUBGCP2	22.5	8.3	10.8	2.5	5.1	7.1	10.8	2.0	8.9
ZMYND19	17.8	7.4	8.8	1.6	3.0	4.0	17.7	2.9	14.7
LPCAT1	25.9	10.2	12.1	2.2	4.3	5.4	9.5	1.5	6.8
FAM53C	20.1	7.7	9.5	2.9	3.4	5.6	12.4	3.6	12.7
DGCR2	26.5	9.5	12.7	2.3	4.3	5.3	9.0	1.6	6.8
ADAMTS1	10.5	3.3	5.5	2.1	3.8	5.3	25.1	4.4	17.9
INE1	26.4	4.8	11.1	2.1	2.8	3.6	18.4	1.2	7.4
HDAC5	21.6	8.3	10.5	2.2	3.7	5.2	13.3	2.4	10.7
NSFL1C	21.4	9.2	11.3	1.9	4.1	5.7	11.6	2.4	10.3
MRPS16	19.7	6.5	10.3	2.4	5.3	6.3	14.3	1.9	11.3
TPM2	23.3	8.5	13.4	2.2	4.3	5.9	10.6	1.6	8.1
NDUFA10	20.7	7.9	10.3	1.5	3.7	5.2	12.7	2.8	13.2
TMEM33	20.1	6.7	8.4	0.6	2.5	3.9	18.7	2.7	14.4
LAMB1	22.1	8.3	9.9	1.9	5.3	6.6	12.5	1.7	9.4
AHR	22.9	8.1	10.4	1.2	3.9	5.4	11.6	2.0	12.4
WDR46	18.0	6.6	9.1	1.4	4.0	5.6	17.7	2.3	12.9
MAPK9	20.9	7.0	11.8	1.2	3.3	4.0	16.0	2.5	11.1

FYTTD1	20.6	8.2	9.9	1.0	3.7	4.6	14.8	2.2	12.7
ARAF	22.6	8.2	11.6	2.0	4.2	5.0	12.4	2.7	9.0
RBM14-RBM4	20.6	8.0	10.7	1.3	0.9	7.2	16.5	1.3	11.3
DNAJC10	17.7	6.7	8.4	0.5	1.8	3.4	22.5	2.1	14.6
ARL6IP5	30.1	10.0	15.5	1.3	3.1	4.4	7.8	1.0	4.6
LOC101928597	24.6	5.7	10.3	1.5	3.2	3.7	18.6	1.9	8.3
SNAPIN	23.0	8.7	13.8	2.2	6.0	7.0	9.0	1.4	6.5
PKD1	23.8	8.2	11.6	3.0	4.5	5.6	10.7	1.9	8.2
NMRAL1	21.7	8.1	10.5	3.0	5.1	8.4	10.3	1.7	8.6
ZC3H14	22.1	8.2	10.6	1.1	3.6	4.8	12.9	2.1	12.2
SIN3A	19.8	8.0	9.8	1.6	4.0	5.1	12.8	2.8	13.6
RAB5B	21.4	9.4	10.1	1.2	4.0	3.8	12.6	2.2	12.7
TOB2	20.6	7.7	9.4	1.9	3.5	5.9	13.1	3.2	12.0
ELAC2	19.4	7.3	9.2	1.9	3.7	5.0	16.0	2.5	12.3
SLC50A1	20.0	7.5	9.4	1.9	4.8	7.9	13.4	1.8	10.6
ALDH18A1	21.0	8.4	9.9	1.3	3.1	4.4	15.1	2.3	11.7
EVI5L	20.1	7.7	10.1	3.2	4.9	6.5	13.5	2.5	8.9
SRSF11	22.9	8.2	10.9	1.0	3.1	3.8	13.2	2.1	12.2
BRD3	21.7	6.9	9.7	3.0	5.0	5.6	11.9	2.6	11.0
XXYLT1	24.1	10.0	12.4	1.9	4.2	6.6	11.0	1.0	6.0
PFDN6	17.9	7.9	10.9	1.1	4.7	6.6	13.6	1.6	12.9
GSTM3	22.0	6.7	10.3	1.8	3.3	4.4	18.3	1.7	8.7
LOC101927178	24.7	5.2	10.7	1.4	2.9	3.4	19.0	1.4	8.3
LOC101927476	25.2	4.9	10.8	1.4	3.1	3.5	18.8	1.3	8.1
RBM15B	19.8	7.5	9.6	2.5	3.6	5.0	13.8	3.6	11.6
DRAM1	25.1	10.9	13.8	0.9	2.2	3.8	9.3	1.9	9.1
LOC101929491	21.2	7.3	9.8	0.7	5.6	5.5	9.2	2.8	15.1
ATP5J	21.4	9.0	12.3	1.2	3.4	5.3	9.5	2.1	12.9
MKKS	21.0	8.7	12.1	0.8	2.9	4.9	12.6	2.0	11.9
RARA	18.0	7.5	8.4	1.8	3.3	4.2	17.5	2.8	13.4
ARL4C	20.4	7.8	9.5	3.3	6.8	8.7	9.9	2.2	8.4
DDX23	24.0	9.3	11.8	1.2	2.5	3.6	13.7	1.7	9.2
MLST8	15.9	6.8	9.5	3.8	5.5	7.4	13.6	2.8	11.6
PGAM5	16.7	6.3	8.5	2.2	3.9	5.0	16.8	3.3	14.2
PIP5K1A	17.5	6.4	8.4	0.8	3.1	4.2	18.4	3.0	15.2
RPS27L	22.7	8.6	12.5	1.6	5.2	5.6	10.2	1.0	9.5
PPIL1	17.4	8.0	11.7	1.2	3.8	5.2	13.1	2.5	14.0
GRSF1	20.1	7.9	10.0	1.7	2.8	4.9	15.5	2.5	11.5
OTUD5	23.4	7.7	9.8	2.1	3.7	4.4	12.8	2.2	10.8
DCAF15	20.9	7.9	11.0	2.2	4.0	5.0	12.7	2.6	10.5
POLR2A	18.5	6.5	8.6	1.5	3.0	4.0	17.6	3.1	14.0
FERMT2	17.2	7.0	8.8	0.9	3.1	3.5	19.5	2.3	14.4
WIPI1	20.6	8.0	10.5	1.0	3.8	5.0	14.5	1.9	11.5
SAFB2	20.4	6.7	9.4	2.0	3.6	4.5	16.3	2.8	11.0
ELP4	24.1	5.7	10.9	0.9	2.7	3.7	18.5	1.2	9.1
ARFIP2	20.4	7.0	10.0	1.3	3.8	5.7	14.2	2.3	12.0
NBL1	23.5	11.0	11.4	2.5	3.6	5.7	9.3	1.6	8.1
PRKACA	22.5	9.6	11.1	1.9	4.8	6.8	8.8	1.6	9.4
ELL	20.5	7.2	9.8	2.7	4.6	5.6	12.8	2.6	10.9
REEP4	21.0	8.1	9.5	2.2	4.2	5.4	12.9	2.3	11.0
LIX1L	22.9	7.9	11.5	2.0	3.0	5.1	14.3	1.8	8.1
SLC35B1	23.1	7.6	11.4	1.0	3.2	3.6	15.3	1.7	9.7
MVD	23.2	9.8	11.9	3.0	4.7	6.3	9.2	1.4	6.9
SMDT1	18.1	8.5	12.4	2.3	3.7	6.9	14.3	1.4	8.6
ANXA4	21.9	7.8	11.2	1.8	3.6	5.4	13.5	1.5	9.4

CBL	20.1	6.1	8.2	1.2	2.8	3.8	17.5	2.6	14.1
JAGN1	16.0	7.3	11.1	1.5	4.6	5.9	16.2	1.9	11.9
ETV4	19.9	7.4	9.5	2.0	3.9	4.8	12.4	3.7	12.6
CTCF	19.1	7.1	8.0	1.5	3.3	3.9	15.7	2.7	15.0
WDR5	18.0	7.0	9.0	1.3	3.0	4.7	16.7	2.1	14.3
MRPS7	18.2	8.2	10.2	1.2	4.0	4.9	14.2	2.5	12.9
LOC100996251	25.2	4.6	10.9	1.4	2.7	3.2	18.7	1.6	7.9
FAM213B	21.6	8.4	10.0	2.3	3.5	4.9	15.3	1.7	8.5
ZFAND5	24.0	8.9	11.6	1.1	3.4	4.9	11.2	1.6	9.3
INO80B-WBP1	18.9	7.1	10.9	2.1	4.0	5.6	13.1	2.7	11.6
RRAS2	20.9	9.0	12.4	1.1	3.2	5.0	10.5	2.6	11.4
GATAD2B	20.7	6.5	8.6	1.6	3.1	4.2	14.5	2.7	14.1
PIP4K2B	23.9	8.8	12.6	1.7	3.8	4.5	10.3	1.6	8.9
IPO4	15.7	5.8	7.3	2.3	4.4	6.4	19.1	2.7	12.2
SNRPD1	17.1	4.6	8.7	0.8	3.2	3.5	19.8	2.6	15.6
MED24	22.9	8.4	11.0	2.1	4.0	5.8	11.2	2.0	8.6
COMMD6	20.3	8.4	13.3	0.7	3.5	3.5	14.2	1.8	10.1
OPTN	24.4	9.5	13.0	1.2	3.5	4.4	9.5	1.7	8.7
MEAF6	20.8	7.3	10.5	0.8	3.7	4.4	14.3	2.4	11.5
C15orf39	21.2	8.9	10.9	2.6	3.8	5.9	11.1	2.4	9.1
CCDC59	20.7	9.7	12.3	1.1	3.5	4.4	11.2	1.9	11.0
NCRUPAR	22.8	5.0	12.3	1.3	2.5	2.4	20.3	1.8	7.3
PRDX4	17.4	6.9	10.3	1.0	2.5	3.6	19.6	1.5	12.9
GSK3B	18.3	7.2	8.7	1.0	3.1	4.4	15.1	2.7	15.2
MED1	18.2	6.6	8.7	0.9	2.6	3.7	15.3	2.8	16.7
MRPL23	21.0	8.3	11.9	1.8	4.1	4.9	13.4	1.6	8.7
RNASET2	25.8	9.6	11.3	1.3	2.8	5.1	10.1	1.6	7.9
RING1	17.0	7.7	9.7	1.8	4.6	6.8	13.8	2.5	11.8
NCOA6	22.9	7.4	9.9	1.4	3.2	4.1	12.1	2.4	12.2
IKBIP	16.8	6.9	10.5	1.0	2.3	2.9	21.4	1.9	11.9
EPRS	18.9	6.6	8.3	0.7	2.9	3.9	18.7	2.2	13.5
BZW2	20.1	7.5	10.7	1.0	3.7	4.8	14.0	2.2	11.6
BCCIP	18.2	5.7	9.3	1.0	3.7	4.1	16.2	2.3	15.0
DTD1	23.0	7.9	10.6	1.4	4.7	5.7	12.3	1.0	8.9
PTPN23	21.3	8.0	9.8	2.8	4.2	6.0	10.8	2.2	10.5
PLEKHM2	20.8	7.7	10.0	2.3	4.6	5.6	12.4	2.2	9.9
MRPL36	17.3	7.3	7.8	1.3	3.2	6.0	16.4	2.4	13.8
STRAP	19.2	8.3	10.2	0.5	2.3	3.3	16.3	1.8	13.5
SHANK2-AS3	24.8	4.6	10.5	1.4	3.4	3.5	18.6	1.3	7.1
POLR2J	16.4	10.0	11.7	3.0	3.4	6.4	8.2	2.4	13.8
PDCD10	20.0	7.1	11.5	0.6	1.7	3.7	13.4	2.5	14.7
CMTM3	23.1	9.4	11.8	1.5	3.5	4.8	10.1	1.4	9.6
NR3C1	23.2	7.3	10.7	0.6	2.3	3.3	15.1	1.7	11.0
WBP11	18.6	7.7	9.3	1.9	3.6	5.2	13.6	2.7	12.5
SPTSSA	18.7	7.5	11.7	1.5	4.1	6.0	11.2	3.3	11.1
HEXDC	21.7	9.5	12.6	2.7	5.1	6.7	9.1	1.5	6.2
VPS72	21.5	8.6	9.6	1.9	4.1	5.7	10.2	2.6	11.0
NFE2L3	22.9	8.3	10.3	2.5	4.4	5.8	11.8	1.5	7.8
LOC101928118	26.6	4.2	11.6	1.7	3.6	3.5	15.2	0.9	7.8
WTAP	20.4	7.6	10.7	1.1	2.2	3.5	14.8	2.8	12.2
GNA13	20.6	7.5	9.3	1.3	4.0	4.1	15.3	1.9	10.9
SEC31A	21.5	8.4	10.9	1.3	3.5	4.9	11.0	2.0	11.6
FBRS	20.7	8.3	10.4	2.1	3.5	4.6	11.9	2.3	11.2
PPP1R12A	21.7	7.0	9.2	1.1	3.1	4.1	15.4	2.1	11.4
SNRPE	17.9	7.1	10.9	0.6	1.2	3.2	17.0	1.4	15.8

CEBPG	16.4	5.5	8.6	1.2	2.7	4.7	18.4	2.2	15.2
COA3	19.3	6.9	10.7	2.3	4.4	5.1	16.2	1.9	8.1
SLC25A22	18.5	6.7	8.6	2.5	5.1	6.0	14.1	2.7	10.9
SEPHS1	18.7	7.3	8.9	1.4	4.4	5.0	14.5	2.3	12.6
MVP	22.5	9.4	11.1	2.5	4.5	6.4	9.8	1.8	7.0
PUM1	20.1	7.3	9.2	1.5	3.5	4.4	14.4	2.3	12.1
GTF2I	20.7	7.6	9.7	0.6	3.1	5.2	14.9	1.9	11.2
ACOT7	18.4	7.3	9.0	2.3	3.4	4.8	15.5	3.0	11.2
HIST1H4A	16.5	5.6	9.0	2.4	5.0	6.8	16.2	2.2	11.1
DDX42	19.0	7.0	9.5	1.5	3.4	4.9	14.2	2.4	12.8
TIMM17B	19.7	7.3	9.8	2.3	3.7	6.0	14.9	2.3	8.8
FAM83G	19.4	7.2	8.7	1.7	2.9	4.6	15.7	2.7	11.8
CLINT1	18.9	7.4	9.1	1.2	3.6	4.1	13.9	2.4	14.2
PAPOLA	20.8	7.9	11.1	0.8	2.8	4.4	12.6	1.9	12.5
QRICH1	19.6	8.2	9.5	1.9	4.3	5.1	10.6	2.9	12.6
B3GAT3	21.8	9.1	11.5	2.9	4.9	6.9	10.2	1.4	6.1
ILF3-AS1	21.5	5.6	10.5	1.4	3.0	3.8	18.7	1.6	8.6
PSMD6	20.1	7.9	13.0	1.2	3.9	4.5	11.4	1.8	10.8
DPH1	23.7	8.8	10.7	2.0	3.2	5.4	11.0	1.9	7.9
NDUFA6	21.4	7.7	11.9	1.4	3.0	4.4	11.6	2.2	11.0
C1orf95	2.8	9.6	6.5	23.4	11.0	0.3	6.0	9.6	5.4
FAM91A1	21.0	7.0	9.5	0.5	2.6	3.6	16.6	1.8	12.0
P2RX5-TAX1BP3	22.8	6.2	10.8	1.7	3.4	4.6	15.6	1.7	7.7
RPRD1B	24.4	7.4	10.0	0.8	3.5	3.8	13.7	1.5	9.4
ASB6	19.5	7.1	8.9	1.8	3.4	4.3	16.3	2.5	10.6
DEGS1	23.1	9.2	10.4	0.8	2.8	3.6	12.0	1.6	11.0
APBB1IP	20.0	7.9	8.2	2.7	3.9	4.8	13.0	2.7	11.3
PAFAH1B1	20.6	7.1	9.2	0.7	2.6	3.3	17.1	2.1	11.8
PLAGL2	17.8	6.7	8.6	1.7	3.1	4.2	15.2	2.8	14.2
LOC101929770	24.1	5.2	10.7	1.2	2.6	3.2	17.8	1.5	8.2
SLC35A2	21.8	8.3	9.1	1.7	3.8	4.7	12.1	2.1	10.7
RSAD1	15.6	6.8	7.4	1.6	3.1	4.6	20.4	2.5	12.4
DDX1	19.6	7.3	9.2	0.6	3.5	4.6	16.7	1.5	11.5
DPP3	21.8	8.9	10.6	2.1	3.5	5.0	13.1	1.6	7.8
AGFG2	23.5	9.9	12.2	1.5	3.2	4.8	9.6	1.8	7.8
AK2	16.5	5.5	8.4	1.4	3.3	4.1	18.7	2.8	13.7
CTBP2	18.0	7.0	8.6	1.8	3.2	4.1	15.5	2.5	13.6
LOC100129269	24.3	5.1	10.3	2.1	3.1	3.8	16.5	1.7	7.3
SRA1	19.4	7.1	10.5	2.5	4.0	5.0	12.4	2.5	10.8
UBA2	18.0	6.8	9.4	0.8	2.9	4.0	15.4	2.3	14.5
IMPAD1	21.6	6.5	10.8	1.5	2.7	3.3	16.3	1.9	9.7
EXTL3	23.1	8.9	11.2	1.2	3.4	4.0	11.9	1.7	8.8
FBXO32	21.6	8.2	10.4	1.8	5.1	7.1	9.9	1.8	8.3
TMEM120B	18.7	6.9	8.6	1.8	3.5	4.7	15.5	2.3	12.2
RSRC2	20.1	7.6	7.6	1.2	2.0	4.7	15.6	2.5	12.8
VPS11	21.1	7.7	9.4	1.8	4.1	5.5	12.7	2.3	9.6
UBE2G2	19.8	5.2	8.7	2.0	3.6	5.9	15.9	1.7	11.3
RUFY1	20.9	7.1	9.7	1.4	3.4	4.2	14.7	2.2	10.3
L3HYPDH	20.3	7.5	11.7	2.4	4.4	6.0	11.7	1.8	8.2
MPLKIP	14.9	7.9	10.5	1.4	2.3	4.1	14.4	2.6	16.0
GALNT2	21.5	8.0	11.4	1.5	3.6	5.0	12.4	1.7	8.9
ALKBH2	19.2	8.2	9.8	1.4	3.6	5.3	12.4	2.4	11.8
ADK	16.4	5.7	9.5	0.6	2.8	3.8	18.9	2.2	14.1
MGRN1	22.0	7.9	10.3	3.0	4.5	5.9	10.5	2.1	7.8
MRPS5	20.0	7.5	10.8	1.4	3.4	4.8	13.2	1.9	10.9

PFDN4	19.9	8.7	9.7	0.5	3.1	3.8	13.9	1.6	12.8
RAPGEF1	20.4	7.5	9.4	1.5	3.3	4.4	14.5	2.3	10.6
NPIPB3	22.0	5.5	10.4	2.7	4.9	5.6	10.7	2.1	9.9
CCNY	20.9	6.3	8.8	1.1	4.2	5.0	13.3	2.3	11.9
LEPREL4	21.6	8.5	11.8	2.7	4.7	6.2	10.1	1.5	6.7
SMG9	20.8	8.1	10.2	2.3	4.6	6.1	10.2	2.3	9.4
LOC101928936	24.5	4.7	11.7	1.5	3.6	4.4	14.8	1.3	7.4
UBL7	18.4	8.1	9.9	1.1	3.6	3.7	14.4	2.4	12.3
RYBP	16.7	6.5	9.1	1.1	3.8	5.1	11.6	3.4	16.5
SMS	18.4	7.2	8.5	1.1	2.5	3.9	16.8	2.4	13.0
RBX1	22.1	7.2	13.4	1.2	2.3	3.6	14.4	1.4	8.0
NPRL3	20.1	8.6	10.4	2.9	4.9	7.4	9.2	2.0	8.0
FBXO7	19.6	7.7	9.6	1.2	2.9	4.4	14.2	2.5	11.5
TERF2IP	18.3	6.9	8.7	1.8	3.5	4.8	16.8	2.2	10.6
MAGOH	20.6	9.0	10.5	1.0	3.3	4.0	10.9	2.1	12.0
DNAJC11	19.9	7.3	8.7	1.3	3.4	4.3	14.7	2.2	11.7
KIF3B	22.0	7.5	9.9	1.0	2.7	3.8	14.2	2.0	10.3
WHSC1	16.5	5.2	10.0	1.2	3.2	3.7	18.5	2.4	12.8
FIS1	20.2	8.7	11.9	2.3	4.1	7.9	9.2	1.1	7.9
BCAS2	20.5	8.0	10.7	1.3	3.1	4.4	12.8	1.3	11.1
PCNX	21.6	6.4	9.0	1.5	3.2	4.3	14.8	2.1	10.5
ICT1	19.8	8.0	12.4	2.6	5.9	5.8	9.4	2.3	7.1
MSMO1	22.7	11.2	14.0	0.1	0.9	1.4	13.2	1.1	8.6
NT5C	18.4	8.8	11.1	2.4	4.4	6.0	11.0	1.8	9.3
COX11	18.7	6.9	10.7	2.2	4.1	4.3	13.5	2.1	10.8
DNMBP	18.3	6.6	7.8	1.8	4.2	5.6	14.0	2.2	12.7
HOXB9	12.6	5.1	6.3	2.1	3.0	3.8	20.7	3.1	16.5
KCTD12	18.0	5.8	8.2	3.7	5.0	6.6	13.5	2.6	9.7
TRAF4	16.9	6.8	9.1	1.6	3.4	4.4	16.3	2.6	12.0
DCXR	16.1	6.1	8.7	2.7	5.4	5.5	16.3	2.3	10.0
TAOK3	18.8	6.5	9.0	1.3	3.5	4.5	14.7	2.3	12.3
BRPF3	17.8	6.2	7.8	1.4	2.6	3.8	17.7	2.9	12.9
FBXW8	21.6	6.6	9.5	2.0	4.3	5.2	14.4	1.6	7.9
TRMT61A	15.3	6.2	7.8	3.3	4.2	6.8	16.1	2.2	10.9
SLC20A1	21.2	7.1	10.5	0.8	2.6	3.4	14.7	1.9	10.8
VPS26B	20.5	7.3	9.7	1.7	3.8	4.2	14.0	1.7	10.0
WDR82	16.8	5.9	8.0	0.7	2.8	3.9	15.6	3.2	16.0
TRNH	24.2	18.2	11.7	1.8	2.6	3.3	2.1	3.5	5.3
NENF	21.6	7.1	11.0	1.5	4.3	4.4	11.5	1.5	9.9
ATP6V1D	19.7	8.1	10.5	0.4	2.4	3.0	16.1	1.9	10.7
FAM134A	16.6	6.0	8.3	2.6	5.0	6.3	14.8	2.5	10.6
TFPI2	16.6	6.5	7.9	1.2	3.7	5.0	17.6	2.5	11.7
PGPEP1	20.6	8.7	9.4	2.5	5.1	6.5	8.8	1.6	9.5
TRNQ	17.6	9.2	6.6	3.8	2.0	6.6	9.7	3.0	14.1
EIF2B1	19.0	7.2	9.3	1.2	4.0	4.4	14.8	1.7	11.2
ZBTB38	21.7	7.9	9.8	1.0	3.4	4.0	11.6	2.0	11.2
APBA2	20.3	8.0	10.1	2.1	3.9	4.7	11.4	2.1	9.9
PPP6R3	20.8	6.1	8.5	1.0	3.1	4.1	14.3	2.1	12.7
FLJ30403	24.5	5.2	11.2	1.4	2.4	2.6	17.0	1.4	6.9
MAPK8IP3	24.0	9.2	13.5	1.9	3.2	4.0	8.2	1.7	6.7
PJA2	20.2	7.8	9.3	0.6	2.5	3.7	14.6	1.7	12.2
GOLGA2	22.3	7.9	10.3	1.7	3.1	4.1	11.9	1.8	9.5
PRPF38B	18.7	7.5	9.0	1.2	2.7	3.4	13.7	2.7	13.7
NGDN	17.7	8.1	9.8	0.6	2.9	3.6	15.9	2.3	11.4
ARRDC2	22.3	7.6	10.8	2.4	3.6	4.1	12.1	2.1	7.4

SETD5	19.5	6.5	8.9	1.2	3.3	4.6	13.3	2.3	12.8
PCNA	16.0	6.9	9.9	0.9	2.6	3.7	16.6	1.8	14.0
SPAG9	19.3	6.7	8.7	1.0	2.9	3.7	15.5	2.0	12.6
GRK6	18.3	7.1	9.0	2.3	4.4	5.4	14.4	2.0	9.4
PPP2CB	19.7	6.9	9.1	1.2	3.2	3.9	13.8	3.0	11.5
WIPF2	21.2	7.3	8.2	2.3	4.0	5.7	9.2	2.8	11.8
TBC1D16	22.9	7.6	11.3	2.4	3.9	5.7	9.8	1.7	7.0
NPIPB5	23.3	4.8	10.4	1.1	3.0	3.8	15.4	0.9	9.6
SNORD65	15.8	6.0	6.6	5.8	4.0	5.6	12.2	0.7	15.7
TACC2	22.6	8.2	9.9	1.2	2.1	3.9	11.9	2.2	10.3
SNTB1	20.4	7.3	9.5	2.7	3.9	5.8	11.0	2.2	9.4
PVT1	19.7	5.5	9.6	1.2	2.7	4.0	17.6	1.8	10.1
SMNDC1	19.7	7.3	9.7	0.4	1.7	2.7	17.4	1.7	11.8
RRP7A	13.4	4.7	7.0	2.6	5.3	6.7	16.2	3.0	13.3
ABHD17C	22.1	7.7	9.4	3.1	3.8	5.8	9.6	2.4	8.2
PPARD	21.1	7.3	9.2	2.1	4.0	4.9	12.3	2.2	9.0
IER3IP1	16.4	7.2	8.9	1.2	3.1	5.6	11.3	3.1	15.2
LINC00294	22.6	5.1	10.2	1.3	2.5	3.2	16.7	1.6	8.8
LOH12CR2	23.5	4.4	10.8	1.1	2.1	2.8	18.1	1.5	7.7
TIMM10	16.5	7.8	10.1	1.1	1.7	4.0	17.0	2.3	11.5
SMPD4	19.6	7.3	9.7	2.0	3.4	5.5	12.3	2.6	9.7
BAG1	20.3	7.3	9.6	2.1	3.8	4.1	14.8	1.7	8.4
SMAD2	20.1	7.3	9.7	0.8	2.4	3.8	14.8	1.8	11.2
LOC101927710	22.4	4.7	10.1	1.2	3.0	3.6	18.1	1.3	7.6
TBC1D14	17.4	7.2	9.5	1.4	3.2	4.9	13.7	2.3	12.3
NUDT5	17.7	6.2	8.6	1.2	2.3	4.0	17.5	1.6	12.8
TOMM70A	18.7	6.8	9.1	1.1	3.1	4.0	15.4	2.2	11.6
ADCY3	20.1	7.3	9.5	1.8	3.9	5.1	12.2	1.8	10.1
PCYT1A	21.4	6.9	9.1	1.4	3.4	4.8	14.1	1.4	9.4
MAPK14	18.8	7.7	8.5	1.4	3.0	3.7	16.7	1.9	10.2
B4GALT2	20.5	8.2	10.1	2.5	3.6	5.8	10.5	2.3	8.5
TCOF1	17.6	6.6	9.1	1.8	3.4	4.4	14.4	2.3	12.2
LOC101927929	22.8	4.5	10.5	1.1	2.8	3.1	17.5	1.4	8.2
HERC4	20.5	5.8	11.3	0.8	3.1	2.5	15.8	1.3	10.8
KANK2	21.1	8.4	10.6	2.6	4.4	5.6	9.7	1.9	7.4
ME3	17.2	6.5	9.3	2.5	4.2	5.7	13.5	1.8	11.0
MAD1L1	19.5	7.4	8.6	1.9	3.6	5.5	12.9	2.2	10.2
CMSS1	15.7	7.2	9.1	1.2	3.2	4.7	15.0	2.1	13.5
ASPSCR1	20.4	7.6	10.0	2.3	4.3	5.3	12.2	1.8	7.5
FTSJ3	19.3	7.2	8.9	1.6	3.5	4.7	13.1	2.2	11.1
HMBS	19.7	8.2	10.7	1.5	3.6	4.9	13.2	1.3	8.6
PHF15	16.2	6.3	7.8	2.2	3.5	4.9	15.6	2.8	12.2
CHORDC1	17.3	7.5	9.8	0.7	1.7	2.9	16.4	2.3	12.9
POLR2K	21.4	8.0	8.9	0.2	4.0	2.5	12.5	1.2	12.8
NRD1	18.1	6.8	8.9	0.9	2.9	4.1	16.3	1.9	11.5
TJP1	17.3	5.5	7.0	1.0	2.8	3.8	16.6	2.6	14.8
FHOD1	19.1	7.6	9.6	2.0	3.7	5.0	13.1	2.1	9.2
PCYOX1	23.3	8.3	10.4	1.0	2.9	4.1	11.3	1.5	8.6
AKAP8	21.6	7.5	10.1	1.4	3.1	4.1	12.3	2.2	9.0
ADRBK1	20.5	8.6	10.5	1.7	3.7	4.6	10.7	1.8	9.2
TPST2	21.5	7.9	11.1	1.9	2.8	4.3	11.8	1.9	8.0
CCDC130	20.4	7.7	11.2	2.5	4.2	4.7	10.1	2.1	8.4
KRR1	17.6	7.5	10.0	0.6	2.3	3.2	15.4	1.8	12.8
HMOX2	19.7	6.1	7.3	1.5	3.4	4.9	15.4	1.9	10.9
SLC38A10	21.0	7.9	11.1	2.6	4.1	5.9	10.2	1.5	6.9

ATG3	16.5	7.3	10.4	1.2	3.3	4.3	14.5	2.4	11.3
NARF	22.3	7.9	11.9	1.2	2.8	4.0	11.1	1.8	8.2
TBRG1	19.7	7.2	9.8	1.0	3.0	4.1	13.9	2.1	10.3
PTP4A1	21.2	9.5	10.0	0.4	1.8	3.7	11.2	1.7	11.6
MEF2A	22.1	7.6	11.4	1.4	3.7	5.0	9.7	1.5	8.8
EHMT2	20.7	7.5	9.3	2.0	4.4	5.2	11.1	1.9	8.8
ITGA2	22.4	7.0	9.9	0.7	2.8	4.0	13.0	1.4	9.8
RSU1	17.5	7.1	8.4	1.1	3.4	4.4	14.0	2.4	12.6
CARD8	20.7	5.9	8.2	1.1	2.8	2.8	17.6	1.8	10.1
FOXO3	16.4	6.9	9.1	2.0	3.5	4.9	14.2	2.5	11.4
MRPS30	16.9	6.2	8.9	2.3	4.1	5.7	13.9	2.3	10.6
DUSP7	16.8	6.8	7.5	2.5	3.6	5.9	12.0	3.5	12.3
TBC1D2	19.8	7.4	9.5	2.0	4.3	5.7	11.8	1.9	8.4
SLC4A7	22.9	6.9	11.5	2.1	3.2	4.6	10.7	1.4	7.6
FLJ42102	22.5	5.0	9.9	1.0	2.6	3.1	17.8	1.3	7.6
LOC101928303	24.0	4.9	10.8	1.0	2.3	2.9	16.6	1.2	7.3
CHD2	20.3	7.1	9.4	0.8	3.1	3.2	13.5	2.5	10.8
DNPEP	20.8	7.3	10.8	1.7	3.6	5.1	11.2	1.9	8.2
PFKFB3	21.4	7.2	10.8	1.4	2.3	2.8	12.6	2.1	10.0
CHTF8	17.0	7.2	9.1	1.2	3.1	5.7	14.0	1.9	11.4
CKB	13.8	6.1	8.0	4.0	5.9	6.9	11.6	3.1	11.1
GALNT10	20.1	8.2	9.9	2.1	4.3	4.7	10.6	1.7	8.9
WNK1	18.6	6.3	8.1	1.6	3.5	4.7	13.1	2.7	12.0
KMT2D	18.7	5.0	8.1	1.9	3.3	4.1	15.4	2.6	11.5
EIF2AK1	21.3	8.0	9.3	0.8	3.3	3.7	12.3	2.2	9.7
LOC101929333	23.0	5.2	10.6	1.6	3.6	3.8	14.2	1.4	7.2
LOC101928868	23.0	5.0	10.8	1.2	2.9	2.9	16.1	1.3	7.3
SMN1	16.5	6.4	8.9	1.0	2.8	3.6	15.2	2.6	13.5
HCLS1	20.1	7.5	9.9	1.3	3.4	4.4	12.6	1.5	9.9
WBSCR22	20.4	6.1	10.0	1.4	3.5	4.2	14.7	1.7	8.4
IP6K2	19.0	7.2	11.0	1.9	4.2	5.5	12.1	1.8	8.0
C10orf32-AS3MT	22.2	4.8	10.0	1.4	2.9	3.4	17.1	1.2	7.6
RHOT2	17.9	7.8	10.0	3.0	4.4	6.1	10.8	1.9	8.5
BHLHE40	24.0	11.4	13.0	0.8	1.5	2.3	8.0	1.5	7.9
FLJ13197	22.3	5.2	10.5	1.7	3.4	3.9	14.9	1.5	7.0
DHX33	17.9	6.3	7.5	1.6	3.2	5.3	14.5	2.4	11.7
ARIH2	18.1	6.4	8.1	1.0	2.4	3.7	15.7	2.5	12.6
FAM21C	22.6	9.0	11.2	0.7	2.3	3.6	11.7	1.2	8.1
HDGFRP2	20.2	7.9	9.7	2.8	3.1	4.8	11.2	2.4	8.4
EXOSC1	18.2	6.6	8.9	2.0	3.4	4.2	13.5	2.1	11.5
TIMMDC1	20.2	7.3	11.6	0.7	2.4	4.4	14.7	1.2	7.9
ZFP36L2	15.4	6.7	7.8	2.8	4.0	4.9	12.9	3.6	12.2
RAB11FIP3	21.6	7.6	9.8	2.4	3.4	4.6	11.4	1.9	7.5
SSB	18.8	7.0	9.9	0.4	2.2	3.6	13.7	1.7	12.9
CTIF	18.3	6.7	8.0	2.7	4.5	4.8	11.5	2.6	11.1
PIP4K2A	21.9	7.1	8.9	1.2	2.8	3.9	12.2	2.0	10.2
CLIP2	22.6	7.9	10.6	1.7	2.9	3.8	11.9	1.8	7.1
FAM106A	23.1	4.7	9.4	1.4	3.1	3.1	17.4	1.1	6.9
C7orf55	21.2	5.8	11.5	1.5	2.9	3.6	15.6	1.1	6.8
PCID2	17.9	6.8	10.6	0.7	2.4	4.6	13.3	1.9	11.9
ZNF395	19.2	7.3	8.9	2.1	3.4	6.1	11.9	2.0	9.1
PDS5A	18.4	7.9	8.5	0.7	2.5	3.2	14.7	1.9	12.3
PLSCR4	25.2	10.0	12.2	0.6	2.9	3.9	7.9	0.8	6.6
ANKRD13B	18.8	7.4	9.9	2.4	3.3	4.9	11.7	2.4	9.3
UTP18	18.1	6.4	7.6	0.9	2.6	3.1	18.7	1.8	10.8

WWP2		20.1	6.7	10.8	1.6	3.2	4.3	14.4	1.4	7.5
USP5		21.3	7.7	10.6	1.9	4.1	6.0	10.0	1.2	7.3
HAGH		18.1	6.6	10.2	2.2	4.3	5.0	12.8	1.6	9.1
HINFP		19.5	7.5	9.6	1.4	2.3	3.3	13.6	2.2	10.6
GNL3		13.3	5.1	6.8	0.8	2.8	4.1	19.1	2.7	15.3
MBNL2		19.2	7.1	9.0	1.1	3.0	3.8	12.7	2.4	11.7
NFYC		18.1	7.6	9.2	1.7	3.3	5.0	11.0	2.2	11.6
PSPH		16.1	7.0	9.2	1.5	3.6	4.5	13.7	2.3	12.0
NXT1		17.2	7.7	10.1	1.7	3.0	5.3	11.5	2.3	11.2
KIF5B		17.1	6.1	7.6	0.8	2.8	3.7	16.1	2.2	13.5
NISCH		21.1	7.7	10.9	1.9	3.4	5.0	9.6	2.1	8.2
OSBPL2		23.1	7.7	9.0	1.4	2.8	3.2	12.6	2.1	7.9
ZNF706		19.3	8.3	9.7	1.0	3.1	4.5	10.4	2.3	11.3
PGF		24.0	8.5	12.7	1.9	2.8	5.0	8.7	1.0	5.1
GUCD1		20.1	8.9	10.3	1.8	3.5	5.1	10.8	1.8	7.3
MRPS27		17.4	6.4	8.3	1.0	3.0	3.5	15.0	2.5	12.5
HMGN4		19.5	5.8	9.0	0.7	2.4	2.9	14.8	2.0	12.6
UBASH3B		14.2	4.5	6.4	2.3	4.6	6.4	16.6	2.4	12.3
ZAK		17.7	7.0	8.3	0.5	2.5	3.2	15.3	2.2	12.9
SLC39A3		18.2	7.5	9.0	2.1	3.4	4.5	13.2	2.5	9.2
SNORA38B		13.5	8.0	8.2	1.2	4.6	3.1	21.6	1.7	7.5
	44811.0	18.9	7.4	8.9	0.6	3.0	3.4	14.9	1.6	11.0
TSTA3		16.5	6.5	9.1	1.9	4.6	5.8	13.3	1.7	10.1
RPH3AL		20.8	8.0	10.7	1.7	3.5	4.0	10.9	2.0	7.7
ACD		16.0	6.7	8.6	2.0	3.3	4.7	13.3	2.6	12.2
RAI1		17.8	6.5	8.3	2.1	3.0	4.1	12.7	2.7	12.2
THBD		19.7	8.4	9.6	3.0	4.3	5.1	11.1	1.8	6.4
MRPL21		18.4	6.8	9.1	1.5	3.5	5.3	12.4	1.6	10.6
DANCR		18.4	7.4	9.7	1.0	2.4	3.9	12.8	2.4	11.2
ZER1		21.2	8.0	10.3	1.7	2.8	4.8	10.2	1.9	8.4
ZC3H4		18.2	7.4	8.5	2.3	3.8	5.2	11.3	2.4	10.0
CCDC137		18.5	7.6	9.5	1.4	3.4	5.1	13.1	1.9	8.7
C20orf112		18.3	7.1	9.2	1.6	3.2	4.2	10.8	3.0	11.8
CCDC12		17.5	8.4	9.9	1.8	3.2	3.8	12.1	1.7	10.7
SMN2		16.2	6.2	8.6	1.0	2.8	3.5	15.1	2.7	13.2
OSBP		18.5	6.3	7.9	1.0	2.7	4.0	14.8	2.1	11.8
UBE2H		23.9	7.0	8.9	1.0	2.7	4.4	11.2	1.8	8.2
	44815.0	16.7	6.3	7.9	0.8	3.0	4.4	15.2	2.5	12.4
CANT1		18.2	7.0	8.8	1.9	3.5	4.7	12.9	2.2	10.0
EGLN1		17.8	6.2	7.8	2.4	3.3	4.3	14.0	3.1	10.3
DNAJC9		16.7	6.0	6.9	1.5	3.6	3.2	17.3	2.2	11.8
PPM1F		18.9	6.5	8.7	2.7	5.2	6.6	11.5	1.9	7.0
TLE1		18.4	7.0	9.1	2.0	3.9	6.0	10.8	2.4	9.5
RPUSD4		18.7	7.0	8.6	1.4	3.2	4.0	13.6	2.2	10.4
IFT52		18.9	6.1	9.3	0.7	3.0	3.5	15.8	1.8	10.0
GNPDA1		17.9	7.7	10.5	1.0	3.1	4.0	12.5	1.7	10.8
GPATCH2L		18.9	6.4	9.3	0.9	2.9	4.5	14.9	1.6	9.6
LOC100131564		22.3	4.8	9.7	1.2	2.7	3.3	16.3	1.3	7.4
JMJD8		18.0	7.3	8.2	2.4	4.0	5.1	11.9	2.4	9.7
SURF2		14.5	5.1	7.9	2.5	4.0	6.3	14.7	2.3	11.6
LOC101929165		22.7	4.3	9.7	1.2	2.6	2.8	17.2	1.3	7.1
TIMM8B		18.1	6.6	10.3	1.0	3.4	3.5	14.5	1.7	9.9
TYK2		20.7	8.3	11.4	2.2	3.8	5.6	8.8	1.4	6.8
CMPK1		20.0	6.8	9.8	1.7	3.3	4.0	12.2	1.7	9.4
OTUD1		19.7	7.6	9.7	2.3	3.3	4.1	11.0	2.7	8.4

SMYD3		15.6	6.0	8.4	1.2	3.3	4.0	17.5	1.8	10.8
RAB4B-EGLN2		20.4	7.6	9.6	2.1	3.4	5.3	11.7	1.8	6.9
SLC25A24		17.5	6.9	7.8	0.9	2.5	3.2	16.2	2.2	11.7
UPP1		16.3	5.8	9.1	0.8	2.2	3.3	17.7	2.7	10.9
ATP6V0B		16.3	6.8	11.7	1.1	2.8	4.9	12.5	1.7	11.0
ITCH		17.8	7.0	9.4	0.8	2.7	3.4	13.0	2.3	12.3
PRKDC		18.4	4.5	7.4	1.2	3.4	4.2	16.4	1.9	11.3
CD96		19.4	8.6	11.6	0.9	3.4	4.7	9.2	1.7	9.3
SP1		18.8	7.4	9.6	1.5	3.9	5.8	8.2	1.9	11.5
ABT1		17.9	7.1	10.1	1.5	3.5	3.3	12.8	2.3	10.3
PHACTR2		20.3	7.5	9.7	1.0	2.5	3.9	10.7	2.3	10.9
SDF2L1		12.1	4.4	6.3	1.1	2.4	2.8	23.6	3.4	12.5
GAS2L1		19.7	8.0	10.5	2.6	4.2	5.4	9.6	1.5	7.2
FAM134C		19.5	7.2	10.5	1.0	2.8	3.8	13.7	1.7	8.4
IRS2		15.2	5.0	6.6	4.0	4.7	6.1	12.7	3.5	10.8
ZNF592		19.4	7.0	8.9	1.7	3.4	4.4	11.3	2.2	10.2
IVD		17.3	6.7	8.9	1.3	3.1	4.3	14.5	2.4	10.2
TXN2		15.7	7.9	9.3	2.5	4.9	7.2	9.6	2.1	9.4
SEC23B		16.5	6.9	8.4	1.0	2.4	4.1	14.5	2.2	12.6
GLO1		16.7	6.3	8.8	0.4	2.7	3.6	17.2	2.1	10.7
EGR1		15.8	6.8	10.6	2.9	3.5	6.1	10.8	2.4	9.6
ACO1		19.8	6.7	9.0	1.0	3.6	5.0	12.9	1.6	8.9
UBR4		19.2	5.5	8.3	1.3	3.2	3.9	14.8	1.9	10.3
GNPTG		20.0	6.0	9.2	3.3	5.0	7.2	9.8	1.5	6.4
TRIP12		17.8	5.8	7.9	0.8	2.3	3.7	15.7	2.0	12.5
FASTK		19.7	7.6	9.7	2.3	4.1	5.3	11.0	1.6	7.0
ATXN7L3		17.8	7.3	8.9	1.2	2.2	3.9	13.2	1.9	12.0
LOC101928501		24.9	4.3	9.0	1.5	2.4	3.5	14.5	1.0	7.3
	44626.0	19.7	6.4	9.3	1.2	3.0	4.1	13.8	1.5	9.4
TMEM165		18.8	6.5	9.7	1.5	3.4	4.7	12.3	1.8	9.6
DDX49		15.9	6.7	8.3	2.0	3.3	4.9	13.7	2.4	11.2
SIKE1		15.6	6.6	9.1	1.1	2.8	4.3	14.3	2.2	12.3
COA1		19.4	5.5	10.1	0.9	3.0	3.3	16.1	1.2	8.7
TMEM115		19.1	6.9	9.5	1.5	2.8	4.7	13.2	2.1	8.5
MTG2		17.5	6.4	8.5	1.6	2.9	3.8	15.2	2.5	9.8
PPP1R11		15.8	7.5	7.5	2.2	5.4	8.6	9.1	2.4	9.5
SDHAF2		16.5	7.1	10.0	0.5	2.1	5.1	12.9	2.6	11.2
ASAH1		24.6	9.2	11.2	1.3	2.7	4.6	8.1	1.0	5.4
CDK9		16.2	7.2	7.7	2.6	5.0	6.2	11.5	2.2	9.6
VAMP2		15.2	7.1	9.7	2.5	5.0	6.7	8.9	2.7	10.2
RPS6KA4		17.6	6.4	8.3	1.9	3.6	4.1	14.1	2.2	9.8
LOC101927903		16.8	5.6	9.2	2.4	4.4	5.7	14.0	1.5	8.6
NAA60		18.5	7.7	9.5	2.1	2.5	3.2	11.5	2.0	11.0
SNORD29		11.6	5.8	7.6	5.8	8.3	6.5	9.1	4.9	8.5
STT3B		17.1	6.7	9.5	0.9	2.7	3.6	14.1	1.6	11.8
C16orf72		16.1	6.3	6.6	1.7	2.7	4.1	16.1	3.2	11.1
ATF6		19.1	7.2	9.2	0.9	3.2	4.5	11.3	2.0	10.7
TPM1		14.0	6.2	8.3	2.1	4.5	5.9	15.2	2.4	9.5
NAA30		17.0	6.4	7.4	2.5	3.4	4.9	14.1	2.5	9.7
AGAP1		18.9	5.4	7.4	1.3	2.7	4.2	16.0	1.6	10.3
UBTF		18.4	6.6	8.3	1.8	3.2	4.4	12.6	2.3	10.5
ATXN2		17.8	6.6	8.7	2.3	4.1	5.5	10.2	2.2	10.5
YIF1B		16.7	6.9	9.3	2.8	5.5	7.3	10.5	1.6	7.3
ETNK1		17.9	6.5	10.4	1.6	3.3	4.3	12.7	2.4	8.7
EID2		16.7	6.1	7.7	1.7	3.3	4.3	15.5	3.1	9.3

C1orf198	17.0	6.4	7.7	1.2	2.5	2.9	15.2	2.5	12.5
PDZD11	20.2	8.5	11.6	1.1	4.2	5.1	8.8	1.4	6.8
HEXA	21.8	8.7	11.4	2.2	4.7	6.8	6.2	1.0	4.9
ADAT1	20.9	4.7	9.4	1.1	2.5	3.1	16.9	1.4	7.8
MEPCE	15.3	6.4	7.2	2.3	3.5	5.2	13.7	2.8	11.3
DGAT1	19.1	7.2	10.8	2.6	4.2	5.9	9.5	1.5	6.7
PPP1CB	16.2	6.9	8.0	0.6	2.6	3.3	17.0	2.0	11.1
PPARG	20.0	7.1	9.8	1.0	4.1	5.5	10.6	1.6	8.1
LUC7L	21.5	7.0	9.7	1.8	3.3	3.9	11.5	1.6	7.5
IFI27L2	15.7	8.1	10.6	2.9	6.9	7.2	8.2	1.7	6.2
PFDN1	16.7	8.5	10.9	0.5	3.5	3.5	9.4	2.2	12.5
TAF10	13.0	5.3	6.4	2.6	4.8	5.4	16.5	2.7	10.6
GGT5	20.0	8.0	10.9	2.8	6.1	7.2	6.6	0.8	5.1
DCTN3	19.9	8.8	10.6	1.4	3.2	4.2	9.7	1.5	8.3
TMEM30A	20.5	8.0	10.2	0.6	2.8	4.3	10.2	1.8	9.0
UXT	16.1	8.8	11.3	1.5	3.8	5.2	9.3	1.6	9.8
POLDIP3	18.3	7.1	8.7	1.5	2.8	4.5	12.3	2.2	10.1
ITGA6	19.9	7.7	9.2	1.2	3.3	5.4	10.0	1.3	9.4
RAB2B	19.7	5.9	9.5	0.7	2.9	3.0	15.1	2.0	8.6
RNPEP	21.8	7.7	9.9	1.7	2.7	4.8	10.6	1.3	7.0
PPP1R2	16.5	7.4	8.0	1.0	3.4	3.5	13.3	2.4	11.8
GPBP1L1	15.7	5.8	7.8	0.8	2.5	3.3	16.5	2.1	12.7
RASSF3	17.5	6.7	8.8	1.2	2.2	4.6	12.9	1.8	11.7
PDCD11	15.4	5.5	7.2	1.1	2.5	3.5	17.0	2.2	12.8
YTHDF2	16.0	6.6	8.7	0.8	2.6	3.7	13.6	2.6	12.7
HNRNPA1L2	18.7	6.1	8.2	0.6	2.4	3.2	15.9	1.8	10.4
C4orf32	17.1	6.7	8.3	2.1	2.3	3.9	14.3	2.6	9.8
SLC39A13	15.6	6.3	8.2	2.1	4.4	6.0	12.9	2.3	9.5
WWC1	15.8	7.6	7.9	1.3	2.6	3.6	15.2	2.1	11.1
IFI16	18.9	7.7	9.2	1.2	3.6	4.7	9.6	2.1	10.0
EPS15L1	19.0	6.9	8.7	1.7	3.2	4.1	11.9	2.2	9.5
LSM3	16.6	6.4	10.4	1.0	4.4	3.4	11.9	1.5	11.4
FBXL12	20.1	7.2	10.0	2.3	3.1	5.1	10.4	2.2	6.7
PCSK9	26.8	10.3	13.4	0.6	1.2	1.5	7.6	1.2	4.5
CECR5	18.8	7.3	9.6	2.0	3.0	3.8	11.9	1.9	8.6
TNFAIP1	15.9	7.7	7.9	1.6	2.9	4.9	12.2	2.7	11.3
TBCD	21.2	7.4	9.6	2.3	4.4	5.7	9.0	1.2	6.2
DDT	30.6	13.2	2.7	0.4	7.9	1.3	3.1	4.7	3.1
PELO	16.6	5.7	8.3	1.3	2.5	3.7	14.7	2.2	11.9
LPPR2	19.1	9.1	9.8	3.1	3.9	5.4	8.2	1.7	6.6
DPH2	16.6	6.9	8.3	0.9	2.6	3.1	16.5	2.0	9.9
MRTO4	15.7	6.5	8.0	1.4	3.3	3.8	13.5	2.4	12.2
KLHL29	18.6	6.7	9.4	2.7	3.7	4.8	10.2	2.1	8.7
MKRN1	18.9	8.0	8.2	1.2	2.9	3.4	12.3	2.3	9.6
PPP3R1	16.6	7.0	7.8	0.2	1.8	2.6	15.9	1.7	13.1
UQCRC2	15.4	5.8	8.3	0.8	3.3	4.1	15.1	1.9	12.0
PPA2	16.9	6.8	9.0	2.4	4.6	6.9	10.5	1.8	7.9
AKIRIN1	16.6	6.0	8.1	1.6	3.0	3.9	13.6	3.2	10.6
SNX21	16.5	7.0	8.3	2.7	5.9	7.0	10.8	1.3	7.3
MRPL33	18.4	8.1	12.0	0.5	1.5	2.6	12.0	1.5	10.0
C11orf80	20.3	6.4	9.4	1.3	1.9	3.4	14.9	1.4	7.8
SFT2D1	17.7	7.4	9.9	0.8	1.6	3.8	13.9	1.2	10.4
FAM168A	19.5	7.5	9.1	2.1	4.4	5.3	7.0	2.8	9.1
EMD	15.6	7.0	8.2	1.6	4.3	5.2	11.8	2.4	10.7
KANSL3	18.8	7.0	9.0	1.3	4.0	4.0	12.2	1.9	8.5

MRPL47	19.2	6.6	10.3	0.4	3.0	3.6	12.9	1.6	9.0
SNX9	21.4	7.0	9.5	0.7	2.5	3.1	11.9	1.8	8.7
MARK4	19.8	7.6	8.8	2.3	3.5	5.2	9.4	1.9	8.2
ATL3	19.9	6.9	9.3	0.7	2.6	3.6	12.5	1.3	9.7
LOC100289019	20.6	4.7	9.3	1.3	2.7	3.1	15.5	1.4	8.1
DENND4B	19.2	7.9	10.3	1.6	3.1	4.3	10.1	1.9	8.3
RNF214	17.9	6.3	8.7	1.2	2.7	4.2	11.8	2.4	11.4
SAP30BP	19.4	7.9	8.0	1.2	2.4	5.1	14.3	1.6	6.6
ZNF444	16.0	6.4	8.7	2.7	3.9	5.0	13.6	2.6	7.6
GNE	20.6	7.5	10.0	1.0	2.8	4.1	11.2	1.3	8.1
RELB	22.8	7.6	10.1	2.2	3.4	5.1	8.6	1.7	4.9
UBE2B	16.8	8.5	8.2	1.3	2.7	4.3	10.1	2.6	12.2
EMC1	19.6	6.3	8.9	1.0	2.6	3.4	13.5	1.8	9.3
ATG13	15.1	5.9	6.4	1.0	2.7	4.0	15.9	2.5	13.0
WASL	18.6	7.8	9.2	2.0	3.0	4.9	8.9	2.1	10.1
PLA2G15	22.2	8.5	11.0	1.9	3.1	4.5	8.3	1.5	5.4
TBL3	15.8	5.6	7.0	2.6	4.1	5.6	14.1	2.3	9.3
CWC25	20.1	5.4	9.5	1.1	2.5	2.7	14.6	1.8	8.7
TBCC	15.1	6.6	7.7	1.4	3.2	3.9	14.7	2.2	11.7
OVCA2	19.3	8.0	10.7	1.8	3.5	4.3	8.7	1.5	8.6
MRPS15	16.0	7.5	10.2	1.1	3.9	4.5	10.5	1.9	10.7
SNORA78	12.7	6.0	8.9	2.9	5.0	5.5	14.5	1.9	9.0
OSBPL8	19.8	6.5	9.2	0.6	2.3	3.1	12.5	1.5	11.0
ELOVL5	18.3	7.8	9.0	0.8	2.6	4.3	9.9	2.4	11.3
SMARCD2	18.8	6.8	8.8	1.4	3.6	4.5	11.1	2.0	9.4
ZNF317	18.2	6.1	8.4	1.4	2.9	3.9	12.4	2.1	10.9
CENPT	17.8	7.4	11.4	1.8	3.7	5.2	9.0	1.7	8.3
ACOT8	19.0	7.2	11.1	2.0	4.2	5.4	9.5	1.5	6.5
CHKA	17.3	6.7	7.9	1.5	2.6	3.4	13.2	2.1	11.6
PRKD2	19.7	7.4	8.8	1.9	2.9	4.5	10.6	2.3	8.1
NCOR1	17.9	7.1	9.3	0.9	2.9	4.5	11.2	2.0	10.4
ZGPAT	19.0	7.5	9.7	1.6	3.0	3.8	10.8	1.7	9.0
LOC550643	16.8	7.5	7.9	0.9	2.5	3.9	13.9	2.3	10.3
TTC1	17.9	8.9	11.1	0.6	3.7	3.4	10.6	1.5	8.5
XIAP	20.7	4.7	8.9	1.0	2.3	2.9	16.1	1.2	8.3
LOC730268	17.6	6.0	9.1	0.8	1.0	1.0	16.9	2.0	11.7
LIF	17.0	6.6	10.6	3.1	5.1	6.7	7.4	1.6	8.0
LRRC24	18.0	7.8	9.3	2.9	3.3	5.9	10.8	1.5	6.5
OCIAD1	17.6	7.8	9.0	0.5	3.1	4.1	10.5	1.4	12.1
UBFD1	18.9	6.0	8.1	1.5	2.9	3.5	14.3	1.8	9.0
ZNHIT6	15.4	5.6	7.2	1.1	2.9	4.2	15.1	2.1	12.5
BCAM	21.5	8.9	11.7	2.2	3.7	4.6	6.7	1.2	5.4
DIDO1	18.0	5.1	8.0	1.7	3.3	4.7	12.9	1.9	10.2
ATP6V0A1	16.4	5.9	8.4	0.7	2.1	2.5	16.1	2.2	11.5
MEX3C	15.0	7.0	8.2	2.9	3.3	3.0	13.1	2.9	10.4
CDR2L	21.2	7.6	9.8	1.6	2.0	3.3	12.0	1.9	6.4
SIK2	18.2	6.1	8.6	1.7	3.2	5.0	10.9	2.5	9.6
RAI14	15.0	5.5	7.2	1.2	3.0	4.3	14.1	2.2	13.2
ANAPC2	19.9	7.2	10.6	2.0	3.2	4.2	9.9	1.7	7.1
MAN2B1	18.1	7.0	9.6	1.8	3.8	5.4	10.6	1.8	7.6
COPB2	18.1	7.1	8.4	0.6	2.6	3.2	13.8	1.6	10.2
IDH3A	17.6	6.5	9.2	0.7	2.6	3.6	13.5	1.6	10.3
LOC101928686	17.3	6.7	7.3	1.9	3.3	3.7	13.4	2.4	9.5
LOC286437	22.8	4.2	9.8	0.8	3.0	3.3	13.0	1.1	7.7
PAQR7	16.8	6.5	7.7	1.9	4.1	5.1	11.5	2.1	9.9

PACSIN2	18.9	6.9	8.6	2.1	4.2	4.8	10.1	2.0	8.1
ZNF703	17.6	7.5	8.5	2.3	3.0	5.1	10.3	2.7	8.7
IARS2	18.5	6.5	8.9	0.6	2.0	3.1	14.9	1.4	9.8
PCNXL3	18.0	6.7	9.2	2.1	3.6	5.0	10.9	2.0	8.1
RPF1	16.0	7.5	9.0	0.9	3.4	4.3	12.0	2.2	10.4
CDC42EP4	19.4	7.2	9.2	1.7	3.2	4.6	10.1	1.9	8.2
LOC101929360	21.1	4.6	8.3	2.0	3.1	3.0	15.5	1.1	6.7
THOC5	17.7	6.5	8.6	1.1	3.1	3.7	12.7	2.0	10.0
COPZ1	17.3	7.3	9.3	0.5	2.5	3.5	12.9	1.5	10.7
AKIRIN2	16.0	5.9	7.0	1.6	2.3	4.3	13.0	2.8	12.7
SH3BGRL	20.0	7.6	11.4	0.3	2.6	4.6	10.9	1.4	6.7
CLEC4C	21.4	4.1	9.3	1.2	2.3	3.0	16.1	1.3	6.7
MRPS18A	14.0	7.0	8.6	1.7	5.0	6.1	11.7	2.1	9.2
ECT2	15.6	6.5	8.9	0.4	2.3	2.6	14.0	1.9	13.2
NEK9	20.5	7.3	10.9	1.5	3.7	4.4	9.2	1.2	6.7
NFAT5	18.2	5.2	7.8	1.0	3.1	3.7	12.0	2.1	12.4
MCOLN1	17.8	7.6	8.8	1.8	3.4	4.8	9.5	2.1	9.5
LOC100506385	21.3	4.9	8.2	1.5	2.5	2.9	15.8	1.5	6.7
CHURC1	16.8	7.9	9.8	1.0	3.5	3.8	14.5	1.5	6.6
ZIC2	14.6	4.9	7.0	4.2	5.9	7.4	9.6	2.7	9.0
BBC3	18.8	7.5	8.7	2.1	4.6	4.4	9.8	1.6	7.9
MED10	16.9	7.3	7.7	0.9	1.5	2.9	14.2	1.9	12.1
MED16	16.3	6.1	7.1	2.7	3.7	5.9	12.4	2.0	9.1
SLC25A46	18.6	6.9	10.6	0.9	2.9	3.1	12.2	1.3	8.8
RANBP9	13.7	8.4	7.3	5.9	3.7	3.0	9.8	4.6	8.9
SUZ12	16.9	6.1	7.9	1.3	3.2	3.8	13.7	2.0	10.3
SRC	18.9	7.0	8.7	2.3	3.0	4.3	10.8	2.1	8.1
GATM-AS1	20.5	3.9	9.4	1.5	2.4	3.2	15.6	1.3	7.4
OSGEP	16.0	7.0	9.3	1.6	3.3	4.5	13.8	1.4	8.4
RGCC	13.5	4.6	8.1	1.2	1.6	2.6	18.3	3.7	11.7
SRGAP2B	17.6	6.4	9.1	1.4	3.2	4.0	11.9	1.9	9.5
PURB	17.0	5.8	8.4	2.7	3.9	4.2	12.5	1.8	8.8
MRPL54	17.3	7.5	11.5	1.4	2.9	4.4	12.0	1.4	6.6
BRAT1	19.0	8.1	10.6	1.9	3.2	4.4	9.9	1.9	6.0
PRPF3	16.5	5.9	8.4	1.0	2.3	3.5	13.4	2.1	11.9
VPS25	17.7	7.5	9.6	1.5	4.2	5.2	9.1	1.5	8.8
TAP2	14.7	5.5	7.0	1.4	3.1	4.4	15.8	2.4	10.7
AP2A2	20.3	7.0	9.1	2.2	4.1	5.1	9.0	1.7	6.5
SMARCA5-AS1	17.9	5.8	7.9	3.1	3.2	4.9	10.3	2.6	9.2
ZBTB44	20.8	6.1	9.0	0.6	2.2	3.5	11.4	1.5	9.9
GMFB	17.6	6.5	9.7	0.5	1.2	2.9	14.6	1.1	10.8
MED13L	17.5	5.8	7.2	0.9	2.2	3.2	14.1	2.0	12.0
CDKN1B	17.3	7.6	9.8	1.6	3.7	4.6	10.0	2.0	8.3
KIAA0907	17.2	7.2	8.8	0.8	2.6	3.2	12.0	1.8	11.4
FCF1	19.8	5.6	9.3	0.8	2.4	3.5	13.7	1.4	8.4
CAMSAP1	16.1	5.4	7.3	1.6	2.9	4.0	13.6	2.5	11.4
PPP5D1	22.0	4.5	9.1	1.5	2.6	3.1	14.4	1.2	6.4
SNX2	18.1	7.4	8.9	1.0	3.0	4.1	11.4	1.5	9.3
GGA1	18.0	7.9	10.1	1.6	4.0	5.0	8.1	1.9	8.2
PITPNB	17.1	6.4	8.4	0.5	2.4	3.0	12.5	2.0	12.5
SGK223	21.7	5.6	8.4	2.4	4.9	6.1	8.9	1.1	5.8
LOC101928291	17.7	5.6	7.2	2.8	3.0	3.7	14.1	2.4	8.3
AGPAT5	15.5	5.9	7.7	1.8	2.9	3.7	13.8	2.4	11.0
SH3BP2	17.9	7.0	9.4	2.5	3.4	5.1	9.8	2.1	7.6
SYNJ2	23.6	7.7	11.1	1.6	3.5	4.7	6.6	0.9	5.1

SEC11C	14.4	7.1	8.0	0.6	2.3	4.2	12.5	2.6	13.1
COMMD4	17.5	6.8	10.1	1.9	3.5	3.6	12.7	1.3	7.5
GSTK1	17.8	7.2	10.1	1.7	3.4	5.0	11.4	1.2	7.0
EIF5B	13.5	5.0	6.0	0.6	2.6	3.4	16.9	2.0	14.5
COPB1	16.4	5.5	8.2	0.4	1.8	2.6	15.6	2.0	12.2
LIN7C	15.8	7.1	8.7	1.1	3.1	4.6	10.9	2.2	11.2
SKI	18.0	6.0	7.5	2.4	3.6	4.2	11.6	2.2	9.2
CRCP	17.6	5.8	7.9	1.1	2.4	3.2	14.3	2.0	10.3
SETD1B	17.2	6.3	8.4	2.5	3.6	5.0	10.2	2.3	9.3
GFPT1	16.1	5.2	7.2	0.4	1.5	2.3	18.3	1.9	11.6
SMARCC1	15.7	5.9	7.3	1.4	3.0	4.0	12.8	2.3	12.1
ERO1L	17.3	5.9	8.1	0.3	1.8	2.2	15.6	1.6	11.8
SDHC	17.8	7.2	9.7	1.1	3.0	4.0	11.0	1.9	8.8
GPAA1	15.5	6.3	9.2	2.9	5.3	7.1	10.1	1.7	6.3
EIF4E	16.5	5.8	8.0	0.8	1.8	3.0	13.8	2.0	12.7
MAN2A1	16.7	5.5	7.5	0.7	2.3	3.6	15.9	1.8	10.4
LOC101927447	19.4	4.2	9.4	0.7	2.0	2.7	17.3	1.4	7.4
SRSF8	17.6	5.5	8.1	1.5	2.6	3.6	14.6	1.9	9.1
THOP1	16.8	6.3	7.9	1.8	3.1	4.4	13.2	1.9	8.9
DHX34	16.8	6.4	8.2	1.9	3.3	4.2	12.5	2.3	8.8
ATF6B	16.8	6.2	8.9	2.8	5.2	7.1	8.1	1.6	7.7
YME1L1	17.0	7.0	7.9	0.4	2.0	2.6	14.4	1.6	11.4
INTS1	18.4	6.3	8.7	2.5	4.2	5.6	10.0	1.7	7.0
MRPL37	16.8	5.9	7.8	1.2	3.0	4.8	12.4	2.3	10.1
LUC7L3	16.8	6.8	9.2	0.8	2.6	3.7	12.0	1.6	10.6
KIFC2	18.2	8.2	11.4	2.7	3.8	5.9	7.0	1.3	5.8
AHCYL1	18.1	7.5	9.2	1.3	3.6	5.0	9.7	1.6	8.1
ARHGAP1	19.3	7.5	8.6	2.4	3.5	6.4	7.3	1.6	7.6
TAB2	15.2	5.8	8.1	0.9	2.2	3.6	11.9	2.4	14.0
NUAK1	12.5	4.9	6.2	1.2	2.2	3.1	17.5	3.1	13.4
EHMT1	19.0	6.0	8.4	2.0	3.4	4.3	11.4	2.0	7.8
SLC6A9	16.5	6.6	8.4	1.5	3.6	5.2	10.2	2.5	9.6
ERBB2IP	15.7	5.8	7.4	0.4	2.1	3.0	13.9	1.9	14.0
FDX1	16.9	6.9	9.0	1.8	3.0	4.1	12.4	1.9	8.1
SMIM12	14.8	7.1	8.0	1.3	2.5	3.9	12.1	2.1	12.4
ARHGAP39	14.3	5.5	6.8	1.9	2.7	3.8	15.3	2.8	10.9
TOR1AIP1	17.1	6.2	8.4	0.9	2.7	3.8	13.0	2.0	10.1
NR2F1	22.8	7.3	10.6	1.9	3.0	4.6	7.2	1.6	5.0
ANKRD10	16.1	6.4	9.3	1.5	2.7	3.7	12.5	2.0	9.8
LPHN2	19.1	7.2	8.9	0.5	2.5	3.3	11.0	1.5	9.9
GALC	21.3	8.8	10.2	1.2	3.0	4.1	7.8	1.2	6.3
LATS2	16.1	5.9	7.6	1.8	2.9	3.8	14.1	2.7	9.1
MAP3K9	19.6	6.6	9.8	1.5	2.5	3.4	10.6	2.0	8.0
PRR12	17.2	5.9	8.3	2.9	3.7	5.1	10.2	2.3	8.4
MACF1	18.1	4.8	7.7	0.7	2.5	2.9	15.6	1.7	10.1
MPP5	15.0	5.7	6.9	0.6	2.4	3.1	14.5	2.3	13.4
C14orf23	20.6	4.6	10.0	1.1	2.3	2.7	14.2	1.3	7.2
SIAH2	13.4	4.7	5.7	1.4	2.3	3.7	17.1	2.8	12.6
TSSC1	15.4	5.4	7.8	1.2	3.0	4.1	14.7	2.0	10.2
DCUN1D2	19.0	4.3	9.1	0.9	2.3	2.9	16.0	1.5	7.7
FHL2	16.2	6.3	7.6	1.0	3.4	4.0	14.6	1.8	8.8
CISD3	13.3	6.0	7.4	2.5	5.2	7.4	11.4	2.4	8.1
LEPRE1	18.2	5.9	8.9	1.6	3.2	3.8	12.2	1.6	8.2
SNHG10	15.9	5.5	10.1	1.3	2.1	3.4	16.6	0.9	7.9
AGPAT6	17.9	6.9	9.3	1.0	2.6	3.8	11.8	1.4	9.1

C11orf1	17.3	7.3	7.9	1.8	4.0	6.6	8.1	2.4	8.3
SEC23A	19.3	7.0	8.8	0.6	2.2	3.4	11.2	1.4	9.9
ICAM2	16.6	6.4	9.3	1.3	3.0	4.2	13.1	2.1	7.8
RYK	17.0	7.3	8.8	1.1	2.8	4.1	10.8	1.8	10.0
SFN	19.4	7.7	9.7	1.1	3.2	3.8	9.5	1.9	7.2
RPA1	16.0	6.1	7.5	1.2	3.3	4.0	13.1	1.8	10.6
MED19	17.5	6.4	8.7	1.3	3.1	4.3	11.1	1.8	9.4
ARRB2	17.0	6.6	9.0	1.1	3.0	3.7	11.3	2.2	9.8
SMAGP	14.3	5.7	7.0	1.0	2.6	5.1	12.6	2.6	12.7
TOX4	17.2	7.2	8.5	1.3	2.7	4.8	9.5	2.1	10.2
ITPA	16.2	6.3	8.9	1.2	3.0	4.3	13.0	1.2	9.5
MTCH2	14.4	6.9	8.4	1.0	2.8	3.6	15.1	1.5	10.0
KDM5C	18.0	7.0	8.8	1.3	2.5	3.3	11.7	2.1	8.7
HIST1H2AI	11.5	4.2	8.8	2.7	5.2	6.7	13.8	1.9	8.7
ZMAT2	16.4	6.9	8.6	1.1	2.8	4.1	10.9	1.4	11.2
NDRG3	18.5	6.1	8.1	0.8	2.4	3.8	12.0	1.6	10.1
PDXDC1	18.8	7.5	8.8	1.0	2.4	3.1	11.4	1.5	8.9
DNAJA3	16.4	7.1	8.1	1.5	3.1	4.5	12.8	1.5	8.5
RAP1B	17.4	6.3	10.0	0.5	2.6	3.1	12.2	2.1	9.2
ANKLE2	18.0	5.5	8.5	1.1	2.6	2.7	13.6	2.0	9.4
PIM1	13.6	5.0	6.2	3.0	4.7	5.4	11.8	2.7	10.9
KIAA0930	19.6	6.6	8.9	1.6	3.2	4.4	10.1	2.0	7.0
MCRS1	19.1	7.0	10.4	2.2	3.1	4.5	9.2	1.2	6.7
PPP1R37	19.0	7.4	9.6	1.7	3.5	3.9	9.1	1.7	7.5
NDUFB8	15.6	6.5	10.0	1.7	3.8	3.2	11.2	1.8	9.7
RNF168	18.3	6.2	8.5	0.9	3.1	3.6	11.7	1.7	9.5
PMPCA	15.4	6.2	8.8	1.2	3.4	3.9	14.2	1.9	8.2
PNKD	19.2	7.8	10.5	1.7	3.2	4.4	7.6	1.3	7.7
LOC100288162	19.3	5.8	10.5	2.0	3.3	4.0	10.4	1.4	6.6
ABHD11	19.5	4.7	8.8	1.2	2.6	3.0	16.0	1.1	6.3
MRPS36	18.9	8.1	9.9	0.7	3.0	3.5	8.6	2.7	7.9
TP53RK	12.8	6.8	7.5	2.1	2.6	3.7	15.4	2.6	9.7
SS18	15.6	6.4	7.3	1.6	3.0	5.4	9.9	2.9	11.0
SETD1A	17.5	6.4	8.5	1.9	3.0	3.9	10.0	2.5	9.4
HPS1	18.4	6.9	8.2	2.3	3.7	4.4	10.0	1.3	7.8
LYPLA2	15.7	7.3	9.3	1.7	3.4	3.7	11.4	1.8	8.9
LIMD2	12.6	7.6	11.5	1.9	3.9	4.5	8.6	2.3	10.1
NEURL1B	16.1	5.9	8.0	2.6	3.6	4.9	12.1	1.8	8.0
MOB3A	18.9	5.5	7.9	2.5	3.7	4.4	9.4	2.0	8.7
EML2	14.7	6.2	8.3	1.5	3.1	4.7	12.2	1.9	10.3
DDX6	15.9	5.2	7.2	0.8	3.1	3.8	12.9	1.9	12.2
ZC3H3	18.6	6.8	9.5	2.0	3.1	4.1	9.0	1.9	7.9
PPP1R26	16.2	5.7	7.2	1.7	3.0	4.3	12.3	2.2	10.3
MICAL2	15.1	5.1	6.1	1.5	3.8	5.1	13.9	2.0	10.3
SEC24B-AS1	22.6	3.7	7.9	1.4	2.6	3.3	15.4	1.1	4.9
CD33	16.2	6.9	9.2	1.8	3.7	5.0	8.5	2.2	9.4
TPRG1L	19.4	6.6	9.7	1.8	2.9	3.7	9.9	2.0	6.7
RER1	16.8	5.8	7.6	0.9	3.6	4.4	12.1	1.9	9.7
ATP2B1	16.9	5.9	8.3	0.4	2.3	3.2	14.3	1.4	9.9
MGST3	16.4	7.5	11.3	1.0	3.7	5.8	9.5	1.0	6.4
RNF5	16.9	6.6	9.3	0.9	3.9	4.1	11.7	1.4	8.0
NDUFB6	15.4	7.1	9.5	2.2	3.1	4.4	12.2	1.0	7.8
C9orf78	15.3	6.9	9.5	1.4	3.1	5.0	9.4	1.9	10.2
POP7	14.1	6.5	8.4	2.0	3.5	6.0	10.7	2.2	9.3
C14orf2	13.6	6.1	10.4	0.3	2.4	3.2	14.4	2.1	10.1

PURA	14.8	5.1	7.1	2.7	3.2	3.7	15.1	2.5	8.5
RAB43	23.2	6.9	8.6	1.4	2.1	4.5	9.1	1.2	5.7
PPP2R4	18.4	6.1	8.3	1.4	3.2	4.0	11.6	1.3	8.4
ARMC6	13.3	5.5	6.6	1.8	3.6	4.6	15.1	2.2	9.9
PRRC1	17.1	6.5	7.5	0.7	2.3	2.9	13.1	1.8	10.7
ACTR1B	16.0	6.1	9.2	2.3	4.9	7.0	9.3	1.3	6.5
SEMA4C	18.5	7.3	9.8	1.4	2.0	3.7	9.9	1.8	8.1
HIAT1	17.1	7.0	9.6	0.6	1.9	2.6	12.2	1.7	9.8
UBE4A	20.0	6.8	9.0	0.7	2.7	3.6	9.9	1.5	8.4
PI4KB	16.5	6.4	8.3	1.5	3.0	4.3	11.1	1.7	9.7
MED29	15.6	6.4	8.8	2.4	3.8	5.6	7.9	1.9	10.0
RBM22	15.1	6.2	8.8	0.7	2.3	3.1	11.9	2.7	11.5
DDX47	15.6	5.3	8.6	0.8	2.7	3.3	13.3	1.8	10.9
C10orf2	14.8	5.0	6.1	0.7	2.4	3.3	16.6	2.0	11.5
ACSS2	22.2	10.7	12.1	0.6	1.4	2.0	7.2	1.1	5.0
FKRP	19.6	7.8	10.6	1.6	2.8	4.0	8.8	1.3	5.8
PPAP2A	18.1	7.4	9.8	1.2	2.6	4.3	9.7	1.9	7.3
ACSF2	18.9	6.9	9.8	1.7	3.6	5.1	8.4	1.5	6.4
FDX1L	17.9	7.0	10.5	1.5	2.9	3.4	9.5	1.4	8.2
RTCB	14.9	5.4	8.3	1.0	2.4	3.3	15.4	1.9	9.7
PPP3CA	16.9	6.1	7.8	0.9	3.1	3.8	12.0	1.8	9.9
PROCR	11.5	6.3	8.3	1.9	4.3	4.5	12.3	2.2	11.1
XPO1	15.7	5.6	7.0	0.4	1.9	3.1	14.5	1.6	12.3
ZMYND11	16.3	7.1	7.3	0.9	2.5	3.6	11.5	2.0	11.0
TMOD3	15.4	5.8	6.5	0.3	2.2	2.5	15.5	1.7	12.4
PEX5	16.9	5.3	7.2	1.2	2.7	3.1	14.4	1.6	9.7
DPYD	16.4	5.6	7.9	0.6	2.9	3.2	15.6	1.1	8.9
MRPL35	17.4	6.6	9.9	0.6	2.0	4.1	11.3	1.8	8.3
MRPL55	15.3	6.2	10.5	2.2	4.0	4.1	9.5	1.7	8.4
S100A13	17.1	6.8	10.3	1.4	3.3	4.1	10.1	1.5	7.6
GTF2H5	16.3	6.0	11.3	0.4	2.7	3.6	11.7	2.0	8.1
SCO1	16.4	6.4	7.9	1.1	2.8	3.5	13.7	1.4	8.8
EFNA5	21.6	6.2	8.7	1.4	3.5	4.4	7.9	1.5	6.9
NIPAL3	15.8	6.1	7.4	1.4	3.2	4.3	13.0	1.6	9.3
TMEM9	13.9	6.8	10.8	1.1	2.9	3.3	12.0	1.5	9.8
MBD2	16.6	6.1	8.3	1.4	2.4	3.9	11.0	2.5	9.8
FLJ31356	20.6	4.1	8.8	1.3	2.4	2.5	14.6	1.2	6.5
RBM17	16.1	6.3	8.1	1.1	2.7	3.3	11.6	1.7	11.0
SNORD3C	17.5	5.8	7.8	3.8	2.4	1.3	14.5	2.0	6.8
ZMYM3	18.5	8.4	7.3	1.2	3.3	3.4	9.1	1.4	9.2
HSPG2	19.3	6.8	10.0	2.5	3.8	4.6	7.7	1.5	5.6
CUL4A	13.9	5.5	6.8	1.0	2.8	3.7	13.3	2.1	12.8
HIF1AN	16.8	6.3	8.8	0.9	3.0	3.5	11.8	1.6	9.2
RHBDF2	17.5	7.8	10.3	1.8	2.6	3.3	8.7	1.6	8.2
AFF1	17.1	5.4	7.1	1.3	2.5	3.6	11.9	2.3	10.6
FAM162A	12.5	6.1	8.5	0.6	2.5	3.5	15.3	1.9	10.9
SRPK1	14.4	5.5	7.2	0.9	2.5	3.3	14.2	1.9	11.8
CD2BP2	17.3	6.2	7.6	1.2	3.4	2.7	12.6	1.6	9.3
LOC101929450	20.0	4.1	8.1	1.2	2.5	3.0	14.7	1.4	6.8
TMED5	16.8	6.7	8.9	0.7	1.5	2.7	12.6	2.0	9.7
MAGT1	16.0	5.0	7.4	0.8	2.4	3.1	15.9	1.4	9.7
TRNE	19.3	9.2	6.6	2.1	3.7	5.3	7.5	1.2	7.0
ZNF460	16.8	4.2	8.6	1.5	3.1	3.2	12.6	1.9	9.8
WWC3	17.8	5.8	8.1	1.7	3.5	4.5	10.5	1.6	8.2
ITFG3	20.7	8.0	10.0	1.9	4.4	4.8	6.5	1.2	4.1

NUB1	14.2	5.5	7.3	1.0	2.8	3.6	13.6	2.0	11.6
RRS1	13.5	4.8	6.0	2.4	3.5	4.7	13.8	2.7	10.2
TMEM11	16.1	6.5	7.9	1.6	2.6	3.3	11.2	2.3	10.1
DALRD3	15.8	6.5	8.5	1.7	3.4	4.7	10.9	1.9	8.3
USF1	18.0	6.6	9.8	1.5	2.6	3.1	10.4	1.5	8.0
PPAP2C	18.7	7.1	10.3	1.2	2.9	3.2	10.1	1.5	6.4
YPEL5	18.9	5.5	8.2	0.8	2.0	3.6	13.3	1.3	7.9
NDEL1	16.7	6.0	7.6	0.9	1.5	3.2	13.2	1.8	10.5
KANSL1	16.3	6.0	7.5	1.3	2.5	3.8	12.4	1.9	9.8
MVK	19.5	8.7	11.0	1.0	1.3	2.0	9.5	1.6	6.7
FAM115A	21.5	7.2	9.5	1.2	3.2	3.8	8.1	1.3	5.6
ARHGEF26-AS1	21.1	4.2	9.4	0.6	2.7	3.0	12.2	0.9	7.3
ALDH9A1	16.4	6.9	8.5	1.1	2.9	3.5	11.8	1.4	8.9
C14orf1	17.5	7.1	9.0	1.0	3.1	3.7	10.5	1.4	8.2
ARHGEF11	17.1	5.7	7.9	1.2	2.4	3.3	10.4	2.2	11.0
LAMTOR4	16.5	7.3	10.1	2.3	3.9	4.0	8.5	1.9	6.8
NUP85	15.8	6.1	8.1	0.9	3.0	3.9	13.4	1.7	8.4
MRI1	16.9	5.5	9.1	1.6	2.9	4.1	12.7	1.5	6.8
MNT	16.3	6.1	7.9	3.4	4.2	5.8	7.4	2.4	7.8
ACAD8	18.2	6.5	9.4	1.3	2.6	3.3	12.6	1.3	6.1
NMNAT1	19.0	4.0	8.6	1.0	2.4	3.0	15.2	1.2	6.8
SLC25A38	15.0	5.3	6.3	0.9	1.9	2.9	16.3	2.1	10.6
CLCN3	16.9	6.6	8.4	0.6	2.3	3.3	11.2	1.7	10.3
FKBP14	17.8	4.1	8.1	0.7	1.8	2.4	16.4	1.3	8.7
LOC101928287	19.8	4.2	8.6	1.4	2.2	2.6	14.6	1.5	6.1
XPO6	14.5	5.3	6.6	1.3	3.0	4.2	14.3	2.1	10.0
CAD	12.9	4.3	6.4	1.8	3.7	4.8	15.1	2.0	10.0
TUBB3	20.0	7.1	9.8	1.7	3.2	3.8	6.9	2.0	6.6
NAT9	15.9	4.9	9.1	1.3	3.1	4.4	12.0	1.4	8.8
EIF4G3	18.5	4.6	8.8	1.2	2.2	4.2	10.7	1.5	9.5
NEK7	12.8	5.0	7.2	0.6	2.2	3.0	14.2	1.8	14.4
AKR7A2	16.7	6.7	8.5	2.2	3.5	5.4	10.2	1.3	6.5
PSMA2	17.7	7.3	9.1	0.5	2.9	3.0	10.1	1.2	9.1
MAPK12	16.5	6.2	9.4	2.3	3.8	5.4	9.0	1.8	6.6
TBC1D13	17.9	6.2	7.8	1.0	3.0	4.0	10.9	1.5	8.6
BCR	18.6	6.3	8.0	1.5	2.9	3.5	11.3	1.6	7.2
CAPN15	15.3	5.7	7.7	2.1	3.1	4.0	12.2	2.1	8.7
SASH1	17.1	5.2	6.9	1.1	2.3	3.3	13.5	2.2	9.2
LOC654433	16.3	4.4	8.2	1.3	2.8	3.2	14.9	1.5	8.3
GLYR1	15.3	6.3	6.9	1.4	2.2	4.2	13.6	1.8	9.2
LOC101929374	15.4	7.2	7.1	2.2	3.8	5.1	10.9	1.7	7.4
LOC101929493	11.6	4.4	6.2	0.8	2.0	2.4	18.4	2.2	12.6
ANKRD13D	18.3	7.4	9.2	1.9	3.6	4.2	7.9	1.8	6.3
SNX1	16.5	6.8	8.6	1.0	3.5	4.4	9.7	1.5	8.9
PGM1	16.5	6.6	9.2	0.8	2.4	3.1	12.6	2.0	7.5
STAP2	18.9	4.5	8.3	1.3	2.9	3.2	13.9	1.3	6.5
TJAP1	16.8	6.4	7.8	1.4	2.4	2.9	12.1	2.0	8.8
MYEOV2	19.3	6.5	10.0	1.8	2.1	3.4	10.5	1.8	5.2
PITPNA	14.2	6.2	7.8	1.0	3.5	4.5	11.6	2.1	9.6
DDX56	15.0	5.5	6.9	1.2	3.2	4.0	12.8	1.9	10.1
FAM83H-AS1	20.4	3.8	8.3	1.0	2.3	2.9	14.8	1.1	6.1
GDE1	16.5	6.7	9.2	1.1	2.9	3.8	10.7	1.3	8.3
USP7	15.0	5.0	6.4	0.8	1.8	2.9	14.6	2.1	11.9
NOC2L	15.3	5.8	7.6	1.7	3.3	4.8	11.4	1.7	8.9
LOC100506022	19.2	4.0	8.4	1.3	2.8	2.5	15.7	0.9	5.8

PBDC1	11.6	4.5	5.4	0.3	2.9	3.3	17.7	2.2	12.6
OSBPL10	13.4	4.6	6.2	1.2	2.0	3.6	14.4	2.3	12.9
LMO4	16.0	6.7	7.1	1.2	2.5	3.1	13.0	1.5	9.4
RNF13	17.0	6.8	9.2	0.4	2.4	3.4	9.8	1.9	9.4
TMEM134	16.7	7.6	9.5	2.5	4.1	6.6	6.7	1.1	5.5
BAZ2A	17.3	5.7	8.0	1.2	2.7	3.3	11.1	1.8	9.3
PSMC2	17.0	6.0	8.0	0.6	3.2	3.3	12.3	1.4	8.6
DHX16	15.2	6.1	7.5	1.3	2.6	3.4	13.3	2.0	9.1
AGRN	18.7	7.1	10.2	3.1	4.2	6.2	6.0	1.0	3.9
SLU7	15.9	6.2	7.3	0.6	2.4	3.3	11.6	1.9	11.2
TSSC4	14.8	6.5	7.3	1.4	2.6	3.5	13.6	1.7	9.0
LOC101928229	13.2	7.2	9.1	1.4	4.2	4.4	8.9	2.1	10.0
UBE2O	17.8	6.8	8.3	1.2	2.4	3.3	10.4	2.1	8.1
LOC100507131	19.4	4.2	8.7	1.1	2.4	2.5	14.7	1.1	6.3
ZRANB1	16.0	5.1	6.8	0.5	1.4	2.3	16.4	1.7	10.1
RASAL2	14.8	4.8	7.0	1.1	2.7	3.8	13.4	2.2	10.5
LOC100506302	18.3	3.7	8.7	1.1	2.6	3.0	14.5	1.2	7.2
C11orf49	16.7	6.8	9.3	1.3	3.5	4.2	9.5	1.7	7.2
EXOSC10	17.8	6.3	7.6	0.9	2.3	3.9	10.3	1.6	9.5
SLC20A2	15.9	5.3	6.7	1.1	2.1	3.3	14.7	2.0	9.1
INE2	20.8	3.9	8.5	1.4	2.2	2.6	13.7	1.3	5.8
ZCCHC14	18.2	6.1	8.0	1.5	2.7	3.5	10.6	1.8	7.9
UNQ9370	20.4	3.7	8.6	1.1	2.1	2.8	14.6	1.1	5.9
MAST2	16.0	6.0	8.3	1.7	3.1	4.2	10.7	1.6	8.4
MMADHC	16.3	5.4	7.8	0.4	1.8	2.7	15.8	1.2	8.7
RRP9	13.8	5.1	6.7	1.5	2.9	4.0	14.0	1.8	10.2
SPG7	18.8	5.9	9.6	1.9	3.3	4.4	9.0	1.3	5.9
MAPK3	17.6	8.1	10.6	1.9	3.7	5.3	6.2	1.3	5.5
C17orf89	14.9	5.0	7.5	1.0	2.8	3.3	16.8	1.6	7.1
NCKAP1	16.6	5.6	7.2	0.8	2.3	3.4	12.5	1.6	10.1
KDM2B	15.6	5.9	7.0	1.8	2.4	4.1	12.0	1.8	9.3
PKN3	16.1	6.6	8.1	1.8	3.0	4.3	10.8	1.8	7.5
ZNF410	16.8	5.0	8.1	0.7	1.8	2.2	14.6	1.8	9.0
PM20D2	13.9	5.1	6.4	1.3	2.5	3.7	15.0	2.2	9.8
LAMA5	21.7	7.9	11.6	2.0	2.9	4.0	5.4	0.8	3.6
LDLRAD3	14.6	5.1	6.4	2.2	3.2	3.8	12.6	2.5	9.6
C6orf1	16.3	7.0	10.0	2.8	4.3	5.2	8.1	1.1	5.1
PPP1R10	14.5	5.5	7.5	1.5	3.5	4.6	11.2	1.7	9.9
PSKH1	17.3	7.2	9.1	2.1	4.7	6.5	6.5	1.1	5.3
GSDMD	18.1	7.7	10.1	2.3	3.5	4.7	7.3	1.1	5.1
EIF2B4	14.1	5.8	7.7	1.0	2.6	3.6	13.5	1.8	9.7
PRR14	17.1	7.0	7.5	1.9	3.8	5.2	7.4	2.2	7.7
TFPT	15.1	6.2	8.5	1.4	2.5	3.4	12.9	1.7	8.2
WDR59	19.8	7.2	9.9	0.9	2.4	3.3	8.2	1.3	6.9
FADD	14.6	5.7	7.1	1.6	3.0	3.4	12.7	2.0	9.7
HDAC9	14.8	5.0	8.4	1.1	2.8	3.5	10.5	2.3	11.5
ADNP	18.2	6.5	7.8	0.7	2.1	3.2	10.0	1.5	9.9
MRPS10	14.9	7.0	9.6	0.3	1.6	4.0	10.8	1.4	10.2
GBF1	17.4	5.8	8.0	1.4	3.1	4.1	10.3	1.9	7.8
MIA3	17.6	6.0	8.3	0.8	2.3	3.1	11.7	1.6	8.3
MLXIP	15.9	6.2	7.3	3.5	4.3	4.9	8.7	1.9	7.1
ATP8B2	15.6	5.7	8.2	0.9	2.4	3.3	11.9	1.9	9.8
MAGI1	16.5	5.0	7.4	1.7	2.9	3.6	11.8	2.0	8.9
CKS2	17.7	6.2	11.3	1.0	1.9	3.0	9.4	1.1	8.0
KAT5	16.6	6.7	7.9	1.6	2.9	4.7	9.1	1.8	8.4

MOCS2	15.0	6.6	8.2	0.7	1.5	3.0	15.2	1.4	8.1
MARK2	15.0	6.0	7.2	1.7	2.9	3.8	10.5	2.6	10.1
DSP	15.1	4.8	6.4	0.7	2.0	2.6	14.3	2.1	11.8
IQSEC1	16.4	5.8	7.6	2.2	3.4	4.7	9.1	1.9	8.8
NUP188	15.1	5.4	7.0	1.2	2.9	4.0	12.5	2.0	9.7
MTG1	17.4	6.0	8.8	2.8	4.5	5.3	9.7	0.9	4.3
RAB2A	18.1	7.6	9.6	0.8	2.4	3.4	8.9	1.3	7.6
SNF8	17.0	5.3	6.2	1.4	4.5	5.1	11.4	1.3	7.4
RBM33	16.5	5.5	8.0	1.7	2.8	3.8	9.9	2.0	9.4
USP36	14.6	5.6	7.8	1.8	3.7	4.1	10.4	2.1	9.6
STAT6	15.3	6.8	8.9	1.4	3.4	4.5	9.4	1.6	8.3
MPZL1	15.8	5.8	7.8	0.4	1.7	2.8	12.4	2.1	10.8
QPCTL	14.8	5.9	7.7	2.0	5.1	5.7	9.3	1.7	7.5
LINC01002	17.0	6.3	9.2	2.3	2.9	4.2	9.9	1.6	6.2
WDR3	13.9	5.2	5.9	0.5	2.3	3.1	15.0	1.6	12.0
CALD1	15.3	5.5	7.3	1.4	2.8	4.4	12.5	1.9	8.6
SORBS3	16.3	6.6	7.8	1.8	4.2	4.0	9.2	2.0	7.6
RBM25	14.0	4.7	6.6	1.1	2.7	2.8	13.6	1.9	12.3
ZC3H12C	19.6	5.9	8.7	1.2	2.5	3.7	9.6	1.5	6.6
MKRN2	13.6	5.0	7.3	0.7	2.7	3.6	14.4	1.6	10.5
POLR1C	14.0	5.7	7.5	1.0	2.5	3.7	13.2	1.9	9.9
RPAIN	15.1	5.9	8.4	0.8	2.0	2.6	13.4	2.0	9.3
GIGYF1	18.6	7.4	10.4	1.6	2.7	3.5	7.9	1.5	5.9
GCSH	14.6	6.2	8.4	1.4	3.4	5.1	10.4	1.3	8.5
LPP	16.8	5.0	7.4	1.6	3.7	4.2	10.0	1.9	8.8
LOC101926996	19.8	3.8	8.0	1.3	2.2	2.7	14.3	1.2	6.2
TSG101	16.9	6.8	9.0	1.0	2.7	4.0	9.3	1.6	8.1
SNAI2	14.1	6.3	8.0	1.6	4.6	6.2	7.5	1.7	9.2
ELMO2	15.8	5.7	7.8	0.7	2.9	3.3	13.5	1.5	8.2
PLEKHM1	18.4	6.8	9.3	1.6	3.0	4.1	8.5	1.5	6.1
MIEN1	18.6	7.1	10.9	1.0	3.1	3.7	8.1	0.9	6.0
PRSS3	15.6	6.2	8.1	1.2	2.3	3.4	10.5	1.9	10.1
ZNFX1	14.9	5.2	6.7	1.3	3.1	4.4	11.9	1.8	10.0
HDAC10	17.2	6.8	9.8	1.9	2.9	4.0	8.6	1.5	6.8
FRMD4A	14.6	5.3	7.0	2.6	3.4	5.0	9.8	2.4	9.3
RAB8B	14.4	6.0	7.5	1.0	3.6	4.1	10.0	1.5	11.2
MED6	15.7	6.4	8.9	0.4	2.0	2.6	11.7	1.8	9.8
SCARNA5	15.6	6.3	9.3	0.5	1.8	2.0	12.2	1.2	10.5
HIGD1A	15.7	7.7	10.3	0.9	3.2	3.7	7.6	1.0	9.1
ZSCAN18	17.0	6.0	8.3	1.5	3.2	4.8	10.9	1.3	6.2
RMND5A	16.8	4.9	7.5	1.0	2.0	3.1	12.9	1.8	9.2
ASXL2	15.3	4.7	7.3	1.2	2.7	3.5	10.8	2.2	11.5
HLTF-AS1	16.9	5.9	9.1	1.5	3.4	4.7	9.5	1.3	6.9
PBXIP1	21.1	7.8	9.5	1.6	2.9	3.7	6.6	1.2	4.7
PPP2R5C	15.9	6.0	8.6	0.9	2.4	3.6	10.6	1.8	9.4
LOC101928750	14.9	5.6	9.3	2.0	3.2	4.4	11.6	2.2	6.0
GTF2H3	14.9	6.0	8.6	0.5	2.9	4.5	10.7	1.5	9.4
PNP	11.4	4.4	5.8	0.8	2.2	3.5	17.1	1.9	12.0
TRIM52-AS1	18.4	4.7	8.5	1.2	2.1	2.5	13.4	1.4	6.9
SGSM2	16.5	5.8	8.5	2.2	3.3	4.7	9.2	1.7	7.2
POMP	15.5	6.8	8.5	0.3	2.0	2.9	12.4	1.4	9.2
SPTLC2	16.7	5.7	7.8	1.0	2.9	3.2	12.1	1.4	8.2
C6orf89	18.1	6.8	8.4	0.9	3.5	3.5	9.6	1.2	6.9
EVI2A	12.4	5.6	8.2	0.6	3.8	4.9	10.6	1.7	11.1
SLC1A4	11.6	4.4	5.4	1.7	3.1	3.8	14.7	2.3	12.1

DDAH2	16.8	6.2	8.8	1.4	2.8	4.0	10.4	1.8	6.6
ARID1A	15.5	5.3	7.3	1.7	3.2	4.2	9.7	2.3	9.6
RNF213	20.0	5.0	8.2	1.2	2.2	3.6	10.5	1.1	6.9
TATDN2	16.9	5.7	7.2	0.8	2.3	3.2	11.2	2.2	9.3
DFFB	18.8	3.9	8.5	1.1	2.3	2.6	14.2	1.3	6.0
MAGEA6	15.1	5.5	7.3	0.9	2.7	3.7	11.7	2.0	9.9
TIMM9	15.1	5.7	8.1	0.3	1.7	3.1	6.3	1.7	16.7
TAX1BP1	15.7	5.4	7.7	0.6	2.4	2.7	13.0	1.4	9.8
MPI	14.4	6.4	7.9	1.3	3.0	4.7	10.7	1.7	8.6
NFKBIL1	15.7	6.6	7.5	2.3	3.0	4.7	9.5	1.7	7.8
KIAA1430	14.8	5.9	8.5	0.9	3.1	4.4	9.9	1.7	9.2
ESAM	14.4	5.3	7.1	1.0	2.0	2.6	14.1	2.2	9.9
C19orf24	14.2	7.3	8.6	2.3	3.4	4.6	8.5	1.9	7.8
UBTD2	17.0	6.8	8.3	0.8	2.4	3.0	9.4	2.0	9.0
SAP30L	16.7	5.7	6.6	1.1	2.8	3.8	11.7	2.0	8.2
HABP4	16.7	6.9	7.7	1.3	2.8	3.2	11.2	1.6	7.1
TP53I3	15.7	6.2	8.7	0.5	2.2	2.7	12.9	1.6	8.1
RSRC1	16.0	6.1	9.4	0.9	2.8	3.1	8.9	1.4	9.7
C9orf37	18.1	4.6	7.8	0.8	2.7	3.4	13.4	1.0	6.7
GIT2	16.4	5.3	8.1	1.4	2.9	4.4	9.2	1.7	9.0
LOC101928115	11.8	5.8	8.3	2.7	4.5	7.1	7.8	1.5	8.9
GTF3C2	15.9	5.8	8.2	0.9	2.5	3.4	11.0	1.8	8.9
GFER	17.4	5.6	7.6	2.5	3.2	4.5	8.3	1.9	7.4
LSG1	16.7	5.3	7.4	1.0	2.3	3.6	12.1	1.5	8.5
SMC3	15.8	5.5	6.7	0.5	2.3	3.1	12.9	1.7	10.1
NUTM2A-AS1	14.5	5.3	7.3	1.2	2.6	3.4	12.4	2.3	9.4
SUCLG2	17.3	7.1	9.0	0.9	3.8	4.6	7.9	1.1	6.6
NUDCD3	17.8	6.0	8.4	1.2	3.1	3.7	8.7	1.5	7.8
CHRDL1	18.6	6.9	9.2	0.8	3.7	3.8	7.6	1.2	6.5
C8orf44	19.4	3.9	7.8	0.9	1.9	2.7	13.1	2.2	6.4
SYPL1	17.2	6.1	8.7	0.4	1.8	2.9	10.4	1.4	9.3
UBAC1	13.2	5.8	7.2	1.4	3.3	4.7	12.2	1.9	8.7
ATP6AP2	17.4	7.0	10.5	0.7	2.6	4.3	8.6	1.1	6.1
UNC119B	17.3	5.9	8.1	1.9	4.1	5.5	6.8	2.0	6.6
KDM3B	17.8	6.4	8.2	0.9	2.2	3.2	10.3	1.5	7.7
GPBP1	14.0	5.4	8.0	0.5	1.8	2.3	12.9	1.8	11.6
MON1B	17.2	5.6	7.8	1.7	3.0	3.8	9.7	2.3	7.0
CNPPD1	15.5	5.8	8.3	2.4	3.3	4.7	9.2	1.3	7.6
RNASEH2C	12.3	4.7	7.2	2.0	3.5	4.4	13.6	1.9	8.7
TFE3	14.3	5.5	7.1	2.3	4.2	5.5	9.1	1.9	8.2
MMP24-AS1	16.7	6.1	8.6	3.0	4.9	6.5	6.6	1.3	4.5
DCAF11	17.0	6.0	8.0	1.3	3.1	4.0	10.6	1.3	6.8
TTC19	16.3	6.5	8.3	2.2	3.7	4.8	9.0	1.0	6.3
MAP4K5	13.8	5.7	7.2	0.7	2.4	3.8	11.4	2.1	11.0
CTU1	15.2	5.1	6.2	2.2	3.0	4.4	12.4	2.2	7.2
TERF2	14.4	5.4	7.2	1.5	2.7	3.6	10.7	1.9	10.6
POLR3D	15.9	5.8	7.4	1.2	2.8	3.3	11.4	1.6	8.6
ABCC1	16.3	5.8	7.7	1.7	3.3	4.4	9.5	1.5	7.7
NACC2	17.1	5.6	8.0	2.3	3.3	4.3	9.3	1.8	6.3
GMPR2	15.3	6.1	8.6	1.0	3.4	4.3	10.4	1.3	7.7
FAM171A1	15.2	5.7	7.2	1.9	3.4	4.2	10.2	2.0	8.1
SRGAP1	14.6	4.5	6.4	1.2	2.5	4.3	12.5	2.0	9.9
LAGE3	16.5	5.4	7.7	1.8	2.3	4.3	12.0	1.4	6.6
COQ9	18.8	7.3	9.5	1.8	3.6	3.7	7.3	1.0	4.9
SERPINB6	16.5	6.1	8.4	1.2	3.5	5.0	9.9	1.2	6.0

ENTPD6	18.5	6.6	8.2	1.8	2.9	4.7	7.6	1.1	6.4
PTEN	15.6	5.8	7.5	0.4	2.0	2.3	12.0	1.5	10.8
STK4	12.8	4.3	6.5	1.0	3.0	4.1	13.7	2.2	10.4
PHF5A	12.8	4.9	7.0	0.9	2.1	2.1	15.4	2.2	10.4
ZKSCAN1	18.4	5.0	7.8	1.3	3.3	3.9	8.9	1.2	8.1
OPA1	15.2	5.3	7.2	0.5	2.4	3.2	12.5	1.5	9.9
PLEKHJ1	18.7	6.4	7.2	1.5	2.7	3.9	9.7	1.2	6.5
PIGY	15.8	5.7	7.8	0.5	2.2	1.8	14.6	1.5	7.9
NUP93	14.5	5.6	7.5	1.1	2.6	3.5	11.7	1.8	9.3
ANAPC7	13.9	4.9	7.2	1.0	3.0	4.4	12.4	1.6	9.4
PSPC1	15.5	5.4	8.2	1.0	2.3	3.2	12.0	1.5	8.6
RBM19	15.2	5.6	7.7	1.5	2.8	4.1	10.3	1.9	8.4
TAF1C	13.8	6.1	7.9	2.8	3.8	5.7	8.7	1.9	7.0
EMC7	16.4	6.4	8.3	0.7	2.1	3.1	10.4	1.6	8.5
STAT2	17.9	6.7	8.9	1.1	3.6	4.9	7.9	1.1	5.5
SMCR7L	12.5	4.4	6.8	1.1	2.5	3.3	14.4	2.2	10.4
NUP50	14.8	5.2	7.5	0.4	1.8	2.6	12.8	1.8	10.6
CSGALNACT2	15.5	4.8	6.5	0.6	2.0	3.2	12.9	1.7	10.4
GET4	16.0	5.6	7.5	1.2	2.8	3.2	11.7	1.8	7.8
PNO1	14.5	6.0	8.1	1.6	2.6	4.2	9.1	1.7	9.8
CPSF3	15.6	5.4	7.3	0.6	2.1	3.1	12.6	1.7	9.1
C3	25.7	8.5	13.5	1.6	2.8	3.8	0.8	0.2	0.6
ACTR10	17.1	6.7	9.5	0.5	2.2	2.7	10.8	1.1	6.9
CD47	17.6	7.2	9.8	0.6	1.7	3.1	9.0	1.0	7.6
SETD3	14.0	4.8	6.0	0.6	1.9	2.8	14.9	1.3	11.2
RAMP2-AS1	17.9	3.9	8.2	0.9	2.3	2.3	14.8	1.2	6.0
COPS5	14.9	5.9	8.9	0.7	2.1	2.6	12.4	1.3	8.6
NME3	16.0	5.3	9.1	2.0	4.3	4.2	8.5	1.7	6.4
NDUFAF2	12.2	6.1	8.7	0.9	4.3	4.7	8.3	1.9	10.4
SMCR8	15.5	5.3	6.9	0.9	2.2	3.4	11.8	2.0	9.4
CAMTA2	16.8	6.7	8.8	1.6	3.3	3.9	8.0	1.4	6.9
SATB2	16.3	5.9	8.1	1.1	3.2	4.1	9.1	1.6	8.0
ENTPD1-AS1	18.6	3.6	7.9	1.0	2.3	2.6	14.0	1.0	6.3
LOC285033	14.6	5.7	6.9	1.5	2.9	3.4	13.0	1.8	7.8
MAML1	16.2	5.2	7.1	1.7	2.8	3.9	9.6	2.1	8.8
ARL6IP6	15.7	6.1	8.7	1.3	1.9	2.5	11.1	1.8	8.3
METTL17	15.4	6.8	8.4	0.8	2.8	3.5	10.5	1.7	7.4
MRPL13	15.0	7.2	9.0	0.5	2.9	4.1	9.7	0.7	8.3
RNF149	14.8	5.2	7.3	1.4	2.7	3.0	12.7	1.7	8.7
UFD1L	15.1	5.7	9.2	0.9	2.5	3.6	10.3	1.6	8.4
SUDS3	15.3	5.6	8.0	1.1	2.3	3.8	10.4	1.7	9.2
PPM1A	15.5	5.1	7.3	0.5	2.4	2.9	12.6	1.2	9.8
ARHGAP5	17.0	5.3	7.6	0.5	2.2	3.0	11.6	1.4	8.7
CREB3L1	21.2	8.0	9.0	1.0	1.9	2.8	7.2	1.3	4.9
NAT10	14.1	5.2	6.5	1.3	3.0	4.0	11.9	1.7	9.5
TBC1D20	15.2	6.1	8.4	1.0	2.5	3.2	11.7	1.7	7.6
BSN-AS2	18.8	3.6	8.4	1.1	2.1	2.5	14.3	1.0	5.5
IVNS1ABP	17.4	6.1	9.0	0.3	1.3	1.5	12.6	1.3	7.7
RNF216	15.5	5.7	8.6	0.9	2.1	3.1	10.0	1.9	9.4
RRP36	15.0	6.4	8.3	0.9	2.9	3.3	9.9	1.8	8.7
HNRNPUL2	16.3	6.8	9.0	0.8	2.4	3.6	8.9	1.2	8.4
LOC100506746	17.7	4.1	8.1	1.1	2.1	2.3	14.1	1.2	6.6
PIN4	13.6	6.8	7.0	0.4	4.5	4.8	7.4	1.9	10.8
SNORA37	12.6	7.2	7.6	3.3	2.5	3.1	12.0	0.8	8.0
THOC7	14.9	6.1	8.4	0.5	2.1	4.0	10.7	1.5	9.0

UBP1	15.1	5.8	7.5	0.7	2.3	3.9	9.9	1.7	10.1
DIMT1	11.9	4.7	6.9	0.7	2.1	3.4	13.5	1.9	12.1
TRAPPC3	16.3	6.6	8.3	1.1	2.7	3.6	10.6	0.9	7.1
IGFBP6	18.4	8.1	10.1	2.4	3.5	5.1	4.9	1.1	3.6
LOC101927131	18.6	3.6	8.1	1.1	2.1	2.5	14.1	1.1	6.0
FAM20C	17.3	6.2	8.0	4.1	6.2	8.8	3.4	0.6	2.5
HDAC7	14.9	5.7	7.6	1.3	2.5	3.8	10.7	2.4	8.1
CTU2	13.6	5.1	6.9	1.9	3.0	3.8	12.9	1.7	8.3
DPM2	17.2	6.7	7.9	2.9	4.4	4.9	5.7	2.1	5.3
QKI	15.5	5.4	7.2	0.7	2.2	3.2	12.2	1.4	9.0
CTNNAL1	13.7	5.3	7.6	0.6	2.3	3.4	12.4	1.5	10.3
RARS	13.3	4.9	7.0	0.3	1.7	2.6	15.9	1.3	9.9
LOC101928925	14.9	6.5	7.1	1.4	2.4	3.9	9.7	1.7	9.4
SH2B1	17.3	7.8	8.3	1.4	2.3	3.3	8.7	1.5	6.4
PANK3	14.5	5.6	7.4	0.4	2.3	2.5	13.5	1.3	9.5
TEAD1	12.2	4.3	5.7	0.8	2.2	3.2	11.9	2.4	14.2
SSBP1	17.0	6.4	8.0	0.5	2.3	3.3	10.5	1.4	7.7
TBC1D10B	14.8	5.7	7.2	1.9	3.0	3.8	11.2	1.7	7.6
FGD5-AS1	17.1	5.4	8.3	0.9	2.5	3.1	11.1	1.2	7.3
EIF4E2	15.8	6.6	8.8	0.9	2.3	3.3	8.5	1.6	9.1
MAD2L2	15.7	6.2	7.6	1.3	3.2	4.5	9.8	1.7	7.0
ADH5	14.7	6.4	9.0	0.6	1.8	2.7	12.9	1.1	7.7
KCTD5	13.7	6.0	7.7	1.7	3.2	4.1	8.3	2.2	9.9
NSUN2	16.0	5.7	7.4	1.0	2.1	3.1	11.5	1.6	8.5
DNLZ	15.2	5.5	7.2	1.4	2.8	3.2	12.4	1.9	7.3
RALBP1	15.4	5.3	7.6	0.7	2.3	2.7	12.2	1.4	9.2
NEURL4	18.3	7.5	10.2	1.2	2.3	2.8	8.0	1.2	5.4
FLYWCH2	14.7	5.6	7.1	2.3	3.8	4.6	9.2	2.1	7.3
SPATA20	17.2	7.1	9.3	1.8	3.5	4.8	7.2	0.9	4.9
PTPLAD1	14.6	5.8	8.2	0.5	1.9	3.4	11.6	1.5	9.3
C17orf61-PLSCR3	15.0	6.4	7.9	1.8	3.4	5.0	8.4	1.6	7.2
CLMP	11.9	5.0	5.7	1.5	3.3	5.0	12.6	1.9	9.9
ZNF865	15.9	6.0	7.1	3.0	3.8	5.1	7.8	1.9	6.2
ATP6V0A2	14.3	4.7	7.0	0.7	2.1	2.3	13.9	1.5	10.2
SEC63	12.8	5.0	6.5	0.6	1.9	2.8	13.3	2.1	11.6
MRPS35	14.0	6.5	7.9	1.2	2.3	3.7	11.1	1.4	8.7
SLC25A11	12.8	6.0	8.2	1.6	4.0	4.4	10.0	1.7	8.0
CCNG1	18.3	6.6	8.2	0.4	1.5	2.4	11.3	1.3	6.8
FBXO27	12.0	4.5	6.8	1.7	2.2	4.0	14.3	2.4	8.8
TSEN15	16.7	5.6	7.0	0.8	2.4	2.7	11.9	1.3	8.2
CPOX	15.2	5.3	6.7	1.5	2.9	3.8	11.9	1.8	7.4
MCM3	11.3	3.9	5.4	1.0	2.2	3.6	14.8	2.1	12.3
NOA1	15.8	5.4	6.7	1.8	2.4	3.9	11.2	1.8	7.6
LOC101928144	19.1	3.6	8.3	0.8	1.9	2.2	13.7	1.0	5.9
ELK1	12.3	5.6	7.5	2.2	3.3	4.5	10.0	2.2	9.1
LOC101929426	19.3	4.3	8.8	1.1	1.9	2.5	11.8	1.0	5.9
ORMDL3	17.8	6.8	7.5	1.6	3.2	3.1	8.2	1.8	6.6
PLCB3	14.9	5.6	7.6	2.1	3.9	4.9	8.7	1.6	7.2
SLC41A3	16.2	6.0	8.7	1.3	2.9	4.5	8.7	1.5	6.6
LOC101929586	17.8	3.3	7.6	1.1	2.3	2.5	14.7	1.1	6.1
TEX261	16.8	6.7	8.2	1.2	4.2	5.8	4.8	1.7	7.1
FEN1	12.8	4.9	6.1	1.1	2.6	3.8	13.3	2.0	9.8
SNAP23	14.3	4.8	9.0	0.7	3.4	3.6	9.3	1.5	9.9
LOC646329	17.3	3.0	7.1	1.3	2.7	2.9	14.1	1.0	6.9
CTBS	16.6	6.4	8.6	1.6	3.2	4.7	8.9	1.4	5.0

EIF1AX	12.6	4.9	7.7	0.5	2.5	3.0	13.7	1.2	10.2
TRA2A	16.3	6.1	7.3	1.3	3.0	4.2	8.5	0.9	8.6
EXOSC2	14.6	4.0	7.1	1.0	2.4	3.0	14.5	1.5	8.3
CKAP5	14.5	5.1	6.5	0.7	2.3	3.1	12.1	1.6	10.3
TMEM160	16.0	5.2	6.7	1.5	2.6	2.9	14.1	1.3	6.0
LRRC14	16.1	6.2	8.3	1.3	2.8	3.7	10.3	1.1	6.4
ATP6V1B2	15.2	5.6	7.7	0.6	2.3	2.9	11.1	1.4	9.4
TLE3	14.7	5.8	6.9	2.4	3.5	4.1	7.6	2.0	9.3
ASB1	13.6	5.2	6.8	1.5	3.8	3.4	13.0	1.1	8.0
C19orf66	15.2	6.5	8.0	2.7	3.5	5.3	7.7	1.3	6.1
LOC101927034	19.2	3.5	8.5	0.6	2.3	2.8	11.4	0.9	7.1
CALB2	15.3	5.4	7.2	1.5	2.8	3.5	10.3	1.7	8.6
C15orf61	15.4	6.1	8.3	2.3	2.5	4.0	8.5	1.6	7.6
GEMIN4	13.3	4.8	6.3	1.3	2.8	3.9	13.0	1.8	9.2
CLIP1	15.9	5.5	7.1	1.1	2.5	3.3	10.4	1.6	8.8
BACE1	18.3	6.4	9.3	1.4	3.1	4.9	6.5	0.9	5.3
LOC101927415	18.8	3.8	7.9	1.0	2.0	2.4	13.5	0.9	5.8
SCARF1	17.7	6.0	7.3	1.6	2.6	4.3	9.5	1.4	5.7
ATG4D	16.4	6.5	8.2	1.3	2.4	3.3	9.3	1.5	7.2
IMPDH1	14.4	5.4	6.7	1.3	3.2	3.5	11.7	1.3	8.5
RHBDF1	16.4	6.2	8.0	2.4	4.3	5.1	7.1	1.2	5.4
STX4	14.8	6.1	7.6	1.1	3.2	4.1	10.2	1.2	7.8
RABGGTB	14.5	5.6	7.6	0.3	1.7	2.1	13.6	1.0	9.5
BIN1	15.6	6.2	8.2	1.8	3.4	4.3	7.6	2.1	6.9
SMEK1	13.5	4.6	6.3	0.8	1.8	2.6	13.5	2.2	10.7
UGGT1	17.3	4.0	7.4	0.9	2.2	2.5	13.4	1.2	7.0
C11orf24	15.0	6.2	5.5	1.8	4.1	5.9	6.9	2.0	8.5
NDUFB2	14.6	4.9	7.5	1.1	2.3	3.9	11.6	1.3	8.7
MORC2	13.9	4.6	6.1	1.2	2.9	3.6	12.2	1.9	9.6
PLEKHO1	15.8	5.0	7.1	1.0	2.4	3.3	10.8	1.8	8.6
TTLL5	13.5	5.1	6.3	1.0	2.5	4.0	10.6	2.0	10.9
SH3GLB1	15.5	6.2	9.2	0.6	2.4	3.2	9.2	1.7	8.0
LOC100996598	17.7	3.8	8.5	0.8	2.2	2.1	14.1	1.3	5.6
EIF2AK4	15.7	5.4	6.9	1.1	2.9	3.4	11.1	1.5	7.8
SMIM15	13.9	5.2	5.4	0.3	1.8	2.9	12.8	2.1	11.4
THOC6	12.6	4.3	5.8	1.8	3.1	5.0	12.8	1.8	8.7
UPF3A	14.7	5.0	6.7	1.7	3.2	4.0	10.4	1.7	8.4
PPFIA1	15.3	5.1	7.4	1.1	2.6	3.4	10.8	1.6	8.6
FAF2	16.6	5.9	8.8	0.9	2.7	3.8	7.5	1.5	8.1
CLDN12	13.6	5.3	7.3	1.0	2.9	3.4	10.4	1.8	10.2
ELMSAN1	15.9	6.1	7.8	2.0	3.2	4.2	7.7	1.7	7.1
MYEOV	11.8	4.2	4.9	2.7	5.1	7.3	10.1	1.5	8.2
SDR39U1	13.7	5.2	7.7	1.2	2.5	4.0	11.9	1.5	8.1
DHX8	15.9	5.7	7.0	0.8	2.5	2.9	10.8	1.6	8.7
TMEM150A	15.7	6.7	8.4	1.5	3.2	4.4	7.9	1.3	6.6
AIMP2	13.5	5.4	6.6	1.2	2.8	4.5	11.4	2.3	8.1
LOC101927550	17.5	3.7	7.8	1.2	2.4	2.8	13.0	1.1	6.2
PTPRE	16.6	6.4	8.2	1.5	3.1	3.9	7.9	1.5	6.6
RRP1	12.9	5.1	6.3	2.0	2.9	3.9	11.6	2.0	9.0
LINC00311	18.2	4.0	7.8	0.9	2.1	2.5	13.1	0.9	6.3
KHNYN	16.9	6.1	8.4	1.1	2.4	3.3	8.7	1.2	7.4
BNIP2	13.7	5.1	6.6	0.8	2.7	3.1	13.3	1.8	8.6
PHYHD1	17.9	5.2	8.6	1.4	2.7	4.1	10.0	0.9	5.0
ACAT1	12.3	6.0	6.8	0.7	2.3	3.3	11.5	1.8	10.9
ARMCX2	14.0	5.6	7.5	1.1	2.0	2.7	13.0	1.4	8.2

DARS	15.5	5.0	6.7	0.6	2.2	3.0	13.0	1.5	8.2
YTHDC1	13.2	4.7	6.2	0.6	2.3	2.7	13.2	1.9	10.7
MBD1	14.1	4.7	6.0	1.6	2.4	3.7	13.0	2.1	7.9
CCBE1	16.2	6.8	8.4	1.0	2.1	3.7	9.4	1.3	6.5
FBXL6	12.8	5.9	7.8	1.9	3.0	3.8	10.9	1.6	7.7
SLC8B1	16.5	6.4	8.1	1.4	2.8	4.0	8.7	1.4	6.3
C3orf37	14.9	5.5	7.6	0.9	2.4	2.6	11.2	1.6	8.8
SNAP29	15.8	5.7	7.9	1.8	2.8	4.0	7.8	2.1	7.5
ACTL6A	14.9	6.1	8.9	0.4	2.1	2.9	9.8	1.3	9.2
DPM3	18.6	6.2	10.8	1.3	3.0	3.9	7.4	0.6	3.8
PPAN-P2RY11	18.8	4.4	7.9	1.4	3.8	3.5	6.4	1.4	7.7
LOC729737	17.2	4.9	8.1	2.4	3.6	4.5	7.5	1.4	5.8
SRP19	11.0	6.2	7.8	0.3	2.1	2.3	10.2	1.8	13.6
C9orf142	12.4	5.2	7.7	2.1	3.4	4.1	11.0	1.3	8.1
USP21	15.1	6.2	8.3	1.0	2.5	3.4	10.3	1.5	7.0
FBXO31	14.0	4.9	6.3	1.5	2.8	3.9	11.8	1.8	8.3
RNF41	15.7	4.0	7.5	1.0	2.2	2.7	13.3	1.3	7.6
TMEM129	18.1	6.2	8.3	3.5	5.1	6.7	4.6	0.5	2.4
DHX37	14.1	5.5	7.2	1.3	2.2	3.4	11.5	1.6	8.5
FLJ31662	19.2	3.4	8.6	0.7	2.6	3.2	10.9	0.9	5.7
REXO1	14.4	5.2	6.8	1.6	2.4	3.5	11.0	2.0	8.4
ATG2A	15.7	5.9	7.5	2.0	3.7	4.5	9.0	1.4	5.6
PHC1	13.2	5.4	7.2	1.5	2.7	4.0	9.5	2.0	9.6
SH3BP5L	13.4	5.6	7.6	1.7	3.5	3.8	10.0	2.0	7.7
MALSU1	13.0	5.4	7.8	1.9	3.9	3.0	10.6	1.6	8.0
MSL1	14.2	5.7	7.2	0.9	2.2	2.8	12.3	1.9	8.0
PFKL	16.9	6.3	8.2	2.4	4.3	5.6	6.1	1.0	4.3
CSNK2A3	16.9	1.9	7.3	0.2	1.5	1.0	11.9	2.2	12.3
ABCC5	19.1	6.1	7.4	0.6	1.1	2.7	10.3	1.4	6.6
SEMA4B	19.0	7.2	9.5	0.9	2.1	2.8	7.7	1.2	4.8
BREA2	17.1	4.5	7.7	1.2	2.5	2.1	13.2	1.1	5.8
HYAL2	19.0	6.7	9.8	1.5	2.7	3.8	6.5	1.0	4.2
MED17	14.9	5.6	7.1	0.9	2.1	2.8	11.2	1.5	9.1
FHL3	13.8	5.0	6.6	1.1	2.6	3.4	12.0	2.0	8.8
ZNF121	12.6	5.2	6.6	0.4	2.3	3.0	12.0	1.3	11.7
PDCL3	15.7	5.8	7.5	0.8	2.8	3.6	10.2	1.5	7.4
DHX38	13.3	4.7	6.4	1.2	2.6	3.7	12.5	1.7	9.0
FRG1	13.0	6.7	9.1	0.4	2.4	4.7	8.5	0.8	9.5
FKBP11	11.5	3.7	5.7	1.2	2.5	3.6	14.3	2.3	10.4
FAM84B	15.7	5.3	6.4	1.2	2.0	2.7	11.4	1.8	8.5
PPP4R2	14.2	5.0	5.6	0.5	1.6	2.1	14.8	1.3	10.0
GALNT1	16.6	6.6	7.9	0.4	2.3	2.6	9.3	1.3	8.1
TSKU	13.9	7.3	7.5	2.4	4.2	5.1	6.8	1.5	6.4
ABCE1	13.5	5.5	6.2	0.2	1.8	3.2	12.6	1.2	10.7
LOC101927884	19.2	3.7	8.2	1.1	2.6	2.9	9.3	1.1	6.9
DEDD	17.4	5.9	7.4	1.2	2.4	4.2	7.8	1.3	7.3
DPM1	12.6	5.9	7.5	0.7	3.7	3.6	9.9	1.5	9.5
TSN	12.8	4.8	7.7	0.7	2.0	2.6	13.7	1.1	9.6
RHOJ	17.0	6.2	7.2	0.7	2.0	2.4	10.7	1.6	7.1
TIMM23	12.6	5.6	8.4	1.0	2.7	3.6	9.9	1.8	9.4
LYAR	11.8	4.9	5.6	0.8	2.4	3.0	14.0	2.3	10.1
RPRD1A	14.8	5.0	7.9	0.7	2.1	2.9	10.7	1.4	9.3
MYO10	13.6	4.6	6.1	1.5	3.5	4.7	10.7	1.6	8.6
LRRC8D	13.4	4.6	6.1	0.6	1.9	3.3	11.7	1.9	11.3
HIPK1	14.4	5.0	6.6	1.1	2.4	3.4	9.5	2.3	10.2

MRPL46	13.0	5.7	8.8	1.2	3.2	3.7	9.8	1.7	7.9
WSB1	13.0	5.5	6.8	0.8	1.8	2.3	13.5	1.7	9.5
ZNF282	12.8	4.8	6.0	1.9	2.9	3.8	11.7	2.2	8.8
GALE	14.4	6.2	8.0	1.3	2.9	3.8	10.5	1.4	6.3
YRDC	12.3	5.0	6.4	1.8	2.6	2.8	14.2	2.0	7.8
DBNL	12.8	4.6	7.2	2.0	3.2	4.8	11.0	1.8	7.5
RPS6KB2	13.8	6.1	7.4	1.6	3.7	4.0	8.9	2.0	7.2
TOP3A	14.4	4.9	6.6	0.9	2.3	2.9	11.9	1.6	9.3
PISD	13.7	5.3	7.0	1.2	2.5	3.3	11.5	1.7	8.5
RIN2	16.5	6.1	8.2	1.5	3.5	4.9	7.2	1.2	5.7
ZFYVE21	16.4	5.6	6.7	1.3	2.8	3.5	11.1	1.0	6.3
APEH	15.0	5.6	8.0	1.7	3.7	5.3	8.4	1.2	5.7
SLC25A51	15.8	4.0	7.7	1.0	2.1	3.0	13.4	1.2	6.5
DOHH	13.4	5.2	7.1	2.1	2.2	3.1	11.7	2.7	7.3
LOC101929652	17.2	4.3	8.4	1.1	2.2	2.8	12.3	1.1	5.4
FLOT2	16.6	6.6	8.0	1.7	3.9	4.7	7.2	1.1	4.8
NOV	12.5	4.5	5.9	0.9	1.5	2.1	15.0	2.8	9.4
POLD4	15.8	4.8	7.2	1.6	3.6	6.4	8.8	1.3	5.2
PPTC7	12.6	5.8	6.2	1.4	2.9	2.9	11.9	1.8	9.1
ZSWIM6	14.1	4.9	6.5	1.4	2.6	3.6	10.3	1.7	9.4
FNTA	13.6	4.2	6.6	1.4	2.4	3.0	12.0	1.9	9.3
SNORA53	18.7	9.9	13.3	0.2	1.0	0.6	6.1	1.1	3.7
SMG6	15.4	5.0	7.2	1.3	3.1	3.9	9.8	1.6	7.4
MFI2	16.1	6.8	8.1	3.0	5.5	7.2	4.1	0.5	3.1
ATG9A	14.0	5.6	6.3	1.4	2.5	3.6	10.7	1.9	8.5
RUSC2	10.9	4.5	5.6	1.8	2.9	4.1	11.6	2.4	10.6
GAMT	13.4	5.0	6.9	3.2	5.0	7.4	7.2	1.1	5.4
CIAPIN1	14.6	5.9	8.0	0.8	1.9	2.7	11.8	0.9	7.7
SEPHS2	13.6	5.1	7.1	0.8	2.2	2.9	12.6	1.6	8.6
FOXJ3	13.3	4.9	6.0	1.3	2.4	3.5	8.9	2.5	11.7
AP1B1	14.9	5.2	7.1	2.1	3.9	4.8	7.9	1.5	6.9
SLC27A4	13.6	5.0	5.6	1.8	3.4	4.5	10.0	2.1	8.4
VSTM2L	19.3	8.3	10.1	1.9	2.0	3.0	3.8	1.1	4.9
NME1	12.3	4.5	6.9	0.4	2.0	2.8	12.8	1.6	11.1
TMEM101	17.6	5.9	9.1	1.5	2.7	4.6	7.2	0.8	4.8
TRIB1	12.4	5.2	7.1	1.4	2.3	2.8	11.9	2.3	8.8
COMMD3-BMI1	13.4	5.5	6.3	0.5	1.9	2.6	11.5	1.9	10.7
FGFRL1	14.3	5.8	7.7	2.6	4.4	5.9	6.6	1.3	5.7
CCZ1	15.0	5.6	7.5	0.4	1.7	3.5	10.8	1.3	8.4
MAN2A2	16.3	6.2	9.0	1.5	2.7	3.5	7.4	1.4	6.4
NMT2	13.0	4.5	6.5	1.7	4.8	6.2	8.8	1.4	7.4
MAPK1	14.3	6.1	7.2	0.9	2.7	3.8	8.4	1.4	9.4
PSMG4	14.3	5.2	6.9	3.1	4.9	7.0	6.3	1.2	5.3
C7orf26	13.6	5.5	6.3	1.0	2.1	2.9	12.4	2.0	8.4
STX1A	15.9	6.4	8.5	1.7	2.6	3.8	7.2	1.7	6.3
PPP1R8	13.1	5.4	7.4	1.0	2.2	3.5	9.4	1.7	10.4
PSMC6	14.9	5.8	8.4	0.6	2.3	3.0	9.7	1.3	8.2
SGPL1	15.6	6.1	7.7	0.5	1.8	3.2	10.7	1.1	7.5
SNHG3	16.0	5.5	7.8	0.6	1.8	2.6	11.3	1.4	7.1
PARD6B	14.1	4.7	7.2	0.9	3.5	4.6	9.6	1.3	8.3
KIAA1522	16.1	6.5	9.7	0.8	1.9	2.4	8.3	1.6	6.8
LAMP2	16.2	6.5	8.9	0.6	2.3	3.2	7.7	1.2	7.5
TYMS	7.8	3.7	5.3	2.2	3.1	4.7	13.2	2.8	11.4
NOTCH1	13.8	4.6	6.3	2.1	2.6	3.6	10.8	2.0	8.2
RALGDS	15.1	5.5	8.0	1.9	3.4	3.9	8.0	1.3	7.1

DGKD	16.0	5.8	8.4	1.0	2.2	2.9	9.3	1.4	6.9
PROSC	14.8	5.5	9.0	1.4	3.2	3.1	9.1	1.2	6.7
ZCCHC24	19.7	7.5	9.8	1.5	3.1	3.4	4.7	0.8	3.5
LINC00958	16.9	3.5	8.3	0.8	1.5	2.4	13.9	1.0	5.8
NFRKB	15.4	5.8	8.1	0.9	2.2	3.1	9.1	1.7	7.6
GNAI3	14.3	5.6	7.2	0.7	2.4	2.1	11.5	1.4	8.6
RBPJ	14.6	5.2	7.4	0.6	2.2	3.8	8.9	1.7	9.4
ARFGEF2	16.5	5.3	7.7	1.0	2.9	3.2	9.1	1.1	7.0
NDOR1	14.6	5.6	7.5	1.4	2.6	3.5	9.7	1.6	7.3
PRPF40A	13.4	4.8	6.3	0.7	1.8	2.7	12.4	1.7	10.0
SUPT7L	15.5	5.2	8.0	1.1	2.3	3.6	9.1	1.6	7.4
TRAF3IP2	17.1	4.0	7.4	1.0	2.1	2.6	12.7	1.1	5.9
UTP6	15.1	6.0	7.0	0.3	1.9	2.3	11.8	1.0	8.4
ZDHHC3	15.8	5.7	8.1	0.7	2.8	2.8	9.4	1.1	7.5
SPOP	14.0	3.9	6.0	0.4	1.9	2.3	15.6	1.4	8.2
NLE1	12.5	4.6	6.3	1.6	3.1	3.7	11.8	1.9	8.4
PHF10	14.3	4.9	7.0	0.6	2.1	3.0	11.7	1.6	8.7
ALDH7A1	11.7	5.8	6.6	0.9	2.9	3.5	11.4	1.7	9.3
PAPSS2	13.4	5.3	6.3	0.7	1.6	2.3	13.0	1.7	9.3
GOLGA8B	17.4	6.4	9.4	0.9	1.9	2.5	8.3	1.2	5.7
FAM60A	10.5	3.7	6.3	0.4	1.3	2.4	14.2	1.7	13.1
ABLIM1	13.6	5.2	5.2	0.9	3.1	3.3	11.0	1.9	9.4
WDR55	14.3	5.6	7.5	1.0	2.9	3.3	10.5	1.4	7.2
MOB1A	14.9	4.7	6.6	0.3	2.3	3.2	10.3	1.1	10.3
LSM10	15.6	6.6	6.8	1.4	2.2	2.9	8.7	1.6	7.8
MSANTD2	17.0	4.4	8.1	1.4	2.7	2.9	10.3	1.4	5.4
DOCK6	13.9	5.6	7.7	1.8	2.7	3.5	9.5	1.7	7.2
SNAP47	15.1	5.9	7.9	1.0	1.7	3.2	10.2	1.4	7.2
CHSY1	14.5	5.5	6.9	0.8	2.5	3.1	10.5	1.6	8.4
NDUFB1	13.5	6.4	8.1	0.6	1.8	3.9	9.9	1.6	7.6
MAPKAPK5	15.8	5.9	6.8	0.8	2.6	3.5	9.6	1.4	7.2
E2F7	14.9	5.2	6.6	1.2	2.8	4.3	8.7	1.7	8.1
PDHX	15.7	6.8	7.4	0.9	2.4	3.3	8.1	1.4	7.4
PIGH	12.6	5.1	6.2	1.1	1.8	3.1	12.4	1.9	9.3
SIRT6	16.2	6.7	7.4	2.1	3.6	3.6	6.6	1.6	5.6
UBXN7	14.7	5.2	7.6	0.6	1.9	3.5	10.3	1.4	8.2
CNIH4	16.3	5.2	9.0	0.6	1.6	2.9	8.3	1.2	8.3
SCARB1	14.0	5.0	6.2	1.6	2.5	4.8	9.2	1.7	8.3
WDR34	14.4	5.8	7.7	2.1	3.4	4.5	8.5	1.3	5.7
TMEM222	14.1	5.6	7.8	1.4	3.6	3.8	9.2	1.5	6.4
METTL2A	11.7	4.6	7.2	1.0	2.7	3.2	11.2	1.9	9.9
VPS37C	15.3	5.8	6.6	1.3	2.7	3.2	8.9	1.8	7.7
NME6	15.8	3.8	7.9	0.9	2.2	2.6	12.8	1.1	6.3
PRO2852	16.8	3.5	7.2	1.0	1.8	2.2	13.7	1.0	6.1
UBL4A	12.3	5.2	7.1	1.2	3.2	3.7	10.5	1.5	8.6
XPNPEP1	14.4	5.3	7.0	1.0	2.2	3.5	10.4	1.5	7.9
USP4	15.8	4.4	7.1	0.9	2.4	3.2	10.6	1.3	7.5
LITAF	15.7	5.8	7.6	0.9	2.4	2.8	9.1	1.4	7.6
HIP1R	16.6	6.2	8.1	1.6	2.6	3.3	8.0	1.3	5.5
HIST1H3A	12.6	5.8	8.2	2.3	3.7	5.9	6.7	1.4	6.6
ZCCHC3	13.4	4.7	5.7	1.2	2.1	2.7	13.4	1.9	8.2
TRERF1	13.6	5.0	6.9	1.8	2.9	3.8	8.8	2.1	8.4
RPE	11.0	4.2	6.2	1.0	2.7	3.3	13.9	1.2	9.7
PPID	15.4	5.0	7.2	0.4	1.7	2.2	11.0	1.5	8.7
COLGALT2	21.1	6.8	8.9	1.5	2.2	3.3	5.2	0.6	3.5

POFUT2	15.3	6.7	8.7	0.9	2.2	2.8	8.2	1.5	6.8
TGIF2	11.0	5.1	6.3	1.9	2.8	3.6	10.1	2.0	10.1
RFNG	13.3	6.4	7.5	1.8	4.0	5.9	6.3	1.6	6.3
FZR1	14.9	5.4	7.3	2.0	3.2	4.0	8.8	1.5	5.8
LOC101928809	18.0	3.8	7.3	1.1	1.9	1.9	12.6	0.8	5.6
MRPS22	14.4	5.4	7.0	0.8	2.5	3.9	9.0	1.4	8.5
LOC101929078	14.2	5.2	6.4	1.4	2.6	3.1	11.3	2.1	6.6
GEMIN7	13.2	5.1	7.5	0.7	2.5	3.0	12.0	1.2	7.7
NAA16	16.6	3.6	6.7	1.0	1.9	2.8	12.4	1.4	6.6
PBX2	12.9	6.0	7.4	1.5	2.7	3.4	8.5	1.7	8.8
PIP5K1C	15.4	5.6	7.1	1.9	3.1	4.0	7.9	1.4	6.6
VPS29	12.5	4.8	6.2	0.7	2.8	3.3	11.6	1.2	9.8
UBR5	15.2	4.8	7.1	1.0	2.3	2.9	10.1	1.6	7.9
LOC100128239	16.8	3.4	8.2	1.0	1.8	2.3	11.6	1.5	6.3
TULP4	13.3	3.5	6.2	1.7	2.7	3.3	11.1	1.8	9.3
RAP2A	12.3	4.0	6.3	1.5	2.3	2.3	13.2	2.2	8.6
CDK7	13.8	5.6	7.3	0.4	2.4	3.0	9.3	1.6	9.3
CREBRF	14.9	4.9	6.9	0.5	1.8	2.3	11.3	1.3	8.9
COG8	17.2	5.9	7.3	1.6	2.7	4.0	7.1	1.2	5.8
ATXN7L3B	15.0	5.0	8.0	0.7	1.6	3.0	11.2	1.4	7.1
MUS81	12.5	5.3	7.2	1.4	3.2	4.1	9.8	1.5	7.7
ANKRD17	12.9	4.8	6.8	0.8	2.1	2.8	11.4	1.7	9.4
CAMK2N2	13.3	7.1	6.1	2.2	3.5	5.7	6.5	1.9	6.5
SDAD1	13.8	4.8	6.9	0.4	2.0	2.8	12.2	1.4	8.3
ELOVL6	18.8	6.3	8.7	0.5	1.6	2.6	7.2	0.7	6.0
RAPH1	13.8	4.0	6.0	1.7	3.3	4.4	8.8	2.0	8.8
HID1	19.4	7.3	10.5	0.6	1.2	1.4	6.5	1.0	4.6
HIRA	15.1	5.1	7.6	1.4	2.4	3.6	9.0	1.5	6.9
GMPS	12.2	4.7	5.6	0.4	2.6	3.3	12.3	1.7	9.9
DCPS	14.6	5.5	6.9	1.9	3.6	3.9	7.4	1.8	7.0
PRR7	14.0	5.6	7.7	3.6	3.7	6.2	5.7	1.5	4.6
ARRDC1	11.7	5.2	7.4	1.8	3.3	4.8	8.5	1.6	8.2
JMJD6	14.4	4.7	5.7	0.9	1.4	2.0	11.9	1.8	9.7
TNPO1	14.0	4.8	6.0	0.6	2.2	3.0	11.5	1.3	9.3
GOLGA7	14.1	5.6	9.3	1.2	1.8	3.6	8.7	1.4	6.8
PDF	11.1	3.6	5.7	1.3	1.6	3.4	15.3	1.9	8.5
BRF1	15.5	4.5	6.8	1.6	4.5	3.4	8.6	1.7	5.8
PSENEN	11.9	6.3	7.8	1.0	1.9	4.0	8.7	1.5	9.4
FLJ44255	17.6	3.0	7.2	1.1	1.9	2.2	12.7	1.0	5.8
PEX16	14.8	5.6	7.3	2.3	3.6	4.8	8.0	1.4	4.7
ACBD3	15.5	5.7	7.2	0.9	2.5	2.6	9.7	1.6	6.9
ZC3H15	12.3	4.5	6.8	0.4	1.5	2.3	13.7	1.2	9.8
MSTO1	12.5	4.5	6.9	1.3	2.7	3.1	10.5	1.9	9.0
IRAK2	20.0	7.2	10.0	0.9	1.8	2.1	6.0	1.0	3.6
AEN	10.6	4.2	5.3	1.2	3.0	3.5	12.2	2.0	10.5
NSMCE4A	13.7	5.5	7.3	1.4	2.7	4.1	10.1	1.3	6.4
GFOD2	13.8	4.9	5.6	0.9	1.7	2.2	14.5	1.6	7.1
ADAM10	17.3	6.1	7.7	0.6	2.3	2.8	8.4	0.9	6.3
RBM12	14.8	5.0	6.5	0.9	2.0	3.0	9.3	1.5	9.5
SIPA1	14.1	4.9	6.9	2.4	3.4	4.3	8.9	1.6	5.8
FZD6	15.5	5.6	7.1	0.6	1.8	2.6	10.3	0.7	8.1
FRY-AS1	16.8	3.3	7.3	0.8	2.2	2.4	12.6	1.1	5.9
ERI3	14.5	5.3	7.9	0.9	3.1	3.2	8.4	1.2	7.7
VPS35	13.5	5.7	7.4	0.5	2.1	2.7	10.3	1.5	8.6
PLLP	18.5	8.2	9.2	2.1	3.0	5.0	3.5	0.5	2.4

LOC101929352	16.8	3.8	7.4	0.6	1.8	2.1	12.8	0.9	6.0
SNX33	15.2	6.1	7.6	1.3	3.1	4.4	7.5	1.3	5.7
TBC1D2B	15.8	5.5	7.4	1.0	2.2	2.4	9.4	1.6	7.0
SMU1	12.8	5.2	7.3	0.5	1.7	1.9	12.5	1.2	9.2
CDC73	14.0	4.1	6.2	0.6	2.0	3.0	11.3	1.7	9.3
NSMCE1	11.6	4.5	6.3	1.7	2.9	4.2	12.0	1.5	7.6
RRAS	15.6	7.3	9.8	1.5	4.1	4.2	4.8	0.9	4.0
PDP1	14.5	5.8	7.3	0.9	3.3	4.0	7.6	1.4	7.5
FKBP9	20.1	7.2	9.0	1.2	2.2	3.2	5.1	0.6	3.5
CCDC94	13.9	6.2	7.0	1.8	2.7	3.8	7.2	2.0	7.6
DCAF13	11.4	4.6	6.7	0.5	2.0	3.0	11.6	1.6	10.7
RNF4	16.0	4.4	5.6	1.0	1.7	1.9	12.9	1.2	7.6
HMGXB3	14.1	5.3	6.9	0.9	2.1	3.0	10.4	1.5	7.9
NCBP2-AS2	14.6	5.3	7.2	1.0	2.4	3.2	9.6	1.7	7.1
POLR3H	14.2	4.8	6.3	1.3	2.3	3.2	10.3	1.8	8.0
UROS	14.7	6.5	8.1	1.3	3.3	5.6	6.6	0.6	5.3
NCF2	14.3	5.9	7.9	1.2	2.5	4.3	7.8	1.8	6.5
PYGO2	14.0	6.7	7.0	2.1	3.5	5.1	6.3	1.2	6.3
DPH7	13.9	5.1	7.8	1.1	2.8	3.3	9.5	1.5	7.1
ACBD6	14.0	5.3	6.6	0.5	2.9	2.8	10.5	1.4	8.2
LOC100130451	16.0	3.8	8.7	1.4	3.0	2.7	10.9	0.8	4.8
DSG2	13.9	5.5	6.9	0.4	1.5	2.1	11.2	1.5	9.1
RXRA	14.1	5.4	7.4	2.2	3.3	5.0	6.4	1.7	6.5
ALKBH3	14.1	3.7	5.8	0.9	2.2	2.8	11.8	1.4	9.3
FAM195A	16.2	4.9	9.2	1.9	2.9	3.4	7.9	0.7	4.9
TXNDC17	11.5	6.2	7.7	0.9	2.0	3.6	10.4	1.8	7.9
PATZ1	11.8	4.5	5.0	1.6	2.8	3.5	11.2	2.2	9.3
RNF31	14.2	5.3	6.8	1.7	2.6	4.2	8.8	1.4	6.9
TMEM245	14.8	4.8	7.2	1.2	2.2	3.0	10.4	1.4	6.9
NAP1L4	13.7	4.8	6.4	0.4	2.2	2.5	12.2	1.6	8.1
CSTF2	12.8	4.8	6.0	1.1	2.8	3.9	10.3	1.7	8.6
CDK12	13.8	4.4	6.0	1.1	2.6	3.5	9.8	1.7	9.0
LOC101927340	17.0	3.7	8.5	0.6	1.3	1.8	11.9	0.9	6.1
TMEM25	17.9	6.4	8.7	1.0	2.5	3.8	5.8	1.0	4.8
HAGHL	14.1	6.0	8.5	2.8	4.1	5.7	6.0	0.9	3.7
AVPI1	13.8	5.2	6.2	1.0	2.4	3.0	10.4	1.8	7.9
DTX2	13.3	5.8	6.8	1.6	2.8	3.3	8.7	2.0	7.4
ICMT	14.6	4.8	5.8	1.8	2.8	3.0	11.1	1.1	6.8
MZF1	12.5	4.2	6.4	2.2	3.4	4.5	9.9	2.1	6.6
RNF145	17.7	6.2	7.8	0.3	1.4	2.0	9.2	1.0	6.0
DDX10	13.1	4.8	6.1	0.4	1.9	2.4	12.0	1.5	9.6
HOMER3	15.8	6.0	8.3	1.6	2.6	3.5	7.6	1.3	5.0
TXNDC12	13.2	4.8	6.0	0.8	2.3	3.0	11.5	1.6	8.6
TCF12	13.2	4.1	6.0	0.6	1.8	2.9	11.6	1.5	9.9
FEZF2	3.4	8.4	6.2	12.0	4.3	0.4	6.1	6.3	4.5
TUSC2	14.0	4.1	6.6	0.7	1.9	1.7	13.2	1.8	7.5
ZFYVE19	16.0	5.7	8.2	1.3	3.2	3.7	6.5	1.0	6.0
STX8	14.5	5.7	8.8	0.9	2.7	4.2	7.4	1.5	5.9
TEAD3	13.6	5.4	6.4	1.5	2.9	3.5	8.1	2.2	7.9
ARL16	12.2	5.3	8.1	0.9	3.0	3.2	9.6	1.5	7.7
UBE3C	12.8	4.5	6.0	0.6	2.2	2.9	11.9	1.6	9.2
MAPKAP1	14.3	4.9	6.6	0.7	2.8	4.1	9.2	1.3	7.8
MPC2	16.3	4.2	7.5	1.1	2.0	2.9	11.1	1.1	5.2
CDC26	14.1	6.5	7.1	0.5	1.5	1.5	10.0	1.8	8.6
EDN1	6.0	3.6	4.1	0.7	1.8	3.0	15.9	3.1	13.4

POLM	13.7	5.8	6.6	1.1	2.3	2.6	10.2	1.7	7.5
ZMIZ2	12.9	5.4	6.4	2.2	3.1	5.1	7.7	1.9	7.0
GPSM1	13.6	5.2	6.5	1.8	3.0	3.5	8.9	2.0	7.1
DLG5	16.6	5.4	7.5	1.1	2.5	3.1	8.0	1.2	6.1
FEM1A	11.7	4.3	5.6	2.0	2.9	4.0	10.4	2.0	8.5
ZDHHC4	17.2	5.9	7.5	0.8	2.5	3.1	8.0	1.1	5.4
LOC100507236	16.3	3.4	7.1	0.9	1.7	2.1	13.4	0.9	5.7
KATNB1	11.2	4.8	6.0	2.8	4.8	6.4	7.6	1.5	6.3
CDK5RAP1	16.5	6.3	8.9	0.5	1.6	2.5	7.8	0.9	6.5
RAB21	11.4	6.8	6.0	1.8	2.3	2.7	7.8	2.7	9.8
UCKL1-AS1	16.6	3.8	7.8	1.0	1.8	2.0	11.6	1.1	5.6
PIGS	16.8	6.2	8.5	0.9	2.7	3.3	7.0	1.0	5.0
ZNF696	11.4	3.9	6.1	1.8	2.8	3.7	12.0	1.8	7.8
HIST1H3J	10.4	3.5	5.0	2.1	3.4	5.7	11.0	2.1	8.0
BDNF	9.9	3.5	5.4	1.0	2.4	3.5	12.3	2.4	10.8
NIPSNAP1	16.5	7.0	9.0	1.3	2.3	3.9	5.9	0.9	4.5
PKP2	14.0	4.9	6.8	1.0	1.8	2.3	10.6	1.5	8.4
EMC10	16.2	5.1	8.4	1.5	2.6	4.4	8.1	0.8	4.4
NXPH4	10.1	4.6	4.4	1.2	2.5	3.9	11.0	2.2	11.4
DERL2	14.5	4.6	6.4	0.8	1.5	2.0	10.5	1.3	9.6
ENTPD4	12.9	5.0	5.8	0.6	2.4	3.7	11.4	1.0	8.3
POLR3K	10.9	5.2	5.5	0.8	2.1	3.5	12.0	2.0	9.2
CSTF1	14.8	6.3	7.4	1.0	2.7	3.3	7.6	1.5	6.6
C8orf76	12.4	5.2	9.1	0.5	1.6	2.8	10.0	1.2	8.3
ADAMTS12	13.5	4.8	6.7	1.2	2.9	4.1	9.6	1.4	7.0
HS1BP3	16.2	6.0	9.7	1.2	3.4	3.6	6.1	1.1	4.0
IFNGR2	14.5	6.5	7.5	1.2	2.1	4.2	6.5	2.0	6.6
EPB41L2	14.3	4.7	6.5	1.0	2.4	3.0	8.9	1.5	8.8
RASA3	11.6	4.4	5.7	1.8	3.5	5.1	9.0	1.7	8.2
HECTD1	14.6	4.5	5.8	0.4	2.0	2.4	12.0	1.2	8.2
CNOT7	12.9	5.3	6.5	0.3	1.2	2.1	12.7	1.3	8.9
ZSWIM8	16.2	6.2	9.4	1.6	2.9	3.7	5.7	1.0	4.4
RLIM	13.7	4.4	6.2	0.5	1.9	2.8	12.3	1.0	8.4
TCERG1	13.9	4.9	6.8	0.9	1.8	2.7	9.5	1.6	8.8
HNRNPUL2-BSCL2	13.6	4.9	6.2	1.8	2.7	3.4	9.9	1.7	6.9
IGDCC4	15.1	5.5	8.2	1.4	2.3	3.1	8.2	1.4	5.9
TNFRSF10A	13.3	5.3	6.7	0.9	2.9	2.7	9.0	1.6	8.6
PDRG1	12.4	4.7	7.3	0.8	1.3	2.0	12.1	1.7	8.5
TIGD5	12.4	4.8	6.0	1.6	2.7	3.4	11.3	1.6	7.1
SNX18	13.7	5.5	6.7	2.1	2.8	4.3	8.0	1.4	6.4
STARD10	14.8	5.8	8.6	1.0	2.2	2.7	7.5	1.8	6.4
GATA6	13.0	5.7	6.9	2.6	3.2	4.7	7.4	1.5	5.9
TRUB2	14.7	6.0	7.0	1.1	2.5	3.9	8.5	1.5	5.7
ADSS	13.1	5.8	7.0	0.8	1.9	2.7	11.2	1.1	7.3
SCP2	15.7	6.6	9.0	0.4	2.0	2.4	8.6	0.9	5.3
GPX8	12.7	4.7	6.5	0.5	1.6	2.7	11.3	1.7	9.0
ARHGAP17	14.2	5.1	5.7	1.2	2.4	3.3	9.3	1.6	8.0
ATP2C1	13.3	4.3	5.9	0.4	1.8	2.6	12.3	1.1	9.0
HTRA1	18.9	6.4	9.2	1.6	2.8	3.6	4.4	0.6	3.3
TOM1	16.1	5.2	8.3	1.6	2.4	3.1	7.8	1.3	4.9
IDH3G	12.9	5.8	7.3	2.1	4.2	5.0	6.2	1.2	6.1
FIP1L1	15.2	5.9	7.3	1.0	2.5	3.3	7.7	1.4	6.6
TAOK2	14.9	5.6	7.5	1.4	2.2	3.7	7.9	1.5	6.1
CASP2	14.3	3.9	5.5	0.8	1.7	1.9	12.4	1.3	9.0
SNHG15	8.8	3.4	4.7	1.1	2.0	2.8	14.9	2.4	10.8

DCTN6	14.6	7.3	7.5	0.7	2.1	2.6	7.7	1.4	6.9
TOR1B	14.6	5.2	7.0	0.7	1.9	1.7	11.5	1.5	6.7
LINC00092	16.5	3.3	6.9	0.9	1.9	2.2	12.7	0.9	5.3
MAPRE2	14.0	5.1	6.1	0.8	2.1	2.9	9.4	1.7	8.7
C19orf52	12.6	4.5	5.8	1.2	2.5	2.4	13.0	1.2	7.5
PTPRJ	15.8	5.8	7.5	1.5	3.1	4.5	6.1	0.9	5.4
KMT2B	14.2	5.0	6.9	1.4	2.6	3.2	8.8	1.8	6.7
PRR11	14.6	4.3	7.1	0.9	2.4	2.9	11.3	1.1	6.0
FLJ31104	15.6	3.3	7.2	0.7	1.7	2.4	12.8	0.9	5.9
PAICS	12.8	5.3	5.2	0.4	1.8	2.3	12.6	1.4	8.9
PTPN12	13.7	4.9	6.1	0.5	2.2	3.1	9.0	1.7	9.3
HIST1H2AJ	8.2	4.7	6.2	2.6	4.2	7.3	6.4	2.1	8.8
TSPAN17	13.4	6.4	7.9	0.9	2.5	3.9	7.5	1.2	6.9
HSD17B1	13.2	5.1	6.9	1.9	2.7	3.4	9.2	1.6	6.5
KDM1A	13.8	4.9	6.2	0.7	1.9	2.7	11.0	1.4	7.8
LOC100134868	15.4	3.3	7.1	1.0	1.8	2.8	12.4	1.2	5.5
PFAS	11.7	3.2	6.1	1.2	2.3	3.2	12.4	1.5	8.8
AP5Z1	16.6	5.7	7.7	1.7	2.7	3.7	6.8	1.1	4.3
GTF2A1	11.9	4.5	6.0	1.3	3.4	4.4	7.2	1.7	10.0
IFFO2	14.5	5.1	6.5	1.6	2.1	2.7	8.2	2.0	7.7
CCSER2	15.4	4.9	5.9	0.6	1.7	2.6	9.6	1.1	8.4
API5	13.1	5.1	6.5	0.6	2.1	2.7	10.3	1.2	8.8
FAM221A	16.4	3.7	7.1	0.8	1.9	2.4	11.4	1.0	5.6
OSBPL3	13.9	4.5	6.2	0.7	1.6	2.2	11.1	1.8	8.2
CD40	11.3	5.2	6.1	1.5	2.6	4.8	9.2	1.9	7.7
CHCHD4	13.0	5.0	5.3	0.5	2.4	2.8	10.0	1.6	9.7
ATG12	13.4	4.9	6.8	0.6	2.1	2.7	9.0	2.0	8.8
EDC3	13.5	5.1	6.7	1.0	2.0	3.0	8.8	1.7	8.4
KIF2A	13.5	5.2	6.2	0.4	1.9	2.8	10.5	1.4	8.4
SLC25A32	12.6	3.4	5.7	1.0	2.1	3.2	12.9	1.3	7.9
ABHD12	16.8	6.4	7.9	1.1	2.7	3.9	5.8	0.9	4.7
MRPL22	13.8	5.2	8.5	0.5	2.7	3.1	7.3	1.6	7.4
PIAS3	15.8	5.7	8.4	1.0	2.3	2.9	7.4	1.2	5.6
TARS2	15.1	4.8	7.5	0.8	2.0	3.1	10.1	1.1	5.7
TMEM185B	12.3	3.9	5.5	1.3	2.7	4.2	9.5	1.6	9.1
XPO7	13.4	4.6	6.5	0.8	2.4	3.1	9.0	1.5	8.9
BRD8	15.5	5.0	7.6	0.9	2.4	3.0	8.1	1.2	6.4
AAGAB	12.1	4.5	5.3	0.5	1.1	2.1	12.4	1.3	10.7
LLGL1	14.3	5.3	7.0	1.9	3.5	4.5	7.6	1.0	4.9
ZNF185	10.6	2.8	3.9	0.9	2.5	4.3	10.7	1.7	12.7
METTL5	13.9	5.2	5.3	0.2	1.7	3.0	11.3	1.3	8.0
GLI4	14.2	5.5	8.2	2.6	3.5	4.7	5.6	1.2	4.5
NABP2	12.2	5.6	6.9	1.9	3.4	3.7	7.9	1.7	6.6
DAB2	15.3	6.1	7.0	0.6	1.8	2.8	7.2	1.4	7.8
IP6K1	12.7	4.8	6.2	1.0	2.0	2.8	9.9	2.3	8.4
RAB32	14.3	6.0	10.0	0.9	2.4	3.0	6.8	1.3	5.5
DENND1A	13.3	4.2	5.1	1.2	2.0	2.9	11.2	1.7	8.2
ZZZ3	11.7	4.3	5.7	0.6	2.0	2.9	11.3	1.5	10.2
ZNF276	13.2	5.0	7.5	1.4	2.7	3.6	8.8	1.4	6.5
LOC101927918	17.6	3.6	7.4	0.8	1.1	2.4	11.1	1.1	4.9
MYO1D	15.6	5.8	7.0	0.6	1.6	2.2	9.1	1.2	6.9
SNRNP27	12.3	5.1	6.1	1.1	2.4	3.4	9.4	1.7	8.5
RANGRF	12.7	4.6	6.5	1.4	3.1	3.2	10.0	2.0	6.4
RAB5A	13.3	4.3	6.5	0.3	2.1	3.1	10.4	1.6	8.5
PLRG1	12.1	4.5	5.9	0.3	1.9	2.6	11.8	1.5	9.4

ALPP	10.9	4.5	6.0	1.2	2.0	2.4	11.2	2.3	9.3
EDEM2	13.4	5.8	7.1	1.4	1.9	3.0	8.7	1.6	7.0
UGDH	14.6	5.3	6.5	0.2	1.6	1.9	11.4	1.1	7.2
FAM219B	13.1	4.6	8.2	1.0	2.5	2.9	9.5	1.3	6.8
PPIH	11.7	4.8	5.5	0.4	1.8	2.4	12.0	1.4	9.9
CDK16	12.3	5.0	7.0	1.7	3.5	4.2	7.3	1.7	7.1
RNPEPL1	13.8	5.5	7.0	2.3	3.0	4.3	7.0	1.3	5.7
INSR	14.0	4.5	6.0	0.9	2.1	2.8	10.4	1.4	7.6
FPGS	10.0	3.8	5.7	2.0	4.0	5.1	10.6	1.4	7.3
MNAT1	12.3	5.1	7.0	0.5	1.9	2.6	11.3	1.0	8.1
MLLT10	13.2	4.1	5.7	1.2	2.7	2.9	10.0	1.3	8.8
RBM10	13.4	5.0	6.6	1.6	2.7	3.9	8.0	1.5	7.0
CLPB	13.9	4.7	6.0	1.1	2.7	3.5	9.1	1.5	7.2
ANKRD33B	11.4	3.5	5.0	1.8	2.7	3.8	11.4	2.1	8.1
KLF11	22.1	8.5	9.9	0.5	0.9	1.3	3.4	0.6	2.6
SLC39A6	13.2	5.0	6.1	0.5	1.7	2.6	9.8	1.6	9.3
ARHGAP18	11.4	3.9	5.2	0.7	2.1	2.8	12.6	1.3	9.5
DRG1	11.9	4.6	7.4	0.4	1.8	2.4	12.2	1.4	7.6
YIPF5	14.5	7.0	7.1	0.5	1.7	2.7	8.4	1.3	6.4
ARG2	6.9	3.2	4.4	0.6	1.6	2.1	16.8	2.1	12.0
CHST15	17.0	6.5	8.2	1.4	2.7	4.0	4.9	1.0	4.0
JAK1	13.1	4.4	5.2	1.0	2.4	2.7	10.8	1.4	8.7
ARHGEF17	15.5	6.0	8.6	1.5	2.3	3.1	7.1	1.2	4.4
ABL1	12.5	4.4	5.8	1.3	2.6	3.3	10.5	1.6	7.6
NFIB	13.6	4.9	5.9	0.8	2.2	2.7	9.8	1.4	8.3
SNRNP40	11.9	4.2	6.5	0.8	1.8	3.1	12.0	1.3	8.0
ACAT2	11.1	5.0	7.4	0.3	1.4	1.9	12.8	1.3	8.3
DUSP12	11.3	4.6	6.5	1.3	1.9	2.7	11.7	1.9	7.7
LOC101927875	16.5	3.1	7.1	0.9	2.0	2.1	12.0	0.9	5.0
C1orf174	10.8	4.9	6.2	0.9	2.5	2.9	10.7	1.9	8.7
DOCK5	14.7	4.5	6.3	1.1	3.1	4.1	7.8	1.1	6.8
LOC100129637	15.1	3.6	6.8	1.2	2.0	2.4	11.1	1.2	6.3
EGFL7	10.1	7.4	8.5	1.9	3.4	5.5	5.8	1.4	5.6
JMJD1C	13.6	5.0	7.1	0.6	1.9	2.6	8.8	1.4	8.7
PRDM8	14.1	5.6	7.2	1.6	2.4	2.8	9.0	1.2	5.5
NFYA	12.1	4.8	6.8	1.1	2.6	4.3	8.2	1.9	7.7
PPP1R15B	11.8	4.3	5.9	0.7	1.5	2.0	12.4	1.7	9.2
TP53BP2	10.8	3.7	4.7	0.6	1.9	2.7	12.3	2.1	10.7
IFT57	13.4	5.6	6.8	0.6	2.3	3.1	9.1	1.2	7.4
ABI1	13.5	4.6	6.3	0.8	2.1	3.0	9.0	1.3	8.9
NASP	11.6	4.6	5.7	0.7	2.2	2.7	10.8	1.6	9.7
UHMK1	14.0	5.0	7.2	0.6	1.4	2.5	11.4	1.2	6.0
WBP1L	15.0	6.0	8.0	1.6	2.3	2.8	6.0	1.7	6.1
DCAF4	12.3	4.9	6.5	1.3	2.8	4.2	8.9	1.1	7.4
SOWAHC	8.9	3.7	4.2	1.2	2.2	3.1	12.9	2.7	10.5
SFSWAP	14.0	5.1	5.9	1.3	2.2	2.8	8.7	1.8	7.6
MYLK	16.2	5.9	7.3	1.5	4.4	4.4	5.1	0.8	4.0
ZNF146	10.3	3.5	5.1	0.3	1.5	2.5	13.5	1.1	11.7
ABI2	13.6	5.1	6.8	1.0	3.0	3.7	7.4	1.5	7.1
FH	12.1	4.8	6.2	0.9	2.8	3.6	9.9	1.4	7.7
C4BPB	15.4	3.4	6.9	1.2	2.4	2.4	11.7	0.8	5.3
SELO	16.0	5.5	8.0	1.5	3.0	3.4	6.8	1.0	4.1
FZD7	12.7	4.7	5.2	2.0	2.8	4.0	9.7	1.6	6.6
STX16	15.1	4.3	6.6	1.1	2.5	2.6	10.1	0.9	6.2
CLK2	14.5	6.2	8.1	1.0	2.0	3.0	6.8	1.3	6.4

UBN1	15.2	5.4	6.4	0.8	1.6	2.5	9.7	1.3	6.5
NDUFAF3	9.1	4.4	4.8	1.9	3.9	5.2	10.8	1.7	7.7
FAM199X	10.7	4.3	5.0	0.8	2.1	3.0	11.5	2.2	9.7
DNAJB2	12.2	5.6	6.9	1.2	2.2	3.0	8.6	1.9	7.7
TRIM41	13.8	5.6	7.3	1.5	3.2	4.0	7.1	1.3	5.5
MIRLET7BHG	14.8	4.6	7.8	1.3	2.0	2.5	9.8	1.1	5.4
ANKLE1	16.3	4.0	6.9	0.8	1.9	2.4	10.7	1.0	5.2
PUS1	11.9	4.2	6.0	1.8	2.3	3.3	10.3	1.7	7.8
MCM5	10.8	5.0	5.0	2.1	3.2	4.6	8.1	1.9	8.4
REPS1	12.2	4.2	5.5	0.9	1.9	2.4	11.0	2.0	9.1
C14orf169	10.6	4.1	5.3	1.7	2.7	3.7	11.2	2.1	7.9
LIMK1	16.7	6.1	7.8	1.4	2.6	4.1	5.8	1.0	3.7
RBM38	12.1	5.2	6.5	2.1	2.2	4.5	7.3	2.4	6.8
C1orf85	15.3	5.9	7.1	1.6	3.2	4.6	5.1	1.4	5.0
GAK	14.9	5.3	7.4	1.5	2.3	3.4	7.1	1.2	5.9
SLC7A6OS	12.8	4.9	7.0	1.2	2.9	2.8	9.9	1.2	6.5
TCF20	12.1	4.3	5.6	0.7	1.9	2.6	9.9	1.8	10.2
HDGFRP3	11.8	5.5	7.2	1.3	2.4	4.0	7.9	1.7	7.4
METRNL	8.3	3.9	4.5	2.6	3.8	5.8	10.3	1.9	8.2
CYB5R1	12.6	4.7	6.6	1.3	2.1	4.1	9.8	1.1	6.9
GADD45A	12.9	5.2	7.0	1.5	2.0	3.2	9.9	1.6	5.8
TBCEL	14.2	5.2	6.6	0.4	1.6	2.2	10.2	1.4	7.3
TTL	10.3	3.8	6.2	1.1	2.5	3.1	9.8	2.0	10.4
AKAP10	14.3	5.1	7.3	0.7	1.8	2.7	9.5	1.1	6.6
UNK	15.0	5.1	8.0	1.7	2.6	3.8	6.5	1.3	5.1
BRD9	12.5	4.4	5.7	1.4	2.5	3.4	10.5	1.4	7.4
MICALL1	12.4	4.7	6.2	1.9	2.8	4.2	8.3	1.8	6.8
SMYD5	15.1	5.1	7.2	0.9	2.6	3.1	8.4	0.8	5.7
NDUFA9	13.5	5.2	8.6	0.7	2.0	3.0	9.0	1.0	6.1
BACE2	15.0	5.2	6.4	1.0	2.1	2.9	8.8	1.1	6.5
NDUFA5	12.9	5.4	7.6	0.5	2.4	2.6	9.5	1.2	7.0
CCDC122	16.6	3.2	7.4	0.7	2.0	2.3	10.6	0.9	5.3
CPA4	14.0	5.4	8.4	1.3	4.1	4.7	6.1	1.0	4.1
SELRC1	10.5	4.3	5.2	0.6	1.4	2.1	12.4	1.8	10.7
B3GNT1	17.3	5.9	9.1	1.5	2.6	3.2	5.9	0.7	2.9
PRPS1	13.7	5.0	6.5	0.5	2.0	2.4	10.1	1.0	7.7
C1orf122	12.6	5.1	6.5	1.4	2.7	3.0	10.0	1.0	6.6
KIAA0226	14.4	4.9	6.5	1.1	2.4	3.1	8.4	1.6	6.5
PHF12	13.7	5.0	6.6	1.1	2.4	3.2	7.7	2.0	7.2
CCND3	11.9	4.9	7.4	1.5	3.2	4.0	7.5	1.8	6.7
FKBP3	13.6	5.1	6.5	0.6	2.9	4.0	8.0	1.3	7.0
DOCK1	13.4	4.4	6.2	1.0	2.6	3.2	9.2	1.4	7.5
HIST1H2BE	8.5	3.6	5.9	2.6	5.9	7.3	5.4	1.9	7.7
MOK	16.5	3.4	6.4	1.3	2.5	3.5	9.7	0.9	4.7
URI1	13.3	4.6	5.7	0.9	1.8	2.4	10.6	1.6	8.0
PRR3	13.0	4.3	5.8	1.2	2.8	3.9	8.1	2.1	7.6
ZBTB45	11.7	5.0	6.4	2.4	2.9	4.3	7.9	1.8	6.5
CYSTM1	14.0	5.7	6.4	0.4	1.9	3.0	8.1	1.7	7.5
FCHSD1	12.9	5.6	7.0	1.1	2.3	2.9	9.3	1.3	6.4
FBXL18	13.0	4.6	6.2	2.0	2.5	3.9	8.3	1.9	6.5
EIF4EBP2	13.0	6.0	6.7	2.3	2.4	4.8	4.8	2.1	6.8
UNC119	16.6	5.9	7.4	1.3	2.0	2.5	6.3	1.0	5.6
PHC3	15.8	4.9	7.3	0.8	2.5	3.1	6.4	1.1	7.0
SEMA6B	13.8	6.0	7.9	1.8	2.7	3.4	6.7	1.4	5.0
TMPPE	13.0	6.9	5.8	1.3	2.9	3.3	7.3	1.5	6.7

ARPC5L	12.5	4.3	7.5	1.3	2.9	3.4	6.8	2.3	7.8
SKA1	15.9	3.2	6.6	0.9	1.9	2.4	11.7	1.0	5.1
PSMD12	13.4	4.4	5.3	0.5	2.8	2.4	9.9	1.3	8.8
GCNT2	14.9	5.0	6.2	1.0	2.4	3.8	8.4	1.1	5.9
KIAA0319L	16.2	4.5	7.5	1.1	2.6	3.3	8.0	0.9	4.6
E2F1	9.5	3.3	4.2	1.8	3.5	4.7	10.7	1.8	9.0
LOC101929017	15.8	3.2	6.9	0.8	1.8	2.1	11.7	0.9	5.4
NPRL2	12.7	5.1	6.2	1.3	2.4	2.5	9.5	1.4	7.6
SCO2	12.0	4.1	6.7	1.1	2.2	2.5	11.0	1.9	7.0
C1orf52	14.0	4.8	7.9	0.8	1.8	2.8	8.0	1.6	6.8
RNU6-2	2.9	20.9	11.9	0.7	4.6	0.0	3.5	0.5	3.5
PRR24	13.4	5.3	7.0	1.8	3.1	3.9	8.1	0.7	5.2
PACS1	14.8	4.8	6.9	1.9	3.2	4.1	6.5	1.2	5.0
PDHB	14.0	5.3	7.5	0.6	2.2	2.9	8.8	1.3	6.0
TPRN	12.0	4.5	5.5	1.5	2.3	2.9	10.1	2.0	7.8
DEXI	13.1	5.5	7.2	0.8	1.9	2.5	8.9	1.8	6.8
C9orf16	16.5	6.5	6.4	2.7	3.1	2.9	4.9	1.0	4.5
FTX	15.0	4.0	7.3	0.9	2.5	2.6	9.0	1.3	6.0
TSNARE1	14.2	5.2	7.3	2.5	3.6	4.4	5.8	1.2	4.3
GRAMD1A	13.4	4.5	6.0	1.9	2.8	4.4	8.2	1.5	5.7
RNASEH1	12.3	4.8	5.4	1.0	2.3	3.3	10.4	1.2	7.9
PDE8A	14.6	5.3	7.3	0.9	2.6	3.2	7.3	1.1	6.1
MRPS17	12.2	5.8	6.1	0.4	2.0	3.5	9.8	0.9	7.8
ATF1	12.2	5.2	5.9	0.7	1.8	2.7	9.7	1.6	8.7
RAB12	13.6	5.4	6.0	1.0	1.9	3.6	8.1	1.3	7.5
IKBKB	14.7	4.9	8.6	0.8	2.2	2.5	7.7	1.0	6.0
ARGLU1	12.4	3.9	6.3	1.2	3.0	4.0	9.7	1.2	6.7
SFXN1	13.3	4.8	6.8	0.5	2.3	2.4	9.6	1.2	7.6
MLLT4	13.2	4.0	6.6	1.2	2.4	3.0	9.7	1.2	7.1
AMBRA1	15.2	4.1	6.5	1.2	2.1	2.7	9.5	1.2	5.9
DNPH1	13.5	4.4	7.1	2.6	5.0	6.1	6.0	1.0	2.5
MPV17	13.0	5.4	7.4	0.8	3.7	3.8	8.1	0.9	5.3
HTR7	11.1	3.4	5.4	1.5	2.4	2.9	12.3	1.4	8.0
FUT11	14.7	4.8	6.8	1.3	2.5	2.8	7.9	1.9	5.6
POM121	13.0	5.0	6.5	1.5	2.4	3.7	7.5	1.6	7.2
SNORA65	10.5	9.3	6.3	2.6	2.1	2.5	7.7	2.7	4.6
PEX14	13.1	5.5	7.2	1.3	2.7	3.8	7.5	1.5	5.7
CAMKK2	12.3	4.8	6.3	1.4	2.8	3.7	9.5	1.3	6.2
EIF1B	11.8	4.7	6.6	0.2	2.4	1.9	9.4	2.0	9.2
METTL13	13.3	4.4	5.7	0.8	1.8	2.5	11.4	1.1	7.2
CCNH	11.3	4.1	5.1	0.5	2.5	3.0	10.3	1.6	9.7
ZBTB18	12.4	5.2	6.6	1.1	2.5	3.5	8.3	1.6	7.0
C6orf62	8.3	3.8	4.8	0.4	1.5	1.9	16.1	1.4	9.9
LINC00507	16.1	3.1	7.8	0.9	2.8	2.7	8.1	0.8	5.8
ZNRF3	4.5	8.1	6.2	6.1	4.1	1.6	7.0	5.9	4.7
ATF5	11.3	4.7	5.8	1.6	2.9	4.3	8.9	1.8	6.8
FBXW2	12.9	4.3	6.1	0.6	1.8	2.4	11.3	1.1	7.5
KCTD2	14.4	5.1	6.7	1.1	2.1	2.5	9.0	1.4	5.9
WDR77	10.7	3.9	6.0	1.0	1.9	2.7	12.5	1.6	7.9
ZNF24	14.0	3.9	6.3	0.4	1.6	1.7	12.3	0.7	7.1
TOB1	13.7	5.0	6.9	0.6	2.0	3.0	7.1	1.6	8.1
ELP5	10.4	4.1	6.4	1.2	2.8	4.3	9.5	1.7	7.7
PI4KA	14.4	5.3	6.5	1.2	2.8	2.8	7.5	1.3	6.3
IRGQ	13.5	4.5	6.8	1.7	2.7	3.3	9.1	1.2	5.2
TONSL	11.6	4.5	6.5	2.4	3.5	5.1	7.4	1.4	5.6

LAMC2	17.3	6.3	8.3	0.8	2.0	2.7	5.6	0.6	4.3
NOL3	13.3	5.5	7.2	1.1	2.2	3.5	9.1	1.2	4.8
PCCB	11.7	5.5	8.2	1.2	2.7	3.5	7.7	1.3	6.2
ARHGAP21	12.8	4.2	5.7	0.9	2.0	2.9	9.2	1.6	8.6
ROCK2	12.3	3.7	5.3	0.3	1.9	2.2	11.5	1.5	9.2
LOC101927796	15.1	3.2	7.2	0.8	1.8	1.9	11.6	0.9	5.4
IFT46	12.5	5.3	7.9	0.9	1.9	3.1	7.9	1.5	6.8
ANKRD13C	13.1	4.9	6.2	0.8	2.5	2.9	9.0	1.3	7.2
NRBF2	12.0	4.8	7.0	0.8	2.0	3.1	8.7	1.2	8.4
GNPNAT1	9.3	3.7	5.7	0.3	1.6	1.6	13.3	1.7	10.8
LOC100129361	8.8	5.6	5.7	2.7	2.6	5.6	6.5	3.2	7.2
TANC2	15.4	4.8	6.9	1.0	2.3	2.9	6.9	1.3	6.3
DLG4	12.6	4.2	5.7	1.2	2.2	3.1	9.3	1.6	7.7
SERPINB1	15.3	5.1	8.0	0.8	2.3	3.1	7.9	0.7	4.6
LOC101929767	14.5	3.8	6.0	2.5	2.4	4.2	6.5	2.0	6.0
DNTTIP2	10.9	4.3	6.3	0.4	1.9	2.3	10.4	1.7	9.6
RBM7	13.1	5.8	6.3	0.5	2.3	2.8	6.3	1.7	9.0
ANKRD52	13.4	4.1	5.8	1.1	2.4	3.1	9.4	1.4	7.0
KIAA0754	13.0	3.9	5.6	1.7	2.6	3.3	9.1	1.7	6.9
ROBO4	9.0	3.9	4.5	1.7	3.1	4.1	10.6	2.3	8.5
HIST1H2BB	11.2	5.3	7.0	1.9	3.5	5.4	5.4	1.3	6.7
NAA25	13.2	4.3	5.4	0.2	1.6	1.8	11.7	1.2	8.3
BOLA3	13.8	4.5	8.1	1.0	2.5	3.2	6.5	1.1	7.0
RTN2	15.9	6.4	7.3	1.4	2.2	3.2	5.8	1.0	4.3
TM2D1	14.6	5.6	8.5	0.6	1.5	2.9	7.4	1.0	5.5
LOC101927063	15.6	3.2	6.6	0.8	1.9	2.3	11.3	0.9	5.1
UNC93B1	14.7	6.0	6.9	1.6	2.7	3.8	7.0	1.1	3.9
NUP98	12.6	4.6	5.3	0.6	2.1	2.3	9.9	1.6	8.6
GHDC	14.5	5.5	7.3	1.9	3.2	4.2	6.1	0.9	3.9
PTTG1	9.6	4.9	5.6	0.6	1.7	3.4	11.3	2.2	8.3
C19orf54	14.4	4.8	7.5	1.2	2.1	2.8	8.2	1.0	5.5
SMG7	11.0	4.7	5.1	0.8	2.4	3.2	8.0	2.2	10.2
CAPZA2	11.8	4.9	8.1	0.3	1.1	2.0	10.7	1.1	7.5
MFAP1	12.9	5.1	6.7	0.7	1.5	2.4	9.3	1.8	7.2
SLTM	13.0	4.7	5.6	1.2	2.5	3.6	8.2	1.3	7.5
RAB3GAP1	12.8	4.7	5.5	0.4	2.0	2.3	10.8	1.3	7.7
VPS33A	12.0	5.0	6.2	0.6	1.8	2.6	10.9	1.3	7.1
GTF3C1	14.2	4.7	6.6	1.1	2.3	3.3	8.5	1.3	5.6
SIL1	14.1	5.1	7.0	1.5	2.6	3.6	7.1	1.2	5.2
UBE2D1	15.0	6.7	7.8	0.4	1.4	2.6	6.1	1.0	6.4
LINC00923	15.7	4.1	7.2	0.8	2.4	2.6	7.9	0.8	5.8
HIPK3	13.3	4.7	7.2	0.6	1.5	2.0	9.5	1.5	7.2
RPIA	12.3	4.5	5.5	0.8	2.0	2.6	11.0	1.4	7.3
GGNBP2	12.3	4.5	6.2	0.4	1.6	2.3	11.6	1.1	7.3
SIK3	13.7	4.0	5.9	1.2	2.8	3.4	7.1	1.6	7.8
FAM20B	13.4	4.2	6.0	0.9	2.1	2.6	9.7	1.3	7.2
BMS1	13.1	4.4	5.8	0.6	2.0	2.5	10.4	1.3	7.2
PRDM4	12.7	4.6	5.7	0.7	2.2	2.7	9.6	1.5	7.7
VPS53	14.3	4.1	6.0	1.0	2.2	3.1	8.7	1.4	6.6
ADPRHL2	13.2	5.2	6.0	1.1	1.9	2.8	9.6	1.1	6.4
POLR1E	10.9	3.7	4.7	0.8	2.1	2.3	12.0	2.2	8.6
PHLDB2	11.9	4.1	5.7	0.9	3.5	3.9	8.8	1.0	7.5
MOV10	13.8	5.9	8.5	1.1	2.3	2.5	6.4	1.1	5.6
STAT5B	11.6	3.9	5.7	1.1	2.3	2.8	10.7	1.6	7.6
NAGS	9.5	4.5	5.5	1.8	2.5	4.1	11.3	1.4	6.7

SLC2A3	14.3	4.9	6.2	0.3	0.9	1.3	11.2	1.0	7.2
RHBDD2	17.4	5.8	7.7	1.5	2.4	3.1	4.8	0.9	3.7
HDDC3	12.6	4.5	6.4	1.3	2.7	4.2	7.8	1.5	6.1
MFSD3	13.3	4.7	6.7	1.9	3.1	4.1	8.3	1.1	4.0
BCL7C	13.1	5.5	5.2	2.0	3.4	4.3	5.6	1.3	6.7
MTX1	12.5	5.0	6.5	1.3	2.2	3.4	8.9	1.1	6.3
IL4R	13.9	5.3	6.7	1.3	2.3	4.0	6.8	1.2	5.7
MCUR1	12.4	3.7	6.0	1.0	1.8	2.6	11.6	1.2	7.0
TMEM156	13.0	5.1	7.3	0.3	1.7	2.2	9.3	1.1	7.2
ACSL3	14.3	5.3	7.2	0.3	1.5	2.4	8.9	0.8	6.4
TUSC3	11.0	4.1	5.9	0.2	2.4	3.0	10.5	1.5	8.6
IAH1	14.1	5.5	7.5	0.5	2.4	3.4	6.7	1.1	6.0
XPO5	11.1	3.7	4.9	0.6	1.7	2.4	12.1	1.3	9.2
CCDC57	13.9	5.6	7.8	1.1	2.5	3.2	7.4	1.0	4.7
SNORD80	6.1	4.7	3.0	3.4	2.0	8.3	5.8	2.3	11.3
TMEM170A	11.8	4.2	5.8	0.7	1.6	2.1	10.8	1.8	8.3
NIPA2	13.4	4.5	6.1	0.3	2.0	2.1	10.5	1.0	7.1
ETS2	12.5	4.6	5.7	1.0	1.9	2.4	8.8	2.0	8.2
AGPS	13.0	4.8	5.6	1.0	2.0	2.8	9.7	1.2	6.9
WWC2	13.2	4.2	6.1	0.5	1.7	1.9	10.2	1.5	7.8
CHMP7	12.5	4.1	5.4	0.8	1.9	2.6	11.3	1.3	7.2
FNBP4	12.2	4.5	6.5	0.9	2.1	2.8	8.8	1.5	7.8
RRP1B	12.5	4.0	5.2	0.9	1.8	2.7	10.7	1.5	7.8
SLC25A28	12.5	5.5	7.8	1.0	2.0	3.3	7.2	1.4	6.3
MAMLD1	12.9	4.6	7.4	1.4	3.3	3.0	6.0	1.8	6.6
TRIM11	12.5	4.6	6.1	1.1	2.0	2.8	9.5	1.6	6.8
CRBN	13.1	5.8	7.1	0.7	1.6	2.8	8.1	1.4	6.4
SNORD105B	7.8	2.0	2.2	7.0	5.5	9.5	2.1	2.8	8.3
PRKCI	11.2	3.7	5.6	0.6	2.0	2.3	11.9	1.2	8.6
YTHDF3	12.0	4.5	5.9	1.0	2.4	3.2	8.5	1.1	8.3
AZI2	10.8	4.3	5.3	0.2	1.2	2.5	11.2	1.4	10.0
TMEM63B	13.8	5.8	7.1	1.4	2.1	3.8	5.9	1.3	5.6
RUNDC1	14.3	4.1	6.6	1.1	1.9	2.5	9.1	1.2	6.1
XRCC1	13.6	4.6	6.3	1.4	3.0	3.8	6.6	1.4	6.1
LOC101929264	14.3	4.3	7.2	2.0	2.2	2.5	7.7	1.8	4.8
ZNF707	11.8	4.0	5.6	1.2	2.5	3.0	9.6	2.1	7.1
PPP2R2D	12.7	4.7	6.4	1.0	2.3	2.9	9.3	1.0	6.5
SPATS2	14.2	3.4	6.4	0.8	1.7	2.0	10.9	1.1	6.3
MYPOP	12.1	4.2	5.7	1.9	3.1	3.7	8.4	1.9	5.9
GLUL	15.2	5.7	7.7	0.5	1.6	2.2	7.1	0.9	5.9
UBE3B	14.1	5.4	7.1	1.1	2.6	4.0	6.3	0.9	5.2
ZNF687	13.7	5.4	7.0	1.2	2.0	3.2	6.9	1.3	6.0
PPP1R7	13.1	4.8	7.1	1.3	3.0	3.3	7.7	1.0	5.5
ADIPOR2	12.5	4.8	5.9	0.5	1.7	2.2	10.2	1.5	7.5
LINC00114	14.8	2.8	6.5	1.0	2.1	2.1	12.0	1.1	4.4
CES2	13.7	4.5	7.1	1.4	2.5	3.5	8.1	1.0	5.0
BCL2L2	9.5	4.7	4.7	0.7	2.3	1.4	12.6	2.1	8.7
MARVELD1	10.6	5.8	5.9	3.4	4.3	6.3	4.6	1.5	4.4
LOC101929291	16.2	0.7	6.7	0.3	3.0	4.9	5.5	2.0	7.4
SUPV3L1	13.4	5.7	6.9	1.0	1.8	3.0	7.3	1.3	6.3
DUSP11	12.1	4.5	7.2	0.6	2.0	2.3	9.6	1.1	7.2
HDDC2	11.4	4.6	5.8	0.6	2.4	2.6	9.9	1.1	8.3
SGSH	15.4	6.7	8.8	1.7	2.1	2.7	4.8	0.9	3.6
GLB1	17.0	4.4	9.4	0.6	1.5	2.8	6.9	0.7	3.3
LTB4R	12.8	5.0	7.2	2.0	3.5	3.5	7.3	0.9	4.4

RALA	10.5	4.6	6.3	0.3	1.2	2.4	11.0	1.8	8.4
SNRPA1	11.6	4.8	6.6	0.6	2.2	2.7	10.2	1.1	6.9
MECP2	11.0	5.0	5.6	1.6	2.5	3.5	8.3	1.4	7.7
RRAGA	13.9	5.1	6.7	1.0	2.4	2.9	7.3	1.1	6.1
PAPD4	12.5	4.5	6.3	0.6	1.7	2.8	10.0	1.1	7.1
GBA2	13.0	5.1	8.3	0.6	1.7	2.1	8.8	1.3	5.8
SLC2A4RG	11.6	4.6	6.3	3.1	3.8	5.7	5.7	1.1	4.7
RBMS1	12.9	5.1	6.6	1.1	2.6	3.1	6.3	1.4	7.5
POLR2B	12.3	4.4	5.7	0.4	1.5	2.3	10.6	1.3	8.0
UBA3	14.5	5.2	6.8	0.6	1.6	2.2	8.4	1.1	6.1
CRTC3	13.2	4.8	6.3	0.9	1.8	3.0	7.7	1.7	7.1
DCUN1D5	10.9	4.4	5.8	0.4	1.6	2.0	11.6	1.1	8.7
PSMB10	12.1	4.4	6.5	1.9	3.2	4.7	7.2	1.3	5.0
BACE1-AS	14.3	3.3	6.4	0.8	1.7	1.9	11.9	0.8	5.3
EP400	13.0	3.5	5.8	1.8	2.9	3.5	8.1	1.4	6.5
METTL23	13.1	5.5	7.1	1.1	2.0	2.7	9.3	0.6	5.2
RHOQ	10.6	4.5	4.5	1.2	2.3	2.5	11.1	1.7	7.9
SWAP70	13.0	4.8	5.7	0.6	1.7	2.1	9.2	1.5	7.7
PRKRIR	12.3	4.5	5.8	0.4	1.8	2.1	9.9	1.3	8.3
VTA1	12.2	5.2	6.8	0.6	2.0	2.6	7.8	1.6	7.7
NDUFA8	13.5	4.0	8.2	0.4	1.7	2.7	9.4	0.9	5.6
MTFR1L	13.1	4.8	7.2	0.6	2.1	3.3	7.5	1.5	6.2
RRNAD1	14.9	5.1	7.1	0.6	1.7	2.4	8.3	1.0	5.2
SLC25A36	12.5	4.8	5.9	0.3	1.8	2.4	9.9	1.0	7.7
PTGS2	12.7	4.2	6.7	0.2	1.2	1.4	10.4	1.9	7.7
SERF2-C15ORF63	12.2	4.5	7.1	1.3	2.6	2.8	7.8	1.4	6.7
RUSC1	13.4	5.2	6.9	1.0	1.8	2.1	8.3	1.4	6.3
FLJ42842	15.5	3.1	6.7	0.8	1.7	1.9	11.6	0.9	4.3
PAGR1	10.8	3.2	5.1	1.4	2.0	3.4	11.5	1.2	7.4
ATF7IP	14.2	5.0	6.4	0.8	2.0	3.2	7.3	1.1	6.3
LOC100132077	14.3	3.4	6.6	0.9	1.7	2.4	10.9	1.0	5.2
TACO1	13.0	4.6	5.5	1.3	2.0	3.1	8.7	1.6	6.5
CPSF4	11.8	5.1	6.0	0.8	2.0	2.9	8.4	1.5	7.8
TIAF1	18.6	6.4	7.1	0.8	2.5	2.3	4.5	0.4	3.7
BCKDK	9.5	3.9	5.4	1.7	3.5	4.4	8.5	1.8	7.4
CCDC88C	12.5	4.5	5.8	1.4	1.9	2.9	8.8	1.4	7.0
CREB3	11.2	4.8	5.8	0.8	2.2	2.7	9.7	1.5	7.4
ZNF532	15.2	5.3	6.9	0.9	2.1	3.0	5.6	1.1	6.0
EVI2B	12.6	4.5	5.6	0.7	2.8	3.7	7.2	1.2	7.8
FAM174A	13.7	5.0	7.4	1.3	2.6	3.2	7.5	1.0	4.6
GPN2	12.4	4.5	5.8	1.3	2.3	3.4	8.4	1.3	6.8
CPT1A	13.0	4.2	5.7	0.8	1.6	2.1	9.7	1.5	7.7
FUCA2	12.1	4.5	5.9	1.1	1.8	3.0	9.1	1.4	7.3
SARNP	11.2	5.1	6.9	0.3	2.0	2.2	8.7	1.8	7.9
LTBP3	18.4	6.2	8.9	2.1	2.6	3.6	2.4	0.4	1.5
MRPL15	11.0	5.1	5.9	0.5	2.5	2.7	9.7	1.4	7.3
DPY30	11.9	4.9	7.2	0.1	1.5	2.0	9.3	0.8	8.4
ZBTB1	13.1	4.5	6.3	0.3	1.4	2.3	8.9	1.0	8.2
RABGGTA	10.8	4.9	6.2	2.0	3.3	4.6	7.4	1.3	5.5
OSER1-AS1	14.6	4.1	6.8	0.9	1.5	2.6	9.9	0.9	4.7
ZNF202	14.8	5.8	7.9	0.6	1.4	2.0	7.5	1.0	5.1
C12orf23	11.9	4.3	7.3	1.2	1.9	3.6	7.2	1.3	7.5
TAOK1	12.8	4.2	5.7	0.7	1.8	2.5	8.6	1.5	8.4
NUDCD2	11.9	5.7	6.9	0.7	2.0	2.9	8.5	1.2	6.3
TIPRL	12.2	5.2	6.8	0.7	2.2	2.6	7.7	1.2	7.6

ARL8A	12.3	4.6	7.9	0.7	2.4	2.8	7.3	1.4	6.6
TPR	12.2	3.8	5.1	0.5	1.8	2.7	10.3	1.3	8.3
EPN2	13.1	5.1	6.3	1.3	3.1	3.4	6.5	1.1	6.2
ARMCX1	12.7	5.9	6.7	0.8	2.6	2.9	7.7	0.9	5.8
GTF2IRD1	13.4	5.1	6.3	1.1	2.2	2.9	7.5	1.3	6.2
TAF4	8.2	6.4	6.5	3.6	2.6	2.2	7.5	2.9	6.2
MRPL32	10.3	5.4	8.7	0.8	1.7	2.8	8.2	1.3	6.9
SH3PXD2A	12.7	4.0	5.9	1.6	3.3	4.0	7.1	1.4	5.9
LINC00941	13.7	2.9	6.2	0.8	1.6	2.5	11.7	0.9	5.6
POLD1	9.8	4.1	5.5	1.8	2.9	3.8	9.1	1.5	7.3
SLC5A6	11.7	4.4	5.7	0.9	2.2	3.1	9.4	1.2	7.2
MINOS1	13.9	3.7	6.6	0.4	1.5	2.2	11.7	1.0	4.9
MAPK8IP1	13.1	5.0	6.3	1.5	2.7	3.6	6.9	1.4	5.3
PITHD1	12.7	4.5	5.3	1.2	1.6	3.3	8.3	1.3	7.6
FAM127C	13.9	6.1	5.7	1.7	2.9	3.6	5.6	0.9	5.4
PRICKLE2-AS3	14.4	3.0	6.8	0.6	1.7	2.1	11.2	0.9	5.1
PDP2	9.8	4.1	4.6	1.0	2.3	2.9	10.4	1.8	8.9
SLC17A9	9.4	4.1	5.4	1.1	2.1	2.3	11.1	1.8	8.5
TMEM167B	11.8	5.4	5.9	0.3	1.3	1.6	10.4	1.5	7.6
BAZ1B	12.0	4.1	5.2	0.7	1.9	2.6	10.1	1.4	7.7
SLAIN2	13.4	4.7	6.0	0.8	2.3	2.7	7.7	1.5	6.6
FAF1	12.0	3.9	5.4	0.6	1.9	2.5	10.4	0.9	8.2
GID4	10.8	5.3	5.7	1.6	2.5	2.5	8.0	1.8	7.6
ZNF22	10.8	4.9	6.3	0.5	2.1	2.1	8.9	1.5	8.4
TIPARP	11.6	4.4	5.6	0.8	3.2	4.3	8.0	1.0	6.8
LOC100130373	15.2	3.5	6.9	1.1	1.5	1.9	9.9	0.9	4.7
EMX2OS	15.0	3.0	6.2	0.8	1.7	2.0	11.1	0.8	5.0
RNF121	13.2	5.0	7.1	0.7	2.4	3.2	7.3	1.0	5.8
DNMT3A	13.8	4.5	7.4	1.1	2.5	2.3	7.8	1.0	5.3
LLPH	10.0	5.3	5.7	0.4	1.5	2.1	9.9	1.2	9.5
OGFOD3	10.7	3.5	5.8	1.7	3.4	4.1	8.9	1.4	6.1
JUP	19.7	7.1	8.8	1.1	2.1	2.2	2.2	0.6	1.9
LARP4	10.8	4.0	5.7	0.5	1.6	2.3	10.2	1.3	9.2
GTF2H4	11.4	4.8	6.1	0.8	1.8	2.7	8.6	1.4	7.9
FLJ41200	14.7	3.9	6.3	0.7	1.4	2.0	10.3	0.9	5.2
LOC101927084	15.1	2.7	6.4	0.9	1.8	2.0	11.2	0.8	4.6
DCAF10	13.4	4.5	6.1	1.2	2.0	2.6	8.6	1.3	5.8
RFXANK	11.8	3.5	5.4	1.4	2.6	3.4	9.2	1.1	7.0
DUXA	11.3	4.5	6.9	0.5	2.1	2.2	7.4	1.2	9.4
MRPL27	12.4	4.1	6.7	0.7	2.9	3.5	7.7	1.1	6.4
CAMK2N1	12.0	4.4	5.8	2.8	3.5	6.0	5.0	1.6	4.4
OAZ2	14.2	5.7	5.9	0.7	2.3	2.0	8.2	0.9	5.6
SPATA2	12.8	5.3	5.5	1.5	2.4	2.9	7.5	1.6	5.9
ERN1	13.5	4.6	6.4	0.9	1.5	2.6	8.4	1.4	6.0
ITGA10	18.7	5.6	9.5	0.6	1.6	2.4	3.8	0.6	2.6
LIPH	17.6	6.5	9.1	0.3	1.4	1.7	4.4	0.6	3.8
COQ4	11.9	4.4	6.2	2.3	4.2	4.7	6.7	1.2	3.9
LRRC41	12.4	5.4	6.6	1.2	2.4	3.2	7.6	1.0	5.6
MTMR14	14.1	5.0	7.0	0.9	1.8	2.5	7.3	1.1	5.6
HECA	12.5	4.1	5.5	1.2	1.7	2.7	8.9	1.6	7.2
ECE2	9.6	4.0	5.1	1.2	2.2	2.7	11.7	1.4	7.5
NDFIP2	11.5	4.5	6.7	0.7	1.6	3.1	8.1	1.3	7.7
TBL1X	12.5	4.4	5.8	1.2	2.5	2.9	7.5	1.4	7.2
NOL11	11.1	4.9	5.8	0.4	1.9	2.7	9.0	1.1	8.2
FLYWCH1	14.0	4.6	6.4	1.6	2.8	3.6	6.9	0.9	4.2

PCK2	9.8	3.7	4.8	1.5	3.9	4.7	9.5	1.0	6.5
LOC101928805	14.1	4.8	6.5	0.8	2.5	2.8	8.0	0.8	4.9
IFRD1	11.4	3.8	4.7	0.5	1.3	1.5	12.3	1.6	8.3
ZFHX3	11.5	3.7	5.2	1.8	2.3	2.6	8.8	1.8	7.6
AP1G1	12.5	5.1	5.9	0.6	1.8	2.7	7.9	1.3	7.5
MAPK7	12.4	4.8	6.1	1.3	2.0	2.9	7.5	1.8	6.5
TNFSF9	14.5	6.9	8.3	0.6	0.8	0.8	7.8	0.9	4.5
RAE1	11.6	3.9	5.7	0.5	1.4	2.2	10.2	1.1	8.7
MED30	10.1	5.3	6.5	1.2	2.1	2.7	9.4	1.7	6.0
MIB2	13.4	5.2	7.2	1.8	2.5	3.4	6.2	1.2	4.4
SCCPDH	11.4	3.9	5.2	0.7	2.5	2.5	11.2	1.1	6.6
ARMCX3	11.1	4.4	5.0	0.6	1.3	2.2	11.6	1.2	7.8
APOPT1	13.5	5.6	6.6	1.0	1.5	2.5	6.5	1.2	6.7
ZNRD1	9.1	4.0	5.6	0.5	1.9	2.3	11.9	1.4	8.4
RBM23	13.4	4.6	6.2	0.6	1.8	2.6	9.2	0.9	5.8
ZNF574	11.0	3.8	4.5	1.6	2.6	3.6	8.8	1.6	7.5
ISG20L2	10.3	3.9	5.1	0.8	2.0	2.5	10.7	1.7	8.2
FZD4	10.8	4.6	6.1	0.9	2.4	3.4	8.9	1.5	6.4
CTTNBP2NL	10.9	4.4	5.6	1.3	2.2	3.3	7.5	1.6	8.2
HIST1H2BN	10.4	4.1	5.7	2.3	3.5	4.6	6.9	1.6	6.1
SLC22A3	14.2	3.7	5.6	1.1	1.6	2.2	9.3	1.2	6.1
CACUL1	13.4	4.2	6.1	0.5	1.8	2.9	8.1	1.2	6.9
LINC00547	15.2	3.3	6.7	0.6	2.1	2.3	8.6	0.9	5.4
CDC5L	12.7	4.4	5.5	0.4	1.7	2.4	8.6	1.4	7.8
KAT7	12.8	3.7	4.5	0.5	2.0	3.4	8.6	1.3	8.4
PITRM1	12.2	4.9	6.3	1.2	2.6	3.6	7.1	1.1	5.9
PREB	8.8	4.0	5.7	1.2	2.5	3.3	11.4	1.6	6.6
ZFYVE28	11.6	3.2	6.3	1.7	3.2	3.9	8.5	1.4	5.2
B3GALNT2	11.5	3.6	5.5	0.9	1.8	2.0	11.6	1.1	7.0
POMT2	12.9	5.6	6.8	1.0	2.4	3.4	6.6	1.0	5.3
ITGA5	13.4	5.3	6.3	1.1	2.5	3.5	6.9	0.9	5.1
TRIM35	11.8	4.5	5.6	1.4	2.4	2.8	9.3	1.4	5.8
JKAMP	13.7	6.0	7.3	0.4	1.7	3.3	7.2	0.8	4.5
NCOA3	12.5	4.1	5.3	0.4	1.3	2.3	9.4	1.6	7.9
YEATS2	12.4	3.9	6.0	0.8	2.0	3.0	7.9	1.4	7.5
PDIA5	13.1	4.5	7.1	0.8	1.8	2.6	7.9	1.2	5.8
SYS1	11.7	4.5	6.8	1.2	2.8	3.4	7.0	1.6	5.9
PDK2	13.0	5.4	6.0	2.3	2.8	4.7	5.0	1.0	4.7
ARID1B	12.8	4.4	6.1	1.6	2.4	3.1	6.8	1.4	6.3
MSLN	9.2	4.2	5.5	2.4	4.5	5.6	7.3	1.3	4.9
LMF2	11.5	4.6	6.0	2.0	3.4	4.5	6.6	1.4	4.9
LOC101929524	16.1	3.0	6.6	0.9	1.7	1.8	9.7	0.9	4.1
TSG1	15.3	3.3	6.6	0.6	2.0	2.4	9.0	0.7	4.9
TGIF2-C20orf24	9.1	2.1	5.1	0.1	0.5	3.2	13.7	1.5	9.5
LOXL1	13.7	6.4	7.3	2.3	3.3	4.6	3.9	0.8	2.6
PSMB8	11.7	4.4	7.1	1.1	2.9	3.7	7.5	0.8	5.6
RAB22A	13.9	4.9	5.1	0.5	2.4	2.7	7.2	1.1	7.0
NUP153	11.2	3.9	4.9	0.4	1.9	2.3	9.5	1.6	9.0
TNRC6C-AS1	13.6	4.4	6.5	0.7	1.8	2.0	9.3	1.1	5.3
MICA	11.6	4.8	7.0	0.7	1.6	2.3	8.0	1.2	7.4
SP3	11.8	4.2	5.4	0.8	2.0	2.5	8.5	1.4	8.2
FRMD8	13.1	4.2	5.3	1.3	2.5	2.9	9.1	1.2	5.1
SUMO1	11.0	6.0	5.5	0.4	1.7	2.5	8.0	0.7	8.8
SPRYD7	12.0	3.7	6.5	0.9	2.0	2.0	10.2	1.2	6.3
LOC101929130	13.4	2.8	6.4	0.7	1.9	2.1	11.6	0.9	4.8

PRPSAP2		10.3	3.9	5.5	0.7	2.0	2.5	10.8	1.3	7.9
LOC100128288		14.7	3.2	6.1	0.9	1.9	1.9	10.8	0.9	4.3
SRPX		13.1	5.3	6.3	1.2	2.9	4.3	6.0	0.8	4.8
SEC24D		11.0	4.3	5.2	0.7	1.7	2.3	8.8	1.8	8.8
OPA3		11.7	4.4	5.6	1.8	2.0	2.5	9.2	1.5	5.9
TMEM208		11.0	3.7	6.2	0.9	1.9	2.9	10.8	1.3	5.9
SPICE1		13.8	3.5	7.3	0.9	1.9	2.7	7.7	1.0	5.6
RNF34		10.2	3.6	5.1	0.9	1.7	3.0	11.2	1.5	7.4
LOC101927061		12.3	6.2	8.7	0.3	2.2	2.4	4.6	1.1	6.8
TBL2		9.6	3.5	4.9	1.4	2.0	3.1	10.4	1.6	8.1
TPT1-AS1		12.9	4.2	6.1	1.2	2.2	2.7	9.7	0.9	4.7
LOC100129034		14.5	5.4	6.7	1.0	2.2	2.8	5.9	0.9	5.3
	44812.0	12.1	5.1	5.9	1.0	2.9	3.9	7.0	1.1	5.5
HMGN3		11.6	5.1	5.8	0.5	0.9	2.5	9.0	1.4	7.7
RFX1		13.6	4.1	5.8	1.9	2.5	3.1	6.8	1.2	5.4
DSCR3		11.1	4.3	6.1	0.9	2.4	2.8	7.5	1.6	7.8
GTPBP1		12.9	4.7	5.9	1.3	2.4	3.0	6.7	1.2	6.3
SCAF4		12.7	4.1	5.3	1.0	1.9	2.6	7.6	1.4	7.8
STK38		12.7	5.0	6.2	0.5	1.5	2.4	8.2	1.1	6.9
TTC3		14.5	3.9	5.1	0.4	1.9	2.5	8.4	1.0	6.7
MED9		11.3	5.7	6.2	2.3	2.5	3.7	4.9	2.0	5.8
LINC01012		14.9	2.8	6.0	0.7	1.7	2.3	10.6	1.1	4.4
INO80C		13.0	4.4	7.3	0.8	1.8	2.9	7.2	1.2	5.8
SP2		11.5	4.7	5.6	1.5	2.7	3.4	6.8	1.8	6.4
AKT3		11.2	4.2	5.9	0.3	2.0	2.4	9.8	1.0	7.6
SHANK2		9.0	3.2	4.0	1.4	2.6	3.3	10.6	1.7	8.6
R3HDM2		13.7	5.2	6.6	1.4	3.3	4.1	4.4	0.9	4.7
EP300		11.8	3.8	5.2	1.0	2.1	2.6	7.7	1.8	8.4
ALAS1		11.9	4.6	5.5	0.6	1.4	2.2	9.4	1.5	7.3
C21orf59		11.8	4.6	5.9	0.6	1.9	2.3	8.6	1.3	7.3
ETV1		15.1	5.5	7.3	1.4	2.6	4.3	3.2	1.0	3.9
ZFP91-CNTF		11.0	4.0	6.1	1.0	2.2	3.1	8.0	1.7	7.2
SH3RF1		9.9	3.0	4.4	1.3	2.6	3.6	8.7	2.0	8.7
MTMR4		11.5	4.3	5.6	0.9	2.4	2.8	8.6	1.4	6.7
FTO		13.3	4.2	5.6	0.8	2.7	3.1	8.7	0.9	5.1
ZBED6		12.2	4.0	5.4	0.3	1.5	2.1	9.1	1.3	8.2
FBXO46		10.8	3.9	5.7	2.0	2.9	3.6	7.8	1.8	5.8
CDKN2D		12.9	4.6	6.4	1.7	2.7	3.2	7.2	1.2	4.3
STC1		12.9	5.2	6.6	1.1	2.1	3.3	6.2	1.4	5.6
MCCC2		13.5	4.2	6.1	0.7	2.2	2.8	8.4	0.9	5.4
RREB1		13.0	4.0	5.7	1.4	2.1	2.7	7.2	1.5	6.6
TRAK1		11.2	5.0	5.6	1.3	2.1	3.1	8.2	1.3	6.4
ZXDC		12.0	4.3	5.3	1.5	2.4	3.5	8.3	1.4	5.5
SH3KBP1		12.0	4.8	5.0	0.8	1.8	3.0	6.9	1.2	8.7
TFIP11		12.2	4.7	5.6	0.8	2.0	2.3	8.4	1.4	6.7
CAMK2G		12.4	5.1	7.0	1.1	2.2	3.1	6.1	1.3	5.9
COPS7B		9.9	4.7	6.1	0.7	1.2	2.1	8.3	1.6	9.4
LAS1L		10.3	3.7	4.7	1.0	2.7	3.5	10.3	1.1	6.8
PTDSS2		9.7	4.1	5.5	1.8	3.4	4.4	7.8	1.3	5.9
PDE4C		14.1	3.0	6.4	0.7	1.6	1.8	10.9	0.9	4.6
AARSD1		11.1	4.5	5.1	1.0	2.4	3.6	9.0	1.0	6.5
MOB2		12.0	5.0	7.0	1.0	2.4	2.7	5.9	1.8	6.3
RPA2		10.6	4.9	5.7	0.7	2.6	2.5	7.7	1.2	8.2
NUDT19		12.1	4.0	4.7	1.8	2.3	3.0	8.0	1.9	6.3
CCDC58		9.8	4.5	7.0	0.4	1.9	3.4	8.0	1.5	7.4

SLC12A4	13.2	5.0	7.1	1.2	2.4	3.1	6.4	1.1	4.3
LOC100507303	12.5	5.2	8.2	0.5	1.1	2.6	7.8	0.8	5.3
SENP2	13.0	5.0	5.6	0.5	1.8	2.7	7.6	1.2	6.6
FGFR1OP2	12.7	3.9	7.1	0.5	0.9	1.6	8.7	1.0	7.4
HS3ST1	11.2	4.5	5.3	0.8	1.7	2.5	9.0	1.8	7.0
PHRF1	11.4	4.2	5.6	1.4	2.2	2.8	8.6	1.5	6.2
SIRT7	13.7	4.9	7.1	1.0	1.2	1.6	8.1	1.2	5.1
LOC101928061	14.9	3.2	6.8	0.7	1.6	1.8	10.3	0.8	3.7
SMEK2	12.1	3.6	5.7	0.4	1.4	1.6	10.0	1.3	7.8
DGCR6L	12.0	5.1	6.3	2.3	3.3	4.4	6.1	0.9	3.4
PARD3	11.8	3.8	4.7	0.6	1.9	2.2	10.8	1.1	6.9
LAP3	10.6	3.9	5.2	0.6	2.2	2.6	9.4	1.5	7.6
OGFOD2	13.6	5.3	6.5	1.2	2.3	3.2	6.1	1.2	4.3
AGPAT3	14.2	4.8	6.1	1.2	1.9	2.8	6.9	0.7	5.1
LINC00665	14.3	3.0	6.6	0.8	1.9	2.0	9.6	0.9	4.7
KLF10	17.2	7.1	8.5	0.6	1.5	2.1	3.1	0.4	3.2
SPNS2	12.5	5.6	6.0	1.0	1.7	2.7	7.1	1.2	5.7
IPO9	10.2	3.9	4.7	0.9	2.6	3.0	9.3	1.4	7.6
JOSD1	11.7	5.0	5.5	0.7	1.7	2.5	7.4	1.2	7.9
USP34	12.1	3.7	5.2	0.3	1.5	1.9	9.5	1.2	8.1
PHAX	12.3	4.8	5.9	0.8	1.9	2.5	7.6	1.2	6.7
CDCA4	9.6	4.0	5.2	1.2	2.3	3.0	10.8	1.3	6.1
NCAPD3	10.1	3.8	5.2	0.7	1.9	3.0	9.6	1.5	7.8
ABRACL	13.7	3.9	6.5	0.2	1.1	1.5	9.3	1.4	6.0
COMMD2	12.0	4.7	6.9	0.3	1.6	2.2	8.1	0.9	6.8
C5orf45	13.1	5.9	7.1	0.9	2.6	2.3	6.2	1.0	4.5
AKR1A1	11.5	4.1	6.8	1.2	3.0	3.6	7.6	1.0	4.9
POLE3	11.2	3.9	5.3	0.9	1.9	3.1	9.2	1.5	6.6
CDS2	11.4	4.6	5.9	0.6	1.5	2.3	9.4	1.2	6.7
GPR135	14.4	2.7	6.7	2.1	3.2	4.0	6.2	0.7	3.4
MRPL16	11.8	4.7	6.1	0.8	2.4	3.1	8.5	0.8	5.3
WDR11-AS1	14.0	3.1	6.2	0.6	1.8	1.9	10.0	0.9	4.9
PANX1	12.1	5.1	5.7	0.5	1.6	2.0	7.2	1.2	8.2
MRPL19	10.0	3.8	5.8	0.7	2.1	2.1	11.2	0.8	6.8
FAM120AOS	10.7	3.2	5.2	0.3	2.6	3.9	10.1	2.0	5.4
HPS3	14.4	5.4	7.0	0.6	1.8	2.6	6.4	0.9	4.4
DAGLA	13.6	5.0	6.2	1.4	2.2	3.0	6.0	1.1	4.9
UBE2G1	11.8	4.2	4.5	0.4	2.0	2.4	10.8	1.0	6.4
ENDOG	10.9	4.6	5.0	1.7	2.7	3.5	8.1	1.1	5.9
HSF4	11.8	4.9	7.7	1.2	3.1	3.7	5.8	1.1	4.2
TYSND1	13.3	4.9	7.0	1.4	2.4	3.3	6.5	0.6	3.9
ST3GAL2	11.0	4.0	5.4	1.5	2.5	3.1	8.0	1.3	6.5
FBXO18	11.3	4.1	6.0	0.8	1.7	2.7	9.3	1.1	6.3
IER5L	15.5	5.8	8.2	1.7	2.3	2.9	3.0	0.9	3.2
PSMD10	13.1	4.7	6.2	0.6	1.9	2.0	8.6	1.1	5.1
CEP57	11.7	4.6	6.7	0.4	1.4	2.4	8.2	1.3	6.7
WRAP73	11.4	4.6	5.4	1.0	2.1	2.4	8.9	1.4	6.3
DND1	13.7	4.8	6.9	1.4	2.6	2.6	6.2	1.0	4.1
UHRF1	6.7	2.8	3.2	1.6	2.7	3.2	11.2	2.3	9.7
ALDH16A1	9.6	3.8	5.4	1.9	3.3	4.4	9.0	1.3	4.6
AGTRAP	11.4	4.9	6.1	1.8	4.0	4.2	4.1	1.2	5.5
SNORA40	12.5	5.1	5.2	0.2	1.1	2.4	8.6	0.4	7.7
SNORD60	7.6	4.2	3.7	2.4	5.2	3.4	6.4	2.1	8.2
LOC100506548	12.0	3.5	5.7	0.7	2.1	2.5	9.9	0.9	6.0
FCGRT	17.0	5.7	7.8	1.2	1.9	2.7	3.9	0.5	2.6

RAB14	12.1	4.2	5.6	0.5	1.2	1.9	8.6	1.3	7.8
RECQL4	9.9	4.4	5.9	1.6	2.7	3.6	7.5	1.5	6.2
SMC1A	11.9	4.3	5.1	0.9	1.9	2.3	8.5	1.1	7.2
ARFRP1	11.6	4.6	5.7	1.7	2.9	3.6	7.3	1.0	4.7
PDHA1	10.8	4.2	6.0	0.9	2.4	2.9	8.8	1.1	6.0
PSMD14	11.0	4.0	5.4	0.3	1.4	2.1	9.7	1.1	8.3
AGFG1	10.2	3.8	5.4	0.6	1.5	2.1	9.7	1.4	8.4
TMEM39B	10.6	4.2	5.6	0.9	2.4	3.2	7.9	1.5	6.9
TXNDC15	11.2	4.5	6.1	0.7	1.4	2.0	9.0	1.3	6.8
TRIM4	11.5	5.3	4.8	1.0	1.6	2.9	8.3	1.2	6.6
NDUFS1	12.0	4.1	5.6	0.4	1.5	2.3	9.3	0.9	7.1
AFG3L2	12.0	4.1	5.3	0.6	2.1	2.6	9.2	1.2	6.0
NDE1	12.1	3.6	5.5	1.0	1.8	2.5	9.4	1.1	6.1
FBXO21	13.9	4.5	5.9	0.9	2.1	2.6	6.5	1.2	5.4
SLC1A3	10.2	4.5	4.7	0.4	1.6	2.3	10.0	1.1	8.2
SNRNP35	10.5	4.0	5.7	0.7	2.4	2.3	7.4	1.7	8.3
ADCK3	11.6	4.7	6.3	1.2	2.6	3.8	6.7	1.1	5.1
MTX2	12.6	3.9	6.5	0.8	2.2	2.7	7.7	1.0	5.6
LINC00338	12.8	2.9	6.0	0.9	2.0	2.6	9.9	1.1	5.0
GOLPH3L	10.1	4.9	6.1	0.6	1.3	2.3	8.4	1.3	8.1
BSDC1	11.8	5.1	6.2	0.9	2.3	2.7	7.0	1.1	5.8
TRAF2	11.2	4.2	5.5	1.1	2.3	2.8	9.3	1.4	5.2
RNF115	11.7	3.8	5.9	0.7	2.2	3.1	7.7	1.2	6.6
SC5D	13.4	5.6	7.6	0.1	0.9	1.4	5.7	0.9	7.3
MSRB3	9.3	3.5	4.3	0.6	1.8	2.3	12.1	0.8	8.2
LRCH3	13.2	4.0	5.4	0.8	1.6	2.5	8.3	1.2	6.1
LOC100128398	14.4	3.2	5.9	1.0	2.0	2.2	9.2	0.8	4.2
RCAN1	14.5	4.9	7.1	0.7	1.8	2.6	4.9	0.8	5.6
PTPN21	11.6	3.8	4.7	1.5	2.3	2.9	7.7	1.7	6.7
ZNF205-AS1	13.7	3.2	5.6	0.7	1.3	1.9	10.7	0.9	4.9
LARS2-AS1	12.9	3.2	6.0	0.6	1.4	1.9	10.8	0.9	5.3
KAT6A	11.0	3.9	5.0	1.1	2.1	2.9	7.5	1.7	7.6
HMGCR	13.8	5.1	7.0	0.2	1.0	1.3	7.5	0.8	6.1
ZNF324	10.4	4.0	5.4	1.7	2.3	3.2	8.1	1.6	6.2
PRPSAP1	11.4	4.2	5.4	1.0	2.2	2.5	9.0	1.2	6.0
TMEM106C	11.3	4.8	7.5	0.9	2.4	3.0	7.0	0.9	4.9
MAN1A2	11.8	4.3	6.4	0.3	1.8	2.3	7.6	1.5	6.7
ANKRD27	11.3	4.4	5.5	0.7	2.1	2.7	8.6	1.3	6.2
OSGEPL1-AS1	14.3	2.6	5.8	0.8	1.3	1.6	11.0	0.9	4.6
FTSJ2	11.1	4.1	5.8	1.0	1.8	2.5	9.8	0.9	5.7
E2F6	9.6	3.6	4.9	0.5	2.0	1.9	9.9	1.1	9.3
NOP58	8.5	2.9	3.9	0.1	1.6	1.9	12.5	0.9	10.4
NUDT16L1	10.5	4.2	6.3	1.7	2.8	3.4	8.1	1.3	4.4
LOC100287792	14.2	2.8	6.3	0.8	1.5	1.9	10.2	0.6	4.6
SMIM13	10.9	1.8	4.6	1.0	1.3	2.3	11.7	1.1	7.9
LOC101928738	13.8	2.8	6.3	0.6	1.4	1.6	11.0	0.8	4.5
ASS1	12.6	4.4	6.6	0.6	1.7	2.2	7.2	1.3	6.1
NEU1	10.7	4.6	6.7	0.8	2.3	2.6	8.3	1.2	5.4
USP14	11.5	4.1	5.2	0.4	1.4	2.0	9.6	1.0	7.4
ISY1	9.1	6.6	7.6	1.0	3.0	2.1	6.9	1.5	4.9
NABP1	11.4	3.3	6.1	0.9	2.7	3.4	7.6	1.0	6.3
DNAJC15	11.9	4.9	6.5	0.9	1.6	2.5	7.4	1.4	5.7
CUL4B	11.2	4.0	5.0	0.3	2.0	2.3	8.7	0.9	8.1
CMC2	11.0	3.3	6.9	0.7	1.6	2.6	9.7	1.2	5.7
GGA3	12.3	4.7	6.2	1.0	2.2	2.9	6.5	1.2	5.6

RCOR3	10.6	4.1	5.7	1.1	2.2	2.9	7.1	1.5	7.4
LOC101928493	13.1	3.9	6.6	0.7	2.1	1.8	7.8	1.3	5.3
PPME1	11.3	4.4	4.8	0.4	1.3	1.3	11.5	1.2	6.6
PSIP1	10.6	4.3	4.9	0.7	2.1	3.3	6.9	1.7	8.2
IL6	18.1	7.5	11.4	0.4	0.9	1.2	1.0	0.3	1.8
LOC151475	14.3	2.8	6.1	0.8	1.4	2.0	10.3	0.7	4.2
SLC35C1	12.2	5.5	6.7	1.4	2.0	2.5	6.1	0.9	5.4
ZFAND1	11.3	2.9	5.4	0.3	1.3	2.7	10.5	0.9	7.3
RBM15	11.1	3.5	4.9	1.0	2.0	2.1	9.4	1.4	7.1
PHF20L1	12.3	3.4	5.8	0.4	2.8	2.5	8.5	0.9	6.0
CCDC53	12.1	5.8	6.9	0.6	2.9	2.7	5.3	0.8	5.4
SOCS7	12.6	4.1	6.0	1.0	1.9	2.1	7.8	1.4	5.6
MAP3K7	12.3	3.4	5.4	0.6	1.7	2.0	8.5	0.9	7.6
RFTN1	10.9	4.7	5.1	0.7	1.5	2.3	8.6	1.5	7.3
LOC101928684	14.3	2.6	5.7	0.9	1.6	2.1	9.6	1.0	4.6
CHST12	11.0	3.9	5.5	1.7	2.4	3.5	7.7	1.7	5.0
TMEM136	9.4	4.2	4.5	0.9	1.4	3.1	10.9	1.7	6.2
NOP9	10.8	4.0	5.9	1.0	2.5	3.1	8.6	1.1	5.5
PRKAG2	10.0	3.8	4.0	0.6	1.9	2.6	9.3	1.2	9.0
ZMYND8	11.2	4.3	4.9	1.2	2.1	2.7	7.6	1.7	6.8
SCARB2	12.5	4.9	5.5	0.2	1.4	1.9	8.2	1.0	6.8
MGEA5	15.5	4.3	7.4	0.7	1.7	2.5	5.4	0.7	4.3
MARK3	10.3	4.9	5.6	0.6	1.8	2.3	8.2	1.4	7.2
CUL3	10.6	4.0	5.0	0.5	1.7	2.4	8.9	1.4	7.9
C12orf43	12.7	4.3	5.7	0.9	2.8	2.5	7.1	1.3	5.1
ANP32E	8.5	3.0	5.1	0.5	2.0	2.1	12.2	1.4	7.5
SNX27	12.9	5.0	6.0	0.8	1.7	3.1	5.6	1.1	6.1
NSMAF	12.0	4.4	5.8	0.4	1.9	2.3	7.7	1.2	6.5
ANKHD1-EIF4EBP3	12.6	4.6	5.6	0.6	1.6	2.3	7.7	0.9	6.4
SMC6	12.6	4.6	6.3	0.3	1.6	1.9	7.9	1.0	6.1
C15orf52	8.8	3.5	4.5	2.2	4.5	5.6	6.8	1.1	5.3
PIAS4	11.6	3.7	5.4	1.2	2.0	2.7	9.0	1.3	5.3
CHD8	12.4	3.9	5.5	0.6	1.8	2.4	8.3	1.2	6.2
MAN2B2	11.7	4.9	6.0	2.0	2.9	4.4	5.0	1.0	4.3
C17orf96	8.3	3.5	4.0	1.6	2.3	3.3	10.3	2.1	6.6
PCSK7	12.1	4.0	5.7	1.1	1.9	2.6	8.2	1.0	5.5
USP19	12.3	4.5	5.8	0.9	2.0	2.5	7.5	1.0	5.5
LOC728755	14.3	2.6	6.1	1.2	2.3	2.7	8.1	0.7	4.1
CEPT1	12.9	4.4	5.0	0.3	1.3	1.9	7.8	0.9	7.6
TRRAP	12.0	3.3	5.1	0.9	1.8	2.3	9.1	1.3	6.3
PRPF39	13.0	4.9	7.0	0.3	1.0	1.5	7.1	1.1	6.1
CALCOCO1	13.0	5.5	7.1	1.0	1.8	3.0	5.5	1.0	4.2
CHMP4A	9.7	4.1	4.7	0.7	1.6	2.4	9.7	1.2	7.9
TMEM192	12.9	4.3	5.8	0.6	1.5	2.2	6.8	1.2	6.7
MAP7D3	9.9	3.7	4.3	0.8	2.5	3.3	8.2	1.5	7.7
ALDH1B1	10.8	4.0	5.5	0.9	2.4	2.4	8.8	1.4	5.9
ZNF697	10.2	3.2	4.6	1.4	2.5	3.2	9.0	1.7	6.1
CMTR1	14.4	5.2	6.8	0.7	2.0	2.7	5.6	0.6	4.0
TUG1	11.6	4.2	5.5	0.5	2.0	2.8	7.7	1.2	6.6
TGFBR3L	9.6	4.3	4.8	2.6	2.9	4.1	7.7	1.7	4.2
MAST3	13.8	4.6	5.9	1.6	2.0	3.3	5.4	1.0	4.4
ST6GAL1	10.5	4.6	4.9	0.8	2.6	2.9	7.9	1.2	6.6
SENP5	12.1	4.1	5.0	0.3	1.5	1.5	9.6	0.8	7.0
C16orf58	10.1	4.8	5.2	1.6	3.4	4.4	6.5	1.2	4.7
NR1D2	8.5	3.2	5.1	0.3	1.1	2.1	10.7	1.4	9.4

SLC35D2	14.2	3.1	6.5	0.8	2.2	2.6	7.0	0.6	4.9
B4GALT7	10.5	4.5	5.2	1.4	3.0	3.3	7.5	1.1	5.3
CTSF	14.0	5.1	6.8	1.5	3.3	4.1	3.8	0.3	2.7
UBLCP1	11.7	4.5	6.7	0.3	1.7	2.2	7.6	1.1	5.9
C9orf123	10.8	3.5	5.4	1.4	2.3	4.1	8.8	1.1	4.4
DNAJB12	12.2	4.7	5.7	1.2	2.8	3.6	5.2	1.0	5.4
LRTOMT	13.7	3.0	5.9	0.7	1.7	2.0	9.5	0.8	4.4
GTF3C4	10.5	3.9	4.9	0.8	1.9	1.8	8.9	1.4	7.7
LOC90784	12.3	3.8	5.0	0.7	1.7	2.4	8.3	1.1	6.4
UIMC1	11.8	3.4	5.8	0.4	1.2	1.9	10.3	1.0	5.8
ANKH	10.9	3.6	5.1	1.0	2.4	3.3	7.9	1.1	6.5
CSTF3-AS1	10.8	4.2	5.8	1.4	2.3	3.6	5.8	1.5	6.3
CACNG7	13.8	5.3	7.4	2.3	3.1	4.4	3.1	0.6	1.7
TUBA4A	13.0	4.8	6.3	1.4	2.3	3.5	5.9	0.8	3.6
HIST1H2AB	8.3	4.1	5.4	1.5	2.7	4.8	8.6	1.1	5.0
RABGAP1	14.1	3.8	5.2	0.5	1.6	2.1	7.4	1.0	6.1
ABCA3	12.4	4.8	6.2	1.5	2.7	3.8	5.3	0.8	4.3
VPS4B	11.0	3.7	6.4	0.3	1.6	1.8	8.9	1.0	6.9
SNORD118	9.3	6.7	8.8	0.4	2.2	0.0	7.8	1.0	5.3
RNMTL1	10.8	3.8	5.4	0.8	1.4	2.1	9.5	1.1	6.7
TBXA2R	14.0	2.3	5.9	0.9	1.4	1.8	10.8	0.8	3.8
AEBP2	9.5	4.6	5.9	1.5	1.8	2.8	7.4	1.5	6.7
CCDC93	11.1	3.7	5.3	0.6	1.5	2.0	9.2	1.5	6.7
DUS3L	9.8	3.7	4.9	1.3	2.4	3.1	9.1	1.5	5.8
RQCD1	10.5	3.5	5.6	0.6	1.7	2.7	8.5	1.0	7.3
BRD7	10.7	4.1	5.2	0.6	2.0	2.9	7.7	1.3	6.9
ZFYVE27	11.5	4.4	6.9	1.0	1.5	2.2	8.3	1.1	4.8
PPP2R5E	11.4	4.5	5.2	0.4	0.9	2.0	7.7	1.5	7.9
GOSR1	11.5	4.6	5.4	0.4	1.7	2.3	6.9	1.1	7.7
KIF3C	13.1	5.3	6.5	1.1	2.4	3.0	4.7	1.1	4.3
TSR2	12.0	4.2	5.9	0.5	1.9	2.6	7.3	1.2	5.7
CRLF3	11.4	3.3	5.1	0.5	1.5	2.0	10.4	1.0	6.1
BPNT1	10.4	3.3	5.6	0.8	1.5	2.1	9.5	0.9	7.2
FNTB	11.7	4.7	6.4	0.9	2.0	2.6	7.1	1.1	5.1
FAM210B	11.1	4.5	5.3	2.3	3.4	3.8	5.8	0.8	4.5
IRF2	11.4	5.0	4.8	0.8	2.0	2.7	8.0	1.3	5.4
BAHCC1	12.4	5.0	6.6	1.3	1.8	2.5	6.0	1.3	4.5
KCTD13	11.6	4.0	5.7	1.2	2.9	3.2	7.1	1.3	4.4
ITGAV	14.0	4.9	6.9	0.4	1.7	2.1	6.5	0.8	4.1
MYRF	11.8	4.6	6.1	1.9	2.9	4.0	4.6	1.0	4.5
KLHL36	13.3	4.5	6.1	0.9	1.6	2.3	7.0	0.9	4.8
ALG2	10.3	3.7	4.8	0.5	1.7	2.3	10.7	1.3	6.0
ALKBH6	11.5	5.6	5.4	1.6	2.6	4.0	5.3	0.9	4.4
ZEB1	10.2	3.6	5.1	0.7	2.2	3.3	7.4	1.4	7.6
TGIF1	11.6	4.5	6.8	0.6	1.7	2.9	5.7	1.3	6.2
LINC00672	13.8	2.6	5.9	0.7	1.4	1.7	10.3	0.8	4.1
SNORA18	8.9	4.9	5.1	0.8	1.4	2.2	12.0	1.6	4.4
OCIAD2	14.4	4.4	6.5	0.3	1.7	2.4	7.0	0.7	4.0
GOT1	10.5	4.2	4.9	0.7	2.2	2.8	8.3	1.1	6.6
POLR3E	9.8	3.6	4.7	0.9	1.9	2.7	8.9	1.3	7.5
NAV2	14.2	4.2	6.5	1.5	3.5	3.7	4.1	0.7	3.0
LOC100996702	12.6	3.8	6.0	0.9	1.4	1.7	7.6	0.9	6.3
DAGLB	9.2	3.3	4.8	1.2	2.4	2.9	9.4	1.3	6.7
PNKP	8.8	3.6	5.1	1.5	2.6	3.9	8.1	1.5	6.3
SLC7A11	10.5	3.0	4.9	0.4	2.3	2.5	9.2	1.2	7.1

NDUFA7	11.4	5.0	6.5	2.4	3.1	5.4	4.0	1.0	2.3
JMY	11.3	3.3	4.8	0.8	1.8	2.1	8.7	1.5	6.9
TCFL5	10.6	4.2	4.9	1.6	2.9	2.9	7.5	1.2	5.5
ST3GAL1	13.1	4.0	5.7	1.0	1.5	2.6	7.0	1.0	5.3
C19orf68	10.1	4.1	5.2	1.7	2.1	2.6	9.0	1.4	5.1
LOC101928050	13.2	3.3	5.6	0.7	1.6	2.1	8.7	0.9	5.0
ABHD10	10.2	3.2	6.2	0.7	2.1	2.4	8.7	1.4	6.2
MNF1	10.0	4.5	6.7	0.7	2.3	3.6	6.5	1.3	5.5
ESYT1	10.7	3.9	4.8	0.8	2.4	2.9	8.9	1.1	5.7
ASCC2	10.6	4.7	5.6	1.2	2.3	3.0	7.0	0.9	5.9
MICU1	12.3	4.9	5.7	0.7	2.0	3.2	6.6	0.8	4.9
MAP3K3	11.1	4.2	6.0	0.9	2.2	2.7	6.9	1.1	6.0
HECTD3	12.8	4.4	6.5	1.0	2.0	3.2	6.1	0.8	4.3
MIER2	10.3	3.6	5.8	1.0	1.9	2.3	8.3	1.3	6.6
POLR3C	10.3	4.3	5.2	0.7	2.1	2.9	8.1	1.1	6.5
TCEB3	10.6	3.2	4.7	0.8	1.9	2.2	8.5	1.3	7.9
RAB24	12.0	3.5	5.6	1.3	2.1	2.6	7.9	0.9	5.1
GNL3L	10.2	3.1	4.6	0.6	2.2	2.0	9.9	1.4	7.0
LMAN2L	12.2	5.1	6.5	0.7	1.8	2.8	6.0	0.8	5.0
SIX4	9.7	3.4	5.0	1.3	2.2	2.9	8.3	1.6	6.5
BCKDHA	10.8	3.8	6.3	2.3	3.7	5.6	4.2	0.9	3.4
ZNF584	8.6	3.4	4.4	0.5	2.3	3.0	9.7	1.4	7.8
COPS2	10.4	3.7	5.0	0.2	1.8	1.9	8.2	0.9	8.8
KLF3	13.2	4.4	5.5	0.7	2.0	2.4	6.2	0.9	5.6
HNRNPDL	10.5	4.2	5.8	0.9	1.9	2.6	6.7	1.3	6.9
TPX2	10.8	4.2	4.5	0.6	2.4	2.9	8.3	1.0	6.1
DIS3	11.5	3.7	4.8	0.3	1.2	1.8	9.4	1.0	7.1
POP4	11.0	4.4	6.3	0.9	2.5	2.9	6.3	1.0	5.7
NDUFB3	13.1	3.4	7.8	0.4	2.3	2.2	5.4	1.0	5.2
STAT5A	11.5	4.3	5.6	1.1	1.9	2.9	7.2	1.1	5.3
DNAJC1	9.8	4.0	5.4	0.6	2.0	2.6	8.1	1.6	6.7
KDM6B	12.2	4.2	5.9	1.3	2.0	2.6	5.9	1.4	5.4
PRPF4B	10.6	4.1	5.0	0.4	1.9	2.2	8.2	1.2	7.2
TRMT10B	13.4	2.8	5.8	0.7	1.5	1.8	9.3	0.8	4.9
PDGFA	10.9	2.8	5.2	1.5	2.6	3.4	8.0	1.2	5.4
HSD17B4	10.6	4.2	5.4	0.4	2.1	2.8	8.5	1.0	5.7
TMEM167A	9.2	4.3	6.5	0.2	0.5	1.8	9.8	0.9	7.7
SRRM2-AS1	13.3	2.6	5.3	0.7	1.6	1.6	10.8	0.6	4.2
PTCSC3	14.3	3.3	6.1	0.7	2.1	2.2	7.1	0.8	4.3
THAP4	9.8	4.0	5.1	1.3	2.5	3.0	7.2	1.5	6.4
CCDC84	11.3	3.9	6.5	1.0	2.2	2.9	6.5	1.2	5.4
UTP3	10.3	3.2	5.0	0.9	1.9	2.7	8.3	1.6	6.8
SPATC1L	10.1	3.6	4.6	1.8	2.9	3.1	8.0	1.2	5.5
ZDHHC16	10.4	4.2	4.5	0.8	3.2	3.9	7.0	1.1	5.5
ARHGAP31	10.4	4.3	5.0	0.7	1.5	2.0	8.1	1.7	7.0
FRMD6-AS1	13.0	2.5	6.0	1.2	2.1	1.9	9.3	0.9	3.8
PHLPP1	10.1	3.5	4.6	1.2	2.2	2.3	9.2	1.4	6.3
MAP3K10	11.5	4.6	6.1	1.7	2.5	3.3	5.4	1.0	4.6
NMD3	8.5	3.7	4.6	0.3	1.6	2.3	11.0	0.9	7.7
ST3GAL4	12.7	3.4	6.1	1.0	2.3	3.2	6.3	1.3	4.6
LOC101927723	12.8	2.4	5.7	0.8	1.6	1.6	10.5	0.6	4.7
TLK2	10.6	4.0	5.1	0.4	1.3	1.9	8.6	1.4	7.3
HS3ST3B1	11.4	4.0	5.2	1.4	2.6	3.3	6.6	1.5	4.8
GMPPA	9.9	3.8	5.3	1.2	1.9	3.4	7.8	1.6	5.8
STRIP1	10.7	4.1	5.4	1.2	2.0	3.2	6.7	1.3	6.0

SNX8	10.4	4.3	6.0	1.7	2.8	3.4	6.4	1.1	4.5
FBXO17	11.2	4.5	5.7	0.8	1.9	1.9	8.0	1.1	5.4
MCM4	7.5	2.4	3.0	1.1	2.9	3.5	9.5	1.7	8.9
CHML	7.8	2.7	3.9	0.4	1.9	2.9	10.3	1.3	9.5
DUSP10	9.0	3.8	5.3	1.5	2.8	3.6	6.2	1.4	7.0
PTPRK	12.6	4.8	5.8	0.4	1.4	2.0	7.0	1.1	5.3
CPNE2	10.1	4.3	5.7	1.2	2.4	3.8	6.1	1.4	5.6
LINC00663	12.7	3.1	5.6	0.8	1.8	1.9	9.4	0.8	4.4
TADA2B	10.0	3.4	4.9	1.2	1.9	2.0	9.3	1.6	6.2
DLEU2	13.6	2.9	6.2	0.7	1.5	1.9	8.7	0.8	4.3
ATG14	11.2	4.1	5.2	0.7	2.3	2.5	7.2	1.1	6.3
SLC33A1	8.8	2.6	4.3	0.4	1.5	2.3	11.0	1.5	8.1
PANK2	10.0	3.5	5.3	0.9	1.5	2.3	10.4	1.2	5.4
MAP3K13	12.7	3.5	6.0	0.6	1.8	1.9	8.4	0.8	4.9
THSD4	10.7	3.8	4.7	1.7	3.0	4.4	6.3	1.0	4.9
UTP11L	11.4	4.7	5.9	0.2	1.3	2.5	6.7	1.1	6.6
C4orf26	13.3	2.7	5.8	0.7	1.7	2.0	9.7	0.7	3.9
CCDC97	10.6	3.9	4.6	1.4	2.2	3.4	7.5	1.6	5.3
NUP43	10.3	3.8	4.7	0.3	1.9	1.8	9.4	1.2	7.1
MED22	9.8	4.2	4.8	1.5	2.8	3.6	7.5	1.1	5.1
METTL10	12.4	3.0	5.4	1.0	1.6	1.7	9.7	1.1	4.6
LOC100188947	12.7	3.4	6.7	0.6	2.3	2.4	5.8	0.9	5.7
VARS2	11.1	4.5	6.5	0.8	1.7	2.5	7.2	1.0	5.0
COPS8	11.7	5.0	5.4	1.0	2.2	2.4	6.4	1.0	5.4
UMPS	9.8	3.5	5.5	0.5	1.3	2.1	9.5	1.2	6.9
HCN2	13.4	5.3	6.4	1.8	2.8	3.5	3.4	0.8	2.9
UBAP1	10.3	4.0	5.3	0.6	1.8	2.2	7.3	1.7	7.2
ZNF512B	9.2	3.8	5.0	1.8	2.7	3.7	7.7	1.3	5.2
STX6	10.7	3.4	4.5	0.7	1.7	2.8	8.1	1.0	7.5
BTF3L4	9.9	3.4	5.6	0.5	2.0	2.7	7.8	1.0	7.5
HEBP2	10.7	4.3	6.0	0.6	2.1	2.4	6.5	0.9	7.0
PRPS2	9.7	3.8	4.9	1.0	2.1	3.4	7.6	1.2	6.7
LOC100287015	12.5	2.7	5.8	0.8	1.7	1.5	9.8	0.8	4.7
UBE2J2	10.7	3.5	5.0	0.8	1.7	2.1	8.0	1.6	6.8
PDE12	9.8	2.9	4.2	1.1	1.9	2.8	9.3	1.3	6.9
TRIM37	11.4	4.0	5.5	0.4	1.5	2.0	8.4	0.9	6.2
HS2ST1	10.5	4.0	5.2	0.3	1.6	1.7	9.1	1.3	6.6
TSEN54	9.8	3.9	6.0	0.9	1.4	2.2	9.2	1.2	5.7
VPS26A	11.5	4.1	6.3	0.3	1.9	1.9	7.4	0.9	5.9
SPPL2B	12.5	5.0	6.8	1.5	2.2	3.5	5.0	0.8	2.9
ZNF408	10.7	3.8	4.7	1.0	1.7	2.3	7.9	1.7	6.4
FBXL19	10.0	4.0	5.0	1.4	2.2	3.1	7.6	1.4	5.5
ATP6V1C1	10.7	4.3	5.3	0.3	1.3	1.4	9.4	1.0	6.5
CRHR1-IT1	13.8	2.8	5.5	0.7	1.5	1.9	9.4	0.8	3.9
UBE2J1	11.0	3.9	4.7	0.5	1.2	1.8	9.8	0.8	6.6
PROSER1	10.1	3.2	4.1	0.6	1.6	2.1	7.6	1.4	9.5
GSR	12.8	4.4	5.4	0.7	1.1	2.4	7.4	1.1	4.9
CNOT8	9.8	4.6	5.2	0.4	1.0	1.8	10.3	0.8	6.3
SNHG12	8.9	4.2	4.9	0.7	1.1	2.1	10.3	1.1	6.8
DDX19A	9.7	4.2	5.6	0.7	1.5	3.0	8.3	1.0	6.0
ZCCHC8	11.5	3.8	5.8	0.5	1.3	2.0	7.6	1.1	6.5
PRPF4	9.1	3.7	4.8	0.3	1.7	1.7	10.1	1.3	7.6
BFSP1	12.3	5.0	6.7	0.4	1.6	1.6	6.4	0.9	5.2
FAM21A	14.7	2.5	6.4	0.7	1.5	1.2	7.8	0.7	4.5
GPATCH4	8.5	3.6	4.2	1.3	2.6	3.5	7.5	1.7	7.2

ULK3	11.6	5.3	7.9	0.6	1.4	1.8	5.8	1.1	4.8
SOCS2-AS1	12.4	2.7	5.2	0.8	1.6	1.9	10.1	0.6	4.8
HIATL1	12.5	4.3	5.8	0.6	1.2	2.3	7.0	0.9	5.4
HTRA2	10.2	4.2	5.0	1.2	2.5	3.9	7.3	0.8	4.9
FGF13	10.2	3.9	6.4	0.7	1.8	2.9	6.5	1.1	6.5
TRADD	12.6	4.8	6.9	1.5	1.9	2.7	6.3	0.9	2.6
TMX2-CTNND1	12.4	4.2	5.8	0.6	1.7	2.4	6.7	0.9	5.3
ARIH1	10.4	3.8	4.5	1.6	2.6	1.8	8.4	1.1	5.9
KIAA0368	10.8	3.8	4.6	0.5	1.9	2.6	8.4	1.0	6.4
CCDC9	10.7	4.7	5.5	0.9	2.0	2.5	7.2	1.2	5.3
IL15RA	11.0	4.3	5.8	1.1	2.1	3.1	6.3	1.1	5.3
PHF3	10.1	3.4	4.7	0.4	1.6	2.3	8.7	1.3	7.5
HSPA14	11.0	3.2	5.0	0.5	1.5	1.9	9.9	0.9	6.1
LIME1	10.7	4.9	7.8	1.5	1.7	2.2	6.3	1.2	3.8
ATMIN	11.0	4.3	5.0	0.6	1.9	2.6	7.4	1.2	6.0
NLK	9.0	3.9	4.5	0.6	1.7	2.4	8.5	1.4	8.0
PITPNC1	11.7	3.2	4.3	0.6	1.4	2.0	9.1	1.2	6.6
LOC101929676	13.6	2.7	5.3	0.8	1.9	2.1	9.2	0.5	3.8
STK25	10.0	4.0	5.4	1.7	3.0	4.2	5.7	0.9	4.9
TTC38	11.0	4.3	5.2	1.5	2.4	3.6	6.4	0.9	4.7
LOC101928224	13.1	2.9	5.1	0.8	1.6	1.8	9.5	0.7	4.3
GLIS2	11.4	3.9	4.7	1.3	2.4	2.6	6.8	1.3	5.5
MSANTD3	9.5	2.3	2.8	2.1	1.0	2.0	9.8	1.0	9.3
LOC100126584	12.7	2.8	5.3	0.7	1.2	1.9	10.8	0.5	3.9
VPS16	12.6	5.4	7.4	0.9	2.1	2.6	4.8	0.5	3.5
CHPT1	10.7	4.3	5.5	1.3	2.3	3.0	6.3	1.0	5.3
SHOX2	11.1	4.4	5.5	1.6	2.3	3.3	5.8	1.4	4.5
TBC1D9	10.6	3.7	4.8	0.7	1.7	2.6	8.2	1.2	6.3
VRK3	10.5	4.8	6.2	0.8	2.0	2.5	6.9	1.1	5.1
MPV17L	13.5	2.9	6.3	1.0	1.5	1.7	8.6	0.5	3.7
COMMD9	11.8	5.3	6.5	0.7	1.9	3.1	5.4	0.8	4.3
LOC101927257	13.0	2.8	5.2	0.6	1.5	1.9	10.0	0.7	4.1
BPTF	12.3	3.9	5.4	0.8	1.5	2.1	6.8	1.2	5.7
PRKAB1	9.6	4.7	5.1	1.1	2.3	3.2	6.5	1.5	5.8
TSPYL2	10.7	4.6	6.1	1.0	1.5	2.1	5.6	1.7	6.6
UTP14A	9.1	3.6	4.9	0.4	1.8	2.2	8.9	1.1	7.7
TRIM56	10.5	2.8	4.6	1.2	2.5	3.0	8.2	1.3	5.7
MAPKAPK5-AS1	10.7	3.6	5.4	0.9	2.0	1.9	8.6	1.0	5.5
UBXN4	10.5	3.7	6.3	0.5	1.6	2.5	7.2	0.9	6.4
MFF	10.9	3.7	4.7	0.7	2.5	2.8	7.7	1.2	5.7
MCTP1	10.7	4.0	5.2	0.4	1.5	1.8	8.3	1.1	6.7
TAF1	12.8	3.8	5.6	0.5	1.6	2.0	7.1	0.8	5.5
PARP4	11.6	4.1	5.2	0.6	1.8	2.8	7.0	1.1	5.5
TOX2	11.5	5.3	6.7	2.3	3.8	4.8	2.1	0.7	2.4
NUP88	10.3	3.7	5.2	0.7	1.9	2.9	8.2	0.9	5.9
CCDC106	13.0	4.8	6.8	1.4	2.5	3.2	4.1	0.7	3.2
FLAD1	10.2	3.8	5.7	0.8	1.7	2.8	7.4	1.3	5.9
ZNF639	8.5	3.0	4.4	0.2	1.4	1.6	10.2	1.1	9.3
ERBB2	10.7	4.1	5.4	1.2	2.5	2.8	7.0	1.0	4.9
TMEM159	10.3	4.3	7.0	0.6	0.8	2.1	7.7	0.9	6.0
TDP2	10.3	3.4	4.9	0.6	1.9	2.7	8.1	1.3	6.3
C11orf73	10.5	3.7	7.2	0.6	2.4	3.0	6.0	0.6	5.5
RIN3	10.4	4.1	4.9	1.4	2.1	2.8	6.8	1.2	5.7
UBAP2	10.0	3.9	4.6	1.1	2.0	3.0	6.5	1.4	7.0
WWOX	10.8	5.0	5.3	0.9	1.7	2.9	6.9	1.4	4.6

G6PC3	11.3	4.4	5.2	1.7	3.6	4.5	4.5	0.5	3.6
OCEL1	11.8	5.0	6.5	1.2	2.4	3.1	5.1	0.7	3.6
WDR54	9.2	3.9	4.7	1.4	2.5	4.7	6.9	1.0	5.1
FBXL19-AS1	12.4	2.7	5.4	0.6	1.6	1.8	9.6	0.8	4.5
CDK11A	10.7	4.2	5.9	1.0	1.9	2.7	6.6	1.3	5.3
LEPROT	12.8	6.1	8.9	0.4	1.3	2.5	4.2	0.2	2.9
MESDC1	7.7	3.0	4.1	1.5	1.6	2.8	10.3	1.7	6.7
EVA1A	9.3	2.8	5.7	1.1	1.7	1.9	10.5	1.2	5.2
OIP5-AS1	11.6	3.5	5.3	0.7	1.7	2.0	8.8	0.8	5.0
FAM129C	12.6	2.7	5.6	0.7	1.5	1.8	9.1	0.7	4.6
TRNC	11.4	5.1	6.6	1.8	1.6	3.0	4.0	1.6	4.1
FAM107B	10.5	4.2	5.1	0.6	1.9	2.6	6.8	1.1	6.5
CTH	7.9	3.1	3.5	0.6	1.9	2.7	9.8	1.1	8.8
SMARCA5	11.1	4.0	4.3	0.4	1.4	1.9	8.3	1.2	6.8
KLHL42	10.4	2.9	4.5	1.3	2.2	2.9	7.9	1.3	5.9
TMEM223	8.8	4.1	5.2	0.9	2.2	2.5	8.4	1.1	6.2
ZNF316	9.3	3.6	4.0	1.4	1.9	2.4	9.6	1.6	5.5
BRIX1	10.2	3.7	5.0	0.3	1.7	2.1	8.2	1.1	6.9
SPRED1	11.1	3.5	4.9	0.4	1.8	2.1	8.0	0.8	6.7
TFDP2	11.0	3.3	5.4	0.7	1.6	2.9	7.5	1.0	5.9
MAU2	12.1	4.9	5.5	1.4	2.1	3.0	5.2	1.1	4.0
NR2C2	11.3	3.2	4.8	0.5	2.1	2.6	6.5	1.2	7.1
MGC16142	12.8	2.4	4.7	0.9	1.6	2.1	10.1	0.5	4.2
YES1	11.0	4.2	4.9	0.3	1.3	2.0	7.9	0.9	6.7
GTPBP6	10.0	4.3	5.2	1.4	2.7	2.8	6.2	1.5	5.2
GTPBP6	10.0	4.3	5.2	1.4	2.7	2.8	6.2	1.5	5.2
ZBED4	9.2	3.0	4.2	0.8	1.6	2.3	9.3	1.6	7.1
PACSIN3	8.3	3.1	4.6	1.3	2.8	3.5	8.6	1.3	5.7
VAPB	9.9	4.0	4.7	0.6	2.0	2.1	8.4	1.5	5.9
TEAD2	11.5	4.9	6.2	0.8	1.7	2.7	6.0	0.9	4.4
EXOSC3	8.3	2.5	4.8	1.0	1.9	2.9	10.5	1.1	6.3
ATE1	10.5	3.5	5.0	0.5	1.5	1.9	9.2	1.0	6.0
GAS8	11.4	4.5	6.2	1.1	2.1	2.8	5.4	0.8	4.9
HIVEP2	12.9	4.2	5.7	0.7	1.8	2.5	5.2	1.0	5.0
SH3D19	11.9	4.2	5.6	0.6	1.5	2.2	7.1	0.9	5.1
EML3	11.1	4.4	6.3	1.6	2.7	3.8	5.1	0.8	3.4
LOC101928451	11.2	4.1	5.8	0.8	1.7	2.0	6.7	1.2	5.5
WAPAL	9.9	3.4	4.5	0.3	1.1	2.0	8.8	1.1	8.0
CPSF2	9.4	3.7	4.8	0.2	1.7	1.9	8.8	1.0	7.4
PAPD7	9.3	3.4	4.9	1.1	1.4	2.3	8.1	1.8	6.8
ELK4	11.6	3.3	4.7	0.5	2.0	2.6	6.0	1.0	7.2
ORAI2	8.4	3.9	4.8	1.4	2.2	3.0	8.4	1.6	5.4
MTIF2	10.7	4.0	5.9	0.3	1.6	1.7	7.9	1.0	5.9
STX18	10.8	3.9	5.0	0.5	2.1	2.4	7.7	1.2	5.7
UCHL3	8.7	3.5	4.7	0.2	1.2	1.8	10.9	1.1	6.9
PAM16	8.8	3.3	5.0	0.9	2.2	2.3	10.4	0.0	6.0
PDPR	10.3	3.1	4.4	0.7	1.7	2.6	8.8	1.1	6.4
DDX51	11.6	4.2	5.6	1.3	1.7	2.4	6.4	1.0	4.7
LIMCH1	10.2	3.7	4.7	0.7	2.0	2.5	8.1	1.3	5.8
ZWINT	9.2	3.8	4.9	1.0	1.8	3.4	8.5	1.2	5.1
GGPS1	12.5	3.4	6.3	0.6	1.3	2.0	7.7	1.0	4.2
FOXQ1	7.5	3.2	4.2	3.3	4.6	5.2	4.9	1.4	4.6
DPH5	10.2	3.8	6.3	0.6	1.3	2.2	6.9	1.0	6.7
NOL6	9.9	2.8	4.3	1.3	2.2	3.1	8.1	1.5	5.7
TIMM22	9.9	3.8	4.1	0.3	1.7	2.1	10.0	1.3	5.7

GRIPAP1		10.2	3.7	5.1	1.0	2.0	2.8	7.2	1.1	5.9
RNF6		8.9	3.4	4.9	0.4	1.5	2.3	9.0	1.3	7.3
METAP1		8.8	3.5	4.8	0.4	1.1	1.9	8.5	1.5	8.5
HINT2		10.7	5.0	5.4	1.1	2.5	3.1	6.6	0.9	3.6
HDAC6		11.4	4.9	6.5	0.8	2.0	3.0	5.1	0.8	4.2
DLD		10.6	4.0	5.1	0.2	1.8	1.9	8.4	0.8	6.2
PTPN2		9.0	3.4	5.5	0.3	1.3	1.8	9.1	1.2	7.4
CERK		12.2	4.2	5.0	1.0	1.8	2.7	6.5	0.9	4.7
LOC101928174		12.3	2.4	5.2	0.6	1.8	1.7	10.2	0.6	4.2
MRPL40		10.4	5.6	4.9	1.1	3.5	3.3	4.8	0.7	4.6
EFCAB14		13.2	5.3	5.0	0.5	1.2	1.8	6.3	0.8	4.7
MRPS25		9.0	3.7	4.9	1.1	3.1	3.8	6.2	1.0	6.0
MAD2L1BP		10.8	4.1	5.5	0.4	2.3	2.5	6.7	0.9	5.6
ZNF205		9.6	3.8	5.1	1.6	2.2	2.5	6.3	1.9	5.8
DCAF12		9.9	3.6	4.2	0.5	1.5	2.0	8.6	1.2	7.3
COPS7A		12.3	3.4	5.6	1.3	3.0	3.5	4.9	0.7	4.2
LPIN2		11.6	4.3	5.8	0.5	1.3	2.0	7.0	0.9	5.5
MED12		9.1	3.3	4.8	1.2	2.6	3.5	6.9	1.3	6.1
LINC00888		12.0	3.9	6.2	0.8	1.2	2.0	7.2	0.9	4.6
ALG13		12.7	3.6	5.6	0.6	1.7	1.8	7.2	0.8	4.8
PWWP2B		10.0	4.0	5.6	1.4	2.4	2.5	6.9	1.2	4.9
LTB4R2		10.8	3.7	6.2	1.9	2.5	2.8	7.0	0.7	3.3
DHX40		11.8	4.1	5.5	0.4	1.5	1.9	7.2	0.9	5.4
NAB1		12.1	3.6	6.2	0.5	1.3	1.8	7.2	0.6	5.4
AP1S2		11.8	4.2	5.1	0.3	1.8	2.4	6.8	0.9	5.3
	44814.0	11.1	4.1	5.7	0.6	1.8	2.4	6.8	0.9	5.3
INPP4B		9.8	3.6	4.6	0.5	1.4	1.9	8.0	1.4	7.5
LINC00094		12.0	3.2	5.2	0.8	1.6	2.0	8.5	0.9	4.6
RPL22L1		8.8	3.7	4.7	0.2	0.8	2.1	8.1	1.5	8.8
TRMT6		10.5	4.6	5.5	0.4	1.1	2.3	6.8	1.0	6.4
IKZF3		12.9	2.4	5.4	0.7	1.4	1.7	9.5	0.7	4.0
VKORC1L1		8.9	3.5	5.1	1.0	2.3	2.2	8.1	1.6	5.9
ZBTB17		10.9	3.6	5.3	1.4	1.9	2.7	6.8	1.3	4.8
ESD		9.7	4.3	5.4	0.3	1.6	2.6	8.2	1.0	5.6
C9orf114		8.6	3.5	4.0	1.3	2.2	2.4	8.5	1.4	6.7
MUL1		11.8	4.4	5.2	0.7	1.1	2.6	7.0	0.9	5.0
CHCHD6		9.1	3.9	5.6	1.4	2.7	3.4	6.5	1.1	4.8
GSTZ1		10.2	4.7	7.0	1.4	4.7	4.7	3.3	0.4	2.3
FOXRED1		9.2	4.1	5.0	1.0	2.5	2.9	7.3	0.7	5.8
MAN1B1-AS1		12.4	2.9	5.6	0.6	1.5	1.7	8.9	0.8	4.2
USP32		10.6	3.7	4.8	0.4	1.3	2.1	8.3	1.0	6.3
TBP		8.9	3.9	5.3	1.4	1.9	2.1	7.2	1.3	6.7
MRPL20		8.7	3.6	5.2	0.7	1.6	2.4	8.2	1.5	6.7
RFC2		9.6	3.7	5.1	0.7	1.9	2.8	7.4	1.2	6.3
CDYL		9.6	3.9	4.9	0.6	1.5	2.1	7.6	1.5	7.0
LOC101927559		12.3	2.7	5.1	0.8	1.6	1.8	9.2	0.8	4.2
GPATCH8		10.6	3.7	4.8	0.9	1.8	2.6	6.4	1.2	6.5
SMARCD1		9.5	3.4	4.7	0.7	1.9	2.3	7.9	1.2	7.0
SUGP1		10.2	4.2	4.7	1.1	2.1	3.4	6.0	1.1	5.7
FITM2		10.4	4.4	5.2	1.3	1.9	3.2	6.7	1.3	4.2
STAG2		10.1	3.3	4.7	0.3	1.2	2.2	8.8	1.1	6.9
SORT1		11.7	4.3	4.9	0.9	1.7	2.4	6.7	1.1	4.9
HHLA3		12.6	2.8	4.0	0.7	1.2	2.1	8.4	1.3	5.5
RALGAPB		10.9	3.2	4.6	0.4	1.6	2.3	8.3	1.0	6.2
PTPLB		11.9	5.4	5.4	0.5	1.3	2.1	6.3	1.0	4.7

SERTAD1	9.3	4.6	7.2	0.6	1.3	1.6	7.9	1.5	4.7
SAT2	11.6	5.2	6.2	0.4	1.5	1.0	8.1	0.5	4.1
CNTROB	10.4	3.8	4.7	1.0	2.0	2.8	6.9	1.3	5.6
GNB1L	9.5	2.9	4.3	1.2	1.6	2.0	9.7	1.7	5.5
KDELC2	11.7	4.6	6.0	0.9	1.7	2.6	6.2	0.7	4.1
FAM178A	13.7	3.3	5.8	1.1	1.7	2.2	7.1	0.5	3.3
AP3M1	10.2	4.0	5.2	0.3	1.8	2.4	7.0	1.1	6.5
ATM	12.1	3.2	6.1	0.3	1.5	1.7	7.7	0.7	5.2
PI4K2A	10.6	3.8	4.4	0.8	1.5	2.1	8.1	1.2	6.0
MOCS1	9.9	3.9	4.7	1.2	2.7	3.5	6.8	1.1	4.8
CCAR1	11.8	4.3	5.5	0.6	1.5	2.2	5.7	0.8	6.0
DST	10.9	2.7	4.8	0.4	1.6	2.0	9.0	0.9	6.1
GOLGB1	11.0	3.1	4.7	0.4	1.6	2.0	8.3	1.0	6.4
ALG8	11.0	4.5	5.0	0.5	1.3	2.5	6.9	1.0	5.8
U2SURP	10.3	4.0	5.6	0.4	1.6	2.4	7.5	1.0	5.7
MFSD5	10.4	4.4	4.8	0.7	1.9	2.4	7.2	1.1	5.4
SNORA14B	7.9	5.1	3.9	1.4	1.6	3.4	5.5	1.3	8.3
PDCD7	10.1	3.7	4.5	1.4	2.1	3.0	6.6	1.4	5.6
BCL3	15.6	6.5	8.0	1.1	1.7	2.0	1.5	0.5	1.4
DIEXF	9.9	3.6	4.3	0.5	1.9	2.4	8.3	1.3	6.2
TP53INP2	11.8	4.9	5.1	1.8	2.8	4.0	3.8	0.8	3.3
PCGF3	10.1	3.7	5.3	1.1	1.8	2.5	5.4	1.2	7.1
SLC35E2B	12.0	4.9	6.7	1.3	2.0	3.0	4.0	0.3	4.1
RRP15	8.5	3.0	4.0	0.5	1.5	2.1	9.5	1.6	7.5
TMA16	9.1	3.5	4.8	0.5	1.9	2.6	7.0	1.2	7.8
TAF13	8.1	2.7	4.1	0.1	1.1	1.3	11.4	1.1	8.3
ZCCHC9	8.9	3.9	5.1	0.3	1.5	2.0	8.0	1.4	7.2
LOC101929694	12.5	2.8	5.5	0.7	1.6	1.6	9.2	0.6	3.8
CHFR	11.0	3.2	5.6	1.0	1.7	1.9	7.5	1.4	5.0
SGPP1	10.2	3.8	5.6	0.9	1.8	2.6	6.8	1.1	5.5
GUSB	11.6	4.5	6.5	0.7	1.4	2.3	6.0	1.0	4.3
SEMA3C	13.9	5.1	6.7	0.5	1.6	2.1	4.6	0.8	3.0
TARBP2	10.2	4.4	5.6	1.1	2.3	2.8	6.2	0.8	4.9
TTPAL	9.6	2.6	4.4	0.8	1.7	2.3	9.9	0.9	5.9
GTF2B	8.3	4.3	5.8	0.4	1.6	1.8	8.1	1.4	6.5
LINC00910	12.2	2.9	5.8	0.7	1.7	1.7	8.3	0.7	4.1
LOC100506990	11.5	2.7	5.3	0.6	1.9	2.2	7.9	0.9	5.3
TRIOBP	10.8	4.2	4.4	2.0	3.7	4.5	5.8	0.8	2.1
WDR13	9.5	3.6	5.1	1.2	2.5	3.8	6.5	0.9	5.1
TSC1	12.2	4.7	6.3	0.6	1.5	1.9	5.7	0.9	4.3
GRAMD3	13.7	4.9	7.0	0.5	1.3	1.4	5.1	0.4	3.9
NEXN-AS1	12.5	2.5	5.0	0.8	1.8	2.0	8.5	0.9	4.0
WDTC1	10.2	4.1	5.6	0.9	2.2	2.6	6.1	1.2	5.2
LOC101926928	11.4	2.4	5.7	0.6	1.7	1.8	9.9	0.7	3.9
RGS19	8.2	3.5	4.6	1.2	2.2	2.9	8.5	1.3	5.9
TFAM	10.4	3.6	4.8	0.4	1.3	1.8	9.0	0.8	6.0
C17orf85	9.5	3.0	4.6	0.9	2.0	2.5	8.0	1.3	6.4
MAML2	10.9	3.7	5.7	1.1	2.3	2.8	4.8	1.1	5.7
EPS15	9.9	4.4	5.1	0.3	1.5	1.8	8.0	0.9	6.2
APBA3	10.7	4.2	5.4	1.6	2.1	2.9	5.8	1.1	4.3
KAT8	10.1	3.4	5.0	0.9	1.6	2.3	8.1	1.1	5.6
C22orf39	12.1	4.3	3.4	1.5	1.8	2.8	6.4	1.1	4.6
PORCN	9.2	3.7	5.0	1.9	3.2	5.0	5.4	1.1	3.6
MRC2	12.5	4.7	6.7	1.2	2.1	3.1	3.8	0.8	3.3
POLR3GL	9.5	5.2	5.8	1.0	1.9	2.7	6.5	0.9	4.6

SPNS1	11.4	3.9	5.1	1.2	1.7	2.6	6.5	1.1	4.6
TMEM97	7.7	4.6	6.2	0.8	1.5	2.1	5.2	1.7	8.3
PSMD1	10.2	4.1	4.9	0.7	1.8	2.5	7.4	0.9	5.4
DNAJB9	7.6	3.7	4.5	0.1	1.3	1.7	9.4	1.1	8.6
TMEM183A	9.7	3.8	5.2	0.6	1.3	1.8	8.5	1.1	5.9
RTF1	10.9	4.2	5.0	0.9	1.9	2.7	5.7	1.2	5.5
MECOM	9.0	3.3	4.0	0.5	1.5	2.2	8.5	1.5	7.5
LOC101928413	12.4	3.4	5.6	0.5	1.4	1.7	7.7	0.5	4.7
ARL2BP	9.8	4.3	5.1	0.3	1.5	2.2	7.5	1.0	6.3
SPG20	10.7	3.8	5.1	0.3	1.6	1.8	7.2	1.0	6.5
SOAT1	10.4	3.5	5.6	0.5	1.6	2.2	7.2	1.0	6.0
WASIR2	11.3	2.6	5.9	0.6	1.0	1.7	10.5	0.7	3.8
MRPL48	9.4	4.5	5.6	0.4	1.0	1.5	9.2	0.8	5.6
WIPF1	10.0	4.1	5.0	1.0	1.9	2.8	5.0	1.6	6.4
DCTN5	10.4	3.9	5.7	0.7	1.5	2.1	7.1	1.0	5.5
GLIPR1	8.8	3.3	4.5	0.6	2.9	2.8	6.4	1.3	7.3
PPCS	8.9	3.5	5.0	0.8	2.3	2.4	7.8	1.1	6.1
EXOSC7	9.4	3.5	4.9	0.4	0.9	1.4	9.3	1.2	6.9
UXS1	11.8	4.4	5.5	1.0	2.4	2.6	5.5	0.7	3.9
CSNK1G3	9.2	4.4	4.3	0.3	1.1	1.6	8.8	1.1	7.1
GAR1	9.2	4.0	4.2	0.7	1.5	1.6	7.4	1.0	8.1
PIGU	11.6	4.1	5.5	0.9	2.2	2.4	5.6	0.9	4.6
DENND3	12.3	4.2	6.0	1.2	2.5	2.8	4.6	0.8	3.5
LOC101927838	10.5	2.7	4.0	1.0	1.6	2.1	8.6	1.3	5.9
PPP1R35	11.5	3.9	5.6	1.2	2.4	2.5	5.9	0.9	3.9
CHMP2B	10.4	4.6	5.8	0.3	1.2	1.4	7.6	1.0	5.4
MKI67IP	9.7	3.9	4.5	0.3	1.7	2.2	7.0	1.1	7.4
SLC41A1	9.7	3.1	4.3	0.7	1.6	2.2	8.1	1.2	7.0
SPEN	9.6	2.7	4.2	0.9	1.7	2.5	7.7	1.4	7.1
LOC101926911	11.5	3.0	5.0	0.7	1.8	1.5	9.4	0.8	4.1
EPG5	10.6	2.9	4.7	0.7	1.9	2.6	8.2	0.8	5.3
MRPS18C	10.7	4.3	5.2	0.4	1.3	2.3	6.7	0.9	6.0
AMD1	10.8	3.4	5.6	0.2	0.9	1.6	8.6	0.7	5.9
GTPBP2	8.6	3.8	4.7	1.1	2.6	3.3	6.9	1.3	5.3
GMDS	9.6	3.4	4.7	0.6	1.8	1.9	8.9	1.1	5.8
ZNF462	10.4	3.1	4.3	1.0	2.2	3.1	6.7	0.9	6.1
NFKB1	9.8	3.3	3.9	0.4	1.0	1.6	10.0	1.1	6.7
DYNLT3	11.5	4.8	5.2	0.3	1.7	2.9	6.1	0.8	4.4
ZNF777	8.2	3.1	3.9	1.2	1.7	2.3	7.7	1.5	8.0
GPN1	9.7	3.8	5.5	0.4	1.3	2.1	7.3	0.9	6.7
C7orf50	10.3	4.0	5.1	0.9	2.0	2.3	7.8	0.7	4.6
PIGK	10.8	3.9	5.2	0.4	1.6	2.4	6.9	1.0	5.4
PMPCB	10.0	4.0	5.4	0.3	1.7	2.1	7.3	0.7	6.0
RABIF	9.2	2.4	3.5	0.5	2.0	2.0	10.2	1.2	6.7
MCAT	10.0	3.8	4.8	0.9	1.7	1.9	8.3	1.3	4.9
LOC101927692	12.0	2.7	5.0	0.5	1.6	2.1	9.0	0.6	4.1
NR1D1	8.6	3.4	4.5	1.2	2.1	2.9	6.6	1.7	6.6
GBE1	10.0	3.3	4.9	0.5	1.6	2.1	8.2	0.9	6.0
TRAPPC10	10.9	3.3	5.0	0.8	1.7	2.2	7.2	1.1	5.3
RSPRY1	9.6	4.0	5.4	0.6	1.5	2.1	7.8	1.1	5.5
GRPEL2	9.0	3.7	4.3	0.5	1.6	2.1	8.8	1.0	6.6
GPHN	9.6	2.9	4.0	0.6	1.6	2.2	7.8	1.6	7.2
ZBTB22	9.4	4.0	5.5	1.1	2.2	2.8	6.5	1.2	4.9
ACAA2	10.7	4.1	5.3	0.4	1.2	1.8	8.2	0.9	4.9
GPR125	10.0	3.4	4.6	0.6	1.6	2.0	7.6	1.0	6.8

LOC100996302	9.7	4.2	5.0	0.5	1.1	1.6	7.7	0.8	6.8
LYRM2	10.3	3.9	6.9	0.5	1.3	1.9	5.7	0.9	6.1
TMEM231	10.4	3.9	5.5	0.9	1.8	2.5	6.9	0.9	4.7
UBE2V2	10.6	4.1	4.9	0.5	0.8	1.4	6.4	1.5	7.3
WASF1	10.3	3.8	5.4	0.8	1.7	2.5	5.3	1.1	6.5
MSRA	8.6	3.1	4.5	1.2	2.5	3.6	6.8	1.3	5.8
NAT14	13.7	4.7	5.7	1.4	2.7	2.6	4.6	0.3	1.7
ENTHD2	9.3	4.0	5.7	1.3	2.2	2.4	6.4	1.2	4.8
POU2F2	10.7	4.9	5.2	1.6	2.6	3.4	4.0	0.9	3.9
ATP6V1G2-DDX39B	12.9	5.0	6.2	0.1	0.3	4.4	5.3	0.2	3.1
DOT1L	10.1	3.8	5.2	1.4	1.7	2.7	6.1	1.3	5.0
AK4	10.0	3.6	6.2	0.5	1.6	2.6	6.3	1.3	5.3
BANP	9.9	3.5	4.2	1.1	1.7	2.6	7.7	1.1	5.5
PTPN9	11.1	3.8	5.0	0.7	1.6	2.5	6.5	0.9	5.3
MED28	8.5	3.8	4.5	0.5	2.3	2.4	7.7	1.0	6.6
VIPAS39	10.4	4.3	5.0	0.6	2.1	2.6	6.6	0.7	5.0
PRKAR2A	9.3	3.0	4.3	0.9	2.1	2.2	8.3	1.1	6.3
LOC101929403	10.7	2.8	4.8	1.2	2.3	2.3	8.0	0.8	4.3
ELF1	9.2	3.6	4.4	0.3	1.3	2.2	7.0	1.3	8.0
MYEF2	9.9	4.1	5.7	0.5	1.5	1.9	7.1	0.8	5.8
AFAP1L1	9.1	3.5	4.7	1.3	2.5	2.8	6.8	1.3	5.2
PHF19	7.3	3.4	4.4	0.7	1.8	2.2	9.0	1.8	6.6
CDC16	9.8	3.9	5.5	0.7	1.9	2.0	7.0	1.0	5.5
TMEM50A	10.4	3.5	6.0	0.1	0.7	1.8	7.3	0.8	6.6
ST3GAL3	9.5	3.7	4.7	1.5	3.3	3.2	6.2	0.8	4.4
SNORD56	11.2	2.9	3.7	1.4	2.0	3.6	3.7	2.6	6.0
OXSR1	9.1	3.5	4.8	0.4	1.5	2.0	7.9	1.1	7.0
C12orf65	8.2	3.4	4.0	0.6	1.7	2.5	9.2	0.9	6.8
SLC9A1	9.5	3.6	4.7	0.9	1.7	2.3	6.7	1.3	6.4
TUSC1	8.6	2.8	4.8	2.0	1.8	2.7	7.5	1.2	5.8
PMVK	11.1	3.7	5.5	0.7	1.8	2.6	6.5	0.7	4.6
FBXO11	10.2	3.3	4.4	1.4	1.8	2.4	6.9	1.3	5.5
NOM1	9.5	3.1	4.3	0.7	1.8	1.9	8.8	1.4	5.7
DGCR14	11.7	4.1	5.3	1.2	2.3	2.2	5.2	0.9	4.3
PCGF5	8.7	4.3	5.1	0.5	1.6	2.7	7.0	1.0	6.3
TCEAL1	9.4	4.0	6.3	0.6	2.8	3.2	5.3	1.2	4.3
WHSC1L1	9.2	3.3	4.4	0.4	1.3	2.0	7.7	1.3	7.7
HOOK2	10.8	3.7	5.6	1.4	2.8	3.3	5.7	0.7	3.1
AP3B1	10.1	3.2	4.6	0.5	1.3	1.7	8.6	1.0	6.2
PPP4R1	9.6	3.8	4.6	0.5	1.3	2.2	8.2	1.0	6.1
ZNF644	9.8	3.6	4.6	0.2	1.2	1.4	8.8	0.9	6.6
LRRC42	8.9	3.4	5.4	0.3	1.3	1.7	8.3	1.0	6.9
ANKRD28	8.9	3.2	4.3	0.6	1.6	2.3	8.4	1.2	6.7
CUL9	12.3	4.0	6.6	1.0	2.1	2.8	4.6	0.6	3.1
LOC100507431	12.3	2.7	5.3	0.6	1.6	2.0	8.0	0.7	4.0
AIG1	10.5	3.7	6.8	0.3	2.2	1.3	7.9	0.8	3.7
SERTAD4-AS1	9.6	3.7	6.8	0.5	0.9	1.2	8.4	0.8	5.1
RNF208	10.5	4.8	6.0	1.8	2.6	3.2	4.0	0.7	3.5
IQCH-AS1	12.5	2.9	4.4	1.1	1.5	1.7	8.4	0.9	3.6
LOC101927319	11.8	2.3	4.8	0.9	1.4	2.4	9.3	0.7	3.4
RGP1	10.1	4.0	5.8	1.0	2.0	3.1	5.4	1.0	4.6
ZNF628	9.3	3.9	4.9	1.4	1.7	2.3	6.5	1.5	5.5
HMG20A	10.0	4.3	5.2	0.5	1.7	2.4	5.7	1.0	6.2
OTUD4	9.6	4.0	4.3	0.5	1.6	1.7	8.0	1.2	6.1
PABPC1L	8.9	3.6	5.5	1.6	2.9	3.5	5.3	0.8	4.8

EXOC5	9.9	4.1	4.7	0.2	1.3	1.7	7.4	0.8	7.0
ALDH3B1	12.2	4.7	6.1	1.7	2.4	3.5	3.6	0.6	2.2
HECTD4	12.0	3.3	5.6	0.8	1.8	2.1	6.2	0.9	4.3
MRPS21	9.5	4.7	5.3	0.9	2.3	3.1	5.4	1.0	4.9
FAM102A	11.5	3.9	6.3	1.4	1.9	3.3	4.7	0.8	3.2
SLC29A2	7.0	3.4	4.4	1.5	2.1	3.3	6.4	1.3	7.5
TSPAN15	11.6	5.2	6.3	0.5	1.6	2.5	4.0	0.8	4.5
LOC101929368	10.8	4.3	6.5	0.3	1.4	2.3	2.9	1.6	7.1
KLF9	10.1	4.1	5.2	1.5	2.0	3.8	4.7	1.1	4.5
CNOT11	11.1	4.0	5.3	0.9	2.1	2.5	5.7	1.0	4.2
SLC35E2	11.1	4.4	5.7	1.0	2.6	2.4	5.4	0.7	3.7
GNL2	8.2	3.2	4.1	0.5	1.2	1.9	8.7	1.3	7.8
CDC42BPA	10.0	2.9	4.4	0.4	1.5	2.1	8.4	1.0	6.1
SS18L1	10.9	2.4	4.5	0.9	2.1	2.3	7.2	1.3	5.2
RASSF7	10.2	4.2	5.5	0.6	1.5	2.4	7.1	1.0	4.4
ZFYVE1	11.2	4.0	5.4	0.7	1.4	1.7	6.8	1.1	4.6
SUN2	10.3	3.8	4.5	1.6	2.9	4.1	5.2	1.1	3.4
AKAP13	9.5	3.0	4.7	0.7	1.8	1.9	8.6	1.1	5.7
CRLS1	10.4	3.8	5.4	0.9	1.7	2.5	6.4	1.0	4.9
NOC4L	9.0	4.0	4.8	1.5	2.4	3.4	5.5	1.3	5.0
TK1	7.1	2.3	3.6	0.8	2.4	3.0	9.7	1.5	6.5
PRPF38A	9.6	2.9	4.1	0.8	1.6	2.1	8.5	1.4	5.7
BRAP	9.1	3.5	5.0	0.5	1.7	2.3	7.3	1.0	6.6
LOC729867	8.8	3.4	4.6	1.6	1.9	2.2	7.9	1.3	5.1
SCAF8	10.0	3.7	5.0	0.6	1.4	2.4	6.8	1.1	5.9
MSANTD4	10.7	3.5	4.9	0.4	1.4	2.3	7.3	1.0	5.2
ANKS1A	11.1	3.5	5.2	1.1	1.8	2.1	5.7	1.2	5.2
EXOC4	9.9	3.6	4.9	0.8	2.2	2.8	6.4	0.8	5.5
NPB	10.9	4.6	5.4	1.7	1.5	3.6	4.7	0.9	3.6
VDAC3	8.6	4.3	5.5	0.2	1.1	1.7	8.6	1.0	6.0
UBE2A	9.7	4.8	5.6	0.4	1.8	1.8	6.1	1.0	5.6
SIK1	8.3	3.3	4.0	1.5	2.1	3.1	7.4	1.5	5.6
CHD6	11.1	3.0	5.0	0.9	1.9	2.3	6.5	0.8	5.3
UBAC2	11.0	3.7	5.6	0.9	1.9	2.8	5.4	0.9	4.6
UGP2	9.9	3.9	5.6	0.2	1.4	2.0	6.8	0.9	6.0
GALNT6	11.8	4.8	5.9	0.9	1.7	2.6	4.5	0.8	3.7
NCOA7	11.2	3.1	5.2	0.5	1.0	1.9	7.8	0.8	5.4
AIDA	10.1	3.8	4.8	0.5	1.8	2.2	7.1	0.8	5.6
TPD52	8.1	3.0	4.3	0.5	2.1	2.2	8.2	0.8	7.5
PTBP3	9.4	3.8	4.7	0.4	1.4	2.3	7.2	1.1	6.5
FAM74A1	10.4	2.5	5.8	0.6	0.8	1.3	9.0	1.1	5.3
CMBL	10.8	5.1	5.0	0.4	1.2	1.6	6.1	1.3	5.1
SCARF2	9.4	3.4	4.4	1.3	2.0	2.6	7.1	1.5	5.1
GON4L	10.6	3.0	5.0	0.6	1.3	2.3	7.4	1.0	5.4
LOC729652	12.2	2.3	5.5	0.8	1.3	1.8	8.5	0.7	3.7
ZBTB7B	10.5	4.1	4.9	1.2	1.7	2.5	5.9	1.1	4.8
ERCC3	10.4	3.7	5.6	0.5	1.5	2.0	6.4	1.1	5.5
ZNF609	8.7	2.9	3.9	0.9	1.7	2.1	7.9	1.3	7.3
ESM1	2.0	1.1	1.2	0.5	0.8	1.1	15.1	1.6	13.3
ZNF76	11.8	5.0	7.0	1.4	2.6	3.5	2.7	0.5	2.1
FAM45A	12.2	4.2	5.3	0.4	1.3	2.0	6.3	0.7	4.2
PGBD2	12.0	2.6	5.5	0.4	1.7	1.5	7.9	0.6	4.3
TCHP	9.0	3.8	4.4	1.1	2.1	2.8	6.4	1.0	5.9
GNG11	9.7	4.7	5.7	0.2	2.3	3.3	4.9	1.2	4.6
ZNF503	7.1	3.0	4.0	2.2	1.7	2.7	7.0	2.4	6.6

SAC3D1		9.6	4.6	6.7	1.5	1.7	2.5	6.1	0.7	3.2
PARP10		10.5	4.8	6.1	1.7	1.9	3.0	5.2	0.6	2.8
UBIAD1		8.1	2.7	4.1	1.3	2.1	3.3	6.7	1.4	6.9
C5orf30		7.4	2.8	3.2	0.4	1.9	2.4	9.9	1.1	7.5
CAB39		9.9	4.2	4.0	0.4	1.9	2.4	6.8	1.0	6.0
LIPA		11.3	4.5	6.4	0.2	1.1	1.9	5.5	0.9	4.7
C14orf142		12.8	4.4	4.6	0.7	2.3	1.4	6.5	0.7	3.3
LIMD1		9.9	3.5	4.8	0.6	1.5	1.9	7.9	1.1	5.4
C4orf46		8.6	3.7	4.5	1.4	1.6	1.6	8.7	1.3	5.1
SUGP2		10.7	3.8	5.6	0.7	1.7	2.3	6.2	0.9	4.7
EBPL		8.6	3.4	6.0	0.9	2.0	1.9	7.5	0.6	5.7
GPN3		8.1	3.4	4.6	0.8	1.3	2.1	8.4	1.1	6.6
RPRD2		10.3	3.9	4.8	0.8	1.7	2.6	5.4	1.2	5.9
RBM5		9.7	3.5	4.7	0.7	2.0	2.4	6.2	1.2	6.2
ARMC10		10.3	3.9	4.7	0.5	1.4	1.9	6.8	0.9	6.1
ZSWIM1		10.1	4.3	4.9	1.3	2.1	3.1	4.4	1.1	5.1
BRD1		11.1	3.4	6.2	0.7	1.3	1.8	6.1	0.9	5.0
TCTN3		10.8	3.9	5.3	0.7	2.2	3.1	5.2	0.9	4.5
ZNF414		8.7	3.6	4.9	2.7	3.2	4.4	4.3	1.1	3.6
FCHSD2		10.0	3.8	4.7	0.6	1.9	2.0	6.6	1.2	5.8
CCDC25		10.4	4.6	5.7	0.8	1.7	2.5	5.6	0.9	4.3
ZNF655		10.5	3.0	4.6	0.4	2.0	2.6	7.2	0.8	5.5
GAS6-AS2		11.0	3.0	4.8	0.5	1.5	1.9	8.9	0.9	3.9
UACA		10.8	3.5	4.5	0.3	1.5	2.2	7.1	0.9	5.7
BLOC1S4		8.9	3.6	4.2	1.8	2.2	2.5	7.2	1.1	4.9
FAM222B		9.3	3.3	4.7	1.4	2.5	3.2	5.0	1.4	5.5
RHPN1		10.0	4.2	6.0	1.4	2.2	3.0	5.4	0.8	3.3
ZDHHC6		10.2	3.6	5.1	0.1	1.1	1.3	7.5	0.8	6.7
KIAA1737		9.0	3.4	4.0	0.8	1.7	2.3	8.0	1.2	5.9
HMGCS1		11.8	5.0	6.2	0.1	0.8	0.7	6.2	0.7	5.0
HUS1		11.1	2.8	4.9	0.6	1.2	1.8	8.1	0.8	4.9
TAMM41		10.0	4.5	4.8	0.9	2.2	2.4	5.9	0.8	4.8
SMG1		10.1	2.6	4.3	0.4	1.4	1.6	8.6	1.0	6.2
RPP25L		8.6	3.1	4.9	1.1	2.2	2.9	7.2	1.3	5.0
ZNF830		9.0	3.7	4.1	0.8	1.9	2.3	6.9	1.5	6.1
DNM1L		10.1	3.6	4.8	0.5	1.5	2.3	7.0	0.9	5.5
AP4B1-AS1		10.9	2.9	5.0	0.5	1.3	1.5	9.3	0.8	4.1
C11orf95		10.9	4.2	5.0	1.6	2.2	2.8	4.9	0.7	4.0
SFXN3		9.2	3.4	4.2	0.8	2.3	2.8	7.5	1.1	5.0
ATXN7		9.0	3.5	4.9	0.8	1.6	2.2	6.5	1.3	6.5
NRARP		6.6	2.6	4.2	1.6	2.5	3.4	7.0	1.6	6.9
LSM1		9.3	4.4	5.5	0.2	1.2	2.0	7.2	0.8	5.7
ADPGK		10.7	4.5	5.9	0.6	1.7	2.6	5.4	0.6	4.3
SYAP1		9.8	3.3	4.3	0.5	1.7	1.8	8.1	1.1	5.6
MINA		9.6	3.3	4.5	0.4	1.6	2.4	7.8	0.8	5.9
	44625.0	9.3	4.0	5.5	0.4	1.3	1.5	7.2	1.2	5.9
B3GALT6		10.5	3.0	4.3	1.1	1.9	2.2	7.5	1.3	4.4
C9orf40		8.0	3.8	5.3	1.1	1.4	2.5	7.1	1.3	5.7
PRKAA1		9.1	3.4	5.1	0.7	1.7	1.8	7.0	1.0	6.3
FAM109A		10.3	4.4	5.7	1.8	1.9	2.7	5.3	1.0	3.0
YIPF6		9.5	3.7	6.0	0.5	2.3	2.3	6.3	0.5	5.0
MBD6		8.9	4.0	4.3	1.2	2.1	2.9	5.3	1.2	6.2
ARFGAP3		9.4	3.6	5.0	0.3	1.1	1.8	8.0	1.4	5.5
STAG1		9.2	2.8	4.0	0.4	1.6	1.9	9.2	0.8	6.3
TRAF3IP2-AS1		11.5	2.6	5.1	0.7	1.4	1.9	8.5	0.6	3.8

SVIL	12.3	4.1	5.4	0.7	1.8	2.0	5.0	0.8	4.0
SKIV2L	9.3	3.8	5.2	1.1	2.1	2.8	6.5	0.9	4.5
RPUSD3	9.0	4.2	4.7	1.2	1.8	3.6	6.4	0.9	4.4
TMCO3	8.7	3.3	4.8	0.7	1.5	2.2	7.6	1.2	6.0
MSANTD3-TMEFF1	9.0	3.9	5.4	0.0	1.8	2.7	5.8	1.4	5.9
RWDD1	8.5	3.7	5.4	0.3	1.0	2.5	7.4	1.1	6.2
SLCO4A1	9.6	4.2	5.5	1.8	2.7	3.9	4.0	0.8	3.7
RABGEF1	9.7	3.1	4.0	0.7	1.7	1.9	7.4	1.0	6.6
BROX	9.1	3.7	4.7	0.3	1.4	2.3	7.3	0.9	6.3
LOC284865	12.0	2.4	4.9	0.6	1.3	1.3	9.2	0.6	3.8
BID	10.1	3.6	5.5	0.6	1.5	2.0	6.9	1.0	4.9
METTL1	9.7	3.8	4.4	0.7	2.0	2.1	6.6	1.1	5.6
ACSS3	11.0	4.2	6.4	0.6	1.7	2.3	5.0	0.6	4.2
SLC10A3	9.9	4.4	5.2	1.1	2.0	3.0	4.4	1.1	5.0
NRAS	10.0	3.5	5.5	0.2	1.5	1.7	6.6	0.9	5.9
MERTK	7.6	2.8	3.9	0.5	1.6	2.0	8.7	1.5	7.4
TAF6L	10.0	3.9	4.5	1.0	1.8	2.3	6.7	1.0	4.7
TANC1	9.0	3.0	4.2	0.8	1.7	2.3	8.2	1.1	5.6
INTS5	10.0	3.4	4.7	0.9	1.8	2.3	7.3	0.9	4.7
LOC100506596	11.4	2.7	5.6	0.9	1.4	1.6	7.4	0.9	4.0
WDR36	9.4	3.2	4.3	0.3	1.4	1.9	7.8	0.9	6.7
EIF1AD	8.7	3.5	4.9	0.6	1.3	2.3	6.8	1.2	6.7
BUD13	9.7	4.2	4.4	0.5	2.0	2.4	5.8	1.4	5.6
C7orf49	9.1	2.8	4.3	0.6	3.0	2.5	6.9	1.1	5.8
USP48	11.4	4.0	5.2	0.4	1.2	1.5	7.3	0.6	4.2
IFT43	10.2	3.7	6.9	0.7	2.7	3.1	5.3	0.7	2.7
NARFL	10.9	4.4	5.0	0.9	1.8	2.5	5.5	0.9	4.2
TP53BP1	10.0	3.4	5.1	0.5	1.4	1.8	7.3	1.0	5.3
PTPLA	6.1	2.7	3.4	2.9	3.7	5.1	5.9	1.4	4.8
LOC100506054	7.5	3.1	3.6	0.6	1.4	2.5	8.2	1.6	7.6
CHRNB1	8.9	3.3	4.1	1.0	2.3	2.7	6.2	1.2	6.2
ASH1L	9.8	3.0	4.3	0.4	1.5	1.9	7.9	0.9	6.2
SNAPC4	8.8	4.4	5.2	1.1	1.7	1.9	6.5	0.9	5.3
SNORA26	10.4	5.0	2.4	0.7	1.8	1.8	7.1	1.8	4.9
PHACTR4	10.7	3.5	4.4	0.7	1.5	2.2	5.7	1.2	6.0
TRIM65	9.8	3.3	5.4	1.6	2.6	3.7	5.0	0.8	3.7
EBF4	10.1	4.0	5.1	1.5	2.3	3.4	4.4	1.0	4.2
DOM3Z	9.9	3.9	5.4	0.7	1.6	2.6	5.9	0.8	5.1
STXBP1	9.7	3.2	4.2	0.7	1.3	2.3	6.5	1.1	6.8
USP9X	10.2	3.1	4.2	0.4	1.4	1.7	7.8	1.0	6.1
FLJ23867	6.2	2.6	3.4	0.7	1.7	2.1	9.5	1.5	7.9
GPANK1	8.0	3.0	3.6	0.9	1.7	2.6	7.6	1.4	7.0
SLX1A-SULT1A3	11.3	4.3	6.5	1.2	1.6	2.4	4.5	0.7	3.2
RDH14	9.4	4.2	4.4	0.8	1.4	1.7	7.8	1.1	4.9
BFAR	9.4	3.8	5.3	0.4	1.6	2.1	6.2	0.8	6.3
DHRS7B	9.2	3.9	4.8	0.5	1.8	2.3	7.9	0.6	4.6
MPHOSPH8	11.2	3.1	5.0	0.6	1.3	1.8	7.5	0.8	4.4
TNKS2	9.8	3.5	5.0	0.5	1.4	1.6	7.6	0.9	5.6
ATP6V0E2	9.1	3.4	4.9	1.2	1.9	2.6	6.9	1.0	4.7
EXOC3	9.6	3.2	4.3	1.1	1.9	3.1	6.5	1.0	5.0
ZNF131	7.7	3.0	4.0	0.3	1.4	2.3	8.7	1.1	7.2
HDAC11	10.5	5.4	6.6	0.8	2.1	3.1	3.7	0.5	2.8
CHST7	8.8	3.6	5.3	0.9	1.2	1.8	7.1	1.2	5.7
ANKRD13A	9.3	3.2	4.0	0.6	1.5	2.4	8.0	1.3	5.4
MAN2C1	8.8	3.4	4.7	1.4	2.6	3.1	6.4	0.9	4.3

AP5M1	11.0	4.4	5.4	0.2	1.4	1.9	6.0	0.6	4.8
LOC100505794	11.4	2.4	5.6	0.7	1.3	1.7	8.3	0.8	3.5
EEFSEC	11.4	4.0	6.1	1.6	2.3	3.5	3.5	0.7	2.6
SMAD5	8.9	3.6	4.7	0.5	1.5	2.3	7.2	0.9	6.0
PSMG1	7.8	3.0	4.1	1.1	2.0	2.9	8.1	1.0	5.6
RCBTB1	10.6	3.1	5.2	0.7	1.8	1.6	6.6	0.8	5.3
GINM1	10.7	3.9	5.8	0.8	1.4	2.6	5.4	0.7	4.4
ACACA	10.9	3.3	4.6	0.4	1.3	1.5	7.3	0.8	5.5
ZNF526	8.1	2.8	4.0	1.0	2.3	2.5	7.5	1.2	6.1
AOX1	7.3	2.7	3.5	0.5	2.0	2.8	8.9	1.1	6.9
CUL1	9.7	3.3	4.2	0.4	1.3	1.9	7.6	0.9	6.3
ANGEL2	10.8	3.1	5.0	0.6	1.7	1.9	7.7	0.7	4.0
LOC101927014	11.4	5.4	6.8	0.5	1.5	2.0	4.6	0.7	2.5
KIAA1279	11.1	3.8	5.3	0.8	1.3	1.7	6.3	0.9	4.3
USE1	9.7	3.3	4.8	0.8	1.8	2.7	6.8	1.1	4.4
GATAD1	10.6	3.2	5.0	0.6	1.4	1.6	7.4	0.8	4.7
RP9	8.7	3.1	4.3	1.4	1.3	2.2	6.0	1.8	6.6
TOR1A	7.9	2.9	4.4	0.8	2.0	2.3	7.7	1.3	6.2
RBMS3	9.1	3.4	4.7	1.2	1.7	4.0	4.2	1.2	6.0
NUP160	9.8	3.4	4.6	0.3	1.3	1.6	8.1	0.9	5.6
HEIH	8.3	3.5	4.2	1.3	2.3	2.3	6.6	1.2	5.6
IGSF8	11.9	3.8	5.5	1.0	1.7	2.4	5.0	0.5	3.6
NADSYN1	10.3	3.7	5.1	1.3	2.1	3.3	4.8	0.9	4.0
HLTF	11.1	4.2	5.0	0.2	1.4	2.0	5.9	0.6	4.9
ZNF251	8.1	4.2	5.1	0.8	1.9	2.2	7.3	1.0	4.7
MOCS3	7.6	3.4	4.5	1.1	2.0	2.8	7.8	1.1	5.1
PRMT6	9.2	3.1	4.9	0.7	2.1	2.7	7.4	0.7	4.4
LOC101927126	12.6	2.0	4.5	0.6	1.4	1.6	8.3	0.7	3.5
SNHG9	10.6	5.7	4.8	0.8	2.0	2.1	2.8	0.9	5.6
STK32C	9.4	3.5	4.8	1.3	1.8	3.0	6.4	0.9	4.1
NOL10	8.9	3.4	4.6	0.4	1.5	1.9	7.8	1.0	5.9
DNAJC3	8.1	2.7	3.8	0.4	0.9	1.4	9.7	1.1	7.1
FAM96A	9.3	3.8	5.1	0.4	1.7	2.0	7.8	1.0	4.3
LINC01000	10.1	3.0	5.0	0.9	1.9	2.1	6.8	0.9	4.6
RBM27	8.4	3.2	4.1	0.6	1.7	2.1	7.3	1.3	6.6
URM1	8.1	3.8	4.7	1.0	1.8	2.8	6.7	1.1	5.1
DUSP23	10.7	4.0	6.4	1.4	2.8	2.8	3.9	0.7	2.6
ZNHIT2	7.5	3.6	4.0	0.9	1.9	3.0	8.2	0.9	5.3
TCEA3	8.0	3.6	4.9	0.4	1.6	1.6	6.5	1.8	6.8
SLMAP	8.1	3.3	3.8	0.4	1.2	1.9	7.9	0.9	7.7
RPP30	10.2	2.9	5.4	0.7	1.6	1.5	6.6	1.1	5.4
TMEM201	8.9	2.7	3.7	1.3	1.6	2.6	6.9	1.5	6.0
INPP5E	9.8	4.1	5.4	1.0	1.4	2.4	5.6	1.2	4.3
TUFT1	9.2	4.3	4.7	0.4	1.1	1.9	7.0	1.0	5.7
TPGS2	9.8	2.9	5.2	0.9	3.0	3.1	5.3	0.6	4.6
IKBKG	11.7	4.2	5.0	0.9	1.7	1.6	5.6	0.7	3.8
LOC101928565	12.5	2.3	4.6	0.4	2.0	1.8	6.6	0.6	4.4
DDX18	7.8	2.8	4.3	0.2	1.2	2.1	9.0	0.8	7.1
SERTAD3	10.4	4.3	5.5	1.2	2.0	1.8	4.7	1.2	4.1
RHPN1-AS1	11.4	2.6	5.6	0.8	1.4	1.4	8.3	0.6	3.2
CASP8	11.4	2.8	5.6	0.8	1.4	1.7	7.4	0.5	3.5
APIP	10.0	3.2	6.2	0.2	0.9	1.8	6.3	1.0	5.7
ARSJ	7.0	2.6	3.7	0.4	1.8	2.3	7.5	1.6	8.4
SCYL2	10.3	3.6	4.6	0.3	1.1	1.4	6.7	0.9	6.3
CCNO	6.8	2.6	3.9	0.8	1.8	1.8	10.4	1.4	5.7

RNF103	10.8	4.3	5.9	0.2	1.1	1.9	5.7	0.4	4.8
LZTS3	7.7	2.6	3.5	1.6	2.8	3.8	7.2	1.0	4.8
TGFBR1	10.3	3.6	5.2	0.5	1.7	2.2	6.2	0.7	4.7
FAM114A1	11.4	3.1	5.1	0.7	1.5	2.0	6.9	0.7	3.7
WDR81	9.9	4.0	5.6	1.0	1.9	2.1	5.2	0.9	4.6
FUNDC2	9.4	3.7	4.9	0.6	0.9	1.5	7.5	1.2	5.5
FBXL14	8.8	3.0	3.9	0.9	1.5	2.1	7.6	1.3	5.9
SMTN	9.6	3.8	4.7	1.2	2.3	3.0	5.3	1.1	4.1
EPB41L1	11.0	4.4	5.4	1.0	2.1	2.5	4.2	0.9	3.5
SLC7A2	10.3	3.8	5.6	0.4	1.3	1.7	6.3	0.5	5.1
KPNA1	10.1	3.6	4.5	0.5	1.3	1.9	6.9	0.9	5.3
SLC12A9	9.5	3.6	5.1	1.2	2.4	3.0	5.2	0.9	4.1
NCDN	9.0	3.4	5.0	1.9	2.3	3.8	5.3	0.9	3.5
CRELD1	9.3	4.2	5.9	0.6	1.5	2.3	5.5	0.9	4.7
MAPK13	11.0	2.9	4.9	0.7	1.4	1.6	8.0	0.7	3.8
GPKOW	9.7	3.9	4.5	0.6	1.7	2.4	6.4	1.0	4.8
COA5	9.4	3.3	4.2	1.1	3.0	2.8	4.8	0.8	5.6
PSEN2	9.5	3.5	5.0	0.9	1.9	2.6	6.5	0.7	4.4
ENOPH1	8.1	3.6	4.2	0.4	1.4	2.3	8.6	1.0	5.3
ADRA1A	11.0	2.1	5.0	0.9	1.2	1.2	9.1	0.7	3.7
LINC01010	11.5	2.0	4.6	0.7	1.2	1.7	9.1	0.6	3.5
BAHD1	9.8	3.8	4.5	1.0	2.0	2.5	6.0	1.0	4.4
TMEM44	9.6	3.6	5.4	1.0	1.7	2.2	6.5	0.8	4.0
ZBTB40	11.5	3.8	5.5	0.7	1.5	2.0	4.9	0.9	4.1
TMEM41A	8.9	3.2	4.3	0.6	0.8	1.9	8.6	1.1	5.5
CRELD2	8.8	3.0	4.8	1.0	1.9	2.3	7.6	0.9	4.6
MED27	8.2	3.1	4.8	0.6	1.6	2.4	7.2	0.9	6.1
PRR16	9.3	3.5	3.5	0.4	1.7	2.0	7.3	1.4	5.6
FBXW4	11.6	4.4	5.9	1.2	2.0	2.7	3.6	0.6	2.7
NT5DC3	8.8	2.9	3.5	0.6	2.4	2.0	7.7	1.3	5.6
NAGK	8.8	4.0	5.7	0.8	1.7	2.6	5.8	0.9	4.5
LOC101929037	9.6	2.6	5.7	0.4	1.6	1.3	8.6	0.8	4.3
ZNF516	9.2	3.2	4.4	1.2	1.7	2.4	7.0	0.9	4.8
ZDHHC1	13.3	4.8	6.4	1.3	1.9	3.1	2.1	0.3	1.5
RAD21-AS1	11.3	3.7	4.5	0.8	1.5	1.7	5.9	1.2	4.2
COG1	8.3	3.2	4.2	0.7	1.9	2.4	7.2	1.1	5.6
DSE	9.7	3.4	4.9	0.4	1.3	1.7	7.2	0.9	5.2
LRRC58	7.9	2.2	3.5	0.5	1.0	1.7	11.2	1.1	5.7
CRY1	7.6	2.8	3.8	0.3	1.3	1.7	9.7	1.1	6.4
CDKN2C	11.7	5.1	7.0	0.4	2.1	2.2	3.4	0.5	2.3
UTP23	7.7	2.8	4.1	0.7	1.6	1.6	7.2	1.5	7.4
SEMA3A	8.1	3.3	4.2	0.6	1.8	2.6	6.9	1.1	5.9
DNAJB14	8.8	3.7	5.0	0.4	1.3	1.7	7.2	0.6	6.1
DGCR8	8.1	2.9	3.9	0.7	2.0	2.5	8.2	1.0	5.3
BRPF1	9.0	3.0	4.4	0.9	1.5	2.2	6.4	1.4	5.7
QSER1	9.3	3.2	4.1	0.3	1.4	2.1	6.2	1.0	7.0
DDX19B	9.3	3.0	4.9	0.5	1.8	2.3	6.2	1.0	5.6
PDCD4-AS1	10.4	2.4	4.4	0.6	1.6	1.6	9.2	0.6	3.7
AIMP1	8.6	4.4	4.3	0.3	1.7	1.8	7.6	0.5	5.5
SSBP3	11.7	5.1	5.0	0.9	2.1	3.0	2.9	0.9	3.0
FAM204A	8.5	2.9	4.9	0.3	1.3	1.7	8.9	0.9	5.2
NAF1	8.7	2.5	3.6	0.6	1.3	1.7	8.8	0.9	6.4
SCN5A	9.3	3.1	4.3	1.3	2.3	3.3	5.3	1.0	4.5
PLEKHA1	9.9	3.4	5.3	0.4	1.3	1.2	6.8	0.9	5.3
GCDH	7.2	3.0	3.7	1.4	2.6	3.3	7.3	1.0	5.1

LOC101926913	11.8	2.2	4.8	0.6	1.5	1.5	7.8	0.6	3.6
PRMT7	8.6	3.0	4.1	1.2	1.9	2.6	7.0	1.0	5.2
UCHL5	8.5	3.2	4.0	0.8	1.8	2.0	7.4	1.0	5.9
MIER1	8.3	3.6	5.1	0.3	1.3	1.9	6.7	1.4	5.8
POMGNT1	9.3	4.3	5.2	1.2	2.3	3.4	4.6	0.8	3.4
GNG10	8.5	3.8	6.2	0.3	1.1	2.4	6.1	1.6	4.5
GOPC	8.1	3.1	4.7	0.4	1.6	1.8	7.3	1.1	6.3
TRAF3	8.5	3.4	4.7	0.9	1.6	2.4	6.6	1.0	5.2
RFWD3	8.6	3.6	4.2	0.6	1.7	2.0	6.3	1.1	6.3
PCGF1	9.0	4.1	5.9	0.5	1.6	2.2	5.0	1.1	5.1
FAM98A	8.0	3.3	4.0	0.6	1.5	2.0	6.8	1.2	6.8
ZG16B	10.2	4.4	5.7	0.8	2.1	2.0	4.5	0.5	4.2
TENC1	9.7	3.9	5.2	1.4	2.3	3.0	4.4	1.0	3.6
NDNL2	8.5	3.3	4.1	0.6	2.0	2.0	7.4	1.2	5.4
NSD1	10.0	3.0	4.2	0.4	1.3	1.8	7.4	0.9	5.3
SP100	10.0	3.4	4.4	0.5	1.8	2.0	5.9	0.9	5.3
SIRT2	9.1	3.9	4.8	0.6	1.8	2.5	6.0	1.2	4.4
HERC2	9.7	2.6	4.4	0.9	2.0	2.4	6.8	0.8	4.6
CCDC90B	8.6	5.0	5.7	0.6	1.5	3.0	5.1	0.6	4.1
TNC	9.9	3.6	4.5	0.3	0.5	1.0	7.7	1.2	5.6
ZDHHC17	11.0	2.9	4.6	0.5	1.1	1.6	7.1	0.7	5.0
SLK	10.0	3.3	4.6	0.3	1.3	1.7	6.7	0.8	5.6
DDR1	11.5	4.2	6.5	0.6	1.3	2.3	4.4	0.5	3.1
SIX5	10.9	4.5	6.0	1.7	2.1	2.5	3.3	0.7	2.6
LOC100128233	11.4	2.9	4.6	0.5	1.1	1.6	8.0	0.6	3.4
VMA21	7.0	3.5	4.3	0.6	1.8	3.1	6.1	1.2	6.6
C4orf3	9.9	4.3	5.5	0.9	1.1	1.4	6.1	0.7	4.4
ZC3HAV1L	6.3	3.4	4.5	1.1	2.2	2.6	7.2	1.0	5.9
OTUD7B	9.9	3.4	4.3	0.5	1.2	1.9	6.4	1.1	5.5
SPIN1	8.4	3.0	4.2	0.5	1.2	1.3	7.7	0.9	7.0
SCARNA20	11.0	5.2	4.5	1.1	1.4	1.1	6.4	0.8	2.8
DCAKD	10.0	4.0	5.6	1.5	2.4	3.3	3.8	0.6	3.0
AP1AR	8.8	2.5	3.7	0.4	1.0	1.9	8.2	0.9	6.8
ADARB1	8.3	2.5	4.0	1.0	1.7	2.3	6.9	1.2	6.2
TRIM33	9.9	2.8	4.1	0.5	1.6	1.8	6.6	1.1	5.7
AK1	10.2	3.5	5.6	0.9	1.2	2.3	4.9	1.0	4.5
STK35	9.2	3.7	3.6	0.7	1.4	1.7	7.4	1.0	5.7
LOC101927512	11.2	2.4	4.9	0.4	1.5	1.5	8.2	0.5	3.7
ACAP2	9.0	3.4	4.1	0.3	1.3	1.4	7.8	1.2	5.7
LOC101927528	11.0	2.4	5.0	0.6	1.3	1.3	8.2	0.6	3.7
ARL3	8.7	3.0	4.8	0.5	1.3	1.9	6.6	1.0	6.2
ARHGEF7	10.8	3.7	4.9	0.6	1.0	1.6	5.6	1.1	4.8
MITD1	9.4	3.8	5.3	0.5	1.4	2.4	5.1	1.1	5.2
BRWD3	8.6	2.9	4.0	0.3	1.0	1.3	8.5	1.0	6.6
BLMH	9.2	3.4	4.7	0.5	1.9	1.9	6.9	0.8	4.9
KPNA3	8.3	3.3	4.5	0.5	1.3	2.2	6.2	1.0	6.7
BTBD10	8.0	3.4	4.4	0.3	1.0	1.6	8.2	1.1	6.1
SAP130	10.1	3.3	4.7	1.1	1.8	2.4	4.5	1.3	4.8
LOC101929035	11.6	1.5	4.6	0.8	1.1	1.4	9.3	0.5	3.4
ANKHD1	10.9	1.8	6.2	0.4	0.3	1.2	6.5	0.9	5.8
SH3GLB2	8.4	4.3	4.9	1.6	2.1	4.6	3.9	0.8	3.5
ZMPSTE24	8.4	3.2	4.7	0.3	1.1	1.6	7.5	1.0	6.3
YJEFN3	10.8	3.7	5.0	1.0	2.0	1.7	5.6	0.9	3.4
CDKN2AIPNL	7.9	2.8	5.7	0.8	2.3	3.0	5.4	0.7	5.6
CCNDBP1	9.7	3.7	6.5	0.5	1.9	2.0	5.2	0.5	3.9

ZNF106	8.6	3.3	4.0	0.4	1.3	1.8	7.9	1.0	5.7
LOC100505989	11.4	2.4	4.7	0.6	1.3	1.4	7.8	0.7	3.7
TST	9.5	3.6	5.3	1.1	1.9	2.9	5.8	0.4	3.6
GFM1	8.3	3.3	4.4	0.4	1.5	2.0	7.3	1.0	5.7
RNF38	9.2	3.6	5.0	0.8	1.6	2.9	4.5	1.2	5.3
ATG4B	10.5	3.5	4.7	0.6	1.4	1.7	6.5	0.9	4.3
TVP23C	9.4	2.9	4.2	0.3	1.5	1.4	8.4	1.0	4.8
TNPO3	9.2	3.3	3.9	0.6	1.5	1.9	6.5	1.2	6.0
TMEM181	8.9	2.8	4.3	0.7	2.2	2.0	7.2	0.8	5.1
SNUPN	7.8	3.4	4.1	0.7	1.3	1.8	7.1	1.2	6.6
WDFY1	8.4	3.3	4.3	1.1	2.8	2.7	5.3	1.0	5.0
KTN1-AS1	7.9	2.3	4.0	1.3	2.2	2.4	8.7	0.7	4.3
TBC1D25	8.0	3.2	4.4	1.2	1.8	2.5	7.3	1.0	4.6
PGS1	9.5	3.7	4.2	0.7	1.8	2.2	6.5	0.8	4.6
TANK	9.7	4.8	6.1	0.3	1.2	1.9	4.6	0.7	4.5
KLHL2	8.6	2.9	4.0	0.2	1.3	1.7	8.3	1.1	5.9
ROBO3	7.7	3.5	4.6	1.4	2.2	3.1	5.3	1.1	4.8
ATF2	8.5	4.0	5.5	0.5	1.2	1.3	6.3	1.1	5.6
BIRC2	8.5	3.4	5.5	0.3	1.0	1.3	7.4	0.8	5.8
CCDC104	9.3	3.5	4.7	0.2	1.6	1.9	6.1	0.6	5.9
KDM4A	8.9	3.1	3.9	0.5	1.5	2.0	7.5	0.9	5.4
TLDC1	7.9	3.4	4.0	0.8	2.3	3.3	6.1	1.1	5.0
CUEDC1	10.8	4.0	5.4	1.0	1.7	2.4	4.4	0.7	3.5
CACNA2D4	10.5	4.7	5.8	1.0	2.0	2.6	3.6	0.6	3.1
LMCD1	8.1	3.8	5.3	0.4	1.3	1.3	6.5	0.8	6.3
ZNF148	9.0	3.0	3.9	0.4	1.3	1.8	7.5	0.9	6.1
PPP6C	9.7	3.2	4.3	0.2	1.1	1.8	6.6	0.8	6.1
CTC1	9.0	3.4	4.8	0.8	1.9	2.6	6.0	0.9	4.6
LINC00864	10.9	2.0	4.9	0.4	1.3	1.5	8.5	0.4	3.9
FAM129A	8.9	3.1	3.8	0.7	2.0	2.6	6.8	0.9	5.0
H6PD	11.6	4.1	6.0	1.7	2.3	3.5	2.3	0.4	1.9
FAM173A	9.1	3.4	5.0	1.2	2.7	3.0	6.2	0.7	2.5
NCKAP5L	7.5	3.1	4.2	1.1	1.8	2.5	6.5	1.6	5.6
CBLB	11.5	3.5	5.4	0.3	0.9	1.1	5.4	1.0	4.7
C8orf59	9.1	3.3	7.4	0.2	1.4	2.0	5.3	0.7	4.4
SLIT2	10.1	3.3	4.4	0.6	1.6	1.8	6.5	0.9	4.5
ALDH6A1	8.0	3.4	3.5	0.6	2.2	3.6	6.4	0.9	5.1
LOC101928255	11.0	2.3	4.8	0.7	1.3	1.4	8.6	0.6	3.1
RNF20	9.0	2.9	4.0	0.5	1.5	1.9	7.1	1.1	5.9
IDI1	9.0	4.8	6.1	0.1	0.6	1.1	6.7	0.5	4.9
CTDP1	10.3	3.5	4.9	0.9	1.8	2.3	5.4	0.9	3.8
KPNA6	9.6	3.3	4.4	0.7	1.3	2.0	6.3	1.0	5.2
FBXO34	8.5	3.0	4.1	0.3	1.0	1.5	8.0	1.0	6.3
TAF11	8.4	3.5	4.2	0.5	1.5	1.7	7.6	0.9	5.3
MED13	9.7	3.0	4.2	0.3	1.3	1.8	6.8	1.0	5.7
ZHX3	8.8	3.3	4.1	1.2	2.0	2.8	5.4	1.1	5.0
REST	8.6	3.1	4.0	0.4	1.2	1.8	7.6	1.0	6.1
UBE4B	10.5	3.6	4.3	0.7	1.6	2.4	5.4	0.8	4.6
KIDINS220	9.4	3.2	4.3	0.5	1.4	2.2	6.4	1.1	5.2
MID1	8.8	3.0	4.0	0.9	1.8	2.1	6.3	1.1	5.7
HTATSF1	8.9	3.5	4.1	0.3	1.3	1.6	7.4	1.1	5.6
RC3H2	9.0	2.9	3.8	0.3	1.4	2.0	7.0	1.1	6.3
MIF4GD	8.5	3.9	4.3	0.5	1.6	1.9	7.0	1.0	4.9
KCNQ1OT1	11.4	2.2	5.1	0.4	1.5	1.7	6.7	0.6	4.1
CD99	9.0	4.0	5.2	0.6	1.8	2.7	4.5	1.2	4.6

CD99	9.0	4.0	5.2	0.6	1.8	2.7	4.5	1.2	4.6
CACNB1	8.7	3.6	4.6	0.6	1.0	1.7	5.9	1.2	6.4
TRAPPC9	10.6	3.5	4.5	0.9	1.7	2.6	5.4	0.7	3.6
CNNM3	9.4	3.6	4.9	1.0	1.3	2.0	5.9	0.8	4.6
LOC101928151	9.9	2.2	5.0	0.5	1.4	1.8	8.6	0.7	3.5
LHFP	8.3	3.6	3.6	1.2	2.1	2.5	6.7	0.9	4.7
SGK196	9.5	3.0	4.0	0.4	1.2	2.0	7.5	1.0	5.1
ORMDL1	8.8	3.6	5.2	0.1	1.4	1.8	5.7	1.0	6.0
OGFOD1	6.5	2.7	3.1	0.4	1.1	1.7	9.4	1.2	7.4
MSL2	7.8	2.5	3.9	0.4	1.0	1.5	8.2	1.3	7.1
SLC12A7	11.2	4.2	5.7	0.9	1.6	2.3	4.1	0.7	2.9
SNORA5C	9.8	2.6	5.5	0.2	1.9	0.9	4.2	1.1	7.3
NGLY1	9.9	2.4	4.3	0.6	1.6	1.9	7.9	0.7	4.1
LOC400958	11.1	2.2	4.8	0.6	1.2	1.3	8.6	0.5	3.4
LINC00476	10.7	2.9	4.7	0.7	1.7	1.8	6.9	0.9	3.2
CDC20	6.3	2.4	2.8	1.0	2.3	2.4	7.8	1.7	6.8
TAZ	10.6	4.0	6.0	0.9	1.5	2.4	4.2	0.7	3.1
LOC101928101	10.9	2.4	4.7	0.5	1.1	1.2	8.6	0.5	3.5
ATRX	9.2	2.8	4.1	0.3	1.2	1.5	7.4	0.8	6.0
BCL10	9.5	4.3	4.4	0.5	1.8	1.9	5.5	0.6	4.8
CBFA2T2	9.8	2.8	4.7	0.8	1.4	2.0	6.0	1.5	4.6
DBNDD2	7.9	3.4	4.8	1.3	1.4	2.1	5.0	1.9	5.7
CACTIN	9.0	3.5	4.4	1.3	1.9	2.1	5.4	1.2	4.7
C2orf47	8.8	4.0	6.0	0.7	1.6	1.8	5.0	0.9	4.6
SMC4	8.7	3.6	4.2	0.4	1.6	2.0	6.8	0.8	5.4
PPIL3	10.0	3.2	5.5	0.5	0.7	1.5	6.6	0.9	4.6
PSMG2	11.1	3.6	4.7	0.2	1.3	1.5	6.0	0.4	4.6
TRAFD1	11.1	4.6	6.6	0.5	1.4	1.6	3.8	0.6	3.2
ERC1	8.9	2.9	3.9	0.4	1.5	2.1	6.9	0.9	5.9
ZNF330	8.2	3.2	4.0	0.3	1.1	1.9	7.8	0.7	6.2
MOCOS	8.3	3.0	4.3	0.6	1.6	2.1	7.5	0.9	5.1
TMEM57	10.5	3.1	3.3	0.4	1.5	1.6	5.7	0.9	6.4
CAMTA1	6.8	2.6	3.2	0.7	1.8	1.7	10.3	0.8	5.5
ABHD16A	8.4	3.3	4.2	0.7	1.9	2.4	6.7	1.0	4.6
DCAF6	10.5	4.0	4.8	0.4	1.3	1.5	5.2	0.9	4.7
GCC1	7.9	2.7	3.4	0.5	1.0	1.5	8.7	1.2	6.5
CLCN6	9.9	3.9	5.2	0.5	1.1	1.5	5.9	0.8	4.6
TBC1D4	7.8	2.6	3.6	0.6	1.2	1.7	8.5	1.4	6.0
GPR107	9.9	4.2	4.9	0.4	1.2	2.2	5.1	0.9	4.6
ZKSCAN8	8.5	3.1	4.3	0.4	1.2	1.7	7.0	1.2	6.0
CD276	9.7	4.4	5.3	1.2	1.7	2.0	4.6	0.8	3.6
CSTF3	10.4	3.8	5.7	0.2	1.0	1.9	5.5	0.6	4.3
BCAS4	9.3	3.8	4.8	1.1	1.4	2.1	6.1	0.7	4.0
MRPL44	9.9	3.5	5.4	0.3	0.9	1.4	6.9	0.8	4.3
ZNF217	9.5	3.2	4.3	0.5	1.6	2.0	6.3	1.1	5.0
MFAP3	8.8	3.2	4.5	0.3	1.5	1.8	7.5	0.8	4.8
TSPAN5	8.7	3.0	4.3	0.5	1.3	1.8	7.7	1.0	5.1
SDC2	9.1	3.9	5.5	0.6	1.4	2.0	6.1	0.6	4.1
ENKD1	8.7	3.0	5.2	1.0	1.6	2.5	5.9	1.1	4.2
PMF1	9.0	3.3	5.3	1.1	1.5	3.1	5.1	0.7	4.2
DFNA5	9.4	3.2	3.9	0.8	1.8	2.1	6.8	0.6	4.8
CLIP4	10.3	3.2	4.8	0.3	1.1	1.4	7.1	0.8	4.3
NUPL1	9.0	3.7	4.3	0.4	1.2	1.6	5.6	0.9	6.5
RBBP7	6.9	3.3	4.1	0.5	1.6	2.5	7.6	1.0	5.6
LONP2	9.6	3.5	4.1	0.9	1.6	2.5	5.4	0.9	4.6

TLK1	10.0	3.3	4.7	0.3	1.6	1.7	5.6	0.7	5.3
SHOC2	7.6	3.1	4.6	0.3	1.6	1.9	7.3	1.2	5.7
CHKB-CPT1B	11.6	4.9	7.1	0.6	1.2	1.6	3.1	0.5	2.5
RBM26	8.7	3.1	4.6	0.5	1.4	1.8	6.3	1.1	5.7
SARS2	9.7	4.3	4.8	1.4	2.2	3.2	3.3	0.9	3.5
FEZ2	8.2	3.2	4.7	0.7	1.6	2.0	7.2	0.9	4.7
BTBD7	9.8	3.0	3.6	0.5	1.2	1.7	6.8	0.7	5.8
LOC101928423	11.5	1.9	4.5	0.6	0.9	1.3	8.3	0.6	3.5
GSN	10.1	3.8	5.3	0.7	1.4	2.0	5.6	0.8	3.5
ECHDC1	9.2	3.4	5.0	0.1	1.1	2.2	5.9	0.8	5.4
WDR35	8.6	3.0	4.6	0.4	1.5	2.0	6.8	1.0	5.2
MAPK8	7.5	2.3	3.1	0.2	1.1	1.6	9.2	1.0	7.0
LTV1	10.2	3.0	4.6	0.3	1.4	1.7	6.4	0.9	4.7
PPAPDC1B	7.9	2.9	3.2	0.5	0.9	1.7	9.4	0.6	6.1
LINC00452	8.5	2.2	3.5	0.9	1.5	1.7	8.5	1.4	4.9
ATXN1L	8.5	3.4	4.7	0.8	1.6	2.3	5.5	1.0	5.2
ERLIN1	7.8	3.5	4.4	0.5	1.8	2.4	6.7	0.7	5.3
RNF25	8.9	3.7	4.7	0.8	1.8	2.1	5.5	1.2	4.6
GALNS	8.9	3.9	4.8	1.1	2.3	2.9	4.8	0.7	3.8
ATP6V1H	10.1	3.5	4.4	0.5	1.1	1.4	7.0	0.7	4.4
HCG18	7.2	2.6	4.4	0.7	1.1	2.2	7.8	0.9	6.3
LOC101927924	10.5	2.2	4.5	0.6	1.3	1.6	8.4	0.4	3.6
SCML1	9.0	3.1	5.1	0.4	1.8	2.1	5.6	1.2	4.8
NRG1	8.3	2.5	3.5	0.9	2.1	2.6	6.1	1.2	6.0
NUDCD1	6.4	2.3	3.5	0.3	1.1	1.6	9.3	1.1	7.5
ANAPC1	8.7	2.8	4.1	0.3	1.4	2.0	7.1	1.0	5.7
CDK17	9.2	3.5	4.3	0.5	1.3	1.8	5.8	1.0	5.6
ECD	8.6	2.8	3.8	0.5	1.7	2.3	6.7	1.3	5.2
CREBL2	8.0	3.6	4.4	1.0	2.5	1.8	7.2	0.8	3.8
DCUN1D1	8.3	3.6	5.1	0.4	1.9	1.3	6.0	0.6	5.9
SKA2	8.0	3.5	4.3	0.4	1.2	1.8	6.6	1.0	6.2
WDR73	8.5	2.7	3.9	0.8	1.4	2.2	7.8	0.7	5.0
CERCAM	9.6	3.4	5.2	1.5	2.7	3.6	3.6	0.7	2.8
ZC3H12A	12.4	5.4	7.7	0.6	0.9	1.6	2.1	0.4	1.8
EHBP1	8.3	2.8	3.1	0.3	1.2	2.0	8.6	0.9	5.7
ULK1	9.5	4.3	5.3	1.6	2.0	3.0	3.0	0.9	3.2
LSM6	8.1	2.9	5.6	0.5	2.1	2.1	4.8	0.9	6.0
SMPD1	10.8	4.7	6.4	1.1	2.1	3.1	2.7	0.4	1.6
SLC25A26	8.9	3.4	4.9	0.3	1.1	1.6	7.3	0.5	4.8
IGFBP3	7.7	3.4	3.5	1.3	2.3	3.0	6.9	1.4	3.4
MED26	8.0	3.5	4.1	1.2	1.5	2.5	5.2	1.4	5.3
LINC00514	9.9	2.2	4.5	0.6	1.3	1.7	8.5	0.6	3.6
PREP	7.3	3.2	4.0	0.7	1.2	2.1	6.9	1.2	6.3
STAU2-AS1	10.2	2.1	4.4	0.6	1.3	1.4	8.6	0.7	3.6
WDR12	8.6	2.7	3.3	0.3	1.2	1.9	7.5	1.0	6.4
C7orf73	10.5	4.0	3.8	0.0	1.6	3.9	4.1	0.6	4.4
FBXL17	8.5	4.2	4.4	2.1	1.5	1.9	5.0	1.4	3.8
TPGS1	10.0	3.6	6.4	1.5	1.7	2.0	4.6	0.8	2.4
FAM175B	9.3	3.4	4.6	0.5	1.1	1.7	6.2	1.1	4.8
BCS1L	8.9	2.7	4.7	0.7	1.7	2.4	6.9	0.7	4.2
SAMM50	9.4	3.8	4.7	0.6	2.1	1.8	5.9	0.8	3.7
CAMK2D	10.6	3.6	5.6	0.6	1.3	2.0	5.1	0.7	3.3
KIF13A	10.0	2.3	4.3	1.0	1.3	2.0	6.7	0.7	4.6
CEP170B	9.0	3.4	4.3	1.4	1.9	2.6	5.4	1.0	3.8
PHF20	10.1	3.2	4.3	0.6	1.4	2.0	6.2	0.7	4.4

GOLGA5	9.1	3.4	4.2	0.3	1.2	1.9	6.3	0.8	5.6
COX14	8.1	3.6	5.1	0.8	1.1	1.8	7.3	1.1	3.9
ASB13	8.5	4.0	5.1	0.9	1.7	2.0	5.6	0.8	4.1
SUCO	8.5	3.0	3.9	0.4	1.4	1.8	7.4	0.9	5.5
DOCK4	8.6	2.7	3.8	0.5	1.3	1.8	7.1	1.0	5.7
SFXN4	8.3	3.3	4.4	0.7	1.7	2.1	6.3	0.9	5.2
BAG4	7.8	3.3	3.9	0.7	1.3	2.5	5.9	1.3	6.1
ZBTB2	6.3	2.1	2.8	0.7	1.4	1.8	8.0	1.9	7.7
CEBPZ	7.7	3.2	3.9	0.2	1.0	1.5	8.1	0.8	6.3
CSTF2T	7.8	3.1	3.8	0.6	1.5	2.3	6.9	1.1	5.5
GORASP1	9.8	3.9	4.6	0.5	1.2	2.2	5.6	0.7	4.1
CTNS	9.1	3.4	4.8	0.8	2.0	2.5	4.7	0.7	4.8
CCDC115	8.7	3.6	4.5	1.1	1.7	2.5	5.6	0.9	4.0
PIGC	8.1	3.6	4.7	0.5	1.0	1.8	6.7	0.8	5.2
ACP2	7.7	2.8	3.8	1.4	2.2	3.6	6.4	0.8	3.7
DTNBP1	9.3	3.5	4.0	0.7	1.7	2.0	5.8	0.9	4.8
ANKFY1	9.8	3.0	4.5	0.6	1.4	2.0	6.0	1.0	4.4
MYO1B	8.5	3.3	3.6	0.3	1.2	1.7	6.9	1.1	6.0
ZDHHC24	9.3	3.5	5.1	1.7	3.3	3.9	3.3	0.7	1.9
ARHGAP32	9.4	3.0	4.1	0.6	1.4	2.1	5.8	0.9	5.0
MKLN1	9.1	3.1	4.0	0.4	1.3	1.5	6.6	0.9	5.5
MYLIP	11.9	4.5	5.6	1.0	1.4	2.0	2.8	0.5	2.6
SOGA1	9.1	2.7	4.5	1.0	1.6	1.8	6.5	1.0	4.4
GGA2	8.6	3.3	4.1	0.6	1.4	1.8	6.3	0.8	5.6
ZNF506	10.6	2.4	4.7	0.6	1.3	1.8	7.0	0.6	3.5
MTFR1	7.3	2.4	3.6	0.2	0.9	1.7	9.4	1.1	5.8
SPHK2	7.7	3.5	4.0	1.5	2.3	3.0	5.6	1.2	3.8
RNF103-CHMP3	6.9	3.2	6.5	0.3	1.2	1.6	5.9	0.6	6.3
COL12A1	8.2	2.4	3.4	0.5	1.7	2.1	7.7	1.0	5.4
TWF1	8.4	3.4	4.9	0.2	0.9	1.7	6.9	0.8	5.3
PET117	8.1	4.5	4.1	1.1	2.2	3.2	4.5	1.5	3.3
LOC101928099	11.2	2.4	4.5	0.5	1.1	1.5	7.4	0.5	3.2
EMG1	5.7	2.6	4.0	0.7	1.4	2.5	8.1	1.1	6.3
TCEAL3	7.4	2.8	3.6	1.2	2.1	2.9	5.8	1.1	5.4
RABEP1	8.3	3.1	3.9	0.5	1.4	1.9	6.4	1.0	5.9
LOC339803	10.2	2.2	4.4	0.5	1.2	1.7	8.2	0.6	3.4
TMEM14B	9.9	3.1	4.1	0.5	2.9	2.4	4.5	0.6	4.4
DROSHA	8.3	2.9	4.1	0.7	1.9	2.1	5.4	1.2	5.7
LOC100506083	9.8	2.4	4.3	0.5	1.1	1.4	8.6	0.6	3.6
LOC101929220	9.2	4.3	5.0	0.6	1.3	1.9	5.7	0.7	3.7
STARD3NL	7.6	3.3	5.1	0.4	1.3	2.1	5.5	1.1	6.0
LOC101928846	10.6	2.1	4.6	0.6	1.3	1.4	7.5	0.7	3.6
IFT20	9.6	3.4	4.5	0.4	0.9	1.8	6.5	0.8	4.6
CXCL2	11.9	6.2	8.2	0.5	0.9	1.6	1.4	0.0	1.6
SLC2A6	11.0	4.8	5.8	1.1	1.9	2.3	2.9	0.5	2.0
TSFM	9.0	2.7	4.0	0.5	1.4	2.3	7.2	0.8	4.5
LOC100507175	10.9	2.1	4.3	0.6	1.3	1.5	7.9	0.7	3.1
CCDC101	7.7	2.6	4.1	0.7	1.9	2.1	7.2	1.2	4.7
NME7	9.6	2.9	4.5	0.2	1.4	1.6	6.9	0.7	4.5
TMEM216	7.1	4.4	5.0	0.8	2.0	2.1	6.3	0.8	3.8
KLF15	3.6	1.5	1.7	1.4	2.6	4.2	8.8	1.6	7.0
ZNF264	10.4	2.2	4.5	0.5	1.0	1.3	7.8	0.6	3.9
NUMBL	8.6	3.1	4.9	1.4	1.6	2.4	5.4	1.1	3.8
SLC37A4	7.6	3.9	5.1	1.1	2.2	3.5	4.6	0.7	3.5
FAM131A	6.1	2.6	3.4	0.9	1.8	2.3	8.1	1.4	5.6

RASA1	9.2	3.5	4.7	0.4	1.2	1.7	6.1	0.8	4.7
GLRX2	7.9	2.4	4.0	0.7	1.1	1.6	8.6	1.1	4.8
IRF9	9.2	4.2	5.7	0.6	1.4	1.9	4.5	0.6	4.1
ING1	7.0	3.2	3.4	1.5	1.6	1.9	6.2	2.0	5.4
ABCA2	9.9	4.0	5.2	1.6	2.5	3.3	3.0	0.6	2.3
C17orf70	7.7	3.1	3.7	1.3	2.6	3.6	5.7	1.0	3.5
CEP170	8.0	2.9	3.7	0.3	1.2	1.9	6.9	1.0	6.3
CAPRIN2	9.0	3.3	5.0	0.4	1.4	2.0	5.4	0.8	4.8
NOMO2	8.0	3.3	3.9	0.7	1.4	2.0	6.4	1.2	5.4
ID1	11.1	5.6	6.6	0.7	0.8	1.6	3.1	0.6	2.0
CBX5	7.5	2.8	4.1	0.3	1.6	1.5	7.6	0.8	6.0
MAP3K1	8.7	3.1	4.0	0.5	1.6	1.6	7.0	1.0	4.8
LOC731424	9.8	2.2	4.8	0.5	1.1	1.4	8.2	0.6	3.5
SNORD12	5.4	2.7	2.6	2.3	2.4	3.8	5.6	1.9	5.5
CAND1	8.6	2.9	4.0	0.2	1.0	1.4	7.4	0.8	5.7
ZMAT3	9.1	2.8	4.0	0.4	1.7	1.9	5.9	1.1	5.1
C1orf35	8.3	3.0	3.8	1.0	1.5	1.9	6.3	1.1	5.3
DHRS4	8.1	3.5	4.7	1.2	1.9	3.1	5.4	0.7	3.5
IDUA	9.1	3.5	5.2	2.5	2.9	3.8	2.8	0.5	1.8
MMAB	8.9	3.2	4.3	1.4	1.5	2.3	5.7	0.7	4.0
LINC00485	10.8	2.1	4.9	0.5	1.4	1.8	6.3	0.6	3.7
LBR	9.3	3.5	4.2	0.2	1.4	2.0	6.5	0.6	4.3
RSF1	9.3	2.9	3.9	0.4	1.4	1.8	6.5	0.8	4.9
LOC101928336	10.4	2.5	4.8	0.9	1.6	1.8	6.1	0.6	3.3
LOC101927969	9.9	2.4	4.5	0.5	1.2	1.6	8.1	0.5	3.2
C1orf63	7.7	2.6	5.6	0.6	1.3	1.6	7.6	0.9	4.1
LOC101929295	10.1	2.5	4.6	0.6	1.2	1.5	7.6	0.6	3.3
ANKRD9	10.1	3.2	4.5	1.1	1.9	2.4	5.4	0.8	2.6
ZNF335	9.0	3.8	4.4	1.1	2.1	2.7	4.4	1.0	3.5
MORC4	10.4	3.8	5.2	0.4	1.4	1.9	4.5	0.6	3.8
SMURF1	9.0	3.3	4.0	0.6	1.4	2.0	5.5	1.0	5.2
NEK4	9.6	3.1	4.7	0.5	1.3	1.8	6.0	0.7	4.2
MCM3AP	9.4	3.0	4.4	0.7	1.2	2.0	6.4	0.8	4.2
LOC101927419	10.2	2.2	4.0	0.6	1.4	1.5	8.2	0.6	3.3
PTGER4	6.6	2.5	3.1	1.0	1.3	2.4	7.0	1.7	6.3
VAV2	8.5	3.0	4.2	0.9	1.6	2.3	5.7	0.9	4.8
FAHD1	8.3	2.9	4.7	1.1	1.8	2.4	6.8	0.6	3.4
TTC37	9.7	3.1	4.5	0.3	1.3	1.7	5.9	0.7	4.6
FAM193B	8.7	3.7	5.0	1.1	1.6	2.1	4.9	1.0	3.7
NSRP1	6.9	3.0	3.5	0.6	1.6	2.4	5.8	1.2	7.0
DHX36	8.5	3.0	4.1	0.4	1.0	1.8	7.2	0.7	5.2
HES1	8.7	3.4	5.0	1.2	1.9	2.9	4.4	0.5	3.9
KLHDC4	8.2	2.5	3.8	0.9	1.9	1.7	7.0	0.8	5.2
TBKBP1	8.4	3.6	4.4	1.1	1.5	1.9	5.8	0.9	4.2
ZNF544	8.4	2.3	4.1	0.3	1.0	1.2	8.4	0.7	5.5
TNKS	9.7	3.1	4.3	0.8	1.6	2.0	5.7	0.8	3.9
HRNR	8.9	1.6	4.1	0.9	1.7	1.8	8.3	0.7	3.8
TMEM38B	8.0	2.7	4.8	0.2	1.3	1.9	6.9	0.7	5.4
FABP5	6.3	2.6	4.4	0.5	2.1	2.5	7.2	0.8	5.3
CABIN1	10.1	3.7	4.8	0.7	1.3	2.0	5.0	0.8	3.5
UBE2E2	6.9	3.5	5.2	0.6	1.5	2.0	4.5	1.3	6.2
LFNG	11.0	3.9	5.8	1.6	2.2	2.7	2.5	0.6	1.4
CCNB1	9.5	3.6	4.7	0.5	1.9	2.6	4.3	0.6	4.1
OBFC1	9.0	3.6	5.1	0.7	1.8	2.7	3.9	0.6	4.4
CRAT	10.4	4.5	5.4	1.2	2.1	3.0	2.7	0.4	2.0

MPC1	6.6	3.4	4.5	0.6	1.3	2.5	6.1	1.0	5.7
ENDOV	10.3	3.5	4.8	0.8	1.3	1.9	5.6	0.6	2.8
CDK2	8.0	2.9	3.7	0.4	1.1	1.8	6.9	0.8	6.0
CBWD5	9.0	3.1	4.3	0.3	1.1	1.8	7.3	0.8	4.0
FAM49B	8.6	3.2	4.6	0.4	1.4	1.6	6.3	0.8	4.8
GLI3	8.9	2.8	3.8	1.1	1.9	2.6	5.0	0.9	4.7
TM7SF3	9.7	2.6	4.3	0.5	1.2	1.4	7.1	0.7	4.1
DAB2IP	9.6	3.7	4.3	0.9	1.4	2.0	4.9	0.9	4.0
VPS13B	10.0	2.9	3.9	0.4	1.3	1.6	6.2	0.6	4.8
FAM161A	10.1	2.4	4.4	0.6	1.3	1.5	7.3	0.8	3.4
TMEM161A	8.4	3.6	4.4	1.4	2.3	3.3	4.5	0.7	3.1
SNX6	8.3	3.6	5.1	0.2	1.0	1.6	6.5	0.6	4.8
LOC101928104	9.9	1.9	4.4	0.6	1.3	1.4	7.9	0.7	3.5
MZT1	7.2	3.1	3.5	0.8	2.2	1.5	5.8	1.5	6.1
SMYD2	7.9	2.9	4.0	0.4	1.8	2.3	6.6	0.6	5.2
DNAJC27-AS1	10.5	2.1	4.5	0.6	1.1	1.4	7.5	0.5	3.3
BIN3	8.3	2.9	4.0	1.1	1.5	2.6	5.5	1.2	4.5
CDR2	7.8	2.9	3.5	0.4	1.4	1.6	6.8	1.5	5.7
LOC101929380	10.2	1.9	4.9	0.4	1.0	1.3	7.6	0.7	3.5
GABARAPL1	9.7	4.0	4.8	0.2	1.2	2.0	5.1	0.9	3.6
LRWD1	8.5	3.4	4.5	0.9	1.7	2.2	5.7	1.0	3.7
MFAP5	9.8	2.2	4.3	0.5	1.2	1.3	8.3	0.7	3.3
RMDN3	8.9	3.6	4.5	0.4	1.6	1.6	6.4	0.7	3.9
LOC101928953	10.0	3.5	4.5	1.0	1.5	2.1	4.9	0.8	3.2
AAAS	8.4	2.7	3.8	0.7	1.6	2.5	6.0	0.9	4.9
C17orf51	7.5	2.5	4.1	0.6	1.6	2.5	5.3	1.3	6.1
GLE1	10.0	3.6	4.1	0.6	1.7	2.0	5.3	0.7	3.6
TBC1D1	8.6	3.1	4.4	0.6	1.7	2.3	5.7	0.8	4.2
PEX19	9.0	3.6	4.7	0.5	1.5	2.3	6.0	0.7	3.2
TIMM10B	9.1	2.6	4.6	0.6	1.0	1.5	7.1	0.8	4.2
NAPG	9.5	3.4	4.6	0.2	1.1	1.2	6.1	0.8	4.6
PNISR	9.0	3.0	4.2	0.6	1.8	2.5	4.9	0.9	4.6
LOC101926943	9.8	2.4	4.1	0.5	1.5	1.4	7.1	0.6	4.0
DYNC1LI1	7.8	2.5	4.5	0.6	0.8	1.8	7.6	0.8	5.3
CAPN5	9.2	4.3	4.9	0.9	1.4	2.3	4.0	1.0	3.5
WASF3	8.8	3.0	4.3	1.1	1.6	2.1	4.6	1.1	5.0
WBP1	9.4	4.6	5.7	1.4	1.3	2.2	4.0	0.5	2.2
GLTPD1	7.7	3.2	3.8	1.9	1.4	2.7	5.2	1.1	4.3
PRKCE	7.9	2.7	3.3	0.9	1.5	1.9	6.8	1.0	5.5
ADCY6	9.1	3.1	4.6	0.7	1.6	1.9	5.3	0.9	4.2
STK17A	9.0	3.1	4.6	0.5	1.2	1.9	5.5	0.9	4.7
SHISA2	6.4	2.0	2.8	3.0	4.7	7.1	2.6	0.9	1.9
LOC440894	5.1	2.3	3.1	1.0	2.0	2.8	7.1	1.2	6.7
RAD1	9.4	2.5	4.3	0.5	1.3	1.7	7.0	0.6	4.1
POGLUT1	8.9	3.6	4.9	0.3	1.2	1.4	5.8	0.8	4.4
ISG15	8.7	3.0	4.4	0.8	2.2	2.2	5.8	0.7	3.7
PTPN14	7.7	2.6	3.4	0.9	1.9	2.5	5.9	1.3	5.2
TBC1D15	10.1	2.3	4.7	0.5	1.1	1.3	7.1	0.7	3.6
LRFN3	9.4	3.3	4.1	1.4	2.1	3.0	4.3	0.9	3.0
MXD1	7.4	2.5	4.2	0.4	1.4	1.4	7.1	1.1	5.8
RHBG	10.3	1.9	4.2	0.7	1.2	1.4	7.9	0.7	3.1
GOSR2	7.0	2.0	3.9	1.2	1.9	2.9	6.3	0.9	5.0
ACYP2	7.8	2.4	5.3	0.6	1.1	2.3	7.1	1.1	3.6
GOLGA4	7.9	2.5	3.5	0.2	0.9	1.3	7.4	1.0	6.5
SIPA1L1	10.1	3.4	4.7	0.5	1.5	2.0	4.7	0.8	3.7

MRPS28	8.5	3.6	4.3	0.6	1.3	1.3	7.1	0.6	4.0
LOC101929680	10.1	2.1	4.6	0.6	1.0	1.2	7.9	0.6	3.2
ZNF692	8.5	3.4	5.0	0.7	1.4	2.1	5.2	0.9	3.9
TK2	10.5	3.9	5.0	0.7	1.3	2.3	3.9	0.5	3.0
THAP7	6.7	3.3	4.1	0.9	1.8	2.8	5.1	1.6	5.0
FNIP1	9.4	3.0	4.6	0.3	1.1	1.2	6.0	0.8	4.8
C19orf25	8.9	3.0	4.7	1.5	2.2	2.5	5.1	0.5	2.7
GTF2F2	7.0	3.0	3.6	0.2	1.4	1.8	7.0	1.1	6.2
DYRK1A	8.8	3.3	4.5	0.4	1.2	1.6	5.1	0.9	5.5
CCM2	9.6	3.3	4.7	0.7	1.4	2.1	5.0	0.8	3.7
TMEM206	6.8	2.8	3.7	0.7	1.4	2.1	7.6	1.2	5.0
HNMT	8.5	4.9	5.6	0.3	1.7	1.8	4.3	0.7	3.4
CENPV	7.4	3.1	3.7	1.9	1.5	1.6	6.6	1.2	4.2
TBC1D3F	9.4	4.0	5.4	0.8	1.5	1.8	4.2	0.8	3.3
PCBD1	5.9	4.4	5.6	1.0	2.3	2.7	3.6	1.0	4.8
VPS18	8.7	3.3	4.3	0.9	1.5	2.1	5.5	1.0	4.0
MICU2	9.0	4.0	4.9	0.5	1.8	2.3	4.3	0.6	3.8
BMP1	11.1	4.4	5.3	0.9	1.6	2.3	2.8	0.5	2.3
TTC7B	8.9	3.0	3.8	0.7	1.2	1.8	6.1	0.9	4.7
TESK1	8.5	3.3	4.3	0.9	1.5	2.3	4.9	1.0	4.5
ETV3	8.2	3.3	4.3	0.6	1.3	2.1	4.9	0.8	5.6
GTF3C6	8.4	3.6	4.0	0.1	1.1	1.9	6.4	0.8	4.9
SS18L2	8.1	3.7	3.3	0.7	1.8	1.8	6.5	0.8	4.3
RFX5	6.0	2.6	3.6	0.8	1.6	2.5	7.0	0.9	6.0
RETSAT	9.4	3.8	4.8	1.0	2.2	3.3	3.3	0.6	2.7
CINP	7.7	2.3	4.3	0.5	1.7	2.6	6.1	0.8	5.1
FAM193A	9.1	2.9	4.5	0.7	1.5	2.2	4.8	1.1	4.4
TXNDC9	7.1	3.4	4.5	0.3	0.9	1.5	6.5	0.8	6.1
KIAA1143	9.0	3.3	4.6	0.5	1.1	1.5	4.3	1.2	5.5
ANKRD50	10.5	3.2	4.3	0.3	1.2	1.5	5.4	0.7	4.1
CCDC167	7.3	3.4	5.5	0.8	1.1	2.3	5.7	1.2	3.9
EFCAB4B	7.8	2.4	3.7	0.5	1.7	1.5	7.7	0.7	5.0
TRMU	8.8	3.2	4.6	0.6	1.1	1.7	5.7	0.8	4.6
LOC643770	10.6	1.9	4.1	0.5	1.2	1.2	8.0	0.6	3.0
C10orf54	8.9	4.6	4.2	0.7	1.2	1.6	4.6	1.1	4.1
FMN1	8.3	2.6	3.5	0.7	1.5	2.0	6.1	1.0	5.3
MMD2	8.8	2.3	5.3	0.5	1.1	1.3	7.3	0.6	3.9
TBC1D3C	10.3	1.9	6.9	0.9	0.3	2.2	3.9	0.1	4.3
LOC101927622	8.2	3.1	4.5	0.6	1.5	2.0	5.9	1.1	4.1
FIZ1	8.6	3.4	4.1	1.1	1.5	1.8	5.6	1.1	3.9
DNAJC21	7.2	3.2	4.0	0.3	1.6	1.9	6.4	0.9	5.4
LOC101928378	7.6	4.5	5.9	0.8	1.6	1.8	3.9	1.1	3.8
GPR89A	7.5	3.0	3.7	0.4	1.2	1.3	7.2	0.9	5.8
DNAAF2	7.4	2.6	4.1	0.7	1.3	1.7	7.0	1.1	5.1
KLC4	10.0	3.6	5.3	1.1	1.4	2.7	4.0	0.4	2.4
PLEK2	6.9	2.9	3.4	0.8	1.5	2.6	6.7	1.1	5.2
PNMA2	8.2	3.4	3.6	0.7	1.5	2.4	5.9	0.9	4.6
FLRT2	9.5	3.1	4.3	0.6	1.5	2.1	4.5	0.6	4.8
PAPSS1	7.8	2.9	4.2	0.5	1.6	2.0	6.6	0.8	4.5
AUH	8.4	2.5	3.3	0.2	1.3	1.4	8.5	0.9	4.5
INCENP	8.2	3.0	3.9	1.4	2.2	2.5	4.8	1.1	3.9
CAAP1	8.6	3.1	3.7	0.7	1.2	1.9	6.6	0.7	4.5
PIGG	10.4	3.0	4.6	0.4	1.4	1.8	5.4	0.6	3.3
ARHGAP27	10.4	3.3	4.9	1.3	1.4	2.2	4.6	0.7	2.1
ZNF587	8.9	2.1	3.8	0.5	1.2	1.6	7.3	0.8	4.7

LOC101928496	10.2	2.2	4.6	0.5	1.1	1.2	7.5	0.5	3.0
TNFRSF11B	7.3	2.8	3.5	0.3	1.0	1.7	7.6	1.1	5.6
EPHB4	7.8	3.4	4.2	0.8	1.6	2.2	5.5	1.0	4.3
EML4	7.9	2.7	3.5	0.4	1.3	1.8	6.9	0.9	5.6
HBP1	9.0	3.2	5.2	0.3	0.9	1.6	5.7	0.8	4.1
FAM57A	6.8	3.2	4.4	1.5	2.0	2.8	4.5	1.3	4.3
NCAPH2	7.7	2.2	3.9	0.7	1.3	1.8	7.6	0.9	4.9
WRAP53	8.7	3.3	4.3	0.5	1.3	1.8	5.9	1.0	4.0
RFC1	8.4	2.9	3.7	0.3	0.9	1.5	6.6	1.0	5.5
C18orf8	7.9	3.2	3.5	0.4	1.5	1.8	5.9	1.1	5.6
E2F5	8.2	3.0	3.4	0.5	1.3	1.6	6.3	1.1	5.5
CLN3	8.9	3.6	4.8	1.2	2.3	2.9	3.2	0.9	2.9
ABHD14B	8.8	3.8	5.0	1.5	3.1	3.6	2.2	0.6	2.3
TAP1	9.3	4.0	5.0	0.7	1.2	2.3	4.2	0.7	3.1
SEC24A	9.5	3.2	4.5	0.3	1.1	1.5	5.0	0.9	4.7
PKN2	8.6	3.2	4.1	0.3	1.3	1.4	6.1	0.9	4.9
PPP6R2	9.0	3.0	4.9	1.3	2.3	2.9	4.3	0.6	2.5
WDR72	10.6	2.6	5.4	1.1	1.5	2.0	3.9	1.1	2.6
NCAPD2	8.2	2.8	3.8	0.6	1.9	2.0	6.1	0.9	4.4
RIOK3	8.4	2.5	3.6	0.3	0.9	1.6	7.0	0.9	5.6
TBX1	10.0	3.4	4.4	0.9	1.0	1.9	4.5	0.8	3.9
NT5C3A	7.2	2.4	3.8	0.4	1.3	1.4	6.5	0.9	7.0
GBAS	10.6	3.8	4.9	0.5	1.2	2.2	4.2	0.5	2.7
LOC101927181	9.6	2.1	4.2	0.6	1.2	1.4	7.8	0.6	3.3
PLEKHH3	10.3	3.9	5.1	0.9	1.6	2.3	3.4	0.7	2.5
FEM1B	7.4	2.7	3.5	0.5	1.2	1.7	6.8	1.0	5.9
MAP4K4	11.5	3.1	4.4	0.8	1.6	2.1	3.6	0.8	2.9
TAF1B	8.3	2.8	3.8	0.3	1.2	1.6	6.8	0.9	5.0
FAM53B	9.2	3.4	5.0	1.0	1.8	2.0	4.0	0.6	3.7
ZNF740	5.5	2.2	3.7	0.3	0.9	1.5	8.5	1.1	7.0
TMEM173	8.1	3.7	3.3	0.9	1.6	1.6	5.4	1.1	5.0
IKBKAP	9.0	3.3	4.7	0.3	0.9	1.2	5.9	0.7	4.6
IL1B	10.6	5.0	7.0	0.8	2.0	2.3	1.5	0.3	1.2
PFKM	6.8	2.8	3.5	0.7	2.2	2.1	7.4	0.7	4.6
ISOC1	7.9	3.4	4.7	0.5	1.3	2.0	5.1	1.1	4.7
DDX31	7.4	3.0	3.5	0.6	1.5	1.9	6.3	1.2	5.3
KDM5B	9.3	3.0	3.9	0.3	1.3	1.5	6.4	0.8	4.2
ZDHHC9	9.0	3.1	3.4	0.6	1.3	2.4	5.4	0.8	4.7
LNPEP	9.3	2.7	3.8	0.4	1.1	1.5	6.1	0.6	5.2
NTHL1	9.2	4.2	4.7	0.9	1.4	2.9	3.3	0.8	3.2
PDPK1	8.6	2.5	3.8	0.6	1.3	2.2	6.0	0.7	4.9
SPIDR	9.2	3.0	4.6	0.6	1.1	1.8	5.8	0.6	4.0
SORD	8.1	3.1	4.1	0.7	1.8	2.9	5.3	0.6	3.9
LOC648987	9.4	2.3	3.9	0.7	1.7	1.5	7.0	0.6	3.5
WDR33	9.3	3.7	4.6	0.6	1.3	1.5	4.4	0.8	4.4
CRYM-AS1	9.9	2.0	3.8	0.6	1.1	1.6	7.7	0.6	3.3
CLDN15	7.9	3.1	4.2	0.8	1.7	1.9	6.1	1.0	4.1
NUBP1	9.2	3.0	4.5	0.7	1.1	2.2	4.8	0.7	4.3
TROVE2	8.5	3.1	3.8	0.2	1.1	1.5	5.9	0.8	5.7
PIGQ	8.1	2.9	4.7	1.7	2.6	3.6	3.8	0.8	2.4
SLC39A10	10.3	3.6	5.4	0.4	1.2	1.6	4.0	0.7	3.5
RBFA	6.9	2.4	3.7	0.6	2.1	3.0	5.9	1.0	4.8
PEX11B	9.9	3.2	4.6	0.3	1.9	2.2	4.8	0.6	3.1
TMEM63A	9.0	2.8	4.9	1.0	1.6	2.6	4.5	0.6	3.5
COL4A2	8.6	3.2	3.9	0.9	2.0	2.5	5.0	0.7	3.8

BCL2L11	10.1	2.3	4.6	0.6	0.7	1.2	6.3	1.0	3.8
NCKIPSD	9.1	3.3	4.1	1.3	2.1	2.6	4.3	0.7	3.1
LOC100129917	8.0	2.2	4.0	0.9	1.6	1.5	6.6	1.0	4.6
LOC100505711	9.8	2.0	3.8	0.7	1.7	1.9	6.8	0.7	3.2
LOC100505876	9.1	2.6	4.7	0.4	1.1	1.6	7.1	0.6	3.3
PDE4DIP	8.7	2.7	4.6	0.8	2.0	2.5	5.0	0.8	3.5
FBXL15	9.8	3.5	4.7	1.2	1.4	2.0	4.8	0.5	2.5
GNAQ	8.7	2.8	3.7	0.5	1.6	1.6	5.1	1.0	5.5
LOC646471	10.0	2.2	4.4	0.6	1.0	1.4	7.5	0.5	3.0
SNORA62	8.5	1.5	4.6	3.8	1.5	3.3	1.5	2.4	3.3
MRPL50	7.9	3.0	4.4	0.6	0.8	1.8	4.9	1.4	5.7
FLJ37453	9.6	2.2	4.5	0.6	1.1	1.4	7.3	0.6	3.1
CHST3	7.6	2.8	4.1	0.8	1.1	1.6	6.4	1.3	4.7
LOC100505564	8.2	3.3	4.0	0.9	1.5	2.2	5.6	0.9	3.8
AP5S1	8.4	3.3	4.8	1.1	2.2	2.6	4.2	1.0	2.8
ACSF3	8.1	3.0	4.2	1.1	2.1	2.7	5.2	0.6	3.3
PEX26	9.4	3.1	4.0	1.1	2.4	3.0	4.7	0.4	2.3
ERICH1	8.1	3.3	4.1	0.9	1.7	2.3	4.8	0.8	4.5
MGA	9.1	2.6	4.7	0.3	1.1	1.6	5.6	0.7	4.6
LINC00632	9.7	1.9	4.2	0.5	1.2	1.6	7.2	0.8	3.2
LINC00269	10.3	1.9	4.1	0.6	1.0	1.2	7.7	0.4	3.1
KIAA0020	6.9	2.3	3.5	0.4	1.6	2.1	6.7	0.8	6.0
CCDC80	9.0	3.2	4.2	0.9	2.9	2.7	3.9	0.7	2.9
PPARA	7.8	2.7	3.5	0.6	1.4	1.8	6.6	1.1	4.8
LOC646762	7.9	2.5	3.6	0.6	1.2	1.9	6.8	1.0	4.8
PUS3	7.3	2.9	4.4	0.3	1.2	1.6	6.0	1.0	5.5
GYS1	7.1	2.6	3.8	1.2	2.3	2.9	5.1	1.1	4.2
C6orf226	9.9	3.7	5.6	1.1	2.8	1.8	3.1	0.1	2.0
SLFNL1-AS1	9.7	2.1	4.3	0.7	1.0	1.3	7.5	0.6	3.2
PGP	6.7	2.4	3.7	0.8	1.7	2.3	6.8	1.0	4.8
BET1L	6.4	2.1	4.0	0.7	0.7	1.2	8.4	1.3	5.4
SEPW1	6.5	2.9	7.0	0.6	1.7	2.0	4.5	1.0	4.2
POLR2M	8.0	3.6	4.5	0.5	1.4	1.9	4.8	1.0	4.5
FRAT2	6.2	2.9	3.0	1.0	1.7	2.1	7.6	1.2	4.5
CEP164	8.4	3.2	4.3	0.7	2.0	2.4	4.8	0.9	3.5
FILIP1L	5.9	2.6	3.0	0.2	1.1	1.0	7.5	1.4	7.4
PRKCD	8.1	3.3	4.4	0.8	1.3	2.4	4.9	0.8	4.3
STXBP6	8.4	3.1	4.3	0.3	1.2	1.3	4.7	1.1	5.8
SAMD8	8.3	3.2	4.7	0.3	1.0	1.6	5.3	0.8	4.8
HGSNAT	9.1	3.1	5.0	0.9	1.5	2.1	4.6	0.8	3.1
NPIPA2	9.9	3.5	4.8	0.3	1.5	2.3	3.1	0.6	4.2
DNAJC14	7.7	3.1	3.6	0.7	1.5	2.0	5.8	0.7	4.9
ANKIB1	8.7	3.0	4.4	0.3	1.2	1.5	5.8	0.7	4.4
TMEM87A	6.7	3.4	5.0	0.4	1.2	1.6	6.4	0.9	4.5
TMEM39A	7.5	3.3	4.3	0.2	1.4	1.5	5.7	1.1	5.2
AP3S2	9.3	2.8	4.6	0.7	1.4	1.9	5.5	0.6	3.3
TRMT5	7.6	2.8	4.1	0.3	1.4	2.2	6.1	0.8	4.8
CERS4	10.1	3.8	5.5	0.6	1.2	2.1	3.2	0.8	2.7
DOLPP1	6.6	2.6	4.2	0.8	2.1	2.7	5.0	1.1	5.0
COG3	8.9	3.1	4.6	0.4	0.8	1.3	5.6	0.8	4.6
SMAP1	8.7	3.1	3.6	0.7	1.2	1.8	5.9	0.9	4.2
GNB4	7.3	2.2	3.1	0.3	1.0	1.7	7.2	0.8	6.5
ACTR8	8.2	3.1	3.9	0.3	1.2	1.8	6.3	0.8	4.4
ALG5	7.3	3.1	4.4	0.2	1.1	1.5	6.6	0.8	5.1
COA6	7.3	2.7	5.2	1.0	1.6	1.4	5.6	0.6	4.6

BTN2A1	8.2	2.9	4.2	0.5	1.2	1.0	5.6	1.3	5.1
MAK16	7.0	1.9	2.9	0.3	1.7	1.6	7.2	1.2	6.2
INTS10	7.7	3.0	3.6	0.5	1.3	1.8	6.9	0.8	4.4
LOC101927134	11.0	2.1	4.4	0.6	1.0	1.2	6.6	0.4	2.7
TTI1	7.9	3.0	4.0	0.6	2.0	1.9	5.1	0.8	4.7
UNC50	7.5	3.3	4.7	0.5	1.3	1.8	5.7	0.7	4.5
KLF13	7.7	3.5	3.5	1.5	1.8	2.7	4.6	0.8	3.8
CREBZF	8.1	3.2	4.4	0.6	1.3	1.5	5.9	0.9	4.1
AGBL5	8.3	3.3	3.9	0.6	1.5	2.2	5.2	0.7	4.3
TNFAIP3	9.5	3.6	5.4	0.6	0.9	1.5	4.0	0.9	3.6
LOC101060037	10.7	4.6	7.1	0.4	0.9	1.2	2.0	0.5	2.5
C11orf54	9.2	3.5	4.7	0.1	1.3	1.0	6.0	0.4	3.6
MED8	7.2	2.6	4.3	0.5	1.1	1.2	6.8	0.9	5.3
LOC100505812	8.6	1.9	4.1	0.6	1.2	1.5	7.6	0.6	3.8
SMCO4	10.1	3.0	3.8	0.7	1.0	1.2	5.4	1.3	3.5
FAM160B2	8.1	3.3	4.7	1.1	1.8	2.7	3.9	0.9	3.3
ZW10	9.2	3.4	4.3	0.5	1.0	1.6	5.2	0.7	3.9
NR2C1	8.4	3.6	5.5	0.4	1.2	1.7	4.2	0.8	4.1
RIN1	6.9	3.1	4.0	1.5	2.9	3.3	4.0	0.8	3.3
SGK1	5.4	1.8	2.4	0.5	1.3	1.7	8.8	1.4	6.4
MOSPD3	9.4	3.0	4.3	1.7	1.8	3.0	2.9	0.7	3.1
TAF5L	8.3	2.6	2.7	0.3	1.6	1.9	6.2	1.1	5.2
SPECC1L-ADORA2A	8.4	2.9	3.8	0.6	1.3	1.8	5.8	0.9	4.4
NADK	7.2	3.0	3.9	0.8	1.7	2.1	6.2	0.9	4.1
PWP2	6.9	2.3	3.5	0.6	1.5	1.8	7.3	0.9	4.9
NBPF16	7.6	2.7	3.8	0.4	1.4	1.8	6.9	0.3	4.9
HINT3	8.9	3.0	3.7	0.8	1.0	1.8	5.4	1.1	4.1
KIAA0513	8.5	3.2	3.9	0.6	1.4	1.6	5.2	0.9	4.5
CCDC43	8.6	2.8	3.3	0.5	0.8	1.6	6.1	1.0	5.1
ZFP41	9.4	3.3	4.2	0.7	1.4	1.9	4.4	1.1	3.5
DNMBP-AS1	9.7	2.0	4.2	0.5	1.1	1.4	7.4	0.4	3.0
TMEM243	8.2	3.3	4.1	0.2	1.2	1.1	5.2	0.5	6.0
ARNT	8.4	3.2	4.5	0.5	1.1	2.1	4.7	0.9	4.3
ADCK5	7.8	4.0	5.4	0.7	1.7	2.0	4.3	0.9	2.9
NAGLU	10.4	4.2	5.2	1.1	1.7	2.7	2.2	0.4	1.9
NIN	8.4	2.5	3.6	0.4	1.2	1.4	6.4	0.8	5.1
INTS6	8.8	2.4	3.0	0.4	1.0	1.4	7.2	0.8	4.7
RABEPK	6.0	2.7	3.4	0.4	1.4	1.7	7.3	0.9	5.9
LOC100506325	10.3	2.0	4.6	0.6	2.0	2.0	4.8	0.4	3.1
RNF14	7.4	3.2	4.0	0.2	1.1	1.6	7.0	0.6	4.7
UBE3A	7.5	2.7	3.6	0.2	1.1	1.2	7.7	0.6	5.0
SNORD41	8.8	1.5	3.4	0.6	1.8	2.6	4.2	0.8	6.1
CAT	8.2	3.7	4.7	0.8	2.4	3.1	3.7	0.5	2.6
CDC14B	9.5	2.2	3.8	0.5	1.1	1.4	6.6	0.7	3.8
PPFIBP1	7.2	2.5	3.4	0.4	1.6	2.1	6.4	0.8	5.2
RRM2	4.0	1.7	2.4	1.6	2.3	3.3	6.7	1.5	6.2
SLC35F3	8.0	2.8	3.7	1.0	2.2	2.5	5.0	0.6	3.8
TRPT1	7.7	3.4	5.1	1.1	1.8	2.5	4.7	0.6	2.7
MAP4	8.3	3.1	4.1	0.6	1.4	2.0	4.5	1.0	4.7
ZFAND2B	7.6	3.3	4.3	0.4	1.6	2.3	4.4	0.9	4.8
CHST14	9.2	3.4	4.4	1.0	1.8	2.6	4.0	0.6	2.8
FBXW7	8.6	3.7	3.6	0.2	1.2	1.1	6.6	0.6	4.0
MTMR2	8.2	2.8	3.4	0.3	0.9	1.3	7.0	0.9	4.8
IFT27	6.9	2.4	4.0	0.6	1.8	2.4	6.9	1.0	3.5
POLR3A	7.6	2.2	3.6	0.4	1.2	1.6	7.4	0.9	4.7

MSI2		8.3	2.7	4.8	0.6	1.8	2.0	4.5	1.1	3.9
CDK13		9.0	3.0	4.0	0.4	1.2	1.3	5.9	0.8	4.0
FLJ31306		8.1	2.6	3.9	0.3	1.2	1.6	6.5	0.7	4.7
UBQLN2		8.1	3.2	3.6	0.6	1.4	2.0	5.5	0.8	4.4
PCNT		7.9	2.4	3.8	0.8	1.5	1.8	6.3	0.8	4.1
E4F1		7.5	2.8	4.1	1.1	1.5	1.8	5.6	1.0	4.2
RNF169		7.1	2.5	3.3	0.6	1.5	2.1	5.7	1.1	5.8
PEX6		8.6	3.2	4.4	0.9	1.8	2.3	5.0	0.6	2.8
ZNF274		8.6	3.2	3.8	0.5	1.5	1.6	5.4	0.7	4.2
GTF2A2		7.5	3.4	4.2	0.4	1.8	2.1	4.0	0.8	5.4
TULP3		8.4	2.2	3.4	0.4	0.8	1.2	6.9	1.0	5.4
CKMT2-AS1		9.2	2.3	4.7	0.5	1.3	1.5	6.2	0.6	3.2
SESN2		7.0	3.1	3.6	1.0	1.7	2.9	5.4	0.8	3.9
LOC100630923		8.2	2.9	4.0	0.8	1.3	1.8	5.8	0.8	3.7
DHX32		10.4	4.0	5.5	0.4	1.3	1.8	3.5	0.3	2.3
TSIX		9.6	2.8	4.6	0.4	1.2	1.4	5.0	0.7	3.7
MRRF		8.1	3.0	4.4	0.3	1.2	1.4	6.1	0.7	4.3
SIMC1		8.2	2.9	4.0	0.6	1.4	1.7	5.0	1.0	4.6
PLSCR1		8.3	3.3	4.6	0.7	1.5	2.0	5.2	0.7	3.2
SHANK2-AS1		9.2	1.9	4.1	0.5	1.4	1.6	7.1	0.7	3.0
ELP3		8.4	2.6	4.1	0.3	1.4	1.7	5.9	0.6	4.6
SLC39A4		6.5	4.6	3.5	1.8	3.0	2.3	3.9	1.2	2.7
TAB3		8.8	3.1	4.5	0.5	1.7	2.0	3.9	0.6	4.3
ARHGAP12		8.1	2.9	3.8	0.3	1.0	1.7	5.6	0.9	5.2
SAR1B		8.7	4.1	5.3	0.3	0.7	0.9	5.3	0.4	3.7
VPS52		7.9	3.0	4.0	0.7	2.1	2.3	4.5	0.9	4.0
STAG3L5P-PVRIG2P-PILRB		8.8	3.3	5.2	0.9	1.7	1.9	4.0	0.5	3.1
NOC3L		8.0	2.3	3.2	0.2	1.1	1.4	7.0	0.6	5.7
HSDL2		8.7	2.6	4.1	0.5	1.8	2.2	4.6	0.7	4.1
SAT1		7.6	3.9	4.7	0.2	1.3	1.6	4.9	1.0	4.2
	44622.0	10.7	3.3	5.6	1.0	1.8	2.4	2.4	0.5	1.7
FBXO28		9.1	3.1	4.1	1.2	1.3	1.5	4.4	0.9	3.9
FBXL8		8.9	4.0	5.1	1.8	1.7	2.2	2.9	0.5	2.3
PCM1		8.9	2.7	3.9	0.3	1.4	1.7	5.6	0.6	4.3
USP54		9.6	2.3	3.9	0.6	1.1	1.4	6.1	0.7	3.7
LINC00996		9.3	2.1	4.3	0.4	1.2	1.4	7.3	0.5	2.9
ZNF524		9.2	4.2	4.6	0.8	1.7	1.8	3.3	0.6	3.1
CCRN4L		6.3	1.9	2.5	0.7	1.1	1.1	7.5	1.4	6.8
BBS2		8.9	3.3	4.4	0.6	1.3	2.0	4.7	0.8	3.4
MOAP1		7.8	3.1	3.3	0.3	0.8	1.2	7.2	0.8	4.8
LYPLA1		6.3	2.8	4.9	0.4	1.5	2.2	5.2	0.6	5.4
TMEM87B		7.9	2.8	4.6	0.2	1.0	1.2	6.6	0.6	4.6
NIPA1		8.9	2.6	4.2	0.7	1.4	1.7	4.8	0.9	4.0
CAP2		8.8	3.1	5.0	0.5	1.7	2.0	3.9	0.7	3.6
ASAP1-IT2		9.0	2.4	4.2	0.4	1.1	1.3	7.1	0.6	3.1
ATP6V1A		9.8	3.0	4.1	0.3	1.0	1.7	5.4	0.5	3.4
NUDT16		6.5	2.5	3.0	1.2	1.8	3.0	6.0	0.8	4.6
TMEM70		6.8	2.6	4.4	0.4	1.1	1.4	7.2	0.7	4.8
DEF6		9.4	3.6	4.6	0.9	1.3	1.6	4.0	0.7	3.2
SLC30A5		7.6	2.6	4.1	0.3	0.9	1.2	6.8	0.7	4.8
TAB1		8.4	3.1	4.1	0.9	1.3	2.1	5.2	0.7	3.3
TANGO2		9.3	3.4	4.4	1.0	0.9	2.3	3.7	0.8	3.5
CARKD		6.9	2.5	3.6	1.0	1.5	1.4	7.5	0.8	4.1
FOXO4		9.0	3.4	4.6	0.9	1.7	2.0	3.6	0.8	3.2
VWA9		9.0	3.5	3.7	0.2	1.4	1.4	5.0	1.0	4.0

ZNF8	7.4	2.8	4.0	0.5	1.2	1.6	6.0	0.9	4.7
TGFBRAP1	8.0	2.5	3.5	0.5	1.0	1.6	6.0	1.1	4.9
VAMP8	7.8	4.0	5.6	0.5	2.1	2.3	3.4	0.9	2.8
SCOC	8.3	3.6	4.9	0.9	1.8	2.4	3.4	0.8	3.0
PNRC2	6.9	3.5	4.0	0.2	1.2	1.3	5.1	1.2	5.8
CCDC102A	7.4	2.8	3.9	1.0	1.4	1.8	6.2	0.9	3.8
LTBP1	11.3	3.7	5.3	0.5	1.3	1.6	2.9	0.5	2.0
LOC101929390	9.3	2.0	4.1	0.4	1.1	1.3	7.1	0.5	3.3
ACADM	7.3	3.2	3.6	0.3	1.3	1.4	6.8	0.8	4.4
CTSL	9.9	4.1	4.5	0.6	1.0	2.2	4.2	0.3	2.4
ARHGAP26	9.3	3.5	5.2	0.5	1.1	1.6	3.9	0.6	3.4
LMBR1	7.8	2.5	4.1	0.2	0.7	1.5	6.5	0.8	5.0
TMEM41B	8.5	3.6	4.5	0.6	1.3	1.8	3.8	1.0	3.9
ZNF771	5.9	2.2	3.5	1.8	2.1	3.0	6.0	1.0	3.6
SECISBP2	8.6	3.2	4.5	0.5	1.5	1.9	4.0	0.6	4.2
SCMH1	8.0	2.8	3.5	0.6	1.6	2.4	5.1	0.8	4.3
C1orf123	7.1	3.5	4.8	1.0	1.9	2.6	4.7	0.5	2.9
CDC27	8.7	3.1	3.9	0.3	1.0	1.6	5.3	0.7	4.5
LOC286186	9.9	1.9	4.0	0.6	1.0	1.1	7.3	0.5	2.6
CIDEB	7.0	3.1	3.9	0.5	1.6	2.2	5.3	0.9	4.5
MGC57346	8.6	2.6	3.7	0.5	1.1	1.5	6.6	0.6	3.9
DHRS13	8.8	3.0	4.2	0.4	1.1	1.7	5.7	0.8	3.3
ZFP64	7.4	2.7	3.4	1.1	1.8	2.4	4.7	1.2	4.4
CLDN7	7.1	3.9	4.1	0.9	1.4	2.3	5.0	0.8	3.5
ZNF638	8.8	2.7	4.1	0.3	1.2	1.3	5.5	0.6	4.5
GLIS3	8.0	2.7	4.3	0.9	1.4	2.0	5.0	1.0	3.7
COG4	9.3	3.5	5.0	0.5	1.4	1.7	4.0	0.6	2.9
KIAA1199	9.7	3.5	5.0	1.2	2.7	3.2	1.9	0.5	1.3
DNAJC13	9.6	3.1	4.3	0.2	1.4	1.8	4.7	0.5	3.4
BTBD3	6.5	2.5	2.7	0.5	1.2	1.5	7.0	1.1	5.9
SMOX	9.4	2.3	2.7	0.5	1.1	1.2	6.4	0.7	4.5
RBL2	9.6	3.1	4.3	0.6	1.8	2.2	4.0	0.5	2.8
FBXW9	8.0	2.5	4.8	0.8	1.6	1.9	4.5	0.9	4.0
TOM1L2	8.7	3.7	4.6	0.7	1.9	2.3	4.2	0.5	2.4
P4HTM	7.4	3.1	4.6	1.0	1.4	2.2	5.1	0.8	3.3
MIIP	6.0	2.8	3.5	0.7	1.5	1.9	6.5	1.4	4.5
KCNK1	7.1	3.1	3.5	0.6	1.5	1.3	6.0	0.9	4.9
HIBADH	8.1	3.0	3.5	0.3	1.9	2.1	5.9	0.8	3.5
LOC101928527	9.3	1.6	4.2	0.7	0.8	1.6	7.2	0.4	3.1
ZNF623	7.4	2.8	3.5	0.4	1.2	1.3	6.3	0.9	5.1
XPNPEP3	8.0	2.9	3.9	0.5	0.8	1.5	5.9	0.6	4.9
CCDC107	6.5	2.4	3.8	1.1	2.1	2.5	5.4	1.2	3.8
ANP32A-IT1	9.4	1.7	4.1	0.6	1.1	1.2	7.0	0.5	3.2
HPS6	7.1	3.2	4.0	0.7	1.5	1.9	6.1	1.0	3.4
HRH1	7.3	2.6	3.7	0.8	1.2	2.2	5.1	1.1	5.0
GDPD5	8.4	3.4	3.7	0.9	1.2	1.8	4.5	0.8	4.1
STK16	8.9	3.6	4.8	0.5	1.3	1.8	4.0	0.6	3.3
LOC400548	9.6	2.1	3.9	0.6	0.9	1.6	6.5	0.7	3.0
LINC00243	9.7	1.7	3.7	0.4	1.1	1.3	7.5	0.4	2.9
LOC101927033	7.7	1.5	4.4	0.9	1.4	1.1	7.3	0.7	3.8
PPIL4	7.6	3.2	3.5	0.2	1.0	1.4	5.9	0.8	5.0
ATPAF2	8.0	3.0	4.3	0.6	1.8	1.8	5.2	0.7	3.6
FAM58A	7.3	3.4	4.6	0.5	1.2	2.0	5.1	0.7	4.0
LOC100505685	9.0	2.2	4.0	0.5	1.0	1.2	7.2	0.5	3.1
NBR2	8.4	2.8	4.2	1.2	1.9	2.5	4.2	0.6	3.0

AFTPH	8.2	3.2	3.4	0.2	0.8	1.5	6.4	0.8	4.3
ATP11B	8.0	3.0	3.9	0.3	1.2	1.6	5.4	0.7	4.7
NCK1	7.1	2.4	3.5	0.1	1.1	1.2	6.9	0.8	5.7
SLC25A29	6.8	2.2	3.4	0.6	1.4	1.8	6.7	1.1	4.7
LOC101928464	9.3	1.7	4.2	0.5	0.8	1.5	6.5	0.8	3.4
HSDL1	8.2	3.0	3.7	0.3	1.0	1.6	6.6	0.6	3.8
NUP205	7.1	2.7	3.4	0.3	1.1	1.4	6.8	0.7	5.2
FLJ44342	10.0	2.6	5.0	0.3	1.1	1.4	4.5	0.5	3.2
NSDHL	7.7	3.4	4.5	0.4	1.1	1.3	4.7	1.0	4.6
NRP2	6.7	2.4	2.8	0.5	0.9	1.2	7.2	1.3	5.7
FOSB	6.1	3.6	5.1	0.9	1.6	2.3	5.2	0.9	2.9
ARHGAP24	10.4	3.4	4.6	0.3	1.1	1.4	3.9	0.6	2.8
WDYHV1	9.6	2.8	4.9	0.3	1.2	1.8	4.0	0.6	3.5
C11orf71	8.6	2.9	5.2	0.9	1.8	1.7	4.0	0.8	2.7
ZDHHC20	8.5	3.8	4.0	0.2	0.9	1.2	4.7	0.8	4.6
SCPEP1	9.7	3.7	5.0	0.4	1.3	1.8	3.4	0.5	2.9
AIFM2	7.7	3.0	4.2	0.9	1.2	2.2	4.9	0.8	3.8
SCARNA9L	7.8	2.4	3.6	0.4	1.0	1.8	4.3	0.5	6.9
STX7	8.3	2.9	4.4	0.2	1.1	1.9	4.1	0.5	5.2
FBXO8	8.7	3.2	4.5	0.4	1.2	1.8	4.5	0.6	3.7
TENM3	7.6	2.3	3.3	0.6	1.2	1.7	6.4	0.9	4.5
NAA38	7.8	2.5	4.7	0.3	1.5	1.8	4.9	0.7	4.3
SIDT2	8.3	3.5	4.4	1.0	1.6	2.8	3.5	0.6	2.9
KLHL24	9.8	3.3	4.5	0.3	0.9	1.5	4.7	0.4	3.2
SCAF11	7.6	2.7	3.6	0.3	1.1	1.5	5.5	0.9	5.4
CRNKL1	8.0	3.0	3.5	0.5	1.0	1.7	5.9	0.9	4.1
PIGO	8.7	2.2	3.9	0.5	1.4	1.7	6.2	0.5	3.5
FBN1	9.8	2.9	4.0	0.3	0.9	1.3	5.4	0.6	3.3
EXOSC8	6.1	2.2	4.2	0.3	0.9	2.0	7.0	0.7	5.2
BOK	8.1	2.4	3.2	1.1	1.7	2.2	5.6	0.6	3.4
SOCS5	7.6	2.4	3.8	0.3	1.1	1.5	6.1	0.9	4.9
GRAMD4	9.0	3.2	3.9	0.5	1.1	1.6	5.1	0.7	3.4
LOC255177	8.9	1.9	3.9	0.5	1.0	1.3	7.2	0.5	3.2
BAGE2	7.5	2.3	3.7	0.6	1.7	1.5	6.4	0.9	3.9
NDUFAF4	7.1	3.2	4.3	0.5	1.0	0.9	6.3	0.5	4.5
KDM3A	9.0	3.2	4.1	0.3	0.9	1.2	5.2	0.6	3.9
JPX	8.4	1.9	4.1	0.7	1.5	1.2	6.5	0.6	3.4
CEP104	8.5	2.9	4.0	0.5	0.8	1.6	5.6	0.7	4.0
TOP2B	8.4	2.6	3.5	0.2	1.0	1.3	6.0	0.8	4.5
KDM4B	8.9	3.1	4.1	1.0	1.6	2.0	4.0	0.8	2.9
PSD3	7.6	2.3	3.8	0.4	1.4	1.4	6.0	0.7	4.7
TRMT2A	6.0	2.6	3.2	0.9	1.6	2.9	5.7	1.2	4.4
INTS4	7.6	2.7	3.4	0.4	0.9	1.6	6.3	0.6	4.8
ACN9	6.7	3.3	4.7	0.9	1.1	1.9	4.6	0.5	4.5
DDIT3	6.3	3.2	3.0	0.7	1.2	1.9	6.5	0.9	4.4
SNORA54	7.7	2.9	3.6	1.1	1.5	2.1	6.3	1.1	2.1
BAIAP2-AS1	8.6	2.3	3.9	0.6	1.2	1.6	6.0	0.6	3.4
ZFYVE26	8.3	2.6	3.8	0.6	1.4	1.9	5.2	0.8	3.7
GMEB2	7.3	2.5	3.7	0.9	1.6	2.1	4.8	1.4	4.0
IMPA1	7.0	3.0	4.3	0.3	1.1	1.4	6.9	0.6	3.8
LOC101928935	7.7	2.3	4.3	0.3	0.8	1.1	5.9	1.0	4.9
LOC101928254	9.0	2.4	4.4	0.7	1.5	1.6	4.3	0.6	3.6
ZCRB1	7.9	3.3	4.5	0.3	1.3	2.0	4.1	0.7	4.0
TOPBP1	6.8	2.3	3.3	0.2	1.0	1.2	6.6	1.0	5.9
AFMID	7.2	3.0	3.7	0.8	2.2	2.1	5.2	0.5	3.6

SRD5A1		7.1	2.7	4.2	1.2	2.1	2.2	4.4	1.0	3.3
PJA1		7.4	2.6	3.0	0.6	1.2	2.0	6.0	1.1	4.3
C9orf172		7.7	3.0	4.2	1.3	1.6	2.2	4.4	0.9	3.0
HBS1L		6.5	2.7	3.1	0.3	1.0	1.4	6.8	1.0	5.4
LRP11		8.1	3.0	4.4	1.1	1.6	2.3	3.9	0.8	2.9
FANCE		6.4	2.3	2.9	0.7	1.3	1.8	6.9	1.0	4.9
RPS6KB1		8.0	3.4	4.1	0.4	1.0	1.4	4.9	0.8	4.1
HNRNPLL		7.7	2.1	3.3	0.4	1.0	1.7	6.9	0.6	4.5
PRICKLE1		6.7	2.3	3.0	0.5	1.4	1.8	6.5	0.8	5.1
PROSER2		6.8	2.2	3.3	0.8	1.4	1.4	6.0	1.3	4.9
SCFD1		7.4	2.9	4.0	0.2	1.3	1.7	5.2	0.6	4.7
RBM4B		7.7	3.0	4.4	0.6	1.5	1.7	4.5	0.7	4.0
PPM1B		7.1	2.3	3.0	0.1	1.1	1.4	6.9	0.9	5.4
NDUFV3		6.2	2.5	3.1	0.9	1.9	2.9	5.1	1.0	4.5
PSMC3IP		7.3	2.6	3.5	0.5	1.2	1.6	4.9	0.9	5.6
CXorf56		8.5	2.8	3.3	0.3	1.0	1.3	5.6	0.8	4.6
NDUFC2-KCTD14		9.9	0.0	0.0	2.6	3.1	0.0	9.3	0.0	3.1
PARG		7.9	2.7	4.2	0.4	1.2	1.7	5.1	0.7	4.3
LOC101927274		9.8	1.8	4.2	0.5	1.2	1.5	5.8	0.5	3.0
SLC25A44		8.1	4.1	4.2	0.4	1.2	1.4	4.0	0.5	4.2
	44627.0	7.5	3.1	4.4	0.2	0.9	1.4	5.0	0.5	5.0
ZNF512		8.2	2.8	3.5	0.5	1.6	1.9	4.3	0.8	4.5
MYD88		8.4	2.6	3.6	0.6	1.2	1.5	5.8	0.7	3.6
RNF2		6.5	3.4	4.6	0.3	1.2	1.6	4.3	1.0	5.1
FLJ42627		7.9	1.9	3.7	0.6	1.2	1.6	6.7	0.7	3.8
BLVRA		8.6	3.4	4.0	0.3	0.8	1.3	6.2	0.5	2.9
LOC101929431		8.9	1.8	4.1	0.4	1.1	1.3	6.9	0.5	3.1
CHMP6		7.3	2.0	4.1	1.1	1.8	2.0	5.1	0.9	3.8
STRADA		8.9	2.9	4.0	0.9	1.1	2.5	4.3	0.4	3.0
SNTB2		9.4	2.9	3.6	0.5	1.2	1.7	5.0	0.6	3.2
TMEM126B		6.3	2.9	4.6	0.3	1.3	1.1	5.8	0.7	5.1
LINC00116		7.6	2.8	3.7	1.0	1.4	1.8	5.9	0.7	3.2
SHROOM3		8.7	3.3	4.3	0.4	0.9	1.0	4.6	0.9	4.0
RAB27B		9.1	3.6	4.1	0.3	1.1	2.4	4.0	0.4	2.9
MB21D1		7.3	2.5	3.3	0.8	1.3	1.8	5.9	0.7	4.3
VSTM4		9.0	1.9	4.1	0.5	1.1	1.2	6.4	0.5	3.2
HAUS4		8.9	2.8	4.4	0.5	1.0	1.8	4.6	0.8	3.2
NRIP1		9.4	3.4	4.4	0.3	1.1	1.4	3.7	0.5	3.7
HPN-AS1		9.5	1.6	3.4	0.5	1.1	1.2	7.3	0.4	2.9
LOC100287042		8.5	1.8	3.8	0.7	1.0	1.4	6.4	0.7	3.5
PRRG1		7.4	2.4	3.5	0.5	1.0	1.8	6.3	0.9	4.1
UCK1		7.9	3.1	3.6	0.7	1.9	2.4	4.6	0.6	3.2
FBXO45		6.3	2.5	2.9	0.4	1.4	1.4	6.6	1.2	5.1
RPF2		6.2	2.9	3.0	0.2	0.8	1.3	7.1	0.7	5.7
FARSB		6.9	2.6	3.2	0.3	1.7	2.5	5.1	0.7	4.8
PPP2R2A		6.2	2.7	3.1	0.2	1.2	1.2	5.5	1.0	6.6
STAM		6.1	2.4	2.9	0.5	1.4	1.9	6.1	1.0	5.5
SURF1		7.3	2.4	4.4	0.5	1.9	1.8	5.8	0.4	3.4
PDCD2L		6.5	2.6	3.8	0.7	1.6	1.8	5.8	1.0	4.1
NQO1		8.3	2.6	4.2	0.5	2.7	2.9	4.1	0.3	2.3
RRN3		7.0	3.0	3.2	0.2	0.9	1.7	6.3	0.9	4.7
LOC100133091		7.4	1.9	3.6	0.9	1.6	1.7	6.4	0.7	3.6
ALG9		8.6	3.0	4.2	0.4	1.1	1.3	4.3	0.6	4.4
TMEM9B		8.4	3.0	4.2	0.3	1.1	1.8	4.4	1.0	3.5
PPAP2B		8.4	3.7	4.1	1.2	2.5	4.0	1.2	0.4	2.3

FNDC3A	8.0	2.8	3.4	0.3	0.8	1.2	5.9	0.6	4.7
OCLN	7.9	2.6	3.5	0.3	0.5	0.8	5.7	0.9	5.6
IRF1	8.2	3.0	3.3	0.7	1.5	2.1	4.9	0.5	3.6
TM2D2	6.6	3.4	4.4	0.3	0.7	1.5	5.6	1.0	4.2
ANK3	8.2	2.7	3.4	0.3	1.4	1.8	5.6	0.7	3.8
LENG1	6.9	2.2	3.2	1.2	1.7	1.8	5.1	0.8	4.8
PHF8	7.5	2.3	3.4	0.7	1.1	1.6	6.1	0.8	4.3
ZFAND2A	8.3	2.3	4.5	0.5	1.0	1.5	4.4	0.8	4.5
RBM41	8.9	1.8	4.1	0.3	0.8	1.3	6.8	0.5	3.2
NFKBIE	8.8	2.6	4.1	0.9	1.3	2.0	4.8	0.7	2.5
PROS1	8.9	2.8	4.1	0.1	1.0	1.2	5.0	0.6	3.9
PLEKHG4	8.0	3.2	4.4	0.8	1.8	2.2	3.9	0.7	2.7
RECQL5	7.6	3.4	4.9	1.1	1.7	2.5	3.7	0.4	2.4
ZBTB10	8.6	2.5	3.5	0.4	1.0	1.3	5.7	0.7	3.9
RAP1GDS1	7.2	2.4	3.8	0.2	1.0	1.2	6.1	0.8	5.0
LOC100507437	9.2	1.6	3.7	0.4	0.9	1.6	6.7	0.5	3.1
LOC100128593	9.1	1.7	4.0	0.8	1.2	1.4	6.6	0.6	2.4
FLT3LG	7.8	3.8	3.7	1.3	1.9	2.4	3.6	1.0	2.2
MTFP1	5.6	2.5	3.1	0.5	1.1	1.7	7.5	0.9	4.8
SYNPO	7.1	3.1	3.3	0.9	1.3	1.8	4.2	1.0	4.9
XRCC3	5.5	1.7	3.3	0.6	1.1	1.6	9.2	0.8	3.7
NDUFAF6	7.1	3.3	4.2	0.4	1.3	1.0	5.6	0.5	4.3
TXNRD2	7.0	2.6	3.5	0.7	1.1	1.7	6.4	0.8	3.9
CMC1	7.6	3.4	4.5	1.2	1.7	2.6	3.2	0.5	3.0
C19orf47	6.3	2.5	3.3	0.8	1.8	2.0	4.4	1.3	5.3
SUGT1	6.9	2.9	3.2	0.3	1.0	1.2	6.6	0.8	4.7
PHF13	8.4	2.7	4.0	0.4	1.0	1.3	5.0	0.8	3.9
SLC35A5	8.1	3.1	4.5	0.2	1.2	1.2	4.5	0.5	4.2
RNF123	7.9	3.2	4.7	0.7	1.6	2.0	4.0	0.6	2.9
LMTK2	7.5	2.6	3.5	0.6	1.5	1.8	5.2	1.0	4.0
SMAD4	7.4	2.5	3.4	0.4	1.3	1.6	6.0	0.9	4.0
TUT1	7.4	3.0	4.0	0.8	1.6	2.2	4.5	0.7	3.4
INHBE	4.6	2.2	1.9	0.4	0.6	0.9	9.3	1.3	6.4
MSH6	7.1	2.2	3.0	0.4	1.0	1.5	6.7	0.8	4.9
OXR1	7.1	3.4	3.9	0.3	1.1	1.2	5.8	0.7	4.1
TMX4	8.3	3.4	4.6	0.6	1.5	2.3	3.8	0.4	2.7
MED31	7.0	3.4	4.8	0.4	1.0	1.8	4.7	0.2	4.3
DTYMK	7.0	2.8	3.6	0.5	0.9	2.0	5.7	0.4	4.6
USP24	7.7	2.3	3.3	0.3	1.1	1.3	6.2	0.7	4.7
GNA15	8.5	3.5	4.4	0.7	1.4	1.9	3.6	0.7	2.8
LOC101928218	7.1	1.9	2.5	0.3	0.9	1.5	6.3	1.5	5.5
MED21	7.6	3.5	4.1	0.4	1.3	2.0	3.3	1.2	4.2
HHEX	6.7	2.9	3.0	1.3	1.7	2.1	4.3	1.2	4.2
C9orf3	8.2	2.8	4.4	0.9	1.7	2.3	3.8	0.7	2.7
LOC284577	8.5	2.0	3.5	0.6	1.3	1.3	6.9	0.5	3.0
HADH	7.3	3.0	4.0	0.4	1.6	2.0	5.0	0.6	3.5
SOCS4	6.2	2.8	3.7	0.2	0.9	1.3	6.2	0.8	5.4
MAPKAPK3	7.6	2.5	3.7	0.8	1.5	2.8	4.3	0.7	3.6
LOC285696	9.5	1.7	3.5	0.7	1.2	1.6	5.3	0.5	3.4
MKL1	6.9	2.8	3.6	0.8	1.3	1.8	5.0	0.9	4.4
MFAP2	9.2	4.0	5.1	1.1	1.7	1.7	2.0	0.6	2.1
TRIM26	7.3	2.4	3.5	0.9	1.5	1.7	5.7	0.8	3.7
BRSK1	7.6	2.7	3.9	0.7	0.9	1.4	5.7	0.9	3.5
TIA1	8.0	2.9	5.2	0.4	1.0	1.6	4.2	0.4	3.6
ZNF219	7.0	2.4	3.8	1.0	1.6	2.0	5.1	1.1	3.6

NBAS	8.2	2.7	4.0	0.4	1.3	1.7	4.8	0.6	3.8
LINC00875	9.1	2.0	3.9	0.6	1.2	1.4	6.0	0.6	2.5
ZNF629	7.3	2.8	3.2	1.0	1.7	1.8	4.6	1.0	4.0
TPRKB	7.1	2.1	4.2	0.4	1.2	1.7	6.2	0.6	3.8
INPP1	6.1	3.7	3.4	0.5	1.1	1.6	4.8	0.8	5.4
ZNF7	8.8	2.5	3.8	0.4	1.4	1.5	4.3	0.7	3.9
TUBB2A	7.1	3.0	4.1	0.9	0.7	2.6	4.6	1.0	3.3
TMEM209	7.6	2.5	3.3	0.3	1.0	1.5	5.5	0.8	4.8
LINC-ROR	9.1	2.8	4.1	0.6	1.2	1.3	4.5	0.8	3.0
OSTM1	7.6	3.1	3.9	0.7	1.1	2.0	5.2	0.5	3.3
TELO2	6.6	2.4	3.5	1.2	1.8	2.3	5.5	0.7	3.4
LOC100506476	8.5	1.7	3.9	0.5	0.9	1.4	6.7	0.6	3.1
GLRB	6.6	3.1	3.8	0.2	1.1	1.2	6.4	0.6	4.5
INPP5A	7.0	3.4	3.3	0.6	1.0	2.1	5.1	0.7	4.0
HERPUD2	7.5	2.6	3.1	0.3	1.2	1.6	5.0	0.9	5.2
LZTR1	7.1	2.8	4.0	1.0	2.0	2.3	4.3	0.6	3.2
APPL2	6.5	2.3	3.7	0.6	1.5	2.2	5.5	0.8	4.4
ZMYM4	7.6	2.5	3.4	0.6	1.3	1.8	5.2	0.7	4.3
CNOT6	7.8	3.0	3.4	0.2	0.8	1.5	5.5	0.6	4.5
NKTR	7.9	3.1	4.6	0.2	0.9	1.2	4.4	0.6	4.3
ING4	7.8	2.9	4.4	0.4	1.1	2.2	4.4	0.8	3.3
ECI2	7.5	2.8	3.8	0.5	1.5	1.9	5.1	0.7	3.4
TPP2	8.5	3.1	3.7	0.3	1.1	1.6	4.7	0.5	3.8
PSME4	6.3	2.0	2.5	0.2	1.0	1.1	7.4	0.9	5.9
ATRN	8.6	2.4	3.4	0.5	1.2	1.7	5.2	0.6	3.8
DNAJC19	4.9	3.3	4.3	0.7	1.6	1.8	4.4	1.2	5.2
VTI1A	8.4	2.5	3.5	0.3	1.3	1.4	5.2	0.6	4.1
RCCD1	7.6	2.8	4.5	1.4	2.1	3.0	3.5	0.4	2.0
RNF19A	8.1	3.1	3.9	0.4	1.1	1.4	4.8	0.7	3.7
WBSCR16	8.0	2.2	3.9	0.6	1.2	1.7	5.9	0.6	3.1
PEX10	6.0	2.5	2.4	1.3	2.2	2.5	6.2	0.8	3.4
RBBP6	8.1	3.1	3.5	0.3	0.6	1.0	4.6	1.0	5.1
N4BP2L2	8.6	2.6	3.9	0.2	1.1	1.7	4.8	0.5	3.7
RNF185	6.8	3.1	4.7	0.4	1.4	1.5	4.6	0.7	4.0
NBPF9	7.3	2.3	3.2	0.3	1.4	1.9	5.5	0.9	4.5
LOC100506922	6.6	2.3	3.1	1.5	1.7	2.0	5.1	1.4	3.6
TSPAN6	7.1	2.8	4.0	0.2	1.0	1.6	5.6	0.3	4.6
RELN	6.4	1.7	2.7	0.2	0.7	0.7	7.9	1.1	5.6
LOC642934	8.3	2.2	3.4	0.4	1.3	1.8	5.6	0.5	3.8
CLCC1	7.7	2.0	3.5	0.5	1.4	1.8	5.6	0.8	3.8
IL27RA	7.5	2.9	4.0	0.6	0.9	1.6	5.1	0.9	3.8
CHAF1A	5.4	1.9	2.7	0.7	1.3	2.2	6.7	1.1	5.1
LUZP6	9.4	4.3	3.5	0.0	1.2	1.6	3.4	0.4	3.3
NOL9	7.8	2.6	3.6	0.6	1.4	1.9	4.8	0.7	3.7
FOXC1	4.7	2.4	2.4	1.5	1.6	2.3	5.0	1.7	5.5
FAM120B	8.4	3.4	4.3	0.6	1.8	2.1	3.4	0.5	2.6
MRPS14	5.6	2.5	4.0	0.5	0.8	1.4	6.6	0.7	5.0
LOC101928703	8.5	1.7	3.7	0.5	1.3	1.7	6.2	0.5	3.0
PIKFYVE	8.9	2.6	4.2	0.2	0.7	1.0	4.4	0.9	4.2
LOC100287166	8.2	2.5	3.8	0.4	1.1	1.5	5.3	0.7	3.6
KCTD3	7.7	3.0	3.8	0.3	1.2	1.5	4.9	0.5	4.2
PNPT1	6.8	3.2	3.8	0.2	0.8	1.5	5.6	0.8	4.4
LOC101928053	9.4	1.7	3.5	0.4	1.0	1.3	6.5	0.4	2.8
RAPGEF2	6.6	2.4	3.0	0.5	0.9	1.6	5.7	1.0	5.5
PHYKPL	7.1	2.7	4.0	0.8	1.9	2.3	4.2	0.7	3.4

ACYP1	6.8	3.4	6.0	0.4	1.0	1.5	4.1	0.4	3.4
SLC4A11	7.2	2.9	4.3	0.9	1.8	1.9	3.6	0.8	3.5
RBBP9	7.9	3.2	3.8	0.4	1.6	1.6	3.6	0.7	4.2
WDR24	7.3	2.4	3.1	1.4	1.7	2.3	4.7	0.9	3.3
LIN37	6.6	2.3	3.8	0.9	1.7	2.1	4.9	1.0	3.6
TRUB1	7.6	3.3	4.3	0.3	1.0	1.9	4.8	0.5	3.3
GTF2E2	6.5	2.6	3.4	0.3	0.8	1.3	5.9	0.8	5.5
LRIG1	8.1	2.5	3.9	0.9	1.9	2.5	3.1	0.6	3.5
BRWD1	6.7	1.0	4.7	0.1	1.1	1.0	6.2	0.2	6.0
CPNE3	8.1	3.1	4.4	0.3	1.1	1.4	4.3	0.4	4.0
CLEC16A	8.5	2.7	3.8	0.9	1.1	1.8	4.2	0.7	3.2
ZNF263	6.8	2.4	3.1	0.5	1.0	1.2	6.1	0.7	5.1
ETV6	7.1	2.9	3.8	0.4	1.5	1.9	3.9	1.0	4.3
PRKAG1	6.9	3.4	3.9	0.3	1.6	1.9	4.8	0.7	3.4
SNORD55	5.9	1.4	1.4	2.7	1.9	1.9	6.0	1.4	4.2
KIF22	5.3	2.1	2.9	0.8	1.8	1.9	6.4	1.0	4.8
SGMS1	7.2	2.6	3.3	0.3	1.0	1.4	5.4	0.9	4.8
TACC3	5.9	2.4	3.1	0.8	1.7	2.3	6.0	0.9	3.9
CD27-AS1	8.9	2.1	4.7	0.6	1.3	1.3	4.4	0.4	3.3
LOC101928074	6.2	2.1	3.3	1.0	1.4	1.3	5.3	1.4	4.9
KMT2C	7.7	1.9	3.3	0.4	1.1	1.3	5.8	0.8	4.6
RNF19B	7.7	2.6	3.7	0.8	1.1	1.7	4.9	0.9	3.5
PBRM1	7.8	2.4	3.2	0.4	1.1	1.3	5.5	0.8	4.4
DDX11	6.9	2.9	3.9	0.7	1.5	1.8	5.0	0.7	3.5
AP5B1	7.9	2.8	3.7	1.0	1.5	2.3	4.3	0.7	2.8
ZNF74	7.3	2.4	3.4	0.6	0.9	1.3	5.8	0.9	4.3
ZNF281	6.0	2.6	3.5	0.5	1.1	1.5	5.6	0.9	5.3
CLPX	6.8	3.2	2.8	0.2	1.1	1.2	5.6	0.8	5.2
CLASP1	8.3	3.0	4.4	0.4	1.2	1.6	4.2	0.6	3.2
LOC101927556	6.5	2.5	2.9	0.2	0.8	1.1	6.7	0.8	5.3
LINC00674	8.1	2.3	3.8	0.6	1.2	1.5	5.4	0.7	3.2
PDE7B	9.5	3.4	4.1	0.8	1.8	2.2	2.3	0.4	2.4
C11orf57	6.7	2.6	3.4	0.2	1.0	1.3	5.3	0.9	5.5
MYLK2	8.6	1.8	3.9	0.5	1.0	1.1	6.8	0.5	2.7
TIMM23B	9.8	2.5	0.6	0.1	0.1	1.4	7.3	0.4	4.7
MTMR11	6.7	2.6	3.3	0.4	1.4	1.4	5.6	0.9	4.6
NUDT6	6.3	2.1	3.9	0.5	1.0	1.5	6.0	1.0	4.5
LGR4	9.9	4.3	5.2	0.4	1.1	1.5	2.3	0.3	1.7
PRAF2	7.4	3.3	2.6	2.0	2.9	4.0	2.1	0.5	2.1
KIAA0101	6.5	1.4	3.5	0.4	0.6	1.1	8.2	1.1	4.0
DDX50	8.2	3.1	4.1	0.3	1.0	1.1	5.1	0.6	3.3
SRPK2	5.9	2.5	3.4	0.3	1.0	1.4	5.9	1.0	5.3
NNT-AS1	8.9	1.9	3.7	0.5	1.1	1.7	5.3	0.6	3.1
TET3	6.7	2.0	3.0	0.7	1.3	1.5	5.7	1.1	4.8
SLC31A1	6.4	3.3	3.8	0.3	0.8	1.7	4.8	0.6	4.9
LOC101927655	7.9	2.3	3.1	0.9	1.5	2.2	4.9	0.7	3.2
ARHGEF26	6.5	2.2	2.9	0.7	1.7	2.2	5.8	0.7	4.1
TEX10	6.3	2.6	3.0	0.3	1.1	1.5	5.7	0.7	5.5
MTMR3	7.6	2.7	3.7	0.4	1.2	1.3	5.1	0.7	4.0
TNRC6B	7.1	1.9	3.4	0.6	1.4	1.6	4.9	1.0	4.8
ATAD3B	6.9	2.2	3.4	0.8	1.5	1.6	5.5	1.0	3.8
ARFGAP2	7.6	3.3	3.3	0.6	1.5	1.7	4.6	0.7	3.4
LCMT1	7.5	2.9	4.0	0.3	1.4	1.4	4.9	0.5	3.6
NBPF1	6.7	2.2	3.4	0.2	1.1	1.2	6.4	0.8	4.7
RALB	8.0	2.5	2.9	0.7	1.3	1.2	5.4	0.7	4.1

SETD2	8.0	2.4	3.4	0.3	1.0	1.3	5.1	0.9	4.4
C2orf49	6.2	2.7	3.7	0.5	1.5	1.9	5.6	0.7	4.0
POP5	4.9	2.2	3.7	0.7	2.7	2.7	4.9	1.3	3.6
BHLHE40-AS1	8.7	1.7	3.8	0.4	1.1	1.2	6.7	0.5	2.6
ZNF746	6.0	2.5	3.3	1.0	1.4	1.8	5.3	0.9	4.4
BCL11A	5.6	2.0	2.5	0.9	1.3	1.7	5.3	1.4	6.1
SECTM1	5.8	3.3	4.1	1.2	2.0	3.1	3.8	0.5	2.9
LOC101927797	9.0	1.8	3.6	0.4	1.3	1.1	4.9	0.4	3.9
LYSMD2	5.5	3.1	3.2	0.7	1.3	2.0	5.0	1.0	4.9
AGAP2-AS1	7.3	2.5	4.1	0.9	1.5	1.8	4.1	0.8	3.5
PHF2	8.0	3.0	3.7	0.6	1.3	1.6	3.9	0.7	3.9
PMF1-BGLAP	8.2	3.0	2.1	1.1	1.8	1.2	3.9	1.5	3.9
EVI5	7.6	2.5	3.4	0.3	1.4	1.8	5.2	0.7	3.8
CDC42EP5	7.4	4.3	5.3	1.6	1.2	2.1	2.2	0.4	2.1
VPRBP	7.2	2.5	3.2	0.4	1.1	1.2	5.9	0.8	4.3
SAAL1	6.9	2.7	3.3	0.4	0.8	1.6	5.5	0.8	4.6
DESI1	6.5	2.4	3.0	0.6	1.9	2.7	5.4	0.9	3.3
KCTD17	7.9	2.9	3.6	1.4	1.8	1.7	3.5	0.7	3.0
ZNF517	8.3	2.2	3.6	0.7	1.6	1.9	5.4	0.5	2.3
ARL14EP	7.2	2.1	3.3	0.3	1.0	1.6	6.5	0.5	4.0
POLR3G	4.7	1.4	2.6	0.3	1.2	1.1	8.4	1.1	5.7
ACBD5	8.4	2.6	3.6	0.4	1.2	1.5	4.8	0.4	3.5
LUZP1	7.4	2.3	3.4	0.3	1.1	1.6	5.4	0.7	4.3
AGGF1	7.1	3.0	4.3	0.5	1.5	1.6	4.3	0.6	3.7
SETDB1	8.4	2.8	4.3	0.5	1.2	1.8	3.7	0.6	3.2
C6orf136	6.0	2.4	3.0	1.1	1.5	2.3	5.0	0.9	4.3
C17orf59	7.8	2.5	3.7	1.0	1.5	2.1	4.2	0.8	3.1
MEGF9	9.3	3.0	4.5	0.9	1.1	1.6	3.2	0.5	2.3
TJP2	6.7	3.0	3.6	0.7	1.0	1.5	4.8	1.0	4.2
GPR137B	7.0	2.6	3.4	0.7	1.4	1.6	5.1	0.9	3.8
PWWP2A	7.7	2.4	3.2	0.1	1.2	1.6	5.4	0.9	4.0
PRC1	6.2	2.8	3.2	0.5	2.3	2.6	4.6	0.5	3.5
CLP1	7.9	3.1	3.2	0.4	1.3	1.1	4.9	0.6	4.0
EXOC2	7.2	2.8	3.5	0.4	1.2	1.6	4.9	0.9	4.1
USO1	6.3	2.5	3.3	0.2	1.1	1.6	5.4	0.7	5.3
SIRT1	6.6	3.1	2.9	0.5	1.0	1.2	6.2	0.7	4.2
APOM	6.7	2.5	2.5	0.4	0.5	1.4	7.1	0.9	4.4
ANGEL1	7.4	2.7	4.7	0.6	1.3	1.7	4.9	0.5	2.6
BTBD6	6.7	2.9	3.1	0.8	1.5	1.9	5.2	0.9	3.4
MTRR	6.1	2.3	2.9	0.5	1.2	1.7	5.8	0.9	4.9
NBPF10	7.9	2.1	3.7	0.5	1.6	1.8	4.7	0.6	3.4
VEZT	7.4	2.5	3.1	0.3	0.8	1.2	5.9	0.6	4.5
ATG7	8.0	2.4	3.9	0.3	1.4	1.5	5.2	0.5	3.2
RICTOR	7.8	2.5	3.5	0.2	0.8	1.2	5.4	0.6	4.4
PDCL	7.5	2.6	3.1	0.7	1.2	2.1	5.3	0.6	3.3
SEC22C	7.6	2.8	3.7	0.2	0.8	1.6	5.1	0.5	3.9
HYKK	8.8	1.6	3.5	0.6	1.1	1.3	6.3	0.5	2.7
LOC284950	8.6	1.7	3.7	0.5	1.1	1.1	6.5	0.4	2.7
HDAC8	8.4	2.8	3.6	0.4	1.0	1.8	3.9	0.8	3.6
SEC23IP	7.6	2.8	3.6	0.5	1.3	1.6	4.5	0.7	3.7
TNFSF18	4.7	1.8	2.2	0.0	0.7	0.8	8.4	1.1	6.7
CTNNBIP1	9.1	2.0	5.1	0.5	0.9	1.9	4.2	0.7	1.9
LOC101928228	9.3	1.9	4.0	0.3	0.8	1.4	5.2	0.6	2.9
ZDHHC14	6.2	2.5	3.0	1.1	1.3	1.7	5.2	1.2	4.3
DMAP1	6.9	2.6	3.5	0.6	1.4	2.0	5.2	0.9	3.3

LOC101928150	8.0	1.8	3.8	0.5	1.3	1.3	6.1	0.6	2.9
EAF1	5.9	2.9	3.5	0.5	1.1	1.5	4.7	0.8	5.4
GIGYF2	8.0	2.6	3.6	0.4	1.0	1.3	4.7	0.6	4.1
ACTR5	7.1	2.8	3.6	0.2	1.0	1.1	5.9	0.6	3.8
LOC101928401	8.9	1.9	3.6	0.4	0.9	1.2	6.5	0.4	2.7
ELF4	7.3	3.0	3.5	0.4	1.3	1.6	4.8	0.8	3.5
IDH1	8.5	3.4	5.2	0.3	0.9	1.2	3.6	0.6	2.5
ACVR1	8.1	2.5	3.2	0.3	1.3	1.5	4.9	0.7	3.7
COMMD10	5.6	2.8	3.8	0.4	1.5	1.6	5.5	0.6	4.4
PP13	7.9	1.9	3.9	0.7	1.4	1.4	5.2	0.5	3.0
UBE2F-SCLY	6.2	2.9	3.5	0.9	1.6	2.0	4.6	0.7	3.8
CHTF18	5.4	2.1	3.4	1.2	1.7	2.4	5.3	1.1	3.7
GMCL1	7.1	2.2	3.2	0.6	1.4	1.6	5.4	0.8	3.9
GGCT	5.0	2.6	2.3	0.2	0.7	1.3	7.2	0.8	6.1
RFFL	8.2	2.3	3.4	0.4	1.2	1.4	5.2	0.6	3.4
DIP2A	7.4	2.3	3.5	0.8	1.2	1.7	4.8	0.7	3.7
ERP44	6.4	2.5	3.4	0.3	1.3	1.7	5.0	0.9	4.7
RAB40C	7.1	2.0	3.2	1.0	1.1	2.0	5.4	1.0	3.4
DCBLD1	7.8	2.7	4.2	0.7	1.4	1.5	3.8	0.5	3.5
IGFBP1	5.7	2.5	2.6	2.0	2.3	4.2	3.7	0.5	2.6
AMMECR1L	7.0	2.1	2.6	0.5	1.2	1.2	5.0	1.1	5.4
MAP1LC3B2	13.3	1.6	3.7	0.2	0.7	0.7	3.5	1.1	1.5
MAPKBP1	7.0	2.3	3.7	0.7	1.3	1.5	5.3	0.7	3.6
SUSD5	7.5	2.9	3.7	0.5	1.1	1.6	4.6	0.7	3.4
FAM118B	6.9	2.1	3.5	0.2	1.0	1.4	5.9	0.9	4.2
ZDHHC12	7.4	2.9	2.9	1.2	1.4	2.7	4.1	0.7	2.9
TSNAX	6.4	2.6	4.0	0.2	1.2	1.7	5.2	0.6	4.2
TEX2	7.4	2.4	3.1	0.5	1.3	2.0	4.8	0.6	3.8
COX10	7.5	2.3	3.4	0.4	1.4	1.4	4.8	1.0	4.0
PCMTD1	7.1	2.4	3.8	0.3	0.9	1.6	5.6	0.5	3.9
CLU	9.6	4.3	5.5	0.6	1.3	1.2	1.7	0.5	1.3
TXNDC11	7.8	2.8	3.4	0.5	1.1	1.6	4.9	0.7	3.3
NAGPA	8.7	3.0	3.8	0.8	1.3	1.9	3.4	0.6	2.4
PXK	7.5	2.7	3.4	0.5	1.3	1.9	4.8	0.5	3.4
BRE	8.4	2.9	3.7	0.4	1.3	1.6	3.8	0.6	3.3
MED4	7.4	3.7	4.1	0.4	1.4	1.4	3.4	0.7	3.6
TVP23B	6.1	3.1	3.8	0.2	0.9	1.5	5.8	0.6	4.0
USP28	7.6	2.6	3.1	0.3	0.9	1.3	5.1	0.8	4.4
BMPR2	8.0	2.8	3.5	0.3	1.0	1.4	4.5	0.7	3.8
SLC14A1	4.6	2.2	3.2	1.0	3.5	4.5	3.6	0.5	2.9
GAB2	5.9	2.6	3.1	0.6	0.9	1.4	5.1	1.2	5.1
SNORD88C	9.8	2.7	1.6	0.5	2.0	1.9	4.0	0.6	2.9
PGM2	4.5	1.5	2.0	0.4	1.2	1.8	8.0	0.9	5.7
KIAA0355	7.2	2.3	3.4	0.8	1.6	1.9	4.0	0.9	3.9
ADNP2	9.2	2.5	3.9	0.6	1.1	1.7	3.3	0.5	3.0
ANKZF1	7.7	3.5	4.5	0.7	1.1	1.9	3.2	0.5	2.8
LYST	7.9	1.8	3.7	0.2	1.0	1.3	6.6	0.4	3.0
DTX3L	8.5	3.1	3.6	0.3	1.1	1.4	4.3	0.5	3.2
BCAS3	8.4	3.1	3.6	0.6	1.3	1.7	3.3	0.7	3.2
SCAMP1	7.3	3.1	4.5	0.3	1.3	1.7	3.6	0.6	3.5
LRSAM1	7.7	3.0	4.2	0.9	1.7	2.1	3.6	0.6	2.2
YPEL3	8.6	3.3	4.5	0.7	1.2	2.1	2.0	0.6	3.0
TICAM1	6.5	2.1	3.0	0.9	1.6	1.8	5.0	1.1	3.8
DENND2A	7.2	2.6	3.6	0.8	2.1	3.1	3.1	0.5	3.0
TMEM120A	6.9	3.5	4.4	0.8	1.5	2.5	2.7	0.7	2.8

TMEM180	6.7	3.0	3.5	0.8	1.4	1.4	4.9	1.0	3.3
VGLL3	5.5	1.9	2.3	1.0	1.8	2.8	3.6	1.3	5.6
THAP3	7.0	2.9	3.7	0.7	1.2	2.3	3.7	0.9	3.3
SRP54	6.6	2.6	3.4	0.2	0.8	1.5	5.5	0.6	4.6
TMEM140	7.2	1.8	3.3	1.0	1.8	2.1	3.6	0.6	4.5
S100A4	6.9	3.2	4.2	0.4	0.6	1.1	4.3	1.2	4.0
PPT2-EGFL8	5.8	2.6	3.4	0.7	1.4	1.5	5.0	0.8	4.5
NFS1	7.3	2.7	3.5	0.4	1.4	2.1	4.4	0.6	3.5
ALG1	7.6	2.1	2.8	0.6	1.5	2.0	5.5	0.5	3.1
CNST	6.2	2.2	2.9	0.2	1.1	1.4	5.7	0.7	5.4
MTOR	8.1	2.7	3.5	0.6	1.6	2.0	3.9	0.5	2.9
WARS2	6.2	2.4	4.4	0.6	1.3	2.0	4.3	0.6	4.0
EFNB1	7.3	3.4	3.8	0.8	1.4	2.0	3.0	0.7	3.3
FAM177A1	6.4	3.0	4.1	0.5	1.1	1.6	4.2	0.9	4.1
RNF113A	4.7	2.0	2.8	0.3	1.1	1.8	7.1	1.2	4.8
RIT1	7.7	2.9	4.0	0.2	1.1	1.1	4.6	0.7	3.5
KIAA1033	7.0	2.3	3.5	0.2	1.2	1.3	5.6	0.6	4.0
LINC00889	8.1	1.9	4.3	0.4	1.2	1.3	5.0	0.4	3.2
TOM1L1	6.6	2.5	3.5	0.3	1.0	1.3	5.2	0.6	4.7
B3GNTL1	6.4	2.7	3.6	0.8	1.6	1.8	5.1	0.8	3.0
KIAA1704	7.0	2.7	3.7	0.3	0.8	1.5	5.1	0.8	3.7
FAM167A	6.2	2.4	2.5	1.1	1.5	2.4	5.1	0.9	3.6
ERAP1	5.5	2.0	2.3	0.4	1.3	1.6	6.3	0.8	5.5
PMM1	7.4	3.2	3.3	0.8	2.0	1.7	4.0	0.5	2.8
NFYB	7.2	2.6	3.7	0.0	1.1	1.6	5.4	0.6	3.5
GLTSCR1	6.8	2.4	3.3	1.5	1.7	2.0	3.5	1.0	3.4
NEMF	6.6	2.4	3.3	0.3	1.2	1.6	5.6	0.7	4.0
FAM203A	5.3	2.6	2.6	1.0	2.0	2.5	5.6	0.9	3.2
UBL3	6.5	2.6	4.2	0.3	1.4	1.3	4.3	0.8	4.3
LYSMD3	6.2	3.5	3.8	0.1	1.1	1.2	3.8	0.8	5.1
MSL3	7.9	2.3	3.4	0.4	1.5	2.0	4.1	0.7	3.5
DUSP16	6.9	2.4	2.9	0.2	0.9	1.2	6.0	0.8	4.4
TMEM65	6.7	2.3	4.0	1.0	1.6	2.1	3.5	0.8	3.6
LOC440173	8.6	1.5	3.8	1.1	1.3	1.6	4.7	0.3	2.7
TYW3	7.1	2.7	3.8	0.3	0.9	1.2	4.9	0.6	4.1
ALG12	6.9	2.5	3.4	0.6	1.4	2.1	4.5	0.9	3.3
TDP1	6.3	2.4	3.2	0.2	1.0	1.3	5.7	0.8	4.8
ABCC10	8.0	2.8	3.8	0.6	1.3	1.5	4.3	0.5	2.7
FBXO38	8.3	3.1	3.5	0.3	1.0	1.2	4.1	0.6	3.6
EIF2D	7.0	3.2	3.9	0.8	2.1	2.4	3.4	0.5	2.2
BOLA2B	4.9	2.5	3.7	1.1	1.3	1.8	5.3	0.8	4.2
RBM6	6.9	2.6	3.3	0.3	1.1	1.3	5.3	0.6	4.2
LOC101929494	5.4	3.5	4.8	0.4	1.1	2.0	3.0	0.6	4.9
NET1	6.5	2.3	3.2	0.4	1.5	1.6	4.8	1.1	4.1
INO80	7.3	2.5	3.1	0.4	1.0	1.7	4.8	0.7	4.1
PAK1IP1	4.8	1.7	3.3	0.4	1.0	1.4	6.4	0.7	5.9
AGPAT4	8.7	3.1	4.8	0.3	1.0	1.1	3.6	0.5	2.3
NFATC2IP	6.6	2.0	3.4	0.7	1.3	2.0	5.3	0.8	3.4
PPHLN1	7.3	3.0	3.6	0.4	1.2	1.5	4.2	0.5	3.9
MRS2	6.7	2.5	3.9	0.3	0.8	1.1	6.1	0.6	3.6
PNPO	6.5	2.6	3.1	0.8	1.6	2.3	5.1	0.6	3.0
ALS2	9.2	3.2	3.3	0.2	0.8	1.0	4.8	0.5	2.5
AVL9	7.4	2.4	3.7	0.3	0.8	1.2	4.8	0.7	4.3
CPE	9.3	3.4	4.2	0.5	1.4	1.6	2.7	0.3	2.0
ACOX1	7.4	2.8	3.6	0.5	1.2	1.9	4.9	0.4	2.8

ANO6	6.8	2.4	3.3	0.2	1.1	1.4	5.3	0.8	4.2
PTPRN2	5.7	2.1	2.7	1.3	2.3	3.1	4.5	0.8	3.1
FOXF2	4.3	3.0	2.6	2.1	2.1	1.9	4.1	1.8	3.5
XYLT2	7.7	3.0	4.1	0.9	1.5	2.1	2.9	0.5	2.8
NAE1	7.0	2.3	3.2	0.2	1.2	1.4	5.0	0.6	4.6
LIG3	7.1	2.4	3.2	0.5	1.4	1.8	4.5	0.6	3.9
LRRC8E	6.8	2.6	3.3	0.7	1.2	1.8	4.4	1.0	3.5
ZNF250	6.5	2.3	3.6	0.6	1.4	1.9	5.1	0.7	3.5
PTS	7.3	2.7	3.3	0.2	1.2	1.4	4.7	0.5	4.1
LOC101929357	5.7	2.2	3.2	0.8	1.6	1.6	5.7	1.0	3.5
CCDC14	7.6	2.6	4.0	0.4	1.4	1.9	3.6	0.6	3.4
ZNF48	5.9	2.0	2.6	0.7	1.1	1.5	6.5	0.8	4.3
NAV1	8.6	2.7	3.9	0.8	1.4	1.9	3.0	0.6	2.4
KDM4A-AS1	7.6	1.9	3.9	0.4	1.0	1.3	6.2	0.5	2.6
PARP2	6.9	2.4	3.2	0.3	1.1	1.3	5.2	0.7	4.4
POLR1B	6.2	2.1	2.8	0.3	1.3	1.3	6.1	0.8	4.4
SUFU	7.2	2.1	2.8	0.7	1.4	1.7	5.0	0.8	3.7
MICAL3	7.3	2.0	3.7	0.8	1.7	2.2	4.5	0.5	2.8
AAK1	7.3	2.5	3.7	0.7	1.6	1.8	3.5	0.7	3.4
RBM28	6.2	1.9	2.7	0.4	1.0	1.3	6.4	0.7	4.8
ARHGEF10	6.5	2.1	3.0	0.7	1.3	1.6	4.9	0.8	4.2
SNORD21	6.1	2.0	3.1	1.1	1.1	1.0	4.7	1.3	4.9
LURAP1L	5.8	3.2	3.9	1.0	1.5	1.7	4.6	0.5	3.1
UAP1L1	7.1	3.1	3.8	0.9	1.3	1.8	4.3	0.6	2.4
PNMAL1	6.0	2.1	2.8	0.4	1.1	1.4	6.5	0.9	4.0
SAMHD1	6.9	2.4	3.1	0.3	0.7	1.3	5.7	0.8	4.1
SLC15A4	5.2	3.7	3.5	1.0	1.6	1.7	4.8	1.0	2.8
SQRDL	7.3	3.1	4.4	0.4	1.3	1.9	4.1	0.3	2.5
ASCC1	7.3	2.4	3.5	0.3	0.9	1.2	5.4	0.6	3.7
KIAA0947	6.2	2.2	2.6	0.4	0.9	1.3	5.8	0.8	4.9
PRKAR1B	5.9	3.0	3.2	0.6	1.2	1.4	5.1	0.8	3.9
URB1	7.1	2.3	3.3	0.7	1.5	2.0	4.4	0.6	3.3
FBXL5	7.2	2.7	4.2	0.2	1.1	1.3	4.6	0.5	3.5
CTPS1	6.0	2.0	3.0	0.3	1.4	1.6	5.5	0.7	4.6
SERHL2	6.2	1.8	3.1	0.7	1.4	2.0	5.7	0.7	3.6
EPHB2	7.3	3.0	3.4	0.5	1.1	1.4	4.0	0.9	3.4
APEX2	5.5	2.4	2.2	0.7	1.4	2.2	5.4	0.9	4.4
PSMG3	6.8	2.9	3.2	0.3	1.0	1.8	5.0	1.0	3.2
COL4A3BP	7.0	2.4	3.7	0.5	0.8	1.4	5.3	0.6	3.4
CARD10	6.2	2.5	3.1	1.1	1.7	2.3	4.6	0.8	2.9
FMNL3	6.1	2.4	3.3	0.7	1.4	1.3	5.2	0.8	3.9
TBC1D8	8.0	2.4	3.1	0.8	2.1	2.2	3.8	0.4	2.3
KLHL18	6.5	2.2	2.9	0.6	1.0	1.7	5.3	0.9	3.9
C21orf2	7.0	2.6	3.4	1.5	1.4	1.8	4.5	0.8	2.1
RTCA	6.4	2.7	2.7	0.2	1.1	0.9	6.1	0.6	4.3
STK11IP	8.8	3.2	4.7	0.5	0.8	1.3	2.9	0.6	2.3
LOC101929372	8.4	1.7	3.1	0.6	1.0	1.1	6.2	0.5	2.5
TVP23C-CDRT4	6.7	1.9	3.1	0.3	0.8	1.2	6.2	0.8	4.1
ZZEF1	6.8	2.3	3.5	0.5	1.3	1.4	4.7	0.8	3.7
MRPS9	6.7	2.8	3.1	0.5	1.4	1.8	4.3	0.6	3.9
AHCTF1	6.0	2.0	2.9	0.2	0.9	1.1	6.3	0.8	4.8
MRPL42	6.7	4.1	4.3	0.2	0.8	1.4	4.3	0.3	3.1
TMBIM4	5.6	2.6	3.2	0.7	1.3	1.7	4.8	0.9	4.3
KIAA1429	7.5	2.6	3.3	0.2	1.0	1.4	4.5	0.7	3.8
BMPR1A	7.4	2.3	3.3	0.2	1.0	1.0	5.4	0.5	3.9

LINC00913	7.0	2.0	4.2	0.5	1.2	1.6	4.4	0.7	3.4
LRP6	6.6	2.1	3.1	0.3	1.1	1.3	5.5	0.7	4.3
FYCO1	7.5	2.6	3.4	0.6	1.0	1.3	4.3	0.7	3.6
PPP2R1B	7.2	2.2	3.3	0.3	1.0	1.5	5.1	1.0	3.4
SCARNA1	6.0	2.8	4.0	0.4	1.3	1.5	4.8	0.3	3.8
NNT	6.6	2.6	4.0	0.3	1.2	1.4	4.6	0.6	3.6
LINC00998	7.8	3.4	4.4	0.2	1.0	1.0	3.6	0.5	3.0
JRK	6.4	2.8	3.0	0.6	1.9	1.8	5.1	0.6	2.8
BBS1	7.3	2.3	3.2	1.2	2.1	3.0	3.3	0.2	2.4
NTPCR	7.0	2.8	3.2	0.6	1.8	1.4	4.2	0.7	3.2
HPRT1	6.2	2.1	2.4	0.1	0.7	1.1	7.0	0.7	4.6
PEAR1	6.2	2.5	2.8	0.6	1.1	1.7	5.1	0.9	4.2
CDC6	4.6	1.7	2.1	0.4	1.0	1.5	7.1	0.9	5.7
GLT8D1	7.2	2.4	3.7	0.3	1.3	1.6	4.3	0.7	3.6
BTG3	6.8	2.5	3.0	0.3	1.0	1.5	4.9	0.6	4.4
C12orf45	6.6	2.7	3.7	0.3	0.6	1.1	5.4	0.9	3.6
IL31RA	5.1	1.5	2.3	0.3	1.0	1.2	7.8	1.0	4.7
ZNF346	5.7	2.0	3.2	0.5	1.8	2.4	5.6	0.7	3.2
SOX13	6.9	2.8	3.3	0.8	1.3	1.8	3.9	0.8	3.3
EMC2	6.9	2.9	3.7	0.3	1.0	1.3	4.8	0.6	3.4
LDOC1L	7.1	2.7	3.1	1.0	1.5	2.9	3.2	0.8	2.4
LOC101929010	8.2	1.7	3.4	0.6	1.0	1.1	6.0	0.4	2.4
DCP1A	6.6	2.2	3.2	0.5	1.0	1.2	4.1	1.0	5.0
R3HCC1	6.0	2.3	3.0	0.5	1.1	1.8	5.4	0.8	4.0
MIR143HG	8.0	1.7	3.6	0.4	1.0	1.0	6.1	0.5	2.5
EYA4	6.7	2.6	3.4	0.4	1.1	1.7	3.6	0.9	4.5
SLC16A4	7.7	1.6	3.5	0.4	0.8	1.0	6.5	0.5	2.9
IFT140	7.6	3.0	4.1	0.7	1.3	1.6	3.8	0.5	2.2
LINC00907	7.8	1.9	3.8	0.4	0.8	1.0	5.8	0.4	2.8
RUNX2	5.9	2.1	2.7	1.7	2.7	3.8	3.1	0.6	2.4
CASKIN2	5.9	2.0	3.0	0.9	1.5	1.9	5.0	0.8	3.8
MON1A	6.0	2.0	2.9	0.5	1.4	1.7	5.3	1.2	3.9
COMMD1	6.4	3.1	4.5	0.2	1.5	1.3	4.0	0.3	3.4
IDE	7.7	2.9	4.0	0.2	1.2	1.3	4.3	0.5	2.7
BOD1L1	6.7	2.1	2.8	0.4	1.1	1.3	5.4	0.7	4.3
SCAMP5	8.0	3.0	3.8	0.5	1.1	1.9	3.4	0.4	2.6
HARS2	6.0	2.5	3.1	0.2	0.6	1.3	5.7	0.8	4.4
EVPL	7.4	3.1	4.0	0.8	1.3	1.5	3.5	0.6	2.5
SETD6	5.5	1.9	2.7	0.8	1.0	1.5	6.4	0.9	4.0
VPS41	8.2	2.9	3.6	0.3	1.0	1.3	4.1	0.4	3.0
PPP2R5B	6.4	2.9	4.2	0.6	1.2	1.7	4.0	0.7	3.1
SNORA77	8.6	3.0	2.1	0.3	0.6	2.0	5.7	0.4	2.0
OTUD6B	5.5	1.8	2.7	0.3	1.0	1.2	6.6	0.7	4.9
ARID5A	6.9	2.7	3.5	1.0	1.6	2.1	3.3	0.9	2.8
BTAF1	6.5	2.1	3.1	0.3	0.9	1.6	5.6	0.7	4.0
HSD17B14	8.0	3.7	4.7	0.6	1.3	1.7	2.4	0.5	1.8
NAA15	5.9	2.0	2.6	0.3	0.7	1.1	6.3	0.6	5.2
PI3	11.1	4.2	6.5	0.1	0.3	0.2	1.3	0.3	0.7
FLJ37448	8.6	1.6	3.7	0.4	0.9	1.1	5.5	0.6	2.4
REV1	7.1	2.4	3.6	0.3	0.9	1.4	4.4	0.7	4.0
TM7SF2	7.6	3.4	4.5	0.7	1.2	1.5	2.8	0.5	2.5
ZNF783	6.6	2.3	3.6	0.5	1.4	1.8	5.0	0.6	2.8
C3orf17	5.9	2.9	3.5	0.6	1.4	1.5	3.9	0.9	4.1
PPIC	7.9	3.0	4.0	0.4	1.1	1.3	4.4	0.3	2.3
LOC101929007	8.2	1.5	3.1	0.5	1.0	1.0	6.5	0.5	2.4

PANK4	7.8	2.6	3.9	0.7	1.4	1.7	3.8	0.4	2.5
GTF2E1	6.2	2.3	3.4	0.3	1.1	1.2	5.1	0.8	4.3
FAM216A	4.3	1.6	2.7	0.9	1.9	2.9	5.4	0.8	4.0
HS6ST1	7.9	2.6	3.4	0.9	1.2	1.3	3.9	0.7	2.7
HSD17B11	7.0	3.1	3.9	0.1	0.7	1.0	5.1	0.6	3.0
LOC101929678	6.0	3.1	3.3	1.0	1.8	2.8	2.8	0.5	3.2
SKIL	5.7	1.7	2.6	0.1	0.6	1.0	6.7	0.7	5.6
MPHOSPH6	5.7	1.9	2.9	0.3	1.0	1.8	5.4	0.7	4.8
TNFSF12	7.1	2.6	3.7	0.7	1.7	2.5	3.4	0.5	2.4
BLOC1S3	8.2	2.4	3.4	0.6	1.4	1.5	4.0	0.5	2.5
LEPROTL1	7.1	2.5	3.9	0.2	1.1	1.5	4.1	0.7	3.6
LOC100130938	5.7	2.7	3.3	0.4	1.0	1.5	5.7	0.6	3.7
CDCA5	5.2	2.0	2.6	0.4	1.7	1.6	5.8	1.3	4.0
LINC00963	7.6	2.0	3.7	0.5	0.8	1.2	5.7	0.5	2.7
ADAMTSL5	6.0	2.5	3.0	1.1	1.6	2.1	4.7	0.7	2.8
SDPR	5.9	2.4	3.0	0.6	1.4	2.0	4.4	1.0	3.9
ARMC7	5.6	2.7	3.0	1.0	1.3	1.4	5.0	1.3	3.2
TMEM71	9.0	2.9	5.3	0.4	0.9	1.1	2.5	0.2	2.3
MPPE1	7.8	1.8	3.5	0.3	0.9	1.0	5.9	0.5	2.7
CBX2	5.3	1.5	2.7	0.8	0.9	1.6	5.9	1.4	4.4
C10orf55	5.0	3.0	3.9	0.4	0.9	2.4	4.3	0.6	4.1
PVRL1	6.2	2.7	3.5	0.8	1.1	1.7	4.0	1.0	3.5
POLR2D	7.0	2.1	4.6	0.7	0.9	2.9	2.2	0.9	3.3
CSRNP1	5.8	1.9	3.0	0.4	0.6	1.0	5.9	1.4	4.4
PDLIM4	7.1	3.3	3.9	1.4	1.8	2.4	2.2	0.6	1.8
TMF1	6.4	2.5	3.2	0.2	0.9	1.3	5.1	0.7	4.1
IPO13	6.8	2.8	3.6	0.6	1.5	1.7	4.0	0.8	2.7
LOC101927582	8.3	1.4	3.3	0.5	0.8	1.1	6.1	0.4	2.5
SNORD99	3.2	1.0	1.3	2.3	0.5	1.8	5.7	1.3	7.4
NRROS	6.1	2.4	3.2	0.7	1.2	1.6	4.7	0.6	4.0
RNF217	6.5	2.4	3.4	0.6	1.1	1.5	4.6	0.7	3.9
CXorf40A	6.8	2.4	3.3	0.9	1.2	1.0	4.7	0.5	3.4
TMEM189-UBE2V1	9.1	4.0	3.5	0.1	0.5	2.2	3.9	0.4	0.8
STAT1	6.4	2.3	3.1	0.3	1.2	1.7	5.4	0.7	3.3
RIPK2	5.4	1.9	3.1	0.7	1.0	1.2	5.4	1.2	4.5
TBC1D24	6.0	2.1	3.1	0.7	1.4	1.5	5.2	0.8	3.6
ASB7	6.1	2.6	3.2	0.4	0.7	1.1	5.3	0.9	4.3
RBMX2	6.4	2.7	3.4	0.6	1.1	1.8	3.6	0.8	4.1
SLC43A1	6.9	2.2	3.6	0.7	1.6	2.4	3.8	0.4	2.8
CHD1	5.9	1.7	2.6	0.1	0.8	1.0	6.1	0.6	5.5
DYM	7.2	2.8	2.9	0.2	1.0	1.9	3.8	0.7	3.8
NTAN1	6.8	3.1	3.5	0.4	1.0	1.6	4.2	0.6	3.3
EDEM3	6.7	1.9	3.1	0.2	1.0	1.1	5.5	0.6	4.3
GCFC2	7.9	1.9	4.4	0.3	0.9	1.1	4.4	0.4	3.1
TBC1D22A	7.3	2.2	3.3	0.6	1.2	1.8	4.1	0.6	3.2
CBY1	5.0	3.2	2.9	0.3	1.7	1.8	4.9	1.1	3.6
ERLIN2	7.5	2.9	3.5	0.6	1.6	2.3	3.0	0.3	2.7
ARHGAP22	5.4	1.9	2.5	0.9	1.8	2.2	5.0	0.8	3.8
TIMM8A	5.9	2.2	3.0	0.5	1.2	1.1	5.2	0.4	4.7
SNORD57	5.5	3.2	1.5	1.4	2.0	1.6	2.9	0.4	5.9
SH3RF3	6.7	2.5	3.3	0.9	1.5	1.9	3.5	0.7	3.3
TADA2A	6.6	2.3	3.3	0.4	1.1	1.8	4.3	0.5	3.9
ARMC9	6.1	2.2	2.7	0.8	2.0	2.2	4.6	0.5	3.2
MTHFSD	7.2	2.4	3.0	0.9	2.1	2.1	3.3	0.6	2.7
CNOT2	7.2	2.4	3.3	0.3	0.9	1.3	4.2	0.6	4.0

GATA2	6.5	2.8	3.0	0.7	0.9	1.3	4.1	1.0	4.1
SHANK1	6.7	2.4	3.1	1.1	1.6	2.3	3.8	0.6	2.8
NAPRT1	7.2	2.5	4.5	0.6	1.1	2.0	3.2	0.5	2.7
CMAS	8.6	2.8	3.5	0.4	1.0	1.3	3.6	0.6	2.6
USP3	7.2	2.5	3.0	0.4	1.0	0.9	4.7	0.7	3.9
CHST10	6.2	2.1	2.4	0.4	1.5	1.5	5.5	0.7	4.0
ITGA1	9.5	3.1	4.5	0.2	0.7	0.8	3.0	0.4	2.2
NPIPA5	7.6	2.3	3.4	0.4	1.3	1.7	3.7	0.7	3.2
MIB1	8.0	2.4	3.3	0.3	1.1	1.3	4.4	0.5	2.9
NRSN2	7.2	2.6	3.0	1.0	2.0	2.8	3.0	0.5	2.2
FAR1	6.1	2.0	2.9	0.2	0.8	1.0	6.4	0.6	4.3
ABHD5	6.3	2.2	3.2	0.3	0.6	1.3	5.1	0.8	4.4
CDK5	6.2	2.8	2.9	0.8	2.0	2.8	3.1	0.6	3.0
LOC101927408	7.4	1.9	3.3	0.9	0.8	2.1	3.6	0.8	3.3
RNF138	6.1	2.0	3.2	0.3	1.4	0.9	5.0	0.8	4.5
LINC00472	6.1	1.9	3.4	0.7	1.2	1.6	5.3	0.7	3.4
RHOT1	6.6	2.3	3.4	0.2	0.9	1.4	4.7	0.6	4.1
LOC100507002	7.5	3.2	5.0	0.7	1.5	1.6	2.3	0.3	2.1
SQLE	7.1	2.6	3.8	0.2	0.3	0.7	4.9	0.7	4.0
DDX52	6.5	2.1	2.9	0.2	1.1	1.4	5.4	0.5	4.0
SEC24B	6.5	2.4	3.3	0.3	1.1	1.5	4.4	0.6	4.0
BST2	8.7	2.4	3.7	0.5	1.0	1.3	3.5	0.6	2.5
KNSTRN	5.0	2.1	3.5	0.6	1.1	2.2	4.6	1.1	4.1
LOC101927860	8.0	1.6	3.3	0.5	1.0	0.8	5.9	0.5	2.7
SGCB	6.0	3.2	3.3	0.2	1.3	2.0	3.4	0.7	3.9
SYNGR1	7.2	2.2	3.6	1.2	2.2	2.4	2.9	0.4	2.0
ZNF668	5.4	2.1	3.0	0.9	1.4	1.4	5.3	1.0	3.6
FAM166A	5.9	2.8	3.0	1.1	1.8	2.2	2.8	1.1	3.5
FAM208B	6.2	1.9	2.7	0.2	0.9	1.1	5.6	0.7	4.7
HAT1	6.0	2.6	2.9	0.2	1.1	1.6	4.7	0.7	4.4
MICB	6.7	2.4	2.9	0.3	0.9	1.7	4.2	0.9	4.1
RAB3GAP2	6.5	2.2	2.9	0.3	1.0	0.9	5.6	0.6	4.2
LMNB1	5.6	1.9	2.9	0.6	1.8	1.9	4.8	0.9	3.6
MDM2	5.6	2.0	2.7	0.1	0.5	1.3	5.7	0.9	5.3
PTPRG-AS1	8.4	1.5	3.5	0.5	0.9	1.0	5.5	0.4	2.5
CAMSAP2	6.7	1.9	2.5	0.2	0.9	1.3	5.3	0.7	4.7
LOC101928281	3.7	1.4	1.7	0.3	0.9	1.2	8.3	1.0	5.5
RPAP1	6.8	2.6	3.5	0.6	1.3	1.9	4.0	0.6	2.8
SDE2	7.3	2.1	3.0	0.4	1.0	1.0	5.2	0.6	3.4
PRKD3	6.4	2.4	3.1	0.2	1.0	1.3	4.8	0.7	4.1
ZHX1	7.1	2.5	3.4	0.2	0.9	1.3	4.6	0.6	3.6
SBF2	6.8	2.2	3.0	0.3	1.0	1.4	4.9	0.7	3.7
E2F3	5.7	1.6	2.2	0.3	1.3	1.5	5.6	0.6	5.0
AKR1C1	9.9	3.9	6.0	0.4	1.0	0.9	0.9	0.1	0.9
HEATR6	7.6	2.7	3.4	0.4	1.0	1.5	3.9	0.7	3.0
DFNB31	7.7	3.5	4.2	0.7	0.9	1.6	2.6	0.6	2.3
TSPAN9	6.4	2.8	4.5	0.6	1.7	1.6	2.9	0.3	3.1
GFOD1	4.1	1.4	2.1	1.0	1.0	1.5	6.3	1.4	5.2
LOC100128242	7.9	2.1	3.5	0.6	0.9	1.4	3.7	0.8	3.0
CTDSPL	7.0	2.4	3.2	0.6	1.0	1.6	4.8	0.4	3.0
THG1L	6.6	2.4	3.3	0.5	1.2	1.7	4.0	0.7	3.4
FIGN	6.7	2.2	3.1	0.5	1.2	1.7	4.0	0.9	3.7
DDHD1	7.4	2.3	3.1	0.7	1.2	1.5	3.9	0.5	3.3
REEP2	7.7	3.6	4.1	0.6	1.2	1.6	2.6	0.4	2.2
RARG	7.1	2.8	3.8	0.7	1.5	2.5	2.9	0.5	2.1

LOC101927842	8.1	1.6	3.2	0.4	0.9	1.0	5.9	0.3	2.5
GPR126	6.3	2.2	2.9	0.2	0.8	1.0	5.5	0.6	4.4
QTRTD1	6.8	2.0	3.1	0.3	0.6	1.0	5.8	0.7	3.7
ZNF710	5.9	2.2	2.8	1.0	1.0	1.6	4.7	0.8	3.9
ESF1	5.8	2.4	2.8	0.2	0.9	1.2	5.2	0.7	4.8
PTER	6.0	1.7	2.7	0.3	1.0	1.3	6.2	0.5	4.2
TBCE	5.5	2.3	3.2	0.2	1.0	1.6	4.9	0.7	4.5
SLC19A2	5.9	2.1	2.9	0.4	0.8	1.1	5.3	0.9	4.4
TRIM16	7.1	2.2	3.5	0.5	1.2	1.4	4.4	0.4	3.2
UCN	6.3	2.8	2.8	0.9	1.0	2.9	4.5	1.0	1.8
MFSD1	7.5	2.5	3.7	0.4	1.0	1.5	4.0	0.6	2.6
MCU	6.5	2.4	3.1	0.5	1.1	1.2	4.7	0.8	3.7
CLCN4	5.3	2.2	3.1	0.5	0.9	1.7	5.2	1.0	4.0
SLC4A1AP	6.6	2.3	3.6	0.5	0.9	1.5	4.1	0.8	3.6
CBWD2	6.9	2.3	3.0	0.6	0.7	1.2	4.7	0.8	3.7
COG2	6.7	2.3	3.1	0.3	0.9	1.3	5.1	0.6	3.6
NIF3L1	5.5	2.4	3.5	0.3	1.1	1.5	5.8	0.4	3.4
HEATR1	5.9	1.9	2.6	0.3	1.0	1.2	5.9	0.7	4.4
FAM220A	6.8	1.9	2.8	0.3	0.8	1.2	5.3	0.5	4.3
DPH3	6.4	2.8	3.3	0.2	1.0	1.7	4.0	0.8	3.7
SNORD14D	5.7	2.2	2.2	0.5	1.6	3.4	5.2	0.3	2.7
CCDC82	7.0	2.2	3.2	0.1	0.7	1.0	5.3	0.5	4.0
MTRF1L	6.8	2.3	3.2	0.3	0.9	1.5	4.4	0.5	4.0
ATAD2	5.3	2.0	2.1	0.3	1.0	1.3	6.4	0.8	4.6
METTL3	5.7	2.1	3.1	0.3	1.1	1.6	5.4	0.8	3.8
RIOK1	5.4	2.1	3.0	0.2	1.0	1.1	5.7	0.8	4.6
LOC100507501	8.1	1.5	3.6	0.5	0.8	1.1	5.2	0.4	2.6
RWDD4	6.7	3.2	3.3	0.3	1.2	1.5	2.8	0.6	4.2
LIN52	5.6	2.3	3.9	0.3	1.0	1.3	6.0	0.3	3.2
PCMTD2	6.1	2.6	3.1	0.3	1.0	1.8	4.6	0.6	3.7
LOC101928485	7.4	3.2	4.4	0.8	1.1	1.5	2.1	0.9	2.5
C17orf80	6.0	1.9	2.8	0.2	0.8	1.2	4.8	0.7	5.2
TMEM64	6.7	2.3	3.5	0.6	1.1	1.1	4.5	0.6	3.4
SPIRE2	5.6	2.3	3.2	0.8	1.3	1.4	5.1	0.9	3.2
MSMP	8.3	3.4	3.6	0.4	0.9	1.0	3.4	0.6	2.1
RC3H1	6.7	2.3	2.7	0.4	1.2	1.4	4.4	0.7	3.9
LINC01021	7.8	1.7	3.4	0.6	0.9	0.9	5.7	0.5	2.2
HYLS1	5.8	2.3	2.9	0.3	1.3	1.8	4.6	0.5	4.3
SYNRG	7.3	2.2	3.1	0.3	0.9	1.2	4.5	0.6	3.4
ANKS6	7.7	2.5	2.8	0.8	1.5	1.9	3.4	0.7	2.5
CEP120	6.9	2.1	3.0	0.2	0.9	1.2	4.9	0.8	3.6
SLC37A3	7.3	2.1	3.8	0.2	1.1	1.2	5.0	0.5	2.6
CCHCR1	6.3	2.4	3.7	0.6	1.1	1.6	4.2	0.6	3.1
TMCO6	4.9	2.5	3.2	0.8	1.4	1.8	4.9	0.7	3.6
ANO8	7.7	3.1	4.3	0.8	1.4	1.7	2.6	0.4	1.8
FICD	5.8	1.9	2.5	0.5	1.2	1.2	5.4	0.8	4.3
ATF3	4.0	1.3	2.9	0.1	0.9	1.0	7.8	0.8	4.8
TGFBR3	8.4	2.8	3.8	0.3	0.5	0.9	3.7	0.6	2.7
CHST2	6.6	2.5	2.9	0.8	1.4	1.9	4.3	0.9	2.3
LOC101928152	7.3	1.0	2.9	0.4	1.1	1.5	6.6	0.4	2.5
PEX13	7.2	2.5	2.9	0.3	0.9	1.4	4.6	0.7	3.1
KIAA1147	8.3	3.1	3.9	0.4	1.1	0.9	3.7	0.4	1.9
RFWD2	6.3	2.0	3.2	0.4	1.0	1.6	4.8	0.6	3.6
LOC401098	7.7	1.7	3.4	0.2	0.8	0.9	6.0	0.4	2.6
SLC12A2	6.9	2.3	3.2	0.4	0.8	0.9	5.0	0.6	3.6

TMEM199	6.2	2.3	3.5	0.4	1.0	1.5	4.2	0.9	3.6
YARS2	5.8	2.4	3.6	0.4	0.7	1.0	5.6	0.5	3.6
ZNF672	5.8	2.8	3.1	1.0	1.3	2.2	3.7	0.7	2.8
AURKB	3.9	1.8	2.8	0.8	2.2	2.8	5.0	0.8	3.6
ZNF142	5.8	1.8	2.7	0.7	1.3	1.8	4.6	0.8	4.2
GK5	7.7	1.5	3.3	0.4	0.9	1.1	5.6	0.5	2.6
MED20	6.8	2.4	3.2	0.3	1.0	1.5	4.8	0.7	2.7
CMTM6	6.3	3.0	3.1	0.4	0.7	1.4	5.0	0.6	3.1
RCOR1	4.8	2.4	2.9	0.6	1.3	1.5	4.6	1.0	4.3
NUP107	6.2	2.1	2.7	0.2	0.7	1.1	6.0	0.6	4.0
OSBPL9	7.1	2.6	3.3	0.3	1.0	1.4	4.0	0.7	3.1
USP25	6.5	2.6	2.9	0.3	0.9	1.3	4.3	0.6	4.2
LUCAT1	7.2	2.7	3.6	0.6	0.8	1.5	4.8	0.2	2.2
LOC101927954	7.5	1.5	3.2	0.4	1.0	0.9	6.0	0.5	2.5
ZNF674-AS1	7.6	1.9	3.4	0.4	0.7	1.0	5.8	0.4	2.4
QDPR	6.0	2.3	3.1	0.6	1.4	1.7	5.0	0.4	3.0
NUP155	6.5	2.4	3.2	0.3	1.0	1.4	4.5	0.5	3.7
ZXDB	5.3	1.9	2.4	0.6	1.3	1.4	5.3	1.1	4.4
BAG5	6.3	2.6	2.9	0.2	0.8	1.2	5.1	0.7	3.7
ONECUT2	4.3	2.8	3.5	3.4	1.4	0.9	2.9	2.0	2.4
ALAD	7.6	2.2	3.3	0.6	1.4	1.8	3.9	0.4	2.2
ATAD3A	6.2	1.8	2.5	1.1	1.8	2.5	4.2	0.6	2.8
RPP14	6.4	1.6	3.0	0.4	0.8	1.0	6.1	0.6	3.6
LOC648691	8.0	1.4	3.2	0.4	0.7	0.9	6.1	0.5	2.3
MDFIC	6.5	2.2	3.3	0.7	1.2	1.7	4.8	0.7	2.5
LINC00261	8.3	3.4	4.3	0.3	0.9	1.1	2.8	0.4	2.0
SPRY1	6.8	2.4	3.0	0.4	1.6	1.8	3.3	0.6	3.7
TMEM141	7.1	2.3	3.2	0.7	1.3	2.1	4.1	0.4	2.3
CDC23	6.5	2.4	3.2	0.3	0.9	1.0	5.0	0.6	3.7
C3orf38	5.4	2.3	3.0	0.7	1.6	1.4	4.4	0.6	4.0
DNAAF1	7.7	1.6	3.4	0.4	1.0	0.9	5.9	0.5	2.1
NUDT9	7.0	2.3	3.2	0.2	1.0	1.1	4.5	0.7	3.4
NUP54	5.9	2.3	3.2	0.3	1.1	1.7	4.1	0.7	4.2
TWISTNB	5.5	2.1	3.1	0.5	1.2	1.4	4.6	0.9	4.1
LOC101928231	7.3	1.8	2.8	0.7	1.2	1.1	4.9	0.6	3.0
SOBP	7.0	2.6	3.5	0.8	1.6	1.6	2.7	0.8	2.7
COQ6	7.6	2.5	4.2	0.5	1.6	2.0	2.6	0.3	2.0
FAM208A	6.8	2.4	3.7	0.4	0.9	1.3	4.1	0.6	3.2
HCG11	7.3	1.6	3.2	0.4	0.9	1.1	5.9	0.4	2.6
LOC101928454	6.8	2.6	2.9	0.6	1.3	2.2	4.0	0.5	2.5
LY6K	6.1	2.9	2.7	1.1	1.5	2.2	3.6	0.8	2.5
TBC1D22B	6.5	2.4	3.2	0.4	1.0	1.4	4.1	0.8	3.6
RBMXL1	6.5	2.2	2.8	0.3	1.3	1.7	4.4	0.9	3.3
MMACHC	4.4	2.1	2.8	0.7	1.6	2.5	4.1	1.0	4.2
WDR61	7.6	3.1	3.8	0.4	1.2	1.7	3.7	0.2	1.7
LLGL2	9.5	4.1	4.7	0.2	0.4	0.7	2.1	0.3	1.5
UPK3BL	6.8	1.8	3.1	0.7	1.2	2.2	4.2	0.6	2.7
NPHP4	7.6	2.8	3.7	0.7	1.4	1.7	3.1	0.4	2.0
CSNK1G1	7.4	1.7	3.4	0.3	0.9	1.1	5.1	0.5	2.9
TMEM92	3.9	2.4	2.1	0.7	1.5	1.9	5.3	1.2	4.4
TMEM128	6.5	3.1	3.4	0.7	1.6	1.9	3.5	0.5	2.3
CLN6	4.1	1.8	1.9	1.0	2.1	2.4	4.6	1.0	4.4
LOC101927060	6.3	2.3	2.5	1.1	1.6	2.1	4.5	0.6	2.2
MGC27345	7.2	1.6	3.3	0.4	1.1	1.4	4.6	0.5	3.2
PLD6	6.6	2.2	2.6	0.6	0.9	1.4	4.4	0.7	3.8

KAT6B	5.5	2.1	2.8	0.5	1.1	1.5	4.5	0.8	4.5
LOC100506469	7.2	1.9	3.4	0.5	1.0	1.4	5.1	0.4	2.4
SOCS2	5.9	2.4	3.0	0.4	0.9	1.3	4.9	0.6	3.9
GGT7	7.7	3.0	4.5	0.9	1.0	1.7	2.4	0.4	1.8
LRRC45	7.2	2.8	4.0	1.1	1.4	1.8	2.7	0.6	1.8
MPDU1	5.7	2.4	2.9	0.6	1.4	1.8	4.1	0.6	3.8
SPSB1	6.4	2.7	3.4	0.6	1.0	1.5	3.9	0.4	3.5
GULP1	7.9	3.5	4.1	0.1	0.6	0.9	3.2	0.3	2.7
CYP1A1	5.2	2.1	2.8	1.1	2.6	3.7	2.7	0.5	2.5
LINC00965	7.5	2.2	3.8	0.4	1.1	1.5	3.6	0.4	3.0
UTRN	5.9	1.6	2.4	0.3	1.1	1.5	5.7	0.6	4.2
LHPP	9.0	3.5	4.0	0.7	1.0	2.3	1.2	0.3	1.2
FBXO44	6.9	3.4	3.7	0.7	0.9	1.8	2.9	0.6	2.5
SLC36A4	5.4	1.7	2.8	0.2	0.7	0.8	6.3	0.8	4.5
NSF	6.0	2.1	2.6	0.2	1.0	1.6	5.3	0.7	3.7
LOC643623	7.6	1.4	3.1	0.5	0.8	1.0	5.5	0.5	2.7
DYNC1I2	5.6	2.0	2.7	0.3	1.3	1.9	5.5	0.5	3.5
SMCR7	5.7	2.1	3.6	0.8	1.7	1.9	4.9	0.5	2.0
ATP1A3	4.8	1.8	2.4	0.9	1.5	2.1	4.6	0.9	4.3
CWC27	6.2	3.1	3.6	0.2	0.9	1.2	4.1	0.7	3.2
POU3F2	2.1	4.1	2.9	4.5	1.1	0.2	3.5	2.6	2.0
LRP8	5.9	2.2	3.1	0.7	1.2	1.8	4.0	0.7	3.6
RIOK2	5.3	2.2	3.0	0.2	0.8	0.9	5.5	0.9	4.5
S100A2	6.5	3.3	5.9	0.1	1.0	1.3	2.6	0.2	2.4
IL13RA1	7.3	2.6	3.6	0.2	0.9	1.2	3.9	0.5	3.1
APPBP2	6.4	2.6	3.1	0.2	0.8	1.1	4.4	0.6	4.0
GTF2H1	7.0	2.5	3.3	0.2	0.8	1.3	3.8	0.6	3.7
MDM4	6.8	1.9	2.9	0.4	1.0	1.1	5.3	0.5	3.3
SNIP1	6.2	2.9	3.0	0.5	1.1	1.5	3.7	0.6	3.6
LOC100128573	7.3	1.4	3.2	0.3	0.7	1.1	5.7	0.5	3.1
EFEMP1	6.0	2.7	3.3	0.6	1.6	1.8	3.4	0.8	3.0
H1FX-AS1	7.0	1.5	3.1	0.6	1.2	1.2	5.3	0.7	2.6
NFU1	6.6	3.2	4.1	0.2	1.1	1.7	2.7	1.0	2.5
RAP1A	6.3	2.2	2.3	0.1	0.7	1.1	5.8	0.6	4.2
USP30	5.9	2.5	3.0	0.5	1.6	1.9	3.4	0.8	3.5
PARP3	7.4	3.0	3.6	0.9	1.3	2.1	2.5	0.6	1.8
BLID	7.1	1.4	3.5	0.3	0.8	0.7	5.2	0.4	3.7
CDC42SE1	5.4	2.0	3.3	0.6	0.7	1.4	4.7	0.5	4.6
TBC1D23	6.1	2.3	3.2	0.2	0.7	1.1	5.3	0.5	3.8
CDKN1C	6.4	2.9	3.5	1.0	1.5	1.8	3.1	0.5	2.5
C16orf45	6.4	2.5	3.2	0.6	1.8	1.9	3.9	0.5	2.4
GEMIN5	5.5	1.9	2.5	0.4	1.0	1.5	5.4	0.8	4.0
KIAA0556	7.0	2.3	3.5	0.7	1.1	1.6	3.4	0.7	2.9
THRA	5.9	2.7	3.4	0.6	1.0	1.9	3.8	0.7	3.1
LOC101928555	7.4	1.7	3.2	0.4	0.8	1.0	5.4	0.6	2.6
C19orf12	5.9	3.3	4.0	0.4	0.9	1.2	4.1	0.5	2.8
FLJ37644	8.3	1.6	3.7	0.4	1.2	1.2	4.0	0.4	2.3
SUV420H1	6.9	2.2	3.0	0.2	0.7	1.2	4.5	0.6	3.8
CYLD	6.4	1.9	2.5	0.3	1.1	1.4	5.7	0.4	3.4
BCL2L13	6.3	2.0	3.6	0.4	1.0	1.9	4.1	0.4	3.3
GAPVD1	6.7	2.1	3.1	0.2	1.0	1.2	4.8	0.6	3.4
CETN3	6.0	3.1	2.9	0.1	0.7	1.2	5.0	0.4	3.7
LOC100507342	7.0	1.9	3.2	0.7	1.1	1.9	4.5	0.5	2.2
PLEKHA5	6.0	2.5	3.1	0.6	1.1	1.8	4.1	0.8	3.2
TUBG2	7.0	2.8	3.4	0.7	1.7	1.9	2.7	0.7	2.2

F11R	7.8	2.9	4.2	0.2	0.7	0.8	3.2	0.7	2.4
RASSF1	5.6	2.8	2.7	0.7	1.1	1.3	4.1	0.9	3.8
FOPNL	6.9	2.5	3.3	0.1	1.1	1.6	4.6	0.5	2.4
KLHL26	6.4	2.1	2.8	0.8	0.8	1.6	4.8	0.9	2.8
SNX25	6.4	2.4	3.0	0.3	0.9	0.9	4.8	0.6	3.8
AK3	5.7	2.2	3.3	0.8	1.4	1.4	4.1	0.6	3.6
MAPK6	6.4	2.3	3.2	0.3	1.0	1.3	4.2	0.5	3.8
AACS	6.7	2.5	3.4	0.4	1.1	1.2	4.2	0.6	2.8
NR1H3	7.4	3.0	4.4	0.6	1.2	1.8	2.1	0.5	2.0
NOMO3	5.8	2.2	3.0	0.4	1.0	1.3	4.4	0.7	4.0
TCF19	4.2	1.5	1.7	0.9	1.6	2.1	5.8	0.6	4.4
CUL5	6.6	2.4	2.9	0.3	0.9	1.2	4.7	0.5	3.5
ZC3H13	6.1	1.9	2.9	0.4	0.9	1.7	4.6	0.6	3.8
RFC4	5.2	2.1	2.8	0.3	1.1	1.8	5.4	0.5	3.7
LOC101928495	7.1	1.5	3.4	0.5	0.8	1.6	5.3	0.2	2.5
ZNF286A	4.9	2.1	3.6	0.1	0.6	0.7	6.3	0.7	3.8
TMX3	6.3	2.4	3.4	0.3	1.4	1.5	4.6	0.3	2.6
NUCB2	4.6	1.9	2.0	0.1	0.6	0.9	7.0	0.7	5.0
CD58	7.4	3.0	4.7	0.2	0.8	1.1	2.5	0.3	2.9
LOC100506119	7.8	1.5	3.4	0.5	1.0	1.1	5.2	0.4	2.0
ORMDL2	5.4	1.8	2.6	0.5	1.4	1.5	4.7	1.1	3.9
C19orf44	6.3	2.4	3.1	0.8	1.4	2.2	3.0	0.6	3.1
LOC100130285	7.0	1.5	3.4	0.6	1.1	0.9	5.5	0.4	2.5
CADM4	6.8	2.9	3.7	0.3	0.8	1.2	3.8	0.6	2.7
WHAMM	6.6	2.3	2.9	0.4	0.8	1.2	4.8	0.7	3.3
SLC11A2	6.5	2.4	3.6	0.3	1.0	1.2	4.3	0.6	3.0
SPIN4	5.4	2.4	2.5	0.5	0.5	0.9	6.1	0.6	3.9
VPS9D1-AS1	5.5	2.0	2.6	1.1	1.9	2.4	3.9	0.7	2.7
TMEM251	5.8	2.6	4.4	0.4	1.0	2.2	2.5	0.5	3.5
ROBO1	7.2	2.6	3.7	0.5	1.3	1.8	2.6	0.5	2.7
NAV3	4.0	1.4	2.0	0.4	1.4	1.8	5.5	0.8	5.4
IGHMBP2	6.4	2.6	3.3	0.5	0.9	1.3	4.3	0.7	2.9
CYTH1	7.0	2.6	3.4	0.4	0.8	1.4	3.9	0.7	2.7
SHQ1	5.7	2.2	3.0	0.2	0.8	0.9	5.8	0.5	3.8
KIAA0430	6.8	2.2	2.9	0.5	1.1	1.6	4.1	0.6	3.1
STIM1	6.7	2.4	3.3	0.7	1.1	1.8	3.6	0.6	2.8
TFB2M	6.0	1.6	3.2	0.2	0.8	0.9	5.7	0.6	3.8
TMEM91	8.7	2.6	3.8	0.8	1.3	1.1	2.8	0.3	1.5
TMEM164	6.3	3.0	2.4	0.2	0.8	2.2	3.3	0.6	3.9
PCF11	7.4	2.5	3.7	0.2	0.8	1.1	3.4	0.5	3.1
PAG1	6.8	2.3	3.2	0.2	1.2	1.3	3.6	0.8	3.5
TAF2	6.5	2.1	3.2	0.2	1.0	1.3	4.5	0.5	3.5
GZF1	5.8	2.0	2.8	0.5	0.9	1.4	4.5	0.8	4.2
WDFY3	5.6	1.7	2.6	0.3	0.9	1.2	5.7	0.7	4.1
LOC101927588	7.8	1.4	3.1	0.4	0.7	0.7	5.9	0.5	2.3
CASK	7.2	2.6	3.2	0.3	1.0	1.7	3.4	0.6	2.8
ACP6	6.4	2.3	2.7	0.6	1.5	2.2	3.8	0.6	2.7
ADC	6.0	2.5	3.2	0.5	1.2	1.7	4.1	0.6	2.8
ZNF3	6.5	2.2	2.9	0.4	0.9	1.1	5.1	0.3	3.3
PTGR2	7.8	2.1	3.3	0.5	0.7	0.9	4.2	0.5	2.8
ZBTB11	6.8	2.4	2.9	0.1	0.8	1.2	4.1	0.6	3.7
LOC729083	7.0	2.1	4.3	0.5	1.1	1.0	3.7	0.5	2.5
CNNM2	7.1	2.1	2.9	0.6	1.0	1.6	4.1	0.7	2.8
CDK19	5.5	2.3	3.0	0.7	1.1	1.9	3.5	0.9	3.9
PHF6	6.2	2.5	2.6	0.1	0.9	1.3	3.8	0.6	4.7

JOSD2	7.4	2.5	3.9	1.1	1.7	1.9	2.7	0.3	1.3
DDX20	5.7	2.1	2.9	0.3	0.9	1.2	5.3	0.6	3.6
FAM3A	6.2	2.4	3.6	0.6	1.4	2.0	3.9	0.5	2.1
MMP15	7.9	3.0	3.4	0.6	1.1	1.1	2.9	0.7	1.9
LMLN	7.5	2.5	3.4	0.4	1.0	1.2	3.8	0.4	2.4
NPIPB15	8.0	2.4	3.4	0.5	1.8	1.4	2.8	0.4	2.0
PLXDC2	7.9	3.1	3.9	0.3	1.1	1.4	2.4	0.4	2.0
PCGF6	6.2	2.2	2.6	0.6	1.1	1.4	4.8	0.6	3.2
PGM3	6.7	2.7	3.2	0.1	0.6	1.0	4.4	0.6	3.3
LOC728730	7.1	1.6	3.1	0.5	1.0	1.2	5.0	0.4	2.6
ZBTB11-AS1	6.8	2.0	3.0	0.6	1.0	1.4	4.6	0.7	2.5
NADK2	6.9	2.9	3.4	0.3	0.8	0.9	4.1	0.5	2.9
CETN2	6.6	2.0	3.2	0.3	0.7	2.0	4.3	0.7	2.9
LOC100506085	6.9	2.0	3.3	0.3	0.8	1.1	4.2	0.6	3.3
EPC2	5.6	2.1	2.7	0.3	1.1	1.1	4.4	0.9	4.3
POU3F3	1.7	4.6	3.0	4.4	1.4	0.0	2.7	2.7	1.9
IREB2	6.8	2.1	3.3	0.1	0.7	1.0	4.7	0.6	3.2
LOC439994	6.3	2.3	3.2	1.0	1.4	2.2	3.4	0.6	2.2
IWS1	6.4	1.9	2.4	0.2	1.0	1.5	5.0	0.6	3.5
MGST2	7.8	3.0	3.7	0.1	1.2	1.1	2.5	0.4	2.7
TRIM39	7.0	2.2	3.2	0.3	0.9	1.4	4.5	0.4	2.8
PIK3C3	6.2	2.5	3.2	0.1	0.9	1.3	4.4	0.5	3.5
DNAAF3	6.7	2.7	3.4	0.7	1.4	1.9	2.7	0.6	2.5
NFATC2	5.9	1.8	2.4	0.5	0.9	1.2	4.9	1.0	4.0
MPHOSPH10	6.2	2.4	2.7	0.3	0.7	1.1	4.9	0.6	3.6
PHF11	6.9	1.7	3.4	0.5	0.7	1.3	5.0	0.7	2.4
LOC100129046	6.0	1.9	2.6	1.0	1.8	1.8	3.6	0.7	3.0
LOC100507217	6.3	2.0	2.6	0.6	1.4	1.1	4.6	0.7	3.3
LOC100129516	7.9	1.0	3.2	0.6	1.0	0.8	5.7	0.3	2.0
FSTL3	5.3	2.1	2.6	1.4	2.4	3.0	3.2	0.4	2.0
FRMD5	4.8	1.8	2.4	0.5	0.9	1.4	5.1	1.0	4.6
GTSE1	6.2	2.4	2.8	0.5	1.1	1.5	4.1	0.8	3.0
GGH	5.5	1.8	3.5	0.6	1.0	1.8	4.2	0.8	3.3
LINC00342	6.9	1.3	3.2	0.4	1.1	1.3	5.0	0.4	2.8
ZCCHC7	6.3	2.0	3.1	0.3	0.9	1.4	4.3	0.7	3.5
UBE2Q2	6.9	2.6	3.2	0.3	0.9	1.4	3.5	0.5	3.0
GRAMD1B	7.1	2.4	4.6	0.4	1.0	1.3	3.0	0.4	2.2
EFTUD1	5.4	2.2	2.9	0.3	0.9	1.2	4.7	0.6	4.2
CXorf40B	6.2	2.0	3.5	0.3	1.0	1.0	4.2	0.6	3.7
ZSCAN25	6.1	1.8	3.7	0.6	1.0	1.3	4.4	0.7	2.9
KLC3	5.5	2.2	2.8	1.0	2.3	2.8	3.0	0.6	2.3
WEE1	5.3	1.9	2.4	0.6	1.0	1.4	5.3	0.7	3.7
AKAP11	6.7	2.1	3.0	0.2	0.8	1.2	4.6	0.5	3.3
GNB5	6.7	2.1	3.4	0.6	1.2	1.4	3.7	0.3	2.9
RFK	5.9	2.2	2.8	0.0	0.2	0.5	5.6	0.8	4.5
RBBP8	5.2	1.9	2.7	0.2	0.7	1.3	5.0	0.7	4.7
GNPAT	6.1	2.3	3.3	0.2	0.8	1.2	4.5	0.5	3.5
TECPR2	6.8	2.0	3.9	0.5	0.9	1.4	3.8	0.5	2.6
PAN3	6.3	2.2	2.7	0.3	1.0	1.2	4.5	0.6	3.6
RDH13	6.2	1.6	2.8	0.8	1.3	1.5	4.2	0.7	3.1
CTAGE5	6.2	2.4	3.0	0.2	0.7	0.9	4.8	0.4	3.7
RIPK1	5.9	2.0	2.7	0.5	0.9	1.5	4.4	0.5	3.9
ARMC5	5.8	1.9	3.0	0.5	1.1	1.4	4.5	1.0	3.3
TMEM69	4.6	1.9	3.4	0.2	0.7	1.4	4.1	0.9	5.1
OGG1	6.7	2.3	2.7	0.6	1.2	2.1	3.7	0.6	2.6

MTMR12	5.6	2.4	2.7	0.3	0.9	1.3	4.9	0.8	3.5
ETFB	6.8	2.4	2.9	1.0	2.2	3.2	2.2	0.4	1.4
PLTP	5.5	2.1	2.9	1.0	2.2	2.3	3.2	0.6	2.6
RRAGB	6.7	2.0	2.6	0.1	0.7	1.2	5.2	0.5	3.3
SERPINB8	5.1	1.9	2.7	0.3	1.1	1.6	4.7	0.8	4.2
RNF111	5.4	2.1	2.9	0.4	0.9	1.5	3.7	0.8	4.7
DYRK3	5.2	1.9	2.5	0.4	0.8	1.4	5.2	0.7	4.2
LEMD3	6.2	1.9	3.0	0.4	1.0	1.3	4.0	0.9	3.6
SMAD1	6.5	2.0	3.2	0.3	0.7	1.3	4.0	0.7	3.6
MIR210HG	7.2	1.6	3.1	0.4	0.8	0.8	5.5	0.3	2.6
F8A3	5.8	2.0	2.5	0.9	1.3	1.6	4.6	0.8	2.9
F8A2	5.8	2.0	2.5	0.9	1.3	1.6	4.6	0.8	2.9
F8A1	5.8	2.0	2.5	0.9	1.3	1.6	4.6	0.8	2.9
SPHK1	5.3	2.0	2.9	0.7	1.1	1.9	5.2	0.6	2.7
CORO7	6.9	2.0	3.2	1.0	1.8	2.4	2.7	0.4	1.9
DDX58	7.3	2.9	4.1	0.1	0.4	0.5	3.6	0.4	3.1
EEF1E1-MUTED	5.9	2.1	3.1	0.3	1.0	1.3	4.4	0.6	3.6
ARHGEF40	7.1	3.0	3.4	0.7	1.1	2.1	2.8	0.4	1.8
RPP38	6.5	2.7	3.2	0.4	0.6	1.8	3.5	0.6	2.9
FBXO22	4.4	1.2	1.8	0.4	1.1	1.3	7.0	0.7	4.4
KCNQ5	4.1	1.5	2.0	0.3	0.8	1.0	6.5	0.8	5.3
PAK1	6.9	2.6	3.0	0.3	0.7	1.4	3.7	0.6	3.1
ZMAT5	6.2	2.4	3.3	0.7	1.6	2.2	3.1	0.6	2.3
SELK	6.0	1.5	3.3	0.1	0.7	0.6	6.7	0.5	3.0
SYNGAP1	6.1	2.4	3.3	0.7	1.4	1.7	3.1	0.7	2.8
SLC30A9	6.4	2.8	3.5	0.4	1.2	1.5	3.2	0.5	2.7
COPS4	5.4	2.7	3.4	0.2	1.2	1.4	4.3	0.4	3.3
UFM1	6.1	1.9	4.2	0.2	0.6	0.5	4.1	0.6	4.1
HCCS	6.2	1.6	2.8	0.2	0.9	1.6	4.4	0.6	3.8
ATP8B1	5.4	2.0	3.0	0.3	0.9	1.4	4.8	0.6	3.8
CEACAM19	4.6	1.7	3.2	1.1	1.5	2.1	3.7	0.8	3.5
SATB1	6.2	2.0	3.1	0.5	1.1	1.6	3.6	0.9	3.3
MED14	6.6	2.3	2.8	0.3	0.9	1.2	3.8	0.6	3.8
CXCL3	8.9	2.9	6.0	0.4	0.9	1.0	1.0	0.1	0.8
HILPDA	6.3	1.9	2.4	0.3	0.5	1.6	5.0	0.4	3.8
SYT17	5.7	1.9	2.6	0.6	0.8	1.2	4.9	1.0	3.6
GPRC5B	5.8	2.3	2.9	0.7	1.2	1.3	4.4	0.8	2.9
DBNDD1	5.6	3.0	3.4	0.6	1.0	1.6	3.1	0.6	3.4
BLOC1S2	6.1	1.7	3.2	0.4	1.0	1.2	4.9	0.6	2.9
LOC101929144	6.7	1.4	3.3	0.3	0.8	1.0	5.9	0.5	2.2
LEMD1-AS1	7.0	1.4	3.1	0.4	0.9	1.3	5.6	0.3	2.2
TRIM14	4.8	1.8	2.6	1.1	1.8	2.4	3.9	0.9	2.8
SNX4	6.3	2.4	3.4	0.2	1.0	1.0	3.9	0.7	3.2
DCP2	5.7	1.8	2.7	0.1	0.6	1.0	6.1	0.5	3.6
ELMOD3	5.9	2.7	3.5	0.5	1.3	1.8	3.7	0.5	2.3
KLHDC2	7.0	2.3	2.8	0.5	1.0	1.6	3.8	0.4	2.7
C12orf76	6.5	2.2	2.8	0.5	0.9	1.4	5.3	0.4	2.1
FAM73B	6.2	2.7	3.0	0.5	0.8	1.3	3.7	0.7	3.2
STAMBPL1	5.4	2.0	2.3	0.2	0.8	1.2	5.5	0.6	4.3
GALNT7	6.1	2.4	3.1	0.2	0.9	1.1	4.4	0.5	3.4
LOC101928657	5.4	2.3	3.2	1.5	1.7	1.3	3.5	0.8	2.5
EMP2	5.3	2.5	2.5	0.6	0.9	1.7	3.3	0.8	4.6
NUP214	5.9	2.1	2.8	0.6	1.2	1.7	3.8	0.7	3.5
ZNF71	5.6	2.8	2.8	0.9	1.5	2.0	3.3	0.7	2.7
C1QTNF5	7.8	3.1	4.1	0.8	1.2	1.3	2.0	0.6	1.3

TCP11L1	6.2	1.9	2.8	0.2	1.1	1.4	4.3	0.5	3.9
LINC00671	7.5	1.4	3.2	0.5	0.8	1.1	4.7	0.5	2.4
PDXP	4.1	1.7	2.9	0.9	1.8	1.9	4.1	0.7	4.1
MFHAS1	6.5	2.2	2.9	0.5	0.8	1.3	4.2	0.5	3.2
FAM179B	6.7	2.4	3.3	0.3	0.9	1.2	4.1	0.5	2.8
COG7	7.3	2.5	3.8	0.6	1.1	1.3	2.6	0.5	2.4
NEDD1	5.6	1.8	2.6	0.2	0.7	0.7	5.4	0.6	4.6
C18orf25	5.9	2.4	2.9	0.3	0.6	0.8	5.0	0.7	3.5
TMEM138	5.9	2.4	2.4	0.4	1.0	1.5	3.7	0.7	4.0
SLC6A6	6.9	2.3	3.2	0.5	1.1	1.3	3.3	0.5	3.0
CENPN	3.7	1.9	1.9	0.3	1.0	1.6	6.6	1.1	4.1
TUBGCP6	7.1	2.8	4.1	0.7	1.2	1.4	2.6	0.4	1.8
NINL	7.3	2.4	3.6	0.6	1.2	1.4	3.0	0.5	2.0
ABHD14A-ACY1	6.0	2.7	3.3	0.8	1.8	2.6	2.5	0.5	1.9
TMEM185A	6.7	2.6	3.0	0.3	0.8	0.9	4.2	0.5	3.0
TUBGCP3	5.0	2.0	2.4	0.5	1.0	1.6	4.9	0.8	3.8
PPIG	5.9	2.3	3.1	0.2	0.9	1.2	4.0	0.5	4.1
SLC48A1	5.0	3.0	2.7	1.0	0.7	1.5	4.7	0.8	2.7
MARS2	4.4	1.4	2.2	0.4	1.0	0.9	6.7	0.7	4.5
RDH10	7.7	2.6	3.6	0.4	0.9	1.2	3.5	0.4	1.8
POC1B	5.2	2.4	3.4	0.2	0.6	1.1	5.0	0.5	3.7
APOE	6.5	2.5	3.6	0.3	0.6	0.6	4.8	0.9	2.3
NSUN5	5.4	2.2	2.9	0.6	1.3	1.6	4.3	0.9	2.8
C9orf91	4.8	1.5	2.3	0.5	1.0	1.0	5.8	0.7	4.4
LOC101929086	7.2	1.8	3.7	0.5	1.1	1.4	3.9	0.3	2.1
BRCC3	4.8	2.5	2.2	0.5	1.0	1.2	5.0	0.8	4.0
UVSSA	6.7	1.9	3.5	0.5	0.9	1.4	4.3	0.5	2.2
GSTM4	7.8	2.5	3.2	0.8	1.5	2.0	2.2	0.2	1.7
LCOR	5.9	1.9	2.8	0.4	0.8	1.0	5.0	0.6	3.7
COX15	6.4	2.3	3.8	0.4	1.2	1.4	3.2	0.4	2.8
ZBTB8OS	5.5	2.2	3.1	0.2	1.0	0.9	4.5	0.6	3.9
VPS45	7.1	2.6	3.4	0.2	0.9	1.1	3.2	0.5	2.9
GABRA3	5.5	2.5	2.9	0.4	1.1	1.6	3.4	0.8	3.8
GALNT5	7.6	2.7	3.5	0.3	0.9	1.1	3.0	0.5	2.4
MELK	6.4	1.7	2.9	0.4	1.1	1.2	4.9	0.5	3.1
GTF2H2	4.8	2.5	3.9	0.1	0.6	0.8	4.9	0.4	4.0
LIG1	5.9	1.7	3.0	0.8	1.9	2.0	3.9	0.6	2.2
EVC	6.1	1.9	2.7	0.9	1.5	2.1	3.9	0.7	2.2
CENPM	5.5	1.7	3.0	0.4	1.0	1.3	5.7	0.6	2.9
BIRC5	5.4	2.7	2.3	0.8	1.5	2.1	3.8	0.7	2.8
LYPLAL1	6.7	2.5	3.3	0.1	0.6	1.6	3.9	0.5	2.7
RAB4A	5.9	2.6	2.8	0.5	1.4	0.9	4.0	0.7	3.1
SUPT3H	5.9	1.8	2.7	0.4	1.2	1.4	4.3	0.6	3.8
SNORD110	4.8	2.1	2.5	1.1	0.5	3.0	3.5	0.7	3.8
SDHAF1	5.8	2.5	2.9	0.6	1.0	1.0	5.2	0.7	2.1
C1orf109	5.2	1.8	2.4	0.2	1.1	1.5	5.5	0.9	3.2
LOC646626	6.6	2.6	2.9	0.8	1.2	1.9	2.8	0.6	2.3
ZDHHC18	5.1	2.4	3.1	0.8	0.8	1.6	3.8	0.7	3.6
MBTD1	6.5	1.7	2.6	0.2	0.8	0.8	4.6	0.7	4.0
METTL2B	5.0	1.6	2.5	0.4	0.9	1.1	5.8	0.8	3.7
UNKL	6.5	2.3	3.4	0.8	1.5	1.8	2.4	0.4	2.8
PCAT1	7.4	1.5	3.3	0.4	0.8	0.8	4.9	0.4	2.3
OSTF1	5.7	2.6	2.9	0.5	1.0	1.5	4.2	0.5	3.0
IFNGR1	6.4	2.6	3.9	0.2	0.6	0.9	3.2	0.6	3.4
FAM160A2	6.0	2.8	3.5	0.5	0.8	1.4	3.5	0.6	2.8

SLC38A7	6.7	2.8	3.6	0.5	1.0	1.3	3.5	0.4	2.0
DSERG1	7.3	1.2	3.6	0.3	0.5	1.2	5.7	0.2	1.8
C12orf73	5.8	1.1	2.2	0.7	0.9	1.6	3.4	1.1	4.9
NARS2	5.8	2.3	3.1	0.3	0.8	1.0	4.8	0.6	3.1
KIAA0247	5.4	2.0	2.9	0.6	0.7	1.3	4.0	1.0	3.9
TTC5	5.5	2.3	2.7	0.4	1.3	1.6	4.2	0.4	3.4
GATSL3	6.8	2.0	3.4	0.7	1.6	2.5	2.0	0.6	2.1
LOC100507460	6.1	1.8	3.0	0.2	1.0	1.1	4.6	0.3	3.7
MAP3K4	6.6	2.2	2.8	0.4	1.1	1.3	3.9	0.6	3.0
NR2C2AP	4.9	2.8	3.1	0.1	1.1	1.3	4.2	0.6	3.7
SRFBP1	5.2	2.0	2.3	0.1	0.5	1.2	5.2	0.6	4.7
HIF1A-AS2	7.2	2.4	3.6	0.1	0.7	1.0	3.4	0.3	3.1
ZNF507	6.2	2.2	2.6	0.2	1.1	1.2	4.0	0.4	3.9
IFIT2	7.3	2.5	3.6	0.1	0.9	0.9	3.4	0.6	2.4
IL17RC	6.7	2.6	3.6	0.8	1.2	2.0	2.9	0.3	1.8
C5orf51	5.2	2.1	2.4	0.2	0.6	1.1	5.7	0.7	3.8
NOL8	5.2	2.1	2.3	0.2	0.7	1.0	5.1	0.5	4.6
EXOC8	6.0	2.0	3.0	0.5	1.1	1.2	4.2	0.6	3.2
CCDC74B-AS1	7.4	1.5	3.2	0.4	0.7	0.9	4.8	0.5	2.3
GEMIN6	4.2	2.7	3.4	0.4	1.5	1.5	3.8	0.6	3.7
PLEKHG2	5.8	2.6	2.9	0.5	1.3	1.5	4.0	0.5	2.7
ZNF670-ZNF695	6.9	1.6	3.1	0.6	1.1	1.3	4.5	0.4	2.3
MUM1	5.5	2.0	2.7	0.7	1.3	1.6	4.4	0.5	3.0
RGS3	5.5	2.7	2.9	0.6	1.2	1.5	3.9	0.7	2.8
NRM	5.7	2.8	2.9	0.9	1.1	2.4	3.1	0.7	2.2
MYCBP2	5.8	1.5	2.5	0.3	0.8	1.0	5.3	0.6	4.0
LOC729732	7.3	1.5	3.1	0.5	1.1	1.0	4.6	0.5	2.2
LOC728769	6.6	1.9	3.2	0.3	1.0	1.3	4.5	0.5	2.4
TOP3B	7.5	2.4	3.8	0.4	1.0	1.3	2.9	0.6	1.9
SPPL2A	6.3	2.4	3.3	0.2	0.6	1.4	4.4	0.3	2.9
CCDC51	6.1	1.9	2.6	0.4	0.8	1.0	5.4	0.7	2.9
KIAA0141	5.5	2.1	2.8	0.6	1.4	1.7	3.7	0.7	3.4
ALKBH4	6.3	2.1	2.8	1.0	1.1	1.5	3.5	0.6	2.8
ZNF212	5.5	2.3	3.0	0.3	1.0	1.0	5.5	0.5	2.7
TRABD2A	4.8	2.0	2.3	0.3	1.2	0.9	5.0	0.7	4.4
RANBP10	5.6	2.4	3.4	0.7	1.6	1.6	3.3	0.6	2.6
LINC00670	8.0	1.4	3.6	0.2	0.9	1.0	3.7	0.3	2.7
ZNF154	6.8	1.4	2.9	0.3	0.9	0.9	5.5	0.5	2.5
SYNJ2BP	7.1	2.0	3.5	0.2	0.9	1.3	3.0	0.5	3.3
IDNK	7.8	1.3	2.6	0.6	0.8	0.9	5.2	0.5	2.1
FKBP7	5.0	2.4	4.3	0.3	1.1	1.2	3.1	0.5	3.9
PCED1B-AS1	7.7	1.3	2.8	0.6	0.9	0.9	4.9	0.4	2.1
LOC728485	7.5	1.3	3.0	0.6	1.0	0.9	4.6	0.6	2.1
LONRF3	6.0	1.9	2.9	0.5	0.8	1.0	4.5	0.7	3.5
LOC101928893	7.0	1.7	3.1	0.3	0.8	1.0	5.5	0.3	2.0
MBOAT2	6.0	2.5	3.3	0.7	1.4	1.6	3.0	0.5	2.6
MRE11A	5.4	2.1	2.8	0.1	1.2	1.0	4.8	0.8	3.2
POLE	5.7	2.1	2.6	0.6	1.1	1.7	4.1	0.7	3.1
NHEJ1	6.6	2.1	2.8	0.4	0.6	1.0	4.2	0.4	3.5
SPATA2L	4.4	2.6	3.0	0.7	1.2	1.6	4.8	0.7	2.9
HAUS7	5.2	1.9	2.5	0.8	1.2	1.6	4.2	0.7	3.6
SERGEF	6.5	2.9	3.1	0.3	1.0	1.1	3.6	0.5	2.6
ST3GAL6	5.6	2.2	3.0	0.1	0.8	1.1	4.9	0.5	3.5
P2RX4	6.8	2.5	3.2	0.8	1.5	2.3	1.8	0.3	2.4
C16orf70	5.8	2.1	3.0	0.3	0.8	1.3	4.2	0.6	3.7

SVIP	4.2	2.8	3.4	0.2	0.5	2.0	4.3	1.1	3.1
CCDC69	7.0	2.6	2.4	0.8	1.6	2.1	2.5	0.3	2.3
LOC100996291	7.3	1.4	3.0	0.5	0.8	1.0	5.3	0.4	2.0
GXYLT2	6.5	2.3	2.7	0.7	1.5	1.9	3.3	0.6	2.1
APOOL	6.5	2.4	2.9	0.2	0.8	1.2	3.4	0.7	3.5
NUP37	5.2	2.2	3.2	0.0	0.8	1.2	4.4	0.6	4.0
PAQR4	5.0	2.1	2.8	1.1	1.5	2.6	3.5	0.7	2.3
BRMS1L	5.8	2.6	3.1	0.2	0.8	1.0	3.8	0.8	3.4
FBXL3	5.1	1.7	2.6	0.2	0.7	1.2	5.0	0.8	4.4
ATXN3	7.3	1.5	3.0	0.4	0.7	1.2	4.7	0.4	2.4
MIR503HG	4.3	1.9	2.7	0.4	1.1	1.5	5.5	0.8	3.3
RMND5B	6.3	1.8	2.7	0.4	1.1	1.4	4.5	0.5	2.8
FCHO1	6.4	2.9	3.0	0.9	1.3	1.5	2.5	0.6	2.3
LOC101928826	4.7	2.1	3.1	0.2	1.0	0.0	8.4	0.0	1.9
MCM6	4.8	2.0	2.2	0.2	0.9	1.6	5.2	0.6	3.9
SHISA4	6.6	2.9	3.2	0.6	1.0	1.8	2.7	0.6	2.2
DPP8	6.4	1.9	3.1	0.2	0.8	0.9	4.5	0.5	3.1
EGFR-AS1	5.4	2.0	2.8	0.6	1.2	1.5	3.6	0.6	3.9
CASP9	6.0	2.4	3.3	0.7	0.8	1.2	4.2	0.5	2.5
ELF3	9.9	3.3	5.5	0.4	0.6	0.8	0.6	0.1	0.4
NIPBL	6.4	1.8	2.7	0.2	0.8	1.0	4.4	0.5	3.7
KLHDC10	5.7	2.2	3.0	0.3	0.9	1.4	3.6	0.5	4.0
LILRB3	6.9	1.5	3.3	0.4	0.8	1.1	5.0	0.4	2.1
ABCC4	6.2	1.6	2.7	0.3	0.8	1.2	4.8	0.5	3.4
DEPDC4	6.8	1.4	3.1	0.6	1.0	1.1	4.8	0.5	2.3
BIRC3	7.3	2.2	3.3	0.1	0.7	0.8	3.9	0.5	2.8
ELF2	6.1	2.1	2.8	0.3	0.7	1.2	4.2	0.7	3.4
LOC100507459	6.8	1.6	2.9	0.4	1.1	1.2	4.4	0.6	2.5
THUMPD1	6.0	2.3	3.1	0.4	0.9	1.6	3.1	0.8	3.2
CRYZ	5.8	2.6	3.5	0.1	0.8	1.5	3.8	0.6	2.9
PRKCDBP	6.6	2.7	3.3	0.9	1.5	1.3	2.7	0.5	1.9
ASUN	5.0	1.8	2.2	0.3	1.2	1.2	5.0	0.7	4.0
KLHL12	6.4	2.0	3.0	0.3	0.9	1.2	4.0	0.4	3.3
LOC100996283	7.2	2.4	0.8	0.6	1.3	0.8	6.0	0.2	2.0
TRIM47	6.3	2.5	2.8	0.7	1.0	1.4	3.8	0.6	2.3
TEX40	5.1	2.2	2.8	0.9	1.1	1.6	3.9	0.5	3.5
TINF2	4.9	1.9	2.3	0.3	1.3	1.6	4.8	0.7	3.6
SMYD4	5.9	2.0	2.6	0.4	0.9	1.3	4.3	0.6	3.4
SAMD12	5.5	1.8	2.1	0.2	1.0	0.9	4.8	0.8	4.5
ACAD9	4.8	1.8	2.4	0.7	1.6	2.0	4.4	0.6	3.2
RNMT	6.0	1.8	2.7	0.1	0.5	0.9	5.0	0.5	3.8
KIAA0895L	5.3	2.7	4.2	1.2	1.7	2.0	2.2	0.4	1.7
EEF2K	4.9	1.7	2.4	0.8	1.3	1.7	4.4	0.7	3.6
GTDC2	6.1	2.3	3.2	0.7	1.4	1.4	3.2	0.7	2.4
UBA5	5.0	1.8	2.7	0.3	0.9	1.4	5.2	0.8	3.3
NUP133	5.8	2.3	2.6	0.2	0.8	1.1	4.8	0.5	3.1
STOM	7.0	1.5	2.8	0.5	0.8	1.2	5.2	0.4	2.1
MTMR1	5.0	2.1	2.5	0.3	0.6	1.1	5.3	0.8	3.6
PRICKLE3	6.2	2.5	3.2	0.7	1.0	1.5	2.8	0.8	2.7
PLEKHA3	6.6	1.9	3.3	0.1	1.0	1.0	3.5	0.7	3.4
ALDH3A2	7.0	2.8	3.4	0.4	1.7	1.7	2.5	0.2	1.7
MSH2	4.7	1.8	2.2	0.2	0.7	1.0	5.9	0.8	3.9
ACAA1	6.0	2.4	3.3	0.6	0.9	1.7	3.3	0.5	2.7
OXLD1	6.7	2.8	3.5	0.8	1.4	2.6	2.1	0.3	1.3
C18orf61	6.8	1.5	3.0	0.4	0.8	0.9	5.1	0.4	2.4

DTX3	5.8	2.5	4.0	0.6	1.2	1.8	2.8	0.5	2.1
MLYCD	6.3	2.4	3.4	0.9	1.4	1.9	2.8	0.5	1.8
C6orf123	6.4	1.1	3.3	0.4	1.0	1.2	5.1	0.4	2.4
CPEB2	6.7	2.7	3.4	0.9	1.2	1.1	2.5	0.6	2.1
LACTB	5.9	2.4	2.5	0.6	1.3	1.2	4.1	0.4	2.8
FAM105B	4.7	1.9	3.0	0.5	1.3	1.0	4.6	0.7	3.6
FAM138C	5.4	2.1	1.2	0.1	0.1	1.6	7.1	0.2	3.4
ZSWIM8-AS1	6.6	3.1	3.4	0.6	1.1	1.5	2.4	0.3	2.3
ZNF576	4.9	2.2	2.8	0.8	0.8	1.5	4.2	0.7	3.3
LRR1	4.5	2.6	3.8	0.1	0.9	0.8	3.6	0.5	4.5
ACAD10	7.0	2.4	3.8	0.4	1.2	1.6	2.7	0.4	1.9
GALT	5.0	1.7	2.8	0.5	1.5	1.9	3.5	0.7	3.6
RHBDL1	5.9	3.3	3.7	0.6	0.9	1.4	3.1	0.5	1.8
RAB11FIP1	6.2	2.0	2.9	0.3	0.7	0.8	4.4	0.6	3.2
ARMC8	5.7	2.2	3.1	0.2	0.9	1.1	4.0	0.6	3.4
ERI1	5.6	1.9	2.5	0.5	0.7	0.9	4.7	0.6	3.8
AGAP10	6.7	1.3	3.0	0.5	1.5	1.3	4.1	0.5	2.3
KBTBD2	5.2	1.8	2.7	0.1	0.7	0.9	5.6	0.4	3.8
CSF2RA	6.4	1.7	3.1	0.4	0.7	1.0	4.7	0.5	2.6
CSF2RA	6.4	1.7	3.1	0.4	0.7	1.0	4.7	0.5	2.6
WDR11	6.3	2.1	3.1	0.3	1.0	1.2	3.9	0.4	2.9
ZFYVE20	5.6	2.1	3.3	0.3	0.9	1.3	4.2	0.4	3.0
TMEM242	6.5	2.1	2.9	0.1	0.7	1.1	4.4	0.5	2.8
DMTF1	6.6	2.3	2.9	0.2	1.2	1.5	3.6	0.5	2.4
PCBP4	5.8	1.9	2.4	0.7	1.6	1.6	3.1	0.5	3.3
MEST	6.0	2.6	2.6	0.3	0.6	1.1	4.1	0.6	3.1
TXLNG	5.4	2.0	2.5	0.2	0.9	1.1	4.5	0.6	3.9
COQ10B	6.1	1.7	4.0	0.4	0.7	1.0	3.2	0.6	3.4
EXOSC9	4.9	2.3	2.8	0.2	0.9	0.9	4.5	0.7	3.8
CASP3	4.8	1.8	2.6	0.1	0.7	0.8	5.4	0.3	4.7
MTUS1	5.2	1.9	2.4	0.2	0.9	1.0	5.2	0.6	3.7
ZSCAN29	5.1	2.0	2.7	0.1	0.6	0.9	5.8	0.5	3.3
DIS3L2	6.0	2.5	3.3	0.5	1.4	1.8	2.8	0.4	2.3
CXXC5	5.5	2.2	3.1	0.6	0.9	1.4	3.5	0.7	3.0
SLC35F5	5.6	2.0	2.8	0.1	0.8	1.3	4.4	0.5	3.5
ENTPD3-AS1	7.3	1.3	2.7	0.3	0.8	1.1	5.3	0.4	2.0
ADCY9	5.3	1.7	2.6	0.5	1.0	1.3	4.3	0.8	3.6
ISY1-RAB43	6.3	1.0	2.6	0.0	0.4	0.3	6.2	0.2	4.1
MEMO1	5.5	2.2	2.9	0.4	0.8	1.1	4.1	0.7	3.3
TAGLN	4.3	1.7	2.5	0.2	0.8	1.0	5.0	0.9	4.8
CIR1	6.4	2.7	3.2	0.3	0.9	1.5	2.6	0.4	3.2
LSM5	6.0	1.5	3.0	0.2	0.6	1.2	4.5	0.3	3.7
LOC100506472	7.0	1.3	3.1	0.3	0.9	1.0	5.1	0.3	2.1
ANKS3	5.7	2.0	2.9	0.7	1.0	1.3	4.0	0.4	3.0
RRAGC	5.4	2.2	3.1	0.3	0.7	1.4	3.9	0.5	3.7
CTR9	6.4	2.3	2.8	0.4	1.0	1.2	3.6	0.4	2.9
ENO2	5.7	2.3	3.3	0.4	1.0	1.1	3.9	0.5	2.8
C7orf43	5.3	2.6	2.8	0.5	0.8	1.4	3.7	0.6	3.3
FBXO41	5.9	2.1	2.7	0.7	1.3	1.6	3.5	0.6	2.5
TMEM175	6.3	2.3	3.0	1.1	1.6	2.1	2.3	0.6	1.6
ILKAP	5.3	2.1	2.9	0.3	0.8	1.1	4.7	0.7	3.1
FOXA1	5.2	2.3	2.8	0.6	0.8	1.3	3.5	1.0	3.7
EEA1	5.6	1.9	2.4	0.2	0.9	1.2	3.9	0.6	4.3
ZFP90	6.6	1.9	3.4	0.2	0.6	1.5	3.8	0.3	2.6
UBXN2A	6.2	2.1	2.5	0.1	0.4	0.9	4.5	0.4	3.8

RFC5		4.6	1.7	2.3	0.4	1.4	1.4	4.6	0.8	3.7
PIK3CB		6.5	2.0	2.9	0.2	0.6	1.1	4.3	0.5	2.9
YIPF4		5.8	3.0	3.7	0.1	0.8	0.9	3.5	0.3	2.8
FAM160B1		6.5	2.1	3.0	0.1	0.7	1.0	4.4	0.5	2.7
KYNU		8.5	2.8	3.7	0.1	0.5	0.6	3.0	0.2	1.6
ZNF446		5.6	1.9	2.7	0.8	1.7	2.1	3.4	0.5	2.1
NPIPA1		6.3	2.0	3.1	0.5	1.5	1.4	2.6	0.6	2.9
PHLDB3		5.6	2.3	3.1	0.5	1.3	1.6	3.8	0.4	2.3
PDLIM2		4.9	2.1	3.0	0.6	1.2	1.4	3.8	0.7	3.1
TMEM5		5.3	2.3	2.6	0.2	0.8	1.1	4.7	0.5	3.4
EAPP		5.8	1.8	3.0	0.2	0.9	1.0	3.5	0.6	4.1
LOC100287225		6.9	1.4	3.0	0.4	0.9	0.8	4.8	0.4	2.2
METTL16		5.3	2.0	2.3	0.2	0.7	0.7	4.9	0.7	4.2
KLF12		6.0	2.2	2.6	0.5	0.9	1.5	3.7	0.7	2.7
DENND5A		5.0	1.7	2.4	0.5	1.1	1.6	4.2	0.6	3.7
GGCX		5.1	2.0	2.8	0.4	1.1	1.6	4.4	0.5	2.9
GNPTAB		6.3	2.3	3.2	0.3	0.7	1.3	3.4	0.5	2.8
LOC100996455		6.2	1.5	2.6	0.4	0.9	1.2	5.2	0.4	2.4
DHRS7		6.0	1.6	2.8	0.4	0.6	0.7	4.9	0.7	3.2
PLEKHO2		6.3	2.2	2.6	0.5	1.1	1.5	3.6	0.6	2.4
ATP1A1OS		7.9	2.0	2.4	0.3	0.9	1.0	4.3	0.1	1.9
ZHX2		4.2	1.6	2.0	0.6	0.9	1.3	4.6	1.1	4.7
LOC100127888		6.5	2.9	3.4	1.0	1.9	2.0	1.6	0.3	1.2
STK19		6.6	2.5	3.0	0.4	1.2	1.4	3.2	0.4	2.1
PSMD5		5.3	2.0	2.5	0.3	0.9	1.0	5.3	0.4	3.2
SH2B2		6.3	2.1	2.8	0.8	1.1	1.5	3.2	0.6	2.4
ADCK4		6.3	2.7	3.8	0.7	1.0	1.5	2.8	0.4	1.7
COX10-AS1		6.0	1.3	3.2	0.4	1.1	1.3	4.0	0.4	3.0
LINC00337		6.4	1.3	3.2	0.5	0.8	0.8	5.0	0.3	2.4
MSRB2		5.7	2.3	3.2	0.6	1.2	1.7	3.3	0.3	2.4
C1orf50		5.0	1.7	2.4	0.6	1.7	1.7	3.8	0.8	2.9
P4HA1		6.3	2.2	3.0	0.1	0.6	1.1	4.0	0.5	3.0
SLC27A3		7.1	2.2	4.2	0.4	0.9	1.1	2.6	0.4	1.7
ARID3B		4.3	1.8	2.3	0.3	0.6	0.8	5.3	1.0	4.3
IFIT5		5.0	1.9	2.2	0.4	0.9	1.1	4.9	0.6	3.7
MTO1		5.8	2.4	3.1	0.3	0.8	1.1	3.8	0.5	2.9
ZNF324B		5.2	1.8	2.6	0.7	1.7	2.1	3.6	0.5	2.5
	44629.0	6.1	2.2	2.6	0.7	0.9	1.4	3.6	0.6	2.6
KIF4A		6.0	1.5	2.9	0.4	1.0	1.2	5.0	0.3	2.4
GPR161		4.3	1.6	2.0	0.4	0.8	1.1	5.7	0.9	4.1
L3MBTL1		6.8	2.1	3.6	0.6	1.2	1.2	3.1	0.3	1.9
AKAP12		5.3	1.9	2.4	0.5	1.1	1.3	4.2	0.6	3.4
CHAMP1		4.8	1.9	2.2	0.2	1.0	1.2	4.6	0.9	3.8
EPS8		6.1	2.3	2.9	0.3	1.0	1.2	3.3	0.6	2.9
PKD2		6.4	1.8	3.5	0.7	1.0	1.7	3.2	0.5	2.0
AHDC1		5.6	2.2	3.0	0.6	1.1	1.6	3.6	0.5	2.4
LOC100130954		6.6	1.2	3.1	0.5	0.8	0.7	5.0	0.6	2.3
UTP14C		5.1	1.7	2.5	0.3	0.7	1.3	4.5	0.6	4.0
TTC7A		5.4	2.0	2.8	0.5	1.1	1.6	3.9	0.6	2.7
MTHFS		5.2	1.7	2.9	0.4	0.8	0.8	5.5	0.8	2.6
PMM2		5.8	1.9	2.4	0.1	0.7	1.0	4.9	0.2	3.7
ZNF646		5.6	2.2	2.9	0.6	1.1	1.5	3.2	0.7	2.9
METTL6		6.5	1.8	2.8	0.1	0.8	1.1	4.3	0.6	2.6
RTKN		5.3	2.2	2.6	0.6	1.2	1.5	3.8	0.6	2.9
HLX		4.1	1.6	2.0	1.0	1.5	2.2	3.2	1.0	4.2

P4HA2	8.0	3.1	3.6	0.2	0.7	0.8	2.2	0.4	1.7
TIMM21	5.6	2.2	2.8	0.4	0.7	1.5	2.9	0.6	4.0
CASP10	7.2	1.6	3.1	0.3	0.8	0.9	4.0	0.4	2.4
ITSN2	5.5	1.9	3.0	0.2	0.8	1.2	4.2	0.5	3.2
LPGAT1	5.6	2.2	2.9	0.2	0.9	0.8	4.3	0.4	3.3
DCAF16	4.4	1.3	2.1	0.5	1.1	1.8	4.5	0.7	4.2
IQSEC2	8.1	2.9	3.9	0.5	0.8	1.4	1.4	0.4	1.1
CCDC91	4.8	2.2	2.5	0.2	0.8	0.9	4.7	0.6	3.9
WDR37	5.8	2.0	2.5	0.6	1.0	1.6	3.8	0.6	2.6
LOC100507156	6.7	1.3	3.0	0.3	0.8	0.9	4.7	0.5	2.3
NFATC3	5.0	2.0	2.5	0.3	0.6	1.0	4.5	0.5	4.2
LOC101929159	6.7	1.1	2.9	0.4	0.8	0.9	5.4	0.3	2.1
IFT81	7.4	2.3	2.5	0.3	1.0	1.0	3.1	0.4	2.6
BICC1	6.7	2.3	2.9	0.3	1.1	1.3	2.7	0.4	2.8
KDM5A	6.2	2.0	2.9	0.2	0.8	0.9	4.5	0.4	2.7
PQLC2	5.4	2.7	2.6	0.5	1.1	1.6	3.4	0.7	2.6
ZNF362	6.2	2.3	2.7	1.3	1.1	1.8	2.2	0.7	2.3
FBXL7	4.6	2.2	2.3	0.8	1.1	1.8	4.2	0.7	2.9
TMEM131	5.8	2.0	2.6	0.5	1.2	1.5	3.3	0.6	3.0
RHBDD1	4.9	1.9	2.8	0.2	1.0	1.2	4.3	0.7	3.4
CBLL1	5.6	2.5	3.3	0.2	1.0	1.6	3.0	0.5	2.9
PEAK1	4.7	1.5	2.0	0.3	0.9	1.4	4.8	0.7	4.2
EXD2	5.7	2.4	3.1	0.3	0.8	1.0	3.8	0.5	2.8
ZNF333	5.8	2.1	2.8	0.3	1.1	1.4	3.7	0.4	2.8
ABHD12B	7.0	1.4	2.7	0.5	0.6	0.7	5.2	0.6	1.7
KIAA1324	6.6	1.3	3.1	0.4	0.8	1.0	4.9	0.4	2.1
TRMT10C	5.0	2.0	2.4	0.2	0.6	1.0	4.3	0.5	4.5
RTN4RL2	5.5	2.6	3.3	1.0	1.2	1.8	2.2	0.6	2.3
LOC101928024	5.7	1.8	2.4	0.6	1.2	1.3	4.6	0.5	2.5
FOXM1	4.7	1.9	2.3	0.8	1.8	2.2	3.1	0.9	3.0
PAN2	7.3	3.0	4.0	0.3	0.8	1.2	2.1	0.3	1.5
ISCA1	4.3	1.7	3.0	0.5	0.5	1.9	4.3	0.7	3.7
TTC13	6.0	2.0	3.1	0.3	0.8	1.1	4.1	0.5	2.6
MAP3K14	5.4	1.8	3.0	0.7	1.3	1.6	3.6	0.6	2.6
MBIP	6.3	3.0	3.9	0.2	0.8	1.3	2.2	0.3	2.6
BMP2K	5.5	1.7	2.4	0.3	0.9	1.1	4.1	0.8	3.6
EZH1	5.7	2.1	2.6	0.2	0.9	1.6	3.4	0.5	3.5
TTC28	5.5	2.0	2.5	0.5	1.1	1.4	3.5	0.7	3.2
LINC00921	6.4	1.3	2.9	0.5	0.8	0.9	5.0	0.4	2.2
PXN-AS1	4.8	2.5	2.3	0.5	1.2	2.3	4.4	0.5	1.9
PPAT	4.7	1.8	2.1	0.2	0.5	0.9	6.0	0.5	3.6
LOC101927185	5.9	1.8	3.3	0.4	1.1	1.0	4.8	0.3	1.8
LOC101929127	7.3	1.6	2.5	0.3	1.1	1.2	3.8	0.6	2.1
CC2D1B	4.6	1.7	2.4	0.7	1.3	1.7	4.1	0.7	3.2
ITFG1	6.5	2.3	3.0	0.4	1.0	1.6	2.8	0.4	2.5
ANKRD54	5.8	2.1	3.0	0.6	0.9	1.3	3.4	0.8	2.5
PTGES2-AS1	6.3	1.7	2.7	0.3	1.2	1.5	4.0	0.4	2.4
RAB18	4.8	2.2	2.3	0.2	0.5	1.2	4.5	0.4	4.4
C19orf26	4.7	2.0	2.8	0.8	1.2	1.3	3.7	0.9	3.0
LOC101927357	6.5	1.4	2.8	0.3	0.8	0.9	5.3	0.4	2.0
LRIG2	6.3	1.8	2.7	0.3	0.7	1.0	4.2	0.4	3.1
DHX35	5.7	2.2	2.8	0.3	1.1	1.1	4.1	0.4	2.8
CDKN1A	4.3	1.4	2.2	0.3	0.5	0.6	5.9	1.0	4.2
WDR41	5.3	1.8	2.7	0.2	0.8	1.1	4.6	0.6	3.2
RALGPS2	4.5	2.2	2.4	0.2	0.8	1.4	4.9	0.5	3.6

EMR1	6.2	2.5	3.1	0.2	0.6	0.9	3.9	0.5	2.5
DHDDS	6.2	1.8	2.7	0.6	1.0	1.1	3.5	0.5	3.1
CERS5	5.5	1.7	3.0	0.2	0.8	0.8	4.7	0.4	3.2
TBC1D10A	6.2	2.4	3.4	0.6	1.2	1.5	2.8	0.4	1.8
TRIM16L	5.9	2.5	3.7	0.5	1.4	1.3	2.9	0.3	1.9
ZSWIM7	6.1	1.9	3.5	0.5	1.1	1.5	2.2	0.3	3.2
C20orf96	6.2	2.7	3.6	0.4	0.7	1.9	1.9	0.5	2.4
ABHD14A	6.5	3.1	2.8	0.9	1.0	1.6	2.0	0.3	2.1
RBM12B	5.3	1.6	2.7	0.4	1.0	1.4	4.5	0.6	2.9
STYXL1	5.6	2.1	2.2	0.3	1.3	1.1	4.5	0.3	2.8
SNORD35A	3.9	1.2	3.1	1.7	1.7	1.1	4.5	0.6	2.5
CLK1	5.7	2.1	2.9	0.2	0.7	1.0	3.8	0.7	3.2
DPCD	5.9	2.7	3.3	0.4	1.6	1.5	2.8	0.5	1.6
VPS37A	4.6	2.1	2.7	0.3	0.8	1.4	3.9	0.8	3.7
VGF	5.5	2.1	2.6	0.6	0.8	1.3	3.9	0.8	2.7
PRDM10	6.3	1.7	2.5	0.5	1.0	1.0	3.5	0.8	2.8
DAK	5.9	1.8	3.1	0.8	1.4	1.9	3.2	0.4	1.8
ITGB2-AS1	5.4	2.6	3.8	0.4	1.1	1.4	2.7	0.5	2.3
UVRAG	5.0	1.5	2.5	0.2	0.6	0.9	5.3	0.7	3.6
NXPE3	6.5	2.0	3.2	0.2	0.7	1.2	3.7	0.5	2.2
HSPBAP1	7.2	2.3	3.3	0.1	0.6	0.8	3.0	0.4	2.6
CDH24	4.9	2.3	3.0	0.7	1.3	1.6	3.3	0.5	2.7
EED	5.0	2.3	2.5	0.4	0.9	1.2	4.0	0.6	3.5
DNHD1	7.3	1.3	3.5	0.5	0.9	1.0	3.4	0.2	2.0
USP20	5.4	2.1	3.0	0.9	1.2	1.8	3.4	0.3	2.2
GABPB1-AS1	6.0	1.5	2.6	0.6	0.9	1.3	4.5	0.5	2.3
VRK1	4.9	2.2	2.9	0.2	0.9	1.0	4.3	0.4	3.2
SLC16A5	6.0	2.6	3.1	0.7	1.8	2.1	2.0	0.3	1.7
ASH2L	6.4	2.2	3.0	0.4	1.1	1.3	3.4	0.5	1.9
C12orf29	4.1	1.6	2.3	0.1	0.8	1.0	5.2	0.5	4.6
TTC4	5.6	1.9	2.3	0.3	0.8	0.9	4.4	0.6	3.4
STXBP3	6.1	2.9	3.1	0.1	0.6	1.0	3.5	0.3	2.6
FLJ45256	6.3	1.2	2.9	0.4	1.0	0.9	4.9	0.5	2.0
SERTAD4	4.5	1.3	1.6	0.3	0.4	0.8	6.1	0.9	4.2
USP1	4.2	1.5	2.0	0.1	0.6	1.0	6.0	0.6	4.1
WRB	6.2	2.2	3.0	0.5	0.6	1.0	3.8	0.6	2.4
BNIP1	5.2	1.9	3.5	0.6	0.7	1.1	3.0	0.9	3.3
LOC101926960	6.9	1.4	2.9	0.3	0.7	0.8	4.8	0.4	2.0
SNORD100	3.1	1.4	1.7	1.6	2.4	1.1	1.2	2.5	5.2
EMC9	4.6	1.7	2.4	0.5	0.8	1.2	4.9	0.8	3.2
SNHG11	5.6	2.1	2.9	0.3	0.8	1.1	4.1	0.6	2.7
RANBP2	5.7	1.6	2.2	0.1	0.7	1.1	5.0	0.5	3.3
ARL5A	5.3	1.7	2.6	0.2	1.1	0.9	4.9	0.4	3.0
BAK1	5.0	2.1	3.1	0.5	1.3	1.2	3.4	0.6	3.1
NANP	5.9	1.6	2.3	0.3	0.6	1.2	4.1	0.6	3.6
BTRC	5.9	2.1	2.4	0.2	0.6	1.0	4.0	0.6	3.3
TRNAL7	5.3	1.4	1.9	0.2	0.4	1.0	6.3	0.3	3.1
C8orf49	6.3	1.3	3.0	0.3	0.8	0.9	5.1	0.4	2.0
LOC101928269	7.3	1.4	2.3	0.6	0.9	0.8	4.5	0.2	2.2
CDK11B	6.3	1.9	2.4	0.4	0.9	1.1	3.5	0.8	2.8
ARFIP1	5.9	2.4	3.0	0.4	0.7	1.0	3.1	0.6	3.0
BCL7A	4.6	2.1	2.2	0.8	0.9	1.1	4.2	0.7	3.5
PARD3B	6.4	2.0	2.6	0.3	0.5	0.9	3.6	0.7	3.2
LOC101928045	6.3	1.3	3.1	0.3	0.6	0.9	5.0	0.4	2.2
ARL4A	4.6	1.3	2.7	0.1	0.4	0.9	6.1	0.5	3.5

CBX7	4.8	2.2	3.9	1.1	1.3	2.1	2.4	0.5	1.8
BLOC1S6	4.9	1.9	3.1	0.4	1.1	1.0	4.2	0.5	3.0
KCTD9	5.2	1.5	2.9	0.2	0.9	0.8	4.5	0.6	3.5
RAB11B-AS1	5.1	2.3	3.4	0.7	1.7	1.7	2.5	0.6	2.1
ASNSD1	4.6	2.1	2.6	0.0	0.6	0.8	5.2	0.5	3.6
SIGLEC8	7.2	1.2	2.7	0.3	0.7	0.9	4.7	0.5	1.8
LOC101927302	4.6	1.7	2.5	0.5	1.0	1.2	5.2	0.5	3.1
PHKA1	5.7	1.5	1.8	0.3	0.8	0.9	4.9	0.6	3.4
ZNF653	6.9	2.2	3.2	0.8	1.4	1.1	2.2	0.5	1.8
HFE	6.4	1.8	2.9	0.4	0.9	1.0	4.1	0.4	2.1
INO80D	5.9	1.7	2.7	0.5	0.8	1.1	3.3	0.8	3.4
TMEM106B	6.2	2.7	2.6	0.2	1.2	1.5	2.6	0.3	2.7
MAGED1	6.6	2.5	3.3	0.7	1.4	2.2	1.7	0.4	1.2
PAPL	4.0	1.2	2.3	0.6	1.3	1.7	4.5	0.7	3.8
ARNT2	5.0	2.1	2.4	0.6	1.4	2.1	3.6	0.4	2.4
HELZ	5.7	2.0	2.6	0.3	0.9	1.2	3.7	0.6	3.1
JARID2	5.2	1.7	2.6	0.7	1.2	1.7	3.8	0.6	2.5
C10orf76	6.3	2.2	2.9	0.4	1.1	1.5	2.8	0.5	2.2
SPATA5L1	5.1	2.2	2.7	0.6	0.8	1.1	5.1	0.5	2.1
PAXBP1-AS1	6.4	1.6	2.9	0.4	0.7	0.9	4.5	0.4	2.2
SRR	5.6	1.9	2.6	0.2	1.0	1.0	3.2	0.7	3.9
NQO2	6.4	2.2	2.8	0.6	1.3	1.3	3.3	0.3	1.7
LOC101928103	7.1	1.4	3.4	0.2	0.9	1.0	3.3	0.3	2.3
DNAJC4	6.6	3.0	2.6	0.8	1.2	1.7	2.1	0.3	1.7
MYH10	4.9	1.5	2.2	0.3	1.1	1.3	4.8	0.6	3.3
FN3KRP	5.2	1.7	2.9	0.3	1.3	1.1	4.1	0.6	2.8
EARS2	5.6	1.8	2.5	0.3	0.8	1.1	4.1	0.7	3.1
ROCK1	5.2	1.8	2.2	0.1	0.7	0.8	4.6	0.7	3.9
FAM196B	3.6	1.2	1.6	0.7	1.5	2.6	4.0	0.9	3.8
SLC9A7	5.3	2.0	2.8	0.5	1.2	1.2	3.5	0.7	2.7
WDR20	5.2	2.0	2.5	0.2	1.0	1.1	3.5	0.6	3.6
MBOAT1	6.3	1.5	3.3	0.3	0.6	0.9	4.2	0.5	2.4
VCPIP1	5.4	1.8	2.4	0.3	0.8	1.2	4.1	0.6	3.4
PSRC1	6.4	2.3	2.8	0.5	1.3	1.3	2.4	0.6	2.4
KCTD21	5.3	2.3	3.1	0.8	0.8	2.1	2.8	0.7	2.1
LOC101927312	7.0	1.3	3.0	0.3	0.8	1.1	3.3	0.2	3.0
RAD54L2	5.6	1.8	2.6	0.3	0.8	1.0	3.9	0.7	3.2
SPTY2D1	5.4	2.5	2.4	0.2	0.7	0.9	3.8	0.6	3.3
MCTS1	4.4	2.2	3.1	0.1	0.8	1.0	4.1	0.4	3.8
ICAM5	4.5	1.8	2.8	1.1	1.6	2.4	3.1	0.5	2.2
ATG16L1	5.1	2.0	2.8	0.3	0.7	1.3	4.3	0.7	2.8
AFAP1-AS1	5.5	1.8	2.4	0.4	0.9	1.1	4.0	0.7	3.1
KAL1	3.3	1.0	1.5	0.6	1.5	2.3	5.0	1.0	3.6
MEGF8	7.0	2.3	3.1	0.8	1.4	1.8	2.1	0.3	1.2
MIR22HG	4.4	1.9	2.7	0.3	0.8	1.1	4.7	0.5	3.4
ATP5J2-PTCD1	5.4	1.9	2.9	0.5	1.0	0.7	3.7	0.5	3.4
DHX29	5.1	1.9	2.4	0.2	0.7	1.0	4.3	0.6	3.7
ACOT9	5.5	2.0	2.5	0.2	1.1	1.5	3.6	0.4	3.2
POLG2	4.8	2.0	2.9	0.4	0.8	1.0	4.6	0.6	2.7
SNORD104	5.7	1.8	1.7	0.5	1.8	1.8	3.5	0.7	2.4
KCTD1	5.3	2.0	2.6	0.7	1.2	1.5	3.3	0.7	2.6
PPWD1	6.1	2.1	2.7	0.3	0.9	1.1	3.2	0.6	2.8
MAP3K12	5.7	2.0	3.3	0.4	1.0	1.3	3.3	0.6	2.2
TECPR1	6.7	2.1	3.0	0.6	1.1	1.4	2.7	0.4	1.7
APBB3	5.8	2.3	3.2	0.6	1.0	1.6	2.8	0.4	2.1

LOC101929591	6.3	1.3	2.8	0.4	0.8	0.9	4.4	0.5	2.4
REEP6	6.9	2.9	3.7	0.8	1.3	2.0	1.1	0.3	0.9
IMPA2	5.7	2.3	3.3	0.4	0.8	1.3	3.3	0.6	2.1
RAP1GAP2	4.6	2.0	2.4	0.5	0.8	1.6	3.3	0.7	3.8
AGPAT9	4.6	1.9	2.3	0.3	1.0	1.1	4.7	0.7	3.2
LOC100507033	6.5	1.2	2.5	0.5	0.7	1.0	4.8	0.3	2.2
ABTB2	5.6	2.1	2.6	0.6	1.2	1.7	3.1	0.5	2.2
HELZ2	5.5	2.0	2.7	0.9	1.3	1.9	3.1	0.5	1.9
ELP2	5.2	2.0	2.6	0.3	0.6	1.2	4.5	0.4	3.0
MRAS	5.4	2.5	3.2	0.4	1.0	1.4	2.4	0.7	2.8
WNT5B	4.6	2.1	2.7	0.7	0.8	1.1	4.8	0.5	2.4
DUSP22	5.5	2.3	3.3	0.8	0.9	1.7	2.0	0.5	2.7
STRN3	5.8	1.9	3.0	0.3	0.7	1.2	3.7	0.5	2.6
GLCE	4.7	2.2	2.9	0.3	0.6	0.9	4.3	0.5	3.3
PPIE	6.6	2.0	2.2	0.3	1.2	1.2	3.4	0.4	2.5
BCOR	5.2	1.8	2.3	0.4	0.9	1.5	3.4	0.7	3.6
TEN1-CDK3	5.5	2.1	3.2	0.5	1.0	1.2	3.1	0.6	2.6
CD163L1	5.2	1.5	2.3	0.2	0.6	0.9	5.1	0.5	3.3
KCTD14	5.7	1.4	2.6	0.3	0.7	1.3	4.1	0.5	3.3
EIF2AK2	5.0	1.9	2.3	0.2	0.5	1.0	4.5	0.5	3.8
PIAS1	6.1	2.3	2.4	0.3	0.7	1.4	2.7	0.7	3.2
LOC100131691	6.5	2.0	2.9	0.5	1.0	1.2	3.5	0.4	1.9
USP6NL-IT1	5.9	1.4	3.0	0.2	0.7	1.0	4.7	0.4	2.3
CFLAR	5.7	1.7	2.8	0.3	0.9	1.1	3.9	0.3	2.9
ZNF559	5.9	1.5	3.2	0.3	1.1	0.9	4.3	0.4	2.1
LOC101927802	6.8	1.4	2.8	0.3	0.9	1.0	3.9	0.4	2.3
ATXN7L2	4.9	2.3	2.8	0.5	1.0	1.1	3.7	0.7	2.7
PHKG2	5.1	1.9	3.5	0.5	1.2	1.1	3.6	0.4	2.4
MUTYH	5.3	2.0	2.6	0.6	1.2	1.2	3.5	0.5	2.8
STX2	5.8	2.1	2.8	0.2	1.0	1.2	3.7	0.5	2.5
EXOC6B	5.4	1.9	2.5	0.3	0.9	1.2	4.3	0.4	2.9
AMH	5.5	2.3	3.0	0.7	1.1	1.3	3.0	0.6	2.0
MGAT5	5.5	1.8	2.5	0.3	0.9	1.2	3.9	0.4	3.2
SETMAR	6.6	1.8	2.5	0.3	1.2	1.0	3.5	0.5	2.3
NAB2	5.1	1.9	2.4	0.4	0.9	1.4	4.2	0.7	2.8
GTF2H2C	6.4	1.3	1.9	0.2	0.7	1.1	4.7	0.4	3.0
LBX2	5.6	1.5	2.4	0.8	1.3	1.8	3.9	0.5	1.7
TMEM45A	4.9	2.3	2.7	0.1	0.2	0.6	4.9	0.6	3.4
CC2D2A	5.6	2.6	2.3	0.3	1.3	1.4	2.2	0.5	3.5
LOC101928173	6.3	1.5	2.9	0.6	0.7	1.0	3.7	0.4	2.6
MGME1	5.8	1.9	3.1	0.4	1.2	1.2	3.1	0.3	2.9
ATXN1	6.3	2.5	3.2	0.6	0.9	1.1	2.5	0.5	2.0
MRPS33	5.5	1.5	2.5	0.2	1.3	2.6	2.7	0.7	2.8
LINC00330	6.0	1.2	3.1	0.4	0.7	0.8	5.0	0.5	2.1
NOL12	4.9	2.2	2.0	0.5	1.3	1.4	3.8	0.7	2.8
UBR7	4.8	2.0	2.4	0.4	1.1	1.7	3.9	0.7	2.6
CREM	6.2	2.0	2.5	0.3	0.9	0.9	4.4	0.5	2.0
ZNF770	4.9	1.7	2.0	0.2	0.8	1.0	4.3	0.6	4.1
SNRNP25	5.1	1.5	2.7	0.6	0.6	1.8	4.3	0.6	2.5
LACTB2	5.3	2.3	3.4	0.3	1.3	1.2	2.9	0.3	2.5
CCDC28A	5.1	2.3	2.9	0.4	0.9	1.0	3.1	0.4	3.6
RSBN1	6.1	2.0	3.0	0.4	0.9	1.0	3.5	0.5	2.3
C19orf71	4.3	1.8	1.6	0.6	1.9	2.2	4.0	0.5	2.6
ZFX	5.1	1.7	3.0	0.1	0.7	1.1	4.6	0.6	2.7
LOC101928887	6.4	1.2	2.9	0.4	0.8	0.9	4.3	0.4	2.3

ADD3	5.8	2.3	3.2	0.3	1.0	1.3	2.8	0.5	2.2
BIRC6	5.5	1.5	2.4	0.2	0.8	0.9	4.4	0.5	3.4
LOC101929716	6.3	2.1	2.6	0.4	1.0	1.2	3.5	0.2	2.3
DRAM2	4.7	2.6	3.4	0.2	0.6	1.1	3.5	0.4	3.1
LOC100130872	6.2	1.3	2.7	0.3	0.8	1.0	4.8	0.3	2.2
LIMS1	5.4	1.6	2.5	0.3	0.9	0.7	4.8	0.7	2.7
NVL	5.6	2.2	2.7	0.2	0.8	1.2	3.5	0.4	2.9
LOC442075	6.1	1.4	2.8	0.4	0.8	1.0	4.5	0.4	2.3
USP15	4.7	1.7	2.5	0.1	1.0	0.8	4.4	0.5	3.8
SCRN2	5.7	1.8	3.0	0.8	1.4	1.8	2.9	0.4	1.8
TNFRSF14	5.7	2.8	3.4	0.7	1.0	1.3	2.4	0.4	1.9
CBX8	5.6	1.7	3.0	0.3	0.8	1.1	3.6	0.7	2.8
TRIM5	5.6	1.6	3.1	0.3	0.6	0.9	4.4	0.4	2.6
RERG	7.4	2.1	3.4	0.2	0.5	0.7	2.7	0.7	1.9
L3MBTL2	5.1	2.0	2.6	0.4	0.9	1.0	4.2	0.5	2.9
NPAT	5.0	1.8	2.4	0.2	0.9	1.3	3.8	0.5	3.7
IL17RA	5.8	2.4	2.8	0.5	0.8	1.2	3.3	0.6	2.2
MLF1	4.7	2.0	3.6	0.2	1.0	1.4	4.0	0.4	2.2
LOC101929132	6.1	1.4	3.0	0.4	0.8	0.8	4.8	0.5	1.8
DNAJC17	5.0	2.4	2.9	0.4	0.8	1.5	3.2	0.4	2.9
RHNO1	5.6	2.1	2.9	0.5	0.9	1.6	3.3	0.4	2.4
PPP3CB	6.4	2.4	2.7	0.4	1.0	1.6	2.8	0.4	2.0
MOB4	5.1	2.1	2.9	0.3	1.1	1.1	3.2	0.3	3.3
MIS12	4.2	1.9	2.2	0.1	0.5	0.5	5.0	0.6	4.4
NCBP1	4.9	1.5	2.3	0.2	0.9	1.4	4.2	0.5	3.6
STARD4	5.9	2.4	3.6	0.1	0.4	0.5	3.9	0.2	2.5
USP53	5.2	2.0	2.1	0.1	0.6	0.7	5.0	0.5	3.3
MMGT1	6.8	1.8	2.9	0.1	1.3	1.5	2.6	0.2	2.2
LRP12	6.7	2.0	2.8	0.2	0.7	0.9	3.3	0.4	2.5
GLA	5.4	2.0	2.7	0.2	0.9	1.0	4.0	0.5	2.8
CENPO	4.9	1.4	2.1	0.4	1.1	1.1	4.4	0.6	3.3
APOL6	6.1	1.9	2.2	0.3	1.1	1.3	3.5	0.5	2.6
LOC100507283	6.1	1.3	2.8	0.5	0.8	1.1	4.8	0.3	1.8
LINC00578	7.2	1.3	2.9	0.3	0.7	0.9	4.2	0.3	1.7
AGTPBP1	5.2	2.0	2.5	0.2	0.9	1.3	3.9	0.4	3.1
SGMS2	6.0	2.0	3.2	0.2	0.7	1.0	3.1	0.6	2.7
LOC101929301	6.6	1.5	2.9	0.6	1.1	0.6	3.8	0.4	1.9
OSR1	5.6	2.8	2.9	0.8	1.1	1.6	2.1	0.4	2.0
PDE4D	5.9	2.1	2.7	0.3	0.7	1.0	3.5	0.5	2.7
MBD4	5.1	1.9	2.5	0.3	0.6	1.3	4.3	0.4	3.1
C17orf75	5.3	2.4	3.1	0.5	0.9	1.5	2.8	0.4	2.5
HTT	5.6	1.9	2.7	0.6	1.1	1.5	3.1	0.6	2.3
PRUNE	5.7	1.8	2.5	0.3	0.6	1.0	3.9	0.9	2.7
TRMT2B	6.8	2.0	2.2	0.3	0.9	1.3	3.3	0.3	2.1
C14orf105	5.8	1.2	3.0	0.3	0.9	0.8	5.0	0.3	2.1
RPS6KA1	6.1	1.9	2.3	0.4	1.0	1.0	3.6	0.6	2.5
LOXL1-AS1	5.2	1.6	2.5	0.4	0.9	1.2	4.3	0.4	2.8
R3HDM1	5.1	1.9	2.7	0.4	1.0	1.2	3.0	0.6	3.5
YIPF1	6.0	2.0	3.1	0.2	0.9	1.6	3.0	0.4	2.3
SMARCA1	5.6	2.0	2.5	0.2	0.8	1.0	4.0	0.5	2.8
PSMD5-AS1	5.6	1.5	2.8	0.3	0.8	0.9	4.8	0.4	2.3
PIK3CA	4.9	1.5	2.4	0.1	0.7	0.7	5.2	0.6	3.3
MDP1	5.7	1.8	3.3	0.0	0.9	1.1	4.0	0.6	1.9
BIN3-IT1	6.8	1.2	2.7	0.4	0.8	0.9	4.3	0.3	1.8
SIAE	6.4	2.1	3.3	0.4	0.9	1.5	2.5	0.3	2.1

MTIF3	5.1	2.1	2.8	0.1	0.5	1.4	3.5	0.4	3.4
PARP14	6.4	2.2	2.8	0.2	0.8	1.0	3.2	0.4	2.3
LOC100506714	5.4	2.0	3.0	0.4	1.1	1.2	3.5	0.4	2.2
LOC100289511	3.8	1.5	2.2	0.5	0.6	1.2	5.6	0.7	3.2
VPS8	6.5	2.2	3.2	0.2	0.6	1.1	2.9	0.4	2.3
SAPCD2	3.8	1.4	1.6	0.8	0.7	0.9	6.1	1.0	3.0
ABCD3	4.8	2.8	2.6	0.3	0.6	1.3	3.6	0.4	3.0
NKAP	5.9	2.0	3.1	0.5	1.0	1.2	3.2	0.4	2.0
NFX1	5.2	1.6	2.4	0.3	0.8	1.1	4.2	0.5	3.2
FASTKD5	5.6	2.2	2.8	0.2	0.7	0.8	3.7	0.6	2.8
MLKL	4.6	1.9	2.3	0.3	1.4	1.8	3.9	0.5	2.6
AMMECR1	4.7	1.1	2.5	0.4	0.6	1.3	4.7	0.9	3.1
CUL7	4.9	1.8	2.9	0.7	1.6	1.8	3.0	0.4	2.1
AAED1	6.1	1.9	2.8	0.4	0.7	0.9	3.0	0.4	2.9
LHFPL2	6.2	1.9	2.7	0.2	0.9	1.2	3.2	0.5	2.3
SNORA4	4.6	1.8	1.4	0.1	0.8	1.0	4.6	0.6	4.3
LINC00973	3.8	2.4	2.3	0.3	0.7	1.0	4.6	0.5	3.7
ZC3H7A	4.7	1.8	2.2	0.2	0.8	1.2	4.6	0.6	3.2
INTS7	5.2	1.7	2.5	0.2	0.5	0.9	4.5	0.5	3.2
ALKBH1	5.6	2.0	2.9	0.3	0.7	1.0	3.7	0.7	2.3
LOC101927919	6.7	2.1	3.2	0.2	0.4	0.8	3.7	0.4	1.7
LOC100507462	6.8	1.5	2.5	0.6	0.6	0.4	4.5	0.5	1.8
SACM1L	5.7	1.8	2.5	0.1	0.8	1.1	4.0	0.5	2.7
FBXO3	6.7	1.8	3.1	0.3	0.7	0.8	3.1	0.5	2.1
GYG1	5.1	2.2	3.2	0.3	0.8	1.3	3.0	0.6	2.7
ZSWIM4	5.5	1.9	2.9	0.8	1.2	1.7	2.6	0.5	1.9
ANKRD49	5.0	2.3	2.9	0.1	0.9	0.4	4.0	0.5	3.0
ADAT3	4.5	1.6	2.1	1.0	0.8	1.2	4.2	1.0	2.8
RSPH3	4.9	1.6	2.6	0.6	1.0	1.3	3.7	0.5	3.0
STRIP2	5.6	2.5	3.3	0.3	0.9	1.1	3.1	0.2	2.1
ATG5	4.8	1.9	2.6	0.2	0.3	0.5	4.2	0.5	4.0
TNRC6A	5.7	1.8	2.8	0.3	0.8	1.0	3.1	0.5	3.2
TLR4	5.0	1.6	2.6	0.1	0.6	0.8	4.3	0.5	3.6
SETX	5.5	1.6	2.5	0.2	0.6	1.0	4.2	0.4	3.2
NHLRC2	5.2	1.6	2.6	0.3	1.0	1.2	3.5	0.6	3.0
CRX	6.4	1.3	2.8	0.4	0.8	1.0	4.2	0.3	1.9
CD82	6.4	2.7	3.1	0.7	1.7	2.3	1.1	0.2	1.0
CARS2	5.1	1.8	2.9	0.3	0.8	1.2	3.5	0.5	2.9
RHOBTB2	4.7	1.8	2.4	0.8	1.1	1.4	3.5	0.8	2.7
MDN1	5.1	1.2	2.3	0.3	0.9	1.0	4.6	0.5	3.1
LOC101928847	6.6	1.2	2.7	0.3	0.8	0.8	4.3	0.4	2.2
PTAR1	5.2	1.7	2.9	0.2	1.0	1.1	3.7	0.5	2.8
TMEM42	5.7	2.4	3.3	0.6	1.4	1.6	2.3	0.3	1.3
LOC101928238	6.1	1.2	2.4	0.4	0.9	1.3	4.5	0.4	1.8
SRGAP2	5.4	1.8	2.6	0.4	0.9	1.2	3.4	0.5	2.7
ATPAF1	5.5	2.2	3.1	0.2	0.6	1.1	4.1	0.4	1.9
AURKA	5.7	2.8	2.7	0.3	0.9	1.0	2.4	0.4	2.7
LOC729887	5.9	2.5	2.8	0.8	1.0	1.9	2.1	0.3	1.7
CKAP2	5.4	2.1	2.6	0.2	0.9	1.2	3.3	0.5	2.9
SSH3	6.2	2.7	3.3	0.7	1.0	1.7	1.7	0.4	1.4
LOC101928029	6.3	1.1	2.4	0.3	0.7	0.9	4.9	0.4	2.1
LOC100133286	4.9	2.4	2.5	0.9	1.2	2.2	2.2	0.6	2.0
SEMA4D	5.9	1.8	2.8	0.5	0.8	1.0	3.3	0.6	2.3
GPR39	4.4	1.5	2.1	0.9	1.8	2.3	3.1	0.6	2.2
SUMF1	6.3	1.9	3.2	0.6	1.7	2.0	2.2	0.2	1.0

NDST2	5.0	2.2	2.7	0.5	1.1	1.5	3.0	0.4	2.4
CPED1	4.5	1.6	2.5	0.4	1.0	1.3	3.9	0.6	3.2
DNAL4	5.5	2.1	3.6	0.5	1.4	0.6	2.7	0.3	2.3
MED11	4.8	2.1	3.3	0.5	1.3	1.6	3.2	0.4	1.8
CD2AP	4.5	1.7	2.1	0.1	0.9	1.0	4.6	0.7	3.5
THEMIS	6.0	2.0	2.8	0.4	0.5	0.5	4.6	0.4	1.7
SMG8	4.6	1.6	2.0	0.3	0.8	1.1	4.2	0.7	3.6
LRP1	6.6	1.9	3.0	0.8	1.3	1.6	2.1	0.3	1.3
STK39	5.5	2.1	2.8	0.3	0.8	0.6	3.9	0.4	2.5
AGO1	5.1	1.8	2.4	0.4	1.0	1.1	3.5	0.7	3.0
IFI30	5.4	2.4	3.1	0.5	1.2	1.1	2.7	0.4	2.2
C19orf55	4.4	1.9	2.1	0.9	1.3	1.8	3.4	0.6	2.6
ZNF394	4.9	1.4	2.0	0.2	1.2	1.1	5.6	0.5	2.0
LOC101927844	6.5	1.3	2.2	0.4	0.8	1.0	4.7	0.2	1.8
ISYNA1	6.0	2.2	3.5	0.6	1.1	1.3	2.5	0.4	1.4
RUNDC3A	6.7	1.3	2.7	0.3	0.8	0.7	4.2	0.4	1.8
HIST1H2BM	2.6	1.1	2.3	1.2	2.1	4.1	1.9	0.6	3.0
GPC1	6.0	2.2	3.2	0.9	1.1	1.5	2.2	0.4	1.4
AGO3	5.5	1.5	2.4	0.2	0.5	0.7	4.7	0.4	3.1
PLS1	5.7	2.1	2.7	0.2	0.7	0.8	3.8	0.3	2.7
MAP3K7CL	2.5	1.2	1.1	0.2	0.5	0.4	8.3	0.4	4.3
ZNF689	5.0	1.6	2.2	0.4	0.9	1.3	3.4	0.9	3.2
SERF1A	5.2	2.0	3.2	0.2	0.9	1.1	3.9	0.4	2.1
SERF1B	5.2	2.0	3.2	0.2	0.9	1.1	3.9	0.4	2.1
CCBL2	5.0	1.7	2.6	0.1	0.6	1.1	4.1	0.5	3.2
PHF17	4.6	1.3	1.9	0.2	0.7	0.9	5.1	0.5	3.6
MYO6	5.2	1.7	2.3	0.3	0.8	1.2	4.1	0.4	2.9
CAPN10	5.3	1.5	3.0	0.7	1.3	1.5	3.0	0.4	2.1
SUCLA2	4.8	2.1	3.0	0.2	0.5	0.7	4.1	0.5	3.0
URGCP	5.3	2.0	2.3	0.4	1.1	1.2	3.7	0.4	2.4
IRX3	4.4	1.9	2.7	0.7	1.0	1.7	2.9	0.9	2.9
BEND3	4.5	1.3	1.8	0.5	0.9	1.3	4.7	0.6	3.3
MALT1	4.8	1.9	2.3	0.2	0.6	0.9	4.7	0.5	3.0
CHUK	5.1	1.7	2.4	0.1	0.5	0.6	4.7	0.5	3.3
EPHX1	6.4	2.4	3.3	0.5	1.0	1.5	1.9	0.4	1.4
SENP6	5.2	1.5	2.4	0.3	0.7	0.8	4.1	0.5	3.4
LY6G5B	4.6	1.7	2.6	0.3	0.6	1.9	3.8	0.7	2.7
DHRS4-AS1	6.2	1.6	2.9	0.3	0.8	0.9	3.8	0.3	2.0
DSN1	4.9	1.7	2.2	0.3	1.1	0.9	3.7	0.6	3.5
ZNF277	6.1	1.6	3.2	0.2	0.5	0.9	3.2	0.5	2.7
CREB1	3.9	1.8	2.2	0.5	1.6	0.9	3.2	1.1	3.6
RGS14	4.6	1.7	2.3	0.5	1.1	1.3	3.9	0.4	3.0
CYP2U1	6.7	2.7	2.9	0.4	1.0	1.3	2.0	0.5	1.3
LOC400655	6.0	1.6	2.6	0.4	0.8	1.0	3.6	0.6	2.2
CRIPAK	5.6	1.3	2.2	1.2	1.3	1.4	3.7	0.5	1.6
FAM210A	5.0	1.8	2.8	0.2	0.5	0.8	4.2	0.6	3.1
SGSM3	5.7	2.4	3.1	0.5	1.1	1.0	2.4	0.4	2.1
LINC00346	6.2	1.3	2.9	0.2	0.9	1.1	3.8	0.3	2.2
C18orf32	5.4	3.1	3.9	0.1	0.5	0.9	2.0	0.4	2.4
SNX30	5.8	1.8	2.0	0.3	0.8	1.0	3.5	0.6	2.9
LDLRAP1	5.5	2.6	2.3	0.6	1.1	1.9	2.2	0.5	2.2
AKR1C3	7.4	2.3	4.1	0.2	1.0	1.2	1.2	0.1	1.2
MYNN	3.7	1.7	2.8	0.2	0.9	1.0	5.0	0.7	2.8
TLCD1	4.2	1.5	1.9	0.9	1.0	1.7	3.6	0.7	3.3
IFIT3	5.4	2.7	2.5	0.1	0.8	0.6	3.4	0.4	2.8

MEX3A	5.0	1.5	2.8	0.7	1.3	1.3	2.9	0.8	2.5
TOR3A	4.5	1.6	2.5	0.4	1.2	0.9	4.1	0.5	3.1
LOC92249	5.8	1.9	2.4	0.4	0.9	1.6	2.8	0.5	2.5
CCNYL1	3.9	1.7	1.9	0.8	0.8	1.5	4.0	0.6	3.6
XAF1	6.4	1.3	2.8	0.2	0.8	1.0	3.7	0.3	2.2
KBTBD4	5.1	2.2	3.0	0.3	0.8	1.2	3.0	0.4	2.8
NRBP2	5.3	2.0	3.0	0.9	1.5	2.2	1.9	0.5	1.5
CLSTN3	5.9	2.2	3.4	0.6	0.8	1.0	2.5	0.4	2.0
AMPH	5.2	1.7	2.5	0.2	0.8	0.9	3.6	0.6	3.0
TMEM116	4.2	1.5	2.6	0.5	0.8	0.9	4.0	0.8	3.5
TSEN2	4.6	1.4	2.1	0.4	1.2	1.4	3.5	0.4	3.6
AKTIP	3.9	1.8	2.4	0.2	1.1	1.5	3.9	0.6	3.4
LOC101928716	5.6	1.1	2.6	0.2	0.7	0.9	5.1	0.3	2.1
SNORA20	3.9	1.3	2.4	0.6	0.8	0.9	5.6	0.6	2.6
NLRP1	5.5	2.1	2.8	0.4	0.7	1.0	3.0	0.4	2.8
UHRF1BP1	5.4	1.7	2.5	0.3	0.8	1.2	3.6	0.4	2.9
WDR70	4.5	1.8	2.7	0.4	1.1	1.3	3.8	0.5	2.6
LRBA	5.6	1.7	2.6	0.2	0.8	1.0	3.8	0.5	2.5
LOC101928672	3.4	1.5	2.1	0.6	1.2	1.1	4.3	0.6	3.9
RACGAP1	5.0	2.1	2.5	0.3	0.9	1.2	3.6	0.5	2.6
IFITM10	6.0	2.3	1.9	0.6	0.5	1.2	3.0	0.5	2.6
LOC728975	6.6	1.2	2.5	0.3	0.7	0.7	4.5	0.4	1.7
CALHM2	5.0	1.6	2.4	0.7	1.5	2.3	2.7	0.4	2.0
NUDT1	4.6	2.0	2.2	0.6	1.2	1.3	3.6	0.4	2.7
DESI2	4.6	1.9	2.7	0.4	0.5	0.8	3.4	0.7	3.7
FANCG	4.8	1.6	2.4	0.4	1.1	1.2	3.9	0.6	2.6
KIAA1875	6.2	1.3	2.8	0.4	0.7	0.9	4.1	0.3	2.0
LOC101927157	6.3	1.3	2.6	0.4	0.9	1.0	3.4	0.3	2.3
AKAP17A	4.7	1.8	2.1	0.6	0.8	1.2	3.9	0.7	2.8
AKAP17A	4.7	1.8	2.1	0.6	0.8	1.2	3.9	0.7	2.8
LOC101929181	6.3	1.5	2.9	0.4	0.8	0.9	3.1	0.4	2.5
AMER1	4.5	1.4	1.9	0.5	1.0	1.2	4.1	0.7	3.3
GPD2	5.5	1.7	2.6	0.2	1.0	0.8	3.9	0.4	2.4
TMEM55A	6.1	1.6	3.0	0.3	0.8	1.2	2.8	0.2	2.5
ARID5B	5.9	2.1	2.9	0.3	0.7	1.0	2.7	0.5	2.4
MRPL39	4.6	2.2	2.8	0.2	0.7	1.1	3.6	0.5	2.9
FBXO33	5.8	1.9	2.7	0.4	0.9	1.0	2.8	0.6	2.5
SLC39A8	4.9	2.0	3.0	0.1	0.5	0.9	3.8	0.5	2.7
LINC00483	6.4	1.4	1.9	0.5	0.5	0.9	4.5	0.3	2.1
FGFR1OP	6.0	1.8	2.6	0.2	0.7	0.8	4.1	0.6	1.7
VPS36	3.8	1.5	1.8	0.2	1.3	1.1	4.7	0.5	3.7
KLHL17	6.0	2.2	3.4	0.5	1.0	1.2	2.0	0.5	1.7
ING5	5.0	2.1	2.1	0.4	0.6	0.9	3.3	0.6	3.6
DDX28	3.9	1.7	1.8	0.7	1.2	1.5	3.6	0.9	3.4
FARS2	5.3	2.1	3.1	0.4	1.0	1.4	2.6	0.4	2.3
ANAPC15	4.2	2.0	3.2	0.6	1.2	1.1	3.2	0.3	2.6
TMEM51	4.8	1.5	1.7	0.4	0.7	1.5	3.8	0.4	3.8
LOC101929047	5.4	1.9	2.8	0.1	0.7	1.5	3.6	0.5	2.1
ZSCAN12	5.8	1.2	2.4	0.4	0.9	0.8	4.6	0.3	2.1
ATF7	6.1	1.9	2.7	0.4	0.8	1.2	2.3	0.5	2.6
NLRC3	6.3	1.2	2.5	0.3	0.8	0.8	4.4	0.3	1.9
MED18	4.6	2.4	2.2	0.3	1.1	1.3	3.3	0.5	2.9
C11orf30	5.1	1.8	2.5	0.3	0.8	1.2	2.6	0.6	3.5
TRIM52	4.9	2.1	2.1	0.3	0.9	1.2	3.7	0.4	2.8
SIX2	4.5	2.4	2.4	0.4	1.1	1.3	2.4	0.8	3.2

AASS		3.7	1.1	1.7	0.3	1.2	1.4	4.9	0.6	3.6
METTL21B		5.8	1.9	2.3	0.5	1.2	1.1	2.9	0.4	2.4
SECISBP2L		5.5	1.7	2.6	0.2	0.8	1.1	3.0	0.4	3.1
C6orf141		5.4	2.3	4.1	0.6	0.9	1.0	2.3	0.3	1.5
PAQR3		4.8	2.1	2.4	0.5	0.9	1.2	3.3	0.6	2.6
C1D		5.5	2.6	2.9	0.0	0.6	0.7	3.1	0.3	2.8
ZNF652		5.7	1.9	2.2	0.4	0.9	1.4	2.9	0.5	2.6
CWF19L1		4.6	2.0	2.3	0.3	0.9	0.9	4.1	0.4	3.0
PID1		3.7	1.9	1.7	0.5	1.1	2.0	3.5	0.6	3.4
KIAA1598		5.4	1.3	2.5	0.2	0.8	1.2	3.5	0.4	3.1
TCF7L2		4.9	1.8	2.5	0.5	1.4	1.4	2.2	0.7	2.9
LINC00960		4.5	1.6	2.3	0.3	0.9	1.2	4.2	0.5	3.0
C1GALT1		4.5	2.0	2.9	0.1	0.7	1.0	3.6	0.4	3.2
COG5		4.8	1.5	2.2	0.3	0.8	1.4	3.6	0.6	3.1
PHF14		5.3	1.6	2.3	0.3	0.7	1.0	3.6	0.5	3.2
NFIL3		4.6	1.8	2.4	0.1	0.4	0.6	4.1	0.6	3.9
TRIM32		4.1	1.8	2.4	0.3	1.0	1.2	4.1	0.5	3.0
LOC100130557		6.0	1.5	2.6	0.4	0.8	0.9	3.9	0.4	2.0
IPO8		5.0	1.7	2.4	0.2	0.7	1.0	3.7	0.4	3.2
	44628.0	5.4	2.0	2.4	0.5	0.9	0.9	3.1	0.5	2.5
CA11		5.8	2.7	3.5	0.5	0.9	1.5	2.0	0.2	1.2
LOC101927663		5.3	2.0	2.4	0.7	1.4	1.4	3.1	0.3	1.8
NIT2		6.0	2.0	2.5	0.2	0.9	1.7	2.4	0.3	2.4
TBC1D12		4.8	1.7	2.0	0.4	0.9	0.8	4.5	0.6	2.6
CRAMP1L		5.0	1.5	2.5	0.5	0.7	1.2	3.7	0.6	2.5
FBXL4		5.7	1.6	2.8	0.3	0.8	0.9	3.5	0.3	2.6
VPS9D1		5.2	2.4	3.3	0.9	1.4	1.8	1.8	0.4	1.2
SPATA5		4.3	1.3	1.9	0.3	0.7	0.9	4.8	0.5	3.4
SAP30		4.5	1.3	2.6	0.3	1.0	1.2	3.8	0.4	3.3
ANKRD39		5.4	1.7	2.7	0.6	1.3	1.1	3.0	0.4	2.1
ST7		4.6	1.5	2.1	0.2	0.7	1.0	4.0	0.6	3.6
ZBTB21		4.3	1.6	2.0	0.3	0.7	1.1	4.0	0.7	3.6
LINC00883		4.5	1.8	2.1	0.2	1.0	1.1	4.3	0.5	2.8
PLEKHG3		5.4	1.7	2.3	0.5	0.8	1.2	3.0	0.5	2.9
CCS		4.5	2.2	2.8	0.7	1.1	1.7	2.6	0.6	2.1
NPIPB4		5.5	1.0	2.1	0.4	1.2	1.2	4.5	0.2	2.3
ZBTB9		4.7	1.6	2.1	0.4	1.0	1.1	3.5	0.7	3.3
EIF5A2		4.6	2.1	3.3	0.2	0.5	0.8	3.2	0.6	3.1
NUP35		4.8	1.7	2.3	0.2	0.5	0.5	4.2	0.6	3.6
PART1		6.4	1.2	2.4	0.4	0.6	0.7	4.4	0.3	1.9
NBPF3		5.2	1.9	2.5	0.3	0.6	0.8	3.9	0.5	2.7
GPC6		6.4	2.0	3.1	0.3	0.7	1.1	2.7	0.3	1.8
ZBTB43		4.4	1.6	2.4	0.6	0.8	1.3	3.6	0.5	3.2
MGC21881		4.6	1.6	2.4	0.4	0.9	1.6	3.6	0.5	2.6
HMBOX1		4.9	1.7	2.5	0.2	0.5	1.1	4.0	0.6	2.7
VPS39		5.7	2.2	3.2	0.5	1.2	1.3	2.2	0.2	1.8
COMTD1		4.6	2.2	2.6	1.0	1.6	2.1	2.2	0.6	1.3
DTD2		4.7	1.6	2.8	0.5	1.1	1.3	3.2	0.6	2.6
CNKSR3		4.5	1.6	2.0	0.3	0.9	1.4	3.9	0.4	3.3
RIC8B		5.6	2.0	2.3	0.2	0.7	0.7	3.6	0.4	2.6
GDAP2		4.6	1.8	2.4	0.1	0.5	1.0	4.1	0.4	3.3
SUV39H2		5.7	1.3	2.4	0.3	0.7	0.7	4.2	0.4	2.5
UBR2		5.9	1.8	2.3	0.2	0.8	1.1	3.3	0.3	2.4
TMEM184C		4.7	1.9	2.4	0.1	0.8	1.2	3.8	0.5	2.8
SKAP2		5.6	1.9	3.3	0.2	0.7	1.2	2.8	0.3	2.2

C6orf47	4.1	1.5	2.2	0.6	0.8	2.0	4.2	0.6	2.2
F12	4.0	1.7	2.2	0.7	1.1	1.2	4.0	0.5	2.7
SFT2D2	5.8	1.7	2.4	0.1	0.7	0.9	3.1	0.3	3.2
ABHD16B	3.7	1.4	2.0	0.6	1.2	1.8	4.3	0.7	2.6
UBE2T	4.4	1.9	3.1	0.3	1.1	1.2	2.4	0.8	3.1
LOC101928082	5.9	1.4	2.8	0.3	0.9	0.9	3.8	0.1	2.0
LOC100288748	6.1	1.2	2.7	0.3	0.7	0.7	4.3	0.3	1.8
PARN	4.7	2.0	2.4	0.2	0.8	1.2	3.6	0.6	2.7
CDK1	3.3	1.5	2.4	0.1	1.0	1.3	4.4	0.5	3.8
USP33	5.1	1.8	2.8	0.2	0.9	1.0	3.0	0.4	2.9
THUMPD3	5.3	2.0	2.7	0.3	0.8	1.2	2.8	0.5	2.5
HELB	5.8	1.1	2.3	0.3	0.8	0.7	4.9	0.3	2.0
C1orf216	4.2	1.9	2.8	0.4	1.1	1.0	3.2	0.8	2.7
PUSL1	3.4	1.0	1.7	0.8	0.8	1.7	4.9	0.7	3.1
LINC00926	6.0	0.9	2.9	0.4	0.6	0.8	4.3	0.4	2.0
TCTN1	5.6	2.1	2.7	0.6	1.8	2.1	1.7	0.3	1.3
TOR2A	5.6	2.2	2.5	1.1	0.7	1.5	2.9	0.3	1.4
LEO1	4.2	1.9	2.2	0.2	0.9	1.0	4.0	0.5	3.2
S100PBP	5.5	2.0	2.9	0.3	0.6	0.9	3.0	0.2	2.7
WDR48	5.6	1.9	2.0	0.1	0.6	0.8	3.6	0.5	3.0
STYX	4.7	2.0	2.4	0.2	0.9	1.4	3.2	0.8	2.6
MTR	5.7	1.7	2.8	0.4	1.0	1.4	2.8	0.3	2.0
VBP1	5.4	2.0	2.9	0.2	0.5	0.6	3.7	0.3	2.4
ZNF468	5.1	1.4	2.8	0.3	0.9	1.0	3.9	0.4	2.4
NEXN	4.0	1.4	2.2	0.2	0.8	1.4	4.0	0.7	3.2
TUBGCP4	5.1	1.7	2.3	0.3	0.7	0.9	3.6	0.5	3.2
LPCAT3	5.6	2.9	2.9	0.4	0.6	1.3	2.1	0.3	2.0
TAPT1-AS1	4.5	1.6	2.7	0.2	0.7	0.9	4.1	0.5	2.9
C18orf21	4.7	2.3	2.6	0.4	0.6	1.2	2.8	0.7	2.8
LOC729444	5.5	1.4	2.9	0.5	1.1	1.2	2.8	0.4	2.2
HSBP1L1	5.4	2.4	2.5	0.0	1.0	1.1	2.9	0.8	1.9
FBXO25	4.6	1.6	2.1	0.2	0.9	1.3	3.5	0.6	3.1
EIF4ENIF1	4.2	1.6	2.3	0.3	0.9	1.2	3.3	0.7	3.5
IL18BP	5.1	1.9	2.3	0.8	0.8	1.2	3.6	0.4	2.1
LOC101929059	6.2	1.1	2.7	0.2	0.8	1.0	3.3	0.3	2.4
ZMAT1	6.4	1.4	2.5	0.4	0.7	0.9	3.8	0.4	1.7
ZNF558	4.8	2.1	2.5	0.2	1.2	1.0	3.4	0.4	2.6
TRIM23	5.3	1.7	2.5	0.1	0.8	0.9	3.7	0.4	2.6
FAM27A	3.4	1.4	2.1	0.5	1.2	1.2	4.3	0.8	3.0
STX12	5.1	2.1	2.5	0.3	1.1	1.4	2.9	0.3	2.3
KCTD7	5.1	1.8	1.9	0.6	0.8	1.1	3.9	0.5	2.3
ZC3HC1	4.9	2.1	2.5	0.3	0.7	0.9	4.0	0.4	2.2
ZCCHC10	4.0	2.3	2.9	0.2	0.9	1.1	2.9	0.3	3.4
CNOT10	5.2	1.6	2.5	0.3	0.7	1.1	3.5	0.5	2.5
SHPK	6.2	1.7	2.6	0.7	0.9	1.3	3.1	0.2	1.4
FAM118A	5.2	1.5	2.9	0.3	0.6	0.5	3.9	0.5	2.5
CACFD1	5.8	1.9	2.7	1.0	0.8	1.6	2.3	0.1	1.8
RPUSD2	5.1	1.8	2.6	0.4	1.0	1.3	3.1	0.7	2.0
LOC100128682	6.2	1.2	2.4	0.3	0.7	0.9	4.4	0.3	1.7
STK36	5.8	2.2	3.1	0.4	1.0	1.3	2.5	0.3	1.4
MIS18BP1	4.7	1.9	2.2	0.1	0.9	1.1	3.2	0.5	3.4
NDUFAF1	4.5	1.4	2.4	0.1	0.7	0.8	4.5	0.7	2.8
FXN	4.6	2.0	2.6	0.6	0.8	1.3	2.9	0.6	2.6
ZNF785	5.7	1.5	2.2	0.8	1.1	1.1	3.2	0.5	1.9
MPP4	5.6	1.2	2.3	0.3	0.8	0.8	4.5	0.4	2.2

TFAP2C	3.9	1.5	1.8	1.0	2.1	2.9	2.5	0.5	1.7
U2AF1L4	4.5	2.2	2.6	0.4	1.4	1.3	2.4	1.0	2.1
ZNF791	4.5	1.8	2.6	0.2	0.8	0.7	4.0	0.4	3.0
RRP8	4.8	1.6	2.6	0.4	0.8	1.1	3.3	0.6	2.7
KCTD21-AS1	5.7	1.6	2.6	0.5	0.6	0.8	3.9	0.2	2.1
USP8	4.7	1.5	2.1	0.1	0.6	1.1	4.0	0.5	3.3
MDC1	4.8	1.6	2.3	0.4	1.1	1.2	2.8	0.6	3.1
C1orf220	5.6	1.2	2.2	0.3	0.8	0.9	4.6	0.4	1.9
PRKRA	4.1	2.1	2.7	0.3	1.2	0.9	2.9	0.6	3.0
TMTC3	6.0	2.2	2.4	0.1	0.7	1.1	2.4	0.4	2.7
ACVR2B	4.8	1.6	2.6	0.4	1.0	1.1	3.4	0.5	2.5
IGFBPL1	4.2	1.9	2.7	0.7	0.7	1.3	3.4	0.8	2.3
SOS2	5.0	1.8	2.1	0.2	0.8	0.9	3.7	0.5	2.9
SSX1	5.3	1.9	2.6	0.3	1.1	1.7	2.5	0.3	2.2
XPC	4.8	2.0	2.4	0.5	1.1	1.5	3.0	0.4	2.4
SRP14-AS1	5.8	1.3	2.2	0.3	0.6	0.8	4.3	0.4	2.1
UG0898H09	6.0	1.2	2.9	0.4	1.0	1.0	3.4	0.3	1.8
RAB40B	5.4	2.2	2.8	0.8	1.5	1.4	2.0	0.2	1.6
LINC00574	5.6	1.1	2.5	0.3	0.9	0.9	4.2	0.5	1.9
GNAI1	4.4	1.7	2.4	0.2	0.9	0.8	4.2	0.6	2.8
KLRC2	7.2	2.7	3.9	0.2	0.7	1.4	0.9	0.2	0.6
PAPD5	3.9	1.9	2.2	0.5	0.8	1.0	3.4	0.9	3.2
KMT2E	5.0	2.0	2.6	0.3	0.8	1.2	2.4	0.5	2.9
MRPL1	4.4	1.4	2.3	0.2	0.7	1.0	4.1	0.3	3.6
C10orf88	4.7	2.1	3.0	0.1	0.5	0.8	3.8	0.3	2.7
SOD3	5.0	2.4	3.1	0.7	1.4	1.7	2.4	0.2	1.0
SKIV2L2	4.5	2.0	2.2	0.2	0.6	1.1	3.8	0.5	3.0
NPIPA8	5.7	2.0	2.8	0.4	1.1	1.2	2.0	0.4	2.2
NPIPA7	5.7	2.0	2.8	0.4	1.1	1.2	2.0	0.4	2.2
FAM35A	5.3	1.8	2.8	0.1	0.5	0.8	3.2	0.4	2.9
SPTLC3	4.2	1.6	1.9	0.5	1.3	1.9	3.2	0.5	2.6
DERA	4.5	1.8	2.3	0.3	0.9	1.3	3.5	0.4	2.9
THTPA	5.2	1.9	2.7	0.7	1.1	1.5	2.5	0.4	1.8
LONRF1	5.8	2.2	2.5	0.4	0.8	0.8	2.9	0.4	2.1
NECAB3	4.4	1.7	2.3	0.9	1.4	1.8	3.0	0.5	1.9
RPS6KA2	5.4	2.0	2.6	0.7	1.6	1.8	2.0	0.3	1.5
WDR75	4.2	1.6	2.2	0.1	0.7	1.1	4.2	0.5	3.4
ZRSR2	4.7	1.4	2.1	0.3	0.6	0.9	3.5	0.6	3.7
AP3M2	4.4	1.4	1.8	0.2	0.8	1.0	4.1	0.6	3.4
TRAK2	5.2	2.3	2.7	0.2	0.8	1.1	2.6	0.6	2.3
IMPACT	5.0	2.4	2.6	0.4	0.9	1.4	2.5	0.2	2.4
FOXRED2	5.2	1.5	2.1	0.7	1.8	1.8	2.2	0.5	2.0
TRPS1	5.2	1.5	2.3	0.3	0.8	1.2	3.2	0.5	2.7
TBC1D5	5.0	1.9	2.2	0.2	0.9	1.2	2.9	0.5	2.9
STX16-NPEPL1	5.1	2.0	2.7	0.6	1.3	1.5	2.5	0.4	1.6
LOC101928309	6.3	1.3	2.5	0.3	0.7	0.6	3.9	0.3	1.9
MYO5A	4.6	1.7	1.9	0.4	0.8	1.2	3.6	0.5	3.0
NDUFB2-AS1	5.6	1.1	2.7	0.4	0.8	0.9	3.9	0.4	2.0
RELL2	4.9	2.3	2.6	0.7	1.1	1.3	2.3	0.5	2.2
ARRDC3-AS1	6.1	1.4	3.0	0.3	0.9	1.3	2.4	0.4	2.0
RBM5-AS1	5.5	1.1	2.7	0.3	0.6	0.6	4.3	0.2	2.4
CLASP2	4.5	1.5	2.2	0.2	0.7	0.6	4.3	0.6	3.1
SLC12A6	4.8	1.8	2.2	0.2	0.9	1.1	3.4	0.5	2.7
PTPN3	5.4	1.7	2.2	0.3	0.7	0.9	3.7	0.4	2.4
MIS18A	6.1	1.8	2.5	0.4	0.8	0.9	3.0	0.2	1.9

LOC101928589	5.0	2.2	2.6	0.2	0.7	1.1	3.1	0.5	2.3
NAA35	4.2	1.5	1.9	0.1	0.5	0.7	4.6	0.6	3.5
KCTD6	4.7	2.1	2.9	0.2	0.6	0.8	2.7	0.7	3.0
LOC101928280	5.1	0.9	2.6	0.5	0.9	0.7	4.9	0.2	1.9
C3orf18	4.7	2.3	2.7	0.8	1.8	2.4	1.6	0.4	1.1
RHBDD3	4.6	1.8	2.2	0.4	0.8	1.4	3.9	0.5	2.0
MAFB	6.1	1.5	3.0	0.8	1.4	1.9	1.7	0.4	1.0
UBXN2B	5.7	1.5	2.6	0.2	0.9	1.1	3.0	0.5	2.1
LOC100507373	4.9	1.4	2.8	0.4	1.0	1.2	2.9	0.5	2.5
ZNF23	4.1	1.8	2.0	0.2	0.4	0.8	4.3	0.6	3.5
RCHY1	4.5	2.3	2.5	0.3	0.8	1.0	3.5	0.4	2.4
CGRRF1	4.3	1.6	2.7	0.1	0.5	0.8	3.7	0.6	3.3
LOC100506365	4.0	1.5	2.5	0.2	0.9	1.1	4.2	0.7	2.6
ITPKC	5.2	1.9	2.4	0.3	0.8	0.8	3.5	0.4	2.3
CXCR4	3.4	1.2	2.0	0.3	0.4	0.7	5.1	0.8	3.8
URB2	4.3	1.1	1.7	0.2	0.7	0.9	4.7	0.5	3.5
ZNF688	5.6	2.3	3.0	0.5	0.8	1.4	2.1	0.5	1.3
TRIP11	5.2	1.9	2.7	0.1	0.6	1.0	2.9	0.3	2.7
SLC9A8	5.7	2.4	2.8	0.5	1.1	2.1	1.5	0.2	1.2
KLHL11	4.5	1.5	2.3	0.3	0.8	1.2	3.7	0.5	2.7
STRN	5.0	1.7	2.2	0.2	0.7	1.1	3.4	0.4	2.9
C11orf35	6.2	2.6	3.3	0.5	0.8	1.4	1.5	0.3	1.0
RASAL2-AS1	5.1	1.3	2.6	0.7	1.1	0.7	3.5	0.5	2.1
ATAT1	5.0	1.8	2.4	0.5	1.2	1.4	2.4	0.9	2.0
APPL1	5.2	1.6	2.5	0.3	0.8	1.0	2.7	0.5	2.9
RRM1	3.7	1.6	1.7	0.2	0.8	1.1	4.6	0.6	3.3
FMR1	4.9	1.9	2.6	0.4	0.5	0.9	3.2	0.5	2.6
ATL2	5.1	1.7	2.3	0.2	0.7	0.8	3.3	0.4	3.1
TAF3	4.6	1.7	1.8	0.3	0.7	1.1	3.7	0.6	3.2
ARMC1	5.3	1.9	3.2	0.3	1.0	1.3	2.6	0.3	1.7
BLOC1S1	5.6	1.4	2.4	0.6	0.8	1.3	2.1	1.0	2.3
PARD6A	3.7	1.5	2.1	0.5	1.0	0.9	4.5	0.6	2.7
ORAI3	6.5	2.6	2.9	0.6	1.1	1.1	1.2	0.3	1.2
GMPPB	4.0	1.5	2.0	0.4	0.7	1.2	4.4	0.7	2.6
CABLES1	5.5	2.4	2.6	0.5	1.0	1.4	2.2	0.3	1.6
NUFIP1	4.5	1.8	2.4	0.4	0.6	1.2	3.4	0.7	2.5
PWARSN	5.6	1.2	2.6	0.3	0.6	0.6	4.7	0.3	1.5
LOC101927294	4.1	1.6	1.6	0.6	0.8	0.6	4.9	0.8	2.7
NF1	5.6	1.5	2.5	0.2	0.6	0.7	3.5	0.4	2.5
SLC23A2	5.1	1.8	2.4	0.3	1.0	1.2	2.6	0.6	2.5
C6orf132	3.2	1.4	1.9	0.4	0.8	0.8	4.0	1.0	4.0
KSR1	5.0	1.7	2.4	0.5	1.0	1.5	2.6	0.6	2.1
CHD1L	4.3	1.6	2.3	0.3	0.9	1.4	3.3	0.4	2.9
LOC101928649	4.3	1.9	2.3	0.4	1.2	1.2	3.1	0.5	2.4
SMARCAL1	4.9	1.7	2.4	0.3	1.0	1.3	3.3	0.3	2.1
MKLN1-AS2	5.7	1.3	2.7	0.4	0.8	0.7	3.4	0.3	2.1
NIT1	4.5	2.2	2.6	0.5	1.1	1.3	3.1	0.5	1.7
SPATA33	4.2	1.6	2.3	0.6	1.0	1.6	3.1	0.4	2.7
PYCRL	4.2	1.2	2.4	0.8	1.6	2.0	2.6	0.4	2.2
TRIQK	5.6	1.6	2.6	0.1	0.9	0.8	3.4	0.6	1.8
GBP2	8.0	2.8	3.9	0.2	0.4	0.7	0.8	0.1	0.5
SPDL1	3.4	1.2	1.8	0.2	0.7	0.8	4.7	0.7	3.8
NUPL2	4.5	1.8	2.5	0.5	1.0	1.1	2.9	0.5	2.6
PRPF18	4.9	1.9	2.2	0.1	0.5	1.0	3.4	0.2	3.2
MRPS31	5.1	2.0	2.5	0.2	0.6	0.9	3.1	0.5	2.5

MAST1	5.9	2.6	3.4	0.4	0.7	1.2	1.6	0.3	1.3
ARHGAP40	6.1	1.7	3.1	0.3	0.7	0.9	2.4	0.4	1.7
NMNAT2	6.5	2.2	2.4	0.4	0.9	1.3	1.7	0.3	1.8
LINC00260	5.4	1.1	3.1	0.3	0.5	0.6	4.4	0.4	1.6
SH3PXD2B	6.0	1.9	2.7	0.4	0.7	1.2	2.1	0.3	2.0
CCNT1	4.7	1.8	2.2	0.3	0.7	0.7	3.1	0.6	3.2
PPIP5K1	5.2	1.6	2.4	0.4	1.0	1.1	3.1	0.4	2.2
LOC101927040	5.6	1.2	2.3	0.3	0.7	1.0	4.0	0.3	1.9
ANO10	4.0	1.6	2.5	0.2	0.9	1.1	3.8	0.3	2.9
HPS4	4.8	1.9	2.5	0.5	0.8	1.4	3.0	0.5	2.1
METTL15	4.8	1.7	2.7	0.2	0.8	1.2	3.1	0.5	2.4
LAMA4	7.0	1.9	2.8	0.5	1.1	1.6	1.4	0.1	0.9
ZNF716	5.5	1.4	2.3	0.2	0.7	0.7	4.1	0.3	2.1
RPA3	3.9	1.4	2.6	0.6	1.0	1.1	3.8	0.4	2.5
C16orf91	3.8	1.5	2.2	0.5	0.6	1.1	3.5	0.7	3.4
LOC101928294	5.8	2.2	2.2	0.5	0.7	1.0	2.3	0.8	1.8
EPT1	4.6	1.6	2.1	0.2	0.7	1.2	3.6	0.5	2.8
MLLT4-AS1	5.7	1.0	2.1	0.4	1.1	0.9	3.9	0.4	1.8
CMTM1	3.4	1.3	1.8	0.9	1.3	1.6	4.0	0.7	2.3
TTC14	4.3	1.6	2.7	0.2	0.8	0.8	3.8	0.5	2.6
RAB33B	4.4	1.4	2.0	0.4	0.8	0.7	3.7	0.7	3.1
ZBTB5	3.9	1.6	2.2	0.3	0.7	0.9	3.4	0.8	3.5
DPY19L4	4.8	2.1	2.4	0.1	0.6	0.8	3.5	0.3	2.7
NR4A2	5.4	1.7	3.0	0.3	0.5	0.9	2.5	0.7	2.3
RAD51D	5.0	1.2	2.1	0.3	0.9	1.0	3.7	0.4	2.7
TMEM47	4.9	1.6	2.7	0.4	1.0	1.0	2.9	0.7	2.1
AQP4-AS1	6.2	1.2	2.5	0.3	0.6	0.8	4.0	0.3	1.5
SLMO2	4.6	2.0	2.3	0.2	0.3	0.7	2.9	0.8	3.6
RELT	5.1	1.2	2.0	0.5	0.8	0.9	3.1	0.8	2.8
FRA10AC1	4.8	1.8	2.2	0.2	0.7	0.8	3.8	0.3	2.8
ZNF367	2.6	0.9	1.7	0.6	1.4	1.3	4.3	1.1	3.3
C22orf46	4.0	1.6	2.1	0.4	0.8	0.8	4.2	0.7	2.5
MECR	5.3	2.4	2.3	0.4	1.2	1.2	2.4	0.3	1.8
LZIC	4.9	1.9	2.5	0.2	0.6	0.4	3.9	0.5	2.3
ZNF134	4.7	1.6	1.9	0.2	1.0	1.2	3.3	0.3	3.1
TMEM256	3.6	1.4	2.5	0.9	1.4	2.1	2.7	0.4	2.2
WDFY2	4.5	1.6	2.4	0.3	0.5	0.8	4.1	0.5	2.3
TCTA	5.0	2.4	3.7	0.7	0.8	0.5	1.7	0.2	2.0
ANKRD6	5.3	2.0	2.8	0.3	0.9	1.1	2.4	0.3	1.9
DBP	6.1	1.9	2.4	0.6	0.7	1.2	2.6	0.5	1.1
GPSM2	4.7	1.9	2.2	0.2	0.7	0.9	3.7	0.4	2.4
LOC101929708	6.7	2.1	3.2	0.2	0.4	0.8	2.0	0.3	1.4
HOMER2	4.0	1.1	1.3	0.3	1.1	1.2	3.8	0.6	3.7
VPS33B	5.7	1.6	2.8	0.3	0.7	1.2	2.8	0.3	1.8
MRPL30	4.9	1.9	2.5	0.2	0.8	1.0	2.7	0.4	2.7
LOC101928808	5.9	1.1	2.6	0.3	0.8	0.9	3.4	0.3	1.8
C9orf64	5.3	1.9	2.1	0.2	0.7	0.9	2.3	0.4	3.3
DNAJC16	4.9	2.1	2.7	0.2	0.7	1.0	2.6	0.4	2.5
MTX3	4.9	1.5	2.6	0.1	0.8	1.0	3.4	0.3	2.5
GJD3	4.1	2.2	3.2	1.1	0.9	1.6	1.7	0.4	1.9
TINCR	5.5	1.2	2.5	0.3	0.7	0.9	3.8	0.4	1.8
IBTK	4.3	1.5	2.2	0.1	0.5	0.8	3.9	0.4	3.3
ZNF319	4.7	1.9	2.5	0.8	1.4	1.8	1.8	0.5	1.8
FAM13B	4.0	1.7	1.9	0.2	0.7	1.2	3.4	0.5	3.5
PSMB9	4.2	2.0	3.1	0.4	1.3	1.1	2.8	0.5	1.8

IFT80		4.8	2.1	2.8	0.1	1.1	1.1	2.6	0.2	2.2
LARS2		5.0	1.6	2.5	0.3	0.7	1.0	3.2	0.3	2.5
LOC101927004		4.5	1.7	3.2	0.5	0.9	1.3	3.3	0.5	1.3
KIF3A		4.7	1.4	2.5	0.1	0.6	0.6	3.6	0.4	3.2
CCDC71L		4.3	2.4	3.1	1.0	1.5	1.3	2.0	0.5	1.1
WNK4		4.5	1.8	2.6	0.3	0.7	0.7	3.4	0.5	2.6
FANCC		4.9	1.4	1.7	0.3	0.9	1.3	3.3	0.5	2.8
APTX		4.7	1.9	2.5	0.1	0.8	1.1	3.3	0.4	2.3
HIF3A		5.5	1.3	2.0	0.4	0.7	1.0	3.8	0.4	2.0
KPTN		4.9	1.8	2.5	0.5	0.9	1.1	3.0	0.5	1.8
RNF144B		3.5	1.5	2.1	0.4	1.0	1.5	3.9	0.6	2.5
POMT1		5.8	1.8	2.5	0.3	0.8	0.9	3.0	0.3	1.6
SH3RF3-AS1		5.2	1.4	2.2	0.7	0.7	1.3	3.2	0.5	1.9
MEF2BNB-MEF2B		4.7	1.9	2.2	0.5	0.9	1.4	2.8	0.6	2.2
SNORA5A		4.5	2.0	2.5	0.2	0.5	1.1	2.6	1.0	2.7
AZI1		5.3	2.0	2.9	0.8	1.2	1.5	1.7	0.4	1.3
LRRFIP1		4.5	2.1	1.9	0.4	0.7	1.0	3.6	0.2	2.6
LOC101928580		6.0	1.2	2.5	0.3	0.6	0.7	3.9	0.2	1.7
TUBE1		4.3	1.9	2.6	0.3	0.7	1.0	2.5	0.6	3.3
ZC3HAV1		4.9	1.7	2.3	0.3	0.8	1.0	3.0	0.6	2.4
UBE2D4		3.3	1.6	1.5	0.6	1.5	2.1	2.9	0.2	3.4
FCHO2		4.7	1.7	2.3	0.3	0.5	1.0	2.9	0.5	3.0
LOC101929227		5.6	1.1	2.3	0.3	0.6	0.7	4.3	0.3	1.7
DIS3L		4.4	1.6	2.2	0.3	0.8	1.3	3.5	0.4	2.5
PLK3		4.1	1.6	2.5	0.5	1.1	1.3	3.1	0.5	2.2
PGGT1B		4.1	1.1	2.5	0.2	0.7	0.7	3.9	0.4	3.3
TRMT11		3.6	1.3	1.7	0.1	0.4	0.9	5.0	0.4	3.6
C2CD2		4.2	1.7	2.3	0.4	1.1	1.0	3.3	0.5	2.4
DOLK		4.2	1.8	2.6	0.3	1.0	1.6	2.7	0.4	2.4
	44624.0	2.6	1.0	1.0	0.2	0.6	0.6	5.6	0.7	4.7
LETMD1		3.6	1.2	2.0	0.4	0.8	1.2	4.4	0.5	3.0
ISG20		5.4	2.1	2.6	0.3	0.6	1.1	2.6	0.4	1.8
FECH		4.9	1.9	2.7	0.5	1.3	1.4	2.3	0.3	1.7
SLC26A6		5.2	2.3	3.4	0.4	0.8	0.9	2.0	0.3	1.6
PIDD		4.8	2.0	2.8	0.6	1.1	1.1	2.4	0.4	1.8
SIRT3		5.4	2.0	2.9	0.7	1.1	1.6	1.7	0.1	1.3
PLK1		4.2	2.1	2.3	0.8	1.7	1.4	2.5	0.5	1.5
LINC00662		5.3	1.2	2.4	0.3	0.7	0.9	3.5	0.4	2.3
LOC100507367		4.9	2.1	2.2	0.0	0.7	0.9	3.5	0.4	2.2
TMEM241		4.9	1.4	2.5	0.4	0.7	0.8	3.9	0.4	2.0
ENDOD1		4.9	1.6	2.2	0.5	1.0	1.4	3.0	0.3	1.9
BRAF		5.1	1.8	2.3	0.2	0.7	0.9	2.9	0.3	2.6
ARL4D		4.2	1.4	2.3	0.2	0.9	1.2	4.2	0.4	2.1
MROH6		5.4	2.1	3.2	0.4	0.7	0.7	2.3	0.5	1.5
KANSL2		3.8	1.6	2.4	0.4	0.6	0.7	4.1	0.6	2.8
LRRK1		5.1	1.6	2.2	0.5	0.9	1.2	3.0	0.4	2.1
CEP63		4.6	1.6	2.1	0.1	0.9	1.0	2.7	0.4	3.5
NBN		4.5	1.7	2.1	0.3	1.0	1.3	2.7	0.6	2.7
FAM161B		5.1	2.1	2.7	0.5	1.2	1.2	2.2	0.3	1.6
UBA6		4.5	1.6	2.0	0.1	0.7	1.1	3.7	0.3	2.9
ASCC3		4.8	1.1	2.1	0.1	0.5	0.6	4.2	0.4	3.1
AGK		4.2	1.7	2.1	0.3	1.0	1.4	3.1	0.4	2.7
PPP2R3A		4.5	1.7	2.0	0.2	0.8	1.1	3.2	0.4	2.9
SNX11		4.5	1.6	2.2	0.1	0.3	1.2	4.1	0.3	2.5
LOC375295		5.0	1.2	2.1	0.3	0.7	1.3	3.1	0.5	2.7

LOC100131496	4.7	1.2	2.2	0.3	0.6	0.8	4.6	0.5	1.9
ZFC3H1	4.2	1.5	1.9	0.2	0.8	1.0	3.7	0.5	3.0
GEMIN2	4.1	2.0	2.9	0.2	0.5	0.7	3.8	0.4	2.3
KIAA0753	4.5	1.3	2.6	0.2	0.7	1.0	3.5	0.4	2.6
USP13	4.1	1.4	1.8	0.3	1.0	1.2	4.0	0.4	2.6
MTPAP	4.7	1.2	2.4	0.2	0.6	0.8	3.3	0.4	3.2
SLC39A11	4.9	1.6	2.2	0.4	0.6	1.0	4.0	0.5	1.7
GPATCH3	4.7	1.7	2.0	0.3	0.4	0.8	3.6	0.5	2.8
IL21R-AS1	5.3	1.2	2.4	0.2	0.6	0.7	4.3	0.4	1.6
B9D1	4.6	1.6	3.5	0.5	1.0	1.7	2.0	0.2	1.7
EIF2B3	4.3	1.6	2.2	0.2	0.9	0.8	4.1	0.3	2.5
MAP2K5	4.7	1.9	2.3	0.2	0.9	1.2	2.9	0.3	2.2
ADCK2	4.6	1.9	2.6	0.3	0.7	1.1	3.0	0.6	2.0
LRRN1	7.0	2.3	3.0	0.4	1.3	1.4	0.6	0.1	0.8
ELP6	4.2	1.6	2.4	0.5	0.7	1.6	3.7	0.2	1.9
MBLAC2	5.0	1.6	2.4	0.9	0.6	1.2	2.6	0.5	2.3
RNF152	4.4	1.5	1.4	0.3	0.6	0.9	3.8	1.0	2.7
HSD11B1L	5.3	2.0	2.8	0.8	1.5	1.4	1.8	0.3	0.9
MAD2L1	3.9	1.5	2.1	0.3	0.8	0.8	3.9	0.4	3.2
C7orf63	5.8	1.1	2.4	0.2	0.7	0.7	3.5	0.2	1.9
LOC101927098	6.4	1.8	3.3	0.6	0.7	1.7	1.4	0.0	0.9
IKBKE	5.5	2.1	2.9	0.5	1.1	1.1	1.8	0.3	1.4
HIVEP1	5.1	1.5	1.9	0.3	0.8	1.1	3.0	0.5	2.6
GTF3C3	4.1	2.3	2.3	0.2	0.7	1.2	3.2	0.5	2.4
CDYL2	4.8	1.6	2.0	0.5	1.0	1.2	2.7	0.4	2.6
FAM214B	4.2	1.8	2.2	0.6	1.0	1.7	2.7	0.4	2.2
CEP350	4.9	1.4	2.2	0.2	0.5	0.7	3.8	0.4	2.6
ZNF621	4.4	1.4	3.0	0.2	0.6	0.8	3.2	0.4	2.9
LOC283299	5.5	1.2	2.6	0.3	0.6	0.9	3.5	0.3	1.8
FER	4.4	1.4	2.4	0.1	0.6	0.9	3.9	0.4	2.6
LRRC8C	3.6	1.2	1.7	0.2	0.6	0.7	4.4	0.6	3.7
INHBA-AS1	5.4	1.3	2.3	0.3	0.6	0.6	4.1	0.4	1.8
COQ2	4.9	1.9	2.4	0.6	0.7	1.3	2.8	0.4	1.8
C5orf38	5.3	1.3	2.8	0.9	0.5	1.4	2.9	0.5	1.2
MKNK1	5.1	1.5	2.5	0.4	0.6	0.8	2.9	0.6	2.2
GALM	5.2	2.4	2.5	0.3	0.7	0.9	2.7	0.3	1.8
KRCC1	5.1	1.8	2.7	0.1	0.7	1.6	2.3	0.3	2.1
SERPINB2	1.6	0.4	0.6	0.8	1.7	2.6	5.0	0.5	3.4
DICER1	4.9	1.5	2.1	0.2	0.6	0.9	3.4	0.5	2.7
WDR53	4.4	1.6	2.1	0.2	0.9	1.2	3.2	0.3	2.9
LOC101928946	4.8	0.8	2.2	0.6	1.0	1.0	4.1	0.3	1.9
MFN1	4.6	1.7	2.3	0.2	0.6	0.8	3.5	0.3	2.7
OXNAD1	4.0	1.3	2.0	0.2	0.7	0.8	4.1	0.5	3.0
RASSF5	4.7	1.6	2.6	0.4	0.6	0.7	3.2	0.5	2.4
BMP2	3.3	1.1	1.4	0.5	0.7	1.2	4.1	0.5	3.8
LOC100289283	5.9	1.7	2.9	0.1	0.9	0.5	2.8	0.0	1.6
PINX1	4.0	1.6	3.0	0.2	0.7	1.2	2.8	0.2	2.9
TMEM237	3.9	1.6	2.3	0.2	1.1	1.3	3.0	0.6	2.6
ZNF322	4.2	1.5	1.9	0.1	0.6	0.9	4.2	0.4	2.9
TIMELESS	3.6	1.0	1.7	0.5	1.0	1.2	3.9	0.6	3.1
SFXN2	5.2	1.0	2.3	0.3	0.6	1.0	3.9	0.4	1.9
MPDZ	4.8	1.3	2.4	0.2	0.5	0.8	3.4	0.4	2.8
MAP3K2	4.7	1.4	1.5	0.2	0.4	0.6	4.0	0.6	3.2
ZBTB47	4.0	1.7	2.0	0.7	1.0	1.8	2.6	0.5	2.2
GTPBP8	4.4	1.7	2.1	0.2	1.1	1.1	2.7	0.5	2.8

MKS1	4.4	1.8	2.6	0.4	1.2	1.3	2.6	0.4	2.0
PPIL2	3.6	1.8	2.0	0.6	1.5	2.1	2.3	0.5	2.2
NPEPL1	5.7	1.6	2.3	0.7	0.3	1.5	2.1	0.1	2.3
FAM98C	4.7	1.6	2.5	0.7	0.8	1.7	2.4	0.4	1.8
THOC2	5.2	1.6	2.3	0.1	0.6	0.8	3.3	0.4	2.3
ZCCHC11	4.4	1.6	2.2	0.2	0.7	1.0	3.5	0.4	2.6
SLC25A33	3.1	1.3	1.9	0.1	0.7	0.8	4.3	0.6	3.7
PDE6D	4.5	2.4	2.3	0.0	0.5	0.7	3.2	0.7	2.3
LIPE	4.2	1.5	2.3	0.7	1.1	1.5	2.6	0.5	2.2
PRR7-AS1	4.8	1.2	2.4	0.2	0.5	0.8	4.3	0.3	2.0
AHI1	5.0	1.9	2.3	0.2	0.8	1.1	2.6	0.3	2.4
EBI3	4.1	2.1	3.1	1.2	1.4	2.1	1.0	0.3	1.4
CCDC78	4.6	2.4	3.0	0.5	0.9	1.2	2.0	0.3	1.7
TFB1M	4.9	1.6	2.7	0.3	0.7	1.1	2.9	0.3	2.1
GPX3	5.9	2.1	2.9	0.4	0.7	0.9	2.3	0.2	1.2
PHLDA3	5.0	2.2	2.3	0.3	0.8	0.8	2.8	0.5	1.9
PCNXL2	4.4	1.4	2.4	0.5	1.2	1.5	2.7	0.4	2.0
TRIML2	4.3	1.8	2.4	0.2	0.3	0.7	3.5	0.6	2.8
ME1	4.8	1.9	2.9	0.1	0.5	0.6	3.2	0.4	2.2
PER1	3.5	1.5	1.7	0.4	0.8	1.0	3.6	0.8	3.3
AQP6	5.3	1.0	2.2	0.3	0.7	0.7	4.1	0.3	1.8
PCDHGB7	5.9	2.4	2.8	0.5	0.6	1.1	1.6	0.3	1.3
RELL1	5.1	1.9	2.2	0.6	0.9	1.5	2.5	0.2	1.5
PGM2L1	5.9	1.8	3.2	0.1	0.4	0.4	2.7	0.2	1.9
RAD9A	4.4	1.8	2.3	0.4	0.6	1.1	3.1	0.5	2.3
LOC101929199	5.4	1.1	2.3	0.3	0.7	0.8	4.1	0.2	1.7
ZNF296	3.9	1.7	2.2	0.5	0.8	1.0	3.0	0.8	2.7
TDG	4.0	1.9	2.1	0.2	0.4	0.7	4.1	0.5	2.6
TTC31	4.2	1.5	2.6	0.5	1.2	1.7	2.6	0.4	1.9
ABHD15	4.6	1.6	2.2	0.6	0.9	1.2	3.0	0.6	1.7
B3GNT9	4.7	2.0	2.4	0.6	1.0	0.9	3.0	0.4	1.5
MUC1	4.5	2.4	2.7	1.2	1.3	1.5	1.3	0.4	1.0
LOC152225	4.3	1.2	1.9	0.1	0.7	0.9	3.8	0.5	3.1
ACVR1B	4.4	1.9	2.2	0.3	0.7	1.0	2.9	0.5	2.5
UHRF2	4.4	1.4	2.0	0.2	0.5	0.6	4.0	0.5	2.7
LOC338799	5.3	1.5	2.6	0.3	0.8	1.0	3.0	0.4	1.7
PSTK	4.5	1.9	2.2	0.4	0.9	1.0	3.0	0.3	2.3
KCNJ14	5.9	1.8	2.6	0.5	0.7	1.0	2.4	0.4	1.2
MCPH1	5.0	1.3	2.0	0.2	0.6	0.8	4.0	0.5	2.0
SNX24	3.2	1.2	1.4	0.1	0.6	1.0	5.3	0.3	3.2
KIAA1958	4.4	1.2	1.8	0.3	0.8	0.8	3.2	0.7	3.1
PRR5L	2.9	1.2	2.1	0.3	0.5	1.0	4.5	0.8	3.0
SH2D5	3.3	1.3	1.6	0.8	1.2	2.1	4.0	0.4	1.8
KLF7	4.2	1.9	2.3	0.7	0.9	1.0	2.3	0.4	2.7
LOC100506834	5.3	1.1	2.2	0.3	0.5	0.7	4.2	0.3	1.8
FAM172A	5.0	1.4	2.5	0.3	0.9	1.0	3.2	0.3	1.8
ABCB7	4.3	1.8	2.8	0.1	0.9	0.8	3.1	0.3	2.2
PAXIP1	4.7	1.7	2.1	0.4	0.8	1.1	2.6	0.5	2.6
DISC1	4.7	1.0	2.0	0.3	0.6	0.8	4.1	0.4	2.3
MKLN1-AS1	4.9	1.4	2.5	0.3	0.9	1.0	3.0	0.3	1.9
PHF21A	4.8	1.7	2.4	0.3	0.7	1.2	2.3	0.4	2.5
RAB7L1	4.6	1.5	1.8	0.3	0.4	1.4	3.1	0.5	2.8
TTC8	4.8	1.7	2.8	0.4	0.9	1.3	1.9	0.5	2.0
FAM219A	4.8	2.1	2.4	0.5	1.4	1.3	1.5	0.5	1.9
WDR60	4.9	1.5	2.5	0.4	0.8	1.0	2.9	0.4	2.0

STAM2	5.1	2.4	2.7	0.1	0.6	1.0	2.3	0.3	1.8
LAMA3	6.0	1.8	2.9	0.5	1.2	1.5	1.3	0.2	0.9
SAYSD1	4.3	1.8	1.8	0.3	0.7	0.9	3.6	0.6	2.3
LOC101929571	4.5	1.3	2.3	0.5	0.6	0.6	4.2	0.4	1.9
CERS6	3.9	1.5	2.0	0.2	0.6	0.8	4.3	0.6	2.5
LMO7	5.0	1.7	2.5	0.1	0.6	0.7	2.9	0.4	2.3
SHANK3	3.9	1.4	1.8	0.7	0.9	1.2	3.2	0.7	2.5
CD74	4.6	1.7	2.9	0.9	1.9	2.1	1.1	0.2	0.8
CHD9	4.7	1.4	2.1	0.1	0.7	0.9	3.5	0.4	2.5
RBBP5	4.5	1.7	2.3	0.2	0.6	0.9	3.3	0.4	2.5
GSTT2B	4.4	1.1	2.2	0.4	1.2	1.1	4.0	0.3	1.7
NUDT15	3.7	1.5	2.3	0.3	0.4	1.0	4.6	0.4	2.1
PELI1	4.6	1.9	2.4	0.1	0.3	0.6	3.2	0.5	2.7
COIL	4.0	1.6	2.5	0.1	0.5	0.9	3.2	0.4	3.1
ASF1A	4.2	1.7	2.4	0.1	0.8	1.0	2.8	0.5	2.7
LARP7	4.6	1.8	2.2	0.1	0.7	0.9	2.8	0.4	2.7
PPP1R26-AS1	5.3	1.1	2.4	0.2	0.7	0.9	3.5	0.3	1.8
NSL1	4.5	1.6	3.2	0.1	0.7	1.5	2.4	0.2	2.0
FERMT1	4.0	1.3	1.6	0.1	0.7	0.9	3.7	0.6	3.3
THAP9-AS1	3.5	1.2	1.7	0.1	0.6	0.6	4.3	0.6	3.7
OPHN1	4.1	1.5	2.0	0.3	0.8	0.9	3.5	0.5	2.8
LOC101929279	5.1	0.9	2.3	0.4	0.8	0.8	4.2	0.2	1.5
TRIM46	5.3	0.9	2.7	0.4	0.7	0.6	3.8	0.4	1.5
CEP68	4.9	1.7	2.7	0.5	1.1	1.5	2.3	0.2	1.3
DOCK7	5.6	1.2	2.0	0.1	0.6	0.8	3.3	0.4	2.2
FUBP1	4.0	1.7	2.4	0.5	1.0	1.3	2.6	0.4	2.4
DIP2C	4.8	1.5	2.3	0.4	0.9	1.2	2.6	0.5	1.9
ZNF784	4.6	1.5	2.2	0.8	1.0	1.5	2.8	0.3	1.6
SHMT1	4.7	1.6	2.5	0.3	0.7	0.8	2.8	0.5	2.3
CD274	3.4	1.5	1.7	0.2	0.7	1.0	3.4	0.4	3.9
FAM229A	4.8	1.6	3.4	0.4	1.0	0.8	2.7	0.6	0.9
MAP4K3	4.2	1.7	2.0	0.1	0.7	0.7	3.3	0.3	3.1
SMCHD1	4.4	1.3	1.8	0.1	0.6	0.9	3.6	0.4	3.1
KIF13B	5.3	1.6	2.1	0.4	0.7	0.9	2.9	0.5	1.7
ASL	3.2	1.1	1.9	0.5	1.1	1.7	3.7	0.5	2.5
CTBP1-AS2	4.3	1.3	2.1	0.4	0.9	1.0	3.5	0.4	2.2
ZNF273	5.2	1.1	2.2	0.3	0.6	0.8	3.8	0.4	1.7
ZADH2	3.3	1.3	1.8	0.4	0.9	1.0	4.1	0.4	3.0
LOC100996335	4.8	1.4	2.1	0.9	1.0	1.1	2.9	0.4	1.6
KIFC1	3.7	1.6	1.9	0.6	1.2	2.0	2.5	0.5	2.2
LOC100287098	5.4	1.4	1.8	0.8	0.8	1.5	2.0	0.2	2.3
LOC729987	5.5	1.2	2.2	0.2	0.7	0.7	3.1	0.3	2.3
UTP20	4.6	1.3	2.0	0.2	0.7	1.0	3.6	0.4	2.5
C10orf12	4.1	1.2	1.7	0.2	0.9	0.9	3.5	0.6	3.2
AQR	4.2	1.4	2.0	0.3	0.7	1.0	3.1	0.5	2.8
ANKFN1	4.3	1.6	2.4	0.2	0.7	1.0	3.0	0.5	2.3
RAD17	4.5	1.7	2.2	0.1	0.7	0.9	3.3	0.3	2.5
GALK1	3.8	2.0	2.3	0.8	1.5	2.2	1.7	0.4	1.4
LINC00839	4.1	1.9	2.5	0.4	0.6	1.0	3.1	0.5	2.0
KLHL20	4.7	1.5	2.1	0.3	0.8	1.0	3.3	0.3	2.1
PHACTR1	4.7	1.6	2.5	0.4	1.0	1.0	2.5	0.5	2.0
D2HGDH	5.3	1.1	2.5	0.8	1.1	1.5	2.3	0.3	1.2
PUS7	3.8	1.1	1.8	0.4	0.7	1.0	3.9	0.4	2.9
NAA40	4.1	1.0	2.1	0.5	0.8	1.2	4.0	0.4	2.1
RAB9A	4.5	1.6	2.5	0.0	0.6	0.7	3.3	0.4	2.4

MPP6	3.5	1.1	1.5	0.2	0.5	1.0	4.5	0.5	3.3
PKNOX1	4.5	1.5	1.8	0.3	0.7	1.1	2.9	0.8	2.4
SLC4A8	5.2	1.1	2.2	0.3	0.8	0.7	3.7	0.3	1.8
LGALSL	5.7	2.3	2.6	0.1	0.5	0.5	2.0	0.2	2.1
ZFPM1	4.4	1.9	1.9	1.1	1.1	1.5	2.0	0.5	1.7
LOC100126784	5.4	1.5	2.4	0.9	1.1	1.4	2.0	0.2	1.1
PIP4K2C	4.4	2.1	1.9	0.2	0.9	0.6	3.4	0.3	2.3
LOC101927045	5.0	1.2	2.3	0.2	0.7	0.8	3.5	0.3	2.0
IL10RB	4.7	1.7	2.5	0.2	0.7	1.3	2.4	0.4	2.1
KATNBL1	5.1	1.0	2.7	0.2	0.3	0.7	3.8	0.4	1.9
RIF1	4.4	1.3	2.0	0.1	0.6	0.7	3.5	0.4	3.0
NLRX1	4.8	2.1	2.8	0.5	1.2	1.5	1.5	0.4	1.3
WNT5A	5.4	1.8	2.1	0.4	1.0	1.3	2.0	0.4	1.7
RPS6KA3	4.5	1.6	2.1	0.2	0.6	0.7	3.3	0.4	2.6
GCAT	3.1	1.2	1.8	0.7	1.6	1.6	3.2	0.5	2.3
MSH3	4.3	1.8	2.4	0.3	0.7	1.1	2.8	0.4	2.3
NPIPB11	4.6	1.4	2.8	0.6	1.0	1.0	2.2	0.3	2.0
B3GALNT1	4.8	1.6	2.2	0.2	0.6	0.9	2.9	0.3	2.4
PKP3	5.0	2.1	2.2	0.5	0.9	1.3	2.0	0.4	1.6
FAM13A	4.9	2.0	2.2	0.2	0.5	0.8	2.8	0.4	2.3
KNOP1	4.0	1.3	1.8	0.5	0.8	1.2	2.9	0.6	3.0
MYCBP	3.7	2.1	1.6	0.1	0.6	0.3	4.2	0.6	2.7
LTB	7.8	3.1	3.4	0.2	0.2	0.2	0.5	0.1	0.5
OCRL	4.3	1.6	2.1	0.2	0.5	1.1	3.5	0.4	2.4
DYRK2	4.0	1.3	2.3	0.3	0.7	1.0	3.2	0.5	2.6
RNF8	4.5	1.2	2.1	0.2	0.5	0.7	3.9	0.5	2.4
ARNTL2	4.1	1.6	2.2	0.1	0.7	0.7	3.5	0.5	2.5
LOC256880	4.9	0.9	2.3	0.2	0.6	0.8	4.0	0.4	1.9
LOC100506730	5.3	0.9	2.2	0.3	0.6	0.7	3.7	0.4	1.8
DNAJC9-AS1	5.2	0.9	2.2	0.3	0.7	0.9	3.6	0.3	1.9
CCDC23	4.0	1.6	4.0	0.3	0.9	0.8	2.0	0.3	2.0
GABPA	4.5	1.7	2.4	0.1	0.7	1.3	2.7	0.3	2.2
LOC441124	3.6	1.8	2.1	0.9	1.3	2.4	1.8	0.5	1.6
SREK1IP1	4.5	1.9	2.3	0.1	0.6	0.3	3.0	0.4	2.8
PLXNA3	4.4	1.8	2.2	0.5	0.8	1.0	2.6	0.5	2.0
ARL5B	3.8	1.8	2.6	0.0	0.7	0.7	3.1	0.2	2.9
HAUS5	3.6	1.7	2.1	0.5	0.8	1.5	2.9	0.5	2.2
PNPLA8	4.6	1.9	2.7	0.2	0.6	0.7	2.8	0.2	2.3
LOC101927530	6.3	1.2	2.7	1.1	1.8	1.8	0.5	0.1	0.3
TSHZ1	4.2	1.6	2.2	0.6	1.0	1.4	2.0	0.5	2.3
SPTBN2	4.2	1.4	2.0	0.6	0.9	1.2	3.3	0.4	2.1
LOC100294362	5.5	1.2	2.6	0.3	0.6	0.7	3.2	0.3	1.5
GINS1	3.9	1.0	2.3	0.4	1.0	0.9	4.1	0.3	2.0
MEF2BNB	3.9	1.2	2.5	0.6	0.8	1.3	3.1	0.5	2.0
RBM18	4.7	1.7	1.7	0.1	0.7	0.8	3.0	0.4	2.9
SLX1A	4.5	2.0	2.5	0.5	1.0	1.4	1.9	0.4	1.8
SLX1B	4.5	2.0	2.5	0.5	1.0	1.4	1.9	0.4	1.8
HSPB11	4.8	1.9	1.9	0.1	0.8	0.7	2.9	0.4	2.4
ZBTB48	4.0	1.7	2.5	0.4	1.0	1.3	2.6	0.4	1.9
PPP1R3B	4.9	1.8	2.0	0.4	0.4	0.9	2.8	0.2	2.4
TAF4B	3.4	1.3	1.6	0.2	0.6	1.0	3.6	0.6	3.5
MAP2K4	4.3	1.7	2.7	0.2	1.0	0.8	2.6	0.3	2.3
SCD5	3.8	1.4	2.0	0.3	0.6	0.9	3.6	0.7	2.6
SCIMP	6.5	1.1	1.8	0.1	0.9	0.7	3.7	0.0	1.1
SMIM4	4.6	1.7	1.6	0.4	0.6	1.2	1.8	0.7	3.2

LOC728606	5.1	1.1	1.8	0.3	0.8	0.8	4.0	0.3	1.8
C16orf74	4.9	1.9	2.2	0.7	1.1	1.1	1.7	0.6	1.7
LYPD1	3.4	1.5	1.9	0.6	1.2	2.0	1.9	0.7	2.7
SRRD	4.8	1.3	1.9	0.2	0.3	0.9	3.1	0.5	3.0
LOC101929497	5.3	0.8	2.0	0.5	0.6	0.8	3.9	0.2	1.7
MLH1	4.7	1.7	2.5	0.2	1.0	1.3	2.2	0.4	1.9
CSRP2BP	5.8	1.6	2.1	0.4	0.9	1.2	2.0	0.2	1.5
ASF1B	3.9	1.9	2.7	0.6	1.4	2.1	1.8	0.2	1.2
TTC23	4.7	1.6	2.1	0.3	0.8	1.2	2.8	0.4	1.9
PLEKHA2	5.4	1.7	2.2	0.2	0.7	1.1	2.5	0.5	1.6
GAN	3.6	1.3	1.9	0.2	0.5	0.6	4.1	0.5	3.1
RRAGD	4.8	1.9	1.8	0.2	0.3	0.7	3.4	0.4	2.2
ICK	3.1	1.2	1.6	0.3	0.6	1.0	3.8	0.8	3.6
LOC100506518	4.2	0.9	1.9	0.6	1.1	1.1	3.6	0.4	1.9
SLC25A30	4.0	1.5	1.9	0.3	0.7	1.0	3.9	0.3	2.2
ZCCHC17	3.7	1.4	1.9	0.1	0.9	0.8	2.9	0.6	3.4
FKBP15	4.5	1.6	2.0	0.4	0.9	1.1	2.5	0.4	2.4
XKR8	5.3	2.1	2.6	0.3	0.7	1.0	2.2	0.4	1.3
LOC101929695	5.3	1.4	2.4	0.5	1.3	0.8	2.4	0.4	1.4
MCM8	3.9	1.2	1.8	0.1	0.5	0.8	4.1	0.4	2.8
DOC2A	5.0	2.0	2.9	0.5	0.8	1.1	1.9	0.3	1.3
SNX7	4.1	1.7	2.3	0.3	0.6	0.7	3.3	0.4	2.4
CMTM4	4.7	1.5	2.3	0.2	0.6	0.9	3.6	0.3	1.6
NXPE2	5.8	0.7	2.0	0.3	0.6	0.7	3.4	0.3	1.9
ACAP3	5.1	1.8	2.4	0.4	1.1	1.0	2.5	0.4	1.2
ZNF426	4.6	1.7	2.1	0.1	0.6	0.6	3.6	0.3	2.2
PSPN	4.7	1.7	2.4	0.7	1.0	0.9	2.7	0.4	1.2
NECAP1	4.0	1.5	2.0	0.2	0.9	1.2	3.0	0.3	2.6
NKX3-1	3.6	1.3	1.8	0.6	0.8	1.1	3.1	0.5	3.0
CISD1	3.5	1.3	1.8	0.1	0.7	0.7	4.2	0.5	2.9
BAIAP2L1	4.4	1.5	2.0	0.3	1.0	1.1	3.1	0.5	1.9
CPB2-AS1	5.2	1.0	2.3	0.3	0.6	0.7	3.6	0.3	1.7
PIGX	4.7	2.1	2.0	0.4	0.9	1.1	2.2	0.4	1.8
CEP95	5.2	1.7	2.7	0.1	0.7	0.6	2.2	0.3	2.0
SNORD91B	2.3	1.2	2.3	0.7	2.5	1.0	2.2	0.3	3.1
PTP4A3	4.2	2.5	2.1	0.6	1.6	1.5	1.7	0.2	1.3
IFNAR1	4.6	1.6	2.1	0.1	0.5	0.7	3.1	0.4	2.6
ASB16-AS1	4.7	0.8	2.6	0.4	0.9	0.9	3.4	0.4	1.6
LOC101929012	3.9	1.7	2.6	0.4	1.0	1.1	2.4	0.6	2.0
NRF1	4.1	1.5	2.0	0.3	1.1	1.4	2.8	0.4	2.1
ZBED3	3.6	1.1	1.5	0.9	0.8	1.0	4.4	0.4	2.0
HIRIP3	4.0	1.8	2.0	0.4	0.7	1.5	2.7	0.4	2.0
RNFT2	5.0	1.9	2.2	0.3	0.7	0.9	2.6	0.4	1.6
AARS2	3.6	1.5	1.9	0.4	0.7	1.0	3.6	0.5	2.4
CTDSPL2	4.1	1.5	2.2	0.1	0.5	0.8	2.8	0.2	3.3
ZCCHC6	4.6	1.4	2.2	0.1	0.5	0.8	3.1	0.4	2.5
TIPARP-AS1	4.3	1.5	2.2	0.7	1.0	1.3	2.9	0.3	1.4
CDK5RAP2	4.5	1.6	2.3	0.3	0.8	1.1	2.5	0.4	2.1
ZNF398	4.1	1.3	1.8	0.5	1.1	1.2	2.8	0.4	2.5
BCORL1	4.5	1.5	2.2	0.4	0.8	0.9	2.5	0.5	2.2
TRAPPC6B	4.0	1.4	1.7	0.3	1.0	1.0	2.8	0.4	3.0
TRIT1	3.6	1.5	2.5	0.2	0.6	0.9	3.5	0.3	2.4
PIK3CD	5.1	1.9	2.3	0.5	0.8	1.4	1.8	0.2	1.6
RIMKLB	4.3	1.7	2.3	0.1	0.6	0.9	3.0	0.3	2.3
LINC00857	4.9	1.5	2.2	0.2	0.7	0.8	2.9	0.5	2.0

FAM138D	5.4	1.0	2.2	0.3	0.7	0.6	3.8	0.2	1.4
SPEG	3.7	1.3	2.1	0.6	0.9	2.1	3.2	0.4	1.3
MC1R	5.0	1.8	2.9	0.4	0.7	1.0	1.9	0.4	1.4
YAE1D1	4.0	1.5	2.7	0.3	0.4	1.6	2.1	0.4	2.5
ARID4A	4.1	1.4	1.8	0.1	0.5	0.7	3.6	0.4	3.0
WDR89	2.8	1.6	1.7	0.1	0.6	0.7	4.4	0.4	3.3
B4GALT6	4.0	1.9	1.8	0.2	1.1	0.6	3.0	0.2	2.8
DCLRE1B	3.5	1.4	1.3	0.2	0.9	1.4	3.2	0.6	3.0
RABEP2	4.7	1.5	2.1	0.7	0.8	1.2	2.5	0.4	1.5
LOC101927610	5.0	1.2	2.4	0.2	0.6	0.7	3.8	0.2	1.5
CASP7	4.2	1.8	2.0	0.2	0.7	1.0	3.1	0.5	1.9
EZH2	3.5	1.4	1.9	0.3	0.6	0.8	3.7	0.5	2.9
C3orf58	4.5	1.4	2.2	0.4	0.6	1.0	3.2	0.3	1.7
LANCL1	4.6	2.1	2.1	0.4	0.8	1.7	1.8	0.2	2.0
KIF7	2.5	0.8	1.1	0.5	0.9	1.0	4.7	0.8	3.2
FAM102B	4.5	1.6	2.1	0.1	0.2	0.8	3.5	0.3	2.2
SOS1	3.8	1.5	1.8	0.2	0.8	0.8	3.0	0.5	3.1
MORC3	3.8	1.4	2.2	0.2	0.5	0.9	3.3	0.6	2.6
INTS2	5.1	1.4	2.2	0.2	0.5	0.7	3.0	0.3	2.0
ADAMTS6	2.5	0.7	1.2	0.2	0.6	0.9	4.8	0.7	3.8
ZNF551	5.1	1.1	2.2	0.2	0.6	0.7	3.6	0.3	1.6
TYW1	4.2	1.3	1.9	0.1	0.6	0.8	3.4	0.4	2.7
USP31	3.6	1.3	1.7	0.4	0.9	0.9	3.0	0.6	3.1
ABCB8	4.5	1.7	2.5	0.5	1.3	1.0	2.2	0.3	1.5
ZNF521	3.0	1.0	1.4	0.3	0.8	1.1	3.9	0.6	3.5
PITPNA-AS1	3.7	1.6	2.3	0.4	0.8	0.9	3.1	0.6	2.1
EBAG9	4.3	2.3	2.8	0.2	0.9	1.4	1.9	0.2	1.5
CIT	4.0	1.5	2.0	0.6	1.3	1.5	2.3	0.4	1.7
CSRNP2	4.1	1.4	2.0	0.2	0.5	0.8	3.7	0.5	2.4
SLC45A4	3.9	1.3	2.0	0.6	1.1	1.0	2.9	0.6	2.2
TMCC1	5.0	1.9	2.4	0.3	0.7	0.9	2.1	0.3	1.8
LOC91548	4.9	1.0	2.2	0.3	0.6	0.8	3.3	0.3	1.8
ADM2	4.1	1.9	2.0	0.3	0.7	0.8	2.5	0.7	2.3
MTA3	4.7	1.5	2.4	0.2	0.9	0.9	2.3	0.2	2.2
INPP4A	4.1	1.3	1.7	0.4	0.8	0.9	3.5	0.4	2.2
TERF1	4.5	1.8	2.0	0.4	0.6	1.0	2.7	0.4	2.0
SLC25A19	3.1	1.4	1.6	0.4	0.7	1.2	3.8	0.4	2.9
LOC284551	5.2	0.9	2.2	0.3	0.6	0.8	3.6	0.2	1.6
GPD1L	4.3	1.7	2.3	0.2	0.4	0.9	3.2	0.3	2.0
WDR90	4.5	1.8	2.5	0.6	1.0	1.3	1.9	0.3	1.5
FNBP1L	4.3	1.5	2.3	0.2	0.6	1.0	2.3	0.6	2.6
TOE1	3.0	1.4	1.8	0.4	0.8	1.3	3.2	0.5	2.9
IGF2BP3	4.4	1.5	2.1	0.4	1.1	1.0	2.8	0.3	1.9
FEM1C	4.4	1.7	1.9	0.1	0.5	0.8	2.9	0.4	2.5
FAM89A	5.5	1.2	1.7	0.7	0.8	1.0	3.2	0.4	0.9
CNEP1R1	3.9	1.9	2.6	0.1	1.0	0.9	2.7	0.4	2.1
LOC101926915	4.7	1.0	2.0	0.2	0.6	0.7	4.2	0.4	1.6
LINC00381	5.7	1.2	1.8	0.4	0.5	1.0	2.7	0.4	1.6
TMED8	3.6	1.5	2.0	0.4	0.4	1.5	3.5	0.4	1.9
MRM1	2.8	1.7	2.2	0.5	1.1	1.9	3.0	0.5	1.7
KRIT1	5.2	1.6	2.3	0.1	0.7	0.8	2.0	0.3	2.4
MAPK11	3.5	1.9	2.2	0.7	0.8	1.3	2.8	0.3	1.9
GMNN	4.3	1.3	2.2	0.0	0.3	0.7	3.5	0.4	2.8
UBR3	4.3	1.3	1.9	0.2	0.5	0.8	3.2	0.5	2.6
ZNF839	4.0	1.5	2.0	0.5	0.8	1.1	3.3	0.2	2.0

LOC101928992	5.2	0.8	2.1	0.2	0.6	0.6	3.8	0.4	1.6
SHC3	4.3	1.6	2.5	0.3	0.8	1.0	2.7	0.4	1.8
LINC00696	5.1	1.1	1.7	0.2	0.6	0.7	4.0	0.3	1.6
LOC100506241	5.7	0.8	1.9	0.3	0.6	0.6	3.8	0.2	1.5
TCAP	4.7	2.1	2.5	0.2	0.6	0.8	1.9	0.4	2.1
CPEB4	3.7	1.2	1.6	0.4	0.8	1.1	3.2	0.5	2.8
NDC1	3.9	1.5	1.9	0.1	0.7	0.7	3.1	0.4	2.9
TTBK2	4.4	1.4	2.0	0.2	0.6	0.7	2.9	0.4	2.6
TRIP4	4.6	1.6	2.3	0.2	0.6	0.9	2.6	0.5	2.0
SNX14	4.5	1.6	2.3	0.2	0.5	0.8	3.2	0.3	2.0
MSRB1	3.2	1.2	1.9	0.5	0.8	1.4	2.8	0.5	3.0
LINC00959	5.0	0.9	2.1	0.4	0.7	0.6	3.7	0.2	1.6
ZNF32	4.1	1.5	2.9	0.2	0.5	1.0	3.2	0.4	1.6
SMIM20	4.5	1.8	3.0	0.5	1.5	1.7	1.1	0.3	0.9
ING2	4.7	1.7	1.7	0.4	0.9	0.9	2.2	0.5	2.3
ANKRD42	4.0	1.6	1.8	0.6	0.6	1.4	2.3	0.4	2.4
RHOU	4.7	1.0	2.2	0.5	1.1	0.9	2.8	0.2	1.8
FLJ33360	4.9	1.0	2.0	0.3	0.7	0.8	3.6	0.3	1.7
RGAG4	4.8	1.7	2.2	0.4	0.8	0.9	2.3	0.5	1.8
USP38	4.1	1.5	2.0	0.2	0.5	0.9	3.3	0.4	2.4
FUNDC1	4.9	1.1	2.1	0.1	0.8	0.4	2.9	0.5	2.5
AVEN	4.0	1.1	1.4	0.2	0.4	0.7	4.3	0.5	2.7
HSD17B7	6.0	1.7	3.1	0.1	0.2	0.6	1.9	0.1	1.5
POLR3B	3.5	1.2	1.5	0.1	0.7	0.9	3.9	0.6	2.8
MTF2	4.1	1.7	1.9	0.1	0.4	0.8	3.8	0.4	2.2
COQ5	4.7	1.9	3.4	0.2	0.7	0.9	1.9	0.2	1.4
STIM2	3.6	1.5	1.9	0.4	0.7	0.7	3.2	0.7	2.5
CHM	4.2	1.6	1.9	0.2	0.7	1.0	2.9	0.3	2.4
SEMA3B	4.6	1.7	2.3	0.5	1.1	1.3	2.0	0.2	1.4
DYX1C1-CCPG1	4.2	1.9	2.2	0.1	0.6	1.0	2.8	0.1	2.3
WNT7B	3.9	1.1	1.7	1.2	2.0	2.8	1.2	0.4	1.0
DCUN1D3	3.8	1.6	2.1	0.3	0.3	0.8	3.6	0.3	2.5
SOCS6	3.6	1.5	2.1	0.3	0.8	0.7	3.3	0.5	2.4
PHTF2	2.9	1.3	1.4	0.1	0.4	0.6	4.3	0.6	3.5
DPY19L1	3.9	1.6	2.0	0.2	0.5	1.0	3.2	0.3	2.6
LINC00700	4.8	0.9	2.1	0.2	0.6	0.6	3.8	0.3	1.7
CNNM4	3.7	1.6	2.1	0.5	0.7	1.2	3.0	0.5	1.8
PET112	4.2	2.0	2.6	0.3	0.8	0.8	2.3	0.4	1.8
LOC100288911	4.4	1.7	2.1	0.4	0.4	0.8	2.4	0.6	2.4
KLHL4	4.1	1.5	2.2	0.2	0.9	1.2	2.5	0.4	2.1
VRK2	4.0	1.5	2.3	0.2	0.5	0.9	3.1	0.4	2.3
CYP20A1	4.1	1.1	1.9	0.2	0.8	0.8	3.1	0.3	2.9
MIR181A1HG	4.0	0.9	2.4	0.6	1.3	1.2	2.9	0.3	1.7
CYP26B1	3.9	1.5	2.0	0.5	1.0	1.5	2.5	0.5	1.7
ARL13B	3.7	1.2	1.9	0.2	0.6	0.8	3.4	0.5	2.9
SCARNA18	3.8	3.1	2.0	0.6	0.8	0.6	2.1	0.6	1.5
RFC3	3.0	1.5	1.9	0.3	0.8	0.9	3.3	0.7	2.8
ETNK2	4.3	1.8	2.1	0.5	1.0	1.0	2.2	0.4	1.9
SZT2	4.8	1.7	2.5	0.5	1.0	1.2	1.9	0.3	1.3
NLRC5	4.6	1.9	2.7	0.5	1.0	1.1	1.9	0.3	1.1
PTGR1	3.7	1.5	1.6	0.2	0.7	0.8	3.7	0.5	2.4
AIFM1	4.2	1.8	2.2	0.3	0.7	0.8	2.3	0.5	2.3
TRIM38	4.6	1.3	2.0	0.2	0.6	0.6	3.5	0.4	1.9
PSD4	3.5	1.5	2.1	0.5	1.2	1.2	2.6	0.4	2.0
GCLC	3.7	1.3	1.6	0.3	0.6	0.9	3.1	0.5	3.0

FLJ22184	4.8	1.8	2.3	0.4	0.6	0.8	2.1	0.4	1.9
TMEM80	4.8	1.9	2.1	0.4	0.6	1.0	2.4	0.4	1.6
LOC100506713	4.7	1.2	2.0	0.5	0.8	0.9	2.9	0.2	1.9
DMD	4.9	1.5	2.2	0.3	0.8	1.1	2.2	0.3	1.8
CNOT4	3.9	1.5	2.1	0.3	0.6	0.8	2.3	0.5	3.0
TAX1BP3	4.8	1.8	2.0	0.6	1.4	1.0	1.6	0.4	1.5
CFB	6.5	2.9	3.6	0.1	0.5	0.5	0.4	0.1	0.4
CATSPER1	3.8	0.9	1.9	0.4	1.2	1.3	3.6	0.4	1.7
RNF24	3.6	1.7	1.9	0.1	0.9	1.0	3.2	0.4	2.3
PANK1	3.5	1.4	2.0	0.3	0.5	0.8	3.5	0.6	2.6
RB1	4.7	1.5	2.1	0.1	0.5	0.8	2.5	0.4	2.4
GALNT14	3.9	1.5	1.8	0.5	0.6	0.9	2.9	0.6	2.3
DNAJC30	3.8	1.4	1.8	0.5	1.1	1.4	2.6	0.5	1.9
PPCDC	4.6	1.4	2.2	0.4	0.9	1.4	2.0	0.4	1.7
TTLL4	3.9	1.3	2.1	0.3	0.7	0.9	3.1	0.4	2.3
ZBTB37	3.9	1.4	2.2	0.2	0.8	0.9	3.2	0.5	2.0
RB1CC1	4.5	1.5	2.2	0.1	0.8	1.2	2.5	0.3	1.9
CDKN2AIP	3.7	1.5	2.0	0.2	0.4	1.1	2.9	0.5	2.6
ANKMY1	4.8	1.4	2.6	0.7	0.8	1.1	2.0	0.3	1.3
TMEM154	4.3	1.0	1.9	0.2	0.2	0.7	3.8	0.5	2.4
LOC100126447	4.6	1.4	2.4	0.1	0.4	0.5	2.4	0.5	2.7
ERO1LB	3.4	1.3	2.0	0.2	0.3	0.7	3.7	0.6	2.8
LOC100144602	5.2	1.8	2.2	0.7	0.9	1.0	1.5	0.4	1.3
LOC101928243	3.9	1.8	2.2	0.4	0.6	0.9	2.4	0.5	2.2
MYBL2	3.2	1.3	1.6	0.4	1.0	1.2	2.9	0.4	3.0
LOC101928795	4.2	1.0	2.3	0.5	0.8	0.7	3.4	0.4	1.6
USP47	3.7	1.2	1.8	0.1	0.5	0.9	3.8	0.3	2.6
PARP8	3.8	1.6	2.2	0.3	1.0	1.1	2.5	0.3	2.1
RAD52	5.1	1.5	2.4	0.4	0.7	1.0	2.0	0.4	1.6
DTNB	4.7	1.3	2.4	0.4	0.6	1.1	2.3	0.2	1.9
HDAC4	4.4	1.3	2.2	0.6	0.8	1.0	2.5	0.4	1.8
LOC101927054	5.1	1.0	2.3	0.2	0.6	0.8	3.2	0.3	1.4
BPHL	4.7	2.0	2.4	0.3	0.8	1.3	2.1	0.2	1.1
LOC100272217	4.5	1.0	2.3	0.4	0.6	0.8	3.6	0.3	1.5
FAM122B	3.5	1.1	1.6	0.4	1.2	1.1	2.3	0.7	3.1
RHPN2	3.6	1.2	1.5	0.3	0.5	0.7	3.8	0.7	2.7
WIBG	3.7	1.8	2.3	0.5	0.7	2.0	1.8	0.4	1.7
LOC101060691	4.5	1.1	1.6	0.1	0.7	0.8	3.3	0.3	2.5
ZDHHC2	3.5	1.3	2.2	0.3	1.1	1.5	2.4	0.5	2.3
FANCL	3.5	1.6	1.5	0.1	0.7	1.0	3.7	0.3	2.4
MIER3	4.1	1.1	2.0	0.1	0.6	0.7	3.8	0.3	2.3
FAH	4.3	1.4	1.9	0.7	1.5	2.2	1.6	0.2	1.1
ARHGEF9	3.6	1.5	1.7	0.3	0.8	1.4	2.7	0.4	2.3
POLL	4.4	1.7	2.4	0.4	1.1	1.2	1.9	0.3	1.5
SLC25A43	4.2	1.5	2.1	0.3	0.7	0.9	2.4	0.5	2.3
NF2	3.1	1.1	1.5	0.3	0.6	1.2	3.9	0.6	2.7
CEP89	4.1	1.2	2.1	0.3	0.6	0.9	2.8	0.4	2.4
C17orf103	3.9	1.9	1.7	0.5	1.1	1.1	2.9	0.3	1.7
TRNAA1	2.8	0.4	0.4	1.4	1.0	0.8	4.0	0.8	3.4
PRSS12	4.0	1.5	2.0	0.5	1.0	1.4	2.5	0.3	1.6
DHFR	3.7	1.7	1.9	0.2	0.4	0.9	3.0	0.4	2.7
RCL1	4.5	1.5	2.1	0.2	0.5	0.7	2.8	0.4	2.1
RASSF8	3.9	1.3	1.9	0.1	0.7	0.8	3.2	0.3	2.6
ASH1L-AS1	3.7	1.3	2.2	0.5	0.7	1.1	3.0	0.4	2.1
ZFP91	3.2	1.6	1.5	0.1	0.5	0.5	3.5	0.3	3.6

MID2	5.0	1.5	2.2	0.2	0.4	0.6	2.5	0.4	2.0
POC1A	3.1	1.4	2.0	0.3	0.9	1.6	2.9	0.3	2.3
RAD50	3.8	1.5	1.8	0.1	0.6	0.8	3.2	0.5	2.5
GALNT11	3.6	1.5	1.8	0.3	0.6	1.3	3.1	0.5	2.3
LOC100379224	5.5	1.6	2.5	0.1	0.5	0.5	2.2	0.2	1.7
TATDN1	4.2	1.6	2.3	0.1	0.4	0.7	2.7	0.3	2.5
LYRM1	3.4	1.4	3.0	0.2	1.2	1.2	1.4	0.7	2.3
SPATA6L	4.9	1.2	1.7	0.2	0.6	0.6	3.5	0.3	1.8
CYP4V2	4.3	1.7	2.0	0.5	1.4	1.5	1.9	0.2	1.2
METTL22	3.5	1.4	2.0	0.5	0.6	1.0	2.7	0.5	2.6
AMOT	3.3	1.1	1.7	0.3	0.8	1.0	2.6	0.7	3.3
KIAA1551	5.6	1.6	2.4	0.1	0.6	0.9	1.9	0.2	1.5
SNORD83B	4.5	1.2	1.4	0.9	1.2	1.0	3.0	0.9	0.7
LOC339166	4.9	1.2	2.3	0.3	0.6	0.8	2.3	0.3	1.9
PAPLN	4.8	1.1	2.1	0.3	0.6	0.6	3.5	0.3	1.5
SLC35A1	4.5	1.4	2.4	0.1	0.5	0.8	3.2	0.3	1.7
RECQL	3.6	1.7	1.9	0.1	0.5	0.8	3.1	0.4	2.6
MTERFD1	3.8	1.2	2.0	0.2	0.5	0.8	3.3	0.3	2.8
CUTC	3.0	1.5	2.2	0.2	0.6	0.7	3.3	0.4	2.8
TAF5	4.1	1.4	2.0	0.3	0.5	0.7	3.1	0.4	2.2
WDR92	3.9	1.9	1.8	0.2	0.9	1.2	2.6	0.3	2.0
GALNT3	4.0	1.4	1.6	0.1	0.6	0.7	3.3	0.4	2.6
ZNF16	3.7	1.5	1.8	0.2	0.9	0.8	3.2	0.4	2.2
PLA2G12A	4.4	1.8	1.7	0.3	0.6	1.0	2.5	0.4	2.0
VANGL1	4.2	1.9	2.2	0.5	1.0	1.3	1.6	0.5	1.6
IQCB1	4.2	1.2	2.3	0.2	0.5	0.7	2.8	0.4	2.4
ZKSCAN5	4.0	1.1	1.7	0.4	0.7	1.1	3.0	0.3	2.4
VSIG10	4.4	1.8	2.1	0.3	0.7	1.0	2.5	0.3	1.5
ECM1	3.7	1.8	2.2	0.5	0.8	1.4	1.6	0.5	2.2
LOC101929157	2.6	0.6	1.9	0.4	0.7	0.9	3.7	0.5	3.5
SNORD45A	5.4	2.0	1.1	0.2	0.9	0.3	2.1	0.6	2.0
C6orf70	4.8	1.5	2.0	0.1	0.6	0.7	2.6	0.3	2.0
HSF2	3.7	1.3	1.7	0.1	0.4	0.8	3.6	0.5	2.6
FBXL20	4.2	1.7	1.8	0.1	0.6	1.0	2.7	0.4	2.2
CCNC	4.0	1.6	2.5	0.2	0.6	0.9	2.3	0.6	2.0
LANCL2	3.6	1.3	2.0	0.3	1.0	0.9	2.9	0.4	2.2
LINC00678	4.3	1.6	2.6	0.2	0.7	0.8	2.0	0.3	2.1
TM4SF19-TCTEX1D2	4.4	1.4	2.0	0.5	0.9	1.2	2.6	0.2	1.3
SEMA4F	5.2	1.1	2.7	0.3	0.5	0.8	2.6	0.2	1.3
SLC25A25	3.0	1.3	1.5	0.4	0.5	0.6	3.9	0.6	2.9
TPH2	5.0	1.0	2.3	0.2	0.7	0.8	2.7	0.3	1.7
TRAF6	3.9	1.0	2.0	0.3	0.5	0.8	3.0	0.5	2.7
SPA17	4.2	1.7	3.2	0.5	0.4	1.3	1.2	0.5	1.7
LOC101927152	4.9	1.0	2.4	0.2	0.5	0.6	3.4	0.3	1.4
PRKRIP1	3.2	1.9	2.2	0.5	0.9	0.8	2.1	0.8	2.3
BOLA3-AS1	4.6	1.2	2.0	0.3	0.5	0.7	3.4	0.3	1.6
ATR	4.6	1.3	2.1	0.2	0.6	0.8	3.1	0.3	1.7
WTIP	2.8	1.3	1.3	0.6	0.8	0.8	4.1	0.6	2.3
SNORA11	5.2	3.0	1.2	0.3	0.4	0.4	1.9	0.2	2.0
THAP1	3.5	1.8	1.9	0.4	0.5	1.1	3.3	0.2	1.9
ZNF805	4.2	1.4	2.0	0.2	0.4	0.7	3.2	0.3	2.3
PHKA2	4.8	1.8	2.6	0.2	0.7	0.8	2.0	0.3	1.6
DDHD2	4.1	1.6	2.1	0.3	0.7	0.8	2.7	0.4	1.9
FAM171A2	4.1	1.7	2.0	0.5	0.7	1.0	2.3	0.5	1.6
NR3C2	4.3	1.4	1.8	0.4	0.8	1.4	2.3	0.4	1.9

PAXBP1	4.0	1.4	2.0	0.2	0.5	0.7	3.2	0.4	2.1
ANKRD12	4.1	1.4	1.8	0.1	0.4	0.7	3.1	0.3	2.6
APOC1	4.1	2.1	3.7	0.0	0.4	1.1	1.6	0.2	1.4
TTC12	4.9	1.6	2.2	0.2	0.8	0.8	2.1	0.1	1.8
PLAC1	4.9	1.5	2.4	0.3	0.6	0.8	1.6	0.5	1.9
SCFD2	3.8	1.5	1.9	0.4	0.7	1.4	2.6	0.4	1.9
LOC101928020	4.4	1.3	2.2	0.2	0.8	0.9	2.5	0.3	1.9
AMIGO3	4.3	1.3	2.2	0.5	1.0	0.9	2.4	0.5	1.3
UBN2	4.1	1.3	2.0	0.3	0.7	0.9	2.5	0.4	2.3
CPNE8	4.4	1.7	2.0	0.2	0.7	0.7	2.6	0.3	2.0
LOC100128006	4.2	1.0	2.3	0.3	0.7	0.7	3.5	0.2	1.7
IPO11	4.1	1.6	1.9	0.1	0.5	0.8	2.7	0.4	2.5
MTMR10	4.1	1.6	2.1	0.2	0.6	1.0	2.6	0.4	2.0
WBP4	4.2	1.9	2.0	0.1	0.6	0.7	2.4	0.4	2.3
LOC101927469	4.6	1.1	2.1	0.4	0.5	0.8	2.8	0.4	1.7
SLC22A15	3.0	1.1	1.8	0.2	0.7	0.9	3.5	0.5	2.8
CAMK1	3.4	1.8	2.1	0.4	0.7	1.3	2.3	0.5	2.0
TYRO3	4.0	1.1	1.8	0.3	0.6	0.8	3.2	0.5	2.2
NR4A1	3.3	1.6	3.2	0.3	0.4	0.9	2.5	0.4	1.9
GPR78	4.6	1.2	2.0	0.3	0.6	0.7	3.4	0.3	1.5
SPG20OS	4.6	1.5	2.3	0.6	0.7	0.8	2.5	0.3	1.1
GTF2IRD2	6.3	1.2	0.9	0.2	0.5	0.2	3.7	0.2	1.4
GCC2	4.0	1.7	2.0	0.1	0.7	0.8	2.8	0.3	2.2
ZNF473	2.9	1.0	1.3	0.2	0.7	0.7	3.8	0.6	3.3
SAMD10	3.6	1.3	1.7	0.8	1.2	1.6	2.3	0.4	1.6
SLC26A1	4.8	1.5	2.2	0.6	0.9	1.2	1.5	0.3	1.4
LOC101928262	3.8	1.4	2.2	0.4	0.6	1.0	3.0	0.3	1.8
LOC101926954	4.1	1.4	1.7	0.3	0.6	1.1	2.8	0.3	2.2
FOXJ2	3.5	1.4	1.6	0.4	0.7	1.2	2.4	0.5	2.7
TBC1D7	3.3	1.7	2.2	0.2	0.5	0.8	3.2	0.2	2.4
DLG5-AS1	5.0	0.9	1.6	0.3	0.7	0.7	3.5	0.3	1.5
SLC30A7	4.0	1.7	1.9	0.2	0.6	0.9	2.6	0.3	2.3
CELSR1	4.4	1.5	1.9	0.6	1.2	1.2	2.1	0.3	1.4
NUDT14	5.5	1.5	2.0	0.6	1.0	1.2	1.4	0.4	1.0
PTENP1-AS	4.8	1.3	1.6	0.5	0.5	0.7	3.3	0.3	1.3
MOB1B	4.2	1.1	1.6	0.0	0.7	0.7	2.4	0.3	3.4
STAU2	4.3	1.6	2.2	0.1	0.4	0.8	2.3	0.3	2.4
ARID4B	3.8	1.3	1.9	0.1	0.6	0.7	3.1	0.4	2.4
SPRED3	4.4	0.9	2.2	0.3	0.6	0.9	3.3	0.3	1.5
FAM122A	3.0	1.0	1.8	0.4	1.0	1.2	2.8	0.5	2.8
LOC101928927	4.1	1.6	1.9	0.3	0.4	0.8	2.3	0.6	2.4
SMARCAD1	4.4	1.7	2.0	0.1	0.4	0.7	2.7	0.3	2.1
LOC101929717	4.4	1.1	1.7	0.3	0.6	0.8	3.3	0.4	1.7
PRH1-PRR4	3.6	1.5	2.0	0.4	0.7	1.1	2.5	0.4	2.2
HTATIP2	5.2	1.4	2.6	0.2	0.4	1.1	2.3	0.2	1.0
TRAF3IP1	4.3	1.7	1.6	0.3	0.9	0.8	2.3	0.5	2.0
LOC646021	4.0	1.4	1.7	0.4	0.7	0.9	3.2	0.4	1.8
WDR25	4.1	1.5	1.8	0.7	1.0	1.2	2.2	0.4	1.6
SCARNA4	4.0	1.2	3.3	0.2	0.6	0.5	3.0	0.5	1.2
LOC101926964	4.8	1.0	1.9	0.2	0.6	0.6	3.3	0.3	1.8
C1GALT1C1	3.9	1.8	2.4	0.1	0.5	0.7	3.2	0.3	1.6
C14orf80	3.6	1.4	1.7	0.7	1.1	1.0	3.2	0.4	1.3
PARS2	3.7	1.4	2.0	0.3	0.6	1.1	2.7	0.5	2.1
PKMYT1	2.7	1.2	1.6	0.6	1.3	1.5	2.7	0.5	2.4
PIK3C2A	4.0	1.3	1.8	0.1	0.4	0.9	3.4	0.4	2.2

LRRFIP2	3.5	1.3	1.7	0.2	0.6	0.8	3.2	0.5	2.7
C8orf58	3.4	1.2	2.2	0.5	0.9	1.2	2.2	0.5	2.3
ZNF143	3.5	1.5	1.5	0.2	0.6	1.1	2.7	0.5	2.8
ST6GALNAC5	3.4	1.6	1.7	0.5	1.0	1.1	2.4	0.4	2.2
DUS2	4.0	1.3	1.9	0.4	0.7	1.1	2.7	0.4	1.9
LINC00488	4.9	1.0	1.7	0.3	0.6	0.7	3.4	0.2	1.6
C1R	6.4	2.5	3.2	0.2	0.5	0.8	0.3	0.1	0.2
TRIM24	3.3	1.5	1.5	0.3	0.7	1.0	3.0	0.5	2.5
FGFBP3	3.9	1.5	2.1	0.6	1.0	0.8	2.5	0.5	1.4
ZNF701	4.7	1.2	2.0	0.3	0.6	0.6	3.3	0.3	1.3
LCTL	4.5	0.8	2.1	0.3	0.6	0.8	3.5	0.3	1.6
MAP4K2	3.7	1.5	2.0	0.4	0.9	1.2	2.3	0.5	1.9
CCDC117	4.0	1.2	2.0	0.2	0.6	0.9	3.4	0.4	1.8
LOC286190	4.7	0.9	1.9	0.3	0.6	0.7	3.5	0.2	1.5
FUT4	3.5	1.4	2.0	0.4	1.0	0.9	3.1	0.4	1.6
CCNG2	4.8	1.9	2.7	0.1	0.6	0.5	2.0	0.2	1.6
PREPL	3.6	1.2	1.8	0.2	1.1	1.0	2.8	0.4	2.2
CDKAL1	4.0	1.5	1.8	0.2	0.6	0.8	3.0	0.3	2.3
ANKRD36B	4.2	1.1	2.1	0.4	0.6	1.0	2.5	0.3	2.1
SFI1	4.1	1.5	2.4	0.6	0.9	1.2	2.2	0.3	1.3
STXBP2	4.6	1.7	2.0	0.3	0.5	0.6	2.3	0.5	1.7
DBT	4.0	1.3	1.9	0.1	0.6	0.7	3.0	0.4	2.4
SMC5	4.1	1.5	1.9	0.1	0.4	0.8	2.9	0.4	2.4
WWP1	4.1	1.4	2.2	0.1	0.7	0.7	2.9	0.4	1.9
C1R	6.4	2.2	3.8	0.1	0.4	0.5	0.4	0.1	0.4
MPHOSPH9	3.8	1.4	1.7	0.1	0.7	0.9	2.8	0.3	2.6
ACTR3B	3.9	1.0	1.5	0.4	0.5	0.8	3.3	0.5	2.3
RSBN1L	4.3	1.3	2.2	0.3	0.6	0.7	2.3	0.4	2.1
TBCCD1	3.6	1.2	2.0	0.2	0.8	0.9	3.2	0.2	2.2
CLIC6	3.9	1.7	2.0	0.4	0.6	0.8	2.6	0.4	1.9
NEIL2	3.7	1.3	1.9	0.1	0.8	0.9	3.0	0.4	2.2
TMEM238	2.9	1.7	2.1	0.9	1.2	0.7	2.6	0.5	1.8
LZTFL1	4.2	1.8	2.2	0.1	0.4	0.7	2.2	0.4	2.2
LINC00942	4.8	0.8	1.9	0.3	0.5	0.5	3.6	0.2	1.7
MLF1IP	3.5	1.2	1.6	0.1	0.9	1.1	2.9	0.3	2.7
PACRGL	4.5	1.1	2.2	0.2	0.7	0.8	2.1	0.3	2.3
CCDC127	4.6	1.3	2.0	0.2	0.6	0.7	2.7	0.3	1.9
TMEM86B	3.9	0.9	1.8	0.4	0.7	0.5	3.1	0.5	2.4
LOC399900	4.6	0.9	2.2	0.3	0.6	0.6	3.3	0.2	1.4
GJA1	4.3	1.7	2.0	0.1	0.8	0.7	2.2	0.5	1.9
TAF1A	3.1	1.1	1.7	0.0	0.2	0.6	3.8	0.3	3.3
SMUG1	3.7	1.0	2.0	0.4	0.5	1.5	3.2	0.2	1.6
SPG11	4.6	1.5	2.1	0.2	0.6	0.8	2.2	0.3	1.8
HRSP12	3.7	2.0	2.5	0.3	0.9	1.0	1.8	0.3	1.7
FBXO42	3.0	1.4	1.8	0.2	0.6	0.8	3.5	0.4	2.5
USP6NL	3.6	1.2	1.8	0.4	0.7	1.0	2.5	0.5	2.7
LOC101928142	4.0	1.5	2.1	0.9	0.9	1.4	2.4	0.1	0.9
LCLAT1	3.8	1.8	2.0	0.1	0.4	0.7	2.8	0.4	2.3
BDP1	3.9	1.0	1.6	0.1	0.6	0.7	3.4	0.4	2.5
EPGN	4.5	0.8	2.1	0.2	0.6	0.6	3.4	0.3	1.6
LOC101928458	3.1	1.2	1.3	0.4	1.4	1.4	2.4	0.4	2.5
FRS3	3.6	1.5	1.8	0.7	1.0	1.6	1.8	0.4	1.7
SLC44A1	4.5	1.7	2.3	0.3	0.6	0.8	2.0	0.3	1.8
POLD3	3.9	1.6	2.0	0.2	0.6	0.7	2.5	0.4	2.3
ASB8	4.4	1.2	1.7	0.2	0.5	0.9	2.9	0.3	2.0

ARHGAP10	3.6	1.6	1.8	0.2	0.6	1.0	2.9	0.4	2.1
POLB	4.2	1.6	2.6	0.1	0.3	0.8	2.2	0.4	1.9
KIAA2018	4.4	1.3	1.9	0.3	0.7	1.1	2.1	0.4	2.0
THOC1	3.9	1.4	2.0	0.2	0.6	0.8	2.6	0.2	2.5
DNAH17-AS1	4.7	1.0	2.0	0.2	0.6	0.6	3.4	0.3	1.4
KRAS	4.1	1.8	2.1	0.2	0.6	0.8	2.5	0.3	1.9
PLAA	3.4	1.1	1.5	0.2	0.8	0.8	3.3	0.5	2.5
PRRT3-AS1	3.1	1.5	1.2	0.4	0.5	1.0	3.1	0.3	3.0
LOC100505811	4.0	1.0	2.0	0.6	0.7	0.8	2.7	0.4	1.8
HEXIM2	4.4	1.6	2.6	0.4	0.9	0.9	2.1	0.2	1.2
TMCO4	4.2	1.5	1.8	0.4	0.7	1.3	2.6	0.2	1.5
TOPORS	3.3	1.4	1.7	0.2	0.6	0.9	3.1	0.3	2.7
BAZ1A	3.2	1.1	1.4	0.1	0.6	0.8	3.6	0.4	2.9
ZNF226	4.5	1.6	2.3	0.2	0.2	0.6	2.7	0.2	1.9
TAPSAR1	5.1	1.3	2.0	0.3	0.7	0.8	2.4	0.3	1.2
GSTT2	2.6	1.9	1.0	0.5	0.6	1.8	2.8	0.3	2.5
ZNF236	4.2	1.3	1.9	0.4	0.8	1.0	2.2	0.3	1.9
SPRYD3	4.4	1.7	1.7	0.4	1.2	1.1	1.8	0.3	1.5
NUDT2	3.8	1.9	2.2	0.2	0.9	1.4	1.5	0.2	2.0
AP4B1	4.2	1.2	2.1	0.2	0.6	0.9	2.5	0.3	1.9
LOC101928509	4.6	0.8	2.1	0.3	0.6	0.6	3.4	0.2	1.4
C2CD5	3.5	1.3	1.4	0.1	0.6	0.8	3.3	0.4	2.8
NSUN4	4.0	1.0	1.9	0.2	0.7	0.8	3.2	0.4	1.9
PRADC1	3.8	1.5	1.9	0.3	1.3	1.2	1.7	0.8	1.6
LOC101927578	4.4	0.8	1.9	0.3	0.6	0.7	3.2	0.3	1.9
GINS3	3.7	1.4	1.4	0.3	1.1	0.7	2.9	0.6	1.9
LINC00466	4.5	0.8	1.8	0.4	0.7	0.9	3.2	0.3	1.5
IQCE	4.5	1.6	2.1	0.4	0.7	1.1	1.8	0.3	1.4
FGFR4	3.8	1.8	2.9	0.3	0.6	0.7	2.1	0.3	1.5
LOC100996442	4.4	1.4	2.4	0.1	0.7	0.9	2.3	0.1	1.8
MORN4	4.4	1.3	2.1	0.3	0.8	0.8	1.7	0.3	2.3
LOC101060542	4.8	1.0	2.2	0.3	0.5	0.5	3.2	0.2	1.3
RNF146	4.9	1.4	1.9	0.2	0.4	0.8	2.4	0.3	1.6
STK17B	4.1	1.6	1.6	0.2	0.5	0.9	2.7	0.2	2.3
SH2D4A	3.7	1.5	1.9	0.2	0.6	0.8	2.3	0.3	2.6
HEY1	3.7	1.3	2.0	0.5	0.6	0.9	2.7	0.4	1.9
DLEU1	3.6	1.3	2.0	0.2	0.5	1.0	2.7	0.5	2.3
STK3	4.1	1.6	1.8	0.3	0.7	1.0	2.8	0.4	1.5
DDI2	3.2	1.4	2.0	0.4	0.5	0.8	2.8	0.5	2.4
AOC2	4.2	1.5	2.4	0.3	0.3	0.5	2.7	0.4	1.8
LOC101928120	4.0	2.0	2.5	0.4	0.6	0.9	2.1	0.3	1.2
MKL2	4.2	1.1	1.7	0.4	0.9	1.1	2.2	0.4	2.0
ZEB2	3.6	1.3	1.4	0.3	0.6	1.0	2.8	0.5	2.5
USP42	3.4	1.2	1.6	0.2	0.7	0.8	3.1	0.5	2.5
ZNF354A	3.5	1.4	1.8	0.2	0.6	0.7	3.0	0.3	2.5
KLC1	4.1	1.2	1.8	0.2	0.5	0.9	3.2	0.4	1.7
PRR14L	4.0	1.2	1.8	0.1	0.5	0.8	2.6	0.4	2.5
TNFRSF1B	4.7	1.0	2.0	0.2	0.6	0.6	3.2	0.3	1.5
RTEL1-TNFRSF6B	4.0	1.4	2.2	0.4	1.1	1.3	2.1	0.3	1.2
ATP11A	3.6	1.2	1.6	0.3	0.5	0.9	3.1	0.5	2.4
SHROOM1	4.0	1.1	2.1	0.5	0.8	1.1	2.4	0.5	1.6
GABPB1	3.0	1.2	1.9	0.2	0.5	0.7	3.4	0.4	2.6
PGM5	3.5	0.9	1.6	0.2	0.4	0.6	4.1	0.3	2.4
LINC00984	4.0	0.8	1.3	0.5	0.5	0.7	3.6	0.4	2.0
WDR4	3.6	0.8	1.6	0.3	0.7	0.9	3.4	0.4	2.2

FN1	5.5	1.5	2.2	0.2	0.5	0.7	1.6	0.2	1.4
ACOT13	4.0	1.1	2.5	0.2	0.5	0.7	2.9	0.3	1.7
PLEKHA8	3.3	1.3	1.7	0.1	0.3	0.5	3.9	0.2	2.6
CARD11	4.2	1.4	2.0	0.4	0.7	0.8	2.2	0.5	1.6
CWF19L2	4.3	1.3	1.9	0.1	0.6	0.7	2.8	0.2	1.9
TCF7	4.4	0.9	1.9	0.3	0.5	0.9	3.0	0.3	1.7
FANCF	3.4	1.3	2.1	0.3	0.4	0.9	3.4	0.6	1.5
KIAA1468	3.7	1.4	1.8	0.4	0.7	1.0	2.7	0.4	1.8
CDC40	4.3	1.7	1.8	0.3	0.7	0.7	2.3	0.3	1.9
FASTKD2	3.9	1.2	2.0	0.1	0.6	0.7	2.9	0.4	2.2
DOK3	4.1	1.2	2.6	0.3	0.6	0.8	2.4	0.4	1.5
EOGT	3.9	1.3	2.3	0.2	0.6	0.8	2.6	0.3	2.0
ARRDC3	4.8	1.9	2.7	0.2	0.7	1.0	1.3	0.2	1.1
APC	3.7	1.1	1.7	0.2	0.5	0.7	3.1	0.4	2.5
LOC101927211	4.5	0.9	1.9	0.3	0.7	0.9	3.2	0.1	1.4
CBR4	4.8	1.4	3.0	0.3	0.7	0.8	1.6	0.3	1.0
CLN8	3.5	1.0	1.6	0.3	0.6	0.7	2.9	0.4	2.8
PAXIP1-AS1	4.2	1.1	1.9	0.3	0.5	0.8	3.5	0.3	1.4
HES7	2.4	0.6	1.3	0.7	0.7	0.8	3.8	0.7	2.9
CHEK1	3.6	1.4	1.8	0.2	0.6	0.7	2.9	0.3	2.4
ZNF561	3.6	1.3	2.0	0.1	0.5	0.7	3.0	0.4	2.4
ANAPC10	3.1	1.2	1.8	0.1	0.7	0.8	3.2	0.3	2.7
ARHGEF19	3.2	1.4	1.7	0.5	0.8	0.9	3.2	0.3	1.9
PTK6	3.8	1.5	2.4	0.5	0.9	1.4	2.0	0.3	1.0
ANKRD46	3.2	1.3	2.6	0.1	0.4	0.4	3.3	0.3	2.3
ATG16L2	4.2	1.3	2.4	0.6	1.1	1.2	1.7	0.3	1.0
PLCXD1	4.6	1.0	1.9	0.3	0.5	0.6	3.2	0.3	1.5
PLCXD1	4.6	1.0	1.9	0.3	0.5	0.6	3.2	0.3	1.5
IRF7	3.6	1.2	2.2	0.6	0.8	0.9	2.2	0.5	1.9
EFCAB2	4.4	0.9	1.9	0.2	0.5	0.7	3.3	0.2	1.8
ZNF451	3.6	1.1	1.8	0.2	0.5	0.9	3.0	0.3	2.5
RNPC3	4.3	1.4	2.3	0.1	0.6	1.0	2.0	0.3	1.9
ZNF195	4.5	1.1	1.9	0.3	0.6	0.8	2.6	0.3	1.7
BRCA1	3.9	0.9	1.7	0.2	0.6	0.7	3.6	0.3	1.9
N4BP1	3.9	1.4	1.8	0.2	0.5	0.7	2.8	0.3	2.2
LOC101929479	3.4	1.3	2.2	0.4	1.0	1.0	2.3	0.4	1.9
C9orf41	3.4	1.5	2.0	0.5	0.6	0.8	2.6	0.3	2.1
ORAOV1	4.0	1.5	2.0	0.3	0.6	0.9	2.8	0.3	1.4
GCH1	4.4	2.0	2.0	0.2	0.4	0.3	2.6	0.2	1.6
EFNA4	3.5	1.4	2.3	0.4	0.5	0.6	2.6	0.3	2.2
DPH3P1	3.7	1.7	2.5	0.3	1.5	0.4	1.2	0.6	1.9
PEX2	3.3	1.7	2.2	0.0	0.3	0.8	3.2	0.2	2.0
GRK5	4.0	1.2	1.7	0.4	0.5	0.8	2.6	0.3	2.3
LIPG	4.8	1.9	2.7	0.1	0.2	0.4	2.2	0.2	1.2
PIAS2	3.6	1.4	2.0	0.1	0.9	0.8	2.4	0.4	2.2
JRKL	4.1	1.3	1.7	0.2	0.7	1.1	2.4	0.3	2.1
UCP2	3.7	1.4	1.8	0.3	0.7	1.0	2.6	0.3	2.1
ADM5	4.4	1.5	1.8	0.3	0.5	0.7	2.2	0.6	1.8
LOC100287497	3.9	1.2	1.7	0.3	0.6	0.6	3.0	0.3	2.1
ZFYVE9	3.8	1.5	1.6	0.1	0.4	0.6	2.9	0.4	2.4
DSCAM-AS1	5.0	1.0	1.8	0.2	0.6	0.4	3.1	0.3	1.3
C16orf95	4.2	0.7	2.0	0.4	0.9	0.6	2.7	0.5	1.8
HOOK3	3.9	0.9	1.6	0.2	0.7	0.9	3.0	0.4	2.2
LOC285540	4.3	1.0	2.0	0.3	0.4	0.7	3.4	0.2	1.4
AHCYL2	4.1	1.4	1.8	0.2	0.5	0.8	2.9	0.4	1.7

SLC5A10	4.1	1.2	1.9	0.4	0.6	0.8	2.5	0.5	1.9
TMEM102	4.6	1.4	2.0	0.4	0.4	0.8	2.2	0.4	1.4
ZMYM6NB	3.6	0.9	2.3	0.3	0.5	0.7	3.5	0.3	1.6
DHRS4L1	3.5	1.4	1.8	0.3	0.8	1.2	2.9	0.2	1.7
FANCA	2.5	0.8	1.4	0.4	0.8	1.1	3.5	0.5	2.8
CCDC88A	3.2	1.1	1.4	0.1	0.5	0.7	3.4	0.5	2.8
C17orf58	3.9	1.8	1.6	0.4	0.8	1.0	2.2	0.4	1.7
CSRP2	3.4	1.5	1.7	0.0	0.5	0.5	3.7	0.7	1.7
NAGA	4.1	1.2	1.8	0.6	0.8	1.4	2.0	0.4	1.5
DIP2B	4.0	1.4	1.6	0.2	0.6	0.8	2.6	0.4	2.1
EPOR	3.4	1.5	1.9	0.6	1.0	1.0	2.6	0.3	1.5
COMMD8	4.2	1.8	1.9	0.1	0.3	0.5	2.9	0.3	1.6
SLC16A7	4.2	0.9	1.4	0.2	0.7	0.9	2.3	0.4	2.7
MOSPD1	4.1	1.4	2.0	0.1	0.5	0.4	3.4	0.1	1.6
MADD	4.4	1.5	2.0	0.2	0.6	0.7	2.0	0.3	1.9
CRNDE	2.8	1.1	1.9	0.4	0.9	0.8	3.2	0.4	2.2
DNAL1	4.3	1.0	1.3	0.1	0.8	0.8	3.0	0.3	2.1
ERCC8	3.4	1.3	2.0	0.1	0.5	0.6	2.8	0.3	2.6
UTP15	3.5	1.2	1.5	0.1	0.3	0.6	3.2	0.4	2.9
SPSB2	3.8	1.6	2.7	0.7	0.6	0.7	2.1	0.2	1.3
DIRC2	4.6	1.6	1.9	0.3	0.5	0.9	1.9	0.2	1.9
ZNF608	4.9	2.0	2.3	0.2	0.7	1.0	1.2	0.3	1.0
ITFG2	3.9	1.1	2.1	0.4	0.5	0.7	2.5	0.4	1.9
CENPA	3.5	1.7	1.8	0.4	0.9	1.8	2.0	0.2	1.3
A4GALT	4.2	1.4	2.3	0.3	0.9	1.0	1.8	0.5	1.4
SSH2	4.0	1.3	2.0	0.2	0.4	0.7	2.4	0.4	2.2
CYB5R4	3.8	1.3	2.6	0.1	0.2	0.4	2.7	0.3	2.3
ZFYVE16	3.6	1.3	1.6	0.1	0.6	0.7	3.2	0.3	2.3
ORC3	4.2	1.6	1.9	0.2	0.6	0.8	2.1	0.2	2.1
ZNF500	3.7	1.3	2.1	0.7	1.1	1.4	1.7	0.4	1.4
DUSP28	3.3	1.4	2.0	0.8	1.1	1.1	2.3	0.4	1.2
JMJD1C-AS1	3.8	1.1	2.4	0.5	0.7	0.7	2.5	0.4	1.6
PARD6G	4.4	1.4	2.5	0.6	0.4	0.7	1.9	0.4	1.5
ZNF438	3.8	1.5	2.0	0.3	0.6	1.0	2.2	0.3	1.9
AIM1	4.0	1.4	1.9	0.2	0.7	0.8	2.4	0.3	1.9
PLEKHF1	3.5	1.3	1.7	0.5	0.4	1.1	2.4	0.5	2.1
FAHD2A	3.6	1.5	2.0	0.5	1.1	1.4	1.7	0.3	1.4
PRMT3	2.9	1.2	1.8	0.2	0.7	1.0	3.4	0.4	2.1
CFL2	3.0	1.4	1.7	0.3	0.6	0.8	2.7	0.4	2.6
CCDC22	3.8	1.7	2.0	0.4	0.8	1.1	2.1	0.2	1.5
GSG2	2.5	1.2	1.4	0.4	0.9	1.1	3.0	0.6	2.5
OARD1	3.0	1.4	1.5	0.2	0.7	1.0	2.8	0.3	2.6
LOC101928861	4.6	0.8	1.8	0.4	0.7	0.7	2.9	0.2	1.5
COL18A1	4.6	1.6	2.3	0.6	1.0	1.2	1.3	0.2	0.8
INSIG2	4.1	1.5	2.6	0.0	0.3	0.6	2.5	0.2	1.9
TTF2	3.6	1.1	1.7	0.2	0.5	0.9	3.0	0.4	2.2
XRRA1	4.4	1.0	1.8	0.2	0.6	0.7	2.5	0.3	2.0
ANKRA2	4.0	1.5	2.5	0.2	0.4	0.8	2.3	0.3	1.5
GALK2	3.8	1.2	1.5	0.1	0.5	0.9	3.5	0.2	1.9
CDIP1	3.5	1.4	1.9	0.4	1.0	0.8	1.8	0.7	2.1
USP49	3.6	1.0	2.0	0.5	0.6	0.8	2.8	0.4	1.8
SNORD3B-2	4.0	1.8	1.1	0.8	0.7	0.6	1.6	0.9	1.9
SNORD3B-1	4.0	1.8	1.1	0.8	0.7	0.6	1.6	0.9	1.9
BRF2	3.5	1.4	1.8	0.2	0.5	0.9	3.0	0.4	2.0
HOMER1	2.7	1.2	1.5	0.2	0.6	0.8	3.1	0.6	2.9

PTOV1-AS1	4.2	1.3	1.8	0.2	0.4	0.6	2.8	0.3	1.9
KCTD18	3.8	1.7	2.4	0.4	0.7	0.9	1.9	0.2	1.4
RGL1	3.7	1.4	2.1	0.2	0.7	0.9	2.2	0.4	2.0
LCAT	3.3	1.6	2.8	0.4	0.7	1.0	1.7	0.4	1.6
LOC101928295	4.6	0.9	1.9	0.2	0.5	0.6	3.2	0.3	1.3
SGTB	3.8	1.2	1.2	0.1	0.3	0.9	3.2	0.3	2.5
MED23	3.7	1.5	2.1	0.1	0.4	0.6	2.8	0.3	2.0
ADA	3.4	1.5	2.2	0.4	0.8	1.2	2.0	0.3	1.7
DHODH	3.8	1.6	1.8	0.5	0.8	0.9	2.3	0.4	1.5
LOC100272216	5.3	0.7	2.3	0.3	0.6	0.6	2.4	0.2	1.0
KLHL7	3.5	1.1	2.0	0.3	0.6	0.7	3.0	0.2	2.1
SLC7A11-AS1	4.7	0.6	1.4	0.2	0.4	0.4	4.3	0.1	1.3
SLC38A6	4.2	1.2	2.0	0.3	0.7	0.9	2.7	0.2	1.3
UBE2W	4.3	1.2	2.0	0.1	0.5	0.7	2.4	0.2	2.0
LOC101928591	5.1	0.6	2.0	0.2	0.7	0.7	2.6	0.2	1.4
USP40	4.0	1.3	1.6	0.2	0.7	1.2	2.3	0.2	1.9
TMEM99	4.1	2.1	2.6	0.1	0.5	0.6	2.1	0.2	1.3
LINC00924	4.3	0.9	1.8	0.3	0.4	0.7	3.4	0.3	1.4
SLC9A6	4.2	1.8	2.0	0.2	0.5	0.5	2.1	0.4	1.7
PRKACB	4.2	1.9	1.9	0.1	0.6	0.5	1.8	0.2	2.2
ST3GAL6-AS1	3.8	0.9	1.7	0.6	0.6	1.0	2.4	0.4	1.9
FGD1	3.7	1.5	2.0	0.4	0.8	1.1	2.1	0.4	1.5
ZNF266	3.8	1.2	2.1	0.1	0.4	0.6	3.0	0.3	2.0
TANGO6	4.1	1.1	2.1	0.2	0.8	1.0	2.3	0.3	1.5
HOXB6	3.6	1.1	2.2	0.4	0.6	0.5	2.6	0.5	1.9
RAB31	4.2	2.0	2.6	0.1	0.6	0.4	1.7	0.3	1.5
INTS9	3.5	1.2	1.8	0.2	0.5	0.8	2.3	0.6	2.5
DECR1	4.4	1.7	2.0	0.2	0.7	1.0	2.2	0.1	1.2
RFX7	3.5	1.3	1.7	0.2	0.6	0.7	2.7	0.4	2.4
LOC283731	4.1	1.0	1.9	0.2	0.5	0.7	3.3	0.2	1.6
FAM98B	2.8	1.6	1.7	0.3	0.6	0.9	2.6	0.4	2.5
LOC101929068	3.6	1.9	2.1	0.6	0.5	0.9	1.9	0.5	1.2
TMPO-AS1	3.1	1.3	1.5	0.4	0.8	0.8	3.2	0.4	1.8
PHYH	5.1	1.6	2.8	0.3	0.9	1.2	0.6	0.2	0.8
RAPGEF6	3.7	1.1	1.7	0.1	0.5	0.6	3.0	0.4	2.1
ZNF778	3.3	1.0	1.8	0.2	0.6	0.7	3.1	0.5	2.3
C5orf22	3.9	1.5	1.9	0.1	0.5	0.6	2.5	0.3	2.0
ZNF160	3.8	1.0	2.2	0.2	0.5	0.8	2.6	0.3	2.0
GINS2	2.7	0.8	2.1	0.4	0.6	1.2	3.2	0.4	2.0
RAB11FIP2	4.1	1.5	1.9	0.2	0.7	0.8	1.9	0.2	2.1
LOC101927462	4.2	1.6	1.9	0.4	0.6	0.9	2.4	0.2	1.3
PHKB	4.6	1.5	2.3	0.2	0.5	0.9	1.8	0.3	1.3
PPFIA3	4.2	1.6	2.1	0.4	0.7	0.9	1.9	0.2	1.4
LOC101060179	3.8	1.0	1.8	0.3	0.5	0.8	3.1	0.2	2.0
B3GALTL	3.7	1.4	2.3	0.1	0.6	0.8	2.3	0.3	1.8
LOC729683	4.4	0.9	1.8	0.2	0.5	0.5	3.3	0.2	1.5
HDHD1	3.7	1.5	1.9	0.2	0.5	0.7	2.4	0.4	2.1
PAPPA2	3.1	1.1	1.3	0.4	1.0	1.2	2.8	0.3	2.2
ZSCAN9	3.7	1.5	2.3	0.2	0.6	0.8	1.8	0.4	2.0
LOC101928091	4.3	1.2	1.9	1.5	1.1	0.7	1.6	0.3	0.7
TTLL11	3.8	1.5	1.8	0.6	0.8	0.9	2.2	0.3	1.4
ARFGEF1	3.6	1.2	1.6	0.1	0.6	0.8	2.5	0.3	2.6
ZNF33A	3.9	2.0	1.3	0.1	0.5	0.7	2.0	0.2	2.5
CYB561D1	3.3	1.3	1.6	0.3	0.6	0.9	2.6	0.5	2.2
RUFY3	4.2	1.0	1.8	0.3	0.5	0.6	2.7	0.3	1.8

CBWD1	3.4	1.0	1.9	0.1	0.6	0.8	2.7	0.4	2.4
SLC35G2	3.3	1.2	1.9	0.0	0.3	0.7	3.1	0.2	2.6
TOP2A	2.6	1.2	1.5	0.2	1.3	1.5	2.9	0.3	1.8
ERGIC2	3.5	1.6	2.0	0.1	0.4	1.0	2.4	0.3	1.9
FAM73A	3.4	1.3	1.8	0.2	0.5	0.6	2.9	0.4	2.3
GMFG	3.4	1.5	2.7	0.3	1.0	2.0	1.5	0.2	0.7
ENPP1	4.6	1.6	2.1	0.1	0.4	0.6	2.2	0.3	1.5
RASA4	4.3	1.5	2.1	0.2	0.4	0.6	2.4	0.3	1.5
USP12	4.0	1.3	1.9	0.1	0.6	0.7	2.3	0.4	2.0
CCDC134	3.9	0.9	1.7	0.1	0.6	0.5	3.0	0.3	2.1
L2HGDH	3.4	1.2	1.8	0.2	0.5	0.9	3.3	0.2	1.8
AGAP6	3.9	1.0	1.8	0.2	0.8	0.7	2.7	0.3	1.8
GFM2	3.7	1.7	2.3	0.2	0.6	0.9	2.3	0.3	1.4
EVA1C	3.2	1.1	1.7	0.4	0.4	1.0	3.2	0.4	1.8
DGKQ	4.2	1.6	2.0	0.5	0.8	1.1	1.8	0.3	1.1
SLC25A5-AS1	3.1	1.4	2.3	0.3	0.7	1.0	1.8	0.6	2.0
LOC101927814	4.4	0.9	1.7	0.3	0.5	0.5	3.4	0.3	1.2
ATP5S	3.8	1.1	1.9	0.4	0.7	0.7	2.5	0.2	1.8
NFIA	3.9	1.1	1.7	0.4	0.8	1.1	1.7	0.4	2.3
LINC00339	4.1	1.4	1.9	0.2	0.6	1.0	2.3	0.3	1.3
LOC101927339	4.2	0.8	1.7	0.3	0.7	0.7	3.1	0.3	1.4
NIPSNAP3A	3.5	1.6	2.0	0.3	1.0	1.1	2.0	0.2	1.5
LOC100506178	4.4	2.3	3.1	0.2	0.3	0.4	1.1	0.2	1.1
PCBP3	3.2	1.1	1.3	0.6	1.2	1.6	2.2	0.4	1.6
L1CAM	2.6	0.9	1.2	0.3	0.6	0.8	3.6	0.6	2.7
LOC101929081	4.3	0.8	1.9	0.2	0.5	0.5	3.3	0.4	1.3
LINC00085	3.6	1.6	2.4	0.4	0.7	0.8	1.9	0.4	1.5
RUFY2	3.7	1.1	1.8	0.1	0.7	0.9	2.5	0.3	2.1
MTFMT	4.7	1.4	2.4	0.2	0.5	1.1	1.7	0.2	1.0
XPO4	3.8	1.2	1.6	0.2	0.5	0.6	2.9	0.3	2.0
PLCE1	4.5	1.6	2.0	0.2	0.6	0.9	1.7	0.3	1.4
GLTSCR1L	3.9	1.4	1.8	0.3	0.7	1.0	1.9	0.4	1.9
TRIM2	3.9	1.7	2.1	0.3	0.5	0.7	2.2	0.4	1.5
CCDC109B	3.0	1.3	1.4	0.1	0.7	1.0	2.6	0.6	2.5
FHOD3	3.9	1.4	1.7	0.5	1.0	1.4	1.8	0.3	1.3
PPIP5K2	3.4	1.3	1.8	0.1	0.6	0.8	3.0	0.2	2.1
ZMYM2	3.7	1.4	2.2	0.1	0.4	0.8	2.2	0.2	2.1
SBF2-AS1	4.2	0.9	2.3	0.2	0.5	0.6	2.6	0.2	1.6
RNASEH2B	3.6	1.6	2.1	0.2	0.8	0.8	1.9	0.3	1.8
CMTR2	3.0	1.2	1.4	0.1	0.2	0.4	3.5	0.4	2.8
FARP1	4.3	1.3	1.9	0.3	0.6	0.9	1.9	0.5	1.5
SLC36A1	3.6	1.3	1.6	0.4	0.7	0.8	2.5	0.3	1.8
LRCH1	3.6	1.3	1.9	0.2	0.6	0.6	2.6	0.3	2.2
BCL2	2.5	0.9	1.5	0.5	0.5	0.8	3.9	0.4	2.1
GPNMB	5.2	1.7	2.5	0.1	0.7	0.7	1.3	0.2	0.7
LENG9	3.5	1.5	1.7	0.4	0.8	1.0	2.0	0.3	1.9
EIF2AK3	3.3	1.5	1.5	0.2	0.3	0.5	2.9	0.4	2.5
GPATCH2	3.9	1.1	1.7	0.2	0.6	0.7	2.5	0.3	2.2
PDSS2	3.7	0.9	1.8	0.1	0.4	0.6	3.4	0.3	2.0
FAM49A	3.7	1.2	1.4	0.2	0.9	0.9	2.5	0.4	1.7
LOC101929631	4.1	1.0	1.8	0.4	0.5	0.6	3.0	0.3	1.5
LIMK2	3.7	1.4	1.6	0.3	0.7	0.6	3.1	0.3	1.4
ACER3	4.2	1.2	1.4	0.3	0.3	0.7	2.7	0.5	1.9
LINC00992	4.1	0.9	1.6	0.2	0.5	0.7	3.2	0.3	1.5
DECR2	3.5	1.8	2.5	0.4	0.7	1.3	1.5	0.3	1.0

OSBPL11	4.0	1.2	1.6	0.1	0.5	0.6	2.7	0.4	2.0
RNF170	3.8	1.0	2.1	0.1	0.6	0.8	2.4	0.2	1.9
ST20	4.1	1.6	2.4	0.1	0.3	0.5	2.6	0.4	1.1
MAFG-AS1	3.4	1.0	1.5	0.3	0.8	0.9	2.8	0.6	1.7
KIAA1109	4.1	1.1	1.8	0.1	0.5	0.8	2.5	0.2	1.9
AMDHD2	3.2	1.4	2.0	0.5	1.1	1.1	2.0	0.4	1.3
C1orf140	4.1	0.8	1.9	0.2	0.4	0.6	3.5	0.2	1.4
ERCC5	4.2	1.0	1.8	0.3	0.6	0.9	2.3	0.4	1.6
CNTNAP1	4.3	1.7	2.5	0.3	0.7	0.8	1.4	0.3	1.0
SERINC5	3.7	1.0	1.7	0.4	0.6	0.6	2.9	0.2	1.8
LOC148413	3.4	1.2	1.6	0.2	0.7	0.8	2.6	0.3	2.1
LINC00673	4.0	1.1	1.4	0.2	0.5	0.8	2.9	0.3	1.7
PXMP2	3.2	0.9	1.5	0.4	1.0	0.8	3.0	0.2	2.1
KIF1B	3.8	1.0	1.7	0.1	0.6	0.8	2.7	0.3	2.1
TBK1	3.3	1.4	1.6	0.1	0.4	0.5	2.6	0.4	2.7
SHROOM4	4.2	0.8	1.7	0.3	0.5	0.5	3.1	0.3	1.5
GCA	5.4	1.9	2.2	0.2	0.4	0.7	1.4	0.2	0.6
FBXL16	3.7	1.6	1.9	0.5	0.7	0.9	2.0	0.4	1.4
ZRANB2	2.9	1.0	1.9	0.3	0.7	1.1	2.5	0.4	2.4
FBXL2	3.9	1.3	1.9	0.2	0.5	0.6	2.7	0.2	1.6
LIN7B	4.6	2.0	1.9	0.3	0.9	0.8	1.4	0.2	0.9
GTF2IRD2B	4.6	1.7	2.3	0.2	0.5	0.7	1.2	0.2	1.6
PGAP2	3.1	1.5	2.2	0.4	0.8	1.5	1.7	0.3	1.5
PFKFB2	4.1	1.4	2.1	0.2	0.6	1.1	2.0	0.2	1.2
MAML3	3.5	1.2	1.3	0.4	0.7	1.0	2.0	0.5	2.3
LINC00167	4.2	0.8	2.0	0.2	0.4	0.6	3.3	0.2	1.3
DMKN	3.6	1.5	2.1	0.2	0.6	0.9	1.8	0.4	1.8
FAM74A2	3.8	0.7	2.2	0.2	0.6	0.5	3.5	0.3	1.1
UST	3.2	1.5	1.7	0.3	0.5	0.9	2.4	0.5	1.9
MAP3K5	5.1	1.5	2.3	0.2	0.5	0.7	1.6	0.2	1.0
LOC642852	3.7	1.3	2.0	0.3	0.6	0.9	2.1	0.3	1.7
CIITA	4.0	0.9	1.7	0.4	0.8	0.8	2.9	0.2	1.2
LIMD1-AS1	4.2	1.1	1.7	0.1	0.5	0.6	2.4	0.3	2.1
SMC2	3.1	1.3	1.6	0.1	0.5	0.6	3.1	0.3	2.4
LOC727978	3.1	0.7	1.7	0.4	0.9	0.7	3.4	0.3	1.7
TIRAP	3.7	1.3	1.7	0.5	1.0	1.2	1.9	0.3	1.3
LOC284581	4.7	1.3	2.0	0.1	0.4	0.6	1.9	0.3	1.6
DARS2	3.3	1.1	1.9	0.1	0.7	0.8	2.7	0.2	1.9
TASP1	3.5	1.2	1.8	0.1	0.5	0.4	2.8	0.4	2.1
LYN	3.5	1.5	2.0	0.1	0.4	0.6	2.5	0.4	1.8
KHDC1	4.1	0.9	1.9	0.1	0.2	0.6	3.1	0.4	1.5
IPMK	2.8	1.5	1.8	0.2	0.6	0.6	3.0	0.3	2.2
ITSN1	3.4	1.2	1.6	0.3	0.8	0.9	2.5	0.4	1.9
ZFAT	3.4	1.0	1.7	0.3	0.6	1.0	2.4	0.4	2.0
ZNF619	4.1	1.2	1.7	0.2	0.5	0.5	2.9	0.3	1.5
LOC100147773	3.8	0.9	1.8	0.3	0.5	0.7	3.2	0.3	1.4
LOC731275	4.3	1.0	2.1	0.3	0.7	0.9	1.9	0.3	1.4
LINC00630	3.8	1.2	2.1	0.2	0.6	0.8	2.3	0.4	1.5
GOLGA1	3.9	1.4	1.9	0.2	0.7	0.7	2.2	0.2	1.6
RNF215	3.4	1.3	2.0	0.5	0.8	1.3	1.9	0.3	1.3
TMC8	4.4	1.8	2.2	0.3	0.5	0.9	1.6	0.2	1.1
LINC00327	4.2	0.8	1.8	0.3	0.5	0.5	3.3	0.3	1.1
BAG2	3.0	0.8	1.7	0.1	0.4	0.9	2.9	0.4	2.6
ACADSB	3.4	1.2	1.7	0.1	0.6	0.7	3.0	0.3	1.7
ARHGEF39	3.9	0.9	1.9	0.2	0.5	0.6	3.0	0.3	1.5

WDR27	4.1	1.1	1.7	0.2	0.6	0.7	2.9	0.3	1.3
TSTD2	3.5	1.1	1.5	0.3	0.8	0.9	2.9	0.3	1.7
LOC388692	3.8	1.0	1.8	0.2	0.5	0.7	2.8	0.3	1.6
SLFN5	4.3	1.4	1.8	0.3	0.9	1.0	1.8	0.2	1.2
SH3RF3	3.9	1.0	1.9	0.1	0.7	0.8	2.1	0.1	2.1
OTUD3	3.3	1.2	1.6	0.2	0.5	0.3	3.0	0.4	2.4
RNF139	3.8	1.4	1.8	0.1	0.4	0.7	2.3	0.3	2.0
HKR1	3.5	1.5	2.0	0.2	0.6	1.1	1.9	0.3	1.7
KDM1B	3.0	1.1	1.7	0.3	0.6	0.9	2.9	0.5	1.9
LINC00704	2.7	1.1	1.3	0.4	1.1	1.2	2.8	0.3	2.0
ZC3H8	2.5	1.0	1.4	0.2	0.6	0.4	3.1	0.4	3.3
ANLN	2.6	1.1	1.5	0.2	0.9	1.2	2.8	0.5	2.1
NICN1	4.0	1.5	2.4	0.7	0.9	0.9	1.2	0.3	0.9
BTN3A1	3.1	1.3	1.7	0.1	0.8	0.7	3.0	0.4	1.9
MYO5C	3.2	1.1	1.3	0.2	0.7	0.9	2.8	0.3	2.2
THAP5	3.7	1.3	1.7	0.1	0.4	0.6	2.6	0.3	2.1
ARRDC4	5.8	2.4	3.0	0.1	0.2	0.2	0.6	0.1	0.4
PTBP2	4.2	1.6	2.0	0.1	0.4	0.7	2.1	0.2	1.5
MST4	3.7	1.2	1.8	0.1	0.3	0.5	2.5	0.2	2.4
EPDR1	4.4	1.3	1.7	0.3	0.6	0.9	2.2	0.2	1.2
DNAJC18	2.8	1.4	1.6	0.2	0.6	0.9	2.5	0.4	2.4
HAR1A	4.5	0.5	1.1	0.2	0.3	0.3	4.5	0.2	1.2
LOC101926895	5.2	0.8	1.6	0.3	0.3	0.5	2.9	0.2	1.1
LRP4-AS1	4.3	0.8	1.7	0.1	0.6	0.6	3.0	0.3	1.4
UPF2	3.4	1.2	1.6	0.1	0.5	0.7	2.5	0.4	2.4
BET1	3.1	0.8	1.7	0.1	0.5	0.6	3.7	0.1	2.2
FRAS1	3.7	1.0	1.5	0.3	0.7	0.7	2.7	0.3	1.8
PEX12	3.4	1.6	1.8	0.1	0.6	0.9	2.5	0.2	1.7
LOC101927859	3.6	1.0	1.7	0.7	0.8	1.2	2.3	0.4	1.2
C15orf37	3.9	1.3	2.1	0.4	0.5	0.7	2.4	0.3	1.3
YBEY	3.6	1.4	1.8	0.3	0.5	1.1	1.6	0.3	2.1
POLI	4.1	1.4	2.2	0.2	0.4	0.5	2.1	0.3	1.6
TRAPPC8	3.7	1.3	1.9	0.1	0.5	0.6	2.5	0.3	1.9
SLC25A16	4.0	1.0	1.6	0.3	0.6	0.6	2.8	0.3	1.6
CPPED1	3.7	1.5	1.8	0.4	0.8	0.7	2.0	0.3	1.6
LOC100289187	3.7	1.9	1.6	0.1	0.3	0.5	2.5	0.3	1.8
SH3BP1	4.1	1.6	1.7	0.5	0.9	1.2	1.3	0.3	1.2
TNFAIP8L1	3.9	1.0	1.8	0.6	0.8	1.1	2.2	0.3	1.1
TTLL3	4.2	1.2	2.2	0.3	0.7	0.9	1.8	0.2	1.3
TMEM53	3.7	1.6	1.9	0.5	0.6	1.8	1.5	0.3	0.8
ITPR1	3.9	1.2	1.6	0.2	0.4	0.6	2.6	0.3	1.9
BBS10	3.4	1.4	2.2	0.2	0.4	0.6	1.9	0.4	2.3
YEATS4	2.5	1.6	1.1	0.2	0.2	0.8	2.6	0.6	3.1
NRGN	1.1	0.8	0.9	0.2	0.4	0.7	4.6	0.8	3.1
THADA	3.7	1.3	1.8	0.1	0.4	0.7	2.5	0.2	1.8
BEND7	2.8	0.9	1.5	0.2	0.6	0.6	3.4	0.3	2.3
FAN1	3.3	1.3	1.8	0.2	0.6	0.7	2.6	0.2	2.0
SMAD6	3.6	1.5	2.0	0.6	0.9	0.9	1.7	0.3	1.1
NLN	3.3	1.1	1.6	0.2	0.5	1.0	2.6	0.3	2.0
BAMBI	4.9	1.3	2.2	0.2	0.3	0.5	1.5	0.3	1.4
PROB1	4.2	1.4	1.9	0.5	0.8	1.1	1.4	0.3	0.9
LOC101928780	4.0	1.0	1.8	0.3	0.7	0.8	2.5	0.3	1.3
TCTN2	4.4	1.5	2.4	0.2	0.6	0.7	1.6	0.2	1.0
NOXA1	4.0	1.6	2.7	0.5	1.2	1.0	0.9	0.2	0.6
CCDC174	3.3	1.0	1.7	0.2	0.5	0.6	2.9	0.2	2.1

CHCHD7		3.1	1.3	1.3	0.2	0.7	1.1	2.9	0.4	1.8
RPAP3		3.4	1.2	1.8	0.1	0.6	0.7	2.3	0.4	2.2
HOXD4		3.3	1.3	1.5	0.3	0.6	0.7	2.6	0.8	1.7
ICAM3		2.9	1.2	1.6	0.5	0.8	1.0	2.3	0.4	2.0
CSPP1		3.0	1.2	1.6	0.2	0.5	0.8	2.6	0.4	2.5
	44810.0	3.5	1.3	2.0	0.3	0.7	1.0	2.1	0.4	1.4
CACNB3		3.7	1.7	2.0	0.4	0.5	0.9	1.5	0.3	1.7
ANKRD36C		3.1	0.7	1.5	0.1	0.5	0.5	3.6	0.3	2.4
TMEM194A		3.3	1.0	1.3	0.2	0.6	0.9	3.0	0.5	1.9
TP73-AS1		4.2	0.7	1.6	0.2	0.4	0.7	3.2	0.2	1.3
MKI67		2.6	1.0	1.2	0.4	1.1	1.3	2.2	0.5	2.2
HES4		3.8	1.4	2.2	0.6	0.8	1.4	1.3	0.4	0.8
SPANXC		5.9	0.7	1.6	0.1	0.8	0.8	1.0	0.4	1.4
SFT2D3		4.2	1.4	1.9	0.8	0.8	0.7	1.7	0.4	0.6
ZMYM6		4.0	1.7	2.4	0.1	0.3	0.5	1.9	0.3	1.4
L3MBTL4		4.3	0.8	1.7	0.2	0.4	0.5	3.0	0.2	1.3
USP46		2.8	1.3	1.4	0.2	0.6	0.8	3.0	0.3	2.0
FLJ27243		4.1	0.7	1.8	0.2	0.6	0.6	3.1	0.2	1.2
CARD16		2.8	1.8	2.3	0.2	1.0	1.0	1.4	0.2	1.9
FAM76A		3.9	1.0	2.0	0.1	0.4	0.6	3.0	0.2	1.3
JMJD4		2.3	0.9	1.4	0.2	0.9	1.1	3.3	0.3	2.1
SH3TC2		3.5	1.4	1.8	0.3	0.8	0.8	2.2	0.3	1.5
NBEAL2		3.8	1.7	2.4	0.3	0.5	0.8	1.5	0.3	1.2
ASIC3		4.0	1.5	1.9	0.5	0.7	1.4	1.0	0.4	1.3
GPATCH11		3.4	1.4	1.4	0.1	0.3	0.8	2.1	0.3	2.6
KCTD11		3.8	1.6	1.7	0.6	0.7	0.8	1.4	0.3	1.7
KRT81		2.6	1.3	1.2	0.3	0.7	0.9	2.9	0.6	2.0
PCBD2		3.5	1.3	1.6	0.3	0.7	1.2	1.4	0.4	2.1
INTS12		2.9	1.4	1.7	0.1	0.7	1.0	1.9	0.4	2.3
CD209		4.2	0.8	1.8	0.2	0.4	0.4	3.2	0.3	1.1
SLC35B4		4.4	1.6	2.0	0.3	0.6	1.0	1.3	0.3	1.0
GPATCH1		3.5	1.2	1.6	0.1	0.5	0.7	2.7	0.3	1.9
ARHGAP42		4.3	1.4	2.0	0.1	0.3	0.5	1.9	0.3	1.7
CA13		2.9	1.3	1.8	0.1	0.8	0.4	1.8	0.7	2.8
FAM120C		2.8	1.9	1.7	0.4	0.5	1.0	2.8	0.4	1.0
BBIP1		3.5	1.2	1.9	0.1	1.2	0.8	1.4	0.4	1.9
NUSAP1		2.6	1.5	1.8	0.3	0.9	1.0	2.0	0.4	1.9
SLC22A25		4.1	0.8	1.9	0.3	0.5	0.7	2.8	0.2	1.1
ERVK13-1		3.9	1.0	1.9	0.3	0.6	0.7	2.5	0.2	1.5
ANKRD16		2.8	1.2	1.7	0.5	0.9	1.4	2.1	0.3	1.6
GPR15		3.4	0.4	1.6	0.7	1.1	1.6	2.3	0.1	1.3
LOC100506963		3.0	0.9	1.4	0.2	0.7	0.4	3.2	0.5	2.2
LOC101928446		4.2	0.7	1.7	0.3	0.4	0.6	3.0	0.2	1.3
SUSD1		3.8	1.3	1.8	0.1	0.4	0.6	2.0	0.4	2.1
LOC101928131		4.5	0.9	1.6	0.3	0.8	0.7	1.9	0.2	1.5
XPR1		3.2	1.1	1.7	0.2	0.7	1.0	2.4	0.3	2.0
ZNF800		3.5	1.2	1.7	0.1	0.2	0.7	2.8	0.3	2.0
RAD18		2.5	0.9	1.4	0.1	0.5	0.6	3.2	0.3	2.9
GSKIP		3.6	1.1	1.6	0.0	0.7	0.2	3.3	0.4	1.4
ARSB		3.4	1.5	1.5	0.4	0.7	1.0	2.1	0.3	1.6
RAB30		3.0	1.1	2.1	0.3	0.6	0.7	2.5	0.3	1.9
DDX60L		3.5	1.1	1.5	0.1	0.6	0.6	3.0	0.3	1.7
STARD13		2.4	0.8	1.4	0.3	0.7	0.8	3.2	0.5	2.4
SH3RF2		3.5	1.4	2.0	0.5	0.8	1.3	1.5	0.4	1.0
SLFN12		2.9	1.0	1.2	0.1	0.4	0.4	3.8	0.4	2.2

LINC00934	3.9	0.8	1.4	0.2	0.4	0.6	3.2	0.3	1.5
LOC101929672	3.6	1.2	2.0	0.7	0.7	1.3	1.7	0.0	1.2
SEMA3F	3.6	1.8	1.4	0.4	0.7	1.2	1.6	0.3	1.4
BCKDHB	2.9	1.0	1.4	0.5	0.6	0.9	2.6	0.4	2.1
LOC283585	3.8	0.7	1.9	0.5	0.8	0.7	2.6	0.3	1.1
SEL1L3	4.1	1.7	2.2	0.2	0.5	0.5	1.6	0.2	1.5
B3GNT3	3.7	1.7	1.9	0.4	0.6	0.9	1.6	0.4	1.2
LOC101928771	2.9	1.2	2.1	0.1	0.9	1.2	1.8	0.2	1.8
B3GNT2	3.4	1.3	1.9	0.1	0.7	0.6	2.4	0.2	1.8
AIF1L	2.5	1.1	1.3	0.1	0.3	0.6	3.1	0.6	2.8
ZNF284	4.2	1.4	2.1	0.1	0.4	0.4	1.8	0.3	1.6
EXOC1	3.5	1.7	1.9	0.1	0.5	0.6	2.1	0.3	1.6
LRRC28	3.1	1.0	1.2	0.2	0.8	0.8	3.1	0.1	1.9
LINC00620	3.9	0.7	2.0	0.2	0.6	0.5	2.8	0.2	1.4
SNAI1	4.0	1.1	1.9	0.1	0.3	0.3	1.8	0.8	1.9
UBXN11	3.9	1.2	1.4	0.6	0.8	1.6	1.4	0.2	1.2
FRS2	3.0	1.3	1.7	0.1	0.5	0.7	2.4	0.3	2.5
LOC101928434	3.9	0.7	1.7	0.4	0.6	0.6	3.1	0.3	1.1
UBE2F	3.0	1.7	1.7	0.1	0.5	0.6	2.2	0.3	2.2
ZBTB39	2.9	1.0	1.5	0.2	0.6	0.9	2.3	0.5	2.5
C3orf14	3.8	1.7	1.5	0.1	0.1	0.7	1.7	0.3	2.4
FLJ42393	4.5	0.4	1.1	0.1	0.3	0.3	4.5	0.1	1.0
ANK2	2.7	1.0	0.9	0.2	0.5	0.7	3.3	0.4	2.6
LOC101926980	4.4	0.6	1.9	0.2	0.5	0.6	2.2	0.2	1.7
PAAF1	3.1	1.3	2.1	0.3	0.6	0.8	2.3	0.4	1.4
CLCF1	2.9	1.3	1.9	0.6	1.6	1.0	1.5	0.3	1.2
FNIP2	3.4	1.4	1.7	0.2	0.7	0.6	2.3	0.4	1.7
PIK3R4	3.4	1.0	1.5	0.1	0.5	0.6	2.8	0.2	2.2
UPF3B	2.9	1.0	1.4	0.2	0.5	0.6	2.9	0.3	2.5
LOC101929451	3.4	0.9	1.3	0.5	1.2	1.1	2.0	0.4	1.5
PMS2	3.9	1.3	1.8	0.3	0.6	0.8	2.0	0.2	1.4
SNAPC3	3.3	1.2	1.6	0.2	0.5	0.6	2.5	0.3	1.9
TSSK3	3.7	1.4	2.2	0.3	0.7	0.5	1.6	0.2	1.7
SLC30A1	2.9	1.3	1.6	0.2	0.9	0.6	2.7	0.3	1.8
HSCB	2.4	1.6	1.4	0.6	0.6	1.1	2.0	0.8	1.8
ABCC3	3.3	0.9	1.7	0.9	1.4	2.5	0.8	0.2	0.6
HDHD2	3.4	1.1	1.6	0.2	0.7	1.2	2.4	0.3	1.6
ZNF12	2.8	0.9	1.3	0.1	0.6	0.8	2.9	0.3	2.5
TMEM186	3.3	0.8	1.9	0.4	0.7	0.6	3.1	0.2	1.3
SDCCAG8	3.3	1.4	1.5	0.2	0.8	0.7	2.1	0.3	1.8
ZNF862	4.1	1.3	1.9	0.3	0.6	0.7	1.8	0.2	1.2
LOC100505622	3.7	1.3	1.5	0.3	0.4	0.5	2.7	0.3	1.6
ACOX3	3.6	1.2	2.0	0.3	0.6	0.7	2.3	0.4	1.3
LOC101927671	4.7	0.7	1.4	0.2	0.6	0.6	2.5	0.4	1.1
STK38L	3.7	1.4	1.8	0.1	0.4	0.6	2.2	0.3	1.8
OPLAH	3.0	1.2	1.7	0.6	0.9	1.3	1.8	0.3	1.4
SYCE2	3.2	1.3	1.2	0.4	0.8	0.9	2.1	0.2	2.1
FAM174B	3.4	0.9	1.6	0.8	0.5	0.9	2.2	0.4	1.6
LOC101928986	3.9	0.7	1.8	0.2	0.5	0.4	3.1	0.2	1.5
ANGPTL4	3.3	1.3	1.8	0.5	0.9	0.9	2.0	0.3	1.1
FAM21B	1.0	3.6	1.6	0.4	1.0	1.7	0.6	1.0	1.4
TRIP13	2.5	1.0	0.9	0.2	1.0	1.0	2.9	0.4	2.3
EXTL2	3.2	1.4	1.7	0.1	0.4	0.5	2.3	0.3	2.1
POMZP3	3.4	1.4	2.2	0.2	0.6	0.9	2.1	0.3	1.2
SACS	3.1	0.8	1.1	0.1	0.5	0.6	3.2	0.3	2.4

P2RX7	4.1	0.8	1.7	0.2	0.4	0.5	3.0	0.2	1.2
FOXN2	3.5	1.5	1.5	0.1	0.4	0.5	2.2	0.3	2.0
METTL14	3.1	1.1	1.6	0.1	0.6	0.5	2.8	0.3	2.1
PLD1	5.1	1.8	2.4	0.1	0.3	0.4	0.9	0.1	1.0
RP2	3.2	1.0	1.5	0.2	0.6	0.4	2.6	0.4	2.2
PTRH1	3.3	1.3	1.5	0.6	1.0	1.2	1.9	0.3	1.1
LNX2	3.3	1.0	1.5	0.2	0.6	0.7	2.2	0.5	2.2
TMEM161B	3.6	0.9	1.3	0.0	0.5	0.5	2.9	0.2	2.2
C2orf42	3.9	1.2	1.4	0.2	0.5	0.9	2.2	0.2	1.5
SCARA3	3.7	2.0	1.8	0.1	0.3	0.6	1.8	0.2	1.5
LOC100129636	3.8	0.7	1.2	0.1	0.2	0.8	3.4	0.2	1.9
LRRC57	3.6	1.1	1.4	0.2	0.4	0.7	2.4	0.3	2.1
C2orf43	2.7	1.1	1.8	0.1	0.6	0.9	2.5	0.2	2.3
SLC25A10	2.8	1.1	1.3	0.8	1.0	1.8	1.6	0.3	1.3
INPP5F	4.0	1.3	1.9	0.1	0.6	0.5	1.9	0.2	1.7
LATS1	3.2	1.2	1.5	0.1	0.3	0.5	2.6	0.3	2.3
PELI3	3.5	1.3	1.8	0.6	0.9	1.5	1.3	0.3	0.9
LMBRD1	4.5	1.5	1.8	0.1	0.3	0.5	1.6	0.4	1.3
COL27A1	3.3	1.3	1.8	0.3	0.6	0.7	1.9	0.4	1.8
HCG4B	3.5	1.3	1.7	0.5	0.9	0.9	1.7	0.4	1.0
IGSF9B	2.8	0.9	1.3	0.4	0.9	1.2	2.2	0.5	1.8
TRNT1	3.3	0.7	1.3	0.1	0.5	0.8	3.4	0.4	1.8
EPHX4	3.4	1.6	1.8	0.3	0.4	0.4	2.2	0.5	1.4
HAUS2	2.3	1.1	1.8	0.1	0.3	0.9	3.4	0.5	1.7
TTC9	3.2	1.2	1.7	0.1	0.3	0.5	2.2	0.6	2.3
LOC100996873	4.0	0.8	1.7	0.2	0.4	0.5	3.0	0.2	1.3
BLOC1S1-RDH5	3.2	1.2	2.1	0.2	0.7	1.1	1.7	0.2	1.6
ZNF786	2.6	1.2	1.4	0.3	0.5	0.8	2.8	0.4	2.1
LOC339535	2.2	0.8	1.3	0.2	0.7	1.4	2.3	0.3	2.7
C14orf159	3.2	1.1	1.9	0.3	0.6	0.8	2.0	0.4	1.7
UBR1	3.5	1.2	1.6	0.1	0.4	0.6	2.5	0.3	1.8
LOC101928670	4.6	0.7	2.0	0.1	0.4	0.7	2.5	0.2	0.8
RASA4B	3.9	1.3	1.8	0.2	0.4	0.5	2.1	0.3	1.4
SLC17A5	3.6	1.1	1.6	0.1	0.7	0.7	2.1	0.3	1.8
FIG4	3.4	1.3	1.9	0.2	0.5	0.7	2.1	0.3	1.8
CENPBD1	3.2	0.7	1.5	0.1	0.7	0.3	2.5	0.5	2.4
PIGV	3.7	1.4	1.9	0.3	0.7	0.8	1.8	0.2	1.3
LOC101928068	4.0	0.8	1.7	0.3	0.5	0.6	2.9	0.2	1.1
RABGAP1L	3.1	1.1	1.4	0.2	0.4	0.5	3.3	0.2	1.9
FAM86A	3.0	1.1	1.4	0.5	0.9	1.1	2.5	0.2	1.4
KIAA0586	3.1	1.1	1.2	0.2	0.6	1.0	2.4	0.4	2.2
LOC101928562	3.8	0.8	1.6	0.2	0.4	0.5	3.1	0.3	1.3
C15orf57	2.9	1.3	1.7	0.4	0.4	0.6	2.8	0.2	1.8
PLCB1	4.1	1.5	1.9	0.2	0.7	0.7	1.5	0.2	1.2
GPRIN1	2.8	1.2	1.4	0.3	0.4	0.7	3.0	0.5	1.7
CCSAP	3.5	1.0	1.4	0.2	0.7	0.7	2.5	0.2	1.7
ARID2	3.0	1.0	1.3	0.1	0.5	0.8	2.2	0.5	2.5
LGALS8-AS1	3.6	1.2	1.5	0.5	1.0	1.5	1.1	0.3	1.3
ABCB9	3.2	1.0	1.5	0.4	0.7	1.2	2.0	0.4	1.6
DNAJC2	2.9	1.3	1.2	0.0	0.3	0.6	2.9	0.5	2.2
CHAC2	3.0	1.0	1.3	0.1	0.3	0.4	2.6	0.5	2.9
HIC1	3.5	1.3	2.1	0.3	0.8	0.6	1.6	0.3	1.4
ZBTB25	3.2	0.9	1.8	0.3	0.7	1.2	1.7	0.4	1.8
SLC30A6	3.4	1.4	1.5	0.1	0.4	0.5	2.4	0.2	2.0
LOC101929280	4.0	0.8	1.5	0.2	0.4	0.5	3.0	0.3	1.2

LOC100505761	4.1	0.8	1.8	0.3	0.7	0.6	2.1	0.3	1.3
ZNF326	3.3	1.0	1.4	0.2	0.8	0.7	2.1	0.3	2.0
DHRS1	3.7	1.3	1.8	0.4	0.8	1.0	1.4	0.3	1.2
SULT1C2	4.8	1.3	1.9	0.2	0.6	0.8	1.4	0.1	0.8
SPAST	2.7	1.2	1.7	0.2	0.5	0.5	2.8	0.2	2.1
TRAPPC11	3.9	1.5	1.8	0.2	0.6	0.9	1.7	0.2	1.2
FAM173B	3.7	0.9	1.5	0.2	0.9	0.7	2.2	0.2	1.5
RGMB-AS1	2.2	0.7	1.1	0.2	0.5	0.8	3.2	0.4	2.7
ZNF625	3.9	0.9	1.9	0.2	0.5	0.6	2.5	0.1	1.4
IL4I1	3.0	1.2	1.4	0.5	0.7	0.9	2.0	0.4	1.9
CHD7	3.4	1.0	1.5	0.2	0.4	0.5	2.5	0.4	2.1
LOC101928669	3.8	0.7	1.7	0.1	0.6	0.7	2.5	0.2	1.5
CCDC142	2.7	1.1	1.6	0.4	0.7	0.9	2.6	0.3	1.5
KIFAP3	3.4	1.5	1.4	0.1	0.6	0.6	2.1	0.3	2.0
GALNT9	3.6	1.5	1.6	0.3	0.7	0.8	1.6	0.4	1.4
MTF1	3.1	1.2	1.5	0.3	0.4	0.9	2.1	0.3	2.2
ZSCAN2	4.2	0.8	2.3	0.2	0.6	0.8	1.2	0.4	1.3
FAM8A1	3.5	1.6	1.7	0.5	0.5	0.9	1.6	0.5	1.0
SLC22A5	4.3	1.6	1.8	0.3	0.5	0.5	1.5	0.2	1.1
LOC340515	4.2	0.9	1.8	0.3	0.6	0.6	1.9	0.3	1.2
CCL28	4.2	0.7	1.8	0.3	0.5	0.4	2.7	0.1	1.2
PLEKHG5	3.5	1.7	2.2	0.3	0.6	0.8	1.2	0.2	1.3
LOC100506974	3.8	0.8	1.7	0.1	0.3	0.6	3.0	0.2	1.4
LOC101928209	3.6	1.2	1.5	0.2	0.4	0.7	2.1	0.4	1.6
IPO11-LRRC70	3.6	1.4	1.3	0.1	0.4	0.7	2.5	0.3	1.6
LOC101926916	3.7	0.9	1.6	0.2	0.5	0.5	2.6	0.3	1.5
LINC00263	3.4	0.9	1.6	0.2	0.7	0.7	2.1	0.3	1.9
LOC101927746	2.1	1.2	1.3	0.2	0.6	0.8	2.5	0.6	2.6
PYROXD2	3.7	1.7	2.4	0.4	0.9	1.0	0.9	0.1	0.9
LOC284191	3.9	0.8	1.7	0.1	0.3	0.6	2.8	0.3	1.3
TPCN2	3.3	1.1	1.9	0.3	0.8	0.8	2.2	0.3	1.2
DNAJB4	3.0	1.2	1.8	0.1	0.5	0.4	2.6	0.3	2.0
MFSD6	4.5	1.6	1.9	0.2	0.4	0.6	1.4	0.2	1.1
LINC00847	3.0	1.3	1.6	0.2	0.4	0.9	2.6	0.4	1.4
ADAMTSL1	2.3	0.9	1.2	0.5	0.9	1.1	2.7	0.3	1.7
GABBR1	3.7	1.3	1.7	0.5	0.9	1.0	1.5	0.2	1.1
HAUS8	2.5	0.9	1.2	0.2	0.7	1.0	2.5	0.4	2.4
ENTPD7	2.5	0.7	1.2	0.1	0.4	0.5	3.3	0.4	2.7
MYCT1	1.7	1.1	1.1	0.1	0.6	1.0	2.5	0.5	3.3
PIM2	4.0	1.5	1.6	0.2	0.4	0.6	1.6	0.2	1.6
LOC283575	3.7	0.8	1.6	0.2	0.5	0.6	2.8	0.3	1.3
SNORD67	1.4	2.2	1.5	1.1	0.3	0.9	2.1	1.2	1.0
FAM53A	3.0	0.9	1.3	0.4	0.8	1.2	2.0	0.3	1.7
PP2672	3.9	0.7	1.6	0.2	0.3	0.5	2.9	0.3	1.4
LOC100507507	2.9	1.4	1.8	0.4	0.4	0.8	2.8	0.3	1.1
SNORA66	2.8	2.0	1.2	0.8	0.3	0.6	2.3	0.2	1.5
COL5A1	3.5	1.2	1.8	0.4	1.0	1.2	1.5	0.3	0.8
BATF3	2.5	0.9	0.8	0.2	0.9	0.9	2.8	0.2	2.5
NEGR1	1.9	1.0	0.9	0.2	0.6	1.1	3.3	0.4	2.5
UBOX5	3.1	1.1	1.2	0.2	0.7	0.9	2.2	0.3	2.0
RARS2	2.8	1.3	2.0	0.1	0.5	0.5	2.3	0.2	2.0
ZNF780A	3.2	0.9	2.4	0.1	0.5	0.4	2.5	0.2	1.5
RBM12B-AS1	3.2	1.1	1.7	0.2	0.4	0.5	2.6	0.4	1.5
STMN3	3.6	1.5	2.1	0.4	0.7	0.8	1.4	0.3	1.0
PP7080	3.1	0.9	1.5	0.3	0.7	0.6	2.4	0.4	1.7

KIF18B	2.6	1.1	1.3	0.6	0.8	1.2	2.0	0.5	1.7
C6orf147	3.4	1.0	1.5	0.1	0.6	0.5	2.6	0.2	1.7
ZNF227	3.3	1.0	1.5	0.1	0.3	0.6	2.5	0.4	2.0
HERC1	3.6	0.9	1.6	0.2	0.7	0.7	2.2	0.2	1.5
BIVM	3.5	0.7	1.7	0.1	0.7	1.0	2.2	0.2	1.6
CWC22	3.2	1.1	1.5	0.1	0.4	0.6	2.2	0.3	2.3
ACPL2	3.1	1.6	1.4	0.2	0.4	0.8	2.0	0.4	1.8
TRPM7	3.3	1.1	1.7	0.1	0.3	0.7	2.4	0.3	1.8
FAM69B	2.8	1.3	1.3	0.6	0.8	0.8	2.0	0.5	1.6
ATP11C	3.4	1.3	1.6	0.1	0.4	0.5	2.2	0.3	1.9
PRRG4	4.0	1.3	2.0	0.1	0.4	0.3	1.9	0.4	1.4
C4orf19	3.1	1.4	1.6	0.3	0.6	0.9	1.9	0.2	1.6
SPRTN	3.1	0.9	1.3	0.3	0.5	0.4	2.7	0.3	2.3
ENGASE	3.3	1.8	2.0	0.3	0.6	0.9	1.3	0.2	1.2
FANCI	2.5	0.8	1.2	0.1	0.6	0.7	3.1	0.3	2.4
NCAPH	2.5	1.2	1.3	0.3	0.6	1.1	2.6	0.3	1.9
S1PR2	3.4	1.1	1.8	0.5	0.7	1.0	1.3	0.3	1.4
CLN5	3.0	1.7	1.6	0.1	0.4	0.9	2.3	0.3	1.2
RALGAPA1	3.6	1.1	1.7	0.1	0.4	0.5	2.3	0.2	1.8
HACL1	2.7	1.0	1.6	0.1	0.4	0.8	3.0	0.2	1.8
CTSH	3.7	2.0	2.4	0.2	0.6	0.7	1.1	0.1	0.8
OTX2-AS1	3.9	0.7	1.7	0.2	0.4	0.5	2.8	0.3	1.1
NKRF	2.8	0.9	1.7	0.1	0.3	0.7	2.8	0.3	2.0
NFXL1	3.3	1.0	1.5	0.2	0.5	0.5	2.5	0.4	1.6
LOC100505676	3.7	0.7	1.5	0.2	0.6	0.6	2.9	0.2	1.3
LINC00598	3.7	0.8	1.5	0.2	0.4	0.5	2.7	0.2	1.4
NCAPG2	2.3	0.6	1.2	0.1	0.7	0.7	3.3	0.5	2.4
CEP85	2.4	1.2	1.5	0.1	0.4	0.7	2.5	0.4	2.3
ASMTL	3.4	1.2	1.7	0.4	0.7	1.0	1.6	0.3	1.4
ASMTL	3.4	1.2	1.7	0.4	0.7	1.0	1.6	0.3	1.4
ZCCHC2	3.3	1.2	1.5	0.3	0.5	0.7	2.1	0.4	1.7
LOC653375	3.6	3.0	1.1	0.3	0.9	0.8	0.7	0.2	1.1
LOC101927820	3.4	0.7	1.8	0.1	0.6	0.4	3.1	0.1	1.4
THEM4	2.8	1.5	1.5	0.2	0.5	0.8	2.1	0.5	1.8
AGL	3.7	1.1	1.7	0.1	0.4	0.6	2.0	0.2	1.8
KAT2B	3.9	1.6	1.7	0.2	0.6	1.0	1.2	0.4	1.0
TEX30	2.2	1.1	2.5	0.1	0.3	0.5	3.0	0.2	1.7
BLZF1	3.1	1.0	1.8	0.1	0.7	0.8	2.1	0.3	1.8
MICAL1	3.0	1.4	1.8	0.3	0.6	0.8	2.0	0.3	1.4
THAP8	3.4	1.2	2.1	0.3	0.8	1.0	1.2	0.1	1.3
LOC101928940	4.0	0.6	1.5	0.3	0.5	0.5	2.8	0.2	1.2
TRPM4	3.5	1.2	1.9	0.5	1.0	1.0	1.4	0.2	1.0
KIAA1549	3.3	1.0	1.5	0.3	0.6	0.8	2.1	0.4	1.6
CPVL	3.9	1.1	2.0	0.2	0.5	0.8	1.9	0.2	1.0
LOC101927678	3.8	0.8	1.6	0.2	0.4	0.4	2.8	0.3	1.3
RAB27A	3.8	1.3	1.7	0.0	0.3	0.6	2.4	0.3	1.2
PPP2R5A	3.6	1.0	1.8	0.2	0.5	0.7	2.1	0.3	1.3
PRTG	3.6	1.2	1.5	0.2	0.4	0.5	2.1	0.4	1.7
SNORD16	3.0	0.7	0.8	0.6	1.8	0.6	3.1	0.3	0.7
LRRC27	3.4	1.3	1.6	0.2	0.7	1.0	1.6	0.4	1.2
UPRT	3.2	1.3	1.5	0.3	0.6	0.9	2.1	0.3	1.2
EFNA3	2.7	1.3	1.7	0.4	0.6	0.7	2.1	0.3	1.7
CNPY4	4.1	1.2	1.5	0.4	0.7	0.9	1.3	0.3	1.1
PCDHGA10	4.3	1.5	1.8	0.2	0.7	0.8	1.3	0.2	0.7
KLHL1	3.5	0.7	1.6	0.4	0.6	0.6	2.9	0.2	1.1

LOC642846	2.6	1.1	1.7	0.3	0.6	0.8	2.2	0.4	1.7
DISP2	3.2	1.3	1.7	0.4	0.8	1.0	1.6	0.4	1.1
LOC101927104	2.0	1.0	1.6	0.3	0.1	0.6	3.4	0.3	2.2
PHIP	3.3	1.0	1.5	0.1	0.5	0.6	2.6	0.2	1.7
KIAA1715	2.7	1.2	1.9	0.1	0.5	0.8	2.3	0.3	1.6
RPS6KL1	2.6	0.8	1.4	0.3	0.5	0.8	2.6	0.6	2.0
FAM92A1	3.6	1.2	2.0	0.1	0.3	0.7	1.8	0.3	1.4
HMGXB4	3.0	1.0	1.6	0.1	0.4	0.7	2.2	0.3	2.1
TFCP2	3.0	1.2	1.4	0.2	0.4	0.7	2.3	0.3	1.9
HIBCH	2.8	1.0	1.4	0.1	0.5	0.5	2.9	0.4	1.9
RPS6KC1	3.6	1.1	1.9	0.1	0.5	0.6	1.9	0.3	1.4
DIAPH2	3.4	1.1	1.6	0.1	0.4	0.6	2.0	0.3	1.8
FAM76B	3.5	1.2	1.8	0.1	0.3	0.6	1.7	0.3	2.0
BDH2	4.4	1.4	1.8	0.2	0.4	0.6	1.3	0.2	1.1
LINC00887	3.9	0.9	1.5	0.3	0.4	0.6	2.5	0.3	1.1
DGKH	4.0	0.8	1.8	0.1	0.5	0.5	2.0	0.2	1.4
FLJ41941	3.8	0.7	1.5	0.2	0.5	0.5	2.8	0.2	1.3
GPAT2	3.3	1.3	1.5	0.3	0.8	0.9	1.7	0.2	1.3
ZBTB34	2.7	1.0	1.6	0.2	0.4	0.6	2.3	0.3	2.3
CABLES2	3.1	1.2	1.4	0.4	0.9	1.1	1.5	0.4	1.5
ZBTB24	3.3	0.9	1.5	0.1	0.4	0.6	2.2	0.4	2.0
FLJ41423	3.9	0.8	1.5	0.2	0.3	0.6	2.7	0.2	1.3
PLK1S1	3.0	1.1	1.4	0.2	0.4	0.6	2.2	0.4	2.2
DOPEY2	3.5	1.2	1.6	0.4	0.7	0.9	1.7	0.2	1.2
SIRT5	3.2	0.9	1.9	0.2	0.4	0.7	2.5	0.2	1.3
TGFBI	3.5	1.5	2.0	0.2	0.8	0.7	1.4	0.3	1.1
TRMT44	2.9	1.1	1.6	0.2	0.5	0.5	2.4	0.3	1.7
DOK4	4.0	1.2	1.8	0.1	0.4	0.8	1.9	0.3	0.9
SLC22A18	3.3	1.8	1.9	0.5	1.3	1.0	0.9	0.2	0.6
MESP1	2.8	1.4	1.9	0.1	0.6	0.7	2.2	0.2	1.4
LOC440028	3.6	0.8	1.8	0.2	0.5	0.5	2.6	0.2	1.1
AHNAK2	2.9	0.9	1.3	0.3	0.5	0.7	2.6	0.3	1.8
LOC101928733	4.1	0.6	1.5	0.2	0.4	0.5	3.0	0.1	1.0
TMEM50B	3.5	1.1	1.6	0.1	0.3	0.6	2.6	0.2	1.4
SNAPC5	2.3	1.4	1.9	0.3	1.1	1.0	1.5	0.5	1.4
UFSP2	3.0	1.2	1.3	0.1	0.5	0.7	2.2	0.3	2.0
HOXD-AS1	2.9	1.0	1.4	0.2	0.4	0.7	2.3	0.5	2.0
HNRNPU-AS1	3.6	1.0	1.7	0.1	0.4	0.5	2.4	0.2	1.4
STAC	3.0	1.2	1.4	0.3	0.5	0.6	1.7	0.3	2.2
EPHA5	4.0	1.3	1.9	0.2	0.4	0.6	1.8	0.2	1.0
YOD1	3.0	0.9	1.4	0.1	0.3	0.5	2.7	0.3	2.1
EFCAB4A	3.6	1.7	2.0	0.3	0.7	0.8	1.1	0.1	0.9
KLHL22	3.2	1.5	2.0	0.4	0.8	1.0	1.2	0.2	1.0
TMEM60	3.3	1.4	2.2	0.2	0.5	0.7	1.4	0.2	1.3
MAMDC4	3.2	1.5	1.8	0.3	0.5	0.8	1.7	0.3	1.2
APOO	2.2	1.2	1.7	0.3	0.7	0.8	2.5	0.4	1.6
LOC100506679	3.6	0.8	1.4	0.2	0.5	0.6	2.9	0.2	1.2
SLC2A4	3.4	0.8	1.5	0.4	0.5	0.6	2.4	0.2	1.5
PEX3	3.0	1.4	1.6	0.1	0.5	0.6	2.1	0.4	1.6
COPG2	3.4	1.2	1.5	0.1	0.5	1.0	2.0	0.1	1.4
LOC100287177	2.8	1.2	2.1	0.4	0.2	0.5	2.7	0.4	1.1
CUL2	3.2	1.0	1.2	0.1	0.4	0.6	2.6	0.3	1.7
LOC100507101	3.1	0.8	2.1	0.2	0.7	0.9	1.6	0.2	1.6
IL7R	3.0	1.3	1.4	0.2	0.3	0.4	2.5	0.5	1.7
THBS3	3.9	1.5	2.4	0.3	0.7	0.9	0.9	0.1	0.6

LOC101927683	3.9	0.8	1.6	0.2	0.5	0.5	2.6	0.1	1.2
KRT10	2.2	1.1	1.1	0.6	0.8	0.9	1.7	0.6	2.3
KAZALD1	3.9	1.4	1.9	0.2	0.4	0.7	1.5	0.3	1.0
LOC101929550	3.7	0.8	1.6	0.2	0.4	0.6	2.6	0.2	1.2
LOC100132356	3.4	1.1	1.7	0.3	0.6	0.9	2.3	0.1	0.9
LOC101928163	3.8	0.6	1.5	0.2	0.5	0.5	2.8	0.3	1.1
LINC00189	4.6	0.7	1.3	0.4	0.3	0.4	2.4	0.2	0.9
C9orf156	3.6	1.1	1.1	0.2	0.5	0.7	1.8	0.5	1.8
ESPN	3.5	1.2	1.8	0.5	0.6	0.9	1.3	0.3	1.1
RABL3	3.4	1.5	2.2	0.1	0.7	0.6	1.2	0.0	1.5
TBPL1	2.8	0.9	1.8	0.1	0.3	0.7	2.0	0.3	2.4
NALCN	3.4	1.1	1.5	0.2	0.6	0.8	1.7	0.3	1.6
LOC151009	2.3	0.9	1.3	0.3	0.5	0.8	2.7	0.4	1.9
FKBPL	2.6	1.1	1.3	0.2	0.4	0.8	2.6	0.5	1.8
ZNF45	3.4	1.2	1.4	0.1	0.3	0.6	1.9	0.2	1.9
SLC29A3	3.4	1.8	1.9	0.3	0.8	0.8	0.9	0.2	1.0
C16orf89	4.2	0.6	1.4	0.2	0.5	0.6	2.4	0.2	1.1
RNF144A-AS1	3.6	0.8	1.6	0.3	0.5	0.4	2.3	0.3	1.4
LOC101927837	3.5	0.6	1.4	0.4	0.5	0.8	2.3	0.3	1.3
BTN3A3	3.0	1.2	1.6	0.1	0.6	0.6	2.3	0.3	1.4
IRX2	2.6	0.9	1.3	0.5	0.8	0.7	1.6	0.7	1.9
MYO1E	3.2	1.2	1.2	0.2	0.4	0.7	2.1	0.3	1.7
PLD2	3.0	1.2	1.3	0.5	0.6	1.0	1.7	0.3	1.6
EPM2AIP1	2.9	1.1	1.5	0.4	0.6	0.7	2.3	0.2	1.4
PRDM2	3.1	0.9	1.2	0.2	0.3	0.8	2.0	0.3	2.3
MTMR6	2.8	1.2	1.6	0.1	0.5	0.6	2.4	0.3	1.8
RNGTT	2.8	1.3	1.6	0.0	0.4	0.7	2.5	0.4	1.4
SLC9A5	2.8	1.1	1.4	0.3	0.6	0.5	2.2	0.4	1.8
LOC101929765	3.1	0.9	1.7	0.2	0.5	0.6	2.9	0.2	1.2
ORC5	3.4	0.9	1.9	0.1	0.4	0.8	1.9	0.2	1.6
MAGEH1	2.9	1.4	1.6	0.6	0.5	0.9	1.8	0.2	1.3
KATNA1	3.1	1.3	1.4	0.2	0.5	0.5	2.4	0.3	1.5
KBTBD6	2.6	1.0	1.3	0.4	0.6	0.8	2.5	0.4	1.7
XRN1	3.5	1.2	1.7	0.1	0.6	0.6	1.8	0.2	1.4
CEP41	2.6	1.2	1.2	0.1	0.8	0.9	1.8	0.4	2.1
UGT8	3.4	1.4	1.8	0.2	0.4	0.6	1.8	0.4	1.3
CAPN7	3.1	1.0	1.3	0.3	0.5	0.5	2.4	0.3	1.8
LINC00909	3.9	0.7	1.6	0.2	0.6	0.5	2.2	0.2	1.3
DIAPH3	2.1	0.8	1.0	0.2	0.4	0.7	3.1	0.4	2.4
ZBED5-AS1	3.7	1.4	2.0	0.1	0.6	0.9	1.5	0.1	0.8
ZNF557	3.0	1.1	1.5	0.1	0.4	0.6	2.9	0.3	1.3
EFNA1	4.0	1.1	2.4	0.0	0.0	0.3	2.2	0.1	0.9
LOC100506860	3.3	0.6	1.3	0.2	0.8	0.6	2.7	0.2	1.3
C5orf28	2.6	0.9	1.7	0.2	0.7	0.5	2.6	0.1	1.9
TSPYL4	2.0	0.8	1.4	0.4	0.7	1.0	2.1	0.2	2.4
LOC100129940	4.0	0.6	1.3	0.2	0.7	0.3	2.7	0.1	1.3
LOC101929468	3.6	0.9	1.6	0.3	0.4	0.5	2.4	0.2	1.2
RAB4B	3.8	1.4	1.8	0.4	0.6	0.4	1.6	0.3	0.8
NCS1	3.6	1.0	1.3	0.2	0.2	0.8	2.1	0.4	1.5
EDA	3.9	1.1	1.6	0.2	0.5	0.6	1.6	0.2	1.4
LOC101928033	3.5	0.7	1.7	0.1	0.2	0.5	2.8	0.2	1.3
TSC22D1-AS1	3.4	1.0	1.5	0.3	0.6	0.8	1.9	0.3	1.3
KIAA1804	3.2	1.4	1.5	0.3	0.3	0.6	1.9	0.3	1.5
MIPOL1	2.8	1.0	1.1	0.1	0.6	0.5	2.6	0.3	2.1
ZNF34	3.6	1.1	2.0	0.2	0.5	0.7	1.5	0.2	1.2

COQ10A		1.9	0.9	1.4	0.2	0.5	0.5	3.2	0.5	1.9
LOH12CR1		2.8	0.9	1.6	0.7	0.5	1.1	2.0	0.2	1.3
ESCO1		2.7	0.9	1.3	0.1	0.4	0.5	2.8	0.3	2.2
PAX8		2.6	1.0	1.5	0.4	0.5	0.8	2.5	0.3	1.4
PAPOLG		2.9	1.1	1.3	0.1	0.6	0.5	2.1	0.4	2.1
CCDC68		3.4	0.8	1.5	0.0	0.3	0.4	2.4	0.2	2.0
CYB561A3		2.4	1.1	1.1	0.3	1.2	1.0	2.2	0.3	1.4
TADA1		2.9	1.1	1.8	0.2	0.4	0.9	1.7	0.4	1.6
LINC00637		3.5	0.7	1.4	0.2	0.5	0.5	2.8	0.2	1.2
KCNQ3		1.9	0.7	1.2	0.3	1.2	1.2	2.8	0.2	1.6
BCL9		3.3	1.3	1.6	0.2	0.5	0.9	1.5	0.4	1.5
LINC00314		3.8	0.6	1.1	0.4	0.4	0.6	2.7	0.1	1.4
LOC101927727		3.3	0.9	1.7	0.4	0.6	0.8	1.5	0.3	1.5
PPOX		3.0	1.4	1.6	0.5	0.7	0.7	1.7	0.3	1.2
LOC100507316		2.7	1.3	2.1	0.4	0.4	0.8	1.4	0.2	1.5
PRKAB2		3.4	1.3	1.9	0.2	0.3	0.6	1.7	0.2	1.5
GPR153		2.9	1.3	1.8	0.6	0.8	1.0	1.6	0.2	0.9
LINC00607		3.4	0.8	1.5	0.2	0.5	0.5	2.6	0.2	1.3
TCAIM		3.4	1.2	1.2	0.0	0.4	0.6	2.1	0.2	2.0
SNORA33		2.5	1.0	1.0	0.3	0.8	2.2	1.2	0.2	1.8
FHIT		3.5	1.1	1.1	0.4	0.4	1.3	1.7	0.3	1.4
LOC648044		3.6	0.9	2.0	0.3	0.3	0.9	1.3	0.3	1.5
KRT7		3.4	1.3	1.5	0.4	0.6	0.9	1.7	0.3	1.1
LCORL		3.3	1.1	1.7	0.2	0.5	0.4	2.1	0.4	1.4
SHROOM2		3.5	1.2	1.6	0.5	0.8	1.0	1.2	0.2	1.0
RAB3IP		2.8	0.8	1.3	0.1	0.4	0.6	3.0	0.2	1.8
LINC00083		3.8	0.8	1.3	0.2	0.5	0.5	2.7	0.2	1.1
TMEM2		3.1	1.2	1.5	0.2	0.4	0.6	2.1	0.3	1.7
ZSCAN21		3.1	1.3	1.1	0.2	0.6	0.8	2.0	0.3	1.6
AP1M2		3.3	1.6	2.2	0.1	0.3	0.7	1.6	0.1	1.1
SNORA51		1.3	1.5	1.8	0.8	0.5	1.2	2.0	0.4	1.5
SH3YL1		3.0	1.1	1.6	0.1	0.4	0.6	2.4	0.4	1.4
RASA2		3.3	1.1	1.5	0.1	0.4	0.6	2.2	0.2	1.7
HOXD1		3.1	1.4	1.6	0.2	0.2	0.7	1.6	0.5	1.6
LEPREL2		3.3	1.1	1.4	0.4	0.7	0.7	1.9	0.3	1.2
TTF1		3.1	1.2	1.5	0.1	0.3	0.5	2.2	0.4	1.6
CAMKMT		2.4	1.1	1.7	0.1	0.4	0.6	2.3	0.3	2.1
RCAN3		4.3	1.5	1.6	0.0	0.3	0.5	1.4	0.2	1.2
RILP		3.9	1.7	1.7	0.3	0.6	0.6	1.1	0.2	0.8
LOC101928093		2.9	1.2	1.5	0.3	0.7	0.8	2.4	0.3	1.0
ARMCX5		2.9	1.1	1.3	0.1	0.5	0.7	2.2	0.4	1.8
CASP1		2.9	1.4	1.6	0.1	0.2	0.7	2.2	0.2	1.7
TRIM13		3.2	0.9	2.0	0.1	0.4	0.3	2.0	0.2	2.0
ZNF133		3.2	1.4	1.7	0.3	0.7	0.8	1.5	0.2	1.0
MXD3		2.8	1.4	2.2	0.5	0.5	1.0	1.5	0.3	0.9
TEFM		3.2	1.1	1.3	0.1	0.3	0.6	2.4	0.2	1.9
CRY2		3.5	1.5	1.6	0.3	0.4	0.9	1.6	0.2	1.1
HS3ST3A1		2.5	1.4	1.6	0.5	1.0	0.8	1.4	0.2	1.6
	44622.0	2.8	0.8	1.3	0.2	0.6	0.7	2.8	0.3	1.5
UHRF1BP1L		3.3	1.0	1.4	0.1	0.4	0.4	2.1	0.3	2.0
TGS1		2.7	0.9	1.2	0.1	0.4	0.7	2.5	0.3	2.2
DPF3		2.7	1.0	1.6	0.4	1.0	1.1	1.3	0.3	1.6
STRADB		3.3	1.3	1.9	0.1	0.6	1.0	1.2	0.3	1.2
SNPH		3.3	0.8	2.0	0.3	0.6	0.6	2.0	0.3	1.0
RAB23		3.0	1.0	1.3	0.1	0.5	0.7	2.2	0.3	1.9

LTN1	2.7	1.1	1.3	0.1	0.3	0.4	2.5	0.3	2.2
PCDHGB1	3.7	1.1	1.7	0.3	0.6	0.8	1.5	0.2	1.0
SLC23A3	3.3	1.0	1.6	0.1	0.3	0.4	2.5	0.4	1.4
OSGIN2	3.3	1.1	1.4	0.1	0.4	0.7	1.7	0.2	1.9
KCNN4	2.3	1.1	1.5	0.5	0.7	0.9	2.0	0.3	1.4
G2E3	3.1	1.3	1.8	0.1	0.2	0.5	2.0	0.2	1.6
MMD	3.5	1.4	1.9	0.1	0.6	1.1	1.2	0.1	0.8
LOC440600	3.8	0.6	1.5	0.2	0.4	0.4	2.6	0.2	1.1
C11orf91	2.9	0.9	1.8	0.7	0.6	0.9	1.5	0.2	1.3
INIP	2.8	1.5	2.0	0.1	0.4	0.9	1.5	0.2	1.3
FAM86C1	2.8	1.0	1.3	0.2	0.7	1.0	2.1	0.4	1.4
FBXO5	3.1	0.9	1.2	0.1	0.6	0.8	2.4	0.2	1.6
TRMT61B	3.2	1.3	1.5	0.1	0.6	0.5	1.9	0.2	1.5
LOC101928897	3.1	0.8	1.9	0.4	0.6	0.2	2.8	0.2	0.9
DENND5B	3.2	0.9	1.4	0.2	0.6	0.6	2.3	0.3	1.3
MTRF1	3.6	1.2	2.0	0.1	0.4	0.7	1.4	0.3	1.3
ABHD6	3.4	1.3	1.8	0.2	0.3	0.7	1.9	0.2	1.2
USP16	2.9	0.9	1.8	0.1	0.4	0.7	2.3	0.2	1.5
KIAA1671	1.8	0.7	2.9	0.2	0.4	0.8	1.4	0.8	2.1
MTHFD2L	3.1	0.8	1.5	0.1	0.4	0.6	2.3	0.4	1.6
TEP1	2.9	0.9	1.5	0.2	0.5	0.6	2.5	0.3	1.5
CNIH3	3.2	1.5	2.0	0.2	0.7	0.5	1.4	0.1	1.2
MAF	0.7	2.4	1.5	1.3	0.7	0.1	1.7	1.2	1.2
SLC5A2	3.0	1.0	1.4	0.4	0.6	1.1	1.8	0.3	1.4
ATP10D	3.0	1.1	1.5	0.1	0.6	0.7	2.2	0.2	1.4
LINC00908	3.5	0.7	1.4	0.3	0.4	0.5	2.8	0.2	1.0
TTLL10-AS1	3.6	0.7	1.5	0.2	0.4	0.5	2.7	0.2	1.1
SLC46A1	2.8	1.1	0.9	0.2	0.4	0.8	2.4	0.4	1.7
PRPF40B	3.0	1.0	1.7	0.3	0.8	0.8	1.7	0.3	1.1
LARP1B	2.2	0.5	1.1	0.1	0.4	0.5	2.9	0.3	2.8
TRPV1	3.4	1.2	1.7	0.4	0.6	0.8	1.5	0.2	0.9
NRTN	2.1	0.8	1.7	0.5	0.5	0.8	2.6	0.6	1.3
SPAG5	3.3	1.3	1.6	0.2	0.6	0.7	1.9	0.3	1.1
PTPN13	2.6	0.9	1.2	0.1	0.3	0.6	2.6	0.4	2.1
MUT	3.4	1.2	1.4	0.1	0.6	0.7	1.6	0.1	1.6
FAM83D	2.8	0.8	1.1	0.4	0.7	1.0	2.1	0.5	1.4
AP4M1	2.5	1.1	1.0	0.3	0.8	0.9	2.2	0.4	1.7
CLK4	3.0	1.1	1.9	0.1	0.4	0.6	2.0	0.2	1.5
HLA-G	3.2	1.4	1.8	0.4	0.8	0.8	1.0	0.2	1.1
EPHA1-AS1	3.1	0.7	1.5	0.1	0.4	0.4	3.0	0.2	1.2
COL4A1	3.1	1.1	1.5	0.2	0.6	0.8	2.1	0.2	1.1
BTD	3.4	0.9	1.4	0.3	0.7	1.0	1.9	0.2	1.0
LEF1	2.3	0.9	1.2	0.2	0.5	1.1	1.6	0.4	2.4
C12orf61	2.3	1.0	1.8	0.6	0.5	1.0	2.7	0.2	0.6
ACSL1	3.4	1.3	1.6	0.2	0.4	0.4	1.8	0.3	1.3
FBXO9	2.8	1.4	1.7	0.1	0.6	0.8	1.8	0.3	1.4
CYB561D2	2.8	1.0	1.1	0.5	0.9	0.8	2.2	0.3	1.2
LOC100996842	3.0	1.2	1.8	0.2	0.7	0.8	1.5	0.3	1.1
HDHD3	2.4	0.9	1.6	0.2	0.6	0.9	2.4	0.3	1.4
SAP25	3.3	1.4	2.1	0.1	0.5	0.5	1.0	0.3	1.5
B4GALT4	3.8	1.0	1.5	0.0	0.4	0.5	2.2	0.1	1.2
ZNF530	3.5	0.8	1.4	0.2	0.4	0.5	2.5	0.2	1.2
ULBP2	2.3	1.3	1.4	0.3	0.8	1.1	1.8	0.3	1.3
CCDC28B	3.5	0.7	1.6	0.4	0.4	0.9	1.9	0.2	1.0
LOC101929747	3.1	1.2	1.9	0.4	0.5	0.6	1.4	0.4	1.4

PYGO1	3.2	1.0	1.1	0.1	0.4	0.6	2.0	0.3	1.9
TNXB	3.3	1.1	1.6	0.3	0.6	0.7	1.8	0.2	0.9
LINC00638	3.1	0.9	1.6	0.2	0.6	0.6	2.4	0.1	1.1
TBC1D3G	3.1	1.4	1.8	0.3	0.7	0.7	1.3	0.3	1.1
MFSD4	3.4	0.6	1.5	0.2	0.4	0.5	2.4	0.2	1.3
FAM109B	2.9	0.9	1.2	0.1	0.5	0.6	2.7	0.3	1.4
TTC27	3.2	1.0	1.5	0.1	0.5	0.7	2.0	0.1	1.5
CYB5D2	3.3	1.4	1.8	0.2	0.6	0.9	1.3	0.1	1.0
STPG1	3.3	1.2	1.4	0.2	0.7	0.8	1.2	0.4	1.5
S100A3	3.4	1.8	1.5	0.1	0.3	1.2	1.3	0.2	0.9
MYO9A	3.1	0.8	1.4	0.1	0.4	0.6	2.2	0.3	1.6
C1orf27	3.0	0.9	1.4	0.1	0.4	0.7	2.0	0.4	1.8
KIAA1432	2.6	0.8	1.2	0.1	0.4	0.5	2.5	0.4	2.0
SYTL2	3.5	1.4	2.1	0.1	0.4	0.4	1.4	0.2	1.2
LOC100506551	3.4	0.7	1.6	0.3	0.4	0.5	2.4	0.2	1.1
XK	2.8	1.0	1.4	0.3	0.4	0.5	1.8	0.5	1.8
HLCS	3.2	1.0	1.5	0.3	0.7	0.8	1.6	0.2	1.2
SUV420H2	2.9	0.8	1.2	0.5	0.6	1.0	1.9	0.5	1.4
ZNF283	3.6	0.7	1.5	0.2	0.3	0.5	2.5	0.2	1.1
GAB1	3.4	1.0	1.4	0.2	0.4	0.6	1.7	0.4	1.5
FAM215A	3.8	0.9	1.4	0.2	0.3	0.8	2.0	0.3	0.9
CRIP2	3.3	1.0	1.7	0.2	0.6	0.5	2.0	0.5	0.7
SIM2	2.4	0.8	1.0	0.4	0.5	1.0	2.1	0.3	1.9
LOC101928604	3.3	0.8	1.5	0.3	0.4	0.6	2.5	0.3	1.0
IFI6	2.9	1.5	2.1	0.2	0.6	1.0	1.1	0.2	0.9
FDXR	2.3	0.9	1.3	0.3	0.7	1.2	2.3	0.3	1.2
PLEKHA4	2.7	1.4	1.3	0.4	0.8	0.9	1.6	0.3	1.1
NUBPL	3.8	1.0	1.6	0.1	0.5	0.7	1.7	0.0	1.1
YAF2	2.6	1.3	1.5	0.2	0.4	0.5	2.4	0.3	1.2
LOC100128818	3.1	0.7	1.4	0.2	0.4	0.6	2.7	0.2	1.2
RGS12	3.3	1.4	1.4	0.3	0.4	0.7	1.7	0.3	1.1
CHIC1	3.5	1.4	1.3	0.2	0.6	0.3	1.7	0.3	1.2
NARG2	2.7	1.2	1.6	0.1	0.3	0.5	1.8	0.2	2.1
RAP2C	2.7	1.1	1.9	0.1	0.4	0.5	2.2	0.4	1.3
ZNF550	3.0	1.3	1.6	0.2	0.6	0.5	1.7	0.3	1.3
FAM111A	3.0	1.1	1.3	0.2	0.4	0.6	2.2	0.4	1.3
C5AR1	1.1	0.9	0.7	0.1	0.3	0.2	3.5	0.8	2.9
C3orf52	2.6	1.0	1.4	0.2	0.3	0.4	2.6	0.3	1.7
RGS4	2.9	1.2	1.4	0.2	0.6	0.7	1.8	0.3	1.5
LOC401242	2.7	0.9	1.1	1.0	0.7	1.0	1.4	0.4	1.3
ZNF83	2.9	1.2	2.1	0.1	0.5	0.7	1.4	0.1	1.5
ZNF699	2.5	1.1	1.2	0.1	0.5	0.6	2.3	0.3	1.6
OAS3	3.2	1.1	1.5	0.4	0.7	0.8	1.5	0.3	1.0
ALKBH8	2.7	0.8	1.5	0.1	0.5	0.5	2.4	0.4	1.6
NEU3	2.2	0.5	0.9	0.2	0.4	0.7	3.2	0.4	2.0
LOC550112	2.6	1.0	1.3	0.2	0.4	0.5	2.3	0.3	1.8
SPECC1	2.6	1.0	1.2	0.1	0.3	0.5	2.5	0.4	1.9
OSBPL5	3.1	1.2	1.5	0.3	0.5	0.9	1.6	0.2	1.1
IFI35	3.0	1.0	1.9	0.3	0.7	0.8	1.6	0.2	1.0
C9orf85	2.8	1.2	1.4	0.1	0.4	0.7	1.8	0.5	1.5
CCDC8	2.0	0.9	1.5	0.5	0.7	0.8	1.9	0.4	1.7
RPP40	2.6	1.2	1.3	0.2	0.8	0.7	1.9	0.1	1.7
ANKRD29	3.2	1.1	1.4	0.3	1.0	1.3	1.2	0.1	0.9
CERS1	2.3	1.0	1.3	0.3	0.6	0.8	1.8	0.4	1.8
RBM24	2.5	1.3	1.0	0.3	0.4	1.1	1.4	0.5	1.9

BBS7	3.3	1.2	1.5	0.1	0.4	0.5	1.7	0.2	1.6
MLLT3	2.7	0.9	1.5	0.2	0.5	0.7	1.9	0.3	1.9
SH2D3A	3.7	1.1	1.7	0.2	0.5	0.5	1.4	0.2	1.2
LOC101928719	3.1	1.2	1.7	0.2	0.5	0.5	1.8	0.1	1.3
H2AFJ	3.2	1.3	1.9	0.2	0.6	0.5	1.5	0.2	1.0
TOMM40L	2.1	1.0	1.0	0.3	0.4	1.2	2.6	0.3	1.5
APOBEC3F	2.8	1.7	1.4	0.4	0.7	0.9	1.3	0.2	1.0
FLJ20444	2.4	1.2	1.3	0.4	0.5	0.9	2.0	0.4	1.5
NR2F1-AS1	3.6	1.0	1.9	0.2	0.3	0.6	1.5	0.2	1.2
ASAP3	3.6	1.3	1.5	0.2	0.6	1.0	1.3	0.2	0.7
CROCC	3.1	1.0	1.5	0.4	0.6	0.7	1.8	0.2	1.0
NPHP3	3.6	0.9	1.5	0.2	0.5	0.6	2.1	0.2	1.0
C9orf116	2.7	1.0	1.8	0.3	0.5	1.1	1.2	0.3	1.4
LOC728323	2.7	1.1	1.2	0.1	0.7	0.7	2.0	0.3	1.6
AGA	3.4	1.1	1.7	0.3	0.7	0.9	1.2	0.2	0.9
CDK5R1	2.9	0.8	0.9	0.2	0.4	0.5	2.1	0.5	2.2
LOC101928300	3.7	0.8	1.4	0.1	0.3	0.5	2.5	0.2	0.9
RBL1	2.6	1.0	1.3	0.2	0.6	0.8	1.9	0.3	1.7
FOXO1	2.9	1.2	1.4	0.6	0.6	0.7	1.5	0.4	1.1
EMR2	3.1	1.5	1.5	0.2	0.4	0.7	1.5	0.2	1.1
LOC100289495	3.5	0.8	1.6	0.1	0.4	0.7	1.9	0.2	1.2
DNASE1	3.1	0.8	1.5	0.3	0.6	0.7	1.8	0.3	1.3
HAUS1	3.3	1.0	1.5	0.1	0.4	0.7	1.5	0.3	1.7
GNG7	2.7	1.4	2.1	0.1	0.5	1.6	0.4	0.2	1.4
LOC285638	3.4	0.8	1.6	0.1	0.4	0.4	2.3	0.2	1.3
LINS	3.2	0.9	1.7	0.2	0.5	0.6	2.0	0.2	1.2
LOC101928164	3.8	0.7	1.9	0.3	0.4	0.7	1.5	0.2	1.0
LINC00652	3.5	0.8	1.6	0.2	0.6	0.5	1.8	0.1	1.2
SYK	2.6	1.1	1.2	0.2	0.4	0.4	2.3	0.3	1.8
KRBOX1	1.8	1.0	1.7	0.3	1.2	1.0	1.9	0.4	1.0
LOC642361	2.7	1.3	1.3	0.5	0.7	0.8	1.5	0.2	1.4
LOC100505495	3.3	0.7	1.5	0.2	0.5	0.5	2.4	0.2	1.1
ZNF514	3.0	0.8	1.3	0.1	0.6	0.8	1.9	0.2	1.7
KIAA0391	2.8	1.0	1.3	0.1	0.2	0.5	2.2	0.5	1.7
TMEM260	3.0	1.1	1.8	0.2	0.4	0.6	1.6	0.2	1.4
USP35	3.1	1.2	1.6	0.2	0.6	0.6	1.6	0.3	1.2
PPP2R3B	2.5	1.1	1.4	0.3	0.4	0.6	2.2	0.3	1.6
PPP2R3B	2.5	1.1	1.4	0.3	0.4	0.6	2.2	0.3	1.6
MREG	2.6	1.2	1.8	0.2	0.4	0.6	1.9	0.1	1.5
TYW5	3.2	1.0	1.6	0.1	0.4	0.5	2.1	0.1	1.2
NANOS1	1.7	0.7	0.8	0.7	0.7	0.9	2.7	0.5	1.5
ZBTB33	2.6	1.3	1.3	0.1	0.5	0.6	1.8	0.2	2.0
ARSK	2.8	1.1	1.6	0.1	0.4	0.3	1.9	0.2	2.0
DYRK4	2.9	1.0	1.6	0.2	0.7	0.8	1.1	0.4	1.7
TMEM68	3.2	1.2	1.6	0.1	0.3	0.3	1.9	0.3	1.3
CCBL1	3.4	1.1	1.6	0.4	0.7	0.7	1.3	0.1	1.0
MYBL1	2.0	0.7	1.1	0.1	0.6	0.6	2.6	0.3	2.2
SYDE2	2.6	0.9	1.2	0.2	0.3	0.3	2.8	0.3	1.8
ZNF497	3.1	1.1	1.8	0.5	0.5	0.7	1.6	0.2	0.8
WDR44	3.2	1.0	1.1	0.1	0.4	0.5	2.1	0.3	1.6
LOC101929020	3.1	0.9	1.4	0.5	0.8	0.9	1.5	0.3	0.9
LRIF1	2.8	1.3	1.4	0.0	0.5	0.5	1.9	0.3	1.6
LOC646719	3.2	0.8	1.4	0.2	0.4	0.5	2.4	0.2	1.2
WDR19	3.1	1.1	1.2	0.1	0.5	0.7	2.0	0.2	1.3
UBA7	2.9	1.6	2.0	0.2	0.6	0.6	1.2	0.2	0.9

LOC100129550	3.4	0.9	1.5	0.1	0.5	0.6	1.9	0.2	1.0
LOC101927150	3.7	0.8	1.5	0.1	0.2	0.6	2.1	0.1	1.3
LOC101929225	2.8	0.7	1.6	0.3	0.5	0.7	2.3	0.2	1.1
TMEM254-AS1	3.6	0.8	1.5	0.1	0.3	0.5	2.2	0.2	1.1
FLJ12825	3.4	0.6	1.3	0.2	0.4	0.6	2.5	0.2	1.1
RAB17	3.9	1.6	2.3	0.0	0.1	0.2	1.2	0.1	0.8
DBF4B	2.8	1.1	1.3	0.4	0.5	0.9	1.6	0.3	1.4
EIF4EBP3	3.1	1.5	1.5	0.4	0.7	0.6	1.3	0.0	1.2
ZNF513	2.3	1.1	1.8	0.3	0.6	0.8	1.6	0.2	1.4
TPST1	2.3	1.1	1.6	0.1	0.6	0.5	2.2	0.4	1.5
PPP2R2C	3.1	1.0	1.9	0.2	0.9	0.8	0.9	0.2	1.3
SERPINF1	3.7	1.5	1.8	0.3	0.5	0.6	0.9	0.2	0.6
CA12	3.2	1.8	1.7	0.4	0.6	1.2	0.5	0.2	0.6
C14orf79	3.0	0.9	1.2	0.3	0.6	0.8	1.7	0.3	1.4
KIF16B	2.9	0.8	1.3	0.1	0.4	0.5	2.3	0.3	1.5
SYT15	3.1	0.7	1.4	0.4	0.6	0.8	1.7	0.3	1.2
METTL12	2.3	0.8	1.5	0.3	0.6	0.4	2.6	0.2	1.5
CHSY3	2.9	1.2	1.4	0.3	0.5	0.6	1.7	0.4	1.1
C2CD3	2.6	0.7	1.2	0.2	0.5	0.7	2.1	0.4	1.9
POLR3F	2.7	1.2	1.7	0.1	0.3	0.6	1.8	0.2	1.6
RMND1	2.7	1.0	1.1	0.1	0.5	0.7	2.1	0.2	1.8
GUF1	2.6	0.9	1.3	0.1	0.7	0.7	2.2	0.3	1.4
WDR7	2.7	1.1	1.6	0.1	0.6	0.7	1.7	0.3	1.3
LOC101929543	3.1	1.0	1.1	0.4	0.6	0.7	2.4	0.2	0.7
ZNF776	2.9	1.3	1.3	0.1	0.4	0.6	1.8	0.3	1.5
DCUN1D4	2.9	1.0	1.9	0.1	0.5	0.7	1.5	0.2	1.4
DAAM1	3.2	1.0	1.6	0.2	0.5	0.5	1.6	0.2	1.4
KLF8	3.0	1.0	1.6	0.1	0.5	0.6	1.7	0.3	1.4
MAGEA3	2.6	1.1	1.2	0.3	0.5	0.8	1.7	0.4	1.6
CLCN2	2.2	0.8	1.1	0.3	0.4	0.5	2.5	0.4	1.8
LOC101928718	3.4	0.6	1.3	0.2	0.6	0.4	2.5	0.3	0.8
ENOSF1	2.9	1.2	1.5	0.2	1.0	0.8	1.7	0.2	0.7
ASIC1	2.5	1.3	1.6	0.3	0.8	1.1	1.3	0.2	1.0
GPR35	2.3	0.5	1.5	0.3	0.5	0.4	2.7	0.5	1.4
APITD1	2.7	1.2	1.9	0.1	0.6	0.8	1.3	0.2	1.2
SNORA11D	2.3	1.3	1.2	0.2	1.2	0.4	1.3	0.1	2.1
SNORA11E	2.3	1.3	1.2	0.2	1.2	0.4	1.3	0.1	2.1
ANKRD23	3.1	0.8	1.6	0.3	0.3	0.7	1.5	0.3	1.5
ZNF589	3.0	0.9	1.3	0.3	0.6	0.5	1.6	0.2	1.8
LOC101927819	3.5	0.7	1.6	0.1	0.4	0.4	2.1	0.2	1.1
ST7L	3.0	1.0	1.4	0.1	0.5	0.6	1.7	0.3	1.5
ALMS1	3.0	0.9	1.4	0.1	0.5	0.7	1.8	0.2	1.5
FAM206A	2.2	1.3	2.0	0.1	0.3	0.6	1.6	0.4	1.6
VPS13C	3.2	0.8	1.5	0.1	0.4	0.5	2.0	0.2	1.4
LOC101927792	3.3	0.7	1.4	0.2	0.4	0.5	2.5	0.2	1.0
MMP11	3.4	1.5	2.2	0.4	0.5	0.8	0.6	0.1	0.5
LOC101927905	3.0	1.1	1.0	0.4	0.5	0.7	1.4	0.2	1.6
SNX29	2.9	0.6	1.3	0.2	0.7	0.9	1.9	0.2	1.4
CCDC77	2.4	0.9	1.3	0.2	0.3	0.7	2.0	0.4	1.8
MAP3K15	3.3	0.9	1.5	0.3	0.4	0.5	2.1	0.2	1.0
RFT1	2.6	0.8	1.4	0.2	0.5	0.8	1.9	0.3	1.5
AHSA2	3.2	1.0	1.4	0.1	0.4	0.7	2.2	0.1	0.9
ZBTB41	2.9	1.1	1.1	0.1	0.4	0.4	2.4	0.3	1.6
MIR17HG	3.0	0.8	1.2	0.2	0.4	0.3	2.8	0.2	1.1
LIN54	2.3	0.8	1.2	0.1	0.5	0.7	2.1	0.4	1.8

ARHGEF5	3.2	1.2	1.6	0.2	0.4	0.5	1.5	0.3	1.2
CCAT1	3.1	0.7	1.6	0.2	0.4	0.4	2.4	0.2	1.0
MGC32805	2.9	1.1	1.9	0.1	0.3	0.3	1.7	0.3	1.5
CHCHD5	3.5	1.4	1.7	0.6	0.9	0.9	0.5	0.1	0.5
FAM114A2	2.6	1.3	1.7	0.1	0.6	0.6	1.5	0.2	1.4
ZNF320	3.2	1.0	1.9	0.1	0.5	0.7	1.4	0.1	1.1
DBR1	2.8	0.8	1.1	0.2	0.5	0.7	2.2	0.2	1.6
GAL3ST3	2.4	0.7	1.6	0.6	0.6	0.8	2.3	0.2	0.8
KLHL8	2.6	0.9	1.3	0.1	0.4	0.4	2.2	0.3	1.8
CSF3	3.7	1.8	2.2	0.3	0.5	0.6	0.4	0.0	0.4
PMS1	2.7	0.8	1.2	0.1	0.3	0.4	2.6	0.2	1.7
ZFHX4	2.7	0.8	1.1	0.2	0.5	0.6	2.0	0.3	1.7
CDK8	3.0	1.0	1.4	0.1	0.4	0.6	1.6	0.2	1.8
TMEM79	2.8	1.0	1.6	0.3	0.5	0.7	1.5	0.2	1.3
FAM225A	3.0	1.3	1.2	0.1	0.5	0.4	1.8	0.3	1.4
MST1	3.0	1.1	1.6	0.2	0.4	0.6	1.7	0.3	1.0
KHK	2.6	0.8	1.1	0.3	0.5	0.8	2.0	0.4	1.4
ZNF721	3.1	0.8	1.3	0.1	0.4	0.4	2.3	0.2	1.5
GPR75-ASB3	3.0	1.1	1.1	0.1	0.2	0.5	1.8	0.3	2.0
C2orf44	2.3	1.0	1.4	0.1	0.3	0.7	2.1	0.4	1.7
CEP290	3.3	0.9	1.5	0.0	0.3	0.5	1.8	0.2	1.4
APC2	2.9	1.1	1.6	0.4	0.5	0.8	1.4	0.3	1.0
KIF2C	1.9	0.9	1.3	0.3	1.0	0.9	1.9	0.4	1.3
LSM11	2.5	1.0	1.6	0.2	0.3	0.5	2.2	0.2	1.5
FAM57B	3.1	1.1	1.5	0.6	0.4	0.7	1.1	0.4	1.1
DHTKD1	2.6	0.9	1.4	0.2	0.6	0.8	1.9	0.2	1.4
POT1	3.0	1.0	1.5	0.1	0.5	0.5	1.6	0.2	1.5
ISM1	3.6	1.1	1.4	0.3	0.7	0.9	0.6	0.3	1.0
ZNF28	3.0	0.9	1.4	0.2	0.5	0.5	2.1	0.2	1.2
MLH3	2.5	0.8	1.3	0.1	0.3	0.5	2.4	0.3	1.8
ODF2L	3.1	1.0	1.4	0.1	0.5	0.6	1.8	0.2	1.4
ACADS	3.1	1.3	1.9	0.4	0.6	0.6	1.2	0.2	0.9
RPAP2	2.6	1.0	1.4	0.0	0.4	0.4	2.3	0.2	1.6
PRTFDC1	3.0	0.9	1.3	0.1	0.2	0.6	1.6	0.4	1.8
TNFAIP8	2.4	1.3	1.8	0.2	1.0	0.6	1.3	0.4	1.1
TTI2	2.5	1.2	1.2	0.0	0.3	0.6	2.1	0.1	1.8
GMEB1	2.7	1.1	1.2	0.1	0.5	0.7	1.7	0.3	1.6
HIGD1B	4.4	0.8	1.5	0.1	0.4	0.9	1.1	0.3	0.5
LINC00648	3.4	0.8	1.7	0.1	0.5	0.5	1.6	0.1	1.2
TSPAN31	3.0	1.1	1.6	0.2	0.5	0.9	1.4	0.1	1.1
PPM1K	2.8	0.7	1.2	0.1	0.4	0.4	2.5	0.1	1.7
PCDHB11	3.5	1.1	1.5	0.4	0.6	0.6	1.2	0.2	0.9
MPND	2.4	1.0	1.2	0.5	1.0	1.0	1.5	0.1	1.1
CPT1C	2.4	1.2	1.3	0.5	0.8	1.1	1.4	0.2	1.1
VPS54	2.8	1.0	1.4	0.1	0.4	0.5	2.1	0.3	1.4
ZNF543	2.7	0.7	1.2	0.1	0.6	0.6	2.1	0.2	1.7
KIAA1024	2.5	0.6	1.2	0.2	0.5	0.8	2.1	0.3	1.8
RAB28	2.2	0.6	1.7	0.0	0.3	0.7	2.6	0.4	1.4
PBX3	3.1	1.0	1.2	0.2	0.4	0.5	1.5	0.3	1.7
KIAA1467	3.1	1.2	1.2	0.2	0.3	0.7	1.6	0.3	1.2
LXN	3.0	1.5	2.1	0.1	0.5	0.7	1.2	0.2	0.8
COQ7	2.2	0.9	1.2	0.2	0.9	0.8	2.2	0.3	1.4
PLCL2	3.1	1.3	1.5	0.0	0.3	0.4	1.6	0.2	1.4
FLJ46320	3.5	0.7	1.4	0.1	0.4	0.5	2.3	0.1	0.9
VAMP7	2.9	1.0	1.4	0.1	0.5	0.6	1.6	0.2	1.4

VAMP7	2.9	1.0	1.4	0.1	0.5	0.6	1.6	0.2	1.4
PPM1D	2.6	1.0	1.2	0.1	0.4	0.5	2.1	0.3	1.6
ZYG11B	3.3	1.3	1.3	0.1	0.6	0.7	1.3	0.2	1.0
SLC31A2	3.1	1.2	1.4	0.0	0.8	0.6	1.2	0.4	1.2
LINC01023	2.6	1.2	1.7	0.1	0.5	0.5	1.7	0.5	1.0
LOC730338	3.4	0.6	1.8	0.1	0.4	0.6	1.9	0.1	1.0
RAD51-AS1	3.3	0.8	1.3	0.1	0.4	0.8	1.9	0.1	1.2
CCNA2	1.6	1.0	1.0	0.4	0.7	0.8	2.3	0.4	1.8
BRI3BP	1.9	0.6	1.3	0.4	0.7	0.5	2.2	0.5	1.7
VASH1	2.6	1.1	1.4	0.3	0.4	0.9	1.8	0.4	1.0
LOC101928139	3.1	0.8	1.5	0.2	0.4	0.4	2.3	0.2	1.0
LOC100506411	2.1	0.8	1.2	0.1	0.7	1.0	2.3	0.3	1.3
LOC100128653	2.4	0.8	1.6	0.2	0.4	0.5	2.3	0.3	1.3
PGAM2	2.7	1.0	1.6	0.2	0.4	0.6	1.7	0.2	1.3
CELSR3	3.0	1.1	1.8	0.3	0.5	0.6	1.4	0.2	1.0
PPP1R3D	2.7	0.8	1.0	0.5	0.5	0.8	1.7	0.6	1.2
MCTP2	3.8	1.1	1.2	0.1	0.2	0.4	1.7	0.2	1.0
LOC100129518	3.0	0.9	1.5	0.2	0.4	0.4	2.3	0.2	0.9
LOC101927160	2.9	0.9	1.4	0.6	0.7	1.2	0.7	0.5	1.0
EPN2-AS1	3.6	0.5	1.3	0.2	0.7	0.5	2.0	0.2	0.7
MYCNOS	2.9	0.7	1.4	0.2	0.4	0.6	2.7	0.2	0.9
TMEM135	3.1	1.0	1.7	0.1	0.3	0.6	1.3	0.2	1.5
BMF	4.0	1.6	1.6	0.1	0.4	0.3	0.9	0.2	0.9
SIGIRR	3.1	1.2	1.7	0.4	0.6	1.0	1.2	0.2	0.6
PTPRCAP	2.5	1.0	1.3	0.4	0.2	1.2	1.8	0.3	1.1
SLC2A11	2.7	1.0	1.5	0.3	0.4	0.5	1.7	0.3	1.5
SMAP2	2.1	1.0	1.5	0.1	0.5	0.7	1.8	0.4	1.6
EEF1E1	2.3	1.1	1.9	0.1	0.3	0.5	1.4	0.2	2.0
PCDHB14	3.5	1.0	1.5	0.2	0.5	0.6	1.3	0.2	0.9
NBPF14	2.8	0.8	1.2	0.1	0.6	0.6	1.9	0.3	1.5
SNRNP48	2.9	1.2	1.3	0.1	0.2	0.7	1.7	0.3	1.3
ALDH4A1	2.8	0.8	1.2	0.5	0.7	1.0	1.4	0.2	1.1
LOC100507642	2.9	0.7	1.4	0.1	0.3	0.4	2.4	0.1	1.3
C1orf159	2.6	1.2	1.4	0.2	0.5	0.8	1.6	0.3	1.1
MAOA	2.5	0.9	1.5	0.2	0.5	0.8	1.8	0.2	1.5
LOC101927707	2.5	1.1	1.1	0.5	0.8	1.0	1.2	0.2	1.3
ASZ1	3.3	0.6	1.3	0.3	0.3	0.5	2.2	0.2	1.0
LOC100129148	3.7	0.0	1.0	0.4	0.0	0.0	2.7	0.0	1.8
ATP9B	3.2	1.0	1.5	0.3	0.6	0.9	1.4	0.1	0.9
KIAA1524	2.2	0.8	1.2	0.1	0.5	0.6	2.2	0.3	1.7
LOC101927770	3.5	0.7	1.3	0.2	0.3	0.3	2.3	0.1	1.1
KIAA0196	2.9	1.1	1.2	0.1	0.6	0.5	1.5	0.3	1.4
ZSCAN30	2.8	1.0	1.6	0.1	0.4	0.5	1.9	0.2	1.2
ORC2	2.7	0.8	1.3	0.1	0.3	0.5	2.0	0.2	1.7
BTN3A2	2.6	0.9	1.3	0.1	0.6	0.5	2.3	0.2	1.1
MAP3K6	3.1	1.1	1.6	0.3	0.7	0.7	1.3	0.2	0.9
ABI3	1.7	0.6	1.0	0.4	0.5	0.6	2.6	0.5	1.7
MAP6D1	2.5	1.2	1.1	0.4	0.5	0.4	1.9	0.2	1.5
PPP1R3F	3.2	1.0	1.4	0.4	0.7	0.7	1.4	0.2	0.7
OSBPL7	2.4	1.4	1.8	0.4	0.7	0.9	1.0	0.2	0.9
LTBP2	2.8	0.9	1.4	0.4	0.6	1.0	1.4	0.2	1.0
HOXB3	2.1	1.4	1.2	0.2	0.8	0.7	1.2	0.8	1.5
FBXO30	2.5	0.8	1.1	0.1	0.2	0.5	2.3	0.3	1.7
P4HA3	3.2	0.6	1.4	0.1	0.3	0.4	2.4	0.1	1.1
ZGLP1	3.5	1.3	1.7	0.4	0.4	0.7	1.2	0.1	0.5

SMIM14	3.1	0.9	1.6	0.1	1.1	0.9	1.1	0.4	0.4
CDCA8	2.4	0.8	1.3	0.3	0.9	1.1	1.2	0.2	1.5
AKNA	2.6	1.0	1.5	0.2	0.5	0.7	1.4	0.3	1.4
KCNRG	2.7	0.5	1.7	0.1	0.2	0.5	2.2	0.3	1.5
SNX16	2.4	0.9	1.5	0.0	0.0	0.6	2.3	0.2	1.7
SNORD14A	1.3	2.5	1.9	0.2	0.4	1.2	0.6	0.3	1.2
IPPK	2.6	0.8	1.2	0.2	0.4	0.8	1.9	0.3	1.6
CEP128	2.4	1.0	1.2	0.2	0.4	0.4	2.0	0.3	1.8
NCOA1	3.2	1.0	1.4	0.2	0.5	0.7	1.1	0.3	1.3
ASMTL-AS1	3.0	0.5	1.5	0.2	0.5	0.5	2.2	0.2	1.0
ASMTL-AS1	3.0	0.5	1.5	0.2	0.5	0.5	2.2	0.2	1.0
LOC100288175	2.3	0.7	1.4	0.2	0.5	1.0	1.7	0.2	1.5
TBC1D31	2.8	1.1	1.5	0.1	0.4	0.5	1.9	0.2	1.3
ZNF337	3.1	0.8	1.4	0.1	0.5	0.5	1.8	0.3	1.3
LOC100131626	3.9	1.1	1.4	0.2	0.2	0.7	1.6	0.1	0.6
MR1	2.8	1.2	1.4	0.1	0.3	0.5	1.6	0.2	1.6
HM13-AS1	2.2	0.7	1.4	0.3	0.3	0.4	2.7	0.3	1.4
ZNF385A	3.1	1.3	1.3	0.5	0.3	0.6	1.4	0.2	1.0
LOC101927479	2.5	1.0	1.4	0.3	0.4	0.5	1.9	0.3	1.4
PARP12	2.7	0.8	1.2	0.2	0.6	0.8	1.6	0.2	1.5
MPP3	2.5	0.9	1.3	0.4	0.6	0.7	1.7	0.3	1.4
NEIL1	3.4	1.4	2.0	0.3	0.4	0.5	0.9	0.1	0.6
SLC25A17	2.6	1.3	1.8	0.1	0.4	0.6	1.6	0.1	1.2
LOC101928122	3.2	0.7	1.4	0.1	0.4	0.4	2.1	0.2	1.0
RMDN1	3.0	1.0	1.7	0.2	0.6	1.0	1.1	0.2	0.8
CEP192	2.7	0.9	1.4	0.1	0.4	0.6	1.9	0.2	1.4
RAMP1	2.6	1.5	1.6	0.3	0.8	0.9	0.8	0.4	0.8
APLF	3.4	1.0	1.8	0.2	0.5	0.8	1.0	0.3	0.8
SLC38A9	2.1	0.9	1.1	0.1	0.3	0.4	2.7	0.3	1.9
AADAT	2.6	0.9	1.5	0.1	0.5	0.5	1.7	0.3	1.5
LOC101927714	2.7	0.8	1.5	0.4	0.5	0.5	1.4	0.2	1.6
LOC100506023	3.4	0.6	1.4	0.1	0.4	0.5	1.8	0.1	1.2
ZNF747	2.0	0.8	1.3	0.4	0.8	1.1	1.9	0.2	1.0
CRABP2	2.3	1.1	1.5	0.1	0.7	0.1	1.9	0.3	1.7
ZNF529	3.1	0.8	1.4	0.1	0.5	0.5	1.8	0.3	1.1
VPS13D	3.0	0.9	1.3	0.1	0.4	0.7	1.9	0.2	1.2
KIF23	1.8	0.9	1.0	0.1	0.4	0.8	2.5	0.3	1.8
RSBN1L-AS1	2.1	0.9	1.1	0.1	0.3	0.6	2.8	0.3	1.6
WDR47	2.7	1.0	1.6	0.1	0.3	0.4	1.8	0.2	1.6
CXXC4	1.3	1.3	1.2	1.6	0.8	0.3	1.2	1.1	0.9
LOC257396	2.9	0.9	1.3	0.1	0.5	0.6	1.7	0.2	1.3
NAIP	3.2	1.0	1.6	0.1	0.4	0.6	1.5	0.2	1.0
FN3K	3.2	1.6	1.9	0.2	0.3	0.7	0.9	0.1	0.8
PIK3R1	2.2	0.8	0.9	0.2	0.5	0.7	2.0	0.3	2.1
DNAJC6	2.3	1.0	1.0	0.2	0.4	0.7	1.8	0.4	1.8
KLHL28	2.5	0.9	1.1	0.1	0.4	0.4	2.3	0.3	1.7
TGIF2LX	3.0	1.1	1.7	0.1	0.3	0.9	0.8	0.2	1.5
SH3BP5	2.7	0.7	1.1	0.1	0.3	0.5	2.2	0.4	1.5
LOC101928525	2.3	1.3	1.4	0.3	0.1	0.7	1.5	0.3	1.7
CDKN2B-AS1	3.5	0.5	1.4	0.1	0.3	0.4	2.3	0.1	1.0
SHPRH	2.9	0.8	1.3	0.1	0.4	0.5	2.1	0.2	1.3
C1orf53	2.5	1.2	1.3	0.6	0.8	0.5	1.3	0.2	1.1
LOC100132249	3.0	0.9	1.9	0.3	0.4	0.7	1.5	0.1	0.7
SKP2	2.8	1.0	1.3	0.1	0.2	0.4	2.2	0.3	1.3
MTERFD2	2.7	0.9	1.4	0.1	0.5	0.6	1.6	0.3	1.5

LOC101928629	2.3	0.5	0.9	0.8	0.7	1.1	1.8	0.3	1.2
TMEM254	2.3	1.0	1.8	0.2	0.4	0.7	1.3	0.2	1.6
KIN	2.4	0.8	1.3	0.1	0.2	0.5	2.6	0.1	1.5
DLX6-AS1	2.6	1.0	1.2	0.1	0.5	0.7	2.1	0.3	1.1
SFXN5	3.3	1.1	1.6	0.3	0.3	0.8	1.2	0.2	0.9
LOC100506123	3.2	0.5	1.4	0.2	0.4	0.6	1.7	0.2	1.4
LOC100506014	3.2	1.3	1.5	0.1	0.4	0.8	1.4	0.1	0.8
TNFRSF25	2.8	1.2	1.7	0.2	0.5	0.6	1.4	0.2	0.9
SMARCA2	2.7	0.8	1.2	0.2	0.5	0.8	1.7	0.3	1.2
USP43	2.7	1.0	1.5	0.2	0.5	0.7	1.6	0.3	1.0
CREB5	2.6	1.0	1.0	0.5	0.8	1.2	0.8	0.4	1.3
SLAIN1	2.6	1.3	1.5	0.2	0.5	0.6	1.3	0.3	1.2
ENTPD5	2.3	1.1	1.3	0.1	0.4	0.7	2.0	0.2	1.4
LINC00967	3.4	0.6	1.2	0.1	0.4	0.5	2.1	0.1	1.0
LETM2	2.0	0.9	0.9	0.2	0.3	0.5	2.3	0.5	2.1
ATRNL1	2.1	1.5	1.6	0.2	0.5	0.3	2.0	0.4	0.8
STXBP5	2.6	0.8	1.1	0.1	0.3	0.5	2.3	0.2	1.7
HCN3	2.6	1.2	1.4	0.3	0.7	0.7	1.5	0.2	0.9
ERCC6L2	2.7	1.0	1.3	0.1	0.5	0.6	1.7	0.2	1.3
MON2	2.8	0.7	1.2	0.1	0.4	0.6	2.1	0.2	1.4
EPC1	2.6	1.0	1.3	0.1	0.3	0.5	2.0	0.3	1.5
NKIRAS1	2.9	1.3	1.5	0.0	0.4	0.5	1.6	0.3	0.9
BCHE	2.8	1.1	1.6	0.1	0.3	0.4	1.6	0.2	1.3
ZNF577	3.2	1.0	1.7	0.2	0.8	0.4	1.1	0.1	0.9
SNORA59B	3.1	0.8	1.3	0.1	0.7	0.6	1.5	0.4	0.9
SNORA59A	3.1	0.8	1.3	0.1	0.7	0.6	1.5	0.4	0.9
FLJ32790	3.1	0.5	1.4	0.2	0.4	0.5	2.2	0.2	0.8
TRMT1L	3.4	1.1	1.4	0.2	0.5	0.7	1.2	0.2	0.8
TRIM62	2.8	0.8	1.2	0.3	0.3	0.8	1.8	0.3	1.1
DNAJC24	2.2	1.1	1.2	0.2	0.3	0.5	2.3	0.2	1.5
NRDE2	2.6	0.9	1.2	0.1	0.4	0.5	2.2	0.2	1.3
LOC100130899	3.1	0.6	1.2	0.2	0.4	0.4	2.4	0.2	1.0
LINC00869	2.1	0.9	1.5	0.3	0.4	0.5	2.0	0.4	1.5
LOC101929185	3.0	0.6	1.6	0.3	0.4	0.5	2.0	0.4	0.8
FUK	3.3	1.4	1.7	0.2	0.4	0.6	1.2	0.1	0.6
ATP10A	1.8	0.8	1.0	0.3	0.5	0.6	2.3	0.3	1.7
ACBD4	2.5	1.4	1.7	0.2	0.5	0.8	1.1	0.1	1.1
ATG2B	2.9	0.8	1.3	0.1	0.4	0.6	1.9	0.3	1.3
MAP9	2.5	0.9	1.4	0.1	0.3	0.5	2.1	0.2	1.4
C14orf93	2.5	1.1	1.0	0.4	0.6	0.7	1.5	0.4	1.4
ADPRM	2.3	1.1	1.1	0.2	0.4	0.8	1.7	0.2	1.7
AREG	2.7	1.2	1.6	0.0	0.1	0.3	2.0	0.1	1.3
NOXO1	3.2	1.1	1.6	0.4	0.4	0.4	1.1	0.3	0.9
ARSG	2.9	0.9	1.5	0.2	0.6	0.9	1.2	0.1	1.0
FUCA1	2.9	1.1	1.8	0.3	0.6	1.2	0.7	0.0	0.8
ASPHD1	2.3	0.9	1.3	0.4	0.4	1.0	1.9	0.1	1.1
POLK	3.3	1.0	1.0	0.1	0.3	0.8	1.5	0.2	1.3
LOC100293704	2.9	1.0	1.4	0.3	0.4	0.6	1.5	0.2	1.0
RPS6KA5	2.3	0.7	0.8	0.1	0.3	0.4	2.5	0.4	2.0
INO80B	0.9	2.3	1.6	0.5	0.0	1.3	0.5	0.9	1.3
ZFP1	3.0	1.0	0.9	0.1	0.2	0.5	1.7	0.2	1.7
FLVCR1	2.3	0.8	1.2	0.2	0.2	0.4	2.2	0.3	1.8
HOXB5	2.2	0.6	1.3	0.5	1.0	0.5	1.7	0.2	1.3
LOC101927910	3.3	1.0	1.9	0.2	0.2	0.3	1.6	0.1	0.8
LOC101929758	2.5	0.9	1.3	0.2	0.8	0.1	2.1	0.3	1.1

FLG	2.5	0.6	1.1	0.2	0.5	0.6	2.5	0.2	1.1
LOC101929668	2.2	1.0	1.4	0.5	0.5	1.0	1.2	0.4	1.2
GMIP	3.1	1.0	1.3	0.3	0.4	0.5	1.5	0.2	1.1
LOC101927400	3.4	0.8	1.2	0.1	0.7	0.3	1.6	0.2	0.9
SSX2IP	2.3	0.8	1.1	0.1	0.2	0.4	2.2	0.3	2.0
ME2	2.6	0.8	1.1	0.1	0.4	0.7	1.9	0.2	1.5
FLJ35282	3.1	0.6	1.5	0.1	0.5	0.5	1.8	0.2	1.0
TAF9B	2.5	0.7	1.1	0.1	0.8	0.6	1.5	0.3	1.8
POU2F1	2.9	0.8	1.3	0.2	0.5	0.6	1.7	0.2	1.1
C15orf41	2.8	0.7	1.2	0.1	0.6	0.7	1.6	0.2	1.4
GDF11	1.7	1.1	0.8	0.3	0.6	0.8	1.6	0.4	2.0
PLXNA2	1.8	0.6	1.0	0.2	0.4	0.5	2.5	0.4	2.1
EPB41L4B	2.9	1.1	1.6	0.2	0.5	0.6	1.0	0.2	1.1
LILRB1	3.1	0.5	1.1	0.2	0.4	0.5	2.4	0.2	1.0
R3HCC1L	2.2	0.9	1.7	0.1	0.4	0.3	1.7	0.2	1.8
LOC100288102	2.8	1.3	1.1	0.1	0.1	0.9	1.5	0.1	1.3
REV3L	2.9	0.9	1.2	0.1	0.4	0.5	1.7	0.2	1.3
GORAB	2.7	1.1	1.5	0.0	0.5	0.5	1.4	0.3	1.1
RAD51C	2.1	1.1	1.1	0.1	0.4	1.0	1.7	0.4	1.5
FDXACB1	3.0	0.7	1.1	0.1	0.4	0.6	1.9	0.1	1.3
FAM126A	2.3	0.8	1.1	0.1	0.4	0.7	1.6	0.3	2.1
C10orf137	2.5	0.9	1.2	0.1	0.4	0.6	2.0	0.2	1.4
PQLC3	3.1	1.3	1.5	0.4	0.6	0.7	0.8	0.2	0.8
RAB15	1.4	0.5	0.7	0.5	0.9	1.1	2.0	0.5	1.6
LOC645553	2.7	0.9	1.4	0.3	0.6	0.7	1.6	0.1	1.0
NUP210	2.9	1.1	1.4	0.2	0.4	0.6	1.3	0.2	1.2
ANKRD37	2.0	0.9	1.1	0.1	0.4	0.4	2.7	0.4	1.3
IKZF5	2.3	0.8	1.2	0.2	0.5	0.7	1.7	0.3	1.6
LOC101929645	3.3	0.6	1.4	0.2	0.5	0.4	1.6	0.2	1.0
C3orf62	2.5	0.9	1.3	0.3	0.3	0.6	1.8	0.1	1.5
ARHGAP11A	1.7	0.8	0.7	0.1	0.4	0.5	3.3	0.4	1.3
CCDC41	2.0	0.7	1.0	0.0	0.3	0.4	2.2	0.3	2.2
C7orf41	1.1	0.8	0.7	0.4	1.1	0.6	2.4	0.1	2.1
BCDIN3D-AS1	3.1	0.6	1.2	0.1	0.4	0.4	2.3	0.2	0.9
SLC38A4	2.8	1.0	1.4	0.0	0.5	0.4	1.5	0.2	1.3
KIAA0922	2.8	0.9	1.3	0.2	0.5	0.6	1.3	0.3	1.3
CLOCK	2.3	0.9	1.2	0.2	0.6	0.6	1.5	0.3	1.8
LOC101060153	0.7	0.9	0.6	0.2	0.5	0.1	4.3	0.7	1.3
ALG11	2.4	1.2	1.1	0.0	0.3	0.3	1.9	0.3	1.7
FZD5	2.2	0.9	0.9	0.3	0.6	0.6	1.6	0.3	1.8
LINC01003	2.9	0.9	1.7	0.3	0.7	0.5	1.5	0.2	0.6
EMB	3.0	1.0	1.0	0.2	0.3	0.6	1.4	0.2	1.5
LOC101929097	2.7	0.9	1.2	0.4	0.8	1.0	1.3	0.3	0.7
ZSCAN22	2.5	0.8	0.8	0.2	0.6	0.8	1.8	0.3	1.4
SNRK-AS1	2.6	1.0	1.2	0.1	0.6	0.5	1.8	0.2	1.2
RAB20	2.3	0.8	1.4	0.2	0.4	0.7	1.6	0.3	1.4
GPAM	2.8	1.1	1.6	0.1	0.4	0.5	1.6	0.2	1.0
PRKX	2.1	1.0	1.4	0.3	0.4	0.7	1.6	0.3	1.3
SLC16A1	3.3	1.1	1.4	0.1	0.2	0.5	1.3	0.2	1.0
TMEM218	2.6	1.1	1.5	0.1	0.4	0.6	1.4	0.1	1.4
MIOS	2.1	0.9	1.0	0.1	0.3	0.4	2.3	0.3	1.9
LOC101929693	2.4	1.1	1.1	0.3	0.4	0.7	1.8	0.4	1.0
ZNF275	2.4	0.6	1.1	0.3	0.5	0.6	2.1	0.2	1.4
ZNF445	2.7	0.7	1.0	0.1	0.4	0.4	1.9	0.2	1.7
KCNC4	1.9	0.8	0.9	0.4	0.5	0.7	2.0	0.3	1.7

DNAJC25	2.4	0.9	1.4	0.2	0.5	0.5	1.9	0.4	1.0
WDSUB1	2.8	1.1	1.1	0.1	0.2	0.4	1.9	0.2	1.6
ZNF343	2.0	0.9	1.2	0.1	0.5	0.5	2.2	0.3	1.4
CDH18	2.4	0.8	1.0	0.2	0.4	0.5	1.8	0.3	1.6
LOC101927642	2.4	0.9	1.9	0.2	0.9	1.1	1.0	0.1	0.6
ZNF211	2.4	1.1	1.6	0.2	0.4	0.6	1.5	0.2	1.1
FABP6	2.5	1.2	1.6	0.1	0.6	0.9	1.1	0.3	0.8
ELMOD2	2.9	0.9	1.6	0.0	0.2	0.4	1.4	0.2	1.6
LOC101928983	2.9	0.6	1.3	0.1	0.3	0.4	2.3	0.1	1.0
KRBOX4	3.0	0.7	0.7	0.2	0.4	0.7	2.8	0.1	0.5
TMCC3	3.7	1.2	1.4	0.3	0.5	0.9	0.5	0.1	0.5
SGK494	3.0	0.6	1.6	0.2	0.4	0.5	1.5	0.2	1.1
LOC645967	2.9	0.8	1.0	0.2	0.5	0.5	1.9	0.2	1.0
PLEKHF2	2.1	0.8	1.1	0.1	0.4	0.4	2.0	0.3	1.9
CSPG4	2.4	0.9	1.3	0.4	0.7	0.8	1.5	0.3	1.0
MOB3C	2.5	1.2	1.8	0.1	0.5	0.8	1.0	0.3	1.0
PPP1R3E	1.7	1.1	1.3	0.5	0.6	0.9	1.8	0.3	0.9
LOC150935	3.4	0.6	1.2	0.2	0.2	0.5	1.9	0.2	0.9
GOLGA7B	4.1	1.0	1.7	0.2	0.3	0.2	1.1	0.1	0.4
SYTL3	2.2	0.6	1.0	0.1	0.3	0.5	2.7	0.4	1.4
LOC100130744	2.9	0.8	1.4	0.2	0.5	0.5	1.6	0.2	1.0
EIF3J-AS1	1.9	0.9	1.2	0.1	0.5	0.7	2.3	0.2	1.4
CYB5D1	2.8	0.8	1.5	0.1	0.4	0.6	1.4	0.2	1.4
SYNE3	1.8	0.7	0.8	0.2	0.4	0.4	3.1	0.2	1.5
PIBF1	2.4	0.9	1.2	0.0	0.3	0.5	1.8	0.3	1.7
LOC101928551	3.3	0.6	1.3	0.1	0.3	0.4	2.1	0.1	1.0
BGLAP	2.4	0.6	1.4	0.1	0.7	0.7	1.4	0.3	1.4
NAIF1	2.2	1.0	1.0	0.3	0.7	0.8	1.5	0.3	1.3
INTS8	2.9	1.0	1.4	0.1	0.3	0.5	1.6	0.2	1.1
CNFN	3.3	1.4	1.1	0.3	0.5	0.5	1.3	0.2	0.5
ZNF667	2.8	0.6	1.2	0.1	0.4	0.5	2.1	0.2	1.2
TMPRSS15	2.7	0.9	1.5	0.2	0.3	0.5	1.5	0.2	1.3
ACTR6	2.3	0.9	1.3	0.1	0.3	0.3	2.3	0.2	1.4
LMBR1L	2.6	1.2	1.7	0.1	0.3	0.5	1.3	0.3	1.1
SRPX2	3.1	1.1	1.4	0.2	0.5	0.7	1.1	0.2	0.7
ZWILCH	1.9	0.8	1.2	0.0	0.3	0.5	2.2	0.2	1.9
NLGN4Y-AS1	3.3	0.6	1.3	0.2	0.3	0.2	2.1	0.1	0.9
LOC254896	3.1	0.9	1.5	0.3	0.5	0.6	1.1	0.2	0.9
CDCA7	2.0	0.7	1.0	0.1	0.6	0.5	2.2	0.3	1.6
PPP1R21	3.0	1.1	1.3	0.1	0.4	0.4	1.4	0.2	1.2
ERCC4	2.8	0.7	1.3	0.1	0.4	0.5	2.0	0.2	1.0
IQCG	3.4	1.2	1.2	0.1	0.7	0.5	1.1	0.1	0.6
SLX4	2.6	1.0	1.5	0.3	0.6	0.5	1.4	0.2	1.1
C22orf23	2.8	0.4	1.3	0.1	0.3	0.4	2.5	0.3	1.1
SPOPL	2.5	0.8	1.0	0.1	0.4	0.5	1.9	0.3	1.5
OAS1	3.3	1.3	1.6	0.1	0.5	0.6	0.8	0.2	0.4
B9D2	2.7	1.1	1.3	0.2	0.3	0.7	1.6	0.2	1.0
TDRP	2.8	1.0	1.8	0.0	0.5	0.4	1.5	0.2	0.9
ZBTB14	1.8	0.7	1.3	0.2	0.3	0.5	2.3	0.2	1.7
XRCC4	2.7	0.9	1.3	0.0	0.3	0.5	1.9	0.1	1.3
C10orf40	3.1	0.5	1.2	0.2	0.3	0.4	2.2	0.2	0.8
DHRS11	2.9	0.8	1.2	0.4	0.7	0.8	1.2	0.2	0.8
LOC101928388	3.0	0.6	1.2	0.2	0.4	0.3	2.3	0.2	1.0
LOC101927826	2.9	0.8	1.1	0.3	0.5	1.0	1.5	0.1	0.8
LOC644083	2.6	0.6	1.2	0.1	0.3	0.4	2.6	0.1	1.1

QRSL1		2.5	0.8	1.0	0.1	0.2	0.4	2.3	0.2	1.5
ZNF329		2.9	0.7	1.5	0.2	0.4	0.3	1.9	0.2	0.9
HEATR5B		2.5	0.7	1.2	0.1	0.3	0.4	2.1	0.2	1.4
LOC100131372		3.0	0.7	1.3	0.2	0.3	0.4	2.0	0.2	0.9
SERINC2		1.5	0.6	0.6	0.4	0.6	0.8	2.1	0.5	1.8
LOC101927752		2.3	1.3	1.2	0.4	0.6	0.5	1.6	0.3	0.8
SCAPER		2.3	0.8	1.1	0.2	0.4	0.8	1.6	0.3	1.6
TIPIN		2.1	1.1	1.0	0.1	0.4	0.8	1.7	0.2	1.6
PCCA		2.7	1.0	1.1	0.1	0.3	0.5	1.7	0.1	1.4
LOC101928138		2.7	0.5	1.2	0.2	0.4	0.6	2.2	0.1	0.9
LOC101928498		2.6	1.0	1.0	0.3	0.6	0.8	1.6	0.2	1.0
ZFP28		2.9	0.7	1.2	0.1	0.3	0.5	1.9	0.3	1.0
TRAPPC13		2.5	0.7	1.3	0.1	0.5	0.4	1.5	0.2	1.8
LOC100506639		2.6	0.8	1.2	0.2	0.4	0.7	1.4	0.2	1.5
SEC14L2		2.9	0.9	1.4	0.2	0.3	0.6	1.4	0.2	1.1
PDS5B		2.5	0.9	1.1	0.1	0.4	0.5	1.8	0.2	1.4
ZNF292		2.8	0.9	1.2	0.1	0.3	0.4	1.6	0.2	1.3
FUT10		2.4	0.7	1.0	0.1	0.4	0.5	1.9	0.3	1.5
LOC100287195		2.6	0.9	1.4	0.5	1.2	0.7	0.6	0.3	0.8
KIAA1656		2.9	0.7	1.3	0.2	0.3	0.4	2.1	0.2	0.9
STOML1		3.0	1.0	1.7	0.3	0.6	0.5	1.0	0.2	0.8
CFH		3.2	1.0	1.4	0.1	0.3	0.4	1.3	0.2	1.0
LOC101928855		2.7	0.6	1.3	0.2	0.3	0.4	2.3	0.2	0.9
ZCCHC4		2.4	0.9	1.1	0.1	0.4	0.3	2.2	0.2	1.3
FAT4		2.4	0.7	0.9	0.2	0.6	0.7	1.8	0.3	1.3
CORO7-PAM16		2.4	0.8	1.1	0.3	0.6	0.5	1.8	0.3	1.1
CD22		2.3	0.7	1.3	0.4	1.2	1.3	1.0	0.1	0.7
ZNF318		2.7	1.0	1.3	0.2	0.5	0.7	1.2	0.2	1.0
ZNF764		2.3	0.7	1.4	0.3	0.7	0.5	1.4	0.3	1.2
FARP2		2.5	0.9	1.3	0.3	0.4	0.7	1.4	0.2	1.2
DENND4A		2.6	0.8	1.1	0.1	0.4	0.5	1.7	0.2	1.5
GXYLT1		2.5	0.8	0.8	0.1	0.3	0.3	2.5	0.2	1.3
CDKN3		2.2	1.4	1.1	0.2	0.7	1.2	1.1	0.1	0.9
TMTC2		2.6	1.1	1.5	0.1	0.3	0.5	1.2	0.3	1.3
CYFIP2		2.8	1.0	1.3	0.2	0.4	0.5	1.3	0.2	1.1
ABCD1		2.9	1.0	1.4	0.3	0.4	0.8	1.0	0.2	0.8
WDHD1		1.8	0.7	0.7	0.1	0.3	0.4	2.6	0.3	2.0
LOC101929383		2.3	1.1	1.3	0.3	0.2	0.2	1.7	0.7	1.2
LOC101927881		3.3	0.5	1.4	0.2	0.5	0.3	1.9	0.1	0.8
PCDHGC3		1.7	0.9	0.8	0.1	0.2	0.3	1.9	0.1	2.8
WWTR1-AS1		2.9	0.8	1.2	0.1	0.2	0.5	2.0	0.2	1.0
	44621.0	2.6	1.2	1.4	0.2	0.5	0.5	1.4	0.3	0.9
FOXO6		2.8	0.6	0.9	0.2	0.4	0.4	2.0	0.4	1.2
LOC101927587		2.8	0.5	1.2	0.1	0.3	0.4	2.2	0.2	1.1
DONSON		2.0	0.8	1.4	0.2	0.5	0.5	1.8	0.1	1.4
TIAM1		2.0	0.8	1.1	0.2	0.5	0.6	2.0	0.2	1.5
NS3BP		2.3	0.9	1.4	0.4	0.4	0.6	1.6	0.2	1.2
AGAP5		2.5	1.0	1.6	0.3	0.5	0.6	1.4	0.2	0.9
DOCK10		2.9	1.0	1.5	0.1	0.4	0.4	1.3	0.2	1.1
UBXN8		2.2	1.1	1.6	0.1	0.3	0.4	1.8	0.3	1.1
NMI		2.4	0.8	1.3	0.0	0.3	0.4	1.9	0.2	1.5
MIPEP		3.1	1.1	1.2	0.1	0.3	0.6	1.4	0.1	0.9
EGFLAM-AS4		2.6	0.7	1.0	0.2	0.5	0.6	1.9	0.3	1.0
ZNF197		3.0	1.0	1.1	0.1	0.5	0.4	1.5	0.2	1.0
ZNF174		2.2	0.9	1.0	0.1	0.3	0.6	1.6	0.2	1.9

ETAA1	3.0	1.0	1.6	0.1	0.5	0.4	1.2	0.1	0.9
PIGM	2.2	0.8	0.8	0.2	0.6	0.9	1.8	0.4	1.2
LINC00997	2.8	0.6	1.2	0.2	0.4	0.5	2.0	0.1	1.1
HLA-DMA	2.7	1.2	1.4	0.3	0.7	0.6	1.0	0.2	0.8
COX18	2.5	1.2	1.6	0.2	0.3	0.7	1.3	0.2	0.9
CRYBG3	2.3	0.6	1.0	0.1	0.4	0.5	2.0	0.2	1.6
LOC100506282	2.4	0.6	1.4	0.3	0.6	0.5	1.9	0.2	1.0
NTNG2	2.6	1.4	1.5	0.4	0.7	1.0	0.6	0.2	0.4
C2orf74	2.8	0.7	1.0	0.1	0.3	0.4	1.8	0.2	1.5
LBX2-AS1	2.5	1.0	1.5	0.2	0.5	0.7	1.2	0.2	1.0
DCP1B	2.7	1.0	1.4	0.2	0.6	0.7	1.0	0.3	1.0
ZNF808	2.7	0.7	1.3	0.1	0.3	0.5	1.5	0.2	1.5
FAM110A	3.2	0.9	1.1	0.3	0.4	0.8	1.1	0.2	0.7
PPT2	1.4	0.4	0.8	0.1	0.0	0.3	3.4	0.6	1.8
LOC101929533	2.8	0.9	1.0	0.1	0.1	0.4	2.3	0.2	1.0
STX17	2.9	0.7	1.5	0.2	0.6	0.4	1.5	0.2	0.8
MUC15	2.9	0.9	1.2	0.1	0.6	0.5	1.5	0.2	0.9
CD37	3.2	1.0	1.6	0.1	0.3	0.5	1.0	0.2	0.8
DDX59	2.7	0.9	1.2	0.1	0.6	0.6	1.0	0.2	1.4
DNMT3B	2.6	1.0	1.4	0.3	0.5	0.8	1.2	0.2	0.7
TRNP1	2.1	0.6	1.2	0.3	0.3	0.7	2.3	0.2	1.1
NAT6	2.0	0.9	1.2	0.5	0.7	0.9	1.0	0.3	1.2
GAS2L3	2.4	0.9	1.3	0.1	0.4	0.5	1.5	0.1	1.5
CCDC132	2.2	0.8	1.4	0.1	0.4	0.5	1.8	0.2	1.5
RAB38	2.1	0.9	1.5	0.1	0.5	0.4	1.4	0.2	1.6
C11orf96	3.3	0.9	1.4	0.1	0.1	0.3	1.1	0.5	1.1
NCOA2	2.6	0.8	1.0	0.2	0.5	0.7	1.5	0.3	1.2
ZNF586	2.8	0.6	1.2	0.2	0.3	0.4	1.8	0.2	1.2
ZC3H10	2.4	0.9	1.4	0.1	0.5	0.9	1.1	0.2	1.3
RRM2B	3.0	1.3	1.4	0.2	0.2	0.2	1.3	0.2	0.9
NUTM2A	2.0	0.5	1.3	0.3	0.7	0.8	1.8	0.2	1.2
GPR132	1.9	0.8	0.9	0.4	0.6	0.5	2.3	0.3	1.1
LOC101928170	2.6	0.9	1.3	0.1	0.4	0.5	1.4	0.2	1.2
ZC2HC1A	2.5	1.2	1.5	0.1	0.3	0.5	1.1	0.2	1.3
RNF219	2.0	0.9	0.9	0.1	0.5	0.5	2.2	0.2	1.5
ATP11A-AS1	3.0	0.5	1.2	0.2	0.3	0.4	1.9	0.2	0.9
FRMD3	2.9	1.4	1.4	0.1	0.4	0.8	1.2	0.1	0.4
LOC101927623	2.6	0.4	1.1	0.1	0.4	0.4	2.3	0.2	1.2
CLGN	2.7	1.1	1.3	0.0	0.4	0.6	1.4	0.1	1.0
PITPNM2	2.7	0.9	1.4	0.3	0.5	0.6	1.1	0.2	0.9
FAM63A	2.6	1.0	1.1	0.2	0.6	0.9	1.0	0.3	1.1
PIGF	2.0	0.7	1.2	0.1	0.5	0.4	2.6	0.1	1.2
NEK11	2.2	0.9	1.5	0.1	0.5	0.4	1.7	0.3	1.1
C5orf42	2.6	0.7	1.2	0.1	0.4	0.4	1.9	0.2	1.3
DCLRE1C	2.5	0.7	1.4	0.2	0.4	0.6	1.5	0.2	1.2
ZNF587B	2.2	0.7	1.2	0.2	0.5	0.4	2.0	0.2	1.2
ZNF704	3.0	0.8	1.1	0.3	0.3	0.5	1.1	0.2	1.2
LOC101927832	2.7	0.9	1.0	0.1	0.5	0.4	1.8	0.2	1.0
XPA	1.6	0.7	1.2	0.1	0.5	0.5	2.4	0.2	1.4
FAM188A	2.6	0.8	1.4	0.1	0.4	0.6	1.4	0.1	1.1
C6orf211	2.8	1.0	1.4	0.1	0.2	0.4	1.3	0.2	1.2
UEVLD	2.4	1.1	1.6	0.1	0.4	0.4	1.2	0.2	1.1
NFATC4	2.6	1.2	1.7	0.1	0.5	0.4	1.0	0.2	0.9
LINC00667	2.6	0.9	1.0	0.2	0.6	0.7	1.4	0.2	1.2
LOC101927463	1.7	0.6	1.1	0.1	0.7	0.4	2.3	0.2	1.6

INSC	2.5	0.7	1.4	0.1	0.3	0.3	2.2	0.2	0.9
MPP2	2.2	1.0	1.2	0.3	0.6	0.5	1.6	0.2	1.0
SWI5	1.7	0.5	0.9	0.2	0.5	1.2	1.6	0.2	1.6
ADAT2	1.3	0.8	1.0	0.2	0.5	0.4	2.1	0.2	2.2
AFF3	1.6	0.6	1.0	0.5	0.6	1.2	1.4	0.3	1.4
PAQR8	2.4	0.8	1.5	0.3	0.7	0.9	1.1	0.1	0.8
IDI2	1.9	0.6	0.9	0.1	0.4	0.4	2.2	0.4	1.8
ZC4H2	2.4	0.8	1.4	0.2	0.4	0.5	1.4	0.1	1.4
VPS13A	2.2	0.7	1.1	0.1	0.3	0.4	2.1	0.2	1.5
FAM217B	2.0	0.8	1.5	0.1	0.4	0.5	1.3	0.5	1.5
TMEM234	2.5	0.9	1.0	0.2	0.4	0.4	2.0	0.2	1.0
CXorf38	2.6	1.1	1.3	0.1	0.3	0.5	1.4	0.1	0.9
PRX	3.2	1.3	1.1	0.3	0.3	0.7	0.9	0.2	0.6
ST5	2.5	1.0	1.1	0.2	0.3	0.8	1.3	0.3	1.1
STEAP1	2.6	0.8	1.5	0.1	0.5	0.7	1.2	0.2	1.1
LOC730227	2.7	0.6	1.2	0.1	0.3	0.4	2.1	0.2	0.9
TTC36	2.9	1.2	1.4	0.1	0.5	0.7	0.8	0.4	0.5
C2orf69	2.5	1.1	1.2	0.1	0.4	0.5	1.3	0.2	1.3
FLJ46836	3.0	0.7	1.2	0.1	0.3	0.3	1.9	0.1	1.1
FAM133B	2.4	0.8	1.3	0.0	0.5	0.3	1.8	0.1	1.4
CRYZL1	2.9	0.8	1.3	0.1	0.4	0.5	1.6	0.1	1.1
CHEK2	1.5	0.7	1.1	0.2	0.5	0.6	2.0	0.3	1.6
FIGNL1	2.2	0.9	1.3	0.1	0.3	0.3	1.6	0.2	1.7
TPMT	2.0	0.8	0.9	0.1	0.3	0.5	2.2	0.2	1.5
SCXA	2.6	0.9	1.8	0.6	0.5	0.5	0.7	0.3	0.5
SCXB	2.6	0.9	1.8	0.6	0.5	0.5	0.7	0.3	0.5
DTWD2	2.1	0.7	1.0	0.1	0.7	0.7	1.5	0.3	1.5
LOC100996255	3.5	0.9	1.7	0.2	0.4	0.4	1.1	0.1	0.3
CEP72	2.0	0.6	1.1	0.3	0.5	0.8	1.7	0.3	1.3
LOC101926997	3.0	0.6	1.1	0.2	0.2	0.4	2.1	0.1	1.0
FRAT1	1.9	0.8	1.1	0.4	0.4	0.6	1.6	0.3	1.4
HIST1H2BL	1.3	1.0	1.1	0.5	1.1	1.5	0.7	0.2	1.2
LOC101927348	3.5	0.7	0.9	0.1	0.4	0.4	1.7	0.1	0.8
GKAP1	2.0	1.2	1.2	0.1	0.3	0.2	2.2	0.2	1.2
IL1RAP	2.1	0.8	1.2	0.1	0.3	0.5	1.8	0.3	1.4
ZNF700	2.3	0.7	1.3	0.0	0.3	0.5	1.9	0.2	1.2
CCDC34	2.6	0.8	0.9	0.2	0.6	0.7	1.3	0.2	1.2
C11orf74	2.9	1.0	1.5	0.1	0.3	0.4	0.8	0.3	1.1
CACTIN-AS1	2.3	1.2	1.4	0.3	0.2	0.6	1.2	0.3	0.9
THRB	2.6	0.6	1.2	0.1	0.6	0.5	1.4	0.2	1.3
ZNF286B	2.1	0.6	1.1	0.0	0.2	0.4	2.1	0.2	1.7
COG6	2.7	0.9	1.1	0.1	0.3	0.4	1.7	0.1	1.2
TIFA	2.0	0.9	1.6	0.1	0.2	0.4	1.8	0.2	1.1
NOS3	2.7	0.6	1.1	0.2	0.3	0.4	2.1	0.2	0.8
KCNIP3	2.5	0.9	1.2	0.2	0.7	0.9	1.1	0.1	0.8
ITGB3	3.5	1.0	1.7	0.1	0.3	0.2	0.9	0.1	0.8
RPL36A-HNRNPH2	3.7	1.2	1.6	0.1	0.0	0.0	1.7	0.0	0.3
RIMS1	1.8	0.8	1.1	0.1	0.4	0.4	1.7	0.4	1.7
BTN2A2	1.9	0.9	1.3	0.2	0.3	0.6	1.5	0.4	1.2
CDK14	2.5	0.6	1.4	0.1	0.2	0.4	1.7	0.2	1.3
ERAP2	2.7	1.0	1.6	0.1	0.2	0.3	1.7	0.2	0.8
TMEM14A	2.0	0.8	1.7	0.1	0.2	0.4	1.8	0.4	1.1
PFKFB4	2.8	1.1	1.5	0.1	0.3	0.4	1.3	0.1	0.9
FAIM	2.4	0.8	1.2	0.1	0.5	0.7	2.0	0.3	0.5
LOC100862671	2.1	0.9	1.4	0.2	0.6	0.2	1.7	0.4	0.9

MOBP	2.6	0.6	1.2	0.2	0.3	0.4	2.2	0.1	0.8
MATN2	1.9	1.0	1.1	0.1	0.3	0.3	1.9	0.3	1.5
LINC00581	3.1	0.3	1.2	0.2	0.4	0.3	2.1	0.1	0.8
ICA1	2.7	1.1	1.7	0.1	0.7	0.6	0.8	0.1	0.7
ECHDC2	2.7	0.9	1.7	0.4	0.4	0.5	0.9	0.1	0.8
SNORA55	1.4	0.6	0.8	1.0	1.1	1.2	1.1	0.8	0.5
EME2	2.2	0.6	1.6	0.3	0.3	0.6	1.6	0.2	0.9
SYNJ2-IT1	3.1	0.5	1.0	0.2	0.4	0.4	1.7	0.2	0.9
KDELR3	2.7	0.7	1.2	0.1	0.2	0.4	1.8	0.3	1.0
B3GNT5	2.5	1.0	1.6	0.0	0.4	0.2	1.5	0.2	0.9
DAAM2	2.1	0.9	1.3	0.2	0.4	0.8	1.4	0.3	1.1
LOC101927371	2.4	1.0	1.2	0.1	0.3	0.4	1.5	0.3	1.3
POC5	2.1	0.9	1.2	0.1	0.5	0.5	1.5	0.3	1.4
FAHD2B	1.6	1.0	1.4	0.2	0.9	1.1	1.1	0.1	1.0
LOC100287082	1.9	1.0	0.8	0.1	0.3	0.3	2.3	0.3	1.3
ADAMTS15	1.5	0.7	0.9	0.8	1.5	1.8	0.6	0.1	0.5
ZNF749	2.0	0.6	1.0	0.1	0.3	0.4	1.9	0.1	1.9
METTL21A	2.3	1.0	1.1	0.2	0.3	0.5	1.6	0.2	1.2
OSBPL1A	2.3	0.9	1.3	0.2	0.7	0.9	1.2	0.2	0.8
FKBP5	3.0	0.6	1.2	0.1	0.6	0.8	0.8	0.2	1.0
IL32	2.7	1.1	1.4	0.0	0.5	0.4	1.4	0.3	0.7
TXNRD3	2.5	0.8	1.4	0.2	0.5	0.8	1.4	0.1	0.8
GBP3	1.6	1.2	1.0	0.2	0.9	0.8	1.4	0.3	1.0
UPK1A-AS1	3.0	0.6	1.1	0.2	0.5	0.3	1.9	0.1	0.8
LOC101927077	3.7	0.7	1.4	0.0	0.0	0.7	1.2	0.0	0.7
CYP1B1	2.1	0.7	1.0	0.7	1.0	1.5	0.8	0.1	0.5
RAD51B	2.2	0.8	1.1	0.1	0.4	0.4	2.0	0.2	1.2
CIRBP-AS1	2.5	1.1	1.7	0.2	0.4	0.4	1.3	0.2	0.6
HEATR3	2.6	0.9	1.1	0.1	0.3	0.4	1.7	0.2	1.0
AQP3	3.0	1.7	1.4	0.2	0.3	0.4	0.8	0.1	0.5
LOC100505776	2.8	0.9	1.5	0.2	0.5	0.4	1.3	0.1	0.7
LOC102723630	3.4	0.5	1.0	0.1	0.2	0.1	1.9	0.1	0.9
RTBDN	3.5	1.3	2.0	0.1	0.1	0.2	0.6	0.0	0.5
ABCB10	2.3	0.9	1.0	0.2	0.5	0.6	1.5	0.2	1.2
METTL21D	1.8	0.9	1.4	0.0	0.2	0.5	2.3	0.1	1.1
DGAT2	2.1	1.2	1.6	0.2	0.5	0.7	1.1	0.1	0.9
HAS2	2.1	0.8	0.9	0.2	0.6	0.7	1.4	0.1	1.4
CCR10	3.0	0.9	1.4	0.3	0.3	0.6	1.0	0.3	0.6
LOC101929211	2.1	1.1	1.2	0.2	0.3	0.3	2.0	0.4	0.9
SMIM3	2.3	0.5	1.2	0.2	0.3	0.4	1.3	0.0	2.1
JHDM1D-AS1	2.7	0.7	1.2	0.2	0.4	0.4	1.5	0.2	1.1
DLG3	2.9	0.9	1.5	0.2	0.4	0.4	1.0	0.1	0.8
LOC101929539	3.0	0.5	1.2	0.2	0.4	0.4	1.5	0.2	0.9
C20orf166-AS1	2.7	0.5	1.3	0.1	0.3	0.4	1.9	0.2	0.9
SLC25A13	2.5	0.8	1.3	0.1	0.4	0.5	1.4	0.1	1.2
FAM211A	2.6	0.7	1.2	0.2	0.4	0.5	1.5	0.2	1.0
PLEKHH1	3.1	0.8	1.6	0.2	0.4	0.4	0.9	0.2	0.7
ANGPTL2	3.1	1.6	1.6	0.2	0.6	0.7	0.2	0.0	0.2
RMI2	1.6	0.8	0.8	0.3	0.6	0.8	2.2	0.3	0.9
SDCBP2-AS1	2.7	0.6	1.2	0.1	0.4	0.3	1.6	0.1	1.2
LOC101927284	3.0	0.6	1.2	0.2	0.4	0.5	1.5	0.1	0.7
YTHDC2	2.5	0.9	1.2	0.1	0.3	0.4	1.4	0.1	1.2
CTF1	2.2	0.8	1.0	0.5	0.6	1.1	1.4	0.0	0.6
C4orf27	1.7	1.2	1.5	0.0	0.3	0.6	1.7	0.2	1.1
CYP27A1	2.8	1.3	1.4	0.2	0.5	0.4	0.9	0.1	0.6

IL17RD	2.9	0.8	0.8	0.2	0.2	0.5	1.5	0.3	1.0
XKR6	2.0	0.8	1.1	0.4	0.3	0.6	1.7	0.2	1.2
TRMT13	2.1	0.7	1.2	0.0	0.3	0.6	1.7	0.2	1.5
JPH1	2.0	0.6	0.8	0.2	0.3	0.6	1.9	0.3	1.6
CD302	3.5	0.8	1.6	0.1	0.5	0.4	0.6	0.1	0.6
TLR9	1.7	0.7	0.9	0.1	0.3	0.3	1.9	0.4	1.9
CELSR2	3.0	1.1	1.3	0.2	0.3	0.4	1.1	0.2	0.8
PPARGC1A	2.9	1.0	1.6	0.2	0.4	0.4	0.9	0.2	0.7
EFCAB11	2.2	0.8	1.8	0.0	0.5	0.2	1.7	0.1	1.0
OFD1	2.4	0.7	1.2	0.1	0.5	0.6	1.5	0.2	1.1
LOC101927934	3.0	0.5	1.2	0.3	0.3	0.3	1.6	0.1	0.9
LDB2	2.0	1.5	1.7	0.2	0.7	0.8	0.6	0.1	0.6
ATP9A	3.1	1.3	1.4	0.2	0.5	0.6	0.6	0.1	0.4
ANK1	2.8	1.0	1.3	0.2	0.5	0.6	1.0	0.2	0.8
OSGIN1	2.7	0.9	1.2	0.2	0.5	0.7	0.9	0.3	0.9
DENND1B	2.7	0.8	1.2	0.1	0.5	0.3	1.2	0.1	1.5
ZNF302	2.0	1.0	1.4	0.1	0.3	0.5	1.2	0.4	1.3
TMEM171	2.0	1.0	1.0	0.1	0.1	0.4	1.9	0.2	1.6
FBXO2	2.5	1.2	1.7	0.2	0.4	0.6	0.9	0.2	0.5
SMPD2	1.9	0.9	1.1	0.3	0.2	0.7	1.3	0.4	1.4
ABHD17B	2.3	0.5	1.1	0.1	0.1	0.3	1.7	0.1	2.0
ATHL1	1.8	1.0	1.4	0.4	0.8	1.1	0.8	0.2	0.7
NSUN6	2.2	0.8	1.2	0.1	0.4	0.5	1.7	0.2	1.1
BUB1	1.6	0.7	0.8	0.2	0.6	0.8	1.7	0.3	1.5
ZSCAN26	2.2	0.9	1.2	0.1	0.4	0.7	1.5	0.2	1.1
LOC101927356	2.9	0.6	1.2	0.1	0.3	0.4	1.4	0.2	1.0
RAD51	1.8	0.5	1.1	0.1	0.5	0.5	2.0	0.3	1.4
DCLRE1A	1.8	0.8	1.0	0.1	0.3	0.5	1.6	0.3	1.7
UFL1	2.8	0.9	1.1	0.1	0.3	0.5	1.4	0.2	1.1
EFNB2	2.8	1.0	0.8	0.4	0.5	0.7	0.7	0.3	0.9
ZNF436	2.3	0.9	1.1	0.1	0.3	0.3	1.5	0.2	1.5
ZFP62	2.2	0.8	1.0	0.1	0.6	0.6	1.4	0.2	1.2
LOC100130705	2.4	0.9	0.8	0.2	0.5	0.6	1.3	0.2	1.2
CCDC126	2.8	0.6	1.1	0.1	0.1	0.4	1.5	0.3	1.3
SAMD5	2.7	1.2	1.2	0.4	0.3	0.4	1.2	0.2	0.5
LINC00336	2.4	0.5	1.1	0.2	0.4	0.5	2.1	0.1	1.0
GATA3	2.1	0.5	0.5	0.5	0.6	0.7	1.4	0.5	1.3
MDM1	2.6	0.9	1.4	0.1	0.3	0.4	1.4	0.1	0.9
FBXO10	2.3	0.8	1.1	0.2	0.4	0.5	1.5	0.2	1.0
LINC00471	2.3	0.7	1.2	0.2	0.3	0.4	1.9	0.2	0.9
NNAT	2.6	0.6	1.1	0.5	0.6	0.4	0.7	0.3	1.4
IBA57	2.4	0.6	0.7	0.2	0.6	0.7	1.8	0.3	0.9
ZNF75A	2.3	1.0	1.3	0.2	0.5	0.6	1.3	0.1	0.9
LOC100134822	2.5	0.8	1.4	0.1	0.1	0.9	1.4	0.1	0.9
FLJ36777	2.6	0.5	1.2	0.1	0.4	0.3	2.1	0.1	0.7
LOC100507670	2.9	1.0	1.4	0.2	0.4	0.5	0.8	0.2	0.7
N6AMT1	3.0	0.7	1.2	0.2	0.3	0.4	1.1	0.2	0.9
CFD	1.4	1.1	1.2	0.3	0.4	0.7	1.5	0.5	1.1
SLPI	3.7	1.5	1.6	0.1	0.2	0.1	0.4	0.2	0.4
TNFRSF11A	1.8	1.1	1.2	0.2	0.6	0.6	1.1	0.3	1.2
MAGEE1	2.5	1.0	1.1	0.3	0.5	0.6	1.1	0.1	0.9
CYB5R2	1.8	0.6	1.3	0.2	0.5	0.9	1.3	0.2	1.4
DYNC2LI1	3.2	1.4	0.9	0.1	0.2	0.6	0.8	0.1	0.8
SMARCD3	2.4	0.9	1.2	0.3	0.7	0.4	1.2	0.2	0.8
ZBTB46	2.4	0.8	1.3	0.3	0.4	0.3	1.4	0.3	0.8

LOC101929052	3.0	0.5	1.5	0.1	0.3	0.6	1.3	0.1	0.9
TFAP2A	1.9	0.9	1.2	0.3	0.8	0.6	1.1	0.3	0.9
HACE1	1.7	0.8	1.0	0.1	0.3	0.4	2.0	0.2	1.6
FAM157C	3.0	0.6	1.4	0.3	0.4	0.3	1.2	0.1	0.8
NBPF12	2.4	0.8	1.2	0.1	0.3	0.4	1.4	0.2	1.3
SLC26A2	2.5	0.9	1.0	0.1	0.5	0.6	1.2	0.1	1.2
LOC101928844	2.0	0.4	1.1	0.2	0.5	0.5	1.5	0.4	1.6
LOC101928934	2.7	0.7	1.0	0.1	0.3	0.5	1.8	0.2	0.8
SUOX	2.0	0.8	1.3	0.2	0.5	0.6	1.6	0.2	0.9
SNORA72	1.8	0.7	0.9	0.2	1.1	0.6	1.2	0.2	1.4
CADM2-AS2	2.6	0.4	1.4	0.1	0.4	0.5	1.7	0.2	0.8
CHN1	1.8	0.6	0.8	0.1	0.2	0.3	2.2	0.3	1.8
MAGOHB	1.7	0.8	1.4	0.2	0.6	0.9	1.3	0.1	1.1
CADPS2	2.6	0.8	1.0	0.1	0.3	0.3	1.6	0.2	1.2
IL1RAPL1	2.3	0.8	1.0	0.1	0.3	0.5	1.4	0.2	1.4
CENPL	2.7	0.6	1.0	0.2	0.4	0.3	1.9	0.1	1.0
HELLS	1.7	0.5	0.6	0.1	0.3	0.7	2.1	0.3	1.9
DNASE1L1	1.9	0.8	1.5	0.5	0.6	0.7	1.1	0.3	0.6
FAM66B	2.9	1.0	1.3	0.1	0.2	0.5	1.2	0.1	0.8
HJURP	1.3	0.8	0.9	0.3	0.7	1.0	1.6	0.3	1.3
MTBP	2.1	0.7	1.2	0.0	0.5	0.5	1.7	0.1	1.2
RNF207	2.2	0.6	1.5	0.4	0.8	0.8	0.9	0.2	0.7
PARP16	2.9	0.9	1.3	0.1	0.1	0.6	0.9	0.3	0.9
METAP1D	2.7	0.8	1.5	0.1	0.6	0.5	0.9	0.1	0.8
C21orf91	2.2	0.8	1.2	0.1	0.3	0.5	1.4	0.2	1.4
DENND6A	2.6	0.8	1.3	0.1	0.5	0.5	1.3	0.1	0.9
CXorf61	1.9	1.6	1.0	0.1	0.3	0.5	1.4	0.2	1.0
CARD6	2.5	1.0	1.0	0.1	0.2	0.3	1.6	0.2	1.3
LOC100996485	2.0	0.6	1.2	0.2	0.0	0.5	2.7	0.0	0.7
WNT2B	2.8	0.7	1.1	0.3	0.5	0.4	1.1	0.4	0.8
DZIP3	2.8	1.1	1.3	0.1	0.4	0.7	0.7	0.1	0.8
ARHGAP11B	2.1	0.8	0.7	0.1	0.6	0.3	2.0	0.4	1.0
LIFR	2.8	0.9	1.4	0.0	0.3	0.3	1.2	0.1	1.0
DAPL1	2.9	0.4	1.1	0.3	0.2	0.6	1.6	0.1	0.8
CEP55	2.0	1.0	1.2	0.1	0.4	0.5	1.5	0.2	1.1
LOC100190940	2.4	0.4	0.8	0.3	0.5	0.6	1.6	0.3	1.0
LOC101927326	2.7	0.4	0.6	0.1	0.5	0.4	2.0	0.2	1.1
LOC101927373	2.4	1.0	1.3	0.1	0.4	0.5	0.8	0.2	1.4
CAPN10-AS1	2.7	0.6	1.3	0.2	0.3	0.4	1.5	0.1	0.9
ZNF70	1.8	0.6	1.3	0.3	0.5	0.8	1.1	0.4	1.3
MANBA	2.9	1.1	1.5	0.1	0.3	0.4	1.1	0.1	0.6
VEPH1	2.1	0.9	0.8	0.1	0.3	0.8	1.4	0.3	1.3
RLF	2.4	0.8	1.2	0.0	0.3	0.3	1.6	0.2	1.2
THAP6	1.2	0.8	0.9	0.1	0.6	0.3	1.9	0.2	1.8
SPDYE1	2.8	0.6	1.3	0.2	0.4	0.5	1.2	0.2	0.8
AR	2.6	1.0	1.2	0.2	0.4	0.6	1.0	0.2	0.8
MAGI1-IT1	2.9	0.5	1.1	0.1	0.4	0.4	1.8	0.1	0.8
VAC14-AS1	2.5	0.5	0.9	0.2	0.4	0.4	2.1	0.2	0.9
NXNL2	3.1	0.6	1.2	0.1	0.5	0.3	1.6	0.1	0.5
ZDHHC21	1.7	0.5	0.8	0.1	0.2	0.4	2.5	0.3	1.4
EPB41L5	2.5	0.7	1.4	0.1	0.3	0.5	1.6	0.1	0.9
GALNT18	1.4	0.6	0.9	0.4	0.4	0.7	1.8	0.3	1.4
LOC100216479	2.6	0.5	1.2	0.2	0.2	0.4	1.9	0.1	0.9
LOC729609	2.7	0.5	1.1	0.1	0.1	0.4	1.9	0.1	1.0
SORBS2	2.8	1.1	1.4	0.2	0.4	0.8	0.5	0.1	0.6

WDR62		1.5	0.7	0.9	0.3	0.6	0.7	1.7	0.4	1.2
LOC100288069		2.3	0.7	1.2	0.2	0.5	0.5	1.4	0.2	1.0
CECR7		2.4	0.7	1.1	0.3	0.6	0.6	1.3	0.2	0.8
ZNF841		2.6	0.7	1.3	0.1	0.5	0.4	1.2	0.2	1.0
ATG4A		2.1	0.8	1.5	0.0	0.2	0.6	1.3	0.1	1.1
LOC101928415		2.4	1.1	1.6	0.2	0.7	0.2	1.1	0.1	0.6
DTL		1.7	0.6	0.7	0.1	0.4	0.7	1.8	0.3	1.6
CLCN5		2.4	0.6	1.2	0.1	0.3	0.4	1.9	0.2	0.9
LOC101929636		2.9	0.6	1.2	0.2	0.3	0.3	1.5	0.1	0.8
DMTN		2.4	0.9	1.3	0.2	0.5	0.4	1.2	0.2	0.8
PALB2		2.5	0.8	1.1	0.1	0.3	0.7	1.2	0.2	0.9
ARAP2		2.0	0.7	1.2	0.0	0.4	0.4	1.6	0.3	1.3
LOC101927570		2.8	0.3	1.2	0.2	0.3	0.3	1.9	0.0	0.8
GPR65		1.7	0.6	0.5	0.0	0.2	0.2	2.4	0.3	1.8
MYL5		2.9	0.5	1.6	0.2	0.6	0.7	0.8	0.2	0.4
EPB41		1.8	0.6	0.9	0.1	0.3	0.4	1.8	0.3	1.7
ZNF2		1.9	0.6	1.2	0.1	0.4	0.4	1.7	0.3	1.3
USP37		2.0	0.8	1.1	0.1	0.2	0.3	2.0	0.3	1.2
C21orf58		2.6	0.6	0.9	0.2	0.4	0.5	1.7	0.1	0.8
HEMK1		2.0	0.7	1.3	0.3	0.5	1.0	1.1	0.1	0.9
SPC24		1.2	0.5	0.8	0.6	1.0	0.8	2.1	0.1	0.7
ERCC6		2.4	0.8	1.1	0.1	0.4	0.5	1.4	0.2	1.0
LOC101926955		2.5	0.6	1.1	0.1	0.3	0.4	1.9	0.1	0.9
MCM9		2.3	0.6	1.2	0.1	0.4	0.4	1.6	0.1	1.2
ORC4		2.2	0.7	1.3	0.0	0.2	0.3	1.5	0.3	1.4
DBF4		2.0	0.7	1.0	0.1	0.4	0.4	1.6	0.2	1.6
NBPF15		2.2	0.7	1.1	0.1	0.4	0.5	1.4	0.3	1.1
DISC2		2.7	0.5	1.0	0.1	0.3	0.3	1.8	0.1	0.8
DHRS12		2.6	1.0	1.4	0.3	0.3	0.9	0.8	0.1	0.5
LOC101927848		2.2	0.6	1.5	0.3	0.7	0.4	1.1	0.2	1.0
GPR157		2.3	0.8	1.0	0.2	0.4	0.5	1.3	0.3	1.1
GTPBP10		1.7	0.7	1.2	0.0	0.2	0.4	1.9	0.2	1.4
C7orf61		2.8	0.9	1.0	0.1	0.1	0.1	2.1	0.1	0.8
FAM155A		1.9	0.5	0.8	0.3	0.4	0.7	1.7	0.2	1.2
KIF27		2.5	0.6	1.2	0.1	0.3	0.5	1.4	0.2	1.1
CLUAP1		1.7	0.9	0.7	0.1	0.4	0.6	1.7	0.2	1.4
LOC645513		2.4	0.7	1.1	0.2	0.4	0.4	1.5	0.1	0.9
MANEA		2.3	1.0	1.3	0.0	0.4	0.6	1.0	0.1	1.1
FAM117B		1.8	0.9	1.1	0.2	0.5	0.8	1.2	0.2	1.1
C1QTNF6		2.1	1.1	1.2	0.3	0.8	0.8	0.9	0.1	0.3
RHOBTB1		2.3	1.0	1.2	0.1	0.3	0.3	1.4	0.2	1.0
DHX57		2.5	0.8	1.1	0.1	0.3	0.5	1.4	0.1	0.9
DKFZP434L187		2.5	0.6	1.1	0.1	0.3	0.3	2.0	0.2	0.8
PTPN18		2.4	0.8	1.0	0.4	0.6	0.7	1.1	0.2	0.7
	44623.0	1.5	0.6	1.1	0.2	0.2	0.3	2.1	0.4	1.3
FAM175A		2.0	0.9	1.0	0.2	0.3	0.6	1.2	0.2	1.4
TTC32		2.1	1.0	1.3	0.2	0.2	0.3	1.3	0.4	1.0
LOC101928706		2.2	0.7	1.6	0.8	1.0	0.4	0.6	0.0	0.6
RABL5		2.7	0.8	1.2	0.2	0.5	0.6	0.8	0.1	0.9
PHF16		1.6	0.6	1.0	0.1	0.3	0.4	1.9	0.2	1.6
C5orf27		2.6	0.7	1.1	0.2	0.4	0.3	1.4	0.1	0.9
FAM160A1		2.0	0.6	0.9	0.1	0.2	0.3	2.0	0.4	1.3
ZCCHC18		1.5	0.6	0.9	0.1	0.4	0.4	2.2	0.2	1.4
ACVR2A		2.4	0.8	1.3	0.1	0.3	0.2	1.2	0.1	1.2
ZBED5		2.3	0.8	1.0	0.0	0.3	0.4	1.5	0.1	1.3

LHB	2.4	0.8	1.1	0.3	0.5	0.7	1.1	0.3	0.6
CTSS	2.3	0.8	1.5	0.1	0.4	0.6	1.1	0.1	0.8
PPP3CC	2.3	0.8	0.6	0.1	0.3	0.4	1.9	0.2	1.1
C20orf194	2.6	0.8	1.2	0.1	0.3	0.5	1.3	0.2	0.9
C4orf22	2.7	0.6	1.0	0.1	0.3	0.5	1.6	0.2	0.8
LOC101060060	2.1	0.7	0.9	0.2	0.9	0.9	0.7	0.2	1.1
RBFOX3	2.8	1.2	1.1	0.2	0.3	0.3	0.6	0.3	1.0
LOC101928550	2.6	0.5	1.3	0.1	0.4	0.4	1.4	0.2	0.9
SENP1	2.2	0.8	1.0	0.1	0.3	0.5	1.3	0.2	1.3
TRG-AS1	2.7	0.6	1.2	0.1	0.2	0.2	1.8	0.1	0.7
NPFF	3.4	0.9	1.6	0.1	0.1	0.2	0.4	0.3	0.7
APOL1	3.0	0.9	1.4	0.1	0.5	0.6	0.8	0.2	0.4
CEP78	1.9	0.7	0.9	0.2	0.3	0.5	1.8	0.2	1.3
RWDD2B	1.5	0.7	0.8	0.1	0.5	0.8	2.0	0.1	1.3
EN2	1.4	0.3	0.5	0.3	0.3	0.6	2.2	0.6	1.5
VMAC	2.7	1.0	0.7	0.2	0.6	0.7	1.5	0.1	0.3
FAM185A	2.4	0.6	1.0	0.2	0.8	0.5	1.4	0.2	0.7
DHRSX	2.1	0.9	1.2	0.2	0.4	0.6	1.4	0.2	0.7
DHRSX	2.1	0.9	1.2	0.2	0.4	0.6	1.4	0.2	0.7
NDUFAF7	1.7	0.9	1.0	0.1	0.2	0.5	1.7	0.2	1.5
SUV39H1	1.7	0.7	0.8	0.3	0.6	0.5	1.8	0.3	1.0
IFI27L1	2.5	0.9	2.1	0.1	0.3	0.5	0.6	0.3	0.4
MFSD9	1.8	0.7	1.1	0.2	0.2	0.5	1.7	0.2	1.3
CT64	2.7	0.6	1.1	0.1	0.4	0.4	1.2	0.0	1.1
SOGA3	2.3	0.8	1.1	0.3	0.4	0.5	1.1	0.2	1.0
LINC00999	2.3	0.6	1.1	0.2	0.5	0.6	1.4	0.2	0.8
ZNF827	2.1	0.7	1.0	0.2	0.4	0.5	1.4	0.3	1.1
LOC100505767	2.4	0.5	1.1	0.3	0.6	0.7	1.1	0.2	0.7
POLA2	1.7	0.7	0.9	0.1	0.3	0.5	1.7	0.3	1.4
CCNF	2.2	0.8	0.6	0.3	0.5	0.9	1.3	0.1	0.8
C11orf92	2.4	0.5	1.1	0.2	0.3	0.3	1.9	0.1	0.9
SEC22A	1.9	0.9	1.7	0.0	0.2	0.4	1.0	0.3	1.3
FUT1	1.8	0.8	1.0	0.1	0.2	0.2	2.0	0.3	1.3
ANKMY2	2.5	0.5	1.2	0.0	0.3	0.5	1.5	0.3	0.9
SP140L	2.8	0.9	1.2	0.0	0.2	0.5	1.1	0.1	1.0
DENND5B-AS1	2.6	0.6	1.6	0.1	0.4	0.2	0.9	0.1	1.2
LOC441242	1.8	0.7	1.3	0.1	0.3	0.5	1.7	0.3	1.0
LOC101928728	2.9	0.6	1.0	0.3	0.3	0.2	1.5	0.2	0.7
LOC283683	2.4	0.5	1.2	0.2	0.3	0.3	1.9	0.1	0.8
PRDM5	2.7	0.8	1.0	0.1	0.2	0.4	1.1	0.2	1.1
LIMS2	1.9	0.8	1.1	0.2	0.3	0.4	1.9	0.2	1.0
GPR173	2.2	0.6	1.0	0.3	0.7	0.5	1.4	0.1	0.7
CDAN1	2.0	0.6	1.1	0.2	0.4	0.5	1.7	0.2	1.0
SLC24A1	2.0	0.6	0.9	0.1	0.5	0.7	1.3	0.2	1.3
GBP1	2.4	0.9	1.2	0.2	0.6	0.7	0.9	0.1	0.7
NXT2	2.3	0.8	1.4	0.1	0.6	0.5	1.0	0.1	0.9
PKIA	2.1	0.6	1.4	0.2	0.6	0.6	0.6	0.1	1.4
ZNF691	2.1	0.9	0.7	0.3	0.4	0.8	1.0	0.1	1.3
LOC100507071	2.3	1.0	1.2	0.0	0.3	0.4	1.2	0.2	1.0
IQGAP3	1.3	0.6	0.9	0.3	0.9	1.1	1.3	0.3	1.0
MTHFR	2.3	1.0	1.2	0.2	0.4	0.5	1.1	0.2	0.8
ZNF213	2.2	0.9	1.2	0.3	0.6	0.7	1.1	0.2	0.5
FAM64A	1.7	0.6	0.5	0.4	0.7	0.8	1.7	0.3	0.9
LOC338817	2.4	0.6	1.2	0.2	0.4	0.4	1.3	0.2	0.9
NKX2-3	0.3	1.9	0.8	0.6	0.6	0.4	1.2	0.7	1.0

SLC43A2	3.4	1.0	1.5	0.2	0.3	0.3	0.5	0.1	0.4
TMEM9B-AS1	1.9	1.1	1.4	0.2	0.3	0.6	1.4	0.0	0.7
FLJ14186	2.2	0.7	1.3	0.3	0.6	0.7	0.8	0.2	0.8
LOC101928214	2.0	0.5	0.6	0.1	0.6	0.3	2.4	0.1	1.0
CCZ1B	1.1	0.7	0.9	0.4	1.6	0.4	0.7	0.6	1.2
SMIM19	2.7	0.4	1.5	0.1	0.5	0.6	0.7	0.1	0.8
PHLPP2	2.4	0.7	1.0	0.1	0.2	0.4	1.5	0.2	1.1
SETD4	2.0	0.8	1.0	0.2	0.3	0.3	1.8	0.1	1.0
ZNF91	2.3	0.8	1.1	0.1	0.3	0.4	1.3	0.2	1.0
SLC35B3	1.8	0.7	1.2	0.1	0.3	0.3	1.6	0.2	1.5
NAAA	2.2	0.7	0.7	0.2	0.7	0.5	1.5	0.2	0.8
PRRT3	2.4	0.8	1.0	0.3	0.4	0.6	1.1	0.1	0.7
SLC35E4	2.2	0.8	1.1	0.3	0.5	0.7	1.0	0.3	0.7
COX20	2.3	0.7	1.0	0.0	0.0	0.6	1.5	0.2	1.2
PPP2R3C	2.1	0.6	1.1	0.0	0.2	0.5	1.7	0.1	1.2
UNC13B	2.1	0.6	0.8	0.1	0.3	0.5	1.6	0.2	1.3
LOC339975	2.8	0.7	1.0	0.2	0.4	0.4	1.3	0.1	0.6
OXSM	2.1	0.7	1.2	0.2	0.3	0.3	1.4	0.1	1.2
LOC101928196	2.7	0.5	1.1	0.0	0.2	0.2	2.2	0.1	0.6
ALG14	2.3	0.5	1.3	0.1	0.3	0.4	1.4	0.2	1.1
VDR	2.2	0.8	1.2	0.3	0.5	0.4	1.0	0.2	1.0
LINC00605	2.6	0.4	0.9	0.1	0.3	0.4	1.9	0.1	0.7
LOC101929204	2.2	0.6	1.3	0.2	0.6	0.3	1.5	0.1	0.6
PPAPDC2	2.2	0.8	1.4	0.3	0.3	0.7	1.1	0.0	0.7
ZSWIM3	2.3	0.7	1.2	0.1	0.4	0.6	1.1	0.2	1.0
TRAF5	1.8	0.9	1.1	0.1	0.5	0.6	1.3	0.1	1.1
TMEM194B	2.0	0.5	1.1	0.1	0.3	0.5	1.9	0.2	0.8
CDKL1	2.2	0.4	1.0	0.1	0.3	0.5	1.8	0.2	1.1
TRIM68	2.0	0.6	1.2	0.1	0.2	0.4	1.7	0.3	1.1
MYH7B	2.2	0.6	1.2	0.3	0.4	0.4	1.2	0.2	1.0
NDUFAF5	1.8	0.8	1.3	0.0	0.2	0.3	1.9	0.2	1.1
HEATR5A	2.4	0.7	1.0	0.0	0.2	0.3	1.7	0.2	1.0
LOC101927368	2.2	0.6	1.0	0.1	0.3	0.3	1.7	0.2	1.1
POU6F1	2.5	0.8	1.1	0.2	0.4	0.5	1.2	0.1	0.7
ZSCAN32	1.6	0.7	1.0	0.1	0.3	0.5	1.7	0.3	1.4
BTBD19	2.2	0.6	1.3	0.4	0.3	0.4	1.2	0.2	1.1
LPIN3	2.6	0.9	1.6	0.2	0.4	0.5	0.5	0.1	0.7
LOC101927666	2.3	0.5	1.2	0.1	0.3	0.3	1.8	0.1	0.8
FAM86B1	2.0	0.6	0.8	0.5	0.8	1.1	1.0	0.1	0.7
AKAP9	2.0	0.6	1.0	0.1	0.3	0.4	1.7	0.2	1.3
LOC101929025	2.5	0.6	1.2	0.1	0.2	0.3	1.8	0.1	0.7
LRRC40	2.4	0.9	1.1	0.0	0.4	0.4	1.1	0.2	1.0
C4orf29	2.2	1.0	0.9	0.0	0.1	0.4	1.5	0.1	1.3
KNTC1	2.0	0.5	0.9	0.1	0.3	0.4	1.8	0.2	1.2
HOXC10	1.9	1.1	1.3	0.4	0.3	0.7	1.0	0.2	0.6
ALS2CL	1.8	0.8	1.1	0.2	0.4	0.5	1.5	0.2	1.0
C17orf49	1.7	0.7	1.2	0.0	0.3	0.7	1.3	0.4	1.1
LOC101928884	1.6	0.5	0.9	0.1	0.2	0.2	2.8	0.1	1.1
DNASE1L2	2.5	0.8	1.2	0.2	0.4	0.4	1.0	0.1	0.7
PAQR6	1.8	0.7	1.5	0.1	0.6	0.6	1.1	0.2	0.8
LOC101927009	2.3	0.4	1.0	0.1	0.3	0.3	2.0	0.2	0.9
GSN-AS1	2.5	0.5	1.2	0.1	0.3	0.3	1.6	0.1	0.8
RANBP6	2.0	0.5	0.8	0.0	0.4	0.2	1.9	0.1	1.4
LOC100653005	2.6	0.5	1.1	0.1	0.3	0.4	1.4	0.1	1.0
SDSL	1.7	1.2	0.9	0.4	0.6	0.7	1.1	0.2	0.6

SNORA2A	1.6	1.2	1.2	0.2	0.8	0.0	1.6	0.2	0.7
PLCXD2	1.3	0.3	0.9	0.2	0.3	0.5	2.2	0.4	1.4
KIAA1731	2.2	0.7	1.1	0.1	0.3	0.5	1.2	0.2	1.1
FAM46C	2.2	0.9	1.1	0.2	0.2	0.5	1.0	0.3	1.1
LOC730183	2.5	0.7	1.2	0.1	0.4	0.4	1.3	0.0	0.8
ELFN2	1.5	0.9	1.0	0.4	0.7	1.0	0.8	0.3	0.9
RFX3	1.9	0.7	1.0	0.0	0.3	0.2	1.9	0.3	1.2
MMP24	2.0	0.7	1.1	0.2	0.3	0.3	1.6	0.2	1.0
INPP5B	2.4	0.8	1.0	0.1	0.2	0.4	1.3	0.1	1.0
LOC101928660	2.3	0.7	1.0	0.2	0.4	0.5	1.0	0.2	1.3
NFE4	1.7	0.6	1.4	0.1	0.3	0.3	1.7	0.2	1.2
TCTEX1D2	1.3	1.0	1.7	0.2	0.2	0.7	1.3	0.1	0.8
LOC101927056	2.3	0.4	1.1	0.2	0.3	0.4	1.7	0.1	0.9
LOC101927115	2.6	0.6	0.9	0.1	0.3	0.3	1.7	0.1	0.8
LRIG3	2.4	0.8	0.8	0.1	0.2	0.4	1.5	0.1	1.0
LOC101929141	2.7	0.7	1.4	0.0	0.2	0.2	1.6	0.1	0.6
FAM46A	2.5	1.1	1.2	0.1	0.3	0.5	0.9	0.1	0.8
ZNRF2	2.0	0.5	0.8	0.4	0.3	0.6	1.9	0.1	0.8
LCMT2	1.3	0.6	0.7	0.2	0.4	0.5	2.0	0.3	1.4
LINC00641	2.2	0.6	1.3	0.1	0.3	0.3	1.4	0.2	1.0
KDM6A	2.0	0.7	1.0	0.1	0.3	0.5	1.3	0.2	1.2
FNDC8	2.0	0.6	0.7	0.2	0.2	0.5	1.5	0.2	1.3
LOC654841	2.5	0.5	1.0	0.1	0.2	0.2	2.0	0.1	0.8
ZNF654	2.1	0.9	1.0	0.1	0.3	0.3	1.5	0.2	1.1
LOC101927402	1.7	0.6	1.6	0.1	0.2	0.3	0.9	0.1	1.9
LOC101927902	2.1	0.5	1.2	0.2	0.5	0.6	1.4	0.1	0.7
PPP1R13B	2.2	0.8	1.1	0.2	0.3	0.6	1.1	0.3	0.9
CENPF	1.5	0.6	0.7	0.1	0.5	0.6	2.0	0.2	1.2
CDON	2.4	1.0	1.2	0.1	0.4	0.4	1.0	0.1	0.7
LINC00886	2.6	0.6	1.1	0.2	0.3	0.3	1.5	0.1	0.7
NEK8	2.5	1.0	1.5	0.2	0.4	0.5	0.5	0.3	0.6
LOC101927803	2.7	0.5	1.1	0.2	0.4	0.5	1.2	0.1	0.7
IRX5	2.0	0.9	0.9	0.3	0.4	0.7	0.9	0.4	0.9
C12orf5	1.8	0.6	1.1	0.1	0.1	0.2	1.7	0.3	1.4
ATRIP	2.1	0.9	0.8	0.2	0.3	0.6	1.3	0.2	0.9
PDGFD	2.1	0.8	0.8	0.0	0.1	0.2	1.8	0.2	1.2
NT5DC1	2.5	1.1	1.3	0.2	0.1	0.7	0.6	0.1	0.7
ZNF341	2.2	0.6	1.0	0.2	0.4	0.6	1.1	0.2	1.0
LOC101927631	2.9	0.7	1.0	0.3	0.3	0.3	1.0	0.1	0.7
TMTC4	2.0	0.9	1.1	0.1	0.4	0.6	1.3	0.2	0.7
CERS6-AS1	1.8	0.7	1.0	0.1	0.3	0.4	1.6	0.2	1.3
LOC101928086	1.5	0.5	1.3	0.2	0.2	0.5	1.7	0.2	1.1
RINT1	2.1	0.8	1.0	0.1	0.4	0.4	1.2	0.2	1.2
ZNF490	2.0	0.6	1.1	0.1	0.5	0.6	1.5	0.1	0.8
LAMTOR3	2.1	0.6	1.5	0.1	0.2	0.4	1.3	0.2	0.9
SMAD7	2.0	0.6	1.0	0.3	0.5	0.9	1.1	0.2	0.7
SDR42E1	2.4	0.7	1.0	0.1	0.4	0.5	1.1	0.1	1.2
LOC101928510	2.4	0.4	1.0	0.2	0.4	0.4	1.8	0.1	0.7
LOC101927970	2.1	0.4	0.6	0.1	0.6	0.6	1.7	0.1	1.0
KMT2E-AS1	2.4	0.7	1.2	0.2	0.4	0.3	1.3	0.2	0.7
APOLD1	2.0	0.6	1.1	0.4	0.5	0.8	1.0	0.1	0.8
NKX2-8	2.3	0.5	0.9	0.4	0.5	0.6	1.3	0.4	0.5
LRRC6	2.3	0.9	1.2	0.1	0.3	0.7	1.1	0.1	0.6
AGAP2	1.7	0.8	0.8	0.3	0.4	0.7	1.3	0.3	1.0
PAOX	2.5	0.9	1.3	0.2	0.3	0.7	0.8	0.1	0.5

STX11	1.5	0.7	0.6	0.2	0.1	0.4	1.9	0.3	1.4
SNTA1	2.2	1.2	1.2	0.2	0.4	0.5	0.9	0.1	0.6
C1orf56	1.8	0.9	0.9	0.2	0.3	0.5	1.4	0.2	1.0
ZNF138	2.0	0.6	1.2	0.1	0.2	0.5	1.3	0.2	1.2
TGDS	1.5	0.7	0.8	0.1	0.2	0.2	1.7	0.3	1.7
BHLHE41	2.2	0.5	1.0	0.5	0.5	0.7	1.0	0.2	0.7
TRNAU1AP	2.0	0.5	1.3	0.1	0.3	0.5	1.6	0.2	0.7
LOC101927598	2.3	0.4	1.0	0.2	0.3	0.4	1.7	0.1	0.8
CEP97	2.2	0.6	1.0	0.0	0.3	0.4	1.5	0.1	1.1
LOC101928353	2.1	0.6	1.2	0.2	0.4	0.4	1.5	0.2	0.7
EXO5	1.6	0.6	0.6	0.1	0.5	0.4	1.9	0.2	1.5
LOC101928786	2.2	0.5	1.1	0.1	0.3	0.3	1.7	0.1	0.9
KLHL15	1.8	0.5	0.8	0.1	0.4	0.4	1.7	0.1	1.3
LINC00331	2.5	0.5	1.3	0.1	0.4	0.2	1.1	0.2	1.0
SPATA24	2.2	0.8	1.7	0.1	0.3	0.4	0.8	0.2	0.6
LOC646736	2.7	0.4	1.2	0.3	0.3	0.2	1.3	0.1	0.7
HCFC2	1.9	0.7	1.2	0.1	0.3	0.5	1.3	0.2	1.1
SUN3	2.3	0.7	1.0	0.1	0.2	0.2	1.8	0.1	0.9
FAM220BP	1.6	0.8	1.1	0.3	0.5	0.4	1.1	0.3	1.2
DLK2	1.3	0.8	0.8	0.2	0.4	0.6	1.7	0.3	1.2
LOC100507032	2.4	0.6	0.9	0.1	0.3	0.4	1.3	0.1	1.1
ITPKB	2.1	0.6	1.0	0.2	0.4	0.5	1.3	0.2	0.9
FAM63B	1.8	0.6	1.0	0.2	0.4	0.7	1.4	0.2	0.9
C9orf96	2.0	0.5	1.1	0.2	0.4	0.3	1.6	0.2	0.9
KCNS3	2.4	0.8	1.2	0.1	0.5	0.6	0.9	0.2	0.6
ACSS1	2.0	0.8	1.3	0.2	0.3	0.8	1.0	0.1	0.6
NUTM2B	2.7	0.5	0.8	0.2	0.3	0.5	1.1	0.2	0.9
C7orf13	1.6	0.7	0.9	0.2	0.3	0.5	1.9	0.2	1.0
UBE3D	1.8	0.6	1.1	0.2	0.4	0.6	1.3	0.1	1.1
MTMR9	1.6	0.7	1.0	0.1	0.4	0.5	1.6	0.2	1.1
ZBTB49	1.8	0.7	0.9	0.1	0.2	0.4	1.7	0.2	1.2
CELF2	2.1	0.9	1.2	0.2	0.2	0.4	1.1	0.2	1.0
DGKA	2.1	0.5	1.2	0.1	0.3	0.4	1.4	0.2	1.0
CAMKK1	2.4	0.9	1.2	0.3	0.4	0.6	0.6	0.1	0.6
MBTPS2	2.6	0.8	1.0	0.1	0.3	0.4	1.2	0.1	0.6
ATAD2B	2.3	0.8	1.3	0.1	0.2	0.4	1.1	0.2	0.9
PGBD1	2.1	0.8	1.1	0.1	0.2	0.3	1.5	0.3	0.9
LINC00102	2.5	0.5	0.9	0.1	0.2	0.3	1.8	0.2	0.8
LINC00102	2.5	0.5	0.9	0.1	0.2	0.3	1.8	0.2	0.8
ARHGAP5-AS1	2.7	0.9	1.1	0.1	0.2	0.4	0.7	0.2	0.8
SPAG1	2.0	0.8	1.1	0.1	0.2	0.2	1.3	0.2	1.1
LOC101927575	2.7	0.5	1.0	0.1	0.2	0.4	1.5	0.2	0.8
LOC101928189	2.4	0.5	1.2	0.4	0.2	0.5	1.0	0.1	1.0
APOBEC3C	2.3	0.9	1.2	0.2	0.3	0.9	0.8	0.2	0.5
ZMYM5	2.0	0.9	1.4	0.0	0.3	0.1	1.8	0.1	0.6
CCRL2	2.1	0.9	1.0	0.1	0.6	0.6	0.8	0.1	0.9
PLAG1	2.3	0.7	0.8	0.1	0.2	0.5	1.2	0.2	1.2
LOC100505616	1.6	1.0	1.2	0.1	0.4	0.5	1.2	0.2	0.9
LOC553103	2.9	0.4	0.9	0.2	0.3	0.3	1.2	0.2	0.7
LYRM7	1.3	0.6	1.3	0.0	0.3	0.7	1.1	0.3	1.5
LOC101927681	2.7	0.8	1.1	0.2	0.1	0.3	1.4	0.2	0.5
LOC101928405	2.6	0.6	0.9	0.2	0.4	0.3	1.5	0.1	0.7
CLUHP3	2.1	0.8	1.2	0.2	0.5	0.6	0.8	0.1	0.8
LOC101927167	1.8	0.7	1.0	0.2	0.2	0.3	1.6	0.3	1.1
FUZ	1.7	1.1	0.8	0.3	0.4	0.5	1.0	0.3	1.2

ITGB4	2.2	1.0	1.0	0.4	0.7	0.9	0.5	0.1	0.4
LOC101929718	2.0	0.8	1.4	0.2	0.2	0.3	1.2	0.1	1.0
SENP3	1.3	0.8	0.7	0.6	0.6	1.0	0.4	0.5	1.3
PPP1R9A	2.3	0.7	1.0	0.1	0.1	0.3	1.4	0.1	1.1
LINC00639	2.3	0.8	1.1	0.2	0.5	0.6	0.8	0.2	0.7
MICU3	1.9	0.8	1.2	0.1	0.2	0.5	1.4	0.1	0.9
LOC101927187	2.1	0.8	1.0	0.2	0.1	0.5	1.4	0.2	0.9
PLA2G6	2.3	1.1	1.4	0.2	0.4	0.5	0.7	0.2	0.3
FLJ35934	2.4	0.4	0.9	0.1	0.2	0.3	1.9	0.2	0.9
LOC100652999	2.0	0.9	1.0	0.1	0.4	0.5	1.0	0.1	1.2
RBM48	2.6	0.7	0.9	0.0	0.2	0.5	0.9	0.1	1.2
LRP2BP	2.0	0.7	1.3	0.1	0.4	0.2	1.6	0.2	0.6
ZNF248	1.7	0.6	1.2	0.2	0.2	0.5	1.3	0.2	1.2
ZNF792	2.3	0.8	1.2	0.1	0.4	0.7	1.0	0.1	0.7
CASP8AP2	2.0	0.7	1.0	0.1	0.3	0.4	1.4	0.2	1.0
LOC100134368	2.2	0.5	1.0	0.1	0.3	0.5	1.4	0.1	0.9
PRICKLE4	1.7	0.8	1.1	0.1	0.6	0.5	1.1	0.2	0.9
TBCK	2.3	0.7	1.2	0.1	0.3	0.5	1.1	0.1	0.9
APH1B	2.1	0.7	1.2	0.1	0.4	0.6	0.8	0.2	1.1
LOC401320	2.4	0.7	1.2	0.1	0.2	0.5	1.3	0.1	0.6
EBP	2.1	0.9	1.1	0.2	0.4	0.7	1.1	0.0	0.5
TMEM200B	1.9	0.8	0.9	0.2	0.4	0.8	1.4	0.3	0.5
SNORD3D	0.8	0.2	1.0	0.0	0.1	0.0	4.9	0.0	0.1
ZNF775	2.0	0.8	0.9	0.4	0.3	0.7	1.1	0.1	0.7
WDR66	2.1	0.6	0.9	0.1	0.2	0.4	1.5	0.2	1.0
LOC100506422	2.7	0.4	0.9	0.2	0.6	0.3	1.3	0.1	0.6
CYP27B1	2.3	0.8	1.4	0.2	0.4	0.3	1.1	0.2	0.5
KLHL25	2.1	0.6	1.1	0.2	0.4	0.4	1.3	0.2	0.9
GCLM	1.9	0.8	0.9	0.3	0.6	0.9	0.7	0.1	1.0
ZNF175	1.9	0.7	0.8	0.1	0.3	0.5	1.3	0.2	1.2
LOC101928697	2.5	0.7	0.9	0.1	0.5	0.5	0.9	0.0	1.0
PAXIP1-AS2	1.9	0.9	1.4	0.1	0.5	0.5	1.0	0.1	0.7
GMDS-AS1	2.0	0.8	0.9	0.3	0.5	0.4	1.4	0.1	0.7
FAM27E3	1.8	0.6	0.9	0.3	0.3	0.4	1.2	0.3	1.3
LOC101928732	1.7	0.8	1.2	0.2	0.3	0.6	1.1	0.2	1.0
RPA3-AS1	2.1	0.8	1.2	0.1	0.4	0.5	0.9	0.1	0.9
TNFSF13	1.8	0.8	1.0	0.2	0.2	0.5	1.2	0.2	1.1
CDC7	1.7	0.6	1.0	0.1	0.2	0.3	1.6	0.1	1.4
STARD8	1.9	0.6	0.9	0.2	0.4	0.5	1.4	0.2	0.9
MYPN	1.6	0.5	0.8	0.1	0.2	0.3	1.4	0.3	1.7
HCG9	2.4	0.5	0.8	0.3	0.2	0.5	1.5	0.1	0.7
SEMA3E	1.6	0.5	0.9	0.1	0.3	0.4	1.7	0.2	1.3
HYAL3	1.7	0.4	0.9	0.3	0.2	0.7	1.4	0.3	1.3
GDPD1	1.7	0.8	1.0	0.1	0.3	0.1	1.4	0.3	1.5
JMJD7	2.2	0.7	0.8	0.1	0.4	0.6	1.3	0.0	0.8
TRIM3	1.7	0.8	1.1	0.2	0.5	0.4	1.3	0.2	0.7
FLJ43879	2.4	0.5	0.9	0.2	0.3	0.3	1.6	0.1	0.7
SPATA13	1.6	0.5	0.6	0.2	0.3	0.4	1.7	0.3	1.4
AMY2B	2.7	0.6	0.9	0.1	0.3	0.4	1.2	0.1	0.7
RNASE4	1.4	0.7	0.7	0.0	0.4	0.4	2.1	0.2	1.0
PCDHB2	2.1	0.7	1.0	0.2	0.4	0.4	1.2	0.3	0.7
MGC34034	2.2	0.4	0.8	0.2	0.4	0.3	1.8	0.1	0.9
C16orf46	2.2	0.5	1.0	0.2	0.4	0.3	1.4	0.2	0.8
VLDLR	1.7	0.7	0.6	0.1	0.2	0.2	1.9	0.3	1.3
ZNF141	1.7	0.6	0.9	0.0	0.2	0.4	1.6	0.2	1.2

ACCS	2.0	0.5	1.1	0.2	0.5	0.5	1.1	0.1	0.9
SNN	2.7	0.6	1.1	0.2	0.4	0.1	1.2	0.0	0.6
LOC100288798	1.8	0.8	0.9	0.2	0.3	0.7	1.0	0.2	1.3
LRRC1	1.9	0.7	1.0	0.1	0.4	0.4	1.4	0.2	1.1
ADAMTS16	1.9	0.6	0.8	0.2	0.5	0.6	1.4	0.2	0.8
DRD4	2.0	0.8	1.4	0.3	0.4	0.6	0.8	0.1	0.5
ADAP1	1.5	0.8	1.2	0.3	0.4	0.4	1.3	0.1	1.0
CMTM8	1.7	0.7	1.2	0.1	0.1	0.4	1.3	0.3	1.2
NLGN3	2.1	0.7	1.1	0.3	0.4	0.5	1.1	0.1	0.7
ZKSCAN3	2.6	0.7	0.8	0.2	0.4	0.3	1.1	0.1	0.7
LOC101928874	2.3	0.2	1.0	0.1	0.1	0.3	1.6	0.2	1.1
LOC440704	2.2	0.5	0.8	0.1	0.3	0.3	1.8	0.2	0.7
FAM230A	1.7	0.6	0.9	0.3	0.5	0.7	1.1	0.2	0.9
SP5	2.0	0.9	0.7	0.3	0.5	0.5	0.8	0.3	0.9
SIGLEC15	1.3	1.1	0.6	0.4	0.7	0.8	1.0	0.2	0.9
TYMP	1.9	0.7	1.0	0.4	0.5	0.6	1.0	0.2	0.6
MBNL3	1.8	0.8	0.8	0.2	0.4	0.5	1.2	0.2	1.0
CCDC61	2.0	0.6	0.9	0.3	0.5	0.9	0.8	0.2	0.7
GLCCI1	1.9	0.8	1.3	0.2	0.4	0.5	0.7	0.2	0.8
ZNF331	1.7	0.6	0.8	0.2	0.1	0.3	1.7	0.2	1.1
FAM211B	2.2	1.0	1.0	0.1	0.3	0.4	0.9	0.1	0.9
ZNF761	2.2	0.5	1.1	0.1	0.2	0.4	1.2	0.1	1.0
LOC101927625	2.3	0.9	1.2	0.2	0.2	0.4	1.0	0.2	0.6
CREB3L4	1.7	0.7	1.1	0.1	0.5	0.5	1.1	0.3	1.0
BHLHB9	1.7	0.5	0.8	0.1	0.5	0.5	1.7	0.2	1.0
PPM1M	2.1	0.6	0.9	0.1	0.5	0.4	1.5	0.2	0.7
GNPDA2	2.0	1.1	0.9	0.1	0.3	0.5	0.9	0.1	0.9
TTLL1	2.2	0.9	1.1	0.3	0.6	0.8	0.6	0.1	0.5
DYRK1B	1.8	0.6	0.8	0.4	0.6	0.7	1.2	0.2	0.8
LOC101927968	2.1	0.8	1.2	0.4	0.4	0.4	1.0	0.0	0.7
SARM1	1.9	0.6	0.9	0.3	0.4	0.5	1.3	0.2	0.8
FAM104B	1.8	0.4	1.1	0.2	0.2	0.6	1.2	0.2	1.4
HES6	2.2	0.8	1.5	0.2	0.3	0.3	1.0	0.1	0.6
MX1	2.2	0.9	1.2	0.2	0.4	0.4	0.8	0.2	0.5
ZMYM1	2.0	0.4	0.9	0.0	0.3	0.3	1.6	0.1	1.2
PTPDC1	1.9	0.5	1.0	0.1	0.3	0.4	1.4	0.3	1.1
ARV1	1.7	0.7	1.0	0.1	0.5	1.3	0.9	0.1	0.5
HHIP	1.0	0.5	0.5	0.0	0.2	0.2	2.3	0.2	2.0
ARNTL	1.7	0.5	1.0	0.0	0.4	0.3	1.5	0.2	1.2
EXD3	2.0	0.8	1.1	0.6	0.7	0.9	0.4	0.1	0.3
KIAA0232	2.1	0.6	1.0	0.1	0.2	0.4	1.3	0.2	1.0
NFKBID	2.4	0.6	1.0	0.1	0.5	0.2	1.2	0.1	0.8
ANAPC4	2.0	0.7	1.1	0.1	0.2	0.5	1.3	0.1	0.9
DENND6B	2.5	0.7	1.5	0.3	0.5	0.4	0.7	0.1	0.2
PI4K2B	2.2	0.7	1.1	0.2	0.4	0.5	1.2	0.0	0.7
FTH1P18	2.1	0.6	0.9	0.1	0.2	0.1	1.8	0.1	0.9
LINC00936	2.1	0.6	1.1	0.2	0.2	0.4	1.3	0.2	0.8
PCOLCE2	1.6	0.8	1.3	0.1	0.3	0.5	1.2	0.2	1.0
KIF9-AS1	2.5	0.5	0.8	0.1	0.3	0.4	1.4	0.1	0.8
RINL	1.5	0.9	1.0	0.1	0.4	0.4	1.3	0.2	0.9
LOC51145	2.6	0.4	0.8	0.1	0.2	0.2	1.6	0.2	0.8
ZNF417	2.0	0.5	0.9	0.1	0.2	0.5	1.5	0.1	1.1
POLR2J2	2.0	0.3	0.9	0.3	0.3	0.9	0.9	0.1	1.2
CCNB2	1.6	0.6	0.9	0.1	0.5	0.7	1.4	0.1	1.0
FZD1	1.8	0.9	1.1	0.2	0.2	0.4	1.3	0.2	0.9

SYNE2	1.8	0.7	0.8	0.1	0.4	0.3	1.4	0.3	1.1
HAUS6	1.7	0.6	0.9	0.0	0.4	0.4	1.4	0.2	1.3
DCAF17	2.1	0.8	1.0	0.1	0.4	0.5	1.0	0.2	0.8
LOC101928526	2.3	0.4	1.2	0.0	0.2	0.3	1.5	0.1	0.7
CACNG8	0.4	1.2	1.2	1.0	0.4	0.2	1.2	0.7	0.6
ID2	1.8	1.3	0.8	0.1	0.2	0.2	0.9	0.3	1.3
LOC100505771	2.0	0.7	1.3	0.2	0.6	0.6	0.9	0.1	0.6
AGMAT	1.5	0.4	0.8	0.1	0.4	0.4	1.5	0.2	1.5
PRSS53	1.7	0.8	1.3	0.3	0.2	0.6	1.0	0.3	0.8
LOC100996660	2.1	0.9	0.9	0.1	0.3	0.4	1.3	0.1	0.7
FGF17	2.3	0.8	0.9	0.2	0.2	0.3	0.9	0.2	1.1
TBC1D3	2.0	0.9	1.2	0.2	0.3	0.4	0.9	0.2	0.7
CAPS	2.5	0.8	0.9	0.2	0.6	0.5	0.7	0.2	0.4
PHTF1	2.0	0.7	0.9	0.1	0.3	0.5	1.3	0.2	0.9
STS	2.0	0.7	1.1	0.1	0.4	0.7	0.7	0.2	0.8
AMIGO1	1.5	0.7	0.9	0.3	0.4	0.7	1.2	0.1	1.1
WRN	1.8	0.5	0.7	0.1	0.2	0.4	1.6	0.2	1.3
POLH	1.8	0.5	0.8	0.1	0.4	0.4	1.5	0.2	1.2
ULBP3	1.4	0.7	1.0	0.1	0.2	0.5	1.6	0.2	1.1
THSD1	2.5	0.6	1.1	0.1	0.3	0.2	0.9	0.1	0.9
FAM69A	1.8	0.9	1.0	0.0	0.3	0.2	1.2	0.1	1.3
HMGCL	1.8	0.8	0.8	0.2	0.5	0.8	0.9	0.2	0.8
IFT74	1.9	0.7	1.0	0.1	0.4	0.3	1.2	0.1	1.1
FLJ36848	2.1	0.8	1.4	0.1	0.2	0.3	0.9	0.2	0.8
RNF141	2.0	0.9	0.8	0.1	0.4	0.6	1.0	0.1	0.8
USPL1	2.3	0.8	0.9	0.0	0.2	0.2	1.3	0.2	0.9
GDF1	1.8	0.6	0.9	0.5	0.4	0.5	1.3	0.2	0.6
HOXC5	1.9	0.6	0.8	0.2	0.2	0.5	1.5	0.3	0.9
TESK2	2.1	0.8	1.0	0.1	0.4	0.3	1.1	0.1	0.9
BCAP29	1.5	0.5	0.7	0.0	0.4	0.3	1.7	0.2	1.4
NBEA	1.9	0.6	1.0	0.1	0.3	0.4	1.3	0.2	1.1
PHKG1	1.8	0.3	1.4	0.1	0.5	0.4	1.4	0.2	0.8
BICD1	1.6	0.6	0.9	0.2	0.4	0.5	1.2	0.2	1.1
TDRKH	1.8	0.4	0.7	0.0	0.2	0.4	1.6	0.3	1.3
LOC101926969	2.0	0.6	0.9	0.1	0.4	0.6	1.3	0.1	0.7
TARBP1	2.5	0.8	1.1	0.1	0.2	0.4	1.0	0.1	0.7
ARHGAP6	1.8	0.6	0.7	0.2	0.3	0.6	1.4	0.3	0.8
HPS5	1.9	0.5	0.8	0.0	0.3	0.4	1.8	0.1	0.9
NRXN3	2.0	0.4	0.9	0.1	0.3	0.3	1.6	0.2	0.8
C16orf59	1.4	0.6	0.8	0.2	0.5	0.6	1.2	0.4	1.0
RPL34-AS1	2.3	0.4	0.9	0.1	0.3	0.3	1.8	0.1	0.7
EML6	1.7	0.6	0.9	0.1	0.4	0.3	1.4	0.2	1.1
LINC00861	2.1	0.5	1.0	0.1	0.2	0.3	1.7	0.1	0.7
ZNF80	2.2	0.5	0.8	0.2	0.4	0.4	1.1	0.1	1.0
LINC00852	2.6	0.5	0.9	0.1	0.3	0.3	1.2	0.1	0.7
LRP5L	2.7	0.4	1.5	0.2	0.4	0.4	0.6	0.0	0.4
SKA3	1.3	0.7	0.7	0.1	0.3	0.5	1.6	0.3	1.4
BRICD5	1.8	0.6	1.2	0.2	0.2	0.5	1.1	0.2	1.0
EID2B	1.9	0.6	1.1	0.2	0.3	0.3	1.9	0.1	0.4
LOC100652768	1.8	0.8	0.8	0.2	0.4	0.5	1.2	0.1	0.9
LINC00623	1.6	0.7	0.5	0.2	0.1	0.5	1.5	0.0	1.7
LIMS3L	2.2	0.6	1.0	0.1	0.3	0.3	1.4	0.2	0.6
DKFZp686K1684	2.4	0.4	0.9	0.1	0.3	0.3	1.4	0.1	0.6
LOC101928052	2.3	0.6	0.7	0.1	0.1	0.4	1.6	0.1	0.7
CA5B	1.6	0.9	0.8	0.1	0.2	0.6	0.9	0.3	1.2

LOC101928492	1.8	0.7	0.8	0.1	0.2	0.4	0.9	0.1	1.6
LINC00937	1.9	0.3	0.9	0.4	0.3	0.3	1.4	0.2	1.0
C6orf52	2.2	0.6	0.5	0.1	0.4	0.4	1.0	0.2	1.2
LOC100505534	2.3	0.4	0.9	0.1	0.2	0.3	1.6	0.1	0.7
IQCK	1.9	0.8	1.3	0.1	0.3	0.6	0.8	0.1	0.8
ZNF595	2.0	0.7	0.7	0.0	0.3	0.4	1.5	0.1	1.0
RFPL1S	2.3	0.5	0.8	0.1	0.2	0.3	1.6	0.1	0.7
MEF2B	1.8	0.6	0.9	0.2	0.3	0.6	1.3	0.3	0.8
SPDYE5	1.7	0.5	1.1	0.2	0.4	0.6	1.3	0.0	0.8
TMEM177	1.6	0.6	1.3	0.2	0.5	0.8	0.7	0.2	0.7
ARHGEF3	2.6	1.0	1.3	0.1	0.1	0.3	0.6	0.1	0.5
LOC101928215	2.7	0.5	0.9	0.2	0.2	0.4	1.0	0.1	0.7
TDRD3	2.0	0.9	0.9	0.1	0.4	0.4	0.8	0.2	1.0
LOC145757	2.5	0.7	1.2	0.1	0.3	0.5	0.8	0.2	0.4
STRA6	2.2	0.5	1.1	0.1	0.2	0.3	1.4	0.1	0.8
LOC101927619	2.2	0.4	1.1	0.1	0.3	0.3	1.4	0.1	0.8
PCDHB13	2.3	0.7	1.1	0.2	0.4	0.3	0.9	0.1	0.5
N4BP2	0.9	0.3	0.4	0.0	0.1	0.2	2.6	0.3	1.7
ZBTB8A	1.4	0.5	0.6	0.1	0.3	0.4	1.5	0.3	1.5
PRKAA2	2.0	0.6	1.0	0.1	0.3	0.3	1.3	0.1	1.0
SPAG16	1.7	0.4	0.9	0.2	0.5	0.6	1.4	0.2	0.8
FOXD4L1	2.0	0.8	1.0	0.2	0.4	0.4	1.1	0.1	0.6
C19orf82	1.9	0.6	0.7	0.4	0.3	0.6	1.4	0.1	0.8
IMMP2L	1.5	0.6	0.8	0.0	0.3	0.3	2.2	0.1	0.8
ARHGAP9	1.9	0.8	0.8	0.2	0.2	0.3	1.2	0.3	0.9
PCDHB9	2.1	0.7	1.0	0.2	0.5	0.4	0.8	0.2	0.7
ABCB6	1.9	0.9	1.1	0.2	0.4	0.6	0.9	0.2	0.6
ITGB8	1.6	0.5	0.8	0.0	0.2	0.2	1.8	0.1	1.4
DYNC2H1	1.8	0.6	0.9	0.1	0.3	0.4	1.4	0.1	1.0
FRYL	1.9	0.6	0.9	0.1	0.3	0.5	1.3	0.1	1.0
C2orf82	1.7	0.5	1.7	0.2	0.1	0.8	1.1	0.3	0.4
ZNF304	1.9	0.7	1.1	0.1	0.3	0.3	1.1	0.2	0.9
MAST4	2.0	0.7	1.0	0.2	0.4	0.5	1.1	0.1	0.7
PRDM15	2.0	0.9	1.0	0.2	0.4	0.4	0.9	0.2	0.6
LOC100996447	1.8	0.6	1.0	0.1	0.1	0.3	1.5	0.2	1.0
RABL2B	1.6	0.7	1.0	0.2	0.5	1.0	0.9	0.2	0.5
LOC101927580	2.1	0.4	1.0	0.1	0.3	0.3	1.5	0.1	0.7
ZNF268	1.8	0.6	0.8	0.1	0.3	0.5	1.2	0.2	1.1
LOC100133957	2.0	0.5	1.1	0.1	0.3	0.3	1.5	0.2	0.6
KIAA1919	1.7	0.5	0.9	0.1	0.2	0.4	1.7	0.1	1.0
SERAC1	2.0	0.7	1.1	0.1	0.2	0.3	1.0	0.2	0.9
TMEM133	2.1	0.6	1.3	0.1	0.1	0.4	0.8	0.1	1.1
LIPT1	1.4	0.7	1.1	0.1	0.3	0.4	1.7	0.1	0.9
TET2	2.1	0.6	1.0	0.1	0.3	0.4	1.1	0.1	0.8
RWDD2A	2.2	0.7	0.9	0.1	0.3	0.3	1.2	0.2	0.8
HCAR1	2.2	0.7	0.9	0.1	0.4	0.6	1.0	0.2	0.5
NFATC1	2.0	0.8	0.7	0.3	0.4	0.7	0.8	0.2	0.8
C21orf67	1.6	0.7	0.9	0.2	0.6	0.7	0.9	0.2	0.8
LOC101927593	2.3	0.5	1.2	0.2	0.2	0.4	1.0	0.2	0.6
AGER	1.8	0.7	1.6	0.3	0.3	0.4	0.8	0.2	0.6
C1orf192	2.2	0.8	1.0	0.1	0.2	0.3	1.2	0.1	0.6
KANSL1L	2.0	0.5	1.1	0.1	0.2	0.2	1.5	0.1	0.8
MKX	1.4	0.4	1.0	0.3	0.2	0.6	1.3	0.3	1.1
TENM1	2.5	0.7	1.1	0.1	0.2	0.3	0.9	0.2	0.7
C17orf53	1.6	0.5	0.9	0.2	0.2	0.4	1.4	0.3	1.1

ZSCAN16-AS1	2.0	0.8	0.9	0.1	0.3	0.4	1.0	0.1	0.9
MIR137HG	1.0	0.4	0.4	0.2	0.5	0.4	2.2	0.3	1.3
DIO2-AS1	2.3	0.5	0.8	0.2	0.3	0.4	1.4	0.1	0.6
SHE	2.0	0.3	1.0	0.1	0.3	0.3	1.6	0.1	0.7
CCDC24	1.8	0.4	1.5	0.2	0.2	0.6	0.7	0.3	0.7
LOC101929486	2.3	0.6	1.0	0.1	0.3	0.3	1.2	0.1	0.7
CTSO	2.6	1.0	1.2	0.3	0.2	0.8	0.1	0.1	0.3
RCE1	1.2	0.6	0.7	0.5	1.0	0.8	0.6	0.4	0.7
DSTYK	1.8	0.7	0.9	0.1	0.3	0.4	1.4	0.1	0.9
LOC101927132	2.2	0.6	1.0	0.1	0.4	0.4	1.1	0.1	0.7
KIAA1161	2.7	0.8	1.2	0.2	0.4	0.4	0.4	0.0	0.4
FAAH	1.6	0.6	1.1	0.1	0.2	0.4	1.4	0.2	1.0
C1RL	2.3	0.8	1.1	0.2	0.5	0.7	0.4	0.1	0.4
CRISPLD2	2.2	0.7	0.9	0.3	0.8	1.1	0.4	0.1	0.2
EYA3	1.7	0.7	0.9	0.0	0.3	0.3	1.2	0.2	1.1
NYAP1	2.2	0.7	1.4	0.2	0.2	0.3	0.7	0.2	0.6
MCM3AP-AS1	1.5	0.5	0.9	0.2	0.3	0.3	1.5	0.2	1.2
LIMS3	2.2	0.6	1.0	0.1	0.2	0.3	1.4	0.1	0.6
ENO3	1.7	0.8	1.2	0.1	0.3	0.5	1.1	0.1	0.8
OMA1	2.2	0.6	1.0	0.0	0.3	0.3	1.2	0.2	0.7
TSSK2	2.1	0.8	0.7	0.1	0.2	0.3	1.5	0.2	0.8
CDPF1	2.3	0.9	0.7	0.2	0.2	0.5	0.8	0.1	0.6
PPARGC1B	1.4	0.4	0.6	0.2	0.4	0.5	1.5	0.2	1.3
ODF2	1.9	0.6	1.0	0.1	0.3	0.4	1.1	0.2	0.8
LOC100507424	1.9	0.6	0.7	0.2	0.5	0.5	1.0	0.1	0.9
REPS2	2.0	0.5	0.9	0.1	0.3	0.5	1.0	0.1	1.0
ARL17B	2.2	0.3	1.3	0.1	0.2	0.2	1.0	0.3	0.9
TMPRSS3	2.1	0.5	1.1	0.1	0.2	0.2	1.5	0.1	0.7
CASD1	2.3	0.8	0.8	0.1	0.2	0.3	1.1	0.1	0.8
PLGLB2	1.7	0.4	0.7	0.2	0.2	0.2	1.5	0.2	1.3
PLGLB1	1.7	0.4	0.7	0.2	0.2	0.2	1.5	0.2	1.3
PRDM11	1.5	0.5	0.8	0.2	0.4	0.4	1.4	0.2	1.0
MED7	1.8	0.4	1.2	0.1	0.5	0.7	0.8	0.0	0.8
C2orf81	2.2	0.6	1.0	0.2	0.4	0.7	0.7	0.1	0.6
LOC101928015	2.2	0.6	0.7	0.3	0.3	0.5	1.2	0.2	0.6
SPTY2D1-AS1	1.6	0.7	0.9	0.1	0.4	0.3	1.1	0.1	1.2
LOC101926935	2.1	0.5	1.1	0.1	0.2	0.3	1.3	0.1	0.7
PUS7L	1.5	0.6	0.7	0.1	0.3	0.4	1.5	0.2	1.2
SLC25A42	1.9	0.6	0.9	0.3	0.4	0.5	0.8	0.1	0.9
IFT122	1.8	0.8	0.8	0.2	0.3	0.5	1.1	0.1	0.8
FLJ10038	2.0	0.5	0.8	0.1	0.3	0.3	1.6	0.1	0.6
LOC100131094	1.6	0.7	1.0	0.3	0.5	0.4	1.2	0.1	0.6
ERMP1	2.2	0.7	1.0	0.2	0.4	0.5	0.9	0.1	0.5
LOC440386	1.8	0.5	0.9	0.2	0.3	0.5	1.3	0.2	0.8
BAZ2B	2.1	0.6	1.1	0.1	0.3	0.3	1.0	0.2	0.8
LOC389247	1.9	0.5	0.9	0.1	0.3	0.3	1.6	0.1	0.8
KIF11	1.4	0.4	0.6	0.0	0.3	0.4	1.7	0.2	1.3
NOP14-AS1	1.7	0.5	0.8	0.1	0.3	0.4	1.4	0.1	1.0
HAPLN3	1.8	0.6	1.0	0.1	0.3	0.3	1.2	0.2	0.9
BTBD9	2.0	0.6	0.7	0.1	0.2	0.6	1.0	0.1	1.0
SYNM	2.2	0.8	1.2	0.2	0.4	0.5	0.6	0.1	0.5
LINC00174	2.1	0.4	1.0	0.2	0.4	0.4	0.9	0.1	0.8
ZNF234	1.6	0.8	1.0	0.0	0.3	0.3	1.2	0.2	1.1
GPR180	2.0	0.6	1.1	0.1	0.2	0.2	1.2	0.1	0.8
LOC101926897	2.1	0.5	0.8	0.1	0.3	0.3	1.4	0.1	0.8

SEPSECS	2.2	0.6	1.0	0.1	0.4	0.3	1.0	0.2	0.8
METTL4	1.7	0.7	1.0	0.1	0.3	0.2	1.2	0.1	1.0
ZNF480	1.4	0.5	1.1	0.1	0.3	0.4	1.3	0.1	1.2
CCNJ	1.6	0.8	0.6	0.1	0.4	0.4	1.1	0.1	1.4
SYTL4	1.7	0.7	1.0	0.1	0.3	0.5	1.4	0.2	0.7
CLSPN	0.8	0.5	0.5	0.1	0.3	0.3	1.9	0.3	1.6
TRAPPC6A	2.3	1.0	1.2	0.1	0.5	0.7	0.3	0.1	0.2
NEDD9	1.4	0.6	0.7	0.1	0.1	0.2	1.5	0.2	1.6
FKTN	1.4	0.6	0.9	0.0	0.4	0.5	1.5	0.1	1.0
LOC100996579	1.9	0.9	1.1	0.1	0.6	0.6	0.5	0.1	0.6
RNU5D-1	2.0	0.9	0.9	0.2	0.4	0.5	0.8	0.3	0.4
SCARNA11	1.4	1.3	0.3	0.1	0.3	0.8	0.6	0.2	1.2
SNX13	2.1	0.6	1.1	0.0	0.1	0.3	1.0	0.2	0.9
CXorf31	2.0	0.5	1.2	0.1	0.2	0.3	1.5	0.0	0.5
RALGAPA2	2.1	0.7	0.8	0.1	0.3	0.4	1.1	0.2	0.7
SLC16A13	1.4	0.6	1.0	0.2	0.4	0.7	0.9	0.2	0.8
GP6	1.8	0.3	1.0	0.2	0.4	0.3	1.5	0.1	0.7
ANKRD44	1.5	0.6	0.6	0.1	0.4	0.4	1.5	0.2	1.0
LOC101928645	2.3	0.4	1.1	0.1	0.2	0.5	0.5	0.2	1.0
RASEF	1.7	0.6	0.9	0.1	0.0	0.2	1.4	0.2	1.3
CSAD	1.9	0.5	1.1	0.1	0.7	0.4	0.8	0.2	0.6
DZIP1L	1.9	0.5	0.9	0.3	0.4	0.3	1.0	0.2	0.8
PTPRC	2.2	0.6	0.9	0.0	0.1	0.8	1.2	0.1	0.6
LOC101928424	1.5	0.7	1.0	0.1	0.6	0.3	0.9	0.4	0.9
OSBPL6	1.9	0.4	0.6	0.1	0.2	0.3	1.7	0.1	1.1
LOC100130111	1.7	0.6	0.9	0.3	0.6	0.7	0.7	0.1	0.6
ADHFE1	2.0	0.5	1.1	0.2	0.3	0.4	0.9	0.2	0.8
LOC100287515	1.9	0.7	1.0	0.1	0.3	0.3	1.0	0.1	1.0
DOPEY1	2.1	0.6	1.0	0.1	0.2	0.2	1.2	0.1	0.8
C17orf100	2.0	0.7	0.4	0.3	0.5	0.4	1.0	0.2	0.7
MAPRE3	2.1	0.6	0.8	0.2	0.3	0.5	0.9	0.1	0.8
CEP44	2.1	0.5	0.7	0.0	0.3	0.4	1.4	0.1	0.9
REC8	2.1	0.7	1.1	0.1	0.2	0.3	1.0	0.2	0.7
PLXNB1	2.1	0.9	1.2	0.2	0.3	0.4	0.7	0.1	0.4
EXPH5	1.4	0.3	0.7	0.1	0.3	0.4	1.5	0.2	1.5
ZNF503-AS2	1.4	0.7	0.5	0.3	0.4	0.4	1.2	0.4	1.0
DUSP8	1.6	0.5	0.8	0.2	0.3	0.4	1.3	0.3	0.9
MASTL	1.4	0.6	0.7	0.1	0.3	0.4	1.2	0.2	1.3
WDR76	1.1	0.4	0.5	0.1	0.3	0.8	1.5	0.2	1.3
COX19	1.8	0.9	0.9	0.1	0.2	0.6	1.0	0.1	0.7
CCDC71	1.3	0.4	0.6	0.2	0.5	0.6	1.4	0.2	1.1
APOBEC3D	1.4	1.0	1.1	0.1	0.2	0.6	0.8	0.2	0.9
TIGD7	1.5	0.7	0.6	0.0	0.4	0.4	1.3	0.3	1.1
IFNAR2	2.4	0.9	0.8	0.0	0.2	0.3	0.6	0.1	0.9
LINC00310	2.3	0.3	0.9	0.1	0.3	0.2	1.3	0.2	0.7
CPS1	1.6	0.6	0.8	0.1	0.4	0.5	1.2	0.2	0.9
ZKSCAN2	1.5	0.6	0.7	0.1	0.2	0.5	1.3	0.1	1.1
DNAJB5	1.0	0.6	0.6	0.3	0.5	0.6	0.9	0.5	1.2
TBC1D3B	1.9	0.8	1.0	0.2	0.3	0.4	0.8	0.2	0.6
CCDC111	1.7	0.5	1.1	0.1	0.3	0.4	1.2	0.1	0.9
C18orf54	1.5	0.3	0.9	0.0	0.4	0.2	1.6	0.2	1.3
BMP8B	1.8	1.1	1.0	0.3	0.4	0.6	0.6	0.1	0.5
RNLS	1.9	0.3	0.9	0.2	0.3	0.4	1.2	0.1	0.9
TUBGCP5	1.9	0.7	0.9	0.1	0.3	0.4	1.3	0.1	0.6
KRT86	1.7	0.7	0.7	0.2	0.2	0.2	1.3	0.2	0.9

LOC101928949	2.7	0.7	1.0	0.0	0.1	0.5	0.9	0.0	0.4
NKX6-1	1.6	0.4	0.8	0.3	0.4	0.8	1.1	0.2	0.7
ADCY7	1.9	0.6	0.6	0.2	0.3	0.6	1.0	0.1	0.9
ALDH1L2	2.1	0.4	0.8	0.1	0.2	0.2	1.6	0.1	0.6
VWA3A	1.7	0.6	0.9	0.1	0.2	0.4	1.3	0.2	1.1
STL	2.1	0.4	0.8	0.1	0.2	0.3	1.5	0.1	0.7
BPGM	1.4	0.7	1.0	0.1	0.2	0.3	1.6	0.2	0.8
BDNF-AS	2.2	0.6	0.8	0.1	0.2	0.3	1.2	0.1	0.8
PRELID2	2.3	0.6	1.1	0.1	0.1	0.1	0.8	0.1	1.0
LOC100506100	2.1	0.5	0.9	0.0	0.4	0.6	0.7	0.0	0.7
LOC399715	2.2	0.4	0.9	0.2	0.2	0.2	1.5	0.1	0.6
ARVCF	2.0	0.7	1.1	0.2	0.2	0.5	1.0	0.1	0.5
TROAP	1.5	0.5	0.7	0.3	0.4	0.5	1.2	0.2	1.0
A1BG-AS1	2.1	0.6	1.2	0.2	0.3	0.2	1.0	0.1	0.6
LOC101929396	1.5	0.2	0.7	0.2	0.4	0.7	1.6	0.1	0.7
RABL2A	1.7	0.8	1.0	0.4	0.2	0.5	1.0	0.1	0.5
ERVW-1	2.5	0.5	0.9	0.1	0.5	0.4	0.8	0.0	0.4
TDRD7	1.6	0.7	1.0	0.1	0.3	0.4	1.2	0.1	0.9
ZBTB6	1.7	0.5	0.8	0.0	0.3	0.1	1.5	0.1	1.3
PNPLA3	2.4	0.7	1.0	0.2	0.4	0.4	0.5	0.1	0.5
ASB16	2.1	0.4	0.8	0.1	0.3	0.3	1.2	0.1	0.9
LPP-AS2	2.0	0.5	0.9	0.1	0.3	0.4	1.4	0.1	0.6
TNFRSF10C	2.6	0.7	1.2	0.1	0.4	0.3	0.5	0.0	0.3
CHRNA1	2.6	0.8	1.3	0.1	0.4	0.4	0.3	0.1	0.2
SP110	2.2	0.7	0.9	0.1	0.3	0.5	0.9	0.1	0.5
C8orf37	2.4	0.9	1.3	0.1	0.2	0.3	0.7	0.1	0.2
ST6GALNAC3	1.1	0.3	0.9	0.0	0.3	0.2	1.8	0.2	1.5
SCYL3	1.8	0.8	0.9	0.1	0.3	0.5	1.1	0.1	0.7
TMED7-TICAM2	1.8	0.7	0.8	0.0	0.1	0.2	1.0	0.1	1.5
NSMCE2	1.7	0.4	1.0	0.1	0.4	0.2	1.2	0.2	0.9
EXOC6	1.7	0.6	0.9	0.1	0.2	0.4	1.4	0.2	0.7
RBAK	1.7	0.7	0.8	0.0	0.2	0.2	1.2	0.1	1.2
ZNF35	1.4	0.6	0.9	0.1	0.3	0.4	1.3	0.1	1.0
ATP2B4	2.1	0.7	1.0	0.1	0.3	0.3	0.8	0.1	0.7
MMP19	2.3	0.5	1.0	0.1	0.4	0.6	0.4	0.1	0.8
NUDT7	1.9	0.5	0.8	0.1	0.4	0.3	1.4	0.1	0.7
KLHDC8B	2.2	0.5	0.7	0.1	0.3	0.4	1.1	0.2	0.7
ATXN7L1	1.5	0.4	1.1	0.1	0.4	0.3	1.0	0.1	1.2
ZNF789	2.4	0.2	0.7	0.1	0.2	0.4	1.4	0.1	0.6
TMEM75	1.7	0.2	0.9	0.1	0.2	0.5	1.7	0.1	0.9
GJC2	2.0	0.5	0.7	0.2	0.1	0.2	1.4	0.2	0.9
YPEL2	2.2	0.4	0.8	0.2	0.2	0.3	0.7	0.2	1.2
NSUN3	1.8	0.8	0.8	0.0	0.3	0.2	1.1	0.1	1.0
ZNF844	1.7	0.7	0.8	0.1	0.3	0.3	1.1	0.1	1.0
C5orf56	1.8	0.4	1.0	0.3	0.6	0.3	1.0	0.2	0.6
RPGRIP1L	1.8	0.6	0.8	0.1	0.3	0.4	1.2	0.1	0.8
NPR2	1.2	0.5	0.7	0.1	0.2	0.2	1.7	0.3	1.2
LOC101929712	1.9	0.3	1.3	0.1	0.1	0.2	1.1	0.0	1.1
ZNF230	2.0	0.7	1.2	0.1	0.2	0.3	0.9	0.1	0.7
GOLGA8A	2.2	0.5	1.0	0.2	0.3	0.4	0.8	0.2	0.5
THAP10	1.5	0.5	0.9	0.2	0.2	0.3	1.6	0.2	0.7
EVL	1.6	0.6	1.0	0.2	0.5	0.5	0.9	0.1	0.6
INVS	2.1	0.6	0.9	0.1	0.3	0.4	1.0	0.1	0.6
AASDH	2.0	0.5	1.1	0.0	0.2	0.3	1.0	0.2	0.9
REM2	1.7	1.0	1.2	0.1	0.1	0.2	0.8	0.1	1.0

APOL2	2.0	0.9	1.0	0.2	0.3	0.4	0.6	0.2	0.6
LINC00964	2.0	0.4	0.9	0.1	0.2	0.3	1.5	0.2	0.5
MYL10	2.0	0.3	0.8	0.2	0.1	0.2	1.7	0.1	0.6
LOC101059976	1.5	0.5	0.8	0.2	0.3	0.4	1.4	0.2	0.7
LOC100130093	1.7	0.6	0.9	0.2	0.3	0.4	1.0	0.2	0.8
ARIH2OS	1.6	0.8	0.8	0.1	0.2	0.2	1.2	0.2	1.0
KATNAL1	2.1	0.6	0.9	0.1	0.1	0.3	1.1	0.0	0.8
TMC7	1.9	0.7	0.7	0.1	0.2	0.2	1.3	0.1	0.8
ZNF407	1.6	0.5	0.7	0.1	0.3	0.4	1.4	0.2	0.9
LRRC16A	2.0	0.5	0.7	0.1	0.2	0.4	1.1	0.2	0.9
HERC5	1.7	0.7	0.6	0.1	0.2	0.3	1.3	0.3	0.9
MAGI2-AS3	1.8	0.5	0.8	0.2	0.2	0.2	1.4	0.2	0.7
ID4	2.0	1.0	0.8	0.5	0.5	0.9	0.2	0.1	0.2
SESTD1	2.3	0.7	1.2	0.1	0.2	0.3	0.8	0.1	0.4
WNT3	1.8	0.6	1.2	0.2	0.3	0.4	1.0	0.1	0.4
FAM126B	1.8	0.7	0.9	0.1	0.3	0.3	0.9	0.1	0.9
LOC100133612	1.9	0.5	0.9	0.1	0.2	0.3	1.4	0.1	0.6
SASS6	1.6	0.5	0.9	0.0	0.2	0.3	1.3	0.2	1.1
KIF21A	1.4	0.4	0.6	0.0	0.2	0.3	1.6	0.2	1.3
ZNF33B	1.3	0.6	0.7	0.1	0.3	0.3	1.3	0.2	1.3
CNOT6L	1.7	0.7	0.7	0.1	0.4	0.5	1.0	0.1	0.9
TRAPPC2	1.9	0.5	0.8	0.2	0.3	0.4	1.0	0.0	0.9
PCSK4	2.0	0.9	1.1	0.4	0.4	0.6	0.3	0.1	0.4
LOC101928100	2.4	0.5	1.1	0.1	0.2	0.3	0.8	0.1	0.6
ZNF614	1.8	0.9	0.8	0.0	0.3	0.4	0.8	0.3	0.8
PDLIM3	1.9	0.7	1.0	0.1	0.2	0.4	1.0	0.2	0.6
ADCK1	2.3	0.5	0.8	0.2	0.7	0.6	0.7	0.1	0.3
SNED1	2.5	0.8	1.2	0.2	0.4	0.3	0.3	0.1	0.2
DEPDC5	1.9	0.5	0.9	0.1	0.3	0.4	1.0	0.1	0.7
SPDYE6	1.8	0.4	1.0	0.1	0.3	0.3	1.4	0.1	0.6
PIGN	1.4	0.6	0.9	0.1	0.3	0.4	1.3	0.2	0.9
HOXB4	2.0	0.9	1.0	0.2	0.4	0.2	0.6	0.2	0.5
ACVR2B-AS1	1.9	0.4	1.0	0.1	0.2	0.3	1.4	0.1	0.6
MGC16275	2.0	0.6	0.8	0.1	0.2	0.3	1.3	0.1	0.7
NHS	1.7	0.5	0.8	0.2	0.3	0.6	0.9	0.1	0.7
TRIM21	1.9	0.9	1.2	0.1	0.3	0.3	0.6	0.2	0.6
ZNF79	1.4	0.5	0.7	0.1	0.4	0.5	1.2	0.1	1.1
STBD1	1.7	0.9	0.8	0.1	0.3	0.3	1.0	0.1	0.8
SLCO1B3	1.3	0.4	0.7	0.0	0.2	0.3	1.7	0.2	1.2
FAM150B	2.0	0.7	0.6	0.2	0.2	0.8	0.8	0.0	0.6
HHAT	1.8	0.7	0.6	0.1	0.1	0.4	1.2	0.2	0.8
GAD1	1.7	0.5	0.9	0.1	0.3	0.3	1.3	0.1	0.8
CDA	1.1	0.4	1.2	0.2	0.2	0.5	1.4	0.0	1.0
GPR124	2.0	0.8	0.9	0.2	0.4	0.4	0.7	0.2	0.5
KRBA2	2.5	0.7	1.0	0.0	0.3	0.4	0.7	0.1	0.3
DUSP2	1.1	0.7	0.5	0.1	0.2	0.5	1.6	0.3	1.0
LOC101928518	2.2	1.2	1.1	0.1	0.3	0.1	0.4	0.1	0.4
LOC101928066	1.4	0.7	1.0	0.1	0.3	0.4	1.0	0.4	0.6
LOC100130238	1.4	0.6	0.8	0.3	0.5	0.7	0.9	0.1	0.6
C11orf63	2.5	0.7	0.5	0.0	0.2	0.4	0.8	0.1	0.6
C1orf131	1.7	0.6	1.0	0.0	0.4	0.3	1.1	0.1	0.8
C16orf62	2.2	0.7	0.8	0.2	0.3	0.4	0.8	0.1	0.5
TAF12	1.7	0.9	1.1	0.1	0.4	0.5	0.8	0.1	0.4
USP2-AS1	1.1	0.5	0.6	0.3	0.5	0.4	1.1	0.2	1.3
JHDM1D	1.7	0.4	0.7	0.1	0.2	0.2	1.3	0.2	1.2

PDSS1	1.1	0.4	1.0	0.1	0.2	0.4	1.5	0.2	1.1
LOC101927673	1.6	0.4	1.3	0.2	0.3	0.4	1.0	0.1	0.7
CAMK4	0.9	0.4	0.5	0.1	0.3	0.4	1.7	0.3	1.3
DPF1	1.4	0.8	1.0	0.2	0.4	0.4	0.9	0.2	0.6
TSNAX-DISC1	1.5	0.5	0.8	0.1	0.2	0.3	1.5	0.2	1.0
STIL	1.4	0.6	0.7	0.0	0.2	0.4	1.2	0.2	1.2
PRC1-AS1	1.4	0.5	0.7	0.2	0.2	0.6	1.5	0.1	0.8
SLX4IP	1.7	0.5	0.7	0.1	0.4	0.3	0.9	0.2	1.1
TMEM249	1.4	0.7	0.8	0.2	0.4	0.7	1.0	0.2	0.6
ZNF821	1.5	0.4	1.3	0.1	0.2	0.2	1.2	0.2	0.9
BLACE	2.0	0.5	0.9	0.1	0.3	0.3	1.2	0.1	0.5
APAF1	1.6	0.6	0.9	0.0	0.2	0.2	1.4	0.1	0.8
SIAH1	1.6	0.7	1.0	0.1	0.4	0.5	0.0	0.5	1.0
GSAP	2.1	0.8	1.1	0.1	0.4	0.4	0.6	0.1	0.4
ELMO3	1.9	0.6	0.9	0.1	0.3	0.3	1.1	0.2	0.6
KIRREL3	1.0	0.4	0.6	0.1	0.2	0.2	2.0	0.2	1.1
ARL6	1.6	0.5	1.0	0.1	0.2	0.3	1.2	0.1	0.9
PEX1	1.7	0.7	0.9	0.1	0.3	0.3	1.0	0.1	0.7
MCIDAS	1.2	0.4	0.7	0.2	0.3	0.6	1.2	0.3	1.1
LRRC17	1.9	0.6	0.8	0.0	0.2	0.2	1.0	0.1	0.9
LOC100507412	1.8	0.4	0.8	0.1	0.3	0.3	1.2	0.1	0.9
SP140	2.5	0.5	0.9	0.1	0.3	0.8	0.3	0.0	0.4
FLJ38717	1.9	0.4	0.8	0.1	0.3	0.2	1.3	0.1	0.7
CCDC114	2.1	1.0	1.3	0.2	0.3	0.2	0.5	0.0	0.3
MICALL2	1.9	0.8	1.0	0.2	0.3	0.5	0.6	0.1	0.4
SNORD15A	0.0	1.1	0.9	0.1	0.5	0.3	2.0	0.0	1.0
LOC643837	1.7	0.6	0.7	0.2	0.3	0.5	0.9	0.2	0.8
TEF	2.0	0.8	1.0	0.1	0.3	0.2	1.1	0.1	0.4
TENM4	1.9	0.7	1.0	0.2	0.5	0.7	0.5	0.1	0.4
LINC00568	1.9	0.4	0.7	0.1	0.1	0.3	1.3	0.2	0.7
PKI55	1.7	0.6	1.0	0.1	0.4	0.5	1.0	0.1	0.7
LEPR	1.8	0.6	0.8	0.1	0.3	0.3	1.1	0.1	0.7
SETD5-AS1	1.5	0.4	1.1	0.1	0.2	0.2	1.5	0.1	0.8
SCML2	1.6	0.4	0.8	0.1	0.2	0.3	1.5	0.1	0.9
LOC377711	1.8	0.7	1.0	0.3	0.3	0.6	0.7	0.1	0.5
S1PR5	0.9	0.6	0.7	0.1	0.5	0.5	1.3	0.3	1.0
SLC22A20	2.0	0.5	0.9	0.1	0.3	0.5	0.9	0.1	0.6
TMEM19	1.9	0.8	0.8	0.0	0.2	0.4	0.9	0.2	0.7
ZNF573	1.9	0.4	0.8	0.1	0.3	0.2	1.2	0.2	0.7
L3MBTL3	1.4	0.4	0.8	0.0	0.2	0.2	1.2	0.2	1.3
IL12A	0.8	0.5	0.6	0.0	0.3	0.5	1.2	0.3	1.7
GEMIN8	1.8	0.6	0.9	0.2	0.5	0.6	0.7	0.2	0.5
ARL15	1.3	0.8	0.9	0.0	0.1	0.3	1.5	0.1	0.9
MKRN3	1.6	0.4	1.0	0.1	0.2	0.3	1.1	0.1	1.1
IRAK3	2.0	0.6	1.0	0.1	0.1	0.2	1.0	0.1	0.7
SUPT20H	1.8	0.7	0.9	0.2	0.3	0.4	0.6	0.1	0.9
MROH8	1.4	0.7	0.7	0.1	0.3	0.5	1.2	0.2	0.7
TTC28-AS1	1.5	0.4	0.6	0.1	0.1	0.3	1.3	0.3	1.1
ZNF804A	1.3	0.4	0.5	0.0	0.2	0.3	1.4	0.3	1.4
TGFA	1.6	0.8	1.4	0.1	0.4	0.8	0.3	0.0	0.4
RGS9	1.5	0.6	0.6	0.1	0.4	0.5	1.1	0.1	0.9
METTL8	1.2	0.5	0.7	0.1	0.2	0.5	1.6	0.1	1.1
AQP11	1.7	0.2	1.0	0.2	0.6	0.3	1.0	0.1	0.8
LOC101928133	1.8	0.7	0.8	0.0	0.1	0.3	1.4	0.0	0.7
GTDC1	1.2	0.8	0.8	0.1	0.2	0.3	1.1	0.2	1.1

HMOX1	1.8	0.9	1.0	0.2	0.5	0.2	0.7	0.2	0.4
GPR3	1.5	0.5	0.7	0.1	0.3	0.5	1.1	0.2	0.9
FAM95C	2.0	0.5	0.9	0.1	0.3	0.4	1.0	0.1	0.7
POPDC3	1.9	0.6	0.7	0.1	0.2	0.3	1.0	0.1	1.0
TAPT1	1.5	0.7	0.8	0.1	0.3	0.3	1.1	0.1	0.9
BARD1	1.3	0.6	0.7	0.1	0.1	0.5	1.3	0.1	1.0
CENPC	1.4	0.6	0.7	0.1	0.3	0.4	1.3	0.2	1.0
LIG4	1.6	0.6	0.7	0.0	0.2	0.2	1.4	0.1	0.9
LOC105379420	1.7	0.5	1.0	0.1	0.2	0.4	0.9	0.1	0.9
PIGA	1.6	0.6	0.7	0.0	0.2	0.3	1.3	0.1	1.0
KIAA1217	2.1	0.7	0.9	0.1	0.3	0.4	0.6	0.2	0.6
CHAD	2.1	0.6	1.1	0.2	0.2	0.1	1.1	0.1	0.3
SFMBT1	1.5	0.5	0.7	0.1	0.3	0.2	1.5	0.1	0.8
SHISA3	1.6	0.8	0.7	0.1	0.3	0.6	0.8	0.1	0.8
CLIC3	1.7	0.9	1.0	0.1	0.4	0.6	0.6	0.1	0.5
KIAA1244	1.6	0.5	0.7	0.1	0.2	0.3	1.4	0.2	0.9
TCF7L1	1.1	0.6	1.1	0.4	0.3	0.3	0.8	0.3	0.8
MAPT	1.6	0.8	0.7	0.2	0.4	0.3	0.8	0.2	0.8
C3orf33	1.9	0.8	0.9	0.2	0.2	0.5	0.8	0.0	0.4
FOXD2-AS1	1.6	0.6	0.9	0.1	0.3	0.6	0.8	0.1	0.8
LOC283352	1.7	0.6	0.8	0.2	0.2	0.4	1.0	0.3	0.6
NUDT17	1.5	0.6	1.1	0.2	0.2	0.4	1.0	0.1	0.6
LOC101927702	2.0	0.4	1.0	0.1	0.3	0.3	0.9	0.1	0.6
GMPR	1.2	0.5	0.8	0.1	0.3	0.2	1.3	0.2	1.2
C7orf60	1.3	0.4	1.1	0.1	0.3	0.2	1.2	0.2	0.9
CASP6	2.0	0.7	0.8	0.0	0.3	0.2	0.8	0.2	0.8
ZNF564	1.6	0.7	0.9	0.1	0.3	0.4	1.1	0.1	0.6
TMEM145	2.2	0.6	0.9	0.2	0.3	0.3	0.7	0.0	0.5
NAP1L3	1.6	0.5	0.8	0.1	0.5	0.6	1.2	0.1	0.5
OSBP2	1.9	0.5	0.9	0.2	0.3	0.5	0.7	0.2	0.7
NHSL1	1.2	0.4	0.5	0.1	0.3	0.3	1.4	0.2	1.3
PCYOX1L	1.6	0.7	0.8	0.3	0.5	0.7	0.6	0.1	0.4
DLGAP5	1.5	0.5	0.7	0.1	0.3	0.6	1.1	0.1	0.8
SLC35G1	1.5	0.4	0.8	0.3	0.3	0.3	1.4	0.1	0.7
TRAC	1.8	1.0	0.8	0.0	0.2	0.3	0.7	0.1	0.8
LOC101929125	2.1	0.6	0.9	0.1	0.2	0.4	0.8	0.2	0.4
KCNN2	1.9	0.7	0.9	0.2	0.2	0.5	0.6	0.1	0.6
CCDC144NL	1.5	0.7	1.0	0.1	0.5	0.3	0.7	0.1	0.8
RECK	1.8	0.6	1.0	0.1	0.3	0.4	0.7	0.2	0.7
PACRG	1.7	0.4	1.0	0.2	0.3	0.2	1.3	0.1	0.6
IFI44	1.9	0.8	1.0	0.0	0.1	0.3	0.7	0.1	0.8
LOC100507419	1.5	0.6	0.8	0.4	0.5	0.5	0.6	0.2	0.6
LOC101929410	2.0	0.3	0.9	0.1	0.2	0.2	1.3	0.1	0.6
TMEM67	1.6	0.6	1.0	0.1	0.3	0.4	1.0	0.2	0.6
ACOT1	2.0	1.0	1.1	0.2	0.4	0.3	0.3	0.0	0.4
ASIP	1.2	0.3	0.5	0.1	0.3	0.5	2.0	0.1	0.8
LOC101929070	1.6	0.4	1.0	0.2	0.3	0.3	0.8	0.1	1.0
SLC2A8	1.6	0.8	1.0	0.2	0.4	0.5	0.6	0.1	0.5
LOC644794	1.7	0.4	1.0	0.2	0.5	0.4	0.7	0.1	0.6
LIPT2	2.2	0.5	0.9	0.2	0.4	0.6	0.6	0.1	0.2
CCNT2	1.6	0.6	0.9	0.1	0.2	0.4	0.9	0.1	0.9
USP45	1.5	0.6	0.9	0.0	0.2	0.3	1.4	0.1	0.8
LOC642633	2.0	0.4	0.6	0.3	0.3	0.5	0.9	0.1	0.6
LOC101927925	1.8	0.7	0.8	0.1	0.2	0.5	0.7	0.2	0.7
PCBP1-AS1	1.8	0.4	0.9	0.2	0.4	0.4	0.9	0.1	0.6

EXOG	1.8	0.6	0.5	0.1	0.1	0.2	1.4	0.1	0.9
ITPR2	1.5	0.4	0.7	0.1	0.3	0.4	1.3	0.1	0.9
AOC3	1.7	0.5	1.0	0.1	0.1	0.2	1.0	0.2	1.0
SPERT	2.1	0.8	0.8	0.2	0.2	0.3	0.6	0.1	0.5
RAB3IL1	1.5	0.5	0.5	0.2	0.2	0.3	1.2	0.1	1.2
NEK1	1.6	0.6	0.8	0.1	0.2	0.3	1.1	0.1	0.8
BEND2	1.9	0.4	0.9	0.2	0.2	0.2	1.2	0.1	0.6
SAP30L-AS1	1.7	0.3	0.8	0.1	0.3	0.3	1.4	0.1	0.7
FAM72D	1.1	0.9	0.7	0.0	0.1	0.0	1.3	0.2	1.3
LINC00221	1.9	0.4	0.8	0.1	0.1	0.3	1.4	0.0	0.6
ZNF488	1.3	0.5	0.8	0.2	0.5	0.6	0.9	0.2	0.7
TMEM110	0.8	1.2	1.5	0.2	0.5	0.0	0.3	0.1	1.2
FAM78B	1.3	0.7	1.2	0.3	0.2	0.3	0.5	0.2	0.9
ZSCAN16	1.9	0.5	1.0	0.1	0.1	0.3	0.6	0.1	0.9
NDUFA6-AS1	1.6	0.6	0.8	0.1	0.3	0.3	1.1	0.2	0.7
PTGER1	1.4	0.7	0.8	0.3	0.2	0.3	1.0	0.2	0.6
ZNF484	1.7	0.6	0.8	0.0	0.2	0.2	1.0	0.2	1.0
PDE1C	0.9	0.4	0.5	0.1	0.4	0.5	1.1	0.3	1.4
SLITRK5	1.9	0.7	0.9	0.2	0.3	0.4	0.6	0.1	0.6
LOC101927017	2.1	0.4	0.6	0.1	0.2	0.2	1.5	0.1	0.5
ASGR1	1.1	0.2	0.5	0.2	0.5	0.4	1.4	0.2	1.2
C17orf107	1.4	0.7	0.6	0.1	0.2	0.4	1.3	0.1	0.8
LOC100289561	2.0	0.5	0.9	0.0	0.1	0.2	0.8	0.3	0.8
LOC101928538	2.1	0.4	0.9	0.1	0.3	0.5	0.8	0.1	0.6
SWSAP1	1.6	0.5	0.6	0.3	0.2	0.5	1.1	0.2	0.6
MFSD7	1.5	0.6	0.9	0.2	0.4	0.4	0.9	0.2	0.5
ZNF232	1.6	0.3	0.9	0.1	0.2	0.5	1.2	0.2	0.6
ZNF765	1.2	0.6	0.9	0.0	0.3	0.5	1.1	0.1	0.9
PXMP4	1.7	0.6	1.0	0.1	0.7	0.7	0.4	0.1	0.3
KIF20A	1.4	0.6	0.6	0.2	0.6	0.7	0.7	0.1	0.6
ERMAP	1.9	0.7	1.1	0.1	0.3	0.5	0.5	0.1	0.3
LOC729291	1.3	0.4	0.8	0.2	0.4	0.3	1.2	0.2	0.8
NRG2	1.6	0.6	0.8	0.4	0.3	0.5	0.7	0.1	0.6
LOC100507547	1.6	0.8	0.6	0.1	0.3	0.5	0.9	0.0	0.8
DSCC1	1.0	0.4	0.8	0.1	0.3	0.4	1.4	0.2	0.9
TBX6	1.8	0.8	1.0	0.2	0.3	0.6	0.4	0.2	0.4
BATF2	1.3	1.0	0.8	0.1	0.3	0.4	0.8	0.2	0.7
NEURL2	2.1	0.2	0.7	0.3	0.6	0.5	0.8	0.1	0.3
MYLK-AS1	1.7	0.5	0.9	0.1	0.3	0.3	1.1	0.1	0.6
SNRK	1.5	0.6	0.9	0.1	0.2	0.4	0.9	0.2	1.0
LINC00478	2.1	0.5	0.6	0.1	0.2	0.2	1.0	0.1	0.9
LOC101928480	1.3	0.2	0.4	0.2	0.3	0.3	2.2	0.1	0.6
TMEM184A	1.3	0.7	0.8	0.1	0.2	0.2	1.1	0.1	1.0
RNU6-15P	3.0	0.0	0.0	0.0	1.3	0.0	0.8	0.1	0.4
HECTD2	1.4	0.5	0.7	0.1	0.2	0.2	1.5	0.1	0.8
SCRN3	1.4	0.7	0.8	0.1	0.2	0.5	0.8	0.1	0.9
RGL4	1.5	0.5	0.8	0.1	0.4	0.5	1.0	0.1	0.6
SORBS1	1.9	0.3	0.7	0.2	0.2	0.2	1.3	0.1	0.6
LOC100506207	1.7	0.4	0.9	0.1	0.2	0.4	1.2	0.1	0.6
RGL3	1.0	0.7	0.8	0.3	0.4	0.7	0.8	0.1	0.7
GPRASP2	1.4	0.7	1.0	0.1	0.2	0.5	1.0	0.1	0.5
LOC100507458	1.6	0.5	0.9	0.1	0.4	0.4	1.0	0.2	0.6
TCTEX1D4	1.3	0.6	1.1	0.2	0.4	0.4	1.1	0.1	0.3
ASTE1	1.8	0.6	0.8	0.0	0.2	0.3	1.2	0.1	0.6
ZFPM2	1.7	0.6	0.7	0.0	0.2	0.4	1.0	0.1	0.8

TP63	2.2	0.9	0.9	0.2	0.5	0.5	0.2	0.1	0.2
PAIP2B	1.5	0.4	0.9	0.1	0.2	0.3	1.0	0.1	0.9
MEX3B	1.4	0.6	0.8	0.1	0.1	0.3	1.2	0.3	0.8
DENND4C	1.7	0.6	0.8	0.1	0.3	0.3	1.0	0.1	0.7
PPP1R12B	2.1	0.5	0.7	0.2	0.3	0.3	0.8	0.1	0.6
ELOVL2-AS1	2.2	0.4	0.9	0.1	0.2	0.2	0.9	0.1	0.6
PTPN4	1.7	0.5	0.8	0.0	0.2	0.2	1.0	0.2	0.9
ZNF548	1.4	0.6	1.1	0.0	0.2	0.3	1.1	0.1	0.7
EID3	1.7	0.3	1.0	0.1	0.5	0.2	1.3	0.0	0.4
CCDC18	1.5	0.6	0.8	0.1	0.2	0.3	1.1	0.2	0.9
ATP8A1	2.5	0.8	1.2	0.0	0.1	0.2	0.4	0.0	0.2
PIGW	1.6	0.5	0.7	0.0	0.1	0.4	1.1	0.1	0.9
TMEM229B	1.8	0.5	0.8	0.2	0.3	0.4	1.0	0.1	0.5
DPY19L3	1.6	0.5	0.6	0.1	0.3	0.4	1.2	0.2	0.8
SOCS1	1.8	0.8	0.6	0.2	0.4	0.4	0.8	0.1	0.4
RAB40AL	2.2	0.3	0.7	0.2	0.3	0.4	1.0	0.0	0.3
ZNF627	1.6	0.6	0.9	0.1	0.2	0.3	1.0	0.1	0.7
LOC101927266	2.5	0.2	0.5	0.1	0.4	0.3	1.0	0.3	0.3
TLN2	1.2	0.4	0.6	0.1	0.3	0.4	1.3	0.1	0.9
OSR2	1.3	0.5	0.8	0.1	0.3	0.3	1.0	0.2	0.9
TRIM61	2.1	0.3	1.0	0.1	0.2	0.2	0.9	0.1	0.6
DNAJC22	1.9	0.6	0.9	0.1	0.3	0.4	0.6	0.2	0.5
MUC5B	2.4	0.8	1.3	0.2	0.3	0.2	0.2	0.1	0.1
MYZAP	2.1	0.2	0.7	0.1	0.2	0.1	1.2	0.2	0.7
MFSD11	1.4	0.4	1.0	0.0	0.1	0.2	1.3	0.1	0.8
TMEM143	1.7	0.6	0.9	0.1	0.4	0.6	0.6	0.1	0.4
CCDC125	1.7	0.5	0.9	0.0	0.3	0.3	0.9	0.1	0.7
LOC100505727	1.7	0.5	0.8	0.1	0.2	0.5	0.9	0.1	0.7
LOC101929209	2.1	0.3	0.3	0.2	0.1	0.2	1.4	0.3	0.6
DISP1	1.5	0.4	0.6	0.1	0.3	0.3	1.0	0.3	0.9
FAM84A	1.2	0.5	0.5	0.2	0.3	0.5	1.0	0.3	1.0
LINC00176	2.0	0.7	0.9	0.2	0.2	0.4	0.4	0.1	0.5
ZNF766	2.1	0.6	0.6	0.1	0.3	0.4	0.7	0.1	0.7
LOC100506421	2.0	0.4	0.8	0.1	0.2	0.2	1.1	0.1	0.6
LOC101928277	1.8	0.4	0.8	0.1	0.3	0.2	1.4	0.1	0.5
ADAMTSL4	1.5	0.3	0.6	0.1	0.2	0.3	1.3	0.2	0.9
LOC150622	1.9	0.3	0.8	0.1	0.2	0.2	1.3	0.1	0.5
IL24	1.1	0.3	0.4	0.1	0.5	0.6	1.2	0.2	1.2
SPIN3	1.5	0.5	0.6	0.2	0.3	0.4	0.8	0.2	0.9
C1RL-AS1	1.7	0.6	0.9	0.1	0.3	0.3	0.8	0.1	0.6
RAB3D	1.6	0.5	0.8	0.1	0.2	0.4	0.9	0.1	0.9
LINC00601	1.5	0.7	1.3	0.1	0.3	0.3	0.5	0.1	0.6
PTPRH	1.9	0.8	1.0	0.0	0.1	0.2	0.9	0.2	0.4
LOC100505666	1.2	0.5	0.8	0.2	0.5	0.6	0.8	0.1	0.9
INS-IGF2	0.8	0.4	0.5	0.5	0.7	0.9	0.7	0.2	0.8
SRD5A3	1.6	0.4	0.8	0.3	0.4	0.6	0.7	0.1	0.7
RXFP4	2.2	0.6	1.1	0.1	0.4	0.3	0.5	0.0	0.3
GPR68	1.7	0.6	0.8	0.4	0.3	0.5	0.5	0.1	0.6
LOC101927270	2.3	0.4	0.5	0.3	0.4	0.3	0.6	0.2	0.5
LOC101928578	1.7	0.4	0.6	0.1	0.3	0.3	1.4	0.1	0.6
MATN3	1.9	0.6	0.8	0.1	0.2	0.2	0.9	0.1	0.6
HLA-F-AS1	1.8	0.5	0.9	0.1	0.2	0.3	1.1	0.1	0.5
ENPP4	1.6	0.6	0.6	0.0	0.2	0.1	1.2	0.1	1.0
LOC101927810	1.7	0.3	1.0	0.1	0.3	0.3	1.1	0.1	0.6
MCOLN2	1.2	0.7	0.7	0.0	0.3	0.3	1.4	0.1	0.8

FAM200B	1.2	0.5	0.7	0.1	0.2	0.4	1.2	0.2	1.0
CEP152	1.2	0.6	0.7	0.1	0.2	0.3	1.3	0.2	1.0
CDC14A	1.6	0.4	0.6	0.0	0.2	0.4	0.9	0.2	1.1
HELQ	1.4	0.5	0.7	0.1	0.3	0.2	1.4	0.1	0.8
TCL6	1.6	0.4	0.8	0.1	0.3	0.3	1.3	0.1	0.6
HOXC8	1.2	0.6	0.5	0.2	0.7	0.6	1.1	0.1	0.5
GPER	1.3	0.6	0.6	0.2	0.3	0.3	0.9	0.2	1.1
MAP10	1.5	0.5	0.8	0.2	0.4	0.5	0.7	0.1	0.6
LOC101927050	1.7	0.6	0.7	0.0	0.0	0.0	1.2	0.0	1.2
FLJ30838	1.7	0.5	0.7	0.1	0.2	0.2	1.4	0.1	0.6
ZNF419	1.6	0.5	1.0	0.1	0.3	0.5	0.8	0.1	0.6
FUT8-AS1	1.6	0.7	1.0	0.2	0.3	0.3	0.8	0.1	0.5
HMGN5	0.8	0.3	0.4	0.1	0.1	0.4	1.8	0.3	1.2
DPH6	1.3	0.7	0.8	0.1	0.8	0.4	0.7	0.0	0.6
NAPA-AS1	1.9	0.4	0.9	0.1	0.2	0.4	0.9	0.3	0.3
ZNF669	1.4	0.6	0.6	0.1	0.1	0.3	1.1	0.1	1.1
OTUB2	1.4	0.7	1.0	0.2	0.5	0.6	0.5	0.2	0.5
ALDH5A1	1.4	0.6	0.7	0.2	0.2	0.5	0.8	0.1	0.7
PTCD2	1.6	0.6	0.9	0.1	0.2	0.3	1.1	0.1	0.7
LOC101927472	1.6	0.3	0.5	0.2	0.2	0.3	1.6	0.1	0.7
SLC25A40	1.4	0.5	0.7	0.1	0.2	0.3	1.1	0.1	1.0
ZNF503-AS1	1.5	0.4	0.7	0.2	0.2	0.2	1.5	0.1	0.7
SP4	1.9	0.6	0.7	0.1	0.2	0.4	0.6	0.1	0.8
RAB30-AS1	1.4	0.5	0.6	0.0	0.2	0.3	1.5	0.1	0.8
ARHGEF35	1.7	0.4	0.7	0.0	0.1	0.3	1.2	0.2	0.8
C17orf72	1.8	0.7	0.7	0.0	0.3	0.2	0.8	0.2	0.6
LOC101928994	1.6	0.4	0.7	0.1	0.2	0.2	1.3	0.1	0.7
CES3	1.8	0.8	0.9	0.2	0.4	0.3	0.8	0.1	0.2
TUBB2B	1.6	0.7	0.9	0.3	0.5	0.4	0.2	0.0	0.6
GLI2	1.2	0.3	0.5	0.1	0.2	0.3	1.2	0.3	1.2
MGC12916	1.0	0.4	0.7	0.2	0.6	0.6	1.0	0.2	0.7
LOC101927798	1.5	0.3	0.9	0.2	0.3	0.1	1.4	0.1	0.5
RNF180	1.8	0.6	0.7	0.0	0.3	0.4	0.8	0.1	0.7
PLEKHM3	1.6	0.5	0.6	0.1	0.4	0.3	1.0	0.2	0.8
TAPBPL	1.7	0.6	0.7	0.2	0.3	0.4	1.0	0.1	0.3
LOC100289230	1.9	0.5	0.9	0.1	0.2	0.4	0.8	0.1	0.6
CEL	1.7	0.6	0.8	0.2	0.3	0.3	0.7	0.1	0.6
ERC2-IT1	1.8	0.4	0.7	0.1	0.3	0.2	1.2	0.1	0.6
ZNF260	1.4	0.6	0.8	0.0	0.3	0.2	0.7	0.1	1.2
MAGI3	1.7	0.6	0.7	0.1	0.3	0.4	0.8	0.1	0.7
ABI3BP	1.5	0.6	0.7	0.1	0.3	0.4	0.7	0.1	0.9
RAPGEF3	1.4	0.4	0.8	0.1	0.2	0.4	1.2	0.2	0.6
MOSPD2	1.3	0.5	0.8	0.0	0.1	0.4	1.3	0.1	0.9
SPRYD4	1.4	0.6	0.6	0.1	0.4	0.4	1.0	0.1	0.6
PCTP	1.4	0.9	0.8	0.0	0.4	0.3	0.9	0.1	0.4
LOC101927430	1.8	0.2	0.8	0.1	0.3	0.2	1.2	0.1	0.5
GNMT	1.5	0.3	0.5	0.3	0.3	0.6	0.8	0.1	0.9
ZNF17	1.5	0.6	0.7	0.0	0.3	0.3	1.0	0.1	0.7
KPNA5	1.3	0.4	0.9	0.0	0.2	0.4	1.2	0.1	0.8
SETDB2	1.4	0.5	0.6	0.1	0.2	0.2	1.4	0.1	0.8
SMIM10	1.5	0.5	0.7	0.3	0.2	0.5	0.9	0.1	0.6
C7orf55-LUC7L2	1.7	0.6	1.1	0.1	0.3	0.3	0.6	0.1	0.4
OVOS2	1.8	0.4	0.7	0.1	0.2	0.4	1.2	0.0	0.6
LOC101927930	1.0	0.5	0.9	0.1	0.2	0.2	1.3	0.1	1.2
LOC101927088	2.0	0.5	0.8	0.1	0.2	0.3	0.8	0.2	0.6

TBC1D3H	1.5	0.7	0.9	0.1	0.3	0.3	0.7	0.2	0.5
ATOH8	1.5	0.7	0.6	0.5	0.5	0.8	0.3	0.1	0.4
LOC728392	1.4	0.7	0.9	0.1	0.3	0.5	0.8	0.1	0.6
LOC101927608	0.6	1.1	0.4	1.6	0.2	0.0	0.4	0.5	0.4
LOC728463	2.0	0.6	0.7	0.1	0.3	0.5	0.6	0.1	0.5
ZNF397	1.4	0.5	1.1	0.1	0.2	0.4	0.8	0.1	0.7
BOC	1.4	0.6	0.8	0.2	0.3	0.5	0.8	0.2	0.6
ZNF354B	1.3	0.4	0.6	0.0	0.3	0.2	1.2	0.2	1.0
AMT	1.5	0.8	1.1	0.2	0.4	0.5	0.5	0.1	0.4
OIP5	1.4	0.3	1.0	0.1	0.3	0.5	0.8	0.2	0.7
C6orf203	1.7	0.7	0.8	0.0	0.3	0.6	0.6	0.1	0.5
SCAI	1.2	0.5	0.8	0.1	0.3	0.3	1.3	0.1	0.8
PRR22	1.5	0.5	0.9	0.2	0.5	0.4	0.7	0.1	0.6
ZNF252P-AS1	1.7	0.3	0.8	0.1	0.2	0.3	1.2	0.1	0.6
PDZD2	1.2	0.4	0.6	0.1	0.2	0.2	1.3	0.3	1.0
TMCC2	1.6	0.5	0.8	0.2	0.4	0.6	0.7	0.1	0.4
LOC101926918	0.9	0.6	1.3	0.0	0.3	0.8	0.7	0.0	0.7
ZNF575	1.0	0.5	0.5	0.5	0.5	0.6	0.8	0.3	0.6
LMTK3	1.6	0.7	0.7	0.2	0.3	0.3	0.8	0.1	0.5
KBTBD7	1.8	0.4	0.9	0.2	0.2	0.3	0.6	0.1	0.6
KDELC1	1.0	0.4	0.8	0.0	0.1	0.2	1.7	0.2	0.8
MGAT5B	1.3	0.5	0.5	0.2	0.6	0.6	0.8	0.1	0.6
SPINT1	2.1	0.6	1.1	0.1	0.1	0.2	0.5	0.1	0.5
PIK3C2B	2.6	0.7	1.0	0.1	0.2	0.2	0.2	0.0	0.2
CDC45	0.8	0.4	0.4	0.3	0.6	0.6	1.1	0.2	0.9
PARP9	1.9	0.7	1.0	0.1	0.3	0.3	0.6	0.1	0.4
CXorf23	1.7	0.5	0.9	0.1	0.3	0.2	0.9	0.1	0.6
CALML4	1.1	0.6	0.6	0.1	0.3	0.5	0.9	0.2	1.1
LOC101927497	1.1	0.3	0.7	0.0	0.2	0.3	1.6	0.0	1.0
LOC101926920	1.9	0.7	0.6	0.1	0.3	0.4	0.6	0.1	0.8
CCDC62	1.5	0.4	0.8	0.1	0.1	0.2	1.2	0.1	0.8
LOC101928762	1.6	0.6	0.8	0.2	0.4	0.5	0.8	0.1	0.3
LOC101928954	1.9	0.3	0.6	0.1	0.2	0.2	1.3	0.1	0.5
LIFR-AS1	1.7	0.5	0.7	0.0	0.3	0.3	1.0	0.1	0.7
C9orf72	1.2	0.4	0.8	0.0	0.2	0.2	1.3	0.1	1.0
NPPA-AS1	1.7	0.5	1.2	0.0	0.1	0.2	0.9	0.1	0.5
KLRC3	1.8	0.5	1.6	0.1	0.2	0.4	0.3	0.1	0.2
LOC101927352	1.8	0.3	0.6	0.1	0.2	0.2	1.4	0.2	0.4
HPSE	1.4	0.3	0.7	0.1	0.3	0.4	1.1	0.2	0.7
HMSD	1.6	0.5	0.3	0.0	0.3	0.1	1.0	0.2	1.2
LOC284757	1.7	0.3	0.8	0.1	0.2	0.2	1.3	0.1	0.6
LOC101929340	1.9	0.5	1.0	0.1	0.2	0.2	0.8	0.1	0.4
PTCH1	1.0	0.3	0.5	0.2	0.4	0.5	1.1	0.2	1.0
PDIK1L	1.3	0.3	0.5	0.1	0.4	0.2	1.3	0.3	0.8
LOC101927064	1.3	0.6	0.9	0.2	0.3	0.5	0.7	0.2	0.6
SP6	0.9	0.4	0.3	0.1	0.4	0.4	1.1	0.1	1.5
CNGA1	1.8	0.4	0.7	0.1	0.2	0.3	1.2	0.1	0.6
MAMSTR	1.2	0.5	0.6	0.2	0.3	0.6	1.0	0.2	0.5
LOC101928335	1.7	0.4	0.9	0.1	0.2	0.2	1.2	0.1	0.5
KIAA2026	1.7	0.5	0.7	0.0	0.1	0.3	0.9	0.2	0.8
FAM135A	1.5	0.6	0.7	0.0	0.1	0.3	1.0	0.1	0.9
LOC101929188	1.5	0.7	0.8	0.1	0.2	0.3	0.9	0.1	0.7
HARBI1	1.4	0.6	0.9	0.0	0.4	0.3	0.8	0.1	0.8
GP1BA	1.4	0.5	0.8	0.2	0.4	0.4	0.7	0.1	0.6
ZNF718	1.6	0.3	0.7	0.0	0.2	0.2	1.1	0.0	1.0

SLC25A12	1.5	0.5	0.7	0.1	0.3	0.3	0.8	0.1	0.8
LOC101928694	1.9	0.4	0.6	0.1	0.2	0.3	1.2	0.1	0.4
KIF18A	1.5	0.4	0.6	0.0	0.3	0.3	1.3	0.1	0.7
LINC00939	1.7	0.3	0.7	0.2	0.2	0.4	1.1	0.1	0.5
NAPB	1.1	0.3	1.0	0.1	0.5	0.3	1.0	0.2	0.8
MNX1	1.0	0.7	0.5	0.2	0.4	0.4	1.1	0.2	0.8
GALNT13	1.3	0.4	0.6	0.0	0.1	0.2	1.5	0.2	0.9
ANKEF1	1.8	0.7	0.9	0.0	0.2	0.3	0.8	0.1	0.5
DLEU7-AS1	1.9	0.4	0.6	0.1	0.2	0.2	1.2	0.0	0.6
LOC643339	2.2	0.1	1.0	0.1	0.2	0.2	0.9	0.1	0.4
ZNF140	1.5	0.4	0.8	0.1	0.2	0.3	1.1	0.1	0.7
TTC26	1.7	0.7	0.6	0.0	0.2	0.3	0.8	0.1	0.7
PIK3CG	1.5	0.4	0.5	0.1	0.2	0.4	1.2	0.1	0.7
ZKSCAN4	1.6	0.7	0.7	0.1	0.2	0.3	0.9	0.2	0.5
C12orf4	1.5	0.7	1.0	0.1	0.1	0.4	0.8	0.1	0.6
ZNF439	1.3	0.4	0.5	0.1	0.2	0.2	1.6	0.1	0.8
NDRG4	1.4	0.6	0.7	0.2	0.3	0.3	1.1	0.1	0.6
DZANK1	2.0	0.5	0.7	0.1	0.2	0.3	0.8	0.0	0.5
NIPAL2	1.2	0.5	0.7	0.1	0.3	0.5	1.0	0.2	0.7
ZNF416	1.4	0.4	0.6	0.1	0.2	0.4	1.1	0.2	0.8
OVGP1	1.9	0.4	0.7	0.0	0.0	0.1	1.4	0.0	0.5
SPATA21	1.8	0.3	0.7	0.1	0.3	0.1	1.3	0.2	0.5
TSSK6	1.5	0.5	0.7	0.2	0.4	0.2	1.0	0.1	0.6
KIAA0087	1.6	0.3	0.7	0.1	0.2	0.2	1.5	0.1	0.5
CCDC112	1.3	0.6	0.7	0.0	0.2	0.3	1.0	0.2	0.8
PDK1	1.5	0.6	0.7	0.1	0.3	0.4	0.8	0.1	0.7
FAM214A	1.7	0.6	0.7	0.0	0.1	0.2	0.8	0.1	0.8
ATP7A	1.7	0.5	0.8	0.0	0.2	0.3	0.9	0.1	0.6
LMF1	1.2	0.6	0.9	0.4	0.5	0.5	0.7	0.1	0.3
LOC400768	1.8	0.2	0.8	0.1	0.3	0.2	1.1	0.1	0.6
SNORA60	0.6	1.0	0.6	0.1	0.6	0.6	0.6	0.4	0.6
POLA1	1.2	0.4	0.6	0.1	0.2	0.3	1.3	0.1	0.9
LOC101928895	1.8	0.3	0.8	0.2	0.4	0.4	0.7	0.1	0.4
PRKCH	1.6	0.7	1.0	0.1	0.2	0.2	0.6	0.1	0.5
LOC101927758	1.3	0.7	1.1	0.2	0.2	0.3	0.8	0.1	0.6
LOC101929319	1.7	0.3	0.7	0.2	0.2	0.3	1.2	0.1	0.6
SYNE1	1.4	0.4	0.6	0.1	0.2	0.3	1.3	0.1	0.8
HCG25	1.8	0.3	0.7	0.0	0.2	0.8	0.6	0.2	0.4
CEP85L	1.6	0.4	0.7	0.1	0.2	0.3	1.2	0.1	0.6
RTEL1	1.7	0.3	0.6	0.4	0.1	0.1	1.2	0.0	0.7
LOC101926892	1.8	0.3	0.9	0.1	0.2	0.3	1.1	0.1	0.4
LOC100996574	1.8	0.5	0.9	0.1	0.2	0.2	0.8	0.1	0.6
GSTCD	1.7	0.6	0.7	0.1	0.2	0.3	0.8	0.1	0.7
TFR2	1.0	0.5	0.4	0.2	0.3	0.5	1.0	0.2	0.9
LIAS	1.0	0.5	0.7	0.1	0.3	0.2	1.4	0.1	0.8
SAA1	2.1	0.6	0.8	0.1	0.2	0.2	0.6	0.1	0.6
ZNF254	1.4	0.6	0.9	0.0	0.2	0.2	1.0	0.0	0.7
DLGAP1-AS1	1.6	0.5	0.8	0.1	0.4	0.3	0.6	0.3	0.6
IKZF4	1.4	0.6	0.9	0.1	0.3	0.5	0.8	0.1	0.4
ZNF425	1.7	0.3	0.5	0.1	0.2	0.3	1.0	0.2	0.8
LOC646652	1.8	0.2	0.8	0.1	0.3	0.3	1.1	0.0	0.4
SLC2A1-AS1	1.6	0.4	0.6	0.2	0.1	0.3	1.4	0.1	0.4
TMEM187	1.3	0.4	0.7	0.3	0.1	0.6	1.1	0.1	0.6
LOC100505502	2.0	0.3	0.6	0.1	0.2	0.2	1.2	0.0	0.5
FUT2	1.5	0.5	0.7	0.3	0.4	0.2	1.1	0.2	0.4

MFI2-AS1	1.2	0.4	0.7	0.2	0.6	0.4	0.9	0.1	0.4
CEP135	1.3	0.5	0.6	0.0	0.1	0.2	1.1	0.1	1.0
FGF13-AS1	1.9	0.4	0.6	0.2	0.3	0.3	0.9	0.2	0.4
LOC101929182	1.4	0.5	0.6	0.1	0.3	0.3	1.0	0.2	0.7
TTC39C	1.4	0.5	0.9	0.1	0.3	0.2	1.1	0.1	0.5
DYNLL1-AS1	1.5	0.6	0.7	0.1	0.2	0.3	0.8	0.2	0.6
CEP70	1.5	0.6	0.8	0.1	0.3	0.4	0.7	0.0	0.7
LRP4	1.3	0.5	0.7	0.1	0.3	0.4	1.1	0.2	0.6
LOC100287036	1.9	0.2	0.8	0.2	0.3	0.7	0.4	0.1	0.5
SLC25A20	1.4	0.6	0.9	0.1	0.1	0.5	0.9	0.1	0.4
HIP1	1.7	0.5	0.8	0.1	0.3	0.4	0.8	0.1	0.6
OVCH2	1.5	0.4	0.6	0.0	0.2	0.3	1.2	0.1	0.8
TTC21B	1.4	0.4	0.8	0.1	0.2	0.2	1.0	0.1	0.8
BBS12	1.4	0.6	0.9	0.0	0.2	0.3	0.8	0.1	0.8
CTCFL	1.8	0.3	0.7	0.1	0.3	0.3	0.9	0.1	0.6
MESTIT1	1.5	0.4	0.8	0.1	0.2	0.3	1.1	0.1	0.6
KIF17	0.7	0.3	0.5	0.2	0.2	0.1	1.4	0.3	1.3
PCDHB12	2.1	0.4	0.8	0.2	0.3	0.2	0.6	0.1	0.4
CCDC66	1.4	0.4	0.7	0.1	0.2	0.2	1.1	0.1	1.0
DMXL2	1.7	0.6	0.8	0.0	0.2	0.3	0.7	0.1	0.7
NHLRC3	1.5	0.5	0.7	0.0	0.3	0.5	1.0	0.1	0.5
LINC01001	1.2	0.7	0.7	0.2	0.4	0.3	0.8	0.1	0.6
EPS8L1	1.7	0.4	0.8	0.1	0.3	0.1	0.9	0.1	0.6
ZNF124	1.2	0.4	0.7	0.2	0.3	0.4	1.0	0.1	0.9
FLJ20021	1.6	0.4	0.8	0.1	0.3	0.6	0.5	0.2	0.6
MAP3K14-AS1	1.2	0.6	1.1	0.1	0.3	0.2	1.0	0.1	0.5
ZBTB3	1.1	0.7	0.9	0.2	0.3	0.3	0.7	0.2	0.6
DNAH11	1.4	0.5	0.6	0.1	0.2	0.3	1.0	0.1	0.7
TIMP4	0.7	0.5	0.4	0.0	0.2	0.4	1.3	0.4	1.1
NUF2	1.2	0.4	0.6	0.0	0.6	0.3	1.2	0.2	0.6
RBAK-RBAKDN	0.6	0.3	1.2	0.1	0.1	0.4	1.7	0.2	0.4
F2RL2	1.1	0.5	0.6	0.1	0.5	0.4	1.0	0.2	0.7
LOC644554	2.4	0.2	0.7	0.0	0.1	0.2	0.9	0.0	0.5
AVIL	1.7	0.5	1.1	0.1	0.3	0.2	0.6	0.1	0.5
CYP2R1	1.5	0.5	0.7	0.1	0.3	0.4	0.7	0.2	0.7
CKAP2L	1.2	0.5	0.6	0.1	0.3	0.5	0.9	0.1	0.8
GDAP1	1.3	0.4	0.6	0.2	0.3	0.4	1.0	0.1	0.6
THUMPD2	1.4	0.4	0.6	0.1	0.2	0.2	1.0	0.1	1.0
LOC100133077	1.6	0.3	0.7	0.2	0.2	0.2	1.3	0.1	0.5
GLMN	1.2	0.5	0.8	0.0	0.1	0.2	1.1	0.1	1.0
DACH1	1.3	0.5	0.8	0.2	0.3	0.3	0.8	0.2	0.8
ZNF37A	1.0	0.5	0.4	0.1	0.3	0.3	1.2	0.2	1.0
ADAMTS9	1.6	0.5	0.8	0.1	0.3	0.5	0.6	0.1	0.4
CLTCL1	1.3	0.4	0.7	0.1	0.3	0.5	0.9	0.1	0.7
HOXB-AS3	1.4	0.6	0.6	0.1	0.0	0.3	1.1	0.2	0.8
LOC100996573	1.2	0.4	0.9	0.2	0.2	0.3	1.0	0.2	0.6
LOC101928083	1.3	0.5	0.6	0.1	0.2	0.3	1.2	0.2	0.8
AP4E1	1.6	0.5	0.8	0.0	0.1	0.2	1.1	0.1	0.7
PPFIBP2	2.2	0.6	0.8	0.1	0.2	0.3	0.5	0.1	0.3
LOC101929670	1.8	0.3	0.8	0.1	0.3	0.2	1.0	0.0	0.4
TMEM44-AS1	1.5	0.3	0.7	0.1	0.2	0.3	1.1	0.2	0.6
LOC101928632	1.5	0.3	0.7	0.1	0.2	0.3	1.3	0.0	0.4
NOX4	1.7	0.3	0.7	0.0	0.2	0.2	1.3	0.1	0.5
ADPRHL1	1.6	0.7	0.9	0.1	0.1	0.2	0.9	0.1	0.4
ZNF552	1.5	0.5	0.5	0.1	0.2	0.3	1.2	0.1	0.6

NHEG1	1.7	0.3	0.6	0.1	0.3	0.2	1.3	0.1	0.5
SLC27A5	1.5	0.5	0.7	0.2	0.2	0.2	0.8	0.2	0.6
SLC35D1	1.4	0.4	0.6	0.1	0.4	0.1	1.2	0.1	0.8
LOC101927136	1.8	0.4	0.7	0.1	0.3	0.3	0.9	0.1	0.5
IFFO1	1.4	0.5	0.6	0.1	0.2	0.3	1.0	0.2	0.8
TUBD1	1.4	0.2	0.7	0.1	0.2	0.3	1.1	0.0	1.1
LOC283045	1.7	0.4	0.8	0.1	0.3	0.2	0.9	0.1	0.6
MORC2-AS1	1.4	0.4	0.7	0.1	0.2	0.2	1.2	0.0	0.7
LOC143666	1.2	0.7	0.8	0.1	0.2	0.2	0.8	0.2	0.6
NUAK2	1.4	0.5	0.7	0.1	0.3	0.4	0.7	0.2	0.7
ZYG11A	1.2	0.5	0.9	0.1	0.3	0.1	1.0	0.1	0.7
RAD54L	1.2	0.3	0.5	0.1	0.3	0.4	1.2	0.2	0.8
XYLB	1.1	0.4	0.7	0.2	0.2	0.4	1.0	0.2	0.9
ITGB3BP	1.5	0.7	0.8	0.1	0.3	0.2	0.6	0.1	0.8
LOC285500	1.6	0.3	0.9	0.2	0.2	0.3	0.8	0.3	0.3
MUC5AC	2.0	0.5	0.8	0.3	0.6	0.5	0.3	0.0	0.1
CCNE1	1.5	0.7	0.8	0.2	0.3	0.3	0.7	0.2	0.4
ZNF280D	1.9	0.4	1.0	0.1	0.2	0.2	0.6	0.0	0.5
NOD1	1.4	0.5	0.6	0.1	0.3	0.5	0.8	0.1	0.5
SLC35E3	1.1	0.5	0.6	0.1	0.3	0.6	0.9	0.2	0.8
TOPORS-AS1	1.0	0.4	0.9	0.1	0.6	0.2	0.9	0.2	0.6
TREX1	1.2	0.5	0.5	0.3	0.4	0.6	0.7	0.2	0.4
DDB2	1.1	0.5	0.8	0.1	0.4	0.4	0.8	0.1	0.8
LOC101929166	1.7	0.4	0.8	0.1	0.0	0.7	0.7	0.1	0.5
ARSA	1.4	0.5	0.6	0.2	0.4	0.5	0.6	0.2	0.5
CLMN	1.1	0.5	0.6	0.1	0.3	0.5	0.9	0.1	0.8
ZFP112	1.6	0.5	0.6	0.0	0.3	0.4	0.7	0.1	0.8
LOC101928958	1.6	0.3	0.7	0.1	0.4	0.3	0.9	0.1	0.6
SNORA1	0.6	0.8	0.9	0.0	0.3	0.2	1.1	0.2	0.8
ANKDD1A	1.4	0.3	0.9	0.1	0.2	0.3	1.0	0.1	0.5
DUX4L9	1.3	0.3	0.6	0.2	0.3	0.3	1.2	0.1	0.7
PIK3AP1	1.9	0.5	0.9	0.1	0.3	0.2	0.6	0.1	0.4
ATP6V0E2-AS1	0.7	0.3	0.5	0.1	0.2	0.3	1.8	0.1	0.9
ETFDH	1.5	0.6	0.8	0.0	0.3	0.4	0.8	0.1	0.4
CNTNAP3	1.9	0.6	0.9	0.1	0.2	0.4	0.5	0.1	0.2
CITED1	1.7	0.5	0.6	0.0	0.1	0.1	1.1	0.2	0.5
FAM198A	1.6	0.5	0.8	0.1	0.2	0.3	0.9	0.1	0.5
AGAP4	1.6	0.4	0.7	0.2	0.3	0.2	0.8	0.1	0.5
KCNMB3	1.4	0.5	0.8	0.1	0.1	0.3	0.8	0.1	0.8
MFSD2A	0.5	0.3	0.6	0.0	0.1	0.2	1.4	0.3	1.5
EPM2A	1.9	0.5	0.5	0.1	0.2	0.4	0.6	0.1	0.6
MTM1	1.6	0.6	0.5	0.1	0.2	0.2	1.0	0.2	0.6
LINGO2	1.8	0.4	0.7	0.0	0.3	0.4	0.6	0.1	0.5
C10orf118	1.5	0.5	0.6	0.0	0.2	0.2	0.9	0.1	0.7
GSDMB	1.7	0.7	0.7	0.0	0.2	0.1	0.9	0.1	0.5
TRNAG8	1.1	0.6	0.4	0.5	0.3	0.3	0.8	0.2	0.8
TRNAG9	1.1	0.6	0.4	0.5	0.3	0.3	0.8	0.2	0.8
TRNAG17	1.1	0.6	0.4	0.5	0.3	0.3	0.8	0.2	0.8
TRNAG13	1.1	0.6	0.4	0.5	0.3	0.3	0.8	0.2	0.8
TRNAG24	1.1	0.6	0.4	0.5	0.3	0.3	0.8	0.2	0.8
TRNAG11	1.1	0.6	0.4	0.5	0.3	0.3	0.8	0.2	0.8
HSD3B7	1.5	0.7	1.1	0.1	0.3	0.3	0.4	0.1	0.3
WDR5B	0.9	0.4	0.7	0.0	0.3	0.4	1.2	0.1	0.8
CCDC157	1.5	0.4	0.8	0.2	0.3	0.3	0.7	0.2	0.5
FAM50B	1.4	0.5	0.8	0.2	0.3	0.5	0.7	0.1	0.4

PLA2G4C	1.5	0.8	1.1	0.1	0.2	0.4	0.4	0.0	0.3
DGCR11	1.5	0.2	0.6	0.1	0.2	0.3	1.3	0.1	0.6
ZNF618	1.4	0.5	0.8	0.1	0.3	0.3	0.8	0.2	0.4
LDLRAD2	1.7	0.7	0.6	0.2	0.4	0.3	0.5	0.1	0.5
ZNF611	1.3	0.6	0.7	0.1	0.2	0.3	1.0	0.1	0.6
HCCAT3	1.2	0.3	0.9	0.1	0.2	0.3	1.3	0.1	0.5
PCLO	1.5	0.4	0.6	0.1	0.2	0.3	0.8	0.1	0.7
LOC101928076	1.0	0.2	0.6	0.1	0.0	0.4	1.5	0.1	0.9
KCNMB4	0.6	0.8	0.8	0.4	0.3	0.2	0.8	0.4	0.4
TSSK4	1.6	0.4	0.7	0.1	0.1	0.3	0.9	0.0	0.6
PDE2A	1.4	0.7	0.7	0.2	0.2	0.4	0.5	0.1	0.4
LOC100652736	1.4	0.3	0.7	0.1	0.3	0.3	0.9	0.1	0.7
RPGR	1.2	0.5	0.5	0.1	0.2	0.3	1.0	0.2	0.8
LOC100996681	1.9	0.3	0.6	0.1	0.3	0.2	0.9	0.0	0.5
LOC101928381	1.6	0.3	0.8	0.1	0.2	0.1	1.1	0.1	0.4
TNFSF10	1.6	0.4	0.8	0.0	0.2	0.2	1.1	0.1	0.4
INTU	1.8	0.5	0.8	0.0	0.2	0.3	0.6	0.1	0.5
C2orf27A	1.7	0.4	0.9	0.1	0.2	0.1	0.5	0.1	0.9
C20orf196	1.6	0.8	0.9	0.0	0.1	0.3	0.6	0.0	0.4
CENPJ	1.4	0.5	0.6	0.1	0.3	0.3	0.9	0.1	0.7
CYP2D6	1.5	0.7	0.8	0.2	0.3	0.4	0.3	0.1	0.4
DIXDC1	1.4	0.6	1.0	0.1	0.3	0.3	0.4	0.1	0.6
CCDC103	1.3	0.5	0.9	0.2	0.4	0.3	0.5	0.2	0.5
LOC101927877	1.6	0.3	0.7	0.1	0.2	0.2	1.1	0.1	0.6
ZNF816	1.2	0.6	0.6	0.1	0.3	0.3	1.0	0.1	0.6
CHMP4C	1.4	0.6	0.9	0.1	0.1	0.1	0.8	0.0	0.6
NBEAL1	1.7	0.4	0.6	0.0	0.2	0.3	0.8	0.1	0.7
LINC00629	1.7	0.4	0.7	0.0	0.1	0.3	0.9	0.1	0.6
SLC10A7	1.1	0.4	0.5	0.1	0.2	0.2	0.9	0.2	1.1
ZFP82	1.1	0.4	0.6	0.0	0.2	0.2	1.4	0.0	0.7
LOC101927775	1.5	0.4	0.7	0.0	0.2	0.2	1.2	0.1	0.4
RBM26-AS1	1.4	0.3	0.7	0.1	0.2	0.2	1.3	0.1	0.5
CHAF1B	1.2	0.5	0.5	0.1	0.2	0.3	0.9	0.3	0.7
ZNF432	1.6	0.5	0.8	0.1	0.1	0.3	0.8	0.2	0.5
JAM2	1.5	0.4	0.7	0.1	0.2	0.2	1.2	0.1	0.6
KRTAP2-3	0.4	0.2	0.4	0.1	0.9	0.8	1.2	0.1	0.5
FAM157A	1.4	0.4	0.4	0.3	0.5	0.4	0.7	0.3	0.3
NYNRIN	1.6	0.6	0.8	0.1	0.1	0.2	0.6	0.1	0.6
IQCD	1.0	0.5	0.8	0.1	0.5	0.4	0.6	0.2	0.5
IGKV1D-43	1.5	0.8	0.4	0.1	0.0	0.5	0.5	0.4	0.5
SLC26A11	1.8	0.8	1.0	0.1	0.2	0.2	0.3	0.0	0.4
ZNF267	1.2	0.4	0.8	0.1	0.3	0.3	0.8	0.1	0.8
EHHADH	1.6	0.6	0.7	0.1	0.2	0.3	0.6	0.1	0.5
SPAG4	1.6	0.6	0.9	0.2	0.1	0.3	0.7	0.1	0.4
C21orf37	1.6	0.3	0.8	0.0	0.2	0.4	0.9	0.2	0.3
LOC101927260	1.3	0.5	0.6	0.1	0.3	0.2	0.8	0.1	0.7
LOC100270746	1.5	0.2	0.7	0.1	0.3	0.3	1.1	0.1	0.3
ACOT4	1.1	0.4	0.8	0.2	0.2	0.4	0.9	0.1	0.5
PASK	1.2	0.5	0.7	0.1	0.2	0.2	1.1	0.2	0.6
DUSP18	1.2	0.6	0.4	0.1	0.5	0.3	0.7	0.2	0.6
HIC2	1.2	0.5	0.7	0.2	0.3	0.4	0.7	0.1	0.6
POP1	1.0	0.4	0.5	0.1	0.3	0.3	1.3	0.1	0.7
GPT	1.4	0.5	0.8	0.2	0.4	0.3	0.5	0.1	0.5
ZNF600	1.3	0.6	0.8	0.0	0.2	0.3	1.0	0.1	0.5
BOK-AS1	1.5	0.5	0.5	0.3	0.3	0.3	0.8	0.1	0.5

SHCBP1	0.9	0.3	0.6	0.1	0.2	0.2	1.3	0.1	0.9
CCP110	1.3	0.5	0.7	0.0	0.2	0.3	0.8	0.2	0.7
LOC101928155	1.9	0.5	0.7	0.1	0.3	0.2	0.5	0.0	0.5
LOC101927509	1.4	0.5	0.9	0.0	0.3	0.2	0.7	0.1	0.5
TIGD2	1.1	0.4	0.5	0.1	0.3	0.2	1.1	0.3	0.8
CPLX1	1.3	0.8	0.5	0.3	0.1	0.2	0.8	0.1	0.5
LINC00265	1.3	0.5	0.6	0.2	0.2	0.3	0.9	0.1	0.6
AMPD3	1.4	0.5	0.9	0.1	0.3	0.3	0.6	0.1	0.4
LOC101928054	1.7	0.3	0.8	0.0	0.2	0.3	0.7	0.1	0.6
NOS1AP	1.9	0.6	0.5	0.1	0.3	0.3	0.6	0.1	0.4
LOC101928947	1.6	0.4	0.7	0.1	0.3	0.2	0.9	0.1	0.6
ING3	1.1	0.4	0.6	0.0	0.3	0.3	1.1	0.0	0.9
ADAM19	1.1	0.7	0.6	0.1	0.4	0.4	0.8	0.1	0.6
CGB7	1.5	0.6	1.0	0.3	0.2	0.2	0.4	0.0	0.4
LYSMD1	1.4	0.6	0.5	0.0	0.2	0.3	0.9	0.1	0.5
LOC101928901	1.4	0.4	0.5	0.1	0.2	0.2	1.1	0.2	0.5
PCDHB10	1.7	0.4	1.0	0.2	0.3	0.3	0.5	0.1	0.3
FAM13A-AS1	1.5	0.5	0.7	0.1	0.2	0.2	0.8	0.1	0.6
MYH3	1.3	0.5	0.7	0.1	0.3	0.4	0.7	0.0	0.5
SRBD1	1.3	0.4	0.6	0.1	0.2	0.4	0.9	0.1	0.7
WDR52	1.6	0.5	0.6	0.1	0.2	0.2	0.8	0.1	0.6
LOC101927122	1.6	0.2	0.8	0.1	0.2	0.1	1.1	0.0	0.6
LINC00470	1.6	0.2	0.7	0.0	0.1	0.2	1.1	0.1	0.5
CCDC40	1.7	0.4	1.0	0.1	0.3	0.3	0.4	0.1	0.3
CELF6	1.1	0.3	0.8	0.2	0.2	0.3	0.8	0.2	0.7
LOC286177	1.6	0.4	0.5	0.1	0.2	0.2	1.1	0.1	0.4
CCDC17	1.5	0.4	0.8	0.1	0.4	0.3	0.6	0.0	0.5
LOC100507376	1.1	0.6	0.4	0.1	0.1	0.4	1.3	0.1	0.6
LOC401557	1.6	0.3	0.6	0.1	0.3	0.4	0.7	0.1	0.4
LOC101927047	1.5	0.4	0.6	0.1	0.3	0.3	0.8	0.1	0.6
P2RY2	0.9	0.5	0.5	0.1	0.2	0.2	1.0	0.3	0.9
PRSS27	1.6	0.7	0.5	0.3	0.4	0.3	0.3	0.2	0.4
LOC101927018	1.4	0.4	0.6	0.0	0.3	0.2	1.1	0.1	0.5
ZNF714	1.6	0.3	0.6	0.1	0.2	0.2	0.9	0.2	0.6
SMPDL3A	0.7	0.4	1.0	0.1	0.4	0.3	0.9	0.2	0.6
LINC00938	1.2	0.4	0.6	0.1	0.2	0.4	1.0	0.1	0.5
WDFY3-AS2	1.4	0.4	0.8	0.2	0.2	0.4	0.4	0.1	0.7
SEMA4G	1.2	0.5	0.7	0.1	0.3	0.5	0.6	0.1	0.6
TMEM110-MUSTN1	1.7	0.4	0.5	0.1	0.2	0.4	1.0	0.0	0.4
LOC730101	0.8	0.3	0.4	0.1	0.1	0.3	1.4	0.2	1.1
C1orf233	1.6	0.5	0.5	0.5	0.4	0.8	0.2	0.1	0.0
KCNV2	1.7	0.2	0.8	0.2	0.3	0.4	0.7	0.1	0.2
LOC100507420	0.8	0.3	0.4	0.2	0.4	0.4	1.3	0.0	0.9
ESPL1	1.1	0.4	0.6	0.2	0.3	0.3	1.0	0.2	0.6
LOC100287896	1.4	0.5	0.8	0.1	0.1	0.2	0.8	0.1	0.6
IFT172	1.6	0.5	0.6	0.1	0.3	0.3	0.6	0.1	0.4
LRRC37A	2.4	0.2	0.5	0.0	0.1	0.2	0.4	0.1	0.7
RWDD3	1.4	0.6	0.9	0.1	0.3	0.2	0.5	0.1	0.5
ZNF440	1.3	0.5	0.6	0.0	0.1	0.3	1.0	0.1	0.8
ID3	1.6	0.6	0.9	0.1	0.1	0.0	0.5	0.3	0.5
PIGL	1.3	0.8	0.5	0.1	0.3	0.3	0.7	0.1	0.6
LOC100506691	1.2	0.3	0.8	0.1	0.2	0.2	1.0	0.1	0.5
LOC101928356	0.9	0.2	1.0	0.1	0.3	0.5	0.6	0.0	0.9
LOC90462	1.2	0.4	0.6	0.1	0.3	0.4	0.6	0.1	0.9
ZNF649	1.6	0.4	0.7	0.1	0.2	0.4	0.6	0.1	0.5

SLITRK2	1.4	0.6	0.8	0.1	0.3	0.3	0.6	0.1	0.6
THNSL1	1.2	0.4	0.6	0.0	0.2	0.2	1.0	0.1	0.8
LOC101929140	1.6	0.3	0.7	0.1	0.2	0.2	0.9	0.1	0.5
LYSMD4	1.2	0.7	1.2	0.1	0.3	0.3	0.3	0.0	0.3
LOC101927699	1.2	0.3	0.5	0.1	0.2	0.3	0.8	0.2	1.0
C9orf9	1.3	0.5	0.7	0.2	0.2	0.5	0.7	0.1	0.4
ZDHHC23	1.0	0.4	0.5	0.1	0.2	0.2	1.1	0.1	0.9
SMIM5	1.6	0.2	0.8	0.1	0.2	0.2	1.0	0.1	0.4
MAN1A1	1.8	0.6	0.9	0.1	0.2	0.2	0.5	0.1	0.4
DMXL1	1.4	0.4	0.6	0.0	0.3	0.3	0.8	0.1	0.7
FERMT3	1.6	0.4	0.8	0.1	0.2	0.2	0.6	0.1	0.6
ARL17A	1.2	0.3	1.0	0.1	0.2	0.2	0.7	0.2	0.8
REL	1.8	0.5	0.7	0.0	0.2	0.2	0.6	0.2	0.5
ANKRD34A	0.8	0.3	0.7	0.1	0.3	0.4	1.1	0.2	0.6
ZNF518A	1.6	0.6	0.6	0.0	0.2	0.2	0.6	0.1	0.6
MEIS2	1.2	0.4	0.5	0.2	0.2	0.3	0.8	0.2	0.8
PARPBP	0.8	0.4	0.7	0.0	0.2	0.2	1.3	0.1	0.9
RAB3A	1.2	0.6	0.8	0.2	0.2	0.5	0.2	0.2	0.7
ZDHHC13	1.3	0.5	0.8	0.0	0.2	0.3	0.8	0.1	0.5
CCDC153	1.3	0.6	0.9	0.1	0.5	0.4	0.4	0.1	0.3
CAMK1D	1.4	0.5	0.7	0.2	0.3	0.4	0.6	0.0	0.4
ZNF41	1.4	0.5	0.6	0.1	0.1	0.3	1.0	0.1	0.6
ZNF44	1.3	0.4	0.8	0.0	0.2	0.3	0.8	0.0	0.7
PCOLCE	1.4	0.5	0.7	0.2	0.3	0.4	0.6	0.1	0.4
SSTR5-AS1	1.4	0.3	0.7	0.1	0.2	0.2	1.1	0.1	0.5
FBF1	1.4	0.4	0.7	0.2	0.3	0.5	0.5	0.1	0.3
OR5P3	1.5	0.3	0.5	0.0	0.2	0.3	1.1	0.0	0.8
LOC101927204	1.3	0.3	0.6	0.1	0.2	0.4	1.0	0.1	0.6
KLHL23	1.0	0.4	0.4	0.0	0.1	0.3	1.0	0.1	1.2
PHOSPHO2-KLHL23	1.0	0.4	0.4	0.0	0.1	0.3	1.0	0.1	1.2
CBR3	1.0	0.5	0.7	0.4	0.6	0.4	0.6	0.1	0.3
LINC00702	1.2	0.3	0.8	0.0	0.2	0.2	1.3	0.1	0.5
MCM10	0.9	0.3	0.4	0.0	0.3	0.4	1.0	0.2	1.0
LOC100129617	1.2	0.3	0.6	0.1	0.2	0.2	1.0	0.1	0.8
LOC101928680	1.3	0.8	0.6	0.4	0.3	0.3	0.5	0.0	0.3
FAM225B	1.5	0.2	0.8	0.2	0.2	0.3	0.8	0.2	0.4
LINC01024	1.2	0.4	0.7	0.1	0.3	0.2	1.0	0.1	0.5
RCBTB2	1.1	0.4	0.6	0.1	0.3	0.4	0.7	0.1	0.8
WBSCR27	0.8	0.8	0.6	0.0	0.3	0.5	0.9	0.0	0.5
LPCAT2	1.2	0.5	0.5	0.1	0.2	0.4	0.8	0.1	0.7
LOC100506321	1.2	0.4	0.8	0.1	0.2	0.2	1.0	0.0	0.6
VWA8	1.3	0.5	0.8	0.1	0.2	0.4	0.6	0.1	0.5
ZNF222	1.3	0.5	0.6	0.0	0.1	0.2	0.9	0.1	0.7
NPM2	1.3	0.3	0.7	0.1	0.1	0.2	0.7	0.4	0.7
LOC101927750	1.2	0.4	0.7	0.1	0.2	0.3	0.9	0.0	0.6
APOL3	1.3	0.5	0.8	0.2	0.3	0.3	0.6	0.0	0.5
LOC101060017	1.0	0.7	0.6	0.1	0.3	0.3	0.7	0.1	0.6
FAM74A4	1.5	0.3	0.4	0.1	0.3	0.2	0.9	0.1	0.6
ZBTB42	1.1	0.4	0.5	0.2	0.1	0.3	1.0	0.2	0.7
FSCN2	1.3	0.4	0.5	0.2	0.1	0.3	1.1	0.1	0.6
MEF2C	1.2	0.4	0.7	0.1	0.3	0.4	0.7	0.2	0.6
DEPTOR	1.2	0.4	0.6	0.2	0.4	0.5	0.6	0.1	0.4
TBXAS1	1.3	0.7	0.7	0.1	0.3	0.3	0.8	0.1	0.2
LOC100289098	1.5	0.4	0.5	0.3	0.2	0.3	0.7	0.1	0.5
ZNF594	1.1	0.4	0.4	0.0	0.4	0.3	1.0	0.1	0.7

TP53TG5	1.5	0.4	0.7	0.1	0.3	0.2	0.7	0.1	0.5
DET1	1.5	0.5	0.5	0.1	0.4	0.3	0.5	0.1	0.5
KCNK6	0.8	0.4	0.6	0.2	0.4	0.4	0.8	0.2	0.6
TNRC6C	1.4	0.5	0.6	0.1	0.2	0.3	0.6	0.1	0.6
LOC284578	1.6	0.3	0.7	0.0	0.3	0.3	0.6	0.1	0.5
ZNF620	0.7	0.4	0.5	0.0	0.2	0.2	1.2	0.2	1.2
LOC101927680	1.6	0.2	0.8	0.0	0.2	0.3	0.6	0.1	0.6
ZNF510	1.2	0.4	0.6	0.0	0.1	0.3	0.8	0.2	0.9
SLC30A4	1.3	0.3	0.7	0.0	0.1	0.3	1.0	0.1	0.6
DUS4L	1.2	0.5	0.6	0.1	0.3	0.4	0.9	0.1	0.6
CYP2S1	1.1	0.2	0.6	0.1	0.1	0.2	1.1	0.2	0.9
HVCN1	1.2	0.4	0.9	0.1	0.4	0.2	0.7	0.1	0.5
SYNJ1	1.4	0.4	0.6	0.1	0.1	0.3	0.8	0.1	0.6
POLE2	0.9	0.3	0.4	0.1	0.2	0.4	1.2	0.1	0.9
ASPHD2	1.1	0.4	0.6	0.1	0.4	0.4	0.6	0.2	0.6
HOXD3	1.1	0.4	0.6	0.2	0.2	0.3	0.8	0.2	0.6
C10orf35	1.5	0.4	0.6	0.1	0.0	0.3	0.9	0.1	0.6
ZFP69B	0.8	0.4	0.5	0.0	0.2	0.3	1.3	0.2	1.0
LOC728537	1.5	0.3	0.4	0.1	0.2	0.4	0.9	0.1	0.4
ACTA2	1.2	0.5	0.8	0.1	0.1	0.3	0.8	0.2	0.6
ZDBF2	1.0	0.4	0.5	0.0	0.2	0.2	1.1	0.1	0.8
PIWIL3	1.4	0.3	0.7	0.0	0.2	0.2	1.1	0.1	0.4
AK5	0.9	0.4	0.4	0.4	0.6	0.7	0.6	0.2	0.3
QRICH2	1.7	0.4	0.8	0.2	0.3	0.3	0.5	0.1	0.3
JAK2	1.7	0.6	0.7	0.0	0.2	0.2	0.6	0.1	0.4
BUB1B	0.8	0.5	0.6	0.1	0.2	0.4	0.9	0.2	0.7
RAD51AP1	1.4	0.3	0.6	0.1	0.2	0.1	0.9	0.1	0.7
PRRT1	1.4	0.7	0.9	0.2	0.3	0.4	0.4	0.1	0.2
SCNN1D	1.0	0.6	0.9	0.2	0.4	0.5	0.5	0.1	0.2
ZDHHC11	0.9	0.3	0.6	0.2	0.3	0.4	0.9	0.0	0.7
LOXL3	1.0	0.3	0.5	0.1	0.3	0.2	1.0	0.2	0.8
LOC101927143	1.3	0.4	0.6	0.0	0.2	0.2	1.1	0.2	0.5
LOC100506870	1.2	0.8	0.8	0.1	0.1	0.2	0.5	0.1	0.6
IFT88	1.5	0.6	0.7	0.1	0.2	0.3	0.5	0.1	0.4
CPEB3	1.1	0.4	0.7	0.2	0.2	0.3	0.7	0.2	0.6
C7orf25	1.2	0.4	0.7	0.1	0.3	0.3	0.8	0.1	0.7
MMP3	0.4	0.1	0.2	0.1	0.2	0.1	1.7	0.1	1.4
LOC101928663	1.6	0.2	0.5	0.0	0.1	0.2	1.4	0.1	0.3
ZNF180	1.5	0.3	0.6	0.0	0.1	0.2	0.9	0.1	0.6
HOXC9	1.3	0.3	0.6	0.2	0.3	0.4	0.7	0.1	0.5
GPR133	1.3	0.4	0.8	0.2	0.3	0.4	0.6	0.1	0.3
LINC00276	1.6	0.3	0.6	0.1	0.2	0.3	0.8	0.1	0.5
LOC283914	1.4	0.3	0.7	0.1	0.2	0.2	0.9	0.1	0.6
SSPN	1.6	0.7	0.8	0.1	0.2	0.3	0.3	0.1	0.5
UFSP1	0.7	0.7	0.9	0.1	0.1	0.4	0.5	0.3	0.8
ANKRD26	1.4	0.4	0.5	0.1	0.2	0.2	0.8	0.1	0.7
SEC31B	1.5	0.4	0.8	0.1	0.2	0.3	0.6	0.1	0.4
CDC37L1	1.2	0.4	0.7	0.1	0.2	0.2	0.8	0.1	0.8
RASSF2	1.2	0.3	0.6	0.1	0.4	0.2	0.8	0.1	0.5
LMO2	0.8	0.6	0.6	0.1	0.3	0.2	1.1	0.3	0.5
LOC100289533	1.2	0.5	0.6	0.2	0.4	0.5	0.5	0.1	0.4
ATP6V1B1	1.7	0.5	0.9	0.0	0.1	0.2	0.5	0.1	0.3
RGS16	0.7	0.4	1.1	0.0	0.1	0.3	1.1	0.3	0.4
LOC101928240	1.3	0.4	0.8	0.0	0.1	0.2	0.9	0.0	0.7
HOXB8	0.5	0.2	0.3	0.1	0.1	0.3	1.3	0.3	1.1

ZNF69	1.4	0.4	0.5	0.1	0.1	0.3	0.8	0.1	0.7
SPIN2B	0.8	0.3	0.5	0.1	0.2	0.2	1.1	0.2	1.2
ZNF347	0.9	0.4	0.8	0.1	0.3	0.3	0.8	0.1	0.7
SPANXA2-OT1	1.4	0.3	0.6	0.1	0.1	0.3	1.1	0.0	0.5
LOC100996473	0.8	0.5	1.0	0.2	0.4	0.3	0.7	0.0	0.3
MANSC1	1.2	0.5	0.7	0.0	0.1	0.2	0.6	0.1	0.8
IL1A	1.9	0.6	0.7	0.1	0.0	0.3	0.3	0.1	0.4
ZEB1-AS1	1.1	0.5	0.6	0.1	0.3	0.4	0.8	0.1	0.6
LRRC8B	1.2	0.4	0.4	0.1	0.1	0.3	0.9	0.2	0.8
TUBA8	1.1	0.4	0.6	0.1	0.1	0.2	0.7	0.1	1.0
HAUS3	1.4	0.3	0.4	0.0	0.1	0.2	1.0	0.1	0.9
LOC101927111	1.1	0.4	0.5	0.0	0.2	0.2	0.9	0.2	0.7
ZNF486	1.0	0.5	0.5	0.0	0.2	0.2	0.8	0.1	1.0
LOC100128594	1.3	0.3	0.6	0.1	0.2	0.4	0.9	0.1	0.4
RAPGEFL1	1.1	0.6	0.7	0.1	0.3	0.3	0.7	0.1	0.4
LOC101928993	1.7	0.4	0.6	0.1	0.3	0.3	0.7	0.0	0.3
DAPK1	1.0	0.3	0.7	0.1	0.1	0.2	1.0	0.2	0.7
AGPAT4-IT1	1.1	0.3	0.6	0.0	0.1	0.2	1.3	0.1	0.6
ANO2	1.0	0.4	0.6	0.0	0.1	0.1	1.0	0.2	0.9
LOC339622	1.4	0.3	0.5	0.1	0.2	0.2	1.0	0.1	0.5
LOC101929096	1.2	0.6	0.8	0.1	0.3	0.3	0.4	0.1	0.5
ZIC5	0.2	0.7	0.7	1.0	0.3	0.1	0.4	0.5	0.4
LOC390937	1.3	0.4	0.7	0.0	0.3	0.3	0.5	0.1	0.6
PLEKHA6	1.2	0.4	0.5	0.2	0.3	0.4	0.6	0.2	0.5
SYT1	0.7	0.2	0.4	0.1	0.2	0.1	1.6	0.1	1.0
MAP4K1	1.4	0.4	0.6	0.1	0.1	0.2	1.0	0.1	0.4
SLC2A14	1.4	0.3	0.6	0.0	0.1	0.2	1.1	0.1	0.5
SLC15A3	1.3	0.4	0.7	0.2	0.2	0.5	0.5	0.1	0.5
COL4A5	1.6	0.5	0.7	0.1	0.2	0.2	0.6	0.0	0.3
SNAI3-AS1	1.0	0.5	0.5	0.1	0.1	0.3	1.0	0.1	0.6
ZNF566	0.9	0.3	0.5	0.1	0.2	0.2	1.0	0.0	1.0
GPCPD1	1.3	0.5	0.7	0.1	0.2	0.2	0.8	0.0	0.6
FLJ40448	1.4	0.2	0.6	0.1	0.2	0.2	1.0	0.1	0.5
DACH2	1.5	0.2	0.6	0.1	0.2	0.2	1.0	0.1	0.4
CDCA3	0.5	0.2	0.6	0.2	0.4	0.5	1.0	0.2	0.7
ZNF678	1.7	0.3	0.6	0.1	0.0	0.3	0.7	0.2	0.5
USHBP1	1.6	0.2	0.6	0.1	0.1	0.2	1.0	0.1	0.4
NPIPB6	1.3	0.4	0.5	0.2	0.3	0.4	0.6	0.1	0.6
LOC101929111	1.2	0.3	0.6	0.1	0.2	0.2	1.1	0.1	0.5
ARHGAP33	0.9	0.5	0.6	0.3	0.4	0.5	0.5	0.1	0.4
LOC340017	1.7	0.2	0.6	0.0	0.2	0.2	0.8	0.1	0.4
HSD17B8	1.0	0.7	0.7	0.2	0.3	0.5	0.4	0.1	0.5
POU6F2-AS2	1.5	0.3	0.6	0.1	0.2	0.4	0.8	0.1	0.4
ZNF888	1.2	0.5	0.6	0.1	0.2	0.2	0.8	0.1	0.6
CTHRC1	1.6	0.3	1.0	0.0	0.1	0.1	0.6	0.1	0.3
GREB1L	1.3	0.4	0.6	0.0	0.2	0.2	0.9	0.1	0.6
LINC00266-1	1.2	0.4	0.7	0.1	0.2	0.3	0.6	0.1	0.7
PEX11A	1.1	0.4	0.9	0.0	0.3	0.3	0.8	0.1	0.4
KIF20B	1.0	0.3	0.5	0.0	0.3	0.3	1.0	0.1	0.7
ZNF680	1.3	0.3	0.8	0.0	0.2	0.2	0.9	0.1	0.5
BBS9	1.2	0.5	0.7	0.0	0.2	0.3	0.8	0.1	0.5
KIAA1211	1.1	0.4	0.5	0.1	0.2	0.2	0.9	0.1	0.8
LRRC23	1.2	0.5	0.8	0.1	0.4	0.2	0.4	0.2	0.3
GAS6-AS1	1.4	0.3	0.4	0.2	0.2	0.3	0.8	0.0	0.7
ZNF720	1.2	0.3	0.5	0.1	0.2	0.4	0.9	0.2	0.5

LOC101927599	1.0	0.6	0.7	0.2	0.2	0.4	0.7	0.1	0.4
FANCD2	1.2	0.3	0.6	0.1	0.2	0.3	0.9	0.1	0.6
NDC80	1.2	0.4	0.5	0.0	0.3	0.3	0.8	0.1	0.6
SCLT1	1.2	0.4	0.5	0.0	0.2	0.3	0.7	0.1	0.8
MNX1-AS1	1.4	0.5	0.6	0.2	0.3	0.6	0.3	0.0	0.4
UBL7-AS1	1.3	0.4	0.6	0.2	0.3	0.1	0.8	0.1	0.5
PRIM1	1.1	0.2	0.5	0.0	0.2	0.4	1.0	0.1	0.9
MSR1	1.3	0.4	0.7	0.1	0.2	0.1	0.8	0.1	0.4
SCLY	1.3	0.4	0.5	0.2	0.3	0.2	0.6	0.1	0.6
SLC4A3	1.1	0.5	0.6	0.1	0.2	0.2	0.7	0.2	0.6
FBXO36	1.2	0.6	0.8	0.1	0.2	0.3	0.7	0.1	0.3
RTN4IP1	1.3	0.5	0.7	0.0	0.3	0.1	0.8	0.0	0.5
LOC101927647	1.4	0.3	0.5	0.1	0.2	0.2	1.0	0.1	0.5
STRBP	1.0	0.5	0.6	0.1	0.2	0.3	0.8	0.1	0.7
GK	1.1	0.3	0.6	0.1	0.2	0.2	1.0	0.1	0.7
OPRL1	1.5	0.7	0.6	0.1	0.3	0.2	0.3	0.0	0.5
PIK3IP1	2.0	0.7	0.6	0.1	0.2	0.2	0.1	0.0	0.4
FLJ38668	1.0	0.2	0.7	0.1	0.2	0.2	1.3	0.0	0.6
LOC101929304	1.4	0.4	0.6	0.1	0.2	0.5	0.4	0.1	0.6
CDC25A	1.0	0.2	0.5	0.1	0.1	0.2	1.0	0.2	0.8
ESCO2	1.3	0.3	0.7	0.0	0.2	0.3	0.8	0.1	0.7
SNAR-A2	1.7	0.6	0.8	0.1	0.1	0.1	0.6	0.0	0.1
SNAR-A1	1.7	0.6	0.8	0.1	0.1	0.1	0.6	0.0	0.1
LOC100506358	1.2	0.4	0.6	0.1	0.4	0.3	0.8	0.1	0.4
C16orf93	0.8	0.5	0.7	0.2	0.3	0.2	0.5	0.0	1.0
LINC00880	1.3	0.3	0.6	0.1	0.2	0.4	0.9	0.1	0.4
FBLN5	1.3	0.6	0.6	0.1	0.1	0.4	0.5	0.2	0.5
SGK2	1.5	0.4	0.9	0.1	0.2	0.2	0.7	0.0	0.2
PCA3	1.5	0.3	0.6	0.1	0.3	0.3	0.6	0.1	0.4
MTERF	1.1	0.3	0.7	0.0	0.2	0.1	1.0	0.0	0.7
LOC100996669	1.5	0.3	0.5	0.1	0.2	0.3	0.8	0.1	0.5
DNAH14	0.8	0.2	0.4	0.0	0.1	0.1	0.6	0.1	1.9
STARD9	1.2	0.4	0.6	0.1	0.2	0.3	0.7	0.1	0.6
LOC729162	1.1	0.3	0.5	0.2	0.2	0.4	0.9	0.1	0.5
ZNF814	1.2	0.3	0.7	0.0	0.2	0.2	0.8	0.1	0.7
NEDD8-MDP1	0.4	0.5	0.3	0.2	0.2	0.1	1.5	0.1	0.8
LOC101927027	1.3	0.6	0.4	0.1	0.1	0.4	0.9	0.1	0.4
BMPR1B	1.1	0.3	0.6	0.0	0.2	0.1	1.0	0.1	0.7
SNX15	0.6	0.5	0.3	0.1	0.3	0.2	1.1	0.3	0.7
ZNF549	1.3	0.5	0.5	0.1	0.2	0.2	0.6	0.1	0.7
PITPNM3	1.1	0.5	0.6	0.2	0.3	0.4	0.6	0.1	0.5
FAM201A	1.0	0.7	0.7	0.2	0.3	0.3	0.6	0.0	0.4
HIVEP3	1.5	0.4	0.4	0.1	0.2	0.4	0.6	0.1	0.5
SH3BP5-AS1	1.4	0.5	0.7	0.1	0.2	0.2	0.6	0.1	0.5
TMEM106A	0.7	0.2	0.5	0.1	0.3	0.2	1.4	0.1	0.7
ROGDI	1.4	0.6	0.7	0.2	0.3	0.3	0.3	0.1	0.2
SENP7	1.1	0.5	0.7	0.0	0.2	0.3	0.7	0.1	0.5
XKR9	1.2	0.5	0.6	0.0	0.1	0.1	0.9	0.1	0.6
C9orf84	1.3	0.5	0.9	0.0	0.1	0.2	0.7	0.1	0.4
FAM138B	1.2	0.2	0.6	0.1	0.0	0.3	1.3	0.0	0.5
FASTKD1	1.2	0.5	0.7	0.0	0.1	0.2	0.8	0.1	0.6
CCDC146	1.3	0.3	0.7	0.1	0.1	0.2	0.9	0.1	0.6
ADRB1	0.8	0.4	0.3	0.4	0.3	0.3	0.7	0.3	0.7
LOC101929568	1.4	0.4	0.6	0.1	0.5	0.1	0.4	0.2	0.5
LOC101927446	1.6	0.2	0.6	0.1	0.3	0.2	0.7	0.1	0.4

ANKRD18A	1.2	0.3	0.6	0.1	0.2	0.2	0.9	0.1	0.6
EFHC1	1.3	0.3	0.6	0.1	0.1	0.3	0.8	0.1	0.5
ZNF562	1.0	0.4	0.8	0.0	0.2	0.2	0.8	0.1	0.7
RAB40A	1.4	0.1	0.4	0.1	0.3	0.3	0.9	0.1	0.5
SPIN2A	1.1	0.4	0.6	0.2	0.1	0.3	0.9	0.1	0.4
KIF25-AS1	1.4	0.3	0.6	0.1	0.1	0.2	1.0	0.1	0.4
LOC100127967	1.2	0.2	0.6	0.1	0.1	0.2	1.1	0.1	0.5
CCDC149	1.4	0.5	0.6	0.1	0.2	0.2	0.6	0.1	0.5
PABPC4L	0.9	0.4	0.6	0.1	0.2	0.3	1.0	0.1	0.5
LSP1	1.3	0.4	0.5	0.1	0.3	0.2	0.7	0.1	0.4
RNF32	1.1	0.3	0.5	0.1	0.3	0.3	0.8	0.2	0.6
AGAP8	1.3	0.3	0.5	0.1	0.3	0.3	0.7	0.1	0.6
VAMP4	1.2	0.5	0.5	0.0	0.2	0.2	0.8	0.1	0.7
HYMAI	1.3	0.3	0.6	0.1	0.1	0.2	1.0	0.1	0.4
FOXD4	0.9	0.3	0.7	0.2	0.1	0.5	0.7	0.3	0.6
LINC00685	1.3	0.4	0.5	0.2	0.2	0.3	1.0	0.0	0.4
LINC00685	1.3	0.4	0.5	0.2	0.2	0.3	1.0	0.0	0.4
LOC389602	1.4	0.6	0.5	0.1	0.3	0.5	0.5	0.1	0.2
NANOGNB	1.6	0.2	0.6	0.2	0.2	0.3	0.7	0.1	0.3
RCSD1	1.1	0.8	0.6	0.1	0.4	0.3	0.5	0.1	0.3
ALX3	1.3	0.4	0.7	0.0	0.3	0.2	0.7	0.1	0.4
LOC101928379	1.0	0.5	1.0	0.0	0.0	0.3	0.4	0.3	0.5
LRRC49	1.3	0.6	0.6	0.0	0.1	0.1	0.6	0.1	0.6
LY75	1.4	0.5	0.6	0.0	0.2	0.3	0.5	0.0	0.5
HSF2BP	0.9	0.4	0.6	0.2	0.4	0.5	0.7	0.0	0.3
SLC22A13	1.5	0.3	0.5	0.1	0.1	0.2	0.9	0.1	0.4
SARDH	1.2	0.4	0.6	0.2	0.4	0.5	0.4	0.0	0.4
IDH2	1.2	0.5	0.4	0.2	0.3	0.4	0.4	0.1	0.6
LOC286184	1.5	0.3	0.5	0.1	0.2	0.2	1.0	0.0	0.3
FAM222A	1.1	0.5	0.4	0.2	0.1	0.2	0.8	0.2	0.7
LOC440896	1.3	0.3	0.6	0.1	0.1	0.2	1.0	0.1	0.5
TTK	1.0	0.4	0.4	0.0	0.3	0.3	0.9	0.1	0.7
PSORS1C1	1.0	0.2	0.3	0.1	0.3	0.1	1.0	0.2	0.8
LOC100653515	1.1	0.2	0.5	0.2	0.3	0.3	0.8	0.1	0.7
ADAMTS17	1.0	0.4	0.6	0.1	0.3	0.4	0.8	0.1	0.5
UGGT2	1.1	0.4	0.6	0.1	0.2	0.2	0.9	0.1	0.5
SUSD3	1.4	0.5	0.4	0.1	0.3	0.5	0.5	0.1	0.3
GPR75	1.6	0.3	0.5	0.1	0.2	0.1	0.5	0.1	0.7
PLXNB3	1.0	0.4	0.6	0.2	0.6	0.5	0.5	0.1	0.3
LOC101927614	1.4	0.5	0.6	0.1	0.2	0.1	0.8	0.1	0.3
FAM46B	0.9	0.4	0.5	0.1	0.1	0.4	0.9	0.2	0.6
PIP5KL1	1.2	0.3	0.4	0.1	0.2	0.1	0.9	0.2	0.6
LOC101927994	1.5	0.3	0.6	0.1	0.2	0.2	0.7	0.0	0.5
LOC100506901	1.0	0.4	0.7	0.1	0.3	0.2	1.0	0.1	0.4
LOC389607	1.5	0.3	0.6	0.2	0.2	0.3	0.5	0.1	0.4
FLJ46134	1.3	0.3	0.6	0.1	0.2	0.3	0.9	0.1	0.4
ITPK1-AS1	1.2	0.2	0.7	0.1	0.2	0.2	1.1	0.1	0.5
ZNF860	1.0	0.3	0.5	0.0	0.1	0.3	0.9	0.1	0.9
ST7-AS1	1.2	0.3	0.6	0.1	0.3	0.4	0.5	0.1	0.5
ZNF845	1.2	0.3	0.6	0.0	0.1	0.2	0.8	0.1	0.7
LOC101927989	0.8	0.6	0.6	0.1	0.2	0.3	1.0	0.1	0.6
CRYL1	1.1	0.4	0.5	0.1	0.4	0.6	0.5	0.0	0.4
LOC100506917	1.5	0.3	0.6	0.1	0.1	0.2	0.8	0.1	0.3
ZNF671	1.1	0.5	0.7	0.1	0.2	0.2	0.8	0.0	0.6
C7orf31	1.2	0.5	0.6	0.1	0.1	0.2	0.6	0.1	0.7

PKIB	2.0	0.3	0.9	0.1	0.3	0.1	0.2	0.2	0.0
GSDMC	1.3	0.5	0.7	0.1	0.1	0.1	0.7	0.1	0.5
LOC728431	1.5	0.3	0.6	0.1	0.4	0.3	0.4	0.1	0.4
GNGT2	0.5	0.4	0.0	0.1	0.0	0.4	1.2	0.2	1.3
CENPP	0.7	0.4	0.5	0.0	0.4	0.3	0.7	0.2	0.8
LOC101929089	1.4	0.5	0.6	0.1	0.2	0.5	0.5	0.0	0.2
TMEM81	1.5	0.3	0.5	0.0	0.0	0.1	0.9	0.1	0.6
LOC401324	1.4	0.3	0.6	0.1	0.2	0.3	0.7	0.0	0.5
KIAA0408	1.2	0.3	0.6	0.1	0.1	0.2	0.8	0.1	0.6
FAM201B	1.2	0.3	0.8	0.0	0.2	0.3	0.6	0.1	0.5
PER3	1.4	0.5	0.6	0.1	0.2	0.3	0.5	0.1	0.4
VAX2	1.4	0.6	0.8	0.1	0.2	0.2	0.3	0.1	0.3
PIGP	1.0	0.6	0.5	0.1	0.4	0.5	0.5	0.1	0.3
LOC101927883	1.5	0.4	0.5	0.1	0.2	0.4	0.6	0.1	0.3
LOC101928465	1.2	0.4	0.5	0.1	0.3	0.3	0.7	0.1	0.5
LACE1	1.4	0.3	0.7	0.1	0.2	0.3	0.7	0.1	0.3
ROR1	1.2	0.4	0.5	0.1	0.4	0.5	0.5	0.1	0.3
AGO4	1.2	0.4	0.6	0.1	0.2	0.2	0.7	0.1	0.5
SEMA6C	1.4	0.4	0.7	0.2	0.2	0.3	0.3	0.0	0.4
CPNE7	0.9	0.4	0.5	0.3	0.5	0.7	0.3	0.1	0.3
C16orf87	1.1	0.7	0.7	0.1	0.1	0.4	0.5	0.1	0.4
DLX1	1.3	0.4	0.6	0.1	0.5	0.3	0.5	0.1	0.3
LOC100505702	1.1	0.3	0.5	0.0	0.2	0.2	1.0	0.1	0.5
NPHP3-ACAD11	1.1	0.4	0.7	0.1	0.3	0.3	0.7	0.1	0.4
COL9A3	0.9	0.4	0.6	0.2	0.3	0.3	0.6	0.1	0.6
FAM132B	1.1	0.2	0.5	0.2	0.2	0.3	1.0	0.2	0.4
TRNAK26	0.8	0.5	0.6	0.1	0.1	0.1	1.2	0.1	0.6
TRNAK14	0.8	0.5	0.6	0.1	0.1	0.1	1.2	0.1	0.6
TRNAK2	0.8	0.5	0.6	0.1	0.1	0.1	1.2	0.1	0.6
TRNAK33	0.8	0.5	0.6	0.1	0.1	0.1	1.2	0.1	0.6
TRNAK20	0.8	0.5	0.6	0.1	0.1	0.1	1.2	0.1	0.6
KDM4C	1.1	0.4	0.6	0.1	0.2	0.3	0.7	0.1	0.6
DZIP1	1.4	0.4	0.6	0.0	0.2	0.3	0.5	0.1	0.6
MFAP3L	1.0	0.3	0.4	0.1	0.1	0.3	0.7	0.1	0.8
MAFA	1.7	0.4	0.8	0.1	0.0	0.1	0.3	0.3	0.3
ZFHX2	1.3	0.4	0.6	0.1	0.2	0.3	0.6	0.1	0.4
C9orf169	1.2	0.6	0.9	0.1	0.3	0.4	0.3	0.1	0.2
LOC100507306	1.4	0.3	0.7	0.1	0.2	0.4	0.4	0.1	0.5
LOC101926922	1.5	0.3	0.5	0.1	0.1	0.2	0.8	0.1	0.4
ZUFSP	1.0	0.4	0.6	0.0	0.2	0.3	0.6	0.1	0.7
JAKMIP2	1.3	0.2	0.5	0.1	0.1	0.2	1.0	0.1	0.4
ZNF221	1.1	0.4	0.6	0.0	0.1	0.3	0.6	0.1	0.7
SMPD5	1.0	0.5	0.7	0.2	0.3	0.2	0.6	0.0	0.4
ZNF200	1.2	0.3	0.5	0.1	0.2	0.3	0.6	0.1	0.8
KCND1	1.4	0.5	0.7	0.1	0.1	0.1	0.6	0.1	0.4
GDPD3	1.5	0.5	0.4	0.1	0.1	0.2	0.6	0.1	0.5
NUDT13	1.1	0.3	0.6	0.0	0.2	0.4	0.7	0.1	0.6
CTPS2	1.1	0.5	0.3	0.1	0.3	0.3	0.7	0.0	0.6
RNF39	0.7	0.5	0.7	0.1	0.3	0.3	0.8	0.0	0.6
RFX2	1.2	0.4	0.4	0.2	0.3	0.4	0.5	0.1	0.5
CORO2A	0.8	0.3	0.4	0.2	0.2	0.3	1.2	0.2	0.5
GAPDHS	1.1	0.4	0.5	0.1	0.2	0.2	0.7	0.1	0.6
FAM169A	1.0	0.4	0.5	0.0	0.1	0.1	1.0	0.1	0.7
LOC100505585	1.2	0.5	0.5	0.2	0.4	0.3	0.4	0.1	0.4
FLRT1	0.9	0.1	0.5	0.1	0.2	0.2	1.3	0.1	0.4

THAP7-AS1	1.6	0.2	0.9	0.1	0.1	0.2	0.6	0.1	0.2
EXO1	0.6	0.3	0.3	0.0	0.3	0.2	1.2	0.2	0.9
PCED1B	0.9	0.4	0.5	0.2	0.3	0.3	0.7	0.1	0.6
LAMA1	0.8	0.3	0.5	0.1	0.2	0.2	1.1	0.2	0.7
ACRC	1.0	0.6	0.8	0.1	0.1	0.3	0.7	0.1	0.5
LOC101929768	1.3	0.5	0.6	0.0	0.3	0.1	0.4	0.0	0.6
LOC101927626	1.3	0.6	0.7	0.1	0.1	0.1	0.6	0.1	0.3
LOC100652777	1.4	0.1	0.4	0.2	0.2	0.3	0.8	0.1	0.4
MAP3K8	1.2	0.7	0.8	0.1	0.0	0.2	0.4	0.1	0.4
ATP6AP1L	1.5	0.4	0.7	0.1	0.1	0.1	0.4	0.1	0.5
LAT2	1.3	0.6	0.7	0.1	0.2	0.2	0.6	0.0	0.2
TRNAG27	0.5	0.3	0.3	0.2	0.4	0.2	1.3	0.2	0.7
TRNAG15	0.5	0.3	0.3	0.2	0.4	0.2	1.3	0.2	0.7
TRNAG21	0.5	0.3	0.3	0.2	0.4	0.2	1.3	0.2	0.7
TRNAG23	0.5	0.3	0.3	0.2	0.4	0.2	1.3	0.2	0.7
TRNAG30	0.5	0.3	0.3	0.2	0.4	0.2	1.3	0.2	0.7
FAM171B	1.1	0.4	0.7	0.1	0.2	0.3	0.5	0.2	0.5
VSNL1	1.3	0.4	0.9	0.0	0.1	0.2	0.6	0.1	0.4
SMPDL3B	1.2	0.6	0.8	0.1	0.1	0.3	0.4	0.1	0.4
LOC101927448	1.3	0.3	0.6	0.1	0.1	0.1	0.9	0.1	0.5
SLC25A52	1.6	0.3	0.6	0.0	0.1	0.1	0.4	0.1	0.7
WFIKKN1	1.2	0.4	0.4	0.1	0.2	0.2	0.7	0.1	0.5
RNF112	1.0	0.6	0.6	0.1	0.1	0.2	0.6	0.1	0.6
LINC00410	1.4	0.2	0.5	0.1	0.1	0.1	0.9	0.1	0.5
SPATA7	1.4	0.5	0.8	0.0	0.1	0.2	0.4	0.1	0.4
LSR	1.0	0.5	0.6	0.2	0.2	0.2	0.7	0.1	0.4
ZNF181	0.9	0.5	0.3	0.0	0.2	0.4	0.9	0.1	0.5
PDCD1LG2	0.7	0.4	0.3	0.0	0.3	0.5	1.0	0.2	0.4
PCDH20	1.4	0.5	0.8	0.1	0.2	0.3	0.4	0.0	0.3
ZNF547	1.2	0.3	0.8	0.0	0.1	0.3	0.6	0.0	0.4
FLJ33630	1.3	0.4	0.6	0.1	0.2	0.3	0.7	0.1	0.4
PDE10A	0.9	0.3	0.5	0.1	0.2	0.3	0.7	0.1	0.7
TCN2	1.6	0.4	0.7	0.1	0.2	0.2	0.4	0.1	0.2
CPT2	1.3	0.5	0.7	0.1	0.2	0.4	0.4	0.1	0.3
LDLRAD4	1.0	0.3	0.6	0.0	0.3	0.1	0.8	0.1	0.7
CLDN20	1.0	0.2	0.8	0.1	0.2	0.3	0.8	0.1	0.4
CYP2F1	0.9	0.2	0.5	0.1	0.2	0.2	1.1	0.1	0.6
TEC	1.1	0.4	0.6	0.1	0.2	0.1	0.7	0.1	0.6
UBAP1L	1.2	0.4	0.7	0.2	0.4	0.1	0.5	0.1	0.3
ZNF189	1.0	0.4	0.6	0.0	0.3	0.2	0.8	0.1	0.5
LOC101926978	0.6	0.2	0.5	0.0	0.2	0.4	1.2	0.2	0.6
JAG2	1.5	0.6	0.9	0.1	0.1	0.2	0.4	0.0	0.2
AZGP1	1.2	0.4	0.5	0.1	0.2	0.3	0.6	0.1	0.4
LINC01018	1.2	0.3	0.4	0.1	0.2	0.2	1.0	0.1	0.3
LRRC37B	1.3	0.5	0.9	0.1	0.1	0.1	0.4	0.1	0.4
NGF	0.8	0.4	0.4	0.1	0.3	0.3	0.7	0.1	0.6
LOC101929252	0.7	0.3	0.3	0.4	0.7	0.7	0.4	0.1	0.2
LINC01004	1.4	0.2	0.6	0.1	0.1	0.2	0.7	0.1	0.4
LOC101929420	1.4	0.3	0.6	0.1	0.2	0.2	0.7	0.1	0.4
CDHR5	1.3	0.3	0.8	0.1	0.3	0.1	0.4	0.1	0.3
CD14	1.1	0.4	0.9	0.1	0.2	0.2	0.6	0.1	0.3
FGD6	1.2	0.4	0.6	0.1	0.1	0.2	0.6	0.1	0.5
FZD10-AS1	1.5	0.3	0.5	0.1	0.2	0.2	0.7	0.0	0.4
SPNS3	0.9	0.5	0.6	0.3	0.2	0.3	0.5	0.0	0.5
ZNF772	1.1	0.5	0.6	0.1	0.2	0.4	0.6	0.1	0.3

BCL2A1	1.4	0.4	0.8	0.0	0.0	0.2	0.7	0.1	0.4
KDM8	1.2	0.5	0.5	0.1	0.1	0.2	0.7	0.1	0.5
LOC101929740	0.9	0.2	0.6	0.1	0.2	0.4	0.6	0.0	1.0
APBB1	1.5	0.4	0.7	0.1	0.1	0.1	0.3	0.1	0.5
GUCA1B	1.2	0.6	0.5	0.0	0.1	0.2	0.5	0.2	0.6
ZNF485	1.1	0.5	0.5	0.0	0.3	0.2	0.6	0.1	0.6
CEP76	1.0	0.3	0.6	0.1	0.2	0.3	0.6	0.1	0.7
KIAA1407	1.6	0.6	0.6	0.1	0.1	0.2	0.4	0.0	0.4
PDGFRL	0.9	0.5	0.7	0.1	0.3	0.1	0.7	0.1	0.5
NAPEPLD	1.1	0.4	0.7	0.1	0.1	0.3	0.7	0.0	0.5
ACAP1	1.1	0.5	0.6	0.1	0.2	0.2	0.6	0.1	0.4
TERT	0.9	0.3	0.5	0.2	0.3	0.4	0.7	0.2	0.5
OTX1	1.3	0.4	0.4	0.2	0.3	0.4	0.4	0.1	0.3
MYSM1	1.3	0.4	0.7	0.0	0.1	0.2	0.6	0.0	0.5
RFXAP	0.7	0.3	0.6	0.2	0.4	0.5	0.6	0.1	0.6
HOXC12	0.2	0.8	0.5	0.5	0.3	0.1	0.6	0.2	0.6
PLCD1	1.2	0.4	0.6	0.2	0.3	0.3	0.4	0.1	0.4
TTC22	1.2	0.4	0.5	0.1	0.1	0.2	0.9	0.1	0.4
UPK3B	1.4	0.4	0.5	0.2	0.3	0.2	0.7	0.0	0.3
DNA2	1.2	0.4	0.4	0.0	0.1	0.1	0.8	0.1	0.5
ZNF616	1.0	0.4	0.4	0.1	0.2	0.1	0.8	0.1	0.7
GABRE	1.2	0.6	0.6	0.1	0.3	0.3	0.4	0.1	0.3
RBM45	0.9	0.3	0.5	0.0	0.1	0.3	0.8	0.1	0.7
AMN1	1.2	0.5	0.6	0.0	0.2	0.4	0.6	0.0	0.3
NRK	0.8	0.3	0.4	0.0	0.2	0.2	1.0	0.2	0.8
NEK2	1.0	0.4	0.8	0.0	0.2	0.4	0.6	0.1	0.3
SGOL1-AS1	1.0	0.2	0.6	0.1	0.4	0.2	0.9	0.2	0.4
ZNF383	1.3	0.3	0.5	0.1	0.1	0.3	0.7	0.0	0.5
PRMT10	1.0	0.2	0.5	0.1	0.2	0.2	0.8	0.1	0.6
TICRR	0.9	0.2	0.4	0.1	0.2	0.3	0.9	0.2	0.6
S1PR3	1.1	0.4	0.5	0.1	0.2	0.3	0.6	0.1	0.4
LIMS3-LOC440895	1.1	0.4	0.5	0.0	0.0	0.2	0.9	0.0	0.7
SPDYE2B	1.2	0.3	0.6	0.1	0.2	0.2	0.7	0.1	0.3
KLHL7-AS1	1.3	0.3	0.6	0.0	0.2	0.2	0.8	0.1	0.3
LOC642648	1.1	0.5	0.8	0.1	0.1	0.2	0.5	0.1	0.4
LOC101928639	1.2	0.2	0.6	0.1	0.2	0.1	0.8	0.1	0.4
SCUBE3	0.9	0.4	0.6	0.1	0.3	0.2	0.8	0.1	0.5
HSPA4L	0.9	0.3	0.6	0.0	0.1	0.2	1.0	0.2	0.5
LOC729224	1.2	0.3	0.4	0.1	0.2	0.2	0.9	0.0	0.5
PITRM1-AS1	1.3	0.3	0.6	0.0	0.1	0.1	0.8	0.1	0.5
SMG7-AS1	1.3	0.5	0.5	0.1	0.2	0.2	0.5	0.0	0.4
TRIM59	0.9	0.2	0.6	0.0	0.2	0.2	0.9	0.1	0.6
KIAA1984	1.2	0.4	0.7	0.1	0.2	0.2	0.5	0.1	0.3
SLC18B1	0.9	0.5	0.5	0.0	0.1	0.2	1.1	0.1	0.4
ABCA7	1.2	0.4	0.8	0.1	0.2	0.2	0.4	0.1	0.3
GLIPR2	0.7	0.2	0.5	0.1	0.5	0.2	1.0	0.1	0.4
SYBU	1.1	0.3	0.3	0.0	0.2	0.2	0.9	0.1	0.6
LOC101929475	1.0	0.4	0.4	0.1	0.2	0.3	0.8	0.1	0.4
LOC101928588	1.2	0.4	0.7	0.1	0.2	0.1	0.7	0.1	0.4
ANXA2R	1.0	0.4	0.5	0.1	0.4	0.1	0.7	0.1	0.4
MEIS3	0.9	0.6	0.6	0.1	0.3	0.1	0.6	0.1	0.4
CLDN1	0.8	0.5	0.5	0.0	0.1	0.1	1.0	0.1	0.6
GABPB2	1.3	0.2	0.8	0.1	0.2	0.2	0.5	0.2	0.4
ZSCAN5A	0.7	0.3	0.4	0.1	0.1	0.2	1.2	0.2	0.7
LINC00115	1.0	0.4	0.8	0.2	0.2	0.3	0.4	0.2	0.4

N4BP2L2-IT2		1.6	0.2	0.6	0.0	0.1	0.1	0.7	0.0	0.3
SIRT4		1.1	0.5	0.4	0.0	0.3	0.0	0.6	0.2	0.6
ZXDA		1.1	0.5	0.4	0.2	0.1	0.3	0.6	0.1	0.4
C1orf112		1.1	0.4	0.5	0.0	0.2	0.2	0.6	0.1	0.7
DHX58		1.2	0.6	0.8	0.1	0.2	0.3	0.3	0.1	0.2
LOC101928241		1.5	0.2	0.5	0.0	0.1	0.3	0.6	0.0	0.4
CLDN14		0.8	0.0	0.4	0.2	0.4	0.6	0.4	0.3	0.5
TRIM36		1.1	0.2	0.5	0.0	0.1	0.2	0.8	0.1	0.7
LOC101928371		1.4	0.3	0.5	0.1	0.1	0.1	0.8	0.1	0.5
DNAH1		1.1	0.3	0.5	0.2	0.3	0.3	0.5	0.1	0.4
NES		0.8	0.3	0.5	0.1	0.2	0.3	0.8	0.1	0.6
TNK1		0.9	0.4	0.5	0.1	0.1	0.3	0.7	0.1	0.6
PCDHGB2		1.2	0.3	0.7	0.1	0.2	0.1	0.4	0.3	0.3
C19orf40		0.7	0.6	0.8	0.1	0.2	0.1	0.8	0.0	0.4
SH3PXD2A-AS1		1.2	0.2	0.4	0.0	0.1	0.3	0.9	0.1	0.5
VWA1		1.1	0.5	0.7	0.2	0.2	0.3	0.7	0.0	0.1
ST3GAL5		1.3	0.5	0.5	0.1	0.2	0.4	0.4	0.1	0.3
LOC101929500		1.1	0.2	0.5	0.1	0.1	0.1	1.0	0.1	0.5
RIMBP3		0.9	0.3	0.4	0.1	0.3	0.2	0.7	0.1	0.6
PBK		0.5	0.4	0.4	0.1	0.3	0.2	0.9	0.1	0.9
C21orf119		0.9	0.4	0.8	0.1	0.3	0.1	0.5	0.1	0.5
	44621.0	0.9	0.5	0.6	0.0	0.1	0.3	0.5	0.2	0.4
EFEMP2		1.1	0.3	0.6	0.1	0.3	0.3	0.5	0.1	0.3
NTRK2		1.0	0.3	0.5	0.1	0.1	0.2	0.8	0.1	0.5
MIRLET7DHG		1.1	0.3	0.5	0.0	0.1	0.1	0.9	0.1	0.5
PLCD4		1.1	0.4	0.5	0.1	0.2	0.1	0.6	0.1	0.6
DOCK2		1.0	0.4	0.4	0.1	0.2	0.2	0.7	0.1	0.6
ACTRT3		0.8	0.3	0.4	0.0	0.1	0.2	0.9	0.1	0.8
ZNF431		1.0	0.3	0.6	0.0	0.1	0.2	0.7	0.2	0.7
WDPCP		1.3	0.3	0.5	0.1	0.2	0.1	0.6	0.1	0.4
LOC101929752		1.3	0.2	0.6	0.1	0.1	0.0	0.9	0.1	0.4
USP27X		0.9	0.4	0.6	0.0	0.2	0.4	0.8	0.1	0.4
TRAIP		0.7	0.3	0.5	0.1	0.3	0.3	1.0	0.1	0.4
LOC101927411		1.1	0.3	0.4	0.0	0.2	0.2	0.9	0.1	0.5
SETD9		0.6	0.2	0.5	0.0	0.2	0.3	0.9	0.1	0.8
CTSK		1.6	0.3	0.5	0.1	0.1	0.1	0.6	0.1	0.3
ZNF77		0.8	0.4	0.7	0.1	0.1	0.2	0.5	0.1	0.7
ATG4C		1.0	0.5	0.5	0.1	0.1	0.3	0.6	0.0	0.5
RAET1G		0.8	0.4	0.5	0.1	0.3	0.5	0.4	0.2	0.5
CYP3A43		1.1	0.3	0.6	0.1	0.1	0.1	0.9	0.1	0.3
WSCD1		1.3	0.4	0.6	0.1	0.1	0.2	0.5	0.1	0.5
CCDC169		0.9	0.4	0.5	0.0	0.2	0.2	0.7	0.1	0.6
MORN1		1.0	0.1	0.6	0.2	0.5	0.4	0.4	0.1	0.3
NT5M		1.3	0.5	0.7	0.1	0.5	0.3	0.2	0.1	0.1
A1BG		1.1	0.5	0.6	0.1	0.1	0.2	0.6	0.1	0.3
SFRP5		0.9	0.2	0.4	0.1	0.3	0.3	0.8	0.2	0.4
PDE7A		0.9	0.4	0.7	0.0	0.2	0.2	0.7	0.1	0.5
LOC101928574		0.9	0.3	0.6	0.0	0.2	0.4	0.6	0.1	0.6
FOXA3		0.9	0.4	0.6	0.2	0.1	0.3	0.4	0.2	0.5
SLC25A35		0.8	0.3	0.4	0.1	0.2	0.4	0.8	0.1	0.5
XRCC2		0.6	0.2	0.2	0.0	0.3	0.3	1.2	0.2	0.8
ERCC6L		0.8	0.2	0.4	0.0	0.2	0.2	0.9	0.1	0.8
FAXC		0.9	0.4	0.4	0.1	0.1	0.2	0.9	0.1	0.5
FOXP2		0.9	0.3	0.6	0.1	0.1	0.3	0.7	0.1	0.6
GHRLOS		1.4	0.3	0.6	0.0	0.1	0.1	0.5	0.0	0.5

LOC101929460	1.0	0.3	0.6	0.0	0.1	0.3	0.7	0.1	0.4
FAM71F2	1.1	0.3	0.7	0.1	0.4	0.3	0.3	0.0	0.3
PEX11G	1.2	0.3	0.8	0.0	0.5	0.3	0.3	0.0	0.2
GPX2	1.1	0.3	0.5	0.0	0.4	0.2	0.7	0.0	0.4
MND1	0.8	0.3	0.4	0.0	0.1	0.2	0.9	0.2	0.8
PRKG1	0.7	0.2	0.3	0.1	0.3	0.3	0.8	0.1	0.8
APOBEC3B	1.0	0.5	0.3	0.1	0.5	0.3	0.4	0.1	0.5
APCDD1L	0.8	0.5	0.4	0.3	0.3	0.5	0.4	0.1	0.2
ZNF415	1.0	0.3	0.4	0.1	0.2	0.3	0.7	0.1	0.6
HSPA12B	0.9	0.4	0.4	0.1	0.2	0.1	0.8	0.1	0.5
LOC101928975	1.5	0.4	0.7	0.1	0.2	0.2	0.3	0.1	0.1
DDN	0.8	0.3	0.3	0.1	0.1	0.1	0.9	0.2	0.8
SIGLEC11	1.3	0.2	0.4	0.1	0.1	0.2	0.9	0.1	0.3
ZC3H12B	1.1	0.2	0.5	0.1	0.2	0.3	0.8	0.0	0.5
LOC101927841	0.9	0.4	0.6	0.0	0.1	0.2	0.6	0.1	0.5
PRR19	0.7	0.5	0.3	0.1	0.1	0.2	0.7	0.2	0.8
IQCC	1.0	0.5	0.5	0.1	0.2	0.1	0.6	0.1	0.5
ALOX12	1.1	0.3	0.6	0.1	0.2	0.2	0.7	0.1	0.4
GINS4	0.6	0.3	0.2	0.1	0.2	0.5	1.3	0.2	0.4
ATAD5	0.8	0.2	0.4	0.0	0.2	0.2	1.0	0.1	0.7
PIGB	1.1	0.4	0.8	0.0	0.2	0.3	0.4	0.1	0.4
LOC101929290	1.2	0.2	0.4	0.1	0.1	0.2	1.0	0.1	0.4
LOC101928862	1.2	0.2	0.5	0.1	0.2	0.2	0.7	0.1	0.5
ZNF239	0.6	0.2	0.4	0.1	0.4	0.3	0.7	0.1	0.7
C22orf29	0.8	0.3	0.5	0.1	0.3	0.3	0.8	0.1	0.4
MSH5-SAPCD1	1.0	0.4	0.5	0.2	0.3	0.3	0.5	0.0	0.4
LOC101929729	1.1	0.2	0.4	0.0	0.2	0.0	0.6	0.1	0.9
LOC285692	1.3	0.2	0.6	0.0	0.1	0.2	0.8	0.1	0.4
ZNF555	0.8	0.5	0.4	0.0	0.1	0.3	0.7	0.2	0.5
C1S	1.4	0.4	0.9	0.1	0.2	0.2	0.3	0.0	0.2
DAW1	1.2	0.2	0.5	0.1	0.1	0.2	0.9	0.1	0.3
MBNL1-AS1	0.9	0.3	0.5	0.1	0.1	0.2	0.7	0.1	0.5
TNIK	1.3	0.4	0.6	0.2	0.2	0.3	0.4	0.1	0.2
LOC101928583	1.3	0.3	0.6	0.1	0.1	0.1	0.5	0.0	0.4
HLA-F	0.9	0.4	0.8	0.1	0.3	0.4	0.3	0.1	0.3
ALDOC	1.3	0.3	0.5	0.1	0.3	0.2	0.6	0.1	0.3
ATP2A1	1.3	0.4	0.5	0.1	0.1	0.1	0.6	0.1	0.5
RGPD5	0.9	0.5	0.5	0.0	0.1	0.2	0.8	0.1	0.5
TRDMT1	0.7	0.3	0.6	0.0	0.3	0.2	0.6	0.1	0.7
ABHD3	0.8	0.4	0.5	0.1	0.2	0.2	0.8	0.1	0.5
LOC101927032	1.2	0.3	0.5	0.1	0.1	0.1	0.9	0.1	0.4
CAND1.11	1.1	0.3	0.7	0.2	0.1	0.1	0.7	0.1	0.3
CILP2	0.9	0.2	0.4	0.1	0.1	0.1	1.1	0.2	0.6
GRIK2	1.2	0.3	0.4	0.1	0.4	0.3	0.5	0.1	0.3
LDHD	1.0	0.3	0.7	0.2	0.3	0.3	0.3	0.1	0.3
IL17D	0.6	0.4	0.4	0.3	0.1	0.3	0.5	0.1	0.7
GPR83	1.5	0.4	0.5	0.0	0.3	0.2	0.5	0.1	0.2
LOC101928221	0.9	0.1	0.6	0.1	0.2	0.3	0.6	0.0	0.8
STXBP4	1.2	0.4	0.4	0.0	0.1	0.1	0.6	0.1	0.6
TARSL2	0.9	0.3	0.4	0.1	0.2	0.3	0.8	0.1	0.5
ATF7IP2	1.2	0.3	0.5	0.1	0.1	0.1	0.8	0.1	0.4
FAM115C	1.1	0.3	0.3	0.1	0.1	0.3	0.8	0.1	0.4
LOC642441	1.1	0.0	0.6	0.2	0.1	0.3	0.7	0.0	0.4
C12orf66	1.2	0.3	0.6	0.0	0.1	0.2	0.6	0.1	0.4
RAVER2	0.9	0.3	0.6	0.1	0.1	0.2	0.6	0.1	0.7

SSTR2	0.8	0.2	0.5	0.1	0.2	0.1	1.0	0.1	0.6
APLP1	0.9	0.4	0.4	0.1	0.2	0.4	0.6	0.1	0.4
LOC100130887	1.1	0.3	0.3	0.1	0.2	0.3	0.6	0.1	0.5
CD83	1.2	0.3	0.4	0.1	0.3	0.2	0.6	0.1	0.5
MUC20	1.1	0.2	0.4	0.1	0.2	0.2	0.6	0.2	0.5
DHRS3	1.3	0.5	0.8	0.1	0.3	0.3	0.0	0.1	0.1
LOC100294145	0.8	0.4	0.4	0.1	0.2	0.3	0.7	0.1	0.6
PER2	1.0	0.4	0.4	0.1	0.1	0.3	0.7	0.2	0.6
PEX7	1.6	0.3	0.4	0.1	0.2	0.4	0.4	0.0	0.2
WDR91	1.1	0.6	0.7	0.1	0.2	0.3	0.4	0.0	0.3
PCDHGA12	1.4	0.2	0.7	0.1	0.2	0.2	0.4	0.1	0.3
KRBA1	1.0	0.2	0.5	0.2	0.2	0.3	0.7	0.1	0.5
UBE2E2-AS1	0.8	0.3	0.6	0.2	0.2	0.2	0.7	0.1	0.4
SNAR-A11	1.6	0.4	0.4	0.1	0.2	0.1	0.5	0.0	0.1
SNAR-A10	1.6	0.4	0.4	0.1	0.2	0.1	0.5	0.0	0.1
SNAR-A9	1.6	0.4	0.4	0.1	0.2	0.1	0.5	0.0	0.1
SNAR-A8	1.6	0.4	0.4	0.1	0.2	0.1	0.5	0.0	0.1
SNAR-A7	1.6	0.4	0.4	0.1	0.2	0.1	0.5	0.0	0.1
SNAR-A6	1.6	0.4	0.4	0.1	0.2	0.1	0.5	0.0	0.1
SNAR-A5	1.6	0.4	0.4	0.1	0.2	0.1	0.5	0.0	0.1
SNAR-A14	1.6	0.4	0.4	0.1	0.2	0.1	0.5	0.0	0.1
SNAR-A4	1.6	0.4	0.4	0.1	0.2	0.1	0.5	0.0	0.1
SNAR-A3	1.6	0.4	0.4	0.1	0.2	0.1	0.5	0.0	0.1
SLC34A3	1.2	0.5	0.8	0.1	0.3	0.2	0.3	0.1	0.2
USP30-AS1	0.8	0.2	0.6	0.1	0.2	0.2	0.5	0.2	0.7
CEBPA	0.7	0.5	0.3	0.4	0.2	0.2	0.4	0.3	0.5
LPAR2	0.8	0.4	0.7	0.2	0.2	0.3	0.5	0.1	0.2
ZBED1	1.1	0.3	0.4	0.1	0.2	0.2	0.6	0.1	0.5
ZBED1	1.1	0.3	0.4	0.1	0.2	0.2	0.6	0.1	0.5
NCAPG	1.0	0.3	0.4	0.0	0.2	0.3	0.8	0.1	0.4
LOC101928263	1.2	0.3	0.8	0.0	0.2	0.3	0.4	0.0	0.4
FAIM3	0.7	0.4	0.3	0.3	0.3	0.5	0.4	0.1	0.4
ZNF85	1.1	0.3	0.5	0.1	0.0	0.2	0.7	0.1	0.5
PRRX1	0.8	0.3	0.6	0.1	0.4	0.3	0.5	0.1	0.4
ILDR2	0.5	0.3	0.4	0.1	0.2	0.3	1.0	0.2	0.6
NR6A1	1.1	0.1	0.4	0.1	0.2	0.2	0.6	0.1	0.7
LOC100131655	0.8	0.5	0.4	0.3	0.3	0.3	0.6	0.1	0.3
ZFP37	1.0	0.5	0.5	0.0	0.1	0.1	0.7	0.1	0.4
SPOCK1	0.8	0.4	0.3	0.1	0.4	0.4	0.5	0.0	0.5
ZNF613	0.9	0.3	0.6	0.0	0.1	0.2	0.6	0.2	0.6
CDK18	0.8	0.4	0.5	0.1	0.2	0.3	0.5	0.1	0.5
LOC284930	1.2	0.3	0.5	0.0	0.1	0.2	0.5	0.1	0.5
TSPAN12	0.8	0.4	0.5	0.0	0.2	0.1	0.7	0.1	0.7
PBX4	1.0	0.2	0.6	0.2	0.2	0.3	0.4	0.1	0.5
GPR37	1.2	0.5	0.7	0.1	0.2	0.3	0.3	0.0	0.2
GPR162	1.0	0.5	0.5	0.1	0.2	0.2	0.6	0.1	0.4
SPATA1	0.9	0.3	0.3	0.0	0.2	0.1	0.7	0.2	0.7
DEPDC1B	0.9	0.4	0.6	0.1	0.1	0.3	0.8	0.1	0.2
LOC101928739	1.6	0.2	0.4	0.1	0.5	0.1	0.5	0.0	0.2
ARSD	1.0	0.3	0.6	0.1	0.4	0.2	0.5	0.0	0.3
IL6R	1.3	0.3	0.5	0.0	0.1	0.0	0.5	0.1	0.6
OXCT1	0.9	0.2	0.3	0.1	0.1	0.3	0.8	0.1	0.6
ZNF470	0.7	0.3	0.6	0.0	0.2	0.4	0.7	0.1	0.4
LOC101928998	0.9	0.2	0.5	0.1	0.3	0.3	0.6	0.1	0.6
LOC642643	1.0	0.3	0.5	0.2	0.1	0.3	0.7	0.1	0.4

LOC101927030	0.9	0.3	0.5	0.1	0.3	0.3	0.8	0.0	0.2
ST3GAL4-AS1	0.9	0.3	0.7	0.1	0.1	0.2	0.6	0.2	0.6
HEATR8-TTC4	1.0	0.3	0.5	0.1	0.1	0.2	0.7	0.1	0.5
LOC101928951	1.4	0.2	0.9	0.0	0.0	0.1	0.5	0.0	0.3
CYB5RL	1.0	0.3	0.4	0.0	0.2	0.3	0.5	0.1	0.5
SCIN	1.2	0.2	0.6	0.1	0.1	0.2	0.7	0.1	0.3
LOC401480	1.4	0.3	0.4	0.1	0.1	0.1	0.8	0.1	0.3
LOC100287467	1.0	0.3	0.5	0.2	0.1	0.1	0.8	0.2	0.3
LRRC37A3	0.9	0.4	0.6	0.1	0.2	0.2	0.6	0.0	0.4
LOC101928236	1.2	0.5	0.5	0.0	0.2	0.1	0.4	0.2	0.4
LOC101059918	1.7	0.4	0.6	0.1	0.1	0.2	0.2	0.0	0.1
CNTD2	1.0	0.5	0.6	0.0	0.1	0.1	0.6	0.1	0.4
LOC400748	1.2	0.3	0.4	0.1	0.1	0.1	0.8	0.1	0.3
LOC101928736	1.3	0.3	0.5	0.0	0.2	0.2	0.4	0.1	0.4
CD177	0.6	0.4	0.4	0.3	0.3	0.3	0.5	0.1	0.6
KCNG2	0.9	0.2	0.3	0.1	0.0	0.1	0.9	0.2	0.7
PPM1L	1.2	0.3	0.4	0.1	0.2	0.2	0.5	0.1	0.4
SEC61A2	1.1	0.4	0.7	0.1	0.1	0.2	0.5	0.1	0.4
KIAA1324L	1.0	0.3	0.5	0.1	0.2	0.2	0.6	0.1	0.5
DHRS2	1.1	0.5	0.4	0.0	0.1	0.2	0.5	0.0	0.5
LOC145783	0.9	0.4	0.4	0.1	0.2	0.3	0.8	0.1	0.3
ZNF674	1.0	0.4	0.4	0.0	0.1	0.2	0.6	0.0	0.7
SLC35G6	1.4	0.3	0.6	0.2	0.1	0.2	0.3	0.1	0.2
IFIH1	1.3	0.4	0.5	0.0	0.2	0.2	0.3	0.1	0.4
SLC24A5	0.9	0.5	0.5	0.1	0.1	0.2	0.5	0.0	0.5
LINC00957	1.3	0.4	0.4	0.1	0.1	0.1	0.4	0.1	0.5
LOC101060341	0.9	0.3	0.7	0.1	0.4	0.3	0.3	0.1	0.2
PON2	1.0	0.5	0.8	0.0	0.1	0.2	0.4	0.1	0.3
LOC101927657	1.3	0.2	0.5	0.0	0.1	0.1	0.7	0.1	0.3
FAM117A	1.1	0.5	0.6	0.1	0.1	0.3	0.3	0.1	0.2
LOC730100	1.1	0.1	0.5	0.1	0.2	0.2	0.8	0.1	0.4
FANCM	1.1	0.3	0.3	0.1	0.1	0.2	0.8	0.1	0.5
FIBCD1	0.6	0.4	0.7	0.1	0.2	0.3	0.7	0.0	0.3
LOC101929173	1.2	0.2	0.4	0.1	0.2	0.2	0.6	0.1	0.5
LOC100996437	1.3	0.3	0.5	0.1	0.1	0.4	0.4	0.1	0.3
LOC101929428	1.1	0.2	0.4	0.0	0.2	0.1	0.9	0.1	0.4
LOC101928210	1.3	0.2	0.3	0.1	0.3	0.1	0.6	0.1	0.3
VN1R2	1.1	0.3	0.4	0.1	0.1	0.2	0.7	0.1	0.4
LOC101929177	0.9	0.3	0.6	0.0	0.1	0.1	0.7	0.1	0.5
FBXL13	1.0	0.4	0.6	0.0	0.1	0.3	0.6	0.1	0.4
ZBTB26	0.7	0.3	0.5	0.1	0.2	0.3	0.6	0.1	0.6
TSNAXIP1	1.1	0.5	0.8	0.1	0.2	0.3	0.3	0.0	0.1
LOC101928700	1.3	0.2	0.4	0.1	0.2	0.1	0.6	0.1	0.4
IL12RB1	1.0	0.3	0.5	0.0	0.1	0.2	0.8	0.1	0.4
EMILIN2	0.8	0.3	0.6	0.1	0.2	0.1	0.7	0.1	0.4
LOC101927021	1.2	0.3	0.4	0.1	0.1	0.2	0.6	0.0	0.3
DICER1-AS1	1.0	0.3	0.5	0.1	0.3	0.1	0.5	0.1	0.4
LOC389199	1.1	0.3	0.5	0.1	0.2	0.2	0.6	0.0	0.3
LRRC37A2	0.9	0.4	0.5	0.1	0.1	0.3	0.7	0.0	0.3
LOC283682	1.1	0.3	0.5	0.1	0.2	0.2	0.6	0.1	0.4
JRKL-AS1	1.4	0.2	0.4	0.1	0.1	0.1	0.7	0.0	0.2
LOC101928725	0.8	0.4	0.6	0.0	0.2	0.2	0.8	0.1	0.1
SPECC1L	0.8	0.3	0.8	0.0	0.1	0.2	0.6	0.1	0.5
POC1B-GALNT4	0.7	0.3	0.1	0.2	0.7	0.1	1.0	0.1	0.0
PGAP1	1.0	0.4	0.4	0.0	0.2	0.2	0.7	0.1	0.4

SMPD3	0.8	0.3	0.4	0.1	0.1	0.2	0.9	0.1	0.5
LOC100130100	1.3	0.2	0.5	0.1	0.1	0.3	0.5	0.1	0.3
LOC101929707	1.0	0.3	0.5	0.1	0.2	0.2	0.7	0.1	0.3
LOC101927423	0.9	0.2	0.7	0.3	0.2	0.3	0.4	0.1	0.1
BANK1	1.3	0.5	0.5	0.0	0.1	0.1	0.5	0.1	0.4
ZNF280C	1.0	0.5	0.5	0.0	0.1	0.3	0.3	0.1	0.5
HAS2-AS1	0.7	0.2	0.4	0.2	0.3	0.4	0.5	0.1	0.5
C2orf76	1.1	0.4	0.6	0.0	0.1	0.0	0.4	0.2	0.5
OSGEPL1	0.9	0.3	0.6	0.0	0.1	0.2	0.5	0.1	0.6
FLG-AS1	1.0	0.2	0.4	0.0	0.2	0.2	0.8	0.1	0.5
JAZF1	0.8	0.3	0.3	0.2	0.1	0.7	0.4	0.0	0.4
SLC2A13	1.0	0.3	0.6	0.1	0.1	0.2	0.5	0.1	0.3
LOC101929714	1.1	0.1	0.5	0.1	0.0	0.1	0.9	0.0	0.4
AK9	1.0	0.3	0.5	0.0	0.1	0.2	0.5	0.1	0.4
AXIN2	0.8	0.4	0.6	0.1	0.1	0.3	0.5	0.1	0.3
LOC101928539	1.3	0.2	0.4	0.1	0.1	0.1	0.8	0.1	0.3
LOC101928898	1.3	0.3	0.6	0.1	0.2	0.2	0.5	0.0	0.1
SLC25A14	0.9	0.4	0.6	0.0	0.2	0.3	0.5	0.1	0.4
LOC439951	0.8	0.3	0.5	0.3	0.2	0.1	0.3	0.3	0.5
GRID1	1.3	0.3	0.6	0.1	0.1	0.2	0.4	0.1	0.3
LOC100131530	1.3	0.2	0.4	0.1	0.2	0.2	0.6	0.1	0.3
ZNF184	0.7	0.4	0.4	0.0	0.3	0.2	0.6	0.1	0.7
SLC25A45	1.2	0.4	0.5	0.1	0.4	0.1	0.2	0.1	0.3
MB21D2	0.7	0.2	0.2	0.1	0.1	0.2	1.1	0.1	0.5
OVCH1-AS1	0.6	0.2	0.6	0.2	0.2	0.3	0.5	0.1	0.5
FLJ46906	0.6	0.3	0.2	0.1	0.1	0.2	1.1	0.1	0.6
SLC10A5	1.7	0.3	0.4	0.0	0.2	0.0	0.5	0.0	0.2
PCDH12	1.0	0.2	0.5	0.0	0.1	0.2	0.8	0.1	0.4
C14orf64	1.0	0.3	0.5	0.1	0.2	0.2	0.8	0.0	0.3
IKZF2	1.1	0.3	0.5	0.1	0.2	0.3	0.3	0.0	0.5
VWA5A	1.2	0.4	0.7	0.1	0.2	0.2	0.3	0.0	0.2
PELI2	0.2	0.1	0.2	0.1	0.1	0.2	1.1	0.2	1.1
NKX3-2	0.8	0.5	0.5	0.1	0.3	0.2	0.6	0.1	0.2
ZNF567	0.8	0.4	0.7	0.1	0.1	0.2	0.5	0.1	0.4
LOC101928069	1.1	0.4	0.6	0.0	0.1	0.2	0.4	0.1	0.4
ASB2	1.5	0.4	0.8	0.1	0.1	0.2	0.1	0.1	0.0
KRT34	0.7	0.1	0.4	0.1	0.1	0.2	1.0	0.1	0.6
LINC00622	1.2	0.3	0.5	0.0	0.0	0.0	0.6	0.1	0.5
SLMO1	0.7	0.2	0.3	0.0	0.1	0.0	1.0	0.2	0.8
LOC101927412	1.4	0.2	0.6	0.1	0.1	0.1	0.4	0.0	0.3
GANC	1.2	0.4	0.4	0.1	0.1	0.1	0.5	0.1	0.4
C17orf97	0.8	0.3	0.4	0.1	0.2	0.6	0.3	0.1	0.3
LOC101928853	1.3	0.2	0.4	0.1	0.1	0.1	0.8	0.1	0.1
FKBP6	1.0	0.2	0.3	0.1	0.1	0.1	0.9	0.1	0.4
ZNF823	0.9	0.4	0.4	0.0	0.1	0.1	0.7	0.0	0.5
TXNDC16	1.4	0.3	0.4	0.0	0.1	0.1	0.5	0.0	0.4
LOC101928976	0.6	0.5	0.3	0.1	0.3	0.2	0.6	0.1	0.6
LOC101928437	1.1	0.2	0.4	0.1	0.1	0.1	0.8	0.1	0.4
NR2F2-AS1	0.7	0.2	0.5	0.1	0.2	0.2	0.6	0.0	0.9
LOC100128340	0.9	0.3	0.4	0.1	0.1	0.1	1.0	0.0	0.5
LRRC2	0.4	0.2	0.2	0.0	0.2	0.2	1.0	0.2	0.9
LOC101928445	1.0	0.3	0.6	0.1	0.2	0.1	0.4	0.1	0.3
TIAM2	1.0	0.2	0.4	0.1	0.2	0.2	0.8	0.1	0.3
KCNIP2	0.9	0.3	0.6	0.2	0.3	0.2	0.4	0.0	0.3
PON3	0.9	0.3	0.6	0.1	0.1	0.2	0.7	0.1	0.4

SLC4A5	1.0	0.4	0.5	0.1	0.2	0.3	0.3	0.0	0.3
WT1	0.1	0.6	0.2	0.7	0.4	0.0	0.4	0.5	0.3
C1orf226	1.0	0.5	0.6	0.1	0.2	0.2	0.3	0.1	0.3
ZNF528	0.8	0.3	0.6	0.1	0.3	0.3	0.5	0.1	0.3
LINC00707	0.6	0.2	0.3	0.1	0.3	0.3	0.6	0.1	0.8
SERPINI1	0.9	0.4	0.6	0.0	0.1	0.1	0.6	0.1	0.4
CLDN6	1.3	0.2	0.7	0.1	0.2	0.0	0.4	0.1	0.3
ARMCX4	0.8	0.2	0.4	0.0	0.2	0.1	0.8	0.1	0.5
ZNF136	1.0	0.3	0.4	0.0	0.1	0.2	0.8	0.1	0.4
ADORA2A-AS1	1.3	0.2	0.5	0.1	0.1	0.1	0.5	0.1	0.3
KIAA1984-AS1	0.7	0.3	0.5	0.1	0.2	0.2	0.6	0.1	0.5
TLCD2	1.1	0.4	0.5	0.1	0.1	0.1	0.6	0.1	0.2
ELL3	0.5	0.4	0.4	0.0	0.2	0.1	0.7	0.1	0.7
HSD17B2	0.7	0.2	0.3	0.1	0.2	0.2	0.8	0.1	0.5
CCDC15	0.7	0.2	0.5	0.0	0.1	0.2	0.8	0.1	0.6
LOC153684	0.7	0.2	0.6	0.0	0.3	0.4	0.6	0.1	0.3
FLJ45974	0.9	0.2	0.6	0.0	0.2	0.2	0.6	0.1	0.5
SLC45A3	0.8	0.3	0.4	0.1	0.1	0.2	0.9	0.1	0.3
DEPDC1	0.9	0.3	0.6	0.1	0.2	0.3	0.5	0.1	0.3
MYO1F	0.9	0.3	0.6	0.1	0.3	0.2	0.4	0.1	0.4
ARHGAP4	1.1	0.3	0.5	0.1	0.1	0.2	0.4	0.1	0.4
TTLL7	0.7	0.4	0.4	0.1	0.2	0.3	0.6	0.1	0.5
LOC101928926	0.9	0.2	0.4	0.1	0.1	0.3	0.6	0.1	0.5
RGPD6	0.9	0.1	0.5	0.0	0.1	0.2	0.8	0.1	0.5
RTTN	1.0	0.3	0.4	0.0	0.1	0.2	0.6	0.1	0.4
PGBD4	0.9	0.3	0.4	0.0	0.1	0.2	0.7	0.0	0.5
VWA5B2	0.8	0.3	0.4	0.1	0.1	0.1	0.7	0.1	0.5
PCDHGA4	1.1	0.3	0.5	0.1	0.1	0.2	0.6	0.0	0.2
ROPN1L-AS1	1.1	0.2	0.4	0.0	0.1	0.1	0.7	0.0	0.5
PTGFRN	1.1	0.4	0.5	0.1	0.1	0.2	0.5	0.1	0.2
LTC4S	0.9	0.2	0.6	0.1	0.2	0.1	0.4	0.1	0.4
AMACR	0.8	0.5	0.5	0.0	0.2	0.2	0.6	0.1	0.3
PDE3B	0.7	0.3	0.4	0.0	0.1	0.1	0.8	0.1	0.7
CTRL	0.9	0.3	0.6	0.1	0.1	0.2	0.3	0.1	0.7
LOC441528	0.8	0.2	0.4	0.1	0.2	0.3	0.7	0.1	0.5
NTN5	1.1	0.5	0.5	0.1	0.1	0.1	0.4	0.1	0.2
SLC10A1	1.3	0.2	0.5	0.1	0.1	0.2	0.4	0.1	0.3
LOC100996301	0.6	0.6	0.5	0.0	0.3	0.2	0.4	0.1	0.5
LOC101929515	1.0	0.2	0.5	0.1	0.1	0.1	0.7	0.1	0.3
PHKA2-AS1	1.1	0.6	0.5	0.0	0.0	0.2	0.4	0.1	0.3
IL11RA	0.9	0.4	0.5	0.1	0.3	0.2	0.4	0.1	0.2
BSCL2	1.0	0.4	0.5	0.1	0.1	0.2	0.5	0.0	0.3
PPP1R32	1.2	0.3	0.5	0.1	0.2	0.3	0.4	0.1	0.2
PPAPDC1A	0.5	0.4	0.5	0.2	0.5	0.6	0.3	0.0	0.2
CCDC144A	0.8	0.3	0.6	0.1	0.1	0.1	0.6	0.1	0.5
LOC100499484-C9ORF174	1.3	0.3	0.6	0.1	0.1	0.2	0.4	0.0	0.2
LOC100506465	0.8	0.3	0.5	0.1	0.1	0.2	0.5	0.2	0.5
LMCD1-AS1	1.1	0.2	0.3	0.1	0.2	0.1	0.8	0.0	0.2
AKAP6	1.4	0.4	0.6	0.0	0.1	0.2	0.2	0.0	0.2
ZNF606	1.0	0.3	0.5	0.0	0.2	0.3	0.5	0.0	0.3
LOC729173	0.9	0.2	0.4	0.1	0.1	0.1	0.9	0.1	0.3
CCDC176	1.2	0.3	0.4	0.0	0.1	0.2	0.4	0.1	0.5
TRAM2-AS1	0.9	0.4	0.5	0.1	0.2	0.2	0.4	0.1	0.3
BBS5	1.1	0.3	0.3	0.0	0.1	0.2	0.4	0.1	0.5
RGS20	0.7	0.3	0.4	0.0	0.1	0.3	0.8	0.1	0.5

BORA	0.9	0.3	0.4	0.1	0.2	0.1	0.5	0.1	0.6
LOC100129931	1.0	0.1	0.4	0.1	0.1	0.1	1.0	0.1	0.3
LOC101928796	1.1	0.5	0.4	0.1	0.2	0.4	0.2	0.1	0.1
LOC101927382	0.8	0.2	0.5	0.0	0.1	0.2	0.7	0.1	0.5
B3GNT7	0.7	0.2	0.4	0.2	0.1	0.4	0.6	0.2	0.4
TMPRSS9	0.9	0.2	0.4	0.2	0.2	0.1	0.6	0.2	0.4
ZNF852	0.7	0.2	0.5	0.0	0.1	0.1	0.7	0.1	0.7
BCDIN3D	1.0	0.4	0.5	0.1	0.1	0.1	0.6	0.0	0.3
CKLF-CMTM1	0.3	0.1	0.2	0.2	0.5	0.2	0.8	0.0	0.9
B3GNT4	1.3	0.3	0.5	0.1	0.1	0.1	0.4	0.0	0.3
FOXD1	0.3	0.5	0.5	0.4	0.2	0.1	0.4	0.3	0.3
ARID3A	0.8	0.3	0.3	0.1	0.2	0.2	0.6	0.2	0.5
CHRM4	0.9	0.3	0.2	0.1	0.2	0.2	0.6	0.1	0.5
HTR2C	1.2	0.4	0.6	0.0	0.3	0.2	0.2	0.0	0.1
DBH-AS1	0.7	0.4	0.6	0.2	0.3	0.3	0.4	0.1	0.2
MURC	0.6	0.3	0.5	0.0	0.2	0.2	0.9	0.1	0.3
HDX	1.0	0.3	0.3	0.0	0.1	0.2	0.6	0.1	0.6
FLJ45872	1.1	0.2	0.4	0.1	0.1	0.2	0.5	0.1	0.4
DCDC2	1.2	0.7	0.4	0.0	0.1	0.1	0.2	0.0	0.2
C4B	1.2	0.5	0.7	0.0	0.1	0.1	0.3	0.0	0.2
ZNF420	0.9	0.4	0.5	0.0	0.1	0.1	0.5	0.1	0.5
FPGT	0.8	0.4	0.6	0.1	0.2	0.2	0.5	0.0	0.4
ANGPTL6	0.8	0.3	0.4	0.1	0.2	0.3	0.6	0.1	0.3
SEMA3G	0.9	0.3	0.5	0.1	0.2	0.2	0.4	0.1	0.3
PTPN22	0.7	0.3	0.5	0.0	0.1	0.2	0.8	0.1	0.5
MED12L	0.8	0.3	0.4	0.1	0.1	0.2	0.6	0.1	0.6
FMR1-AS1	0.5	0.5	0.4	0.4	0.1	0.1	0.4	0.3	0.3
LOC283070	1.2	0.4	0.7	0.1	0.1	0.2	0.1	0.0	0.2
ACPT	0.5	0.2	0.5	0.1	0.1	0.2	0.7	0.1	0.6
RBP5	1.3	0.3	0.6	0.1	0.1	0.2	0.2	0.1	0.2
ARHGEF4	1.1	0.3	0.4	0.1	0.2	0.2	0.3	0.0	0.4
LOC284600	1.0	0.3	0.4	0.1	0.2	0.1	0.6	0.0	0.3
SLC25A21-AS1	0.7	0.3	0.5	0.1	0.1	0.2	0.5	0.2	0.5
RBKS	1.2	0.4	0.5	0.1	0.1	0.1	0.4	0.1	0.2
LOC100288142	0.8	0.2	0.3	0.0	0.1	0.1	0.8	0.1	0.6
LOC730139	0.9	0.5	0.5	0.1	0.2	0.3	0.3	0.0	0.2
GALNT16	0.8	0.2	0.4	0.2	0.3	0.3	0.4	0.1	0.3
USP2	0.6	0.4	0.5	0.0	0.1	0.1	0.5	0.1	0.7
CDCA2	0.8	0.4	0.3	0.1	0.2	0.3	0.5	0.1	0.4
KANK3	1.1	0.3	0.4	0.1	0.3	0.1	0.6	0.0	0.2
LOC100507520	0.8	0.4	0.6	0.1	0.1	0.3	0.4	0.1	0.3
ERC2	0.8	0.2	0.3	0.0	0.1	0.2	0.6	0.1	0.6
LOC101929385	1.0	0.3	0.6	0.0	0.1	0.1	0.6	0.0	0.3
HOXB-AS1	0.1	0.2	0.3	1.8	0.1	0.0	0.4	0.1	0.1
GCNT1	0.8	0.3	0.5	0.0	0.2	0.2	0.6	0.1	0.4
MYO3B	0.9	0.2	0.5	0.0	0.2	0.2	0.7	0.1	0.3
POF1B	1.0	0.5	0.5	0.1	0.1	0.2	0.3	0.1	0.3
LOC100134040	1.0	0.2	0.4	0.1	0.1	0.2	0.7	0.0	0.3
C3orf65	0.9	0.1	0.4	0.1	0.2	0.2	0.9	0.1	0.3
CNIH2	0.8	0.4	0.6	0.1	0.2	0.1	0.4	0.1	0.3
LOC101927915	0.7	0.3	0.7	0.1	0.2	0.2	0.4	0.2	0.4
PLXNC1	0.9	0.3	0.5	0.1	0.1	0.2	0.5	0.1	0.5
LOC101927896	1.2	0.2	0.5	0.1	0.1	0.0	0.7	0.1	0.3
ZNF837	0.7	0.4	0.4	0.2	0.2	0.3	0.6	0.1	0.2
LOC100505912	1.2	0.3	0.4	0.0	0.2	0.0	0.6	0.1	0.4

TFEB	1.0	0.2	0.5	0.2	0.1	0.3	0.5	0.1	0.3
GEN1	0.8	0.3	0.4	0.1	0.2	0.2	0.6	0.1	0.5
DGCR6	1.2	0.4	0.6	0.2	0.1	0.1	0.3	0.0	0.2
ZNF182	0.9	0.4	0.4	0.0	0.1	0.2	0.6	0.0	0.5
LOC729970	1.0	0.3	0.4	0.1	0.1	0.2	0.5	0.0	0.4
NPIPA3	0.8	0.3	0.4	0.1	0.2	0.2	0.5	0.1	0.4
DLX4	0.8	0.4	0.4	0.1	0.2	0.2	0.3	0.1	0.5
LOC101059948	1.2	0.4	0.5	0.1	0.1	0.3	0.3	0.0	0.1
PLA2G4A	0.8	0.3	0.5	0.0	0.2	0.2	0.6	0.1	0.4
LOC389332	1.2	0.1	0.4	0.1	0.2	0.2	0.5	0.1	0.2
NUDT18	1.3	0.4	0.4	0.1	0.2	0.3	0.3	0.0	0.1
RBPMS	1.0	0.4	0.5	0.1	0.1	0.2	0.2	0.3	0.4
CRYBB2	0.8	0.5	0.4	0.0	0.2	0.3	0.6	0.0	0.3
D21S2088E	0.9	0.2	0.4	0.1	0.2	0.2	0.7	0.0	0.4
LOC100505938	1.1	0.3	0.5	0.1	0.1	0.2	0.3	0.0	0.3
LOC254128	0.9	0.2	0.3	0.1	0.1	0.2	0.7	0.0	0.4
IGFLR1	0.5	0.3	0.4	0.1	0.2	0.2	0.7	0.1	0.6
ANKRD36	0.8	0.2	0.5	0.0	0.2	0.2	0.6	0.1	0.5
LOC101929661	0.3	0.1	0.0	0.2	0.2	0.5	0.7	0.1	0.8
PIWIL4	1.2	0.4	0.5	0.0	0.1	0.1	0.4	0.1	0.2
BRSK2	0.8	0.3	0.5	0.2	0.2	0.2	0.3	0.1	0.4
LOC101929555	1.3	0.2	0.4	0.0	0.1	0.1	0.5	0.0	0.3
AKR1C2	1.1	0.4	0.7	0.1	0.1	0.3	0.1	0.0	0.2
CHRNA5	0.5	0.2	0.2	0.1	0.2	0.3	0.8	0.1	0.6
C8G	0.8	0.3	0.6	0.1	0.1	0.4	0.3	0.1	0.3
LOC101928288	0.9	0.2	0.5	0.1	0.1	0.2	0.8	0.0	0.3
CHRNE	0.9	0.4	0.6	0.0	0.1	0.1	0.4	0.1	0.3
PBX1	1.0	0.5	0.4	0.1	0.3	0.3	0.2	0.0	0.2
PAMR1	1.1	0.4	0.6	0.0	0.2	0.3	0.1	0.1	0.2
LINC00668	0.9	0.2	0.4	0.1	0.1	0.1	0.7	0.0	0.5
ADAM22	0.7	0.3	0.4	0.1	0.2	0.3	0.5	0.1	0.4
C1orf74	0.9	0.5	0.4	0.1	0.1	0.2	0.4	0.1	0.3
LOC101928621	1.0	0.1	0.4	0.1	0.0	0.0	0.9	0.0	0.4
FEZF1-AS1	0.7	0.2	0.4	0.1	0.1	0.2	0.6	0.1	0.4
LOC101927151	0.8	0.3	0.5	0.1	0.1	0.3	0.4	0.1	0.4
FENDRR	1.1	0.1	0.4	0.1	0.2	0.1	0.6	0.0	0.4
JARID2-AS1	0.8	0.2	0.5	0.2	0.2	0.4	0.5	0.2	0.0
LOC101927316	1.1	0.2	0.4	0.1	0.2	0.1	0.6	0.0	0.3
TRPC1	0.8	0.3	0.6	0.0	0.0	0.3	0.6	0.0	0.3
INADL	1.0	0.3	0.5	0.0	0.1	0.2	0.4	0.0	0.4
APITD1-CORT	0.7	0.2	0.2	0.2	0.2	0.3	0.8	0.1	0.3
CRMP1	0.8	0.2	0.3	0.2	0.2	0.5	0.4	0.1	0.2
ERCC6-PGBD3	0.9	0.3	0.4	0.0	0.2	0.2	0.5	0.1	0.4
PCDHB15	1.0	0.2	0.6	0.1	0.2	0.2	0.4	0.1	0.1
WNK3	0.8	0.3	0.4	0.0	0.1	0.2	0.6	0.1	0.6
SNAP91	1.1	0.4	0.4	0.0	0.2	0.2	0.3	0.0	0.3
SMCR5	1.1	0.1	0.4	0.1	0.2	0.2	0.6	0.1	0.3
LOC101929574	1.1	0.2	0.5	0.1	0.2	0.0	0.5	0.1	0.3
KIAA1683	1.2	0.4	0.6	0.1	0.1	0.1	0.2	0.1	0.2
LOC100507191	0.8	0.3	0.6	0.1	0.1	0.3	0.3	0.1	0.4
DCLK2	0.8	0.2	0.4	0.1	0.1	0.1	0.7	0.1	0.4
LOC255130	0.9	0.3	0.6	0.2	0.2	0.2	0.2	0.1	0.3
EME1	0.6	0.3	0.3	0.1	0.3	0.2	0.6	0.2	0.6
LOC100289637	1.2	0.3	0.5	0.1	0.1	0.1	0.4	0.1	0.2
ERI2	0.6	0.5	0.4	0.1	0.1	0.3	0.5	0.0	0.4

PPM1H	0.9	0.4	0.5	0.1	0.3	0.2	0.3	0.1	0.2
TRPV2	0.9	0.4	0.5	0.1	0.2	0.2	0.3	0.0	0.2
LOC100288152	0.9	0.4	0.4	0.0	0.1	0.2	0.5	0.1	0.3
LOC101928414	0.9	0.2	0.3	0.0	0.2	0.1	0.8	0.1	0.4
LOC101929469	0.9	0.2	0.3	0.1	0.3	0.5	0.2	0.0	0.4
MCHR2-AS1	0.9	0.3	0.4	0.0	0.1	0.0	0.5	0.1	0.7
FGF18	1.2	0.3	0.6	0.1	0.0	0.1	0.3	0.0	0.3
QPCT	0.7	0.4	0.7	0.1	0.1	0.2	0.3	0.1	0.4
ACTL10	0.7	0.3	0.7	0.1	0.2	0.3	0.3	0.1	0.3
LOC101928845	0.8	0.2	0.5	0.1	0.2	0.2	0.7	0.1	0.2
SPTB	0.9	0.3	0.4	0.0	0.1	0.1	0.6	0.1	0.4
CASZ1	0.6	0.2	0.2	0.1	0.1	0.1	0.8	0.1	0.6
DNAJC25-GNG10	0.9	0.7	0.2	0.0	0.0	0.2	0.6	0.0	0.2
FBXO4	0.8	0.4	0.3	0.1	0.3	0.2	0.3	0.1	0.4
HHIP-AS1	0.6	0.2	0.4	0.1	0.2	0.1	0.7	0.1	0.5
TMEM62	0.8	0.3	0.4	0.0	0.1	0.1	0.6	0.1	0.5
TAGLN3	0.8	0.3	0.5	0.0	0.1	0.1	0.7	0.0	0.4
TEKT4	1.0	0.3	0.4	0.1	0.2	0.2	0.3	0.1	0.3
IL2RB	1.1	0.3	0.5	0.0	0.2	0.1	0.4	0.1	0.2
LOC101927166	0.9	0.2	0.5	0.1	0.1	0.1	0.8	0.0	0.3
CD7	0.5	0.4	0.4	0.1	0.2	0.2	0.7	0.1	0.3
ZNF57	0.6	0.3	0.3	0.0	0.1	0.3	0.6	0.1	0.7
NBPF11	1.0	0.2	0.5	0.0	0.1	0.1	0.6	0.1	0.4
DNAH2	1.2	0.3	0.6	0.1	0.2	0.2	0.2	0.0	0.1
DCAF4L1	1.5	0.3	0.3	0.1	0.0	0.1	0.5	0.0	0.1
RRAD	0.9	0.2	0.5	0.0	0.1	0.1	0.4	0.0	0.6
LOC101927081	1.0	0.3	0.6	0.0	0.2	0.1	0.5	0.0	0.2
SLC25A27	0.9	0.2	0.4	0.1	0.2	0.3	0.6	0.0	0.2
TBX19	0.7	0.3	0.5	0.1	0.1	0.1	0.6	0.2	0.4
SKOR1	0.7	0.2	0.4	0.1	0.1	0.2	0.5	0.2	0.5
STEAP2	1.1	0.3	0.7	0.0	0.1	0.2	0.2	0.0	0.3
LOC646976	0.8	0.2	0.3	0.1	0.2	0.2	0.5	0.1	0.4
ZNF345	1.2	0.2	0.2	0.1	0.1	0.2	0.5	0.0	0.4
LOC100131395	0.8	0.2	0.4	0.1	0.1	0.2	0.7	0.1	0.3
SEBOX	0.8	0.1	0.3	0.2	0.3	0.2	0.6	0.0	0.4
C16orf86	0.8	0.3	0.6	0.1	0.1	0.1	0.7	0.1	0.2
FANK1	1.0	0.2	0.5	0.0	0.1	0.2	0.4	0.2	0.3
LOC100506392	1.0	0.1	0.3	0.0	0.0	0.2	0.9	0.0	0.4
LOC101928476	1.0	0.3	0.4	0.0	0.1	0.0	0.7	0.0	0.3
TICAM2	0.5	0.0	0.6	0.1	0.3	0.6	0.6	0.3	0.0
LOC101927790	1.0	0.1	0.3	0.1	0.1	0.2	0.9	0.0	0.1
ZNF92	0.9	0.4	0.4	0.0	0.1	0.1	0.5	0.1	0.5
LOC101928192	0.7	0.4	0.4	0.1	0.1	0.3	0.5	0.0	0.3
CDADC1	0.8	0.4	0.5	0.0	0.1	0.1	0.5	0.0	0.4
FBP1	0.5	0.2	0.3	0.1	0.2	0.2	0.7	0.0	0.7
PRRT2	0.9	0.3	0.5	0.1	0.1	0.3	0.3	0.1	0.2
LOC100506473	0.9	0.3	0.4	0.0	0.1	0.1	0.6	0.1	0.3
MMS22L	0.7	0.2	0.4	0.0	0.1	0.1	0.6	0.1	0.6
ADAMTS7	1.0	0.3	0.6	0.1	0.1	0.2	0.3	0.0	0.3
LOC100996515	0.8	0.1	0.3	0.1	0.2	0.1	1.0	0.0	0.4
NPAS1	1.0	0.3	0.5	0.1	0.2	0.1	0.4	0.1	0.3
FAM78A	1.0	0.2	0.3	0.1	0.4	0.1	0.4	0.2	0.2
LOC101927719	1.0	0.1	0.3	0.0	0.1	0.1	0.8	0.1	0.3
LOC101927106	0.8	0.3	0.4	0.0	0.1	0.2	0.4	0.1	0.4
MFAP4	0.6	0.3	0.6	0.0	0.3	0.1	0.5	0.0	0.4

RARB	1.0	0.3	0.6	0.0	0.2	0.2	0.2	0.1	0.2
PARK2	1.0	0.3	0.5	0.0	0.2	0.3	0.4	0.0	0.1
RUNX1-IT1	0.6	0.1	0.3	0.1	0.2	0.3	0.7	0.0	0.5
MIR4458HG	0.7	0.3	0.4	0.1	0.1	0.2	0.5	0.1	0.5
ATL1	1.0	0.3	0.3	0.0	0.1	0.2	0.3	0.1	0.3
TRIM74	0.5	0.5	0.7	0.1	0.1	0.2	0.5	0.1	0.2
LOC650293	0.7	0.2	0.5	0.0	0.1	0.2	0.9	0.0	0.3
OLFML2A	0.8	0.3	0.4	0.2	0.4	0.2	0.3	0.1	0.2
TAS1R3	0.9	0.4	0.6	0.1	0.1	0.2	0.3	0.1	0.3
FAM66D	0.8	0.3	0.4	0.1	0.2	0.1	0.3	0.0	0.6
OLFML3	0.7	0.5	0.4	0.0	0.1	0.2	0.5	0.1	0.3
NPTXR	0.8	0.4	0.5	0.1	0.1	0.3	0.4	0.1	0.3
LOC157273	1.0	0.2	0.4	0.1	0.1	0.2	0.5	0.1	0.3
PLAC4	0.5	0.3	0.3	0.1	0.1	0.1	0.7	0.1	0.7
GPR63	0.5	0.2	0.3	0.0	0.2	0.1	0.8	0.1	0.6
TMEM190	0.7	0.4	0.3	0.1	0.2	0.2	0.6	0.1	0.1
C19orf57	0.8	0.3	0.4	0.1	0.1	0.3	0.5	0.1	0.4
DENND1C	1.0	0.4	0.6	0.1	0.1	0.1	0.2	0.1	0.3
METTL25	0.8	0.5	0.4	0.0	0.1	0.1	0.4	0.0	0.5
ZNF235	0.9	0.3	0.4	0.1	0.1	0.2	0.6	0.0	0.3
RNF43	1.1	0.3	0.4	0.1	0.1	0.2	0.3	0.1	0.3
GHRL	1.0	0.2	0.5	0.1	0.3	0.2	0.2	0.0	0.3
LOC100131540	0.9	0.3	0.4	0.0	0.1	0.2	0.5	0.1	0.3
ABCC2	0.7	0.3	0.4	0.0	0.0	0.1	0.7	0.1	0.5
ZNF709	1.0	0.3	0.3	0.0	0.1	0.1	0.6	0.0	0.3
ZNF257	0.7	0.3	0.4	0.0	0.1	0.3	0.5	0.1	0.4
ZNF215	0.5	0.4	0.4	0.1	0.1	0.1	0.7	0.1	0.4
LINC00324	0.7	0.3	0.5	0.0	0.1	0.2	0.6	0.1	0.4
OASL	0.8	0.4	0.5	0.0	0.1	0.2	0.5	0.1	0.2
HAS3	0.8	0.2	0.5	0.1	0.3	0.4	0.2	0.1	0.2
OR5P2	0.9	0.1	0.4	0.1	0.3	0.1	0.7	0.0	0.2
AHRR	0.6	0.2	0.2	0.2	0.3	0.4	0.5	0.0	0.4
CARD9	0.6	0.2	0.4	0.1	0.1	0.2	0.7	0.1	0.5
RASSF4	0.8	0.6	0.5	0.1	0.2	0.2	0.2	0.0	0.2
GATSL2	0.9	0.2	0.6	0.0	0.1	0.2	0.2	0.1	0.4
LOC101927100	1.5	0.1	0.4	0.0	0.1	0.0	0.4	0.1	0.2
NUDT8	0.4	0.3	0.4	0.3	0.4	0.5	0.3	0.1	0.2
ATP7B	0.8	0.3	0.4	0.0	0.2	0.2	0.4	0.1	0.3
FLJ45513	1.0	0.1	0.3	0.2	0.0	0.2	0.5	0.1	0.3
ZNF816-ZNF321P	0.6	0.3	0.6	0.0	0.1	0.1	0.2	0.0	0.9
LINC00528	0.9	0.3	0.4	0.1	0.1	0.1	0.7	0.1	0.2
LOC100288123	0.8	0.3	0.4	0.0	0.1	0.2	0.6	0.1	0.2
SYTL1	0.9	0.4	0.3	0.0	0.1	0.2	0.5	0.1	0.2
A2ML1	0.9	0.2	0.4	0.0	0.1	0.1	0.8	0.0	0.3
TRIM66	0.7	0.1	0.3	0.1	0.2	0.2	0.6	0.0	0.5
BCAN	0.7	0.3	0.3	0.1	0.3	0.2	0.5	0.1	0.4
ZNF782	0.9	0.3	0.5	0.0	0.1	0.1	0.5	0.1	0.3
TGFB3	0.9	0.2	0.6	0.1	0.2	0.1	0.4	0.0	0.2
LOC101929399	0.5	0.4	0.5	0.1	0.2	0.1	0.6	0.0	0.4
ZNF774	0.6	0.4	0.3	0.1	0.1	0.3	0.4	0.1	0.4
RNFT1	0.7	0.5	0.4	0.0	0.0	0.2	0.6	0.1	0.4
ZNF736	0.7	0.1	0.2	0.0	0.2	0.2	0.7	0.1	0.5
LOC100996571	1.1	0.2	0.3	0.0	0.1	0.1	0.5	0.1	0.4
SELPLG	0.9	0.2	0.4	0.1	0.1	0.1	0.5	0.1	0.4
HSPA2	0.9	0.3	0.5	0.0	0.1	0.2	0.3	0.1	0.3

ZNF836	1.0	0.3	0.5	0.0	0.1	0.2	0.3	0.1	0.3
ZNF30	0.9	0.2	0.4	0.1	0.1	0.2	0.6	0.0	0.3
BBS4	0.7	0.3	0.6	0.1	0.2	0.3	0.3	0.0	0.4
CLDN22	0.7	0.3	0.5	0.0	0.1	0.1	0.5	0.1	0.4
C10orf25	0.6	0.2	0.3	0.0	0.2	0.3	0.2	0.1	0.8
TNS1	0.5	0.2	0.3	0.1	0.2	0.3	0.5	0.1	0.5
FLJ13224	0.7	0.2	0.3	0.1	0.0	0.1	0.8	0.1	0.4
PDK3	1.0	0.3	0.5	0.0	0.2	0.1	0.3	0.1	0.4
NPIPB9	0.8	0.3	0.3	0.1	0.2	0.3	0.4	0.0	0.3
LRRC4	0.7	0.1	0.6	0.1	0.2	0.0	0.8	0.1	0.2
SWT1	1.0	0.3	0.5	0.0	0.1	0.1	0.4	0.0	0.3
TTC39B	0.6	0.3	0.5	0.0	0.1	0.1	0.6	0.0	0.5
NOTCH3	1.0	0.3	0.5	0.1	0.1	0.2	0.3	0.1	0.2
CROT	0.8	0.3	0.4	0.0	0.2	0.1	0.4	0.0	0.6
CNTRL	0.8	0.3	0.4	0.0	0.1	0.2	0.5	0.1	0.4
LOC101929289	0.6	0.4	0.8	0.0	0.1	0.1	0.3	0.1	0.3
CLDN4	1.0	0.3	0.4	0.1	0.1	0.1	0.4	0.1	0.2
ACAD11	1.0	0.4	0.4	0.0	0.1	0.1	0.4	0.1	0.2
LOC101927624	0.9	0.2	0.4	0.1	0.0	0.2	0.7	0.0	0.2
LINC00617	1.1	0.2	0.4	0.1	0.1	0.2	0.4	0.0	0.3
SH3D21	0.8	0.3	0.5	0.1	0.1	0.2	0.4	0.0	0.3
KIAA0895	0.7	0.4	0.4	0.1	0.2	0.2	0.4	0.1	0.3
DYDC2	0.9	0.3	0.5	0.0	0.1	0.1	0.3	0.1	0.4
VASN	0.9	0.3	0.4	0.1	0.1	0.2	0.4	0.1	0.2
BLM	0.7	0.2	0.4	0.0	0.1	0.2	0.6	0.1	0.4
LOC101927817	0.8	0.1	0.5	0.0	0.2	0.2	0.8	0.1	0.2
ZP3	1.0	0.2	0.3	0.0	0.1	0.2	0.5	0.1	0.4
GAL3ST2	0.8	0.3	0.5	0.1	0.1	0.1	0.4	0.1	0.3
RANBP17	0.8	0.3	0.4	0.0	0.1	0.2	0.5	0.0	0.3
SPTBN4	1.0	0.5	0.4	0.1	0.1	0.1	0.2	0.0	0.2
LOC284837	0.7	0.3	0.4	0.1	0.1	0.2	0.5	0.1	0.4
ST14	0.9	0.3	0.5	0.1	0.1	0.2	0.3	0.1	0.2
CDKL5	0.7	0.3	0.4	0.0	0.2	0.2	0.5	0.1	0.4
SLC6A8	1.0	0.3	0.4	0.1	0.1	0.2	0.3	0.1	0.2
ADPGK-AS1	0.9	0.2	0.4	0.0	0.0	0.2	0.6	0.1	0.3
RAD54B	1.1	0.1	0.2	0.0	0.1	0.1	0.5	0.1	0.4
ZNF780B	0.7	0.2	0.5	0.0	0.1	0.1	0.6	0.0	0.4
LOC101927774	0.8	0.1	0.4	0.0	0.1	0.3	0.4	0.0	0.5
LOC100130691	0.7	0.3	0.4	0.1	0.1	0.3	0.5	0.0	0.4
DNAJC27	0.9	0.2	0.5	0.0	0.1	0.1	0.4	0.0	0.3
LOC113230	0.7	0.3	0.4	0.1	0.1	0.2	0.4	0.1	0.5
P2RX5	0.8	0.3	0.4	0.0	0.1	0.1	0.6	0.1	0.4
TRIM9	0.5	0.2	0.3	0.1	0.1	0.2	0.8	0.1	0.5
CCDC19	0.5	0.3	0.3	0.1	0.2	0.2	0.5	0.1	0.5
RTN4R	0.6	0.3	0.3	0.0	0.1	0.1	0.6	0.1	0.6
FAM24B-CUZD1	0.8	0.3	0.4	0.0	0.0	0.1	0.5	0.1	0.4
LOC100289580	0.8	0.2	0.4	0.0	0.1	0.1	0.6	0.1	0.5
LOC100133331	0.7	0.3	0.5	0.1	0.2	0.2	0.3	0.1	0.3
NIPAL1	0.6	0.2	0.3	0.0	0.2	0.2	0.5	0.0	0.7
ADIPOQ-AS1	0.9	0.2	0.4	0.1	0.1	0.2	0.6	0.0	0.2
C2orf48	0.8	0.3	0.3	0.1	0.2	0.1	0.5	0.1	0.4
IL17RE	0.8	0.3	0.4	0.0	0.1	0.2	0.5	0.0	0.3
GPC4	0.9	0.4	0.4	0.0	0.2	0.2	0.3	0.1	0.2
CACNB4	0.4	0.2	0.2	0.0	0.1	0.1	1.0	0.1	0.5
CARF	1.0	0.4	0.4	0.0	0.1	0.1	0.4	0.0	0.3

C11orf82	0.6	0.2	0.3	0.0	0.1	0.2	0.6	0.1	0.6
LRRC56	0.8	0.1	0.5	0.0	0.2	0.1	0.5	0.0	0.3
ZNF846	0.9	0.2	0.4	0.0	0.1	0.1	0.6	0.0	0.3
TAS2R3	1.1	0.1	0.3	0.0	0.1	0.2	0.5	0.0	0.2
ADRA1B	0.4	0.4	0.4	0.1	0.0	0.1	0.7	0.1	0.5
ZNF101	0.7	0.2	0.4	0.1	0.2	0.1	0.5	0.0	0.4
CNTNAP3B	1.1	0.2	0.5	0.0	0.2	0.1	0.2	0.0	0.3
LOC101928524	0.8	0.3	0.4	0.0	0.1	0.1	0.5	0.1	0.3
GDPGP1	0.5	0.3	0.5	0.1	0.3	0.3	0.5	0.1	0.4
KCNS1	0.8	0.2	0.4	0.1	0.1	0.1	0.6	0.1	0.4
ZNF624	0.7	0.2	0.4	0.0	0.1	0.2	0.4	0.1	0.6
BRIP1	0.6	0.2	0.3	0.0	0.1	0.2	0.6	0.1	0.4
LOC101060494	0.8	0.1	0.6	0.1	0.0	0.3	0.4	0.0	0.4
TBC1D30	0.4	0.2	0.4	0.0	0.1	0.2	0.6	0.1	0.6
LOC100131320	0.7	0.3	0.3	0.1	0.2	0.2	0.5	0.1	0.4
C1orf106	0.8	0.3	0.3	0.1	0.1	0.2	0.6	0.0	0.2
PCDHGA6	1.0	0.3	0.4	0.1	0.2	0.2	0.3	0.0	0.2
INPP5J	0.9	0.4	0.4	0.1	0.1	0.2	0.3	0.1	0.2
FAM222A-AS1	0.9	0.2	0.2	0.0	0.1	0.3	0.3	0.1	0.5
LOC101928063	0.9	0.4	0.4	0.0	0.1	0.2	0.3	0.0	0.3
FCGBP	1.1	0.3	0.5	0.1	0.1	0.2	0.2	0.0	0.1
TMEM144	0.8	0.2	0.5	0.0	0.2	0.2	0.2	0.1	0.5
PAFAH2	0.8	0.3	0.4	0.0	0.1	0.2	0.4	0.0	0.3
PCDH17	1.1	0.4	0.4	0.1	0.1	0.1	0.3	0.1	0.2
LOC285501	0.6	0.2	0.5	0.0	0.2	0.1	0.7	0.0	0.3
LOC100507012	0.2	0.5	0.3	0.5	0.1	0.1	0.5	0.3	0.3
PARP11	0.8	0.3	0.4	0.0	0.1	0.2	0.3	0.1	0.3
LOC101927901	0.7	0.2	0.4	0.0	0.2	0.2	0.4	0.1	0.4
CENPH	0.6	0.3	0.3	0.3	0.2	0.0	0.5	0.1	0.3
ZNF10	0.9	0.2	0.3	0.0	0.1	0.1	0.5	0.0	0.5
MCEE	0.7	0.2	0.4	0.1	0.2	0.2	0.5	0.1	0.3
PROCA1	0.7	0.3	0.4	0.0	0.1	0.2	0.5	0.1	0.4
C1orf110	0.5	0.0	0.3	0.1	0.2	0.3	0.4	0.1	0.6
SFR1	0.7	0.2	0.4	0.1	0.1	0.2	0.4	0.1	0.4
OXTR	0.6	0.1	0.2	0.1	0.2	0.1	0.8	0.1	0.4
KIAA1328	0.6	0.2	0.3	0.0	0.2	0.2	0.4	0.1	0.5
LOC101927365	0.6	0.3	0.5	0.0	0.1	0.1	0.5	0.0	0.4
UNC5CL	0.8	0.2	0.4	0.2	0.2	0.3	0.3	0.0	0.2
FAM110B	1.1	0.1	0.3	0.1	0.1	0.1	0.5	0.1	0.2
GATS	0.7	0.2	0.4	0.1	0.1	0.2	0.4	0.0	0.4
LOC101928043	0.9	0.2	0.5	0.0	0.2	0.1	0.5	0.0	0.2
TMED6	0.8	0.1	0.4	0.1	0.2	0.2	0.5	0.1	0.3
PGM5-AS1	0.8	0.2	0.3	0.2	0.0	0.1	0.5	0.1	0.5
TOX	0.7	0.4	0.3	0.1	0.3	0.2	0.3	0.1	0.3
UCP3	0.9	0.4	0.4	0.1	0.0	0.1	0.4	0.1	0.2
SLC22A4	0.6	0.3	0.4	0.1	0.2	0.4	0.5	0.0	0.2
LOC101927451	0.6	0.3	0.4	0.0	0.1	0.2	0.5	0.1	0.4
CLDN23	0.7	0.3	0.4	0.1	0.2	0.3	0.4	0.1	0.1
SSX4B	1.0	0.2	0.3	0.1	0.1	0.2	0.3	0.1	0.4
SSX4	1.0	0.2	0.3	0.1	0.1	0.2	0.3	0.1	0.4
DLG1-AS1	0.9	0.3	0.4	0.1	0.2	0.2	0.4	0.0	0.2
MIR4697HG	0.7	0.3	0.3	0.1	0.1	0.2	0.6	0.0	0.4
BTBD18	1.0	0.2	0.5	0.0	0.1	0.1	0.4	0.0	0.3
SIRPB1	1.0	0.4	0.6	0.1	0.1	0.1	0.2	0.0	0.1
ANKRD32	0.5	0.2	0.4	0.0	0.1	0.1	0.6	0.1	0.5

BZRAP1	0.6	0.2	0.4	0.1	0.1	0.2	0.5	0.1	0.4
LOC729164	1.0	0.1	0.2	0.0	0.1	0.2	0.5	0.1	0.4
RUSC1-AS1	0.8	0.2	0.6	0.1	0.4	0.1	0.3	0.1	0.1
ESR2	1.0	0.2	0.4	0.1	0.1	0.3	0.4	0.0	0.3
LOC101929065	0.8	0.2	0.3	0.1	0.2	0.2	0.5	0.0	0.2
SAMD9	0.8	0.4	0.3	0.0	0.2	0.2	0.4	0.0	0.4
FAM212A	0.8	0.3	0.5	0.0	0.1	0.1	0.6	0.1	0.2
AUNIP	0.4	0.2	0.2	0.1	0.1	0.1	0.6	0.1	0.6
LOC101927184	1.1	0.2	0.3	0.0	0.1	0.1	0.4	0.0	0.4
CHST5	0.8	0.1	0.4	0.2	0.2	0.2	0.5	0.0	0.1
TRNAN20	0.6	0.2	0.5	0.0	0.1	0.1	0.6	0.1	0.3
TRNAN17	0.6	0.2	0.5	0.0	0.1	0.1	0.6	0.1	0.3
TRNAN7	0.6	0.2	0.5	0.0	0.1	0.1	0.6	0.1	0.3
TRNAN11	0.6	0.2	0.5	0.0	0.1	0.1	0.6	0.1	0.3
TRNAN32	0.6	0.2	0.5	0.0	0.1	0.1	0.6	0.1	0.3
TRNAN1	0.6	0.2	0.5	0.0	0.1	0.1	0.6	0.1	0.3
LOC101928233	1.1	0.2	0.4	0.1	0.2	0.1	0.3	0.0	0.2
VN1R1	0.8	0.3	0.4	0.0	0.2	0.2	0.4	0.0	0.3
ZNF880	0.7	0.3	0.4	0.0	0.1	0.1	0.4	0.0	0.5
ZNF25	0.7	0.2	0.5	0.0	0.2	0.1	0.6	0.0	0.3
NEIL3	0.6	0.2	0.3	0.0	0.3	0.1	0.7	0.1	0.4
LINC00235	0.9	0.4	0.4	0.1	0.1	0.1	0.4	0.0	0.2
ASTN2	0.8	0.1	0.3	0.0	0.1	0.1	0.6	0.1	0.4
LRRC26	0.4	0.2	0.3	0.1	0.1	0.2	0.8	0.1	0.3
LGALS9	0.9	0.4	0.2	0.1	0.1	0.2	0.4	0.0	0.3
C9orf135-AS1	1.0	0.2	0.3	0.0	0.0	0.2	0.7	0.0	0.2
CACNA2D1	0.9	0.3	0.4	0.0	0.1	0.1	0.3	0.0	0.3
LRRC69	0.9	0.4	0.3	0.0	0.1	0.1	0.6	0.0	0.3
LOC284801	0.7	0.1	0.3	0.2	0.1	0.1	0.7	0.1	0.3
LOC100287808	0.7	0.4	0.4	0.1	0.1	0.2	0.5	0.1	0.3
NPHP1	0.9	0.3	0.4	0.0	0.2	0.1	0.3	0.0	0.3
CCNE2	0.6	0.2	0.3	0.1	0.1	0.1	0.7	0.0	0.5
PPM1N	0.8	0.3	0.4	0.1	0.1	0.1	0.3	0.0	0.3
PDZK1	0.9	0.2	0.4	0.0	0.1	0.1	0.4	0.0	0.4
LOC101926907	0.8	0.1	0.7	0.1	0.0	0.1	0.4	0.1	0.3
GPR17	0.2	0.0	0.1	0.0	0.0	0.1	0.9	0.3	0.9
TMOD2	0.6	0.3	0.4	0.0	0.1	0.1	0.5	0.1	0.5
LOC101927595	0.8	0.0	0.4	0.1	0.0	0.1	0.6	0.1	0.5
FGF11	0.5	0.2	0.4	0.1	0.1	0.3	0.3	0.2	0.5
PROSER2-AS1	1.0	0.2	0.4	0.0	0.1	0.1	0.4	0.0	0.3
ALPK1	1.0	0.4	0.5	0.0	0.2	0.1	0.3	0.0	0.1
ULK4	1.0	0.3	0.4	0.0	0.1	0.1	0.4	0.0	0.3
TTN-AS1	0.7	0.2	0.4	0.0	0.2	0.2	0.5	0.1	0.3
LOC100130256	0.9	0.2	0.3	0.1	0.1	0.1	0.7	0.1	0.1
LINC00643	1.0	0.2	0.4	0.1	0.1	0.2	0.3	0.1	0.2
FLJ27354	0.7	0.1	0.4	0.0	0.0	0.2	0.7	0.1	0.3
LOC101927380	0.8	0.1	0.4	0.0	0.0	0.1	0.6	0.1	0.3
TGM1	0.6	0.3	0.4	0.1	0.1	0.2	0.5	0.1	0.3
LOC101927555	0.7	0.3	0.4	0.1	0.2	0.2	0.5	0.1	0.2
SYT7	0.7	0.3	0.3	0.0	0.1	0.1	0.5	0.1	0.4
LINC00989	1.0	0.1	0.3	0.1	0.1	0.2	0.5	0.0	0.2
LOC101928504	0.7	0.3	0.4	0.0	0.2	0.1	0.5	0.1	0.3
PYROXD1	0.7	0.3	0.6	0.0	0.1	0.1	0.5	0.1	0.2
FAM200A	0.7	0.2	0.3	0.0	0.1	0.1	0.6	0.0	0.4
LOC101928707	1.1	0.2	0.3	0.0	0.0	0.2	0.3	0.0	0.4

C19orf73	1.0	0.2	0.3	0.1	0.0	0.3	0.5	0.1	0.1
MAK	0.7	0.1	0.3	0.1	0.2	0.2	0.6	0.0	0.3
KREMEN2	0.7	0.2	0.3	0.1	0.1	0.2	0.5	0.1	0.2
ASPM	0.6	0.3	0.3	0.0	0.2	0.2	0.5	0.1	0.3
SPDYE3	0.7	0.2	0.4	0.1	0.1	0.3	0.4	0.0	0.2
SOX2	0.8	0.2	0.5	0.1	0.1	0.2	0.4	0.1	0.1
NKX2-1	0.5	0.3	0.5	0.2	0.1	0.1	0.4	0.2	0.2
ZDHHC22	0.6	0.1	0.3	0.0	0.1	0.1	0.6	0.1	0.6
PNPLA1	0.5	0.2	0.2	0.0	0.1	0.1	0.8	0.1	0.5
LOC101928966	0.6	0.2	0.4	0.0	0.1	0.2	0.6	0.1	0.3
LOC100996350	0.6	0.2	0.4	0.0	0.1	0.2	0.6	0.1	0.3
LOC101929358	0.2	0.3	0.5	0.2	0.2	0.1	0.6	0.1	0.2
PPFIA2	0.8	0.2	0.3	0.0	0.1	0.1	0.5	0.1	0.4
MSANTD1	0.8	0.1	0.4	0.1	0.1	0.1	0.5	0.0	0.3
LOC115110	0.6	0.2	0.5	0.1	0.1	0.1	0.5	0.1	0.4
SPTBN5	0.8	0.2	0.4	0.1	0.2	0.1	0.4	0.0	0.3
STK33	0.7	0.3	0.3	0.0	0.2	0.2	0.4	0.1	0.4
IRAK1BP1	0.6	0.4	0.4	0.1	0.1	0.2	0.3	0.2	0.2
ADRBK2	0.8	0.2	0.3	0.1	0.1	0.2	0.5	0.1	0.3
ZNF253	0.8	0.4	0.4	0.0	0.2	0.1	0.3	0.0	0.3
LOC101927768	0.6	0.3	0.4	0.1	0.1	0.4	0.5	0.0	0.2
SRCRB4D	0.9	0.4	0.4	0.0	0.1	0.1	0.3	0.1	0.2
EFCAB7	0.7	0.3	0.5	0.0	0.1	0.2	0.4	0.1	0.2
LINC00933	0.8	0.2	0.3	0.1	0.1	0.1	0.6	0.0	0.3
ZNF75D	0.8	0.2	0.3	0.0	0.1	0.1	0.7	0.0	0.3
IFIT1	0.9	0.3	0.5	0.0	0.2	0.1	0.4	0.1	0.1
LOC101927123	0.8	0.1	0.3	0.0	0.1	0.1	0.8	0.1	0.1
KIAA1009	0.8	0.2	0.4	0.0	0.1	0.1	0.4	0.0	0.4
ABCA1	0.9	0.3	0.4	0.1	0.3	0.3	0.1	0.0	0.1
SBSN	0.4	0.1	0.3	0.0	0.1	0.1	0.7	0.3	0.6
LOC101927377	0.9	0.2	0.4	0.0	0.1	0.1	0.5	0.1	0.2
PINLYP	0.5	0.2	0.4	0.0	0.1	0.2	0.5	0.2	0.5
LOC101929397	0.9	0.2	0.2	0.1	0.1	0.1	0.6	0.1	0.2
AGAP9	1.2	0.4	0.6	0.0	0.0	0.0	0.1	0.0	0.1
LACC1	0.7	0.3	0.3	0.0	0.2	0.1	0.5	0.0	0.2
SIDT1	0.7	0.3	0.4	0.0	0.1	0.2	0.4	0.0	0.3
FAM149A	0.9	0.4	0.4	0.1	0.1	0.3	0.2	0.1	0.0
SPOCD1	0.6	0.4	0.4	0.2	0.3	0.3	0.2	0.0	0.1
LOC100996663	0.9	0.2	0.4	0.0	0.1	0.1	0.4	0.1	0.3
PCDHGA11	0.9	0.3	0.5	0.0	0.2	0.1	0.3	0.0	0.2
IL20RB	0.7	0.4	0.4	0.0	0.0	0.1	0.5	0.0	0.4
VSIG10L	0.6	0.3	0.4	0.1	0.2	0.3	0.3	0.0	0.2
ACRBP	0.7	0.3	0.5	0.1	0.1	0.1	0.3	0.1	0.4
CDKL3	0.8	0.2	0.4	0.0	0.0	0.1	0.5	0.1	0.4
ADTRP	0.3	0.3	0.2	0.0	0.1	0.1	0.7	0.2	0.4
GNAT2	1.2	0.2	0.4	0.0	0.0	0.2	0.3	0.0	0.2
ZNF883	0.7	0.3	0.4	0.0	0.1	0.1	0.5	0.0	0.4
LOC100507616	0.8	0.2	0.4	0.0	0.1	0.2	0.4	0.0	0.3
ZNF717	0.7	0.3	0.3	0.0	0.1	0.2	0.5	0.0	0.3
GZMM	0.5	0.4	0.4	0.0	0.2	0.0	0.6	0.1	0.3
PDE5A	0.9	0.3	0.4	0.0	0.1	0.1	0.3	0.0	0.3
MTSS1	0.9	0.3	0.4	0.1	0.1	0.2	0.2	0.1	0.2
LINC00534	0.6	0.2	0.3	0.1	0.1	0.1	0.7	0.1	0.3
LOC100996415	0.7	0.1	0.3	0.2	0.2	0.3	0.5	0.0	0.2
TSGA10	0.8	0.3	0.3	0.0	0.1	0.2	0.4	0.1	0.2

GLYCTK	0.7	0.2	0.4	0.1	0.2	0.2	0.5	0.0	0.2
SPDYE2	0.8	0.2	0.4	0.0	0.1	0.1	0.5	0.1	0.2
NRIP2	0.8	0.2	0.4	0.0	0.1	0.3	0.4	0.0	0.3
LOC100507639	0.8	0.2	0.5	0.1	0.1	0.0	0.4	0.1	0.2
LOC101927228	0.7	0.1	0.3	0.1	0.1	0.1	0.7	0.0	0.2
C6orf164	1.1	0.2	0.4	0.0	0.1	0.1	0.4	0.0	0.1
ZFAND4	0.6	0.2	0.4	0.0	0.1	0.1	0.5	0.1	0.4
LOC100996419	0.5	0.2	0.4	0.0	0.2	0.2	0.2	0.2	0.4
FAM27B	0.0	0.5	0.6	0.0	0.1	0.0	0.9	0.0	0.3
SAMSN1	0.6	0.2	0.4	0.0	0.2	0.1	0.5	0.1	0.5
VHLL	0.7	0.3	0.2	0.1	0.2	0.1	0.5	0.2	0.2
C1QL1	0.8	0.3	0.4	0.1	0.3	0.1	0.2	0.1	0.1
ENOX1	0.4	0.2	0.2	0.0	0.1	0.2	0.7	0.1	0.4
KIAA1586	0.5	0.2	0.4	0.0	0.1	0.2	0.5	0.1	0.4
INTS6-AS1	1.0	0.2	0.5	0.0	0.1	0.0	0.3	0.1	0.3
ZNF169	0.7	0.3	0.3	0.1	0.1	0.2	0.4	0.1	0.2
IL17RB	0.9	0.2	0.4	0.0	0.0	0.2	0.3	0.1	0.4
LOC101928731	0.9	0.1	0.3	0.1	0.2	0.1	0.5	0.1	0.2
UGT2A1	0.9	0.2	0.4	0.0	0.0	0.2	0.4	0.0	0.2
GRAMD1C	0.7	0.4	0.2	0.0	0.2	0.2	0.4	0.0	0.3
ODF3B	0.4	0.4	0.4	0.2	0.4	0.2	0.2	0.0	0.2
ZNF223	0.6	0.4	0.3	0.0	0.1	0.2	0.3	0.0	0.5
CNTN5	0.6	0.3	0.3	0.1	0.1	0.2	0.6	0.1	0.3
LOC643723	0.7	0.2	0.5	0.0	0.1	0.3	0.3	0.0	0.2
RAPSN	0.6	0.2	0.4	0.0	0.1	0.2	0.5	0.0	0.2
CORT	0.5	0.2	0.3	0.0	0.0	0.2	1.0	0.1	0.0
LOC101929731	0.3	0.2	0.3	0.0	0.1	0.2	0.6	0.1	0.6
CAPN9	0.7	0.2	0.4	0.1	0.1	0.1	0.5	0.0	0.2
LOC101929162	0.8	0.2	0.3	0.1	0.0	0.1	0.5	0.1	0.3
COL1A1	0.7	0.3	0.3	0.1	0.2	0.1	0.4	0.1	0.2
LOC100506331	0.7	0.2	0.4	0.0	0.1	0.1	0.6	0.0	0.3
LRRC48	0.4	0.1	0.4	0.1	0.1	0.2	0.6	0.1	0.4
LOC101928910	1.1	0.2	0.5	0.0	0.0	0.1	0.1	0.1	0.3
LOC101927780	0.9	0.2	0.5	0.0	0.0	0.1	0.5	0.0	0.2
GGN	0.7	0.3	0.4	0.1	0.1	0.1	0.4	0.0	0.2
TBC1D8B	0.7	0.2	0.4	0.0	0.2	0.2	0.4	0.0	0.3
DGKI	0.9	0.3	0.4	0.0	0.1	0.1	0.5	0.0	0.1
DTX4	0.7	0.3	0.4	0.0	0.0	0.1	0.5	0.1	0.2
CCDC136	0.9	0.2	0.3	0.1	0.1	0.1	0.3	0.0	0.2
LOC101928989	0.8	0.2	0.5	0.0	0.0	0.1	0.5	0.0	0.3
LOC101928683	0.8	0.2	0.4	0.0	0.1	0.1	0.5	0.0	0.2
LOC101927221	0.7	0.2	0.3	0.0	0.2	0.1	0.4	0.1	0.3
LOC100506157	0.7	0.2	0.4	0.1	0.2	0.1	0.3	0.1	0.4
MBD5	0.9	0.2	0.4	0.0	0.2	0.1	0.3	0.0	0.3
NHSL2	0.7	0.2	0.5	0.1	0.2	0.2	0.1	0.1	0.2
ZNF256	0.5	0.3	0.2	0.0	0.2	0.2	0.5	0.0	0.5
SNAI3	0.8	0.2	0.2	0.1	0.1	0.3	0.4	0.0	0.2
CKMT2	0.9	0.0	0.4	0.0	0.1	0.1	0.4	0.1	0.2
CLIP3	0.8	0.3	0.5	0.0	0.2	0.1	0.1	0.0	0.2
GLUD2	0.6	0.2	0.3	0.1	0.0	0.1	0.5	0.1	0.5
LOC100133182	0.6	0.2	0.4	0.1	0.1	0.1	0.5	0.1	0.3
SHOX	0.7	0.2	0.3	0.0	0.1	0.1	0.6	0.0	0.3
SHOX	0.7	0.2	0.3	0.0	0.1	0.1	0.6	0.0	0.3
TRNAG5	0.2	0.2	0.2	0.1	0.3	0.1	0.1	1.1	0.1
ZNF625-ZNF20	0.8	0.2	0.4	0.0	0.1	0.1	0.4	0.1	0.3

ZNF675	0.6	0.2	0.4	0.0	0.1	0.1	0.6	0.0	0.3
C11orf45	0.7	0.2	0.4	0.1	0.1	0.1	0.4	0.0	0.2
TSPAN1	0.7	0.3	0.4	0.0	0.1	0.1	0.5	0.1	0.2
ALG10	0.8	0.1	0.4	0.0	0.1	0.1	0.5	0.0	0.3
ZNF467	0.6	0.3	0.4	0.1	0.1	0.1	0.4	0.1	0.3
LOC101928537	1.0	0.1	0.4	0.0	0.1	0.1	0.5	0.1	0.2
TRANK1	0.9	0.2	0.3	0.0	0.1	0.2	0.3	0.0	0.2
PCDHGA5	0.7	0.2	0.4	0.1	0.1	0.1	0.4	0.1	0.2
OBSCN	0.7	0.2	0.4	0.1	0.1	0.2	0.4	0.1	0.3
ERV3-1	0.9	0.1	0.5	0.0	0.1	0.1	0.3	0.1	0.3
LOC101927861	0.5	0.1	0.4	0.1	0.5	0.2	0.3	0.0	0.3
INHBB	0.9	0.3	0.2	0.1	0.1	0.1	0.3	0.1	0.2
PTCH2	0.8	0.2	0.4	0.1	0.2	0.1	0.3	0.0	0.2
LOC101927979	0.5	0.3	0.4	0.0	0.2	0.1	0.2	0.0	0.5
TC2N	1.1	0.4	0.4	0.0	0.0	0.0	0.2	0.0	0.2
LOC643696	0.6	0.3	0.7	0.0	0.0	0.1	0.6	0.0	0.1
ZNF641	0.6	0.2	0.1	0.0	0.1	0.2	0.6	0.1	0.3
CCDC102B	0.8	0.3	0.4	0.0	0.1	0.1	0.4	0.0	0.2
LOC644656	0.7	0.2	0.2	0.1	0.1	0.1	0.6	0.0	0.3
LOC286297	0.7	0.1	0.4	0.0	0.1	0.1	0.5	0.1	0.3
CHRM3-AS2	0.4	0.2	0.5	0.0	0.1	0.3	0.4	0.1	0.3
ZNF443	0.8	0.4	0.3	0.0	0.0	0.1	0.4	0.0	0.3
ZNF585B	0.7	0.3	0.4	0.0	0.1	0.2	0.3	0.1	0.3
LOC100507656	0.8	0.3	0.4	0.1	0.1	0.2	0.3	0.0	0.1
CGA	0.6	0.1	0.1	0.2	0.1	0.3	0.6	0.0	0.2
LINC00854	0.4	0.0	0.4	0.0	0.2	0.1	0.7	0.1	0.3
PLK4	0.5	0.2	0.3	0.0	0.1	0.1	0.5	0.1	0.4
LOC150381	0.7	0.3	0.3	0.1	0.1	0.1	0.5	0.1	0.4
PLCG2	0.6	0.2	0.4	0.1	0.1	0.2	0.4	0.0	0.2
NPAS3	0.6	0.3	0.4	0.1	0.1	0.1	0.3	0.1	0.2
ROM1	0.7	0.5	0.5	0.0	0.0	0.1	0.2	0.1	0.2
ADAM32	0.9	0.2	0.3	0.0	0.1	0.1	0.4	0.0	0.2
TTTY15	0.7	0.2	0.3	0.0	0.1	0.1	0.6	0.0	0.3
FRMD4B	0.8	0.4	0.2	0.0	0.1	0.1	0.4	0.1	0.4
CEP57L1	0.8	0.2	0.3	0.1	0.1	0.2	0.3	0.1	0.3
LINC00894	0.7	0.2	0.4	0.0	0.2	0.1	0.4	0.1	0.3
LHX4	0.6	0.1	0.4	0.1	0.1	0.2	0.5	0.1	0.2
MEIS1	0.7	0.2	0.3	0.1	0.1	0.3	0.2	0.1	0.2
LINC00482	0.8	0.1	0.3	0.0	0.1	0.1	0.7	0.0	0.2
EPSTI1	0.5	0.2	0.3	0.1	0.1	0.3	0.3	0.0	0.4
SCT	0.7	0.4	0.4	0.1	0.1	0.1	0.4	0.1	0.2
HERC3	0.6	0.2	0.2	0.0	0.1	0.1	0.6	0.0	0.5
LINC00948	0.9	0.1	0.2	0.1	0.1	0.0	0.5	0.0	0.4
LOC101928615	1.0	0.1	0.4	0.0	0.0	0.1	0.5	0.0	0.2
LOC101926994	0.7	0.2	0.4	0.1	0.2	0.1	0.4	0.0	0.2
LOC101060581	0.7	0.1	0.3	0.1	0.1	0.2	0.3	0.1	0.3
VMO1	0.8	0.3	0.5	0.1	0.1	0.0	0.1	0.1	0.3
LOC101929112	0.3	0.3	0.5	0.0	0.2	0.2	0.4	0.0	0.2
C20orf144	0.7	0.2	0.3	0.1	0.3	0.1	0.4	0.1	0.1
LOC150577	0.8	0.1	0.3	0.1	0.2	0.1	0.5	0.0	0.2
ZNF799	0.7	0.3	0.3	0.0	0.2	0.0	0.3	0.0	0.4
FEZF1	0.5	0.2	0.3	0.0	0.1	0.2	0.4	0.2	0.4
RARRES1	1.0	0.2	0.5	0.0	0.0	0.1	0.2	0.0	0.2
PRKCQ-AS1	0.4	0.2	0.3	0.0	0.1	0.1	0.6	0.1	0.4
MAPK8IP2	0.9	0.2	0.3	0.1	0.2	0.2	0.3	0.0	0.2

IDI2-AS1	0.8	0.2	0.4	0.0	0.1	0.1	0.3	0.0	0.2
LOC283587	0.6	0.3	0.4	0.0	0.2	0.0	0.4	0.0	0.3
MN1	0.8	0.3	0.2	0.1	0.1	0.1	0.4	0.1	0.4
LOC101928885	0.9	0.1	0.3	0.1	0.1	0.1	0.5	0.0	0.2
RSG1	0.7	0.1	0.2	0.1	0.3	0.2	0.2	0.1	0.4
TXK	0.5	0.2	0.3	0.0	0.1	0.2	0.6	0.1	0.3
LOC101929715	0.5	0.3	0.2	0.0	0.1	0.1	0.6	0.0	0.4
USP6	0.7	0.2	0.3	0.0	0.1	0.1	0.4	0.1	0.4
LOC101927105	0.8	0.3	0.4	0.1	0.0	0.1	0.3	0.1	0.2
ORC1	0.4	0.2	0.2	0.1	0.1	0.1	0.6	0.1	0.5
PSMD6-AS2	0.7	0.2	0.4	0.0	0.1	0.1	0.4	0.1	0.3
JPH2	0.6	0.1	0.4	0.1	0.1	0.2	0.4	0.1	0.2
RAB26	0.8	0.2	0.4	0.1	0.4	0.1	0.1	0.0	0.1
CCDC88B	0.8	0.3	0.4	0.1	0.1	0.1	0.3	0.0	0.2
HLA-DRB1	0.7	0.1	0.3	0.1	0.3	0.1	0.4	0.1	0.3
PLEKHN1	0.6	0.2	0.3	0.1	0.2	0.3	0.2	0.0	0.2
ZNF449	0.6	0.3	0.4	0.1	0.1	0.1	0.5	0.0	0.3
LOC100996433	0.4	0.1	0.2	0.3	0.3	0.3	0.3	0.1	0.3
C3orf83	0.6	0.1	0.2	0.0	0.1	0.2	0.6	0.1	0.3
TYW1B	0.5	0.2	0.4	0.1	0.1	0.1	0.5	0.0	0.4
SLC12A5	0.8	0.4	0.4	0.1	0.1	0.2	0.2	0.0	0.1
C8orf31	0.6	0.1	0.2	0.1	0.1	0.3	0.5	0.0	0.2
LINC01011	0.6	0.3	0.4	0.0	0.2	0.2	0.4	0.0	0.2
LOC101928773	0.7	0.2	0.4	0.0	0.0	0.2	0.4	0.1	0.3
KLHDC1	0.6	0.3	0.4	0.0	0.2	0.1	0.2	0.0	0.3
GNG12-AS1	0.7	0.1	0.4	0.0	0.1	0.1	0.5	0.0	0.4
PHOSPHO1	0.6	0.2	0.4	0.0	0.1	0.2	0.5	0.1	0.2
C8orf48	0.6	0.2	0.4	0.0	0.0	0.1	0.3	0.1	0.5
LOC284395	0.9	0.1	0.3	0.1	0.1	0.1	0.5	0.0	0.2
COL9A2	0.7	0.3	0.3	0.0	0.2	0.1	0.3	0.1	0.1
LOC100506444	0.7	0.2	0.4	0.1	0.1	0.2	0.4	0.0	0.2
LOC339978	0.8	0.2	0.3	0.0	0.1	0.1	0.5	0.0	0.3
ARMC4	0.6	0.2	0.3	0.0	0.1	0.2	0.5	0.1	0.2
SGOL1	0.3	0.3	0.3	0.0	0.2	0.2	0.5	0.1	0.5
TP73	0.6	0.3	0.2	0.1	0.1	0.3	0.3	0.1	0.2
DNM1	0.9	0.2	0.4	0.1	0.1	0.2	0.1	0.0	0.1
SERPINF2	0.6	0.1	0.2	0.0	0.1	0.1	0.5	0.1	0.5
MCC	0.4	0.2	0.2	0.0	0.1	0.1	0.7	0.1	0.4
GLS2	0.6	0.2	0.3	0.1	0.0	0.1	0.5	0.1	0.4
ZNF615	0.7	0.3	0.4	0.0	0.2	0.1	0.3	0.0	0.3
TREX2	0.4	0.2	0.4	0.2	0.1	0.1	0.4	0.0	0.3
LOC100507540	0.6	0.2	0.4	0.1	0.1	0.1	0.5	0.0	0.2
ZNRD1-AS1	0.7	0.1	0.4	0.0	0.2	0.2	0.4	0.0	0.2
PPM1J	0.4	0.3	0.3	0.1	0.3	0.3	0.3	0.0	0.2
GLB1L3	0.6	0.2	0.4	0.0	0.2	0.2	0.3	0.1	0.2
TBC1D19	0.7	0.3	0.4	0.0	0.1	0.1	0.3	0.0	0.3
DLL4	0.3	0.1	0.2	0.0	0.1	0.0	0.8	0.2	0.6
MIR497HG	1.0	0.1	0.3	0.0	0.0	0.2	0.4	0.0	0.1
LOC100289333	0.8	0.1	0.4	0.1	0.1	0.1	0.3	0.1	0.2
LINC01016	0.8	0.2	0.3	0.0	0.1	0.1	0.4	0.0	0.2
CBWD3	0.5	0.3	0.2	0.0	0.1	0.2	0.5	0.0	0.3
VASH2	0.4	0.4	0.4	0.2	0.2	0.1	0.2	0.1	0.2
IGHM	0.5	0.1	0.4	0.0	0.0	0.1	0.5	0.1	0.4
LOC401589	0.6	0.2	0.3	0.1	0.1	0.1	0.6	0.0	0.3
KIF14	0.4	0.2	0.3	0.0	0.1	0.1	0.6	0.1	0.4

LRRC71	0.6	0.2	0.3	0.0	0.0	0.2	0.4	0.0	0.4
LOC101929253	0.5	0.3	0.4	0.1	0.1	0.1	0.4	0.1	0.3
LOC101929054	0.7	0.5	0.2	0.1	0.1	0.2	0.3	0.1	0.2
NEK3	0.8	0.2	0.4	0.0	0.1	0.1	0.3	0.1	0.2
CXCR5	0.7	0.2	0.4	0.0	0.2	0.1	0.3	0.0	0.2
KIAA1841	0.8	0.2	0.4	0.0	0.1	0.0	0.4	0.0	0.2
SND1-IT1	0.7	0.1	0.2	0.1	0.1	0.2	0.6	0.0	0.3
GJA3	0.3	0.1	0.1	0.1	0.1	0.1	0.6	0.2	0.7
HSD11B2	0.5	0.2	0.5	0.1	0.1	0.1	0.4	0.1	0.2
ARMC12	0.4	0.4	0.3	0.1	0.1	0.3	0.1	0.1	0.3
ZNF565	0.6	0.2	0.3	0.0	0.1	0.2	0.4	0.0	0.3
HCG4	0.7	0.3	0.3	0.1	0.1	0.2	0.3	0.0	0.2
LOC101927701	0.5	0.2	0.2	0.0	0.1	0.1	0.5	0.0	0.5
TRIM73	0.9	0.1	0.1	0.1	0.2	0.2	0.4	0.1	0.1
LOC100128882	0.6	0.3	0.3	0.1	0.1	0.1	0.4	0.1	0.2
ACSM4	0.7	0.2	0.2	0.0	0.1	0.1	0.5	0.1	0.2
STK4-AS1	0.7	0.1	0.2	0.0	0.1	0.1	0.6	0.1	0.3
GLIS3-AS1	0.9	0.2	0.3	0.0	0.0	0.0	0.4	0.2	0.1
LOC101928803	0.7	0.2	0.2	0.0	0.1	0.1	0.5	0.0	0.2
LOC101929728	0.5	0.1	0.2	0.1	0.3	0.2	0.5	0.1	0.2
ACACB	0.7	0.2	0.3	0.0	0.1	0.2	0.4	0.1	0.2
LOC283856	0.8	0.2	0.3	0.0	0.1	0.1	0.4	0.0	0.2
TRIM45	0.6	0.2	0.3	0.0	0.1	0.2	0.4	0.0	0.3
C1QTNF3-AMACR	0.7	0.2	0.3	0.0	0.1	0.1	0.3	0.1	0.2
LOC101929540	0.4	0.3	0.5	0.1	0.1	0.1	0.3	0.0	0.3
BRE-AS1	0.7	0.2	0.5	0.0	0.1	0.1	0.2	0.1	0.2
LOC101928755	0.7	0.3	0.2	0.1	0.2	0.2	0.0	0.0	0.4
SH3TC1	0.7	0.2	0.3	0.1	0.2	0.1	0.4	0.0	0.2
LINC00691	0.7	0.1	0.2	0.0	0.1	0.1	0.5	0.0	0.2
WDR17	0.6	0.3	0.3	0.0	0.1	0.2	0.3	0.1	0.2
ITGB7	0.7	0.2	0.3	0.0	0.1	0.2	0.4	0.1	0.2
PCSK5	0.6	0.2	0.3	0.0	0.1	0.2	0.3	0.1	0.3
PNPLA7	0.7	0.2	0.4	0.1	0.2	0.2	0.2	0.0	0.1
OAZ3	0.7	0.2	0.2	0.0	0.1	0.1	0.4	0.0	0.4
TFAP2E	0.7	0.1	0.3	0.1	0.2	0.1	0.3	0.1	0.2
BIVM-ERCC5	0.3	0.4	0.2	0.0	0.1	0.1	0.5	0.1	0.5
PVRIG	0.7	0.3	0.3	0.0	0.1	0.0	0.3	0.0	0.3
MASP2	0.7	0.2	0.4	0.0	0.1	0.1	0.3	0.0	0.3
LRRCC1	0.5	0.2	0.3	0.0	0.2	0.2	0.3	0.0	0.3
INCA1	0.8	0.2	0.3	0.1	0.1	0.2	0.2	0.0	0.4
LOC644135	0.9	0.3	0.6	0.0	0.1	0.1	0.1	0.0	0.1
MST1R	0.5	0.2	0.3	0.1	0.2	0.2	0.2	0.0	0.2
ALDH8A1	0.6	0.2	0.5	0.0	0.1	0.1	0.4	0.0	0.2
LOC284344	0.7	0.2	0.1	0.0	0.2	0.2	0.4	0.0	0.3
LOC284080	0.6	0.2	0.4	0.0	0.1	0.2	0.3	0.0	0.1
CTNND1	0.8	0.2	0.3	0.0	0.1	0.1	0.3	0.0	0.3
GRIP1	0.4	0.2	0.3	0.0	0.1	0.1	0.5	0.1	0.4
ZCWPW1	0.4	0.2	0.4	0.1	0.1	0.2	0.5	0.0	0.2
MMP25	0.5	0.3	0.3	0.1	0.1	0.1	0.3	0.1	0.3
LRRC46	0.4	0.3	0.5	0.1	0.2	0.1	0.3	0.0	0.1
NEBL	0.8	0.1	0.4	0.0	0.1	0.2	0.3	0.0	0.2
LOC100507599	0.3	0.3	0.3	0.1	0.1	0.1	0.6	0.1	0.3
KCNJ8	0.8	0.1	0.4	0.1	0.1	0.0	0.4	0.0	0.2
CCDC147	0.7	0.2	0.3	0.0	0.1	0.1	0.3	0.0	0.2
BEND6	0.5	0.2	0.3	0.1	0.1	0.2	0.4	0.1	0.2

LOC101928782	0.5	0.2	0.3	0.1	0.1	0.1	0.4	0.1	0.3
LINC00927	0.6	0.2	0.3	0.0	0.0	0.1	0.6	0.0	0.2
NEO1	0.4	0.1	0.2	0.0	0.1	0.1	0.6	0.1	0.5
DNER	0.3	0.1	0.2	0.1	0.3	0.5	0.4	0.1	0.1
HTR1D	0.5	0.1	0.4	0.1	0.0	0.1	0.4	0.1	0.4
LOC101926888	0.7	0.3	0.2	0.1	0.0	0.1	0.4	0.0	0.2
LINC00477	0.6	0.1	0.5	0.0	0.1	0.1	0.4	0.1	0.2
LOC100527964	0.7	0.2	0.4	0.0	0.1	0.1	0.3	0.1	0.2
TM6SF1	0.6	0.2	0.4	0.0	0.2	0.1	0.3	0.0	0.2
MSX2	0.5	0.2	0.2	0.0	0.1	0.2	0.4	0.1	0.2
CENPI	0.4	0.2	0.2	0.0	0.1	0.1	0.6	0.1	0.4
LOC100505635	0.6	0.1	0.2	0.1	0.1	0.1	0.4	0.1	0.4
PCDHGA9	0.8	0.3	0.3	0.0	0.0	0.1	0.3	0.1	0.2
THEMIS2	0.6	0.2	0.4	0.1	0.1	0.1	0.3	0.0	0.4
LOC101927886	0.3	0.1	0.5	0.0	0.2	0.1	0.4	0.2	0.3
LOC101927890	0.7	0.1	0.4	0.1	0.1	0.1	0.4	0.0	0.2
SLFNL1	0.6	0.1	0.2	0.0	0.1	0.1	0.5	0.1	0.3
ZNF563	0.5	0.3	0.4	0.0	0.1	0.2	0.3	0.0	0.3
FAM182B	0.8	0.2	0.3	0.0	0.1	0.1	0.4	0.0	0.2
LOC149134	0.5	0.2	0.4	0.0	0.0	0.2	0.4	0.1	0.3
NPIPB8	0.6	0.2	0.3	0.1	0.2	0.3	0.2	0.0	0.2
LSAMP	0.6	0.1	0.1	0.0	0.2	0.2	0.4	0.1	0.4
CCDC154	0.7	0.2	0.5	0.0	0.0	0.1	0.3	0.0	0.1
ZNF311	0.4	0.2	0.3	0.0	0.2	0.3	0.4	0.0	0.3
TLR8-AS1	0.7	0.1	0.2	0.1	0.1	0.1	0.6	0.0	0.2
LOC101926967	0.6	0.3	0.3	0.1	0.2	0.1	0.3	0.0	0.2
ZNF433	0.5	0.1	0.4	0.0	0.1	0.2	0.4	0.0	0.2
LOC101929549	0.7	0.2	0.2	0.1	0.1	0.1	0.4	0.1	0.3
HSPA6	0.7	0.3	0.4	0.0	0.0	0.1	0.4	0.0	0.1
ZNF607	0.5	0.2	0.3	0.0	0.1	0.1	0.4	0.0	0.3
PGLYRP1	0.5	0.2	0.3	0.1	0.2	0.0	0.3	0.1	0.3
RANBP3L	0.9	0.3	0.5	0.0	0.1	0.1	0.1	0.0	0.0
LOC101927121	0.6	0.1	0.3	0.0	0.1	0.1	0.4	0.0	0.3
ZNF807	0.5	0.1	0.3	0.0	0.2	0.2	0.5	0.0	0.2
LOC100129215	0.7	0.2	0.3	0.0	0.1	0.1	0.3	0.1	0.2
DOK6	1.0	0.1	0.3	0.0	0.1	0.0	0.3	0.0	0.2
JMJD7-PLA2G4B	0.6	0.2	0.2	0.1	0.1	0.2	0.4	0.1	0.2
LOC101927144	0.5	0.3	0.1	0.0	0.1	0.3	0.4	0.0	0.2
CORO1A	0.4	0.1	0.3	0.1	0.2	0.1	0.4	0.1	0.4
FOXB2	0.2	0.4	0.3	0.4	0.1	0.1	0.2	0.2	0.2
LOC283887	0.7	0.1	0.3	0.0	0.1	0.2	0.4	0.0	0.1
LOC100288846	0.7	0.3	0.4	0.0	0.1	0.1	0.3	0.0	0.1
SDK1	0.3	0.1	0.2	0.1	0.2	0.2	0.4	0.1	0.4
LOC101927278	0.4	0.2	0.3	0.0	0.1	0.1	0.5	0.0	0.3
LOC101929488	0.8	0.1	0.4	0.1	0.0	0.1	0.4	0.0	0.2
ZCWPW2	0.8	0.2	0.2	0.1	0.1	0.2	0.2	0.0	0.3
FAM223B	0.6	0.1	0.3	0.0	0.1	0.1	0.3	0.1	0.4
PRICKLE2-AS1	0.7	0.2	0.3	0.0	0.1	0.1	0.3	0.0	0.2
ZNF554	0.5	0.2	0.3	0.0	0.1	0.1	0.5	0.1	0.2
AKR1B15	0.7	0.1	0.2	0.0	0.1	0.0	0.6	0.0	0.2
LOC100128374	0.8	0.1	0.3	0.0	0.1	0.1	0.4	0.0	0.1
TPK1	0.7	0.2	0.3	0.0	0.1	0.1	0.3	0.0	0.2
LOC100507336	0.8	0.1	0.2	0.1	0.1	0.1	0.5	0.0	0.2
PDE6A	0.6	0.1	0.3	0.1	0.1	0.1	0.4	0.0	0.3
LINC00366	0.5	0.1	0.2	0.0	0.0	0.1	0.8	0.0	0.2

PPM1E		0.6	0.3	0.3	0.0	0.1	0.1	0.3	0.0	0.2
GLB1L		0.7	0.2	0.2	0.1	0.2	0.2	0.2	0.0	0.1
VSIG2		0.5	0.2	0.4	0.0	0.2	0.1	0.3	0.0	0.3
LOC101928671		0.8	0.1	0.2	0.2	0.2	0.0	0.3	0.0	0.2
RGPD8		0.6	0.1	0.3	0.0	0.1	0.1	0.5	0.0	0.3
DDR2		0.4	0.2	0.3	0.0	0.1	0.0	0.6	0.1	0.3
FAM65C		0.6	0.2	0.3	0.0	0.1	0.2	0.4	0.1	0.1
KCNN1		0.2	0.3	0.3	0.1	0.2	0.1	0.4	0.1	0.4
STX18-AS1		0.6	0.1	0.3	0.1	0.1	0.1	0.3	0.1	0.3
LOC101928001		0.5	0.1	0.2	0.1	0.2	0.2	0.7	0.0	0.2
SNCAIP		0.6	0.2	0.3	0.0	0.1	0.1	0.3	0.1	0.3
LOC101929705		0.7	0.2	0.2	0.1	0.1	0.1	0.5	0.0	0.2
CAHM		0.5	0.2	0.4	0.1	0.3	0.1	0.2	0.0	0.2
NR1I3		0.3	0.2	0.2	0.1	0.2	0.1	0.4	0.1	0.2
LANCL3		0.4	0.3	0.2	0.0	0.1	0.2	0.3	0.1	0.3
SKOR2		0.1	0.3	0.2	0.4	0.1	0.0	0.3	0.2	0.2
HLA-DPA1		0.5	0.2	0.6	0.1	0.1	0.2	0.1	0.0	0.0
CLDN18		0.6	0.1	0.4	0.1	0.1	0.1	0.3	0.1	0.3
NCRNA00185		0.7	0.1	0.3	0.0	0.1	0.0	0.4	0.0	0.2
YY2		0.5	0.3	0.3	0.1	0.0	0.3	0.3	0.0	0.1
MEG3		0.6	0.1	0.3	0.0	0.1	0.1	0.5	0.0	0.2
LOC286359		0.7	0.2	0.4	0.0	0.1	0.1	0.2	0.0	0.2
MRPL23-AS1		0.5	0.2	0.2	0.0	0.2	0.1	0.5	0.0	0.3
TRIM6-TRIM34		0.5	0.3	0.4	0.0	0.0	0.1	0.3	0.0	0.3
KIF24		0.5	0.1	0.2	0.1	0.1	0.2	0.3	0.1	0.4
PTPRU		0.6	0.2	0.3	0.1	0.1	0.2	0.3	0.1	0.2
NGEF		0.6	0.3	0.4	0.0	0.1	0.2	0.1	0.0	0.3
LOC284412		0.5	0.1	0.3	0.1	0.1	0.1	0.4	0.0	0.2
ASB9		0.6	0.1	0.4	0.0	0.0	0.1	0.3	0.0	0.2
LOC100507557		0.6	0.2	0.3	0.0	0.1	0.1	0.4	0.0	0.3
DHFRL1		0.6	0.3	0.5	0.1	0.1	0.1	0.2	0.0	0.1
	44805.0	0.7	0.2	0.3	0.0	0.1	0.1	0.3	0.0	0.1
HNF4G		0.7	0.3	0.2	0.0	0.1	0.1	0.3	0.0	0.2
ZACN		0.7	0.1	0.3	0.0	0.0	0.1	0.5	0.0	0.2
SLC22A1		0.6	0.3	0.1	0.1	0.1	0.2	0.3	0.0	0.3
ZNF19		0.7	0.2	0.2	0.0	0.0	0.1	0.4	0.0	0.2
C2orf72		0.0	0.4	0.3	0.3	0.2	0.0	0.3	0.1	0.2
SLC3A1		0.5	0.1	0.3	0.0	0.2	0.1	0.3	0.0	0.3
ZNF18		0.5	0.2	0.3	0.0	0.1	0.1	0.4	0.1	0.3
LOC101927031		0.6	0.2	0.3	0.1	0.1	0.2	0.2	0.1	0.1
APOBEC2		0.4	0.1	0.5	0.1	0.2	0.3	0.2	0.1	0.1
ARHGAP30		0.3	0.2	0.1	0.0	0.1	0.1	0.5	0.1	0.3
MSS51		1.0	0.2	0.3	0.0	0.1	0.1	0.2	0.0	0.1
LINC00521		0.7	0.2	0.2	0.0	0.1	0.2	0.4	0.0	0.2
PCDH9		0.5	0.1	0.3	0.0	0.0	0.1	0.4	0.1	0.4
RGS5		0.4	0.2	0.4	0.1	0.1	0.2	0.3	0.1	0.1
LOC101929608		0.6	0.1	0.3	0.1	0.1	0.1	0.5	0.1	0.1
ZNF790		0.7	0.3	0.3	0.0	0.1	0.1	0.3	0.0	0.1
LAT		0.5	0.2	0.3	0.1	0.1	0.2	0.4	0.0	0.3
GRIK4		0.5	0.2	0.2	0.1	0.2	0.2	0.2	0.0	0.2
SMTNL1		0.7	0.2	0.4	0.0	0.2	0.2	0.1	0.0	0.1
FAM86B2		0.4	0.1	0.3	0.1	0.2	0.2	0.4	0.0	0.2
LOC285758		0.7	0.1	0.3	0.0	0.1	0.1	0.4	0.0	0.1
SGOL2		0.6	0.2	0.2	0.0	0.1	0.1	0.3	0.0	0.2
LOC101929444		0.6	0.1	0.2	0.1	0.2	0.1	0.3	0.1	0.3

MYO5B	0.4	0.1	0.2	0.1	0.1	0.1	0.5	0.1	0.4
ZNF582-AS1	0.6	0.2	0.3	0.0	0.1	0.1	0.4	0.0	0.2
FAXDC2	0.5	0.2	0.2	0.0	0.1	0.2	0.3	0.1	0.3
DTNA	0.6	0.3	0.2	0.0	0.1	0.1	0.2	0.1	0.2
PTGIR	0.7	0.1	0.4	0.1	0.0	0.1	0.3	0.1	0.2
LOC101927964	0.7	0.1	0.3	0.0	0.1	0.1	0.4	0.0	0.2
ANKAR	0.6	0.2	0.3	0.0	0.1	0.0	0.3	0.0	0.3
HSD17B6	0.5	0.1	0.2	0.0	0.2	0.2	0.2	0.0	0.3
KCNK12	0.6	0.1	0.4	0.1	0.1	0.0	0.4	0.0	0.2
LOC100132287	0.6	0.1	0.4	0.1	0.1	0.1	0.3	0.0	0.1
ZNF850	0.6	0.2	0.2	0.0	0.1	0.1	0.4	0.0	0.2
FES	0.5	0.2	0.2	0.1	0.2	0.1	0.4	0.0	0.3
TSPEAR-AS1	0.4	0.2	0.2	0.1	0.1	0.2	0.3	0.0	0.3
NAT8L	0.2	0.2	0.4	0.0	0.2	0.1	0.5	0.1	0.4
LOC101927742	0.6	0.1	0.2	0.1	0.0	0.1	0.5	0.1	0.2
ZNF502	0.7	0.1	0.4	0.0	0.1	0.1	0.2	0.0	0.3
PODNL1	0.6	0.3	0.3	0.1	0.1	0.1	0.1	0.1	0.1
CASC5	0.4	0.2	0.2	0.0	0.1	0.2	0.5	0.0	0.3
ZNF132	0.4	0.2	0.3	0.0	0.1	0.2	0.4	0.0	0.2
TSPAN10	0.6	0.1	0.4	0.1	0.1	0.1	0.3	0.1	0.2
LOC101927756	0.7	0.1	0.1	0.0	0.0	0.0	0.4	0.1	0.4
RGS2	0.5	0.2	0.4	0.0	0.1	0.1	0.2	0.1	0.3
C2orf27B	0.5	0.1	0.2	0.0	0.1	0.1	0.5	0.0	0.3
LOC100129473	0.5	0.1	0.3	0.0	0.1	0.0	0.4	0.1	0.3
ZFP30	0.4	0.2	0.3	0.0	0.1	0.1	0.4	0.0	0.3
PROX2	0.7	0.1	0.3	0.0	0.2	0.1	0.4	0.0	0.2
KIF15	0.3	0.1	0.2	0.0	0.1	0.1	0.5	0.1	0.4
SRCIN1	0.4	0.2	0.3	0.1	0.1	0.1	0.4	0.1	0.2
CGN	0.5	0.2	0.2	0.0	0.2	0.1	0.4	0.1	0.3
CECR6	0.4	0.2	0.2	0.1	0.1	0.1	0.3	0.1	0.3
STXBP5-AS1	0.5	0.2	0.3	0.0	0.1	0.1	0.5	0.0	0.2
LOC100506827	0.5	0.2	0.3	0.0	0.1	0.1	0.5	0.0	0.2
MIOX	0.2	0.2	0.2	0.0	0.0	0.2	0.5	0.1	0.5
LOC101927372	0.3	0.2	0.3	0.1	0.1	0.2	0.4	0.0	0.4
PROZ	0.6	0.3	0.2	0.0	0.1	0.1	0.5	0.0	0.1
LOC285762	0.8	0.1	0.2	0.0	0.1	0.1	0.4	0.0	0.1
NRL	0.6	0.1	0.2	0.1	0.1	0.2	0.4	0.0	0.1
LOC101929330	0.6	0.2	0.2	0.1	0.1	0.1	0.5	0.0	0.2
BRCA2	0.5	0.2	0.2	0.0	0.1	0.1	0.4	0.0	0.3
ITGAE	0.5	0.2	0.3	0.0	0.1	0.1	0.4	0.0	0.2
TLR3	0.6	0.2	0.3	0.0	0.0	0.1	0.4	0.1	0.3
LOC339874	0.5	0.1	0.2	0.0	0.2	0.1	0.4	0.0	0.3
FLJ25917	0.6	0.0	0.2	0.1	0.0	0.0	0.6	0.2	0.2
LOC101928556	0.9	0.1	0.2	0.2	0.2	0.0	0.3	0.0	0.0
PSD	0.6	0.3	0.3	0.0	0.1	0.2	0.2	0.0	0.1
NLRP3	0.4	0.1	0.1	0.1	0.1	0.1	0.5	0.1	0.5
LOC101929561	0.3	0.2	0.3	0.0	0.1	0.2	0.3	0.0	0.4
LINC00971	0.6	0.2	0.2	0.0	0.1	0.1	0.5	0.0	0.3
MYO7B	0.3	0.1	0.2	0.1	0.2	0.3	0.4	0.0	0.2
PSG4	0.5	0.1	0.2	0.1	0.1	0.1	0.4	0.0	0.3
CDH13	0.4	0.1	0.4	0.1	0.0	0.1	0.3	0.0	0.4
LOC101927682	0.5	0.1	0.3	0.1	0.2	0.2	0.2	0.1	0.3
LOC101929244	0.8	0.1	0.2	0.0	0.1	0.1	0.3	0.0	0.1
LINC00950	0.6	0.1	0.3	0.0	0.1	0.1	0.4	0.0	0.1
LOC286370	0.6	0.1	0.3	0.1	0.1	0.1	0.3	0.0	0.2

WISP2	0.3	0.1	0.4	0.1	0.2	0.4	0.3	0.0	0.1
LOC101929051	0.6	0.3	0.2	0.0	0.2	0.1	0.2	0.0	0.1
LOC158435	0.6	0.2	0.3	0.0	0.1	0.1	0.3	0.0	0.3
FAM111B	0.3	0.1	0.2	0.0	0.1	0.2	0.4	0.0	0.4
PCDHGA7	0.8	0.1	0.3	0.0	0.1	0.1	0.2	0.0	0.2
NHLRC1	0.4	0.2	0.3	0.1	0.2	0.1	0.3	0.0	0.2
FAM72B	0.2	0.1	0.1	0.0	0.1	0.2	0.4	0.0	0.7
YPEL4	0.6	0.1	0.4	0.1	0.1	0.2	0.0	0.1	0.2
GPR137C	0.4	0.2	0.1	0.1	0.1	0.1	0.5	0.0	0.3
LOC100996511	0.7	0.1	0.2	0.0	0.1	0.1	0.3	0.0	0.2
LINC00692	0.7	0.0	0.3	0.1	0.2	0.1	0.2	0.0	0.1
RIMS2	0.2	0.1	0.1	0.0	0.1	0.1	1.0	0.0	0.3
COL7A1	0.4	0.2	0.2	0.1	0.2	0.2	0.4	0.0	0.2
TTC30B	0.8	0.1	0.3	0.0	0.1	0.1	0.2	0.0	0.2
C1orf51	0.4	0.4	0.2	0.0	0.0	0.1	0.3	0.1	0.3
LOC101929569	0.6	0.2	0.3	0.0	0.1	0.1	0.3	0.0	0.1
C9orf47	0.4	0.2	0.3	0.1	0.1	0.2	0.4	0.0	0.2
GRTP1	0.7	0.3	0.1	0.1	0.0	0.2	0.2	0.0	0.3
DSEL	0.5	0.2	0.2	0.0	0.1	0.1	0.3	0.0	0.3
FAM72A	0.2	0.0	0.0	0.0	0.1	0.2	0.7	0.1	0.6
CCDC129	0.5	0.1	0.2	0.1	0.1	0.1	0.4	0.1	0.2
LOC101927142	0.7	0.2	0.3	0.1	0.1	0.1	0.2	0.0	0.1
CARD14	0.6	0.2	0.4	0.0	0.1	0.1	0.1	0.0	0.1
SCN1B	0.7	0.4	0.1	0.0	0.1	0.1	0.2	0.0	0.0
LOC100132078	0.7	0.1	0.2	0.0	0.1	0.1	0.4	0.0	0.2
LOC100996738	0.3	0.1	0.5	0.0	0.1	0.0	0.5	0.0	0.2
ITIH4	0.6	0.3	0.4	0.1	0.1	0.1	0.1	0.0	0.1
ZNF100	0.4	0.1	0.3	0.0	0.1	0.1	0.3	0.0	0.3
PARD6G-AS1	0.4	0.2	0.5	0.1	0.1	0.1	0.3	0.0	0.1
FAM212B	0.3	0.2	0.4	0.0	0.1	0.0	0.4	0.1	0.3
LOC101928978	0.5	0.1	0.3	0.0	0.1	0.1	0.3	0.1	0.2
LOC101929072	0.5	0.2	0.3	0.1	0.1	0.2	0.2	0.1	0.3
LOC101929158	0.5	0.1	0.3	0.0	0.1	0.1	0.3	0.1	0.2
INHBA	0.5	0.1	0.2	0.0	0.1	0.2	0.3	0.0	0.2
HERC6	0.5	0.2	0.3	0.0	0.1	0.1	0.3	0.1	0.2
PDE4B	0.6	0.1	0.4	0.0	0.1	0.1	0.2	0.0	0.3
DMBT1	0.6	0.3	0.3	0.0	0.1	0.1	0.1	0.0	0.1
GAREM	0.5	0.1	0.2	0.1	0.1	0.1	0.3	0.1	0.4
GOLGA8K	0.9	0.2	0.1	0.0	0.0	0.2	0.1	0.0	0.2
LINC00520	0.5	0.2	0.2	0.0	0.1	0.1	0.3	0.1	0.3
KIAA1377	0.6	0.2	0.3	0.0	0.1	0.1	0.3	0.0	0.2
DEPDC7	0.4	0.2	0.3	0.0	0.1	0.1	0.3	0.1	0.3
RNASEL	0.6	0.2	0.2	0.0	0.1	0.0	0.3	0.0	0.2
OGFR-AS1	0.4	0.2	0.3	0.1	0.1	0.1	0.2	0.1	0.2
LOC101927393	0.5	0.2	0.2	0.0	0.1	0.2	0.3	0.1	0.3
C6orf163	0.6	0.1	0.2	0.1	0.1	0.1	0.3	0.1	0.3
LOC101928552	0.4	0.1	0.3	0.0	0.0	0.2	0.5	0.0	0.2
LOC100996635	0.8	0.1	0.2	0.0	0.0	0.1	0.3	0.0	0.2
HMGN3-AS1	0.4	0.3	0.3	0.1	0.0	0.1	0.3	0.0	0.2
CLEC18A	0.7	0.1	0.4	0.0	0.1	0.1	0.2	0.0	0.1
LEF1-AS1	0.5	0.1	0.2	0.0	0.1	0.2	0.5	0.0	0.1
UBXN7-AS1	0.6	0.1	0.3	0.1	0.0	0.1	0.3	0.1	0.2
LOC100505501	0.5	0.3	0.5	0.0	0.0	0.1	0.1	0.0	0.1
LOC101928008	0.4	0.2	0.3	0.0	0.0	0.1	0.5	0.0	0.1
FLVCR2	0.6	0.4	0.2	0.0	0.1	0.2	0.1	0.0	0.1

ANXA9	0.6	0.2	0.2	0.0	0.1	0.1	0.2	0.0	0.2
LOC101928841	0.2	0.1	0.2	0.0	0.1	0.2	0.4	0.1	0.4
LOC100287320	0.5	0.0	0.3	0.1	0.1	0.1	0.5	0.0	0.1
SSC5D	0.5	0.1	0.2	0.1	0.1	0.2	0.3	0.0	0.3
STC2	0.2	0.1	0.3	0.1	0.2	0.2	0.4	0.1	0.2
THAP2	0.8	0.2	0.2	0.0	0.1	0.0	0.1	0.0	0.2
LOC105379426	0.5	0.1	0.2	0.0	0.1	0.1	0.4	0.0	0.3
SH2D2A	0.3	0.1	0.2	0.1	0.1	0.1	0.4	0.1	0.3
DSC2	0.5	0.3	0.3	0.0	0.1	0.1	0.3	0.1	0.2
LOC100996402	0.6	0.2	0.2	0.0	0.1	0.2	0.2	0.0	0.2
SEMA6A	0.6	0.2	0.2	0.0	0.0	0.1	0.4	0.0	0.2
SLC52A3	0.6	0.1	0.3	0.0	0.1	0.2	0.2	0.0	0.2
POU5F1	0.5	0.1	0.2	0.1	0.1	0.1	0.4	0.0	0.2
NCR3LG1	0.4	0.2	0.3	0.0	0.1	0.1	0.3	0.1	0.3
TNFSF12-TNFSF13	0.5	0.2	0.4	0.0	0.0	0.1	0.1	0.0	0.3
UCA1	0.6	0.2	0.2	0.1	0.1	0.1	0.3	0.0	0.2
TUBA3D	0.5	0.2	0.3	0.0	0.1	0.1	0.2	0.1	0.0
MYH15	0.6	0.2	0.3	0.0	0.1	0.1	0.4	0.0	0.2
LOC101927572	0.3	0.1	0.2	0.1	0.1	0.0	0.7	0.0	0.2
LOC101929045	0.4	0.1	0.3	0.0	0.1	0.1	0.2	0.1	0.3
LOC100630918	0.5	0.2	0.3	0.0	0.1	0.1	0.2	0.0	0.2
ZNF813	0.2	0.3	0.3	0.0	0.1	0.1	0.3	0.1	0.3
NTF4	0.6	0.2	0.2	0.1	0.1	0.2	0.1	0.1	0.1
NYAP2	0.5	0.1	0.2	0.0	0.1	0.1	0.3	0.1	0.3
LOC101927576	0.5	0.1	0.2	0.0	0.1	0.2	0.4	0.1	0.1
ASB12	0.6	0.1	0.3	0.0	0.1	0.0	0.1	0.1	0.3
ST8SIA4	0.4	0.1	0.3	0.0	0.1	0.2	0.2	0.0	0.2
PCDHB7	0.7	0.2	0.4	0.1	0.1	0.1	0.2	0.0	0.1
LNX1-AS2	0.6	0.1	0.3	0.1	0.1	0.1	0.3	0.0	0.2
CTSW	0.4	0.3	0.3	0.0	0.2	0.2	0.2	0.0	0.2
DNM3	0.4	0.1	0.3	0.1	0.1	0.1	0.3	0.1	0.2
HIST3H2BB	0.4	0.2	0.4	0.1	0.0	0.2	0.4	0.1	0.0
LOC101929079	0.4	0.1	0.2	0.0	0.2	0.1	0.4	0.0	0.3
TLE2	0.3	0.1	0.2	0.1	0.1	0.1	0.3	0.1	0.4
PTPRB	0.6	0.1	0.3	0.0	0.0	0.0	0.3	0.1	0.2
FAM20A	0.7	0.2	0.3	0.0	0.1	0.0	0.2	0.0	0.1
HUS1B	0.4	0.1	0.4	0.1	0.2	0.0	0.4	0.0	0.1
HEXA-AS1	0.6	0.1	0.3	0.0	0.1	0.1	0.3	0.1	0.2
VTN	0.7	0.1	0.3	0.0	0.0	0.1	0.3	0.0	0.1
MIR31HG	0.7	0.1	0.2	0.0	0.1	0.0	0.4	0.0	0.1
SMC5-AS1	0.5	0.1	0.1	0.0	0.2	0.0	0.2	0.0	0.4
BEST1	0.5	0.2	0.3	0.1	0.1	0.0	0.3	0.1	0.1
ZBTB20	0.6	0.1	0.2	0.1	0.1	0.1	0.1	0.1	0.3
DERL3	0.3	0.3	0.2	0.0	0.0	0.0	0.4	0.1	0.3
CST7	0.5	0.2	0.1	0.1	0.0	0.1	0.2	0.1	0.4
C9orf117	0.3	0.2	0.4	0.1	0.1	0.2	0.2	0.0	0.1
LOC100505716	0.5	0.2	0.3	0.0	0.0	0.1	0.2	0.0	0.2
SERPINA1	0.4	0.2	0.2	0.0	0.2	0.4	0.1	0.1	0.1
MUM1L1	0.7	0.2	0.3	0.0	0.0	0.0	0.2	0.0	0.1
NTN3	0.4	0.2	0.2	0.1	0.1	0.1	0.4	0.1	0.1
ZC2HC1C	0.4	0.2	0.2	0.0	0.1	0.1	0.4	0.1	0.1
LOC100132215	0.4	0.2	0.3	0.0	0.1	0.1	0.3	0.1	0.2
MISP	0.4	0.2	0.2	0.0	0.1	0.1	0.3	0.0	0.2
TTC18	0.6	0.2	0.3	0.0	0.1	0.1	0.3	0.0	0.1
LOC101927553	0.5	0.0	0.5	0.1	0.1	0.0	0.2	0.0	0.2

LOC101928376	0.6	0.1	0.3	0.0	0.1	0.1	0.3	0.0	0.2
PCDHGA8	0.7	0.1	0.3	0.1	0.0	0.1	0.2	0.0	0.1
DDX60	0.5	0.1	0.3	0.0	0.1	0.1	0.3	0.0	0.2
ZNF708	0.3	0.2	0.2	0.0	0.1	0.1	0.4	0.0	0.3
EML5	0.6	0.1	0.3	0.0	0.1	0.1	0.2	0.0	0.2
RCOR2	0.6	0.3	0.2	0.1	0.0	0.1	0.2	0.0	0.2
CHGA	0.3	0.1	0.3	0.0	0.1	0.1	0.3	0.0	0.3
LOC100128361	0.4	0.2	0.3	0.1	0.1	0.1	0.3	0.0	0.1
MARK1	0.4	0.1	0.1	0.0	0.1	0.1	0.3	0.1	0.4
FAM218A	0.4	0.2	0.5	0.0	0.1	0.1	0.2	0.1	0.1
MTMR8	0.4	0.1	0.2	0.0	0.1	0.1	0.4	0.0	0.2
LOC90246	0.6	0.2	0.2	0.0	0.0	0.0	0.2	0.0	0.3
TEX9	0.5	0.2	0.3	0.1	0.1	0.1	0.3	0.0	0.1
LOC100288181	0.6	0.1	0.2	0.0	0.1	0.1	0.2	0.0	0.2
FOXN3-AS1	0.4	0.2	0.2	0.1	0.1	0.1	0.4	0.1	0.2
NUTM2G	0.4	0.1	0.2	0.0	0.1	0.1	0.4	0.0	0.2
LSMEM2	0.6	0.3	0.1	0.0	0.0	0.2	0.2	0.0	0.2
LOC101929516	0.4	0.2	0.1	0.0	0.1	0.0	0.3	0.1	0.4
LOC100653021	0.4	0.2	0.3	0.0	0.1	0.2	0.2	0.1	0.2
LOC101929523	0.5	0.2	0.2	0.1	0.0	0.1	0.1	0.1	0.2
TXNRD3NB	0.2	0.1	0.3	0.0	0.1	0.1	0.4	0.1	0.4
C9orf173	0.5	0.1	0.3	0.1	0.1	0.0	0.1	0.0	0.3
SLC1A1	0.6	0.3	0.2	0.1	0.1	0.1	0.2	0.0	0.1
STARD13-AS	0.4	0.1	0.2	0.0	0.1	0.1	0.3	0.0	0.3
C8orf46	0.4	0.1	0.3	0.1	0.1	0.1	0.3	0.0	0.1
LOC101928000	0.1	0.1	0.2	0.1	0.1	0.1	0.5	0.0	0.3
ANGPT4	0.3	0.1	0.2	0.0	0.1	0.0	0.5	0.0	0.2
ZNF81	0.3	0.2	0.3	0.0	0.1	0.1	0.3	0.0	0.2
EFCAB5	0.5	0.1	0.2	0.0	0.0	0.1	0.4	0.0	0.2
ARHGAP19	0.0	0.5	0.4	0.0	0.2	0.2	0.0	0.1	0.0
LOC100130097	0.5	0.1	0.3	0.0	0.1	0.1	0.3	0.0	0.1
HCG16	0.4	0.2	0.2	0.1	0.0	0.2	0.2	0.0	0.3
GRIN2D	0.5	0.3	0.3	0.0	0.1	0.1	0.1	0.0	0.1
FTCDNL1	0.4	0.1	0.2	0.0	0.2	0.2	0.2	0.0	0.2
PDGFRB	0.4	0.2	0.2	0.0	0.0	0.1	0.4	0.0	0.2
ARHGAP19-SLIT1	0.6	0.1	0.1	0.0	0.0	0.1	0.3	0.0	0.3
LOC100506281	0.6	0.2	0.3	0.0	0.1	0.1	0.1	0.0	0.1
ESRP2	0.4	0.1	0.2	0.1	0.1	0.1	0.3	0.1	0.2
ITPKA	0.4	0.1	0.2	0.0	0.0	0.1	0.4	0.1	0.2
MMP13	0.6	0.0	0.3	0.1	0.0	0.0	0.3	0.0	0.2
LINC00893	0.4	0.2	0.4	0.0	0.1	0.2	0.2	0.0	0.1
MAGEA4	0.3	0.3	0.2	0.0	0.1	0.1	0.3	0.1	0.2
ZSCAN31	0.6	0.2	0.2	0.0	0.1	0.2	0.1	0.0	0.1
BACH2	0.3	0.1	0.1	0.0	0.2	0.2	0.3	0.1	0.3
LOC101929416	0.5	0.1	0.2	0.0	0.1	0.0	0.4	0.0	0.2
C4orf21	0.5	0.1	0.2	0.0	0.0	0.1	0.4	0.0	0.2
ZRANB3	0.4	0.1	0.2	0.0	0.1	0.1	0.3	0.0	0.2
SLC5A4	0.6	0.1	0.3	0.0	0.0	0.2	0.1	0.0	0.1
LOC339988	0.5	0.1	0.3	0.0	0.0	0.1	0.4	0.0	0.1
LOC101928004	0.6	0.2	0.2	0.1	0.0	0.1	0.3	0.0	0.1
PCP2	0.5	0.4	0.5	0.0	0.1	0.1	0.0	0.0	0.0
ARHGEF6	0.6	0.2	0.1	0.0	0.1	0.1	0.2	0.0	0.2
LOC101929243	0.3	0.4	0.1	0.1	0.0	0.2	0.3	0.1	0.1
CACHD1	0.4	0.2	0.2	0.0	0.1	0.2	0.3	0.0	0.2
CYorf17	0.6	0.2	0.3	0.0	0.0	0.0	0.2	0.1	0.1

LOC100130876	0.5	0.1	0.2	0.0	0.1	0.1	0.3	0.1	0.2
GAL3ST4	0.6	0.1	0.2	0.0	0.1	0.1	0.2	0.0	0.1
SRRM5	0.5	0.2	0.2	0.0	0.0	0.1	0.2	0.1	0.2
GLP2R	0.6	0.2	0.0	0.1	0.1	0.1	0.2	0.0	0.2
LYPD3	0.7	0.1	0.2	0.0	0.1	0.1	0.2	0.1	0.1
ADD3-AS1	0.4	0.2	0.3	0.1	0.0	0.2	0.2	0.0	0.1
ZNF568	0.7	0.1	0.3	0.0	0.0	0.1	0.1	0.0	0.2
LOC100287175	0.5	0.1	0.5	0.0	0.2	0.1	0.1	0.1	0.1
KIF9	0.3	0.2	0.3	0.1	0.1	0.1	0.2	0.0	0.2
COBLL1	0.4	0.1	0.2	0.0	0.1	0.1	0.3	0.0	0.3
LOC101927498	0.5	0.1	0.2	0.0	0.0	0.0	0.3	0.1	0.2
POTEE	0.4	0.1	0.2	0.1	0.1	0.2	0.2	0.1	0.2
LOC101929356	0.5	0.2	0.1	0.0	0.2	0.0	0.3	0.0	0.2
LINC00565	0.5	0.1	0.2	0.0	0.1	0.0	0.4	0.0	0.2
TRIM67	0.1	0.3	0.2	0.2	0.0	0.0	0.4	0.2	0.1
TLR6	0.5	0.2	0.2	0.0	0.1	0.1	0.3	0.0	0.2
FITM1	0.5	0.1	0.2	0.0	0.1	0.1	0.2	0.0	0.2
LOC646903	0.4	0.2	0.3	0.0	0.1	0.1	0.2	0.0	0.2
CENPE	0.3	0.1	0.2	0.0	0.1	0.1	0.4	0.1	0.3
LOC101927300	0.3	0.2	0.3	0.0	0.2	0.2	0.2	0.0	0.1
FBXO43	0.3	0.1	0.2	0.1	0.2	0.2	0.3	0.0	0.2
GAGE1	0.6	0.1	0.2	0.0	0.0	0.0	0.3	0.0	0.1
ALG10B	0.4	0.2	0.2	0.0	0.0	0.2	0.2	0.0	0.3
UTY	0.5	0.1	0.2	0.1	0.0	0.1	0.4	0.0	0.2
PLIN4	0.5	0.2	0.2	0.1	0.1	0.1	0.1	0.0	0.1
LOC100499194	0.5	0.1	0.2	0.0	0.1	0.0	0.3	0.0	0.3
LOC101928012	0.6	0.0	0.2	0.0	0.1	0.1	0.3	0.0	0.1
ETV2	0.4	0.2	0.2	0.1	0.0	0.2	0.1	0.0	0.2
ZNF493	0.6	0.2	0.3	0.0	0.2	0.1	0.1	0.0	0.1
LOC101926957	0.5	0.1	0.2	0.0	0.1	0.1	0.3	0.0	0.2
LOC101927069	0.4	0.1	0.2	0.0	0.0	0.1	0.4	0.0	0.2
RALGPS1	0.4	0.1	0.3	0.0	0.1	0.1	0.2	0.0	0.2
LOC101927645	0.4	0.1	0.3	0.0	0.0	0.1	0.3	0.1	0.2
FOCAD	0.4	0.1	0.3	0.0	0.0	0.1	0.4	0.0	0.2
HAO1	0.3	0.0	0.2	0.1	0.0	0.0	0.5	0.1	0.2
SYT16	0.5	0.1	0.2	0.1	0.0	0.1	0.3	0.0	0.2
EHMT1-IT1	0.6	0.1	0.2	0.0	0.1	0.1	0.3	0.0	0.2
DOCK11	0.5	0.2	0.2	0.0	0.0	0.1	0.3	0.1	0.2
RIPK4	0.5	0.2	0.2	0.1	0.1	0.1	0.2	0.0	0.1
LOC101928753	0.4	0.2	0.2	0.1	0.1	0.2	0.2	0.0	0.1
OTOP1	0.6	0.1	0.2	0.0	0.0	0.0	0.3	0.1	0.2
KDM4D	0.5	0.2	0.1	0.0	0.0	0.1	0.3	0.1	0.2
IL15	0.5	0.1	0.3	0.0	0.1	0.1	0.2	0.0	0.2
LOC100130698	0.5	0.1	0.2	0.0	0.1	0.1	0.4	0.0	0.1
LOC101927815	0.5	0.1	0.2	0.0	0.1	0.1	0.3	0.0	0.2
GABRB1	0.6	0.2	0.1	0.0	0.0	0.0	0.2	0.1	0.1
LOC101059906	0.3	0.2	0.2	0.1	0.1	0.1	0.3	0.0	0.2
DKFZP434I0714	0.3	0.2	0.2	0.1	0.0	0.1	0.3	0.0	0.3
MORN3	0.4	0.1	0.2	0.1	0.1	0.1	0.2	0.0	0.2
FOXD4L5	0.4	0.2	0.4	0.1	0.0	0.1	0.2	0.0	0.1
PCDH1	0.4	0.2	0.1	0.0	0.0	0.0	0.4	0.0	0.3
TTC21A	0.5	0.2	0.3	0.1	0.1	0.1	0.2	0.0	0.1
PDK4	0.6	0.1	0.3	0.0	0.0	0.1	0.3	0.0	0.1
CDSN	0.4	0.0	0.1	0.1	0.1	0.2	0.5	0.1	0.1
MUC6	0.5	0.1	0.3	0.0	0.1	0.1	0.3	0.0	0.2

LIPE-AS1	0.5	0.1	0.2	0.0	0.1	0.1	0.2	0.0	0.1
C1orf213	0.5	0.2	0.3	0.0	0.1	0.1	0.1	0.0	0.2
DGUOK-AS1	0.5	0.1	0.2	0.1	0.0	0.1	0.4	0.0	0.1
FBXO6	0.6	0.1	0.3	0.0	0.0	0.1	0.2	0.0	0.2
SERINC4	0.5	0.1	0.3	0.0	0.1	0.2	0.1	0.0	0.1
FANCB	0.3	0.1	0.1	0.0	0.1	0.1	0.5	0.0	0.2
SDS	0.6	0.2	0.2	0.0	0.0	0.0	0.2	0.0	0.1
NMU	0.2	0.1	0.2	0.0	0.2	0.1	0.3	0.1	0.2
LRRTM2	0.6	0.0	0.2	0.0	0.1	0.1	0.3	0.0	0.1
PIEZO2	0.5	0.2	0.2	0.0	0.1	0.2	0.1	0.0	0.1
ZSCAN20	0.4	0.1	0.2	0.0	0.0	0.1	0.3	0.0	0.2
FAM184B	0.5	0.1	0.2	0.0	0.1	0.1	0.3	0.0	0.1
LOC101928756	0.5	0.2	0.3	0.0	0.0	0.0	0.2	0.0	0.2
LOC101928673	0.4	0.1	0.2	0.0	0.1	0.1	0.3	0.1	0.2
EDNRA	0.3	0.1	0.3	0.0	0.1	0.1	0.3	0.0	0.2
CDH3	0.4	0.2	0.2	0.0	0.1	0.1	0.2	0.0	0.1
GARNL3	0.5	0.2	0.2	0.0	0.1	0.1	0.1	0.0	0.2
LOC100506286	0.6	0.1	0.1	0.0	0.0	0.1	0.3	0.0	0.1
MAP2	0.5	0.1	0.2	0.0	0.2	0.1	0.2	0.0	0.1
NAT16	0.8	0.1	0.2	0.0	0.1	0.0	0.1	0.1	0.0
HTR5A-AS1	0.4	0.1	0.3	0.0	0.1	0.0	0.3	0.0	0.2
SPATA41	0.4	0.2	0.2	0.0	0.1	0.1	0.2	0.0	0.2
ERBB3	0.4	0.1	0.2	0.0	0.1	0.1	0.3	0.1	0.2
LINC00899	0.4	0.1	0.3	0.1	0.1	0.2	0.1	0.0	0.1
LOC101928172	0.5	0.1	0.2	0.0	0.0	0.1	0.2	0.0	0.1
PRR26	0.5	0.1	0.2	0.0	0.1	0.1	0.3	0.1	0.1
LOC101929609	0.2	0.1	0.4	0.0	0.1	0.1	0.2	0.0	0.4
PODXL2	0.4	0.2	0.3	0.0	0.1	0.1	0.2	0.0	0.2
ARID3C	0.4	0.2	0.2	0.0	0.0	0.1	0.2	0.0	0.3
LOC101929624	0.7	0.1	0.1	0.0	0.0	0.1	0.4	0.0	0.1
LOC285626	0.6	0.1	0.2	0.0	0.1	0.1	0.2	0.0	0.1
LOC101928500	0.3	0.0	0.1	0.0	0.2	0.1	0.5	0.0	0.1
PRDM6	0.4	0.1	0.3	0.0	0.1	0.1	0.3	0.0	0.2
ZFP69	0.5	0.1	0.2	0.0	0.0	0.0	0.3	0.0	0.3
LOC442028	0.5	0.1	0.3	0.1	0.1	0.1	0.2	0.0	0.1
ABHD1	0.4	0.1	0.2	0.0	0.0	0.1	0.3	0.0	0.2
CHADL	0.5	0.1	0.2	0.0	0.1	0.0	0.3	0.0	0.3
PPIL6	0.4	0.2	0.3	0.0	0.1	0.1	0.2	0.0	0.1
TET1	0.5	0.2	0.2	0.0	0.1	0.1	0.2	0.0	0.2
NPR1	0.5	0.2	0.2	0.0	0.1	0.1	0.2	0.0	0.2
ZNF354C	0.4	0.3	0.2	0.0	0.0	0.0	0.3	0.1	0.1
LOC100996619	0.5	0.1	0.2	0.0	0.2	0.1	0.3	0.0	0.0
PRAM1	0.5	0.2	0.3	0.1	0.1	0.0	0.1	0.0	0.0
HECW2	0.2	0.1	0.1	0.0	0.1	0.1	0.3	0.1	0.3
PRICKLE2	0.4	0.2	0.3	0.1	0.1	0.1	0.1	0.1	0.1
PIF1	0.3	0.2	0.2	0.1	0.1	0.1	0.2	0.1	0.2
LOC79015	0.6	0.1	0.2	0.0	0.0	0.0	0.2	0.0	0.1
ZNF429	0.4	0.0	0.2	0.1	0.0	0.1	0.4	0.0	0.2
PCDHA11	0.5	0.1	0.3	0.0	0.1	0.0	0.2	0.0	0.1
C1orf145	0.5	0.1	0.1	0.1	0.0	0.1	0.3	0.0	0.2
MTCP1	0.2	0.0	0.2	0.1	0.0	0.2	0.4	0.2	0.1
DCTN1-AS1	0.4	0.1	0.1	0.1	0.1	0.1	0.4	0.0	0.1
DOCK4-AS1	0.5	0.1	0.3	0.0	0.1	0.1	0.2	0.0	0.1
OR2A1	0.4	0.1	0.2	0.1	0.2	0.2	0.2	0.0	0.2
SALL2	0.4	0.1	0.2	0.0	0.0	0.2	0.3	0.0	0.2

NEK10	0.7	0.2	0.2	0.0	0.0	0.0	0.2	0.0	0.1
ZNF385C	0.3	0.2	0.2	0.1	0.0	0.2	0.2	0.1	0.2
MSH4	0.5	0.1	0.2	0.0	0.1	0.1	0.2	0.0	0.2
MBLAC1	0.4	0.2	0.2	0.1	0.1	0.1	0.2	0.0	0.1
GRIN2C	0.3	0.2	0.3	0.0	0.1	0.1	0.3	0.0	0.1
LOC100506496	0.4	0.1	0.3	0.0	0.1	0.1	0.2	0.0	0.2
GOLGA8H	0.6	0.1	0.3	0.0	0.0	0.1	0.1	0.0	0.1
SPC25	0.1	0.0	0.1	0.0	0.2	0.2	0.4	0.1	0.2
FLJ44511	0.5	0.1	0.2	0.1	0.1	0.1	0.3	0.0	0.2
GPR146	0.4	0.1	0.2	0.1	0.1	0.1	0.3	0.1	0.2
LOC101929513	0.4	0.1	0.2	0.0	0.2	0.1	0.3	0.1	0.2
FAM27C	0.8	0.0	0.0	0.1	0.0	0.0	0.5	0.0	0.0
TMEM51-AS1	0.4	0.1	0.2	0.0	0.1	0.0	0.4	0.0	0.1
EDNRB	0.3	0.1	0.1	0.0	0.1	0.1	0.4	0.1	0.3
SCG2	0.6	0.1	0.2	0.0	0.1	0.2	0.1	0.1	0.1
ADAM11	0.3	0.2	0.2	0.1	0.0	0.2	0.3	0.0	0.1
STARD4-AS1	0.4	0.1	0.2	0.0	0.1	0.1	0.2	0.0	0.1
LOC101928334	0.4	0.1	0.2	0.0	0.0	0.1	0.3	0.0	0.2
POLQ	0.3	0.1	0.2	0.0	0.1	0.1	0.3	0.0	0.2
NCAM2	0.5	0.2	0.2	0.0	0.0	0.0	0.2	0.0	0.2
ZNF519	0.3	0.2	0.2	0.0	0.0	0.1	0.3	0.0	0.3
PUS10	0.4	0.1	0.1	0.0	0.1	0.1	0.2	0.0	0.3
VSIG1	0.5	0.3	0.1	0.0	0.0	0.0	0.3	0.1	0.1
PTGES3L	0.2	0.2	0.2	0.1	0.0	0.1	0.3	0.1	0.2
BAI2	0.3	0.1	0.1	0.0	0.1	0.1	0.4	0.0	0.3
CADM2-AS1	0.5	0.1	0.2	0.0	0.0	0.0	0.3	0.0	0.1
DNAH5	0.5	0.1	0.3	0.0	0.1	0.1	0.2	0.0	0.1
UBOX5-AS1	0.4	0.1	0.2	0.0	0.0	0.0	0.3	0.0	0.2
SOX10	0.5	0.0	0.3	0.0	0.1	0.1	0.2	0.0	0.2
LOC101929029	0.4	0.2	0.3	0.0	0.1	0.1	0.1	0.0	0.2
PSMG3-AS1	0.3	0.1	0.2	0.1	0.2	0.2	0.1	0.0	0.1
GDAP1L1	0.4	0.0	0.1	0.0	0.0	0.1	0.3	0.1	0.3
PCDHGA3	0.5	0.2	0.2	0.0	0.0	0.1	0.1	0.0	0.1
C14orf37	0.6	0.2	0.2	0.0	0.0	0.1	0.2	0.0	0.2
MAGEA11	0.2	0.1	0.2	0.0	0.2	0.1	0.1	0.0	0.4
CYBB	0.3	0.2	0.2	0.0	0.0	0.1	0.3	0.0	0.1
LOC101929194	0.5	0.1	0.2	0.0	0.1	0.0	0.2	0.1	0.1
LOC100507054	0.3	0.2	0.3	0.0	0.1	0.1	0.2	0.1	0.2
CSNK1G2-AS1	0.4	0.1	0.2	0.0	0.0	0.1	0.3	0.1	0.2
AKAP5	0.5	0.1	0.1	0.0	0.1	0.1	0.3	0.0	0.2
LOC101928419	0.3	0.1	0.2	0.1	0.1	0.1	0.2	0.0	0.3
MKNK1-AS1	0.5	0.1	0.2	0.0	0.0	0.0	0.4	0.0	0.1
C5orf34	0.4	0.1	0.1	0.0	0.1	0.2	0.2	0.0	0.3
SEMA5A	0.5	0.2	0.1	0.1	0.1	0.1	0.2	0.0	0.2
NPTN-IT1	0.4	0.1	0.3	0.0	0.1	0.1	0.3	0.0	0.1
PLA2G4B	0.5	0.2	0.1	0.0	0.1	0.1	0.2	0.0	0.1
SYT12	0.5	0.2	0.3	0.0	0.0	0.0	0.1	0.0	0.1
IQCH	0.3	0.2	0.2	0.0	0.1	0.1	0.3	0.0	0.1
NLGN1	0.3	0.2	0.1	0.0	0.2	0.2	0.1	0.0	0.1
CFLAR-AS1	0.6	0.1	0.2	0.0	0.1	0.1	0.2	0.0	0.2
ICAM4	0.7	0.1	0.2	0.0	0.0	0.1	0.1	0.0	0.1
TBC1D32	0.5	0.1	0.3	0.0	0.0	0.1	0.2	0.0	0.1
BST1	0.3	0.1	0.2	0.0	0.1	0.1	0.3	0.1	0.2
CSPG5	0.5	0.1	0.3	0.0	0.1	0.0	0.1	0.0	0.1
ANKRD53	0.3	0.2	0.2	0.0	0.2	0.2	0.2	0.0	0.1

CA14	0.4	0.2	0.2	0.0	0.0	0.1	0.1	0.0	0.2
FAM9B	0.1	0.2	0.2	0.0	0.1	0.3	0.2	0.0	0.1
DRGX	0.2	0.1	0.1	0.1	0.1	0.1	0.2	0.1	0.3
ADAMTS9-AS2	0.4	0.1	0.1	0.1	0.1	0.1	0.3	0.0	0.1
LOC101927215	0.4	0.1	0.2	0.0	0.1	0.1	0.3	0.0	0.2
LOC101929482	0.3	0.1	0.2	0.0	0.0	0.0	0.3	0.1	0.3
LAG3	0.5	0.2	0.2	0.0	0.0	0.0	0.2	0.0	0.1
HPDL	0.4	0.1	0.2	0.1	0.1	0.1	0.3	0.0	0.1
CXorf57	0.5	0.2	0.2	0.0	0.0	0.1	0.2	0.0	0.2
ALOXE3	0.3	0.0	0.2	0.0	0.1	0.1	0.3	0.0	0.3
MYH11	0.4	0.1	0.2	0.0	0.1	0.1	0.2	0.0	0.2
LINC00615	0.4	0.1	0.2	0.0	0.1	0.1	0.2	0.0	0.1
HECW1	0.4	0.1	0.2	0.1	0.1	0.1	0.1	0.0	0.1
GPR89B	0.4	0.1	0.1	0.0	0.0	0.0	0.3	0.1	0.2
CCDC65	0.3	0.1	0.2	0.0	0.1	0.0	0.3	0.0	0.1
LOC101928705	0.3	0.2	0.2	0.1	0.0	0.1	0.3	0.0	0.1
SIGLEC6	0.4	0.0	0.1	0.0	0.1	0.1	0.4	0.1	0.2
LOC101929464	0.3	0.1	0.4	0.1	0.1	0.1	0.2	0.0	0.1
IL12RB2	0.5	0.1	0.2	0.0	0.1	0.1	0.3	0.0	0.1
ARSE	0.3	0.1	0.2	0.1	0.1	0.1	0.3	0.0	0.2
SCN9A	0.4	0.1	0.2	0.0	0.1	0.0	0.2	0.0	0.2
LOC101929438	0.3	0.1	0.1	0.0	0.1	0.1	0.3	0.0	0.2
RBFADN	0.4	0.1	0.1	0.0	0.1	0.0	0.2	0.0	0.3
IGSF23	0.5	0.1	0.2	0.1	0.1	0.2	0.1	0.0	0.1
LOC100996379	0.4	0.2	0.1	0.0	0.1	0.0	0.1	0.0	0.2
HCG27	0.5	0.2	0.1	0.0	0.1	0.1	0.2	0.0	0.1
NIPSNAP3B	0.4	0.2	0.2	0.1	0.1	0.1	0.1	0.0	0.1
HDGFL1	0.2	0.2	0.1	0.4	0.1	0.2	0.0	0.0	0.0
HS3ST5	0.3	0.1	0.2	0.1	0.1	0.1	0.3	0.0	0.2
PRKAR2B	0.4	0.2	0.0	0.0	0.0	0.0	0.4	0.0	0.1
LOC440925	0.3	0.1	0.2	0.0	0.1	0.2	0.2	0.1	0.1
SLC36A3	0.3	0.1	0.2	0.0	0.1	0.1	0.4	0.0	0.1
DSCAM	0.1	0.1	0.1	0.1	0.1	0.1	0.4	0.1	0.2
TMEM72-AS1	0.5	0.1	0.2	0.0	0.0	0.1	0.2	0.0	0.2
SLC7A13	0.4	0.1	0.1	0.0	0.0	0.0	0.4	0.0	0.2
FCRLB	0.3	0.1	0.1	0.1	0.1	0.1	0.4	0.1	0.2
ALOX12B	0.4	0.1	0.2	0.0	0.0	0.1	0.2	0.0	0.1
DNAJC3-AS1	0.4	0.1	0.1	0.0	0.0	0.0	0.4	0.0	0.1
TMEM30B	0.4	0.1	0.3	0.0	0.0	0.1	0.1	0.0	0.1
HSPA1L	0.5	0.1	0.1	0.1	0.1	0.1	0.1	0.0	0.1
CASC9	0.4	0.1	0.3	0.0	0.0	0.0	0.2	0.0	0.1
LNX1	0.4	0.1	0.2	0.0	0.0	0.1	0.2	0.0	0.2
LOC100505940	0.2	0.2	0.1	0.1	0.1	0.1	0.1	0.1	0.4
LOC285593	0.5	0.1	0.2	0.0	0.0	0.1	0.2	0.0	0.1
DUOX1	0.3	0.1	0.3	0.0	0.1	0.1	0.2	0.0	0.1
HYDIN	0.5	0.1	0.2	0.0	0.0	0.1	0.2	0.0	0.1
FAM186B	0.4	0.1	0.1	0.0	0.0	0.1	0.3	0.1	0.2
VWCE	0.4	0.1	0.2	0.0	0.1	0.1	0.2	0.0	0.2
KLHL35	0.4	0.1	0.2	0.1	0.0	0.1	0.3	0.1	0.1
BMP3	0.3	0.1	0.2	0.0	0.0	0.1	0.3	0.1	0.2
TMC4	0.6	0.1	0.1	0.0	0.1	0.0	0.1	0.0	0.1
PCSK1	0.3	0.2	0.2	0.0	0.1	0.0	0.2	0.0	0.2
GGT1	0.4	0.2	0.2	0.0	0.0	0.0	0.2	0.0	0.1
SPATC1	0.4	0.1	0.3	0.0	0.1	0.1	0.1	0.0	0.1
LOC100507291	0.3	0.1	0.2	0.0	0.1	0.0	0.4	0.0	0.1

IGSF3	0.3	0.1	0.2	0.0	0.0	0.0	0.2	0.1	0.2
BTBD11	0.5	0.2	0.2	0.1	0.0	0.1	0.1	0.1	0.0
NEURL	0.2	0.1	0.1	0.1	0.1	0.2	0.2	0.1	0.3
RSPH9	0.5	0.0	0.3	0.0	0.1	0.0	0.2	0.1	0.1
ATP8B3	0.4	0.2	0.2	0.1	0.1	0.1	0.1	0.0	0.1
NBPF8	0.4	0.1	0.2	0.0	0.1	0.0	0.3	0.0	0.1
PRCD	0.3	0.1	0.3	0.0	0.1	0.1	0.2	0.0	0.2
VWDE	0.3	0.1	0.2	0.0	0.0	0.1	0.3	0.0	0.2
SLC22A14	0.6	0.1	0.2	0.0	0.0	0.1	0.1	0.0	0.1
LOC100289650	0.3	0.0	0.1	0.1	0.2	0.1	0.2	0.0	0.1
DLX2	0.2	0.2	0.1	0.1	0.1	0.1	0.2	0.0	0.2
TMEM204	0.3	0.1	0.2	0.1	0.0	0.1	0.3	0.1	0.2
C1orf185	0.5	0.2	0.2	0.1	0.1	0.0	0.0	0.0	0.1
ERVFRD-1	0.4	0.1	0.1	0.0	0.1	0.1	0.3	0.0	0.2
UPB1	0.5	0.1	0.2	0.0	0.1	0.1	0.1	0.0	0.0
C21orf49	0.3	0.1	0.1	0.0	0.1	0.1	0.3	0.0	0.1
BLK	0.2	0.1	0.2	0.0	0.2	0.1	0.2	0.0	0.2
FOXD3	0.4	0.2	0.2	0.1	0.0	0.1	0.1	0.0	0.0
CDH20	0.2	0.1	0.1	0.0	0.1	0.1	0.4	0.0	0.2
EEPD1	0.5	0.2	0.1	0.0	0.1	0.1	0.1	0.0	0.1
ENO1-AS1	0.2	0.2	0.2	0.1	0.0	0.2	0.1	0.1	0.1
NPHP3-AS1	0.3	0.1	0.1	0.0	0.1	0.1	0.3	0.0	0.1
TSPO2	0.3	0.1	0.2	0.0	0.1	0.2	0.1	0.1	0.1
LINC00842	0.2	0.1	0.1	0.0	0.1	0.2	0.3	0.0	0.2
WDR65	0.4	0.1	0.2	0.0	0.1	0.1	0.2	0.1	0.1
OR2A42	0.3	0.1	0.2	0.1	0.2	0.1	0.2	0.0	0.1
GPR116	0.3	0.1	0.2	0.0	0.1	0.1	0.1	0.0	0.1
LTK	0.3	0.2	0.2	0.0	0.1	0.1	0.3	0.0	0.1
HMCN1	0.5	0.1	0.2	0.0	0.1	0.1	0.2	0.0	0.1
SNX10	0.4	0.3	0.2	0.0	0.0	0.1	0.1	0.0	0.1
MLK7-AS1	0.4	0.1	0.1	0.0	0.1	0.1	0.3	0.0	0.1
CCDC113	0.3	0.1	0.3	0.1	0.0	0.2	0.2	0.0	0.1
LOC440910	0.4	0.0	0.2	0.0	0.0	0.1	0.3	0.0	0.2
LOC101928963	0.1	0.1	0.2	0.1	0.1	0.0	0.3	0.0	0.3
CDH12	0.5	0.1	0.2	0.0	0.0	0.0	0.2	0.0	0.2
FSIP2	0.5	0.1	0.2	0.0	0.1	0.1	0.1	0.0	0.1
C1orf204	0.4	0.1	0.1	0.2	0.0	0.1	0.1	0.1	0.2
PCDHGA2	0.4	0.1	0.1	0.0	0.1	0.1	0.1	0.0	0.1
EPHA7	0.4	0.1	0.2	0.0	0.1	0.0	0.2	0.0	0.1
LOC100506124	0.6	0.1	0.2	0.1	0.0	0.0	0.1	0.0	0.1
LOC284825	0.4	0.1	0.1	0.1	0.1	0.1	0.2	0.0	0.1
GOLGA6L4	0.4	0.1	0.2	0.0	0.1	0.2	0.1	0.0	0.1
ELAC1	0.3	0.2	0.2	0.0	0.0	0.2	0.2	0.0	0.1
SLC2A12	0.3	0.1	0.1	0.0	0.1	0.1	0.3	0.0	0.1
CPNE5	0.4	0.1	0.3	0.0	0.1	0.1	0.1	0.0	0.1
CYP2A6	0.3	0.1	0.2	0.0	0.0	0.1	0.2	0.0	0.2
CNKSR2	0.4	0.1	0.1	0.0	0.0	0.1	0.2	0.0	0.2
LOC100996351	0.4	0.1	0.2	0.1	0.0	0.0	0.2	0.0	0.1
KCNAB1	0.4	0.1	0.1	0.0	0.1	0.1	0.2	0.0	0.2
DSCR9	0.4	0.1	0.2	0.0	0.1	0.1	0.2	0.0	0.1
FTCD	0.3	0.2	0.3	0.0	0.1	0.0	0.2	0.0	0.1
ALDH3A1	0.2	0.1	0.1	0.1	0.2	0.3	0.0	0.1	0.0
DGKE	0.4	0.1	0.3	0.0	0.0	0.1	0.2	0.0	0.1
MOV10L1	0.2	0.0	0.1	0.0	0.1	0.1	0.4	0.0	0.2
LOC100506013	0.4	0.1	0.2	0.0	0.0	0.1	0.2	0.0	0.1

LMOD2	0.4	0.1	0.2	0.1	0.1	0.1	0.1	0.0	0.1
GREB1	0.4	0.2	0.2	0.0	0.0	0.1	0.2	0.0	0.1
FAM66C	0.4	0.1	0.2	0.0	0.0	0.1	0.2	0.0	0.2
CCSER1	0.3	0.1	0.1	0.0	0.0	0.1	0.2	0.0	0.2
ARMC2	0.3	0.1	0.1	0.0	0.1	0.1	0.1	0.0	0.2
DHH	0.3	0.1	0.1	0.1	0.0	0.1	0.3	0.0	0.1
LOC101927685	0.3	0.1	0.0	0.0	0.0	0.2	0.3	0.0	0.3
MYOM1	0.4	0.1	0.2	0.0	0.1	0.1	0.2	0.0	0.1
CUBN	0.4	0.1	0.2	0.0	0.0	0.1	0.2	0.0	0.1
RASIP1	0.3	0.1	0.2	0.1	0.0	0.1	0.2	0.0	0.1
LOC101928857	0.4	0.1	0.2	0.0	0.1	0.1	0.2	0.0	0.1
CRLF1	0.3	0.2	0.2	0.0	0.0	0.1	0.1	0.1	0.1
PANX2	0.3	0.1	0.2	0.1	0.1	0.1	0.2	0.1	0.2
LOC729020	0.0	0.1	0.2	0.0	0.0	0.0	0.4	0.0	0.5
LOC646862	0.2	0.1	0.2	0.1	0.1	0.1	0.1	0.1	0.2
SLC25A34	0.4	0.1	0.2	0.0	0.1	0.1	0.2	0.0	0.0
STAC3	0.4	0.1	0.1	0.0	0.1	0.0	0.2	0.0	0.2
PLCH2	0.3	0.1	0.1	0.0	0.1	0.1	0.2	0.0	0.2
MORF4L2-AS1	0.3	0.1	0.2	0.1	0.1	0.0	0.2	0.0	0.1
ARMCX5-GPRASP2	0.3	0.1	0.2	0.0	0.0	0.1	0.2	0.0	0.1
AFAP1L2	0.3	0.1	0.1	0.1	0.0	0.2	0.2	0.0	0.1
MYO7A	0.4	0.2	0.2	0.0	0.0	0.1	0.1	0.0	0.1
ELOVL3	0.4	0.2	0.1	0.0	0.0	0.1	0.1	0.0	0.2
ZNF750	0.3	0.0	0.1	0.0	0.2	0.1	0.3	0.0	0.1
SNAR-A13	0.4	0.1	0.2	0.1	0.1	0.0	0.1	0.0	0.1
SNAR-A12	0.4	0.1	0.2	0.1	0.1	0.0	0.1	0.0	0.1
CCDC150	0.3	0.1	0.2	0.0	0.0	0.1	0.2	0.0	0.1
FLJ36000	0.4	0.1	0.2	0.0	0.1	0.1	0.2	0.0	0.1
MARVELD2	0.4	0.1	0.1	0.0	0.1	0.0	0.1	0.0	0.3
POU5F2	0.3	0.1	0.1	0.0	0.0	0.2	0.2	0.0	0.2
POPDC2	0.4	0.1	0.1	0.0	0.0	0.1	0.2	0.0	0.2
PPP1R36	0.3	0.1	0.1	0.0	0.1	0.0	0.2	0.1	0.2
NPR3	0.3	0.0	0.2	0.1	0.0	0.1	0.1	0.1	0.2
LOC100506276	0.4	0.1	0.1	0.1	0.1	0.1	0.2	0.0	0.1
FLJ31945	0.5	0.1	0.2	0.1	0.0	0.0	0.2	0.0	0.1
COL16A1	0.3	0.2	0.2	0.1	0.0	0.1	0.2	0.0	0.1
SLC25A53	0.2	0.2	0.3	0.1	0.0	0.1	0.1	0.0	0.2
C21orf90	0.2	0.1	0.1	0.0	0.1	0.1	0.2	0.1	0.2
NLRP11	0.4	0.1	0.2	0.0	0.0	0.0	0.2	0.0	0.1
LOC100270804	0.3	0.1	0.2	0.0	0.1	0.1	0.1	0.0	0.2
LOC101060022	0.2	0.1	0.2	0.0	0.0	0.1	0.1	0.0	0.2
CA5A	0.3	0.2	0.2	0.0	0.0	0.0	0.1	0.0	0.2
KIAA1107	0.3	0.2	0.3	0.0	0.0	0.1	0.1	0.0	0.2
RTN1	0.3	0.1	0.2	0.0	0.0	0.1	0.2	0.0	0.1
PCDHGB5	0.4	0.1	0.2	0.0	0.1	0.0	0.2	0.0	0.1
LOC100506606	0.5	0.1	0.1	0.0	0.1	0.0	0.2	0.0	0.1
C19orf35	0.3	0.1	0.1	0.1	0.1	0.0	0.3	0.0	0.2
LOC101928985	0.5	0.0	0.1	0.0	0.1	0.0	0.2	0.1	0.1
TCEAL6	0.3	0.1	0.2	0.0	0.1	0.1	0.1	0.0	0.1
CCDC74A	0.3	0.1	0.1	0.0	0.1	0.1	0.2	0.0	0.2
LOC101928922	0.3	0.0	0.1	0.0	0.0	0.1	0.2	0.1	0.1
LINC01005	0.3	0.1	0.2	0.0	0.0	0.1	0.3	0.0	0.1
KCNK7	0.2	0.2	0.1	0.1	0.1	0.1	0.2	0.0	0.2
PCDHGB6	0.5	0.1	0.2	0.0	0.1	0.0	0.1	0.0	0.0
COL4A4	0.3	0.1	0.2	0.0	0.1	0.0	0.2	0.0	0.1

MATN1-AS1	0.3	0.1	0.2	0.0	0.0	0.1	0.2	0.1	0.1
SAMD11	0.4	0.1	0.1	0.0	0.0	0.0	0.3	0.0	0.0
NR4A3	0.4	0.1	0.1	0.0	0.0	0.1	0.2	0.0	0.1
RAB36	0.3	0.1	0.1	0.1	0.1	0.1	0.3	0.0	0.1
PCDHB3	0.2	0.1	0.2	0.0	0.1	0.1	0.2	0.0	0.0
CMKLR1	0.1	0.1	0.2	0.1	0.0	0.1	0.1	0.0	0.2
LOC101928820	0.3	0.1	0.2	0.0	0.1	0.0	0.2	0.0	0.2
ASAH2	0.3	0.1	0.2	0.0	0.0	0.1	0.1	0.1	0.2
LOC101928592	0.5	0.1	0.0	0.0	0.0	0.1	0.1	0.0	0.0
FLJ43585	0.4	0.1	0.2	0.0	0.0	0.1	0.2	0.0	0.1
SRPK3	0.2	0.1	0.2	0.0	0.1	0.1	0.2	0.0	0.1
LOC101929559	0.3	0.1	0.2	0.0	0.0	0.1	0.2	0.0	0.1
ALK	0.4	0.1	0.2	0.0	0.1	0.0	0.2	0.0	0.1
ABLIM2	0.2	0.2	0.2	0.0	0.1	0.1	0.1	0.0	0.2
LOC729159	0.4	0.1	0.0	0.0	0.1	0.1	0.2	0.0	0.1
PPL	0.3	0.1	0.3	0.0	0.1	0.0	0.1	0.0	0.1
GOLGA8M	0.4	0.2	0.2	0.0	0.0	0.1	0.0	0.0	0.1
GPR98	0.3	0.1	0.1	0.0	0.0	0.0	0.3	0.0	0.2
LCA5	0.3	0.1	0.1	0.0	0.0	0.0	0.2	0.0	0.2
KCNJ4	0.3	0.1	0.2	0.0	0.0	0.0	0.3	0.0	0.1
PYGM	0.4	0.1	0.2	0.0	0.0	0.0	0.1	0.0	0.1
CCDC11	0.3	0.1	0.1	0.0	0.1	0.1	0.1	0.0	0.2
ACTA1	0.3	0.1	0.3	0.0	0.0	0.0	0.1	0.0	0.1
TOB1-AS1	0.3	0.1	0.1	0.0	0.1	0.1	0.3	0.0	0.1
ATP2A3	0.3	0.1	0.1	0.0	0.1	0.1	0.2	0.0	0.1
CASKIN1	0.2	0.1	0.1	0.1	0.1	0.0	0.2	0.1	0.1
RBM47	0.4	0.2	0.2	0.0	0.0	0.1	0.1	0.0	0.0
ZNF727	0.2	0.1	0.2	0.0	0.1	0.1	0.2	0.0	0.2
LOC101928188	0.3	0.1	0.1	0.0	0.1	0.1	0.2	0.0	0.0
FAM188B	0.3	0.0	0.2	0.1	0.1	0.0	0.2	0.0	0.2
TTLL13	0.3	0.2	0.2	0.0	0.0	0.1	0.2	0.0	0.1
BZRAP1-AS1	0.3	0.1	0.1	0.0	0.0	0.1	0.1	0.0	0.2
GPR113	0.3	0.1	0.2	0.0	0.1	0.1	0.2	0.0	0.1
WWC2-AS2	0.3	0.1	0.1	0.0	0.1	0.1	0.2	0.0	0.1
FGD4	0.4	0.1	0.1	0.0	0.1	0.0	0.2	0.0	0.2
PCDHGC5	0.4	0.1	0.2	0.0	0.1	0.0	0.2	0.0	0.0
CORO2B	0.1	0.1	0.1	0.0	0.1	0.1	0.2	0.1	0.3
LOC100130950	0.3	0.1	0.1	0.0	0.1	0.0	0.2	0.0	0.1
C1orf116	0.2	0.2	0.1	0.0	0.1	0.1	0.1	0.0	0.1
HEATR4	0.4	0.1	0.2	0.0	0.1	0.1	0.1	0.0	0.1
MFSD2B	0.2	0.1	0.1	0.0	0.1	0.1	0.2	0.0	0.2
SLC7A7	0.3	0.1	0.1	0.0	0.0	0.1	0.2	0.0	0.0
FAM134B	0.3	0.1	0.1	0.1	0.0	0.1	0.2	0.0	0.0
GPRASP1	0.3	0.0	0.2	0.0	0.1	0.1	0.2	0.0	0.1
FOXD2	0.2	0.1	0.2	0.0	0.1	0.1	0.1	0.0	0.2
PCDHB8	0.4	0.1	0.1	0.0	0.0	0.1	0.1	0.0	0.1
LOC729121	0.2	0.1	0.1	0.0	0.1	0.1	0.3	0.0	0.1
LOC101927898	0.2	0.1	0.2	0.0	0.1	0.1	0.2	0.0	0.1
PCDHB16	0.3	0.1	0.2	0.0	0.1	0.0	0.2	0.1	0.1
MEI1	0.3	0.1	0.1	0.0	0.1	0.1	0.2	0.0	0.1
CYP11A1	0.2	0.1	0.2	0.1	0.0	0.1	0.1	0.0	0.2
NRCAM	0.3	0.1	0.1	0.0	0.1	0.1	0.2	0.0	0.2
POTEI	0.3	0.1	0.1	0.1	0.1	0.1	0.1	0.0	0.1
B4GALNT1	0.2	0.0	0.2	0.0	0.1	0.0	0.2	0.0	0.2
CABYR	0.3	0.1	0.2	0.0	0.0	0.1	0.1	0.1	0.1

WDR86		0.4	0.0	0.2	0.0	0.1	0.1	0.1	0.0	0.1
HES2		0.2	0.2	0.1	0.1	0.1	0.0	0.1	0.1	0.1
FAM196A		0.3	0.1	0.1	0.0	0.1	0.1	0.2	0.0	0.1
PCDH7		0.4	0.1	0.2	0.0	0.1	0.1	0.1	0.0	0.0
LOC728819		0.2	0.2	0.1	0.0	0.1	0.1	0.1	0.1	0.1
ZKSCAN7		0.2	0.1	0.2	0.0	0.1	0.0	0.2	0.0	0.1
GOLGA8R		0.0	0.2	0.4	0.0	0.2	0.1	0.1	0.0	0.0
CCDC64		0.3	0.0	0.2	0.0	0.0	0.1	0.2	0.0	0.1
CBR3-AS1		0.2	0.1	0.3	0.0	0.1	0.1	0.0	0.0	0.1
GCOM1		0.0	0.3	0.2	0.0	0.0	0.0	0.1	0.1	0.2
LOC100506314		0.3	0.0	0.3	0.0	0.1	0.1	0.1	0.0	0.0
SYT14		0.4	0.1	0.1	0.0	0.0	0.1	0.2	0.0	0.1
ZNF461		0.4	0.2	0.2	0.0	0.0	0.1	0.1	0.0	0.1
IRF4		0.4	0.1	0.1	0.0	0.1	0.0	0.2	0.0	0.1
SCNN1A		0.4	0.1	0.2	0.0	0.0	0.1	0.0	0.0	0.0
C1orf191		0.3	0.1	0.2	0.0	0.0	0.0	0.2	0.0	0.1
CCDC41-AS1		0.3	0.1	0.1	0.0	0.1	0.1	0.2	0.0	0.1
ZAP70		0.2	0.0	0.2	0.0	0.1	0.1	0.2	0.1	0.2
TMEM139		0.2	0.1	0.0	0.0	0.1	0.1	0.2	0.0	0.2
LOC101927261		0.4	0.0	0.2	0.0	0.1	0.0	0.2	0.0	0.0
MDS2		0.4	0.0	0.2	0.0	0.1	0.0	0.1	0.0	0.2
LOC284561		0.2	0.1	0.1	0.0	0.0	0.0	0.2	0.0	0.2
LINC00087		0.2	0.2	0.1	0.0	0.1	0.1	0.1	0.1	0.1
EMILIN1		0.3	0.1	0.2	0.0	0.1	0.0	0.1	0.0	0.1
LOC100996246		0.2	0.1	0.2	0.1	0.1	0.1	0.1	0.0	0.1
SAMD15		0.3	0.1	0.2	0.0	0.1	0.1	0.1	0.0	0.1
LOC100506655		0.4	0.1	0.1	0.0	0.1	0.1	0.1	0.0	0.1
MIR600HG		0.3	0.0	0.1	0.0	0.0	0.0	0.2	0.0	0.1
THAP9		0.3	0.1	0.2	0.0	0.0	0.1	0.1	0.0	0.1
E2F2		0.1	0.1	0.2	0.1	0.0	0.1	0.1	0.0	0.2
KIAA1462		0.3	0.2	0.1	0.0	0.1	0.1	0.1	0.0	0.0
ZNF878		0.4	0.0	0.1	0.0	0.0	0.0	0.2	0.0	0.1
GIMAP2		0.2	0.2	0.2	0.0	0.0	0.1	0.2	0.0	0.1
EPHA4		0.2	0.1	0.1	0.0	0.1	0.1	0.2	0.0	0.1
LOC100129726		0.2	0.1	0.1	0.0	0.0	0.0	0.2	0.0	0.2
ZBTB32		0.3	0.1	0.1	0.1	0.1	0.1	0.2	0.0	0.1
PSG9		0.3	0.0	0.2	0.0	0.0	0.1	0.1	0.0	0.1
SLC16A11		0.3	0.1	0.2	0.0	0.0	0.1	0.1	0.1	0.1
KLHL31		0.3	0.1	0.1	0.0	0.0	0.1	0.1	0.1	0.0
CTNNA2		0.2	0.1	0.2	0.0	0.1	0.1	0.1	0.0	0.1
HYAL1		0.3	0.1	0.2	0.0	0.0	0.0	0.2	0.0	0.1
ZNF665		0.2	0.1	0.1	0.0	0.1	0.1	0.1	0.0	0.2
GSG1		0.0	0.1	0.1	0.0	0.1	0.0	0.1	0.1	0.4
LOC283177		0.3	0.1	0.2	0.0	0.1	0.1	0.1	0.0	0.1
C5		0.3	0.1	0.1	0.0	0.1	0.1	0.2	0.0	0.1
COLQ		0.2	0.1	0.1	0.1	0.0	0.1	0.2	0.0	0.1
CSGALNACT1		0.3	0.1	0.1	0.0	0.1	0.1	0.2	0.0	0.1
PRODH		0.3	0.1	0.2	0.1	0.0	0.1	0.1	0.0	0.1
ZNF695		0.3	0.1	0.1	0.0	0.1	0.0	0.3	0.0	0.0
PCDHB4		0.4	0.0	0.2	0.0	0.0	0.0	0.2	0.0	0.0
	44631.0	0.2	0.2	0.2	0.0	0.0	0.0	0.1	0.0	0.1
PIGZ		0.3	0.2	0.2	0.0	0.0	0.1	0.1	0.0	0.1
LOC101929234		0.3	0.1	0.1	0.0	0.0	0.1	0.1	0.1	0.1
CYP2W1		0.2	0.1	0.1	0.0	0.0	0.0	0.2	0.1	0.1
KIAA0825		0.3	0.1	0.1	0.0	0.1	0.0	0.1	0.0	0.1

LOC101929743	0.3	0.1	0.1	0.0	0.0	0.1	0.2	0.0	0.1
ONECUT1	0.2	0.1	0.1	0.0	0.1	0.1	0.1	0.1	0.2
LOC101927658	0.4	0.1	0.0	0.0	0.0	0.1	0.1	0.0	0.1
GOLGA6L9	0.4	0.1	0.0	0.0	0.0	0.1	0.1	0.0	0.1
LOC100506172	0.4	0.0	0.1	0.0	0.0	0.0	0.2	0.0	0.1
WDR88	0.2	0.0	0.2	0.1	0.1	0.1	0.1	0.0	0.1
OPRM1	0.4	0.1	0.1	0.0	0.0	0.0	0.2	0.0	0.1
ZNF43	0.2	0.1	0.2	0.0	0.1	0.1	0.2	0.0	0.1
LOC101927282	0.5	0.0	0.1	0.0	0.0	0.0	0.2	0.0	0.1
MYOM2	0.3	0.1	0.1	0.0	0.1	0.1	0.1	0.0	0.1
NEK5	0.4	0.0	0.1	0.0	0.0	0.0	0.2	0.0	0.1
GNRHR	0.3	0.0	0.1	0.0	0.1	0.0	0.1	0.0	0.2
TBC1D29	0.4	0.1	0.2	0.0	0.0	0.1	0.0	0.0	0.2
RNASE10	0.2	0.2	0.2	0.0	0.0	0.0	0.3	0.0	0.1
CHRNA10	0.2	0.1	0.2	0.0	0.1	0.0	0.2	0.0	0.1
ARPC4-TTLL3	0.0	0.2	0.3	0.1	0.1	0.0	0.0	0.0	0.2
PDZD7	0.2	0.1	0.0	0.0	0.1	0.1	0.2	0.0	0.1
COL17A1	0.2	0.1	0.1	0.0	0.1	0.1	0.1	0.0	0.1
PRDM9	0.3	0.1	0.1	0.0	0.0	0.1	0.1	0.0	0.1
PARP15	0.2	0.1	0.1	0.0	0.0	0.0	0.2	0.0	0.1
PITX2	0.2	0.2	0.2	0.0	0.1	0.0	0.1	0.0	0.1
LOC101928627	0.4	0.0	0.2	0.0	0.0	0.1	0.1	0.0	0.1
ARHGEF10L	0.3	0.1	0.1	0.0	0.0	0.0	0.2	0.0	0.2
DPYSL4	0.3	0.1	0.1	0.0	0.1	0.0	0.2	0.0	0.1
S1PR1	0.1	0.1	0.2	0.0	0.1	0.0	0.2	0.1	0.1
ENO4	0.3	0.1	0.2	0.0	0.1	0.0	0.1	0.0	0.1
ADAMTS2	0.1	0.1	0.2	0.0	0.1	0.0	0.2	0.0	0.1
E2F8	0.2	0.0	0.0	0.0	0.1	0.1	0.2	0.0	0.2
POU4F2	0.2	0.1	0.1	0.1	0.1	0.1	0.1	0.1	0.0
B3GALT5	0.3	0.1	0.1	0.0	0.1	0.0	0.2	0.0	0.0
ALDH1A3	0.2	0.1	0.1	0.0	0.1	0.1	0.0	0.0	0.1
FGGY	0.3	0.1	0.2	0.0	0.1	0.1	0.1	0.0	0.0
SLC37A1	0.4	0.1	0.1	0.0	0.0	0.1	0.1	0.0	0.1
MEGF11	0.3	0.0	0.1	0.0	0.0	0.1	0.1	0.0	0.1
GOLGA8O	0.3	0.2	0.1	0.0	0.0	0.0	0.0	0.0	0.2
TTYH1	0.3	0.1	0.2	0.1	0.0	0.0	0.1	0.0	0.1
MAGEA2	0.3	0.1	0.1	0.0	0.0	0.1	0.1	0.0	0.2
PLEKHG6	0.3	0.1	0.2	0.0	0.0	0.1	0.1	0.0	0.1
LOC101929741	0.3	0.0	0.1	0.0	0.0	0.0	0.2	0.0	0.1
LOC101928613	0.2	0.1	0.2	0.0	0.1	0.0	0.0	0.0	0.1
GLI1	0.2	0.1	0.2	0.0	0.0	0.0	0.2	0.0	0.1
SOWAHD	0.1	0.2	0.2	0.0	0.1	0.1	0.1	0.1	0.1
TMPRSS7	0.2	0.1	0.1	0.0	0.0	0.0	0.2	0.0	0.1
NEU4	0.3	0.0	0.1	0.0	0.0	0.0	0.2	0.0	0.1
CD8A	0.3	0.1	0.1	0.0	0.1	0.1	0.1	0.0	0.1
LRRN4	0.2	0.1	0.2	0.0	0.1	0.1	0.1	0.0	0.0
LOC100505715	0.2	0.1	0.2	0.0	0.1	0.0	0.1	0.0	0.1
LOC101927612	0.2	0.1	0.1	0.0	0.0	0.0	0.2	0.1	0.1
NWD1	0.2	0.1	0.2	0.0	0.0	0.0	0.2	0.0	0.1
SLC51A	0.2	0.2	0.1	0.0	0.0	0.0	0.1	0.0	0.1
SAMD9L	0.2	0.1	0.1	0.0	0.1	0.1	0.1	0.0	0.2
OR2A7	0.2	0.0	0.2	0.0	0.0	0.1	0.2	0.0	0.0
RDH12	0.2	0.1	0.1	0.0	0.1	0.0	0.2	0.0	0.1
TMEM52	0.2	0.0	0.1	0.1	0.1	0.1	0.2	0.0	0.0
BAIAP2L2	0.2	0.2	0.1	0.0	0.0	0.0	0.2	0.1	0.1

SBK1	0.2	0.1	0.1	0.1	0.1	0.0	0.2	0.0	0.1
CSRNP3	0.3	0.1	0.1	0.0	0.0	0.0	0.1	0.0	0.1
LINC00319	0.2	0.1	0.1	0.1	0.1	0.1	0.1	0.0	0.1
DDX3Y	0.4	0.1	0.1	0.0	0.0	0.0	0.1	0.0	0.0
MYO16	0.3	0.1	0.1	0.1	0.1	0.0	0.1	0.0	0.1
FAM166B	0.2	0.0	0.2	0.1	0.0	0.1	0.1	0.0	0.1
LOC100129722	0.3	0.1	0.1	0.0	0.0	0.1	0.1	0.0	0.1
GPC2	0.2	0.2	0.2	0.0	0.1	0.1	0.1	0.0	0.0
CCDC166	0.3	0.1	0.1	0.0	0.0	0.0	0.3	0.0	0.0
MICALCL	0.2	0.1	0.0	0.0	0.0	0.0	0.2	0.0	0.2
ANO7	0.2	0.0	0.2	0.0	0.1	0.0	0.1	0.1	0.1
TECTA	0.3	0.1	0.1	0.0	0.0	0.1	0.2	0.0	0.1
COMP	0.2	0.1	0.1	0.1	0.0	0.1	0.2	0.0	0.1
ALPK3	0.3	0.1	0.1	0.0	0.0	0.0	0.1	0.0	0.1
KCP	0.2	0.1	0.2	0.1	0.0	0.0	0.1	0.0	0.0
TIGD6	0.3	0.0	0.1	0.0	0.0	0.1	0.2	0.0	0.1
CCR7	0.2	0.0	0.1	0.0	0.1	0.0	0.2	0.0	0.1
FLJ10489	0.4	0.1	0.1	0.0	0.0	0.1	0.2	0.0	0.0
RRH	0.3	0.1	0.1	0.0	0.0	0.1	0.1	0.0	0.2
LOC149351	0.3	0.1	0.1	0.0	0.1	0.1	0.0	0.0	0.1
CORIN	0.4	0.1	0.1	0.0	0.0	0.1	0.1	0.0	0.0
LOC101927295	0.3	0.1	0.1	0.0	0.0	0.0	0.2	0.0	0.1
MAP1A	0.4	0.1	0.1	0.0	0.0	0.1	0.1	0.0	0.0
MMP16	0.2	0.1	0.1	0.0	0.1	0.1	0.2	0.0	0.1
PTPRQ	0.2	0.0	0.1	0.0	0.0	0.0	0.3	0.0	0.2
DACT3	0.1	0.1	0.1	0.0	0.0	0.0	0.2	0.0	0.1
DCHS1	0.2	0.1	0.1	0.0	0.1	0.1	0.1	0.0	0.1
TG	0.4	0.1	0.2	0.0	0.0	0.0	0.1	0.0	0.0
ATP6V0A4	0.2	0.0	0.0	0.0	0.0	0.0	0.2	0.0	0.2
LOC101929151	0.3	0.1	0.1	0.0	0.0	0.1	0.2	0.0	0.1
ZNF773	0.2	0.1	0.1	0.1	0.1	0.0	0.1	0.0	0.1
KALRN	0.2	0.0	0.1	0.0	0.1	0.1	0.1	0.0	0.1
LINC00161	0.3	0.1	0.1	0.0	0.0	0.0	0.2	0.1	0.1
LINC00202-2	0.3	0.1	0.1	0.0	0.0	0.0	0.2	0.0	0.1
FLJ39095	0.3	0.0	0.2	0.0	0.1	0.0	0.1	0.0	0.1
HAL	0.3	0.1	0.1	0.0	0.1	0.0	0.0	0.0	0.1
LOC101927506	0.1	0.1	0.1	0.0	0.0	0.0	0.3	0.0	0.1
FLJ16341	0.3	0.1	0.1	0.0	0.0	0.0	0.2	0.0	0.1
SUSD2	0.3	0.1	0.1	0.0	0.0	0.0	0.1	0.0	0.1
LGR5	0.3	0.1	0.2	0.0	0.0	0.1	0.0	0.0	0.0
LOC101927864	0.2	0.1	0.1	0.0	0.1	0.0	0.1	0.0	0.1
HMMR	0.2	0.1	0.1	0.0	0.1	0.1	0.1	0.0	0.1
APOC4-APOC2	0.3	0.0	0.1	0.0	0.0	0.0	0.1	0.0	0.1
CHRNA7	0.3	0.1	0.1	0.0	0.0	0.0	0.2	0.0	0.1
RBM43	0.3	0.1	0.1	0.0	0.1	0.1	0.1	0.0	0.0
KCTD19	0.3	0.1	0.1	0.0	0.0	0.0	0.1	0.0	0.0
LOC101928279	0.3	0.1	0.1	0.0	0.0	0.1	0.1	0.0	0.1
TTC6	0.2	0.1	0.2	0.0	0.1	0.1	0.1	0.0	0.1
LYZL4	0.1	0.2	0.0	0.1	0.1	0.2	0.1	0.1	0.0
CDRT1	0.2	0.0	0.2	0.1	0.1	0.1	0.2	0.0	0.0
LOC101928774	0.3	0.0	0.1	0.0	0.1	0.0	0.1	0.0	0.1
TRIM39-RPP21	0.1	0.0	0.1	0.0	0.1	0.0	0.3	0.0	0.1
POU5F1B	0.1	0.1	0.1	0.0	0.1	0.1	0.1	0.0	0.1
KC6	0.4	0.0	0.1	0.0	0.0	0.1	0.1	0.0	0.1
FILIP1	0.3	0.1	0.1	0.0	0.0	0.0	0.1	0.0	0.1

TRHDE-AS1	0.2	0.1	0.1	0.0	0.0	0.0	0.1	0.0	0.1
TENM2	0.2	0.1	0.1	0.0	0.1	0.1	0.2	0.0	0.1
VAV1	0.2	0.1	0.2	0.0	0.1	0.0	0.1	0.0	0.1
C17orf47	0.2	0.1	0.2	0.0	0.0	0.1	0.1	0.0	0.0
CCDC121	0.2	0.0	0.1	0.0	0.1	0.1	0.2	0.0	0.1
CTAGE4	0.1	0.1	0.1	0.0	0.0	0.0	0.3	0.0	0.1
LOC100128386	0.2	0.1	0.1	0.0	0.1	0.0	0.1	0.0	0.1
SKIDA1	0.2	0.1	0.1	0.0	0.0	0.1	0.1	0.0	0.1
FLJ38723	0.2	0.1	0.1	0.0	0.0	0.0	0.1	0.0	0.1
SPATA32	0.2	0.0	0.2	0.0	0.0	0.0	0.1	0.0	0.1
GJA9-MYCBP	0.3	0.1	0.2	0.0	0.0	0.0	0.1	0.0	0.1
FHAD1	0.2	0.1	0.1	0.0	0.1	0.1	0.1	0.0	0.1
SNTG2	0.2	0.1	0.1	0.0	0.0	0.0	0.1	0.0	0.1
PAPPA	0.2	0.0	0.0	0.0	0.1	0.2	0.0	0.0	0.1
C1orf167	0.2	0.1	0.1	0.0	0.0	0.0	0.2	0.0	0.1
LOC101927703	0.2	0.0	0.1	0.0	0.0	0.0	0.2	0.0	0.1
TEX41	0.3	0.1	0.1	0.0	0.0	0.0	0.1	0.0	0.1
BSN	0.3	0.1	0.1	0.0	0.0	0.0	0.1	0.0	0.1
MEGF6	0.1	0.0	0.1	0.1	0.1	0.1	0.1	0.0	0.0
HSFX1	0.3	0.1	0.1	0.0	0.0	0.0	0.1	0.0	0.1
HSFX2	0.3	0.1	0.1	0.0	0.0	0.0	0.1	0.0	0.1
C14orf183	0.3	0.0	0.1	0.0	0.0	0.0	0.2	0.1	0.0
GJB3	0.0	0.1	0.1	0.1	0.0	0.0	0.2	0.0	0.1
FLG2	0.3	0.0	0.1	0.0	0.1	0.0	0.1	0.0	0.0
ECM2	0.3	0.0	0.1	0.0	0.1	0.0	0.1	0.0	0.1
BTBD8	0.3	0.0	0.1	0.0	0.1	0.0	0.1	0.0	0.1
TBX2	0.2	0.1	0.1	0.1	0.0	0.0	0.1	0.1	0.1
SIGLEC12	0.3	0.1	0.1	0.0	0.1	0.1	0.1	0.0	0.1
PLCH1	0.2	0.1	0.1	0.0	0.0	0.1	0.1	0.0	0.1
LOC101927677	0.2	0.0	0.2	0.0	0.0	0.1	0.1	0.0	0.0
LRRC16B	0.2	0.1	0.1	0.0	0.0	0.0	0.1	0.0	0.1
GRIN3B	0.2	0.1	0.1	0.0	0.0	0.1	0.1	0.0	0.0
CCDC151	0.3	0.1	0.1	0.0	0.0	0.1	0.1	0.0	0.0
ZNF605	0.2	0.1	0.0	0.0	0.1	0.1	0.2	0.0	0.1
PROC	0.1	0.2	0.0	0.1	0.1	0.0	0.2	0.0	0.0
EYS	0.2	0.1	0.1	0.0	0.0	0.0	0.1	0.0	0.1
ENAM	0.1	0.1	0.1	0.0	0.0	0.1	0.2	0.0	0.1
LINC00202-1	0.3	0.1	0.2	0.0	0.0	0.0	0.1	0.0	0.0
B3GALT2	0.1	0.0	0.1	0.0	0.0	0.0	0.4	0.0	0.0
ZC3H6	0.3	0.1	0.1	0.0	0.1	0.0	0.0	0.0	0.0
GPR56	0.2	0.1	0.0	0.0	0.1	0.1	0.1	0.0	0.1
RHD	0.1	0.1	0.1	0.0	0.0	0.0	0.2	0.0	0.1
DMRTA2	0.2	0.1	0.1	0.1	0.0	0.0	0.1	0.1	0.1
SLC16A2	0.3	0.1	0.1	0.0	0.0	0.1	0.1	0.0	0.1
LOC100049716	0.2	0.1	0.1	0.0	0.0	0.0	0.1	0.0	0.2
THBS4	0.2	0.1	0.1	0.0	0.0	0.1	0.1	0.0	0.1
TBC1D10C	0.1	0.1	0.1	0.0	0.0	0.1	0.1	0.0	0.1
AGBL2	0.2	0.1	0.1	0.0	0.0	0.1	0.1	0.0	0.1
LOC101926963	0.1	0.1	0.1	0.0	0.0	0.1	0.1	0.1	0.1
DLGAP1-AS5	0.2	0.0	0.1	0.0	0.0	0.0	0.1	0.0	0.1
DDX25	0.2	0.1	0.0	0.0	0.0	0.1	0.2	0.0	0.1
KBTBD11	0.1	0.0	0.1	0.0	0.1	0.1	0.1	0.0	0.0
LOC285389	0.2	0.0	0.1	0.0	0.0	0.0	0.2	0.0	0.1
SULT1A2	0.1	0.0	0.2	0.0	0.1	0.1	0.1	0.0	0.0
RAET1L	0.1	0.1	0.1	0.0	0.1	0.0	0.1	0.0	0.1

PCOLCE-AS1	0.2	0.1	0.2	0.0	0.0	0.0	0.0	0.0	0.1
MTMR7	0.2	0.1	0.1	0.0	0.0	0.0	0.2	0.0	0.0
KANK1	0.1	0.0	0.0	0.0	0.0	0.0	0.3	0.0	0.2
IL9R	0.2	0.0	0.2	0.0	0.0	0.0	0.1	0.0	0.1
IL9R	0.2	0.0	0.2	0.0	0.0	0.0	0.1	0.0	0.1
RIBC2	0.2	0.1	0.0	0.0	0.0	0.1	0.1	0.1	0.0
FAM179A	0.2	0.1	0.2	0.0	0.0	0.1	0.1	0.0	0.0
RIMBP3B	0.1	0.0	0.1	0.0	0.0	0.0	0.3	0.0	0.1
HS6ST3	0.1	0.1	0.1	0.0	0.0	0.1	0.2	0.0	0.1
LOC101928284	0.2	0.1	0.1	0.0	0.0	0.0	0.1	0.0	0.1
LOC101929124	0.2	0.0	0.1	0.0	0.0	0.0	0.1	0.0	0.1
AIRN	0.2	0.0	0.1	0.0	0.1	0.1	0.1	0.0	0.1
LOC101929387	0.2	0.1	0.1	0.0	0.1	0.0	0.1	0.0	0.1
LOC145474	0.3	0.1	0.1	0.0	0.0	0.0	0.1	0.0	0.0
SLC39A5	0.2	0.1	0.1	0.0	0.0	0.0	0.1	0.0	0.1
LOC100507634	0.1	0.0	0.2	0.0	0.1	0.0	0.1	0.0	0.1
AKR1E2	0.1	0.1	0.0	0.0	0.0	0.0	0.3	0.0	0.1
FOXD4L6	0.3	0.1	0.0	0.0	0.0	0.0	0.2	0.1	0.0
LOC101928484	0.3	0.0	0.1	0.0	0.0	0.0	0.1	0.0	0.1
MATK	0.1	0.1	0.0	0.1	0.1	0.1	0.1	0.0	0.1
TFEC	0.1	0.1	0.1	0.0	0.0	0.1	0.0	0.1	0.2
CTNND2	0.1	0.1	0.1	0.1	0.1	0.0	0.1	0.0	0.1
LIN28A	0.3	0.0	0.1	0.0	0.0	0.0	0.1	0.0	0.1
FAM194A	0.2	0.0	0.1	0.1	0.1	0.0	0.1	0.0	0.1
LOC101927636	0.3	0.1	0.2	0.0	0.0	0.0	0.0	0.0	0.0
POTEJ	0.2	0.1	0.1	0.0	0.0	0.1	0.1	0.0	0.1
ZNF441	0.2	0.1	0.1	0.0	0.0	0.1	0.1	0.0	0.0
GATA6-AS1	0.1	0.1	0.2	0.0	0.0	0.0	0.1	0.0	0.1
HBCBP	0.2	0.0	0.2	0.0	0.0	0.1	0.1	0.0	0.0
GPR155	0.2	0.1	0.0	0.0	0.1	0.0	0.1	0.0	0.1
CARNS1	0.2	0.1	0.1	0.0	0.0	0.0	0.0	0.0	0.1
LOC101928867	0.2	0.1	0.1	0.0	0.0	0.0	0.1	0.0	0.1
FIGNL2	0.2	0.1	0.1	0.0	0.0	0.0	0.2	0.0	0.0
ADAM12	0.2	0.0	0.1	0.0	0.0	0.1	0.1	0.0	0.1
SLC13A4	0.2	0.1	0.1	0.0	0.1	0.0	0.1	0.0	0.0
FUT5	0.2	0.1	0.1	0.0	0.0	0.0	0.1	0.0	0.0
HTRA3	0.1	0.0	0.1	0.1	0.1	0.1	0.1	0.0	0.0
NID2	0.2	0.1	0.1	0.0	0.0	0.0	0.1	0.0	0.1
ZNF107	0.2	0.0	0.1	0.0	0.0	0.0	0.1	0.0	0.1
LOC101928729	0.2	0.1	0.1	0.0	0.0	0.0	0.0	0.0	0.1
MX2	0.2	0.1	0.1	0.0	0.0	0.1	0.0	0.0	0.0
PSG5	0.1	0.0	0.0	0.0	0.1	0.1	0.2	0.0	0.1
KCNH3	0.2	0.1	0.1	0.0	0.1	0.0	0.1	0.0	0.1
STON1	0.2	0.1	0.1	0.0	0.0	0.0	0.1	0.0	0.1
LY86-AS1	0.2	0.1	0.1	0.0	0.0	0.0	0.1	0.0	0.1
LINC00559	0.2	0.0	0.1	0.0	0.0	0.0	0.2	0.0	0.0
CRB2	0.2	0.1	0.1	0.0	0.0	0.0	0.1	0.0	0.1
ADCY4	0.2	0.1	0.1	0.0	0.0	0.0	0.1	0.0	0.1
MCF2L2	0.2	0.0	0.1	0.0	0.0	0.1	0.1	0.0	0.0
NCKAP5	0.1	0.0	0.1	0.0	0.0	0.1	0.2	0.0	0.2
LOC101929607	0.2	0.0	0.1	0.0	0.0	0.1	0.1	0.0	0.1
AS3MT	0.4	0.0	0.1	0.0	0.0	0.1	0.1	0.0	0.0
LOC100505532	0.2	0.0	0.1	0.0	0.0	0.0	0.1	0.0	0.0
AGAP7	0.1	0.0	0.0	0.0	0.0	0.0	0.1	0.0	0.3
LOC101593348	0.2	0.0	0.2	0.0	0.0	0.0	0.1	0.0	0.0

KCNA7	0.4	0.0	0.1	0.0	0.0	0.0	0.0	0.0	0.0
LOC101928741	0.3	0.0	0.1	0.0	0.0	0.0	0.0	0.0	0.0
CNKSR1	0.1	0.0	0.1	0.0	0.1	0.1	0.1	0.1	0.1
CCDC171	0.1	0.1	0.1	0.0	0.1	0.0	0.1	0.0	0.1
GRK4	0.1	0.1	0.1	0.0	0.0	0.1	0.1	0.0	0.1
ATP8A2	0.1	0.0	0.0	0.0	0.1	0.0	0.2	0.0	0.1
WAS	0.1	0.1	0.1	0.0	0.0	0.0	0.1	0.0	0.1
TRIM54	0.1	0.0	0.0	0.0	0.1	0.1	0.2	0.0	0.0
ACTN3	0.2	0.1	0.1	0.0	0.0	0.0	0.1	0.0	0.1
FGD3	0.2	0.1	0.1	0.0	0.0	0.0	0.1	0.0	0.1
IGSF9	0.3	0.1	0.1	0.0	0.0	0.0	0.0	0.0	0.1
GPLD1	0.1	0.1	0.1	0.0	0.0	0.0	0.1	0.0	0.1
HNF1A	0.2	0.0	0.1	0.0	0.1	0.1	0.1	0.0	0.0
USP17L7	0.2	0.0	0.1	0.0	0.1	0.0	0.1	0.0	0.0
BHMT	0.2	0.1	0.1	0.0	0.1	0.0	0.1	0.0	0.1
FOXD4L4	0.2	0.1	0.1	0.0	0.1	0.0	0.0	0.0	0.1
FOXD4L2	0.2	0.1	0.1	0.0	0.1	0.0	0.0	0.0	0.1
FAM182A	0.2	0.1	0.1	0.0	0.0	0.0	0.1	0.0	0.1
WBP2NL	0.2	0.0	0.1	0.0	0.0	0.0	0.1	0.0	0.1
PRKCQ	0.1	0.1	0.1	0.0	0.0	0.0	0.1	0.0	0.2
CCDC87	0.2	0.0	0.0	0.0	0.0	0.0	0.1	0.0	0.1
LINC00624	0.2	0.0	0.1	0.0	0.0	0.0	0.1	0.0	0.1
LINC00273	0.2	0.0	0.1	0.0	0.0	0.0	0.2	0.0	0.1
SLC9A3	0.1	0.1	0.0	0.0	0.1	0.0	0.1	0.0	0.1
LOC339505	0.2	0.0	0.0	0.0	0.0	0.1	0.1	0.0	0.0
MMRN2	0.2	0.1	0.1	0.0	0.0	0.0	0.1	0.0	0.1
SIPA1L2	0.1	0.0	0.1	0.0	0.0	0.0	0.1	0.0	0.1
SEMA6D	0.2	0.1	0.1	0.0	0.1	0.1	0.1	0.0	0.0
CLEC18C	0.2	0.0	0.2	0.0	0.0	0.0	0.1	0.0	0.0
KRT83	0.2	0.1	0.0	0.0	0.0	0.0	0.2	0.0	0.1
SCN2A	0.2	0.1	0.1	0.0	0.0	0.0	0.0	0.0	0.0
SNX32	0.1	0.1	0.1	0.0	0.0	0.0	0.1	0.0	0.1
GALNT12	0.1	0.0	0.1	0.0	0.0	0.1	0.1	0.0	0.0
RHBDL2	0.3	0.0	0.1	0.0	0.0	0.0	0.0	0.0	0.0
HCP5	0.1	0.0	0.1	0.0	0.0	0.0	0.1	0.0	0.1
EPHA1	0.2	0.0	0.0	0.0	0.0	0.0	0.2	0.0	0.1
LOC101927229	0.2	0.0	0.1	0.0	0.0	0.1	0.1	0.0	0.1
LOC440416	0.1	0.1	0.1	0.0	0.0	0.0	0.1	0.0	0.1
PCDHB5	0.2	0.1	0.2	0.0	0.0	0.1	0.0	0.0	0.0
IMPG2	0.2	0.0	0.1	0.0	0.0	0.0	0.0	0.0	0.1
LOC100506801	0.2	0.0	0.1	0.0	0.0	0.0	0.1	0.0	0.1
LOC100507600	0.1	0.0	0.1	0.0	0.0	0.0	0.2	0.0	0.1
H19	0.1	0.0	0.1	0.0	0.1	0.0	0.1	0.0	0.1
COL4A6	0.2	0.0	0.1	0.0	0.0	0.1	0.1	0.0	0.0
BAIAP3	0.2	0.1	0.1	0.0	0.0	0.0	0.0	0.0	0.1
RGPD3	0.1	0.0	0.2	0.0	0.0	0.0	0.1	0.0	0.1
DKFZP434F142	0.1	0.1	0.1	0.0	0.1	0.0	0.0	0.0	0.0
LARGE	0.1	0.0	0.1	0.0	0.0	0.1	0.1	0.0	0.1
SPON2	0.2	0.1	0.0	0.0	0.1	0.1	0.0	0.1	0.1
MAGIX	0.1	0.1	0.1	0.0	0.0	0.1	0.1	0.1	0.0
LOC101928714	0.1	0.0	0.1	0.0	0.0	0.0	0.2	0.0	0.0
FAM95B1	0.2	0.0	0.1	0.0	0.0	0.1	0.1	0.0	0.0
FOXE1	0.1	0.1	0.0	0.0	0.0	0.0	0.1	0.0	0.1
FAT3	0.1	0.0	0.1	0.0	0.0	0.1	0.1	0.0	0.1
LINC00032	0.2	0.0	0.1	0.0	0.0	0.0	0.1	0.0	0.0

SYNPO2		0.2	0.1	0.1	0.0	0.1	0.0	0.0	0.0	0.0
GRIK1		0.2	0.1	0.1	0.0	0.0	0.0	0.1	0.0	0.1
GRM5-AS1		0.1	0.0	0.0	0.0	0.0	0.0	0.2	0.0	0.1
MAPK15		0.1	0.1	0.1	0.0	0.1	0.0	0.0	0.0	0.1
LRFN1		0.1	0.0	0.1	0.1	0.0	0.1	0.1	0.0	0.1
ACOT11		0.1	0.0	0.1	0.0	0.1	0.0	0.2	0.0	0.0
ESYT3		0.2	0.0	0.1	0.0	0.0	0.0	0.1	0.0	0.1
GOLGA6L3		0.2	0.1	0.1	0.0	0.0	0.0	0.0	0.0	0.0
UPK1A		0.1	0.0	0.1	0.0	0.0	0.0	0.1	0.0	0.1
LINC00539		0.0	0.1	0.1	0.0	0.0	0.0	0.1	0.0	0.1
SLC22A23		0.1	0.0	0.1	0.0	0.0	0.0	0.1	0.0	0.1
LOC284632		0.2	0.0	0.1	0.0	0.0	0.0	0.0	0.0	0.1
MIAT		0.1	0.0	0.1	0.0	0.0	0.0	0.1	0.0	0.1
EPN3		0.1	0.1	0.1	0.0	0.0	0.0	0.1	0.0	0.0
LOC101928483		0.1	0.1	0.1	0.0	0.1	0.1	0.0	0.0	0.0
MXRA8		0.1	0.1	0.0	0.0	0.0	0.1	0.0	0.0	0.0
	44808.0	0.2	0.1	0.0	0.0	0.0	0.0	0.1	0.0	0.1
IL3RA		0.2	0.1	0.1	0.0	0.0	0.0	0.1	0.0	0.0
IL3RA		0.2	0.1	0.1	0.0	0.0	0.0	0.1	0.0	0.0
PGBD3		0.2	0.0	0.1	0.0	0.0	0.0	0.1	0.0	0.1
HHIPL1		0.1	0.1	0.0	0.0	0.0	0.0	0.1	0.0	0.1
ABCC9		0.2	0.0	0.1	0.0	0.0	0.0	0.1	0.0	0.0
MATN4		0.2	0.1	0.0	0.0	0.0	0.0	0.1	0.0	0.0
SLC23A1		0.2	0.0	0.1	0.0	0.0	0.0	0.1	0.0	0.0
LOC100132891		0.1	0.0	0.1	0.0	0.0	0.0	0.1	0.0	0.1
LOC100128885		0.2	0.0	0.1	0.0	0.0	0.0	0.1	0.0	0.1
LOC101927156		0.2	0.0	0.1	0.0	0.0	0.0	0.1	0.0	0.0
LOC284661		0.2	0.0	0.1	0.0	0.1	0.0	0.1	0.0	0.0
XDH		0.2	0.0	0.0	0.0	0.0	0.0	0.1	0.0	0.1
COL5A2		0.1	0.1	0.0	0.0	0.0	0.0	0.1	0.0	0.1
RGPD1		0.1	0.1	0.0	0.0	0.0	0.0	0.1	0.0	0.0
C3orf49		0.2	0.0	0.1	0.0	0.0	0.0	0.1	0.0	0.0
C1orf170		0.1	0.0	0.0	0.0	0.0	0.1	0.0	0.0	0.1
ZNF610		0.1	0.1	0.1	0.0	0.0	0.0	0.0	0.1	0.1
CYP2E1		0.1	0.1	0.1	0.0	0.0	0.0	0.0	0.0	0.1
CELF2-AS1		0.1	0.0	0.1	0.0	0.0	0.0	0.1	0.0	0.1
KDM5D		0.2	0.0	0.0	0.0	0.0	0.0	0.1	0.0	0.1
RLTPR		0.2	0.0	0.1	0.0	0.0	0.0	0.1	0.0	0.1
POLN		0.1	0.0	0.0	0.0	0.0	0.0	0.1	0.0	0.1
MEIS1-AS3		0.1	0.0	0.1	0.0	0.0	0.1	0.1	0.0	0.0
RGPD4		0.2	0.0	0.0	0.0	0.0	0.0	0.1	0.0	0.1
MST1L		0.1	0.1	0.1	0.0	0.0	0.0	0.1	0.0	0.1
ANO9		0.1	0.1	0.1	0.0	0.1	0.0	0.1	0.0	0.0
LOC101929719		0.1	0.0	0.1	0.0	0.0	0.0	0.1	0.0	0.1
LILRA6		0.1	0.0	0.1	0.0	0.0	0.1	0.1	0.0	0.0
CBWD7		0.1	0.1	0.0	0.0	0.0	0.0	0.1	0.0	0.1
PHYHIP		0.2	0.0	0.1	0.0	0.0	0.1	0.0	0.0	0.0
TTN		0.2	0.0	0.1	0.0	0.0	0.0	0.1	0.0	0.0
GPR45		0.1	0.0	0.1	0.0	0.0	0.0	0.0	0.0	0.1
DOCK3		0.1	0.0	0.1	0.0	0.0	0.0	0.1	0.0	0.1
IGSF10		0.1	0.0	0.1	0.0	0.1	0.0	0.0	0.0	0.0
SLC1A7		0.1	0.0	0.1	0.0	0.0	0.0	0.0	0.0	0.1
ANKRD20A1		0.1	0.0	0.1	0.0	0.0	0.0	0.1	0.0	0.1
PMFBP1		0.1	0.0	0.1	0.0	0.0	0.1	0.0	0.0	0.1
LOC100505658		0.1	0.0	0.0	0.0	0.0	0.0	0.1	0.0	0.0

KIF26B	0.1	0.0	0.1	0.0	0.0	0.0	0.1	0.0	0.1
SEZ6	0.1	0.0	0.1	0.0	0.0	0.1	0.1	0.0	0.1
KIF6	0.1	0.1	0.0	0.0	0.0	0.1	0.1	0.0	0.0
LOC340512	0.1	0.0	0.1	0.0	0.1	0.0	0.0	0.0	0.0
SLC45A1	0.1	0.1	0.1	0.0	0.0	0.0	0.1	0.0	0.1
FLJ45079	0.2	0.0	0.1	0.0	0.0	0.0	0.1	0.0	0.0
CLEC18B	0.1	0.0	0.1	0.0	0.0	0.0	0.0	0.0	0.1
DYX1C1	0.1	0.0	0.1	0.0	0.0	0.1	0.1	0.1	0.1
LOC101927501	0.1	0.0	0.1	0.0	0.0	0.0	0.1	0.0	0.0
DRP2	0.1	0.1	0.0	0.0	0.0	0.0	0.1	0.0	0.0
CACNA1A	0.2	0.0	0.0	0.0	0.0	0.0	0.1	0.0	0.0
STAG3	0.1	0.0	0.1	0.0	0.0	0.0	0.1	0.0	0.1
PDZD9	0.1	0.1	0.1	0.0	0.0	0.0	0.0	0.0	0.1
CAND2	0.1	0.1	0.1	0.0	0.0	0.0	0.1	0.0	0.1
EFR3B	0.1	0.1	0.0	0.0	0.0	0.0	0.1	0.0	0.1
TEX14	0.2	0.0	0.0	0.0	0.0	0.0	0.1	0.0	0.0
FER1L5	0.1	0.0	0.0	0.0	0.0	0.0	0.1	0.0	0.1
STRC	0.1	0.0	0.1	0.0	0.0	0.0	0.1	0.0	0.0
LOC101929653	0.1	0.0	0.0	0.0	0.0	0.0	0.1	0.0	0.1
ADAMTS5	0.1	0.0	0.0	0.0	0.0	0.0	0.1	0.0	0.0
GRM5	0.0	0.0	0.0	0.0	0.0	0.0	0.1	0.0	0.1
LINC00536	0.1	0.0	0.0	0.0	0.0	0.0	0.1	0.0	0.1
CCDC85A	0.1	0.1	0.0	0.0	0.0	0.1	0.1	0.0	0.0
LOC100996490	0.1	0.0	0.1	0.0	0.0	0.0	0.1	0.0	0.0
PSG1	0.1	0.0	0.1	0.0	0.0	0.0	0.1	0.0	0.0
GPR179	0.1	0.0	0.1	0.0	0.0	0.0	0.1	0.0	0.0
LOC101927450	0.2	0.0	0.0	0.0	0.0	0.0	0.1	0.0	0.0
TNS4	0.1	0.0	0.1	0.0	0.0	0.0	0.1	0.0	0.1
SH2D3C	0.1	0.1	0.1	0.0	0.0	0.0	0.1	0.0	0.0
FAM131B	0.1	0.1	0.0	0.0	0.0	0.0	0.1	0.0	0.1
RASGRF1	0.1	0.0	0.0	0.0	0.0	0.1	0.1	0.0	0.0
LOC101927272	0.1	0.0	0.1	0.0	0.0	0.0	0.1	0.0	0.0
PRDM1	0.1	0.0	0.0	0.0	0.0	0.0	0.1	0.0	0.0
PREX1	0.1	0.0	0.1	0.0	0.0	0.0	0.1	0.0	0.1
SVEP1	0.1	0.0	0.1	0.0	0.0	0.0	0.0	0.0	0.1
SRGAP3	0.1	0.0	0.1	0.0	0.0	0.0	0.1	0.0	0.0
ABCA4	0.1	0.0	0.1	0.0	0.0	0.0	0.1	0.0	0.0
FLJ37035	0.1	0.1	0.1	0.0	0.0	0.0	0.0	0.0	0.0
ANKRD20A3	0.1	0.0	0.1	0.0	0.0	0.0	0.1	0.0	0.1
FBP2	0.2	0.0	0.0	0.0	0.0	0.0	0.1	0.0	0.0
LOC101927754	0.1	0.0	0.1	0.0	0.0	0.0	0.2	0.0	0.0
PDZD3	0.1	0.1	0.0	0.0	0.0	0.0	0.0	0.0	0.0
RGPD2	0.1	0.0	0.0	0.0	0.0	0.0	0.1	0.0	0.0
ANKRD20A2	0.1	0.1	0.1	0.0	0.0	0.0	0.1	0.0	0.0
LOC100507140	0.0	0.0	0.0	0.0	0.0	0.1	0.0	0.0	0.1
LINC00837	0.1	0.0	0.1	0.0	0.0	0.0	0.1	0.0	0.0
LOC100132062	0.2	0.0	0.0	0.1	0.0	0.0	0.0	0.0	0.0
PCSK6	0.1	0.0	0.1	0.0	0.0	0.0	0.0	0.0	0.0
PKDREJ	0.1	0.0	0.1	0.0	0.0	0.0	0.1	0.0	0.0
DTHD1	0.1	0.0	0.1	0.0	0.0	0.0	0.1	0.0	0.0
ELN	0.0	0.0	0.1	0.1	0.0	0.0	0.0	0.0	0.0
FHDC1	0.0	0.0	0.1	0.0	0.0	0.0	0.1	0.0	0.0
LOC643542	0.1	0.0	0.0	0.0	0.0	0.0	0.1	0.0	0.0
ABCA13	0.1	0.0	0.0	0.0	0.0	0.0	0.0	0.0	0.0
LOC101928072	0.1	0.1	0.0	0.0	0.0	0.0	0.0	0.0	0.1

CMYA5	0.1	0.0	0.1	0.0	0.0	0.0	0.1	0.0	0.0
LOC101927407	0.1	0.0	0.0	0.0	0.0	0.0	0.0	0.0	0.1
LINC00664	0.1	0.0	0.0	0.0	0.0	0.0	0.1	0.0	0.0
SORL1	0.1	0.0	0.0	0.0	0.0	0.0	0.1	0.0	0.1
LOC101929445	0.1	0.0	0.0	0.0	0.0	0.0	0.1	0.0	0.0
PTGES3L-AARSD1	0.1	0.1	0.0	0.0	0.0	0.1	0.0	0.0	0.0
CHD5	0.1	0.0	0.0	0.0	0.0	0.0	0.1	0.0	0.1
LOC101928266	0.1	0.0	0.0	0.0	0.0	0.0	0.0	0.0	0.1
LY75-CD302	0.0	0.1	0.0	0.0	0.0	0.0	0.1	0.0	0.0
LOC101929116	0.1	0.0	0.0	0.0	0.0	0.0	0.1	0.0	0.1
PTCHD2	0.1	0.0	0.1	0.0	0.0	0.0	0.0	0.0	0.0
KCNQ4	0.1	0.0	0.0	0.0	0.0	0.0	0.1	0.0	0.1
ITGAX	0.1	0.1	0.1	0.0	0.0	0.0	0.0	0.0	0.0
LINC00689	0.1	0.0	0.1	0.0	0.0	0.0	0.1	0.0	0.0
KIAA1614	0.1	0.0	0.0	0.0	0.0	0.0	0.1	0.0	0.0
CCDC180	0.1	0.0	0.0	0.0	0.0	0.1	0.0	0.0	0.1
OTUD7A	0.1	0.0	0.1	0.0	0.0	0.0	0.0	0.0	0.0
LOC101927183	0.1	0.0	0.1	0.0	0.0	0.0	0.0	0.0	0.0
STON1-GTF2A1L	0.1	0.0	0.0	0.0	0.0	0.0	0.0	0.0	0.0
C2orf16	0.1	0.0	0.0	0.0	0.0	0.0	0.0	0.0	0.0
TRIL	0.1	0.0	0.1	0.0	0.0	0.0	0.0	0.0	0.0
ANKRD24	0.1	0.0	0.1	0.0	0.0	0.0	0.0	0.0	0.0
CDHR3	0.0	0.0	0.0	0.0	0.0	0.0	0.1	0.0	0.0
COL20A1	0.1	0.0	0.1	0.0	0.0	0.0	0.0	0.0	0.0
SEPT5-GP1BB	0.1	0.0	0.1	0.0	0.0	0.0	0.0	0.0	0.0
CDC42BPG	0.1	0.0	0.0	0.0	0.0	0.0	0.1	0.0	0.0
TDRD6	0.1	0.0	0.1	0.0	0.0	0.0	0.0	0.0	0.0
POTEF	0.1	0.0	0.0	0.0	0.0	0.0	0.0	0.0	0.0
LOC100129213	0.1	0.0	0.1	0.0	0.0	0.0	0.1	0.0	0.0
DLEC1	0.1	0.0	0.1	0.0	0.0	0.0	0.0	0.0	0.0
MUC2	0.1	0.0	0.1	0.0	0.0	0.0	0.0	0.0	0.0
MDGA1	0.1	0.0	0.0	0.0	0.0	0.0	0.0	0.0	0.1
IGFN1	0.0	0.0	0.0	0.0	0.0	0.0	0.1	0.0	0.1
GPR64	0.1	0.0	0.0	0.0	0.0	0.0	0.0	0.0	0.0
CCDC177	0.0	0.1	0.0	0.0	0.0	0.0	0.0	0.0	0.0
ELMO1	0.0	0.0	0.0	0.0	0.0	0.0	0.0	0.0	0.0
PCDH11X	0.1	0.0	0.0	0.0	0.0	0.0	0.0	0.0	0.0
TRPV3	0.1	0.0	0.0	0.0	0.0	0.0	0.0	0.0	0.1
EPPK1	0.1	0.0	0.1	0.0	0.0	0.0	0.1	0.0	0.0
KRTAP5-AS1	0.1	0.0	0.0	0.0	0.0	0.0	0.0	0.0	0.0
FRMPD3	0.0	0.0	0.0	0.0	0.0	0.0	0.1	0.0	0.0
DNAH17	0.1	0.0	0.0	0.0	0.0	0.0	0.0	0.0	0.0
LOC101927267	0.1	0.0	0.0	0.0	0.0	0.0	0.0	0.0	0.0
LINC00426	0.1	0.0	0.0	0.0	0.0	0.0	0.0	0.0	0.0
LOC101928559	0.1	0.0	0.0	0.0	0.0	0.0	0.1	0.0	0.0
NLRP6	0.1	0.0	0.0	0.0	0.0	0.0	0.0	0.0	0.0
IGF2-AS	0.0	0.0	0.0	0.0	0.0	0.0	0.1	0.0	0.1
SCN8A	0.0	0.0	0.0	0.0	0.0	0.0	0.1	0.0	0.0
LINC00840	0.0	0.0	0.0	0.0	0.0	0.0	0.1	0.0	0.0
PXDNL	0.1	0.0	0.0	0.0	0.0	0.0	0.0	0.0	0.0
INMT-FAM188B	0.0	0.1	0.0	0.0	0.0	0.0	0.0	0.0	0.0
LOC440934	0.1	0.0	0.0	0.0	0.0	0.0	0.0	0.0	0.0
IGSF1	0.1	0.0	0.0	0.0	0.0	0.0	0.0	0.0	0.1
FOXG1	0.1	0.0	0.0	0.0	0.0	0.0	0.0	0.0	0.0
FREM2	0.0	0.0	0.0	0.0	0.0	0.0	0.1	0.0	0.0

OTOGL	0.1	0.0	0.0	0.0	0.0	0.0	0.0	0.0	0.0
STAB2	0.1	0.0	0.0	0.0	0.0	0.0	0.0	0.0	0.0
LOC440905	0.1	0.0	0.0	0.0	0.0	0.0	0.0	0.0	0.0
PKHD1	0.1	0.0	0.0	0.0	0.0	0.0	0.0	0.0	0.0
CCDC169-SOHLH2	0.1	0.0	0.0	0.0	0.0	0.0	0.0	0.0	0.0
PCDHA12	0.1	0.0	0.0	0.0	0.0	0.0	0.0	0.0	0.0
LOC100996414	0.0	0.0	0.0	0.0	0.1	0.0	0.0	0.0	0.0
ZNF469	0.0	0.0	0.0	0.0	0.0	0.0	0.0	0.0	0.0
GOLGA8J	0.1	0.0	0.0	0.0	0.0	0.0	0.0	0.0	0.0
DCHS2	0.0	0.0	0.0	0.0	0.0	0.0	0.0	0.0	0.0
CGNL1	0.1	0.0	0.0	0.0	0.0	0.0	0.0	0.0	0.0
DNAH10	0.0	0.0	0.0	0.0	0.0	0.0	0.0	0.0	0.0
MAGEA2B	0.0	0.0	0.0	0.0	0.0	0.0	0.0	0.0	0.0
LOC400553	0.0	0.0	0.0	0.0	0.0	0.0	0.0	0.0	0.0
LPA	0.0	0.0	0.0	0.0	0.0	0.0	0.0	0.0	0.0
MUC4	0.0	0.0	0.0	0.0	0.0	0.0	0.0	0.0	0.0
DNAH6	0.0	0.0	0.0	0.0	0.0	0.0	0.0	0.0	0.0
FAT2	0.0	0.0	0.0	0.0	0.0	0.0	0.0	0.0	0.0
DCC	0.0	0.0	0.0	0.0	0.0	0.0	0.0	0.0	0.0
RYR3	0.0	0.0	0.0	0.0	0.0	0.0	0.0	0.0	0.0
C12orf55	0.0	0.0	0.0	0.0	0.0	0.0	0.0	0.0	0.0
RYR1	0.0	0.0	0.0	0.0	0.0	0.0	0.0	0.0	0.0
PCDHA1	0.0	0.0	0.0	0.0	0.0	0.0	0.0	0.0	0.0
P2RY11	0.0	6.1	0.0	1.9	2.5	3.5	7.3	1.4	0.0
SNORD26	1.6	0.0	1.8	2.4	0.5	2.3	2.0	1.1	1.9
RNU6-33P	0.1	0.0	0.0	0.0	0.0	7.5	0.0	0.5	0.0
TRNP	0.0	2.3	0.8	0.6	0.5	0.8	1.5	0.4	1.2
SNORD14E	0.0	1.1	0.0	1.0	0.0	2.5	1.1	0.0	1.2
LOC101928602	0.6	0.0	0.6	0.2	0.0	0.4	0.5	0.1	0.5
LOC101929100	0.9	0.0	0.5	0.0	0.1	0.3	0.3	0.0	0.2
LOC554223	0.7	0.1	0.0	0.2	0.3	0.0	0.3	0.1	0.0
C20orf197	0.2	0.0	0.3	0.1	0.1	0.2	0.2	0.0	0.3
LOC101928383	0.2	0.0	0.4	0.0	0.1	0.1	0.2	0.0	0.1
TPRG1-AS2	0.4	0.0	0.1	0.0	0.0	0.1	0.2	0.1	0.1
LINC00028	0.4	0.0	0.1	0.0	0.0	0.0	0.3	0.0	0.1
LOC101928970	0.3	0.0	0.1	0.0	0.1	0.0	0.3	0.0	0.1
OR7E24	0.3	0.0	0.1	0.0	0.1	0.1	0.1	0.1	0.1
LOC101928814	0.4	0.0	0.1	0.0	0.0	0.1	0.1	0.0	0.1
PARM1	0.3	0.1	0.0	0.0	0.1	0.0	0.2	0.1	0.0
LINC00226	0.3	0.0	0.0	0.0	0.1	0.0	0.2	0.0	0.2
HOXB7	0.1	0.0	0.0	0.0	0.1	0.0	0.2	0.0	0.2
P2RY4	0.2	0.0	0.1	0.1	0.0	0.1	0.1	0.0	0.1
SOX7	0.1	0.0	0.1	0.0	0.0	0.1	0.1	0.1	0.1
WDR31	0.3	0.0	0.0	0.0	0.0	0.1	0.1	0.0	0.1
APOBEC4	0.2	0.0	0.0	0.0	0.1	0.1	0.0	0.0	0.1
PRDM12	0.1	0.1	0.0	0.0	0.0	0.0	0.1	0.0	0.1
RASD2	0.1	0.0	0.0	0.0	0.1	0.1	0.1	0.0	0.1
FOXF1	0.0	0.1	0.1	0.1	0.1	0.1	0.0	0.0	0.0
POU4F1	0.0	0.1	0.0	0.1	0.1	0.0	0.0	0.0	0.0
DOC2B	0.1	0.0	0.0	0.0	0.0	0.1	0.1	0.0	0.0
BCAR4	0.1	0.0	0.0	0.0	0.1	0.0	0.1	0.0	0.0
CD6	0.0	0.0	0.0	0.0	0.0	0.0	0.1	0.0	0.0
LOC101927523	0.2	0.0	0.0	0.0	0.0	0.0	0.0	0.0	0.0
LOC101926894	0.0	0.0	0.0	0.1	0.0	0.0	0.2	0.0	0.0
EYA1	0.0	0.0	0.0	0.0	0.0	0.0	0.0	0.0	0.0

LOC285819	0.0	0.0	0.0	0.0	0.0	0.0	0.0	0.0	0.0
TRIM50	0.0	0.0	0.0	0.0	0.0	0.0	0.1	0.0	0.0
LOC340113	0.0	0.0	0.0	0.0	0.0	0.0	0.1	0.0	0.0
PSG3	0.0	0.0	0.0	0.0	0.0	0.0	0.1	0.0	0.1
FLJ33534	0.0	0.0	0.0	0.0	0.0	0.0	0.0	0.0	0.0
MAGI2	0.0	0.0	0.0	0.0	0.0	0.0	0.0	0.0	0.0
RPS10-NUDT3	219.3	0.1	0.1	5.0	28.1	0.0	0.1	0.1	53.4
NDUFC2	24.4	10.4	17.6	0.0	3.1	11.0	23.3	4.0	16.9
SNORD22	9.4	3.4	6.1	0.0	1.1	1.4	14.2	0.2	3.2
SNORA81	10.1	7.9	8.4	0.0	1.5	0.8	2.1	0.3	1.2
CDC42SE2	6.4	2.0	3.8	0.0	1.0	1.7	5.8	0.6	4.5
C15orf38	4.8	1.8	0.0	0.0	2.0	2.4	3.2	0.7	2.9
SNORA13	4.5	2.6	2.2	0.0	1.4	0.6	2.2	0.4	3.5
SNORA32	5.9	1.2	3.8	0.0	0.5	1.5	1.8	0.5	2.0
SNORD8	6.2	2.0	1.6	0.0	0.3	0.3	2.0	0.2	3.5
LOC101928021	4.6	1.7	2.5	0.0	0.3	0.7	3.0	0.3	2.9
SMIM11	4.3	2.4	2.8	0.0	1.1	0.4	1.2	0.7	2.2
RPL39L	4.7	1.7	1.8	0.0	0.4	0.4	2.7	0.3	1.7
C15orf38-AP3S2	4.7	1.7	2.9	0.8	0.0	0.7	1.3	0.0	1.2
LOC101929546	3.3	0.6	2.0	0.5	0.0	0.5	4.9	0.3	0.7
TRNK	0.6	1.5	2.3	0.0	0.5	1.4	4.2	0.8	1.6
TTC33	3.4	1.7	1.7	0.0	0.4	0.6	2.5	0.5	1.9
TMEM126A	2.5	1.1	2.6	0.0	0.7	0.5	2.3	0.3	2.2
SNORD9	3.3	1.1	1.8	0.0	0.7	0.3	2.1	0.5	1.4
SNORA39	4.4	1.0	1.0	0.8	0.0	1.4	1.4	0.6	0.4
SLC35A3	2.4	1.2	1.1	0.0	0.4	0.3	2.8	0.3	2.3
SNORD88A	2.2	2.1	0.6	0.9	0.0	0.3	2.4	0.3	1.5
DCK	2.8	0.9	1.8	0.0	0.5	0.7	2.0	0.2	1.4
GOLT1B	3.0	0.8	1.8	0.0	0.2	0.3	2.8	0.3	1.0
SNORA11B	1.9	1.8	1.7	0.0	0.7	1.1	1.3	0.4	1.3
CCL26	3.0	1.9	2.7	0.0	0.1	0.0	0.9	0.2	1.0
ASAH2B	2.4	0.8	1.8	0.0	0.3	0.6	1.9	0.2	1.5
RNY4	3.7	1.3	1.4	0.6	0.0	0.0	1.5	0.4	0.4
FAM138E	5.0	0.5	1.3	0.1	0.7	0.0	1.0	0.1	0.5
SNORA42	3.3	1.7	1.6	0.0	0.5	0.5	0.5	0.2	0.4
SNORA31	3.2	1.1	2.1	0.2	0.0	0.4	1.2	0.2	0.4
SLC41A2	2.8	0.9	1.0	0.0	0.3	0.3	1.9	0.2	1.3
MILR1	2.6	0.8	1.0	0.0	0.1	0.6	1.9	0.4	1.0
ARC	2.8	0.8	1.8	0.0	0.1	0.0	1.6	0.2	0.8
CPQ	2.7	1.1	1.6	0.0	0.3	0.4	1.0	0.2	0.9
SLC25A15	1.3	0.8	1.1	0.0	0.5	0.6	2.1	0.1	1.6
RMDN2	2.3	0.7	1.4	0.0	0.2	0.2	1.5	0.2	1.4
URGCP-MRPS24	2.0	0.5	1.4	0.0	0.8	0.8	1.6	0.2	0.5
SNORD44	0.7	1.7	0.4	0.0	1.2	0.5	1.5	0.4	1.4
SNORA25	3.4	0.0	1.8	0.0	0.3	0.3	1.2	0.0	0.7
DNAJC12	1.9	1.2	0.8	0.0	0.3	0.2	1.6	0.1	1.5
CENPK	1.4	0.7	0.8	0.0	0.3	0.3	1.8	0.5	1.8
MAL2	2.6	0.8	1.6	0.1	0.0	0.2	1.3	0.2	0.8
SNORD58A	1.2	0.4	0.4	0.0	0.6	0.9	0.9	0.4	2.6
SCARNA3	2.2	0.6	1.1	0.7	0.0	0.4	1.3	0.2	0.8
SNORD35B	2.7	0.6	1.4	0.0	0.0	0.0	2.2	0.0	0.5
ZNF114	2.1	0.7	0.8	0.0	0.3	0.4	1.4	0.3	1.2
TMEM170B	2.2	0.5	0.7	0.0	0.4	0.1	1.8	0.3	1.2
ABHD13	1.7	0.6	1.2	0.0	0.2	0.4	1.4	0.2	1.4
PRSS1	2.5	1.3	1.7	0.1	0.0	0.3	0.4	0.0	0.5

GALNT4	1.9	0.9	1.3	0.1	0.0	0.6	0.9	0.1	0.8
LOC644634	1.3	0.6	0.8	0.0	0.3	0.5	1.1	0.2	1.1
FAM229B	2.0	0.8	1.1	0.0	0.3	0.3	0.4	0.2	0.9
LOC100289061	1.6	0.4	0.5	0.0	0.2	0.3	1.8	0.1	0.8
LY96	1.4	0.4	0.9	0.0	0.2	0.3	1.3	0.2	1.2
NINJ2	2.4	0.9	1.0	0.2	0.0	0.3	0.6	0.1	0.4
MFSD8	1.3	0.8	0.9	0.0	0.3	0.2	1.2	0.1	0.8
IMMP1L	2.2	0.6	0.8	0.0	0.2	0.3	0.9	0.1	0.5
PLGRKT	1.0	0.4	0.8	0.0	0.6	0.5	0.8	0.3	1.1
ZNF225	1.9	0.8	0.9	0.0	0.1	0.1	0.7	0.1	0.9
FASTKD3	1.5	0.6	0.8	0.0	0.3	0.4	1.1	0.1	0.8
SNORA6	1.1	1.0	0.8	0.4	0.0	0.6	0.8	0.2	0.7
C1orf21	1.3	0.6	0.7	0.0	0.4	0.2	1.0	0.3	1.1
SH3BGRL2	1.1	0.6	1.0	0.0	0.3	0.2	1.0	0.2	1.0
NUDT12	1.5	0.7	0.9	0.0	0.3	0.4	1.1	0.0	0.5
LOC100506844	1.5	0.6	0.7	0.0	0.4	0.6	0.7	0.3	0.5
LINC00882	1.4	0.4	0.5	0.0	0.1	0.0	1.8	0.1	0.7
CEMP1	1.4	0.5	0.8	0.0	0.3	0.4	0.8	0.1	0.7
DTWD1	1.1	0.5	0.7	0.0	0.2	0.4	0.9	0.2	0.9
IRAK4	1.4	0.4	0.8	0.0	0.1	0.2	1.1	0.1	0.9
GABRR2	2.2	0.5	0.9	0.0	0.2	0.1	0.5	0.1	0.4
MORN2	1.1	1.1	1.5	0.0	0.2	0.2	0.2	0.2	0.4
LMBRD2	1.4	0.4	0.7	0.0	0.1	0.4	1.0	0.1	0.8
AP1S3	1.2	0.5	0.5	0.0	0.4	0.3	0.9	0.1	1.0
ZNF32-AS1	1.4	0.5	1.0	0.0	0.5	0.4	0.4	0.1	0.6
EPCAM	1.3	0.3	0.8	0.0	0.1	0.1	0.9	0.1	1.1
HOXC6	1.1	0.5	0.6	0.0	0.1	0.4	1.0	0.2	0.7
CRYGS	1.5	0.3	1.0	0.1	0.0	0.4	1.1	0.1	0.1
CEP19	1.5	0.4	0.6	0.0	0.1	0.2	1.4	0.1	0.4
CENPW	0.8	0.4	0.6	0.0	0.3	0.7	1.1	0.2	0.6
AP4S1	1.2	0.1	0.6	0.0	0.0	0.2	1.1	0.1	1.3
ASB3	1.0	0.4	1.4	0.0	0.0	0.3	1.5	0.0	0.0
LOC101927789	1.2	0.9	0.9	0.0	0.4	0.3	0.4	0.1	0.3
ATP6V1E2	1.4	0.6	0.4	0.1	0.1	0.0	0.8	0.0	1.0
LOC101927164	1.4	0.6	0.7	0.0	0.3	0.3	0.5	0.1	0.6
LYG1	1.7	0.5	0.8	0.0	0.2	0.1	0.8	0.1	0.2
VAMP1	1.3	0.3	1.0	0.0	0.1	0.2	0.5	0.2	0.8
SNORD119	0.6	0.3	0.4	0.2	0.4	0.0	0.8	0.3	1.5
SERPINB7	0.7	0.4	0.8	0.0	0.1	0.3	1.0	0.2	0.8
C5orf54	1.1	0.4	0.6	0.0	0.1	0.2	1.1	0.1	0.7
MTERFD3	1.3	0.4	0.8	0.0	0.1	0.2	0.8	0.0	0.6
FABP3	1.2	0.8	0.7	0.0	0.0	0.4	0.5	0.1	0.6
ACBD7	0.5	0.2	0.5	0.0	0.3	0.1	1.3	0.4	0.8
TRNAL11	0.7	0.3	0.2	0.0	0.5	0.2	1.5	0.4	0.5
TRNAL13	0.7	0.3	0.2	0.0	0.5	0.2	1.5	0.4	0.5
LOC101929496	1.6	0.4	0.6	0.0	0.0	0.2	0.0	0.0	1.3
KISS1R	0.1	1.1	0.7	1.0	0.1	0.0	0.5	0.6	0.1
TP53INP1	1.5	0.7	0.8	0.0	0.1	0.3	0.3	0.0	0.3
LOC101929205	1.3	0.3	0.5	0.0	0.1	0.1	1.1	0.1	0.6
CLHC1	1.5	0.3	0.6	0.0	0.1	0.2	0.8	0.0	0.5
SPATA25	1.8	0.2	0.5	0.0	0.3	0.2	0.2	0.0	0.6
LOC101928943	1.5	0.5	0.7	0.0	0.1	0.2	0.4	0.1	0.4
LOC101929200	1.1	0.3	0.4	0.0	0.2	0.3	0.9	0.1	0.4
CENPQ	0.6	0.2	0.6	0.0	0.1	0.1	1.3	0.1	0.6
ANG	1.0	0.4	0.5	0.0	0.1	0.2	0.8	0.2	0.6

GSTO2	1.5	0.3	0.8	0.0	0.3	0.3	0.3	0.0	0.2
LOC107987361	1.2	0.1	0.5	0.0	0.3	0.2	0.8	0.1	0.5
FAM228B	1.0	0.6	0.7	0.0	0.2	0.2	0.5	0.1	0.5
SPDEF	1.3	0.9	0.9	0.1	0.0	0.1	0.1	0.1	0.0
C2orf88	1.0	0.2	0.6	0.1	0.0	0.2	0.5	0.0	1.0
LOC101928156	1.2	0.2	0.7	0.0	0.2	0.3	0.4	0.1	0.5
QRFP	1.3	0.2	0.6	0.0	0.1	0.1	0.7	0.1	0.4
LOC101927262	0.6	0.3	0.7	0.0	0.0	0.0	0.8	0.2	0.8
RAB9B	0.9	0.1	0.4	0.1	0.0	0.2	1.2	0.0	0.5
TMEM217	0.9	0.4	0.5	0.0	0.1	0.2	0.7	0.1	0.6
TRNAV19	0.4	0.2	0.7	0.1	0.2	0.0	1.0	0.1	0.6
TRNAV11	0.4	0.2	0.7	0.1	0.2	0.0	1.0	0.1	0.6
CAB39L	1.0	0.4	0.2	0.0	0.1	0.2	0.8	0.1	0.6
ZNF670	1.1	0.3	0.6	0.0	0.1	0.1	0.4	0.0	0.7
SNORD124	1.1	0.4	0.7	0.0	0.0	0.3	0.6	0.3	0.0
SDR16C5	1.2	0.5	0.5	0.0	0.2	0.1	0.4	0.0	0.4
ZNF430	0.9	0.3	0.6	0.0	0.1	0.1	0.6	0.1	0.6
HPGD	1.1	0.6	0.7	0.0	0.2	0.1	0.2	0.1	0.4
LGALS2	0.9	0.5	0.5	0.0	0.1	0.1	0.7	0.0	0.5
TPD52L1	1.6	0.8	0.7	0.0	0.0	0.1	0.0	0.0	0.0
LOC101927617	0.4	0.4	0.6	0.0	0.0	0.8	0.5	0.2	0.3
LIN9	0.8	0.3	0.4	0.0	0.2	0.2	0.5	0.1	0.7
LOC101928267	0.9	0.2	1.1	0.0	0.2	0.1	0.3	0.2	0.2
SLCO4C1	1.2	0.4	0.6	0.0	0.0	0.1	0.3	0.1	0.5
N4BP2L1	1.0	0.3	0.6	0.0	0.0	0.1	0.5	0.1	0.4
LOC101927020	0.9	0.2	0.5	0.0	0.1	0.0	0.6	0.0	0.6
CCDC138	0.8	0.3	0.3	0.0	0.1	0.2	0.7	0.1	0.6
LOC101928665	0.9	0.2	0.6	0.0	0.4	0.1	0.5	0.1	0.2
METTL18	0.7	0.5	0.4	0.0	0.1	0.1	0.7	0.1	0.4
SOX1	0.2	0.4	0.3	0.6	0.3	0.0	0.5	0.4	0.3
EVX2	0.4	0.5	0.3	0.4	0.3	0.0	0.7	0.1	0.2
LOC101929433	1.0	0.0	0.4	0.1	0.1	0.0	0.8	0.2	0.2
TRIM17	0.8	0.2	0.7	0.1	0.0	0.1	0.6	0.0	0.3
ZNF391	0.9	0.3	0.2	0.0	0.1	0.2	0.5	0.1	0.5
LOC101929231	0.8	0.3	0.3	0.0	0.0	0.4	0.6	0.1	0.3
SP8	0.2	0.5	0.4	0.6	0.1	0.0	0.2	0.5	0.3
MLLT11	1.0	0.3	0.3	0.0	0.1	0.1	0.7	0.0	0.2
C11orf94	0.3	0.3	0.8	0.1	0.0	0.1	0.3	0.1	0.7
TM4SF19	0.7	0.2	0.5	0.0	0.2	0.2	0.8	0.1	0.3
LINC00467	0.7	0.5	0.7	0.0	0.1	0.2	0.2	0.0	0.4
SAA2	1.4	0.5	0.6	0.0	0.1	0.0	0.0	0.0	0.1
LOC100506161	0.9	0.3	0.4	0.0	0.1	0.2	0.3	0.1	0.4
SLC44A5	1.1	0.4	0.5	0.0	0.1	0.1	0.3	0.1	0.2
PMEPA1	0.5	0.2	0.3	0.1	0.0	0.3	0.8	0.1	0.3
LOC101927562	0.7	0.3	0.4	0.0	0.1	0.1	0.5	0.0	0.6
NPPA	0.6	0.1	0.8	0.0	0.1	0.1	0.6	0.1	0.1
ADAL	0.8	0.2	0.4	0.0	0.1	0.0	0.4	0.2	0.5
LOC101926987	0.6	0.1	0.3	0.0	0.3	0.2	0.4	0.1	0.6
TCEANC2	0.9	0.4	0.3	0.0	0.2	0.1	0.2	0.1	0.4
MRAP	0.9	0.2	0.3	0.0	0.2	0.2	0.4	0.2	0.2
RMI1	0.7	0.3	0.4	0.0	0.1	0.1	0.4	0.0	0.4
TMPRSS4-AS1	0.8	0.1	0.3	0.2	0.0	0.2	0.7	0.0	0.3
MTFR2	0.4	0.1	0.4	0.0	0.0	0.1	0.8	0.1	0.5
SESN1	0.7	0.1	0.3	0.0	0.1	0.2	0.5	0.1	0.5
PBLD	0.6	0.2	0.7	0.0	0.2	0.1	0.3	0.0	0.4

LOC100506504	0.8	0.2	0.2	0.1	0.0	0.0	0.3	0.2	0.6
CD34	0.8	0.4	0.4	0.0	0.1	0.1	0.4	0.0	0.2
SLC6A12	0.8	0.3	0.4	0.0	0.0	0.0	0.5	0.0	0.3
PAN3-AS1	1.1	0.2	0.4	0.1	0.0	0.2	0.1	0.1	0.3
WEE2-AS1	0.5	0.3	0.4	0.0	0.1	0.2	0.5	0.1	0.4
ZNF737	0.7	0.3	0.5	0.0	0.2	0.1	0.3	0.0	0.3
LOC100506388	0.5	0.2	0.8	0.0	0.1	0.2	0.4	0.0	0.1
LOC158960	0.3	0.4	0.5	0.0	0.1	0.3	0.4	0.0	0.4
LOC101929074	0.6	0.4	0.6	0.0	0.1	0.1	0.3	0.1	0.3
LOC101929028	0.6	0.2	0.1	0.1	0.0	0.1	0.6	0.0	0.6
ZNF713	0.6	0.2	0.4	0.0	0.1	0.1	0.5	0.1	0.4
LOC101929463	0.7	0.3	0.3	0.0	0.0	0.3	0.3	0.1	0.3
RASSF9	0.7	0.2	0.5	0.0	0.1	0.1	0.5	0.1	0.3
LCK	0.8	0.4	0.4	0.0	0.0	0.0	0.3	0.1	0.2
LOC101927821	0.5	0.1	0.3	0.0	0.0	0.2	0.6	0.1	0.3
LOC101928453	0.6	0.2	0.4	0.0	0.1	0.2	0.4	0.1	0.3
KCNIP2-AS1	0.7	0.1	0.4	0.0	0.1	0.2	0.5	0.0	0.4
ALG6	0.4	0.4	0.2	0.0	0.1	0.2	0.6	0.0	0.4
LOC101928830	0.6	0.3	0.4	0.0	0.2	0.1	0.5	0.0	0.2
GIN1	0.8	0.3	0.4	0.0	0.1	0.1	0.2	0.0	0.3
C14orf28	0.7	0.2	0.4	0.0	0.0	0.1	0.5	0.0	0.3
LOC101927541	1.0	0.2	0.5	0.0	0.0	0.1	0.3	0.0	0.2
SUCNR1	0.9	0.2	0.6	0.0	0.0	0.0	0.3	0.1	0.2
LOC100130348	0.6	0.4	0.4	0.0	0.0	0.1	0.5	0.1	0.2
LOC101926929	0.9	0.0	0.3	0.0	0.0	0.1	0.6	0.0	0.2
FOXI3	0.1	0.4	0.2	0.5	0.2	0.0	0.2	0.4	0.1
LOC101928323	0.7	0.3	0.3	0.0	0.2	0.1	0.4	0.0	0.2
TCF15	0.2	0.3	0.2	0.3	0.4	0.0	0.2	0.2	0.4
TCP10L	0.8	0.3	0.4	0.0	0.0	0.2	0.2	0.0	0.2
FAM151B	0.4	0.4	0.6	0.0	0.1	0.1	0.3	0.1	0.2
ZNF224	0.8	0.2	0.4	0.0	0.1	0.0	0.3	0.1	0.2
LOC400684	0.6	0.2	0.4	0.1	0.0	0.3	0.3	0.0	0.3
LOC101926909	0.7	0.1	0.3	0.0	0.0	0.2	0.3	0.0	0.4
LOC100505498	0.3	0.6	0.2	0.0	0.1	0.2	0.2	0.1	0.3
CEACAM1	0.9	0.4	0.4	0.0	0.2	0.1	0.0	0.0	0.1
C5orf55	0.3	0.0	0.3	0.0	0.5	0.2	0.5	0.1	0.2
LINC00575	0.7	0.2	0.4	0.0	0.0	0.1	0.5	0.0	0.2
XRCC6BP1	0.8	0.2	0.3	0.0	0.0	0.1	0.4	0.1	0.2
LINC00969	1.0	0.2	0.2	0.0	0.1	0.1	0.3	0.0	0.2
PECR	0.8	0.2	0.4	0.0	0.1	0.2	0.1	0.1	0.2
PTPRR	0.6	0.3	0.2	0.0	0.0	0.1	0.4	0.1	0.3
BCO2	0.6	0.2	0.4	0.0	0.1	0.1	0.5	0.1	0.1
SSSCA1-AS1	0.5	0.3	0.3	0.0	0.1	0.1	0.3	0.1	0.4
HCN1	0.3	0.4	0.3	0.4	0.1	0.0	0.3	0.1	0.1
LOC100128079	0.8	0.0	0.2	0.0	0.2	0.2	0.2	0.0	0.3
ZFP14	0.4	0.2	0.3	0.0	0.1	0.1	0.5	0.0	0.3
ZNF14	0.4	0.1	0.3	0.0	0.0	0.1	0.6	0.0	0.4
SYNC	0.6	0.2	0.3	0.0	0.1	0.2	0.3	0.1	0.2
LOC101928143	0.8	0.1	0.2	0.0	0.0	0.1	0.5	0.0	0.2
SRD5A3-AS1	0.5	0.1	0.3	0.0	0.1	0.1	0.5	0.0	0.3
PMCH	0.5	0.2	0.2	0.0	0.1	0.0	0.5	0.1	0.5
EBLN2	0.5	0.2	0.3	0.0	0.0	0.1	0.5	0.0	0.3
SAMD12-AS1	0.5	0.1	0.1	0.0	0.0	0.1	0.7	0.0	0.5
OVOL3	0.5	0.2	0.2	0.0	0.0	0.1	0.5	0.1	0.4
GDPD2	0.8	0.1	0.2	0.1	0.0	0.1	0.5	0.0	0.2

LOC100499489	0.5	0.1	0.3	0.1	0.0	0.0	0.4	0.1	0.3
LINC00618	0.6	0.0	0.5	0.0	0.0	0.1	0.3	0.1	0.3
CBWD6	0.5	0.2	0.3	0.0	0.1	0.1	0.4	0.0	0.3
PLAC9	1.3	0.3	0.2	0.0	0.0	0.0	0.1	0.0	0.0
LOC101928286	0.6	0.1	0.2	0.0	0.2	0.1	0.3	0.1	0.2
LOC100507165	0.6	0.2	0.3	0.0	0.1	0.2	0.2	0.1	0.1
VIM-AS1	0.5	0.2	0.2	0.0	0.1	0.0	0.5	0.0	0.3
FSD1L	0.6	0.1	0.3	0.0	0.1	0.1	0.3	0.0	0.3
LAMP3	0.7	0.3	0.3	0.0	0.0	0.1	0.2	0.0	0.2
IPP	0.5	0.2	0.3	0.0	0.1	0.1	0.4	0.0	0.2
KLRC4-KLRK1	1.0	0.3	0.3	0.0	0.1	0.1	0.1	0.0	0.0
KLRK1	1.0	0.3	0.3	0.0	0.1	0.1	0.1	0.0	0.0
ZNF891	0.5	0.2	0.3	0.0	0.1	0.0	0.4	0.0	0.3
RTKN2	0.5	0.1	0.1	0.0	0.1	0.1	0.4	0.1	0.4
LOC101928178	0.5	0.3	0.1	0.0	0.1	0.2	0.2	0.1	0.3
TRAF1	0.9	0.1	0.3	0.1	0.0	0.0	0.3	0.1	0.1
LOC101928690	0.6	0.1	0.2	0.0	0.1	0.0	0.4	0.0	0.3
TNNI3	0.5	0.3	0.2	0.0	0.0	0.2	0.1	0.0	0.5
KBTBD3	0.6	0.2	0.3	0.0	0.1	0.1	0.2	0.0	0.3
MTAP	0.5	0.4	0.3	0.0	0.0	0.1	0.2	0.0	0.2
LYPD6	0.3	0.2	0.2	0.1	0.0	0.1	0.4	0.1	0.3
RGS9BP	0.6	0.3	0.1	0.1	0.1	0.0	0.4	0.1	0.1
LOC101928659	0.7	0.1	0.2	0.0	0.1	0.2	0.3	0.1	0.1
FLJ39739	0.5	0.2	0.1	0.0	0.1	0.1	0.4	0.1	0.3
ABAT	0.3	0.1	0.3	0.0	0.1	0.1	0.3	0.0	0.5
C18orf56	0.2	0.2	0.2	0.0	0.2	0.2	0.3	0.0	0.3
SGK3	0.4	0.1	0.1	0.0	0.0	0.1	0.6	0.0	0.4
LOC100289455	0.4	0.1	0.2	0.0	0.1	0.3	0.3	0.1	0.2
LOC286189	0.7	0.1	0.2	0.0	0.1	0.1	0.3	0.0	0.3
NOXRED1	0.7	0.1	0.3	0.0	0.0	0.1	0.3	0.0	0.2
FAM27E1	0.6	0.1	0.2	0.0	0.0	0.0	0.4	0.0	0.2
RAD51L3-RFFL	0.2	0.2	0.4	0.0	0.0	0.0	0.5	0.0	0.4
HLA-DPB1	0.4	0.2	0.5	0.1	0.0	0.1	0.2	0.0	0.2
LOC101929018	0.4	0.2	0.2	0.0	0.1	0.1	0.6	0.0	0.1
CHRM3	0.6	0.2	0.3	0.0	0.0	0.1	0.3	0.0	0.1
TPPP3	0.4	0.4	0.1	0.1	0.0	0.1	0.2	0.1	0.2
GPM6A	0.6	0.1	0.2	0.0	0.0	0.1	0.4	0.0	0.2
CEP112	0.4	0.2	0.3	0.0	0.0	0.1	0.2	0.0	0.3
TRMT10A	0.4	0.1	0.3	0.0	0.0	0.1	0.3	0.0	0.4
LOC101928179	0.3	0.2	0.2	0.0	0.0	0.1	0.5	0.1	0.2
HCG17	0.3	0.1	0.3	0.0	0.1	0.1	0.5	0.0	0.3
TAS2R19	0.4	0.1	0.2	0.0	0.1	0.0	0.4	0.1	0.2
CT62	0.2	0.2	0.1	0.2	0.1	0.0	0.6	0.0	0.3
RARRES3	0.5	0.1	0.5	0.0	0.1	0.1	0.1	0.1	0.1
LRRC29	0.4	0.1	0.2	0.0	0.2	0.1	0.3	0.1	0.1
SYNE4	0.4	0.2	0.2	0.0	0.0	0.1	0.4	0.1	0.2
KAAG1	0.3	0.2	0.4	0.0	0.1	0.0	0.2	0.1	0.2
EFCAB13	0.6	0.2	0.2	0.0	0.0	0.0	0.3	0.1	0.2
LOC101927036	0.5	0.0	0.3	0.2	0.0	0.2	0.1	0.0	0.2
LOC100507066	0.8	0.0	0.3	0.0	0.0	0.0	0.2	0.0	0.1
CD79A	0.4	0.0	0.3	0.0	0.2	0.0	0.3	0.1	0.2
LINC00240	0.6	0.2	0.3	0.0	0.1	0.0	0.1	0.0	0.2
B3GALT4	0.4	0.2	0.5	0.0	0.0	0.1	0.2	0.0	0.1
ANKRD61	0.4	0.1	0.1	0.0	0.1	0.1	0.5	0.0	0.1
IL10RB-AS1	0.4	0.1	0.4	0.0	0.1	0.1	0.3	0.0	0.2

USP18	0.5	0.2	0.2	0.0	0.1	0.1	0.2	0.0	0.1
CXCL16	0.5	0.2	0.4	0.1	0.1	0.0	0.1	0.0	0.2
XXYLT1-AS2	0.4	0.1	0.2	0.0	0.1	0.1	0.3	0.0	0.1
DCLK1	0.1	0.1	0.0	0.0	0.2	0.0	0.4	0.0	0.6
C16orf71	0.4	0.1	0.1	0.0	0.1	0.1	0.5	0.1	0.1
HOXA2	0.1	0.1	0.1	0.4	0.2	0.0	0.2	0.2	0.1
LOC101927597	0.5	0.1	0.2	0.0	0.2	0.1	0.2	0.0	0.1
FAS	0.3	0.2	0.2	0.0	0.1	0.1	0.5	0.0	0.1
INSL3	0.7	0.2	0.1	0.0	0.0	0.1	0.4	0.0	0.0
HSD11B1	0.4	0.3	0.2	0.0	0.1	0.1	0.1	0.0	0.3
B3GALT1	0.4	0.2	0.3	0.0	0.0	0.1	0.2	0.0	0.2
ZNF569	0.3	0.2	0.2	0.0	0.1	0.1	0.2	0.0	0.2
IL13RA2	0.4	0.1	0.3	0.0	0.1	0.3	0.2	0.0	0.1
GPR158	0.4	0.2	0.2	0.0	0.1	0.1	0.2	0.0	0.2
LOC101060363	0.5	0.2	0.2	0.0	0.0	0.0	0.4	0.0	0.1
FAM71E1	0.4	0.3	0.2	0.1	0.0	0.2	0.1	0.0	0.2
EHD3	0.7	0.1	0.3	0.0	0.0	0.1	0.1	0.0	0.1
LOC100130357	0.4	0.3	0.1	0.0	0.0	0.0	0.5	0.0	0.1
LOC101927811	0.5	0.1	0.2	0.0	0.0	0.0	0.4	0.1	0.1
LOC101927275	0.4	0.2	0.2	0.0	0.1	0.1	0.2	0.0	0.2
NMNAT3	0.3	0.2	0.2	0.0	0.1	0.3	0.2	0.1	0.1
C1orf148	0.6	0.1	0.1	0.0	0.2	0.1	0.2	0.1	0.0
DCDC2B	0.4	0.1	0.2	0.0	0.1	0.1	0.2	0.0	0.1
AURKC	0.4	0.0	0.1	0.0	0.1	0.0	0.4	0.0	0.2
LOC101928710	0.3	0.1	0.3	0.0	0.1	0.0	0.4	0.0	0.2
PRSS48	0.5	0.1	0.3	0.0	0.0	0.1	0.3	0.1	0.1
ZNF546	0.3	0.2	0.3	0.0	0.1	0.1	0.3	0.0	0.1
EPHX3	0.4	0.2	0.2	0.0	0.0	0.1	0.3	0.0	0.1
LOC100506674	0.4	0.1	0.2	0.0	0.0	0.0	0.3	0.1	0.2
PSTPIP2	0.4	0.2	0.2	0.0	0.0	0.1	0.1	0.1	0.2
EZR-AS1	0.3	0.1	0.2	0.1	0.0	0.0	0.3	0.1	0.2
CD70	0.6	0.1	0.3	0.0	0.1	0.0	0.0	0.1	0.1
COLEC10	0.1	0.1	0.1	0.0	0.0	0.0	0.6	0.1	0.4
ACP5	0.4	0.1	0.4	0.0	0.0	0.1	0.1	0.0	0.1
SLC25A21	0.4	0.1	0.1	0.0	0.0	0.0	0.5	0.0	0.2
CLDN9	0.5	0.2	0.2	0.0	0.0	0.1	0.1	0.0	0.1
ATP6V0D2	0.5	0.1	0.2	0.0	0.1	0.0	0.2	0.0	0.2
LOC255654	0.5	0.2	0.1	0.0	0.0	0.1	0.1	0.1	0.1
LOC101928403	0.3	0.1	0.2	0.0	0.1	0.0	0.3	0.1	0.2
CLEC11A	0.5	0.1	0.2	0.1	0.0	0.0	0.1	0.1	0.1
KLK14	0.4	0.2	0.1	0.0	0.1	0.1	0.1	0.0	0.1
CYGB	0.6	0.1	0.1	0.0	0.0	0.2	0.1	0.0	0.1
CPT1B	0.4	0.2	0.3	0.0	0.0	0.1	0.1	0.0	0.1
NMB	0.9	0.1	0.1	0.0	0.0	0.0	0.1	0.0	0.1
LOC101928487	0.6	0.0	0.1	0.1	0.0	0.1	0.1	0.0	0.2
RND1	0.4	0.3	0.3	0.1	0.0	0.0	0.1	0.0	0.1
NEUROD2	0.3	0.0	0.2	0.0	0.0	0.0	0.4	0.1	0.1
RFPL4A	0.4	0.0	0.0	0.0	0.0	0.0	0.4	0.1	0.3
MMAA	0.3	0.2	0.2	0.0	0.1	0.1	0.2	0.1	0.1
LOC148696	0.4	0.1	0.2	0.0	0.1	0.1	0.2	0.0	0.1
CKM	0.2	0.1	0.2	0.0	0.0	0.1	0.4	0.1	0.2
SLC25A41	0.5	0.1	0.2	0.0	0.1	0.1	0.1	0.0	0.1
PYY	0.2	0.3	0.1	0.1	0.0	0.2	0.1	0.1	0.1
TBX15	0.2	0.1	0.2	0.0	0.0	0.0	0.3	0.1	0.3
LOC101929560	0.2	0.1	0.2	0.0	0.2	0.2	0.1	0.1	0.1

TGM2	0.5	0.1	0.3	0.0	0.0	0.0	0.1	0.0	0.1
LOC101928491	0.4	0.1	0.2	0.0	0.0	0.1	0.2	0.0	0.2
ZNF763	0.4	0.0	0.1	0.0	0.0	0.1	0.3	0.0	0.2
LOC390956	0.4	0.1	0.2	0.0	0.0	0.0	0.3	0.0	0.2
CD160	0.3	0.2	0.2	0.0	0.1	0.0	0.2	0.0	0.1
TMEM74	0.2	0.2	0.1	0.0	0.0	0.0	0.3	0.1	0.3
ENTPD3	0.3	0.1	0.1	0.0	0.1	0.1	0.2	0.1	0.2
TRNAM14	0.1	0.1	0.0	0.1	0.0	0.0	0.3	0.2	0.2
TRNAM17	0.1	0.1	0.0	0.1	0.0	0.0	0.3	0.2	0.2
TRNAM15	0.1	0.1	0.0	0.1	0.0	0.0	0.3	0.2	0.2
TRNAM18	0.1	0.1	0.0	0.1	0.0	0.0	0.3	0.2	0.2
TRNAM4	0.1	0.1	0.0	0.1	0.0	0.0	0.3	0.2	0.2
TRNAM6	0.1	0.1	0.0	0.1	0.0	0.0	0.3	0.2	0.2
TRNAM1	0.1	0.1	0.0	0.1	0.0	0.0	0.3	0.2	0.2
TRNAM3	0.1	0.1	0.0	0.1	0.0	0.0	0.3	0.2	0.2
TYRP1	0.4	0.1	0.1	0.0	0.0	0.0	0.3	0.0	0.1
LOC100506379	0.4	0.1	0.2	0.0	0.0	0.1	0.2	0.0	0.2
MGAM	0.5	0.1	0.2	0.0	0.1	0.0	0.2	0.0	0.1
PRSS35	0.4	0.1	0.2	0.0	0.0	0.0	0.2	0.0	0.2
ZNF396	0.4	0.2	0.2	0.0	0.1	0.1	0.2	0.0	0.1
USP44	0.4	0.2	0.2	0.0	0.0	0.1	0.2	0.0	0.2
IL23A	0.3	0.1	0.2	0.0	0.0	0.0	0.2	0.0	0.3
RAET1E	0.2	0.1	0.1	0.0	0.1	0.0	0.3	0.1	0.3
LOC101928816	0.4	0.1	0.1	0.0	0.0	0.0	0.3	0.0	0.1
EPHA3	0.4	0.1	0.2	0.0	0.0	0.1	0.2	0.0	0.1
LINC00326	0.4	0.2	0.2	0.0	0.0	0.0	0.1	0.0	0.1
KDR	0.3	0.1	0.1	0.0	0.0	0.1	0.3	0.0	0.1
KAZN	0.4	0.1	0.2	0.1	0.0	0.1	0.2	0.0	0.1
RNASEH2B-AS1	0.4	0.1	0.3	0.0	0.1	0.0	0.1	0.1	0.1
SERPINE3	0.3	0.1	0.1	0.0	0.0	0.1	0.2	0.0	0.1
DSCR8	0.4	0.0	0.2	0.0	0.0	0.0	0.3	0.0	0.2
LOC101928324	0.4	0.1	0.1	0.1	0.0	0.0	0.1	0.1	0.2
ISPD	0.2	0.1	0.2	0.0	0.0	0.1	0.2	0.0	0.3
SLC46A3	0.3	0.1	0.1	0.0	0.2	0.0	0.1	0.0	0.2
LINC00163	0.3	0.0	0.1	0.0	0.0	0.0	0.3	0.0	0.3
NPC1L1	0.3	0.0	0.1	0.0	0.0	0.0	0.3	0.0	0.3
LOC101929594	0.3	0.1	0.1	0.0	0.0	0.2	0.0	0.0	0.2
SIX3	0.3	0.1	0.2	0.0	0.1	0.1	0.2	0.1	0.2
CDRT15	0.4	0.1	0.2	0.0	0.0	0.1	0.2	0.0	0.1
LOC101928948	0.5	0.0	0.1	0.0	0.0	0.0	0.2	0.0	0.2
ZNF492	0.1	0.1	0.1	0.0	0.1	0.1	0.4	0.0	0.1
ZNF117	0.3	0.2	0.2	0.0	0.0	0.0	0.2	0.0	0.1
LOC101928865	0.4	0.1	0.1	0.0	0.1	0.0	0.2	0.0	0.1
KCTD16	0.2	0.1	0.1	0.0	0.0	0.1	0.4	0.0	0.2
LOC101927974	0.4	0.1	0.1	0.0	0.0	0.1	0.1	0.0	0.2
XIRP2	0.4	0.1	0.1	0.0	0.1	0.0	0.2	0.0	0.2
C19orf18	0.4	0.1	0.2	0.0	0.1	0.1	0.1	0.0	0.0
MFNG	0.3	0.1	0.1	0.0	0.1	0.1	0.2	0.0	0.1
OMP	0.1	0.2	0.1	0.0	0.1	0.0	0.2	0.1	0.1
APOBEC3G	0.4	0.1	0.1	0.0	0.0	0.2	0.1	0.1	0.1
CATSPER2	0.3	0.2	0.1	0.0	0.0	0.1	0.1	0.0	0.1
PDE11A	0.2	0.1	0.1	0.0	0.1	0.1	0.3	0.0	0.1
LOC101929748	0.5	0.2	0.1	0.0	0.0	0.0	0.1	0.0	0.0
TMPRSS6	0.2	0.1	0.2	0.0	0.0	0.0	0.2	0.0	0.2
LOC100996318	0.3	0.0	0.3	0.0	0.1	0.0	0.2	0.0	0.1

TAS2R31	0.4	0.0	0.2	0.0	0.0	0.0	0.2	0.0	0.1
PP2D1	0.4	0.0	0.1	0.0	0.0	0.1	0.1	0.0	0.2
AMER2	0.1	0.1	0.2	0.2	0.1	0.0	0.2	0.1	0.2
CFP	0.3	0.1	0.1	0.0	0.0	0.1	0.2	0.0	0.1
ZNF90	0.2	0.1	0.1	0.0	0.1	0.0	0.2	0.0	0.2
CRIP1	0.3	0.2	0.1	0.0	0.0	0.0	0.2	0.1	0.1
LOC101927467	0.4	0.1	0.1	0.0	0.0	0.0	0.2	0.0	0.1
LOC101927124	0.3	0.0	0.1	0.0	0.0	0.1	0.2	0.0	0.2
ST6GALNAC2	0.3	0.1	0.1	0.0	0.1	0.0	0.2	0.0	0.2
PDGFRA	0.4	0.1	0.2	0.0	0.0	0.0	0.1	0.0	0.1
BHLHE22	0.1	0.3	0.2	0.1	0.1	0.0	0.1	0.0	0.0
SLC6A16	0.4	0.1	0.2	0.0	0.0	0.0	0.1	0.0	0.1
ZNF93	0.4	0.0	0.1	0.0	0.0	0.1	0.2	0.0	0.1
ACRV1	0.2	0.1	0.1	0.0	0.1	0.1	0.3	0.0	0.1
POU2F3	0.2	0.1	0.1	0.0	0.0	0.0	0.2	0.0	0.3
COL11A2	0.2	0.1	0.2	0.0	0.0	0.0	0.2	0.0	0.3
FEV	0.2	0.1	0.1	0.0	0.0	0.1	0.1	0.1	0.2
LOC101929222	0.5	0.0	0.1	0.0	0.1	0.0	0.0	0.1	0.2
MPP7	0.3	0.1	0.1	0.0	0.0	0.0	0.2	0.0	0.2
LOC101928838	0.1	0.0	0.0	0.1	0.0	0.1	0.2	0.1	0.3
ACTN1-AS1	0.3	0.0	0.3	0.1	0.0	0.0	0.2	0.0	0.0
LOC101059949	0.3	0.0	0.1	0.0	0.0	0.0	0.3	0.0	0.2
LINC00884	0.2	0.1	0.1	0.0	0.1	0.1	0.1	0.0	0.0
OXCT2	0.2	0.1	0.1	0.1	0.0	0.1	0.2	0.0	0.1
LOC101928825	0.4	0.1	0.0	0.0	0.0	0.0	0.0	0.0	0.2
GAL3ST1	0.1	0.2	0.1	0.0	0.0	0.0	0.3	0.0	0.1
LOC101928417	0.2	0.1	0.1	0.0	0.0	0.1	0.2	0.1	0.1
LOC101929619	0.1	0.2	0.2	0.0	0.0	0.1	0.2	0.1	0.1
HLA-DQB1	0.2	0.1	0.1	0.1	0.0	0.0	0.1	0.1	0.1
LOC101927751	0.2	0.1	0.2	0.0	0.0	0.1	0.2	0.0	0.1
PILRA	0.2	0.1	0.1	0.0	0.1	0.0	0.2	0.0	0.2
MDGA2	0.4	0.1	0.2	0.0	0.1	0.0	0.1	0.0	0.0
ZNF385B	0.4	0.1	0.1	0.0	0.0	0.0	0.1	0.1	0.1
RNF165	0.2	0.2	0.1	0.0	0.1	0.0	0.1	0.0	0.1
DUOXA1	0.2	0.1	0.1	0.0	0.0	0.0	0.3	0.0	0.1
AMZ1	0.2	0.0	0.2	0.0	0.0	0.1	0.2	0.0	0.1
TMEM74B	0.3	0.0	0.1	0.0	0.0	0.0	0.2	0.0	0.0
DKFZP434K028	0.3	0.0	0.1	0.0	0.0	0.0	0.2	0.1	0.2
MNS1	0.2	0.0	0.1	0.0	0.1	0.1	0.1	0.0	0.1
GJC1	0.2	0.1	0.1	0.0	0.1	0.0	0.1	0.0	0.1
TSKS	0.2	0.0	0.2	0.0	0.0	0.1	0.1	0.1	0.1
LOC101929004	0.2	0.1	0.1	0.0	0.1	0.1	0.1	0.0	0.3
LPPR3	0.3	0.1	0.1	0.0	0.0	0.0	0.2	0.0	0.1
EMX1	0.4	0.1	0.1	0.0	0.0	0.0	0.1	0.0	0.0
SMCO2	0.2	0.1	0.1	0.0	0.0	0.1	0.1	0.0	0.2
C20orf173	0.2	0.0	0.2	0.0	0.0	0.1	0.2	0.0	0.1
LY6G5C	0.1	0.1	0.2	0.0	0.0	0.1	0.3	0.1	0.0
SMAD5-AS1	0.3	0.1	0.2	0.0	0.0	0.0	0.2	0.0	0.1
LOC643072	0.3	0.1	0.2	0.0	0.1	0.1	0.1	0.0	0.1
TIGD3	0.2	0.1	0.2	0.0	0.0	0.0	0.1	0.0	0.1
ZNF879	0.2	0.1	0.1	0.0	0.1	0.1	0.1	0.0	0.1
PCDHGC4	0.3	0.0	0.1	0.0	0.1	0.0	0.3	0.0	0.1
ALDH1A2	0.1	0.1	0.1	0.0	0.0	0.0	0.2	0.0	0.2
LOC100129461	0.4	0.1	0.1	0.0	0.1	0.1	0.2	0.0	0.0
GABBR2	0.0	0.3	0.2	0.1	0.0	0.0	0.2	0.0	0.1

TMEM45B	0.2	0.0	0.2	0.0	0.0	0.0	0.2	0.0	0.1
KHDRBS2	0.2	0.1	0.1	0.0	0.1	0.0	0.1	0.1	0.1
RHOV	0.2	0.1	0.1	0.1	0.0	0.0	0.2	0.0	0.1
RIMS3	0.3	0.0	0.1	0.0	0.2	0.0	0.2	0.0	0.0
TNNC1	0.0	0.1	0.2	0.1	0.1	0.0	0.2	0.1	0.1
ALX4	0.2	0.0	0.2	0.0	0.0	0.0	0.2	0.0	0.1
AATK	0.2	0.1	0.1	0.0	0.0	0.0	0.2	0.0	0.1
LHX9	0.2	0.1	0.1	0.0	0.0	0.1	0.3	0.0	0.1
FGF21	0.1	0.1	0.1	0.0	0.2	0.1	0.2	0.0	0.1
SYCP2L	0.2	0.1	0.2	0.0	0.1	0.0	0.2	0.0	0.1
LOC100996695	0.2	0.0	0.1	0.0	0.0	0.1	0.3	0.1	0.1
LOC100505555	0.2	0.1	0.2	0.0	0.1	0.1	0.2	0.0	0.1
LOC100130502	0.3	0.0	0.1	0.0	0.0	0.1	0.1	0.0	0.1
LINC00341	0.2	0.1	0.1	0.0	0.0	0.0	0.1	0.0	0.2
ADAMTS3	0.2	0.1	0.1	0.0	0.1	0.1	0.2	0.0	0.1
LOC101929467	0.2	0.0	0.1	0.0	0.1	0.0	0.2	0.0	0.0
PROX1	0.3	0.1	0.1	0.0	0.0	0.0	0.1	0.0	0.1
CATSPER3	0.2	0.1	0.1	0.0	0.0	0.1	0.1	0.1	0.1
OLAH	0.3	0.0	0.0	0.0	0.1	0.0	0.1	0.0	0.1
LOC101926901	0.2	0.2	0.0	0.0	0.0	0.0	0.0	0.0	0.3
BMPER	0.1	0.1	0.1	0.0	0.1	0.1	0.2	0.0	0.1
PLD5	0.2	0.1	0.1	0.0	0.1	0.0	0.1	0.0	0.2
ACSM3	0.2	0.0	0.0	0.0	0.0	0.1	0.1	0.0	0.2
GSTM2	0.2	0.1	0.2	0.0	0.1	0.0	0.1	0.0	0.1
TMEM232	0.3	0.1	0.1	0.0	0.1	0.1	0.1	0.0	0.1
FAM41C	0.2	0.1	0.1	0.0	0.0	0.1	0.1	0.0	0.1
SUSD4	0.1	0.1	0.0	0.0	0.1	0.1	0.2	0.0	0.2
KCNAB3	0.2	0.1	0.1	0.0	0.0	0.1	0.1	0.0	0.2
IL16	0.2	0.0	0.1	0.0	0.0	0.0	0.2	0.0	0.2
SLFN13	0.2	0.1	0.2	0.0	0.0	0.1	0.1	0.0	0.0
B3GAT2	0.1	0.1	0.1	0.0	0.0	0.1	0.3	0.0	0.0
C3AR1	0.1	0.1	0.1	0.0	0.1	0.1	0.2	0.0	0.2
WNT4	0.2	0.1	0.1	0.0	0.0	0.1	0.1	0.0	0.1
CASC2	0.3	0.1	0.1	0.0	0.0	0.1	0.1	0.0	0.1
ZFP2	0.2	0.1	0.2	0.0	0.0	0.0	0.1	0.0	0.1
LOC339524	0.3	0.1	0.1	0.0	0.0	0.0	0.2	0.0	0.0
LOC101929155	0.2	0.1	0.1	0.0	0.0	0.1	0.1	0.0	0.1
OR8B4	0.3	0.0	0.0	0.0	0.1	0.0	0.1	0.1	0.1
PAQR5	0.2	0.0	0.0	0.0	0.0	0.0	0.3	0.1	0.1
LOC101928222	0.1	0.1	0.0	0.0	0.1	0.0	0.2	0.0	0.2
TUBB1	0.4	0.0	0.2	0.0	0.0	0.0	0.0	0.0	0.1
FSIP1	0.2	0.1	0.1	0.0	0.0	0.1	0.1	0.0	0.1
OR8B8	0.2	0.0	0.2	0.0	0.0	0.0	0.1	0.0	0.1
INSL4	0.3	0.1	0.1	0.0	0.1	0.0	0.1	0.1	0.1
CNN1	0.1	0.1	0.0	0.0	0.1	0.2	0.2	0.0	0.0
LINC00589	0.2	0.1	0.3	0.0	0.1	0.0	0.1	0.0	0.1
TREH	0.4	0.0	0.1	0.0	0.1	0.1	0.1	0.0	0.1
ST7-OT4	0.1	0.1	0.1	0.0	0.1	0.1	0.1	0.0	0.1
LOC100287704	0.3	0.1	0.1	0.0	0.0	0.0	0.1	0.0	0.1
SNCG	0.2	0.1	0.1	0.0	0.1	0.1	0.1	0.0	0.0
CSF1R	0.2	0.1	0.1	0.0	0.0	0.0	0.2	0.0	0.1
LOC729815	0.2	0.1	0.2	0.0	0.0	0.0	0.1	0.0	0.1
SLCO1B1	0.2	0.0	0.1	0.0	0.0	0.0	0.1	0.0	0.2
EGR3	0.1	0.1	0.3	0.0	0.0	0.0	0.0	0.0	0.1
IGLV5-52	0.2	0.1	0.1	0.0	0.0	0.1	0.2	0.0	0.1

LOC100996592	0.2	0.1	0.0	0.0	0.1	0.0	0.1	0.0	0.1
OLFM2	0.3	0.1	0.0	0.0	0.0	0.0	0.2	0.0	0.0
PALM2	0.3	0.1	0.1	0.0	0.0	0.1	0.1	0.0	0.1
PRKCG	0.2	0.1	0.1	0.0	0.0	0.0	0.1	0.1	0.1
SLC9A9	0.2	0.1	0.1	0.0	0.1	0.1	0.1	0.0	0.0
SOX18	0.2	0.1	0.1	0.1	0.0	0.0	0.2	0.0	0.0
CAPN3	0.3	0.0	0.1	0.0	0.0	0.1	0.1	0.0	0.0
ALG1L	0.2	0.1	0.0	0.0	0.1	0.1	0.1	0.0	0.1
NAALADL2	0.2	0.0	0.1	0.0	0.1	0.1	0.1	0.0	0.1
GRB14	0.2	0.1	0.0	0.0	0.0	0.0	0.1	0.0	0.1
TLR1	0.2	0.1	0.1	0.0	0.0	0.0	0.1	0.1	0.1
NKX1-2	0.1	0.1	0.1	0.1	0.1	0.0	0.1	0.0	0.1
LEFTY1	0.1	0.0	0.1	0.0	0.0	0.0	0.3	0.0	0.1
ZNF491	0.3	0.1	0.1	0.0	0.0	0.0	0.1	0.1	0.1
ZNF583	0.2	0.1	0.1	0.0	0.1	0.1	0.1	0.0	0.0
GPBAR1	0.1	0.0	0.1	0.1	0.0	0.1	0.2	0.0	0.1
CHRND	0.3	0.0	0.1	0.0	0.0	0.0	0.1	0.0	0.0
LOC100506127	0.2	0.1	0.1	0.0	0.0	0.1	0.1	0.0	0.1
GPRC5D	0.2	0.1	0.1	0.0	0.0	0.0	0.1	0.0	0.0
MUSTN1	0.0	0.1	0.2	0.0	0.1	0.0	0.2	0.2	0.0
ZNF829	0.3	0.1	0.1	0.0	0.0	0.0	0.0	0.0	0.1
LOC101929276	0.2	0.1	0.2	0.0	0.1	0.0	0.0	0.0	0.0
EPHA6	0.2	0.1	0.1	0.0	0.0	0.0	0.1	0.0	0.1
TXNDC2	0.1	0.1	0.1	0.0	0.0	0.0	0.2	0.1	0.2
ZNF658	0.2	0.1	0.1	0.0	0.0	0.0	0.1	0.0	0.1
GPR52	0.1	0.0	0.1	0.0	0.0	0.1	0.2	0.0	0.1
MLNR	0.1	0.0	0.2	0.0	0.0	0.1	0.0	0.0	0.1
SERPIND1	0.3	0.1	0.1	0.0	0.1	0.0	0.1	0.0	0.1
SLITRK6	0.3	0.1	0.1	0.0	0.0	0.0	0.0	0.0	0.0
UNC5B	0.1	0.0	0.1	0.0	0.1	0.0	0.2	0.0	0.1
PTPN6	0.1	0.0	0.1	0.1	0.0	0.1	0.1	0.0	0.1
LOC100506446	0.2	0.0	0.2	0.0	0.0	0.0	0.2	0.0	0.0
CLIC5	0.1	0.1	0.0	0.0	0.1	0.0	0.2	0.1	0.1
CAPN12	0.2	0.1	0.1	0.0	0.0	0.0	0.1	0.0	0.1
LOC101928147	0.2	0.0	0.1	0.0	0.0	0.1	0.1	0.0	0.1
ZRANB2-AS1	0.3	0.1	0.1	0.0	0.0	0.0	0.1	0.0	0.1
SLC12A8	0.2	0.1	0.1	0.0	0.0	0.0	0.1	0.0	0.1
C2	0.1	0.1	0.1	0.0	0.0	0.0	0.1	0.0	0.1
ASPDH	0.2	0.0	0.1	0.0	0.0	0.0	0.1	0.0	0.0
NRXN1	0.1	0.1	0.0	0.0	0.0	0.0	0.1	0.1	0.1
LOC101927829	0.3	0.0	0.1	0.0	0.0	0.0	0.1	0.0	0.0
PCDH18	0.2	0.1	0.1	0.0	0.0	0.0	0.1	0.0	0.0
ZNF404	0.3	0.1	0.1	0.0	0.0	0.0	0.0	0.0	0.0
CHDH	0.2	0.0	0.2	0.0	0.0	0.0	0.1	0.0	0.1
EPHB3	0.1	0.1	0.1	0.0	0.0	0.0	0.1	0.0	0.1
OR10A3	0.1	0.1	0.1	0.0	0.0	0.0	0.1	0.0	0.2
GNAL	0.2	0.0	0.1	0.0	0.0	0.0	0.1	0.1	0.1
FAM124A	0.1	0.0	0.1	0.0	0.0	0.0	0.1	0.1	0.1
ZNF382	0.2	0.2	0.1	0.0	0.0	0.0	0.1	0.0	0.0
CD5	0.1	0.0	0.1	0.0	0.0	0.0	0.1	0.0	0.2
FBLN2	0.2	0.1	0.1	0.0	0.0	0.0	0.1	0.0	0.0
LOC101927943	0.2	0.1	0.1	0.0	0.0	0.0	0.1	0.0	0.1
AIM2	0.2	0.1	0.1	0.0	0.0	0.0	0.1	0.0	0.0
ABCG2	0.0	0.1	0.0	0.0	0.0	0.0	0.2	0.0	0.2
ABCA5	0.1	0.0	0.1	0.0	0.0	0.0	0.1	0.0	0.1

LOC101927565	0.2	0.1	0.1	0.0	0.0	0.0	0.1	0.0	0.1
LOC729040	0.2	0.1	0.1	0.0	0.0	0.1	0.1	0.0	0.1
LOC101928203	0.3	0.0	0.1	0.0	0.0	0.0	0.1	0.0	0.0
IRGC	0.2	0.0	0.0	0.0	0.0	0.0	0.1	0.0	0.1
WNT10B	0.0	0.1	0.0	0.0	0.0	0.1	0.1	0.0	0.2
HIPK4	0.2	0.0	0.1	0.0	0.0	0.0	0.1	0.0	0.1
SLC17A7	0.2	0.1	0.1	0.0	0.0	0.1	0.1	0.0	0.0
STARD5	0.2	0.0	0.1	0.0	0.1	0.0	0.1	0.0	0.0
HOMEZ	0.2	0.0	0.1	0.0	0.0	0.0	0.1	0.0	0.1
MAMDC2	0.2	0.1	0.1	0.0	0.0	0.0	0.1	0.0	0.1
IGLL5	0.1	0.0	0.1	0.0	0.0	0.1	0.2	0.0	0.1
LOC100132731	0.2	0.0	0.1	0.0	0.0	0.0	0.1	0.0	0.1
SEMA4A	0.2	0.1	0.1	0.0	0.0	0.0	0.1	0.0	0.1
OXER1	0.2	0.0	0.1	0.0	0.0	0.0	0.0	0.0	0.1
LOC100506371	0.1	0.0	0.1	0.0	0.1	0.0	0.1	0.1	0.1
C7orf10	0.1	0.1	0.1	0.0	0.0	0.0	0.1	0.0	0.0
TMEM151B	0.1	0.1	0.0	0.2	0.1	0.0	0.0	0.0	0.0
EIF4E3	0.0	0.1	0.2	0.0	0.1	0.0	0.1	0.1	0.1
SLC8A2	0.2	0.1	0.1	0.0	0.0	0.0	0.1	0.0	0.1
ADSSL1	0.2	0.1	0.1	0.0	0.0	0.1	0.0	0.0	0.0
CCDC39	0.2	0.0	0.0	0.0	0.0	0.0	0.1	0.0	0.1
LOC100506071	0.2	0.1	0.2	0.0	0.0	0.0	0.1	0.0	0.0
LOC100507562	0.1	0.0	0.0	0.0	0.1	0.0	0.1	0.0	0.1
LOC101927827	0.2	0.0	0.1	0.0	0.0	0.0	0.1	0.0	0.1
DACT3-AS1	0.0	0.0	0.3	0.0	0.1	0.1	0.0	0.0	0.0
ANKRD35	0.2	0.1	0.2	0.0	0.0	0.0	0.0	0.0	0.0
SESN3	0.2	0.1	0.0	0.0	0.0	0.0	0.0	0.0	0.1
STON2	0.2	0.1	0.0	0.0	0.0	0.0	0.1	0.0	0.1
GUCY1B3	0.1	0.1	0.1	0.0	0.0	0.1	0.0	0.0	0.1
LOC101927868	0.1	0.0	0.1	0.0	0.0	0.0	0.1	0.0	0.1
LRRC73	0.0	0.1	0.0	0.0	0.0	0.0	0.2	0.0	0.2
TRIM22	0.1	0.0	0.1	0.0	0.0	0.0	0.1	0.0	0.1
C16orf98	0.2	0.0	0.1	0.0	0.0	0.0	0.1	0.0	0.0
ROBO2	0.2	0.1	0.1	0.0	0.0	0.0	0.1	0.0	0.0
ADCY8	0.1	0.0	0.0	0.0	0.0	0.0	0.2	0.0	0.1
NOTCH4	0.1	0.0	0.1	0.0	0.0	0.0	0.1	0.0	0.1
LINC00906	0.2	0.0	0.1	0.0	0.0	0.0	0.1	0.0	0.1
PLCB2	0.1	0.0	0.0	0.0	0.0	0.0	0.1	0.0	0.0
FLJ42969	0.1	0.1	0.1	0.0	0.0	0.0	0.1	0.0	0.0
CP	0.2	0.0	0.1	0.0	0.0	0.0	0.0	0.0	0.1
FAM71E2	0.2	0.0	0.1	0.0	0.0	0.0	0.1	0.0	0.1
ANKRD30B	0.1	0.1	0.1	0.0	0.0	0.0	0.0	0.0	0.1
LOC101927206	0.1	0.1	0.0	0.0	0.1	0.0	0.0	0.0	0.1
ACOX2	0.1	0.0	0.1	0.0	0.0	0.0	0.1	0.0	0.1
LINC00669	0.1	0.0	0.0	0.0	0.1	0.0	0.1	0.0	0.1
PRKG2	0.1	0.1	0.1	0.0	0.0	0.0	0.1	0.1	0.0
NELL2	0.1	0.1	0.1	0.0	0.0	0.1	0.0	0.0	0.0
LINC00943	0.1	0.0	0.1	0.0	0.0	0.1	0.1	0.0	0.0
RASGRP2	0.1	0.1	0.1	0.0	0.0	0.0	0.1	0.0	0.0
LOC440292	0.2	0.1	0.1	0.0	0.0	0.0	0.1	0.0	0.0
LOC100129858	0.1	0.0	0.1	0.0	0.0	0.0	0.1	0.0	0.0
ABCA10	0.2	0.0	0.1	0.0	0.0	0.0	0.1	0.0	0.1
MOXD1	0.1	0.0	0.1	0.0	0.0	0.0	0.1	0.0	0.0
CDH10	0.1	0.0	0.1	0.0	0.0	0.0	0.1	0.0	0.1
GPR22	0.1	0.0	0.1	0.0	0.1	0.0	0.0	0.0	0.0

SPI1	0.1	0.0	0.1	0.0	0.0	0.0	0.0	0.0	0.1
LOC101928745	0.2	0.0	0.0	0.0	0.1	0.0	0.0	0.0	0.0
LOC100996276	0.2	0.0	0.1	0.0	0.0	0.0	0.0	0.0	0.1
GDF9	0.1	0.0	0.1	0.0	0.0	0.0	0.1	0.0	0.0
ODF3L1	0.1	0.1	0.0	0.0	0.1	0.0	0.0	0.0	0.0
LOC101929118	0.1	0.0	0.1	0.0	0.0	0.0	0.1	0.0	0.1
YBX2	0.1	0.1	0.0	0.0	0.0	0.1	0.1	0.0	0.1
LOC101927554	0.1	0.0	0.1	0.0	0.0	0.0	0.1	0.0	0.1
MGC2889	0.1	0.0	0.1	0.0	0.0	0.0	0.0	0.0	0.1
CAMSAP3	0.1	0.0	0.1	0.0	0.0	0.0	0.1	0.0	0.1
CNTD1	0.0	0.1	0.1	0.0	0.0	0.0	0.1	0.1	0.0
SETBP1	0.1	0.1	0.0	0.1	0.0	0.0	0.0	0.0	0.0
C4A	0.1	0.0	0.1	0.0	0.0	0.1	0.0	0.0	0.1
MDH1B	0.2	0.0	0.1	0.0	0.0	0.0	0.0	0.0	0.0
NOTUM	0.1	0.0	0.0	0.0	0.0	0.0	0.1	0.0	0.0
LOC728875	0.0	0.0	0.1	0.0	0.0	0.0	0.2	0.0	0.0
AVPR2	0.1	0.0	0.1	0.0	0.0	0.0	0.1	0.0	0.1
SLC14A2	0.2	0.0	0.0	0.0	0.0	0.0	0.1	0.0	0.0
CTRC	0.1	0.0	0.0	0.0	0.0	0.0	0.1	0.0	0.1
LOC100128554	0.1	0.1	0.1	0.0	0.0	0.0	0.1	0.0	0.0
TJP3	0.1	0.1	0.1	0.0	0.0	0.0	0.1	0.0	0.0
LOC100287834	0.2	0.0	0.1	0.0	0.0	0.0	0.1	0.0	0.0
TMTC1	0.1	0.1	0.0	0.0	0.0	0.0	0.0	0.0	0.0
LOC101060825	0.1	0.1	0.1	0.0	0.0	0.0	0.0	0.0	0.0
LINC00518	0.0	0.0	0.0	0.0	0.0	0.0	0.1	0.0	0.1
ITGA7	0.1	0.0	0.1	0.0	0.0	0.0	0.0	0.0	0.0
GRIN2B	0.2	0.0	0.1	0.0	0.0	0.0	0.0	0.0	0.0
ZNF732	0.1	0.1	0.0	0.0	0.0	0.0	0.0	0.0	0.0
LINC00928	0.1	0.0	0.0	0.0	0.0	0.0	0.1	0.0	0.0
LOC101929103	0.0	0.1	0.1	0.0	0.0	0.0	0.1	0.0	0.0
LOC400685	0.1	0.0	0.1	0.0	0.0	0.0	0.0	0.0	0.1
LOC101927787	0.1	0.0	0.0	0.0	0.1	0.0	0.1	0.0	0.0
ERBB4	0.1	0.0	0.1	0.0	0.0	0.0	0.1	0.0	0.0
LINC00242	0.1	0.0	0.1	0.0	0.0	0.0	0.0	0.0	0.0
LAMA2	0.1	0.0	0.1	0.0	0.0	0.0	0.1	0.0	0.0
IGKV1-12	0.1	0.0	0.1	0.0	0.0	0.0	0.1	0.0	0.0
TLL2	0.1	0.0	0.0	0.0	0.0	0.0	0.0	0.0	0.0
ATG9B	0.1	0.0	0.0	0.0	0.0	0.0	0.1	0.0	0.0
LINC00184	0.0	0.0	0.1	0.0	0.0	0.1	0.0	0.0	0.0
C17orf104	0.1	0.0	0.0	0.0	0.0	0.0	0.1	0.0	0.0
IGLC1	0.1	0.0	0.1	0.0	0.0	0.0	0.1	0.0	0.0
LINC00312	0.0	0.0	0.1	0.0	0.0	0.0	0.1	0.0	0.1
GRM8	0.1	0.0	0.0	0.0	0.0	0.0	0.1	0.0	0.0
C8orf44-SGK3	0.1	0.0	0.0	0.0	0.0	0.0	0.1	0.0	0.1
LOC728158	0.1	0.0	0.0	0.0	0.0	0.0	0.0	0.0	0.0
LRRC43	0.1	0.0	0.0	0.0	0.0	0.0	0.1	0.0	0.0
PDE3A	0.1	0.0	0.0	0.0	0.0	0.0	0.1	0.0	0.0
HHIPL2	0.1	0.1	0.0	0.0	0.0	0.0	0.0	0.0	0.0
LOC101928834	0.1	0.0	0.1	0.0	0.0	0.0	0.0	0.0	0.0
NTRK1	0.1	0.0	0.1	0.0	0.0	0.0	0.0	0.0	0.0
GRHL1	0.1	0.0	0.1	0.0	0.0	0.0	0.1	0.0	0.0
LINC00304	0.1	0.0	0.0	0.0	0.0	0.0	0.0	0.0	0.0
FLJ38576	0.0	0.1	0.1	0.0	0.0	0.0	0.1	0.0	0.0
SPANXA2	0.0	0.1	0.0	0.0	0.0	0.0	0.1	0.0	0.1
SPANXA1	0.0	0.1	0.0	0.0	0.0	0.0	0.1	0.0	0.1

LINC00856	0.0	0.0	0.0	0.0	0.0	0.0	0.2	0.0	0.0
PRKCZ	0.1	0.0	0.0	0.0	0.0	0.0	0.0	0.0	0.1
PAPPA-AS1	0.0	0.0	0.0	0.0	0.1	0.0	0.0	0.0	0.0
ARHGEF16	0.0	0.0	0.0	0.0	0.0	0.0	0.1	0.0	0.0
PCDH19	0.0	0.0	0.0	0.0	0.0	0.0	0.1	0.0	0.0
PDE8B	0.1	0.0	0.1	0.0	0.0	0.0	0.0	0.0	0.0
SCUBE2	0.0	0.0	0.1	0.0	0.0	0.0	0.0	0.0	0.0
NCKAP1L	0.0	0.0	0.0	0.0	0.0	0.0	0.1	0.0	0.0
CNTN1	0.0	0.0	0.0	0.0	0.0	0.0	0.0	0.0	0.0
CACNA2D2	0.1	0.0	0.1	0.0	0.0	0.0	0.0	0.0	0.0
CAGE1	0.1	0.0	0.0	0.0	0.0	0.0	0.1	0.0	0.0
LOC100128770	0.1	0.0	0.0	0.0	0.0	0.0	0.0	0.0	0.0
PRG4	0.1	0.0	0.0	0.0	0.0	0.0	0.0	0.0	0.0
PCDHA13	0.1	0.0	0.0	0.0	0.0	0.0	0.1	0.0	0.0
KATNAL2	0.1	0.0	0.0	0.0	0.0	0.0	0.0	0.0	0.0
PSG8	0.1	0.0	0.0	0.0	0.0	0.0	0.0	0.0	0.0
UNC13A	0.0	0.0	0.0	0.0	0.0	0.0	0.0	0.0	0.0
KCNH1	0.1	0.0	0.0	0.0	0.0	0.0	0.1	0.0	0.0
AIFM3	0.1	0.0	0.0	0.0	0.1	0.0	0.0	0.0	0.0
CYP2A7	0.0	0.0	0.0	0.0	0.0	0.0	0.1	0.0	0.1
TRPV4	0.1	0.0	0.0	0.0	0.0	0.0	0.1	0.0	0.0
LOC100129027	0.0	0.0	0.1	0.0	0.0	0.0	0.0	0.0	0.0
RASGRP4	0.0	0.0	0.1	0.0	0.0	0.0	0.0	0.0	0.0
ELFN1	0.0	0.0	0.0	0.0	0.0	0.0	0.1	0.0	0.0
CCM2L	0.0	0.0	0.0	0.0	0.0	0.0	0.0	0.0	0.0
F2	0.1	0.0	0.0	0.0	0.0	0.0	0.0	0.0	0.0
CEBPA-AS1	0.0	0.0	0.0	0.0	0.0	0.0	0.1	0.0	0.0
CYP21A2	0.0	0.0	0.0	0.0	0.0	0.0	0.0	0.0	0.0
TKTL2	0.0	0.0	0.0	0.0	0.0	0.0	0.0	0.0	0.0
ANKRD30A	0.0	0.0	0.0	0.0	0.0	0.0	0.0	0.0	0.0
VWF	0.0	0.0	0.0	0.0	0.0	0.0	0.0	0.0	0.0
AKNAD1	0.0	0.0	0.0	0.0	0.0	0.0	0.0	0.0	0.0
PRSS36	0.0	0.0	0.0	0.0	0.0	0.0	0.0	0.0	0.1
MCF2L	0.0	0.0	0.1	0.0	0.0	0.0	0.0	0.0	0.0
LOC388210	0.1	0.0	0.0	0.0	0.0	0.0	0.0	0.0	0.0
LOC286114	0.1	0.0	0.0	0.0	0.0	0.0	0.0	0.0	0.0
ANKRD20A4	0.0	0.0	0.0	0.0	0.0	0.0	0.0	0.0	0.0
BAI3	0.0	0.0	0.0	0.0	0.0	0.0	0.0	0.0	0.0
NRXN2	0.0	0.0	0.0	0.1	0.0	0.0	0.0	0.0	0.0
NUTM2F	0.0	0.1	0.0	0.0	0.0	0.0	0.0	0.0	0.0
LRP1B	0.0	0.0	0.0	0.0	0.0	0.0	0.0	0.0	0.0
AKAP3	0.0	0.0	0.0	0.0	0.0	0.0	0.0	0.0	0.0
ZDHHC11B	0.0	0.0	0.0	0.0	0.0	0.0	0.0	0.0	0.0
IL10RA	0.0	0.0	0.0	0.0	0.0	0.0	0.0	0.0	0.0
ANPEP	0.0	0.0	0.0	0.0	0.0	0.0	0.0	0.0	0.0
WDFY4	0.0	0.0	0.0	0.0	0.0	0.0	0.0	0.0	0.0
ANKRD18B	0.0	0.0	0.0	0.0	0.0	0.0	0.0	0.0	0.0
PGR	0.0	0.0	0.0	0.0	0.0	0.0	0.0	0.0	0.0
COBL	0.0	0.0	0.0	0.0	0.0	0.0	0.0	0.0	0.0
LOC101928766	0.0	0.0	0.0	0.0	0.0	0.0	0.0	0.0	0.0
PCDHA6	0.1	0.0	0.0	0.0	0.0	0.0	0.0	0.0	0.0
LOC100130992	0.0	0.0	0.0	0.0	0.0	0.0	0.0	0.0	0.0
RASGRF2	0.0	0.0	0.0	0.0	0.0	0.0	0.0	0.0	0.0
CCDC37	0.0	0.0	0.0	0.0	0.0	0.0	0.0	0.0	0.0
KIF21B	0.0	0.0	0.0	0.0	0.0	0.0	0.0	0.0	0.0

SCN3A	0.0	0.0	0.0	0.0	0.0	0.0	0.0	0.0	0.0
FRY	0.0	0.0	0.0	0.0	0.0	0.0	0.0	0.0	0.0
LOC101929335	0.0	0.0	0.0	0.0	0.0	0.0	0.0	0.0	0.0
PCDHA7	0.0	0.0	0.0	0.0	0.0	0.0	0.0	0.0	0.0
KIAA0125	0.0	0.0	0.0	0.0	0.0	0.0	0.0	0.0	0.0
PCDHA9	0.0	0.0	0.0	0.0	0.0	0.0	0.0	0.0	0.0
PCDHA10	0.0	0.0	0.0	0.0	0.0	0.0	0.0	0.0	0.0
MUC17	0.0	0.0	0.0	0.0	0.0	0.0	0.0	0.0	0.0
TRNS2	4.4	7.0	5.4	4.1	2.4	3.8	5.0	0.0	5.3
SNORD50A	6.3	1.4	0.5	0.3	1.9	1.1	5.3	0.0	3.4
SNORD36A	2.2	2.9	2.2	1.1	1.0	0.4	3.3	0.0	2.7
SNORA50	3.6	1.9	1.4	0.1	0.5	1.9	3.9	0.0	1.8
SNORA45	3.2	1.9	1.1	0.3	0.3	0.4	3.3	0.0	0.7
AAMDC	3.4	1.3	1.7	0.1	0.4	0.8	1.9	0.0	1.6
SNORD27	0.6	1.4	3.0	0.6	0.5	0.8	1.2	0.0	2.7
SNORD117	1.0	1.7	1.0	0.8	1.9	1.5	1.6	0.0	1.1
PGAP3	2.7	1.0	1.3	0.2	0.9	1.2	1.3	0.0	1.1
ECHDC3	3.0	1.1	1.4	0.1	0.3	0.5	0.8	0.0	0.6
SNORD88B	2.6	0.3	0.9	0.0	0.0	0.0	2.1	0.0	1.5
SNORD83A	1.4	0.8	0.8	0.2	0.4	0.6	1.1	0.0	2.1
TFF3	3.1	0.8	1.0	0.1	0.4	0.2	1.0	0.0	0.3
LOC100507577	2.1	0.5	0.7	0.0	0.1	0.3	1.5	0.0	1.0
NREP	2.3	1.1	0.9	0.2	0.4	0.3	0.8	0.0	0.4
TXNL4B	1.8	0.6	0.6	0.0	0.2	0.3	1.8	0.0	0.8
GNG3	1.9	0.6	1.2	0.2	0.2	0.4	0.8	0.0	0.6
C6orf57	1.7	0.6	0.8	0.2	0.2	0.4	1.1	0.0	0.7
C5orf46	0.8	0.5	0.6	0.1	0.3	0.5	1.3	0.0	1.4
LOC101927255	1.5	0.8	1.2	0.3	0.3	0.5	0.3	0.0	0.6
ITM2A	1.9	0.9	1.1	0.0	0.4	0.4	0.3	0.0	0.1
LOC100130987	2.2	0.6	1.2	0.0	0.2	0.3	0.3	0.0	0.3
ZNF155	1.5	0.6	1.0	0.1	0.3	0.3	0.9	0.0	0.6
LOC101927621	1.7	0.3	1.3	0.3	0.3	0.2	0.3	0.0	0.6
SPRN	1.9	0.5	0.8	0.3	0.3	0.4	0.6	0.0	0.1
LOC100505549	1.6	0.6	0.8	0.1	0.2	0.2	0.6	0.0	0.5
TMEM107	1.2	0.5	0.8	0.3	0.3	0.6	0.5	0.0	0.5
TMEM14E	1.4	0.3	0.9	0.1	0.3	0.6	0.6	0.0	0.4
CASC10	0.2	0.2	0.2	0.1	0.5	0.5	1.6	0.0	0.9
ARTN	1.2	0.5	0.6	0.3	0.3	0.2	1.0	0.0	0.2
C17orf67	2.0	0.6	0.7	0.1	0.0	0.2	0.5	0.2	0.0
VPS37D	1.3	0.8	0.5	0.2	0.3	0.3	0.4	0.0	0.2
LCN2	2.2	0.9	0.7	0.1	0.1	0.0	0.0	0.0	0.0
TMEM88	1.0	0.5	1.2	0.1	0.2	0.1	0.2	0.0	0.6
TRNAE24	0.3	0.2	0.1	0.2	0.1	0.4	0.2	0.0	2.4
LOC100505624	0.8	0.4	0.7	0.0	0.0	0.5	0.6	0.0	0.9
IGIP	0.8	0.2	0.7	0.3	0.2	0.3	0.4	0.0	0.7
LOC101927963	1.2	0.3	0.3	0.1	0.2	0.1	0.6	0.0	0.8
RGS17	0.8	0.3	0.8	0.0	0.2	0.3	0.5	0.0	0.7
FLJ22447	1.6	0.6	0.7	0.0	0.1	0.1	0.3	0.0	0.2
LOC101929063	1.2	0.3	0.2	0.1	0.2	0.3	1.0	0.0	0.3
GGACT	1.0	0.2	0.3	0.3	0.5	0.2	0.6	0.0	0.4
LOC101928306	1.4	0.0	0.4	0.1	0.1	0.2	0.9	0.0	0.2
LOC101927696	0.7	0.4	0.8	0.1	0.0	0.1	0.7	0.0	0.6
TMEM198	1.1	0.4	0.5	0.1	0.2	0.3	0.4	0.0	0.3
EVA1B	0.9	0.4	0.5	0.1	0.1	0.3	0.5	0.0	0.4
LOC101928055	1.0	0.2	0.6	0.1	0.2	0.3	0.4	0.0	0.3

ZBED2	1.0	0.1	0.4	0.2	0.3	0.2	0.6	0.0	0.4
C15orf62	1.1	0.2	0.4	0.1	0.1	0.2	0.7	0.0	0.2
ZNF638-IT1	1.2	0.3	0.5	0.0	0.1	0.2	0.5	0.0	0.3
S100A1	1.2	0.8	0.5	0.1	0.0	0.3	0.0	0.0	0.2
C21orf88	0.9	0.3	0.3	0.2	0.1	0.2	0.7	0.0	0.5
CCDC159	0.9	0.5	0.5	0.1	0.2	0.3	0.4	0.0	0.1
CRISPLD1	0.8	0.3	0.6	0.0	0.1	0.2	0.5	0.0	0.4
CXADR	1.6	0.1	0.3	0.0	0.0	0.2	0.3	0.0	0.4
ZNF177	0.9	0.5	0.6	0.0	0.1	0.1	0.4	0.0	0.2
RASL11A	0.8	0.7	0.8	0.1	0.0	0.2	0.1	0.0	0.2
LOC101929580	1.5	0.4	0.3	0.1	0.1	0.1	0.2	0.0	0.4
FAM149B1	1.0	0.2	0.4	0.1	0.2	0.3	0.5	0.0	0.2
SENP8	0.9	0.3	0.3	0.1	0.3	0.4	0.3	0.0	0.3
ENOX2	1.0	0.3	0.3	0.1	0.1	0.3	0.6	0.0	0.3
LOC101928206	0.7	0.4	0.5	0.2	0.1	0.3	0.5	0.0	0.2
TMEM8B	1.3	0.3	0.5	0.1	0.1	0.2	0.1	0.0	0.1
C16orf3	1.0	0.2	0.7	0.1	0.1	0.2	0.3	0.0	0.1
C10orf95	0.8	0.1	0.4	0.1	0.3	0.3	0.5	0.0	0.3
LOC101929145	0.5	0.1	0.4	0.2	0.2	0.7	0.0	0.0	0.6
ZNF32-AS2	0.9	0.3	0.6	0.0	0.1	0.1	0.4	0.0	0.3
FAM85A	1.0	0.3	0.3	0.0	0.2	0.2	0.3	0.0	0.3
LOC101928772	0.8	0.2	0.5	0.1	0.4	0.3	0.1	0.0	0.3
DIO2	0.9	0.5	0.4	0.1	0.2	0.3	0.2	0.0	0.2
WTH3DI	0.6	0.3	0.4	0.0	0.2	0.2	0.5	0.0	0.4
GALNT9	0.6	0.5	0.4	0.1	0.1	0.0	0.6	0.0	0.4
NMRK1	0.6	0.2	0.6	0.0	0.2	0.3	0.4	0.0	0.3
OSCP1	0.8	0.4	0.5	0.1	0.1	0.3	0.3	0.0	0.1
COQ3	0.7	0.3	0.4	0.0	0.2	0.1	0.5	0.0	0.3
HLA-DRA	0.7	0.2	0.5	0.1	0.2	0.3	0.4	0.0	0.1
NAPSA	0.9	0.4	0.6	0.0	0.2	0.1	0.2	0.0	0.2
LINC00944	0.8	0.3	0.4	0.0	0.0	0.2	0.4	0.0	0.4
SGK110	0.9	0.5	0.4	0.1	0.1	0.2	0.2	0.0	0.1
ARGFX	0.8	0.2	0.3	0.1	0.1	0.1	0.6	0.0	0.2
GALR2	0.8	0.2	0.4	0.1	0.1	0.1	0.4	0.0	0.2
TAS2R5	0.6	0.1	0.4	0.0	0.1	0.2	0.5	0.0	0.4
TMEM178A	1.0	0.2	0.4	0.1	0.1	0.1	0.3	0.0	0.0
LOC101929666	0.6	0.2	0.3	0.0	0.2	0.2	0.5	0.0	0.3
FAM105A	0.9	0.2	0.2	0.0	0.2	0.3	0.2	0.0	0.3
NAT1	0.7	0.3	0.3	0.0	0.2	0.1	0.5	0.0	0.2
TMEM161B-AS1	0.6	0.3	0.5	0.2	0.0	0.2	0.3	0.0	0.2
ACTA2-AS1	0.7	0.2	0.3	0.1	0.2	0.1	0.4	0.0	0.2
LOC101928973	1.0	0.2	0.2	0.0	0.1	0.1	0.4	0.0	0.3
IGFBP5	0.6	0.2	0.3	0.2	0.2	0.4	0.2	0.0	0.2
SOX15	0.9	0.2	0.4	0.1	0.1	0.2	0.3	0.0	0.2
LOC101928979	0.5	0.1	0.3	0.1	0.1	0.1	0.7	0.0	0.3
CADM2	0.8	0.4	0.4	0.1	0.2	0.0	0.2	0.0	0.1
LOC101928429	0.8	0.1	0.5	0.1	0.1	0.2	0.2	0.0	0.1
CREB3L3	0.7	0.4	0.4	0.1	0.3	0.2	0.0	0.0	0.1
FAM27L	0.8	0.0	0.2	0.0	0.1	0.2	0.5	0.0	0.3
LOC101928570	0.3	0.3	0.0	0.1	0.2	0.3	0.5	0.0	0.5
TMEM86A	0.7	0.4	0.4	0.1	0.2	0.0	0.2	0.0	0.1
CLEC3B	0.8	0.1	0.3	0.2	0.1	0.2	0.4	0.0	0.1
LOC100506457	0.3	0.1	0.5	0.1	0.2	0.4	0.2	0.0	0.2
LOC100289092	0.5	0.2	0.3	0.0	0.0	0.3	0.3	0.0	0.4
TTC30A	0.6	0.2	0.3	0.1	0.2	0.3	0.2	0.0	0.1

ZNF571	0.7	0.2	0.3	0.0	0.1	0.1	0.4	0.0	0.3
LOC100506753	0.3	0.3	0.4	0.0	0.0	0.3	0.4	0.0	0.3
CPA3	0.2	0.1	0.2	0.0	0.4	0.2	0.3	0.0	0.5
N6AMT2	0.5	0.3	0.2	0.0	0.2	0.1	0.4	0.0	0.3
LOC101927117	0.7	0.3	0.3	0.0	0.1	0.2	0.2	0.0	0.2
PTN	0.8	0.2	0.4	0.1	0.2	0.1	0.3	0.1	0.0
LOC100499183	0.6	0.1	0.2	0.1	0.2	0.1	0.4	0.0	0.2
TMEFF1	0.9	0.1	0.1	0.1	0.0	0.0	0.5	0.0	0.3
LOC101927712	0.6	0.3	0.2	0.0	0.1	0.1	0.4	0.0	0.3
NDP	0.4	0.2	0.2	0.1	0.3	0.1	0.5	0.0	0.2
ASAP1-IT1	0.6	0.1	0.3	0.0	0.1	0.1	0.5	0.0	0.3
ASPRV1	0.5	0.2	0.3	0.0	0.1	0.2	0.5	0.0	0.2
NPY1R	0.7	0.3	0.3	0.1	0.1	0.0	0.3	0.0	0.2
LOC100506718	0.8	0.1	0.3	0.0	0.0	0.1	0.3	0.0	0.2
TAS2R4	0.6	0.1	0.4	0.0	0.1	0.3	0.2	0.0	0.2
SLCO1B7	0.7	0.2	0.3	0.0	0.1	0.1	0.4	0.0	0.2
MIA-RAB4B	0.3	0.1	0.5	0.0	0.1	0.2	0.5	0.0	0.2
FGF1	0.3	0.0	0.1	0.1	0.3	0.2	0.6	0.0	0.4
LOC101926941	0.5	0.2	0.3	0.1	0.3	0.1	0.2	0.0	0.2
FOXH1	0.6	0.1	0.3	0.1	0.0	0.3	0.2	0.0	0.1
LOC101928304	0.3	0.2	0.3	0.1	0.1	0.4	0.3	0.0	0.2
LOC101929271	0.7	0.2	0.2	0.1	0.0	0.1	0.4	0.0	0.2
ZBED3-AS1	0.5	0.2	0.2	0.0	0.2	0.1	0.6	0.0	0.1
SMKR1	0.6	0.1	0.3	0.0	0.4	0.1	0.0	0.0	0.2
LOC101927305	1.0	0.2	0.1	0.1	0.0	0.1	0.2	0.0	0.1
IL1R1	0.8	0.2	0.3	0.0	0.1	0.2	0.1	0.0	0.1
LOC101928793	0.6	0.1	0.4	0.0	0.0	0.1	0.5	0.0	0.1
FOXD4L3	0.8	0.2	0.5	0.0	0.0	0.0	0.0	0.0	0.2
PITX3	0.6	0.3	0.2	0.1	0.1	0.1	0.2	0.0	0.1
CDK5R2	0.5	0.2	0.2	0.1	0.1	0.1	0.3	0.0	0.2
TMEM191B	1.1	0.2	0.4	0.0	0.0	0.0	0.0	0.0	0.1
KREMEN1	0.6	0.2	0.5	0.0	0.2	0.1	0.0	0.0	0.2
LOC100130476	0.6	0.2	0.3	0.0	0.1	0.1	0.3	0.0	0.2
LOC101927986	0.3	0.1	0.5	0.1	0.2	0.3	0.2	0.0	0.2
CCNI2	0.4	0.3	0.3	0.1	0.0	0.1	0.3	0.0	0.2
GLTPD2	0.3	0.3	0.4	0.1	0.1	0.3	0.1	0.0	0.2
CEACAM16	0.8	0.3	0.4	0.1	0.0	0.1	0.0	0.0	0.0
CHRNG	0.4	0.3	0.3	0.1	0.1	0.1	0.2	0.0	0.2
CA3	0.5	0.2	0.2	0.1	0.1	0.0	0.4	0.0	0.3
CHN2	0.5	0.2	0.2	0.0	0.2	0.1	0.2	0.0	0.3
LOC101928749	0.3	0.1	0.1	0.1	0.1	0.2	0.4	0.0	0.5
LOC101929642	0.7	0.2	0.1	0.0	0.1	0.1	0.2	0.0	0.1
MMP23B	0.5	0.3	0.2	0.1	0.1	0.1	0.2	0.0	0.2
LOC101927343	0.8	0.1	0.3	0.1	0.0	0.1	0.2	0.0	0.2
CHKB-AS1	0.7	0.2	0.3	0.0	0.0	0.0	0.3	0.0	0.1
B3GNT8	0.4	0.1	0.3	0.1	0.2	0.1	0.2	0.0	0.3
HIST3H3	0.5	0.2	0.3	0.1	0.0	0.2	0.1	0.0	0.1
MPZL2	0.6	0.2	0.5	0.0	0.1	0.1	0.1	0.0	0.1
CYTH4	0.6	0.2	0.4	0.0	0.1	0.1	0.1	0.0	0.1
MYLK4	0.4	0.3	0.4	0.1	0.1	0.1	0.2	0.0	0.1
LOC101927716	0.6	0.2	0.2	0.0	0.2	0.0	0.3	0.0	0.0
LINC00526	0.2	0.2	0.3	0.1	0.2	0.2	0.3	0.0	0.1
MMP9	0.5	0.2	0.3	0.1	0.1	0.1	0.1	0.0	0.2
NSUN7	0.4	0.2	0.2	0.0	0.1	0.1	0.3	0.0	0.2
LOC643669	0.5	0.2	0.3	0.0	0.2	0.1	0.1	0.0	0.1

LOC101927208	0.5	0.1	0.2	0.0	0.0	0.1	0.3	0.0	0.3
CYP2J2	0.3	0.2	0.1	0.1	0.1	0.1	0.4	0.0	0.2
SPOCK2	0.6	0.1	0.4	0.0	0.0	0.1	0.2	0.0	0.1
LOC101059954	0.7	0.1	0.2	0.0	0.1	0.2	0.1	0.0	0.1
LOC101929060	0.5	0.1	0.2	0.0	0.1	0.0	0.5	0.0	0.1
LOC100996405	0.5	0.1	0.2	0.1	0.1	0.1	0.2	0.0	0.2
KBTBD8	0.4	0.1	0.2	0.0	0.1	0.0	0.3	0.0	0.3
TRNAG2	0.2	0.2	0.2	0.1	0.3	0.1	0.1	0.0	0.2
TRNAG16	0.2	0.2	0.2	0.1	0.3	0.1	0.1	0.0	0.2
TRNAG6	0.2	0.2	0.2	0.1	0.3	0.1	0.1	0.0	0.2
TRNAG29	0.2	0.2	0.2	0.1	0.3	0.1	0.1	0.0	0.2
TRNAG19	0.2	0.2	0.2	0.1	0.3	0.1	0.1	0.0	0.2
TRNAG31	0.2	0.2	0.2	0.1	0.3	0.1	0.1	0.0	0.2
LOC101928886	0.4	0.1	0.3	0.0	0.1	0.1	0.3	0.0	0.2
LOC101928497	0.7	0.1	0.2	0.0	0.0	0.1	0.3	0.0	0.1
SLC26A7	0.5	0.2	0.3	0.0	0.1	0.0	0.2	0.0	0.2
LOC105379417	0.2	0.1	0.3	0.0	0.2	0.0	0.2	0.0	0.3
GPR182	0.6	0.1	0.3	0.1	0.0	0.1	0.1	0.0	0.2
CCDC96	0.3	0.2	0.2	0.1	0.1	0.2	0.3	0.0	0.2
DUX4L	0.4	0.2	0.2	0.0	0.0	0.1	0.3	0.0	0.1
LOC400752	0.5	0.1	0.2	0.0	0.1	0.1	0.3	0.0	0.2
FNDC5	0.5	0.2	0.2	0.0	0.1	0.1	0.2	0.0	0.1
KCNJ13	0.5	0.1	0.2	0.1	0.1	0.1	0.3	0.0	0.2
AMDHD1	0.4	0.2	0.3	0.0	0.1	0.1	0.1	0.0	0.2
TP53TG1	0.2	0.3	0.2	0.1	0.3	0.1	0.2	0.0	0.1
LINC00862	0.4	0.1	0.2	0.1	0.1	0.1	0.4	0.0	0.1
B4GALNT3	0.6	0.2	0.2	0.0	0.1	0.0	0.1	0.0	0.1
LOC101927724	0.6	0.2	0.4	0.0	0.1	0.1	0.1	0.0	0.0
MPP1	0.4	0.2	0.3	0.0	0.0	0.0	0.2	0.0	0.1
PANX3	0.3	0.1	0.2	0.0	0.1	0.1	0.4	0.0	0.2
UCN2	0.2	0.0	0.2	0.1	0.1	0.5	0.0	0.0	0.3
TMOD4	0.4	0.1	0.1	0.0	0.0	0.1	0.2	0.0	0.3
FLJ35024	0.4	0.1	0.2	0.0	0.0	0.1	0.1	0.0	0.3
LOC101927396	0.2	0.1	0.2	0.1	0.1	0.1	0.4	0.0	0.2
HSPB6	0.3	0.2	0.2	0.1	0.1	0.1	0.3	0.0	0.2
EHHADH-AS1	0.6	0.1	0.2	0.0	0.1	0.1	0.1	0.0	0.0
CBY3	0.5	0.1	0.1	0.1	0.0	0.1	0.3	0.0	0.0
PCDHGA1	0.4	0.2	0.3	0.1	0.1	0.1	0.1	0.0	0.1
OR8A1	0.4	0.1	0.2	0.0	0.3	0.1	0.1	0.0	0.1
LRIT3	0.5	0.1	0.2	0.0	0.0	0.1	0.3	0.0	0.1
SLC9B1	0.4	0.1	0.1	0.0	0.0	0.1	0.4	0.0	0.1
TTYH2	0.3	0.0	0.2	0.0	0.0	0.2	0.3	0.0	0.2
LINC00313	0.4	0.0	0.2	0.1	0.1	0.3	0.1	0.0	0.1
IDH1-AS1	0.3	0.1	0.2	0.1	0.3	0.0	0.3	0.0	0.0
LOC101928909	0.4	0.1	0.2	0.0	0.1	0.1	0.3	0.0	0.2
DDO	0.4	0.1	0.3	0.1	0.1	0.1	0.1	0.0	0.1
LINC00320	0.5	0.1	0.2	0.0	0.1	0.1	0.2	0.0	0.1
PRSS37	0.4	0.1	0.1	0.0	0.1	0.1	0.4	0.0	0.0
OAS2	0.4	0.3	0.3	0.0	0.1	0.1	0.1	0.0	0.0
FGL2	0.5	0.1	0.2	0.0	0.1	0.0	0.2	0.0	0.1
KRTCAP3	0.4	0.1	0.2	0.1	0.1	0.0	0.2	0.0	0.2
AGAP1-IT1	0.5	0.0	0.2	0.1	0.1	0.1	0.2	0.0	0.0
DHRS4L2	0.4	0.1	0.3	0.0	0.0	0.1	0.1	0.0	0.2
LOC101928025	0.6	0.1	0.1	0.0	0.1	0.0	0.2	0.0	0.1
LOC101929083	0.5	0.1	0.1	0.0	0.1	0.1	0.2	0.0	0.2

F8	0.5	0.1	0.1	0.0	0.0	0.1	0.3	0.0	0.1
LOC101927507	0.5	0.1	0.2	0.0	0.1	0.1	0.1	0.0	0.1
DNAJA4	0.2	0.1	0.2	0.1	0.0	0.1	0.3	0.0	0.3
LOC101927709	0.4	0.1	0.1	0.0	0.0	0.0	0.3	0.0	0.2
LOC101929736	0.5	0.1	0.2	0.0	0.1	0.1	0.2	0.0	0.1
LOC388849	0.5	0.1	0.2	0.1	0.0	0.1	0.1	0.0	0.1
GPR85	0.1	0.1	0.1	0.0	0.1	0.1	0.4	0.0	0.3
MAB21L2	0.4	0.1	0.2	0.1	0.1	0.1	0.2	0.0	0.1
ZSCAN23	0.4	0.1	0.1	0.0	0.1	0.1	0.2	0.0	0.2
SERPING1	0.4	0.3	0.2	0.0	0.0	0.1	0.1	0.0	0.0
FAM27E2	0.5	0.0	0.2	0.1	0.1	0.1	0.1	0.0	0.1
IZUMO4	0.3	0.2	0.3	0.0	0.1	0.1	0.1	0.0	0.1
LOC101928873	0.5	0.1	0.0	0.0	0.1	0.0	0.2	0.0	0.2
LOC400043	0.4	0.0	0.2	0.0	0.1	0.2	0.2	0.0	0.1
EPN2-IT1	0.4	0.1	0.1	0.0	0.1	0.0	0.3	0.0	0.2
LOC101927858	0.3	0.1	0.2	0.0	0.1	0.0	0.3	0.0	0.2
LNP1	0.5	0.1	0.1	0.1	0.0	0.2	0.1	0.0	0.1
PHF7	0.3	0.0	0.2	0.0	0.1	0.1	0.2	0.0	0.2
GPR21	0.3	0.1	0.2	0.1	0.1	0.1	0.1	0.0	0.1
LOC100652856	0.6	0.0	0.1	0.0	0.0	0.1	0.2	0.0	0.1
AGT	0.5	0.3	0.2	0.0	0.0	0.0	0.1	0.0	0.0
CHRNA3	0.4	0.1	0.1	0.0	0.1	0.1	0.2	0.0	0.1
BEAN1	0.3	0.2	0.2	0.1	0.0	0.1	0.2	0.0	0.0
CACNB2	0.4	0.1	0.2	0.0	0.1	0.0	0.1	0.0	0.1
CLDND2	0.2	0.2	0.2	0.1	0.2	0.1	0.2	0.0	0.1
ZNF233	0.4	0.0	0.3	0.0	0.1	0.0	0.2	0.0	0.1
GPR160	0.3	0.0	0.2	0.0	0.1	0.1	0.3	0.0	0.1
CDC25C	0.2	0.1	0.2	0.0	0.1	0.1	0.2	0.0	0.2
HPCA	0.2	0.1	0.1	0.0	0.1	0.0	0.3	0.0	0.2
ZNF350	0.4	0.1	0.2	0.0	0.1	0.1	0.1	0.0	0.0
ZNF684	0.2	0.1	0.3	0.0	0.1	0.1	0.2	0.0	0.2
DDX26B	0.3	0.1	0.2	0.0	0.0	0.1	0.2	0.0	0.2
C2orf61	0.4	0.1	0.3	0.0	0.0	0.1	0.2	0.0	0.0
SOX30	0.4	0.2	0.2	0.0	0.1	0.0	0.1	0.0	0.1
LOC100505942	0.4	0.0	0.4	0.0	0.1	0.0	0.1	0.0	0.1
OR5K2	0.4	0.1	0.1	0.0	0.1	0.1	0.1	0.0	0.1
MACROD2	0.2	0.2	0.1	0.0	0.2	0.1	0.1	0.0	0.2
PRKG1-AS1	0.2	0.1	0.2	0.0	0.1	0.1	0.2	0.0	0.1
ZNF165	0.3	0.2	0.1	0.0	0.0	0.0	0.1	0.0	0.2
TMPRSS5	0.2	0.1	0.2	0.0	0.0	0.1	0.2	0.0	0.1
SLC16A6	0.3	0.0	0.1	0.0	0.1	0.1	0.2	0.0	0.1
LOC101060169	0.3	0.1	0.3	0.0	0.0	0.1	0.2	0.0	0.1
CACNG6	0.2	0.2	0.0	0.1	0.2	0.2	0.1	0.0	0.1
SPON1	0.4	0.1	0.2	0.0	0.1	0.0	0.1	0.0	0.1
CAMK1G	0.3	0.1	0.1	0.0	0.1	0.2	0.1	0.0	0.1
FLVCR1-AS1	0.1	0.1	0.1	0.0	0.1	0.0	0.3	0.0	0.2
GTSE1-AS1	0.2	0.2	0.1	0.0	0.2	0.1	0.1	0.0	0.1
FBLN1	0.2	0.0	0.1	0.1	0.1	0.1	0.2	0.0	0.0
ANGPTL1	0.3	0.1	0.1	0.0	0.0	0.1	0.3	0.0	0.1
WNT8B	0.2	0.1	0.3	0.1	0.0	0.1	0.2	0.0	0.0
RAPGEF4	0.4	0.1	0.2	0.0	0.0	0.0	0.1	0.0	0.0
ARHGAP25	0.3	0.1	0.1	0.0	0.1	0.1	0.1	0.0	0.1
LOC283214	0.4	0.0	0.1	0.0	0.1	0.1	0.2	0.0	0.1
PMEL	0.4	0.2	0.1	0.0	0.1	0.0	0.1	0.0	0.0
ANGPT2	0.4	0.0	0.1	0.0	0.0	0.1	0.2	0.0	0.1

TPPP	0.3	0.0	0.1	0.1	0.1	0.1	0.1	0.0	0.1
LOC101927543	0.4	0.0	0.1	0.0	0.0	0.1	0.1	0.0	0.1
LOC101929005	0.4	0.1	0.1	0.0	0.0	0.1	0.1	0.0	0.1
TCL1A	0.3	0.1	0.1	0.0	0.0	0.1	0.2	0.0	0.0
LOC100507487	0.3	0.0	0.2	0.0	0.0	0.0	0.3	0.0	0.1
SPARC	0.2	0.1	0.0	0.0	0.1	0.0	0.3	0.0	0.2
LOC101243545	0.1	0.2	0.2	0.0	0.0	0.1	0.1	0.0	0.1
LINC00900	0.4	0.0	0.1	0.0	0.0	0.0	0.1	0.0	0.1
LOC101927290	0.2	0.1	0.3	0.0	0.0	0.1	0.1	0.0	0.0
ABCC6	0.5	0.1	0.1	0.0	0.0	0.0	0.1	0.0	0.0
GNB3	0.2	0.1	0.1	0.1	0.1	0.1	0.1	0.0	0.1
ATP2C2	0.2	0.1	0.1	0.0	0.1	0.1	0.1	0.0	0.1
LOC100616530	0.1	0.0	0.0	0.1	0.1	0.2	0.3	0.0	0.1
C9orf131	0.2	0.0	0.1	0.0	0.1	0.0	0.3	0.0	0.1
TMPRSS11D	0.4	0.1	0.1	0.0	0.0	0.0	0.1	0.0	0.1
WNT9A	0.4	0.1	0.1	0.0	0.0	0.1	0.1	0.0	0.1
LOC101929191	0.3	0.0	0.1	0.0	0.0	0.0	0.2	0.0	0.1
EFCC1	0.3	0.1	0.2	0.1	0.0	0.1	0.0	0.0	0.0
SYCP3	0.4	0.1	0.1	0.0	0.0	0.1	0.2	0.0	0.0
LOC101928032	0.2	0.1	0.1	0.0	0.1	0.1	0.1	0.0	0.2
LOC101929760	0.3	0.0	0.1	0.0	0.0	0.0	0.2	0.0	0.1
PLCE1-AS1	0.1	0.1	0.3	0.0	0.0	0.1	0.1	0.0	0.1
ZBTB7C	0.2	0.1	0.1	0.0	0.0	0.1	0.1	0.0	0.2
PATL2	0.2	0.1	0.2	0.0	0.1	0.1	0.1	0.0	0.0
TMEM163	0.2	0.0	0.3	0.0	0.0	0.1	0.1	0.0	0.1
LOC101928576	0.2	0.0	0.2	0.0	0.0	0.1	0.1	0.0	0.2
NANOG	0.3	0.0	0.1	0.0	0.1	0.0	0.2	0.0	0.0
LOC729461	0.2	0.1	0.2	0.0	0.1	0.0	0.2	0.0	0.0
LOC101927086	0.3	0.2	0.1	0.0	0.1	0.1	0.1	0.0	0.0
BEST3	0.3	0.0	0.0	0.0	0.0	0.1	0.2	0.0	0.2
ALG1L2	0.3	0.0	0.1	0.0	0.2	0.1	0.1	0.0	0.0
FAM155A-IT1	0.3	0.1	0.1	0.0	0.0	0.1	0.2	0.0	0.1
CPM	0.2	0.1	0.2	0.0	0.0	0.2	0.1	0.0	0.0
LOC101929505	0.4	0.0	0.1	0.0	0.0	0.0	0.1	0.0	0.1
LOC101928111	0.2	0.1	0.0	0.0	0.1	0.1	0.1	0.0	0.1
LOC101927854	0.1	0.2	0.2	0.0	0.1	0.0	0.0	0.0	0.2
SHF	0.1	0.1	0.3	0.0	0.1	0.0	0.0	0.0	0.1
PRSS22	0.4	0.1	0.1	0.0	0.0	0.0	0.0	0.0	0.0
FRRS1	0.2	0.1	0.1	0.0	0.0	0.0	0.1	0.0	0.1
GALR3	0.1	0.1	0.0	0.1	0.1	0.1	0.1	0.0	0.1
SLA2	0.3	0.1	0.1	0.0	0.0	0.0	0.1	0.0	0.2
LOC100996290	0.2	0.1	0.1	0.0	0.2	0.0	0.1	0.0	0.1
GUCY1A2	0.2	0.1	0.2	0.0	0.0	0.1	0.1	0.0	0.1
PPIAL4G	0.2	0.0	0.1	0.0	0.0	0.0	0.4	0.0	0.1
LOC101929746	0.2	0.2	0.1	0.0	0.1	0.0	0.1	0.0	0.1
LOC101929473	0.3	0.0	0.1	0.0	0.0	0.0	0.2	0.0	0.1
BEST2	0.2	0.2	0.1	0.0	0.0	0.1	0.1	0.0	0.1
OR8D1	0.4	0.0	0.1	0.1	0.0	0.1	0.0	0.0	0.0
TMEM121	0.2	0.1	0.0	0.0	0.1	0.1	0.2	0.0	0.1
RASGRP3	0.2	0.1	0.1	0.0	0.0	0.0	0.1	0.0	0.1
HLA-DRB5	0.1	0.0	0.1	0.1	0.1	0.0	0.2	0.0	0.2
MMP21	0.3	0.1	0.1	0.0	0.0	0.1	0.0	0.0	0.1
PTGDR2	0.1	0.0	0.2	0.1	0.1	0.0	0.1	0.0	0.0
PTH1R	0.2	0.1	0.1	0.0	0.0	0.0	0.1	0.0	0.1
AEBP1	0.3	0.1	0.1	0.0	0.0	0.0	0.1	0.0	0.1

LOC101929284	0.3	0.1	0.2	0.0	0.0	0.0	0.1	0.0	0.0
TRIM6	0.3	0.1	0.1	0.0	0.0	0.1	0.1	0.1	0.0
HEPACAM	0.3	0.1	0.1	0.0	0.0	0.0	0.1	0.0	0.1
SGPP2	0.2	0.1	0.0	0.0	0.1	0.1	0.1	0.0	0.1
LOC101926919	0.4	0.0	0.0	0.0	0.0	0.0	0.1	0.0	0.1
SYT11	0.3	0.1	0.1	0.0	0.1	0.1	0.0	0.0	0.1
LOC101929595	0.2	0.1	0.1	0.0	0.0	0.0	0.1	0.0	0.1
HOXB2	0.1	0.2	0.1	0.0	0.0	0.1	0.1	0.0	0.1
LINC00427	0.2	0.0	0.1	0.0	0.2	0.1	0.1	0.0	0.1
LINC00645	0.3	0.1	0.1	0.0	0.0	0.0	0.1	0.0	0.1
C19orf67	0.3	0.0	0.1	0.0	0.0	0.0	0.1	0.0	0.1
SCRT1	0.2	0.0	0.1	0.0	0.0	0.0	0.1	0.0	0.1
LINC00271	0.2	0.1	0.1	0.0	0.0	0.1	0.1	0.0	0.1
DDX11-AS1	0.1	0.1	0.1	0.0	0.1	0.1	0.1	0.0	0.1
SEPP1	0.2	0.0	0.1	0.0	0.0	0.0	0.2	0.0	0.1
PRSS54	0.3	0.1	0.1	0.0	0.0	0.0	0.1	0.0	0.1
SLC29A4	0.3	0.1	0.2	0.0	0.0	0.0	0.0	0.0	0.0
ZDHHC15	0.2	0.1	0.1	0.0	0.0	0.1	0.1	0.0	0.1
FGF22	0.1	0.0	0.1	0.1	0.1	0.0	0.2	0.0	0.1
RNF150	0.3	0.1	0.1	0.1	0.0	0.0	0.0	0.0	0.0
C12orf36	0.3	0.0	0.1	0.0	0.0	0.0	0.1	0.0	0.1
FBXO24	0.2	0.1	0.1	0.0	0.0	0.0	0.1	0.0	0.0
HKDC1	0.2	0.1	0.2	0.0	0.0	0.1	0.1	0.0	0.0
TEKT3	0.2	0.0	0.1	0.0	0.0	0.1	0.2	0.0	0.1
GAS2	0.3	0.1	0.1	0.0	0.1	0.0	0.0	0.0	0.1
C19orf81	0.1	0.2	0.1	0.0	0.0	0.0	0.2	0.0	0.1
PSG7	0.2	0.0	0.1	0.0	0.1	0.1	0.1	0.0	0.1
LOC401317	0.2	0.1	0.1	0.0	0.1	0.1	0.0	0.0	0.0
OPN1SW	0.4	0.1	0.0	0.0	0.0	0.0	0.0	0.0	0.1
FBLN7	0.2	0.1	0.1	0.1	0.0	0.1	0.0	0.0	0.0
SPATA17	0.1	0.0	0.2	0.0	0.0	0.1	0.0	0.0	0.1
PP14571	0.2	0.0	0.1	0.0	0.0	0.0	0.1	0.0	0.1
C6orf118	0.1	0.1	0.1	0.0	0.0	0.0	0.2	0.0	0.1
LOC101927002	0.2	0.0	0.1	0.0	0.0	0.1	0.2	0.0	0.0
LOC101927667	0.3	0.0	0.1	0.0	0.0	0.0	0.1	0.0	0.1
LOC285629	0.2	0.1	0.1	0.0	0.0	0.0	0.1	0.0	0.1
MUSK	0.1	0.1	0.1	0.0	0.1	0.1	0.1	0.0	0.1
ARHGEF33	0.3	0.1	0.1	0.0	0.0	0.0	0.0	0.0	0.1
LOC101927950	0.1	0.0	0.1	0.0	0.0	0.1	0.1	0.0	0.0
CACNA1G-AS1	0.2	0.1	0.0	0.0	0.1	0.1	0.1	0.0	0.0
LOC101927189	0.3	0.0	0.0	0.0	0.0	0.0	0.2	0.0	0.0
FCGR2C	0.2	0.0	0.1	0.0	0.0	0.0	0.1	0.0	0.0
HCAR3	0.2	0.0	0.2	0.0	0.0	0.0	0.1	0.0	0.0
TTLL9	0.1	0.0	0.1	0.1	0.0	0.0	0.2	0.0	0.0
LRG1	0.2	0.1	0.1	0.0	0.0	0.0	0.1	0.0	0.1
LOC100505736	0.2	0.1	0.1	0.0	0.0	0.0	0.1	0.0	0.0
LOC653160	0.2	0.1	0.0	0.0	0.1	0.1	0.1	0.0	0.1
ZNF418	0.2	0.1	0.1	0.0	0.0	0.0	0.1	0.0	0.0
UPP2	0.2	0.0	0.1	0.0	0.0	0.0	0.1	0.0	0.0
FAM151A	0.1	0.1	0.0	0.0	0.1	0.1	0.1	0.0	0.1
LOC101927511	0.2	0.1	0.0	0.1	0.1	0.0	0.1	0.0	0.1
LOC101927539	0.2	0.0	0.1	0.0	0.0	0.0	0.1	0.0	0.1
ARHGAP20	0.3	0.1	0.1	0.0	0.0	0.0	0.1	0.0	0.0
LOC100996368	0.2	0.1	0.2	0.0	0.0	0.0	0.0	0.0	0.0
FAM9A	0.1	0.0	0.1	0.1	0.0	0.0	0.1	0.0	0.1

VSIG8	0.2	0.1	0.1	0.0	0.1	0.0	0.0	0.0	0.0
ST8SIA5	0.2	0.0	0.1	0.1	0.0	0.1	0.1	0.0	0.1
LOC100507534	0.1	0.1	0.1	0.0	0.0	0.1	0.1	0.0	0.1
TSPAN32	0.2	0.1	0.1	0.0	0.0	0.0	0.1	0.0	0.1
EGR2	0.2	0.1	0.1	0.0	0.0	0.0	0.0	0.0	0.1
LOC391322	0.0	0.1	0.1	0.0	0.0	0.2	0.0	0.0	0.2
ALKBH3-AS1	0.2	0.1	0.1	0.0	0.0	0.0	0.1	0.0	0.0
LOC101927394	0.2	0.0	0.1	0.0	0.0	0.0	0.1	0.0	0.0
LOC101927583	0.2	0.0	0.0	0.0	0.0	0.0	0.1	0.0	0.1
VN1R4	0.2	0.0	0.1	0.0	0.1	0.1	0.0	0.0	0.1
HAPLN2	0.2	0.1	0.0	0.0	0.0	0.1	0.1	0.0	0.0
FAR2	0.2	0.0	0.2	0.0	0.0	0.0	0.0	0.0	0.0
SLC46A2	0.1	0.0	0.1	0.0	0.0	0.0	0.1	0.0	0.1
NR2E3	0.3	0.0	0.0	0.0	0.1	0.0	0.1	0.0	0.1
LOC285847	0.1	0.1	0.1	0.0	0.0	0.1	0.1	0.0	0.0
LOC101928595	0.2	0.1	0.1	0.0	0.0	0.0	0.0	0.0	0.1
EXOC3L1	0.2	0.0	0.2	0.0	0.1	0.0	0.0	0.0	0.0
LOC101928433	0.2	0.1	0.1	0.0	0.0	0.0	0.0	0.0	0.1
LOC101927207	0.2	0.0	0.1	0.0	0.0	0.0	0.1	0.0	0.1
WDR78	0.2	0.1	0.1	0.0	0.0	0.1	0.0	0.0	0.1
IFI44L	0.1	0.0	0.1	0.0	0.1	0.1	0.1	0.0	0.1
TMEM253	0.2	0.1	0.1	0.0	0.1	0.0	0.1	0.0	0.0
KCNIP4	0.2	0.0	0.1	0.0	0.0	0.0	0.1	0.0	0.1
LOC254028	0.1	0.0	0.1	0.0	0.0	0.0	0.1	0.0	0.0
ARR3	0.1	0.0	0.0	0.1	0.1	0.0	0.1	0.0	0.0
ZFX-AS1	0.1	0.0	0.1	0.0	0.0	0.0	0.1	0.0	0.1
DBH	0.2	0.1	0.0	0.0	0.0	0.1	0.0	0.0	0.0
GPR156	0.2	0.1	0.1	0.0	0.0	0.0	0.1	0.0	0.1
LOC100506258	0.2	0.0	0.1	0.0	0.0	0.0	0.1	0.0	0.0
LOC101926977	0.1	0.0	0.1	0.0	0.0	0.0	0.1	0.0	0.0
DKK3	0.1	0.0	0.0	0.0	0.0	0.1	0.1	0.0	0.1
SLC22A7	0.2	0.0	0.1	0.0	0.0	0.0	0.1	0.0	0.1
ABCG4	0.2	0.1	0.1	0.0	0.0	0.0	0.1	0.0	0.0
FOXP3	0.2	0.1	0.1	0.0	0.0	0.0	0.0	0.0	0.0
ARHGAP44	0.2	0.0	0.1	0.0	0.0	0.1	0.0	0.0	0.0
LOC101929583	0.1	0.0	0.1	0.0	0.0	0.0	0.1	0.0	0.1
LOC643797	0.1	0.0	0.0	0.0	0.1	0.1	0.0	0.0	0.0
BRWD1-IT2	0.1	0.1	0.1	0.0	0.0	0.0	0.0	0.0	0.1
MYADML2	0.1	0.0	0.1	0.0	0.1	0.1	0.1	0.0	0.0
SLC4A9	0.3	0.0	0.1	0.0	0.0	0.0	0.0	0.0	0.0
KLHL13	0.1	0.0	0.1	0.0	0.0	0.1	0.0	0.0	0.0
NAV2-AS5	0.1	0.0	0.1	0.0	0.0	0.0	0.1	0.0	0.1
LOC100507651	0.2	0.0	0.1	0.0	0.0	0.0	0.1	0.0	0.0
COL10A1	0.2	0.0	0.1	0.0	0.0	0.0	0.0	0.0	0.0
HEPH	0.1	0.1	0.1	0.0	0.0	0.0	0.1	0.0	0.0
LOC101928871	0.1	0.1	0.1	0.0	0.0	0.0	0.0	0.0	0.0
LOC101927200	0.1	0.1	0.1	0.0	0.0	0.0	0.1	0.0	0.0
ZNF681	0.1	0.1	0.1	0.0	0.0	0.0	0.0	0.0	0.1
APCDD1L-AS1	0.1	0.0	0.1	0.0	0.0	0.0	0.1	0.0	0.0
ZNF541	0.0	0.0	0.0	0.0	0.0	0.1	0.1	0.0	0.1
ENTPD8	0.1	0.0	0.1	0.0	0.1	0.0	0.0	0.0	0.1
LRGUK	0.2	0.1	0.1	0.0	0.0	0.0	0.1	0.0	0.0
CXCL17	0.0	0.1	0.1	0.0	0.1	0.0	0.1	0.0	0.0
LOC101928205	0.2	0.0	0.1	0.0	0.0	0.0	0.0	0.0	0.0
HYAL4	0.1	0.1	0.1	0.0	0.1	0.0	0.0	0.0	0.0

C3orf20	0.1	0.0	0.1	0.0	0.0	0.0	0.1	0.0	0.0
FAM132A	0.1	0.1	0.0	0.0	0.0	0.0	0.1	0.0	0.1
LOC153910	0.1	0.0	0.0	0.0	0.0	0.0	0.1	0.0	0.0
SLC4A4	0.1	0.0	0.1	0.0	0.0	0.0	0.0	0.0	0.0
JPH3	0.1	0.0	0.1	0.0	0.0	0.1	0.1	0.0	0.0
NPSR1-AS1	0.2	0.0	0.0	0.0	0.0	0.0	0.1	0.0	0.0
SORCS1	0.1	0.1	0.1	0.0	0.0	0.0	0.1	0.0	0.0
LOC285889	0.1	0.0	0.0	0.0	0.0	0.0	0.1	0.0	0.0
LOC100128714	0.1	0.0	0.0	0.0	0.0	0.0	0.1	0.0	0.0
TDO2	0.1	0.0	0.0	0.0	0.0	0.1	0.0	0.0	0.0
TLR5	0.1	0.0	0.0	0.0	0.1	0.0	0.0	0.0	0.0
AQPEP	0.1	0.0	0.1	0.0	0.0	0.0	0.0	0.0	0.0
OCA2	0.1	0.0	0.1	0.0	0.1	0.0	0.1	0.0	0.0
RGAG1	0.1	0.0	0.1	0.0	0.0	0.0	0.1	0.0	0.1
CGB2	0.0	0.1	0.0	0.0	0.0	0.0	0.1	0.0	0.0
ALS2CR11	0.1	0.0	0.1	0.0	0.0	0.0	0.1	0.0	0.1
PLXDC1	0.1	0.1	0.1	0.0	0.0	0.0	0.0	0.0	0.0
MYO15A	0.1	0.0	0.1	0.0	0.0	0.0	0.0	0.0	0.0
PLXNA4	0.1	0.0	0.0	0.0	0.1	0.1	0.0	0.0	0.0
CATSPERG	0.1	0.0	0.1	0.0	0.0	0.0	0.0	0.0	0.0
USP51	0.1	0.0	0.0	0.0	0.0	0.0	0.1	0.0	0.0
FLJ30679	0.2	0.0	0.0	0.0	0.0	0.0	0.0	0.0	0.0
LOC100506235	0.0	0.0	0.0	0.0	0.0	0.0	0.1	0.0	0.1
LOC101929745	0.1	0.0	0.0	0.0	0.0	0.1	0.0	0.0	0.1
RAP1GAP	0.1	0.0	0.1	0.0	0.0	0.0	0.0	0.0	0.0
MLXIPL	0.1	0.0	0.1	0.0	0.0	0.0	0.0	0.0	0.0
ADAMTSL3	0.1	0.0	0.0	0.0	0.0	0.0	0.1	0.0	0.1
LOC101928422	0.1	0.0	0.0	0.0	0.0	0.0	0.1	0.0	0.0
TRAF3IP3	0.1	0.0	0.1	0.0	0.0	0.1	0.0	0.0	0.0
TMEM191C	0.0	0.1	0.0	0.0	0.0	0.0	0.1	0.0	0.0
PSG6	0.1	0.0	0.0	0.0	0.1	0.1	0.0	0.0	0.0
PADI1	0.0	0.0	0.0	0.0	0.0	0.0	0.1	0.0	0.0
LRRC4B	0.1	0.0	0.0	0.0	0.0	0.0	0.1	0.0	0.0
RP1L1	0.0	0.0	0.0	0.0	0.0	0.0	0.0	0.0	0.0
KIAA1755	0.1	0.0	0.0	0.0	0.0	0.0	0.1	0.0	0.0
LOC101060161	0.1	0.0	0.0	0.0	0.0	0.0	0.0	0.0	0.0
CYP26C1	0.1	0.0	0.0	0.0	0.0	0.0	0.0	0.0	0.0
LOC339788	0.1	0.0	0.0	0.0	0.0	0.0	0.1	0.0	0.0
PLB1	0.0	0.0	0.1	0.0	0.0	0.0	0.0	0.0	0.0
ZNF599	0.0	0.0	0.0	0.0	0.0	0.0	0.0	0.0	0.0
ALAS2	0.0	0.0	0.0	0.0	0.0	0.0	0.1	0.0	0.1
DNAH7	0.1	0.0	0.0	0.0	0.0	0.0	0.0	0.0	0.0
CGB5	0.0	0.1	0.0	0.0	0.0	0.0	0.0	0.0	0.0
LOC101054525	0.0	0.0	0.1	0.0	0.1	0.0	0.0	0.0	0.0
MYOM3	0.1	0.0	0.0	0.0	0.0	0.0	0.0	0.0	0.0
EPHB1	0.0	0.0	0.1	0.0	0.0	0.0	0.0	0.0	0.0
LOC101928195	0.0	0.0	0.1	0.0	0.0	0.0	0.0	0.0	0.0
CGB	0.0	0.1	0.0	0.0	0.0	0.0	0.0	0.0	0.0
CTAGE8	0.1	0.0	0.1	0.0	0.0	0.0	0.0	0.0	0.0
FAM157B	0.0	0.0	0.0	0.1	0.0	0.0	0.0	0.1	0.0
COL18A1-AS1	0.0	0.0	0.0	0.0	0.0	0.0	0.1	0.0	0.0
TTLL6	0.0	0.0	0.0	0.0	0.1	0.0	0.0	0.0	0.0
LOC101927391	0.1	0.0	0.0	0.0	0.0	0.0	0.0	0.0	0.0
AGAP11	0.0	0.0	0.1	0.0	0.0	0.0	0.0	0.0	0.1
GPRIN3	0.1	0.0	0.1	0.0	0.0	0.0	0.0	0.0	0.0

ST8SIA6	0.0	0.0	0.0	0.0	0.0	0.0	0.0	0.0	0.0
TRPM2	0.0	0.0	0.0	0.0	0.0	0.1	0.0	0.0	0.0
PIR-FIGF	0.0	0.1	0.1	0.0	0.0	0.0	0.0	0.0	0.0
PTK2B	0.0	0.0	0.0	0.0	0.0	0.0	0.0	0.0	0.0
SLIT1	0.1	0.0	0.0	0.0	0.0	0.0	0.0	0.0	0.0
LOC100128908	0.1	0.0	0.0	0.0	0.0	0.0	0.0	0.0	0.0
COL6A3	0.1	0.0	0.0	0.0	0.0	0.0	0.0	0.0	0.0
PAX5	0.0	0.0	0.0	0.0	0.0	0.0	0.1	0.0	0.0
LINC00558	0.0	0.0	0.0	0.0	0.0	0.0	0.0	0.0	0.0
PKD1L1	0.0	0.0	0.0	0.0	0.0	0.0	0.0	0.0	0.0
ZNF229	0.1	0.0	0.0	0.0	0.0	0.0	0.0	0.0	0.0
NUP210L	0.1	0.0	0.0	0.0	0.0	0.0	0.0	0.0	0.0
DCST1	0.0	0.0	0.0	0.0	0.1	0.0	0.0	0.0	0.0
LOC283387	0.0	0.0	0.0	0.0	0.0	0.0	0.0	0.0	0.0
LOC101929106	0.1	0.0	0.0	0.0	0.0	0.0	0.0	0.0	0.0
LOC101927188	0.0	0.0	0.0	0.0	0.0	0.0	0.0	0.0	0.0
ITGAM	0.0	0.0	0.0	0.0	0.0	0.0	0.0	0.0	0.0
KIAA1549L	0.0	0.0	0.0	0.0	0.0	0.0	0.0	0.0	0.0
ANKRD31	0.0	0.0	0.0	0.0	0.0	0.0	0.0	0.0	0.0
RBP3	0.0	0.0	0.0	0.0	0.0	0.0	0.0	0.0	0.0
XIST	0.0	0.0	0.0	0.0	0.0	0.0	0.0	0.0	0.0
RIMBP3C	0.0	0.0	0.0	0.0	0.0	0.0	0.0	0.0	0.0
TRNAV16	0.6	0.0	0.7	0.6	0.0	0.4	2.5	0.8	2.6
ZHX1-C8ORF76	2.4	0.0	0.0	0.0	0.0	0.0	1.4	0.4	1.2
TRNAD10	0.0	0.4	0.4	0.0	0.5	0.8	2.1	0.4	0.8
SNORD47	1.0	1.0	0.0	0.3	0.0	0.4	0.8	0.3	0.7
LYRM5	0.9	0.0	0.2	0.0	0.0	0.0	0.4	0.1	0.0
RNU6-16P	0.0	0.0	1.1	0.0	0.3	0.0	0.0	0.1	0.0
RNU6-39P	1.3	0.0	0.0	0.0	0.0	0.0	0.0	0.1	0.0
LOC101929414	0.6	0.0	0.1	0.0	0.0	0.2	0.3	0.1	0.0
LOC100506571	0.4	0.0	0.1	0.0	0.1	0.0	0.4	0.0	0.2
LOC101927632	0.2	0.0	0.2	0.1	0.1	0.0	0.2	0.1	0.3
DRD5	0.1	0.0	0.0	0.0	0.0	0.0	0.5	0.1	0.4
LOC101928906	0.1	0.0	0.0	0.0	0.0	0.2	0.4	0.0	0.2
LOC101927547	0.3	0.0	0.2	0.0	0.0	0.0	0.3	0.0	0.1
TNFRSF13C	0.4	0.0	0.0	0.0	0.0	0.1	0.2	0.1	0.0
HBQ1	0.4	0.1	0.0	0.0	0.1	0.0	0.1	0.1	0.1
TAS2R46	0.3	0.0	0.1	0.0	0.0	0.1	0.2	0.0	0.2
ELMOD1	0.2	0.1	0.0	0.0	0.1	0.1	0.3	0.0	0.2
CST6	0.2	0.2	0.0	0.0	0.0	0.0	0.4	0.0	0.0
CD28	0.3	0.0	0.1	0.0	0.1	0.0	0.3	0.0	0.1
LOC101928153	0.0	0.1	0.1	0.1	0.0	0.1	0.1	0.1	0.2
LOC101928096	0.3	0.0	0.0	0.0	0.0	0.1	0.3	0.1	0.0
KCNJ15	0.1	0.1	0.0	0.0	0.0	0.1	0.2	0.1	0.2
PRG2	0.2	0.0	0.0	0.0	0.0	0.0	0.3	0.1	0.1
PSTPIP1	0.3	0.0	0.1	0.0	0.0	0.0	0.2	0.0	0.0
LOC101928466	0.3	0.0	0.0	0.0	0.0	0.1	0.1	0.0	0.1
TOMM20L	0.1	0.0	0.1	0.0	0.0	0.1	0.1	0.1	0.2
LOC101929484	0.1	0.0	0.2	0.1	0.0	0.1	0.0	0.0	0.1
MVB12B	0.0	0.0	0.2	0.1	0.0	0.0	0.2	0.0	0.0
HLA-DMB	0.1	0.0	0.0	0.0	0.1	0.1	0.2	0.0	0.0
CABP1	0.2	0.0	0.0	0.0	0.0	0.0	0.1	0.0	0.1
SLC13A3	0.2	0.0	0.1	0.0	0.0	0.0	0.1	0.0	0.0
CELF2-AS2	0.2	0.0	0.0	0.0	0.0	0.0	0.1	0.0	0.1
LOC101927741	0.2	0.0	0.1	0.0	0.0	0.0	0.1	0.0	0.0

GDF7	0.0	0.1	0.1	0.0	0.0	0.0	0.1	0.0	0.0
GLRA1	0.1	0.0	0.1	0.0	0.0	0.1	0.0	0.0	0.0
ANGPTL7	0.2	0.0	0.0	0.0	0.0	0.0	0.0	0.0	0.0
FLJ40194	0.0	0.0	0.1	0.0	0.0	0.0	0.0	0.0	0.0
TTLL11-IT1	0.1	0.0	0.0	0.0	0.1	0.0	0.1	0.0	0.1
FLJ16171	0.1	0.0	0.0	0.0	0.0	0.0	0.2	0.0	0.0
LOC101927940	0.0	0.0	0.1	0.0	0.0	0.0	0.1	0.0	0.1
DLX6	0.0	0.0	0.0	0.1	0.0	0.0	0.0	0.0	0.1
TRPC3	0.0	0.0	0.0	0.0	0.0	0.0	0.1	0.0	0.1
BEGAIN	0.1	0.0	0.0	0.0	0.0	0.0	0.1	0.0	0.0
CHRDL2	0.1	0.0	0.0	0.0	0.0	0.0	0.0	0.0	0.0
LOC101927606	0.0	0.0	0.0	0.0	0.0	0.0	0.0	0.0	0.0
ENKUR	0.0	0.0	0.0	0.0	0.0	0.0	0.0	0.0	0.1
FLJ37505	0.1	0.0	0.0	0.0	0.0	0.0	0.1	0.0	0.0
SLC26A8	0.0	0.0	0.0	0.0	0.0	0.0	0.0	0.0	0.0
RBPJL	0.1	0.0	0.0	0.0	0.0	0.0	0.0	0.0	0.0
MGC45800	0.1	0.0	0.0	0.0	0.0	0.0	0.0	0.0	0.0
IGDCC3	0.0	0.0	0.0	0.0	0.0	0.0	0.0	0.0	0.0
C11orf16	0.0	0.0	0.0	0.0	0.0	0.0	0.0	0.0	0.0
LOC100996713	0.0	0.0	0.0	0.0	0.0	0.0	0.0	0.0	0.0
LOC340107	0.0	0.0	0.0	0.0	0.0	0.0	0.0	0.0	0.0
LOC100506007	0.5	0.0	0.3	0.0	0.1	0.2	0.5	0.0	0.2
LOC100996664	0.6	0.0	0.3	0.1	0.1	0.2	0.4	0.0	0.1
MAGI1-AS1	0.4	0.0	0.1	0.0	0.1	0.2	0.5	0.0	0.1
LOC101929737	0.8	0.0	0.2	0.0	0.0	0.0	0.3	0.0	0.0
TSPYL6	0.6	0.0	0.2	0.0	0.0	0.1	0.1	0.0	0.1
USP46-AS1	0.4	0.0	0.1	0.0	0.1	0.0	0.2	0.0	0.1
MIA	0.0	0.1	0.0	0.0	0.2	0.3	0.3	0.0	0.0
CLYBL	0.3	0.0	0.1	0.0	0.0	0.0	0.1	0.0	0.1
GOLGA6L10	0.4	0.0	0.0	0.0	0.0	0.0	0.0	0.0	0.1
LOC101927085	0.0	0.0	0.1	0.1	0.1	0.1	0.0	0.0	0.1
LOC101927767	0.2	0.0	0.1	0.0	0.0	0.0	0.2	0.1	0.0
GABRQ	0.1	0.0	0.1	0.0	0.1	0.0	0.2	0.0	0.0
LOC101927601	0.3	0.0	0.0	0.0	0.0	0.0	0.0	0.0	0.1
LOC101927417	0.1	0.0	0.0	0.0	0.0	0.0	0.1	0.0	0.1
LOC101928389	0.2	0.0	0.0	0.0	0.0	0.0	0.1	0.0	0.0
C19orf38	0.0	0.0	0.2	0.0	0.1	0.0	0.0	0.0	0.1
CPO	0.1	0.0	0.0	0.0	0.1	0.0	0.1	0.0	0.1
SLC7A9	0.1	0.0	0.0	0.0	0.0	0.0	0.1	0.0	0.0
SYNDIG1	0.1	0.0	0.1	0.0	0.0	0.1	0.0	0.0	0.0
DENND2D	0.1	0.0	0.0	0.0	0.1	0.0	0.0	0.0	0.1
CALHM3	0.0	0.1	0.0	0.0	0.1	0.0	0.1	0.0	0.0
SIRPA	0.0	0.0	0.0	0.0	0.0	0.0	0.0	0.0	0.1
DPPA4	0.1	0.0	0.0	0.0	0.0	0.0	0.1	0.0	0.1
SLC7A10	0.1	0.0	0.1	0.0	0.0	0.0	0.0	0.0	0.0
LOC100129203	0.1	0.0	0.0	0.0	0.0	0.0	0.0	0.0	0.0
MYLK3	0.1	0.0	0.0	0.0	0.1	0.0	0.0	0.0	0.0
MSH5	0.0	0.1	0.1	0.0	0.0	0.1	0.0	0.0	0.0
OR4F3	0.1	0.0	0.0	0.0	0.0	0.0	0.0	0.0	0.0
OR4F16	0.1	0.0	0.0	0.0	0.0	0.0	0.0	0.0	0.0
OR4F29	0.1	0.0	0.0	0.0	0.0	0.0	0.0	0.0	0.0
LOC339298	0.1	0.0	0.0	0.0	0.0	0.0	0.0	0.0	0.0
LOC646670	0.0	0.0	0.1	0.0	0.0	0.0	0.0	0.0	0.0
ITGA11	0.0	0.0	0.0	0.0	0.0	0.1	0.0	0.0	0.0
PWAR5	0.0	0.0	0.0	0.0	0.0	0.0	0.0	0.0	0.0

LOC100507391	0.0	0.0	0.0	0.0	0.0	0.0	0.0	0.0	0.0
CGB8	0.0	0.0	0.0	0.0	0.0	0.0	0.0	0.0	0.0
FBN2	0.0	0.0	0.0	0.0	0.0	0.0	0.0	0.0	0.0
FMN2	0.0	0.0	0.0	0.0	0.0	0.0	0.0	0.0	0.0
LOC101928980	0.0	0.0	0.0	0.0	0.0	0.0	0.0	0.0	0.0
FAM74A3	5.6	1.0	2.0	0.0	0.8	0.9	5.2	0.0	1.2
SNORD12B	5.1	1.0	1.5	0.2	0.7	0.0	4.3	0.0	3.0
RNY5	5.9	1.9	0.9	0.0	0.4	0.7	1.4	0.0	0.3
KLRC4	4.3	0.9	1.8	0.0	0.2	0.3	0.1	0.0	0.3
C15orf48	2.2	1.2	1.9	0.0	0.2	0.3	0.1	0.1	0.0
LOC101927807	1.2	0.5	0.5	0.2	0.6	0.0	1.4	0.0	0.9
SNORD42B	0.6	0.4	0.8	0.3	0.5	0.0	1.3	0.0	1.3
SPAG5-AS1	1.7	0.3	0.9	0.0	0.0	0.1	1.5	0.0	0.5
SMIM8	0.7	0.3	0.9	0.0	0.3	0.5	1.2	0.0	0.6
LOC101929262	1.6	0.4	0.3	0.0	0.0	0.0	0.8	0.0	0.6
FAM167B	1.6	0.8	0.5	0.0	0.0	0.1	0.2	0.0	0.2
PLEKHB1	0.8	0.7	0.8	0.0	0.1	0.3	0.5	0.0	0.3
SNORD36B	0.6	0.4	0.4	0.3	0.0	0.4	0.4	0.0	0.8
TSPAN8	1.0	0.3	0.4	0.0	0.2	0.1	0.7	0.0	0.4
GAS5-AS1	0.6	0.3	0.4	0.0	0.1	0.2	0.5	0.0	0.6
LOC728026	0.9	0.1	0.7	0.0	0.1	0.1	0.6	0.0	0.2
LOC101929709	0.9	0.2	0.4	0.0	0.2	0.1	0.4	0.0	0.6
LOC101927809	0.9	0.2	0.6	0.0	0.1	0.1	0.3	0.0	0.4
PLAC8L1	0.9	0.1	0.7	0.0	0.1	0.2	0.5	0.0	0.0
LOC101927218	0.6	0.3	0.5	0.0	0.0	0.1	0.3	0.0	0.3
TATDN3	1.0	0.3	0.3	0.0	0.1	0.1	0.1	0.0	0.3
LOC101928450	0.6	0.4	0.4	0.0	0.2	0.0	0.4	0.0	0.3
FRMD6-AS2	0.4	0.2	0.2	0.0	0.1	0.2	0.7	0.0	0.4
C22orf26	0.8	0.3	0.3	0.0	0.0	0.1	0.6	0.0	0.1
C5orf63	0.9	0.2	0.1	0.0	0.2	0.1	0.3	0.0	0.2
DNM1P50	1.1	0.2	0.2	0.0	0.0	0.1	0.5	0.0	0.0
LOC100130548	0.7	0.2	0.4	0.0	0.0	0.1	0.5	0.0	0.3
PIK3R3	0.7	0.3	0.4	0.0	0.1	0.1	0.1	0.0	0.3
LOC101927765	0.7	0.2	0.3	0.0	0.0	0.1	0.4	0.0	0.2
SAMD13	0.4	0.2	0.4	0.0	0.3	0.1	0.3	0.0	0.3
LOC100996273	0.6	0.2	0.2	0.1	0.0	0.1	0.5	0.0	0.2
LOC101929067	0.7	0.3	0.6	0.0	0.0	0.0	0.2	0.1	0.0
LOC101927983	0.7	0.1	0.3	0.0	0.0	0.0	0.6	0.0	0.1
RAB6B	0.8	0.1	0.3	0.0	0.2	0.1	0.0	0.0	0.4
LURAP1	0.7	0.1	0.2	0.0	0.1	0.3	0.4	0.0	0.1
DPPA3	0.4	0.2	0.4	0.0	0.1	0.1	0.1	0.0	0.3
LOC101927354	0.6	0.1	0.4	0.0	0.0	0.1	0.3	0.0	0.2
RNF122	0.4	0.3	0.3	0.0	0.2	0.0	0.3	0.0	0.3
ZNF626	0.3	0.2	0.6	0.0	0.0	0.2	0.1	0.0	0.3
LOC100996274	0.6	0.1	0.4	0.0	0.0	0.0	0.5	0.0	0.1
PLIN1	0.7	0.3	0.5	0.0	0.0	0.1	0.0	0.0	0.0
LOC284009	0.5	0.3	0.4	0.0	0.2	0.0	0.2	0.0	0.2
RBAKDN	0.4	0.2	0.2	0.1	0.0	0.2	0.4	0.0	0.1
APOA1	0.6	0.1	0.2	0.1	0.0	0.1	0.4	0.0	0.1
ROPN1L	0.5	0.2	0.3	0.0	0.0	0.2	0.2	0.0	0.1
LOC101928835	0.6	0.3	0.1	0.0	0.0	0.0	0.2	0.0	0.2
TDGF1	0.5	0.1	0.1	0.0	0.2	0.1	0.4	0.0	0.1
OSTCP2	0.6	0.0	0.2	0.0	0.0	0.1	0.3	0.0	0.1
PTHLH	0.3	0.2	0.2	0.1	0.0	0.1	0.3	0.0	0.2
LOC101926940	0.4	0.1	0.3	0.0	0.1	0.1	0.2	0.0	0.2

C3orf35	0.3	0.0	0.4	0.0	0.1	0.1	0.4	0.0	0.1
TNFRSF9	0.6	0.4	0.2	0.0	0.0	0.0	0.1	0.0	0.1
LOC101929597	0.3	0.3	0.1	0.0	0.1	0.2	0.3	0.0	0.1
LOC100506088	0.4	0.1	0.2	0.0	0.1	0.1	0.3	0.0	0.2
LOC101926925	0.1	0.2	0.7	0.0	0.1	0.0	0.2	0.0	0.1
C4orf33	0.5	0.1	0.2	0.0	0.1	0.2	0.2	0.0	0.1
RSPH6A	0.7	0.1	0.2	0.0	0.0	0.1	0.1	0.0	0.1
SYP	0.4	0.2	0.1	0.0	0.1	0.0	0.3	0.0	0.1
OMG	0.5	0.0	0.2	0.0	0.1	0.0	0.3	0.0	0.1
TTTY14	0.5	0.0	0.3	0.0	0.1	0.0	0.2	0.0	0.2
FLJ25363	0.3	0.0	0.0	0.0	0.1	0.1	0.6	0.0	0.1
LOC101928351	0.4	0.1	0.2	0.0	0.1	0.1	0.3	0.0	0.1
DKKL1	0.4	0.1	0.2	0.0	0.1	0.0	0.2	0.0	0.2
GPR18	0.4	0.1	0.1	0.0	0.0	0.1	0.4	0.0	0.1
LINC00922	0.5	0.1	0.3	0.0	0.0	0.1	0.1	0.0	0.0
LOC100506737	0.5	0.1	0.1	0.0	0.1	0.1	0.1	0.0	0.1
LOC101929687	0.5	0.0	0.2	0.0	0.1	0.1	0.2	0.0	0.2
LOC101928148	0.2	0.1	0.4	0.1	0.0	0.0	0.3	0.1	0.0
NLRP10	0.3	0.1	0.1	0.1	0.0	0.1	0.3	0.0	0.2
LOC100996535	0.6	0.2	0.2	0.0	0.1	0.0	0.1	0.0	0.0
KLLN	0.2	0.1	0.3	0.0	0.1	0.1	0.3	0.0	0.1
LOC100996693	0.6	0.1	0.2	0.0	0.2	0.0	0.1	0.0	0.1
SPDYA	0.8	0.0	0.2	0.0	0.0	0.0	0.0	0.0	0.1
LOC101928482	0.4	0.0	0.2	0.0	0.1	0.1	0.1	0.0	0.2
MCOLN3	0.4	0.1	0.2	0.0	0.0	0.1	0.2	0.0	0.1
EGFL8	0.2	0.2	0.3	0.0	0.0	0.1	0.1	0.0	0.2
WFDC3	0.3	0.2	0.4	0.0	0.0	0.0	0.1	0.0	0.0
TNFRSF19	0.3	0.2	0.2	0.0	0.0	0.1	0.2	0.0	0.1
PPP1R3G	0.4	0.1	0.1	0.0	0.1	0.0	0.2	0.0	0.1
CLDN24	0.4	0.1	0.2	0.0	0.1	0.0	0.3	0.0	0.0
LSMEM1	0.3	0.2	0.1	0.1	0.0	0.0	0.2	0.0	0.2
UBE2QL1	0.1	0.2	0.3	0.2	0.0	0.0	0.2	0.1	0.0
FAM183A	0.2	0.1	0.3	0.1	0.0	0.1	0.1	0.0	0.2
ZNF570	0.4	0.1	0.2	0.0	0.0	0.0	0.1	0.0	0.1
FAM227A	0.4	0.2	0.2	0.0	0.0	0.1	0.1	0.0	0.1
BNIPL	0.4	0.1	0.2	0.0	0.0	0.0	0.2	0.0	0.1
LOC101927044	0.1	0.2	0.2	0.0	0.1	0.0	0.2	0.0	0.3
FGFBP1	0.4	0.2	0.1	0.0	0.0	0.0	0.1	0.0	0.2
FLJ46026	0.4	0.2	0.1	0.0	0.1	0.1	0.1	0.0	0.1
LOC101927385	0.3	0.0	0.2	0.0	0.1	0.0	0.4	0.0	0.1
HOXC-AS1	0.4	0.1	0.1	0.0	0.0	0.1	0.2	0.0	0.1
TAS2R14	0.4	0.1	0.2	0.0	0.0	0.1	0.2	0.0	0.1
MT1G	0.0	0.1	0.1	0.0	0.0	0.0	0.4	0.0	0.3
BANCR	0.4	0.0	0.2	0.0	0.1	0.1	0.2	0.0	0.1
LOC100133985	0.3	0.1	0.1	0.0	0.0	0.1	0.2	0.0	0.2
LOC101929381	0.3	0.1	0.3	0.0	0.0	0.0	0.2	0.0	0.1
HCG8	0.3	0.1	0.2	0.0	0.1	0.0	0.2	0.0	0.1
CYP3A5	0.4	0.1	0.2	0.0	0.0	0.1	0.2	0.0	0.1
ZNF682	0.3	0.1	0.2	0.0	0.0	0.0	0.2	0.0	0.1
TRHR	0.3	0.0	0.1	0.0	0.1	0.0	0.3	0.0	0.1
TEX19	0.3	0.2	0.2	0.0	0.0	0.0	0.2	0.0	0.1
LOC100129924	0.4	0.1	0.3	0.0	0.0	0.0	0.2	0.0	0.0
LOC101927028	0.2	0.1	0.2	0.0	0.1	0.0	0.2	0.0	0.2
TMEM240	0.2	0.1	0.2	0.0	0.0	0.1	0.2	0.0	0.2
FAM209A	0.1	0.1	0.1	0.0	0.1	0.1	0.5	0.0	0.0

LOC101928569	0.3	0.1	0.2	0.0	0.1	0.2	0.0	0.0	0.0
LOC101927099	0.4	0.1	0.1	0.1	0.0	0.0	0.1	0.0	0.2
LOC101060181	0.5	0.0	0.1	0.0	0.0	0.0	0.2	0.0	0.1
SLAMF9	0.5	0.2	0.1	0.0	0.0	0.0	0.1	0.0	0.0
CXCR6	0.5	0.1	0.1	0.0	0.1	0.1	0.2	0.0	0.0
LOC101928965	0.5	0.1	0.1	0.0	0.0	0.0	0.1	0.0	0.1
LOC100240734	0.3	0.0	0.2	0.0	0.1	0.0	0.2	0.0	0.1
HOXC4	0.4	0.2	0.1	0.0	0.0	0.0	0.0	0.0	0.1
REP15	0.4	0.1	0.2	0.0	0.0	0.0	0.1	0.0	0.2
GRB7	0.4	0.1	0.3	0.0	0.0	0.0	0.0	0.0	0.1
RBMS3-AS3	0.2	0.1	0.2	0.0	0.1	0.1	0.2	0.0	0.1
ADAM20	0.4	0.1	0.1	0.0	0.0	0.0	0.3	0.0	0.2
TMEM61	0.3	0.1	0.2	0.0	0.1	0.0	0.1	0.0	0.1
AKR7A3	0.4	0.0	0.2	0.0	0.1	0.0	0.2	0.0	0.0
LOC100507564	0.2	0.2	0.2	0.0	0.0	0.0	0.1	0.0	0.2
NKPD1	0.3	0.1	0.2	0.1	0.0	0.1	0.1	0.0	0.1
LOC101927708	0.3	0.1	0.0	0.0	0.0	0.0	0.3	0.0	0.2
LOC101928180	0.2	0.0	0.3	0.0	0.3	0.0	0.0	0.0	0.2
CORO6	0.3	0.1	0.2	0.0	0.0	0.1	0.1	0.0	0.1
SPATA9	0.3	0.1	0.1	0.0	0.1	0.1	0.1	0.0	0.2
DHDH	0.2	0.1	0.3	0.0	0.0	0.1	0.1	0.0	0.0
PALM	0.4	0.1	0.1	0.0	0.1	0.0	0.1	0.0	0.1
IL7	0.2	0.1	0.1	0.0	0.0	0.1	0.2	0.0	0.1
SHBG	0.3	0.1	0.1	0.0	0.1	0.0	0.1	0.0	0.3
LOC101927957	0.1	0.3	0.1	0.1	0.0	0.1	0.1	0.0	0.2
LOC101929487	0.2	0.1	0.2	0.0	0.1	0.1	0.1	0.0	0.0
PRR25	0.3	0.1	0.2	0.1	0.0	0.1	0.2	0.0	0.1
MAT1A	0.2	0.2	0.2	0.0	0.0	0.1	0.1	0.0	0.0
DPP6	0.0	0.1	0.2	0.2	0.1	0.0	0.1	0.2	0.0
LOC101927839	0.3	0.0	0.2	0.1	0.0	0.0	0.1	0.0	0.1
LOC101929504	0.3	0.1	0.0	0.0	0.1	0.1	0.2	0.0	0.1
VSX1	0.4	0.1	0.1	0.0	0.0	0.0	0.1	0.0	0.1
GRASP	0.3	0.1	0.1	0.1	0.1	0.0	0.2	0.0	0.1
METTL20	0.3	0.1	0.2	0.1	0.0	0.1	0.1	0.0	0.0
TFAP2A-AS1	0.2	0.1	0.2	0.0	0.0	0.0	0.2	0.0	0.1
LOC101928676	0.2	0.1	0.2	0.0	0.0	0.0	0.1	0.0	0.2
IGSF6	0.2	0.1	0.1	0.0	0.1	0.1	0.2	0.0	0.0
LOC101927376	0.3	0.1	0.1	0.1	0.0	0.0	0.0	0.1	0.1
LOC101929411	0.2	0.0	0.1	0.0	0.1	0.1	0.2	0.0	0.1
CIDEC	0.3	0.1	0.1	0.0	0.0	0.0	0.2	0.0	0.1
NHLH1	0.1	0.1	0.3	0.0	0.1	0.1	0.1	0.0	0.0
LOC101928319	0.1	0.2	0.2	0.0	0.1	0.0	0.2	0.0	0.1
RASSF8-AS1	0.2	0.0	0.0	0.0	0.2	0.1	0.1	0.0	0.2
LOC101927759	0.3	0.1	0.1	0.0	0.0	0.1	0.1	0.0	0.0
C19orf80	0.2	0.1	0.1	0.0	0.0	0.0	0.2	0.0	0.2
NODAL	0.2	0.1	0.2	0.0	0.0	0.0	0.2	0.0	0.2
LOC100129029	0.3	0.1	0.1	0.0	0.0	0.1	0.3	0.0	0.1
LOC101929548	0.2	0.0	0.1	0.0	0.1	0.0	0.2	0.0	0.2
LTA	0.2	0.1	0.1	0.0	0.1	0.1	0.0	0.0	0.1
GRIK1-AS2	0.2	0.1	0.1	0.0	0.0	0.0	0.2	0.0	0.1
HNRNPCL1	0.3	0.0	0.1	0.0	0.0	0.0	0.2	0.0	0.1
LOC100506516	0.2	0.0	0.1	0.0	0.0	0.0	0.3	0.0	0.1
GSTA4	0.2	0.0	0.1	0.0	0.1	0.1	0.1	0.0	0.1
LOC101927057	0.5	0.0	0.2	0.0	0.0	0.0	0.0	0.0	0.1
TRIM7	0.5	0.0	0.0	0.0	0.0	0.1	0.1	0.0	0.0

LOC101928798	0.3	0.1	0.1	0.0	0.0	0.0	0.1	0.0	0.1
LINGO3	0.3	0.0	0.1	0.0	0.0	0.0	0.2	0.0	0.1
LOC101927245	0.2	0.2	0.1	0.0	0.0	0.0	0.2	0.0	0.0
GPHA2	0.2	0.1	0.1	0.0	0.1	0.1	0.0	0.0	0.1
LOC101929249	0.5	0.1	0.1	0.0	0.0	0.0	0.1	0.0	0.0
DLG3-AS1	0.4	0.0	0.1	0.0	0.0	0.0	0.1	0.0	0.0
DNAJC28	0.2	0.0	0.1	0.0	0.1	0.0	0.1	0.0	0.1
LOC101928081	0.3	0.0	0.1	0.0	0.0	0.0	0.1	0.0	0.1
LOC100653133	0.2	0.1	0.1	0.0	0.0	0.0	0.1	0.0	0.2
NUP62CL	0.1	0.1	0.3	0.0	0.0	0.1	0.1	0.0	0.0
LOC101929143	0.2	0.1	0.1	0.0	0.0	0.1	0.2	0.0	0.1
OR8G5	0.2	0.0	0.1	0.0	0.0	0.1	0.2	0.0	0.1
LOC101929283	0.4	0.0	0.1	0.0	0.0	0.0	0.0	0.0	0.2
PLP1	0.1	0.0	0.1	0.1	0.1	0.0	0.3	0.0	0.0
HCST	0.1	0.0	0.0	0.0	0.1	0.1	0.1	0.0	0.1
LRRC34	0.3	0.1	0.2	0.0	0.0	0.0	0.1	0.0	0.0
SSBP2	0.3	0.1	0.1	0.0	0.0	0.1	0.0	0.0	0.1
CTRB1	0.4	0.0	0.2	0.0	0.0	0.0	0.1	0.0	0.0
DEGS2	0.2	0.2	0.1	0.0	0.0	0.1	0.1	0.0	0.1
IGFALS	0.2	0.1	0.1	0.0	0.0	0.0	0.1	0.0	0.1
OR8B12	0.2	0.0	0.1	0.0	0.0	0.0	0.2	0.0	0.0
LOC100129781	0.2	0.1	0.1	0.0	0.0	0.0	0.1	0.0	0.1
LOC101927288	0.2	0.1	0.1	0.0	0.1	0.0	0.0	0.0	0.0
ZNF474	0.2	0.1	0.1	0.0	0.0	0.1	0.1	0.0	0.1
LOC101928900	0.2	0.1	0.1	0.0	0.1	0.0	0.1	0.0	0.0
MGAT4A	0.2	0.1	0.1	0.0	0.0	0.0	0.1	0.0	0.1
PIPOX	0.1	0.1	0.1	0.0	0.0	0.0	0.2	0.0	0.1
TPSB2	0.3	0.2	0.1	0.0	0.0	0.0	0.0	0.0	0.0
SOX21	0.0	0.0	0.1	0.1	0.1	0.0	0.0	0.1	0.1
FTO-IT1	0.2	0.1	0.0	0.0	0.1	0.0	0.1	0.0	0.1
TMEM221	0.1	0.1	0.2	0.0	0.1	0.1	0.1	0.0	0.1
LOC101929313	0.3	0.0	0.1	0.0	0.1	0.1	0.1	0.0	0.0
TNFAIP8L2	0.3	0.0	0.1	0.1	0.0	0.1	0.1	0.0	0.1
LOC101927585	0.3	0.0	0.1	0.0	0.0	0.0	0.1	0.0	0.1
CTSV	0.3	0.0	0.1	0.0	0.1	0.0	0.1	0.0	0.0
HCAR2	0.1	0.1	0.1	0.0	0.0	0.1	0.2	0.0	0.1
LOC100505679	0.3	0.1	0.1	0.0	0.0	0.0	0.2	0.0	0.1
LOC101929347	0.3	0.0	0.1	0.0	0.1	0.0	0.1	0.0	0.1
NTF3	0.2	0.1	0.2	0.0	0.0	0.0	0.1	0.0	0.1
RNF128	0.3	0.0	0.2	0.0	0.0	0.0	0.0	0.0	0.0
C11orf87	0.3	0.1	0.1	0.1	0.0	0.0	0.1	0.0	0.0
FMO4	0.1	0.2	0.1	0.0	0.0	0.0	0.1	0.0	0.1
LOC100506538	0.1	0.0	0.1	0.0	0.0	0.0	0.2	0.0	0.2
HOXC13	0.3	0.0	0.1	0.0	0.0	0.1	0.1	0.0	0.0
LOC101928461	0.5	0.0	0.1	0.0	0.0	0.0	0.0	0.0	0.0
TMEM56	0.3	0.1	0.1	0.0	0.0	0.0	0.0	0.0	0.1
LOC101928794	0.2	0.1	0.1	0.0	0.0	0.0	0.1	0.0	0.2
OR8D2	0.1	0.0	0.1	0.0	0.0	0.0	0.2	0.0	0.1
LOC101927515	0.2	0.0	0.1	0.1	0.1	0.0	0.1	0.0	0.0
LOC101927772	0.2	0.0	0.1	0.0	0.0	0.0	0.2	0.0	0.2
APBA1	0.2	0.1	0.1	0.0	0.1	0.0	0.1	0.0	0.1
GCM2	0.3	0.0	0.1	0.0	0.0	0.0	0.2	0.0	0.0
GRIA4	0.3	0.0	0.1	0.0	0.0	0.0	0.1	0.0	0.0
CHST6	0.2	0.0	0.1	0.0	0.1	0.1	0.1	0.0	0.1
LINC00417	0.4	0.1	0.1	0.0	0.0	0.0	0.0	0.0	0.0

SLC10A6	0.1	0.1	0.1	0.0	0.1	0.1	0.0	0.0	0.2
RNF148	0.1	0.0	0.0	0.0	0.0	0.1	0.2	0.0	0.1
BARHL1	0.1	0.0	0.1	0.0	0.1	0.1	0.1	0.0	0.1
IGHA1	0.2	0.1	0.0	0.0	0.0	0.0	0.1	0.0	0.1
ITGB1BP2	0.1	0.0	0.1	0.0	0.0	0.0	0.1	0.0	0.1
GFI1	0.3	0.0	0.1	0.0	0.0	0.0	0.0	0.0	0.1
SPAG8	0.1	0.1	0.1	0.0	0.1	0.0	0.1	0.0	0.1
LOC101928877	0.3	0.0	0.1	0.0	0.0	0.0	0.1	0.0	0.1
SKAP1	0.1	0.0	0.1	0.0	0.0	0.0	0.2	0.0	0.1
PCAT6	0.2	0.0	0.1	0.0	0.1	0.0	0.1	0.0	0.0
LOC100130207	0.2	0.0	0.0	0.0	0.0	0.0	0.2	0.0	0.0
LOC100192426	0.2	0.0	0.1	0.0	0.0	0.1	0.1	0.0	0.0
C11orf42	0.2	0.1	0.1	0.0	0.0	0.1	0.0	0.0	0.1
LOC101927762	0.1	0.0	0.2	0.0	0.0	0.1	0.1	0.0	0.0
LOC101928763	0.1	0.1	0.1	0.0	0.0	0.0	0.2	0.0	0.0
GPRC5C	0.1	0.1	0.1	0.0	0.0	0.1	0.1	0.0	0.0
ZNF571-AS1	0.2	0.0	0.2	0.0	0.0	0.0	0.1	0.0	0.0
ZMYND12	0.3	0.1	0.1	0.0	0.0	0.0	0.0	0.0	0.0
COLEC11	0.1	0.0	0.1	0.0	0.1	0.0	0.1	0.0	0.0
CIDEA	0.2	0.0	0.1	0.0	0.0	0.1	0.1	0.0	0.0
NAGPA-AS1	0.2	0.1	0.1	0.0	0.0	0.0	0.1	0.0	0.1
IL2RG	0.2	0.0	0.1	0.1	0.0	0.1	0.1	0.0	0.1
MZB1	0.2	0.0	0.2	0.0	0.0	0.1	0.1	0.0	0.1
LOC101927051	0.2	0.1	0.1	0.0	0.0	0.0	0.1	0.0	0.0
AMN	0.2	0.1	0.0	0.0	0.0	0.0	0.2	0.0	0.1
CCDC89	0.2	0.0	0.2	0.0	0.0	0.0	0.0	0.0	0.1
LOC100507548	0.2	0.0	0.1	0.0	0.0	0.0	0.1	0.0	0.0
RDH5	0.3	0.1	0.0	0.1	0.0	0.0	0.0	0.1	0.0
ARL13A	0.2	0.0	0.1	0.0	0.0	0.0	0.1	0.0	0.0
SPEF2	0.2	0.0	0.1	0.0	0.0	0.0	0.1	0.0	0.1
LOC101928105	0.2	0.0	0.0	0.0	0.0	0.0	0.1	0.0	0.1
GIPR	0.2	0.1	0.1	0.0	0.1	0.0	0.1	0.0	0.0
LOC101928044	0.2	0.1	0.0	0.0	0.0	0.0	0.1	0.0	0.0
CCDC64B	0.2	0.1	0.1	0.0	0.0	0.0	0.1	0.0	0.0
LOC101929354	0.1	0.0	0.0	0.0	0.0	0.0	0.2	0.0	0.1
PLEKHH2	0.2	0.1	0.1	0.0	0.0	0.0	0.0	0.0	0.1
RAD9B	0.2	0.0	0.1	0.0	0.1	0.0	0.1	0.0	0.0
LOC100128568	0.2	0.0	0.1	0.0	0.0	0.1	0.1	0.0	0.0
LOC101926984	0.1	0.1	0.1	0.0	0.0	0.0	0.1	0.0	0.0
DMGDH	0.2	0.1	0.1	0.0	0.0	0.1	0.0	0.0	0.0
EOMES	0.1	0.1	0.1	0.0	0.0	0.0	0.1	0.0	0.1
CT45A5	0.2	0.0	0.1	0.0	0.1	0.0	0.1	0.0	0.0
GLRA4	0.2	0.0	0.1	0.0	0.0	0.0	0.1	0.0	0.1
LOC100652782	0.3	0.1	0.0	0.0	0.0	0.1	0.0	0.0	0.0
TCTE3	0.0	0.0	0.1	0.1	0.0	0.0	0.2	0.0	0.0
DNAJB13	0.1	0.1	0.1	0.0	0.1	0.0	0.0	0.0	0.0
LOC101926991	0.1	0.1	0.1	0.0	0.1	0.0	0.1	0.0	0.1
C3orf67	0.2	0.1	0.1	0.0	0.1	0.0	0.0	0.0	0.0
DCST2	0.2	0.1	0.1	0.0	0.0	0.0	0.1	0.0	0.0
LINC00634	0.2	0.1	0.1	0.0	0.0	0.0	0.0	0.0	0.0
VWA7	0.2	0.1	0.1	0.0	0.0	0.0	0.1	0.0	0.0
LOC400794	0.3	0.0	0.1	0.0	0.0	0.0	0.0	0.0	0.0
LOC101929526	0.1	0.1	0.0	0.0	0.0	0.0	0.1	0.0	0.1
ZNF442	0.1	0.1	0.1	0.0	0.0	0.0	0.0	0.0	0.2
LOC81691	0.3	0.1	0.0	0.0	0.0	0.0	0.1	0.0	0.0

CPNE9	0.1	0.1	0.1	0.0	0.0	0.0	0.1	0.0	0.1
ZNF726	0.1	0.1	0.1	0.0	0.0	0.0	0.1	0.0	0.1
ACOT6	0.1	0.1	0.0	0.0	0.0	0.0	0.1	0.0	0.1
SLC16A12	0.1	0.1	0.1	0.0	0.1	0.0	0.2	0.0	0.0
KCNQ5-AS1	0.2	0.1	0.0	0.0	0.0	0.0	0.0	0.0	0.1
LOC101927953	0.1	0.1	0.1	0.0	0.0	0.1	0.1	0.0	0.0
STOX1	0.2	0.1	0.0	0.0	0.0	0.0	0.0	0.0	0.0
MIA2	0.2	0.0	0.1	0.0	0.0	0.0	0.0	0.0	0.0
SLC9C1	0.2	0.0	0.0	0.0	0.0	0.0	0.1	0.0	0.1
LOC101928042	0.1	0.0	0.2	0.0	0.0	0.0	0.0	0.0	0.0
GCNT7	0.2	0.1	0.1	0.0	0.0	0.0	0.1	0.0	0.0
UBAC2-AS1	0.1	0.1	0.1	0.0	0.0	0.0	0.1	0.0	0.1
SLC6A14	0.2	0.0	0.0	0.0	0.0	0.0	0.1	0.0	0.1
RPGRIP1	0.1	0.1	0.1	0.0	0.0	0.0	0.0	0.0	0.1
GAS7	0.2	0.0	0.0	0.0	0.0	0.0	0.0	0.0	0.0
LOC101929643	0.2	0.0	0.1	0.0	0.0	0.0	0.1	0.0	0.1
DAPK2	0.2	0.1	0.1	0.0	0.0	0.0	0.0	0.0	0.0
C12orf60	0.1	0.1	0.1	0.0	0.0	0.0	0.1	0.0	0.0
LOC100507472	0.1	0.1	0.1	0.0	0.1	0.0	0.1	0.0	0.0
F10	0.2	0.0	0.0	0.0	0.0	0.0	0.1	0.0	0.0
FLRT3	0.1	0.1	0.0	0.0	0.1	0.0	0.1	0.0	0.0
ANXA10	0.0	0.0	0.1	0.1	0.0	0.1	0.0	0.0	0.0
LOC101929302	0.2	0.0	0.0	0.0	0.0	0.0	0.1	0.0	0.1
SHC4	0.1	0.0	0.1	0.0	0.0	0.0	0.1	0.0	0.1
LOC101927239	0.1	0.0	0.1	0.0	0.0	0.0	0.1	0.0	0.1
LOC100131107	0.2	0.0	0.1	0.0	0.0	0.0	0.0	0.0	0.0
GRIK5	0.1	0.1	0.1	0.0	0.0	0.0	0.1	0.0	0.0
CCDC42B	0.1	0.1	0.1	0.0	0.0	0.0	0.0	0.0	0.1
LOC100129973	0.1	0.0	0.1	0.0	0.0	0.0	0.2	0.0	0.0
KIAA1257	0.1	0.0	0.0	0.1	0.0	0.0	0.2	0.0	0.0
MAP7	0.2	0.1	0.1	0.0	0.0	0.0	0.0	0.0	0.0
C15orf60	0.1	0.1	0.1	0.0	0.0	0.0	0.0	0.0	0.0
PLA2R1	0.2	0.0	0.1	0.0	0.0	0.0	0.0	0.0	0.0
VCAN	0.1	0.1	0.0	0.0	0.0	0.0	0.0	0.0	0.1
LOC101928917	0.2	0.0	0.1	0.0	0.0	0.0	0.1	0.0	0.0
IL1R2	0.1	0.0	0.1	0.0	0.0	0.0	0.0	0.0	0.0
LOC100996555	0.1	0.0	0.1	0.0	0.0	0.0	0.1	0.0	0.1
LMOD1	0.1	0.0	0.1	0.0	0.0	0.0	0.1	0.0	0.0
LOC101927604	0.1	0.0	0.0	0.0	0.0	0.1	0.0	0.0	0.1
MEGF10	0.1	0.0	0.0	0.0	0.0	0.0	0.1	0.0	0.0
LOC101926931	0.0	0.0	0.2	0.0	0.0	0.0	0.1	0.0	0.0
LOC101928185	0.1	0.0	0.1	0.0	0.0	0.0	0.1	0.0	0.0
C11orf65	0.0	0.1	0.1	0.0	0.0	0.0	0.1	0.0	0.1
ALDH3B2	0.2	0.1	0.0	0.0	0.0	0.0	0.0	0.0	0.0
SIGLEC9	0.1	0.1	0.0	0.0	0.0	0.0	0.1	0.0	0.0
LOC101927948	0.1	0.0	0.1	0.0	0.0	0.0	0.0	0.0	0.1
LOC101927263	0.1	0.0	0.0	0.0	0.0	0.0	0.1	0.0	0.0
SSUH2	0.1	0.1	0.0	0.0	0.0	0.0	0.0	0.0	0.0
LOC339666	0.1	0.0	0.1	0.0	0.0	0.0	0.0	0.0	0.1
XKR3	0.1	0.1	0.1	0.0	0.0	0.0	0.0	0.0	0.0
EFNB3	0.1	0.1	0.0	0.0	0.0	0.0	0.1	0.0	0.0
LOC101929336	0.1	0.0	0.1	0.0	0.0	0.0	0.1	0.0	0.1
OR13D1	0.1	0.1	0.1	0.0	0.0	0.0	0.0	0.0	0.0
FAM87B	0.1	0.1	0.0	0.0	0.0	0.0	0.0	0.0	0.0
DENND2C	0.2	0.0	0.0	0.0	0.0	0.0	0.1	0.0	0.0

RAG1	0.1	0.0	0.0	0.0	0.0	0.0	0.1	0.0	0.1
MGAT4C	0.1	0.0	0.1	0.0	0.0	0.1	0.0	0.0	0.0
NKX2-1-AS1	0.1	0.0	0.1	0.0	0.0	0.0	0.0	0.0	0.0
NT5C1B	0.1	0.0	0.1	0.0	0.0	0.0	0.0	0.0	0.1
SYT2	0.1	0.0	0.0	0.0	0.0	0.0	0.1	0.0	0.0
BMP6	0.0	0.0	0.1	0.0	0.1	0.0	0.1	0.0	0.1
CHRNB2	0.2	0.1	0.0	0.0	0.0	0.0	0.0	0.0	0.0
SELL	0.1	0.1	0.0	0.0	0.0	0.0	0.1	0.0	0.0
BEST4	0.1	0.0	0.1	0.0	0.0	0.0	0.0	0.0	0.0
ZNF208	0.1	0.0	0.0	0.0	0.0	0.0	0.0	0.0	0.0
LOC100505978	0.1	0.0	0.1	0.0	0.0	0.0	0.0	0.0	0.1
LOC345051	0.1	0.0	0.1	0.0	0.0	0.0	0.0	0.0	0.0
LOC285740	0.1	0.0	0.1	0.0	0.0	0.0	0.1	0.0	0.0
TPRG1	0.1	0.0	0.0	0.0	0.0	0.1	0.1	0.0	0.0
TPTE2	0.2	0.0	0.0	0.0	0.0	0.0	0.1	0.0	0.0
ABCB5	0.1	0.0	0.1	0.0	0.1	0.0	0.1	0.0	0.0
LOC101929215	0.1	0.0	0.1	0.0	0.0	0.0	0.0	0.0	0.0
ZP1	0.1	0.0	0.1	0.0	0.0	0.0	0.0	0.0	0.0
LOC101928159	0.0	0.0	0.1	0.0	0.0	0.1	0.0	0.0	0.1
LOC100996643	0.0	0.0	0.1	0.0	0.0	0.0	0.1	0.0	0.0
C21orf15	0.1	0.0	0.1	0.0	0.0	0.0	0.1	0.0	0.0
NCF4	0.0	0.1	0.1	0.0	0.0	0.0	0.1	0.0	0.0
MGC16025	0.1	0.0	0.0	0.0	0.0	0.0	0.1	0.0	0.0
LOC100506497	0.1	0.0	0.0	0.0	0.0	0.0	0.1	0.0	0.0
RADIL	0.1	0.0	0.1	0.0	0.0	0.0	0.0	0.0	0.0
SNRPN	0.1	0.0	0.0	0.0	0.0	0.0	0.1	0.0	0.0
HTR6	0.1	0.0	0.1	0.0	0.0	0.0	0.1	0.0	0.0
BCL11B	0.1	0.0	0.1	0.0	0.0	0.0	0.0	0.0	0.1
MYO1G	0.0	0.0	0.0	0.0	0.0	0.0	0.1	0.0	0.1
COL4A2-AS2	0.1	0.0	0.0	0.0	0.0	0.0	0.1	0.0	0.1
RNF216-IT1	0.0	0.0	0.1	0.0	0.0	0.1	0.0	0.0	0.0
CDCP2	0.1	0.0	0.0	0.0	0.0	0.0	0.1	0.0	0.0
LOC101927843	0.1	0.0	0.1	0.0	0.0	0.0	0.1	0.0	0.0
LOC101929002	0.0	0.0	0.0	0.0	0.0	0.0	0.1	0.0	0.0
CDH8	0.1	0.0	0.0	0.0	0.0	0.0	0.0	0.0	0.0
GATA3-AS1	0.1	0.0	0.0	0.0	0.0	0.0	0.1	0.0	0.0
LOC101928034	0.1	0.0	0.0	0.0	0.0	0.0	0.0	0.0	0.0
HAS1	0.1	0.0	0.0	0.0	0.0	0.0	0.0	0.0	0.1
PRSS55	0.0	0.0	0.0	0.0	0.0	0.1	0.0	0.0	0.1
PKD1L3	0.1	0.0	0.1	0.0	0.0	0.0	0.0	0.0	0.0
LOC286367	0.1	0.0	0.0	0.0	0.0	0.0	0.0	0.0	0.0
PURG	0.1	0.0	0.0	0.0	0.0	0.0	0.1	0.0	0.0
LOC101927830	0.1	0.0	0.0	0.0	0.0	0.1	0.0	0.0	0.0
PKDCC	0.1	0.1	0.0	0.0	0.0	0.0	0.0	0.0	0.0
SOHLH2	0.1	0.0	0.1	0.0	0.0	0.0	0.0	0.0	0.0
FAM184A	0.1	0.0	0.0	0.0	0.0	0.0	0.0	0.0	0.0
FPR1	0.1	0.0	0.0	0.0	0.0	0.0	0.0	0.0	0.0
LOC101928519	0.1	0.0	0.0	0.0	0.0	0.0	0.0	0.0	0.0
LOC100506368	0.1	0.0	0.0	0.0	0.0	0.0	0.0	0.0	0.1
KCNC3	0.1	0.0	0.0	0.0	0.0	0.0	0.0	0.0	0.0
LOC100128002	0.1	0.0	0.0	0.0	0.0	0.0	0.1	0.0	0.0
TEX11	0.1	0.0	0.0	0.0	0.0	0.0	0.1	0.0	0.0
PRPH2	0.2	0.0	0.0	0.0	0.0	0.0	0.0	0.0	0.0
LINC00865	0.1	0.0	0.0	0.0	0.0	0.0	0.0	0.0	0.0
LOC441179	0.1	0.0	0.0	0.0	0.0	0.0	0.1	0.0	0.0

PCCA-AS1	0.2	0.0	0.0	0.0	0.0	0.0	0.0	0.0	0.0
NUTM1	0.1	0.0	0.0	0.0	0.0	0.0	0.1	0.0	0.0
LINC00550	0.1	0.0	0.0	0.0	0.0	0.0	0.1	0.0	0.0
SRMS	0.1	0.0	0.0	0.0	0.0	0.0	0.0	0.0	0.0
DPH6-AS1	0.1	0.0	0.0	0.0	0.0	0.1	0.0	0.0	0.0
ST6GALNAC1	0.1	0.0	0.0	0.0	0.0	0.0	0.0	0.0	0.1
MPL	0.1	0.0	0.0	0.0	0.0	0.0	0.0	0.0	0.0
SPRY3	0.0	0.0	0.0	0.0	0.0	0.0	0.1	0.0	0.0
KLHDC7B	0.0	0.0	0.0	0.0	0.0	0.0	0.1	0.0	0.0
LONRF2	0.1	0.0	0.0	0.0	0.0	0.0	0.1	0.0	0.0
FLJ46284	0.1	0.1	0.0	0.0	0.0	0.0	0.0	0.0	0.0
PLEKHA7	0.0	0.0	0.0	0.0	0.0	0.0	0.1	0.0	0.1
KCNK2	0.0	0.1	0.0	0.0	0.0	0.0	0.0	0.0	0.0
TPO	0.1	0.0	0.0	0.0	0.0	0.0	0.0	0.0	0.1
P2RY8	0.1	0.0	0.0	0.0	0.0	0.0	0.1	0.0	0.0
P2RY8	0.1	0.0	0.0	0.0	0.0	0.0	0.1	0.0	0.0
BTBD16	0.1	0.0	0.0	0.0	0.0	0.0	0.0	0.0	0.0
C1orf228	0.1	0.0	0.0	0.0	0.0	0.0	0.0	0.0	0.0
CD72	0.0	0.0	0.0	0.0	0.0	0.1	0.0	0.0	0.0
RIPK3	0.1	0.0	0.0	0.0	0.0	0.0	0.0	0.0	0.0
LOC101927586	0.0	0.1	0.1	0.0	0.0	0.0	0.0	0.0	0.0
SFTPB	0.1	0.0	0.0	0.0	0.0	0.1	0.0	0.0	0.0
LRRN2	0.1	0.0	0.0	0.0	0.0	0.0	0.0	0.0	0.0
CLRN1	0.1	0.0	0.0	0.0	0.0	0.0	0.0	0.0	0.1
DES	0.1	0.0	0.0	0.0	0.0	0.0	0.0	0.0	0.0
ZDHHC19	0.1	0.0	0.0	0.0	0.0	0.0	0.0	0.0	0.0
ITIH2	0.1	0.1	0.0	0.0	0.0	0.0	0.0	0.0	0.0
EGF	0.0	0.0	0.0	0.0	0.0	0.1	0.0	0.0	0.0
TOX3	0.0	0.0	0.0	0.0	0.0	0.1	0.0	0.0	0.0
LOC101928667	0.1	0.0	0.0	0.0	0.0	0.0	0.1	0.0	0.0
LOC100288897	0.1	0.0	0.0	0.0	0.0	0.0	0.0	0.0	0.0
STK31	0.1	0.0	0.0	0.0	0.0	0.0	0.0	0.0	0.0
LOC100507395	0.1	0.0	0.0	0.0	0.0	0.0	0.0	0.0	0.0
LOC101927277	0.1	0.0	0.0	0.0	0.0	0.0	0.0	0.0	0.0
KLKB1	0.1	0.0	0.0	0.0	0.0	0.0	0.0	0.0	0.0
TNNI3K	0.1	0.0	0.0	0.0	0.0	0.0	0.0	0.0	0.0
JAK3	0.1	0.0	0.0	0.0	0.0	0.0	0.0	0.0	0.0
PIR	0.0	0.0	0.0	0.0	0.0	0.0	0.1	0.0	0.0
KEL	0.1	0.0	0.0	0.0	0.0	0.0	0.0	0.0	0.0
LOC100505921	0.1	0.0	0.0	0.0	0.0	0.0	0.0	0.0	0.0
LOC100507489	0.1	0.0	0.0	0.0	0.0	0.0	0.0	0.0	0.0
TRPC6	0.0	0.0	0.0	0.0	0.0	0.0	0.0	0.0	0.0
SNAP25-AS1	0.0	0.0	0.0	0.0	0.0	0.0	0.1	0.0	0.0
MYT1L	0.0	0.0	0.0	0.0	0.0	0.0	0.0	0.0	0.1
AXDND1	0.1	0.0	0.0	0.0	0.0	0.0	0.0	0.0	0.0
CCDC73	0.0	0.0	0.0	0.0	0.0	0.0	0.0	0.0	0.0
LINC00535	0.1	0.0	0.0	0.0	0.0	0.0	0.0	0.0	0.0
GAREML	0.0	0.0	0.0	0.0	0.0	0.0	0.0	0.0	0.0
CDH23	0.0	0.0	0.0	0.0	0.0	0.0	0.1	0.0	0.0
SPRY3	0.0	0.0	0.0	0.0	0.0	0.0	0.0	0.0	0.0
LOC101928692	0.1	0.0	0.0	0.0	0.0	0.0	0.0	0.0	0.0
FLJ33581	0.1	0.0	0.0	0.0	0.0	0.0	0.0	0.0	0.0
LOC441009	0.0	0.0	0.0	0.0	0.0	0.0	0.0	0.0	0.0
C1orf141	0.0	0.0	0.0	0.0	0.0	0.0	0.0	0.0	0.0
NID1	0.0	0.0	0.0	0.0	0.0	0.0	0.1	0.0	0.0

CDHR2	0.0	0.0	0.0	0.0	0.0	0.0	0.0	0.0	0.0
LOC101927360	0.1	0.0	0.1	0.0	0.0	0.0	0.0	0.0	0.0
MATN1	0.1	0.0	0.0	0.0	0.0	0.0	0.0	0.0	0.0
HR	0.0	0.0	0.0	0.0	0.0	0.0	0.0	0.0	0.0
DGCR5	0.1	0.0	0.0	0.0	0.0	0.0	0.0	0.0	0.0
KRT85	0.0	0.0	0.1	0.0	0.0	0.0	0.0	0.0	0.0
FYB	0.0	0.0	0.0	0.0	0.0	0.0	0.0	0.0	0.0
SPATA18	0.0	0.0	0.0	0.0	0.0	0.0	0.0	0.0	0.0
LINC00940	0.0	0.0	0.0	0.0	0.0	0.0	0.0	0.0	0.0
LOC100507053	0.0	0.0	0.0	0.0	0.0	0.0	0.0	0.0	0.0
COL24A1	0.0	0.0	0.0	0.0	0.0	0.0	0.0	0.0	0.0
DKFZP434A062	0.0	0.0	0.0	0.0	0.0	0.0	0.0	0.0	0.0
LOC283482	0.1	0.0	0.0	0.0	0.0	0.0	0.0	0.0	0.0
GRIA1	0.0	0.0	0.0	0.0	0.0	0.0	0.0	0.0	0.0
FAM83B	0.0	0.0	0.0	0.0	0.0	0.0	0.0	0.0	0.0
PPFIA4	0.0	0.0	0.0	0.0	0.0	0.0	0.0	0.0	0.0
GLIPR1L2	0.0	0.0	0.0	0.0	0.0	0.0	0.0	0.0	0.0
LOC101928182	0.0	0.0	0.0	0.0	0.0	0.0	0.0	0.0	0.0
ACTG2	0.1	0.0	0.0	0.0	0.0	0.0	0.0	0.0	0.0
LOC283038	0.1	0.0	0.0	0.0	0.0	0.0	0.0	0.0	0.0
LOC101927378	0.0	0.0	0.0	0.0	0.0	0.0	0.0	0.0	0.0
CTAGE9	0.1	0.0	0.0	0.0	0.0	0.0	0.0	0.0	0.0
ZNF98	0.0	0.0	0.0	0.0	0.0	0.1	0.0	0.0	0.0
OTOA	0.0	0.0	0.0	0.0	0.0	0.0	0.0	0.0	0.0
IQCJ-SCHIP1	0.0	0.0	0.0	0.0	0.0	0.0	0.0	0.0	0.0
VWA3B	0.0	0.0	0.0	0.0	0.0	0.0	0.0	0.0	0.0
KCNIP4-IT1	0.0	0.0	0.0	0.0	0.0	0.0	0.0	0.0	0.0
NOS2	0.0	0.0	0.0	0.0	0.0	0.0	0.0	0.0	0.0
FUT6	0.0	0.0	0.0	0.0	0.0	0.0	0.0	0.0	0.0
LPHN3	0.0	0.0	0.0	0.0	0.0	0.0	0.0	0.0	0.0
LRRK2	0.0	0.0	0.0	0.0	0.0	0.0	0.0	0.0	0.0
STAB1	0.0	0.0	0.0	0.0	0.0	0.0	0.0	0.0	0.0
ADAMTS4	0.0	0.0	0.0	0.0	0.0	0.0	0.0	0.0	0.0
DNAH12	0.0	0.0	0.0	0.0	0.0	0.0	0.0	0.0	0.0
DNAH3	0.0	0.0	0.0	0.0	0.0	0.0	0.0	0.0	0.0
AMY1C	0.0	0.0	0.0	0.0	0.0	0.0	0.0	0.0	0.0
AMY1B	0.0	0.0	0.0	0.0	0.0	0.0	0.0	0.0	0.0
AMY1A	0.0	0.0	0.0	0.0	0.0	0.0	0.0	0.0	0.0
USH2A	0.0	0.0	0.0	0.0	0.0	0.0	0.0	0.0	0.0
SSPO	0.0	0.0	0.0	0.0	0.0	0.0	0.0	0.0	0.0
SNORD49A	0.0	0.0	0.0	0.6	0.5	1.2	0.4	0.4	2.0
PCDHGB3	0.0	0.0	0.0	0.0	0.1	0.0	0.2	0.0	0.0
LOC400756	0.0	0.0	0.0	0.0	0.0	0.0	0.1	0.0	0.1
TRNAS21	3.9	0.9	1.9	0.0	0.0	0.0	5.8	2.9	4.8
VTRNA1-3	1.6	1.1	0.0	0.0	0.0	0.0	4.5	0.7	2.7
TRNAS16	0.2	0.4	0.2	0.0	0.0	0.0	8.1	0.1	2.4
CRIPT	2.5	0.8	1.3	0.0	0.0	0.0	2.6	0.0	1.0
S100P	1.4	0.7	1.2	0.0	0.0	0.0	0.9	0.0	0.9
ELOVL7	1.7	0.6	0.5	0.0	0.0	0.0	0.7	0.1	0.6
MIR181A2HG	0.9	0.2	0.5	0.0	0.0	0.0	0.8	0.1	0.3
VSTM1	0.6	0.2	0.4	0.0	0.0	0.0	1.0	0.1	0.6
LOC100996557	0.9	0.2	0.6	0.0	0.0	0.0	0.4	0.1	0.3
BEX5	0.6	0.1	0.3	0.0	0.0	0.0	1.1	0.1	0.4
LOC101927733	0.9	0.5	0.4	0.0	0.0	0.0	0.2	0.0	0.6
TMEM182	0.7	0.2	0.4	0.0	0.0	0.0	0.6	0.1	0.4

RHEBL1	0.8	0.1	0.2	0.0	0.0	0.0	0.6	0.1	0.5
MDFI	0.6	0.2	0.4	0.0	0.0	0.0	0.7	0.1	0.2
LOC101927246	0.9	0.1	0.1	0.0	0.0	0.0	0.8	0.0	0.3
HIST1H4I	0.5	0.1	0.3	0.0	0.0	0.0	0.9	0.1	0.4
TSPAN13	0.7	0.1	0.4	0.0	0.0	0.0	0.6	0.1	0.3
TFF2	0.8	0.4	0.2	0.0	0.0	0.0	0.4	0.1	0.3
LOC101927688	0.3	0.3	0.3	0.0	0.0	0.0	0.6	0.1	0.3
GCHFR	0.9	0.0	0.1	0.0	0.0	0.0	0.4	0.1	0.4
LOC101927895	0.4	0.3	0.2	0.0	0.0	0.0	0.3	0.1	0.5
KCNQ5-IT1	0.5	0.0	0.2	0.0	0.0	0.0	0.8	0.1	0.3
TRNAS23	0.4	0.4	0.3	0.0	0.0	0.0	0.5	0.1	0.1
TRNAS18	0.4	0.4	0.3	0.0	0.0	0.0	0.5	0.1	0.1
TRNAS17	0.4	0.4	0.3	0.0	0.0	0.0	0.5	0.1	0.1
GNRH1	0.6	0.0	0.5	0.0	0.0	0.0	0.5	0.1	0.1
LOC101928059	0.9	0.1	0.2	0.0	0.0	0.0	0.2	0.1	0.1
LOC101928249	0.5	0.2	0.3	0.0	0.0	0.0	0.5	0.0	0.1
LOC101928571	0.4	0.3	0.3	0.0	0.0	0.0	0.2	0.0	0.4
LOC101928344	0.5	0.1	0.2	0.0	0.0	0.0	0.5	0.0	0.2
C11orf70	0.3	0.3	0.3	0.0	0.0	0.0	0.2	0.1	0.2
ICOSLG	0.4	0.3	0.4	0.0	0.0	0.0	0.3	0.0	0.2
LOC101928395	0.2	0.0	0.2	0.0	0.0	0.0	0.5	0.1	0.5
LOC101060264	0.4	0.1	0.0	0.0	0.0	0.0	0.4	0.2	0.4
AKAP7	0.5	0.3	0.2	0.0	0.0	0.0	0.3	0.1	0.1
ZNF527	0.5	0.2	0.2	0.0	0.0	0.0	0.4	0.0	0.2
CAPN8	0.6	0.2	0.5	0.0	0.0	0.0	0.1	0.0	0.0
PDE6G	0.2	0.1	0.2	0.0	0.0	0.0	0.6	0.1	0.2
CT49	0.7	0.0	0.5	0.0	0.0	0.0	0.1	0.0	0.0
LOC101927209	0.8	0.0	0.0	0.0	0.0	0.0	0.5	0.0	0.0
LOC100506499	0.3	0.1	0.3	0.0	0.0	0.0	0.2	0.0	0.3
PIH1D2	0.5	0.1	0.2	0.0	0.0	0.0	0.3	0.0	0.2
LOC101928930	0.3	0.1	0.3	0.0	0.0	0.0	0.5	0.1	0.0
TRNAH6	0.1	0.1	0.2	0.0	0.0	0.0	0.3	0.1	0.6
TRNAH2	0.1	0.1	0.2	0.0	0.0	0.0	0.3	0.1	0.6
TRNAH1	0.1	0.1	0.2	0.0	0.0	0.0	0.3	0.1	0.6
TRNAH5	0.1	0.1	0.2	0.0	0.0	0.0	0.3	0.1	0.6
TRNAH3	0.1	0.1	0.2	0.0	0.0	0.0	0.3	0.1	0.6
TRNAH7	0.1	0.1	0.2	0.0	0.0	0.0	0.3	0.1	0.6
TRNAH9	0.1	0.1	0.2	0.0	0.0	0.0	0.3	0.1	0.6
TRNAH10	0.1	0.1	0.2	0.0	0.0	0.0	0.3	0.1	0.6
TRNAH4	0.1	0.1	0.2	0.0	0.0	0.0	0.3	0.1	0.6
TAS2R30	0.7	0.1	0.2	0.0	0.0	0.0	0.3	0.0	0.1
LRRC39	0.4	0.2	0.2	0.0	0.0	0.0	0.4	0.1	0.1
SNORD105	0.0	0.0	0.6	0.0	0.0	0.0	0.0	0.0	0.7
HBE1	0.4	0.1	0.1	0.0	0.0	0.0	0.2	0.1	0.3
LOC101927755	0.5	0.1	0.2	0.0	0.0	0.0	0.2	0.0	0.1
LOC101929115	0.5	0.2	0.0	0.0	0.0	0.0	0.2	0.1	0.2
CLEC2B	0.4	0.2	0.1	0.0	0.0	0.0	0.0	0.1	0.2
TRNAL26	0.2	0.1	0.1	0.0	0.0	0.0	0.4	0.1	0.1
TRNAL6	0.2	0.1	0.1	0.0	0.0	0.0	0.4	0.1	0.1
TRNAL14	0.2	0.1	0.1	0.0	0.0	0.0	0.4	0.1	0.1
TRNAL35	0.2	0.1	0.1	0.0	0.0	0.0	0.4	0.1	0.1
TRNAL30	0.2	0.1	0.1	0.0	0.0	0.0	0.4	0.1	0.1
TRNAL5	0.2	0.1	0.1	0.0	0.0	0.0	0.4	0.1	0.1
TRNAL38	0.2	0.1	0.1	0.0	0.0	0.0	0.4	0.1	0.1
HOGA1	0.3	0.2	0.2	0.0	0.0	0.0	0.2	0.0	0.2

LOC101927155	0.3	0.2	0.2	0.0	0.0	0.0	0.2	0.2	0.1
EFHD1	0.4	0.1	0.2	0.0	0.0	0.0	0.1	0.0	0.1
NHLRC4	0.2	0.1	0.1	0.0	0.0	0.0	0.3	0.1	0.3
CCPG1	0.8	0.0	0.1	0.0	0.0	0.0	0.0	0.1	0.0
LOC101927794	0.6	0.0	0.4	0.0	0.0	0.0	0.0	0.0	0.0
ONECUT3	0.1	0.3	0.1	0.0	0.0	0.0	0.1	0.1	0.1
LOC101927384	0.3	0.1	0.1	0.0	0.0	0.0	0.2	0.1	0.1
TAS2R43	0.5	0.0	0.2	0.0	0.0	0.0	0.2	0.0	0.0
RNF125	0.3	0.0	0.2	0.0	0.0	0.0	0.3	0.0	0.1
PLVAP	0.4	0.2	0.1	0.0	0.0	0.0	0.2	0.0	0.1
LOC101927315	0.3	0.1	0.2	0.0	0.0	0.0	0.2	0.0	0.1
LOC101927659	0.5	0.1	0.0	0.0	0.0	0.0	0.2	0.0	0.1
LOC101929690	0.3	0.1	0.2	0.0	0.0	0.0	0.2	0.0	0.1
SMOC1	0.3	0.1	0.2	0.0	0.0	0.0	0.2	0.0	0.1
FAM209B	0.3	0.2	0.2	0.0	0.0	0.0	0.1	0.1	0.1
CCDC147-AS1	0.4	0.0	0.1	0.0	0.0	0.0	0.2	0.1	0.1
NRN1L	0.2	0.1	0.1	0.0	0.0	0.0	0.3	0.1	0.1
TRNAC29	0.1	0.1	0.2	0.0	0.0	0.0	0.2	0.1	0.2
TRNAC9	0.1	0.1	0.2	0.0	0.0	0.0	0.2	0.1	0.2
TRNAC5	0.1	0.1	0.2	0.0	0.0	0.0	0.2	0.1	0.2
TRNAC16	0.1	0.1	0.2	0.0	0.0	0.0	0.2	0.1	0.2
RASL10B	0.2	0.0	0.1	0.0	0.0	0.0	0.4	0.0	0.2
LOC101928268	0.3	0.0	0.1	0.0	0.0	0.0	0.2	0.0	0.1
GJA4	0.4	0.1	0.1	0.0	0.0	0.0	0.1	0.0	0.0
PRKAG2-AS1	0.1	0.1	0.0	0.0	0.0	0.0	0.3	0.1	0.2
PLA2G10	0.2	0.1	0.2	0.0	0.0	0.0	0.3	0.1	0.0
STYK1	0.4	0.1	0.1	0.0	0.0	0.0	0.1	0.0	0.1
SLC40A1	0.3	0.1	0.2	0.0	0.0	0.0	0.2	0.0	0.0
HTR2B	0.2	0.0	0.1	0.0	0.0	0.0	0.2	0.0	0.3
LOC101929648	0.3	0.1	0.2	0.0	0.0	0.0	0.1	0.0	0.1
CRB3	0.0	0.2	0.1	0.0	0.0	0.0	0.2	0.1	0.2
LOC101927732	0.3	0.0	0.1	0.0	0.0	0.0	0.1	0.0	0.1
ZNF501	0.3	0.1	0.1	0.0	0.0	0.0	0.1	0.0	0.1
GPR62	0.2	0.1	0.1	0.0	0.0	0.0	0.2	0.0	0.1
SOX5	0.3	0.0	0.2	0.0	0.0	0.0	0.1	0.0	0.1
GPC5	0.2	0.1	0.1	0.0	0.0	0.0	0.2	0.0	0.1
OR9A4	0.3	0.0	0.0	0.0	0.0	0.0	0.2	0.0	0.1
CDKL4	0.2	0.2	0.1	0.0	0.0	0.0	0.2	0.0	0.1
C20orf203	0.0	0.1	0.2	0.0	0.0	0.0	0.2	0.1	0.1
WFDC13	0.1	0.1	0.2	0.0	0.0	0.0	0.1	0.0	0.1
DYNAP	0.2	0.1	0.0	0.0	0.0	0.0	0.2	0.0	0.1
C1QTNF4	0.1	0.1	0.1	0.0	0.0	0.0	0.3	0.1	0.1
LOC101928870	0.4	0.0	0.2	0.0	0.0	0.0	0.2	0.0	0.0
LOC101927146	0.2	0.1	0.1	0.0	0.0	0.0	0.1	0.0	0.1
CCDC148	0.1	0.1	0.2	0.0	0.0	0.0	0.1	0.0	0.1
CCT6B	0.3	0.1	0.2	0.0	0.0	0.0	0.0	0.0	0.0
SHC2	0.4	0.0	0.1	0.0	0.0	0.0	0.1	0.0	0.0
FAM81A	0.2	0.0	0.0	0.0	0.0	0.0	0.2	0.0	0.1
RASGRP1	0.3	0.1	0.1	0.0	0.0	0.0	0.1	0.0	0.1
CLDN5	0.2	0.1	0.0	0.0	0.0	0.0	0.2	0.1	0.0
LOC101928560	0.1	0.2	0.1	0.0	0.0	0.0	0.1	0.0	0.1
EGLN3	0.2	0.0	0.2	0.0	0.0	0.0	0.0	0.0	0.1
SEMA3D	0.1	0.0	0.1	0.0	0.0	0.0	0.1	0.1	0.2
TAS2R20	0.2	0.0	0.1	0.0	0.0	0.0	0.1	0.0	0.2
RIBC1	0.1	0.2	0.1	0.0	0.0	0.0	0.1	0.0	0.1

HS1BP3-IT1	0.2	0.0	0.0	0.0	0.0	0.0	0.0	0.0	0.0
ANO3	0.2	0.0	0.1	0.0	0.0	0.0	0.1	0.0	0.1
CHURC1-FNTB	0.0	0.2	0.2	0.0	0.0	0.0	0.0	0.0	0.2
C22orf15	0.2	0.0	0.1	0.0	0.0	0.0	0.2	0.1	0.1
LOC101927941	0.1	0.1	0.0	0.0	0.0	0.0	0.2	0.0	0.0
KRT15	0.2	0.1	0.1	0.0	0.0	0.0	0.1	0.0	0.0
TLL1	0.1	0.0	0.1	0.0	0.0	0.0	0.2	0.0	0.2
HOXD13	0.1	0.1	0.1	0.0	0.0	0.0	0.1	0.1	0.0
IL18R1	0.2	0.1	0.1	0.0	0.0	0.0	0.1	0.0	0.1
LOC100507530	0.1	0.1	0.1	0.0	0.0	0.0	0.1	0.0	0.1
LOC101927952	0.0	0.0	0.1	0.0	0.0	0.0	0.2	0.0	0.1
C4orf36	0.0	0.1	0.0	0.0	0.0	0.0	0.2	0.0	0.2
CRHR2	0.2	0.0	0.0	0.0	0.0	0.0	0.1	0.0	0.2
LHX3	0.2	0.1	0.1	0.0	0.0	0.0	0.1	0.0	0.1
ADRA1D	0.2	0.1	0.1	0.0	0.0	0.0	0.1	0.0	0.1
RFPL3S	0.2	0.0	0.1	0.0	0.0	0.0	0.0	0.0	0.1
LOC101929379	0.1	0.1	0.1	0.0	0.0	0.0	0.0	0.0	0.1
LOC101927043	0.2	0.0	0.1	0.0	0.0	0.0	0.0	0.0	0.1
DMRTB1	0.0	0.1	0.1	0.0	0.0	0.0	0.0	0.0	0.1
HBA1	0.2	0.1	0.1	0.0	0.0	0.0	0.0	0.0	0.0
HBA2	0.2	0.1	0.1	0.0	0.0	0.0	0.0	0.0	0.0
LOC101929519	0.1	0.2	0.0	0.0	0.0	0.0	0.1	0.0	0.0
OLFML2B	0.1	0.1	0.1	0.0	0.0	0.0	0.1	0.0	0.0
ADAM21	0.2	0.0	0.1	0.0	0.0	0.0	0.1	0.0	0.1
M1AP	0.1	0.0	0.1	0.0	0.0	0.0	0.0	0.0	0.1
FZD3	0.1	0.0	0.0	0.0	0.0	0.0	0.2	0.0	0.1
NALCN-AS1	0.2	0.0	0.0	0.0	0.0	0.0	0.1	0.0	0.0
CHRM1	0.2	0.1	0.0	0.0	0.0	0.0	0.0	0.0	0.1
H2BFWT	0.2	0.0	0.1	0.0	0.0	0.0	0.1	0.1	0.1
MBOAT4	0.2	0.1	0.1	0.0	0.0	0.0	0.1	0.0	0.1
LINC01007	0.2	0.0	0.1	0.0	0.0	0.0	0.1	0.0	0.0
LOC100996286	0.1	0.0	0.1	0.0	0.0	0.0	0.1	0.1	0.0
CD109	0.2	0.1	0.1	0.0	0.0	0.0	0.1	0.0	0.0
ARHGEF37	0.2	0.1	0.1	0.0	0.0	0.0	0.0	0.0	0.0
LOC101929323	0.0	0.0	0.1	0.0	0.0	0.0	0.1	0.1	0.1
MAB21L3	0.1	0.1	0.0	0.0	0.0	0.0	0.1	0.0	0.0
AGMO	0.1	0.0	0.1	0.0	0.0	0.0	0.1	0.0	0.1
ACHE	0.1	0.0	0.0	0.0	0.0	0.0	0.0	0.0	0.1
TTC3-AS1	0.2	0.1	0.0	0.0	0.0	0.0	0.1	0.0	0.0
LOC100505550	0.1	0.0	0.1	0.0	0.0	0.0	0.0	0.0	0.1
GPR19	0.1	0.0	0.0	0.0	0.0	0.0	0.1	0.0	0.1
POU4F3	0.1	0.1	0.0	0.0	0.0	0.0	0.1	0.0	0.1
GABRG1	0.1	0.1	0.1	0.0	0.0	0.0	0.0	0.0	0.1
GYS2	0.2	0.0	0.0	0.0	0.0	0.0	0.0	0.0	0.1
LOC101927192	0.2	0.0	0.1	0.0	0.0	0.0	0.0	0.0	0.0
C1orf101	0.1	0.1	0.1	0.0	0.0	0.0	0.1	0.0	0.1
ZNF572	0.0	0.1	0.1	0.0	0.0	0.0	0.1	0.0	0.1
LOC100132741	0.1	0.0	0.1	0.0	0.0	0.0	0.1	0.0	0.1
LINC00891	0.1	0.0	0.1	0.0	0.0	0.0	0.1	0.0	0.1
RNF182	0.1	0.0	0.0	0.0	0.0	0.0	0.1	0.0	0.1
CCDC81	0.1	0.0	0.1	0.0	0.0	0.0	0.1	0.0	0.0
FOXL2	0.1	0.1	0.0	0.0	0.0	0.0	0.1	0.0	0.0
HOXD11	0.1	0.0	0.0	0.0	0.0	0.0	0.1	0.1	0.0
IGKV1D-16	0.1	0.0	0.1	0.0	0.0	0.0	0.1	0.0	0.0
LOC100507194	0.1	0.0	0.0	0.0	0.0	0.0	0.1	0.0	0.0

CCDC30	0.2	0.0	0.0	0.0	0.0	0.0	0.0	0.0	0.1
CASS4	0.2	0.1	0.0	0.0	0.0	0.0	0.1	0.0	0.0
DDX43	0.2	0.0	0.1	0.0	0.0	0.0	0.0	0.0	0.0
TCF4	0.1	0.1	0.0	0.0	0.0	0.0	0.1	0.0	0.0
OR2H2	0.0	0.0	0.1	0.0	0.0	0.0	0.1	0.1	0.0
LOC101928160	0.1	0.1	0.0	0.0	0.0	0.0	0.1	0.0	0.0
LINC00896	0.1	0.0	0.0	0.0	0.0	0.0	0.1	0.0	0.1
P2RX2	0.2	0.0	0.1	0.0	0.0	0.0	0.0	0.0	0.0
LOC284751	0.1	0.1	0.0	0.0	0.0	0.0	0.1	0.0	0.1
FNDC7	0.1	0.0	0.0	0.0	0.0	0.0	0.1	0.0	0.0
TRHDE	0.1	0.1	0.0	0.0	0.0	0.0	0.0	0.0	0.1
FAM83H	0.2	0.0	0.0	0.0	0.0	0.0	0.0	0.0	0.0
HRH2	0.1	0.0	0.0	0.0	0.0	0.0	0.1	0.0	0.1
SLC16A10	0.1	0.0	0.0	0.0	0.0	0.0	0.1	0.0	0.1
LHFPL3-AS2	0.1	0.1	0.1	0.0	0.0	0.0	0.0	0.0	0.0
EN1	0.0	0.0	0.0	0.0	0.0	0.0	0.0	0.1	0.1
NAALAD2	0.2	0.0	0.0	0.0	0.0	0.0	0.1	0.0	0.0
LOC286178	0.1	0.0	0.0	0.0	0.0	0.0	0.1	0.0	0.1
LOC731223	0.0	0.0	0.1	0.0	0.0	0.0	0.0	0.1	0.1
ZNF214	0.1	0.0	0.0	0.0	0.0	0.0	0.0	0.0	0.0
LOC101928768	0.1	0.1	0.0	0.0	0.0	0.0	0.0	0.0	0.0
GRM1	0.1	0.1	0.1	0.0	0.0	0.0	0.1	0.0	0.0
LRRIQ1	0.1	0.0	0.0	0.0	0.0	0.0	0.0	0.0	0.0
ACR	0.1	0.0	0.1	0.0	0.0	0.0	0.0	0.0	0.0
SRRM3	0.0	0.0	0.0	0.0	0.0	0.0	0.0	0.1	0.1
IGKV1-27	0.1	0.0	0.1	0.0	0.0	0.0	0.1	0.0	0.0
FLJ41481	0.1	0.0	0.0	0.0	0.0	0.0	0.1	0.0	0.0
ADORA2A	0.0	0.1	0.1	0.0	0.0	0.0	0.1	0.0	0.0
ASIC2	0.1	0.0	0.0	0.0	0.0	0.0	0.0	0.0	0.1
LOC101927705	0.1	0.0	0.0	0.0	0.0	0.0	0.0	0.0	0.0
SLC27A2	0.1	0.0	0.0	0.0	0.0	0.0	0.1	0.0	0.0
ZFHX4-AS1	0.0	0.0	0.1	0.0	0.0	0.0	0.1	0.0	0.1
ARHGAP8	0.1	0.0	0.0	0.0	0.0	0.0	0.1	0.0	0.0
PIWIL2	0.1	0.0	0.0	0.0	0.0	0.0	0.1	0.0	0.0
RIMKLA	0.0	0.0	0.0	0.0	0.0	0.0	0.1	0.0	0.0
LOC101928605	0.0	0.1	0.0	0.0	0.0	0.0	0.0	0.1	0.0
CRHBP	0.1	0.0	0.0	0.0	0.0	0.0	0.0	0.0	0.0
LOC101927824	0.1	0.0	0.0	0.0	0.0	0.0	0.1	0.0	0.0
IQGAP2	0.1	0.0	0.0	0.0	0.0	0.0	0.0	0.0	0.0
HOXD8	0.1	0.0	0.0	0.0	0.0	0.0	0.0	0.0	0.0
DOK7	0.0	0.0	0.1	0.0	0.0	0.0	0.0	0.0	0.0
CKMT1A	0.0	0.0	0.0	0.0	0.0	0.0	0.0	0.0	0.0
ADAMTS13	0.1	0.0	0.0	0.0	0.0	0.0	0.0	0.0	0.0
PRF1	0.0	0.0	0.0	0.0	0.0	0.0	0.0	0.0	0.0
RNF157	0.0	0.0	0.0	0.0	0.0	0.0	0.0	0.0	0.0
DUOX2	0.1	0.0	0.0	0.0	0.0	0.0	0.0	0.0	0.0
PCDHAC1	0.1	0.0	0.1	0.0	0.0	0.0	0.0	0.0	0.0
TTC24	0.0	0.0	0.0	0.0	0.0	0.0	0.0	0.0	0.0
ITGAL	0.0	0.0	0.0	0.0	0.0	0.0	0.0	0.0	0.0
LOC101927120	0.1	0.0	0.0	0.0	0.0	0.0	0.1	0.0	0.0
ANKDD1B	0.0	0.0	0.0	0.0	0.0	0.0	0.0	0.0	0.1
HFM1	0.1	0.0	0.0	0.0	0.0	0.0	0.0	0.0	0.0
DTX1	0.1	0.0	0.0	0.0	0.0	0.0	0.0	0.0	0.0
NLRP14	0.0	0.0	0.0	0.0	0.0	0.0	0.0	0.0	0.0
NIM1	0.1	0.0	0.0	0.0	0.0	0.0	0.0	0.0	0.0

GOLGA6A	0.1	0.0	0.0	0.0	0.0	0.0	0.0	0.0	0.0
FAM106B	0.1	0.0	0.0	0.0	0.0	0.0	0.0	0.0	0.0
CT60	0.0	0.0	0.0	0.0	0.0	0.0	0.0	0.0	0.0
FER1L6	0.0	0.0	0.0	0.0	0.0	0.0	0.0	0.0	0.0
GOLGA6C	0.0	0.0	0.0	0.0	0.0	0.0	0.0	0.0	0.0
EXTL1	0.0	0.0	0.0	0.0	0.0	0.0	0.1	0.0	0.0
MYBPC1	0.0	0.0	0.0	0.0	0.0	0.0	0.0	0.0	0.0
TTC40	0.1	0.0	0.0	0.0	0.0	0.0	0.0	0.0	0.0
ITGAD	0.0	0.0	0.0	0.0	0.0	0.0	0.0	0.0	0.0
GRM4	0.0	0.0	0.0	0.0	0.0	0.0	0.0	0.0	0.0
LOC100505835	0.0	0.0	0.0	0.0	0.0	0.0	0.0	0.0	0.0
THSD7A	0.0	0.0	0.0	0.0	0.0	0.0	0.0	0.0	0.0
PCDHA4	0.0	0.0	0.0	0.0	0.0	0.0	0.0	0.0	0.0
FAM203B	0.0	0.0	0.0	0.0	0.0	0.0	0.0	0.0	0.0
PCDHA2	0.0	0.0	0.0	0.0	0.0	0.0	0.0	0.0	0.0
CRTAC1	0.0	0.0	0.0	0.0	0.0	0.0	0.0	0.0	0.0
ABCC11	0.0	0.0	0.0	0.0	0.0	0.0	0.0	0.0	0.0
RYR2	0.0	0.0	0.0	0.0	0.0	0.0	0.0	0.0	0.0
PCDHAC2	0.0	0.0	0.0	0.0	0.0	0.0	0.0	0.0	0.0
RNU5F-1	4.8	1.9	1.3	0.0	0.0	0.0	1.1	0.2	1.8
LOC101929534	0.8	0.1	0.0	0.0	0.0	0.0	0.8	0.1	0.2
LOC101927529	0.5	0.1	0.2	0.0	0.0	0.0	0.7	0.1	0.2
TRNAV23	0.4	0.5	0.1	0.0	0.0	0.0	0.4	0.1	0.3
LOC101929408	0.3	0.2	0.3	0.0	0.0	0.0	0.5	0.0	0.4
LOC101927169	0.9	0.1	0.1	0.0	0.0	0.0	0.1	0.0	0.0
LOC101927161	0.4	0.1	0.1	0.0	0.0	0.0	0.4	0.1	0.2
GREM2	0.3	0.1	0.5	0.0	0.0	0.0	0.3	0.1	0.1
ZPLD1	0.4	0.2	0.2	0.0	0.0	0.0	0.3	0.0	0.1
VAMP5	0.6	0.1	0.2	0.0	0.0	0.0	0.1	0.1	0.2
OOEP	0.5	0.1	0.1	0.0	0.0	0.0	0.2	0.0	0.2
GNRH2	0.4	0.1	0.3	0.0	0.0	0.0	0.1	0.1	0.0
CD53	0.3	0.1	0.0	0.0	0.0	0.0	0.2	0.1	0.2
LOC101929136	0.1	0.1	0.2	0.0	0.0	0.0	0.4	0.0	0.0
EAF2	0.2	0.0	0.2	0.0	0.0	0.0	0.2	0.0	0.2
IFNLR1	0.3	0.1	0.0	0.0	0.0	0.0	0.1	0.0	0.2
TAF1D	0.2	0.0	0.2	0.0	0.0	0.0	0.2	0.0	0.2
CD200	0.2	0.0	0.2	0.0	0.0	0.0	0.2	0.0	0.2
LOC101928375	0.1	0.0	0.0	0.0	0.0	0.0	0.3	0.1	0.2
TSPAN33	0.3	0.1	0.1	0.0	0.0	0.0	0.1	0.0	0.1
LOC100506114	0.4	0.1	0.1	0.0	0.0	0.0	0.1	0.0	0.0
USP17L2	0.4	0.0	0.1	0.0	0.0	0.0	0.1	0.0	0.0
RUNDC3B	0.2	0.1	0.1	0.0	0.0	0.0	0.1	0.1	0.1
LOC101929095	0.4	0.1	0.1	0.0	0.0	0.0	0.0	0.0	0.1
ANKRD2	0.1	0.1	0.1	0.0	0.0	0.0	0.2	0.0	0.1
ESPNL	0.2	0.0	0.0	0.0	0.0	0.0	0.2	0.1	0.1
LINC00659	0.3	0.1	0.1	0.0	0.0	0.0	0.0	0.0	0.0
FBXO16	0.2	0.1	0.1	0.0	0.0	0.0	0.0	0.0	0.0
IL21R	0.1	0.0	0.1	0.0	0.0	0.0	0.1	0.0	0.1
LOC101928636	0.1	0.1	0.0	0.0	0.0	0.0	0.0	0.1	0.1
LOC339807	0.3	0.0	0.1	0.0	0.0	0.0	0.1	0.0	0.0
CCDC13	0.1	0.1	0.1	0.0	0.0	0.0	0.1	0.0	0.1
LOC101928302	0.1	0.0	0.0	0.0	0.0	0.0	0.1	0.0	0.0
SGIP1	0.1	0.0	0.1	0.0	0.0	0.0	0.1	0.0	0.1
LOC100505875	0.1	0.1	0.0	0.0	0.0	0.0	0.1	0.0	0.1
CCND2	0.1	0.0	0.0	0.0	0.0	0.0	0.0	0.0	0.1

CCDC181	0.1	0.0	0.0	0.0	0.0	0.0	0.1	0.0	0.1
TAS2R50	0.2	0.0	0.1	0.0	0.0	0.0	0.0	0.0	0.0
LOC100996425	0.1	0.0	0.1	0.0	0.0	0.0	0.1	0.0	0.1
TMEM117	0.1	0.1	0.1	0.0	0.0	0.0	0.1	0.0	0.1
PKD1L2	0.2	0.0	0.0	0.0	0.0	0.0	0.1	0.0	0.0
LOC101928126	0.0	0.1	0.1	0.0	0.0	0.0	0.1	0.0	0.1
VILL	0.1	0.1	0.1	0.0	0.0	0.0	0.0	0.0	0.0
FOXE3	0.1	0.0	0.0	0.0	0.0	0.0	0.0	0.0	0.0
RAB11FIP4	0.1	0.0	0.1	0.0	0.0	0.0	0.0	0.0	0.0
HCG26	0.1	0.0	0.0	0.0	0.0	0.0	0.0	0.0	0.0
LOC284648	0.0	0.1	0.0	0.0	0.0	0.0	0.0	0.0	0.0
RSPH10B2	0.1	0.0	0.0	0.0	0.0	0.0	0.0	0.0	0.0
LOC101927190	0.0	0.0	0.0	0.0	0.0	0.0	0.0	0.0	0.0
EGFLAM	0.0	0.0	0.1	0.0	0.0	0.0	0.0	0.0	0.0
WISP1	0.0	0.0	0.0	0.0	0.0	0.0	0.0	0.0	0.0
NACAD	0.0	0.0	0.0	0.0	0.0	0.0	0.0	0.0	0.0
LCT	0.0	0.0	0.0	0.0	0.0	0.0	0.0	0.0	0.0
LOC389043	0.0	0.0	0.0	0.0	0.0	0.0	0.0	0.0	0.0
RSPH10B	0.0	0.0	0.0	0.0	0.0	0.0	0.0	0.0	0.0
XIRP1	0.0	0.0	0.0	0.0	0.0	0.0	0.0	0.0	0.0
PCDHA3	0.0	0.0	0.0	0.0	0.0	0.0	0.0	0.0	0.0
PCDHA8	0.0	0.0	0.0	0.0	0.0	0.0	0.0	0.0	0.0
PCDHA5	0.0	0.0	0.0	0.0	0.0	0.0	0.0	0.0	0.0
SNORD34	0.6	0.4	0.0	0.3	0.0	3.4	1.8	0.0	1.7
SNORD1C	1.0	0.5	0.0	0.0	0.5	1.3	2.3	0.0	2.5
SNORD31	2.3	0.4	0.0	1.2	0.0	0.8	1.3	0.0	1.7
SNORD24	1.6	0.3	0.0	0.8	0.0	0.4	0.8	0.0	1.1
RNU6-35P	2.3	0.0	0.6	0.2	0.2	0.0	0.4	0.0	0.5
SNORD74	0.6	0.0	0.4	0.0	0.5	0.4	0.8	0.0	0.8
SNORD12C	0.5	0.0	1.0	0.0	0.0	0.4	0.4	0.0	0.4
TRNAG3	0.0	0.0	0.6	0.3	0.0	0.4	0.8	0.0	0.1
TRNAL27	0.0	0.1	0.1	0.0	0.1	0.0	0.9	0.0	0.4
TRNAL1	0.0	0.1	0.1	0.0	0.1	0.0	0.9	0.0	0.4
TRNAL20	0.0	0.1	0.1	0.0	0.1	0.0	0.9	0.0	0.4
TRNAL32	0.0	0.1	0.1	0.0	0.1	0.0	0.9	0.0	0.4
LOC101926903	0.3	0.1	0.0	0.0	0.1	0.1	0.6	0.0	0.3
SNORD62B	0.7	0.0	0.0	0.1	0.0	0.0	0.6	0.0	0.0
SNORD62A	0.7	0.0	0.0	0.1	0.0	0.0	0.6	0.0	0.0
KCTD4	0.4	0.0	0.0	0.0	0.1	0.1	0.3	0.0	0.3
C9orf152	0.5	0.1	0.0	0.0	0.0	0.0	0.3	0.0	0.0
ACY1	0.1	0.1	0.0	0.0	0.2	0.1	0.3	0.0	0.2
LOC101927271	0.4	0.0	0.0	0.1	0.0	0.1	0.1	0.0	0.2
ODF3	0.7	0.0	0.1	0.0	0.0	0.1	0.0	0.0	0.0
LOC100506797	0.1	0.0	0.1	0.0	0.2	0.1	0.2	0.0	0.2
LOC100287290	0.4	0.0	0.1	0.0	0.0	0.1	0.1	0.0	0.1
FLJ32756	0.0	0.0	0.2	0.0	0.1	0.1	0.4	0.0	0.1
LOC340581	0.4	0.0	0.0	0.0	0.1	0.0	0.2	0.0	0.0
LYRM9	0.2	0.0	0.0	0.0	0.2	0.1	0.1	0.0	0.0
C6orf99	0.4	0.0	0.1	0.0	0.0	0.1	0.1	0.0	0.0
LOC100506374	0.1	0.0	0.0	0.1	0.0	0.0	0.1	0.0	0.1
TNFSF4	0.0	0.1	0.1	0.0	0.0	0.0	0.2	0.0	0.1
RNU6-48P	0.4	0.0	0.0	0.0	0.0	0.0	0.0	0.1	0.0
NANOS3	0.1	0.0	0.1	0.0	0.1	0.0	0.1	0.0	0.0
LOC101929272	0.2	0.1	0.0	0.0	0.0	0.1	0.1	0.0	0.0
RNU6-14P	0.0	0.0	0.4	0.0	0.0	0.0	0.0	0.1	0.0

HRASLS	0.0	0.0	0.0	0.0	0.0	0.1	0.1	0.0	0.2
HLA-DOB	0.1	0.0	0.0	0.0	0.1	0.0	0.2	0.0	0.1
LOC101929503	0.1	0.1	0.0	0.0	0.1	0.0	0.2	0.0	0.1
TOLLIP-AS1	0.2	0.1	0.0	0.0	0.0	0.0	0.1	0.0	0.1
LOC101929332	0.1	0.0	0.1	0.0	0.0	0.2	0.1	0.0	0.0
LOC101928190	0.2	0.0	0.1	0.0	0.0	0.0	0.1	0.0	0.1
LOC100505633	0.2	0.0	0.0	0.0	0.1	0.0	0.1	0.0	0.0
LINC00904	0.1	0.0	0.1	0.0	0.0	0.1	0.1	0.0	0.1
ZBTB20-AS1	0.2	0.0	0.2	0.0	0.0	0.0	0.1	0.0	0.0
LOC100506136	0.2	0.1	0.0	0.0	0.0	0.0	0.1	0.0	0.1
SGCD	0.1	0.0	0.0	0.0	0.0	0.1	0.1	0.0	0.0
FBXL22	0.1	0.0	0.1	0.0	0.0	0.1	0.0	0.0	0.0
LOC100240735	0.0	0.0	0.1	0.0	0.0	0.1	0.0	0.0	0.1
LOC101929117	0.1	0.0	0.1	0.0	0.0	0.0	0.1	0.0	0.1
ENHO	0.2	0.0	0.0	0.0	0.2	0.0	0.0	0.0	0.0
ASMT	0.1	0.0	0.1	0.0	0.0	0.1	0.0	0.0	0.1
ASMT	0.1	0.0	0.1	0.0	0.0	0.1	0.0	0.0	0.1
LOC100507554	0.1	0.0	0.0	0.0	0.0	0.0	0.1	0.0	0.1
GPR1	0.1	0.0	0.1	0.0	0.0	0.0	0.1	0.0	0.1
TPSAB1	0.0	0.0	0.1	0.0	0.0	0.0	0.1	0.0	0.1
FSD2	0.1	0.0	0.0	0.0	0.0	0.0	0.1	0.0	0.0
IGHG3	0.2	0.0	0.0	0.0	0.0	0.0	0.0	0.0	0.0
KCNJ1	0.1	0.0	0.1	0.0	0.0	0.0	0.0	0.0	0.0
STX1B	0.0	0.1	0.1	0.0	0.0	0.0	0.0	0.0	0.0
LOC101926948	0.1	0.0	0.0	0.0	0.0	0.0	0.1	0.0	0.0
LOC100129961	0.1	0.0	0.0	0.0	0.1	0.1	0.0	0.0	0.0
HLA-DQB2	0.0	0.0	0.0	0.0	0.0	0.0	0.0	0.0	0.1
VAT1L	0.0	0.1	0.0	0.0	0.0	0.0	0.0	0.0	0.0
FAM227B	0.1	0.0	0.0	0.0	0.0	0.0	0.1	0.0	0.1
LOC101927516	0.1	0.0	0.1	0.0	0.0	0.0	0.0	0.0	0.0
ACVRL1	0.1	0.0	0.1	0.0	0.0	0.0	0.0	0.0	0.0
LOC101927993	0.0	0.0	0.0	0.0	0.0	0.0	0.0	0.0	0.1
CPNE4	0.1	0.0	0.0	0.0	0.0	0.0	0.0	0.0	0.1
ANGPT1	0.1	0.0	0.0	0.0	0.0	0.1	0.0	0.0	0.0
NPY4R	0.0	0.0	0.0	0.0	0.1	0.0	0.0	0.0	0.1
LRRIQ4	0.0	0.0	0.0	0.0	0.0	0.1	0.1	0.0	0.1
OTP	0.0	0.0	0.1	0.0	0.0	0.0	0.0	0.0	0.0
IGSF5	0.0	0.0	0.0	0.0	0.0	0.0	0.0	0.0	0.0
INPP5D	0.0	0.0	0.0	0.0	0.0	0.0	0.1	0.0	0.1
LOC101929325	0.0	0.0	0.0	0.0	0.0	0.0	0.1	0.0	0.0
BIK	0.0	0.0	0.0	0.0	0.0	0.0	0.0	0.0	0.2
LY9	0.0	0.0	0.0	0.0	0.0	0.0	0.1	0.1	0.0
PPP1R16B	0.0	0.0	0.0	0.0	0.0	0.0	0.0	0.0	0.0
LYG2	0.0	0.0	0.1	0.0	0.0	0.0	0.0	0.0	0.0
LOC101927618	0.1	0.0	0.0	0.0	0.0	0.0	0.0	0.0	0.0
KRT12	0.0	0.0	0.0	0.0	0.0	0.0	0.0	0.0	0.1
IRF8	0.0	0.0	0.0	0.0	0.0	0.0	0.0	0.0	0.0
NEFH	0.0	0.0	0.0	0.0	0.0	0.0	0.0	0.0	0.0
FRMPD4	0.0	0.0	0.0	0.0	0.0	0.0	0.0	0.0	0.1
GRID1-AS1	0.0	0.0	0.0	0.0	0.0	0.0	0.1	0.0	0.0
KCNJ9	0.0	0.0	0.0	0.1	0.0	0.0	0.0	0.0	0.0
CPA5	0.0	0.0	0.0	0.0	0.1	0.0	0.0	0.0	0.0
AZU1	0.0	0.0	0.0	0.0	0.0	0.0	0.0	0.0	0.1
C1orf180	0.1	0.0	0.0	0.0	0.0	0.0	0.0	0.0	0.0
INSM1	0.0	0.0	0.0	0.0	0.0	0.0	0.0	0.0	0.0

LZTS1	0.0	0.0	0.0	0.0	0.0	0.0	0.0	0.0	0.0
MAEL	0.0	0.0	0.0	0.0	0.0	0.0	0.0	0.0	0.0
LMO3	0.1	0.0	0.0	0.0	0.0	0.0	0.0	0.0	0.0
HAAO	0.0	0.0	0.0	0.0	0.0	0.0	0.0	0.0	0.0
CSF3R	0.0	0.0	0.0	0.0	0.0	0.0	0.0	0.0	0.0
ITGA4	0.1	0.0	0.0	0.0	0.0	0.0	0.0	0.0	0.0
LOC100128505	0.0	0.0	0.0	0.0	0.0	0.0	0.0	0.0	0.0
LOC100507091	0.0	0.0	0.0	0.0	0.0	0.0	0.0	0.0	0.0
LOC101928474	0.1	0.0	0.0	0.0	0.0	0.0	0.0	0.0	0.0
OR4F21	0.1	0.0	0.0	0.0	0.0	0.0	0.0	0.0	0.0
BCL6B	0.0	0.0	0.0	0.0	0.0	0.0	0.0	0.0	0.0
GLT1D1	0.0	0.0	0.0	0.0	0.0	0.0	0.0	0.0	0.0
LOC402269	0.0	0.0	0.0	0.0	0.0	0.0	0.0	0.0	0.1
SP9	0.0	0.0	0.0	0.0	0.0	0.0	0.0	0.0	0.0
ADCY5	0.1	0.0	0.0	0.0	0.0	0.0	0.0	0.0	0.0
LINC00282	0.0	0.0	0.0	0.0	0.0	0.0	0.0	0.0	0.0
SLC26A4-AS1	0.0	0.0	0.0	0.0	0.0	0.0	0.0	0.0	0.0
GUCY2F	0.0	0.0	0.0	0.0	0.0	0.0	0.0	0.0	0.0
LOC101060553	0.0	0.0	0.0	0.0	0.0	0.0	0.0	0.0	0.0
DOCK8	0.0	0.0	0.0	0.0	0.0	0.0	0.0	0.0	0.0
GPR112	0.0	0.0	0.0	0.0	0.0	0.0	0.0	0.0	0.0
ADORA3	0.0	0.0	0.0	0.0	0.0	0.0	0.0	0.0	0.0
LOC101929724	0.0	0.0	0.0	0.0	0.0	0.0	0.0	0.0	0.0
SOX2-OT	0.0	0.0	0.0	0.0	0.0	0.0	0.0	0.0	0.0
LOC285484	0.0	0.0	0.0	0.0	0.0	0.0	0.0	0.0	0.0
CELF4	0.0	0.0	0.0	0.0	0.0	0.0	0.0	0.0	0.0
LOC101929038	0.0	0.0	0.0	0.0	0.0	0.0	0.0	0.0	0.0
ATP4A	0.0	0.0	0.0	0.0	0.0	0.0	0.0	0.0	0.0
USP17L15	0.0	0.0	0.0	0.0	0.0	0.0	0.0	0.0	0.0
NBPF7	0.0	0.0	0.0	0.0	0.0	0.0	0.0	0.0	0.0
SLC35G4	0.0	0.0	0.0	0.0	0.0	0.0	0.0	0.0	0.0
TCERG1L	0.0	0.0	0.0	0.0	0.0	0.0	0.0	0.0	0.0
C10orf10	1.4	0.4	0.7	0.0	0.1	0.1	0.0	0.0	0.0
C11orf44	0.8	0.1	0.4	0.2	0.5	0.8	0.0	0.0	0.0
BDKRB2	1.1	0.3	0.5	0.1	0.0	0.1	0.0	0.0	0.0
TRNAE19	0.5	0.2	0.1	0.2	0.1	0.4	0.0	0.0	0.0
TRNAE6	0.5	0.2	0.1	0.2	0.1	0.4	0.0	0.0	0.0
TRNAE5	0.5	0.2	0.1	0.2	0.1	0.4	0.0	0.0	0.0
TRNAE18	0.5	0.2	0.1	0.2	0.1	0.4	0.0	0.0	0.0
TRNAE22	0.5	0.2	0.1	0.2	0.1	0.4	0.0	0.0	0.0
TRNAE13	0.5	0.2	0.1	0.2	0.1	0.4	0.0	0.0	0.0
TRNAE21	0.5	0.2	0.1	0.2	0.1	0.4	0.0	0.0	0.0
TMEM183B	0.3	0.3	0.3	0.0	0.0	0.1	0.0	0.0	0.0
HRCT1	0.4	0.1	0.2	0.2	0.0	0.1	0.0	0.0	0.0
LOC101927863	0.4	0.1	0.2	0.1	0.1	0.0	0.0	0.0	0.0
LINC00920	0.4	0.1	0.1	0.0	0.0	0.0	0.0	0.0	0.0
LOC101929536	0.0	0.1	0.3	0.0	0.2	0.1	0.0	0.0	0.0
OR5B21	0.3	0.1	0.1	0.0	0.1	0.0	0.0	0.0	0.0
RARRES2	0.2	0.0	0.1	0.0	0.0	0.1	0.0	0.0	0.0
LRRTM4	0.3	0.0	0.1	0.0	0.0	0.0	0.0	0.0	0.0
TEX22	0.2	0.1	0.0	0.0	0.0	0.0	0.0	0.0	0.0
MADCAM1	0.3	0.0	0.0	0.0	0.0	0.1	0.0	0.0	0.0
SPOCK3	0.2	0.0	0.0	0.0	0.1	0.1	0.0	0.0	0.0
C1orf229	0.1	0.1	0.1	0.1	0.1	0.1	0.0	0.0	0.0
CETP	0.2	0.1	0.0	0.0	0.0	0.0	0.0	0.0	0.0

LOC101927502	0.2	0.0	0.0	0.0	0.0	0.1	0.0	0.0	0.0
MTNR1B	0.1	0.0	0.1	0.0	0.0	0.0	0.0	0.0	0.0
HTR1F	0.1	0.0	0.0	0.0	0.1	0.1	0.0	0.0	0.0
ZMIZ1-AS1	0.1	0.0	0.1	0.0	0.0	0.0	0.0	0.0	0.0
LINC00423	0.2	0.0	0.0	0.0	0.0	0.1	0.0	0.0	0.0
TNFAIP8L2-SCNM1	0.0	0.0	0.0	0.1	0.1	0.0	0.0	0.0	0.0
C1QTNF1	0.1	0.1	0.1	0.0	0.0	0.0	0.0	0.0	0.0
LOC101927503	0.2	0.0	0.0	0.0	0.0	0.0	0.0	0.0	0.0
CRLF2	0.1	0.0	0.0	0.0	0.1	0.0	0.0	0.0	0.0
CRLF2	0.1	0.0	0.0	0.0	0.1	0.0	0.0	0.0	0.0
FBXO15	0.1	0.0	0.1	0.0	0.1	0.0	0.0	0.0	0.0
GRM3	0.0	0.0	0.1	0.0	0.0	0.0	0.0	0.0	0.0
LOC101928921	0.0	0.1	0.0	0.0	0.1	0.0	0.0	0.0	0.0
LOC101928140	0.2	0.0	0.0	0.0	0.0	0.0	0.0	0.0	0.0
LOC101059935	0.1	0.0	0.0	0.0	0.0	0.0	0.0	0.0	0.0
CYP1B1-AS1	0.1	0.0	0.0	0.0	0.0	0.0	0.0	0.0	0.0
COL25A1	0.1	0.0	0.0	0.0	0.0	0.0	0.0	0.0	0.0
RBFOX1	0.1	0.0	0.0	0.0	0.0	0.0	0.0	0.0	0.0
LOC401134	0.0	0.0	0.0	0.0	0.0	0.0	0.0	0.0	0.0
LOC101929439	0.1	0.0	0.0	0.0	0.0	0.0	0.0	0.0	0.0
TIGD4	0.0	0.0	0.0	0.0	0.0	0.0	0.0	0.0	0.0
LOC100132813	0.0	0.0	0.0	0.0	0.0	0.0	0.0	0.0	0.0
LOC644086	0.2	0.1	0.1	0.1	0.1	0.0	0.0	0.0	0.0
RNU6-36P	0.0	0.0	0.0	0.0	0.0	0.0	0.0	0.5	0.0
CABP7	0.0	0.0	0.0	0.1	0.0	0.0	0.0	0.0	0.1
TAF1L	0.0	0.0	0.0	0.0	0.0	0.0	0.1	0.0	0.1
RNU6-1	0.0	0.0	0.0	0.0	0.0	0.0	0.0	0.1	0.0
CAPN14	0.0	0.0	0.0	0.0	0.0	0.0	0.0	0.0	0.0
LOC101929447	0.0	0.0	0.0	0.0	0.1	0.0	0.0	0.0	0.0
LOC101927068	0.0	0.0	0.0	0.0	0.0	0.0	0.0	0.0	0.0
KIRREL3-AS2	0.0	0.0	0.0	0.0	0.1	0.0	0.2	0.1	0.1
GABRG2	0.0	0.0	0.0	0.0	0.0	0.0	0.0	0.0	0.0
TRNAL8	1.9	0.0	0.3	0.0	0.0	0.0	1.4	0.1	0.5
TRNAA34	0.6	0.0	0.4	0.0	0.0	0.0	1.2	1.1	0.4
EIF5AL1	1.2	0.0	0.0	0.0	0.0	0.0	1.2	0.0	1.1
UQCRHL	0.9	0.0	0.3	0.0	0.0	0.0	0.3	0.0	0.0
LOC101928301	0.7	0.0	0.2	0.0	0.0	0.0	0.0	0.0	0.3
C15orf26	0.2	0.0	0.1	0.0	0.0	0.0	0.6	0.1	0.4
LOC101926902	0.0	0.2	0.4	0.0	0.0	0.0	0.4	0.0	0.0
TRNAY7	0.0	0.1	0.1	0.0	0.0	0.0	0.4	0.1	0.1
TRNAY10	0.0	0.1	0.1	0.0	0.0	0.0	0.4	0.1	0.1
TRNAY4	0.0	0.1	0.1	0.0	0.0	0.0	0.4	0.1	0.1
LPAR3	0.0	0.0	0.1	0.0	0.0	0.0	0.4	0.1	0.2
LOC101929212	0.3	0.0	0.2	0.0	0.0	0.0	0.2	0.0	0.0
TAS2R10	0.3	0.0	0.2	0.0	0.0	0.0	0.0	0.0	0.0
C2orf70	0.1	0.0	0.2	0.0	0.0	0.0	0.1	0.1	0.2
LOC100996412	0.3	0.0	0.1	0.0	0.0	0.0	0.1	0.0	0.0
LOC101928817	0.2	0.0	0.2	0.0	0.0	0.0	0.1	0.0	0.0
GJD4	0.2	0.0	0.1	0.0	0.0	0.0	0.1	0.0	0.1
LOC101927125	0.1	0.0	0.0	0.0	0.0	0.0	0.2	0.1	0.1
KLHDC9	0.3	0.0	0.0	0.0	0.0	0.0	0.1	0.0	0.0
C12orf71	0.1	0.0	0.1	0.0	0.0	0.0	0.1	0.0	0.0
CA1	0.2	0.0	0.1	0.0	0.0	0.0	0.1	0.0	0.1
ARL10	0.2	0.0	0.0	0.0	0.0	0.0	0.1	0.0	0.0
BARX2	0.2	0.0	0.0	0.0	0.0	0.0	0.1	0.0	0.0

OPA1-AS1	0.3	0.0	0.0	0.0	0.0	0.0	0.0	0.1	0.1
LOC100507172	0.2	0.0	0.0	0.0	0.0	0.0	0.2	0.0	0.1
JAM3	0.1	0.0	0.1	0.0	0.0	0.0	0.0	0.0	0.1
OGN	0.2	0.0	0.0	0.0	0.0	0.0	0.1	0.0	0.0
RGS7	0.1	0.0	0.1	0.0	0.0	0.0	0.0	0.0	0.1
LOC101927420	0.0	0.0	0.0	0.0	0.0	0.0	0.1	0.0	0.1
LOC728715	0.0	0.0	0.0	0.0	0.0	0.0	0.0	0.1	0.1
P2RY14	0.1	0.0	0.0	0.0	0.0	0.0	0.1	0.0	0.0
ISX	0.0	0.0	0.0	0.0	0.0	0.0	0.2	0.0	0.1
LOC101929702	0.1	0.0	0.0	0.0	0.0	0.0	0.0	0.1	0.0
RUNX1T1	0.2	0.0	0.1	0.0	0.0	0.0	0.0	0.0	0.0
CHST4	0.0	0.0	0.0	0.0	0.0	0.0	0.1	0.0	0.1
PSCA	0.1	0.0	0.0	0.0	0.0	0.0	0.0	0.0	0.0
C9orf163	0.1	0.0	0.0	0.0	0.0	0.0	0.1	0.0	0.0
PLEKHD1	0.0	0.0	0.1	0.0	0.0	0.0	0.1	0.0	0.0
LINC00595	0.0	0.0	0.1	0.0	0.0	0.0	0.0	0.0	0.0
C16orf11	0.0	0.0	0.0	0.0	0.0	0.0	0.1	0.0	0.1
TRIM63	0.0	0.0	0.1	0.0	0.0	0.0	0.0	0.0	0.0
KCNF1	0.1	0.0	0.0	0.0	0.0	0.0	0.0	0.0	0.0
CDK15	0.0	0.0	0.0	0.0	0.0	0.0	0.0	0.0	0.1
LOC101928098	0.0	0.0	0.1	0.0	0.0	0.0	0.0	0.0	0.0
ZNF806	0.1	0.0	0.0	0.0	0.0	0.0	0.0	0.0	0.0
OR2S2	0.0	0.0	0.0	0.0	0.0	0.0	0.1	0.0	0.0
LOR	0.0	0.0	0.0	0.0	0.0	0.0	0.0	0.0	0.0
LOC100287413	0.1	0.0	0.0	0.0	0.0	0.0	0.0	0.0	0.0
ZNF585A	0.1	0.0	0.0	0.0	0.0	0.0	0.0	0.0	0.0
KRT82	0.0	0.0	0.0	0.0	0.0	0.0	0.0	0.0	0.0
CHRFAM7A	0.0	0.0	0.1	0.0	0.0	0.0	0.0	0.0	0.0
LOC101927075	0.0	0.0	0.1	0.0	0.0	0.0	0.0	0.0	0.0
COX6B2	0.0	0.0	0.0	0.0	0.0	0.0	0.0	0.1	0.0
LOC101927545	0.0	0.0	0.0	0.0	0.0	0.0	0.1	0.0	0.0
FGFR3	0.0	0.0	0.0	0.0	0.0	0.0	0.0	0.0	0.0
FLT1	0.0	0.0	0.0	0.0	0.0	0.0	0.0	0.0	0.0
LOC100128276	0.0	0.0	0.0	0.0	0.0	0.0	0.0	0.0	0.0
MTOR-AS1	0.0	0.0	0.0	0.0	0.0	0.0	0.0	0.0	0.0
GOLGA6B	0.0	0.0	0.0	0.0	0.0	0.0	0.0	0.0	0.1
DIRC3	0.0	0.0	0.0	0.0	0.0	0.0	0.0	0.0	0.0
THBS2	0.0	0.0	0.0	0.0	0.0	0.0	0.0	0.0	0.0
KCNT2	0.0	0.0	0.0	0.0	0.0	0.0	0.0	0.0	0.0
USP17L1P	0.0	0.0	0.0	0.0	0.0	0.0	0.0	0.0	0.0
GOLGA6D	0.0	0.0	0.0	0.0	0.0	0.0	0.0	0.0	0.0
CACNA1I	0.0	0.0	0.0	0.0	0.0	0.0	0.0	0.0	0.0
KCNT1	0.0	0.0	0.0	0.0	0.0	0.0	0.0	0.0	0.0
TRNAR20	0.5	0.4	0.0	0.0	0.0	0.0	0.4	0.4	0.8
SCARNA23	0.6	0.0	0.2	0.0	0.0	0.0	0.7	0.0	0.0
SNORA70G	0.0	0.0	0.2	0.0	0.0	0.0	0.4	0.2	0.0
LOC101928332	0.1	0.0	0.0	0.0	0.0	0.0	0.6	0.0	0.0
MTUS2-AS1	0.1	0.0	0.0	0.0	0.0	0.0	0.2	0.0	0.1
LOC101927466	0.0	0.0	0.0	0.0	0.0	0.0	0.2	0.1	0.2
SOWAHA	0.1	0.0	0.1	0.0	0.0	0.0	0.1	0.0	0.1
LOC101927853	0.2	0.0	0.1	0.0	0.0	0.0	0.0	0.0	0.1
ZNF853	0.0	0.1	0.0	0.0	0.0	0.0	0.1	0.0	0.1
C1QTNF7	0.1	0.0	0.0	0.0	0.0	0.0	0.1	0.0	0.0
LOC101928299	0.1	0.0	0.0	0.0	0.0	0.0	0.0	0.0	0.1
CHST13	0.1	0.0	0.0	0.0	0.0	0.0	0.2	0.0	0.0

C16orf90	0.1	0.0	0.1	0.0	0.0	0.0	0.0	0.0	0.1
GPR32	0.0	0.0	0.1	0.0	0.0	0.0	0.1	0.1	0.0
USP3-AS1	0.1	0.0	0.0	0.0	0.0	0.0	0.1	0.0	0.1
NAT8	0.1	0.0	0.0	0.0	0.0	0.0	0.0	0.0	0.0
PPP1R27	0.0	0.1	0.0	0.0	0.0	0.0	0.1	0.0	0.1
FAM47E	0.0	0.0	0.0	0.0	0.0	0.0	0.1	0.0	0.0
KRT4	0.0	0.0	0.0	0.0	0.0	0.0	0.1	0.0	0.1
LOC100506557	0.0	0.0	0.1	0.0	0.0	0.0	0.1	0.0	0.0
DGCR9	0.1	0.0	0.0	0.0	0.0	0.0	0.0	0.0	0.0
SLC35F4	0.0	0.0	0.0	0.0	0.0	0.0	0.0	0.0	0.0
SLC9A2	0.0	0.0	0.0	0.0	0.0	0.0	0.0	0.0	0.0
TVP23A	0.0	0.0	0.0	0.0	0.0	0.0	0.0	0.0	0.0
LILRA3	0.0	0.0	0.0	0.0	0.0	0.0	0.1	0.0	0.0
UBQLNL	0.1	0.0	0.0	0.0	0.0	0.0	0.0	0.0	0.0
LOC101927149	0.1	0.0	0.0	0.0	0.0	0.0	0.0	0.0	0.0
ATP13A4	0.0	0.0	0.0	0.0	0.0	0.0	0.0	0.0	0.0
LOC339568	0.0	0.0	0.0	0.0	0.0	0.0	0.0	0.0	0.0
LOC100652770	0.0	0.0	0.0	0.0	0.0	0.0	0.0	0.0	0.0
PALM3	0.0	0.0	0.0	0.0	0.0	0.0	0.0	0.0	0.0
LOC348761	0.0	0.0	0.0	0.0	0.0	0.0	0.0	0.0	0.0
DACT1	0.0	0.0	0.0	0.0	0.0	0.0	0.0	0.0	0.0
MYT1	0.0	0.0	0.0	0.0	0.0	0.0	0.0	0.0	0.0
C6orf222	0.0	0.0	0.0	0.0	0.0	0.0	0.0	0.0	0.0
MRC1	0.0	0.0	0.0	0.0	0.0	0.0	0.0	0.0	0.0
FTHL17	0.0	0.0	0.0	0.0	0.0	0.0	0.0	0.0	0.0
LOC149684	0.0	0.0	0.0	0.0	0.0	0.0	0.0	0.0	0.0
ACAN	0.0	0.0	0.0	0.0	0.0	0.0	0.0	0.0	0.0
HSPE1-MOB4	0.0	0.0	0.0	0.0	0.0	0.0	0.0	0.0	0.0
ANXA8	0.0	0.0	0.0	0.0	0.0	0.1	0.0	0.0	0.0
GSTM5	0.0	0.0	0.0	0.0	0.0	0.0	0.1	0.0	0.0
CGB1	0.0	0.0	0.0	0.0	0.0	0.0	0.0	0.0	0.0
DUOXA2	0.0	0.0	0.0	0.0	0.0	0.0	0.0	0.0	0.1
HRK	0.0	0.0	0.0	0.0	0.0	0.0	0.0	0.0	0.0
LOC732265	0.0	0.0	0.0	0.0	0.0	0.0	0.0	0.0	0.0
VPS13A-AS1	0.0	0.0	0.0	0.0	0.0	0.1	1.0	0.0	0.0
OR2AG2	0.0	0.0	0.0	0.0	0.0	0.0	0.0	0.0	0.0
C9orf129	5.9	0.2	2.3	0.0	0.0	0.0	0.0	2.4	3.3
SNORA27	1.7	2.3	0.8	0.0	0.0	0.0	0.8	0.0	1.3
SNORA14A	2.7	0.4	0.6	0.0	0.0	0.0	1.5	0.0	0.5
NME2	0.0	0.0	0.0	0.0	0.0	0.0	0.0	0.0	5.6
SNORA58	1.0	0.7	0.5	0.0	0.0	0.0	1.3	0.0	1.1
SNORD58C	1.1	0.3	0.4	0.0	0.0	0.0	0.8	0.0	1.5
SNORD28	0.5	0.3	0.7	0.0	0.0	0.0	1.2	0.0	0.8
TRNAG28	0.4	0.2	0.6	0.0	0.0	0.0	0.0	0.4	1.2
TRNAG1	0.4	0.2	0.6	0.0	0.0	0.0	0.0	0.4	1.2
LOC101928544	1.2	0.2	0.5	0.0	0.0	0.0	0.4	0.0	0.3
SNORA46	1.2	0.2	0.8	0.0	0.0	0.0	0.2	0.0	0.3
PABPC3	0.6	0.8	0.0	0.0	0.0	0.0	1.0	0.0	0.0
SNORD23	0.0	0.4	0.0	0.0	0.0	0.0	0.9	0.0	0.9
LOC101928624	0.7	0.1	0.3	0.0	0.0	0.0	0.7	0.0	0.2
SNORD11B	0.5	0.5	0.6	0.0	0.0	0.0	0.3	0.0	0.3
LOC100133445	0.7	0.3	0.3	0.0	0.0	0.0	0.3	0.0	0.2
LOC101929233	0.6	0.2	0.0	0.0	0.0	0.0	0.3	0.0	0.3
SNORA28	0.0	0.6	0.4	0.0	0.0	0.0	0.4	0.0	0.4
SYNJ2BP-COX16	0.7	0.3	0.0	0.0	0.0	0.0	0.3	0.0	0.3

C12orf39	0.6	0.1	0.1	0.0	0.0	0.0	0.7	0.0	0.1
LOC101929057	0.7	0.1	0.2	0.0	0.0	0.0	0.3	0.0	0.4
RFESD	0.7	0.1	0.2	0.0	0.0	0.0	0.3	0.0	0.2
RBPMS2	0.5	0.2	0.3	0.0	0.0	0.0	0.2	0.0	0.3
VPREB3	0.4	0.1	0.2	0.0	0.0	0.0	0.4	0.0	0.2
KITLG	0.4	0.1	0.3	0.0	0.0	0.0	0.4	0.0	0.3
ATG10	0.3	0.4	0.3	0.0	0.0	0.0	0.3	0.0	0.2
LOC101928373	0.6	0.1	0.3	0.0	0.0	0.0	0.1	0.0	0.2
DNAJB7	0.5	0.2	0.2	0.0	0.0	0.0	0.2	0.0	0.1
LOC101928545	0.6	0.0	0.2	0.0	0.0	0.0	0.3	0.0	0.2
CEND1	0.4	0.2	0.1	0.0	0.0	0.0	0.4	0.0	0.1
C19orf59	0.2	0.1	0.3	0.0	0.0	0.0	0.4	0.0	0.1
PHOSPHO2	0.3	0.1	0.3	0.0	0.0	0.0	0.3	0.0	0.2
LOC101929365	0.4	0.3	0.2	0.0	0.0	0.0	0.2	0.0	0.0
LOC101927110	0.2	0.2	0.2	0.0	0.0	0.0	0.3	0.0	0.3
KANSL1-AS1	0.6	0.1	0.2	0.0	0.0	0.0	0.3	0.0	0.1
ZFAT-AS1	0.5	0.3	0.2	0.0	0.0	0.0	0.2	0.0	0.1
LOC101928158	0.7	0.1	0.2	0.0	0.0	0.0	0.1	0.0	0.1
LOC100129083	0.4	0.3	0.4	0.0	0.0	0.0	0.0	0.0	0.1
LOC101927500	0.6	0.0	0.2	0.0	0.0	0.0	0.2	0.0	0.0
LOC101929525	0.3	0.1	0.2	0.0	0.0	0.0	0.2	0.0	0.1
MED4-AS1	0.5	0.1	0.0	0.0	0.0	0.0	0.1	0.0	0.3
LOC101929296	0.3	0.1	0.1	0.0	0.0	0.0	0.4	0.0	0.2
LOC101929196	0.6	0.0	0.1	0.0	0.0	0.0	0.1	0.0	0.1
UBD	0.6	0.1	0.2	0.0	0.0	0.0	0.1	0.0	0.1
MYO16-AS1	0.4	0.1	0.4	0.0	0.0	0.0	0.1	0.0	0.0
C7orf69	0.3	0.1	0.1	0.0	0.0	0.0	0.3	0.0	0.1
CD79B	0.4	0.1	0.2	0.0	0.0	0.0	0.1	0.0	0.1
LDHAL6A	0.5	0.0	0.1	0.0	0.0	0.0	0.1	0.0	0.2
LOC101928038	0.3	0.1	0.0	0.0	0.0	0.0	0.4	0.0	0.1
LOC101928089	0.5	0.0	0.1	0.0	0.0	0.0	0.1	0.0	0.2
LOC101929458	0.1	0.2	0.2	0.0	0.0	0.0	0.2	0.0	0.1
SUMO4	0.3	0.1	0.1	0.0	0.0	0.0	0.1	0.0	0.2
LINC00705	0.2	0.1	0.2	0.0	0.0	0.0	0.2	0.0	0.2
VIP	0.2	0.2	0.1	0.0	0.0	0.0	0.1	0.0	0.1
C14orf182	0.4	0.2	0.2	0.0	0.0	0.0	0.0	0.0	0.1
LOC101927574	0.3	0.2	0.2	0.0	0.0	0.0	0.1	0.0	0.1
TRNAP16	0.2	0.1	0.1	0.0	0.0	0.0	0.2	0.0	0.2
TRNAP21	0.2	0.1	0.1	0.0	0.0	0.0	0.2	0.0	0.2
TRNAP19	0.2	0.1	0.1	0.0	0.0	0.0	0.2	0.0	0.2
TRNAP7	0.2	0.1	0.1	0.0	0.0	0.0	0.2	0.0	0.2
TRNAP3	0.2	0.1	0.1	0.0	0.0	0.0	0.2	0.0	0.2
PRR15L	0.4	0.1	0.1	0.0	0.0	0.0	0.1	0.0	0.0
ERICH2	0.5	0.0	0.1	0.0	0.0	0.0	0.2	0.0	0.0
LINC00515	0.3	0.1	0.0	0.0	0.0	0.0	0.1	0.0	0.3
C2CD4A	0.4	0.0	0.3	0.0	0.0	0.0	0.0	0.0	0.0
LOC101928902	0.2	0.1	0.1	0.0	0.0	0.0	0.1	0.0	0.1
AGRP	0.1	0.0	0.2	0.0	0.0	0.0	0.2	0.0	0.1
EBLN1	0.2	0.1	0.2	0.0	0.0	0.0	0.1	0.0	0.2
RDH16	0.2	0.1	0.2	0.0	0.0	0.0	0.2	0.0	0.0
LOC100506804	0.3	0.1	0.1	0.0	0.0	0.0	0.2	0.0	0.1
LOC101929270	0.1	0.0	0.2	0.0	0.0	0.0	0.1	0.0	0.1
CISH	0.2	0.0	0.0	0.0	0.0	0.0	0.2	0.0	0.0
LOC101928915	0.2	0.1	0.0	0.0	0.0	0.0	0.2	0.0	0.1
GNLY	0.2	0.1	0.1	0.0	0.0	0.0	0.1	0.0	0.1

SCRT2	0.0	0.2	0.1	0.0	0.0	0.0	0.0	0.1	0.1
KCNE2	0.1	0.2	0.0	0.0	0.0	0.0	0.2	0.0	0.2
TRBC2	0.0	0.1	0.0	0.0	0.0	0.0	0.4	0.0	0.1
LEAP2	0.3	0.0	0.2	0.0	0.0	0.0	0.1	0.0	0.0
LOC100507377	0.2	0.2	0.1	0.0	0.0	0.0	0.1	0.0	0.1
C17orf82	0.2	0.1	0.0	0.0	0.0	0.0	0.2	0.1	0.0
LOC101929584	0.2	0.1	0.0	0.0	0.0	0.0	0.2	0.0	0.0
EFCAB3	0.2	0.1	0.2	0.0	0.0	0.0	0.0	0.0	0.1
LINC00881	0.2	0.1	0.2	0.0	0.0	0.0	0.1	0.0	0.1
LOC100505909	0.3	0.1	0.0	0.0	0.0	0.0	0.2	0.0	0.1
TMEM59L	0.4	0.1	0.1	0.0	0.0	0.0	0.0	0.0	0.1
STX19	0.1	0.1	0.1	0.0	0.0	0.0	0.1	0.0	0.3
C9orf43	0.3	0.0	0.1	0.0	0.0	0.0	0.1	0.0	0.1
SNORA30	0.4	0.2	0.0	0.0	0.0	0.0	0.0	0.0	0.0
LOC101928003	0.1	0.1	0.1	0.0	0.0	0.0	0.2	0.0	0.1
ASB14	0.3	0.0	0.2	0.0	0.0	0.0	0.0	0.0	0.1
TULP2	0.2	0.1	0.1	0.0	0.0	0.0	0.1	0.0	0.1
ZNF596	0.3	0.1	0.1	0.0	0.0	0.0	0.1	0.0	0.0
FRK	0.2	0.1	0.1	0.0	0.0	0.0	0.1	0.0	0.1
LOC101929346	0.2	0.0	0.0	0.0	0.0	0.0	0.1	0.0	0.2
LOC101927314	0.2	0.0	0.1	0.0	0.0	0.0	0.2	0.0	0.1
GJB7	0.3	0.1	0.0	0.0	0.0	0.0	0.1	0.0	0.1
CYP39A1	0.2	0.0	0.2	0.0	0.0	0.0	0.1	0.0	0.0
LOC101929665	0.3	0.0	0.1	0.0	0.0	0.0	0.1	0.0	0.0
SPEF1	0.2	0.0	0.1	0.0	0.0	0.0	0.2	0.0	0.0
LOC101927972	0.2	0.0	0.1	0.0	0.0	0.0	0.1	0.0	0.1
LOC100287934	0.2	0.0	0.2	0.0	0.0	0.0	0.0	0.0	0.1
NXPH3	0.2	0.1	0.2	0.0	0.0	0.0	0.0	0.0	0.0
LOC100289361	0.1	0.1	0.1	0.0	0.0	0.0	0.1	0.0	0.0
LOC101928617	0.2	0.1	0.1	0.0	0.0	0.0	0.0	0.0	0.1
WNT11	0.1	0.0	0.1	0.0	0.0	0.0	0.1	0.0	0.1
LPAR5	0.1	0.1	0.0	0.0	0.0	0.0	0.1	0.0	0.1
SLC35G5	0.3	0.0	0.1	0.0	0.0	0.0	0.0	0.0	0.1
ABCC5-AS1	0.1	0.1	0.1	0.0	0.0	0.0	0.2	0.0	0.1
LOC101929706	0.2	0.1	0.1	0.0	0.0	0.0	0.2	0.0	0.1
RPS6KA2-IT1	0.2	0.1	0.1	0.0	0.0	0.0	0.1	0.1	0.0
LOC101928408	0.1	0.0	0.1	0.0	0.0	0.0	0.2	0.0	0.1
GPR87	0.2	0.0	0.0	0.0	0.0	0.0	0.2	0.0	0.1
LOC728228	0.1	0.1	0.1	0.0	0.0	0.0	0.1	0.0	0.1
TRAM1L1	0.1	0.1	0.1	0.0	0.0	0.0	0.1	0.0	0.1
SH3BGR	0.1	0.1	0.1	0.0	0.0	0.0	0.1	0.0	0.1
LOC101926933	0.1	0.1	0.1	0.0	0.0	0.0	0.0	0.0	0.1
FCGR1B	0.4	0.0	0.0	0.0	0.0	0.0	0.0	0.0	0.0
RPA4	0.2	0.0	0.2	0.0	0.0	0.0	0.0	0.0	0.1
C1QL3	0.2	0.0	0.2	0.0	0.0	0.0	0.1	0.0	0.0
MSTN	0.2	0.0	0.1	0.0	0.0	0.0	0.1	0.0	0.1
HPYR1	0.3	0.0	0.1	0.0	0.0	0.0	0.0	0.0	0.0
IFI27	0.2	0.1	0.1	0.0	0.0	0.0	0.0	0.0	0.1
TCEANC	0.1	0.1	0.0	0.0	0.0	0.0	0.2	0.0	0.1
LRRC2-AS1	0.0	0.1	0.0	0.0	0.0	0.0	0.2	0.1	0.0
TACR2	0.1	0.1	0.0	0.0	0.0	0.0	0.1	0.0	0.1
LOC101927322	0.1	0.0	0.0	0.0	0.0	0.0	0.1	0.0	0.1
TMC5	0.2	0.1	0.1	0.0	0.0	0.0	0.1	0.0	0.1
LOC101927492	0.2	0.0	0.1	0.0	0.0	0.0	0.1	0.0	0.1
CD38	0.2	0.1	0.0	0.0	0.0	0.0	0.1	0.0	0.0

LOC100507258	0.1	0.0	0.1	0.0	0.0	0.0	0.0	0.0	0.1
C4orf47	0.3	0.0	0.0	0.0	0.0	0.0	0.0	0.0	0.1
LOC101927783	0.1	0.1	0.1	0.0	0.0	0.0	0.1	0.0	0.1
LOC101928119	0.1	0.1	0.1	0.0	0.0	0.0	0.1	0.0	0.1
CALCRL	0.1	0.0	0.0	0.0	0.0	0.0	0.2	0.0	0.1
LOC101928191	0.2	0.1	0.1	0.0	0.0	0.0	0.1	0.0	0.0
MAP1LC3A	0.2	0.1	0.1	0.0	0.0	0.0	0.0	0.0	0.1
C10orf68	0.2	0.0	0.1	0.0	0.0	0.0	0.1	0.0	0.0
ACVR1C	0.1	0.0	0.1	0.0	0.0	0.0	0.0	0.0	0.0
GLT8D2	0.1	0.0	0.1	0.0	0.0	0.0	0.1	0.0	0.1
JPH4	0.1	0.1	0.1	0.0	0.0	0.0	0.1	0.0	0.0
CCDC116	0.2	0.1	0.1	0.0	0.0	0.0	0.1	0.0	0.0
FUT7	0.2	0.1	0.1	0.0	0.0	0.0	0.1	0.0	0.1
SLC44A3	0.2	0.1	0.1	0.0	0.0	0.0	0.0	0.0	0.0
TUBAL3	0.1	0.1	0.1	0.0	0.0	0.0	0.1	0.0	0.1
SLC26A4	0.1	0.0	0.0	0.0	0.0	0.0	0.1	0.0	0.1
ASCL5	0.1	0.1	0.0	0.0	0.0	0.0	0.1	0.1	0.0
LUZP2	0.1	0.0	0.1	0.0	0.0	0.0	0.1	0.0	0.0
ZNF843	0.2	0.0	0.1	0.0	0.0	0.0	0.0	0.0	0.0
LOC101927880	0.1	0.1	0.1	0.0	0.0	0.0	0.1	0.0	0.0
LOC101926959	0.2	0.0	0.1	0.0	0.0	0.0	0.1	0.0	0.0
LOC101928123	0.1	0.0	0.1	0.0	0.0	0.0	0.1	0.0	0.0
NDST3	0.2	0.0	0.1	0.0	0.0	0.0	0.0	0.0	0.0
PPP1R14D	0.1	0.0	0.0	0.0	0.0	0.0	0.1	0.0	0.0
SLC45A2	0.2	0.0	0.0	0.0	0.0	0.0	0.1	0.0	0.0
LOC101928486	0.1	0.1	0.0	0.0	0.0	0.0	0.1	0.0	0.1
KCNJ11	0.1	0.0	0.1	0.0	0.0	0.0	0.1	0.0	0.1
LOC101929686	0.0	0.1	0.1	0.0	0.0	0.0	0.2	0.0	0.0
KLHL30	0.1	0.1	0.1	0.0	0.0	0.0	0.0	0.0	0.1
PSORS1C2	0.2	0.0	0.0	0.0	0.0	0.0	0.1	0.0	0.0
LOC101929040	0.1	0.0	0.0	0.0	0.0	0.0	0.1	0.0	0.1
H2AFY2	0.0	0.0	0.1	0.0	0.0	0.0	0.1	0.0	0.1
TLR2	0.2	0.0	0.1	0.0	0.0	0.0	0.1	0.0	0.0
LINC00051	0.1	0.0	0.0	0.0	0.0	0.0	0.2	0.0	0.0
ATP6V1C2	0.1	0.1	0.0	0.0	0.0	0.0	0.0	0.0	0.0
LRRC70	0.1	0.0	0.0	0.0	0.0	0.0	0.0	0.0	0.2
LOC101926887	0.1	0.0	0.1	0.0	0.0	0.0	0.1	0.0	0.0
PEX5L	0.2	0.0	0.1	0.0	0.0	0.0	0.1	0.0	0.0
LRRC7	0.1	0.0	0.0	0.0	0.0	0.0	0.1	0.0	0.1
TEKT2	0.2	0.0	0.0	0.0	0.0	0.0	0.0	0.0	0.0
LOC101929713	0.1	0.0	0.0	0.0	0.0	0.0	0.1	0.0	0.0
LRRC66	0.2	0.1	0.1	0.0	0.0	0.0	0.0	0.0	0.0
TEX13B	0.2	0.1	0.0	0.0	0.0	0.0	0.0	0.0	0.0
NTN1	0.1	0.0	0.1	0.0	0.0	0.0	0.1	0.0	0.0
RAPGEF5	0.1	0.0	0.1	0.0	0.0	0.0	0.1	0.0	0.1
LOC101928823	0.2	0.0	0.0	0.0	0.0	0.0	0.1	0.0	0.1
LOC101928019	0.0	0.0	0.0	0.0	0.0	0.0	0.1	0.0	0.1
WIPF3	0.1	0.1	0.0	0.0	0.0	0.0	0.1	0.0	0.0
CT45A2	0.2	0.0	0.1	0.0	0.0	0.0	0.1	0.0	0.0
LOC400541	0.0	0.1	0.1	0.0	0.0	0.0	0.0	0.0	0.1
AANAT	0.2	0.0	0.1	0.0	0.0	0.0	0.0	0.0	0.0
TPRX1	0.1	0.1	0.0	0.0	0.0	0.0	0.1	0.0	0.1
KRT17	0.0	0.0	0.0	0.0	0.0	0.0	0.1	0.0	0.0
GRPR	0.1	0.1	0.0	0.0	0.0	0.0	0.1	0.0	0.0
SCEL	0.1	0.1	0.0	0.0	0.0	0.0	0.1	0.0	0.0

PHEX	0.1	0.0	0.1	0.0	0.0	0.0	0.1	0.0	0.1
GPM6B	0.1	0.0	0.0	0.0	0.0	0.0	0.0	0.0	0.0
DPEP2	0.0	0.0	0.1	0.0	0.0	0.0	0.1	0.0	0.1
SLC18A2	0.1	0.0	0.0	0.0	0.0	0.0	0.0	0.0	0.1
LOC101929023	0.2	0.0	0.0	0.0	0.0	0.0	0.0	0.0	0.0
PPP1R1C	0.1	0.0	0.0	0.0	0.0	0.0	0.1	0.0	0.1
LOC101928907	0.1	0.0	0.0	0.0	0.0	0.0	0.1	0.0	0.0
LOC101927217	0.1	0.0	0.0	0.0	0.0	0.0	0.1	0.0	0.1
LOC101927620	0.0	0.0	0.1	0.0	0.0	0.0	0.0	0.0	0.1
PIK3R5	0.0	0.0	0.1	0.0	0.0	0.0	0.1	0.0	0.1
LOC101929066	0.0	0.0	0.1	0.0	0.0	0.0	0.0	0.0	0.0
COL3A1	0.1	0.0	0.1	0.0	0.0	0.0	0.0	0.0	0.0
LRRN3	0.1	0.0	0.1	0.0	0.0	0.0	0.1	0.0	0.0
LOC100506134	0.1	0.0	0.1	0.0	0.0	0.0	0.0	0.0	0.0
LOC101928239	0.1	0.0	0.0	0.0	0.0	0.0	0.0	0.0	0.0
HSPA12A	0.1	0.1	0.0	0.0	0.0	0.0	0.0	0.0	0.0
OR2AE1	0.0	0.1	0.1	0.0	0.0	0.0	0.1	0.0	0.0
DKFZp667F0711	0.1	0.0	0.1	0.0	0.0	0.0	0.0	0.0	0.0
LOC101927071	0.1	0.0	0.1	0.0	0.0	0.0	0.1	0.0	0.0
H2BFM	0.0	0.0	0.0	0.0	0.0	0.0	0.1	0.0	0.1
MARVELD3	0.1	0.0	0.0	0.0	0.0	0.0	0.0	0.0	0.0
ICA1L	0.1	0.1	0.0	0.0	0.0	0.0	0.0	0.0	0.0
LOC101929499	0.0	0.0	0.1	0.0	0.0	0.0	0.0	0.0	0.0
LOC101928799	0.0	0.0	0.1	0.0	0.0	0.0	0.1	0.0	0.0
TMEM236	0.0	0.0	0.1	0.0	0.0	0.0	0.1	0.0	0.0
LOC101928829	0.0	0.0	0.0	0.0	0.0	0.0	0.0	0.0	0.1
LOC100996478	0.1	0.0	0.0	0.0	0.0	0.0	0.0	0.0	0.0
ZNF679	0.0	0.0	0.1	0.0	0.0	0.0	0.1	0.0	0.1
CD19	0.1	0.0	0.0	0.0	0.0	0.0	0.0	0.0	0.1
LOC101927592	0.1	0.0	0.0	0.0	0.0	0.0	0.0	0.0	0.0
EGR4	0.1	0.0	0.0	0.0	0.0	0.0	0.0	0.0	0.0
RAPGEF4-AS1	0.1	0.0	0.0	0.0	0.0	0.0	0.0	0.0	0.0
WEE2	0.0	0.0	0.1	0.0	0.0	0.0	0.1	0.0	0.0
LOC100169752	0.1	0.0	0.0	0.0	0.0	0.0	0.1	0.0	0.0
TF	0.0	0.0	0.0	0.0	0.0	0.0	0.0	0.0	0.1
TRIM72	0.0	0.0	0.0	0.0	0.0	0.0	0.1	0.0	0.0
LOC101927471	0.1	0.0	0.0	0.0	0.0	0.0	0.0	0.0	0.1
TMLHE-AS1	0.1	0.0	0.0	0.0	0.0	0.0	0.0	0.0	0.0
CCDC173	0.0	0.0	0.1	0.0	0.0	0.0	0.0	0.0	0.0
FAM95A	0.0	0.0	0.1	0.0	0.0	0.0	0.0	0.0	0.0
C17orf66	0.1	0.0	0.0	0.0	0.0	0.0	0.0	0.0	0.0
C10orf129	0.1	0.0	0.0	0.0	0.0	0.0	0.1	0.0	0.0
CT45A3	0.1	0.0	0.0	0.0	0.0	0.0	0.1	0.0	0.0
GPR110	0.1	0.0	0.0	0.0	0.0	0.0	0.0	0.0	0.0
SULT2B1	0.1	0.1	0.0	0.0	0.0	0.0	0.0	0.0	0.0
WDR49	0.1	0.0	0.0	0.0	0.0	0.0	0.0	0.0	0.0
LRRIQ3	0.1	0.0	0.0	0.0	0.0	0.0	0.0	0.0	0.0
CCNB3	0.1	0.0	0.0	0.0	0.0	0.0	0.0	0.0	0.0
FAM66E	0.1	0.0	0.0	0.0	0.0	0.0	0.1	0.0	0.0
SULF2	0.1	0.0	0.0	0.0	0.0	0.0	0.0	0.0	0.0
TGM4	0.0	0.0	0.0	0.0	0.0	0.0	0.1	0.0	0.1
USP41	0.0	0.0	0.1	0.0	0.0	0.0	0.0	0.0	0.1
LOC101927795	0.0	0.0	0.1	0.0	0.0	0.0	0.0	0.0	0.0
TLE6	0.1	0.0	0.0	0.0	0.0	0.0	0.0	0.0	0.0
EPX	0.1	0.0	0.0	0.0	0.0	0.0	0.0	0.0	0.0

SPARCL1		0.1	0.0	0.0	0.0	0.0	0.0	0.0	0.0	0.0
ARHGAP26-AS1		0.1	0.0	0.0	0.0	0.0	0.0	0.0	0.1	0.0
NLRC4		0.0	0.0	0.0	0.0	0.0	0.0	0.0	0.0	0.0
LOC101929344		0.0	0.0	0.0	0.0	0.0	0.0	0.1	0.0	0.0
ANKS1B		0.0	0.0	0.1	0.0	0.0	0.0	0.0	0.0	0.0
PLD4		0.1	0.0	0.0	0.0	0.0	0.0	0.1	0.0	0.0
FAM65B		0.0	0.0	0.0	0.0	0.0	0.0	0.0	0.0	0.1
SHISA9		0.0	0.0	0.0	0.0	0.0	0.0	0.1	0.0	0.0
WDR96		0.1	0.0	0.0	0.0	0.0	0.0	0.0	0.0	0.0
ZSCAN5B		0.0	0.0	0.0	0.0	0.0	0.0	0.1	0.0	0.0
CCDC120		0.1	0.0	0.0	0.0	0.0	0.0	0.0	0.0	0.0
	44818.0	0.0	0.0	0.0	0.0	0.0	0.0	0.1	0.0	0.0
LOC339593		0.0	0.0	0.0	0.0	0.0	0.0	0.0	0.0	0.0
SMAD9		0.1	0.0	0.0	0.0	0.0	0.0	0.0	0.0	0.0
CCDC7		0.0	0.0	0.0	0.0	0.0	0.0	0.0	0.0	0.0
HPVC1		0.1	0.0	0.0	0.0	0.0	0.0	0.0	0.0	0.0
SOWAHB		0.1	0.0	0.0	0.0	0.0	0.0	0.0	0.0	0.0
LINC00901		0.1	0.0	0.0	0.0	0.0	0.0	0.0	0.0	0.0
TUBB8		0.1	0.0	0.0	0.0	0.0	0.0	0.0	0.0	0.0
LOC101927003		0.0	0.0	0.0	0.0	0.0	0.0	0.0	0.0	0.0
IQUB		0.0	0.0	0.0	0.0	0.0	0.0	0.0	0.0	0.0
REN		0.0	0.0	0.1	0.0	0.0	0.0	0.0	0.0	0.0
TRPM5		0.1	0.0	0.0	0.0	0.0	0.0	0.0	0.0	0.0
CDH15		0.0	0.0	0.0	0.0	0.0	0.0	0.0	0.0	0.0
RORB		0.0	0.0	0.0	0.0	0.0	0.0	0.0	0.0	0.0
EFHB		0.0	0.0	0.0	0.0	0.0	0.0	0.0	0.0	0.0
LOC101927891		0.0	0.0	0.0	0.0	0.0	0.0	0.0	0.0	0.0
PAX6		0.0	0.0	0.0	0.0	0.0	0.0	0.0	0.0	0.0
GRIN1		0.0	0.0	0.0	0.0	0.0	0.0	0.0	0.0	0.0
RBM44		0.0	0.0	0.0	0.0	0.0	0.0	0.0	0.0	0.0
LOC100507506		0.1	0.0	0.0	0.0	0.0	0.0	0.0	0.0	0.0
PADI3		0.1	0.0	0.0	0.0	0.0	0.0	0.0	0.0	0.0
LOC101927035		0.0	0.0	0.0	0.0	0.0	0.0	0.1	0.0	0.0
LOC101928896		0.0	0.0	0.1	0.0	0.0	0.0	0.1	0.0	0.0
CTAGE15		0.1	0.0	0.0	0.0	0.0	0.0	0.0	0.0	0.0
XYLT1		0.0	0.0	0.0	0.0	0.0	0.0	0.0	0.0	0.0
TRIM60		0.0	0.0	0.0	0.0	0.0	0.0	0.0	0.0	0.0
LOC101928251		0.1	0.0	0.0	0.0	0.0	0.0	0.0	0.0	0.0
C16orf96		0.0	0.0	0.0	0.0	0.0	0.0	0.0	0.0	0.0
ZNF26		0.0	0.1	0.0	0.0	0.0	0.0	0.0	0.0	0.0
CNTN2		0.1	0.0	0.0	0.0	0.0	0.0	0.0	0.0	0.0
LOC100996634		0.0	0.0	0.0	0.0	0.0	0.0	0.1	0.0	0.0
TSPEAR		0.0	0.0	0.0	0.0	0.0	0.0	0.0	0.0	0.0
FAP		0.0	0.1	0.0	0.0	0.0	0.0	0.0	0.0	0.0
MAN1C1		0.0	0.0	0.0	0.0	0.0	0.0	0.0	0.0	0.0
CT45A6		0.0	0.0	0.0	0.0	0.0	0.0	0.0	0.0	0.0
CT45A4		0.0	0.0	0.0	0.0	0.0	0.0	0.0	0.0	0.0
ITGA9		0.1	0.0	0.0	0.0	0.0	0.0	0.0	0.0	0.0
CACNA1H		0.0	0.0	0.0	0.0	0.0	0.0	0.0	0.0	0.0
MAMDC2-AS1		0.0	0.0	0.0	0.0	0.0	0.0	0.0	0.0	0.0
GYLTL1B		0.0	0.0	0.0	0.0	0.0	0.0	0.0	0.0	0.0
LINC00351		0.0	0.0	0.0	0.0	0.0	0.0	0.0	0.0	0.0
LINC00552		0.0	0.0	0.0	0.0	0.0	0.0	0.0	0.0	0.0
AGBL3		0.0	0.0	0.0	0.0	0.0	0.0	0.0	0.0	0.0
INSRR		0.0	0.0	0.0	0.0	0.0	0.0	0.0	0.0	0.0

C22orf34	0.1	0.0	0.0	0.0	0.0	0.0	0.0	0.0	0.0
LOC101927721	0.0	0.0	0.0	0.0	0.0	0.0	0.0	0.0	0.0
NTSR1	0.0	0.0	0.0	0.0	0.0	0.0	0.0	0.0	0.0
IRX4	0.0	0.0	0.0	0.0	0.0	0.0	0.0	0.0	0.0
LOC101929530	0.0	0.0	0.0	0.0	0.0	0.0	0.0	0.0	0.0
DSPP	0.0	0.0	0.0	0.0	0.0	0.0	0.0	0.0	0.0
ABCA8	0.0	0.0	0.0	0.0	0.0	0.0	0.0	0.0	0.0
LINC00701	0.0	0.0	0.0	0.0	0.0	0.0	0.0	0.0	0.0
LOC100129316	0.0	0.0	0.0	0.0	0.0	0.0	0.0	0.0	0.0
LOC101929238	0.0	0.0	0.0	0.0	0.0	0.0	0.0	0.0	0.0
LOC101929263	0.0	0.0	0.0	0.0	0.0	0.0	0.0	0.0	0.0
ZNF729	0.0	0.0	0.0	0.0	0.0	0.0	0.0	0.0	0.0
POTEG	0.0	0.0	0.0	0.0	0.0	0.0	0.0	0.0	0.0
NT5C1B-RDH14	0.0	0.0	0.0	0.0	0.0	0.0	0.0	0.0	0.0
CT45A1	0.0	0.0	0.0	0.0	0.0	0.0	0.0	0.0	0.0
SPAG17	0.0	0.0	0.0	0.0	0.0	0.0	0.0	0.0	0.0
MUC12	0.0	0.0	0.0	0.0	0.0	0.0	0.0	0.0	0.0
USP17L4	0.0	0.0	0.0	0.0	0.0	0.0	0.0	0.0	0.0
TRNAG10	1.1	1.1	1.1	0.0	0.0	0.0	0.0	0.8	2.0
SNORD79	0.7	0.0	0.7	0.0	0.0	0.0	0.5	0.0	1.6
CMC4	1.0	0.5	0.8	0.0	0.0	0.0	0.3	0.0	0.8
RNU6-21P	0.7	0.0	0.7	0.0	0.0	0.0	0.6	0.0	0.3
CCL20	0.8	0.3	0.6	0.0	0.0	0.0	0.3	0.0	0.0
VTRNA1-2	0.0	0.6	0.6	0.0	0.0	0.0	0.3	0.0	0.3
GADD45G	0.5	0.2	0.3	0.0	0.0	0.0	0.4	0.0	0.1
KHDC1L	0.6	0.1	0.1	0.0	0.0	0.0	0.5	0.0	0.3
LOC101928529	0.3	0.2	0.3	0.0	0.0	0.0	0.2	0.0	0.2
VSTM5	0.6	0.2	0.2	0.0	0.0	0.0	0.1	0.0	0.0
TRNAK1	0.3	0.2	0.1	0.0	0.0	0.0	0.2	0.0	0.2
TRNAK3	0.3	0.2	0.1	0.0	0.0	0.0	0.2	0.0	0.2
TRNAK22	0.3	0.2	0.1	0.0	0.0	0.0	0.2	0.0	0.2
TRNAK34	0.3	0.2	0.1	0.0	0.0	0.0	0.2	0.0	0.2
TRNAK16	0.3	0.2	0.1	0.0	0.0	0.0	0.2	0.0	0.2
LOC100996668	0.5	0.1	0.1	0.0	0.0	0.0	0.1	0.0	0.3
FOLR1	0.3	0.2	0.2	0.0	0.0	0.0	0.1	0.0	0.1
GCSAM	0.3	0.1	0.3	0.0	0.0	0.0	0.3	0.0	0.0
LOC100506113	0.2	0.2	0.3	0.0	0.0	0.0	0.3	0.0	0.0
LOC101927201	0.2	0.1	0.2	0.0	0.0	0.0	0.1	0.0	0.2
C1orf100	0.0	0.1	0.2	0.0	0.0	0.0	0.0	0.0	0.5
LOC101928421	0.4	0.1	0.2	0.0	0.0	0.0	0.1	0.0	0.1
SCG5	0.3	0.0	0.1	0.0	0.0	0.0	0.2	0.0	0.1
MCCD1	0.4	0.1	0.1	0.0	0.0	0.0	0.0	0.0	0.1
TRNAD17	0.2	0.2	0.2	0.0	0.0	0.0	0.1	0.0	0.0
TRNAD16	0.2	0.2	0.2	0.0	0.0	0.0	0.1	0.0	0.0
TRNAD15	0.2	0.2	0.2	0.0	0.0	0.0	0.1	0.0	0.0
TRNAD12	0.2	0.2	0.2	0.0	0.0	0.0	0.1	0.0	0.0
TRNAD14	0.2	0.2	0.2	0.0	0.0	0.0	0.1	0.0	0.0
TRNAD8	0.2	0.2	0.2	0.0	0.0	0.0	0.1	0.0	0.0
TRNAD5	0.2	0.2	0.2	0.0	0.0	0.0	0.1	0.0	0.0
TRNAD6	0.2	0.2	0.2	0.0	0.0	0.0	0.1	0.0	0.0
TRNAD13	0.2	0.2	0.2	0.0	0.0	0.0	0.1	0.0	0.0
TRNAD11	0.2	0.2	0.2	0.0	0.0	0.0	0.1	0.0	0.0
TRNAD18	0.2	0.2	0.2	0.0	0.0	0.0	0.1	0.0	0.0
LOC100505592	0.3	0.2	0.1	0.0	0.0	0.0	0.1	0.0	0.0
PRH2	0.1	0.2	0.1	0.0	0.0	0.0	0.2	0.0	0.1

SAMD14	0.3	0.1	0.1	0.0	0.0	0.0	0.1	0.0	0.1
C2orf15	0.2	0.1	0.0	0.0	0.0	0.0	0.2	0.0	0.2
RAB42	0.2	0.0	0.1	0.0	0.0	0.0	0.2	0.0	0.0
TAGAP	0.2	0.1	0.1	0.0	0.0	0.0	0.1	0.0	0.1
LOC101929720	0.2	0.1	0.3	0.0	0.0	0.0	0.1	0.1	0.0
THRSP	0.2	0.2	0.1	0.0	0.0	0.0	0.1	0.0	0.0
LOC388813	0.1	0.0	0.3	0.0	0.0	0.0	0.1	0.0	0.1
GPR183	0.2	0.0	0.1	0.0	0.0	0.0	0.2	0.0	0.0
LOC100505683	0.2	0.0	0.1	0.0	0.0	0.0	0.2	0.0	0.0
LOC101928360	0.2	0.1	0.1	0.0	0.0	0.0	0.1	0.0	0.0
CELA2B	0.1	0.0	0.2	0.0	0.0	0.0	0.1	0.0	0.1
HSD3B2	0.2	0.0	0.0	0.0	0.0	0.0	0.2	0.0	0.1
ZNF365	0.3	0.0	0.2	0.0	0.0	0.0	0.0	0.0	0.0
ZNF790-AS1	0.3	0.1	0.0	0.0	0.0	0.0	0.0	0.0	0.0
TM4SF1-AS1	0.1	0.1	0.1	0.0	0.0	0.0	0.1	0.0	0.1
HGFAC	0.2	0.1	0.1	0.0	0.0	0.0	0.1	0.0	0.0
MYRFL	0.2	0.0	0.1	0.0	0.0	0.0	0.1	0.0	0.0
LOC100131060	0.1	0.0	0.1	0.0	0.0	0.0	0.1	0.0	0.1
LOC645262	0.1	0.1	0.0	0.0	0.0	0.0	0.2	0.0	0.1
VNN2	0.1	0.1	0.1	0.0	0.0	0.0	0.0	0.0	0.0
TRIM69	0.1	0.1	0.1	0.0	0.0	0.0	0.0	0.0	0.1
LOC100996288	0.1	0.0	0.1	0.0	0.0	0.0	0.1	0.0	0.1
VENTX	0.2	0.0	0.1	0.0	0.0	0.0	0.1	0.0	0.1
LOC101926975	0.2	0.0	0.1	0.0	0.0	0.0	0.1	0.0	0.1
LOC101929683	0.2	0.0	0.1	0.0	0.0	0.0	0.1	0.0	0.0
TAS1R1	0.2	0.0	0.1	0.0	0.0	0.0	0.0	0.0	0.0
DIRAS3	0.1	0.0	0.1	0.0	0.0	0.0	0.1	0.0	0.0
GIF	0.1	0.0	0.1	0.0	0.0	0.0	0.1	0.0	0.1
EFCAB12	0.2	0.0	0.1	0.0	0.0	0.0	0.0	0.0	0.0
MAPK10	0.2	0.0	0.1	0.0	0.0	0.0	0.0	0.0	0.0
A3GALT2	0.2	0.0	0.1	0.0	0.0	0.0	0.1	0.0	0.0
C20orf195	0.1	0.1	0.1	0.0	0.0	0.0	0.0	0.0	0.0
LOC101929542	0.2	0.0	0.0	0.0	0.0	0.0	0.1	0.0	0.0
GSX2	0.0	0.0	0.0	0.0	0.0	0.0	0.2	0.0	0.1
LOC388436	0.2	0.0	0.1	0.0	0.0	0.0	0.0	0.0	0.1
LOC101927980	0.2	0.0	0.1	0.0	0.0	0.0	0.0	0.0	0.0
LRRN4CL	0.2	0.0	0.1	0.0	0.0	0.0	0.0	0.0	0.0
NXNL1	0.1	0.1	0.1	0.0	0.0	0.0	0.0	0.0	0.0
GRK7	0.1	0.0	0.1	0.0	0.0	0.0	0.1	0.0	0.0
LOC101926979	0.1	0.0	0.1	0.0	0.0	0.0	0.1	0.0	0.0
LOC101928208	0.1	0.0	0.1	0.0	0.0	0.0	0.1	0.0	0.1
UROC1	0.1	0.0	0.0	0.0	0.0	0.0	0.1	0.0	0.1
ATAD3C	0.0	0.1	0.1	0.0	0.0	0.0	0.0	0.1	0.1
MIR4500HG	0.1	0.0	0.1	0.0	0.0	0.0	0.0	0.0	0.1
OCLM	0.0	0.0	0.3	0.0	0.0	0.0	0.0	0.0	0.0
GPR171	0.1	0.1	0.1	0.0	0.0	0.0	0.0	0.0	0.0
EFNA2	0.1	0.1	0.0	0.0	0.0	0.0	0.0	0.0	0.0
TCN1	0.2	0.0	0.0	0.0	0.0	0.0	0.1	0.0	0.0
TSPAN19	0.0	0.0	0.2	0.0	0.0	0.0	0.1	0.0	0.1
SMCO3	0.1	0.1	0.0	0.0	0.0	0.0	0.1	0.0	0.0
MMP10	0.1	0.0	0.0	0.0	0.0	0.0	0.1	0.0	0.0
LOC101929578	0.1	0.0	0.1	0.0	0.0	0.0	0.0	0.0	0.0
LOC100505710	0.1	0.0	0.1	0.0	0.0	0.0	0.0	0.0	0.1
FMO5	0.1	0.0	0.0	0.0	0.0	0.0	0.0	0.0	0.0
IPCEF1	0.1	0.0	0.1	0.0	0.0	0.0	0.0	0.0	0.0

C15orf65	0.0	0.1	0.1	0.0	0.0	0.0	0.1	0.0	0.0
SLC5A5	0.1	0.1	0.0	0.0	0.0	0.0	0.0	0.0	0.0
THPO	0.1	0.0	0.1	0.0	0.0	0.0	0.0	0.0	0.1
LOC101928913	0.1	0.0	0.0	0.0	0.0	0.0	0.1	0.0	0.0
RAD51AP2	0.1	0.0	0.0	0.0	0.0	0.0	0.0	0.0	0.0
LOC100996404	0.1	0.1	0.0	0.0	0.0	0.0	0.0	0.0	0.0
CCDC13-AS1	0.1	0.0	0.1	0.0	0.0	0.0	0.0	0.0	0.0
GBP4	0.1	0.0	0.1	0.0	0.0	0.0	0.0	0.0	0.0
LOC101927552	0.1	0.1	0.0	0.0	0.0	0.0	0.0	0.0	0.0
LOC101928146	0.1	0.0	0.1	0.0	0.0	0.0	0.0	0.0	0.0
TMEM119	0.1	0.0	0.0	0.0	0.0	0.0	0.0	0.0	0.0
LOC101928767	0.1	0.1	0.0	0.0	0.0	0.0	0.0	0.0	0.0
SIX6	0.1	0.0	0.1	0.0	0.0	0.0	0.0	0.0	0.0
MGC42157	0.0	0.0	0.0	0.0	0.0	0.0	0.1	0.0	0.0
MYBPHL	0.1	0.0	0.1	0.0	0.0	0.0	0.0	0.0	0.1
LOC101927223	0.0	0.0	0.0	0.0	0.0	0.0	0.2	0.0	0.0
CAPS2	0.1	0.0	0.0	0.0	0.0	0.0	0.0	0.0	0.0
LOC101929512	0.0	0.0	0.0	0.0	0.0	0.0	0.0	0.0	0.0
RNF219-AS1	0.1	0.0	0.0	0.0	0.0	0.0	0.0	0.0	0.1
LOC101927813	0.1	0.0	0.0	0.0	0.0	0.0	0.0	0.0	0.0
FRG2	0.0	0.0	0.0	0.0	0.0	0.0	0.1	0.0	0.0
FSCN3	0.1	0.1	0.0	0.0	0.0	0.0	0.0	0.0	0.0
ISM2	0.1	0.0	0.0	0.0	0.0	0.0	0.0	0.0	0.1
ENTPD2	0.1	0.0	0.0	0.0	0.0	0.0	0.0	0.0	0.0
EPB41L4A	0.1	0.0	0.0	0.0	0.0	0.0	0.1	0.0	0.0
TBC1D26	0.0	0.0	0.0	0.0	0.0	0.0	0.1	0.0	0.0
FLJ26850	0.1	0.0	0.0	0.0	0.0	0.0	0.1	0.0	0.0
CCDC36	0.1	0.0	0.0	0.0	0.0	0.0	0.1	0.0	0.0
TRIM43B	0.1	0.0	0.0	0.0	0.0	0.0	0.1	0.0	0.0
WDR63	0.1	0.0	0.0	0.0	0.0	0.0	0.0	0.0	0.0
C1QTNF9B	0.0	0.0	0.0	0.0	0.0	0.0	0.0	0.0	0.1
SIGLEC10	0.1	0.0	0.0	0.0	0.0	0.0	0.0	0.0	0.0
SLCO1A2	0.1	0.0	0.0	0.0	0.0	0.0	0.0	0.0	0.0
RAD21L1	0.1	0.0	0.1	0.0	0.0	0.0	0.0	0.0	0.0
GALNT8	0.1	0.0	0.0	0.0	0.0	0.0	0.0	0.0	0.0
MAPT-IT1	0.1	0.0	0.0	0.0	0.0	0.0	0.0	0.0	0.0
STAT4	0.1	0.0	0.0	0.0	0.0	0.0	0.0	0.0	0.0
CDH2	0.1	0.0	0.0	0.0	0.0	0.0	0.0	0.0	0.0
FLJ41278	0.0	0.0	0.0	0.0	0.0	0.0	0.0	0.0	0.1
SLCO3A1	0.0	0.0	0.0	0.0	0.0	0.0	0.0	0.0	0.0
C12orf50	0.0	0.0	0.0	0.0	0.0	0.0	0.0	0.0	0.1
LOC101927870	0.0	0.0	0.0	0.0	0.0	0.0	0.0	0.0	0.0
FBXW12	0.0	0.0	0.0	0.0	0.0	0.0	0.0	0.0	0.0
KLB	0.1	0.0	0.0	0.0	0.0	0.0	0.0	0.0	0.0
LOC101926998	0.0	0.0	0.1	0.0	0.0	0.0	0.0	0.0	0.0
SYCP2	0.0	0.0	0.0	0.0	0.0	0.0	0.0	0.0	0.0
PAK6	0.1	0.0	0.0	0.0	0.0	0.0	0.0	0.0	0.0
RPS4Y1	0.0	0.0	0.0	0.0	0.0	0.0	0.0	0.0	0.0
SMC1B	0.1	0.0	0.0	0.0	0.0	0.0	0.0	0.0	0.0
ALPK2	0.0	0.0	0.0	0.0	0.0	0.0	0.0	0.0	0.0
LOC145837	0.0	0.0	0.0	0.0	0.0	0.0	0.0	0.0	0.0
MYL4	0.0	0.0	0.0	0.0	0.0	0.0	0.1	0.0	0.0
TGM6	0.0	0.0	0.0	0.0	0.0	0.0	0.1	0.0	0.0
FAM83C	0.0	0.0	0.0	0.0	0.0	0.0	0.0	0.0	0.0
F13A1	0.0	0.0	0.0	0.0	0.0	0.0	0.0	0.0	0.0

RAB44	0.0	0.0	0.0	0.0	0.0	0.0	0.0	0.0	0.0
EFCAB14-AS1	0.0	0.0	0.0	0.0	0.0	0.0	0.0	0.0	0.1
KCNB1	0.0	0.0	0.0	0.0	0.0	0.0	0.0	0.0	0.0
LRRD1	0.0	0.0	0.0	0.0	0.0	0.0	0.0	0.0	0.0
CCDC110	0.0	0.0	0.0	0.0	0.0	0.0	0.0	0.0	0.0
GFAP	0.0	0.0	0.0	0.0	0.0	0.0	0.0	0.0	0.0
LOC101929407	0.0	0.0	0.0	0.0	0.0	0.0	0.0	0.0	0.0
LOC101060183	0.0	0.0	0.0	0.0	0.0	0.0	0.0	0.0	0.0
FXYD6-FXYD2	0.0	0.0	0.0	0.0	0.0	0.0	0.0	0.0	0.0
TTLL8	0.0	0.0	0.0	0.0	0.0	0.0	0.0	0.0	0.0
LOC100506731	0.0	0.0	0.0	0.0	0.0	0.0	0.0	0.0	0.0
KIF4B	0.0	0.0	0.0	0.0	0.0	0.0	0.0	0.0	0.0
FLJ44006	0.0	0.0	0.0	0.0	0.0	0.0	0.0	0.0	0.0
KCNU1	0.0	0.0	0.0	0.0	0.0	0.0	0.0	0.0	0.0
SORCS2	0.0	0.0	0.0	0.0	0.0	0.0	0.0	0.0	0.0
SLIT3	0.0	0.0	0.0	0.0	0.0	0.0	0.0	0.0	0.0
CCDC168	0.0	0.0	0.0	0.0	0.0	0.0	0.0	0.0	0.0
SNORD103B	0.6	0.0	0.5	0.1	0.2	0.0	0.0	0.0	0.0
SNORD103A	0.6	0.0	0.5	0.1	0.2	0.0	0.0	0.0	0.0
LOC100652758	0.0	0.1	0.1	0.1	0.2	0.1	0.0	0.0	0.0
CECR5-AS1	0.2	0.1	0.0	0.0	0.2	0.1	0.0	0.0	0.0
RNU105B	0.2	0.0	0.1	0.1	0.0	0.1	0.0	0.0	0.0
TMEM37	0.2	0.0	0.0	0.1	0.1	0.0	0.0	0.0	0.0
LOC101927179	0.3	0.0	0.1	0.0	0.0	0.0	0.0	0.0	0.0
PRSS8	0.2	0.0	0.1	0.0	0.0	0.0	0.0	0.0	0.0
LOC101928109	0.1	0.0	0.1	0.0	0.1	0.0	0.0	0.0	0.0
RBMXL2	0.1	0.0	0.1	0.0	0.1	0.0	0.0	0.0	0.0
LOC101929704	0.1	0.0	0.1	0.0	0.0	0.0	0.0	0.0	0.0
SLC6A4	0.0	0.0	0.0	0.0	0.0	0.0	0.0	0.0	0.0
MASP1	0.1	0.0	0.0	0.0	0.0	0.0	0.0	0.0	0.0
MEIOB	0.0	0.0	0.0	0.0	0.0	0.0	0.0	0.0	0.0
LOC100129434	0.1	0.0	0.0	0.0	0.0	0.0	0.0	0.0	0.0
EIF4A1	5.1	0.6	0.9	0.0	0.0	1.5	0.0	0.0	0.0
TRNAG18	3.9	0.0	1.9	0.0	1.5	0.4	0.0	0.0	0.0
LBP	2.5	1.0	1.2	0.0	0.0	0.0	0.0	0.0	0.0
C10orf91	1.4	0.5	1.4	0.0	0.2	0.2	0.0	0.0	0.0
BCL2L2-PABPN1	1.0	0.3	0.8	0.1	0.0	1.0	0.0	0.0	0.0
MALL	1.1	0.5	0.5	0.0	0.2	0.0	0.0	0.0	0.0
TMSB15B	1.1	0.0	0.3	0.0	0.0	0.2	0.0	0.0	0.0
C1orf54	0.8	0.1	0.1	0.0	0.0	0.4	0.0	0.0	0.0
LOC101927773	0.7	0.3	0.3	0.1	0.2	0.0	0.0	0.0	0.0
MOB3B	0.9	0.1	0.2	0.0	0.0	0.1	0.0	0.0	0.0
LINC00239	0.5	0.3	0.2	0.0	0.0	0.2	0.0	0.0	0.0
WFDC10B	0.5	0.1	0.1	0.0	0.0	0.2	0.0	0.0	0.0
SPINK4	0.5	0.0	0.0	0.1	0.0	0.3	0.0	0.0	0.0
CLDN2	0.3	0.1	0.3	0.0	0.1	0.0	0.0	0.0	0.0
LOC101929756	0.4	0.2	0.2	0.0	0.1	0.1	0.0	0.0	0.0
PCSK1N	0.2	0.1	0.2	0.1	0.1	0.0	0.0	0.0	0.0
FBXO48	0.3	0.1	0.2	0.0	0.1	0.1	0.0	0.0	0.0
LOC101929632	0.1	0.1	0.0	0.0	0.0	0.2	0.0	0.0	0.0
LOC101929639	0.3	0.1	0.2	0.0	0.0	0.0	0.0	0.0	0.0
LOC101927203	0.0	0.1	0.3	0.1	0.0	0.1	0.0	0.0	0.0
CLDN3	0.2	0.0	0.1	0.0	0.2	0.0	0.0	0.0	0.0
LOC101927679	0.4	0.1	0.0	0.0	0.0	0.0	0.0	0.0	0.0
RAET1E-AS1	0.2	0.0	0.0	0.1	0.2	0.0	0.0	0.0	0.0

C10orf67	0.1	0.1	0.0	0.0	0.0	0.1	0.0	0.0	0.0
NPL	0.2	0.0	0.2	0.0	0.0	0.0	0.0	0.0	0.0
TTC9B	0.3	0.1	0.1	0.0	0.0	0.0	0.0	0.0	0.0
KCNK4	0.2	0.0	0.1	0.0	0.0	0.0	0.0	0.0	0.0
RBM11	0.1	0.2	0.1	0.0	0.0	0.1	0.0	0.0	0.0
ZMYND10	0.2	0.1	0.2	0.0	0.0	0.0	0.0	0.0	0.0
LGALS4	0.2	0.0	0.1	0.0	0.0	0.0	0.0	0.0	0.0
PKNOX2	0.2	0.1	0.1	0.0	0.0	0.0	0.0	0.0	0.0
C19orf45	0.2	0.0	0.1	0.0	0.0	0.0	0.0	0.0	0.0
PTGS1	0.1	0.1	0.1	0.0	0.0	0.0	0.0	0.0	0.0
C20orf201	0.1	0.1	0.1	0.0	0.0	0.0	0.0	0.0	0.0
UTS2R	0.2	0.0	0.1	0.0	0.0	0.0	0.0	0.0	0.0
COX7A1	0.2	0.0	0.1	0.0	0.0	0.0	0.0	0.0	0.0
TLX2	0.2	0.0	0.1	0.0	0.0	0.0	0.0	0.0	0.0
SYT9	0.1	0.1	0.0	0.0	0.0	0.1	0.0	0.0	0.0
MTL5	0.2	0.0	0.1	0.0	0.0	0.0	0.0	0.0	0.0
C15orf27	0.2	0.0	0.1	0.0	0.0	0.0	0.0	0.0	0.0
LOC101060569	0.1	0.0	0.0	0.0	0.0	0.0	0.0	0.0	0.0
AK8	0.1	0.0	0.1	0.0	0.0	0.1	0.0	0.0	0.0
ZNF285	0.1	0.1	0.0	0.0	0.0	0.0	0.0	0.0	0.0
SIRPG	0.1	0.1	0.1	0.0	0.0	0.0	0.0	0.0	0.0
RENBP	0.1	0.1	0.1	0.0	0.0	0.0	0.0	0.0	0.0
OR2D2	0.0	0.0	0.1	0.0	0.1	0.0	0.0	0.0	0.0
CD226	0.1	0.0	0.1	0.0	0.0	0.0	0.0	0.0	0.0
ZNF385D	0.1	0.0	0.0	0.0	0.0	0.0	0.0	0.0	0.0
AWAT1	0.1	0.1	0.0	0.1	0.0	0.0	0.0	0.0	0.0
CAMK2B	0.0	0.1	0.1	0.0	0.0	0.0	0.0	0.0	0.0
CDKL2	0.1	0.0	0.1	0.0	0.0	0.0	0.0	0.0	0.0
GSTM1	0.1	0.1	0.0	0.0	0.1	0.0	0.0	0.0	0.0
ISM1-AS1	0.0	0.0	0.0	0.0	0.0	0.0	0.0	0.0	0.0
CACNA2D3	0.1	0.0	0.1	0.0	0.0	0.0	0.0	0.0	0.0
PCDHB6	0.1	0.0	0.0	0.0	0.0	0.0	0.0	0.0	0.0
FAM86KP	0.0	0.0	0.1	0.0	0.0	0.0	0.0	0.0	0.0
ACTC1	0.1	0.0	0.0	0.0	0.0	0.1	0.0	0.0	0.0
LOC644249	0.1	0.0	0.1	0.0	0.0	0.0	0.0	0.0	0.0
SAA2-SAA4	0.1	0.0	0.1	0.0	0.0	0.0	0.0	0.0	0.0
PNLDC1	0.1	0.1	0.0	0.0	0.0	0.0	0.0	0.0	0.0
PRSS16	0.1	0.0	0.0	0.0	0.0	0.0	0.0	0.0	0.0
ZC3H12D	0.1	0.0	0.0	0.0	0.0	0.0	0.0	0.0	0.0
IGHE	0.0	0.0	0.1	0.0	0.1	0.0	0.0	0.0	0.0
LOC101927894	0.0	0.0	0.1	0.0	0.0	0.0	0.0	0.0	0.0
LOC101927333	0.0	0.0	0.0	0.0	0.0	0.0	0.0	0.0	0.0
IZUMO1	0.0	0.0	0.0	0.0	0.0	0.0	0.0	0.0	0.0
LOC101929552	0.0	0.0	0.0	0.0	0.0	0.0	0.0	0.0	0.0
LOC100129307	0.1	0.0	0.0	0.0	0.0	0.0	0.0	0.0	0.0
LOC101927273	0.1	0.0	0.0	0.0	0.0	0.0	0.0	0.0	0.0
ITGA2B	0.0	0.0	0.1	0.0	0.0	0.0	0.0	0.0	0.0
IGFBP2	0.1	0.0	0.0	0.0	0.0	0.0	0.0	0.0	0.0
ALDH1L1	0.1	0.0	0.1	0.0	0.0	0.0	0.0	0.0	0.0
CRTAM	0.0	0.0	0.0	0.0	0.0	0.0	0.0	0.0	0.0
ARG1	0.1	0.0	0.0	0.0	0.0	0.0	0.0	0.0	0.0
C8orf34	0.1	0.0	0.0	0.0	0.0	0.0	0.0	0.0	0.0
CEACAM3	0.1	0.0	0.0	0.0	0.0	0.0	0.0	0.0	0.0
DKFZp434L192	0.0	0.0	0.0	0.0	0.0	0.0	0.0	0.0	0.0
RHCE	0.1	0.0	0.0	0.0	0.0	0.0	0.0	0.0	0.0

SLC47A2	0.1	0.0	0.0	0.0	0.0	0.0	0.0	0.0	0.0
LINC00838	0.1	0.0	0.0	0.0	0.0	0.0	0.0	0.0	0.0
CERKL	0.0	0.0	0.0	0.0	0.0	0.0	0.0	0.0	0.0
SULT1A1	0.0	0.0	0.0	0.0	0.0	0.0	0.0	0.0	0.0
FPGT-TNNI3K	0.0	0.0	0.0	0.0	0.0	0.0	0.0	0.0	0.0
LRFN5	0.0	0.0	0.0	0.0	0.0	0.0	0.0	0.0	0.0
KIRREL2	0.0	0.0	0.0	0.0	0.0	0.0	0.0	0.0	0.0
LOC101929423	0.0	0.0	0.1	0.0	0.0	0.0	0.0	0.0	0.0
LOC101929457	0.0	0.0	0.0	0.0	0.0	0.0	0.0	0.0	0.0
STAR	0.0	0.0	0.0	0.0	0.0	0.0	0.0	0.0	0.0
LOC101927753	0.0	0.0	0.0	0.0	0.0	0.0	0.0	0.0	0.0
LOC100129620	0.0	0.0	0.0	0.0	0.0	0.0	0.0	0.0	0.0
KNDC1	0.0	0.0	0.0	0.0	0.0	0.0	0.0	0.0	0.0
LOC100507351	0.0	0.0	0.0	0.0	0.0	0.0	0.0	0.0	0.0
PRUNE2	0.0	0.0	0.0	0.0	0.0	0.0	0.0	0.0	0.0
RPRM	0.0	0.1	0.3	0.2	0.1	0.2	0.0	0.0	0.0
LOC441956	0.1	0.0	0.1	0.0	0.0	0.0	0.0	0.0	0.0
DGAT2L6	0.0	0.0	0.1	0.0	0.0	0.1	0.0	0.0	0.0
LOC101928416	0.1	0.1	0.2	0.0	0.1	0.1	0.0	0.0	0.0
KCNE1L	0.2	0.1	0.1	0.1	0.0	0.0	0.0	0.0	0.0
CX3CL1	0.1	0.0	0.1	0.0	0.1	0.0	0.0	0.0	0.0
SLC25A2	0.1	0.0	0.1	0.0	0.0	0.0	0.0	0.0	0.0
LOC101928931	0.1	0.0	0.1	0.0	0.0	0.1	0.0	0.0	0.0
SLC22A18AS	0.1	0.0	0.0	0.0	0.0	0.0	0.0	0.0	0.0
TCEB3C	0.0	0.1	0.0	0.0	0.1	0.0	0.0	0.0	0.0
NKAIN3	0.1	0.0	0.0	0.0	0.0	0.0	0.0	0.0	0.0
LOC100652869	0.0	0.0	0.0	0.0	0.0	0.0	0.0	0.0	0.0
GPR37L1	0.1	0.0	0.0	0.0	0.0	0.0	0.0	0.0	0.0
CD93	0.0	0.0	0.0	0.0	0.0	0.0	0.0	0.0	0.0
CASP16	0.0	0.0	0.0	0.0	0.0	0.0	0.0	0.0	0.0
SNORD48	0.0	0.0	0.0	0.3	0.6	0.0	0.5	0.0	1.3
LOC101928117	0.0	0.0	0.0	0.0	0.1	0.1	0.1	0.0	0.2
S100A5	0.0	0.0	0.0	0.0	0.0	0.0	0.3	0.0	0.1
FAM223A	0.0	0.0	0.0	0.0	0.1	0.0	0.3	0.0	0.0
LOC101927435	0.0	0.0	0.0	0.0	0.1	0.0	0.1	0.0	0.1
IGF2	0.0	0.0	0.0	0.0	0.0	0.1	0.3	0.0	0.0
LOC101928631	0.0	0.0	0.0	0.0	0.0	0.0	0.1	0.0	0.1
LINC00570	0.0	0.0	0.0	0.0	0.0	0.0	0.1	0.0	0.1
CLIC2	0.0	0.0	0.0	0.0	0.1	0.0	0.0	0.0	0.0
LOC101928363	0.0	0.0	0.0	0.0	0.0	0.0	0.2	0.0	0.1
LOC100506076	0.0	0.0	0.0	0.0	0.1	0.1	0.0	0.0	0.1
FLJ41309	0.0	0.0	0.0	0.0	0.0	0.0	0.0	0.0	0.0
CMTM2	0.0	0.0	0.0	0.0	0.0	0.0	0.0	0.0	0.1
DCAF12L1	0.0	0.0	0.0	0.0	0.0	0.0	0.0	0.0	0.1
ANKRD63	0.0	0.0	0.0	0.0	0.0	0.0	0.1	0.0	0.0
MIR155HG	0.0	0.0	0.0	0.0	0.0	0.0	0.1	0.0	0.1
TCTE1	0.0	0.0	0.0	0.0	0.0	0.0	0.1	0.0	0.0
UBTFL1	0.0	0.0	0.0	0.0	0.0	0.0	0.1	0.0	0.0
TUBA3E	0.0	0.0	0.0	0.0	0.0	0.0	0.0	0.0	0.1
DPEP1	0.0	0.0	0.0	0.0	0.0	0.0	0.0	0.0	0.0
LOC101928333	0.0	0.0	0.0	0.0	0.0	0.0	0.0	0.0	0.0
LOC727710	0.0	0.0	0.0	0.0	0.0	0.0	0.0	0.0	0.0
FOXC2	0.0	0.0	0.0	0.0	0.0	0.0	0.0	0.0	0.0
DPP4	0.0	0.0	0.0	0.0	0.0	0.0	0.0	0.0	0.0
H2AFB3	0.0	0.0	0.0	0.0	0.0	0.0	0.0	0.0	0.0

H2AFB2	0.0	0.0	0.0	0.0	0.0	0.0	0.0	0.0	0.0
WDR38	0.0	0.0	0.0	0.0	0.0	0.0	0.1	0.0	0.0
SORCS3	0.0	0.0	0.0	0.0	0.0	0.0	0.0	0.0	0.0
CCL15-CCL14	0.0	0.0	0.0	0.0	0.0	0.0	0.0	0.0	0.0
LOC101927641	0.0	0.0	0.0	0.0	0.0	0.0	0.0	0.0	0.0
LOC221122	0.0	0.0	0.0	0.0	0.0	0.0	0.1	0.0	0.0
ADRA2C	0.0	0.0	0.0	0.0	0.0	0.0	0.0	0.0	0.0
PNMA3	0.0	0.0	0.0	0.0	0.0	0.0	0.0	0.0	0.0
MUC16	0.0	0.0	0.0	0.0	0.0	0.0	0.0	0.0	0.0
TRNW	0.0	0.0	0.0	0.3	0.5	0.0	0.4	0.0	0.4
SNORD18A	0.0	0.0	0.0	0.0	0.5	0.4	0.4	0.0	0.0
LOC101929506	0.0	0.0	0.0	0.0	0.0	0.0	0.1	0.0	0.1
LINC00441	0.0	0.0	0.0	0.0	0.0	0.0	0.0	0.0	0.0
LOC101928058	0.0	0.0	0.0	0.0	0.0	0.0	0.0	0.0	0.0
GIMAP8	0.0	0.0	0.0	0.0	0.0	0.0	0.0	0.0	0.0
VAX1	0.0	0.0	0.0	0.0	0.0	0.0	0.0	0.0	0.0
TRNI	1.2	0.4	0.0	0.0	0.0	0.0	1.3	0.0	1.6
SNORA75	0.0	0.3	1.2	0.0	0.0	0.0	1.1	0.0	1.0
RNU6-8	1.7	0.0	0.4	0.0	0.0	0.0	0.4	0.0	0.6
SNORD11	0.7	0.0	0.0	0.0	0.0	0.0	0.7	0.0	1.2
TRNAG-CCC	0.6	0.4	0.0	0.0	0.0	0.0	0.8	0.0	0.8
SNORA56	0.0	1.0	0.4	0.0	0.0	0.0	0.0	0.2	0.2
RNU6-76P	0.4	0.0	0.0	0.0	0.0	0.0	1.0	0.0	0.4
ATP6V1G2	0.8	0.0	0.4	0.0	0.0	0.0	0.2	0.0	0.0
VTRNA2-1	0.6	0.0	0.4	0.0	0.0	0.0	0.5	0.0	0.0
DIO1	0.4	0.0	0.3	0.0	0.0	0.0	0.4	0.0	0.2
VTRNA1-1	0.0	0.4	0.3	0.0	0.0	0.0	0.5	0.0	0.0
LOC101928274	0.4	0.0	0.2	0.0	0.0	0.0	0.3	0.0	0.3
YTHDF3-AS1	0.4	0.0	0.1	0.0	0.0	0.0	0.2	0.0	0.2
LOC101928258	0.3	0.0	0.1	0.0	0.0	0.0	0.1	0.0	0.0
LOC101929480	0.5	0.0	0.1	0.0	0.0	0.0	0.1	0.0	0.1
LOC101926968	0.3	0.0	0.1	0.0	0.0	0.0	0.2	0.0	0.1
CPLX2	0.3	0.0	0.1	0.0	0.0	0.0	0.2	0.0	0.1
LOC101928854	0.4	0.0	0.0	0.0	0.0	0.0	0.2	0.0	0.2
LOC91450	0.3	0.0	0.1	0.0	0.0	0.0	0.1	0.0	0.1
LOC101928516	0.1	0.0	0.1	0.0	0.0	0.0	0.2	0.0	0.2
TSTD3	0.3	0.0	0.0	0.0	0.0	0.0	0.1	0.0	0.2
THRB-AS1	0.2	0.0	0.2	0.0	0.0	0.0	0.2	0.0	0.0
S100A14	0.4	0.0	0.0	0.0	0.0	0.0	0.0	0.0	0.1
DUSP15	0.2	0.0	0.0	0.0	0.0	0.0	0.2	0.0	0.0
HIST1H2AE	0.1	0.0	0.1	0.0	0.0	0.0	0.3	0.0	0.1
ZNF660	0.2	0.0	0.1	0.0	0.0	0.0	0.1	0.0	0.1
OR8G1	0.2	0.0	0.1	0.0	0.0	0.0	0.0	0.0	0.2
C20orf202	0.2	0.0	0.0	0.0	0.0	0.0	0.1	0.1	0.0
OR8B3	0.2	0.0	0.2	0.0	0.0	0.0	0.0	0.0	0.1
RNU6-45P	0.0	0.5	0.0	0.0	0.0	0.0	0.0	0.0	0.0
LOC101928106	0.2	0.0	0.0	0.0	0.0	0.0	0.1	0.0	0.2
LOC101928366	0.0	0.0	0.2	0.0	0.0	0.0	0.2	0.0	0.0
MAP1LC3C	0.2	0.0	0.0	0.0	0.0	0.0	0.1	0.0	0.1
C10orf11	0.2	0.1	0.0	0.0	0.0	0.0	0.0	0.0	0.0
LOC101929732	0.4	0.0	0.1	0.0	0.0	0.0	0.0	0.0	0.0
PGAM4	0.2	0.0	0.1	0.0	0.0	0.0	0.1	0.0	0.0
HIST1H4D	0.0	0.0	0.1	0.0	0.0	0.0	0.2	0.0	0.1
LOC101929691	0.2	0.0	0.1	0.0	0.0	0.0	0.1	0.0	0.0
LOC101929152	0.1	0.0	0.0	0.0	0.0	0.0	0.3	0.0	0.0

LOC101928030	0.0	0.0	0.2	0.0	0.0	0.0	0.2	0.0	0.0
SOX6	0.2	0.0	0.0	0.0	0.0	0.0	0.1	0.0	0.1
TRNAP18	0.2	0.0	0.0	0.0	0.0	0.0	0.0	0.0	0.0
TRNAP9	0.2	0.0	0.0	0.0	0.0	0.0	0.0	0.0	0.0
TRNAP2	0.2	0.0	0.0	0.0	0.0	0.0	0.0	0.0	0.0
TRNAP1	0.2	0.0	0.0	0.0	0.0	0.0	0.0	0.0	0.0
TRNAP6	0.2	0.0	0.0	0.0	0.0	0.0	0.0	0.0	0.0
TRNAP15	0.2	0.0	0.0	0.0	0.0	0.0	0.0	0.0	0.0
TRNAP13	0.2	0.0	0.0	0.0	0.0	0.0	0.0	0.0	0.0
TRNAP4	0.2	0.0	0.0	0.0	0.0	0.0	0.0	0.0	0.0
MAB21L1	0.1	0.0	0.1	0.0	0.0	0.0	0.1	0.0	0.0
METTL7A	0.1	0.0	0.1	0.0	0.0	0.0	0.1	0.0	0.0
TTC34	0.1	0.0	0.1	0.0	0.0	0.0	0.1	0.0	0.0
EGOT	0.2	0.0	0.0	0.0	0.0	0.0	0.0	0.0	0.0
SPP1	0.1	0.0	0.1	0.0	0.0	0.0	0.0	0.0	0.1
LOC101928734	0.1	0.0	0.0	0.0	0.0	0.0	0.2	0.0	0.0
RFPL4AL1	0.0	0.0	0.1	0.0	0.0	0.0	0.2	0.0	0.0
OR10A2	0.1	0.0	0.0	0.0	0.0	0.0	0.1	0.0	0.0
LOC340073	0.1	0.1	0.0	0.0	0.0	0.0	0.1	0.0	0.0
LOC101929069	0.2	0.0	0.0	0.0	0.0	0.0	0.0	0.0	0.1
LOC101927956	0.3	0.0	0.0	0.0	0.0	0.0	0.0	0.0	0.0
ROPN1B	0.1	0.0	0.1	0.0	0.0	0.0	0.1	0.0	0.0
LOC101929401	0.2	0.0	0.1	0.0	0.0	0.0	0.0	0.0	0.0
LOC100287944	0.0	0.1	0.0	0.0	0.0	0.0	0.0	0.0	0.2
DKK2	0.1	0.0	0.1	0.0	0.0	0.0	0.0	0.0	0.1
LOC101927481	0.1	0.0	0.1	0.0	0.0	0.0	0.1	0.0	0.0
HCRTR1	0.0	0.0	0.0	0.0	0.0	0.0	0.1	0.0	0.1
THY1	0.0	0.0	0.1	0.0	0.0	0.0	0.1	0.0	0.1
LCN12	0.1	0.0	0.0	0.0	0.0	0.0	0.1	0.0	0.1
S1PR4	0.1	0.0	0.0	0.0	0.0	0.0	0.1	0.0	0.1
FCER1G	0.0	0.1	0.0	0.0	0.0	0.0	0.0	0.0	0.1
TMOD1	0.1	0.0	0.1	0.0	0.0	0.0	0.1	0.0	0.0
GP5	0.2	0.0	0.1	0.0	0.0	0.0	0.0	0.0	0.0
LOC101927128	0.0	0.0	0.0	0.0	0.0	0.0	0.0	0.0	0.1
MYOZ3	0.1	0.0	0.1	0.0	0.0	0.0	0.1	0.0	0.0
OLFML1	0.1	0.0	0.0	0.0	0.0	0.0	0.0	0.0	0.0
LOC101927427	0.0	0.1	0.1	0.0	0.0	0.0	0.0	0.0	0.1
OR8B2	0.1	0.0	0.0	0.0	0.0	0.0	0.1	0.0	0.0
FOXR1	0.0	0.1	0.0	0.0	0.0	0.0	0.1	0.0	0.0
LINC00571	0.1	0.1	0.0	0.0	0.0	0.0	0.1	0.0	0.1
SFTA3	0.0	0.0	0.0	0.0	0.0	0.0	0.1	0.0	0.1
C1QTNF2	0.0	0.0	0.1	0.0	0.0	0.0	0.1	0.0	0.0
VNN1	0.0	0.1	0.1	0.0	0.0	0.0	0.0	0.0	0.0
CYP7B1	0.1	0.0	0.0	0.0	0.0	0.0	0.1	0.0	0.0
WNT16	0.1	0.0	0.0	0.0	0.0	0.0	0.0	0.0	0.0
LOC101927932	0.0	0.0	0.1	0.0	0.0	0.0	0.0	0.0	0.0
CXorf28	0.1	0.0	0.0	0.0	0.0	0.0	0.0	0.0	0.1
LOC100996583	0.0	0.0	0.0	0.0	0.0	0.0	0.0	0.0	0.0
IRF5	0.1	0.0	0.0	0.0	0.0	0.0	0.1	0.0	0.0
PLG	0.1	0.0	0.0	0.0	0.0	0.0	0.0	0.0	0.0
LOC100996342	0.1	0.0	0.0	0.0	0.0	0.0	0.1	0.0	0.1
DLX3	0.0	0.0	0.0	0.0	0.0	0.0	0.1	0.0	0.1
TARM1	0.0	0.0	0.0	0.0	0.0	0.0	0.0	0.0	0.0
TAS2R60	0.0	0.0	0.0	0.0	0.0	0.0	0.1	0.0	0.1
LOC101928168	0.1	0.0	0.0	0.0	0.0	0.0	0.1	0.0	0.0

RSPH4A	0.1	0.0	0.0	0.0	0.0	0.0	0.0	0.0	0.0
OR2A4	0.1	0.0	0.0	0.0	0.0	0.0	0.0	0.0	0.0
TBC1D27	0.1	0.0	0.1	0.0	0.0	0.0	0.0	0.0	0.0
OR2J3	0.1	0.0	0.0	0.0	0.0	0.0	0.0	0.0	0.0
LOC101927233	0.1	0.0	0.0	0.0	0.0	0.0	0.1	0.0	0.0
LTF	0.0	0.0	0.0	0.0	0.0	0.0	0.1	0.0	0.1
OR9A2	0.1	0.0	0.0	0.0	0.0	0.0	0.1	0.0	0.1
GLIPR1L1	0.1	0.0	0.0	0.0	0.0	0.0	0.0	0.0	0.0
FLJ20712	0.1	0.0	0.0	0.0	0.0	0.0	0.1	0.0	0.0
GPR34	0.1	0.0	0.0	0.0	0.0	0.0	0.1	0.0	0.0
SLC52A1	0.0	0.0	0.1	0.0	0.0	0.0	0.1	0.0	0.0
OR13A1	0.0	0.0	0.1	0.0	0.0	0.0	0.0	0.0	0.0
LOC285627	0.1	0.0	0.1	0.0	0.0	0.0	0.0	0.0	0.0
FLJ25715	0.1	0.0	0.0	0.0	0.0	0.0	0.0	0.0	0.0
LOC338694	0.1	0.0	0.0	0.0	0.0	0.0	0.0	0.0	0.1
CELF5	0.1	0.0	0.0	0.0	0.0	0.0	0.0	0.0	0.0
LINC00511	0.0	0.0	0.0	0.0	0.0	0.0	0.0	0.0	0.1
KCNQ1	0.1	0.0	0.0	0.0	0.0	0.0	0.0	0.0	0.0
HSH2D	0.0	0.0	0.0	0.0	0.0	0.0	0.0	0.0	0.1
KCNS2	0.1	0.0	0.0	0.0	0.0	0.0	0.0	0.0	0.0
ERP27	0.1	0.0	0.0	0.0	0.0	0.0	0.0	0.0	0.1
NCF1	0.0	0.0	0.0	0.0	0.0	0.0	0.0	0.0	0.0
CRYBA1	0.1	0.1	0.0	0.0	0.0	0.0	0.0	0.0	0.0
IKZF1	0.0	0.1	0.0	0.0	0.0	0.0	0.0	0.0	0.0
TACR1	0.1	0.1	0.0	0.0	0.0	0.0	0.0	0.0	0.0
CGREF1	0.0	0.0	0.0	0.0	0.0	0.0	0.1	0.0	0.0
ECT2L	0.1	0.0	0.0	0.0	0.0	0.0	0.0	0.0	0.0
CCDC38	0.0	0.0	0.0	0.0	0.0	0.0	0.0	0.0	0.0
LOC101928220	0.1	0.1	0.0	0.0	0.0	0.0	0.0	0.0	0.0
IGHA2	0.0	0.0	0.1	0.0	0.0	0.0	0.0	0.0	0.0
FAM43B	0.0	0.0	0.0	0.0	0.0	0.0	0.0	0.0	0.0
COL4A3	0.1	0.0	0.0	0.0	0.0	0.0	0.0	0.0	0.0
TSHR	0.1	0.0	0.0	0.0	0.0	0.0	0.0	0.0	0.0
GDF5	0.1	0.0	0.0	0.0	0.0	0.0	0.0	0.0	0.0
EFCAB8	0.1	0.0	0.0	0.0	0.0	0.0	0.0	0.0	0.0
ZSCAN10	0.0	0.0	0.0	0.0	0.0	0.0	0.0	0.0	0.0
LOC285000	0.1	0.0	0.0	0.0	0.0	0.0	0.0	0.0	0.0
USP27X-AS1	0.0	0.0	0.0	0.0	0.0	0.0	0.1	0.0	0.0
DGCR10	0.0	0.1	0.0	0.0	0.0	0.0	0.0	0.0	0.0
ZSWIM5	0.0	0.0	0.0	0.0	0.0	0.0	0.0	0.0	0.0
FER1L6-AS1	0.1	0.0	0.0	0.0	0.0	0.0	0.0	0.0	0.0
DDI1	0.1	0.0	0.0	0.0	0.0	0.0	0.0	0.0	0.0
MMP28	0.0	0.1	0.0	0.0	0.0	0.0	0.0	0.0	0.0
LOC101928658	0.1	0.0	0.0	0.0	0.0	0.0	0.0	0.0	0.0
KLF17	0.0	0.0	0.0	0.0	0.0	0.0	0.0	0.0	0.0
LOC101928892	0.0	0.0	0.0	0.0	0.0	0.0	0.0	0.0	0.0
XAGE1E	0.0	0.0	0.1	0.0	0.0	0.0	0.0	0.0	0.0
XAGE1C	0.0	0.0	0.1	0.0	0.0	0.0	0.0	0.0	0.0
XAGE1A	0.0	0.0	0.1	0.0	0.0	0.0	0.0	0.0	0.0
XAGE1B	0.0	0.0	0.1	0.0	0.0	0.0	0.0	0.0	0.0
LOC101928956	0.0	0.0	0.0	0.0	0.0	0.0	0.1	0.0	0.0
FAM198B	0.1	0.0	0.0	0.0	0.0	0.0	0.0	0.0	0.0
GCM1	0.0	0.0	0.0	0.0	0.0	0.0	0.0	0.0	0.0
CSF2RB	0.0	0.0	0.0	0.0	0.0	0.0	0.0	0.0	0.0
GCSAML-AS1	0.0	0.0	0.0	0.0	0.0	0.0	0.0	0.0	0.0

SLFN12L		0.1	0.0	0.0	0.0	0.0	0.0	0.0	0.0	0.0
MYCBPAP		0.0	0.0	0.0	0.0	0.0	0.0	0.0	0.0	0.0
DLL1		0.0	0.0	0.0	0.0	0.0	0.0	0.0	0.0	0.0
STPG2		0.0	0.0	0.0	0.0	0.0	0.0	0.0	0.0	0.0
	44630.0	0.0	0.0	0.0	0.0	0.0	0.0	0.0	0.0	0.0
CD163		0.0	0.0	0.0	0.0	0.0	0.0	0.0	0.0	0.0
LOC101929692		0.0	0.0	0.0	0.0	0.0	0.0	0.1	0.0	0.0
LOC101929192		0.0	0.0	0.0	0.0	0.0	0.0	0.1	0.0	0.0
PDIA2		0.0	0.0	0.0	0.0	0.0	0.0	0.0	0.0	0.0
DMP1		0.0	0.0	0.0	0.0	0.0	0.0	0.0	0.0	0.0
LGR6		0.0	0.0	0.0	0.0	0.0	0.0	0.0	0.0	0.0
CBS		0.0	0.0	0.0	0.0	0.0	0.0	0.0	0.0	0.0
GFRA3		0.0	0.0	0.0	0.0	0.0	0.0	0.0	0.0	0.0
LOC101927635		0.0	0.0	0.0	0.0	0.0	0.0	0.0	0.0	0.0
DNM3OS		0.0	0.0	0.0	0.0	0.0	0.0	0.0	0.0	0.0
RASAL3		0.0	0.0	0.0	0.0	0.0	0.0	0.0	0.0	0.0
RGS22		0.0	0.0	0.0	0.0	0.0	0.0	0.0	0.0	0.0
SLC44A4		0.0	0.0	0.0	0.0	0.0	0.0	0.0	0.0	0.0
TTBK1		0.0	0.0	0.0	0.0	0.0	0.0	0.0	0.0	0.0
KCNA3		0.0	0.0	0.0	0.0	0.0	0.0	0.0	0.0	0.0
HPD		0.0	0.0	0.0	0.0	0.0	0.0	0.0	0.0	0.0
ACOXL		0.0	0.0	0.0	0.0	0.0	0.0	0.0	0.0	0.0
LOC100507103		0.0	0.0	0.0	0.0	0.0	0.0	0.0	0.0	0.0
LOC101926981		0.0	0.0	0.0	0.0	0.0	0.0	0.0	0.0	0.0
LOC101928070		0.0	0.0	0.0	0.0	0.0	0.0	0.0	0.0	0.0
LGALS9C		0.0	0.0	0.0	0.0	0.0	0.0	0.0	0.0	0.0
B4GALNT4		0.0	0.0	0.0	0.0	0.0	0.0	0.0	0.0	0.0
ADAMTS10		0.0	0.0	0.0	0.0	0.0	0.0	0.0	0.0	0.0
COCH		0.0	0.0	0.0	0.0	0.0	0.0	0.0	0.0	0.0
ACSBG2		0.0	0.0	0.0	0.0	0.0	0.0	0.0	0.0	0.0
BNC1		0.0	0.0	0.0	0.0	0.0	0.0	0.0	0.0	0.0
LOC101926924		0.0	0.0	0.0	0.0	0.0	0.0	0.0	0.0	0.0
SLC25A18		0.0	0.0	0.0	0.0	0.0	0.0	0.0	0.0	0.0
TNN		0.0	0.0	0.0	0.0	0.0	0.0	0.0	0.0	0.0
PLEKHG4B		0.0	0.0	0.0	0.0	0.0	0.0	0.0	0.0	0.0
CNTN4		0.0	0.0	0.0	0.0	0.0	0.0	0.0	0.0	0.0
LILRB2		0.0	0.0	0.0	0.0	0.0	0.0	0.0	0.0	0.0
ADARB2		0.0	0.0	0.0	0.0	0.0	0.0	0.0	0.0	0.0
LOC101927690		0.0	0.0	0.0	0.0	0.0	0.0	0.0	0.0	0.0
JAKMIP2-AS1		0.0	0.0	0.0	0.0	0.0	0.0	0.0	0.0	0.0
CLSTN2		0.0	0.0	0.0	0.0	0.0	0.0	0.0	0.0	0.0
KCNH4		0.0	0.0	0.0	0.0	0.0	0.0	0.0	0.0	0.0
CNDP1		0.0	0.0	0.0	0.0	0.0	0.0	0.0	0.0	0.0
LOC101929481		0.0	0.0	0.0	0.0	0.0	0.0	0.0	0.0	0.0
FLJ16779		0.0	0.0	0.0	0.0	0.0	0.0	0.0	0.0	0.0
FAM186A		0.0	0.0	0.0	0.0	0.0	0.0	0.0	0.0	0.0
MYO18B		0.0	0.0	0.0	0.0	0.0	0.0	0.0	0.0	0.0
USP17L3		0.0	0.0	0.0	0.0	0.0	0.0	0.0	0.0	0.0
HMX3		0.0	0.0	0.0	0.0	0.0	0.0	0.0	0.0	0.0
SNORD5		0.5	0.0	0.7	0.0	0.0	0.0	0.8	0.0	0.4
SNORD70		0.5	0.0	0.3	0.0	0.0	0.0	0.7	0.0	0.3
LOC101929521		1.1	0.0	0.0	0.0	0.0	0.0	0.4	0.0	0.0
SNORD14B		0.8	0.0	0.3	0.0	0.0	0.0	0.3	0.0	0.0
SCARNA8		0.0	0.0	0.2	0.0	0.0	0.0	0.2	0.0	0.6
LOC100287852		0.4	0.0	0.1	0.0	0.0	0.0	0.1	0.0	0.4

LOC101926961	0.5	0.3	0.0	0.0	0.0	0.0	0.2	0.0	0.0
LOC101928997	0.3	0.0	0.1	0.0	0.0	0.0	0.1	0.0	0.2
LOC101928430	0.0	0.2	0.0	0.0	0.0	0.0	0.3	0.0	0.2
LOC101927977	0.0	0.0	0.0	0.0	0.0	0.0	0.4	0.0	0.2
TFF1	0.4	0.1	0.0	0.0	0.0	0.0	0.1	0.0	0.0
RNU6-19P	0.0	0.0	0.0	0.0	0.0	0.0	0.6	0.0	0.0
LOC100506908	0.3	0.0	0.2	0.0	0.0	0.0	0.0	0.0	0.1
LOC100505984	0.4	0.0	0.1	0.0	0.0	0.0	0.1	0.0	0.1
TRNAV7	0.1	0.0	0.1	0.0	0.0	0.0	0.2	0.0	0.2
TRNAV1	0.1	0.0	0.1	0.0	0.0	0.0	0.2	0.0	0.2
TRNAV3	0.1	0.0	0.1	0.0	0.0	0.0	0.2	0.0	0.2
TRNAV5	0.1	0.0	0.1	0.0	0.0	0.0	0.2	0.0	0.2
TRNAV24	0.1	0.0	0.1	0.0	0.0	0.0	0.2	0.0	0.2
LOC101929587	0.2	0.1	0.0	0.0	0.0	0.0	0.2	0.0	0.0
LOC101928149	0.0	0.0	0.0	0.0	0.0	0.0	0.3	0.0	0.2
LOC101928860	0.2	0.0	0.0	0.0	0.0	0.0	0.1	0.0	0.1
ST20-MTHFS	0.3	0.0	0.1	0.0	0.0	0.0	0.0	0.0	0.0
LOC100128108	0.2	0.0	0.1	0.0	0.0	0.0	0.0	0.0	0.1
LOC101927665	0.0	0.1	0.1	0.0	0.0	0.0	0.0	0.0	0.2
HIST1H1A	0.1	0.0	0.1	0.0	0.0	0.0	0.1	0.0	0.1
LOC101927630	0.3	0.0	0.0	0.0	0.0	0.0	0.1	0.0	0.0
TRNAV21	0.1	0.0	0.0	0.0	0.0	0.0	0.1	0.0	0.2
TRNAV6	0.1	0.0	0.0	0.0	0.0	0.0	0.1	0.0	0.2
TRNAV4	0.1	0.0	0.0	0.0	0.0	0.0	0.1	0.0	0.2
TRNAV9	0.1	0.0	0.0	0.0	0.0	0.0	0.1	0.0	0.2
TRNAV2	0.1	0.0	0.0	0.0	0.0	0.0	0.1	0.0	0.2
TRNAV29	0.1	0.0	0.0	0.0	0.0	0.0	0.1	0.0	0.2
CCNA1	0.2	0.1	0.0	0.0	0.0	0.0	0.1	0.1	0.0
HIST1H4E	0.0	0.0	0.1	0.0	0.0	0.0	0.2	0.0	0.1
TMEM56-RWDD3	0.1	0.0	0.1	0.0	0.0	0.0	0.2	0.0	0.0
C3orf80	0.1	0.0	0.0	0.0	0.0	0.0	0.2	0.0	0.1
IHH	0.2	0.0	0.0	0.0	0.0	0.0	0.1	0.0	0.0
OR10A4	0.3	0.0	0.0	0.0	0.0	0.0	0.1	0.0	0.0
LINC00628	0.2	0.0	0.0	0.0	0.0	0.0	0.0	0.0	0.1
LOC101927259	0.0	0.0	0.2	0.0	0.0	0.0	0.1	0.0	0.1
LOC101929425	0.2	0.0	0.0	0.0	0.0	0.0	0.1	0.0	0.1
PDC	0.1	0.0	0.1	0.0	0.0	0.0	0.1	0.0	0.1
LOC101929621	0.1	0.0	0.0	0.0	0.0	0.0	0.1	0.0	0.1
RXFP3	0.1	0.0	0.0	0.0	0.0	0.0	0.2	0.0	0.1
LINC00582	0.1	0.0	0.1	0.0	0.0	0.0	0.1	0.0	0.0
BLOC1S5	0.0	0.0	0.1	0.0	0.0	0.0	0.0	0.2	0.0
LOC101928689	0.1	0.0	0.1	0.0	0.0	0.0	0.1	0.0	0.1
LOC101927445	0.1	0.0	0.1	0.0	0.0	0.0	0.1	0.0	0.0
TMEM150B	0.1	0.0	0.0	0.0	0.0	0.0	0.1	0.0	0.1
CSTA	0.0	0.0	0.0	0.0	0.0	0.0	0.1	0.0	0.1
DIRAS1	0.1	0.0	0.0	0.0	0.0	0.0	0.1	0.0	0.1
FGF8	0.1	0.0	0.0	0.0	0.0	0.0	0.1	0.0	0.0
LOC101928942	0.1	0.0	0.1	0.0	0.0	0.0	0.1	0.0	0.0
LOC101927330	0.1	0.0	0.0	0.0	0.0	0.0	0.2	0.0	0.0
HLA-DQA1	0.1	0.1	0.0	0.0	0.0	0.0	0.1	0.0	0.0
RUNX3	0.2	0.0	0.0	0.0	0.0	0.0	0.1	0.0	0.0
GRIK1-AS1	0.1	0.0	0.0	0.0	0.0	0.0	0.1	0.0	0.0
NKG7	0.0	0.1	0.1	0.0	0.0	0.0	0.1	0.1	0.0
C9orf139	0.0	0.1	0.0	0.0	0.0	0.0	0.1	0.0	0.0
UTS2B	0.0	0.1	0.1	0.0	0.0	0.0	0.1	0.0	0.0

LOC101927944		0.1	0.0	0.0	0.0	0.0	0.0	0.1	0.0	0.0
FAM26E		0.1	0.0	0.0	0.0	0.0	0.0	0.1	0.0	0.0
IL1RL1		0.0	0.0	0.0	0.0	0.0	0.0	0.1	0.0	0.0
OR6A2		0.1	0.0	0.0	0.0	0.0	0.0	0.1	0.0	0.1
AADAC		0.1	0.0	0.0	0.0	0.0	0.0	0.1	0.0	0.0
C17orf98		0.0	0.0	0.0	0.0	0.0	0.0	0.1	0.0	0.1
LOC101927250		0.0	0.0	0.1	0.0	0.0	0.0	0.1	0.0	0.1
GZMB		0.0	0.0	0.1	0.0	0.0	0.0	0.0	0.0	0.0
ENDOU		0.1	0.0	0.0	0.0	0.0	0.0	0.0	0.0	0.1
CLEC19A		0.0	0.1	0.1	0.0	0.0	0.0	0.0	0.0	0.1
IGKV1D-33		0.0	0.0	0.1	0.0	0.0	0.0	0.0	0.0	0.0
IGKV1-33		0.0	0.0	0.1	0.0	0.0	0.0	0.0	0.0	0.0
IL17B		0.1	0.0	0.1	0.0	0.0	0.0	0.1	0.0	0.0
CPSF4L		0.0	0.0	0.0	0.0	0.0	0.0	0.2	0.0	0.0
TCP11		0.0	0.0	0.1	0.0	0.0	0.0	0.1	0.0	0.0
C8orf12		0.1	0.0	0.0	0.0	0.0	0.0	0.1	0.0	0.0
LOC101928837		0.1	0.0	0.1	0.0	0.0	0.0	0.0	0.0	0.1
LOC101928489		0.1	0.0	0.1	0.0	0.0	0.0	0.0	0.0	0.0
IRG1		0.1	0.0	0.1	0.0	0.0	0.0	0.1	0.0	0.0
FGD2		0.1	0.0	0.1	0.0	0.0	0.0	0.0	0.0	0.0
KRTAP5-1		0.1	0.0	0.0	0.0	0.0	0.0	0.0	0.0	0.0
TAS2R41		0.0	0.0	0.1	0.0	0.0	0.0	0.0	0.0	0.0
SYT5		0.1	0.0	0.0	0.0	0.0	0.0	0.0	0.0	0.0
RAB3B		0.0	0.0	0.0	0.0	0.0	0.0	0.1	0.0	0.0
P2RY13		0.0	0.0	0.0	0.0	0.0	0.0	0.1	0.0	0.1
SYN2		0.0	0.1	0.0	0.0	0.0	0.0	0.0	0.0	0.0
SPEM1		0.0	0.0	0.0	0.0	0.0	0.0	0.1	0.0	0.0
SYCE1L		0.1	0.1	0.0	0.0	0.0	0.0	0.1	0.0	0.0
ADH1C		0.0	0.0	0.0	0.0	0.0	0.0	0.1	0.0	0.1
TTC39A		0.1	0.0	0.0	0.0	0.0	0.0	0.0	0.0	0.0
ZNF667-AS1		0.0	0.0	0.0	0.0	0.0	0.0	0.1	0.0	0.0
NAMA		0.1	0.0	0.0	0.0	0.0	0.0	0.0	0.0	0.0
CHRD		0.1	0.0	0.0	0.0	0.0	0.0	0.0	0.0	0.0
OMD		0.1	0.0	0.0	0.0	0.0	0.0	0.0	0.0	0.0
SFTPA2		0.0	0.0	0.0	0.0	0.0	0.0	0.0	0.0	0.0
LMAN1L		0.1	0.0	0.0	0.0	0.0	0.0	0.0	0.0	0.0
TLDC2		0.0	0.0	0.0	0.0	0.0	0.0	0.0	0.0	0.1
RNF151		0.1	0.0	0.0	0.0	0.0	0.0	0.0	0.0	0.0
LOC101929274		0.0	0.0	0.1	0.0	0.0	0.0	0.1	0.0	0.0
LOC101928377		0.1	0.0	0.0	0.0	0.0	0.0	0.0	0.0	0.0
NPFFR1		0.1	0.0	0.0	0.0	0.0	0.0	0.0	0.0	0.0
PRR18		0.0	0.1	0.0	0.0	0.0	0.0	0.0	0.0	0.0
CDH17		0.1	0.0	0.0	0.0	0.0	0.0	0.0	0.0	0.0
C6orf25		0.1	0.0	0.0	0.0	0.0	0.0	0.0	0.0	0.0
GPR61		0.0	0.0	0.1	0.0	0.0	0.0	0.0	0.0	0.0
LOC101805489		0.1	0.0	0.0	0.0	0.0	0.0	0.0	0.0	0.0
GPR174		0.0	0.0	0.0	0.0	0.0	0.0	0.0	0.0	0.1
CYP24A1		0.1	0.0	0.0	0.0	0.0	0.0	0.0	0.0	0.0
LINC00238		0.1	0.0	0.0	0.0	0.0	0.0	0.0	0.0	0.0
LOC285095		0.1	0.1	0.0	0.0	0.0	0.0	0.0	0.0	0.0
TUSC7		0.1	0.0	0.0	0.0	0.0	0.0	0.0	0.0	0.0
LOC101928175		0.0	0.0	0.0	0.0	0.0	0.0	0.0	0.0	0.1
ALDOB		0.1	0.0	0.0	0.0	0.0	0.0	0.0	0.0	0.0
	44807.0	0.0	0.0	0.1	0.0	0.0	0.0	0.0	0.0	0.0
LMOD3		0.0	0.0	0.0	0.0	0.0	0.0	0.0	0.0	0.0

FCN1	0.0	0.0	0.1	0.0	0.0	0.0	0.0	0.0	0.0
LOC101927675	0.0	0.0	0.0	0.0	0.0	0.0	0.0	0.0	0.0
TGM3	0.1	0.0	0.0	0.0	0.0	0.0	0.0	0.0	0.0
ALOX5	0.0	0.0	0.0	0.0	0.0	0.0	0.0	0.0	0.0
LOC101928708	0.0	0.0	0.0	0.0	0.0	0.0	0.0	0.0	0.0
COL23A1	0.0	0.0	0.0	0.0	0.0	0.0	0.0	0.0	0.0
SLC11A1	0.1	0.0	0.0	0.0	0.0	0.0	0.0	0.0	0.0
PGC	0.0	0.0	0.0	0.0	0.0	0.0	0.0	0.0	0.0
IGKV1-6	0.0	0.0	0.0	0.0	0.0	0.0	0.0	0.0	0.0
LOC101929655	0.0	0.0	0.0	0.0	0.0	0.0	0.0	0.0	0.0
LOC101929110	0.0	0.0	0.0	0.0	0.0	0.0	0.0	0.0	0.0
TNFRSF8	0.1	0.0	0.0	0.0	0.0	0.0	0.0	0.0	0.0
GIMAP6	0.0	0.0	0.0	0.0	0.0	0.0	0.0	0.0	0.0
DRC1	0.0	0.0	0.0	0.0	0.0	0.0	0.0	0.0	0.0
HAP1	0.0	0.0	0.0	0.0	0.0	0.0	0.0	0.0	0.0
EML1	0.0	0.0	0.0	0.0	0.0	0.0	0.0	0.0	0.0
TEX28	0.0	0.0	0.0	0.0	0.0	0.0	0.0	0.0	0.0
LOC101928075	0.0	0.0	0.0	0.0	0.0	0.0	0.0	0.0	0.0
LOC100507250	0.0	0.0	0.0	0.0	0.0	0.0	0.0	0.0	0.0
FAS-AS1	0.0	0.0	0.0	0.0	0.0	0.0	0.0	0.0	0.0
GGT2	0.0	0.0	0.0	0.0	0.0	0.0	0.0	0.0	0.0
ELAVL3	0.0	0.0	0.0	0.0	0.0	0.0	0.0	0.0	0.0
PSG11	0.0	0.0	0.0	0.0	0.0	0.0	0.0	0.0	0.0
RASL10A	0.1	0.0	0.0	0.0	0.0	0.0	0.0	0.0	0.0
OOSP1	0.0	0.0	0.0	0.0	0.0	0.0	0.0	0.0	0.0
TMEM63C	0.0	0.0	0.0	0.0	0.0	0.0	0.0	0.0	0.0
PDE6C	0.0	0.0	0.0	0.0	0.0	0.0	0.0	0.0	0.0
PRRT4	0.0	0.0	0.0	0.0	0.0	0.0	0.0	0.0	0.0
TUBB4A	0.0	0.0	0.0	0.0	0.0	0.0	0.0	0.0	0.0
GLIS1	0.0	0.0	0.0	0.0	0.0	0.0	0.0	0.0	0.0
TAS1R2	0.0	0.0	0.0	0.0	0.0	0.0	0.0	0.0	0.0
SIGLEC1	0.0	0.0	0.0	0.0	0.0	0.0	0.0	0.0	0.0
EXOC3L4	0.0	0.0	0.0	0.0	0.0	0.0	0.0	0.0	0.0
LOC101927662	0.0	0.0	0.0	0.0	0.0	0.0	0.0	0.0	0.0
BTK	0.0	0.0	0.0	0.0	0.0	0.0	0.0	0.0	0.0
PROM1	0.0	0.0	0.0	0.0	0.0	0.0	0.0	0.0	0.0
LRRC36	0.0	0.0	0.0	0.0	0.0	0.0	0.0	0.0	0.0
FANCD2OS	0.0	0.1	0.0	0.0	0.0	0.0	0.0	0.0	0.0
CYP2C19	0.0	0.0	0.0	0.0	0.0	0.0	0.0	0.0	0.0
LOC100131047	0.0	0.0	0.0	0.0	0.0	0.0	0.0	0.0	0.0
LOC101928517	0.0	0.0	0.0	0.0	0.0	0.0	0.0	0.0	0.0
LOC101927087	0.0	0.0	0.0	0.0	0.0	0.0	0.0	0.0	0.0
TPTE	0.0	0.0	0.0	0.0	0.0	0.0	0.0	0.0	0.0
ALOX15	0.0	0.0	0.0	0.0	0.0	0.0	0.0	0.0	0.0
CNTNAP2	0.0	0.0	0.0	0.0	0.0	0.0	0.0	0.0	0.0
LOC441493	0.0	0.0	0.0	0.0	0.0	0.0	0.0	0.0	0.0
PPIAL4F	0.0	0.0	0.0	0.0	0.0	0.0	0.0	0.0	0.0
PPIAL4D	0.0	0.0	0.0	0.0	0.0	0.0	0.0	0.0	0.0
LGALS9B	0.0	0.0	0.0	0.0	0.0	0.0	0.0	0.0	0.0
LOC100507389	0.0	0.0	0.0	0.0	0.0	0.0	0.0	0.0	0.0
IQSEC3	0.0	0.0	0.0	0.0	0.0	0.0	0.0	0.0	0.0
LOC101927594	0.0	0.0	0.0	0.0	0.0	0.0	0.0	0.0	0.0
ABCB1	0.0	0.0	0.0	0.0	0.0	0.0	0.0	0.0	0.0
LINC00951	0.0	0.0	0.0	0.0	0.0	0.0	0.0	0.0	0.0
MROH5	0.0	0.0	0.0	0.0	0.0	0.0	0.0	0.0	0.0

LILRB4	0.0	0.0	0.0	0.0	0.0	0.0	0.0	0.0	0.0
LOC100129345	0.0	0.0	0.0	0.0	0.0	0.0	0.0	0.0	0.0
ABCA12	0.0	0.0	0.0	0.0	0.0	0.0	0.0	0.0	0.0
TCHH	0.0	0.0	0.0	0.0	0.0	0.0	0.0	0.0	0.0
LOC101927628	0.0	0.0	0.0	0.0	0.0	0.0	0.0	0.0	0.0
LOC101928623	2.1	0.3	0.0	0.2	0.0	0.0	0.0	0.0	0.0
RNU6-31P	1.0	0.3	0.0	0.4	0.0	0.3	0.0	0.0	0.0
LOC101927173	0.1	0.0	0.2	0.0	0.1	0.0	0.0	0.0	0.0
BTG2	0.0	0.1	0.1	0.1	0.1	0.0	0.0	0.0	0.0
LOC101060091	0.1	0.0	0.0	0.0	0.1	0.0	0.0	0.0	0.0
HESX1	0.0	0.0	0.2	0.0	0.0	0.1	0.0	0.0	0.0
SYPL2	0.1	0.0	0.1	0.1	0.0	0.0	0.0	0.0	0.0
LOC101927401	0.2	0.1	0.0	0.0	0.0	0.1	0.0	0.0	0.0
LOC101929679	0.2	0.0	0.1	0.0	0.1	0.0	0.0	0.0	0.0
CDIPT-AS1	0.1	0.0	0.0	0.0	0.1	0.1	0.0	0.0	0.0
LOC101928513	0.0	0.0	0.1	0.0	0.0	0.0	0.0	0.0	0.0
FAM107A	0.1	0.1	0.0	0.0	0.0	0.0	0.0	0.0	0.0
TPSD1	0.0	0.1	0.1	0.0	0.0	0.1	0.0	0.0	0.0
TMEM17	0.0	0.1	0.1	0.0	0.1	0.1	0.0	0.0	0.0
FGF7	0.1	0.1	0.0	0.0	0.1	0.0	0.0	0.0	0.0
LOC100506098	0.1	0.0	0.1	0.0	0.1	0.0	0.0	0.0	0.0
RNF223	0.1	0.0	0.1	0.0	0.0	0.0	0.0	0.0	0.0
LOC100130298	0.1	0.1	0.0	0.0	0.1	0.0	0.0	0.0	0.0
LOC100506036	0.1	0.0	0.0	0.0	0.0	0.0	0.0	0.0	0.0
CXCR3	0.2	0.0	0.0	0.0	0.0	0.0	0.0	0.0	0.0
RHOH	0.1	0.0	0.0	0.0	0.1	0.0	0.0	0.0	0.0
CRYBA4	0.1	0.0	0.0	0.0	0.0	0.1	0.0	0.0	0.0
FLJ44313	0.0	0.1	0.1	0.0	0.0	0.1	0.0	0.0	0.0
RDM1	0.2	0.0	0.0	0.0	0.0	0.1	0.0	0.0	0.0
MAGEB2	0.0	0.0	0.0	0.0	0.0	0.0	0.0	0.0	0.0
LOC101927569	0.1	0.1	0.0	0.0	0.0	0.0	0.0	0.0	0.0
OR10A5	0.1	0.0	0.1	0.0	0.0	0.0	0.0	0.0	0.0
TNKS2-AS1	0.1	0.0	0.0	0.0	0.1	0.1	0.0	0.0	0.0
PHOX2A	0.1	0.0	0.1	0.0	0.0	0.0	0.0	0.0	0.0
SASH3	0.1	0.0	0.0	0.0	0.0	0.0	0.0	0.0	0.0
GC	0.1	0.0	0.0	0.0	0.0	0.0	0.0	0.0	0.0
SLC22A31	0.1	0.0	0.0	0.0	0.0	0.0	0.0	0.0	0.0
FFAR1	0.0	0.1	0.0	0.0	0.0	0.0	0.0	0.0	0.0
ZNF559-ZNF177	0.0	0.0	0.0	0.0	0.1	0.0	0.0	0.0	0.0
ADAP2	0.0	0.0	0.0	0.0	0.1	0.0	0.0	0.0	0.0
LOC645485	0.0	0.0	0.1	0.0	0.0	0.0	0.0	0.0	0.0
TFAP2B	0.0	0.0	0.0	0.0	0.1	0.0	0.0	0.0	0.0
LOC101928213	0.0	0.0	0.1	0.1	0.0	0.0	0.0	0.0	0.0
CDH1	0.0	0.0	0.0	0.0	0.0	0.0	0.0	0.0	0.0
FBXW10	0.0	0.0	0.0	0.0	0.0	0.0	0.0	0.0	0.0
LOC101927660	0.1	0.0	0.0	0.0	0.0	0.0	0.0	0.0	0.0
OIT3	0.0	0.0	0.0	0.0	0.0	0.0	0.0	0.0	0.0
PHOX2B	0.0	0.1	0.0	0.0	0.0	0.0	0.0	0.0	0.0
FAM183CP	0.0	0.0	0.0	0.0	0.0	0.0	0.0	0.0	0.0
PVRL3-AS1	0.0	0.0	0.0	0.0	0.0	0.0	0.0	0.0	0.0
GPR114	0.0	0.0	0.0	0.0	0.0	0.0	0.0	0.0	0.0
SLCO1C1	0.0	0.0	0.0	0.0	0.0	0.0	0.0	0.0	0.0
EPHA8	0.0	0.0	0.0	0.0	0.0	0.0	0.0	0.0	0.0
ARPP21	0.0	0.0	0.0	0.0	0.0	0.0	0.0	0.0	0.0
UNCX	0.0	0.0	0.0	0.0	0.0	0.0	0.0	0.0	0.0

LOC101927928	0.0	0.0	0.0	0.0	0.0	0.0	0.0	0.0	0.0
ADCY1	0.0	0.0	0.0	0.0	0.0	0.0	0.0	0.0	0.0
CD248	0.0	0.0	0.0	0.0	0.0	0.0	0.0	0.0	0.0
TCEAL5	0.0	0.0	0.0	0.0	0.0	0.0	0.0	0.0	0.0
MUC3A	0.0	0.0	0.0	0.0	0.0	0.0	0.0	0.0	0.0
LOC401357	0.0	0.0	0.0	0.0	0.0	0.0	0.0	0.0	0.0
LOC101929738	0.0	0.0	0.0	0.0	0.0	0.0	0.0	0.0	0.0
MYO1H	0.0	0.0	0.0	0.0	0.0	0.0	0.0	0.0	0.0
CEACAM5	0.0	0.0	0.0	0.0	0.0	0.0	0.0	0.0	0.0
FLJ44635	0.0	0.0	0.0	0.0	0.0	0.0	0.0	0.0	0.0
NOX5	0.0	0.0	0.0	0.0	0.0	0.0	0.0	0.0	0.0
LOC101927137	2.6	0.0	2.0	0.3	0.5	0.0	0.0	0.0	0.0
LOC101929623	0.2	0.0	0.1	0.1	0.2	0.0	0.0	0.0	0.0
IL17C	0.3	0.0	0.0	0.0	0.0	0.0	0.0	0.0	0.0
LHX6	0.2	0.0	0.1	0.0	0.1	0.0	0.0	0.0	0.0
RCAN2	0.2	0.0	0.0	0.1	0.0	0.0	0.0	0.0	0.0
LOC101927241	0.0	0.1	0.0	0.0	0.0	0.0	0.0	0.0	0.0
WFIKKN2	0.1	0.0	0.0	0.0	0.0	0.0	0.0	0.0	0.0
GDNF	0.0	0.0	0.0	0.1	0.1	0.0	0.0	0.0	0.0
HGD	0.0	0.1	0.0	0.0	0.1	0.0	0.0	0.0	0.0
LOC100506474	0.1	0.0	0.0	0.0	0.0	0.0	0.0	0.0	0.0
SMPX	0.1	0.1	0.0	0.0	0.0	0.0	0.0	0.0	0.0
TMEM82	0.1	0.0	0.0	0.0	0.0	0.0	0.0	0.0	0.0
MSX1	0.0	0.0	0.1	0.0	0.0	0.0	0.0	0.0	0.0
HOXA13	0.1	0.0	0.0	0.0	0.0	0.0	0.0	0.0	0.0
NDNF	0.1	0.0	0.0	0.0	0.0	0.0	0.0	0.0	0.0
FAAH2	0.0	0.0	0.0	0.0	0.0	0.1	0.0	0.0	0.0
LOC101927851	0.0	0.0	0.0	0.0	0.1	0.0	0.0	0.0	0.0
CCIN	0.0	0.0	0.0	0.0	0.0	0.0	0.0	0.0	0.0
AUTS2	0.0	0.0	0.0	0.0	0.0	0.0	0.0	0.0	0.0
DNAJB8	0.0	0.0	0.0	0.0	0.1	0.0	0.0	0.0	0.0
GPR55	0.0	0.0	0.0	0.0	0.0	0.0	0.0	0.0	0.0
LOC101927726	0.0	0.0	0.0	0.0	0.0	0.1	0.0	0.0	0.0
RSC1A1	0.0	0.0	0.0	0.0	0.0	0.0	0.0	0.0	0.0
TRNAA39	0.0	0.0	0.0	0.0	0.0	0.0	1.1	0.1	0.6
TRNAA3	0.0	0.0	0.0	0.0	0.0	0.0	1.1	0.1	0.6
SPIB	0.0	0.0	0.0	0.0	0.0	0.0	0.2	0.1	0.2
LOC646513	0.0	0.0	0.0	0.0	0.0	0.0	0.1	0.0	0.2
CLEC4A	0.0	0.0	0.0	0.0	0.0	0.0	0.2	0.0	0.1
PYCARD	0.0	0.0	0.0	0.0	0.0	0.0	0.2	0.0	0.0
KRTAP4-8	0.0	0.0	0.0	0.0	0.0	0.0	0.2	0.0	0.1
C2CD4D	0.0	0.0	0.0	0.0	0.0	0.0	0.1	0.0	0.1
IL17REL	0.0	0.0	0.0	0.0	0.0	0.0	0.1	0.0	0.1
LDHAL6B	0.0	0.0	0.0	0.0	0.0	0.0	0.1	0.0	0.0
CCR1	0.0	0.0	0.0	0.0	0.0	0.0	0.0	0.1	0.1
MYB	0.0	0.0	0.0	0.0	0.0	0.0	0.0	0.0	0.1
CCER1	0.0	0.0	0.0	0.0	0.0	0.0	0.0	0.0	0.0
PRDM7	0.0	0.0	0.0	0.0	0.0	0.0	0.0	0.0	0.0
ACSBG1	0.0	0.0	0.0	0.0	0.0	0.0	0.0	0.0	0.0
CYP2C18	0.0	0.0	0.0	0.0	0.0	0.0	0.0	0.0	0.1
FLI1	0.0	0.0	0.0	0.0	0.0	0.0	0.0	0.0	0.0
CDS1	0.0	0.0	0.0	0.0	0.0	0.0	0.0	0.0	0.0
CTAGE1	0.0	0.0	0.0	0.0	0.0	0.0	0.0	0.0	0.0
MAP6	0.0	0.0	0.0	0.0	0.0	0.0	0.0	0.0	0.0
NLRP12	0.0	0.0	0.0	0.0	0.0	0.0	0.0	0.0	0.0

LOC101929430	0.0	0.0	0.0	0.0	0.0	0.0	0.0	0.0	0.0
LOC101929362	0.0	0.0	0.0	0.0	0.0	0.0	0.0	0.0	0.0
SNORD63	0.0	0.0	0.0	0.0	0.0	0.0	0.4	0.4	0.4
MRGPRD	0.0	0.0	0.0	0.0	0.0	0.0	0.1	0.0	0.1
CAPNS2	0.0	0.0	0.0	0.0	0.0	0.0	0.0	0.0	0.0
TRNAR21	0.0	0.0	0.0	0.0	0.0	0.0	0.4	0.1	0.1
TRNAR2	0.0	0.0	0.0	0.0	0.0	0.0	0.4	0.1	0.1
TRNAR26	0.0	0.0	0.0	0.0	0.0	0.0	0.4	0.1	0.1
LOC100507661	0.0	0.0	0.0	0.0	0.0	0.0	0.2	0.0	0.0
LOC101927684	0.0	0.0	0.0	0.0	0.0	0.0	0.0	0.0	0.2
LOC101928601	0.0	0.0	0.0	0.0	0.0	0.0	0.1	0.0	0.0
PNMT	0.0	0.0	0.0	0.0	0.0	0.0	0.1	0.0	0.0
AMELX	0.0	0.0	0.0	0.0	0.0	0.0	0.1	0.0	0.0
RASL11B	0.0	0.0	0.0	0.0	0.0	0.0	0.0	0.0	0.1
FMOD	0.0	0.0	0.0	0.0	0.0	0.0	0.0	0.0	0.1
C11orf34	0.0	0.0	0.0	0.0	0.0	0.0	0.0	0.0	0.1
OSM	0.0	0.0	0.0	0.0	0.0	0.0	0.1	0.0	0.0
CAPN6	0.0	0.0	0.0	0.0	0.0	0.0	0.0	0.0	0.0
ARX	0.0	0.0	0.0	0.0	0.0	0.0	0.1	0.0	0.0
LMO1	0.0	0.0	0.0	0.0	0.0	0.0	0.0	0.0	0.1
KCNN3	0.0	0.0	0.0	0.0	0.0	0.0	0.0	0.0	0.0
LOC101928757	0.0	0.0	0.0	0.0	0.0	0.0	0.0	0.0	0.0
C7	0.0	0.0	0.0	0.0	0.0	0.0	0.0	0.0	0.0
TMC3	0.0	0.0	0.0	0.0	0.0	0.0	0.0	0.0	0.0
TRNAI4	0.0	0.0	0.0	0.0	0.0	0.0	0.6	0.2	0.1
TRNAI11	0.0	0.0	0.0	0.0	0.0	0.0	0.6	0.2	0.1
TRNAQ21	0.0	0.0	0.0	0.0	0.0	0.0	0.4	0.1	0.1
LOC388780	0.0	0.0	0.0	0.0	0.0	0.0	0.2	0.0	0.1
PDX1	0.0	0.0	0.0	0.0	0.0	0.0	0.0	0.0	0.0
NIPAL4	0.0	0.0	0.0	0.0	0.0	0.0	0.0	0.0	0.0
PRDM13	0.0	0.0	0.0	0.0	0.0	0.0	0.0	0.0	0.0
KTI12	0.0	0.0	0.0	0.1	0.3	0.3	0.0	0.0	0.0
ZCCHC5	0.0	0.0	0.0	0.0	0.1	0.1	0.0	0.0	0.0
GJB4	0.0	0.0	0.0	0.0	0.0	0.0	0.0	0.0	0.0
GATSL1	0.0	0.0	0.0	0.0	0.1	0.0	0.0	0.0	0.0
H2AFB1	0.0	0.0	0.0	0.0	0.0	0.1	0.0	0.0	0.0
GGTLC2	0.0	0.0	0.0	0.0	0.0	0.0	0.0	0.0	0.0
GRIN2A	0.0	0.0	0.0	0.0	0.0	0.0	0.0	0.0	0.0
GJA9	0.0	0.0	0.0	0.0	0.0	0.0	0.0	0.0	0.0
LOC101929710	0.6	0.4	0.3	0.0	0.0	0.0	0.0	0.0	0.0
LOC441155	0.8	0.3	0.0	0.0	0.0	0.0	0.0	0.0	0.0
LOC101929150	0.3	0.1	0.2	0.0	0.0	0.0	0.0	0.0	0.0
RNF224	0.4	0.1	0.5	0.0	0.0	0.0	0.0	0.0	0.0
YPEL1	0.3	0.1	0.4	0.0	0.0	0.0	0.0	0.0	0.0
SNORA5B	0.3	0.2	0.2	0.0	0.0	0.0	0.0	0.0	0.0
HSD17B3	0.3	0.1	0.3	0.0	0.0	0.0	0.0	0.0	0.0
HES5	0.1	0.2	0.2	0.0	0.0	0.0	0.0	0.0	0.0
CYS1	0.3	0.3	0.1	0.0	0.0	0.0	0.0	0.0	0.0
LOC101927850	0.3	0.2	0.2	0.0	0.0	0.0	0.0	0.0	0.0
ARL9	0.2	0.1	0.2	0.0	0.0	0.0	0.0	0.0	0.0
OR8G2	0.3	0.1	0.2	0.0	0.0	0.0	0.0	0.0	0.0
LINC00035	0.2	0.2	0.2	0.0	0.0	0.0	0.0	0.0	0.0
LOC101928635	0.1	0.1	0.1	0.0	0.0	0.0	0.0	0.0	0.0
C10orf111	0.3	0.1	0.1	0.0	0.0	0.0	0.0	0.0	0.0
FKBP1B	0.0	0.3	0.1	0.0	0.0	0.0	0.0	0.0	0.0

MGARP	0.3	0.1	0.1	0.0	0.0	0.0	0.0	0.0	0.0
IGKC	0.4	0.1	0.0	0.0	0.0	0.0	0.0	0.0	0.0
TM4SF18	0.2	0.1	0.1	0.0	0.0	0.0	0.0	0.0	0.0
LOC101928712	0.4	0.0	0.1	0.0	0.0	0.0	0.0	0.0	0.0
MAP2K6	0.2	0.1	0.1	0.0	0.0	0.0	0.0	0.0	0.0
LOC101929149	0.2	0.1	0.1	0.0	0.0	0.0	0.0	0.0	0.0
LOC101926934	0.1	0.1	0.2	0.0	0.0	0.0	0.0	0.0	0.0
TAS2R13	0.2	0.0	0.1	0.0	0.0	0.0	0.0	0.0	0.0
SCGB2B2	0.2	0.1	0.2	0.0	0.0	0.0	0.0	0.0	0.0
RD3	0.1	0.1	0.1	0.0	0.0	0.0	0.0	0.0	0.0
BMP8A	0.2	0.0	0.1	0.0	0.0	0.0	0.0	0.0	0.0
LOC101928402	0.2	0.1	0.0	0.0	0.0	0.0	0.0	0.0	0.0
HLA-DOA	0.1	0.1	0.2	0.0	0.0	0.0	0.0	0.0	0.0
CRYAB	0.2	0.1	0.0	0.0	0.0	0.0	0.0	0.0	0.0
FLJ42351	0.2	0.0	0.2	0.0	0.0	0.0	0.0	0.0	0.0
CALY	0.1	0.1	0.1	0.0	0.0	0.0	0.0	0.0	0.0
RFX4	0.1	0.1	0.1	0.0	0.0	0.0	0.0	0.0	0.0
SBK2	0.1	0.1	0.1	0.0	0.0	0.0	0.0	0.0	0.0
CIB2	0.1	0.1	0.0	0.0	0.0	0.0	0.0	0.0	0.0
FGF2	0.1	0.0	0.1	0.0	0.0	0.0	0.0	0.0	0.0
LEKR1	0.2	0.1	0.0	0.0	0.0	0.0	0.0	0.0	0.0
NKX6-2	0.0	0.1	0.1	0.0	0.0	0.0	0.0	0.0	0.0
DNALI1	0.2	0.0	0.0	0.0	0.0	0.0	0.0	0.0	0.0
ANKK1	0.1	0.0	0.1	0.0	0.0	0.0	0.0	0.0	0.0
DLL3	0.2	0.0	0.1	0.0	0.0	0.0	0.0	0.0	0.0
C10orf62	0.1	0.0	0.1	0.0	0.0	0.0	0.0	0.0	0.0
BTLA	0.1	0.1	0.0	0.0	0.0	0.0	0.0	0.0	0.0
BARHL2	0.1	0.0	0.0	0.0	0.0	0.0	0.0	0.0	0.0
LINC00640	0.2	0.1	0.0	0.0	0.0	0.0	0.0	0.0	0.0
CITED4	0.1	0.0	0.0	0.0	0.0	0.0	0.0	0.0	0.0
OR11H2	0.2	0.1	0.0	0.0	0.0	0.0	0.0	0.0	0.0
NAT2	0.0	0.0	0.0	0.0	0.0	0.0	0.0	0.0	0.0
FUT3	0.2	0.0	0.0	0.0	0.0	0.0	0.0	0.0	0.0
LINC00460	0.1	0.0	0.0	0.0	0.0	0.0	0.0	0.0	0.0
KLHL41	0.1	0.0	0.1	0.0	0.0	0.0	0.0	0.0	0.0
NDUFA4L2	0.1	0.0	0.1	0.0	0.0	0.0	0.0	0.0	0.0
BTNL2	0.1	0.1	0.0	0.0	0.0	0.0	0.0	0.0	0.0
ELOVL2	0.1	0.0	0.1	0.0	0.0	0.0	0.0	0.0	0.0
LOC100129060	0.1	0.0	0.1	0.0	0.0	0.0	0.0	0.0	0.0
KPNA7	0.1	0.0	0.0	0.0	0.0	0.0	0.0	0.0	0.0
TULP1	0.0	0.0	0.0	0.0	0.0	0.0	0.0	0.0	0.0
EXOC3L2	0.1	0.0	0.1	0.0	0.0	0.0	0.0	0.0	0.0
ARL14	0.1	0.0	0.0	0.0	0.0	0.0	0.0	0.0	0.0
CD27	0.0	0.1	0.0	0.0	0.0	0.0	0.0	0.0	0.0
DQX1	0.1	0.0	0.1	0.0	0.0	0.0	0.0	0.0	0.0
TMPRSS11A	0.1	0.0	0.1	0.0	0.0	0.0	0.0	0.0	0.0
P2RY10	0.0	0.0	0.1	0.0	0.0	0.0	0.0	0.0	0.0
FAM221B	0.1	0.0	0.0	0.0	0.0	0.0	0.0	0.0	0.0
LOC101929201	0.1	0.0	0.1	0.0	0.0	0.0	0.0	0.0	0.0
KCNJ12	0.1	0.0	0.0	0.0	0.0	0.0	0.0	0.0	0.0
MAP7D2	0.1	0.0	0.0	0.0	0.0	0.0	0.0	0.0	0.0
OR52D1	0.0	0.0	0.1	0.0	0.0	0.0	0.0	0.0	0.0
LOC101929133	0.0	0.0	0.0	0.0	0.0	0.0	0.0	0.0	0.0
NXF3	0.0	0.0	0.0	0.0	0.0	0.0	0.0	0.0	0.0
PFKFB1	0.0	0.0	0.0	0.0	0.0	0.0	0.0	0.0	0.0

LOC100505918	0.1	0.0	0.0	0.0	0.0	0.0	0.0	0.0	0.0
LOC101929076	0.1	0.0	0.0	0.0	0.0	0.0	0.0	0.0	0.0
QPRT	0.0	0.0	0.0	0.0	0.0	0.0	0.0	0.0	0.0
TRPM8	0.1	0.0	0.0	0.0	0.0	0.0	0.0	0.0	0.0
LOC101928879	0.1	0.0	0.0	0.0	0.0	0.0	0.0	0.0	0.0
SLFN11	0.1	0.0	0.0	0.0	0.0	0.0	0.0	0.0	0.0
ZNF540	0.1	0.0	0.0	0.0	0.0	0.0	0.0	0.0	0.0
LOC101059957	0.1	0.0	0.0	0.0	0.0	0.0	0.0	0.0	0.0
FCRL6	0.1	0.0	0.0	0.0	0.0	0.0	0.0	0.0	0.0
LOC101929730	0.1	0.0	0.0	0.0	0.0	0.0	0.0	0.0	0.0
PRAMEF17	0.1	0.0	0.0	0.0	0.0	0.0	0.0	0.0	0.0
FGR	0.0	0.0	0.0	0.0	0.0	0.0	0.0	0.0	0.0
CTAGE6	0.1	0.0	0.0	0.0	0.0	0.0	0.0	0.0	0.0
MGAT3	0.0	0.0	0.0	0.0	0.0	0.0	0.0	0.0	0.0
SLC35D3	0.0	0.0	0.0	0.0	0.0	0.0	0.0	0.0	0.0
ALS2CR12	0.0	0.0	0.0	0.0	0.0	0.0	0.0	0.0	0.0
LOC101927475	0.0	0.0	0.0	0.0	0.0	0.0	0.0	0.0	0.0
ASIC4	0.1	0.0	0.0	0.0	0.0	0.0	0.0	0.0	0.0
STEAP4	0.1	0.0	0.0	0.0	0.0	0.0	0.0	0.0	0.0
FAM90A1	0.1	0.0	0.0	0.0	0.0	0.0	0.0	0.0	0.0
IGSF22	0.1	0.0	0.0	0.0	0.0	0.0	0.0	0.0	0.0
SLC6A3	0.1	0.0	0.0	0.0	0.0	0.0	0.0	0.0	0.0
ENPP2	0.0	0.0	0.0	0.0	0.0	0.0	0.0	0.0	0.0
LINC00693	0.0	0.0	0.0	0.0	0.0	0.0	0.0	0.0	0.0
FGB	0.0	0.0	0.0	0.0	0.0	0.0	0.0	0.0	0.0
C2orf62	0.1	0.0	0.0	0.0	0.0	0.0	0.0	0.0	0.0
LINC00461	0.0	0.0	0.0	0.0	0.0	0.0	0.0	0.0	0.0
CCDC108	0.0	0.0	0.0	0.0	0.0	0.0	0.0	0.0	0.0
LOC101929572	0.0	0.0	0.0	0.0	0.0	0.0	0.0	0.0	0.0
LOC100505625	0.0	0.0	0.0	0.0	0.0	0.0	0.0	0.0	0.0
TRIM71	0.0	0.0	0.0	0.0	0.0	0.0	0.0	0.0	0.0
LOC101927949	0.0	0.0	0.0	0.0	0.0	0.0	0.0	0.0	0.0
OVOS	0.0	0.0	0.0	0.0	0.0	0.0	0.0	0.0	0.0
CLLU1	0.0	0.0	0.0	0.0	0.0	0.0	0.0	0.0	0.0
COL19A1	0.0	0.0	0.0	0.0	0.0	0.0	0.0	0.0	0.0
LINC00858	0.0	0.0	0.0	0.0	0.0	0.0	0.0	0.0	0.0
SALL4	0.0	0.0	0.0	0.0	0.0	0.0	0.0	0.0	0.0
SLC26A10	0.0	0.0	0.0	0.0	0.0	0.0	0.0	0.0	0.0
COL28A1	0.0	0.0	0.0	0.0	0.0	0.0	0.0	0.0	0.0
AQP9	0.0	0.0	0.0	0.0	0.0	0.0	0.0	0.0	0.0
RBM20	0.0	0.0	0.0	0.0	0.0	0.0	0.0	0.0	0.0
CTNNA3	0.0	0.0	0.0	0.0	0.0	0.0	0.0	0.0	0.0
LOC100506895	0.0	0.0	0.0	0.0	0.0	0.0	0.0	0.0	0.0
GRM7	0.0	0.0	0.0	0.0	0.0	0.0	0.0	0.0	0.0
IPW	0.0	0.0	0.0	0.0	0.0	0.0	0.0	0.0	0.0
LOC100996492	0.0	0.0	0.0	0.0	0.0	0.0	0.0	0.0	0.0
NOVA2	0.0	0.0	0.0	0.0	0.0	0.0	0.0	0.0	0.0
KIAA1239	0.0	0.0	0.0	0.0	0.0	0.0	0.0	0.0	0.0
MTTP	0.0	0.0	0.0	0.0	0.0	0.0	0.0	0.0	0.0
MMEL1	0.0	0.0	0.0	0.0	0.0	0.0	0.0	0.0	0.0
LOC100508046	0.0	0.0	0.0	0.0	0.0	0.0	0.0	0.0	0.0
SLITRK3	0.0	0.0	0.0	0.0	0.0	0.0	0.0	0.0	0.0
UNC79	0.0	0.0	0.0	0.0	0.0	0.0	0.0	0.0	0.0
DSCAML1	0.0	0.0	0.0	0.0	0.0	0.0	0.0	0.0	0.0
NBPF4	0.0	0.0	0.0	0.0	0.0	0.0	0.0	0.0	0.0

WNK2	0.0	0.0	0.0	0.0	0.0	0.0	0.0	0.0	0.0
SNORD84	1.0	0.3	0.7	0.0	0.0	0.0	0.0	0.0	0.0
SNORA15	0.6	0.7	0.4	0.0	0.0	0.0	0.0	0.0	0.0
HYPK	0.0	0.7	0.0	0.0	0.0	0.0	0.0	0.0	0.0
SPTSSB	0.5	0.3	0.1	0.0	0.0	0.0	0.0	0.0	0.0
LOC101927418	0.4	0.2	0.0	0.0	0.0	0.0	0.0	0.0	0.0
HSPB8	0.3	0.2	0.0	0.0	0.0	0.0	0.0	0.0	0.0
TRNAU1	0.7	0.0	0.0	0.0	0.0	0.0	0.0	0.0	0.0
HSPB9	0.2	0.1	0.2	0.0	0.0	0.0	0.0	0.0	0.0
FKSG29	0.2	0.1	0.1	0.0	0.0	0.0	0.0	0.0	0.0
FXYD2	0.4	0.1	0.1	0.0	0.0	0.0	0.0	0.0	0.0
LOC101060626	0.5	0.0	0.0	0.0	0.0	0.0	0.0	0.0	0.0
TSACC	0.1	0.1	0.1	0.0	0.0	0.0	0.0	0.0	0.0
TNNC2	0.2	0.1	0.1	0.0	0.0	0.0	0.0	0.0	0.0
LOC101927563	0.2	0.1	0.1	0.0	0.0	0.0	0.0	0.0	0.0
LOC101927113	0.2	0.0	0.0	0.0	0.0	0.0	0.0	0.0	0.0
BDKRB1	0.3	0.1	0.1	0.0	0.0	0.0	0.0	0.0	0.0
LOC100996618	0.1	0.1	0.1	0.0	0.0	0.0	0.0	0.0	0.0
LOC101928899	0.1	0.1	0.1	0.0	0.0	0.0	0.0	0.0	0.0
LOC101926914	0.1	0.2	0.1	0.0	0.0	0.0	0.0	0.0	0.0
C21orf62	0.2	0.1	0.1	0.0	0.0	0.0	0.0	0.0	0.0
LOC101927308	0.1	0.1	0.1	0.0	0.0	0.0	0.0	0.0	0.0
LOC101928969	0.2	0.1	0.0	0.0	0.0	0.0	0.0	0.0	0.0
SH2D6	0.2	0.0	0.1	0.0	0.0	0.0	0.0	0.0	0.0
SNORA70E	0.1	0.0	0.0	0.0	0.0	0.0	0.0	0.0	0.0
FSBP	0.1	0.0	0.1	0.0	0.0	0.0	0.0	0.0	0.0
HCG14	0.2	0.1	0.0	0.0	0.0	0.0	0.0	0.0	0.0
C12orf79	0.3	0.0	0.1	0.0	0.0	0.0	0.0	0.0	0.0
LOC100505948	0.2	0.0	0.0	0.0	0.0	0.0	0.0	0.0	0.0
LOC100128176	0.2	0.0	0.1	0.0	0.0	0.0	0.0	0.0	0.0
LOC401052	0.1	0.1	0.0	0.0	0.0	0.0	0.0	0.0	0.0
SERPINB4	0.2	0.1	0.1	0.0	0.0	0.0	0.0	0.0	0.0
NEBL-AS1	0.2	0.0	0.1	0.0	0.0	0.0	0.0	0.0	0.0
LOC101928704	0.1	0.0	0.1	0.0	0.0	0.0	0.0	0.0	0.0
MOG	0.2	0.1	0.1	0.0	0.0	0.0	0.0	0.0	0.0
RAB33A	0.1	0.1	0.1	0.0	0.0	0.0	0.0	0.0	0.0
LOC101929384	0.3	0.0	0.1	0.0	0.0	0.0	0.0	0.0	0.0
LOC100134259	0.2	0.0	0.1	0.0	0.0	0.0	0.0	0.0	0.0
KIF25	0.2	0.0	0.1	0.0	0.0	0.0	0.0	0.0	0.0
AKR1B10	0.1	0.0	0.1	0.0	0.0	0.0	0.0	0.0	0.0
ACTL7A	0.0	0.0	0.1	0.0	0.0	0.0	0.0	0.0	0.0
LPAR6	0.2	0.0	0.0	0.0	0.0	0.0	0.0	0.0	0.0
FAM83E	0.1	0.1	0.0	0.0	0.0	0.0	0.0	0.0	0.0
CASC16	0.0	0.1	0.0	0.0	0.0	0.0	0.0	0.0	0.0
LOC101927482	0.1	0.0	0.0	0.0	0.0	0.0	0.0	0.0	0.0
LINC00499	0.1	0.0	0.0	0.0	0.0	0.0	0.0	0.0	0.0
UPK2	0.0	0.0	0.1	0.0	0.0	0.0	0.0	0.0	0.0
SERPINC1	0.1	0.0	0.0	0.0	0.0	0.0	0.0	0.0	0.0
CD207	0.1	0.0	0.0	0.0	0.0	0.0	0.0	0.0	0.0
PRB3	0.1	0.1	0.0	0.0	0.0	0.0	0.0	0.0	0.0
LOC283440	0.1	0.0	0.1	0.0	0.0	0.0	0.0	0.0	0.0
NMBR	0.1	0.0	0.1	0.0	0.0	0.0	0.0	0.0	0.0
SLC16A14	0.1	0.0	0.1	0.0	0.0	0.0	0.0	0.0	0.0
LOC101928852	0.1	0.1	0.0	0.0	0.0	0.0	0.0	0.0	0.0
FGF14	0.1	0.0	0.0	0.0	0.0	0.0	0.0	0.0	0.0

NMRK2	0.1	0.0	0.1	0.0	0.0	0.0	0.0	0.0	0.0
CYSLTR1	0.1	0.0	0.1	0.0	0.0	0.0	0.0	0.0	0.0
LOC101928090	0.2	0.0	0.0	0.0	0.0	0.0	0.0	0.0	0.0
DPYS	0.0	0.0	0.1	0.0	0.0	0.0	0.0	0.0	0.0
TBR1	0.1	0.0	0.0	0.0	0.0	0.0	0.0	0.0	0.0
SLC6A13	0.1	0.0	0.1	0.0	0.0	0.0	0.0	0.0	0.0
BHMT2	0.1	0.0	0.1	0.0	0.0	0.0	0.0	0.0	0.0
TNF	0.1	0.0	0.0	0.0	0.0	0.0	0.0	0.0	0.0
PRB1	0.1	0.1	0.0	0.0	0.0	0.0	0.0	0.0	0.0
CCR5	0.0	0.0	0.1	0.0	0.0	0.0	0.0	0.0	0.0
LOC101928077	0.1	0.0	0.0	0.0	0.0	0.0	0.0	0.0	0.0
PRB2	0.1	0.0	0.0	0.0	0.0	0.0	0.0	0.0	0.0
KLRC1	0.1	0.1	0.0	0.0	0.0	0.0	0.0	0.0	0.0
ACTL7B	0.1	0.0	0.0	0.0	0.0	0.0	0.0	0.0	0.0
XKRX	0.0	0.0	0.1	0.0	0.0	0.0	0.0	0.0	0.0
LOC101928664	0.1	0.0	0.0	0.0	0.0	0.0	0.0	0.0	0.0
LOC101927988	0.0	0.0	0.0	0.0	0.0	0.0	0.0	0.0	0.0
BCL2L14	0.1	0.0	0.0	0.0	0.0	0.0	0.0	0.0	0.0
LOC101927955	0.1	0.0	0.0	0.0	0.0	0.0	0.0	0.0	0.0
CRNN	0.1	0.0	0.0	0.0	0.0	0.0	0.0	0.0	0.0
LOC101929373	0.1	0.0	0.0	0.0	0.0	0.0	0.0	0.0	0.0
OR5B12	0.0	0.0	0.0	0.0	0.0	0.0	0.0	0.0	0.0
BCAS1	0.0	0.0	0.0	0.0	0.0	0.0	0.0	0.0	0.0
KRT32	0.1	0.0	0.0	0.0	0.0	0.0	0.0	0.0	0.0
TMEM108	0.0	0.0	0.0	0.0	0.0	0.0	0.0	0.0	0.0
MEFV	0.1	0.0	0.0	0.0	0.0	0.0	0.0	0.0	0.0
PTPRD	0.0	0.0	0.0	0.0	0.0	0.0	0.0	0.0	0.0
FOXJ1	0.0	0.0	0.0	0.0	0.0	0.0	0.0	0.0	0.0
DUX4L4	0.0	0.0	0.0	0.0	0.0	0.0	0.0	0.0	0.0
SLC8A3	0.1	0.0	0.0	0.0	0.0	0.0	0.0	0.0	0.0
LOC728175	0.0	0.0	0.1	0.0	0.0	0.0	0.0	0.0	0.0
MACC1	0.0	0.0	0.0	0.0	0.0	0.0	0.0	0.0	0.0
ST7-OT3	0.0	0.0	0.0	0.0	0.0	0.0	0.0	0.0	0.0
IL20RA	0.1	0.0	0.0	0.0	0.0	0.0	0.0	0.0	0.0
LOC374890	0.1	0.0	0.0	0.0	0.0	0.0	0.0	0.0	0.0
CATSPERD	0.1	0.0	0.0	0.0	0.0	0.0	0.0	0.0	0.0
SLC9B2	0.0	0.0	0.0	0.0	0.0	0.0	0.0	0.0	0.0
PTCHD3	0.1	0.0	0.0	0.0	0.0	0.0	0.0	0.0	0.0
THSD7B	0.0	0.0	0.0	0.0	0.0	0.0	0.0	0.0	0.0
CATSPERB	0.0	0.0	0.0	0.0	0.0	0.0	0.0	0.0	0.0
GLB1L2	0.0	0.0	0.0	0.0	0.0	0.0	0.0	0.0	0.0
CNTFR	0.0	0.0	0.0	0.0	0.0	0.0	0.0	0.0	0.0
LOC101927734	0.0	0.0	0.0	0.0	0.0	0.0	0.0	0.0	0.0
C1orf173	0.0	0.0	0.0	0.0	0.0	0.0	0.0	0.0	0.0
COL15A1	0.0	0.0	0.0	0.0	0.0	0.0	0.0	0.0	0.0
DCDC5	0.0	0.0	0.0	0.0	0.0	0.0	0.0	0.0	0.0
PCDH15	0.0	0.0	0.0	0.0	0.0	0.0	0.0	0.0	0.0
JAKMIP3	0.0	0.0	0.0	0.0	0.0	0.0	0.0	0.0	0.0
C1QTNF9B-AS1	0.0	0.0	0.0	0.0	0.0	0.0	0.0	0.0	0.0
SHISA8	0.0	0.0	0.0	0.0	0.0	0.0	0.0	0.0	0.0
HOOK1	0.0	0.0	0.0	0.0	0.0	0.0	0.0	0.0	0.0
SHCBP1L	0.0	0.0	0.0	0.0	0.0	0.0	0.0	0.0	0.0
NBPF6	0.0	0.0	0.0	0.0	0.0	0.0	0.0	0.0	0.0
TTTY9A	0.0	0.0	0.0	0.0	0.0	0.0	0.0	0.0	0.0
TTTY9B	0.0	0.0	0.0	0.0	0.0	0.0	0.0	0.0	0.0

ZAN	0.0	0.0	0.0	0.0	0.0	0.0	0.0	0.0	0.0
LOC101928647	0.0	0.0	0.0	0.0	0.1	0.0	0.0	0.0	0.0
CUZD1	0.0	0.0	0.0	0.0	0.1	0.0	0.0	0.0	0.0
LAMTOR5-AS1	0.0	0.0	0.0	0.0	0.0	0.0	0.0	0.0	0.0
TGM5	0.0	0.0	0.0	0.0	0.0	0.0	0.0	0.0	0.0
KLHL32	0.0	0.0	0.0	0.0	0.0	0.0	0.0	0.0	0.0
CADPS	0.0	0.0	0.0	0.0	0.0	0.0	0.0	0.0	0.0
EPHA10	0.0	0.0	0.0	0.0	0.0	0.0	0.0	0.0	0.0
DUSP27	0.0	0.0	0.0	0.0	0.0	0.0	0.0	0.0	0.0
GNG2	2.9	0.8	0.9	0.0	0.0	0.0	0.0	0.0	0.0
CST2	0.9	0.2	0.4	0.0	0.0	0.0	0.0	0.0	0.0
ARL14EPL	0.4	0.2	0.2	0.0	0.0	0.0	0.0	0.0	0.0
C1QL4	0.3	0.1	0.1	0.0	0.0	0.0	0.0	0.0	0.0
TAL2	0.2	0.0	0.1	0.0	0.0	0.0	0.0	0.0	0.0
CA7	0.2	0.1	0.2	0.0	0.0	0.0	0.0	0.0	0.0
RAB41	0.2	0.0	0.1	0.0	0.0	0.0	0.0	0.0	0.0
CD200R1	0.2	0.0	0.0	0.0	0.0	0.0	0.0	0.0	0.0
CH25H	0.1	0.2	0.0	0.0	0.0	0.0	0.0	0.0	0.0
SNORA70B	0.3	0.0	0.0	0.0	0.0	0.0	0.0	0.0	0.0
LOC101926936	0.0	0.1	0.2	0.0	0.0	0.0	0.0	0.0	0.0
SCRG1	0.0	0.0	0.2	0.0	0.0	0.0	0.0	0.0	0.0
PLK5	0.1	0.0	0.1	0.0	0.0	0.0	0.0	0.0	0.0
LOC101929769	0.1	0.1	0.1	0.0	0.0	0.0	0.0	0.0	0.0
ZAR1	0.2	0.0	0.0	0.0	0.0	0.0	0.0	0.0	0.0
PPAPDC3	0.1	0.0	0.0	0.0	0.0	0.0	0.0	0.0	0.0
HSPB2-C11orf52	0.1	0.1	0.0	0.0	0.0	0.0	0.0	0.0	0.0
PLBD1	0.1	0.0	0.1	0.0	0.0	0.0	0.0	0.0	0.0
LDB3	0.1	0.1	0.0	0.0	0.0	0.0	0.0	0.0	0.0
C9orf171	0.0	0.0	0.1	0.0	0.0	0.0	0.0	0.0	0.0
LOC101929314	0.2	0.0	0.0	0.0	0.0	0.0	0.0	0.0	0.0
ASB4	0.0	0.1	0.0	0.0	0.0	0.0	0.0	0.0	0.0
IQCJ	0.1	0.0	0.0	0.0	0.0	0.0	0.0	0.0	0.0
LRRC19	0.1	0.0	0.0	0.0	0.0	0.0	0.0	0.0	0.0
DCAF12L2	0.1	0.0	0.0	0.0	0.0	0.0	0.0	0.0	0.0
OR2D3	0.1	0.0	0.0	0.0	0.0	0.0	0.0	0.0	0.0
IGLON5	0.1	0.0	0.0	0.0	0.0	0.0	0.0	0.0	0.0
IL18RAP	0.1	0.0	0.0	0.0	0.0	0.0	0.0	0.0	0.0
PTGFR	0.0	0.1	0.0	0.0	0.0	0.0	0.0	0.0	0.0
LOC100505851	0.1	0.0	0.0	0.0	0.0	0.0	0.0	0.0	0.0
ENPP3	0.1	0.0	0.0	0.0	0.0	0.0	0.0	0.0	0.0
GPR151	0.0	0.0	0.0	0.0	0.0	0.0	0.0	0.0	0.0
C10orf85	0.0	0.0	0.0	0.0	0.0	0.0	0.0	0.0	0.0
LOC646241	0.0	0.0	0.0	0.0	0.0	0.0	0.0	0.0	0.0
CDHR4	0.0	0.0	0.0	0.0	0.0	0.0	0.0	0.0	0.0
LOC151171	0.0	0.0	0.0	0.0	0.0	0.0	0.0	0.0	0.0
P2RX1	0.0	0.0	0.0	0.0	0.0	0.0	0.0	0.0	0.0
EMILIN3	0.0	0.0	0.0	0.0	0.0	0.0	0.0	0.0	0.0
LOC101927965	0.0	0.0	0.0	0.0	0.0	0.0	0.0	0.0	0.0
SCN4A	0.0	0.0	0.0	0.0	0.0	0.0	0.0	0.0	0.0
DGKG	0.0	0.0	0.0	0.0	0.0	0.0	0.0	0.0	0.0
DNAH8	0.0	0.0	0.0	0.0	0.0	0.0	0.0	0.0	0.0
SNORD87	0.5	0.3	0.4	0.0	0.0	0.0	0.0	0.0	0.0
CXCL5	0.2	0.2	0.5	0.0	0.0	0.0	0.0	0.0	0.0
TRNAS5	0.5	0.3	0.0	0.0	0.0	0.0	0.0	0.0	0.0
TNFAIP6	0.3	0.0	0.1	0.0	0.0	0.0	0.0	0.0	0.0

NFE2	0.2	0.1	0.1	0.0	0.0	0.0	0.0	0.0	0.0
LOC101927429	0.2	0.0	0.2	0.0	0.0	0.0	0.0	0.0	0.0
CNBD2	0.2	0.1	0.1	0.0	0.0	0.0	0.0	0.0	0.0
ANKRD22	0.1	0.1	0.1	0.0	0.0	0.0	0.0	0.0	0.0
GDF3	0.2	0.0	0.1	0.0	0.0	0.0	0.0	0.0	0.0
ATOH7	0.1	0.1	0.1	0.0	0.0	0.0	0.0	0.0	0.0
LOC101928039	0.1	0.0	0.1	0.0	0.0	0.0	0.0	0.0	0.0
CA9	0.1	0.1	0.0	0.0	0.0	0.0	0.0	0.0	0.0
HS3ST2	0.1	0.0	0.0	0.0	0.0	0.0	0.0	0.0	0.0
LOC100133746	0.1	0.0	0.1	0.0	0.0	0.0	0.0	0.0	0.0
FRZB	0.1	0.0	0.1	0.0	0.0	0.0	0.0	0.0	0.0
LOC101928806	0.1	0.1	0.0	0.0	0.0	0.0	0.0	0.0	0.0
FANK1-AS1	0.1	0.0	0.1	0.0	0.0	0.0	0.0	0.0	0.0
SPIC	0.2	0.0	0.0	0.0	0.0	0.0	0.0	0.0	0.0
EHF	0.1	0.0	0.0	0.0	0.0	0.0	0.0	0.0	0.0
LINC00898	0.1	0.0	0.0	0.0	0.0	0.0	0.0	0.0	0.0
OR13C4	0.1	0.0	0.0	0.0	0.0	0.0	0.0	0.0	0.0
RMDN2-AS1	0.1	0.0	0.0	0.0	0.0	0.0	0.0	0.0	0.0
NPFFR2	0.0	0.0	0.1	0.0	0.0	0.0	0.0	0.0	0.0
HOXD12	0.0	0.0	0.0	0.0	0.0	0.0	0.0	0.0	0.0
GPR152	0.1	0.0	0.0	0.0	0.0	0.0	0.0	0.0	0.0
INHA	0.0	0.0	0.1	0.0	0.0	0.0	0.0	0.0	0.0
CFHR3	0.1	0.0	0.0	0.0	0.0	0.0	0.0	0.0	0.0
GRHL3	0.1	0.0	0.0	0.0	0.0	0.0	0.0	0.0	0.0
ART1	0.0	0.0	0.0	0.0	0.0	0.0	0.0	0.0	0.0
LOC101927521	0.0	0.0	0.0	0.0	0.0	0.0	0.0	0.0	0.0
GH1	0.0	0.0	0.0	0.0	0.0	0.0	0.0	0.0	0.0
NR0B2	0.1	0.0	0.0	0.0	0.0	0.0	0.0	0.0	0.0
HK3	0.0	0.0	0.0	0.0	0.0	0.0	0.0	0.0	0.0
PLEKHS1	0.0	0.0	0.0	0.0	0.0	0.0	0.0	0.0	0.0
HRC	0.0	0.0	0.0	0.0	0.0	0.0	0.0	0.0	0.0
LOC101928858	0.0	0.0	0.0	0.0	0.0	0.0	0.0	0.0	0.0
CHDC2	0.0	0.0	0.0	0.0	0.0	0.0	0.0	0.0	0.0
TBX4	0.0	0.0	0.0	0.0	0.0	0.0	0.0	0.0	0.0
RGS7BP	0.0	0.0	0.0	0.0	0.0	0.0	0.0	0.0	0.0
SLC4A1	0.0	0.0	0.0	0.0	0.0	0.0	0.0	0.0	0.0
IMPG1	0.1	0.0	0.0	0.0	0.0	0.0	0.0	0.0	0.0
REXO1L1	0.0	0.0	0.0	0.0	0.0	0.0	0.0	0.0	0.0
FLJ27255	0.0	0.0	0.0	0.0	0.0	0.0	0.0	0.0	0.0
ERN2	0.0	0.0	0.0	0.0	0.0	0.0	0.0	0.0	0.0
SATB2-AS1	0.0	0.0	0.0	0.0	0.0	0.0	0.0	0.0	0.0
LOC101928961	0.0	0.0	0.0	0.0	0.0	0.0	0.0	0.0	0.0
LOC100288562	0.0	0.0	0.0	0.0	0.0	0.0	0.0	0.0	0.0
LOC101929627	0.0	0.0	0.0	0.0	0.0	0.0	0.0	0.0	0.0
LOC101929601	0.0	0.0	0.0	0.0	0.0	0.0	0.0	0.0	0.0
CPAMD8	0.0	0.0	0.0	0.0	0.0	0.0	0.0	0.0	0.0
KY	0.0	0.0	0.0	0.0	0.0	0.0	0.0	0.0	0.0
ABCB4	0.0	0.0	0.0	0.0	0.0	0.0	0.0	0.0	0.0
TCEB3B	0.0	0.0	0.0	0.0	0.0	0.0	0.0	0.0	0.0
SLC7A8	0.0	0.0	0.0	0.0	0.0	0.0	0.0	0.0	0.0
FLT4	0.0	0.0	0.0	0.0	0.0	0.0	0.0	0.0	0.0
SCN7A	0.0	0.0	0.0	0.0	0.0	0.0	0.0	0.0	0.0
SNORD58B	0.0	0.0	0.0	0.0	0.0	0.0	0.9	0.0	0.4
GNG13	0.0	0.0	0.0	0.0	0.0	0.0	0.4	0.0	0.2
TRNAL28	0.0	0.0	0.0	0.0	0.0	0.0	0.0	0.1	0.5

SNORD61	0.0	0.0	0.0	0.0	0.0	0.0	0.2	0.0	0.2
LOC101927771	0.0	0.0	0.0	0.0	0.0	0.0	0.2	0.0	0.0
LOC101927978	0.0	0.0	0.0	0.0	0.0	0.0	0.1	0.0	0.1
LOC101929269	0.0	0.0	0.0	0.0	0.0	0.0	0.1	0.0	0.0
LHFPL5	0.0	0.0	0.0	0.0	0.0	0.0	0.1	0.0	0.0
SMLR1	0.0	0.0	0.0	0.0	0.0	0.0	0.2	0.0	0.0
CA2	0.0	0.0	0.0	0.0	0.0	0.0	0.2	0.0	0.1
LOC101928530	0.0	0.0	0.0	0.0	0.0	0.0	0.3	0.0	0.1
TNNT2	0.0	0.0	0.0	0.0	0.0	0.0	0.2	0.0	0.2
LOC101928747	0.0	0.0	0.0	0.0	0.0	0.0	0.1	0.0	0.1
LOC101928557	0.0	0.0	0.0	0.0	0.0	0.0	0.1	0.0	0.0
HCRT	0.0	0.0	0.0	0.0	0.0	0.0	0.1	0.1	0.0
S100Z	0.0	0.0	0.0	0.0	0.0	0.0	0.0	0.0	0.0
S100A9	0.0	0.0	0.0	0.0	0.0	0.0	0.1	0.0	0.0
LOC100996671	0.0	0.0	0.0	0.0	0.0	0.0	0.1	0.0	0.0
TRNAI23	0.0	0.0	0.0	0.0	0.0	0.0	0.1	0.0	0.1
TRNAI15	0.0	0.0	0.0	0.0	0.0	0.0	0.1	0.0	0.1
TRNAI17	0.0	0.0	0.0	0.0	0.0	0.0	0.1	0.0	0.1
TRNAI20	0.0	0.0	0.0	0.0	0.0	0.0	0.1	0.0	0.1
TRNAI9	0.0	0.0	0.0	0.0	0.0	0.0	0.1	0.0	0.1
GLRA3	0.0	0.0	0.0	0.0	0.0	0.0	0.0	0.0	0.1
LOC101927318	0.0	0.0	0.0	0.0	0.0	0.0	0.1	0.0	0.1
LOC101927975	0.0	0.0	0.0	0.0	0.0	0.0	0.1	0.0	0.1
TNFSF13B	0.0	0.0	0.0	0.0	0.0	0.0	0.0	0.0	0.1
WFDC2	0.0	0.0	0.0	0.0	0.0	0.0	0.1	0.0	0.0
LOC101929641	0.0	0.0	0.0	0.0	0.0	0.0	0.1	0.1	0.0
C6orf195	0.0	0.0	0.0	0.0	0.0	0.0	0.1	0.0	0.1
LOC101929461	0.0	0.0	0.0	0.0	0.0	0.0	0.1	0.0	0.0
LOC101929434	0.0	0.0	0.0	0.0	0.0	0.0	0.2	0.0	0.0
FAM133A	0.0	0.0	0.0	0.0	0.0	0.0	0.0	0.0	0.1
ASTL	0.0	0.0	0.0	0.0	0.0	0.0	0.1	0.0	0.1
LOC100996457	0.0	0.0	0.0	0.0	0.0	0.0	0.1	0.0	0.1
CSAG2	0.0	0.0	0.0	0.0	0.0	0.0	0.1	0.0	0.0
CSAG3	0.0	0.0	0.0	0.0	0.0	0.0	0.1	0.0	0.0
CCDC3	0.0	0.0	0.0	0.0	0.0	0.0	0.1	0.0	0.1
LOC101928802	0.0	0.0	0.0	0.0	0.0	0.0	0.1	0.0	0.0
LOC101928625	0.0	0.0	0.0	0.0	0.0	0.0	0.1	0.0	0.1
HSD17B13	0.0	0.0	0.0	0.0	0.0	0.0	0.1	0.0	0.0
LCP2	0.0	0.0	0.0	0.0	0.0	0.0	0.1	0.0	0.0
LOC100132111	0.0	0.0	0.0	0.0	0.0	0.0	0.0	0.0	0.1
LOC101928723	0.0	0.0	0.0	0.0	0.0	0.0	0.0	0.0	0.0
OR2AT4	0.0	0.0	0.0	0.0	0.0	0.0	0.1	0.0	0.0
DLEU7	0.0	0.0	0.0	0.0	0.0	0.0	0.0	0.0	0.0
LPAR4	0.0	0.0	0.0	0.0	0.0	0.0	0.1	0.0	0.0
TTC23L	0.0	0.0	0.0	0.0	0.0	0.0	0.0	0.0	0.1
LOC101928469	0.0	0.0	0.0	0.0	0.0	0.0	0.1	0.0	0.0
LEFTY2	0.0	0.0	0.0	0.0	0.0	0.0	0.0	0.0	0.0
TAF7L	0.0	0.0	0.0	0.0	0.0	0.0	0.0	0.0	0.0
LOC101929174	0.0	0.0	0.0	0.0	0.0	0.0	0.0	0.0	0.1
LOC101927749	0.0	0.0	0.0	0.0	0.0	0.0	0.0	0.0	0.1
OR1K1	0.0	0.0	0.0	0.0	0.0	0.0	0.0	0.0	0.0
EPB41L4A-AS2	0.0	0.0	0.0	0.0	0.0	0.0	0.0	0.0	0.0
CNTF	0.0	0.0	0.0	0.0	0.0	0.0	0.0	0.0	0.1
OR6V1	0.0	0.0	0.0	0.0	0.0	0.0	0.0	0.0	0.0
A1CF	0.0	0.0	0.0	0.0	0.0	0.0	0.0	0.0	0.0

LOC101926976		0.0	0.0	0.0	0.0	0.0	0.0	0.0	0.0	0.0
KCNC1		0.0	0.0	0.0	0.0	0.0	0.0	0.0	0.0	0.0
CERS3		0.0	0.0	0.0	0.0	0.0	0.0	0.0	0.0	0.0
LOC101927686		0.0	0.0	0.0	0.0	0.0	0.0	0.0	0.0	0.0
SLC47A1		0.0	0.0	0.0	0.0	0.0	0.0	0.0	0.0	0.0
TRIM31		0.0	0.0	0.0	0.0	0.0	0.0	0.0	0.0	0.0
LOC101928938		0.0	0.0	0.0	0.0	0.0	0.0	0.0	0.0	0.0
APOL5		0.0	0.0	0.0	0.0	0.0	0.0	0.0	0.0	0.0
	44809.0	0.0	0.0	0.0	0.0	0.0	0.0	0.0	0.0	0.1
ADAMTS8		0.0	0.0	0.0	0.0	0.0	0.0	0.0	0.0	0.0
NR5A1		0.0	0.0	0.0	0.0	0.0	0.0	0.0	0.0	0.0
GALNT15		0.0	0.0	0.0	0.0	0.0	0.0	0.0	0.0	0.0
ALDH1L1-AS2		0.0	0.0	0.0	0.0	0.0	0.0	0.0	0.0	0.0
TEK		0.0	0.0	0.0	0.0	0.0	0.0	0.0	0.0	0.0
LECT1		0.0	0.0	0.0	0.0	0.0	0.0	0.0	0.0	0.0
ADAM30		0.0	0.0	0.0	0.0	0.0	0.0	0.0	0.0	0.0
LOC285768		0.0	0.0	0.0	0.0	0.0	0.0	0.0	0.0	0.0
ITK		0.0	0.0	0.0	0.0	0.0	0.0	0.0	0.0	0.0
TDRD12		0.0	0.0	0.0	0.0	0.0	0.0	0.0	0.0	0.0
LOC441728		0.0	0.0	0.0	0.0	0.0	0.0	0.0	0.0	0.0
SELP		0.0	0.0	0.0	0.0	0.0	0.0	0.0	0.0	0.0
EFCAB10		0.0	0.0	0.0	0.0	0.0	0.0	0.0	0.0	0.0
GRIN3A		0.0	0.0	0.0	0.0	0.0	0.0	0.0	0.0	0.0
LOC100506351		0.0	0.0	0.0	0.0	0.0	0.0	0.0	0.0	0.0
FAM87A		0.0	0.0	0.0	0.0	0.0	0.0	0.0	0.0	0.0
GAS2L2		0.0	0.0	0.0	0.0	0.0	0.0	0.0	0.0	0.0
NCAN		0.0	0.0	0.0	0.0	0.0	0.0	0.0	0.0	0.0
LOC101929563		0.0	0.0	0.0	0.0	0.0	0.0	0.0	0.0	0.0
C10orf71		0.0	0.0	0.0	0.0	0.0	0.0	0.0	0.0	0.0
GRID2IP		0.0	0.0	0.0	0.0	0.0	0.0	0.0	0.0	0.0
LOC101927283		0.0	0.0	0.0	0.0	0.0	0.0	0.0	0.0	0.0
SLC12A3		0.0	0.0	0.0	0.0	0.0	0.0	0.0	0.0	0.0
PCDH11Y		0.0	0.0	0.0	0.0	0.0	0.0	0.0	0.0	0.0
LINC00890		0.0	0.0	0.0	0.0	0.0	0.0	0.0	0.0	0.0
LINC00333		0.0	0.0	0.0	0.0	0.0	0.0	0.0	0.0	0.0
TRNAA7		0.0	0.0	0.0	0.0	0.0	0.0	0.4	0.0	0.4
TRNAY12		0.0	0.0	0.0	0.0	0.0	0.0	0.3	0.0	0.1
LOC101928822		0.0	0.0	0.0	0.0	0.0	0.0	0.1	0.0	0.1
HCN4		0.0	0.0	0.0	0.0	0.0	0.0	0.1	0.0	0.1
C3orf22		0.0	0.0	0.0	0.0	0.0	0.0	0.1	0.0	0.0
AQP1		0.0	0.0	0.0	0.0	0.0	0.0	0.1	0.0	0.1
OR11H12		0.0	0.0	0.0	0.0	0.0	0.0	0.0	0.0	0.1
RAB25		0.0	0.0	0.0	0.0	0.0	0.0	0.0	0.0	0.1
VN1R5		0.0	0.0	0.0	0.0	0.0	0.0	0.1	0.0	0.0
LOC101928619		0.0	0.0	0.0	0.0	0.0	0.0	0.0	0.0	0.0
KRTAP4-9		0.0	0.0	0.0	0.0	0.0	0.0	0.0	0.0	0.0
SLC38A5		0.0	0.0	0.0	0.0	0.0	0.0	0.1	0.0	0.0
LOC101929470		0.0	0.0	0.0	0.0	0.0	0.0	0.0	0.0	0.0
LOC101927778		0.0	0.0	0.0	0.0	0.0	0.0	0.0	0.0	0.0
LOC101927639		0.0	0.0	0.0	0.0	0.0	0.0	0.0	0.0	0.0
ESRRB		0.0	0.0	0.0	0.0	0.0	0.0	0.0	0.1	0.0
LOC101928579		0.0	0.0	0.0	0.0	0.0	0.0	0.0	0.0	0.0
LOC101928542		0.0	0.0	0.0	0.0	0.0	0.0	0.0	0.0	0.1
KCNG1		0.0	0.0	0.0	0.0	0.0	0.0	0.0	0.0	0.0
ISLR		0.0	0.0	0.0	0.0	0.0	0.0	0.0	0.0	0.0

PPP1R17	0.0	0.0	0.0	0.0	0.0	0.0	0.0	0.1	0.0
SOX11	0.0	0.0	0.0	0.0	0.0	0.0	0.0	0.0	0.0
LOC285441	0.0	0.0	0.0	0.0	0.0	0.0	0.1	0.0	0.0
SLC22A17	0.0	0.0	0.0	0.0	0.0	0.0	0.0	0.0	0.0
ZNF295-AS1	0.0	0.0	0.0	0.0	0.0	0.0	0.0	0.0	0.0
PKD2L2	0.0	0.0	0.0	0.0	0.0	0.0	0.1	0.0	0.0
NRON	0.0	0.0	0.0	0.0	0.0	0.0	0.0	0.0	0.0
GP1BB	0.0	0.0	0.0	0.0	0.0	0.0	0.0	0.0	0.0
SLC5A11	0.0	0.0	0.0	0.0	0.0	0.0	0.0	0.0	0.0
SYN3	0.0	0.0	0.0	0.0	0.0	0.0	0.0	0.0	0.0
EVC2	0.0	0.0	0.0	0.0	0.0	0.0	0.0	0.0	0.0
PDZRN3	0.0	0.0	0.0	0.0	0.0	0.0	0.0	0.0	0.0
C1orf200	0.0	0.0	0.0	0.0	0.0	0.0	0.0	0.0	0.0
RNA18S5	0.0	0.0	0.0	0.0	0.0	0.0	0.0	0.0	0.0
ZNF677	0.0	0.0	0.0	0.0	0.0	0.0	0.0	0.0	0.0
LOC101927320	0.0	0.0	0.0	0.0	0.0	0.0	0.0	0.0	0.0
LOC101927739	0.0	0.0	0.0	0.0	0.0	0.0	0.0	0.0	0.0
LOC101927669	0.0	0.0	0.0	0.0	0.0	0.0	0.0	0.0	0.0
ZNF84	0.0	0.0	0.0	0.0	0.0	0.0	0.0	0.0	0.0
TRNAR18	0.0	0.0	0.0	0.0	0.0	0.0	3.2	0.0	0.8
SNORD42A	0.0	0.0	0.0	0.0	0.0	0.0	0.0	0.4	0.9
TRNL2	0.0	0.0	0.0	0.0	0.0	0.0	0.4	0.0	0.4
TRNG	0.0	0.0	0.0	0.0	0.0	0.0	0.4	0.0	0.4
SNORD78	0.0	0.0	0.0	0.0	0.0	0.0	0.5	0.0	0.5
LOC101929562	0.0	0.0	0.0	0.0	0.0	0.0	0.5	0.0	0.1
LOC101928382	0.0	0.0	0.0	0.0	0.0	0.0	0.6	0.0	0.0
RNU6-66P	0.0	0.0	0.0	0.0	0.0	0.0	0.4	0.0	0.3
SNORA79	0.0	0.0	0.0	0.0	0.0	0.0	0.2	0.0	0.4
LOC100133123	0.0	0.0	0.0	0.0	0.0	0.0	0.4	0.0	0.2
SNORA2B	0.0	0.0	0.0	0.0	0.0	0.0	0.2	0.0	0.2
SCARNA15	0.0	0.0	0.0	0.0	0.0	0.0	0.0	0.0	0.4
TRNAA2	0.0	0.0	0.0	0.0	0.0	0.0	0.1	0.1	0.0
SNORA29	0.0	0.0	0.0	0.0	0.0	0.0	0.0	0.2	0.0
PAGE2B	0.0	0.0	0.0	0.0	0.0	0.0	0.4	0.0	0.1
SMIM18	0.0	0.0	0.0	0.0	0.0	0.0	0.1	0.0	0.1
CLPS	0.0	0.0	0.0	0.0	0.0	0.0	0.0	0.0	0.2
HIST1H1T	0.0	0.0	0.0	0.0	0.0	0.0	0.1	0.0	0.1
LOC101929656	0.0	0.0	0.0	0.0	0.0	0.0	0.1	0.0	0.1
TEX12	0.0	0.0	0.0	0.0	0.0	0.0	0.0	0.0	0.1
TRIM29	0.0	0.0	0.0	0.0	0.0	0.0	0.1	0.0	0.1
LINC00379	0.0	0.0	0.0	0.0	0.0	0.0	0.1	0.0	0.1
MEIS1-AS2	0.0	0.0	0.0	0.0	0.0	0.0	0.0	0.0	0.0
TEX29	0.0	0.0	0.0	0.0	0.0	0.0	0.1	0.0	0.1
CD3D	0.0	0.0	0.0	0.0	0.0	0.0	0.1	0.0	0.0
DIP2A-IT1	0.0	0.0	0.0	0.0	0.0	0.0	0.1	0.0	0.0
MYL3	0.0	0.0	0.0	0.0	0.0	0.0	0.1	0.0	0.0
ZNF728	0.0	0.0	0.0	0.0	0.0	0.0	0.0	0.0	0.2
CD48	0.0	0.0	0.0	0.0	0.0	0.0	0.1	0.0	0.0
DIAPH3-AS1	0.0	0.0	0.0	0.0	0.0	0.0	0.1	0.0	0.0
TMIE	0.0	0.0	0.0	0.0	0.0	0.0	0.1	0.0	0.1
PDGFB	0.0	0.0	0.0	0.0	0.0	0.0	0.1	0.0	0.1
HPCAL4	0.0	0.0	0.0	0.0	0.0	0.0	0.1	0.0	0.0
LOC101929119	0.0	0.0	0.0	0.0	0.0	0.0	0.0	0.0	0.2
TSPAN2	0.0	0.0	0.0	0.0	0.0	0.0	0.0	0.0	0.1
NKX2-5	0.0	0.0	0.0	0.0	0.0	0.0	0.0	0.0	0.1

LOC101928502	0.0	0.0	0.0	0.0	0.0	0.0	0.1	0.0	0.0
IGHV4-4	0.0	0.0	0.0	0.0	0.0	0.0	0.0	0.0	0.1
CD3E	0.0	0.0	0.0	0.0	0.0	0.0	0.0	0.0	0.0
HP	0.0	0.0	0.0	0.0	0.0	0.0	0.0	0.0	0.0
C10orf71-AS1	0.0	0.0	0.0	0.0	0.0	0.0	0.0	0.1	0.1
NCCRP1	0.0	0.0	0.0	0.0	0.0	0.0	0.0	0.0	0.1
HPX	0.0	0.0	0.0	0.0	0.0	0.0	0.1	0.0	0.0
CLDN11	0.0	0.0	0.0	0.0	0.0	0.0	0.0	0.0	0.0
LOC101928216	0.0	0.0	0.0	0.0	0.0	0.0	0.1	0.0	0.0
LYZ	0.0	0.0	0.0	0.0	0.0	0.0	0.1	0.0	0.1
AMTN	0.0	0.0	0.0	0.0	0.0	0.0	0.0	0.0	0.0
AARD	0.0	0.0	0.0	0.0	0.0	0.0	0.0	0.0	0.1
CD5L	0.0	0.0	0.0	0.0	0.0	0.0	0.1	0.0	0.0
LOC101929610	0.0	0.0	0.0	0.0	0.0	0.0	0.0	0.0	0.0
RPL3L	0.0	0.0	0.0	0.0	0.0	0.0	0.0	0.0	0.0
LOC101927546	0.0	0.0	0.0	0.0	0.0	0.0	0.1	0.0	0.0
AQP8	0.0	0.0	0.0	0.0	0.0	0.0	0.1	0.0	0.0
LOC101927163	0.0	0.0	0.0	0.0	0.0	0.0	0.1	0.0	0.0
LOC101927644	0.0	0.0	0.0	0.0	0.0	0.0	0.1	0.0	0.0
LOC101929477	0.0	0.0	0.0	0.0	0.0	0.0	0.0	0.1	0.0
CBFA2T3	0.0	0.0	0.0	0.0	0.0	0.0	0.1	0.0	0.0
EXD1	0.0	0.0	0.0	0.0	0.0	0.0	0.0	0.0	0.0
FAM170A	0.0	0.0	0.0	0.0	0.0	0.0	0.0	0.0	0.0
LILRA4	0.0	0.0	0.0	0.0	0.0	0.0	0.0	0.0	0.0
LOC101927392	0.0	0.0	0.0	0.0	0.0	0.0	0.0	0.0	0.0
POU3F1	0.0	0.0	0.0	0.0	0.0	0.0	0.1	0.0	0.0
CD69	0.0	0.0	0.0	0.0	0.0	0.0	0.0	0.0	0.0
LOC101928801	0.0	0.0	0.0	0.0	0.0	0.0	0.0	0.0	0.0
LOC101928459	0.0	0.0	0.0	0.0	0.0	0.0	0.0	0.0	0.0
LOC101928955	0.0	0.0	0.0	0.0	0.0	0.0	0.0	0.0	0.0
TRIM64C	0.0	0.0	0.0	0.0	0.0	0.0	0.0	0.0	0.0
HCK	0.0	0.0	0.0	0.0	0.0	0.0	0.0	0.0	0.0
RLBP1	0.0	0.0	0.0	0.0	0.0	0.0	0.0	0.0	0.0
OR2J2	0.0	0.0	0.0	0.0	0.0	0.0	0.0	0.0	0.0
SNX20	0.0	0.0	0.0	0.0	0.0	0.0	0.0	0.0	0.0
RFTN2	0.0	0.0	0.0	0.0	0.0	0.0	0.0	0.0	0.0
ADRA2B	0.0	0.0	0.0	0.0	0.0	0.0	0.0	0.0	0.0
GPR143	0.0	0.0	0.0	0.0	0.0	0.0	0.1	0.0	0.0
OR7D4	0.0	0.0	0.0	0.0	0.0	0.0	0.1	0.0	0.0
CKMT1B	0.0	0.0	0.0	0.0	0.0	0.0	0.0	0.0	0.0
TSGA10IP	0.0	0.0	0.0	0.0	0.0	0.0	0.0	0.0	0.0
DSC3	0.0	0.0	0.0	0.0	0.0	0.0	0.0	0.0	0.0
LOC101929109	0.0	0.0	0.0	0.0	0.0	0.0	0.0	0.0	0.0
ASB10	0.0	0.0	0.0	0.0	0.0	0.0	0.0	0.0	0.0
LOC101929198	0.0	0.0	0.0	0.0	0.0	0.0	0.0	0.0	0.0
LOC649133	0.0	0.0	0.0	0.0	0.0	0.0	0.0	0.0	0.0
NUGGC	0.0	0.0	0.0	0.0	0.0	0.0	0.0	0.0	0.0
HSD3B1	0.0	0.0	0.0	0.0	0.0	0.0	0.0	0.0	0.0
GIMAP1	0.0	0.0	0.0	0.0	0.0	0.0	0.0	0.0	0.0
SLC22A16	0.0	0.0	0.0	0.0	0.0	0.0	0.0	0.0	0.0
ZNF300	0.0	0.0	0.0	0.0	0.0	0.0	0.0	0.0	0.0
KCNA6	0.0	0.0	0.0	0.0	0.0	0.0	0.0	0.0	0.0
TRIM43	0.0	0.0	0.0	0.0	0.0	0.0	0.1	0.0	0.0
ASB18	0.0	0.0	0.0	0.0	0.0	0.0	0.0	0.0	0.0
SHD	0.0	0.0	0.0	0.0	0.0	0.0	0.0	0.0	0.0

PACRG-AS1	0.0	0.0	0.0	0.0	0.0	0.0	0.0	0.0	0.0
LOC100996380	0.0	0.0	0.0	0.0	0.0	0.0	0.0	0.0	0.0
SFTPA1	0.0	0.0	0.0	0.0	0.0	0.0	0.0	0.0	0.0
CDK3	0.0	0.0	0.0	0.0	0.0	0.0	0.0	0.0	0.0
TTC25	0.0	0.0	0.0	0.0	0.0	0.0	0.0	0.0	0.0
GPC6-AS2	0.0	0.0	0.0	0.0	0.0	0.0	0.0	0.0	0.0
MTUS2	0.0	0.0	0.0	0.0	0.0	0.0	0.0	0.0	0.0
LOC101928722	0.0	0.0	0.0	0.0	0.0	0.0	0.0	0.0	0.0
BEND4	0.0	0.0	0.0	0.0	0.0	0.0	0.0	0.0	0.0
GBP5	0.0	0.0	0.0	0.0	0.0	0.0	0.0	0.0	0.0
STOX2	0.0	0.0	0.0	0.0	0.0	0.0	0.0	0.0	0.0
KIAA0226L	0.0	0.0	0.0	0.0	0.0	0.0	0.0	0.0	0.0
MS4A15	0.0	0.0	0.0	0.0	0.0	0.0	0.0	0.0	0.0
CPXM2	0.0	0.0	0.0	0.0	0.0	0.0	0.0	0.0	0.0
GIMAP1-GIMAP5	0.0	0.0	0.0	0.0	0.0	0.0	0.0	0.0	0.0
COL8A2	0.0	0.0	0.0	0.0	0.0	0.0	0.0	0.0	0.0
OPN1MW2	0.0	0.0	0.0	0.0	0.0	0.0	0.0	0.0	0.0
OPN1MW	0.0	0.0	0.0	0.0	0.0	0.0	0.0	0.0	0.0
ITGB6	0.0	0.0	0.0	0.0	0.0	0.0	0.0	0.0	0.0
RPH3A	0.0	0.0	0.0	0.0	0.0	0.0	0.0	0.0	0.0
PNCK	0.0	0.0	0.0	0.0	0.0	0.0	0.0	0.0	0.0
RIMBP2	0.0	0.0	0.0	0.0	0.0	0.0	0.0	0.0	0.0
BVES-AS1	0.0	0.0	0.0	0.0	0.0	0.0	0.0	0.0	0.0
CD101	0.0	0.0	0.0	0.0	0.0	0.0	0.0	0.0	0.0
LOC101927691	0.0	0.0	0.0	0.0	0.0	0.0	0.0	0.0	0.0
LINC00654	0.0	0.0	0.0	0.0	0.0	0.0	0.0	0.0	0.0
CASQ1	0.0	0.0	0.0	0.0	0.0	0.0	0.0	0.0	0.0
SNORD20	0.0	0.0	0.0	0.0	0.0	0.0	0.0	0.0	0.0
LOC100506585	0.0	0.0	0.0	0.0	0.0	0.0	0.0	0.0	0.0
TBX21	0.0	0.0	0.0	0.0	0.0	0.0	0.0	0.0	0.0
PLEKHG1	0.0	0.0	0.0	0.0	0.0	0.0	0.0	0.0	0.0
COL5A3	0.0	0.0	0.0	0.0	0.0	0.0	0.0	0.0	0.0
LAMB4	0.0	0.0	0.0	0.0	0.0	0.0	0.0	0.0	0.0
LOC100130880	0.0	0.0	0.0	0.0	0.0	0.0	0.0	0.0	0.0
WDR87	0.0	0.0	0.0	0.0	0.0	0.0	0.0	0.0	0.0
LOC284240	0.0	0.0	0.0	0.0	0.0	0.0	0.0	0.0	0.0
C18orf12	0.0	0.0	0.0	0.0	0.0	0.0	0.0	0.0	0.0
DPRX	0.0	0.0	0.0	0.0	0.0	0.0	0.0	0.0	0.0
MAGEA12	0.0	0.0	0.0	0.0	0.0	0.0	0.0	0.0	0.0
SLC35F1	0.0	0.0	0.0	0.0	0.0	0.0	0.0	0.0	0.0
LINC00905	0.0	0.0	0.0	0.0	0.0	0.0	0.0	0.0	0.0
ZNF99	0.0	0.0	0.0	0.0	0.0	0.0	0.0	0.0	0.0
TRNAA29	0.0	0.0	0.0	0.0	0.0	0.0	0.4	0.0	0.8
SNORD50B	0.0	0.0	0.0	0.0	0.0	0.0	0.0	0.4	0.8
TRNAA5	0.0	0.0	0.0	0.0	0.0	0.0	0.4	0.0	0.4
TRNAQ17	0.0	0.0	0.0	0.0	0.0	0.0	0.4	0.0	0.4
TRNAS13	0.0	0.0	0.0	0.0	0.0	0.0	0.4	0.0	0.3
TRNAL23	0.0	0.0	0.0	0.0	0.0	0.0	0.4	0.0	0.3
SNORD18B	0.0	0.0	0.0	0.0	0.0	0.0	0.4	0.0	0.0
TRNAM8	0.0	0.0	0.0	0.0	0.0	0.0	0.2	0.2	0.0
TRNAM12	0.0	0.0	0.0	0.0	0.0	0.0	0.2	0.2	0.0
TRNAN15	0.0	0.0	0.0	0.0	0.0	0.0	0.2	0.2	0.0
TRNAN27	0.0	0.0	0.0	0.0	0.0	0.0	0.2	0.2	0.0
TRNAT15	0.0	0.0	0.0	0.0	0.0	0.0	0.2	0.0	0.1
TRNAT14	0.0	0.0	0.0	0.0	0.0	0.0	0.2	0.0	0.1

LOC101929613	0.0	0.0	0.0	0.0	0.0	0.0	0.1	0.0	0.1
PSORS1C3	0.0	0.0	0.0	0.0	0.0	0.0	0.1	0.0	0.0
HIST1H3G	0.0	0.0	0.0	0.0	0.0	0.0	0.1	0.0	0.1
ATP1B2	0.0	0.0	0.0	0.0	0.0	0.0	0.1	0.0	0.1
KRTAP4-11	0.0	0.0	0.0	0.0	0.0	0.0	0.1	0.0	0.0
GZMH	0.0	0.0	0.0	0.0	0.0	0.0	0.1	0.0	0.0
TRNAQ1	0.0	0.0	0.0	0.0	0.0	0.0	0.0	0.1	0.1
TRNAQ24	0.0	0.0	0.0	0.0	0.0	0.0	0.0	0.1	0.1
OR11H4	0.0	0.0	0.0	0.0	0.0	0.0	0.0	0.0	0.1
TRNAQ11	0.0	0.0	0.0	0.0	0.0	0.0	0.0	0.1	0.1
TRNAQ16	0.0	0.0	0.0	0.0	0.0	0.0	0.0	0.1	0.1
TRNAQ22	0.0	0.0	0.0	0.0	0.0	0.0	0.0	0.1	0.1
TAS2R39	0.0	0.0	0.0	0.0	0.0	0.0	0.1	0.0	0.0
LRRC25	0.0	0.0	0.0	0.0	0.0	0.0	0.1	0.0	0.0
IFNL3	0.0	0.0	0.0	0.0	0.0	0.0	0.1	0.0	0.0
OR7A10	0.0	0.0	0.0	0.0	0.0	0.0	0.0	0.0	0.0
LOC100505841	0.0	0.0	0.0	0.0	0.0	0.0	0.1	0.0	0.0
IGHV4-61	0.0	0.0	0.0	0.0	0.0	0.0	0.0	0.0	0.1
ACTR3C	0.0	0.0	0.0	0.0	0.0	0.0	0.1	0.0	0.0
PTAFR	0.0	0.0	0.0	0.0	0.0	0.0	0.0	0.0	0.1
BRWD1-AS1	0.0	0.0	0.0	0.0	0.0	0.0	0.0	0.0	0.0
LOC400499	0.0	0.0	0.0	0.0	0.0	0.0	0.0	0.0	0.1
CNR2	0.0	0.0	0.0	0.0	0.0	0.0	0.1	0.0	0.0
SIAH3	0.0	0.0	0.0	0.0	0.0	0.0	0.1	0.0	0.0
C1QB	0.0	0.0	0.0	0.0	0.0	0.0	0.0	0.0	0.0
LOC101928023	0.0	0.0	0.0	0.0	0.0	0.0	0.1	0.0	0.0
HBB	0.0	0.0	0.0	0.0	0.0	0.0	0.0	0.0	0.1
PRAP1	0.0	0.0	0.0	0.0	0.0	0.0	0.0	0.0	0.1
JAKMIP1	0.0	0.0	0.0	0.0	0.0	0.0	0.0	0.0	0.0
MRGPRX4	0.0	0.0	0.0	0.0	0.0	0.0	0.0	0.0	0.0
PRKCB	0.0	0.0	0.0	0.0	0.0	0.0	0.0	0.0	0.0
C5orf47	0.0	0.0	0.0	0.0	0.0	0.0	0.0	0.0	0.1
GNAS-AS1	0.0	0.0	0.0	0.0	0.0	0.0	0.0	0.0	0.0
KRT78	0.0	0.0	0.0	0.0	0.0	0.0	0.0	0.0	0.0
RTP3	0.0	0.0	0.0	0.0	0.0	0.0	0.0	0.0	0.0
IGKV1-16	0.0	0.0	0.0	0.0	0.0	0.0	0.0	0.0	0.0
PDZD4	0.0	0.0	0.0	0.0	0.0	0.0	0.0	0.0	0.0
SERPINA3	0.0	0.0	0.0	0.0	0.0	0.0	0.0	0.0	0.0
HSPB7	0.0	0.0	0.0	0.0	0.0	0.0	0.0	0.0	0.0
C2CD4C	0.0	0.0	0.0	0.0	0.0	0.0	0.0	0.0	0.0
FBXO47	0.0	0.0	0.0	0.0	0.0	0.0	0.0	0.0	0.0
IGF2BP1	0.0	0.0	0.0	0.0	0.0	0.0	0.0	0.0	0.0
ACSM1	0.0	0.0	0.0	0.0	0.0	0.0	0.0	0.0	0.0
ZNF454	0.0	0.0	0.0	0.0	0.0	0.0	0.0	0.0	0.0
LOC101927933	0.0	0.0	0.0	0.0	0.0	0.0	0.0	0.0	0.0
PLCB4	0.0	0.0	0.0	0.0	0.0	0.0	0.0	0.0	0.0
CLCA2	0.0	0.0	0.0	0.0	0.0	0.0	0.0	0.0	0.0
LOC100129603	0.0	0.0	0.0	0.0	0.0	0.0	0.0	0.0	0.0
THEG	0.0	0.0	0.0	0.0	0.0	0.0	0.0	0.0	0.0
LOC101929606	0.0	0.0	0.0	0.0	0.0	0.0	0.0	0.0	0.0
PALD1	0.0	0.0	0.0	0.0	0.0	0.0	0.0	0.0	0.0
ELTD1	0.0	0.0	0.0	0.0	0.0	0.0	0.0	0.0	0.0
KCNJ6	0.0	0.0	0.0	0.0	0.0	0.0	0.0	0.0	0.0
LOC101927911	0.0	0.0	0.0	0.0	0.0	0.0	0.0	0.0	0.0
LOC100652824	0.0	0.0	0.0	0.0	0.0	0.0	0.0	0.0	0.0

KIF5C	0.0	0.0	0.0	0.0	0.0	0.0	0.0	0.0	0.0
GRID2	0.0	0.0	0.0	0.0	0.0	0.0	0.0	0.0	0.0
LOC101927251	0.0	0.0	0.0	0.0	0.0	0.0	0.0	0.0	0.0
ST18	0.0	0.0	0.0	0.0	0.0	0.0	0.0	0.0	0.0
A2M-AS1	0.0	0.0	0.0	0.0	0.0	0.0	0.0	0.0	0.0
TRPM6	0.0	0.0	0.0	0.0	0.0	0.0	0.0	0.0	0.0
LINC00086	0.0	0.0	0.0	0.0	0.0	0.0	0.0	0.0	0.0
ACTN2	0.0	0.0	0.0	0.0	0.0	0.0	0.0	0.0	0.0
LOC101928352	0.0	0.0	0.0	0.0	0.0	0.0	0.0	0.0	0.0
PRDM16	0.0	0.0	0.0	0.0	0.0	0.0	0.0	0.0	0.0
SCAND3	0.0	0.0	0.0	0.0	0.0	0.0	0.0	0.0	0.0
LOC101927147	0.0	0.0	0.0	0.0	0.0	0.0	0.0	0.0	0.0
CR1	0.0	0.0	0.0	0.0	0.0	0.0	0.0	0.0	0.0
LOC101929050	0.0	0.0	0.0	0.0	0.0	0.0	0.0	0.0	0.0
LINC00278	0.0	0.0	0.0	0.0	0.0	0.0	0.0	0.0	0.0
GALNTL6	0.0	0.0	0.0	0.0	0.0	0.0	0.0	0.0	0.0
PRAMEF10	0.0	0.0	0.0	0.0	0.0	0.0	0.0	0.0	0.0
USP17L8	0.0	0.0	0.0	0.0	0.0	0.0	0.0	0.0	0.0
TRNT	0.0	0.0	0.0	0.0	0.5	1.3	0.0	0.0	0.0
SNORD82	0.0	0.0	0.0	0.0	0.0	0.4	0.0	0.0	0.0
APLN	0.0	0.0	0.0	0.0	0.0	0.1	0.0	0.0	0.0
FAM138F	0.0	0.0	0.0	0.1	0.1	0.0	0.0	0.0	0.0
FAM138A	0.0	0.0	0.0	0.1	0.1	0.0	0.0	0.0	0.0
LOC101927938	0.0	0.0	0.0	0.0	0.1	0.0	0.0	0.0	0.0
LOC101448202	0.0	0.0	0.0	0.0	0.0	0.0	0.0	0.0	0.0
LOC101928056	0.0	0.0	0.0	0.0	0.0	0.0	0.0	0.0	0.0
LOC101928674	0.0	0.0	0.0	0.0	0.1	0.1	0.0	0.0	0.0
ALPPL2	0.0	0.0	0.0	0.0	0.0	0.0	0.0	0.0	0.0
MPZ	0.0	0.0	0.0	0.1	0.0	0.0	0.0	0.0	0.0
C17orf74	0.0	0.0	0.0	0.0	0.0	0.0	0.0	0.0	0.0
NAP1L5	0.0	0.0	0.0	0.0	0.1	0.0	0.0	0.0	0.0
CDC20B	0.0	0.0	0.0	0.0	0.0	0.0	0.0	0.0	0.0
LAMP5	0.0	0.0	0.0	0.1	0.0	0.0	0.0	0.0	0.0
RHOXF2	0.0	0.0	0.0	0.0	0.0	0.0	0.0	0.0	0.0
TCL1B	0.0	0.0	0.0	0.0	0.1	0.0	0.0	0.0	0.0
KRT79	0.0	0.0	0.0	0.0	0.0	0.0	0.0	0.0	0.0
LOC392288	0.0	0.0	0.0	0.0	0.0	0.0	0.0	0.0	0.0
KRT16	0.0	0.0	0.0	0.0	0.0	0.0	0.0	0.0	0.0
AWAT2	0.0	0.0	0.0	0.0	0.0	0.0	0.0	0.0	0.0
LOC101927237	0.0	0.0	0.0	0.0	0.0	0.0	0.0	0.0	0.0
TRPC5	0.0	0.0	0.0	0.0	0.0	0.0	0.0	0.0	0.0
LRRC15	0.0	0.0	0.0	0.0	0.0	0.0	0.0	0.0	0.0
MAP3K19	0.0	0.0	0.0	0.0	0.0	0.0	0.0	0.0	0.0
ABCD2	0.0	0.0	0.0	0.0	0.0	0.0	0.0	0.0	0.0
DLGAP1	0.0	0.0	0.0	0.0	0.0	0.0	0.0	0.0	0.0
RNU6-10P	0.0	0.0	0.0	0.0	0.0	0.0	0.0	0.0	0.0
SLC35G3	0.0	0.0	0.0	0.0	0.0	0.0	0.0	0.0	0.0
KIF26A	0.0	0.0	0.0	0.0	0.0	0.0	0.0	0.0	0.0
LOC101927078	0.0	0.0	0.0	0.0	0.0	0.0	0.0	0.0	0.0
ADAMTS14	0.0	0.0	0.0	0.0	0.0	0.0	0.0	0.0	0.0
MIR205HG	0.0	0.0	0.0	0.1	0.0	0.0	0.0	0.0	0.0
AREGB	0.0	0.0	0.0	0.0	0.0	0.0	0.0	0.0	0.0
TRIM34	0.0	0.0	0.0	0.0	0.1	0.0	0.0	0.0	0.0
PIANP	0.0	0.0	0.0	0.0	0.0	0.0	0.0	0.0	0.0
MAGEA8	0.0	0.0	0.0	0.0	0.0	0.0	0.0	0.0	0.0

LRRC32	0.0	0.0	0.0	0.0	0.0	0.0	0.0	0.0	0.0
LILRA1	0.0	0.0	0.0	0.0	0.0	0.0	0.0	0.0	0.0
RNU6-42P	0.0	0.0	0.0	0.0	0.0	0.0	0.0	0.0	0.0
KCND3	0.0	0.0	0.0	0.0	0.0	0.0	0.0	0.0	0.0
LOC442497	0.0	0.0	0.0	0.0	0.0	0.0	0.0	0.0	0.0
SNORD38A	1.1	0.4	0.0	0.0	0.0	0.0	0.0	0.0	0.0
SNORD96A	0.6	0.4	0.0	0.0	0.0	0.0	0.0	0.0	0.0
TNIP3	0.3	0.0	0.3	0.0	0.0	0.0	0.0	0.0	0.0
LOC101928181	0.2	0.2	0.0	0.0	0.0	0.0	0.0	0.0	0.0
ACKR4	0.2	0.0	0.2	0.0	0.0	0.0	0.0	0.0	0.0
LOC101929175	0.3	0.0	0.1	0.0	0.0	0.0	0.0	0.0	0.0
TMEM220	0.1	0.0	0.1	0.0	0.0	0.0	0.0	0.0	0.0
KRTAP20-4	0.2	0.0	0.2	0.0	0.0	0.0	0.0	0.0	0.0
TMEM150C	0.3	0.1	0.0	0.0	0.0	0.0	0.0	0.0	0.0
LOC101927736	0.3	0.0	0.1	0.0	0.0	0.0	0.0	0.0	0.0
ATP6V1G3	0.2	0.0	0.1	0.0	0.0	0.0	0.0	0.0	0.0
LOC101928630	0.2	0.1	0.0	0.0	0.0	0.0	0.0	0.0	0.0
SPATA12	0.1	0.0	0.3	0.0	0.0	0.0	0.0	0.0	0.0
LOC729658	0.2	0.0	0.0	0.0	0.0	0.0	0.0	0.0	0.0
TEX38	0.1	0.0	0.1	0.0	0.0	0.0	0.0	0.0	0.0
LBH	0.3	0.0	0.0	0.0	0.0	0.0	0.0	0.0	0.0
IGLV4-69	0.3	0.0	0.1	0.0	0.0	0.0	0.0	0.0	0.0
LOC100127983	0.1	0.0	0.2	0.0	0.0	0.0	0.0	0.0	0.0
LOC101928387	0.2	0.0	0.1	0.0	0.0	0.0	0.0	0.0	0.0
SMIM1	0.0	0.0	0.2	0.0	0.0	0.0	0.0	0.0	0.0
LOC441461	0.1	0.0	0.0	0.0	0.0	0.0	0.0	0.0	0.0
SNORA41	0.0	0.0	0.0	0.0	0.0	0.0	0.0	0.0	0.0
MYCL	0.1	0.0	0.1	0.0	0.0	0.0	0.0	0.0	0.0
CYTIP	0.1	0.0	0.1	0.0	0.0	0.0	0.0	0.0	0.0
LOC100996654	0.0	0.1	0.0	0.0	0.0	0.0	0.0	0.0	0.0
GPC5-AS1	0.1	0.0	0.0	0.0	0.0	0.0	0.0	0.0	0.0
C11orf88	0.0	0.0	0.1	0.0	0.0	0.0	0.0	0.0	0.0
PRRG2	0.2	0.0	0.1	0.0	0.0	0.0	0.0	0.0	0.0
OR2B6	0.1	0.0	0.1	0.0	0.0	0.0	0.0	0.0	0.0
EFCAB9	0.0	0.0	0.1	0.0	0.0	0.0	0.0	0.0	0.0
FAM101B	0.0	0.0	0.1	0.0	0.0	0.0	0.0	0.0	0.0
LOC101926904	0.0	0.0	0.1	0.0	0.0	0.0	0.0	0.0	0.0
LOC101929015	0.1	0.0	0.1	0.0	0.0	0.0	0.0	0.0	0.0
LOC100653233	0.1	0.1	0.0	0.0	0.0	0.0	0.0	0.0	0.0
LOC101927268	0.0	0.0	0.1	0.0	0.0	0.0	0.0	0.0	0.0
IGHG1	0.0	0.0	0.0	0.0	0.0	0.0	0.0	0.0	0.0
ACER2	0.1	0.0	0.0	0.0	0.0	0.0	0.0	0.0	0.0
CCL5	0.2	0.0	0.1	0.0	0.0	0.0	0.0	0.0	0.0
LOC728586	0.0	0.0	0.1	0.0	0.0	0.0	0.0	0.0	0.0
PPP1R1B	0.1	0.0	0.0	0.0	0.0	0.0	0.0	0.0	0.0
COPZ2	0.0	0.0	0.1	0.0	0.0	0.0	0.0	0.0	0.0
MACROD2-AS1	0.1	0.0	0.0	0.0	0.0	0.0	0.0	0.0	0.0
LOC101927761	0.0	0.0	0.0	0.0	0.0	0.0	0.0	0.0	0.0
LOC101928784	0.1	0.0	0.0	0.0	0.0	0.0	0.0	0.0	0.0
LOC101927347	0.0	0.0	0.1	0.0	0.0	0.0	0.0	0.0	0.0
GHSR	0.1	0.0	0.0	0.0	0.0	0.0	0.0	0.0	0.0
STMN4	0.1	0.0	0.0	0.0	0.0	0.0	0.0	0.0	0.0
TNFRSF18	0.1	0.0	0.0	0.0	0.0	0.0	0.0	0.0	0.0
LOC101928282	0.1	0.0	0.0	0.0	0.0	0.0	0.0	0.0	0.0
LOC101928186	0.2	0.0	0.0	0.0	0.0	0.0	0.0	0.0	0.0

LOC199882	0.1	0.0	0.0	0.0	0.0	0.0	0.0	0.0	0.0
IGLV1-51	0.1	0.0	0.0	0.0	0.0	0.0	0.0	0.0	0.0
LOC101927193	0.0	0.0	0.1	0.0	0.0	0.0	0.0	0.0	0.0
RASGEF1C	0.1	0.1	0.0	0.0	0.0	0.0	0.0	0.0	0.0
KBTBD13	0.1	0.0	0.0	0.0	0.0	0.0	0.0	0.0	0.0
LIN7A	0.0	0.1	0.0	0.0	0.0	0.0	0.0	0.0	0.0
LYL1	0.1	0.0	0.0	0.0	0.0	0.0	0.0	0.0	0.0
RNF113B	0.1	0.0	0.0	0.0	0.0	0.0	0.0	0.0	0.0
LOC101927603	0.1	0.0	0.0	0.0	0.0	0.0	0.0	0.0	0.0
PTTG2	0.1	0.0	0.0	0.0	0.0	0.0	0.0	0.0	0.0
LOC101927256	0.0	0.2	0.0	0.0	0.0	0.0	0.0	0.0	0.0
TPH1	0.1	0.0	0.0	0.0	0.0	0.0	0.0	0.0	0.0
LOC100506725	0.1	0.0	0.0	0.0	0.0	0.0	0.0	0.0	0.0
BAAT	0.0	0.0	0.1	0.0	0.0	0.0	0.0	0.0	0.0
SLIT2-IT1	0.0	0.0	0.0	0.0	0.0	0.0	0.0	0.0	0.0
PLAGL1	0.1	0.0	0.0	0.0	0.0	0.0	0.0	0.0	0.0
ANKRD55	0.1	0.0	0.0	0.0	0.0	0.0	0.0	0.0	0.0
CEACAM8	0.0	0.0	0.0	0.0	0.0	0.0	0.0	0.0	0.0
P2RX6	0.0	0.0	0.0	0.0	0.0	0.0	0.0	0.0	0.0
GRAMD2	0.0	0.0	0.0	0.0	0.0	0.0	0.0	0.0	0.0
LOC349160	0.0	0.0	0.0	0.0	0.0	0.0	0.0	0.0	0.0
RASSF10	0.1	0.0	0.0	0.0	0.0	0.0	0.0	0.0	0.0
GPR4	0.0	0.0	0.0	0.0	0.0	0.0	0.0	0.0	0.0
IGHG2	0.0	0.0	0.1	0.0	0.0	0.0	0.0	0.0	0.0
OR2B11	0.0	0.0	0.0	0.0	0.0	0.0	0.0	0.0	0.0
HSD52	0.1	0.1	0.0	0.0	0.0	0.0	0.0	0.0	0.0
LINC00954	0.1	0.0	0.0	0.0	0.0	0.0	0.0	0.0	0.0
OR12D2	0.0	0.0	0.0	0.0	0.0	0.0	0.0	0.0	0.0
SLC15A2	0.0	0.0	0.0	0.0	0.0	0.0	0.0	0.0	0.0
LOC101929003	0.0	0.0	0.0	0.0	0.0	0.0	0.0	0.0	0.0
ADPRH	0.0	0.0	0.0	0.0	0.0	0.0	0.0	0.0	0.0
LOC101927520	0.0	0.0	0.1	0.0	0.0	0.0	0.0	0.0	0.0
CYP26A1	0.0	0.0	0.0	0.0	0.0	0.0	0.0	0.0	0.0
LOC402160	0.0	0.0	0.0	0.0	0.0	0.0	0.0	0.0	0.0
LRTM2	0.0	0.0	0.0	0.0	0.0	0.0	0.0	0.0	0.0
OR10A6	0.1	0.0	0.0	0.0	0.0	0.0	0.0	0.0	0.0
GYG2	0.0	0.0	0.0	0.0	0.0	0.0	0.0	0.0	0.0
APOA4	0.0	0.0	0.0	0.0	0.0	0.0	0.0	0.0	0.0
OR2AG1	0.0	0.0	0.0	0.0	0.0	0.0	0.0	0.0	0.0
N4BP3	0.0	0.0	0.0	0.0	0.0	0.0	0.0	0.0	0.0
SPDYC	0.0	0.0	0.0	0.0	0.0	0.0	0.0	0.0	0.0
IFIT1B	0.0	0.0	0.0	0.0	0.0	0.0	0.0	0.0	0.0
ST7-AS2	0.0	0.0	0.1	0.0	0.0	0.0	0.0	0.0	0.0
CRYM	0.0	0.0	0.0	0.0	0.0	0.0	0.0	0.0	0.0
LOC101927210	0.1	0.0	0.0	0.0	0.0	0.0	0.0	0.0	0.0
SERPINB9	0.0	0.0	0.0	0.0	0.0	0.0	0.0	0.0	0.0
GATM	0.0	0.0	0.0	0.0	0.0	0.0	0.0	0.0	0.0
FCAMR	0.0	0.0	0.0	0.0	0.0	0.0	0.0	0.0	0.0
LOC100132154	0.0	0.0	0.0	0.0	0.0	0.0	0.0	0.0	0.0
CPNE6	0.0	0.0	0.0	0.0	0.0	0.0	0.0	0.0	0.0
AQP2	0.0	0.0	0.0	0.0	0.0	0.0	0.0	0.0	0.0
LRRC14B	0.0	0.0	0.0	0.0	0.0	0.0	0.0	0.0	0.0
LOC101929350	0.0	0.0	0.0	0.0	0.0	0.0	0.0	0.0	0.0
SULT1C3	0.0	0.0	0.0	0.0	0.0	0.0	0.0	0.0	0.0
MUC13	0.0	0.0	0.0	0.0	0.0	0.0	0.0	0.0	0.0

MAGEA10	0.0	0.0	0.0	0.0	0.0	0.0	0.0	0.0	0.0
CRYBB3	0.0	0.0	0.0	0.0	0.0	0.0	0.0	0.0	0.0
SMIM17	0.0	0.0	0.0	0.0	0.0	0.0	0.0	0.0	0.0
LGALS7B	0.0	0.0	0.0	0.0	0.0	0.0	0.0	0.0	0.0
ACE	0.0	0.0	0.0	0.0	0.0	0.0	0.0	0.0	0.0
LOC101929237	0.0	0.0	0.0	0.0	0.0	0.0	0.0	0.0	0.0
SIGLEC7	0.1	0.0	0.0	0.0	0.0	0.0	0.0	0.0	0.0
LOC101927865	0.0	0.0	0.0	0.0	0.0	0.0	0.0	0.0	0.0
LINC00322	0.0	0.0	0.0	0.0	0.0	0.0	0.0	0.0	0.0
ESRP1	0.0	0.0	0.0	0.0	0.0	0.0	0.0	0.0	0.0
ESR1	0.0	0.0	0.0	0.0	0.0	0.0	0.0	0.0	0.0
MPEG1	0.0	0.0	0.0	0.0	0.0	0.0	0.0	0.0	0.0
SH2D4B	0.0	0.0	0.0	0.0	0.0	0.0	0.0	0.0	0.0
FAM81B	0.0	0.0	0.0	0.0	0.0	0.0	0.0	0.0	0.0
PNLIPRP3	0.0	0.0	0.0	0.0	0.0	0.0	0.0	0.0	0.0
LOC101929260	0.0	0.0	0.0	0.0	0.0	0.0	0.0	0.0	0.0
LCA5L	0.1	0.0	0.0	0.0	0.0	0.0	0.0	0.0	0.0
LOC100652930	0.0	0.0	0.0	0.0	0.0	0.0	0.0	0.0	0.0
LOC100506999	0.0	0.0	0.0	0.0	0.0	0.0	0.0	0.0	0.0
LOC100507003	0.0	0.0	0.0	0.0	0.0	0.0	0.0	0.0	0.0
DPP10	0.0	0.0	0.0	0.0	0.0	0.0	0.0	0.0	0.0
GAB3	0.0	0.0	0.0	0.0	0.0	0.0	0.0	0.0	0.0
SLC8A1	0.0	0.0	0.0	0.0	0.0	0.0	0.0	0.0	0.0
LOC101928751	0.0	0.0	0.0	0.0	0.0	0.0	0.0	0.0	0.0
VWA8-AS1	0.0	0.0	0.0	0.0	0.0	0.0	0.0	0.0	0.0
AQP7	0.0	0.0	0.0	0.0	0.0	0.0	0.0	0.0	0.0
LOC729770	0.0	0.0	0.0	0.0	0.0	0.0	0.0	0.0	0.0
TEKT5	0.0	0.0	0.0	0.0	0.0	0.0	0.0	0.0	0.0
HOXA-AS4	0.0	0.0	0.0	0.0	0.0	0.0	0.0	0.0	0.0
LOC284898	0.0	0.0	0.0	0.0	0.0	0.0	0.0	0.0	0.0
LOC101926962	0.0	0.0	0.0	0.0	0.0	0.0	0.0	0.0	0.0
LOC285766	0.0	0.0	0.0	0.0	0.0	0.0	0.0	0.0	0.0
LOC101927788	0.0	0.0	0.0	0.0	0.0	0.0	0.0	0.0	0.0
DLGAP3	0.0	0.0	0.0	0.0	0.0	0.0	0.0	0.0	0.0
LOC101929657	0.0	0.0	0.0	0.0	0.0	0.0	0.0	0.0	0.0
LOC100505474	0.0	0.0	0.0	0.0	0.0	0.0	0.0	0.0	0.0
LOC100996296	0.0	0.0	0.0	0.0	0.0	0.0	0.0	0.0	0.0
NAALADL1	0.0	0.0	0.0	0.0	0.0	0.0	0.0	0.0	0.0
CNBD1	0.0	0.0	0.0	0.0	0.0	0.0	0.0	0.0	0.0
MRVI1	0.0	0.0	0.0	0.0	0.0	0.0	0.0	0.0	0.0
LOC101927852	0.0	0.0	0.0	0.0	0.0	0.0	0.0	0.0	0.0
PTPN7	0.0	0.0	0.0	0.0	0.0	0.0	0.0	0.0	0.0
UNC5C	0.0	0.0	0.0	0.0	0.0	0.0	0.0	0.0	0.0
ABCA9	0.0	0.0	0.0	0.0	0.0	0.0	0.0	0.0	0.0
BIN2	0.0	0.0	0.0	0.0	0.0	0.0	0.0	0.0	0.0
COL11A1	0.0	0.0	0.0	0.0	0.0	0.0	0.0	0.0	0.0
TRIM64B	0.0	0.0	0.0	0.0	0.0	0.0	0.0	0.0	0.0
LOC101927437	0.0	0.0	0.0	0.0	0.0	0.0	0.0	0.0	0.0
ATP10B	0.0	0.0	0.0	0.0	0.0	0.0	0.0	0.0	0.0
CACNA1B	0.0	0.0	0.0	0.0	0.0	0.0	0.0	0.0	0.0
LGALS7	0.0	0.0	0.0	0.0	0.0	0.0	0.0	0.0	0.0
LOC101927846	0.0	0.0	0.0	0.0	0.0	0.0	0.0	0.0	0.0
USP17L11	0.0	0.0	0.0	0.0	0.0	0.0	0.0	0.0	0.0
SCARNA14	0.0	0.6	0.3	0.0	0.0	0.0	0.0	0.0	0.0
SNORD127	0.4	0.3	0.0	0.0	0.0	0.0	0.0	0.0	0.0

TRNAC7	0.8	0.0	0.1	0.0	0.0	0.0	0.0	0.0	0.0
SNORD71	0.5	0.0	0.4	0.0	0.0	0.0	0.0	0.0	0.0
SPINT4	0.6	0.0	0.1	0.0	0.0	0.0	0.0	0.0	0.0
LOC101929016	0.0	0.2	0.2	0.0	0.0	0.0	0.0	0.0	0.0
HIST1H2BF	0.3	0.0	0.1	0.0	0.0	0.0	0.0	0.0	0.0
SNORA70F	0.3	0.0	0.0	0.0	0.0	0.0	0.0	0.0	0.0
LOC101928981	0.4	0.0	0.0	0.0	0.0	0.0	0.0	0.0	0.0
TRNAK6	0.0	0.2	0.2	0.0	0.0	0.0	0.0	0.0	0.0
TRNAK13	0.0	0.2	0.2	0.0	0.0	0.0	0.0	0.0	0.0
PYDC1	0.3	0.0	0.1	0.0	0.0	0.0	0.0	0.0	0.0
HRASLS2	0.1	0.0	0.2	0.0	0.0	0.0	0.0	0.0	0.0
LOC101060024	0.3	0.0	0.0	0.0	0.0	0.0	0.0	0.0	0.0
LOC101929287	0.2	0.0	0.0	0.0	0.0	0.0	0.0	0.0	0.0
LOC101927413	0.2	0.0	0.0	0.0	0.0	0.0	0.0	0.0	0.0
C9orf24	0.1	0.0	0.1	0.0	0.0	0.0	0.0	0.0	0.0
LOC101927212	0.2	0.0	0.0	0.0	0.0	0.0	0.0	0.0	0.0
LOC101929566	0.1	0.0	0.1	0.0	0.0	0.0	0.0	0.0	0.0
LOC101928764	0.1	0.1	0.0	0.0	0.0	0.0	0.0	0.0	0.0
HIST1H2BI	0.2	0.0	0.1	0.0	0.0	0.0	0.0	0.0	0.0
CBLN3	0.2	0.0	0.0	0.0	0.0	0.0	0.0	0.0	0.0
LOC101927764	0.2	0.0	0.0	0.0	0.0	0.0	0.0	0.0	0.0
TAS2R7	0.2	0.0	0.1	0.0	0.0	0.0	0.0	0.0	0.0
BAALC	0.2	0.0	0.0	0.0	0.0	0.0	0.0	0.0	0.0
MEIG1	0.0	0.1	0.0	0.0	0.0	0.0	0.0	0.0	0.0
SRSF12	0.2	0.0	0.0	0.0	0.0	0.0	0.0	0.0	0.0
DUSP19	0.2	0.0	0.0	0.0	0.0	0.0	0.0	0.0	0.0
LOC400743	0.1	0.0	0.0	0.0	0.0	0.0	0.0	0.0	0.0
C11orf86	0.2	0.0	0.0	0.0	0.0	0.0	0.0	0.0	0.0
C13orf45	0.2	0.0	0.0	0.0	0.0	0.0	0.0	0.0	0.0
MRVI1-AS1	0.1	0.0	0.0	0.0	0.0	0.0	0.0	0.0	0.0
LOC101929322	0.1	0.0	0.1	0.0	0.0	0.0	0.0	0.0	0.0
TCF21	0.1	0.0	0.1	0.0	0.0	0.0	0.0	0.0	0.0
LOC391722	0.2	0.0	0.0	0.0	0.0	0.0	0.0	0.0	0.0
SNORA16B	0.0	0.0	0.2	0.0	0.0	0.0	0.0	0.0	0.0
LOC101928572	0.2	0.0	0.0	0.0	0.0	0.0	0.0	0.0	0.0
SNAP25	0.1	0.0	0.0	0.0	0.0	0.0	0.0	0.0	0.0
TAS2R38	0.1	0.0	0.1	0.0	0.0	0.0	0.0	0.0	0.0
PROP1	0.1	0.0	0.1	0.0	0.0	0.0	0.0	0.0	0.0
LOC100507537	0.1	0.0	0.0	0.0	0.0	0.0	0.0	0.0	0.0
HPGDS	0.1	0.0	0.0	0.0	0.0	0.0	0.0	0.0	0.0
LOC101060385	0.1	0.0	0.0	0.0	0.0	0.0	0.0	0.0	0.0
CDO1	0.1	0.1	0.0	0.0	0.0	0.0	0.0	0.0	0.0
IL31	0.1	0.0	0.1	0.0	0.0	0.0	0.0	0.0	0.0
LOC101928678	0.1	0.1	0.0	0.0	0.0	0.0	0.0	0.0	0.0
TRNAK9	0.0	0.1	0.1	0.0	0.0	0.0	0.0	0.0	0.0
TRNAK15	0.0	0.1	0.1	0.0	0.0	0.0	0.0	0.0	0.0
C9orf50	0.1	0.0	0.0	0.0	0.0	0.0	0.0	0.0	0.0
LOC100130370	0.0	0.0	0.1	0.0	0.0	0.0	0.0	0.0	0.0
GPR25	0.1	0.0	0.0	0.0	0.0	0.0	0.0	0.0	0.0
LOC101929154	0.1	0.0	0.0	0.0	0.0	0.0	0.0	0.0	0.0
METTL21C	0.0	0.0	0.0	0.0	0.0	0.0	0.0	0.0	0.0
PRB4	0.1	0.0	0.0	0.0	0.0	0.0	0.0	0.0	0.0
NAT8B	0.1	0.0	0.0	0.0	0.0	0.0	0.0	0.0	0.0
RNF133	0.1	0.0	0.0	0.0	0.0	0.0	0.0	0.0	0.0
P2RY12	0.1	0.0	0.0	0.0	0.0	0.0	0.0	0.0	0.0

LOC101928370	0.1	0.0	0.1	0.0	0.0	0.0	0.0	0.0	0.0
CALB1	0.0	0.0	0.0	0.0	0.0	0.0	0.0	0.0	0.0
WNT10A	0.1	0.0	0.1	0.0	0.0	0.0	0.0	0.0	0.0
MYLPF	0.1	0.0	0.0	0.0	0.0	0.0	0.0	0.0	0.0
BOLL	0.0	0.0	0.1	0.0	0.0	0.0	0.0	0.0	0.0
LOC101929726	0.1	0.0	0.0	0.0	0.0	0.0	0.0	0.0	0.0
HIST1H1D	0.1	0.0	0.0	0.0	0.0	0.0	0.0	0.0	0.0
CD4	0.1	0.0	0.0	0.0	0.0	0.0	0.0	0.0	0.0
LOC101928666	0.1	0.0	0.0	0.0	0.0	0.0	0.0	0.0	0.0
LOC101929588	0.1	0.0	0.0	0.0	0.0	0.0	0.0	0.0	0.0
GOLGA6L2	0.1	0.0	0.0	0.0	0.0	0.0	0.0	0.0	0.0
LOC101927093	0.0	0.0	0.0	0.0	0.0	0.0	0.0	0.0	0.0
KLHL10	0.1	0.0	0.1	0.0	0.0	0.0	0.0	0.0	0.0
CALR3	0.0	0.0	0.1	0.0	0.0	0.0	0.0	0.0	0.0
LOC375196	0.1	0.0	0.1	0.0	0.0	0.0	0.0	0.0	0.0
WNT1	0.1	0.0	0.0	0.0	0.0	0.0	0.0	0.0	0.0
PSG2	0.0	0.0	0.0	0.0	0.0	0.0	0.0	0.0	0.0
A4GNT	0.0	0.0	0.1	0.0	0.0	0.0	0.0	0.0	0.0
LINC00853	0.1	0.0	0.0	0.0	0.0	0.0	0.0	0.0	0.0
FAM228A	0.1	0.0	0.0	0.0	0.0	0.0	0.0	0.0	0.0
KLHL38	0.0	0.0	0.1	0.0	0.0	0.0	0.0	0.0	0.0
HAPLN1	0.0	0.0	0.1	0.0	0.0	0.0	0.0	0.0	0.0
C1orf195	0.0	0.0	0.1	0.0	0.0	0.0	0.0	0.0	0.0
MTNR1A	0.1	0.0	0.0	0.0	0.0	0.0	0.0	0.0	0.0
FCAR	0.1	0.0	0.0	0.0	0.0	0.0	0.0	0.0	0.0
LOC100505735	0.1	0.0	0.0	0.0	0.0	0.0	0.0	0.0	0.0
LINC00592	0.1	0.0	0.0	0.0	0.0	0.0	0.0	0.0	0.0
GP9	0.1	0.0	0.1	0.0	0.0	0.0	0.0	0.0	0.0
LINGO1	0.1	0.0	0.0	0.0	0.0	0.0	0.0	0.0	0.0
SMCR9	0.1	0.0	0.0	0.0	0.0	0.0	0.0	0.0	0.0
TSPAN11	0.1	0.0	0.0	0.0	0.0	0.0	0.0	0.0	0.0
FCGR1A	0.1	0.0	0.0	0.0	0.0	0.0	0.0	0.0	0.0
KRTAP5-8	0.1	0.0	0.0	0.0	0.0	0.0	0.0	0.0	0.0
BCL2L15	0.1	0.0	0.0	0.0	0.0	0.0	0.0	0.0	0.0
CA10	0.1	0.0	0.1	0.0	0.0	0.0	0.0	0.0	0.0
LINC00681	0.1	0.0	0.0	0.0	0.0	0.0	0.0	0.0	0.0
LINC00162	0.0	0.0	0.1	0.0	0.0	0.0	0.0	0.0	0.0
HAND2	0.0	0.0	0.0	0.0	0.0	0.0	0.0	0.0	0.0
TMEM151A	0.1	0.0	0.0	0.0	0.0	0.0	0.0	0.0	0.0
TSSK1B	0.1	0.0	0.0	0.0	0.0	0.0	0.0	0.0	0.0
CD164L2	0.1	0.0	0.0	0.0	0.0	0.0	0.0	0.0	0.0
FLJ40288	0.0	0.0	0.0	0.0	0.0	0.0	0.0	0.0	0.0
DUSP26	0.1	0.0	0.0	0.0	0.0	0.0	0.0	0.0	0.0
RGS11	0.1	0.0	0.0	0.0	0.0	0.0	0.0	0.0	0.0
PPEF1	0.1	0.0	0.0	0.0	0.0	0.0	0.0	0.0	0.0
LOC100996646	0.1	0.0	0.0	0.0	0.0	0.0	0.0	0.0	0.0
PDE6B	0.1	0.0	0.0	0.0	0.0	0.0	0.0	0.0	0.0
TRMT12	0.1	0.0	0.0	0.0	0.0	0.0	0.0	0.0	0.0
AMHR2	0.1	0.0	0.0	0.0	0.0	0.0	0.0	0.0	0.0
SNX31	0.0	0.0	0.1	0.0	0.0	0.0	0.0	0.0	0.0
HIST1H4L	0.0	0.0	0.0	0.0	0.0	0.0	0.0	0.0	0.0
FCER2	0.0	0.0	0.0	0.0	0.0	0.0	0.0	0.0	0.0
LOC101929725	0.0	0.0	0.0	0.0	0.0	0.0	0.0	0.0	0.0
PRLR	0.0	0.0	0.0	0.0	0.0	0.0	0.0	0.0	0.0
ANP32C	0.1	0.0	0.0	0.0	0.0	0.0	0.0	0.0	0.0

KCNK9	0.0	0.0	0.0	0.0	0.0	0.0	0.0	0.0	0.0
MAGEA1	0.0	0.0	0.0	0.0	0.0	0.0	0.0	0.0	0.0
LOC101927567	0.0	0.0	0.0	0.0	0.0	0.0	0.0	0.0	0.0
BIRC7	0.0	0.0	0.0	0.0	0.0	0.0	0.0	0.0	0.0
NOL4	0.0	0.0	0.0	0.0	0.0	0.0	0.0	0.0	0.0
LOC441239	0.0	0.0	0.0	0.0	0.0	0.0	0.0	0.0	0.0
OLFM3	0.0	0.0	0.0	0.0	0.0	0.0	0.0	0.0	0.0
KLHL3	0.0	0.0	0.0	0.0	0.0	0.0	0.0	0.0	0.0
LOC101929230	0.0	0.0	0.0	0.0	0.0	0.0	0.0	0.0	0.0
MT1M	0.0	0.0	0.0	0.0	0.0	0.0	0.0	0.0	0.0
LOC101928085	0.0	0.1	0.0	0.0	0.0	0.0	0.0	0.0	0.0
LOC101929577	0.1	0.0	0.0	0.0	0.0	0.0	0.0	0.0	0.0
C5orf52	0.0	0.0	0.1	0.0	0.0	0.0	0.0	0.0	0.0
LOC101928883	0.0	0.0	0.0	0.0	0.0	0.0	0.0	0.0	0.0
SPAG6	0.0	0.0	0.0	0.0	0.0	0.0	0.0	0.0	0.0
LOC101928646	0.0	0.0	0.0	0.0	0.0	0.0	0.0	0.0	0.0
LOC100506851	0.0	0.0	0.0	0.0	0.0	0.0	0.0	0.0	0.0
RGMA	0.0	0.0	0.0	0.0	0.0	0.0	0.0	0.0	0.0
SELENBP1	0.1	0.0	0.0	0.0	0.0	0.0	0.0	0.0	0.0
HTR3E	0.0	0.0	0.0	0.0	0.0	0.0	0.0	0.0	0.0
BPI	0.0	0.0	0.0	0.0	0.0	0.0	0.0	0.0	0.0
LINC00473	0.0	0.0	0.0	0.0	0.0	0.0	0.0	0.0	0.0
SYNDIG1L	0.0	0.0	0.0	0.0	0.0	0.0	0.0	0.0	0.0
PRPH	0.0	0.0	0.0	0.0	0.0	0.0	0.0	0.0	0.0
MFRP	0.0	0.0	0.0	0.0	0.0	0.0	0.0	0.0	0.0
AATK-AS1	0.0	0.0	0.0	0.0	0.0	0.0	0.0	0.0	0.0
SOAT2	0.0	0.0	0.0	0.0	0.0	0.0	0.0	0.0	0.0
EPB41L3	0.1	0.0	0.0	0.0	0.0	0.0	0.0	0.0	0.0
SLAMF8	0.0	0.0	0.0	0.0	0.0	0.0	0.0	0.0	0.0
PTGER3	0.0	0.0	0.0	0.0	0.0	0.0	0.0	0.0	0.0
BTN1A1	0.0	0.0	0.0	0.0	0.0	0.0	0.0	0.0	0.0
TRPC7	0.0	0.0	0.0	0.0	0.0	0.0	0.0	0.0	0.0
DCT	0.0	0.0	0.0	0.0	0.0	0.0	0.0	0.0	0.0
ASB15	0.0	0.0	0.0	0.0	0.0	0.0	0.0	0.0	0.0
LOC101928404	0.0	0.0	0.0	0.0	0.0	0.0	0.0	0.0	0.0
TBX22	0.0	0.0	0.0	0.0	0.0	0.0	0.0	0.0	0.0
PLCL1	0.0	0.0	0.0	0.0	0.0	0.0	0.0	0.0	0.0
RORC	0.0	0.0	0.0	0.0	0.0	0.0	0.0	0.0	0.0
OVOL1	0.1	0.0	0.0	0.0	0.0	0.0	0.0	0.0	0.0
FAM217A	0.0	0.0	0.0	0.0	0.0	0.0	0.0	0.0	0.0
SLC10A4	0.0	0.0	0.0	0.0	0.0	0.0	0.0	0.0	0.0
FAM189A2	0.0	0.0	0.0	0.0	0.0	0.0	0.0	0.0	0.0
C20orf141	0.0	0.0	0.1	0.0	0.0	0.0	0.0	0.0	0.0
SAMD3	0.0	0.0	0.0	0.0	0.0	0.0	0.0	0.0	0.0
KRT14	0.0	0.0	0.0	0.0	0.0	0.0	0.0	0.0	0.0
MYRIP	0.0	0.0	0.0	0.0	0.0	0.0	0.0	0.0	0.0
OR52W1	0.0	0.0	0.0	0.0	0.0	0.0	0.0	0.0	0.0
LDHC	0.0	0.0	0.0	0.0	0.0	0.0	0.0	0.0	0.0
FSD1	0.0	0.0	0.0	0.0	0.0	0.0	0.0	0.0	0.0
PICK1	0.0	0.0	0.0	0.0	0.0	0.0	0.0	0.0	0.0
VWA2	0.0	0.0	0.0	0.0	0.0	0.0	0.0	0.0	0.0
LRRC37A5P	0.1	0.0	0.0	0.0	0.0	0.0	0.0	0.0	0.0
GRIP2	0.0	0.0	0.0	0.0	0.0	0.0	0.0	0.0	0.0
SFTPD	0.1	0.0	0.0	0.0	0.0	0.0	0.0	0.0	0.0
DRD2	0.0	0.0	0.0	0.0	0.0	0.0	0.0	0.0	0.0

ETNPPL	0.0	0.0	0.0	0.0	0.0	0.0	0.0	0.0	0.0
ATP2B2	0.0	0.0	0.0	0.0	0.0	0.0	0.0	0.0	0.0
LOC101927048	0.0	0.0	0.0	0.0	0.0	0.0	0.0	0.0	0.0
KRTAP3-3	0.0	0.0	0.0	0.0	0.0	0.0	0.0	0.0	0.0
SLC7A14	0.0	0.0	0.0	0.0	0.0	0.0	0.0	0.0	0.0
ZNF812	0.0	0.0	0.0	0.0	0.0	0.0	0.0	0.0	0.0
LOC727982	0.0	0.0	0.0	0.0	0.0	0.0	0.0	0.0	0.0
LOC388553	0.0	0.0	0.0	0.0	0.0	0.0	0.0	0.0	0.0
SLC7A3	0.0	0.0	0.0	0.0	0.0	0.0	0.0	0.0	0.0
SV2A	0.0	0.0	0.0	0.0	0.0	0.0	0.0	0.0	0.0
LOC100505782	0.0	0.0	0.0	0.0	0.0	0.0	0.0	0.0	0.0
HCG23	0.0	0.0	0.0	0.0	0.0	0.0	0.0	0.0	0.0
GTF2A1L	0.0	0.0	0.0	0.0	0.0	0.0	0.0	0.0	0.0
PPIAL4E	0.0	0.0	0.0	0.0	0.0	0.0	0.0	0.0	0.0
WDR16	0.0	0.0	0.0	0.0	0.0	0.0	0.0	0.0	0.0
CUX2	0.0	0.0	0.0	0.0	0.0	0.0	0.0	0.0	0.0
ZNF711	0.0	0.0	0.0	0.0	0.0	0.0	0.0	0.0	0.0
DDX4	0.0	0.0	0.0	0.0	0.0	0.0	0.0	0.0	0.0
LOC101926942	0.0	0.0	0.0	0.0	0.0	0.0	0.0	0.0	0.0
PARVB	0.0	0.0	0.0	0.0	0.0	0.0	0.0	0.0	0.0
FCRL3	0.0	0.0	0.0	0.0	0.0	0.0	0.0	0.0	0.0
APOBR	0.0	0.0	0.0	0.0	0.0	0.0	0.0	0.0	0.0
LINC00974	0.0	0.0	0.0	0.0	0.0	0.0	0.0	0.0	0.0
LOC101929327	0.0	0.0	0.0	0.0	0.0	0.0	0.0	0.0	0.0
LOC101927715	0.0	0.0	0.0	0.0	0.0	0.0	0.0	0.0	0.0
TMEM200C	0.0	0.0	0.0	0.0	0.0	0.0	0.0	0.0	0.0
NR1H4	0.0	0.0	0.0	0.0	0.0	0.0	0.0	0.0	0.0
ZNF366	0.0	0.0	0.0	0.0	0.0	0.0	0.0	0.0	0.0
KRT5	0.0	0.0	0.0	0.0	0.0	0.0	0.0	0.0	0.0
ADAM28	0.0	0.0	0.0	0.0	0.0	0.0	0.0	0.0	0.0
ADAM23	0.0	0.0	0.0	0.0	0.0	0.0	0.0	0.0	0.0
ZNF578	0.0	0.0	0.0	0.0	0.0	0.0	0.0	0.0	0.0
ATP1A2	0.0	0.0	0.0	0.0	0.0	0.0	0.0	0.0	0.0
CPB2	0.0	0.0	0.0	0.0	0.0	0.0	0.0	0.0	0.0
ANO4	0.0	0.0	0.0	0.0	0.0	0.0	0.0	0.0	0.0
KIAA0319	0.0	0.0	0.0	0.0	0.0	0.0	0.0	0.0	0.0
HUNK	0.0	0.0	0.0	0.0	0.0	0.0	0.0	0.0	0.0
PLA1A	0.0	0.0	0.0	0.0	0.0	0.0	0.0	0.0	0.0
CDH16	0.0	0.0	0.0	0.0	0.0	0.0	0.0	0.0	0.0
LINC00293	0.0	0.0	0.0	0.0	0.0	0.0	0.0	0.0	0.0
LOC645949	0.0	0.0	0.0	0.0	0.0	0.0	0.0	0.0	0.0
PACSIN1	0.0	0.0	0.0	0.0	0.0	0.0	0.0	0.0	0.0
LPO	0.0	0.0	0.0	0.0	0.0	0.0	0.0	0.0	0.0
PRSS56	0.0	0.0	0.0	0.0	0.0	0.0	0.0	0.0	0.0
LINC00548	0.0	0.0	0.0	0.0	0.0	0.0	0.0	0.0	0.0
HEPHL1	0.0	0.0	0.0	0.0	0.0	0.0	0.0	0.0	0.0
DKFZp451B082	0.0	0.0	0.0	0.0	0.0	0.0	0.0	0.0	0.0
ANGPTL3	0.0	0.0	0.0	0.0	0.0	0.0	0.0	0.0	0.0
LRP3	0.0	0.0	0.0	0.0	0.0	0.0	0.0	0.0	0.0
SCN11A	0.0	0.0	0.0	0.0	0.0	0.0	0.0	0.0	0.0
SNORD111B	0.0	0.0	0.0	0.0	0.0	0.0	0.0	0.0	0.0
CAPN11	0.0	0.0	0.0	0.0	0.0	0.0	0.0	0.0	0.0
LOC100288255	0.0	0.0	0.0	0.0	0.0	0.0	0.0	0.0	0.0
LOC101929522	0.0	0.0	0.0	0.0	0.0	0.0	0.0	0.0	0.0
ASXL3	0.0	0.0	0.0	0.0	0.0	0.0	0.0	0.0	0.0

LOC101928549	0.0	0.0	0.0	0.0	0.0	0.0	0.0	0.0	0.0
CHGB	0.0	0.0	0.0	0.0	0.0	0.0	0.0	0.0	0.0
LAMC3	0.0	0.0	0.0	0.0	0.0	0.0	0.0	0.0	0.0
CNTNAP4	0.0	0.0	0.0	0.0	0.0	0.0	0.0	0.0	0.0
LOC101927544	0.0	0.0	0.0	0.0	0.0	0.0	0.0	0.0	0.0
FAM47E-STBD1	0.0	0.0	0.0	0.0	0.0	0.0	0.0	0.0	0.0
CSMD3	0.0	0.0	0.0	0.0	0.0	0.0	0.0	0.0	0.0
LOC101929184	0.0	0.0	0.0	0.0	0.0	0.0	0.0	0.0	0.0
LOC101929031	0.0	0.0	0.0	0.0	0.0	0.0	0.0	0.0	0.0
FRG2B	0.0	0.0	0.0	0.0	0.0	0.0	0.0	0.0	0.0
C8orf56	0.0	0.0	0.0	0.0	0.0	0.0	0.0	0.0	0.0
LRCOL1	0.0	0.0	0.0	0.0	0.0	0.0	0.0	0.0	0.0
UNC80	0.0	0.0	0.0	0.0	0.0	0.0	0.0	0.0	0.0
LINC00113	0.0	0.0	0.0	0.0	0.0	0.0	0.0	0.0	0.0
CACNA1G	0.0	0.0	0.0	0.0	0.0	0.0	0.0	0.0	0.0
PTPRZ1	0.0	0.0	0.0	0.0	0.0	0.0	0.0	0.0	0.0
NME9	0.0	0.0	0.0	0.0	0.0	0.0	0.0	0.0	0.0
SCN1A	0.0	0.0	0.0	0.0	0.0	0.0	0.0	0.0	0.0
PABPN1L	0.0	0.0	0.0	0.0	0.0	0.0	0.0	0.0	0.0
LOC283692	0.0	0.0	0.0	0.0	0.0	0.0	0.0	0.0	0.0
C1QTNF9	0.0	0.0	0.0	0.0	0.0	0.0	0.0	0.0	0.0
STH	0.0	0.0	0.0	0.0	0.1	0.1	0.0	0.0	0.0
LOC101927227	0.0	0.0	0.0	0.0	0.1	0.1	0.0	0.0	0.0
LOC101927289	0.0	0.0	0.0	0.1	0.0	0.1	0.0	0.0	0.0
LOC100652807	0.0	0.0	0.0	0.0	0.1	0.0	0.0	0.0	0.0
LOC101929021	0.0	0.0	0.0	0.0	0.1	0.0	0.0	0.0	0.0
INHBC	0.0	0.0	0.0	0.0	0.0	0.0	0.0	0.0	0.0
BTNL10	0.0	0.0	0.0	0.0	0.0	0.0	0.0	0.0	0.0
PROX1-AS1	0.0	0.0	0.0	0.0	0.0	0.0	0.0	0.0	0.0
MGC15885	0.0	0.0	0.0	0.0	0.0	0.0	0.0	0.0	0.0
FAM13C	0.0	0.0	0.0	0.0	0.0	0.0	0.0	0.0	0.0
PODN	0.0	0.0	0.0	0.0	0.0	0.0	0.0	0.0	0.0
LOC101928533	0.0	0.0	0.0	0.0	0.0	0.0	0.0	0.0	0.0
ADAMTS19	0.0	0.0	0.0	0.0	0.0	0.0	0.0	0.0	0.0
LOC101929022	0.0	0.0	0.0	0.0	0.0	0.0	0.0	0.0	0.0
LOC101929114	0.0	0.0	0.0	0.0	0.0	0.0	0.0	0.0	0.0
TMEM132D	0.0	0.0	0.0	0.0	0.0	0.0	0.0	0.0	0.0
CPXM1	0.0	0.0	0.0	0.0	0.0	0.0	0.0	0.0	0.0
CTRB2	0.0	0.0	0.0	0.0	0.0	0.0	0.0	0.0	0.0
SNORD45C	0.0	0.0	0.0	0.3	0.5	0.0	0.0	0.0	0.0
LOC101929553	0.0	0.0	0.0	0.0	0.2	0.0	0.0	0.0	0.0
TRBV30	0.0	0.0	0.0	0.1	0.1	0.0	0.0	0.0	0.0
LINC01006	0.0	0.0	0.0	0.0	0.0	0.0	0.0	0.0	0.0
FIGF	0.0	0.0	0.0	0.0	0.0	0.0	0.0	0.0	0.0
LOC100128364	0.0	0.0	0.0	0.0	0.0	0.0	0.0	0.0	0.0
TRO	0.0	0.0	0.0	0.0	0.0	0.0	0.0	0.0	0.0
LOC730256	0.0	0.0	0.0	0.0	0.0	0.0	0.0	0.0	0.0
LOC101929647	1.2	0.0	0.2	0.0	0.0	0.0	0.0	0.0	0.0
SCARNA27	0.0	0.0	0.4	0.0	0.0	0.0	0.0	0.0	0.0
LOC101929008	0.3	0.0	0.1	0.0	0.0	0.0	0.0	0.0	0.0
SNORA36B	0.3	0.0	0.0	0.0	0.0	0.0	0.0	0.0	0.0
SCGB1C1	0.2	0.1	0.0	0.0	0.0	0.0	0.0	0.0	0.0
LOC101928984	0.1	0.0	0.0	0.0	0.0	0.0	0.0	0.0	0.0
LOC100505478	0.2	0.0	0.0	0.0	0.0	0.0	0.0	0.0	0.0
LOC101929232	0.2	0.0	0.1	0.0	0.0	0.0	0.0	0.0	0.0

LOC729900	0.2	0.0	0.0	0.0	0.0	0.0	0.0	0.0	0.0
SIRPD	0.2	0.0	0.1	0.0	0.0	0.0	0.0	0.0	0.0
LOC100505946	0.0	0.1	0.1	0.0	0.0	0.0	0.0	0.0	0.0
TAC4	0.1	0.0	0.1	0.0	0.0	0.0	0.0	0.0	0.0
OSBPL10-AS1	0.1	0.0	0.0	0.0	0.0	0.0	0.0	0.0	0.0
METTL7B	0.1	0.1	0.0	0.0	0.0	0.0	0.0	0.0	0.0
KCNE3	0.1	0.1	0.0	0.0	0.0	0.0	0.0	0.0	0.0
LOC101928962	0.1	0.0	0.1	0.0	0.0	0.0	0.0	0.0	0.0
RTP4	0.0	0.0	0.1	0.0	0.0	0.0	0.0	0.0	0.0
LOC101928051	0.1	0.0	0.0	0.0	0.0	0.0	0.0	0.0	0.0
BCL2L10	0.1	0.0	0.0	0.0	0.0	0.0	0.0	0.0	0.0
LOC101928824	0.0	0.0	0.2	0.0	0.0	0.0	0.0	0.0	0.0
RMST	0.1	0.0	0.0	0.0	0.0	0.0	0.0	0.0	0.0
MEOX1	0.1	0.0	0.0	0.0	0.0	0.0	0.0	0.0	0.0
TREM1	0.1	0.0	0.0	0.0	0.0	0.0	0.0	0.0	0.0
OR51B5	0.1	0.0	0.0	0.0	0.0	0.0	0.0	0.0	0.0
C2orf73	0.0	0.0	0.0	0.0	0.0	0.0	0.0	0.0	0.0
LINC00264	0.1	0.0	0.0	0.0	0.0	0.0	0.0	0.0	0.0
FAM71A	0.1	0.0	0.0	0.0	0.0	0.0	0.0	0.0	0.0
PNMA5	0.1	0.0	0.0	0.0	0.0	0.0	0.0	0.0	0.0
ACER1	0.1	0.0	0.0	0.0	0.0	0.0	0.0	0.0	0.0
C5orf60	0.1	0.0	0.1	0.0	0.0	0.0	0.0	0.0	0.0
PPP4R4	0.1	0.0	0.0	0.0	0.0	0.0	0.0	0.0	0.0
SERPINI2	0.1	0.0	0.0	0.0	0.0	0.0	0.0	0.0	0.0
LOC101928199	0.0	0.0	0.0	0.0	0.0	0.0	0.0	0.0	0.0
LOC100507144	0.0	0.0	0.0	0.0	0.0	0.0	0.0	0.0	0.0
BOD1L2	0.0	0.1	0.0	0.0	0.0	0.0	0.0	0.0	0.0
IGKV1D-39	0.0	0.0	0.1	0.0	0.0	0.0	0.0	0.0	0.0
IGKV1-39	0.0	0.0	0.1	0.0	0.0	0.0	0.0	0.0	0.0
FEZ1	0.0	0.0	0.0	0.0	0.0	0.0	0.0	0.0	0.0
C1orf162	0.1	0.0	0.0	0.0	0.0	0.0	0.0	0.0	0.0
LHX5	0.1	0.0	0.0	0.0	0.0	0.0	0.0	0.0	0.0
LOC101929538	0.1	0.0	0.0	0.0	0.0	0.0	0.0	0.0	0.0
CDH26	0.1	0.0	0.0	0.0	0.0	0.0	0.0	0.0	0.0
LOC101929180	0.0	0.0	0.0	0.0	0.0	0.0	0.0	0.0	0.0
LOC389273	0.0	0.0	0.0	0.0	0.0	0.0	0.0	0.0	0.0
SLC36A2	0.1	0.0	0.0	0.0	0.0	0.0	0.0	0.0	0.0
ACADL	0.0	0.0	0.0	0.0	0.0	0.0	0.0	0.0	0.0
TCEB3CL	0.1	0.0	0.0	0.0	0.0	0.0	0.0	0.0	0.0
OR13J1	0.0	0.0	0.0	0.0	0.0	0.0	0.0	0.0	0.0
GUCA1A	0.1	0.0	0.0	0.0	0.0	0.0	0.0	0.0	0.0
LOC101927557	0.0	0.0	0.0	0.0	0.0	0.0	0.0	0.0	0.0
TCEB3CL2	0.0	0.0	0.0	0.0	0.0	0.0	0.0	0.0	0.0
FAM71D	0.0	0.0	0.0	0.0	0.0	0.0	0.0	0.0	0.0
LOC728084	0.0	0.0	0.0	0.0	0.0	0.0	0.0	0.0	0.0
JSRP1	0.0	0.0	0.0	0.0	0.0	0.0	0.0	0.0	0.0
HAVCR1	0.0	0.0	0.0	0.0	0.0	0.0	0.0	0.0	0.0
LOC101928124	0.0	0.0	0.0	0.0	0.0	0.0	0.0	0.0	0.0
FAM180A	0.0	0.0	0.1	0.0	0.0	0.0	0.0	0.0	0.0
TMPRSS11F	0.0	0.0	0.0	0.0	0.0	0.0	0.0	0.0	0.0
CDH5	0.0	0.0	0.0	0.0	0.0	0.0	0.0	0.0	0.0
SGSM1	0.0	0.0	0.0	0.0	0.0	0.0	0.0	0.0	0.0
LOC101928503	0.0	0.0	0.0	0.0	0.0	0.0	0.0	0.0	0.0
KRT84	0.0	0.0	0.0	0.0	0.0	0.0	0.0	0.0	0.0
TP53TG3C	0.0	0.0	0.0	0.0	0.0	0.0	0.0	0.0	0.0

STEAP1B	0.0	0.0	0.0	0.0	0.0	0.0	0.0	0.0	0.0
SYNPO2L	0.0	0.0	0.0	0.0	0.0	0.0	0.0	0.0	0.0
OTOL1	0.0	0.0	0.0	0.0	0.0	0.0	0.0	0.0	0.0
ENTPD1	0.0	0.0	0.0	0.0	0.0	0.0	0.0	0.0	0.0
PADI2	0.0	0.0	0.0	0.0	0.0	0.0	0.0	0.0	0.0
LOC101927700	0.0	0.0	0.0	0.0	0.0	0.0	0.0	0.0	0.0
C12orf42	0.0	0.0	0.0	0.0	0.0	0.0	0.0	0.0	0.0
TP53TG3B	0.0	0.0	0.0	0.0	0.0	0.0	0.0	0.0	0.0
TP53TG3D	0.0	0.0	0.0	0.0	0.0	0.0	0.0	0.0	0.0
PIK3R6	0.0	0.0	0.0	0.0	0.0	0.0	0.0	0.0	0.0
KIAA1211L	0.0	0.0	0.0	0.0	0.0	0.0	0.0	0.0	0.0
LOC729177	0.0	0.0	0.0	0.0	0.0	0.0	0.0	0.0	0.0
C20orf26	0.0	0.0	0.0	0.0	0.0	0.0	0.0	0.0	0.0
LOC100506175	0.0	0.0	0.0	0.0	0.0	0.0	0.0	0.0	0.0
LOC101927452	0.0	0.0	0.0	0.0	0.0	0.0	0.0	0.0	0.0
GPR82	0.0	0.0	0.0	0.0	0.0	0.0	0.0	0.0	0.0
TP53TG3	0.0	0.0	0.0	0.0	0.0	0.0	0.0	0.0	0.0
CASC1	0.0	0.0	0.0	0.0	0.0	0.0	0.0	0.0	0.0
SLC4A10	0.0	0.0	0.0	0.0	0.0	0.0	0.0	0.0	0.0
EIF1B-AS1	0.0	0.0	0.0	0.0	0.0	0.0	0.0	0.0	0.0
LINC00636	0.0	0.0	0.0	0.0	0.0	0.0	0.0	0.0	0.0
LOC100287477	0.0	0.0	0.0	0.0	0.0	0.0	0.0	0.0	0.0
COL2A1	0.0	0.0	0.0	0.0	0.0	0.0	0.0	0.0	0.0
EDAR	0.0	0.0	0.0	0.0	0.0	0.0	0.0	0.0	0.0
PVRL4	0.0	0.0	0.0	0.0	0.0	0.0	0.0	0.0	0.0
LOC100507657	0.0	0.0	0.0	0.0	0.0	0.0	0.0	0.0	0.0
ZIC3	0.0	0.0	0.0	0.0	0.0	0.0	0.0	0.0	0.0
STXBP5L	0.0	0.0	0.0	0.0	0.0	0.0	0.0	0.0	0.0
LOC100996369	0.0	0.0	0.0	0.0	0.0	0.0	0.0	0.0	0.0
RBP2	0.0	0.0	0.0	0.0	0.0	0.0	0.0	0.0	0.0
MYL2	0.0	0.0	0.0	0.0	0.0	0.0	0.0	0.0	0.0
LOC100287294	0.0	0.0	0.0	0.0	0.0	0.0	0.0	0.0	0.0
MUC19	0.0	0.0	0.0	0.0	0.0	0.0	0.0	0.0	0.0
USP17L13	0.0	0.0	0.0	0.0	0.0	0.0	0.0	0.0	0.0
TRNAR25	0.5	0.4	0.0	0.0	0.0	0.0	0.0	0.0	0.0
LOC101927103	0.7	0.0	0.1	0.0	0.0	0.0	0.0	0.0	0.0
SNORD4B	0.0	0.4	0.4	0.0	0.0	0.0	0.0	0.0	0.0
LOC101928217	0.4	0.0	0.0	0.0	0.0	0.0	0.0	0.0	0.0
RNU6-28P	0.0	0.4	0.0	0.0	0.0	0.0	0.0	0.0	0.0
SLURP1	0.3	0.1	0.0	0.0	0.0	0.0	0.0	0.0	0.0
LOC101928386	0.1	0.0	0.2	0.0	0.0	0.0	0.0	0.0	0.0
SNORA69	0.0	0.0	0.3	0.0	0.0	0.0	0.0	0.0	0.0
LOC101929223	0.2	0.0	0.1	0.0	0.0	0.0	0.0	0.0	0.0
PPP1R14A	0.2	0.0	0.1	0.0	0.0	0.0	0.0	0.0	0.0
TNFSF14	0.2	0.0	0.1	0.0	0.0	0.0	0.0	0.0	0.0
TP53AIP1	0.2	0.0	0.1	0.0	0.0	0.0	0.0	0.0	0.0
LOC101927777	0.0	0.1	0.2	0.0	0.0	0.0	0.0	0.0	0.0
PTH2	0.1	0.1	0.0	0.0	0.0	0.0	0.0	0.0	0.0
BEX1	0.2	0.0	0.0	0.0	0.0	0.0	0.0	0.0	0.0
FAM24A	0.1	0.0	0.1	0.0	0.0	0.0	0.0	0.0	0.0
LOC101929266	0.0	0.0	0.2	0.0	0.0	0.0	0.0	0.0	0.0
NME5	0.1	0.0	0.0	0.0	0.0	0.0	0.0	0.0	0.0
LOC101929285	0.0	0.0	0.2	0.0	0.0	0.0	0.0	0.0	0.0
FAM19A3	0.1	0.0	0.1	0.0	0.0	0.0	0.0	0.0	0.0
LOC101928609	0.1	0.0	0.1	0.0	0.0	0.0	0.0	0.0	0.0

LOC101928197	0.1	0.1	0.0	0.0	0.0	0.0	0.0	0.0	0.0
MLANA	0.2	0.0	0.0	0.0	0.0	0.0	0.0	0.0	0.0
TMEM247	0.1	0.0	0.0	0.0	0.0	0.0	0.0	0.0	0.0
ZNF705E	0.2	0.0	0.0	0.0	0.0	0.0	0.0	0.0	0.0
CRISP2	0.1	0.0	0.0	0.0	0.0	0.0	0.0	0.0	0.0
TMEM98	0.1	0.0	0.0	0.0	0.0	0.0	0.0	0.0	0.0
TRBV5-7	0.0	0.1	0.1	0.0	0.0	0.0	0.0	0.0	0.0
PROKR1	0.1	0.0	0.1	0.0	0.0	0.0	0.0	0.0	0.0
LOC101927942	0.1	0.0	0.0	0.0	0.0	0.0	0.0	0.0	0.0
C7orf65	0.1	0.1	0.0	0.0	0.0	0.0	0.0	0.0	0.0
FAM25HP	0.1	0.0	0.0	0.0	0.0	0.0	0.0	0.0	0.0
TNFRSF13B	0.1	0.0	0.0	0.0	0.0	0.0	0.0	0.0	0.0
USP50	0.1	0.0	0.0	0.0	0.0	0.0	0.0	0.0	0.0
LOC101929688	0.1	0.0	0.1	0.0	0.0	0.0	0.0	0.0	0.0
PI15	0.1	0.0	0.0	0.0	0.0	0.0	0.0	0.0	0.0
ATP5L2	0.1	0.0	0.0	0.0	0.0	0.0	0.0	0.0	0.0
LOC101929673	0.1	0.0	0.0	0.0	0.0	0.0	0.0	0.0	0.0
FAM83A	0.1	0.1	0.0	0.0	0.0	0.0	0.0	0.0	0.0
HIST1H2AL	0.0	0.1	0.1	0.0	0.0	0.0	0.0	0.0	0.0
HIST1H2AD	0.0	0.1	0.1	0.0	0.0	0.0	0.0	0.0	0.0
KCNK3	0.0	0.0	0.1	0.0	0.0	0.0	0.0	0.0	0.0
LOC101927882	0.1	0.0	0.1	0.0	0.0	0.0	0.0	0.0	0.0
LOC101927505	0.1	0.0	0.0	0.0	0.0	0.0	0.0	0.0	0.0
GNG8	0.0	0.1	0.0	0.0	0.0	0.0	0.0	0.0	0.0
ALLC	0.1	0.0	0.1	0.0	0.0	0.0	0.0	0.0	0.0
TRNAD9	0.1	0.0	0.0	0.0	0.0	0.0	0.0	0.0	0.0
LOC101928888	0.1	0.0	0.0	0.0	0.0	0.0	0.0	0.0	0.0
LOC101929596	0.0	0.0	0.1	0.0	0.0	0.0	0.0	0.0	0.0
CSDC2	0.0	0.0	0.1	0.0	0.0	0.0	0.0	0.0	0.0
TBPL2	0.1	0.0	0.0	0.0	0.0	0.0	0.0	0.0	0.0
C22orf24	0.1	0.0	0.0	0.0	0.0	0.0	0.0	0.0	0.0
SIX3-AS1	0.1	0.0	0.0	0.0	0.0	0.0	0.0	0.0	0.0
TIGIT	0.1	0.0	0.0	0.0	0.0	0.0	0.0	0.0	0.0
LOC646522	0.1	0.0	0.0	0.0	0.0	0.0	0.0	0.0	0.0
GCNT4	0.1	0.0	0.0	0.0	0.0	0.0	0.0	0.0	0.0
SYCE3	0.0	0.0	0.1	0.0	0.0	0.0	0.0	0.0	0.0
LOC101929107	0.1	0.0	0.0	0.0	0.0	0.0	0.0	0.0	0.0
HIST1H3E	0.1	0.0	0.0	0.0	0.0	0.0	0.0	0.0	0.0
BGN	0.0	0.1	0.0	0.0	0.0	0.0	0.0	0.0	0.0
LOC101927000	0.0	0.0	0.1	0.0	0.0	0.0	0.0	0.0	0.0
CCL17	0.0	0.0	0.1	0.0	0.0	0.0	0.0	0.0	0.0
LOC101928789	0.1	0.0	0.0	0.0	0.0	0.0	0.0	0.0	0.0
HBM	0.1	0.0	0.0	0.0	0.0	0.0	0.0	0.0	0.0
LOC100506498	0.1	0.0	0.0	0.0	0.0	0.0	0.0	0.0	0.0
ASB17	0.1	0.0	0.0	0.0	0.0	0.0	0.0	0.0	0.0
LHFPL3	0.1	0.0	0.0	0.0	0.0	0.0	0.0	0.0	0.0
LOC100506885	0.1	0.0	0.0	0.0	0.0	0.0	0.0	0.0	0.0
CCL3L1	0.0	0.0	0.0	0.0	0.0	0.0	0.0	0.0	0.0
CCL3L3	0.0	0.0	0.0	0.0	0.0	0.0	0.0	0.0	0.0
CASP5	0.1	0.0	0.0	0.0	0.0	0.0	0.0	0.0	0.0
IGKV2D-30	0.1	0.0	0.0	0.0	0.0	0.0	0.0	0.0	0.0
IGKV2-30	0.1	0.0	0.0	0.0	0.0	0.0	0.0	0.0	0.0
GAST	0.0	0.1	0.0	0.0	0.0	0.0	0.0	0.0	0.0
LOC101928472	0.0	0.0	0.1	0.0	0.0	0.0	0.0	0.0	0.0
CPN1	0.1	0.0	0.0	0.0	0.0	0.0	0.0	0.0	0.0

C20orf181	0.1	0.0	0.0	0.0	0.0	0.0	0.0	0.0	0.0
GPR84	0.1	0.0	0.0	0.0	0.0	0.0	0.0	0.0	0.0
TBX10	0.0	0.1	0.0	0.0	0.0	0.0	0.0	0.0	0.0
IFNK	0.0	0.0	0.1	0.0	0.0	0.0	0.0	0.0	0.0
MLC1	0.1	0.0	0.0	0.0	0.0	0.0	0.0	0.0	0.0
FAM26F	0.1	0.0	0.0	0.0	0.0	0.0	0.0	0.0	0.0
LOC101929309	0.0	0.0	0.0	0.0	0.0	0.0	0.0	0.0	0.0
SERPINB3	0.0	0.0	0.0	0.0	0.0	0.0	0.0	0.0	0.0
LOC101927568	0.0	0.0	0.0	0.0	0.0	0.0	0.0	0.0	0.0
MEP1B	0.1	0.0	0.0	0.0	0.0	0.0	0.0	0.0	0.0
TBC1D28	0.0	0.0	0.0	0.0	0.0	0.0	0.0	0.0	0.0
MAGEB17	0.0	0.0	0.0	0.0	0.0	0.0	0.0	0.0	0.0
C9orf106	0.0	0.0	0.0	0.0	0.0	0.0	0.0	0.0	0.0
TLX3	0.0	0.0	0.0	0.0	0.0	0.0	0.0	0.0	0.0
HAR1B	0.0	0.0	0.0	0.0	0.0	0.0	0.0	0.0	0.0
DAND5	0.1	0.0	0.0	0.0	0.0	0.0	0.0	0.0	0.0
RTN4RL1	0.0	0.0	0.0	0.0	0.0	0.0	0.0	0.0	0.0
LOC101928512	0.0	0.0	0.0	0.0	0.0	0.0	0.0	0.0	0.0
LINC00433	0.1	0.0	0.0	0.0	0.0	0.0	0.0	0.0	0.0
LOC101928944	0.1	0.0	0.0	0.0	0.0	0.0	0.0	0.0	0.0
KLRG2	0.0	0.0	0.0	0.0	0.0	0.0	0.0	0.0	0.0
PLSCR2	0.1	0.0	0.0	0.0	0.0	0.0	0.0	0.0	0.0
OLR1	0.0	0.1	0.0	0.0	0.0	0.0	0.0	0.0	0.0
LOC101929441	0.1	0.0	0.0	0.0	0.0	0.0	0.0	0.0	0.0
EDDM3B	0.0	0.0	0.1	0.0	0.0	0.0	0.0	0.0	0.0
LOC101928760	0.0	0.0	0.0	0.0	0.0	0.0	0.0	0.0	0.0
TMEM27	0.1	0.0	0.0	0.0	0.0	0.0	0.0	0.0	0.0
DKK4	0.1	0.0	0.0	0.0	0.0	0.0	0.0	0.0	0.0
PON1	0.0	0.0	0.0	0.0	0.0	0.0	0.0	0.0	0.0
PGK2	0.0	0.0	0.0	0.0	0.0	0.0	0.0	0.0	0.0
CLEC4F	0.0	0.0	0.0	0.0	0.0	0.0	0.0	0.0	0.0
LOC100505798	0.1	0.0	0.0	0.0	0.0	0.0	0.0	0.0	0.0
GPR150	0.0	0.0	0.0	0.0	0.0	0.0	0.0	0.0	0.0
MGC2848	0.0	0.0	0.0	0.0	0.0	0.0	0.0	0.0	0.0
ITGBL1	0.0	0.0	0.0	0.0	0.0	0.0	0.0	0.0	0.0
C1QTNF3	0.0	0.0	0.1	0.0	0.0	0.0	0.0	0.0	0.0
CLNK	0.0	0.0	0.0	0.0	0.0	0.0	0.0	0.0	0.0
PROM2	0.0	0.0	0.0	0.0	0.0	0.0	0.0	0.0	0.0
OR1L4	0.0	0.0	0.0	0.0	0.0	0.0	0.0	0.0	0.0
DNAJB8-AS1	0.0	0.0	0.0	0.0	0.0	0.0	0.0	0.0	0.0
GPR88	0.0	0.0	0.0	0.0	0.0	0.0	0.0	0.0	0.0
LOC101927488	0.0	0.0	0.1	0.0	0.0	0.0	0.0	0.0	0.0
UBASH3A	0.0	0.0	0.0	0.0	0.0	0.0	0.0	0.0	0.0
KRT31	0.0	0.0	0.0	0.0	0.0	0.0	0.0	0.0	0.0
SLCO2A1	0.0	0.0	0.0	0.0	0.0	0.0	0.0	0.0	0.0
OTOP2	0.0	0.0	0.0	0.0	0.0	0.0	0.0	0.0	0.0
GIPC2	0.1	0.0	0.0	0.0	0.0	0.0	0.0	0.0	0.0
PPEF2	0.0	0.0	0.0	0.0	0.0	0.0	0.0	0.0	0.0
DHRS9	0.0	0.0	0.0	0.0	0.0	0.0	0.0	0.0	0.0
LOC101928212	0.0	0.0	0.0	0.0	0.0	0.0	0.0	0.0	0.0
CLVS1	0.0	0.0	0.0	0.0	0.0	0.0	0.0	0.0	0.0
GCNT3	0.0	0.0	0.0	0.0	0.0	0.0	0.0	0.0	0.0
CHRM2	0.0	0.0	0.0	0.0	0.0	0.0	0.0	0.0	0.0
LOC101927781	0.0	0.0	0.0	0.0	0.0	0.0	0.0	0.0	0.0
P2RY6	0.0	0.0	0.0	0.0	0.0	0.0	0.0	0.0	0.0

ORM1	0.0	0.0	0.0	0.0	0.0	0.0	0.0	0.0	0.0
UNC5A	0.0	0.0	0.0	0.0	0.0	0.0	0.0	0.0	0.0
DMBX1	0.0	0.0	0.0	0.0	0.0	0.0	0.0	0.0	0.0
LOC101927958	0.0	0.0	0.0	0.0	0.0	0.0	0.0	0.0	0.0
SLC12A1	0.0	0.0	0.0	0.0	0.0	0.0	0.0	0.0	0.0
KIAA1045	0.0	0.0	0.0	0.0	0.0	0.0	0.0	0.0	0.0
CASC11	0.0	0.0	0.0	0.0	0.0	0.0	0.0	0.0	0.0
FLJ39080	0.0	0.0	0.0	0.0	0.0	0.0	0.0	0.0	0.0
C1orf172	0.0	0.0	0.0	0.0	0.0	0.0	0.0	0.0	0.0
WNT9B	0.0	0.0	0.0	0.0	0.0	0.0	0.0	0.0	0.0
LRRC63	0.0	0.0	0.0	0.0	0.0	0.0	0.0	0.0	0.0
ISPD-AS1	0.0	0.0	0.0	0.0	0.0	0.0	0.0	0.0	0.0
CLYBL-AS2	0.0	0.0	0.0	0.0	0.0	0.0	0.0	0.0	0.0
HTR4	0.0	0.0	0.0	0.0	0.0	0.0	0.0	0.0	0.0
TM4SF20	0.0	0.0	0.0	0.0	0.0	0.0	0.0	0.0	0.0
AIPL1	0.0	0.0	0.0	0.0	0.0	0.0	0.0	0.0	0.0
ITPRIPL1	0.0	0.0	0.0	0.0	0.0	0.0	0.0	0.0	0.0
CHODL	0.0	0.0	0.0	0.0	0.0	0.0	0.0	0.0	0.0
PRTN3	0.0	0.0	0.0	0.0	0.0	0.0	0.0	0.0	0.0
OTOP3	0.0	0.0	0.0	0.0	0.0	0.0	0.0	0.0	0.0
NOBOX	0.0	0.0	0.0	0.0	0.0	0.0	0.0	0.0	0.0
RBM46	0.0	0.0	0.0	0.0	0.0	0.0	0.0	0.0	0.0
UNC5D	0.0	0.0	0.0	0.0	0.0	0.0	0.0	0.0	0.0
ZNF135	0.0	0.0	0.0	0.0	0.0	0.0	0.0	0.0	0.0
ALOX15B	0.0	0.0	0.0	0.0	0.0	0.0	0.0	0.0	0.0
GUCY1A3	0.0	0.0	0.0	0.0	0.0	0.0	0.0	0.0	0.0
LOC101927366	0.0	0.0	0.0	0.0	0.0	0.0	0.0	0.0	0.0
CFI	0.0	0.0	0.0	0.0	0.0	0.0	0.0	0.0	0.0
GRM2	0.0	0.0	0.0	0.0	0.0	0.0	0.0	0.0	0.0
F7	0.0	0.0	0.0	0.0	0.0	0.0	0.0	0.0	0.0
ENPP5	0.0	0.0	0.0	0.0	0.0	0.0	0.0	0.0	0.0
LOC101927096	0.0	0.0	0.0	0.0	0.0	0.0	0.0	0.0	0.0
LOC101928272	0.0	0.0	0.0	0.0	0.0	0.0	0.0	0.0	0.0
GPD1	0.0	0.0	0.0	0.0	0.0	0.0	0.0	0.0	0.0
BAI1	0.0	0.0	0.0	0.0	0.0	0.0	0.0	0.0	0.0
LOC340090	0.0	0.0	0.0	0.0	0.0	0.0	0.0	0.0	0.0
TEX26-AS1	0.0	0.0	0.0	0.0	0.0	0.0	0.0	0.0	0.0
LOC100996609	0.0	0.0	0.0	0.0	0.0	0.0	0.0	0.0	0.0
LOC101928167	0.0	0.0	0.0	0.0	0.0	0.0	0.0	0.0	0.0
NLGN4X	0.0	0.0	0.0	0.0	0.0	0.0	0.0	0.0	0.0
KCNQ1DN	0.0	0.0	0.0	0.0	0.0	0.0	0.0	0.0	0.0
GLP1R	0.0	0.0	0.0	0.0	0.0	0.0	0.0	0.0	0.0
LOC100506319	0.0	0.0	0.0	0.0	0.0	0.0	0.0	0.0	0.0
SLC7A4	0.0	0.0	0.0	0.0	0.0	0.0	0.0	0.0	0.0
NMUR2	0.0	0.0	0.0	0.0	0.0	0.0	0.0	0.0	0.0
LOC400558	0.0	0.0	0.0	0.0	0.0	0.0	0.0	0.0	0.0
LOC101929413	0.0	0.0	0.0	0.0	0.0	0.0	0.0	0.0	0.0
CECR2	0.0	0.0	0.0	0.0	0.0	0.0	0.0	0.0	0.0
EPHB6	0.0	0.0	0.0	0.0	0.0	0.0	0.0	0.0	0.0
ADCY10	0.0	0.0	0.0	0.0	0.0	0.0	0.0	0.0	0.0
ISL2	0.0	0.0	0.0	0.0	0.0	0.0	0.0	0.0	0.0
DGKB	0.0	0.0	0.0	0.0	0.0	0.0	0.0	0.0	0.0
C1orf127	0.0	0.0	0.0	0.0	0.0	0.0	0.0	0.0	0.0
MAG	0.0	0.0	0.0	0.0	0.0	0.0	0.0	0.0	0.0
TSPYL5	0.0	0.0	0.0	0.0	0.0	0.0	0.0	0.0	0.0

VTCN1	0.0	0.0	0.0	0.0	0.0	0.0	0.0	0.0	0.0
CLCNKB	0.0	0.0	0.0	0.0	0.0	0.0	0.0	0.0	0.0
VRTN	0.0	0.0	0.0	0.0	0.0	0.0	0.0	0.0	0.0
RNU6-30P	0.0	0.0	0.0	0.0	0.0	0.0	0.0	0.0	0.0
FREM1	0.0	0.0	0.0	0.0	0.0	0.0	0.0	0.0	0.0
FAM41AY2	0.0	0.0	0.0	0.0	0.0	0.0	0.0	0.0	0.0
FAM41AY1	0.0	0.0	0.0	0.0	0.0	0.0	0.0	0.0	0.0
ADAMTS18	0.0	0.0	0.0	0.0	0.0	0.0	0.0	0.0	0.0
LOC101928346	0.0	0.0	0.0	0.0	0.0	0.0	0.0	0.0	0.0
LOC101926988	0.0	0.0	0.0	0.0	0.0	0.0	0.0	0.0	0.0
NLRP9	0.0	0.0	0.0	0.0	0.0	0.0	0.0	0.0	0.0
DLGAP2	0.0	0.0	0.0	0.0	0.0	0.0	0.0	0.0	0.0
CACNA1D	0.0	0.0	0.0	0.0	0.0	0.0	0.0	0.0	0.0
COX8C	0.0	0.0	0.0	0.0	0.0	0.0	0.0	0.0	0.0
LOC101929213	0.0	0.0	0.0	0.0	0.0	0.0	0.0	0.0	0.0
NPHS1	0.0	0.0	0.0	0.0	0.0	0.0	0.0	0.0	0.0
CCL7	0.0	0.0	0.0	0.0	0.0	0.0	0.0	0.0	0.0
PDZK1IP1	0.0	0.0	0.0	0.0	0.0	0.0	0.0	0.0	0.0
LOC101928049	0.0	0.0	0.0	0.0	0.0	0.0	0.0	0.0	0.0
CHRNA4	0.0	0.0	0.0	0.0	0.0	0.0	0.0	0.0	0.0
EVPLL	0.0	0.0	0.0	0.0	0.0	0.0	0.0	0.0	0.0
LOC101927062	0.0	0.0	0.0	0.0	0.0	0.0	0.0	0.0	0.0
PPP1R1A	0.0	0.0	0.0	0.0	0.0	0.0	0.0	0.0	0.0
HIST1H2BG	0.0	0.0	0.0	0.0	0.0	0.0	0.0	0.0	0.0
C11orf85	0.0	0.0	0.0	0.0	0.0	0.0	0.0	0.0	0.0
HCCAT4	0.0	0.0	0.0	0.0	0.0	0.0	0.0	0.0	0.0
LOC101927067	0.0	0.0	0.0	0.0	0.0	0.0	0.0	0.0	0.0
CACNA1F	0.0	0.0	0.0	0.0	0.0	0.0	0.0	0.0	0.0
LOC101929113	0.0	0.0	0.0	0.0	0.0	0.0	0.0	0.0	0.0
LOC128322	0.0	0.0	0.0	0.0	0.0	0.0	0.0	0.0	0.0
PABPC1L2A	0.0	0.0	0.0	0.0	0.0	0.0	0.0	0.0	0.0
PABPC1L2B	0.0	0.0	0.0	0.0	0.0	0.0	0.0	0.0	0.0
BPIFB2	0.0	0.0	0.0	0.0	0.0	0.0	0.0	0.0	0.0
PRODH2	0.0	0.0	0.0	0.0	0.0	0.0	0.0	0.0	0.0
LOC440243	0.0	0.0	0.0	0.0	0.0	0.0	0.0	0.0	0.0
PLEKHG7	0.0	0.0	0.0	0.0	0.0	0.0	0.0	0.0	0.0
T	0.0	0.0	0.0	0.0	0.0	0.0	0.0	0.0	0.0
SHISA6	0.0	0.0	0.0	0.0	0.0	0.0	0.0	0.0	0.0
KRT6A	0.0	0.0	0.0	0.0	0.0	0.0	0.0	0.0	0.0

Table S2B. RPKM of identified transcriptomes from CRL-1620 line.

Gene	CRL_D1	CRL_D2	CRL_D3	CRL_E1	CRL_E2	CRL_E3	CRL_P1	CRL_P2	CRL_P3
RNA45S5	46865.5	38713.0	66365.7	60013.0	61965.6	63135.0	59372.7	39903.3	67139.8
RN7SL1	41999.4	64955.1	9506.8	22655.7	18909.2	34168.4	50725.4	81272.1	9877.7
RN7SK	54021.9	47737.6	18470.6	24502.4	29617.8	7977.0	39195.8	53821.9	18269.9
RN7SL2	30931.1	52743.9	7836.5	16634.9	14039.3	20035.1	32610.5	54702.9	7833.7
RMRP	20659.7	28720.1	7190.0	7918.3	8330.8	3322.1	13472.9	24340.4	5045.7
RPPH1	4797.3	10318.3	1915.7	4484.1	4402.8	4386.2	7533.7	16583.6	2539.1
RNR1	873.1	326.3	882.2	3837.5	1625.1	1274.4	2197.9	1841.2	3915.3
ATP6	2984.4	3130.3	786.1	1612.4	1014.0	718.4	1398.4	3332.1	945.7
COX2	3232.8	3147.4	848.6	1637.3	1120.7	533.6	1159.7	2780.4	891.7
RNR2	700.1	746.8	631.8	3094.3	713.4	1103.9	2040.7	2912.4	2842.0
ACTB	2783.6	3223.7	956.9	1685.4	1781.7	829.9	952.0	2028.3	532.8
COX3	2410.2	2856.0	661.8	1175.7	795.0	488.1	1124.3	2993.7	829.6
COX1	1970.6	2611.3	533.7	1049.6	727.7	641.7	1050.2	3031.6	720.4
ND4L	1648.0	2110.4	486.1	990.3	647.7	283.7	895.1	2734.5	565.6
ND4	1801.7	1789.4	499.5	1016.9	669.8	454.7	850.9	2126.7	611.7
SPARC	2202.2	2597.4	712.9	663.5	649.0	422.0	558.5	1252.3	320.8
RNY3	613.8	1980.3	294.3	364.0	219.7	237.8	1713.3	3652.8	175.2
RNU2-2P	854.0	2436.9	225.5	368.3	230.4	952.6	822.1	2555.0	141.7
CYTB	1506.2	1622.6	420.5	885.8	589.2	338.3	693.4	1945.6	510.8
EEF1A1	1178.5	1952.8	302.6	880.3	630.8	715.1	597.1	1763.9	274.9
SNORD3A	1046.9	1999.7	376.2	332.9	346.4	1498.0	954.2	1327.3	197.9
ND1	1289.0	1376.0	353.6	836.9	568.6	351.0	718.6	1751.2	526.6
ATP8	1406.8	1150.5	323.1	732.6	480.4	538.8	618.9	1244.6	442.2
ND2	1110.5	1168.5	277.7	685.4	446.5	351.9	627.5	1462.9	439.8
RNU1-28P	548.4	2176.8	299.2	266.8	306.0	713.4	655.7	1275.5	186.8
RNU1-27P	548.4	2176.8	299.2	266.8	306.0	713.4	655.7	1275.5	186.8
RNVU1-18	548.4	2176.8	299.2	266.8	306.0	713.4	655.7	1275.5	186.8
RNU1-2	548.4	2176.8	299.2	266.8	306.0	713.4	655.7	1275.5	186.8
RNU1-4	548.4	2176.8	299.2	266.8	306.0	713.4	655.7	1275.5	186.8
RNU1-3	548.4	2176.8	299.2	266.8	306.0	713.4	655.7	1275.5	186.8
RNU1-1	548.4	2176.8	299.2	266.8	306.0	713.4	655.7	1275.5	186.8
RNVU1-9	543.4	2144.8	297.6	264.6	303.0	696.3	653.0	1268.6	185.9
RNVU1-7	543.4	2144.8	297.6	264.6	303.0	696.3	653.0	1268.6	185.9
S100A6	943.4	1199.0	317.6	650.6	473.7	306.7	638.5	1145.1	234.3
PLAT	780.1	1022.8	213.8	650.6	584.0	352.3	445.2	1176.1	221.4
KRT18	1039.0	1207.3	316.8	745.4	683.1	353.5	101.5	175.9	51.1
S100A4	634.7	827.8	172.2	552.5	406.0	327.1	411.4	877.6	148.5
HSPB1	793.0	837.9	296.4	412.4	446.0	232.8	331.8	617.1	189.8
PKM	584.8	748.3	183.4	393.1	392.3	259.8	397.4	934.5	210.9
ND5	788.4	804.9	207.2	471.5	310.0	217.5	317.1	746.6	216.8
RPL28	713.1	740.2	269.8	381.1	412.5	205.0	380.7	700.6	241.1
TMSB4X	615.8	1206.3	156.6	426.3	324.0	315.7	219.0	593.6	108.0
RNU105A	443.4	728.3	199.3	279.5	235.6	116.5	634.5	1102.0	156.9
VIM	620.2	828.7	227.1	247.8	253.0	143.4	309.5	687.1	179.4

RNU4-2	336.7	833.1	118.1	141.5	121.8	440.3	446.3	908.3	106.4
TMSB10	608.5	430.1	243.0	290.1	276.3	206.3	361.6	550.6	164.2
SNORA73A	354.6	729.9	138.4	215.1	166.5	224.0	416.7	710.5	94.2
PFN1	460.2	674.7	157.8	285.9	273.5	223.2	251.0	588.0	127.3
RPL37A	488.4	578.7	168.8	322.2	270.9	166.1	279.5	627.9	120.1
RNU4-1	314.5	743.1	103.8	138.4	107.7	352.1	362.3	768.5	88.9
FTH1	506.1	557.1	149.4	441.8	398.8	273.5	157.6	361.1	109.1
GAPDH	470.2	597.1	147.5	273.6	254.5	176.2	238.1	582.5	113.3
SCD	578.3	764.0	155.7	204.0	181.6	122.4	197.2	523.2	122.8
LGALS1	410.6	545.0	140.7	229.4	231.4	137.1	296.3	714.9	139.9
RPS11	457.2	403.5	141.8	250.7	233.1	202.0	307.7	539.4	156.7
ACTG1	429.9	492.6	139.7	374.2	370.6	194.2	185.3	398.0	98.1
RPL13A	372.6	575.2	115.7	252.4	203.7	259.2	240.6	554.4	108.5
ND3	515.4	357.6	118.2	276.6	184.9	180.7	319.6	513.7	198.0
ND6	516.6	460.0	137.0	321.2	202.3	192.9	224.4	448.1	142.0
MALAT1	454.2	632.0	90.0	208.1	126.8	218.0	158.5	496.2	83.9
RPS2	415.8	499.0	152.3	251.1	257.7	140.1	199.9	393.1	110.4
RPS16	364.8	494.8	126.4	215.8	182.9	149.2	251.0	509.9	113.1
RPL13	379.9	507.2	151.3	230.9	213.5	202.3	197.6	390.1	106.7
ALDOA	375.7	479.2	121.6	240.5	239.9	134.9	207.2	464.4	101.5
SQSTM1	416.3	536.7	116.9	230.3	211.0	156.8	185.1	410.4	90.7
EEF2	399.4	458.3	130.6	195.8	192.2	141.8	214.9	444.2	104.4
FADS2	440.1	487.5	132.5	137.3	136.2	69.1	229.2	500.6	124.5
FLNA	406.2	449.0	138.0	222.8	220.3	139.7	174.6	387.3	78.8
SNORA48	169.3	444.4	69.6	91.8	62.4	114.9	436.1	734.5	50.9
RPS19	301.7	447.2	100.0	196.1	161.9	183.6	193.4	421.7	78.7
PPIA	315.9	489.1	81.4	232.4	168.8	182.0	133.8	359.0	66.0
IFITM3	443.3	447.3	155.6	145.9	150.9	57.0	158.2	314.1	88.9
ACTN4	386.4	386.5	134.3	208.4	225.5	112.4	139.6	279.7	75.3
CD81	358.3	518.2	113.1	145.8	133.7	85.6	155.0	359.0	73.2
TUBB	301.0	377.8	87.6	260.9	213.2	143.6	126.8	341.9	59.8
RPS15	346.4	355.0	121.9	195.9	202.3	170.1	158.4	260.3	92.7
RPL10	281.9	406.2	77.7	162.6	129.7	181.0	167.5	395.6	71.9
BCYRN1	154.8	175.1	54.0	369.9	343.3	237.8	188.3	260.7	88.2
GNB2L1	321.6	424.6	95.0	173.9	156.2	121.4	142.3	331.8	68.5
RPL8	281.6	317.1	105.6	175.8	170.8	152.6	180.6	324.6	85.8
RPL23A	321.3	296.5	104.0	187.8	174.0	111.1	166.1	323.5	110.1
CD151	328.8	414.8	106.3	182.6	158.5	83.5	144.8	297.4	59.3
RPL18A	272.3	351.4	96.2	166.1	153.9	127.9	175.8	344.0	86.3
CFL1	290.7	353.5	87.6	149.1	145.3	89.7	174.9	405.8	75.6
RPL7A	283.9	382.3	83.5	165.9	138.3	116.9	147.1	368.5	66.0
RPL37	289.5	312.7	102.7	193.5	179.3	93.0	160.5	313.3	81.6
APP	255.8	297.2	79.8	202.8	170.9	121.8	153.4	366.5	75.6
RPLP2	270.2	340.9	88.7	153.6	127.9	117.3	180.9	356.5	67.2
RPS27	293.6	240.6	87.8	181.9	162.4	107.6	168.8	355.6	94.4
RPL35	267.6	329.8	91.2	182.7	178.1	136.1	136.0	291.8	78.1
GNAS	274.0	345.7	87.0	151.2	142.0	102.6	146.2	354.8	83.2

UBB	244.1	312.2	78.9	161.3	149.0	109.5	152.7	387.6	87.9
RPS3	248.5	412.3	80.9	144.0	127.0	168.2	126.5	309.6	56.5
RPS5	249.5	349.5	80.7	144.5	126.3	97.2	171.0	382.2	70.6
TIMP2	321.0	408.8	119.0	122.1	126.2	67.6	131.3	297.8	74.6
OAZ1	264.9	391.7	76.6	156.2	131.2	140.7	131.0	307.1	54.3
DCBLD2	319.9	407.6	93.1	111.8	98.7	74.0	127.2	345.1	75.3
RPL19	277.1	339.9	87.8	163.4	143.1	103.3	140.7	315.2	74.4
PTRF	288.2	306.6	110.8	228.7	240.9	114.1	106.4	181.4	56.6
CSTB	364.3	331.2	118.7	177.2	176.4	93.2	106.7	197.6	62.0
PODXL	401.8	385.8	133.4	127.8	134.8	65.6	88.6	198.3	52.0
RPLP0	245.4	355.5	71.9	152.1	124.0	127.8	130.6	319.5	58.8
RPL15	242.4	399.5	71.5	149.7	125.5	89.5	120.5	316.8	63.9
LOXL2	258.2	334.5	83.8	211.2	190.9	117.7	82.0	192.1	38.6
UBC	268.7	304.3	74.2	138.2	121.8	102.8	126.1	306.0	61.9
RPL32	243.5	322.6	81.8	154.8	142.4	111.4	110.0	261.2	67.2
LMNA	285.1	334.9	100.7	127.5	129.9	67.9	123.3	243.4	57.5
FSTL1	361.0	509.2	108.7	151.5	116.6	90.9	34.2	81.6	15.3
RPS28	261.1	237.5	96.7	156.4	151.7	74.2	148.7	246.6	80.9
RPLP1	215.6	309.4	74.8	158.7	120.2	113.1	137.4	268.2	53.5
UBA52	230.4	299.0	69.7	132.5	123.4	115.8	121.7	289.2	65.3
TAGLN2	237.6	341.4	75.0	114.4	99.6	104.1	117.7	257.9	49.7
HLA-C	231.1	252.4	85.2	102.7	103.4	265.4	107.6	195.2	51.1
S100A10	274.9	379.3	69.5	150.8	117.3	135.3	58.8	161.3	38.4
POLR2L	228.4	266.3	87.7	157.8	180.5	66.1	108.7	212.3	58.6
AES	232.9	271.5	68.3	135.7	118.3	67.4	120.9	289.4	57.1
MBOAT7	233.1	293.0	68.8	109.4	106.0	55.9	130.5	290.3	66.9
ITGA3	249.1	309.5	80.5	193.2	184.8	108.2	55.6	133.3	28.8
ENO1	208.9	259.2	56.1	195.8	157.1	147.5	82.7	200.5	34.6
DKK3	268.7	344.1	92.2	128.7	115.5	74.4	89.6	182.0	41.6
RPS18	208.3	229.6	66.2	140.3	108.4	139.5	134.1	256.0	50.3
EDF1	234.8	202.3	89.5	104.4	108.4	62.5	167.6	276.6	86.3
RNY1	185.6	237.0	111.7	106.4	89.3	44.2	230.4	258.9	60.0
ANXA2	204.2	315.9	58.7	171.3	142.7	117.6	67.7	193.0	36.7
EEF1G	199.9	273.5	57.3	116.3	100.8	112.0	105.1	268.8	48.6
RPL27A	205.9	193.8	82.6	134.7	128.4	101.3	136.4	213.1	70.7
CDKN1A	205.3	306.9	66.8	78.6	68.6	56.1	136.8	278.8	54.4
PSAP	178.6	249.2	50.7	97.8	81.9	67.4	128.1	333.7	57.6
RHOC	267.0	316.4	71.3	90.4	87.4	58.1	94.2	211.7	44.1
DKK1	265.4	428.8	93.3	85.7	81.3	63.9	54.0	121.9	30.2
FASN	246.9	302.9	83.7	110.1	109.6	60.7	81.7	187.9	39.8
B2M	164.5	353.6	48.3	76.2	65.3	172.2	76.6	215.8	39.1
TPM4	242.7	226.4	86.3	134.8	109.2	88.2	99.2	171.5	44.5
CTSB	228.7	303.2	69.8	110.5	92.9	74.0	79.3	186.2	39.8
COL1A2	187.2	272.3	53.7	31.4	24.8	21.8	155.4	373.9	58.4
RPS14	151.8	226.7	45.7	123.0	98.9	137.1	102.6	242.1	40.8
RPL27	174.2	205.9	64.0	112.5	121.1	87.5	104.3	221.9	58.1
PHPT1	171.3	219.9	57.2	99.6	95.1	56.5	126.5	249.4	66.0

BSG	181.7	242.4	60.5	78.2	81.0	60.0	120.3	258.4	55.6
RPL36	191.7	199.9	88.6	113.8	109.9	64.4	118.1	192.7	58.1
SNORA63	96.3	292.7	34.0	63.2	30.0	81.2	153.6	357.0	24.0
RPL23	182.1	246.1	58.2	106.2	86.4	95.5	91.6	216.7	43.7
S100A16	205.7	272.5	70.5	114.6	108.2	63.9	72.5	170.2	44.6
RPL26	181.0	243.0	53.7	118.1	94.5	80.6	84.9	215.2	50.4
TPT1	142.3	191.8	43.3	92.1	70.8	133.3	107.6	277.8	62.2
SCARNA2	104.4	136.8	24.7	82.7	64.4	323.2	145.8	207.3	27.4
CTSD	172.5	205.7	51.1	131.3	122.7	75.6	100.0	208.3	48.2
S100A11	197.7	264.6	53.2	96.9	87.5	54.1	77.4	212.0	44.9
FTL	186.6	234.4	53.3	127.8	118.3	89.4	66.8	159.5	39.7
RPL29	177.6	227.4	56.2	101.4	88.3	77.2	90.8	214.0	42.1
GDF15	296.5	323.0	87.3	39.1	36.8	16.7	85.6	142.6	45.4
TUBA1B	169.5	250.9	53.3	123.1	117.2	69.3	64.6	184.8	35.6
RPL3	169.9	226.5	51.1	93.4	79.7	109.9	85.2	207.6	37.5
CALR	153.7	206.7	49.2	134.0	117.5	88.4	77.6	171.7	41.7
TCEB2	162.4	237.8	59.9	85.4	83.9	37.4	107.2	210.5	55.7
DPYSL3	116.5	135.1	32.5	72.6	64.4	43.2	145.5	352.7	72.0
RPL38	178.3	136.9	61.5	109.8	92.4	69.1	115.2	216.6	53.4
MYL12A	202.6	320.8	57.8	105.8	85.6	84.1	41.5	105.0	19.9
CAPN2	180.8	215.5	53.6	112.8	103.2	77.7	74.7	163.6	36.1
CHCHD2	152.4	225.6	55.8	86.6	74.3	50.6	109.6	224.9	36.9
FAM129B	165.1	209.0	55.7	81.5	80.3	44.4	99.8	226.1	46.7
RTN4	196.2	247.8	60.1	90.8	78.1	67.9	73.6	155.8	35.8
RPS6	149.5	219.7	39.7	106.6	83.1	126.7	72.7	166.2	41.3
RPS8	151.4	222.5	47.7	88.4	72.8	98.2	79.7	204.2	36.0
ARHGDIA	201.4	241.9	66.3	74.0	70.7	53.4	81.2	168.7	39.2
RPS17L	170.1	184.4	69.1	107.8	114.6	77.5	72.6	137.7	57.2
HSP90AB1	158.1	212.2	39.8	90.0	72.7	83.2	79.4	217.3	36.9
PTMA	112.8	99.7	42.9	111.5	87.3	61.3	153.2	255.9	55.2
CCND1	166.2	223.7	55.5	101.6	109.0	49.4	68.3	151.8	42.6
PEA15	210.9	233.8	59.4	120.8	117.0	56.7	39.0	96.6	26.7
ARF1	160.9	169.7	58.9	65.3	69.0	43.4	114.1	221.2	57.3
RPS24	168.4	187.2	52.9	92.6	80.2	73.9	75.9	169.6	49.3
RPS21	113.6	235.6	37.8	89.3	70.6	111.0	81.7	178.5	29.1
GPX1	155.3	198.9	57.5	94.5	91.2	45.4	88.4	170.1	41.8
MYL6	162.6	147.0	51.1	127.4	114.4	70.7	80.2	143.6	36.8
PRNP	112.4	138.9	43.8	54.2	50.9	21.4	126.0	302.4	63.1
PHLDA1	162.3	149.2	54.4	50.0	51.2	26.3	127.3	229.2	62.8
GSTP1	159.7	191.8	50.6	92.0	88.0	52.3	73.3	160.3	41.5
SURF4	126.2	215.2	39.7	62.3	59.5	33.0	115.5	199.8	49.8
TUBB4B	148.0	181.7	52.9	117.2	122.8	66.2	54.2	123.9	30.6
YWHAG	170.5	176.1	57.1	83.9	87.5	39.3	73.4	150.8	43.8
AHNAK	179.0	252.2	61.0	59.3	56.8	64.8	41.2	137.7	20.2
HSPA8	131.8	178.7	33.8	89.8	76.2	70.6	60.3	189.1	38.4
RPS23	126.3	208.8	34.4	77.1	63.1	99.6	63.3	158.2	30.7
TNFRSF12A	160.2	231.7	63.3	95.5	99.3	48.7	52.6	89.8	19.5

UCHL1	112.4	202.7	28.9	90.2	67.1	65.3	71.9	191.0	29.9
CRIM1	156.8	227.0	50.5	126.1	110.6	79.4	26.5	67.4	13.5
OST4	151.6	185.2	61.8	91.7	87.7	34.1	67.8	130.0	47.0
GRN	160.6	194.5	47.1	78.3	72.9	44.7	72.2	145.8	32.0
PSMB4	128.4	199.9	35.1	88.2	76.9	56.1	62.8	163.6	31.7
CST3	137.0	181.2	49.9	62.6	65.3	35.2	87.3	181.3	41.6
JUND	159.5	162.5	72.3	66.1	73.2	60.3	74.4	129.4	42.4
NUPR1	161.2	172.9	48.0	58.3	55.0	34.8	81.1	192.5	33.5
GNAI2	156.4	184.7	49.5	62.1	61.9	40.1	77.6	167.5	35.6
ESRG	118.8	127.3	34.9	169.4	145.7	85.4	34.5	94.9	23.5
COX6A1	103.1	180.3	41.7	74.4	74.3	45.8	78.9	181.2	47.4
THBS2	169.6	208.1	54.0	22.6	22.0	12.8	86.4	210.0	41.2
LRRC17	186.5	328.5	48.8	80.3	62.5	56.7	13.3	38.4	8.0
FAU	124.5	158.9	38.3	68.3	56.3	96.5	82.2	167.0	30.8
CLIC1	148.5	229.9	44.6	66.0	56.0	46.7	54.8	144.9	29.8
HSPA5	117.7	152.2	30.0	51.1	42.3	44.1	92.2	245.9	44.7
TUBA1A	140.3	206.5	42.1	62.4	59.0	33.1	65.3	172.3	37.3
PTTG1IP	137.8	168.4	37.6	79.1	69.2	55.2	73.0	163.3	32.5
RPL31	130.6	144.3	41.9	99.5	68.5	90.4	72.4	137.3	29.1
LGALS3BP	138.1	176.4	42.9	61.4	58.8	35.5	78.1	178.6	38.7
MALL	163.7	186.5	58.7	40.6	41.9	20.7	86.3	168.2	41.5
MT2A	110.5	178.6	31.4	82.1	79.3	46.3	80.6	156.7	42.4
GPC1	175.5	206.0	58.7	76.1	74.1	40.6	51.6	96.3	23.8
P4HB	129.6	169.2	39.1	70.8	60.9	48.7	75.7	169.0	38.8
HLA-B	135.2	148.1	51.3	45.0	43.6	166.4	64.3	117.4	30.3
LDLR	162.8	200.3	50.2	41.2	39.7	25.1	66.7	180.2	35.0
AP2M1	134.6	185.0	40.6	75.5	64.5	54.0	63.6	152.1	30.5
BCL2L1	171.7	221.5	53.3	63.1	55.6	46.5	51.5	112.3	23.8
PTMS	122.4	129.5	35.7	79.8	65.3	33.2	103.0	191.9	38.1
RPS4X	115.0	161.0	34.0	74.0	67.5	91.5	58.5	157.1	38.7
KRT8	156.1	180.6	49.5	141.6	128.4	70.4	21.6	37.0	10.4
FKBP8	138.8	169.8	45.2	71.7	68.3	43.2	76.4	146.5	31.6
CD63	119.9	197.7	31.3	59.2	42.9	53.1	64.1	187.5	29.3
RAC1	144.8	178.1	43.7	73.7	64.8	47.7	56.4	137.1	32.8
RPL12	135.2	167.3	45.5	86.2	80.5	51.0	54.1	127.7	30.1
RPL4	126.9	160.8	37.1	74.6	66.2	63.4	62.4	149.0	34.2
RPL24	115.8	170.2	34.2	79.9	69.7	77.0	57.0	138.0	30.5
HIST1H2BK	112.9	134.4	43.7	113.0	114.3	61.1	53.6	105.9	32.4
MTSS1L	172.1	157.5	59.9	71.6	78.6	40.7	59.5	91.2	34.7
NDUFA3	136.4	104.2	61.9	70.9	82.6	31.0	87.9	126.1	59.9
BHLHE40	166.1	189.1	48.6	55.2	54.6	34.0	57.3	120.2	35.6
PRDX5	138.3	136.3	46.1	72.5	59.6	44.3	81.8	145.1	35.2
PCBP2	134.0	154.8	38.6	62.4	52.8	50.8	65.9	166.4	30.3
HLA-A	130.9	144.1	45.9	47.6	48.0	124.2	67.7	117.0	30.2
SNAR-A2	109.8	82.9	41.0	71.9	35.2	126.8	188.8	80.8	16.6
SNAR-A1	109.8	82.9	41.0	71.9	35.2	126.8	188.8	80.8	16.6
SERPINE2	123.8	209.4	38.5	74.5	65.6	50.9	45.0	117.1	24.3

44813	135.0	147.8	48.1	62.9	64.3	46.3	66.0	141.2	37.3
PLD3	125.1	141.0	39.8	62.1	61.6	36.1	71.7	163.7	38.3
RPL30	109.2	163.0	31.7	68.8	52.9	59.4	60.8	155.5	30.0
RPS15A	100.7	175.0	32.3	69.6	57.6	71.2	49.9	137.0	26.8
NFE2L1	134.5	165.7	41.4	53.0	50.5	29.5	57.7	156.7	30.0
RPL14	109.5	159.5	32.4	67.2	57.3	81.2	48.2	130.5	29.9
NDUFA13	116.8	164.5	37.4	64.6	59.7	36.7	64.6	143.7	26.4
SERPINE1	145.4	204.7	50.3	92.8	91.9	43.1	21.3	49.0	11.7
CAPNS1	125.8	153.5	42.8	71.1	67.2	41.5	56.5	118.9	31.8
HIST2H2BE	131.8	101.8	51.3	93.8	107.3	42.8	56.3	93.4	30.3
YWHAE	115.4	161.0	34.8	68.8	58.6	49.9	53.6	133.8	29.4
H3F3B	124.7	144.0	42.1	57.1	50.2	69.0	64.6	124.9	27.6
LASP1	147.4	163.8	49.0	70.7	68.4	42.2	41.4	96.4	24.2
CYB5B	105.0	145.4	25.9	39.1	35.2	32.6	81.0	208.1	30.0
PPDPF	144.5	125.2	59.1	62.1	69.0	28.8	60.5	113.2	36.5
DYNLRB1	140.5	92.6	44.5	68.1	73.1	48.6	72.9	116.6	38.7
ECM1	167.1	174.4	59.3	63.0	61.8	32.7	38.2	75.4	22.7
RPL6	108.2	156.4	29.1	64.4	50.0	76.8	46.1	134.5	28.2
SLC7A5	128.3	146.8	39.6	33.2	31.7	17.2	77.1	177.5	38.8
TKT	118.4	130.2	39.2	73.2	73.6	37.7	60.7	121.6	34.0
CCL2	59.9	89.0	15.4	83.2	76.9	30.1	69.3	208.2	53.7
UQCR11	127.6	129.1	48.4	70.3	68.1	27.1	68.8	110.2	34.8
ATP6V0C	112.1	159.7	38.7	56.5	59.8	40.3	56.6	129.1	29.6
CAPG	121.1	174.2	37.7	54.4	51.2	39.8	52.6	122.0	23.2
INF2	174.2	147.5	54.9	58.7	60.1	23.0	42.1	85.3	29.5
EIF4H	116.5	127.5	41.3	54.7	53.4	44.5	66.4	138.0	31.5
COL5A2	153.1	190.7	46.3	43.6	34.9	31.7	50.2	102.4	19.9
WBP2	143.1	138.9	45.8	57.2	54.2	31.8	53.7	117.2	28.9
RPS26	93.5	155.3	28.0	58.1	56.1	70.6	53.5	122.0	32.5
GNB2	105.6	139.0	35.2	56.7	54.7	42.5	61.8	138.3	29.3
KDELR2	105.1	122.5	27.9	44.9	37.6	24.5	69.7	190.3	40.7
NEAT1	122.4	162.5	28.3	77.8	42.5	43.1	48.3	114.2	21.3
RPS20	81.6	136.2	23.6	58.3	55.8	147.8	36.6	96.3	21.3
YWHAQ	112.0	152.8	36.1	66.9	57.2	36.6	51.0	117.4	27.0
MDH2	113.8	136.5	39.2	56.8	59.4	28.4	58.5	122.0	39.5
DBN1	118.5	121.1	46.6	64.8	65.1	34.3	58.5	115.0	29.2
SUCNR1	117.5	189.5	27.2	61.6	43.7	44.9	34.1	113.2	17.7
EIF5A	93.6	140.3	26.4	55.4	44.9	52.8	59.3	150.6	22.5
SOD1	92.2	139.6	30.8	68.0	55.5	49.5	54.6	126.4	27.9
RPS13	98.5	144.7	29.3	54.4	36.8	47.9	56.0	148.7	27.9
MARCKS	88.4	89.0	42.3	53.1	54.5	28.2	88.0	137.3	56.8
HCFC1R1	121.4	123.8	42.1	94.3	95.8	47.5	33.8	56.6	20.9
RPS9	85.3	127.2	27.2	55.5	46.6	103.3	55.0	112.5	22.3
ANXA1	116.9	188.3	29.2	50.5	37.8	39.9	39.4	113.4	19.3
PRKCSH	107.7	109.5	35.8	54.6	53.1	32.1	69.1	134.9	33.8
MRFAP1	124.8	103.4	41.0	63.0	58.6	36.7	73.7	95.5	31.9
COPE	94.3	123.1	31.0	44.3	41.3	29.4	75.5	160.2	29.2

RPL18	83.7	115.4	30.2	46.6	46.5	120.8	49.1	107.1	23.4
RCAN1	69.9	84.6	19.5	166.5	147.0	87.6	11.0	26.6	6.8
MIF	112.1	107.5	45.1	59.4	73.0	24.9	60.2	101.2	33.7
ACLY	116.8	145.7	34.5	57.7	52.3	40.0	41.8	105.8	20.4
GDI1	113.3	136.1	29.2	46.5	39.5	30.3	59.2	132.0	28.7
AKR1B1	18.0	25.6	5.1	211.5	162.2	123.4	16.0	40.6	7.1
MRPS6	44.7	66.9	14.1	106.7	84.5	63.0	54.2	141.5	32.2
CANX	89.9	122.8	23.6	52.3	42.1	41.4	52.4	152.0	27.3
L1CAM	114.5	132.5	37.1	71.0	64.0	38.1	39.7	87.1	17.8
COL3A1	109.9	150.5	30.4	33.3	25.1	24.1	62.2	143.3	21.9
MZT2B	100.0	124.2	38.3	69.3	64.0	30.2	52.1	103.2	18.3
MGAT4B	97.2	128.6	28.8	58.4	55.5	37.8	54.3	113.6	25.1
PCBP1	98.2	113.4	31.7	49.8	50.8	42.3	57.1	125.9	29.3
BRI3	123.7	138.6	40.8	65.3	61.2	34.4	35.7	79.5	18.6
TMEM259	105.9	135.7	33.6	46.1	42.9	32.9	55.7	114.2	30.3
DHCR7	119.3	142.0	36.9	48.4	45.9	26.5	46.4	103.6	25.4
GUK1	107.1	134.4	37.1	57.7	53.9	32.0	51.3	96.7	22.1
VAT1	119.4	145.8	39.7	51.8	50.2	34.0	41.2	86.8	17.5
PKN1	102.2	132.0	34.5	54.2	53.4	40.6	48.6	96.4	22.3
MYL12B	114.6	186.0	31.6	54.3	39.2	39.4	28.3	77.6	11.7
COX4I1	89.7	139.7	24.6	54.7	43.0	40.1	46.7	121.8	22.0
CD44	110.1	118.3	27.3	58.9	53.5	43.3	40.2	105.7	24.6
NPDC1	102.9	117.8	34.5	51.2	47.3	23.6	59.6	111.5	32.1
TRMT112	95.7	122.3	31.5	49.4	44.2	43.0	54.8	114.0	24.6
LDHA	86.4	129.3	21.8	59.8	46.7	48.3	41.5	122.4	22.1
RPS27A	73.6	126.9	19.0	55.2	34.2	88.0	46.2	115.5	19.3
TMED9	85.8	113.2	29.8	47.0	39.6	37.7	63.7	132.8	27.4
RPSA	79.4	142.6	22.6	65.8	40.1	52.5	43.2	112.8	17.2
RHOA	96.1	144.8	25.1	57.6	47.6	48.3	39.9	95.5	20.0
C19orf33	108.7	136.9	45.8	77.9	73.9	28.8	36.4	50.0	15.5
RPL10A	83.1	120.5	24.9	50.5	44.8	66.9	44.5	117.7	20.6
RPS7	75.3	133.5	23.8	62.9	46.9	72.4	38.2	100.2	20.2
ARPC1B	100.3	122.6	33.8	64.5	60.5	48.2	45.2	78.0	19.2
TUBA1C	95.9	133.9	28.2	62.7	55.5	29.8	40.9	104.9	18.6
COX8A	89.1	127.3	27.1	50.6	50.9	28.7	55.7	116.0	24.6
CD59	94.8	128.2	28.2	54.5	46.8	32.7	47.7	114.2	22.4
ZFP36L1	104.1	106.4	35.2	46.9	50.6	31.7	56.5	102.8	32.9
ARL4C	76.8	97.1	25.2	34.1	31.4	19.9	80.5	167.9	33.6
PGAM1	91.4	130.7	28.1	58.2	50.4	40.8	47.0	99.0	20.7
TECR	89.6	108.1	29.0	44.6	45.7	25.0	58.7	130.5	34.6
CUTA	95.3	128.6	30.0	55.5	49.6	33.5	42.3	106.5	23.4
ATP5I	101.9	86.0	38.7	56.8	47.6	27.9	71.4	108.7	25.3
CLU	103.3	126.7	31.4	43.4	38.6	25.8	52.7	113.2	28.6
NPM1	85.6	122.0	20.6	50.5	40.3	49.9	44.8	124.3	22.8
GPX4	100.5	118.5	32.5	50.2	46.2	28.3	52.8	104.1	27.7
EPN1	96.8	101.9	37.3	45.8	47.3	27.8	60.9	111.7	30.9
SNORD10	39.2	139.4	12.9	20.7	13.6	145.7	80.9	99.1	6.4

RPL9	70.3	110.1	19.8	55.3	38.3	68.2	48.4	128.9	17.9
CYB5R3	115.1	130.0	40.0	45.8	52.8	23.9	39.6	85.0	23.1
NOG	120.9	162.2	38.7	15.9	15.3	13.7	57.0	105.4	25.6
LAPTM4A	91.1	151.3	24.2	45.1	38.3	40.6	34.4	108.0	21.7
HDLBP	85.7	105.2	24.1	46.8	43.3	31.7	53.7	136.2	27.2
PTOV1	89.1	107.1	28.6	44.3	41.3	23.5	59.2	129.1	29.2
YBX1	99.8	83.1	35.2	54.7	54.1	44.0	53.7	93.2	33.2
SSR4	79.5	99.9	27.7	43.2	34.8	18.4	75.6	143.7	27.9
PHLDA2	99.0	98.6	39.7	33.3	42.0	18.9	61.5	108.5	47.8
WWTR1	68.7	78.1	21.2	24.0	25.3	12.0	74.5	197.7	47.3
COPG1	87.6	109.1	24.9	31.6	27.7	18.4	59.8	158.5	30.2
TERC	88.0	118.7	27.1	63.0	55.2	51.3	45.3	80.0	19.0
COX5B	82.7	120.8	30.7	48.8	44.4	21.1	53.4	123.0	22.6
FDPS	101.1	139.2	30.0	54.0	45.7	44.3	34.4	81.1	16.9
RPS3A	72.9	136.9	18.7	54.9	34.9	77.6	34.4	100.4	15.8
PRDX1	64.7	103.3	14.7	53.8	38.2	46.9	50.5	150.0	22.8
MAZ	91.3	94.2	34.1	57.3	54.9	28.1	51.4	98.8	27.0
PEBP1	96.1	121.6	33.3	50.3	45.8	24.0	44.6	96.8	23.1
CHPF	85.1	110.0	30.2	39.3	39.6	20.8	58.4	121.8	30.1
TRAPPC1	89.8	122.0	27.2	53.1	45.7	31.7	44.0	100.8	20.5
TIMP1	89.3	108.6	28.3	30.4	28.8	14.2	57.9	140.0	34.4
CTSZ	90.9	116.7	29.2	48.4	44.3	36.9	45.4	96.0	22.4
CTGF	150.4	218.7	48.3	35.4	34.7	19.1	6.5	12.4	3.9
GSN	85.5	110.4	25.9	49.2	43.5	26.5	51.9	113.2	22.8
PSMA7	78.7	125.4	24.0	49.4	36.7	35.0	45.8	114.5	18.0
TTYH3	75.6	93.9	24.6	40.2	38.8	23.6	65.0	136.9	28.9
RABAC1	69.5	110.3	22.8	40.8	36.4	31.5	58.6	128.2	27.3
EID1	84.7	94.1	28.5	44.4	44.4	24.1	62.2	110.7	31.5
EIF1	81.7	138.9	15.2	37.1	23.3	43.9	40.6	125.8	17.4
COL6A2	88.7	96.4	31.8	41.4	39.8	21.9	57.8	118.8	27.2
TAPBP	84.2	100.3	31.0	44.6	47.8	35.1	48.0	103.8	27.6
ATP6AP1	99.5	121.8	33.4	40.5	38.1	18.0	44.2	102.5	23.9
SSFA2	90.4	121.7	26.1	28.5	22.7	17.2	55.1	131.6	28.0
DBI	91.3	88.2	29.8	55.6	53.6	26.6	49.5	97.3	29.2
CCT3	81.3	114.2	21.6	48.4	37.5	34.5	43.4	115.0	20.2
PABPC1	83.5	114.4	23.9	58.3	53.0	52.7	30.0	80.9	19.3
PTX3	145.4	199.6	43.0	28.7	23.6	19.3	16.7	30.7	8.2
HIST2H2AA4	76.6	96.9	26.7	65.9	61.4	39.6	43.9	81.9	20.9
HIST2H2AA3	76.6	96.9	26.7	65.9	61.4	39.6	43.9	81.9	20.9
COX6B1	86.5	115.9	23.9	53.1	46.1	29.7	36.4	102.3	19.2
SLC9A3R1	100.3	110.6	34.1	40.1	40.5	30.1	46.6	83.7	22.6
MYLK	104.2	121.0	31.5	42.8	36.5	26.9	40.0	89.7	15.0
SERPINH1	86.2	100.4	28.7	24.7	25.4	11.9	64.9	134.8	29.8
CNBP	81.4	120.6	24.4	46.4	39.5	36.4	37.7	99.6	20.1
AXL	99.0	118.5	35.2	59.6	57.7	37.4	27.6	56.9	13.5
SERF2	91.9	101.4	27.9	40.6	40.3	26.5	51.3	102.5	22.0
SHC1	93.0	124.6	27.8	38.7	35.6	26.1	40.4	95.4	18.6

GLS	42.5	52.2	13.3	89.1	75.6	73.3	43.2	91.9	17.7
SNAR-A11	57.7	62.1	23.9	38.1	20.0	101.7	120.9	60.8	10.6
SNAR-A10	57.7	62.1	23.9	38.1	20.0	101.7	120.9	60.8	10.6
SNAR-A9	57.7	62.1	23.9	38.1	20.0	101.7	120.9	60.8	10.6
SNAR-A8	57.7	62.1	23.9	38.1	20.0	101.7	120.9	60.8	10.6
SNAR-A7	57.7	62.1	23.9	38.1	20.0	101.7	120.9	60.8	10.6
SNAR-A6	57.7	62.1	23.9	38.1	20.0	101.7	120.9	60.8	10.6
SNAR-A5	57.7	62.1	23.9	38.1	20.0	101.7	120.9	60.8	10.6
SNAR-A14	57.7	62.1	23.9	38.1	20.0	101.7	120.9	60.8	10.6
SNAR-A4	57.7	62.1	23.9	38.1	20.0	101.7	120.9	60.8	10.6
SNAR-A3	57.7	62.1	23.9	38.1	20.0	101.7	120.9	60.8	10.6
RPS17	85.0	92.2	34.5	53.9	57.3	38.8	36.3	68.8	28.6
SEC61A1	69.8	94.7	18.5	37.7	34.3	24.7	49.8	138.6	27.3
SH3BGRL3	80.9	122.1	31.0	40.1	43.4	35.5	36.1	85.7	19.7
IDS	77.7	110.8	21.8	33.9	32.8	22.1	42.6	125.8	25.6
CAV1	86.6	111.1	31.9	81.2	79.8	33.6	18.2	38.5	10.2
ATP5B	80.3	113.1	20.7	42.9	34.5	30.6	41.4	107.9	18.6
RPL7	74.9	103.6	18.5	46.1	35.3	63.6	35.4	94.4	17.4
LAMP1	98.8	109.9	30.7	48.5	44.3	28.2	34.6	75.0	18.9
EIF3G	86.0	86.1	29.6	41.8	43.0	27.5	50.7	97.5	24.7
MSN	85.0	112.0	26.6	42.5	36.9	37.5	37.0	86.9	18.7
FBXW5	79.6	95.9	24.0	46.1	46.0	26.8	46.9	91.4	21.3
RPL11	81.4	76.8	26.5	50.0	47.8	63.2	41.5	69.1	20.3
CERS2	81.4	119.8	22.5	42.9	36.4	29.9	33.8	92.0	16.9
NME1-NME2	71.2	87.3	24.8	46.1	43.9	34.1	45.0	98.7	23.8
BTF3	75.4	109.1	21.5	43.2	35.8	35.0	35.0	96.7	22.3
BCAP31	73.6	102.5	19.3	41.5	31.2	34.1	44.8	107.9	18.0
FDFT1	91.1	127.1	23.9	39.7	31.5	28.0	32.8	83.1	15.7
TRIM28	72.7	91.0	22.6	40.1	37.2	27.7	48.1	109.2	21.9
RNF187	84.8	97.9	28.3	30.8	24.6	15.8	57.0	108.0	22.5
RPS10	64.1	78.9	8.9	57.2	31.1	73.3	36.8	97.4	21.0
DPYSL2	72.1	89.6	21.2	33.7	30.7	22.4	48.4	126.7	23.9
MYADM	84.6	114.0	28.6	37.0	39.2	21.4	34.2	87.6	21.7
MYH9	90.3	86.0	31.2	46.5	44.9	41.1	35.5	76.1	16.8
MRPS24	73.6	85.8	26.8	63.8	61.9	38.1	33.4	61.2	20.3
RNASEK	82.1	102.9	24.1	46.5	46.6	29.2	35.2	73.2	24.5
RPL17	70.6	91.2	20.6	43.6	40.1	76.2	29.2	74.1	18.3
RAD23A	81.1	90.8	28.8	40.0	40.2	26.0	45.2	88.8	23.1
H1FX	92.1	71.4	37.2	42.6	41.0	26.9	52.9	75.4	23.6
QPRT	77.7	96.9	27.5	50.6	48.3	27.4	39.1	78.9	16.6
WDR83OS	70.1	111.5	21.6	36.7	37.4	19.3	44.7	100.1	21.3
FLNB	115.2	149.4	35.9	37.4	34.3	23.7	15.6	42.8	7.7
TRNF	36.0	65.2	28.1	59.0	18.0	13.4	100.5	111.5	29.7
FKBP1A	64.4	88.2	18.8	41.3	29.9	38.3	48.2	113.1	18.8
DUSP4	68.4	86.8	22.7	23.8	25.0	13.6	60.1	131.1	29.3
PPP6R1	78.5	83.8	24.6	36.9	35.2	23.1	50.0	103.6	23.7
BLVRB	77.7	100.3	24.4	50.4	47.0	26.2	38.0	75.1	20.3

PPP1R14B	76.4	89.0	31.7	44.0	42.4	28.4	42.3	83.7	21.6
DNAJB1	79.4	100.3	24.8	40.8	39.5	34.4	37.2	82.0	20.2
PPIB	71.7	95.2	21.2	40.4	31.3	30.1	44.8	104.7	18.5
TPI1	72.9	111.9	25.0	45.6	39.8	28.3	35.7	81.5	17.1
JUP	66.7	74.3	22.0	35.0	37.4	16.9	52.1	123.2	29.7
PRDX6	73.9	91.3	20.0	43.7	37.1	28.1	40.6	103.9	18.6
LBH	111.6	123.8	40.5	37.4	37.4	26.5	22.3	44.3	13.1
DRAP1	83.8	71.2	30.3	45.5	41.9	29.7	48.4	79.9	25.4
EIF4G2	68.1	100.5	17.3	34.6	30.3	33.5	35.2	115.5	20.9
NFIC	75.8	76.9	28.1	34.7	34.1	15.3	51.0	110.1	29.3
FAM96B	74.4	84.3	28.3	44.8	37.3	17.7	48.2	95.3	24.8
SEC13	65.8	87.4	22.0	30.0	26.7	14.4	58.2	127.8	22.7
ECH1	73.0	104.0	23.1	33.8	30.6	18.3	43.4	106.4	22.3
ARPC2	76.2	100.3	25.7	43.2	41.6	34.3	32.6	77.6	23.5
GPI	79.6	99.6	23.5	39.3	37.6	31.8	36.6	88.3	18.1
DYNLL1	59.5	108.4	20.3	51.3	44.0	44.5	29.4	81.9	14.9
VKORC1	73.0	116.0	22.2	35.5	30.6	31.7	35.7	91.7	17.4
LONP1	82.8	95.4	27.9	27.8	27.3	17.3	50.4	101.0	23.0
ATP5D	78.8	84.4	28.7	43.3	40.2	15.3	49.9	87.1	24.6
CTXN1	75.8	111.7	27.1	39.6	36.9	21.1	42.8	81.4	15.2
ITGB1	77.7	105.4	19.5	38.9	29.3	31.8	32.8	97.3	18.7
ATP5G2	74.7	99.5	24.5	40.0	42.1	16.5	38.5	94.8	20.6
HMGA1	52.5	61.7	18.9	39.4	36.3	29.5	59.4	124.5	26.5
PRMT1	73.2	93.2	23.0	37.3	35.5	18.8	47.3	99.4	21.0
LRP1	73.3	95.9	25.6	24.7	24.0	16.4	39.5	129.8	19.3
SYT11	78.2	101.1	22.6	23.2	19.5	15.3	46.2	116.9	22.9
EMP3	86.2	86.6	25.8	41.9	35.1	22.7	39.5	86.1	19.1
RNU5A-1	55.7	169.5	21.5	12.5	9.8	25.4	52.3	87.2	9.1
RPL35A	56.6	103.4	17.7	42.5	32.6	57.5	28.9	87.7	15.2
GNB1	72.9	99.5	23.4	35.0	34.6	28.0	37.8	93.5	17.5
EIF6	79.6	100.0	24.9	42.7	38.9	24.5	35.5	76.2	17.5
FADS3	111.4	122.9	32.8	47.6	45.6	18.8	16.5	34.0	9.9
SHISA5	81.0	86.6	31.6	35.2	35.1	20.4	41.3	90.4	17.7
MVP	88.4	100.0	27.6	31.2	29.3	21.1	40.2	81.0	18.6
KRT80	102.2	115.9	33.7	39.5	39.6	23.8	24.1	44.0	11.4
USP22	67.5	82.1	22.4	34.3	36.5	18.8	43.4	104.5	24.7
ENG	78.6	88.5	27.5	40.8	38.7	18.7	37.8	81.4	20.2
MFGE8	75.5	101.3	23.6	45.0	39.8	25.4	32.7	73.5	14.9
ANXA6	81.9	102.7	25.6	38.9	35.1	29.2	30.3	71.3	15.9
C19orf53	80.2	54.0	28.9	46.2	45.6	26.8	56.4	67.6	22.7
CALM2	65.5	112.6	18.3	47.8	31.1	36.5	25.4	78.3	12.6
ILK	70.5	102.1	21.1	33.8	26.9	24.3	37.7	93.4	18.0
LY6E	72.7	99.1	20.9	38.3	39.2	28.7	31.8	80.0	16.7
SNORA57	35.2	113.6	15.5	24.4	18.9	64.2	62.1	77.4	15.4
TGFB1	66.4	75.3	21.8	30.8	30.9	30.0	45.2	97.3	27.9
SET	70.6	92.0	20.8	44.2	34.1	34.3	32.1	78.4	17.4
RPS12	58.1	82.8	16.7	39.7	27.0	48.7	43.9	90.2	14.0

DAP	56.7	60.5	17.8	38.3	38.8	21.4	45.1	111.9	30.0
SNORA7B	51.3	101.9	20.1	33.7	21.1	35.0	55.3	86.9	14.5
GIPC1	81.4	89.6	27.0	45.6	42.5	31.7	30.7	57.8	12.9
RAB1B	69.9	86.6	21.8	34.5	34.3	22.8	39.4	87.7	21.5
PSMD2	64.7	89.3	19.7	44.2	37.0	31.7	35.1	82.8	14.1
SNAR-B2	57.8	58.4	24.3	44.4	25.1	17.1	101.4	75.8	14.2
SNAR-B1	57.8	58.4	24.3	44.4	25.1	17.1	101.4	75.8	14.2
CDK2AP1	61.9	77.8	22.6	44.2	39.6	23.1	44.1	80.9	22.9
UBE2S	69.0	85.2	25.3	53.7	49.6	27.1	27.7	64.3	15.0
NDUFS8	68.3	79.6	23.4	41.3	40.3	17.5	37.9	83.7	24.0
GRINA	76.8	80.2	23.8	39.3	37.5	18.8	35.2	82.1	21.6
IRAK1	67.2	84.8	21.8	42.0	42.5	25.6	35.0	78.5	18.0
CTSA	71.4	89.2	21.0	42.9	40.8	26.3	31.4	74.2	17.6
SSNA1	63.7	82.2	22.0	35.2	34.7	28.3	43.0	87.4	16.8
PRKAR1A	68.9	92.8	19.2	30.2	25.9	24.8	34.6	98.0	18.9
UGDH-AS1	70.2	131.5	16.6	16.8	13.5	36.8	17.9	98.3	11.7
LOXL1	87.8	104.7	36.0	34.5	33.7	15.9	28.2	53.6	16.3
PGK1	66.2	101.0	17.7	35.5	26.7	26.8	33.3	89.4	13.6
DHCR24	69.3	83.0	21.7	34.5	30.7	19.8	40.4	91.9	18.7
HNRNPH1	60.7	81.0	17.0	27.5	22.3	32.3	42.2	105.2	21.9
MRPL41	65.4	89.6	24.7	41.6	36.9	23.8	39.9	70.0	17.8
PSMB3	64.2	101.4	19.5	40.6	33.4	22.1	32.1	80.0	16.4
HIST1H1C	56.3	65.9	18.7	57.2	45.4	59.1	32.2	56.2	17.6
YWHAZ	77.9	107.6	18.8	37.8	30.8	31.6	23.0	66.8	14.0
G6PD	58.8	64.2	18.0	49.1	49.2	28.0	38.2	81.0	21.8
NDUFB4	75.7	70.6	27.1	37.9	39.1	21.9	37.8	69.1	28.6
SPTBN1	72.9	83.3	23.5	33.8	30.6	23.4	34.4	89.5	16.4
IER2	71.0	74.2	25.2	19.8	19.9	41.7	44.2	83.1	26.6
KIAA0754	83.4	103.4	28.6	29.3	27.2	19.1	26.2	72.0	15.5
CTNNA1	70.1	90.2	21.2	36.2	31.6	21.7	31.1	85.6	16.3
DDX5	61.3	94.8	14.7	32.2	26.1	41.8	28.4	89.1	15.0
LMAN2	72.7	88.9	24.3	35.3	31.9	20.9	36.6	72.5	20.1
WDR1	71.6	91.2	22.8	45.2	41.5	35.5	26.1	56.0	13.2
SUMO2	57.2	93.9	15.5	42.7	31.5	34.0	25.6	86.0	16.0
DPP7	78.9	90.5	25.5	40.5	39.6	21.5	32.1	57.8	14.5
INSIG1	80.1	114.3	23.0	33.4	32.5	19.3	26.4	55.6	15.6
GARS	71.3	99.9	19.9	27.0	24.3	18.7	30.6	88.9	19.4
HNRNPUL1	69.6	74.7	23.3	33.6	33.0	26.0	37.8	81.1	20.6
SYNGR2	61.2	85.4	21.7	36.0	37.6	19.7	35.8	84.9	17.5
C17orf76-AS1	60.6	85.9	16.9	35.8	26.7	23.7	36.9	94.1	18.7
TXN	55.6	90.9	13.9	48.2	37.6	32.2	25.5	81.6	12.4
COL6A1	50.5	56.2	17.8	38.3	37.8	19.8	50.7	101.9	25.1
HNRNPA2B1	66.5	80.7	20.5	40.4	33.3	35.6	31.9	71.0	16.5
PPME1	46.8	68.6	14.3	85.6	70.3	59.2	12.5	31.6	6.6
THY1	76.5	79.4	27.0	45.9	45.8	20.4	28.4	57.8	13.7
COLGALT1	75.7	94.0	27.0	31.1	30.3	16.6	33.6	69.5	16.5
EIF3F	75.8	95.7	25.5	33.5	35.0	23.0	27.1	59.6	16.1

EIF4G1	64.4	75.2	20.0	36.7	35.1	25.2	33.5	83.4	17.4
PPP2R1A	67.7	90.0	21.3	33.0	29.9	23.5	34.0	76.2	15.1
ZYX	66.4	69.0	22.4	36.2	36.8	24.5	37.6	77.4	20.2
HIST2H2AC	53.1	62.1	18.3	41.7	44.8	65.9	33.2	57.1	14.1
SNRPD2	65.8	87.4	20.3	35.4	34.8	19.1	33.8	73.2	19.7
CITED2	104.7	101.0	36.8	37.8	35.5	23.2	15.4	25.0	9.7
PSMD8	65.5	80.2	21.8	34.3	30.4	20.3	36.3	79.2	20.6
HSP90AA1	64.5	83.2	17.3	44.7	34.9	34.5	25.7	69.8	14.0
TNNT1	66.5	67.0	25.8	45.8	43.4	26.4	35.9	61.2	15.9
PLEC	71.5	77.4	26.6	30.2	29.7	21.4	34.2	81.1	15.6
PLXND1	76.9	84.1	25.0	30.9	31.6	16.5	33.2	71.0	17.9
ERGIC1	74.5	68.2	24.3	39.2	43.1	23.5	30.6	64.0	19.6
PSMB7	63.5	85.5	19.0	37.7	32.0	30.4	29.5	75.2	14.1
TUBB3	68.5	83.9	23.5	34.5	35.4	16.8	33.3	74.1	16.4
NONO	63.2	72.3	17.5	35.8	29.4	28.5	37.0	86.0	16.7
GABARAP	57.7	95.8	13.7	32.6	28.2	27.9	27.8	83.2	18.4
TUBB6	63.8	82.4	25.4	59.8	63.6	35.5	14.7	32.1	8.1
IGFBP7	79.2	127.4	28.4	31.7	25.0	16.2	24.1	43.1	9.7
CCT7	55.1	95.1	16.7	31.7	32.9	25.8	30.0	82.1	13.9
UQCRQ	52.5	103.7	16.4	37.0	29.6	17.6	33.7	78.4	14.4
RNU5E-1	65.0	151.8	30.5	20.1	15.6	14.6	36.9	38.9	9.7
MLLT11	64.0	101.7	16.4	35.5	31.9	26.4	22.5	68.4	15.4
EHD2	76.5	103.5	26.9	35.3	34.5	19.1	24.4	49.5	12.4
MAP1B	67.2	83.3	20.9	33.9	31.4	22.4	24.9	80.6	13.8
CAP1	50.5	68.6	15.0	23.1	20.5	21.6	41.0	115.2	22.0
MCL1	69.4	89.7	22.4	24.8	22.3	43.9	29.9	60.6	14.0
RCN1	57.6	76.6	17.4	30.7	25.0	23.7	36.8	89.9	19.4
MYO1C	61.7	75.4	19.5	44.4	41.5	29.6	27.1	63.8	12.6
FABP3	119.5	141.6	38.3	15.0	14.6	7.2	12.5	20.0	6.1
SKP1	59.9	74.5	16.2	35.2	29.4	20.8	35.2	86.6	16.8
SLC25A39	60.1	72.6	20.1	34.6	30.3	17.1	38.9	80.5	20.1
PNPLA6	59.4	72.4	18.6	38.3	36.0	24.6	35.2	72.5	16.9
CRTAP	68.3	93.4	20.3	33.2	28.4	19.7	27.7	66.7	15.8
TM4SF1	65.2	122.6	17.9	19.5	15.0	13.0	26.5	81.2	11.9
TIMM13	59.8	74.9	21.5	31.4	28.2	20.2	45.2	72.8	18.1
NDUFB7	63.3	73.8	22.7	39.5	35.5	14.8	38.1	65.4	18.3
MBD3	66.0	68.4	22.7	30.1	34.2	15.1	39.3	73.2	21.3
HDGF	58.4	63.0	24.1	39.5	38.0	28.1	35.6	64.9	17.9
CNN2	61.8	57.7	19.2	39.8	42.1	34.1	31.2	64.3	19.0
RNH1	57.9	80.0	18.4	28.8	28.0	20.2	38.1	81.0	16.5
FAM50A	70.2	64.8	22.7	34.4	33.5	20.7	39.0	68.0	15.5
NACA	66.3	72.3	19.6	33.5	27.8	27.1	33.8	72.3	15.4
TUBG1	57.3	66.3	17.7	35.5	34.8	20.2	36.3	80.4	19.5
TP53	75.6	71.4	25.0	21.7	23.0	18.9	36.7	73.8	21.7
LSS	71.4	90.0	20.5	31.8	29.0	22.2	28.2	60.0	14.5
FSCN1	49.8	62.8	19.8	29.7	29.5	13.6	46.7	92.8	22.5
POLR2E	62.8	74.3	21.4	30.9	30.4	25.4	35.5	72.0	14.2

PERP	64.2	91.9	19.4	40.5	33.4	19.8	23.5	62.6	11.3
CRIP2	71.8	79.8	27.7	34.5	36.2	18.6	30.5	51.7	16.0
FLOT1	71.1	84.4	22.1	32.0	29.5	22.0	29.5	62.4	13.3
CDH13	63.8	89.4	16.6	31.4	30.6	21.0	26.3	73.2	13.9
HNRNPK	56.7	74.1	14.6	34.9	26.6	31.0	28.9	82.6	16.1
RPS25	56.4	71.6	17.4	37.4	30.9	44.5	29.8	59.4	16.2
PLIN3	63.7	81.1	23.2	37.1	36.4	18.6	27.2	60.5	14.8
AARS	70.7	88.0	19.6	25.6	21.3	20.3	28.5	76.0	12.4
AP3D1	62.7	68.4	20.9	30.7	29.5	15.7	37.4	79.1	18.0
HINT1	56.4	72.3	19.9	45.1	36.8	30.0	27.3	59.1	15.4
ADRM1	61.9	71.3	21.2	33.9	32.3	23.8	34.1	67.0	16.7
TALDO1	62.7	73.4	21.6	35.5	35.1	26.4	26.4	65.1	15.3
SNORD15B	27.1	105.3	12.2	13.0	10.2	39.1	62.1	83.1	7.0
SSR2	49.6	72.8	14.6	28.8	23.5	20.0	34.1	95.3	20.3
TBC1D9B	60.9	71.4	18.9	33.1	30.8	19.3	32.9	75.5	15.7
UBE2M	56.9	64.0	18.7	30.7	33.4	20.3	39.3	76.4	18.6
MKX	61.8	70.9	20.4	33.6	30.0	20.1	33.7	69.5	18.4
ATP6V1F	61.9	77.8	19.4	34.4	30.0	22.4	30.2	64.0	18.0
FADS1	69.4	86.4	20.7	25.4	21.5	13.1	31.1	73.6	16.8
CHMP4B	68.3	65.2	21.8	31.8	32.8	22.0	31.6	63.5	19.8
SLC25A6	57.4	70.9	20.9	37.6	37.4	27.8	29.4	59.9	14.7
SLC25A6	57.4	70.9	20.9	37.6	37.4	27.8	29.4	59.9	14.7
PNPLA2	66.4	77.1	22.5	26.6	27.4	15.8	32.1	68.7	19.0
NUCKS1	59.5	69.2	17.1	42.1	36.3	28.9	24.3	61.1	16.4
CHD3	52.7	59.7	17.1	33.0	26.0	19.8	38.2	90.5	17.3
UQCR10	55.7	58.2	21.0	32.9	41.9	9.8	39.3	68.5	26.0
RAB5C	59.2	74.7	18.9	28.7	28.2	23.5	33.1	69.8	16.9
ATP5J2	55.5	67.1	21.5	34.2	32.3	33.9	28.8	63.4	15.3
RAB11B	57.7	64.4	20.5	26.6	27.7	16.0	39.9	75.5	23.1
TUFM	55.9	71.7	18.3	33.8	31.2	24.5	29.9	68.2	17.3
RAD23B	59.3	65.3	17.1	46.6	41.5	24.7	22.9	59.8	12.9
SHMT2	58.1	74.9	18.5	24.8	23.7	16.5	33.7	81.7	17.9
SRP14	60.5	53.2	21.3	36.5	31.3	24.2	41.5	61.5	19.8
CTBP1	60.0	74.3	18.4	29.2	27.0	22.4	35.4	66.0	15.8
ATP5E	56.0	77.7	22.2	34.9	31.6	24.7	25.6	63.8	11.5
BMP1	71.4	82.3	21.6	26.4	23.5	12.4	31.6	63.9	14.7
SPTAN1	70.5	86.9	21.0	30.7	27.5	24.3	19.8	56.9	9.9
PRPF8	62.6	81.0	18.2	21.4	19.4	15.8	28.7	84.0	15.7
TPM2	54.8	46.6	22.1	45.2	43.0	30.1	35.9	51.5	17.5
MZT2A	62.1	79.3	24.3	38.6	40.5	25.8	24.4	38.3	12.8
CALU	55.5	77.7	13.0	27.1	21.5	23.5	26.3	86.0	15.2
IER3	56.4	67.5	18.5	36.9	39.0	16.2	31.1	59.1	21.1
SOX9	60.6	58.9	21.3	16.9	19.2	7.8	50.5	82.5	27.5
GANAB	57.2	79.6	15.4	22.2	18.1	18.5	32.3	87.1	14.8
NDUFB10	59.8	58.5	19.3	33.4	32.6	19.2	33.1	68.7	19.7
C19orf10	61.9	63.6	21.0	27.2	27.9	23.4	39.8	62.3	17.0
PSMD4	58.9	78.6	17.0	30.8	26.5	19.7	29.9	70.8	11.9

CDC37	57.9	51.6	19.9	36.4	37.6	21.7	38.4	63.9	16.2
PFDN5	46.8	69.2	15.7	36.3	27.4	25.5	33.4	73.2	15.9
FAM168B	55.6	68.5	18.6	24.3	25.5	11.9	30.7	86.8	21.3
ARHGEF2	64.9	74.6	20.0	21.1	21.4	15.3	32.8	75.3	17.4
TFPI	65.9	106.8	14.7	16.6	12.1	14.5	23.8	73.9	14.1
TSPAN4	66.9	90.1	20.4	33.0	29.7	13.1	23.7	53.2	12.2
PDXK	64.0	80.7	19.4	30.2	26.6	19.2	27.3	62.0	12.2
PSMB6	55.6	73.0	17.3	30.8	26.8	17.0	34.0	72.7	13.8
CADM4	61.2	71.9	20.3	41.9	36.9	26.8	23.6	48.4	9.5
ANXA5	54.9	87.0	13.4	33.7	24.9	32.4	20.0	61.2	12.1
NOMO1	54.1	72.5	15.2	25.5	21.8	17.9	31.1	85.8	15.6
LRRC59	55.3	59.6	19.6	26.1	23.4	17.1	40.2	80.0	18.1
SLC9A3R2	48.3	60.7	17.7	33.6	37.2	21.4	34.8	68.6	16.5
ATF4	51.0	81.4	12.6	20.6	18.6	23.3	28.7	86.1	16.1
NCL	53.8	53.6	18.2	31.4	27.1	19.8	39.0	77.9	17.2
LAMA5	48.9	62.4	15.5	19.0	18.9	11.6	37.5	105.0	19.0
HIST1H2BD	59.6	52.4	20.7	36.9	31.9	24.8	32.4	60.7	17.9
SARS	59.2	82.6	16.9	24.5	20.7	19.1	28.2	72.1	13.6
RPN1	49.9	66.9	16.1	29.8	25.9	19.4	31.4	81.4	16.1
KIF1C	53.9	61.3	18.0	44.4	39.5	26.7	25.7	54.2	13.0
PRELID1	52.1	72.0	17.8	31.4	27.6	20.0	27.7	71.3	16.7
H2AFV	51.8	67.9	15.0	32.0	24.8	26.2	28.0	79.4	11.4
RABL6	60.4	69.0	20.4	30.4	31.2	20.7	30.1	59.2	14.8
HNRNPU	58.2	54.6	19.0	33.6	31.6	25.0	32.4	65.4	16.5
PXDN	30.7	36.2	10.3	16.5	17.2	10.0	50.9	135.4	28.3
NME4	52.5	69.5	19.3	42.5	44.9	26.0	23.0	45.5	10.7
ITGA5	61.2	79.0	17.8	24.9	23.1	16.8	27.5	69.6	13.9
HNRNPC	52.1	79.8	14.8	30.1	24.1	27.9	24.7	66.7	13.4
NDUFA11	54.1	73.8	18.6	33.8	32.7	20.3	29.6	55.3	15.2
RHBDD2	61.1	72.4	19.1	23.4	20.7	11.9	33.7	73.3	17.8
TGOLN2	58.5	57.9	19.6	29.2	30.6	17.7	32.5	67.6	19.0
TMBIM1	65.3	86.4	18.9	32.2	30.6	17.2	20.4	50.3	11.4
MORF4L1	53.1	71.4	13.9	28.8	23.9	31.6	25.8	70.1	13.1
PGLS	55.0	68.3	17.8	29.9	30.3	14.9	30.6	69.5	14.9
PPP4C	54.0	75.3	16.3	31.5	27.5	21.7	28.8	62.2	13.5
YIPF3	49.5	60.8	14.2	20.6	18.2	12.5	38.7	94.6	21.5
ACTN1	59.9	69.8	18.7	31.7	31.0	19.9	26.5	59.5	13.6
EEF1B2	49.2	70.7	15.8	34.6	26.8	30.2	26.5	64.7	11.7
GOLGA2	56.1	58.7	18.6	17.0	15.4	11.1	43.3	90.2	19.6
HIST1H4E	46.9	62.5	21.0	22.7	21.3	19.6	40.6	74.6	20.4
INPPL1	54.9	64.5	16.3	26.5	24.2	17.3	34.7	72.8	17.9
EIF2S3	54.4	73.3	13.8	27.8	20.4	22.7	29.6	73.4	13.4
C1orf43	49.0	76.5	13.3	24.9	19.3	18.8	28.2	83.0	15.7
SMPD1	60.9	77.8	19.3	30.9	29.3	19.0	23.1	54.2	14.1
TNFRSF10B	58.5	80.5	17.2	21.9	17.3	15.9	29.4	74.9	12.7
PTBP1	52.9	61.1	18.7	27.2	31.7	23.8	29.6	66.6	16.6
EIF4EBP1	60.4	72.3	21.5	21.5	22.3	16.1	30.8	63.3	20.0

CARHSP1	41.5	51.7	14.3	57.4	53.3	27.7	21.6	51.2	9.2
RNPS1	57.7	66.5	17.7	24.8	24.2	17.9	30.7	69.6	18.3
DAZAP2	63.6	62.3	17.8	27.8	26.2	19.3	28.0	63.5	17.9
MRPS34	53.2	56.0	19.6	34.7	36.4	16.8	35.2	57.7	16.5
RAN	47.7	80.7	13.3	38.5	27.1	29.6	20.3	58.0	11.0
TSPO	57.2	79.7	17.4	30.6	27.6	19.6	27.2	56.4	10.1
LSM7	57.0	48.1	20.8	31.7	34.1	17.9	36.4	64.4	15.1
ZNF580	58.1	58.4	21.1	28.8	32.2	13.0	33.0	60.5	20.4
ERGIC3	51.6	72.9	15.6	24.3	21.9	18.2	33.5	73.3	13.9
PSMD3	54.3	64.8	16.1	25.9	26.7	18.0	32.0	72.6	14.4
CSNK2B	54.2	72.9	16.3	26.6	27.7	12.8	25.1	71.2	17.8
CLTA	53.8	73.1	16.4	33.5	26.8	23.3	29.6	55.8	12.1
RGS16	29.7	32.0	6.1	3.9	2.9	2.2	63.0	147.8	35.9
CD99	59.6	77.9	20.7	28.7	26.4	23.8	22.0	51.0	13.0
CD99	59.6	77.9	20.7	28.7	26.4	23.8	22.0	51.0	13.0
TCF3	52.4	53.3	17.9	27.4	30.0	19.2	33.8	70.1	18.1
U2AF2	57.3	58.6	16.5	28.7	27.2	20.5	32.8	63.4	17.3
ROMO1	46.5	83.7	19.6	33.9	34.2	20.8	26.0	47.1	9.9
EEF1A2	55.0	58.4	18.7	37.6	35.6	23.6	27.6	51.9	13.2
PHB	51.0	73.5	16.5	35.1	26.7	25.2	24.8	56.9	11.5
HM13	59.2	70.3	18.5	22.1	21.3	11.5	31.5	68.7	17.6
LAMTOR2	58.6	66.1	18.5	31.0	29.2	17.6	30.6	57.2	12.0
MARS	57.4	74.7	15.8	21.4	17.6	15.2	30.7	74.4	13.6
SNORD13	40.5	52.5	18.6	14.7	14.4	18.0	79.0	69.8	12.9
NAP1L1	60.4	75.9	15.3	34.0	23.4	24.0	21.2	55.4	10.8
SNORD17	43.5	96.0	12.9	20.1	13.5	15.6	37.3	70.0	11.3
C19orf43	50.4	48.6	16.4	32.1	29.6	24.7	38.5	61.8	17.7
MTCH1	47.1	70.9	15.0	29.2	25.7	22.1	28.4	69.2	12.1
CIB1	45.4	67.6	13.3	22.4	20.9	15.7	33.9	85.1	15.2
ERRFI1	42.8	59.4	11.1	13.3	11.0	10.7	34.1	113.5	23.7
PFDN2	57.6	55.0	21.0	21.0	18.0	15.7	40.1	74.1	16.7
PXN	68.0	78.8	24.6	28.0	29.5	17.1	19.5	42.4	11.0
SLC25A3	49.8	80.1	13.7	31.4	26.7	28.1	20.5	57.9	10.6
MAP2K2	46.6	61.2	14.4	35.8	35.0	29.1	25.9	55.8	13.2
KRTCAP2	54.8	59.8	17.6	25.2	24.7	12.5	32.5	72.2	17.2
H2AFY	43.6	69.7	18.5	28.7	20.8	23.4	29.2	66.4	15.3
RPL34	38.6	76.3	10.5	30.0	17.7	50.9	22.2	59.1	10.3
RPS29	44.2	60.6	14.0	30.3	19.4	54.8	33.1	47.6	10.9
RBM8A	49.7	59.1	15.7	27.3	23.3	20.3	35.9	65.3	18.2
PKD1	57.7	76.8	16.8	19.6	17.1	12.2	31.0	70.0	13.5
QSOX1	49.3	67.1	16.9	28.2	26.2	18.2	27.9	67.1	13.6
LENG8	69.2	75.3	16.9	17.9	13.0	14.1	31.4	59.4	17.3
HSPA9	49.8	65.1	12.0	24.4	19.6	21.8	26.6	81.4	13.4
MYOF	63.3	87.8	17.1	33.9	29.4	28.7	11.5	35.4	6.6
NOP10	50.4	78.7	16.9	30.3	31.6	15.2	20.5	52.6	17.5
CAV2	61.0	86.3	22.7	39.1	39.2	25.1	10.9	22.8	6.0
TNFRSF1A	70.5	81.3	18.4	19.6	19.8	12.4	22.0	57.4	11.2

LPPR2	45.6	57.5	18.0	19.6	20.8	9.8	36.3	83.4	20.7
AP2A1	54.4	67.8	17.7	30.6	29.7	17.9	24.5	56.5	12.6
MCFD2	51.5	59.8	15.9	26.1	22.5	16.3	31.3	73.4	14.9
HIST1H1E	54.1	48.3	19.5	30.0	28.5	32.0	32.5	48.5	17.9
CNN3	62.2	81.8	17.4	26.3	23.6	19.1	19.0	50.1	11.7
PLCD3	62.5	69.6	20.2	35.8	35.6	19.1	19.3	39.3	9.1
TBCB	52.1	61.6	17.0	24.5	24.4	16.0	34.3	65.0	15.6
CERCAM	57.6	72.7	19.9	31.3	32.5	16.9	21.7	46.0	11.8
EWSR1	51.6	59.3	18.0	26.5	25.4	15.0	25.9	75.0	13.3
NEFL	35.3	43.9	11.5	20.2	19.8	9.5	50.5	90.0	28.7
SNORA70	27.9	69.9	9.7	18.2	13.2	39.7	51.4	73.6	5.9
TMBIM6	46.9	77.8	11.9	24.4	20.4	25.0	21.9	69.9	10.8
SH3GL1	46.4	57.6	15.8	30.2	30.6	23.1	29.8	60.4	15.0
TMA7	57.6	36.4	22.1	34.5	37.7	25.3	35.0	43.1	17.1
PDAP1	59.0	36.0	23.4	28.6	29.5	22.1	37.9	55.5	16.6
ANXA4	43.1	56.7	12.5	19.0	15.8	11.9	36.2	92.7	20.3
RAB7A	52.7	71.1	15.7	26.3	23.5	22.8	23.2	59.2	11.7
UBXN6	50.6	59.4	17.4	27.5	23.8	16.3	32.1	63.2	15.7
CDH2	62.4	87.1	17.9	21.3	17.5	15.4	18.5	54.5	11.1
G6PC3	51.3	66.5	17.5	27.8	27.5	14.2	26.5	62.0	12.4
APH1A	51.9	68.2	14.6	28.5	24.6	19.8	24.1	60.2	12.1
LSMD1	45.9	63.7	16.2	34.1	33.2	14.9	30.2	51.2	14.3
NDUFS7	55.5	65.2	18.0	23.8	24.8	17.1	29.3	56.6	12.9
PSMC4	44.7	58.9	12.3	19.2	16.9	13.9	35.0	84.6	16.9
NELFB	51.6	69.3	15.1	26.2	22.4	17.8	27.3	60.6	12.1
CYBRD1	59.7	83.9	15.5	34.3	27.2	23.4	14.5	36.6	7.2
SENP3-EIF4A1	50.7	70.0	14.7	26.2	23.3	18.4	25.2	61.3	11.5
SNORA10	34.9	69.7	23.8	15.9	14.7	30.3	51.4	46.1	14.2
DIRAS3	82.1	107.2	19.6	13.0	11.5	6.0	16.8	35.1	9.5
RNU4ATAC	27.0	42.9	24.2	18.7	16.2	6.1	80.9	67.5	17.1
IFITM2	74.8	75.6	29.9	21.5	25.1	9.4	19.6	32.4	11.1
AGRN	47.6	61.8	14.8	30.1	30.4	21.6	26.3	55.4	11.2
EZR	52.1	50.0	16.4	47.8	43.7	36.9	15.7	27.9	8.4
ILF3	51.6	58.9	16.4	26.0	26.4	19.8	25.8	59.9	13.2
HNRNPA1	48.5	60.5	14.1	27.3	22.6	28.6	23.9	61.1	11.5
TMEM158	49.1	56.6	19.8	20.5	23.7	7.6	31.8	70.5	18.5
EEF1D	46.6	52.8	14.6	24.7	21.5	30.6	29.3	64.9	12.6
C4orf48	50.3	53.4	21.7	28.9	30.2	8.6	39.3	51.8	13.0
SCAMP3	47.9	62.0	14.4	22.9	23.7	14.7	28.0	67.6	15.8
HIST1H2BC	42.7	51.7	16.6	28.2	28.4	18.4	32.2	62.1	16.7
TRIM8	54.1	66.9	19.2	24.1	24.2	19.3	24.3	52.8	12.0
EIF3K	44.4	66.5	13.6	27.4	23.8	23.8	24.9	60.5	11.9
TCIRG1	65.5	79.8	17.5	22.6	20.7	15.0	22.6	42.2	10.7
UBA1	49.8	66.3	15.0	24.5	22.2	17.5	24.8	63.5	12.7
KXD1	56.3	63.8	15.4	19.4	22.3	9.1	30.6	61.5	17.7
ARF4	31.1	51.6	7.3	15.9	10.2	14.3	33.6	120.3	11.7
AKT1S1	46.7	53.6	18.2	23.4	23.4	18.2	30.7	64.1	16.9

CCDC124	51.0	44.6	21.1	27.9	29.3	17.1	34.4	53.5	16.3
RIC8A	46.3	59.8	14.4	30.5	27.2	22.0	25.2	58.4	11.0
SRSF9	43.6	57.8	15.6	26.5	23.2	20.3	29.1	64.2	14.0
NDUFB11	44.3	58.6	14.7	26.6	27.3	15.5	31.8	59.4	15.8
RPS4Y1	53.8	66.9	15.8	29.3	20.5	20.6	21.9	54.4	9.8
ALKBH5	43.4	55.3	15.1	22.3	21.8	16.5	32.9	67.3	17.9
PHLDA3	52.7	63.3	18.3	32.8	30.3	14.5	25.4	45.8	9.6
AUP1	45.4	59.1	12.4	20.6	17.6	13.0	33.3	74.8	16.2
COMT	52.7	66.4	16.8	22.2	20.8	11.8	27.8	61.1	12.6
RPL36A	57.6	48.0	18.9	38.2	29.1	24.3	20.9	40.6	13.9
TMED4	49.9	66.7	15.4	24.4	21.1	17.6	26.1	57.2	11.9
RPL21	40.0	71.1	9.7	27.6	16.7	34.2	20.1	60.8	10.1
TBC1D17	48.7	53.8	16.6	31.0	30.9	15.7	25.6	53.3	14.3
RNF181	47.7	60.4	15.9	26.7	23.8	15.0	24.6	64.3	11.4
TMEM66	39.9	59.9	10.6	19.7	17.5	20.9	28.4	77.6	14.5
SCARNA7	40.4	66.3	8.0	29.8	21.6	39.7	23.6	46.0	13.4
RNU5B-1	37.4	102.7	15.1	15.3	16.1	34.6	25.5	32.8	8.7
LIMK1	46.2	57.3	15.5	23.1	23.3	13.6	28.8	64.2	15.8
NDUFS5	49.2	51.5	16.9	27.4	25.8	19.8	31.3	52.4	13.2
APRT	52.1	60.4	18.2	27.6	27.9	13.6	26.4	47.9	13.5
SDF4	52.8	54.3	19.2	25.9	24.2	16.0	29.1	51.4	14.2
COX7C	38.5	66.8	9.7	29.2	19.7	26.5	21.8	66.8	7.7
NEK7	59.9	86.1	14.2	24.4	19.6	21.1	13.7	38.9	8.5
TUBB2A	60.1	73.7	22.3	26.7	25.3	16.1	17.4	34.8	9.7
SLC4A2	43.2	52.0	11.9	28.5	25.6	19.2	28.4	63.0	13.9
NFIX	48.0	56.9	16.0	22.1	22.3	15.5	28.2	62.2	14.6
TRAPPC5	44.5	56.9	17.2	25.7	26.4	13.9	31.2	55.2	14.4
RPL5	40.3	69.2	12.4	26.9	20.3	34.9	18.9	52.1	10.4
HAGH	49.4	58.9	15.8	24.7	23.8	16.1	28.4	56.5	11.6
C19orf70	45.1	59.5	15.6	26.8	23.6	18.3	28.5	54.3	13.1
SLC3A2	52.6	73.2	14.5	14.5	12.6	12.0	26.9	66.4	12.0
TSEN34	45.4	60.1	15.3	27.6	24.0	14.6	27.1	58.1	12.1
SRRM2	54.3	60.7	16.2	18.5	16.6	18.4	23.4	61.7	14.5
HERPUD1	39.0	45.3	11.3	12.9	11.7	9.9	41.5	89.7	22.9
VAMP5	49.6	77.0	17.9	26.6	20.3	23.0	19.9	43.8	6.1
DDAH2	51.3	64.5	18.1	25.6	26.5	14.8	22.8	48.6	11.3
SLC5A3	19.8	30.9	4.6	40.3	28.5	36.4	21.6	89.9	11.3
PDIA3	45.8	65.5	14.3	27.1	22.8	18.5	20.1	56.7	12.2
UQCRC1	44.6	65.3	13.7	26.2	23.3	17.9	24.2	56.2	11.3
FAM122C	48.9	73.1	13.1	16.9	11.0	28.7	21.5	58.8	10.7
LARP1	47.4	54.1	15.2	26.0	23.6	16.5	24.7	60.4	14.7
SCAND1	45.0	59.5	15.7	22.1	24.2	13.3	27.3	60.7	14.3
LTBP3	45.7	53.3	13.0	18.1	18.1	10.9	34.1	72.1	16.7
CSNK1G2	49.8	55.7	16.6	22.5	26.2	17.7	25.6	50.7	17.1
SNORA71A	37.3	22.7	11.7	28.5	22.4	39.1	64.6	47.7	7.6
CLIC3	50.0	55.3	19.9	52.7	55.7	25.0	6.6	11.9	4.1
PFN2	50.3	77.8	16.7	28.2	22.6	19.7	17.1	40.1	8.6

PCDHGC3	44.7	49.1	11.8	13.8	13.6	9.2	35.4	89.3	13.8
TPM3	47.5	61.4	14.8	28.4	24.2	21.3	21.6	50.0	10.8
LRP10	46.2	56.6	15.6	24.2	23.0	15.5	25.4	60.0	13.0
NAA10	41.2	51.2	13.4	28.9	25.7	14.6	30.0	61.8	12.5
RPL22	46.3	56.1	14.5	26.7	20.2	28.6	23.5	52.6	10.7
SREBF1	57.5	69.6	18.3	20.4	18.5	11.1	23.1	48.7	12.2
JUN	48.0	49.4	16.9	25.9	28.0	51.8	16.7	31.8	10.0
PPAP2C	47.7	62.1	13.8	23.9	20.4	14.1	25.0	59.3	12.1
HGS	49.6	53.7	15.4	22.5	22.4	14.8	29.1	56.2	14.5
TMEM147	47.7	64.2	12.0	26.7	22.1	16.2	22.9	54.2	12.0
CLPP	46.3	58.1	16.3	23.0	24.6	13.8	27.6	54.8	13.4
STIP1	47.5	56.4	15.4	23.6	25.1	17.2	23.2	55.2	14.1
METRNL	60.7	70.0	22.6	21.7	22.2	14.5	18.9	37.1	9.3
HOXB6	62.1	71.2	21.5	18.4	17.5	5.8	22.5	45.7	12.3
MRC2	45.3	52.2	15.4	25.3	24.7	13.7	26.3	59.5	13.8
SCARNA21	53.7	28.4	15.4	24.5	27.3	22.4	40.7	47.0	16.7
MMP2	14.4	17.2	4.7	5.5	5.8	3.4	53.1	141.1	30.2
TMEM59L	55.8	64.4	18.5	25.6	26.4	10.4	22.4	40.2	11.2
HPCAL1	41.5	51.1	15.0	21.7	26.9	13.9	27.9	59.2	17.2
CTTN	53.9	59.7	15.2	33.3	28.2	21.8	17.6	36.2	8.4
PSAT1	37.9	56.1	8.8	19.1	14.2	16.2	26.9	83.5	11.2
BAD	44.3	59.6	14.8	29.5	30.9	19.4	23.1	41.7	10.4
SSR3	43.2	68.7	13.1	27.5	22.2	17.3	19.4	51.9	10.1
CIZ1	48.0	50.3	16.0	29.2	30.1	17.4	22.2	46.1	13.9
SELM	41.1	60.1	14.1	26.2	25.8	12.5	25.3	53.6	14.4
PCYT2	55.1	68.7	17.4	24.0	24.2	18.1	17.9	39.2	8.6
DPP9	50.3	61.5	17.3	23.5	22.3	15.9	21.6	47.8	12.0
MICAL2	58.8	83.1	17.5	20.9	18.4	15.8	13.4	37.7	6.5
BRK1	43.2	52.5	10.8	24.1	21.3	25.7	25.6	59.9	8.5
USF2	49.5	47.8	18.0	22.8	22.6	16.7	29.5	49.5	15.0
CDIPT	45.3	54.6	16.1	20.8	21.2	16.6	25.1	58.3	13.6
GNA11	44.9	50.6	14.4	24.9	24.5	13.6	27.1	58.3	12.9
ENSA	46.9	56.4	13.5	20.8	20.1	16.4	21.9	63.2	11.6
YWHAB	47.7	64.7	11.9	24.4	19.3	18.1	21.0	52.7	10.9
FLII	44.5	53.5	14.3	32.5	30.9	20.7	19.2	44.3	9.7
SDPR	58.0	68.1	19.8	32.6	29.6	19.5	12.1	24.1	5.6
BAG6	47.3	55.8	14.7	20.5	19.0	16.0	24.1	58.8	13.0
TNIP1	49.0	55.7	16.0	23.2	23.2	14.4	23.6	51.0	13.0
AP2B1	44.4	72.9	11.0	21.2	14.9	16.5	18.3	59.2	10.0
CSDE1	41.7	59.9	10.2	23.5	18.1	20.9	20.1	63.3	10.8
PSMC3	43.0	46.9	13.6	25.5	22.1	18.9	27.8	59.4	11.1
TLN1	45.9	58.3	14.6	18.7	17.0	18.7	23.2	61.3	10.5
STX10	46.3	53.4	16.7	21.6	20.5	15.5	28.2	55.0	10.9
YIF1A	36.9	48.0	12.0	15.1	14.2	8.7	34.3	82.5	15.4
AMIGO2	39.6	63.5	9.8	50.7	37.5	33.6	8.2	20.2	3.7
PRRC2A	49.2	50.1	16.1	22.3	21.6	16.2	24.3	54.1	12.8
PSMD11	43.1	60.8	12.8	26.8	22.9	20.4	18.6	51.6	9.4

LOC101927338	51.1	83.4	13.2	9.7	9.9	22.7	11.5	56.8	7.8
U2AF1	40.7	52.7	11.3	21.2	16.7	17.3	30.2	64.2	11.8
LOC101928293	60.9	56.3	24.8	22.0	24.9	9.9	19.1	35.4	12.8
HIST1H4K	33.4	41.9	14.8	30.1	35.0	18.1	27.0	50.1	15.2
HIST1H4J	33.4	41.9	14.8	30.1	35.0	18.1	27.0	50.1	15.2
SCRN1	50.5	64.3	14.6	25.7	22.4	20.3	17.0	42.6	8.1
PARK7	40.8	63.7	10.3	23.8	18.3	17.4	21.9	59.3	9.8
HMGN1	40.0	47.1	15.0	28.1	24.3	23.2	23.9	52.2	11.0
RPL39	40.5	56.5	11.9	30.1	19.2	27.0	21.1	49.5	9.0
BTG1	19.9	23.9	4.6	8.3	6.3	31.6	42.9	107.6	19.6
AK1	38.8	49.7	13.2	27.1	24.3	20.9	23.0	54.3	13.1
COL5A1	51.1	61.7	17.2	12.6	11.6	9.9	27.5	59.9	12.4
LOC101927337	41.7	12.6	9.5	19.8	14.8	109.6	19.4	24.6	11.3
EIF4B	42.7	54.0	11.6	22.0	18.7	23.2	23.0	55.2	12.0
EMD	46.3	48.8	13.5	25.7	22.8	12.9	23.9	55.6	12.8
POLR2I	41.4	43.0	16.1	27.6	24.5	9.3	31.2	52.5	16.0
ANKRD10	56.7	75.2	17.5	19.1	17.6	14.8	15.3	38.2	7.1
MXRA7	46.4	48.7	19.1	27.7	22.4	11.6	28.3	44.6	12.4
CSNK1A1	42.7	63.4	11.5	20.5	15.6	20.3	19.8	56.9	10.7
HN1	40.6	51.7	14.8	29.7	24.8	22.7	21.1	46.7	8.7
EBP	44.2	67.8	11.7	24.5	18.3	15.6	20.5	50.7	7.6
TBCA	42.0	45.8	17.1	30.6	23.6	21.4	22.3	43.0	14.6
VCP	43.1	60.8	11.2	22.3	18.1	18.5	20.8	57.4	8.0
COPA	39.2	57.8	9.6	15.8	12.9	13.5	23.3	76.3	11.8
SLC35B2	39.9	64.9	13.5	23.6	21.5	12.7	22.5	50.5	11.1
LINC00458	25.4	25.6	7.9	74.1	59.3	34.9	7.7	21.1	4.0
GPS1	43.0	51.1	14.8	25.6	23.8	13.6	24.9	50.5	12.6
C21orf33	39.8	52.9	15.4	20.6	22.5	12.1	28.3	55.5	12.6
MFSD10	47.9	63.3	15.4	21.8	19.1	12.4	22.6	47.2	9.4
RNF7	45.9	54.2	14.4	24.7	18.8	17.8	23.4	48.9	10.8
ANAPC11	35.8	57.6	10.8	21.6	22.3	10.3	28.8	62.5	8.9
PHGDH	37.8	49.2	10.6	18.3	14.9	12.2	29.3	74.8	11.2
S100A13	48.4	72.2	12.4	28.6	20.1	13.5	17.8	38.0	7.2
PYGB	46.8	58.4	13.6	16.6	16.2	11.5	24.4	58.9	11.6
SRP9	40.7	75.8	9.7	20.6	14.6	17.9	18.5	50.6	9.3
UQCRH	43.0	47.6	13.2	24.6	22.1	14.8	24.2	56.4	11.8
RTN3	40.9	48.0	13.5	28.5	16.7	16.6	23.6	58.6	11.2
ARPC5	40.1	37.9	16.9	24.0	24.4	26.2	28.9	45.7	13.4
NRBP1	45.2	55.3	13.5	16.5	14.5	12.9	23.8	62.3	12.7
PKIG	47.6	50.0	15.3	27.4	28.8	11.9	21.5	41.4	12.9
SF1	42.5	45.1	14.2	20.1	20.7	21.4	24.5	54.3	13.6
C11orf58	39.5	49.5	12.3	24.6	16.9	20.7	25.2	56.0	11.6
LDHB	38.5	63.4	8.8	28.8	17.8	26.2	17.6	47.5	7.7
SERP1	38.1	33.1	13.2	18.2	16.3	12.5	38.6	71.6	14.3
SLC1A5	43.9	53.8	14.0	13.9	13.3	8.7	28.3	66.5	13.3
CPNE1	44.3	59.4	13.2	21.1	18.0	14.4	22.3	52.0	10.2
CCT6A	39.8	54.4	11.7	22.2	17.9	17.7	23.5	55.2	12.2

NDUFA4	37.0	67.1	9.6	24.8	17.4	17.1	19.1	51.9	10.7
NCOR2	45.4	42.1	17.0	22.7	24.6	13.5	25.7	49.3	14.0
BTBD2	43.4	53.7	14.6	22.6	21.1	14.1	23.1	49.2	12.4
DSTN	32.9	59.7	9.5	28.1	19.3	26.9	16.1	51.8	9.8
CTDNEP1	41.6	51.1	11.6	20.4	19.6	12.4	23.8	58.9	14.1
ABHD17A	43.3	49.8	14.5	21.4	22.5	13.7	25.0	50.2	13.1
CAPRIN1	40.7	54.6	11.1	21.1	19.2	15.6	20.5	58.4	11.9
CDK6	44.9	57.0	14.7	29.6	24.1	16.5	15.5	41.2	9.4
PCNA-AS1	28.1	43.2	11.4	32.5	33.1	11.9	24.3	57.1	11.2
MAP7D1	45.2	45.5	16.7	20.7	21.9	11.5	26.3	51.0	14.0
CSRP1	34.0	49.5	10.8	21.5	19.1	16.3	26.1	63.4	12.0
DDOST	42.5	57.5	13.1	23.7	19.7	16.2	20.7	48.9	10.2
DDX39A	44.4	55.6	13.7	18.3	15.9	13.8	25.6	53.1	11.8
TLE4	58.5	70.4	18.2	23.0	21.0	12.8	12.1	29.2	6.7
GADD45GIP1	47.6	41.6	18.7	25.9	27.8	10.7	27.9	38.1	13.5
KDELR1	42.9	53.3	12.7	20.0	17.7	10.9	21.8	62.7	9.7
MRPL17	37.5	45.8	14.3	22.0	23.8	10.4	27.1	55.5	15.2
H2AFZ	34.3	54.2	9.5	31.6	23.1	24.4	18.7	47.9	7.6
TRIM47	40.7	48.2	13.8	24.7	23.8	11.8	24.6	51.5	12.0
TMEM258	35.3	51.3	10.9	19.3	15.6	15.3	24.4	68.8	10.0
CD164	41.0	53.4	14.5	21.4	18.2	12.6	24.1	50.9	14.3
DDA1	46.1	41.4	16.8	25.8	28.0	14.7	24.1	38.4	15.0
TMEM205	36.2	44.6	11.8	15.3	15.9	8.2	30.4	72.3	15.2
FUS	38.4	44.9	11.3	21.8	19.9	14.8	24.8	61.4	12.5
LOC100506634	42.7	65.9	12.6	16.8	14.4	14.0	18.1	56.7	8.6
MFSD12	44.9	59.0	17.3	19.0	20.8	11.3	21.1	45.6	10.9
TMED2	38.0	48.1	10.2	19.4	17.0	15.9	24.5	63.3	13.3
COPS6	40.6	56.1	13.0	24.9	22.9	17.0	20.4	43.8	10.2
ATOX1	42.2	55.6	11.5	32.1	31.7	12.5	15.8	37.3	9.9
SLC39A7	34.3	42.0	10.3	16.1	13.6	9.7	31.6	76.7	13.5
ANKRD11	38.8	40.3	14.0	17.0	16.4	12.3	29.2	63.5	15.8
DAG1	42.8	45.9	13.7	22.0	21.6	12.0	23.1	52.0	13.8
HSPE1	40.1	42.6	9.8	27.8	20.7	26.0	20.6	48.2	11.1
CYC1	37.5	50.8	12.3	22.3	21.9	18.3	22.1	50.5	11.2
GHITM	34.2	60.0	9.2	20.6	15.1	21.2	20.0	58.0	8.5
KIFAP3	46.2	74.6	10.2	8.7	6.3	6.8	19.4	62.6	11.6
STUB1	39.5	44.1	13.6	26.8	24.9	16.8	22.6	45.9	12.0
SH3GLB2	43.7	59.0	13.3	22.1	23.0	16.5	18.5	39.8	10.4
PIN1	40.6	46.1	14.1	26.7	23.8	17.4	22.3	43.5	11.4
OGDH	39.5	52.8	14.7	21.4	24.4	17.1	18.6	46.1	11.3
SNORA67	25.2	50.3	11.8	15.5	8.6	20.2	46.7	61.7	5.6
FKBP9	46.1	70.1	13.8	18.2	15.7	13.2	17.3	42.5	8.2
CRAT	44.7	52.4	14.5	20.3	21.0	10.0	21.1	49.5	11.8
HK1	44.4	53.8	13.7	27.0	25.6	19.6	15.8	37.2	7.6
DLGAP4	60.0	57.5	19.9	15.7	15.6	9.2	17.4	39.1	9.7
ANXA11	44.5	47.1	12.5	28.4	26.1	18.1	17.7	40.2	9.3
MRPL4	41.2	50.2	13.4	22.0	21.2	12.5	22.6	48.6	11.8

PTPRS	41.9	48.1	13.6	20.5	18.2	10.3	23.7	54.9	12.2
COL8A1	54.6	76.1	20.3	2.3	2.2	1.8	21.9	53.3	10.8
TADA3	38.3	54.6	12.2	20.5	18.2	14.7	22.7	51.4	10.4
UCA1	11.2	11.5	2.9	87.2	83.0	42.0	1.4	2.6	1.0
HSF1	41.2	43.7	12.7	18.9	18.8	13.7	25.8	54.1	13.6
EIF3C	48.0	52.6	15.0	20.6	19.3	12.5	20.2	43.1	11.0
CLIC4	37.2	60.1	9.6	22.0	18.2	18.8	16.0	51.5	8.9
SNX17	32.1	53.4	11.2	20.4	24.4	14.7	25.5	50.3	9.7
HSBP1	40.6	59.2	14.0	24.1	22.7	16.1	16.1	36.4	12.3
LINC00657	41.4	55.6	12.4	16.7	14.3	14.8	21.0	53.6	11.6
SERINC3	40.2	56.4	10.5	18.0	16.1	13.9	18.7	57.2	10.3
BAG3	42.2	44.4	14.9	15.8	17.7	8.1	24.6	57.7	15.8
SNX3	34.4	56.1	10.9	25.8	23.7	17.2	18.6	44.0	10.5
TRAF7	41.8	45.7	13.0	24.6	22.3	12.6	23.1	46.6	11.2
CDK2AP2	38.7	40.0	14.6	16.4	16.9	11.7	28.8	58.2	15.5
SOD2	45.9	59.4	14.1	28.3	25.2	17.6	12.4	30.9	6.7
SAP18	39.6	47.5	11.6	20.1	18.8	16.4	23.9	51.7	10.9
EPHA2	40.1	53.5	13.0	21.9	21.1	14.4	19.0	48.6	8.9
MAFK	53.0	63.3	17.1	22.5	20.2	13.5	16.0	28.7	6.0
PSMB5	32.0	54.5	9.1	23.7	17.8	19.2	21.9	54.9	7.2
MAN1B1	42.0	52.6	11.9	20.5	19.0	14.4	21.7	47.4	10.5
UQCRFS1	37.7	54.7	11.1	21.9	19.4	13.4	20.1	50.4	10.9
CKAP4	36.6	44.0	12.8	21.5	19.0	12.4	25.3	56.3	11.2
YKT6	32.0	50.6	7.2	19.1	14.9	12.1	24.8	65.5	13.1
ARHGAP1	47.2	57.7	14.8	18.6	17.8	13.5	17.5	42.5	9.8
HIST1H4H	32.2	49.4	9.5	28.3	23.1	27.7	18.2	44.4	6.3
ALKBH7	38.8	53.5	12.6	19.2	16.4	12.5	24.8	50.8	10.0
HS3ST3A1	45.7	56.4	17.4	21.1	21.5	10.2	16.8	40.3	9.1
LOC100996482	40.5	63.9	10.5	13.4	10.1	26.3	15.8	50.9	7.0
HIST2H4B	27.6	42.5	10.1	43.9	36.7	35.7	13.6	24.6	3.7
HIST2H4A	27.6	42.5	10.1	43.9	36.7	35.7	13.6	24.6	3.7
PIGT	43.4	50.1	13.8	23.1	23.6	11.7	18.8	40.9	12.7
NDUFA2	36.2	39.7	14.3	26.0	26.5	16.8	27.9	42.2	8.6
AHNAK2	55.2	69.9	17.7	15.1	13.5	11.1	13.1	36.8	5.7
CDK16	39.7	45.6	12.6	20.9	19.7	13.1	23.8	50.6	11.8
GTF2F1	40.9	31.3	14.8	19.9	19.6	12.2	33.6	51.7	13.9
JTB	30.1	54.5	8.3	22.6	20.6	13.2	21.8	57.3	9.1
PLP2	30.2	45.4	8.7	16.6	15.4	11.0	21.3	71.5	17.1
HAX1	37.8	52.1	10.7	20.6	15.7	13.3	18.7	56.2	11.2
DDR2	40.7	59.5	10.4	16.4	13.0	10.4	17.9	58.6	9.4
FAM127A	42.4	51.7	13.1	18.7	17.5	11.6	25.7	44.8	10.2
TMEM184B	42.4	47.9	17.1	20.2	21.2	9.1	20.2	45.2	12.1
ACADVL	35.0	45.8	10.7	23.6	20.8	16.8	23.0	47.6	11.6
TCF25	41.5	51.2	12.2	18.1	14.8	11.8	23.3	51.5	10.5
UBTD1	37.4	49.5	15.2	15.9	18.2	7.9	24.5	53.6	12.7
BANF1	34.6	64.6	10.0	16.4	11.3	19.4	18.3	49.6	10.3
CDV3	42.8	41.5	15.1	28.4	29.8	18.5	13.7	35.7	8.6

PSMB2	40.4	52.8	12.2	20.6	18.3	11.7	16.7	50.6	10.9
LAMTOR1	38.8	48.6	12.4	21.2	20.4	8.3	22.7	50.0	11.6
SLC38A2	33.0	48.7	6.7	28.0	21.0	23.1	14.8	49.7	8.7
PSMC5	33.3	41.7	10.3	22.9	20.1	18.4	23.6	52.5	11.0
FXYD5	44.8	44.6	15.9	20.3	20.0	11.3	21.1	43.3	12.4
AHCY	35.7	48.0	11.4	21.0	21.2	13.4	23.2	48.7	10.8
OXTR	59.6	78.9	20.5	28.2	26.7	14.3	1.7	2.9	0.7
ALYREF	40.9	38.6	16.3	25.3	25.0	16.7	21.6	37.0	11.6
POLD2	37.2	50.9	11.7	18.6	19.1	13.0	19.8	52.1	10.5
APOA1BP	34.0	51.7	9.9	26.6	22.0	15.3	18.2	46.3	8.6
44806	37.2	51.4	10.5	20.9	18.6	15.9	18.0	49.4	10.4
HSPD1	36.8	51.0	8.9	22.9	16.9	19.6	16.6	51.4	8.4
RUNX1	44.4	42.1	16.7	21.5	22.1	9.9	20.0	43.1	12.5
ATP5L	39.1	41.8	13.6	28.0	26.9	17.5	18.0	35.7	11.1
POLR2J3	44.6	49.6	12.8	17.5	15.4	8.4	21.5	50.6	11.3
VEGFA	27.7	29.9	7.3	16.7	13.1	8.6	35.2	76.2	17.0
ADAM9	37.8	59.7	9.3	20.7	15.3	18.9	14.6	48.0	7.2
HMG20B	40.4	45.9	16.0	21.7	20.2	13.9	23.1	39.5	10.7
SLC44A2	41.0	60.9	11.6	30.9	25.7	22.2	9.7	24.0	4.9
FAM189B	42.7	48.2	13.9	24.0	25.0	12.1	17.5	36.4	10.9
STOM	54.8	74.9	16.5	20.0	17.0	14.6	8.3	20.2	4.2
PTPRF	53.8	62.2	17.2	17.8	17.8	10.7	14.6	29.1	7.3
NCSTN	41.5	60.1	10.2	15.2	16.6	15.8	16.9	45.1	8.5
PCGF2	42.9	40.3	14.2	21.0	22.0	9.0	21.6	46.0	12.9
NR1H2	34.0	47.9	12.0	16.8	16.4	14.2	25.6	53.0	9.8
UBL5	30.5	59.1	9.0	19.9	13.6	18.9	17.5	53.9	7.2
SGTA	36.8	43.5	13.0	17.3	22.4	12.3	23.0	45.1	16.2
MLF2	34.2	42.9	11.1	20.8	18.5	15.2	23.1	51.5	11.7
SNRPB	34.7	58.2	10.8	19.8	14.8	20.2	18.5	43.9	7.8
SUMO3	28.6	38.1	10.8	29.9	33.5	18.1	20.0	38.1	11.4
ACTR2	35.3	59.2	8.1	17.4	12.8	24.4	14.5	47.7	8.5
PML	38.6	46.9	12.5	19.8	20.0	11.7	20.8	47.1	10.4
MGLL	44.2	52.3	14.3	29.2	26.3	16.8	11.6	28.0	5.1
EHBP1L1	44.0	49.5	14.6	21.3	18.6	14.2	18.5	38.1	9.0
HNRNPF	38.1	48.9	11.5	20.4	18.4	16.5	18.7	45.6	9.6
SNX21	29.6	32.8	11.5	18.3	21.6	10.2	26.6	60.6	16.4
HSP90B1	34.4	47.3	9.5	22.5	17.1	17.1	17.1	52.6	9.8
SF3A2	43.6	37.9	15.1	16.4	18.1	11.4	26.1	44.5	14.2
ELOVL1	41.5	56.5	12.2	19.9	19.1	14.8	15.9	39.8	7.0
VOPP1	28.0	33.6	9.1	20.5	19.9	15.1	26.1	60.6	13.7
UBQLN1	39.0	51.8	10.9	21.9	19.2	14.6	14.7	44.0	10.4
RPS27L	33.1	52.9	9.0	19.8	17.1	18.1	17.9	50.5	7.9
MAGED1	36.7	49.0	11.6	15.2	14.8	9.3	21.1	56.3	11.9
MVD	42.1	44.7	13.5	23.0	21.2	14.5	19.8	38.9	8.3
DHPS	39.8	50.2	13.1	14.9	13.2	8.0	23.9	51.3	11.3
C6orf48	38.3	55.6	9.3	15.2	11.8	8.2	20.0	57.2	10.3
SNORD33	42.0	26.4	26.5	15.4	13.1	7.7	53.6	29.5	11.2

LSM4	36.6	39.4	10.4	26.6	23.7	17.0	21.6	41.4	8.7
MEF2D	43.4	46.5	15.8	14.0	15.2	11.4	21.3	44.4	12.8
POLDIP2	36.6	49.9	11.2	19.1	19.5	11.2	19.2	47.8	10.4
CDK4	35.1	50.5	10.0	22.6	19.4	15.0	17.1	47.2	7.6
SLC25A1	39.0	50.7	13.3	22.0	20.6	11.9	17.9	40.2	8.7
HIGD2A	41.3	42.3	13.9	21.6	23.3	13.0	21.1	36.5	11.4
PRR13	44.5	44.7	11.4	23.7	19.5	13.5	16.1	42.6	8.2
PIEZO1	36.1	44.1	12.0	30.7	28.8	18.5	15.1	31.6	7.3
PRSS23	55.3	74.5	16.6	19.5	16.4	12.8	8.3	16.4	4.3
EIF2S2	41.3	42.0	10.8	21.2	16.8	15.6	18.8	47.2	10.2
ATRAID	34.2	57.7	11.7	21.5	20.4	9.6	18.0	41.1	9.7
UBE2L6	41.4	47.3	12.1	17.3	16.1	12.1	19.4	46.7	11.1
HNRNPL	30.9	43.3	9.8	20.7	19.7	22.3	19.8	47.0	9.8
HIF1A	34.6	50.6	8.2	32.9	27.0	24.3	9.0	30.0	6.4
MKNK2	41.5	46.1	12.5	10.7	11.3	17.9	21.9	49.2	11.7
MACF1	42.4	64.5	11.8	14.5	11.8	13.9	12.5	45.0	6.3
ASNA1	33.6	44.4	11.2	23.1	21.7	12.8	20.1	46.3	9.5
SLC20A1	35.9	57.0	9.0	13.7	10.9	11.2	17.4	59.0	8.4
SCARNA12	22.8	48.7	8.0	17.7	13.6	44.4	20.5	42.0	4.7
PPP1CA	33.5	46.2	11.1	18.6	17.7	16.3	21.0	47.0	10.9
ARF5	34.2	54.3	11.3	19.1	20.3	16.2	18.7	39.9	8.1
SPCS1	38.3	42.9	11.9	20.1	18.0	11.0	25.1	43.5	11.0
CLIP2	33.7	39.7	11.3	29.5	25.2	16.0	19.6	38.3	8.0
DNM2	39.1	46.1	13.1	19.2	18.6	13.3	20.0	42.7	9.2
HOXB8	47.7	52.7	15.3	15.0	12.9	6.0	18.8	41.1	11.4
ATXN2L	42.6	42.2	13.2	18.5	17.9	11.9	19.0	44.3	11.1
LOC101928686	34.4	40.9	12.2	16.9	16.5	10.4	23.2	53.7	12.5
DBNL	33.4	41.1	12.7	22.6	21.7	13.8	21.4	45.3	8.6
VCL	51.5	69.8	15.3	15.5	12.9	12.1	10.5	27.5	5.0
ARPC3	34.1	48.6	10.0	20.0	16.9	17.5	16.9	45.8	10.4
HEG1	34.1	38.7	9.3	39.8	33.9	21.4	9.6	27.3	5.4
CALM1	30.8	49.5	8.6	17.4	15.8	20.6	15.4	52.9	8.4
RRBP1	38.8	33.3	14.7	16.2	15.8	9.5	27.0	50.7	13.4
EHD1	38.8	43.0	13.6	25.4	27.6	17.7	14.8	30.4	8.1
PDLIM1	43.1	57.2	13.4	28.4	25.8	15.8	8.3	22.2	5.1
ABCA2	40.3	49.4	13.3	18.8	17.3	9.6	18.8	42.1	9.2
KIAA1191	34.8	49.6	12.1	18.4	17.7	10.2	17.9	47.7	9.5
IDH3G	36.3	40.9	11.7	17.4	18.6	11.9	22.9	46.1	12.1
ATP5G3	31.3	57.6	8.5	24.0	16.7	20.3	14.6	38.3	6.3
TPD52L2	35.6	52.0	10.3	20.4	18.8	14.7	16.4	41.8	7.6
EIF2AK1	37.3	51.5	10.8	18.1	15.4	12.2	17.0	45.5	9.5
NHP2	32.6	36.4	10.6	21.3	19.9	13.6	23.9	48.3	10.3
SORBS3	38.2	37.8	14.8	25.0	25.4	14.6	17.6	33.3	10.1
C16orf13	35.0	41.7	12.2	19.9	19.4	10.3	23.9	43.9	10.3
RRAS	45.6	58.9	14.3	17.8	16.6	12.4	14.4	28.6	7.4
SEMA6B	35.8	42.9	13.3	20.3	21.3	10.8	20.1	40.5	10.9
LOC101927374	35.3	66.2	9.6	8.6	7.2	22.6	9.0	50.8	6.4

H3F3A	31.1	53.0	9.1	16.8	13.0	17.3	17.9	47.7	9.9
ARPC1A	34.2	49.2	10.5	22.0	18.5	12.9	16.6	42.1	9.8
NPC2	44.7	55.4	13.9	22.8	22.4	12.7	11.8	24.8	6.9
DPM3	35.0	47.3	11.0	16.1	14.7	6.8	25.8	50.8	7.9
ZBTB4	35.8	39.9	12.7	18.7	19.2	10.8	21.0	45.6	11.6
ATP5J	31.8	47.3	7.5	20.0	19.5	16.8	16.5	46.8	9.0
VPS4A	35.3	42.3	11.1	19.5	17.2	17.9	19.5	44.1	8.1
ZNF358	35.6	40.3	12.5	23.7	23.4	11.7	19.7	37.0	11.0
ECE1	29.0	34.6	10.2	14.0	13.2	8.8	25.6	65.0	14.4
EGFL7	44.9	36.8	17.0	25.9	28.1	13.3	17.8	20.8	9.7
MPG	36.8	47.9	14.1	18.7	18.5	11.0	18.2	37.8	11.2
ST6GALNAC6	35.2	41.4	10.5	24.3	24.6	15.2	18.0	37.0	8.1
WARS	38.4	43.7	10.8	13.8	13.5	7.7	20.4	55.5	10.3
TMEM132A	34.1	43.8	10.7	19.1	17.6	11.5	22.0	45.6	9.6
CD97	37.2	45.5	12.4	14.4	12.5	16.1	19.7	45.5	10.3
SEZ6L2	36.7	39.1	12.2	11.9	11.5	7.0	26.0	56.6	12.6
ATP1A1	30.6	42.1	8.4	20.2	16.3	17.8	17.7	51.8	8.3
CSNK1E	42.1	50.4	13.2	13.7	14.0	7.3	18.8	41.8	11.3
ACTR1A	35.4	48.6	10.1	17.2	16.2	10.8	19.1	46.0	8.7
IARS	32.6	49.1	7.2	14.6	10.9	11.2	17.9	59.2	9.6
SCNM1	35.8	45.5	10.7	15.3	12.0	20.6	19.1	42.6	10.4
LOC100288162	37.5	55.2	9.7	12.4	9.9	8.2	21.9	48.9	8.1
TMEM214	32.1	41.3	10.9	15.4	13.1	9.3	22.7	55.4	11.5
SF3B2	37.8	40.8	11.6	17.5	15.9	12.3	21.0	44.5	10.3
CAST	34.6	43.3	8.6	18.2	14.7	14.5	16.5	51.7	9.3
DUSP1	30.3	37.6	9.3	28.7	24.3	46.1	9.0	21.3	4.8
UBE2I	38.6	44.6	11.1	17.5	18.6	14.6	15.4	39.4	11.4
LAMB1	35.6	47.7	10.1	15.1	12.3	10.4	18.3	52.6	9.1
RBCK1	35.5	45.2	10.1	13.3	12.4	11.1	22.4	49.9	11.1
TFG	34.4	41.5	11.3	17.1	15.1	8.4	19.3	50.7	13.1
PLAUR	43.5	58.5	14.9	13.6	11.9	6.6	15.5	37.8	8.5
MAF1	32.8	36.9	11.0	17.4	15.1	10.5	23.4	50.6	13.0
HIST1H4B	21.9	45.8	7.7	25.5	20.4	26.9	17.5	39.0	6.1
NLGN2	37.8	45.1	13.1	16.9	16.2	8.8	20.3	41.9	10.2
CIRBP	40.0	42.1	12.3	16.4	11.9	13.1	22.7	42.8	8.5
ATP5O	30.8	49.2	8.6	23.1	18.3	17.3	15.5	40.3	6.7
ACOT7	35.3	40.3	12.2	20.9	19.8	9.9	20.2	42.0	9.0
CAPN1	35.5	44.9	11.5	20.3	21.0	15.0	16.4	36.0	8.4
CBS	38.1	43.2	10.3	17.2	15.9	10.8	21.2	42.9	8.5
SNRNP200	36.3	46.4	10.1	14.1	12.4	11.4	18.2	49.8	9.3
MAP1LC3B	43.7	34.7	14.3	17.2	17.8	10.5	19.4	40.2	10.0
FSTL3	38.7	55.7	15.5	33.2	31.8	16.4	5.5	8.3	2.5
FKBP10	36.0	43.2	10.3	12.2	10.8	6.8	25.4	51.2	11.5
PLEKHB2	35.5	53.5	8.3	17.1	13.9	15.1	15.3	42.5	6.0
IGFBP6	36.7	45.1	11.7	24.3	22.4	9.3	17.7	30.9	8.8
AP2S1	31.1	49.0	11.2	18.3	13.8	10.9	20.5	43.8	8.2
SNORA52	14.5	61.5	7.8	11.6	5.2	19.4	31.4	50.9	4.2

STAT3	34.5	43.5	10.6	19.2	17.0	14.9	16.0	41.4	9.1
SSBP4	38.0	41.7	12.6	23.0	18.4	12.7	19.8	30.7	9.2
TNFAIP2	42.5	52.0	12.5	14.8	13.0	8.5	17.1	37.7	8.1
CD99L2	37.4	41.0	13.7	18.5	17.3	17.2	16.3	36.4	8.2
TSPAN3	34.7	53.4	10.4	21.0	19.7	15.8	11.4	32.5	7.0
SMIM3	20.9	27.7	5.6	8.3	6.6	4.8	28.2	91.1	12.6
AURKAIP1	33.3	36.0	11.6	20.0	17.4	10.7	24.1	41.5	11.0
HIST2H3D	24.5	40.7	9.7	37.6	39.2	27.6	8.0	14.3	3.9
SF3B1	34.7	49.3	7.9	13.2	10.4	14.0	18.0	48.7	9.3
AP1S1	31.1	49.0	9.8	17.7	14.1	16.3	18.8	40.6	7.8
ITM2C	35.3	40.8	11.4	18.7	17.4	14.9	18.3	39.5	8.8
TOR4A	38.1	49.9	12.8	18.8	17.7	11.0	17.3	32.0	7.5
ATP5A1	30.9	50.4	8.6	18.6	14.5	17.3	15.0	42.9	6.9
CYCS	31.5	40.2	8.5	18.0	10.4	20.1	19.0	47.8	9.6
SF3B4	37.1	37.3	12.3	19.1	20.1	10.5	18.1	39.6	10.9
LAMC1	33.9	44.9	10.1	13.5	10.9	8.0	18.5	56.3	8.9
PDIA6	29.5	45.5	7.7	21.5	15.4	17.2	17.9	43.4	7.0
LAYN	38.5	47.7	10.1	38.3	31.0	23.0	4.2	9.2	2.9
ATP6V0E1	26.1	46.7	7.3	19.5	16.6	29.0	17.0	37.4	5.3
PDLIM7	33.5	41.5	13.5	22.1	24.3	12.3	16.7	32.2	8.6
CDK9	30.4	40.0	10.1	19.6	21.7	12.6	17.6	42.6	10.2
PFKP	38.0	50.7	13.9	19.6	19.2	15.7	15.2	26.5	5.8
MRPL34	29.6	36.0	12.7	27.3	26.5	15.3	15.7	29.9	11.3
AGPAT2	31.6	40.4	11.0	20.1	19.9	10.7	18.6	42.2	9.7
ILF2	29.0	48.8	7.7	20.2	18.0	14.1	14.5	43.7	8.0
CALM3	35.6	43.0	13.8	25.3	24.1	13.1	13.2	28.6	7.1
HNRNPH3	34.4	38.8	10.4	14.5	11.8	13.3	22.9	48.5	9.4
LAGE3	29.9	45.4	10.5	22.3	20.9	15.0	17.9	34.8	7.2
YIPF2	26.8	38.1	9.4	14.1	14.3	8.1	24.8	55.9	12.2
PIGQ	31.4	39.2	9.3	22.2	21.6	10.2	18.5	41.7	9.6
EIF3I	32.6	47.7	10.2	19.7	16.9	14.9	15.4	38.2	8.1
ATP5H	28.8	45.7	6.5	21.8	16.2	19.6	14.6	43.3	7.0
PSMA1	35.7	50.8	8.4	21.3	16.4	16.3	12.2	34.4	8.1
BAX	31.5	47.7	9.5	14.9	12.5	13.2	21.6	43.8	8.8
SNHG7	25.5	46.9	8.4	15.8	13.6	14.5	22.7	47.4	8.2
TMEM203	32.9	48.9	12.1	18.7	16.9	11.8	16.8	36.5	8.4
CDC34	34.8	35.9	13.1	18.4	18.5	10.9	20.5	41.6	9.2
SCAF1	34.3	34.1	13.1	16.7	16.9	10.6	22.8	44.1	10.2
NINJ1	28.5	36.0	11.3	15.3	18.7	9.5	23.5	45.8	14.1
RAC2	37.8	39.9	13.6	20.3	19.4	19.1	13.7	32.2	6.7
RAB43	22.7	37.8	7.8	11.6	13.6	12.5	26.1	59.4	10.9
NDUFB8	36.5	39.3	11.2	22.4	18.8	15.4	17.8	32.9	8.0
RALY	35.3	40.3	12.0	18.9	18.2	12.6	18.8	36.7	9.4
PRAF2	31.6	31.0	11.7	18.6	19.7	11.4	24.6	42.9	10.6
SH3KBP1	20.3	21.4	6.2	19.5	17.9	15.3	23.6	64.7	13.0
GPR137	32.4	46.9	10.3	17.8	15.0	11.0	17.4	41.9	9.2
BABAM1	31.5	47.4	10.6	19.9	18.1	11.6	15.9	38.7	7.7

VPS28	37.6	43.9	10.6	17.7	14.4	13.6	18.2	36.4	8.9
NDUFAB1	30.0	47.1	10.7	19.0	12.8	9.3	20.2	45.3	6.9
FARSA	31.2	37.3	10.1	16.9	16.2	11.9	21.5	45.4	10.7
AKIP1	33.1	47.3	10.2	20.4	16.3	20.9	14.2	33.2	5.5
TXNIP	39.8	50.2	8.7	2.8	2.5	49.6	11.4	29.6	6.6
CLPTM1	35.1	38.1	11.0	16.1	15.8	9.2	21.0	43.8	10.8
CCT5	33.9	47.1	10.2	19.3	14.7	13.7	14.8	40.2	7.1
AMFR	33.7	45.3	9.6	19.0	16.5	12.0	16.0	41.2	7.5
PYCR1	26.8	37.4	8.8	14.5	13.8	7.8	25.1	56.5	10.2
LAMB2	34.7	45.3	9.4	20.4	17.7	11.9	16.6	36.5	7.9
DNAJA1	28.5	37.6	7.3	20.2	15.4	17.5	17.0	49.0	8.0
ANXA7	33.9	53.4	8.7	16.7	13.1	12.4	14.8	40.5	7.0
ATP6V1G1	33.6	32.0	12.5	22.1	21.4	16.7	17.1	33.2	11.7
SF3B5	28.4	43.7	9.6	17.9	18.2	11.7	18.6	43.2	8.9
TRNE	23.2	38.0	10.0	19.5	6.6	4.2	33.6	56.6	8.1
VDAC1	32.6	44.0	9.3	20.2	17.0	14.7	14.7	38.7	8.5
HSPA1A	27.0	38.7	9.5	18.0	19.4	13.7	17.1	47.2	8.9
SLC39A1	31.2	44.4	7.5	20.9	20.6	17.0	17.8	34.5	5.7
GTF2I	31.2	43.4	7.5	17.4	13.9	16.6	17.3	44.0	8.2
SLC2A4RG	35.5	42.6	14.1	21.3	22.4	12.2	14.4	28.8	8.0
ATPIF1	30.7	29.3	13.2	25.5	22.8	15.3	18.0	31.5	13.0
YPEL5	34.3	52.7	8.8	20.9	15.3	16.2	11.3	32.5	7.4
TMEM248	33.0	43.9	10.2	16.7	15.9	12.2	14.8	42.2	10.0
WDR34	31.9	41.2	10.2	28.4	24.5	17.4	13.6	25.1	6.1
PALM	36.8	36.6	14.7	21.9	24.0	11.0	16.5	28.6	8.0
MRPL28	33.8	34.5	10.8	23.0	22.7	11.4	18.9	34.1	8.9
TPM1	41.7	44.5	13.8	27.8	25.6	16.5	8.2	15.7	4.2
BOLA2	30.4	41.4	10.8	19.0	18.2	7.4	20.9	38.8	11.1
TRNA	28.6	39.5	17.0	13.1	12.9	2.2	29.1	47.5	7.9
STK11	32.6	40.2	10.6	18.0	17.0	11.5	19.8	38.9	9.1
CNP	29.3	38.0	9.7	18.0	17.4	11.7	19.2	44.7	9.6
SNAPIN	30.5	48.4	9.9	19.6	18.3	12.5	14.5	36.2	7.8
DAD1	27.2	41.7	7.5	17.3	12.7	15.3	17.9	46.4	11.3
DDX17	28.2	41.7	7.4	16.2	14.4	20.8	16.2	45.3	7.2
ST13	32.6	38.0	8.9	17.8	15.1	13.0	18.2	44.1	9.9
LZTS2	38.8	44.2	12.5	15.8	16.5	8.9	17.8	34.3	8.5
HLA-E	31.4	37.8	10.3	13.4	12.1	50.9	11.9	23.9	5.0
SERBP1	33.8	33.9	11.4	20.4	17.9	16.6	18.1	35.4	9.0
ATP5C1	31.6	50.6	7.8	13.9	11.4	13.2	14.5	44.7	8.8
ZNF428	34.0	39.0	10.1	13.6	12.4	7.6	24.4	45.4	9.8
NACC1	33.6	40.2	12.7	18.7	20.1	9.9	18.1	34.6	8.3
SFPQ	34.6	35.8	12.0	16.3	15.6	16.6	19.1	36.2	9.9
CDK18	48.5	58.4	13.6	17.7	14.3	9.5	9.2	19.7	5.2
PFKL	29.2	36.9	9.6	15.7	14.7	10.6	22.2	47.7	9.5
DUSP3	33.9	38.2	11.1	23.7	23.2	11.9	14.5	33.5	6.1
ARL6IP4	31.7	31.7	12.4	18.4	17.6	13.3	24.5	37.0	9.6
ERP29	37.6	42.0	13.3	17.8	15.9	9.6	15.8	35.9	7.8

COASY	30.1	39.1	9.2	17.5	16.5	11.3	18.4	44.5	9.1
SUMF2	33.0	45.9	8.8	17.5	15.2	13.8	15.5	38.2	7.8
CUEDC2	32.3	43.4	10.2	16.0	14.3	14.8	17.3	39.1	8.0
SUPT6H	33.5	37.2	10.2	14.7	14.0	11.0	18.8	47.0	9.0
MARVELD1	30.1	37.3	12.4	15.5	16.4	6.2	20.1	43.4	13.8
SEC14L1	34.8	48.9	10.1	18.7	17.1	13.2	12.9	33.2	6.3
GORASP2	30.4	38.8	9.5	14.2	13.7	7.2	17.6	50.5	13.1
PSMA2	26.9	47.4	8.0	19.0	12.4	21.2	15.6	38.4	5.9
CENPB	29.4	37.6	10.3	16.9	15.8	11.4	21.4	42.5	9.6
CLTB	31.8	34.4	11.4	24.7	24.5	18.0	15.2	28.2	6.8
SCARNA10	24.7	52.1	6.2	13.3	11.0	20.9	19.7	39.7	7.1
CD276	35.5	45.0	12.5	13.4	12.7	7.3	18.5	40.9	8.8
SLC2A1	34.2	46.3	10.4	24.7	23.0	13.5	10.9	26.1	5.5
PTK7	32.3	41.8	9.3	20.0	16.6	11.5	16.5	38.0	8.7
ZNF207	35.2	47.9	8.0	18.1	14.6	12.1	13.5	37.1	8.1
SNORA7A	13.8	48.6	5.1	9.5	5.6	48.6	25.6	30.8	6.3
RNF167	29.4	37.0	9.2	16.7	15.9	11.0	18.8	46.3	9.5
TRIB3	35.0	48.1	11.0	8.1	8.3	5.0	20.3	47.9	9.3
ATP5G1	27.8	40.6	7.9	21.1	17.1	13.7	16.7	40.6	7.7
DDR1	34.1	44.5	10.1	15.3	12.8	9.7	18.5	40.1	7.7
XRCC5	30.1	45.2	8.2	15.5	12.2	14.6	14.4	44.6	7.9
RNU12	27.7	39.6	16.1	10.5	13.5	5.6	30.9	34.9	13.8
TMEM219	33.2	46.9	9.8	17.4	13.9	8.5	16.2	38.6	7.8
RND3	37.3	56.8	9.2	23.1	21.3	17.9	5.6	17.3	3.9
MAGED2	32.9	42.0	8.1	13.0	10.9	8.8	19.3	47.4	9.9
AAMP	31.5	38.2	8.7	14.2	12.5	10.4	23.1	45.4	8.2
FBLN1	35.2	43.0	11.6	23.5	22.5	12.1	13.4	25.3	5.5
AP1M1	36.4	36.1	10.7	14.8	17.1	11.4	17.0	38.1	10.3
VAMP2	34.7	33.0	10.6	14.1	12.9	10.7	21.2	41.1	13.5
TFE3	35.3	38.8	11.8	18.1	17.9	8.8	16.2	36.0	9.1
SNORA71D	12.5	42.2	6.1	9.8	4.6	55.6	22.3	35.4	3.4
PLXNB2	33.6	38.5	11.2	18.5	18.0	11.5	16.1	36.3	8.1
FAM89B	25.6	32.8	11.5	17.8	18.6	10.3	21.1	41.2	13.1
ASPH	32.1	43.4	8.6	15.3	12.5	12.4	13.7	44.6	9.1
MGRN1	33.6	34.4	11.3	15.4	16.6	9.1	18.8	41.4	11.1
ARF3	28.2	41.2	7.6	14.2	12.6	15.1	20.0	46.4	6.3
POR	34.1	42.2	10.7	13.3	13.8	8.0	20.1	39.8	9.5
IDH1	28.8	42.9	6.8	14.7	11.2	11.2	16.0	52.2	7.6
XRCC6	31.9	46.1	8.4	15.8	12.4	15.1	14.5	39.2	7.5
NUCB1	36.9	39.4	13.2	17.3	16.0	9.6	17.3	33.3	7.8
ATP1B1	18.6	28.2	4.2	43.4	33.7	31.5	7.0	19.9	4.3
NDUFS6	37.5	26.2	13.1	22.6	19.2	15.3	21.4	24.2	11.1
ADD1	34.9	39.4	10.1	16.3	15.3	12.9	15.5	38.2	8.1
CHID1	35.1	48.9	10.8	12.8	11.9	8.3	17.5	36.5	8.7
SDC1	29.4	32.9	11.3	24.8	25.2	12.1	16.6	30.4	7.9
DNAJB6	32.7	43.7	10.0	18.3	16.7	15.3	13.7	33.4	6.6
ARL6IP5	30.6	40.2	8.6	20.5	14.8	17.5	15.6	34.3	7.6

GOLGA3	28.4	32.9	10.2	10.5	10.3	8.5	22.3	56.7	9.7
CORO1B	35.5	40.2	11.7	17.8	16.9	10.2	16.7	32.3	8.2
PPP2R4	35.3	43.1	10.6	14.6	12.5	11.3	16.5	37.0	8.5
NDUFB9	25.1	32.6	10.5	21.7	20.1	11.6	15.7	40.7	11.3
SMG5	29.2	39.9	7.6	17.3	15.4	10.5	17.1	44.3	8.0
KIAA0100	29.6	40.7	7.8	17.4	13.6	11.3	16.2	44.6	8.1
PCBP4	33.6	34.7	10.9	17.7	15.1	8.4	16.0	44.3	8.4
NSMF	32.7	38.9	9.7	20.8	18.5	13.1	14.3	31.9	8.8
REEP2	33.0	41.7	11.4	20.9	18.8	10.9	13.8	30.5	7.7
C19orf24	30.3	35.1	12.1	14.6	17.2	7.8	22.6	35.9	12.9
PYCR2	33.1	38.0	9.1	16.8	12.8	10.2	21.5	38.7	8.2
MPP7	47.3	56.9	9.5	14.3	11.6	11.8	8.3	24.4	4.3
HIST1H2AE	26.9	35.6	10.0	19.3	18.9	30.4	13.0	25.4	8.6
AIMP2	32.1	48.5	8.9	12.7	11.5	8.6	16.3	40.5	9.1
KHSRP	33.9	33.0	10.4	16.8	17.0	16.5	16.7	34.9	8.9
SRXN1	29.6	34.6	12.2	16.0	18.5	9.3	19.1	37.6	11.2
SEC16A	28.2	36.1	9.2	11.3	11.1	8.5	19.4	53.9	10.2
FBN1	35.8	52.7	10.4	7.6	6.2	6.1	14.5	47.3	7.3
AKAP12	52.9	55.3	16.4	11.1	9.3	7.9	9.7	20.3	4.7
CEBPB	34.2	33.8	13.2	12.0	12.9	17.2	20.4	34.1	9.8
MARCKSL1	25.4	31.5	8.9	14.9	13.8	8.8	21.6	52.9	9.4
DTX2	37.9	40.6	11.4	18.3	20.1	9.3	12.8	29.9	6.9
CLTC	28.5	45.5	6.6	16.4	12.7	15.1	12.5	43.3	6.4
MAPK3	34.9	41.7	11.9	18.3	18.2	8.9	14.2	31.1	7.9
TGFB1I1	32.3	36.5	10.7	16.3	15.6	8.5	18.6	38.4	10.2
PSMB1	30.6	44.7	8.2	16.4	13.5	13.2	15.6	38.1	6.7
NT5E	38.3	58.3	12.1	13.9	12.1	9.1	9.9	27.6	5.5
CPA4	35.7	43.9	7.9	28.5	24.0	21.9	7.6	13.3	3.8
TPP1	33.7	43.0	10.8	17.7	14.7	11.6	14.5	33.5	7.2
WIPI2	32.0	37.1	9.4	16.6	15.2	9.8	16.7	41.3	8.1
RAB1A	29.4	43.7	6.4	13.1	10.5	15.9	13.9	45.3	7.8
RNU6ATAC	20.0	35.8	15.2	6.2	6.2	19.7	38.2	39.3	5.6
WDR26	29.5	36.5	10.0	15.2	14.7	11.7	16.9	42.4	9.3
TRIP6	28.5	36.5	9.5	19.3	18.5	12.8	17.3	35.7	7.9
DCAF8	28.7	38.5	8.1	12.8	9.2	8.8	22.1	48.3	9.5
ISOC2	24.0	33.6	8.1	15.6	17.2	8.4	21.3	48.2	9.6
ITPR3	40.6	48.6	12.1	19.1	17.4	10.9	10.2	22.1	5.0
COX5A	34.3	39.2	9.8	20.4	17.5	10.5	15.2	30.1	8.7
BUD31	28.7	46.9	7.8	14.7	12.3	9.0	16.1	41.1	9.1
MPRIP	31.7	39.2	11.3	16.6	15.6	11.3	15.8	36.1	8.0
RPL36AL	29.4	36.6	8.1	18.8	15.5	17.5	17.7	33.5	8.5
TAF10	27.6	39.4	12.6	20.2	20.6	15.3	13.1	29.0	7.7
RGS3	34.9	45.9	10.5	9.3	8.9	7.2	17.9	41.6	9.0
NGRN	27.8	43.2	8.0	14.8	12.6	12.4	16.0	41.1	8.8
CSNK1D	31.6	40.9	9.9	14.7	14.5	12.9	15.9	35.7	8.5
BCAR1	32.7	36.7	11.4	16.3	16.8	8.6	17.5	35.5	9.1
PDE1C	46.5	60.6	12.5	17.2	15.0	11.2	4.9	13.7	2.8

SBDS	33.7	48.7	9.0	16.0	13.6	12.5	10.9	33.8	6.3
MICALL1	32.8	35.8	11.5	20.7	19.4	11.8	14.0	31.4	6.7
LMNB2	29.0	31.7	11.5	26.2	24.3	15.4	14.5	24.8	6.6
IGF2R	34.4	43.4	9.6	13.0	11.3	12.1	13.0	40.9	6.2
SUPT5H	30.4	32.1	9.9	14.9	14.7	11.7	19.5	41.3	9.3
IER5	27.2	31.7	9.5	21.9	19.3	25.2	16.8	25.0	7.1
DMPK	33.4	44.6	10.0	14.7	14.9	9.9	17.2	31.6	7.5
SEC61B	26.5	37.0	6.2	13.7	12.3	13.2	18.3	46.9	9.6
HOXB-AS3	37.4	48.5	10.9	12.9	10.6	5.9	13.4	36.2	7.6
PLK2	42.9	55.9	11.5	23.3	19.6	17.4	3.5	7.4	1.9
HNRNPA0	26.6	37.9	8.3	15.5	13.8	16.8	19.0	38.3	7.3
FAM32A	30.1	26.3	12.5	21.1	19.2	14.8	18.9	30.0	10.4
CCNL2	34.2	42.9	8.7	9.3	6.9	11.3	20.2	40.1	9.6
XPOT	28.6	43.0	5.5	12.2	9.2	10.5	14.5	50.6	9.2
KEAP1	29.1	35.0	9.4	15.3	14.5	8.2	20.1	42.2	9.3
ARHGEF1	26.7	31.3	9.0	12.0	12.2	24.9	18.2	40.2	8.7
C11orf31	27.8	43.9	11.2	17.2	14.5	10.9	16.0	31.9	9.4
GAMT	28.0	37.0	9.7	21.0	23.2	10.6	14.9	30.5	8.0
SH2B3	29.5	38.3	9.5	7.6	6.5	7.5	20.3	52.3	11.2
UNC13D	36.2	43.4	12.7	21.5	21.9	14.9	8.8	18.8	4.4
TCEA1	26.6	38.6	6.0	11.2	8.2	9.6	16.8	56.5	9.2
TMEM109	29.4	36.6	9.0	18.2	17.7	12.8	15.9	36.2	6.8
COX7A2L	30.6	43.1	6.3	15.3	13.5	13.5	13.7	37.8	8.7
MMP14	27.8	38.6	8.3	12.2	10.7	8.8	18.1	48.4	9.4
UBE2H	27.0	36.1	7.5	22.2	17.6	18.6	12.6	33.6	7.1
PRDX3	25.8	41.0	8.3	17.9	14.1	16.0	16.0	36.9	6.3
CBX3	21.1	41.5	4.9	18.9	13.3	20.2	14.7	42.2	5.4
CIC	32.4	36.6	11.4	17.2	17.0	11.3	14.7	33.1	8.2
LOX	25.8	36.2	9.2	14.2	13.4	8.1	17.3	46.6	11.1
TMEM8A	31.9	36.3	9.9	20.6	20.7	11.4	13.4	30.0	7.6
NPLOC4	28.9	40.2	8.2	18.3	15.3	12.6	13.7	36.7	7.8
LOC728752	33.7	44.3	11.4	11.8	11.5	6.8	14.4	40.9	6.8
AMOTL2	44.9	54.2	15.9	21.8	22.6	12.8	3.0	5.0	1.3
PLCG1	33.8	42.5	8.8	11.3	10.5	9.0	16.4	41.2	8.0
WFS1	35.0	37.0	11.2	22.6	22.4	10.7	11.2	24.7	6.5
CCT8	27.6	45.0	7.1	17.2	13.2	15.6	11.8	37.0	6.7
CASC4	27.6	39.3	7.8	14.4	12.7	9.6	16.8	43.6	9.3
ARF6	31.0	40.2	11.3	14.7	15.3	12.9	13.7	33.7	7.9
TMEM120A	31.8	35.1	8.8	20.3	18.1	10.7	14.6	34.2	7.0
MINK1	33.7	35.2	9.7	13.6	12.8	10.4	18.3	37.5	8.9
KANK2	33.3	36.9	11.1	14.1	13.8	6.9	17.6	37.3	9.2
PGPEP1	28.9	35.2	8.4	11.6	8.3	8.5	21.8	49.3	8.1
SRSF2	34.8	43.6	10.3	17.1	16.2	12.3	13.6	24.3	7.9
TUBB4A	28.4	33.7	9.9	23.4	24.8	12.7	13.7	26.5	7.1
UBFD1	27.2	35.3	8.4	16.1	14.5	13.2	17.9	39.4	8.0
FURIN	28.6	33.6	9.8	17.1	17.4	10.5	17.1	37.2	8.6
CTNNB1	26.8	40.9	7.1	15.3	12.0	12.5	15.2	43.4	6.7

TWF2	26.7	34.6	8.2	10.6	10.6	9.7	20.0	49.2	10.1
MEA1	25.0	32.4	8.4	19.1	16.8	11.4	18.0	40.8	8.0
TSR3	29.9	37.9	10.4	18.1	16.4	11.0	17.6	30.5	7.8
INTS1	30.8	37.5	9.5	14.1	14.0	10.3	17.2	38.1	8.1
UBE2D3	27.3	41.4	6.2	13.2	12.1	17.2	13.6	40.0	8.5
COTL1	30.0	36.3	9.8	16.3	17.9	18.1	13.5	30.0	7.4
SERINC2	36.3	43.1	13.0	16.4	15.1	7.6	14.0	27.2	6.7
HIST1H3B	22.4	32.0	8.1	43.5	34.4	23.7	3.4	9.8	1.9
SLC35F6	29.4	37.3	10.2	15.0	14.9	8.9	15.9	39.9	7.8
NUTF2	31.6	41.3	9.3	16.0	16.7	11.6	11.9	32.9	7.9
GBA	31.2	40.5	8.7	22.7	21.8	11.8	9.9	26.9	5.7
OBSL1	30.4	37.9	9.1	18.1	17.9	11.7	15.8	31.1	6.8
SZRD1	30.0	32.2	9.9	15.5	17.0	11.0	16.1	36.8	10.3
PDDC1	29.7	39.3	8.9	15.4	14.0	14.3	14.0	34.8	8.2
PPP1R12C	28.9	35.2	10.0	21.6	20.6	14.3	12.9	27.8	7.0
VDAC2	30.3	43.1	10.1	15.7	13.2	12.9	13.4	31.9	7.6
RBM42	30.7	37.6	10.9	15.8	16.2	11.3	15.2	33.1	7.5
OPTN	31.8	35.3	9.4	19.4	15.7	14.4	13.6	32.5	6.0
TM9SF2	24.3	40.3	6.0	12.9	9.1	11.4	15.1	50.5	8.6
SLC35E1	27.4	37.2	10.5	12.4	13.5	7.9	17.7	40.7	10.6
PDGFRB	27.8	33.0	8.2	14.6	12.4	7.7	19.0	46.5	8.7
IQGAP1	33.7	44.9	9.3	12.7	11.4	12.8	11.6	35.3	6.3
ORAI2	25.5	35.3	9.8	17.7	18.6	7.6	15.5	38.8	9.0
AIP	29.6	36.4	9.7	14.9	12.9	11.1	17.6	37.8	7.4
ITFG3	31.5	41.7	9.7	14.2	14.4	9.5	15.6	33.0	7.9
LOC101928247	28.4	38.6	13.3	13.7	16.6	7.9	12.7	37.5	8.7
DDB2	30.2	41.3	9.4	11.9	10.9	7.3	16.6	40.5	9.2
ELK3	27.2	32.6	8.9	16.2	16.3	9.3	15.5	41.3	10.0
FKBP2	27.9	30.9	10.2	18.0	18.1	8.4	22.2	32.2	9.3
PRPF31	30.8	32.7	9.6	16.7	16.0	9.0	17.7	35.7	9.0
MRPL23	30.6	34.1	10.6	15.7	14.1	12.2	19.2	31.9	8.3
NDUFC1	31.9	30.3	12.9	18.5	19.5	7.4	17.6	29.2	9.3
RHOQ	38.3	46.1	12.9	14.5	12.6	8.6	12.0	25.3	6.2
SNRPC	30.7	35.6	9.1	17.1	15.9	10.3	15.6	35.2	7.0
G3BP1	28.3	36.0	6.8	20.9	16.1	15.6	13.5	33.1	6.1
PLOD3	29.9	40.4	9.2	11.9	11.8	8.2	16.9	39.9	8.1
CLPTM1L	30.1	41.0	9.1	18.6	15.1	14.7	12.9	29.4	5.6
DPM2	27.1	36.5	6.4	18.5	18.5	13.4	14.6	36.7	4.5
ABR	30.3	38.2	9.0	13.2	11.9	9.8	16.3	39.6	8.0
MAP3K7CL	40.6	63.3	12.4	4.7	4.6	2.6	10.8	31.0	6.2
PSMF1	26.9	36.9	8.6	15.7	13.2	11.8	16.0	39.1	7.8
PCSK1N	24.9	32.0	10.5	13.0	14.2	9.1	23.4	35.2	13.4
ZNF664	24.2	28.0	7.6	12.3	12.7	8.6	20.6	51.7	9.5
TRNY	19.8	52.1	9.2	11.3	10.5	1.2	18.6	42.9	9.7
OS9	32.5	34.6	9.2	13.0	11.3	12.3	16.8	37.9	7.7
ABCA3	31.4	37.3	10.1	16.9	14.1	8.1	14.9	34.8	7.7
CARS	30.8	38.8	8.8	10.5	9.1	7.0	17.4	44.5	8.1

ARFGAP1	30.1	40.1	8.7	11.4	10.5	8.0	18.4	38.7	8.9
HSPBP1	29.3	37.0	9.9	12.1	12.5	7.7	19.0	39.6	7.7
PUF60	29.6	35.9	9.1	13.1	11.8	8.3	18.8	40.0	8.3
GATAD2A	30.4	30.4	10.8	16.6	18.7	11.8	15.4	31.4	9.1
TOMM7	32.9	29.9	8.2	14.6	11.5	16.2	22.6	32.7	6.2
CCNI	25.6	38.9	6.5	13.5	10.2	10.0	14.3	46.9	8.7
CCT4	28.8	44.8	7.2	16.3	12.8	13.4	11.3	34.1	5.7
ARHGAP23	35.5	43.5	12.6	18.1	16.6	9.7	9.9	23.2	5.4
DVL3	29.1	29.8	9.5	16.2	16.5	10.9	17.3	35.5	9.3
SEC61G	19.0	42.1	3.8	8.7	6.2	17.6	16.9	54.2	5.6
TMEM129	30.0	41.7	8.3	14.3	11.5	6.2	16.3	38.9	6.8
DDB1	28.3	39.5	8.0	12.2	10.7	9.4	15.3	42.6	7.8
RPN2	26.0	34.2	7.6	15.3	13.6	9.7	15.6	44.0	7.8
COX6C	26.8	51.6	7.4	17.2	10.6	12.7	11.0	32.3	4.3
C1QBP	27.5	41.4	7.6	18.3	15.3	11.4	13.3	32.5	6.4
ERH	26.0	42.9	7.3	12.8	14.4	12.6	13.9	35.5	7.9
EIF4A2	25.0	39.7	5.9	20.4	13.5	22.0	10.6	30.7	5.5
TMEM59	24.2	39.2	6.6	14.1	11.5	11.1	15.9	42.3	8.2
CPPED1	32.7	44.9	12.1	15.3	13.8	10.7	11.7	25.4	6.5
FBL	28.2	32.4	9.2	18.2	15.9	14.1	13.7	33.8	7.5
FIS1	27.4	31.8	8.8	16.7	15.9	9.6	18.3	35.5	8.8
KIF3C	27.5	29.7	9.6	20.5	19.2	12.6	15.3	31.2	7.4
SCAMP4	26.8	38.2	8.7	15.2	14.5	11.8	16.1	35.5	5.9
AKT1	27.3	35.4	9.3	15.8	15.0	10.9	15.9	34.7	8.3
VEGFB	32.4	36.3	9.9	18.7	18.4	9.4	14.3	26.0	7.2
DAB2	43.3	57.0	11.4	11.9	10.1	8.4	6.8	19.5	4.3
SON	28.7	41.5	7.0	12.2	9.9	12.4	13.2	40.0	6.9
PSMD7	29.6	40.7	8.0	14.6	12.4	9.3	13.4	37.0	6.7
FAM134A	28.2	36.2	8.4	17.7	15.0	13.1	14.5	31.8	6.8
PYURF	29.3	30.9	10.1	18.8	14.8	9.6	18.4	29.4	10.3
SIGMAR1	29.5	36.2	8.8	17.7	15.9	10.2	15.1	30.6	7.6
PSMD13	26.1	38.3	7.2	15.8	11.3	12.5	14.8	39.2	6.3
CBR1	25.0	37.7	10.1	16.4	16.7	7.5	14.5	33.8	9.5
GIT1	28.8	33.3	9.8	15.6	17.2	10.2	15.5	32.4	8.5
NDST1	26.8	33.3	8.7	17.8	17.6	10.0	14.0	34.8	8.2
NNMT	33.1	56.7	10.1	12.9	8.9	6.5	10.1	28.1	4.6
DYNLT1	28.5	40.9	8.9	16.2	13.5	12.9	12.6	31.7	5.7
SRSF3	28.8	51.6	8.7	16.3	12.8	15.3	8.2	24.3	4.8
AKAP2	31.4	37.2	9.8	23.0	21.0	13.8	8.5	21.3	4.9
FIBP	26.7	34.8	8.7	14.4	14.2	8.6	16.9	37.2	9.3
IER5L	41.8	43.1	14.4	12.7	11.5	3.8	14.6	20.4	8.1
AMPD2	24.0	28.7	6.7	13.4	12.6	9.4	20.0	44.6	11.0
SLC39A3	27.2	35.7	9.9	13.4	11.6	8.5	17.7	38.8	7.2
C1QTNF1	22.6	29.4	6.8	7.9	7.6	4.4	25.2	53.6	12.6
MORF4L2	22.6	37.9	6.4	10.8	9.9	10.1	16.1	46.7	9.6
DCTN1	27.6	33.0	9.0	17.6	15.3	10.5	15.0	34.4	7.5
COX7B	24.7	32.1	8.3	21.7	19.2	7.9	14.7	34.1	7.2

RGS5	20.6	38.2	4.2	5.6	4.9	4.0	19.7	62.3	10.4
TMEM230	25.1	40.4	7.4	14.4	14.1	11.0	11.8	37.7	8.0
ATG9A	27.4	35.3	9.1	11.9	11.7	7.0	16.9	41.9	8.6
LRRC8A	25.2	34.8	8.7	11.7	12.7	6.2	19.1	41.4	9.9
GRHPR	28.3	33.8	9.0	19.1	17.7	13.1	12.3	28.2	7.9
NUBP2	22.8	33.5	7.4	13.3	12.5	10.7	20.8	40.7	7.7
EGFR	35.1	47.7	9.9	17.8	16.9	14.3	6.6	17.9	3.1
HEATR2	29.5	34.3	10.6	14.3	14.1	7.1	15.7	34.4	9.1
EXOC7	28.7	34.0	8.4	15.5	13.8	10.0	15.5	35.9	7.3
PI4KB	27.7	36.5	7.8	15.3	14.0	9.9	14.6	35.8	7.5
SHKBP1	29.4	38.5	9.4	15.8	15.2	11.5	13.9	28.7	6.4
ADAM15	31.5	40.6	9.3	19.3	16.5	9.4	10.5	25.7	5.9
RAB11A	28.7	37.6	7.5	14.9	12.5	11.8	13.6	34.2	7.8
GAS6	20.7	26.2	6.7	26.2	24.7	15.8	13.6	27.8	6.9
SREBF2	30.2	37.8	9.7	14.4	12.6	9.6	13.7	33.6	6.7
HIST2H2BF	23.5	22.1	7.9	25.1	25.1	25.2	11.6	21.6	6.3
KPNB1	24.4	35.8	6.5	16.5	13.3	14.2	12.6	38.6	6.3
DNASE2	30.9	42.1	9.3	14.9	14.2	10.0	11.9	29.1	5.9
CNOT3	30.0	29.3	10.9	13.7	14.8	7.7	19.8	31.8	10.3
DNAJB2	26.8	33.7	8.9	14.4	13.7	10.3	16.2	35.9	8.1
REPIN1	21.0	24.9	6.7	20.8	21.8	13.8	17.3	32.1	9.8
GNA12	28.9	39.7	9.3	13.5	12.4	9.5	14.2	33.8	6.8
MDH1	25.6	41.4	6.5	18.5	13.3	12.8	11.6	33.6	4.8
44811	26.6	35.6	6.2	17.9	13.5	15.5	12.0	34.0	6.7
SMARCE1	28.0	27.0	9.4	16.0	13.2	10.3	18.6	36.1	9.2
PNMA1	35.2	44.7	9.4	12.0	11.5	13.7	10.8	24.6	6.0
ITGB5	26.2	36.1	9.0	16.8	15.4	12.3	13.1	32.4	6.5
DUSP23	22.9	32.7	8.5	19.0	17.7	8.5	17.1	33.4	7.8
ZNHIT1	24.4	39.5	6.5	11.9	9.3	11.5	17.5	38.9	8.1
PDCD6	27.7	34.2	8.8	13.8	12.6	8.6	17.1	37.8	6.9
GABARAPL2	27.0	43.6	6.2	14.6	9.2	12.1	12.8	35.2	6.7
TNRC18	24.0	27.9	9.0	9.4	9.3	7.9	21.8	47.7	10.5
LOC101928600	29.6	48.8	8.8	7.5	6.0	13.1	10.0	38.7	4.8
UBQLN4	28.2	32.9	9.1	14.4	12.6	8.0	14.9	36.6	10.4
ANP32B	27.8	25.5	10.3	18.4	17.5	13.4	17.7	27.5	8.8
SNORA22	23.6	25.4	16.1	16.1	14.8	5.5	27.0	25.1	13.2
SNORA47	19.9	12.0	15.1	16.2	23.5	2.5	36.4	23.4	17.8
SNORA23	16.6	58.9	2.7	5.2	5.0	19.5	13.6	43.2	2.0
IGFBP5	31.0	43.1	13.6	5.5	6.1	2.9	19.0	34.4	11.1
RNF11	29.2	36.6	8.0	15.6	13.3	13.6	12.6	30.8	7.0
EPHX1	19.1	23.1	4.6	25.7	23.0	15.7	13.5	35.6	6.2
TCP1	23.9	40.4	5.9	13.5	8.9	14.5	12.3	40.3	7.0
ENAH	30.2	30.3	8.3	14.9	13.7	9.2	15.2	36.7	8.1
PMPCA	27.7	37.2	7.5	12.3	11.2	8.0	17.2	37.5	7.8
ANKRD52	31.8	40.9	10.0	12.5	11.2	7.5	13.1	33.2	6.2
HIST2H3A	17.8	22.0	6.1	37.6	37.5	27.7	5.0	10.2	2.4
HIST2H3C	17.8	22.0	6.1	37.6	37.5	27.7	5.0	10.2	2.4

HEXB	28.0	41.7	8.2	18.1	14.7	16.4	8.8	25.4	4.9
ARAF	28.4	30.6	8.3	14.1	11.8	8.1	16.7	38.8	9.2
PRMT2	27.5	36.5	7.3	13.5	11.7	11.8	15.6	35.0	7.1
DCTN2	29.1	37.5	9.1	16.1	14.1	8.8	12.7	32.0	6.5
ATP6V0E2	26.1	34.3	8.6	15.6	15.0	9.5	15.5	32.8	8.4
CARM1	27.7	34.3	9.4	16.6	15.3	10.6	14.7	30.1	6.8
RAB35	28.6	33.1	10.2	13.3	13.7	11.0	13.2	34.1	8.5
TRNL1	22.4	33.6	5.2	11.0	7.4	3.1	15.0	63.4	4.3
NUMA1	28.6	30.7	9.7	12.5	11.3	8.9	16.8	38.3	8.6
MAPKAP1	24.5	37.6	6.7	14.0	12.0	12.6	12.8	37.1	7.9
YIF1B	25.5	35.4	8.9	12.7	12.3	9.5	17.2	35.5	8.3
TNFRSF11B	7.7	11.6	1.9	4.1	3.0	2.0	28.3	91.7	14.7
TRIM44	28.2	31.8	9.0	12.7	11.3	9.9	17.7	36.3	8.0
TMEM9	29.4	39.6	7.5	18.9	12.6	6.9	13.8	30.4	5.8
ANTXR1	31.8	37.9	9.1	17.8	14.4	10.3	10.4	27.0	6.0
GTPBP2	32.7	42.6	8.9	9.3	7.4	5.7	15.2	35.6	7.2
MYO18A	25.2	29.3	7.7	16.9	16.2	11.4	15.7	34.2	8.1
HIST1H2AC	24.2	30.3	7.8	15.8	13.5	13.7	15.7	35.5	8.2
WRNIP1	26.8	34.6	8.3	15.2	14.2	10.2	14.7	34.8	5.9
GOLM1	30.0	31.6	9.1	13.8	11.7	8.6	16.1	36.1	7.2
NDUFC2	21.7	39.5	7.5	20.9	14.7	18.9	11.5	22.5	6.7
CTSL	31.2	41.5	8.8	13.9	12.7	11.9	10.6	27.8	5.4
SDHA	27.5	33.9	8.3	16.5	14.6	11.5	13.8	31.5	6.2
PCMT1	24.1	36.2	6.9	15.0	11.9	11.7	14.8	35.9	7.2
DYNC1H1	31.2	43.6	10.4	11.4	10.1	10.3	9.2	33.1	4.3
CD46	23.5	39.8	5.6	14.5	11.1	11.8	11.7	38.9	6.5
SOX12	25.5	28.4	10.3	12.8	14.7	9.1	19.3	33.3	9.9
NUDCD3	25.0	36.1	8.2	12.5	11.8	10.2	15.6	37.9	6.1
ID1	34.0	41.8	10.9	20.1	16.9	8.4	10.6	15.4	5.1
SDCBP	27.5	48.5	6.5	15.3	10.6	11.9	9.3	28.6	5.0
PPM1G	26.7	28.8	9.0	16.4	13.7	10.5	16.4	34.2	7.5
TMEM30A	25.5	37.9	5.9	12.7	10.1	10.8	13.4	39.1	7.7
FMNL1	29.8	36.2	9.9	10.2	10.2	20.2	12.9	26.9	6.5
RTFDC1	27.1	35.5	7.4	15.5	13.6	10.7	13.5	32.6	6.9
TXNRD1	27.7	40.1	6.5	20.2	14.4	17.3	8.4	24.2	4.0
GDI2	22.8	37.2	6.0	13.1	9.4	13.5	12.3	41.3	6.9
KPNA2	27.6	40.4	7.1	20.5	16.8	14.1	7.7	23.9	4.6
AKT2	27.7	34.5	7.7	11.3	10.8	12.1	15.6	35.4	7.2
UHMK1	26.3	41.4	6.7	15.4	10.6	14.6	11.4	31.1	4.9
AGAP3	27.2	33.6	8.9	17.5	14.7	10.8	14.4	28.7	6.1
CDC42EP3	18.5	24.8	6.4	16.1	14.2	11.9	14.6	45.2	10.1
TMX2	25.2	33.0	5.6	15.1	11.7	9.2	14.1	39.6	8.3
NUDC	27.9	28.8	8.9	14.3	13.4	10.3	18.1	32.9	7.1
STX4	30.9	29.6	7.7	11.6	9.3	9.3	19.3	36.6	7.2
ARL2	29.3	34.6	9.6	17.3	19.0	7.1	11.4	25.4	7.7
FASTK	26.0	32.1	8.3	14.0	13.3	9.0	16.2	34.9	7.4
KIFC3	30.6	36.2	9.7	13.6	13.0	7.9	13.8	30.1	6.1

FXR1	28.3	37.0	6.1	16.2	11.6	14.0	11.0	29.9	7.0
MYL6B	24.3	29.3	7.4	22.8	18.0	11.3	13.0	29.2	5.6
TMEM63B	28.2	33.1	8.5	14.3	12.9	8.3	14.2	34.1	7.2
SEPN1	28.0	32.5	8.9	14.2	13.2	8.8	14.4	32.7	8.0
FAM120A	27.8	31.5	8.7	15.4	14.3	9.6	13.3	32.8	7.3
UAP1L1	33.5	45.2	10.3	10.9	11.2	8.0	12.0	23.6	6.0
PNKD	24.8	34.2	8.7	15.5	11.9	10.9	15.5	32.2	6.9
APLP2	28.2	28.0	7.3	17.8	15.3	13.8	12.1	31.8	6.2
COPRS	27.7	33.8	7.6	15.8	14.5	12.2	11.5	31.1	6.4
GSK3A	25.8	35.0	8.1	12.5	11.9	8.0	15.9	35.8	7.5
C9orf16	28.4	34.3	9.2	12.3	11.1	8.5	15.8	32.1	8.7
MIEN1	27.1	31.3	8.7	15.7	14.4	12.7	19.0	26.6	5.0
C3orf37	27.9	34.4	6.8	14.8	12.8	11.0	13.8	32.8	6.1
ANXA3	28.5	45.4	7.4	23.4	18.3	18.1	4.5	12.1	2.4
FRMD4A	27.5	30.8	9.9	7.2	7.9	3.3	19.4	42.9	11.4
MGAT1	21.7	29.4	7.5	12.6	11.7	9.2	18.2	40.6	9.4
ADAR	26.7	31.8	7.1	14.1	11.8	12.3	12.2	36.4	7.8
PRKACA	28.3	31.2	8.3	13.7	12.1	10.2	14.1	35.2	7.0
PGD	26.9	31.4	8.3	15.9	13.9	10.2	14.0	32.3	7.2
SMARCA4	30.0	29.8	11.1	13.0	12.9	7.9	16.0	31.3	8.2
DNM1	27.6	30.6	9.1	14.4	14.3	8.9	14.3	33.5	7.3
SRSF1	25.9	40.4	7.4	12.8	10.8	10.4	14.8	31.1	6.3
C15orf40	25.7	41.7	6.7	10.1	7.6	15.3	10.3	36.7	5.9
KIAA0930	28.1	31.4	8.5	14.8	13.7	10.1	14.4	32.1	6.5
GPM6A	27.6	46.6	5.7	10.2	6.7	8.6	11.8	36.9	5.3
CDK5RAP3	26.4	32.9	5.4	11.3	9.1	7.7	19.3	39.0	7.9
CBX1	26.7	33.6	7.5	14.9	12.5	12.7	13.0	32.2	6.1
RAB6A	23.9	34.3	7.5	11.4	8.1	9.8	14.3	42.3	7.3
NDUFV1	25.7	34.1	7.0	12.8	10.1	10.1	16.9	35.5	6.8
ZNF598	25.0	27.2	9.2	15.0	15.2	7.4	18.1	32.6	9.2
PTGES2	24.6	32.1	8.1	13.8	13.4	7.6	17.3	35.0	7.0
MEIS2	35.7	43.1	9.4	10.9	10.5	8.4	9.9	24.6	6.3
TCEA2	28.3	34.6	9.2	10.4	10.1	6.4	15.7	36.0	8.0
RNF40	26.8	30.5	8.1	12.0	9.9	9.0	17.9	36.4	8.2
PIK3R2	24.9	28.8	9.3	11.8	12.5	8.7	17.6	36.1	9.0
GPS2	24.3	25.2	8.0	15.7	15.1	8.3	16.0	35.0	11.0
WIPF1	23.5	21.4	9.2	6.6	7.6	6.5	21.6	46.9	15.2
CHMP1A	28.1	35.4	8.7	13.8	12.7	8.0	14.6	31.2	5.9
PINK1	24.2	29.5	7.7	24.7	22.2	17.7	8.7	19.2	4.4
SNORD3B-2	29.5	34.8	10.5	7.9	8.6	19.4	22.8	19.1	5.7
SNORD3B-1	29.5	34.8	10.5	7.9	8.6	19.4	22.8	19.1	5.7
DDX56	28.6	37.5	7.4	12.9	10.2	6.9	14.9	33.5	6.4
LIMA1	34.5	46.0	10.0	18.5	16.6	11.4	5.3	12.9	3.1
NCLN	27.4	32.1	9.9	13.3	13.4	7.2	16.1	31.6	7.2
KIRREL	32.4	39.0	10.1	12.8	12.9	8.7	10.9	25.2	5.9
TRNAV16	18.4	28.8	8.2	4.3	2.9	1.1	51.2	38.7	4.3
SSU72	33.4	31.0	8.3	11.4	12.5	7.7	13.7	30.2	9.5

EIF3B	24.4	31.3	7.7	13.5	11.3	10.9	16.2	35.2	7.3
HIST1H3D	24.2	33.6	7.2	17.2	18.4	14.0	12.1	24.3	6.7
DVL1	27.4	30.9	9.7	15.2	14.9	11.2	14.2	27.0	7.1
LAMTOR4	26.6	33.6	9.3	16.7	13.1	8.8	16.4	27.6	5.6
USP11	27.4	33.5	8.4	11.1	10.2	7.6	15.9	36.1	7.4
COPB2	22.4	31.4	5.7	11.1	8.6	9.1	15.1	46.5	7.7
SLMO2-ATP5E	23.3	31.3	7.3	16.3	13.8	10.3	13.6	34.3	7.4
OSMR	29.0	42.0	5.9	18.8	13.2	10.4	5.7	29.0	3.4
STEAP3	18.6	21.1	6.3	15.7	15.5	7.1	20.9	41.7	10.4
SEC24C	25.4	33.8	7.0	9.9	9.5	7.1	15.7	40.8	8.0
ZC3H11A	25.3	34.2	7.1	10.8	8.7	8.2	13.3	42.2	7.4
B4GALT5	26.6	37.5	7.1	11.0	8.4	7.8	13.2	37.4	7.9
PPP2CA	23.4	38.2	6.3	14.4	11.3	13.4	12.4	32.4	5.1
TNK2	24.6	29.4	7.3	12.8	12.7	11.4	15.3	34.8	8.5
MRPS12	25.3	33.2	7.4	13.6	13.1	7.8	14.8	35.9	5.6
MRPL43	25.7	30.6	9.6	15.6	16.0	6.7	16.1	27.7	8.7
LPIN1	27.3	38.5	8.3	7.2	7.7	8.9	12.7	37.7	7.9
TMEM127	25.5	35.6	8.4	10.9	11.3	8.8	14.2	34.5	6.9
WBSCR22	24.6	34.7	8.0	12.8	9.7	14.7	14.2	31.0	6.1
ERF	31.9	33.2	11.0	12.3	11.6	8.1	14.1	25.6	8.0
CLSTN2	10.8	12.4	2.5	2.4	2.2	1.2	27.6	80.7	16.0
TRNAL7	17.7	44.1	6.5	6.3	3.1	1.9	29.2	44.5	2.3
MRPL12	25.4	33.1	7.4	16.7	16.3	11.3	12.7	26.0	6.5
HNRNPM	21.0	28.7	7.6	12.0	11.2	13.0	17.9	37.8	6.3
B3GNT9	23.2	32.8	9.1	11.5	11.8	6.1	16.1	35.2	9.5
PAF1	26.7	26.1	9.0	13.0	13.1	8.4	16.9	33.1	9.1
PRR7	28.9	30.3	12.5	12.6	16.3	5.5	15.4	24.8	9.1
MAPRE1	24.3	37.6	6.4	16.6	11.8	12.4	10.7	30.5	4.9
ZNF703	29.6	32.1	11.5	10.4	9.6	6.3	17.9	28.0	9.5
TMUB2	27.3	32.1	7.0	12.1	11.5	8.4	15.7	33.6	7.3
NR2F6	23.2	29.9	8.5	15.2	13.8	9.4	15.7	31.6	7.3
SHISA4	26.2	35.2	9.5	13.0	14.2	7.6	13.1	28.4	7.2
BNIP3L	24.7	36.8	6.8	13.5	9.3	7.9	13.2	36.1	6.2
GLUD1	25.9	37.3	7.7	11.0	8.9	9.7	14.4	33.7	5.7
C19orf60	26.3	30.9	8.6	13.6	13.7	9.0	16.9	28.1	7.0
HDGFRP2	27.9	25.3	12.0	15.3	15.5	7.5	16.0	26.4	8.2
SMS	26.7	42.9	8.2	14.2	11.0	12.3	8.9	25.0	5.0
SNHG9	29.0	19.7	9.4	11.2	10.6	1.6	27.8	32.8	12.0
SNORA84	10.2	50.9	4.1	9.3	3.8	11.9	23.5	36.4	3.8
RHBDF1	31.1	37.0	10.1	13.7	13.0	8.1	12.9	23.2	4.7
MAP1LC3A	35.1	37.6	10.5	14.5	12.1	7.9	10.4	21.0	4.7
SCYL1	25.3	30.8	8.3	12.2	11.7	8.2	15.2	34.1	7.9
MTPN	19.9	32.6	5.7	13.6	11.8	16.8	12.9	34.0	6.2
DDX41	26.4	28.5	8.8	14.4	13.2	7.6	15.4	31.7	7.4
DDX3X	21.4	30.3	5.4	14.1	10.3	16.1	13.2	36.6	5.9
FAM198B	34.0	46.4	11.1	11.5	10.1	7.5	8.7	19.1	4.7
RHOG	24.4	32.7	8.5	14.0	14.0	9.1	13.3	29.1	7.8

ADAM19	20.0	22.9	6.2	9.0	7.7	6.2	21.1	50.2	9.5
TAF6	26.9	30.0	7.7	13.7	13.6	8.0	13.0	32.9	6.9
IRF2BPL	23.4	28.1	7.8	9.4	9.6	8.9	17.6	37.8	10.1
HCFC1	25.1	26.8	8.8	10.6	11.4	7.2	15.9	37.8	8.9
UBE2V1	23.0	32.7	5.9	15.5	14.3	11.0	12.1	29.5	8.5
MRPS26	27.2	25.6	10.7	15.1	16.3	7.3	16.9	24.1	9.0
TNKS1BP1	26.0	30.3	8.5	11.3	9.5	7.1	17.1	35.2	7.2
RAB13	23.3	29.4	7.0	18.3	15.3	9.3	12.9	30.0	6.5
C9orf142	26.4	30.9	7.3	12.1	9.5	7.6	17.8	32.4	8.1
HMGCS1	31.7	53.2	7.2	7.3	5.4	6.5	9.3	27.3	4.1
GNG12	28.8	38.4	8.0	12.1	9.5	9.1	10.4	29.9	5.7
CHMP1B	24.1	32.5	7.3	12.5	10.5	12.4	12.4	33.2	6.9
CSNK2A1	27.6	24.4	7.3	12.6	11.9	4.8	16.0	38.1	9.1
ISCU	20.7	27.1	6.8	14.9	11.9	14.5	13.1	36.5	6.2
PSMC1	24.3	34.6	6.3	14.0	11.0	14.5	11.4	30.7	4.8
MRPL38	24.8	33.3	7.0	12.9	11.8	8.1	15.0	31.3	7.3
COL18A1	27.7	33.6	9.7	13.0	12.5	10.4	13.1	25.9	5.7
SLC35A4	22.8	35.0	7.2	11.7	11.7	5.5	13.1	36.3	8.1
GPAA1	23.9	32.0	7.5	13.7	11.7	8.4	14.0	32.6	7.6
MTHFD2	23.4	33.7	6.3	9.7	7.7	7.2	14.3	41.2	7.9
SPEG	33.2	42.2	9.7	11.0	10.2	6.4	10.0	22.8	5.8
SUN1	28.2	39.2	8.4	10.8	9.2	8.5	11.7	29.9	5.5
TOMM6	20.2	36.3	6.2	14.8	11.3	14.8	12.8	28.4	6.5
OGFR	23.2	24.5	9.0	13.7	13.1	8.6	17.0	33.4	8.6
KIAA1522	23.7	26.2	8.5	14.6	13.9	7.4	16.2	32.1	8.1
SHFM1	27.1	20.6	8.1	17.2	12.8	16.3	17.4	25.0	6.1
ITM2B	21.0	29.5	6.6	9.7	9.4	12.7	14.4	39.4	7.9
INO80E	22.7	27.2	7.7	14.7	15.0	10.9	13.8	29.6	8.8
PITPNM1	22.5	26.8	7.6	12.5	12.4	9.6	16.3	33.4	9.2
HSD17B10	25.1	33.5	8.2	11.1	10.0	10.9	14.5	31.2	6.0
ZC3H7B	30.0	33.5	9.4	11.5	11.8	7.6	12.5	27.7	6.2
GADD45G	23.2	23.2	5.4	4.7	5.7	2.4	30.4	42.2	13.2
PET100	16.5	27.7	6.5	15.1	9.2	11.7	18.4	39.4	5.9
CGGBP1	22.0	33.8	6.0	10.6	8.5	8.0	12.7	39.3	9.2
NISCH	29.6	34.7	8.7	13.5	13.0	8.8	10.3	25.8	5.5
ORAI3	27.6	38.2	8.2	11.3	10.9	6.1	11.8	29.7	6.0
SH3BP5	31.5	35.5	10.3	8.1	7.1	6.4	13.6	30.7	6.4
HNRNPD	25.7	22.0	8.7	16.1	13.9	11.9	15.8	28.8	6.6
UBE2Z	23.3	29.5	7.4	15.3	15.2	11.1	11.8	29.1	6.4
UBXN1	27.4	27.4	9.2	12.9	13.3	10.6	15.2	26.6	6.7
BRAT1	22.8	29.7	7.4	11.1	10.7	7.7	16.6	35.0	8.2
USMG5	19.5	43.7	6.0	12.7	9.5	15.8	9.4	27.5	5.1
FGF2	27.1	31.5	9.3	21.4	20.0	11.9	8.6	15.0	4.5
TOMM5	20.6	30.5	8.1	13.3	12.1	6.2	14.1	35.1	9.2
TOLLIP	26.5	31.5	8.6	11.4	12.8	7.2	13.2	30.8	7.1
CORO1C	23.9	33.5	7.1	19.4	15.2	11.8	8.9	24.9	4.3
NOSIP	24.4	30.8	9.0	13.1	12.9	9.1	13.5	30.0	6.2

PSMC2	21.8	36.4	5.6	12.9	10.5	12.5	11.4	31.7	6.1
ATP6AP2	22.5	34.2	4.7	12.1	8.5	9.3	13.5	37.8	6.0
SNORA80	10.8	32.0	6.8	10.1	5.9	38.2	16.7	25.9	2.2
JAZF1	37.3	39.5	11.9	11.3	10.4	6.1	8.5	19.3	4.4
SLC16A3	25.4	37.5	8.7	12.9	11.0	12.0	13.4	23.4	4.3
IDH3B	23.5	32.0	8.2	13.6	12.4	9.3	14.3	29.1	6.3
DHX30	24.8	32.3	7.7	12.1	10.9	10.9	12.6	30.4	6.7
MIDN	28.9	29.8	11.0	8.4	9.8	10.1	15.1	26.8	8.7
NDUFB5	20.5	36.2	6.4	13.5	11.7	11.5	12.6	30.2	5.8
PHB2	23.0	32.2	7.4	13.7	11.4	11.1	12.0	30.4	7.1
ARL8A	26.6	35.0	9.4	12.7	10.0	8.3	11.9	28.4	6.1
IFI27L2	25.1	28.1	8.8	15.8	16.5	10.8	13.2	24.1	5.9
MRPL53	22.8	32.9	8.9	14.6	16.0	9.3	12.2	25.0	6.5
PLXNA1	24.7	29.8	8.3	11.6	11.4	6.4	13.7	34.9	7.3
STXBP1	28.8	35.4	8.7	10.8	10.8	6.4	11.2	29.6	6.5
LOC101926889	23.3	45.5	6.1	5.8	4.7	17.3	6.3	34.7	4.3
TSC22D4	20.5	24.6	6.2	21.2	22.6	17.2	8.8	22.1	5.1
RNF145	21.7	36.5	5.6	11.9	8.7	10.3	11.1	36.3	5.7
SUN2	25.1	31.8	8.9	10.9	10.1	12.8	13.2	28.9	6.1
PPP1CB	21.9	36.7	6.1	11.8	8.4	12.6	10.5	34.9	4.9
FOSL2	23.6	23.8	8.6	16.8	15.5	15.2	12.2	26.0	6.2
PIP5K1A	24.6	33.9	7.2	15.2	12.9	10.6	9.1	28.9	5.6
PCNP	25.7	25.5	6.3	13.7	9.3	11.1	15.9	34.0	6.0
COPZ1	22.8	36.2	6.1	12.5	9.2	10.1	11.3	33.1	5.9
SNORA49	16.3	18.9	6.8	11.9	7.5	13.1	30.8	35.2	6.8
C5orf15	21.6	29.4	6.8	12.7	12.8	7.3	12.4	35.9	8.2
LOC101060037	36.8	42.1	8.8	7.8	7.2	1.3	11.7	22.3	8.8
NDUFAF3	23.5	28.5	8.4	17.7	15.5	8.6	12.1	25.8	6.6
MGST3	22.2	37.8	6.3	16.1	13.8	9.1	10.6	25.2	5.5
ZFAND3	27.4	29.9	8.4	12.6	10.9	7.1	12.3	30.5	7.5
GPSM1	25.7	30.4	9.1	13.5	13.8	7.8	13.3	26.6	6.3
FAM127B	27.0	27.3	10.0	9.7	9.9	5.9	17.4	31.9	7.4
CALD1	27.5	25.1	8.9	22.0	17.9	12.1	9.3	19.3	4.3
SNRPD3	27.2	32.5	6.8	12.1	11.9	9.4	11.6	29.0	5.6
BOD1	23.1	30.8	8.0	11.6	10.0	7.2	17.0	32.1	6.4
FAM114A1	20.1	30.4	5.6	12.4	10.5	17.5	11.1	32.9	5.7
TMEM134	26.4	36.4	8.5	10.7	12.4	7.6	11.3	25.4	7.5
MAVS	24.6	28.3	7.8	11.6	11.2	10.5	13.2	31.8	7.1
ELOF1	22.7	29.5	9.9	10.3	10.7	7.4	16.8	30.1	8.6
DAZAP1	25.6	27.6	9.9	15.3	16.5	13.6	9.6	22.2	5.6
LIF	45.7	47.4	12.3	5.4	6.9	2.0	8.3	13.6	4.3
MMADHC	20.2	39.9	4.2	13.7	9.8	12.1	9.9	31.7	4.4
ALDH3A2	24.3	34.3	6.2	11.2	9.8	8.1	11.0	35.1	5.9
SNRNP70	25.3	22.1	9.0	15.1	15.8	15.3	14.9	21.5	6.8
VPS25	25.2	32.2	7.3	13.9	11.9	11.1	11.8	27.6	4.6
RAMP1	15.5	19.9	7.0	16.8	14.3	5.9	21.1	34.5	10.7
PLA2G16	23.6	37.5	6.6	12.8	11.0	9.3	10.7	29.5	4.5

ITPRIPL2	22.0	30.7	7.5	9.3	8.0	6.8	16.5	38.0	6.7
CREB3	16.7	23.2	4.8	10.4	8.7	6.6	17.9	49.0	8.1
SUB1	23.6	34.9	6.4	13.2	9.8	9.5	10.8	29.5	7.8
B4GALT2	24.7	33.4	8.9	13.3	13.8	7.6	11.6	25.6	6.6
RAB10	24.7	34.6	6.1	10.5	9.0	9.6	12.3	30.7	7.8
BECN1	26.7	31.4	5.9	10.5	11.3	9.6	13.0	30.9	6.0
RAB34	23.2	31.6	7.1	13.6	11.9	9.0	11.7	31.3	5.6
NCKAP1	22.7	34.7	5.8	15.3	12.2	10.7	9.0	29.5	5.3
TMEM104	22.2	25.7	7.3	13.1	13.5	7.5	14.0	33.7	8.1
COPB1	21.8	35.5	5.1	10.3	7.0	7.5	11.2	40.2	6.4
RAB5B	26.9	30.2	7.1	14.0	10.1	8.2	12.7	29.3	6.7
LEPREL4	22.0	29.9	8.2	7.2	8.0	6.4	18.4	35.8	9.2
BAP1	24.5	30.6	7.3	11.7	11.5	8.4	13.7	30.9	6.6
HSPA1B	24.2	19.4	8.5	16.7	16.5	12.2	15.7	24.2	7.4
NBR1	23.6	31.3	6.9	11.4	10.0	8.6	11.8	35.0	6.0
PTPMT1	23.4	31.0	8.5	15.4	14.2	11.7	12.5	21.5	6.4
MAP1A	22.2	25.5	6.8	15.0	13.6	8.7	12.5	34.1	6.3
SGK1	20.0	25.4	4.6	33.8	24.9	19.7	4.4	9.7	2.0
VAPA	23.7	34.2	6.2	13.1	10.7	9.9	10.5	29.8	6.2
GAA	27.3	30.1	8.6	12.5	12.6	7.4	12.8	26.4	6.6
PARP1	21.7	25.9	6.0	12.8	11.2	10.0	13.8	36.2	6.6
NGFRAP1	23.9	29.2	7.6	16.1	12.7	11.1	12.6	24.2	6.8
DGKZ	25.0	30.9	8.1	11.1	11.1	10.4	13.1	27.2	7.2
C9orf69	23.7	28.4	7.4	15.4	14.0	6.8	14.7	27.2	6.5
ARL6IP1	20.9	35.9	4.3	15.8	11.3	18.8	8.0	24.8	4.0
NENF	23.4	32.9	8.1	17.1	12.1	8.1	10.3	26.3	5.3
GLG1	22.0	32.5	6.7	9.1	8.4	10.2	13.9	34.6	6.2
PPP1R18	24.9	27.5	8.5	11.1	10.2	10.2	14.0	30.0	7.2
CSPG4	25.6	31.3	8.9	13.1	13.5	8.8	10.3	26.7	5.4
HMGB1	21.7	26.2	5.7	16.8	12.3	14.7	12.1	28.7	5.3
SCAMP2	24.5	28.9	7.8	11.9	11.5	8.1	12.3	29.7	8.8
C17orf62	22.4	32.4	7.4	11.4	10.1	7.2	15.0	32.2	5.4
ZFAND5	19.9	28.4	5.0	12.0	9.9	10.6	11.0	38.2	8.4
PICALM	25.1	33.6	6.9	14.0	13.1	10.7	8.5	25.6	5.6
NELFE	22.9	27.0	7.4	14.8	12.7	8.2	14.0	28.8	7.4
HNRNPA3	26.7	31.2	6.5	15.4	13.3	11.8	9.8	22.8	5.7
ZMAT3	24.3	31.3	6.3	11.7	8.7	7.5	12.1	35.3	5.9
ATP6V0B	19.6	29.5	5.3	14.8	9.7	9.5	13.7	34.1	6.8
CLCN7	26.4	31.6	8.1	13.8	12.4	9.0	11.1	24.8	5.8
TMEM43	25.3	34.0	7.5	11.2	10.4	10.7	10.9	26.7	6.2
PDIA4	21.1	29.0	6.3	11.5	11.3	8.9	13.1	34.8	6.8
LRRC4B	24.5	26.6	9.6	14.0	15.8	6.3	12.7	25.0	8.0
MPV17	25.2	39.9	5.6	10.6	8.4	10.7	11.9	26.2	4.1
C16orf72	22.0	32.4	7.6	10.6	10.7	9.4	12.2	30.2	7.4
C20orf27	27.4	34.5	8.7	18.1	17.1	9.7	7.1	16.3	3.5
NAGLU	25.7	33.5	7.5	11.1	9.3	7.3	12.7	29.2	6.2
NDUFA7	25.3	26.3	10.7	13.9	14.5	5.1	12.5	26.2	8.0

CDC25B	25.9	30.3	8.3	14.3	14.5	11.2	9.2	23.1	5.6
SOCS2	27.2	33.6	8.4	10.8	11.9	7.9	11.4	24.5	6.5
TRAM1	21.7	35.6	6.0	12.9	10.2	10.9	9.6	29.1	6.2
PCOLCE	27.0	30.1	8.8	10.4	10.1	6.3	13.8	27.8	8.1
TMED3	23.5	29.8	7.2	12.3	9.4	6.7	14.8	32.5	5.9
TSSC4	21.1	27.6	6.6	11.0	10.5	7.2	16.6	34.6	6.7
ARPP19	22.1	28.7	6.5	15.9	11.7	11.5	11.7	26.3	7.4
WDR18	25.4	27.8	8.5	9.5	8.9	5.3	16.2	32.4	7.8
STK25	23.5	28.3	7.5	12.8	12.8	8.0	13.3	29.3	6.2
RNPEPL1	25.4	30.2	7.7	11.2	11.0	7.7	13.1	27.8	7.5
CYP51A1	20.5	41.0	4.3	9.8	7.8	10.6	10.5	32.9	4.2
SDF2	18.1	29.8	5.4	14.0	9.7	9.1	15.3	34.8	5.4
OCIAD2	25.6	44.6	5.9	9.6	7.5	9.2	8.3	26.3	4.5
ZFAS1	19.9	34.7	5.7	10.2	9.0	9.1	11.7	35.9	5.2
RANGAP1	22.4	29.2	8.4	15.8	14.6	10.8	10.8	24.7	4.6
SH3BP2	24.6	28.4	7.7	10.3	8.8	5.5	14.3	33.8	8.0
POLR2J	27.7	18.7	10.2	16.7	15.7	8.2	13.7	23.3	7.1
RAB11FIP5	22.1	25.9	7.9	14.3	14.8	9.8	12.6	27.6	6.3
SPATS2L	22.4	30.4	6.6	9.0	8.1	5.5	12.6	37.9	8.6
NDUFV3	24.4	21.5	7.4	11.8	11.4	5.9	18.8	32.2	7.7
FBRS	25.8	23.4	7.8	12.8	11.3	8.1	14.9	28.8	8.4
KIF5B	24.2	31.9	6.3	12.0	10.0	10.4	9.7	30.6	5.8
SMARCC2	25.9	27.4	7.9	11.9	11.9	8.6	12.5	27.9	6.8
CAPZA1	24.5	35.0	6.6	11.8	10.2	10.2	9.6	26.7	6.0
PRPF3	27.3	34.6	6.1	7.9	7.8	5.5	12.4	31.7	7.4
PRKCA	27.4	37.5	8.0	10.1	7.9	11.0	8.9	25.0	4.7
TIMM17A	20.2	30.3	5.5	12.5	9.2	12.3	10.7	34.8	4.9
GRAMD1A	22.2	26.7	8.3	13.1	13.6	9.3	12.2	27.6	7.4
SPPL2B	23.1	30.3	6.8	10.9	10.2	7.8	13.9	30.5	6.8
PIGS	26.8	36.9	8.8	12.5	11.0	7.3	8.8	23.5	4.4
INTS3	25.3	33.3	6.7	9.4	7.7	5.5	13.4	32.2	6.4
APMAP	22.8	30.4	6.9	13.3	11.1	9.4	11.6	28.5	5.9
NEDD8	22.7	20.1	7.9	15.7	14.1	14.2	13.8	25.8	5.6
ANAPC2	24.1	31.4	7.2	10.5	9.3	7.8	14.9	28.3	6.5
NMRAL1	23.9	30.0	7.8	12.1	11.8	8.1	10.7	28.5	6.9
DNPH1	22.5	30.7	8.8	17.9	13.2	12.3	12.0	18.2	4.3
HUWE1	19.7	27.2	6.0	9.6	8.6	8.7	11.8	41.9	6.3
HARS	24.1	27.4	6.4	13.4	9.7	8.6	14.1	30.6	5.3
KCNN4	15.1	21.8	3.9	8.2	7.7	5.9	21.4	47.0	8.6
CD9	22.1	37.4	7.2	14.7	12.9	10.3	8.4	22.1	4.5
RNF10	21.8	28.9	7.0	14.1	10.8	12.0	11.3	27.9	5.7
DDRGK1	21.5	22.9	7.1	10.6	9.3	6.0	18.3	36.6	6.7
TRAM2	26.2	36.6	8.6	11.9	10.1	7.9	8.6	23.8	5.3
IK	24.0	24.4	7.9	11.2	10.2	9.0	15.4	28.7	8.1
PCID2	25.1	37.4	5.9	13.7	9.8	8.0	9.8	24.5	4.7
MED25	24.8	27.7	8.1	11.7	11.7	8.6	12.9	26.2	7.0
LAMTOR5	22.9	32.2	6.8	12.5	10.8	7.8	11.2	27.8	6.6

TPGS2	23.5	31.9	6.5	13.8	13.0	7.5	9.8	26.9	5.8
WBP1	19.3	30.0	7.1	8.9	8.8	7.8	17.0	32.3	7.3
RELA	24.0	24.6	9.1	12.7	13.0	8.5	13.0	25.9	7.8
MBNL2	22.7	32.3	5.7	18.5	16.7	14.6	6.1	17.9	3.8
PXDC1	28.4	34.9	12.0	12.1	12.7	7.4	8.5	18.2	4.0
MAPKAPK2	23.0	24.8	7.0	13.9	12.0	10.1	11.3	29.3	6.7
SLC25A22	24.2	29.2	7.0	12.7	13.0	5.8	12.4	27.4	6.3
CCM2	24.9	29.5	6.9	11.0	10.7	7.8	12.7	27.4	7.0
TSC22D1	12.0	16.7	3.2	7.3	6.0	6.0	23.0	53.6	10.4
TAX1BP1	20.2	34.7	4.8	10.2	7.7	8.2	10.5	35.3	6.4
DNAJC5	24.0	25.2	7.4	11.0	11.6	7.6	14.6	29.0	7.6
TRAF4	20.0	29.4	6.1	11.9	9.9	9.0	12.7	32.9	6.1
TCEB1	22.8	33.6	4.6	12.9	7.7	8.4	10.7	31.9	5.3
VPS72	23.7	29.4	7.2	10.4	9.6	7.4	12.8	30.2	6.9
CLIP3	28.0	34.9	9.6	10.3	9.3	5.6	10.8	24.2	5.1
ELP5	20.5	33.1	6.7	9.9	9.0	4.7	13.7	33.9	6.4
CDR2L	16.9	22.4	7.0	17.5	16.1	9.1	12.8	29.1	6.5
REEP5	18.2	27.5	4.4	10.7	9.9	7.9	13.6	37.9	7.4
PABPN1	24.5	27.5	7.0	11.2	8.5	12.0	13.4	27.1	5.9
MLLT1	24.5	20.7	9.2	11.2	13.9	7.3	14.3	28.1	8.0
ORMDL3	19.6	29.8	5.6	10.6	9.4	6.2	13.1	36.0	6.7
NUDT16L1	21.3	30.6	6.5	11.3	10.0	8.6	16.7	25.5	6.6
ZMIZ2	22.6	25.9	7.1	12.7	12.3	6.8	12.6	30.0	7.0
TNPO2	24.1	30.0	7.3	10.3	9.9	6.2	12.5	30.0	6.6
FAM168A	22.7	20.9	6.8	9.0	9.7	4.7	14.0	37.9	11.1
QARS	22.3	28.3	6.6	12.1	11.2	8.5	12.7	29.0	6.0
UBL4A	22.6	25.9	8.7	14.1	14.5	8.0	13.2	24.1	5.6
CRMP1	24.0	31.2	7.9	11.1	9.2	7.2	10.9	28.3	6.8
HYOU1	19.9	24.4	5.8	8.8	7.5	7.3	15.6	40.1	7.3
CYR61	22.6	28.4	7.1	29.2	27.4	18.0	1.1	2.4	0.6
NDUFA1	24.3	28.3	7.4	11.5	8.3	11.9	12.4	26.7	5.7
MFI2	32.9	37.7	9.3	14.4	14.0	7.3	6.2	11.9	2.8
LOC101926898	24.1	44.5	5.2	5.3	4.4	13.0	5.1	30.8	4.0
SEC31A	22.0	27.9	5.6	8.7	7.4	6.5	12.0	39.1	7.3
POLR2C	20.0	32.0	5.1	11.1	8.7	7.3	12.1	34.5	5.7
BCL7B	21.0	27.4	7.0	12.8	12.1	8.5	12.3	27.5	7.7
C17orf61-PLSCR3	23.9	29.6	7.7	14.5	13.0	8.7	10.5	22.8	5.5
SLC39A13	22.4	30.4	6.9	11.3	11.4	6.9	12.2	29.2	5.5
SIK1	29.5	35.3	8.5	9.8	9.4	15.8	7.6	16.1	4.1
STARD3	23.0	27.9	6.5	10.5	9.7	7.7	14.4	30.0	6.3
TUBG2	25.1	30.0	6.9	7.9	7.9	3.7	14.5	31.7	8.1
UBAP2L	27.3	29.8	6.5	11.7	10.8	8.4	9.9	26.2	5.3
ZBTB7A	23.9	25.7	9.4	10.7	11.8	8.2	13.1	25.4	7.6
LRPAP1	23.3	23.0	8.3	12.3	11.2	7.6	14.9	28.4	6.7
MOGS	20.9	25.7	5.9	9.4	8.6	6.9	15.2	35.4	7.7
C6orf1	29.0	33.2	8.0	10.2	9.7	4.5	10.9	24.5	5.8
DUSP14	26.6	36.3	7.7	12.6	11.2	6.3	8.7	20.8	5.2

LOC100507303	22.2	24.0	4.9	6.9	4.5	4.9	22.4	39.0	6.5
SLC25A11	22.3	29.7	5.9	13.2	12.3	10.1	10.7	26.1	4.7
ELAVL1	21.8	29.3	6.6	12.9	12.3	9.3	10.4	26.7	5.8
SBF1	20.0	25.0	7.0	9.8	9.1	9.2	15.1	32.9	6.9
NAA20	21.4	31.6	6.1	11.0	7.7	10.1	10.6	31.7	4.7
AFF4	21.9	27.8	5.7	11.1	9.8	8.8	10.4	33.1	6.3
SLC25A37	27.8	31.6	8.4	7.9	8.6	5.5	12.7	25.0	7.2
ATP13A1	20.6	25.8	6.0	9.5	9.4	8.9	15.3	32.3	6.9
SELT	19.1	32.0	5.0	11.4	8.2	10.0	11.3	31.2	6.3
FGFR1	19.0	26.0	5.7	7.8	6.9	5.6	13.4	42.9	7.2
KDM1A	18.2	22.1	5.0	23.7	19.0	17.1	7.2	18.4	3.9
SSRP1	20.8	26.8	6.4	14.0	11.4	8.7	11.3	29.0	5.7
CLNS1A	20.7	30.4	6.1	10.7	10.6	8.5	11.9	29.0	6.2
PSENEN	19.8	26.5	4.4	13.2	11.0	9.4	11.0	32.2	6.3
DNLZ	19.0	26.1	7.7	10.1	9.2	6.1	17.4	31.2	7.2
CLSTN1	22.6	26.6	7.3	10.2	9.7	7.0	12.8	31.1	6.5
SF3B3	23.5	34.9	6.1	11.7	9.3	9.1	8.7	26.4	4.0
CDC42EP1	20.0	22.2	7.2	15.5	16.7	9.0	11.5	24.6	6.8
GADD45A	28.8	37.2	10.0	14.9	12.7	9.4	6.3	11.7	2.5
LARP6	21.5	25.6	7.4	10.5	10.3	5.4	13.8	30.5	8.3
POM121C	24.9	26.1	8.2	11.8	12.9	6.4	11.0	25.0	7.0
MRPS16	19.3	29.3	6.3	12.2	10.9	10.7	11.2	28.6	4.5
EFEMP2	24.7	31.3	8.7	17.7	16.9	9.3	6.6	14.0	4.0
NPRL3	25.0	27.3	6.8	10.5	10.5	6.0	11.4	28.3	7.3
PMEPA1	12.4	13.5	4.5	4.9	4.6	2.9	25.2	50.8	14.1
HABP4	33.7	34.5	10.9	12.8	10.8	8.3	6.8	11.4	3.6
CHMP5	20.2	33.1	5.4	12.4	8.5	9.1	10.1	29.0	4.9
SRI	21.3	30.9	5.8	10.7	8.9	7.9	11.2	30.1	5.9
TMED10	21.7	30.9	5.4	12.4	9.4	7.3	11.2	29.2	5.3
RBBP7	20.0	32.4	4.2	12.8	10.4	12.7	8.6	26.6	5.0
CREB3L1	28.0	29.9	9.6	6.6	7.2	3.0	12.5	28.6	7.2
SURF1	18.1	30.5	5.1	10.5	7.5	7.6	14.4	33.8	4.9
TM9SF4	22.2	29.7	6.4	10.0	8.2	7.0	11.6	31.0	6.3
PAK4	22.7	27.2	8.7	12.9	11.5	8.1	10.9	24.1	6.1
COA3	21.8	30.5	6.2	11.7	12.5	10.5	11.0	23.8	4.4
PSME1	19.0	31.1	5.6	10.1	8.6	10.9	12.1	29.0	5.7
UBE2J1	20.2	28.3	5.4	10.4	8.5	9.0	10.2	34.1	5.8
C5orf45	21.3	30.9	6.1	13.4	11.5	8.8	9.7	25.9	4.3
MTA2	20.7	25.0	5.3	9.5	8.8	8.1	13.8	33.8	6.9
DCTN4	21.3	30.5	5.8	9.0	7.9	6.5	11.0	33.5	6.4
TNNC1	44.2	42.5	16.2	10.6	9.6	3.7	2.2	2.2	0.5
HIST1H4C	13.6	26.7	5.6	13.9	13.6	15.2	10.9	28.2	3.9
SH3BP5L	25.7	29.0	8.0	9.6	9.8	5.0	14.0	24.8	5.7
CPSF3L	23.4	30.1	6.8	10.3	9.1	9.8	10.2	26.4	5.5
PTP4A2	19.4	31.8	6.2	10.7	10.2	12.5	8.0	26.5	6.1
SND1	19.2	25.3	5.7	10.5	9.0	8.5	13.1	33.8	6.3
B4GALNT4	24.5	29.4	6.6	10.9	9.6	6.6	13.5	24.6	5.8

LRP3	23.3	29.1	7.7	13.4	14.3	7.3	11.1	20.3	4.9
TMUB1	20.9	23.3	7.1	12.2	13.4	7.5	12.7	26.8	7.2
MLST8	23.7	28.7	7.5	13.3	13.6	7.1	10.1	20.7	6.5
PDXDC1	18.1	24.1	5.4	7.9	6.0	6.0	15.7	40.8	7.2
LINC00152	23.1	35.0	7.4	17.1	14.0	8.6	6.8	16.5	2.6
STAU1	20.3	22.7	6.4	12.1	10.5	8.7	12.0	30.3	7.9
PSG4	37.6	44.8	10.6	14.8	14.2	8.1	0.3	0.4	0.2
MAP3K11	21.3	24.0	7.5	10.7	10.5	8.8	14.3	26.7	7.0
FPGS	23.5	29.1	7.0	15.3	13.6	9.0	9.2	19.9	4.1
JOSD2	18.4	29.7	5.9	12.2	9.7	8.7	12.5	28.1	5.4
AHCYL1	22.8	30.2	6.4	11.0	9.0	8.1	10.7	27.8	4.6
MRPL52	19.5	21.3	6.4	15.9	16.6	8.3	13.7	23.1	6.0
EIF3A	23.1	23.8	7.2	12.1	10.6	11.5	11.8	24.9	5.5
DDX23	22.9	25.9	7.3	9.1	7.4	7.1	15.2	28.8	6.9
DCTN3	20.3	30.6	6.4	12.2	10.6	7.4	11.6	25.8	5.6
MRPS2	18.3	21.6	6.7	10.2	10.5	5.6	16.5	31.7	9.3
SRA1	20.9	27.0	7.5	9.4	8.2	8.3	13.2	29.9	6.0
IMP4	18.2	22.7	6.4	10.1	10.6	7.3	14.7	31.5	8.8
KIAA1967	21.5	27.3	6.3	10.5	9.4	8.0	12.7	28.6	5.8
GPR108	19.3	25.8	5.3	10.7	9.9	5.8	13.7	32.5	7.3
SNRPG	16.9	28.8	4.6	11.5	7.8	13.7	10.0	31.9	5.0
EPHB4	16.9	20.4	5.2	10.0	9.7	6.5	15.8	37.6	7.9
LOC101927061	26.8	37.3	5.8	16.2	13.3	12.7	3.4	10.9	3.7
IKBKB	25.2	29.5	5.0	10.1	6.6	7.0	10.5	30.2	5.8
ADIPOR1	20.8	30.3	5.4	11.0	10.1	7.8	10.8	27.5	6.1
HSD17B12	21.8	35.5	5.2	12.0	9.6	8.7	8.4	24.1	4.6
GSPT1	15.9	24.9	4.7	15.2	11.4	13.9	10.5	28.2	5.3
NMT1	22.8	28.7	6.9	11.4	10.3	8.5	9.9	25.2	6.0
TRA2B	22.5	28.5	6.8	10.7	8.3	10.6	11.6	25.2	5.5
NUDT22	24.5	27.9	7.0	13.4	10.8	7.1	10.5	22.7	5.8
OTUD5	21.6	25.6	6.8	9.2	7.6	8.1	13.4	30.8	6.5
PCBD1	21.4	24.0	7.1	14.0	13.5	8.1	11.3	25.5	4.5
YARS	20.8	30.2	5.8	7.5	6.1	6.1	12.8	33.6	6.5
PA2G4	21.5	27.6	6.9	10.9	10.0	9.2	10.8	26.4	6.1
MRPL55	19.7	26.3	6.7	10.2	10.5	3.8	16.9	28.0	7.2
RAVER1	19.7	23.1	7.4	10.2	11.0	7.3	14.4	28.1	8.1
PSMA5	17.9	28.0	4.9	11.6	8.6	8.8	11.7	33.0	4.9
SLC25A23	21.4	29.4	7.0	11.4	9.9	8.5	11.7	24.7	5.2
C17orf89	21.6	28.1	5.2	13.6	10.7	9.9	11.8	21.8	6.5
ACTR1B	21.6	31.7	5.7	13.4	11.5	10.5	9.2	22.1	3.5
ADI1	18.5	20.9	6.0	12.3	11.3	9.9	14.4	29.9	5.9
SLC6A8	25.4	31.9	7.1	10.2	8.6	7.8	10.0	23.7	4.1
SLC25A5	19.3	27.2	5.7	11.5	9.8	12.5	10.5	27.0	5.2
TAZ	23.6	29.2	6.1	10.5	9.7	6.5	11.8	25.6	5.9
NOTCH2NL	23.5	38.7	6.7	10.0	8.0	8.8	8.0	22.0	3.0
GNG5	23.4	26.5	5.9	11.5	10.2	6.8	8.6	30.5	5.3
IKBKG	23.4	25.0	6.4	11.4	8.2	14.3	12.0	23.6	4.2

TRAP1	22.9	27.4	7.2	11.4	10.7	8.0	10.5	24.2	6.1
CDC42	20.8	30.6	5.1	11.3	8.9	15.5	7.8	23.4	4.9
EXOSC6	23.8	27.3	8.1	8.9	8.0	5.8	14.3	26.9	5.0
HIST1H3I	18.0	22.4	8.2	20.0	20.3	10.7	7.6	15.6	5.2
PEPD	25.6	33.9	8.2	12.3	12.8	8.4	7.0	16.5	3.2
IP6K2	18.9	22.6	5.3	11.1	11.0	7.3	14.1	29.9	7.6
LEPRE1	20.8	26.8	5.7	8.3	7.3	5.3	13.3	33.5	6.8
TCTA	21.7	24.3	5.8	12.3	11.2	8.4	12.7	25.7	5.6
TRNV	18.0	22.7	10.7	6.6	2.8	2.8	27.9	30.9	5.3
TFDP1	18.4	24.9	5.0	15.2	14.4	9.9	10.5	24.3	5.1
DAPK3	19.5	22.2	6.5	13.1	13.0	9.6	12.7	24.9	6.2
FGFRL1	20.4	26.1	7.4	12.0	12.6	6.5	12.3	24.6	5.7
PCDHGB7	18.7	23.7	6.1	5.4	5.0	4.1	15.9	39.6	8.9
THOP1	19.6	25.8	6.2	13.1	13.1	8.8	11.7	24.7	4.5
FLJ20464	21.0	31.3	6.2	8.5	6.1	14.0	8.8	26.8	4.5
TBRG4	22.0	29.0	6.6	9.9	10.0	7.6	11.1	26.6	4.6
MIR4435-1HG	22.5	38.3	7.1	14.6	12.6	10.5	5.7	13.7	2.3
SPAG7	22.4	23.9	7.5	10.0	8.8	5.4	14.6	29.1	5.5
WSB2	22.7	33.9	6.3	11.1	9.8	8.2	8.6	21.7	4.9
UBE2Q1	22.2	23.4	6.6	12.1	10.5	8.0	11.2	28.1	5.0
ZDHHC12	22.5	29.0	8.4	16.9	16.6	7.2	8.5	14.8	3.4
HOMER3	20.4	25.2	6.1	12.0	12.5	8.0	11.8	25.2	6.1
LSM12	22.4	26.8	5.8	9.7	8.7	8.0	10.5	29.3	6.0
FBLIM1	21.5	26.6	6.3	11.1	11.4	6.1	12.6	26.7	4.7
MPLKIP	18.7	26.5	6.7	11.4	10.3	7.9	12.0	25.8	7.6
HIGD1A	20.5	27.7	5.6	14.4	9.8	11.1	7.5	25.9	4.4
HOXB2	24.7	30.7	8.4	10.0	10.0	6.6	11.1	20.1	5.4
MRPL33	20.3	30.1	5.8	14.5	7.3	11.6	8.1	24.1	5.1
TSPYL1	20.8	23.7	7.0	12.3	10.7	9.8	11.9	24.7	5.9
DCAF7	23.0	29.3	7.3	8.3	8.2	6.1	11.7	27.5	5.4
IRF2BP2	24.6	26.0	7.7	8.6	8.8	12.5	11.5	21.5	5.5
HCN2	20.2	22.8	6.7	9.9	9.4	4.4	14.9	30.6	7.8
MAT2A	24.7	33.7	5.6	8.8	7.8	7.6	10.6	23.0	4.8
MED16	21.2	27.0	7.1	10.2	10.9	6.0	12.0	25.9	6.4
POLR2F	20.4	22.2	7.0	12.5	11.9	8.1	12.8	25.6	6.1
WDR45	21.4	29.1	6.7	9.2	8.9	6.8	11.1	27.4	6.0
GJC1	21.1	27.9	5.1	9.9	8.7	7.2	10.5	31.1	4.9
SRM	20.0	24.6	6.6	12.7	11.7	6.5	11.0	26.9	6.4
DIRAS1	24.1	24.0	7.9	11.7	13.0	7.1	11.6	21.2	5.8
ATP13A2	14.7	17.0	3.9	5.0	4.8	4.2	19.6	47.9	9.4
UROD	21.1	27.8	5.8	12.4	10.6	6.5	10.8	25.7	5.6
ZNF768	21.5	26.6	6.8	11.3	12.0	5.8	11.4	23.9	6.9
ESYT1	20.3	28.3	6.1	10.9	9.3	10.9	9.5	26.1	4.6
MANF	17.2	26.6	5.3	10.4	9.6	7.6	11.4	31.4	6.6
HEXIM1	20.7	21.3	7.0	9.6	8.9	7.9	14.0	28.8	7.9
PQBP1	20.5	22.9	5.7	8.0	8.4	6.7	16.2	31.0	6.5
HYAL2	19.8	29.1	6.9	14.2	12.7	6.4	8.9	22.6	5.2

BLVRA	22.0	34.8	5.3	12.4	8.8	9.3	8.6	20.9	3.9
PAM	20.7	32.5	5.9	10.8	8.3	7.8	9.7	25.3	4.8
ANK1	13.8	16.0	4.2	18.0	17.0	10.4	11.9	28.6	5.9
PCYOX1	19.9	26.2	5.7	11.5	9.7	9.9	10.8	26.5	5.5
RNF130	18.3	29.4	5.2	10.8	8.8	10.6	10.4	26.7	5.6
TM9SF3	18.3	28.2	5.3	9.6	6.4	9.8	11.3	31.6	5.3
CCZ1	19.4	27.6	5.2	11.5	9.3	9.6	9.6	28.6	4.9
TARS	19.8	28.7	4.8	10.2	7.3	7.9	10.3	30.9	5.7
SNHG5	15.6	27.9	3.6	11.0	7.2	14.3	10.4	31.1	4.4
SLC52A2	18.8	26.3	6.0	9.1	8.4	6.2	13.4	31.2	6.1
MEX3D	20.0	22.7	8.7	13.6	14.5	8.5	11.0	20.1	6.3
DDIT4	15.7	19.5	3.8	2.6	2.3	10.8	23.0	39.6	8.2
SV2A	23.7	33.8	6.2	8.5	7.2	4.6	10.3	26.1	4.8
VARS	19.8	25.9	6.2	10.1	9.3	7.9	12.3	27.9	6.0
SMARCB1	19.1	21.6	5.1	10.0	9.7	7.4	15.0	30.3	7.2
STMN1	19.7	27.2	6.8	23.3	17.5	12.3	4.4	11.3	2.9
SRGN	14.6	26.1	4.5	16.7	13.2	32.2	4.5	11.8	1.9
ST6GALNAC4	18.2	22.3	5.9	15.1	14.0	8.2	12.4	24.5	4.7
MVB12A	20.6	27.6	6.6	10.6	9.1	8.9	11.2	24.8	5.9
NDUFS2	19.2	27.4	5.8	12.1	10.3	8.2	10.8	26.7	4.7
SBNO2	21.8	25.7	7.4	10.8	11.1	8.6	11.5	23.0	5.2
RNF216	21.0	24.7	5.6	10.4	9.2	7.9	10.8	29.5	6.1
FZR1	22.2	27.6	7.5	9.1	10.2	5.9	11.2	25.2	6.2
MLEC	18.4	23.3	5.6	10.2	9.3	8.6	12.4	31.6	5.7
KDM5C	20.7	25.6	6.1	8.5	7.5	6.9	13.1	30.4	6.3
ATP2A2	19.7	28.7	5.1	11.4	9.1	7.2	9.5	28.7	5.4
KIAA0195	24.4	27.8	7.0	9.5	9.0	5.5	11.1	24.6	6.1
CHP1	23.0	26.3	5.8	8.4	9.4	7.8	10.7	28.1	5.3
KAT2A	21.9	27.8	5.3	12.5	10.3	8.2	10.8	22.1	6.0
HSPA4	20.5	26.2	5.1	12.1	9.6	10.2	9.9	26.4	4.6
CTDSP1	17.8	24.4	6.0	10.1	9.7	7.2	12.8	30.6	6.1
AGPAT3	22.1	26.3	6.2	11.4	10.3	8.1	11.5	24.3	4.4
SH2B1	19.4	25.7	5.7	9.0	7.3	6.8	12.4	32.9	5.4
MSTO1	17.3	20.9	4.4	8.8	7.6	5.1	15.7	37.5	7.3
LOC101928687	28.2	33.1	9.1	15.1	12.1	11.2	4.7	8.6	2.5
TRIP10	20.2	22.7	6.5	15.4	14.0	10.2	9.9	20.6	5.1
PMP22	20.6	31.4	9.3	9.5	8.9	4.4	10.4	22.9	7.0
SPATA20	24.2	30.8	6.8	12.4	11.1	7.5	9.1	17.9	4.5
EFTUD2	21.1	29.4	6.0	10.1	9.1	8.3	9.6	25.8	5.0
BZW1	18.7	29.8	4.6	10.6	7.7	10.3	9.2	28.8	4.6
TMOD1	13.8	19.0	3.4	2.9	2.7	1.2	18.5	52.5	10.4
GNS	23.0	31.6	6.3	10.7	8.1	8.8	7.7	24.1	4.0
ATP1B3	16.2	25.4	3.6	13.5	10.0	12.9	9.0	29.4	4.1
AGPAT1	19.2	25.7	6.5	9.4	7.8	6.8	12.0	30.3	6.4
NELFCD	21.1	27.8	5.9	9.9	8.8	7.7	10.1	26.9	5.8
TNFAIP1	22.9	28.4	7.0	10.9	11.3	7.3	10.3	21.5	4.5
PHLDB1	27.4	27.7	6.8	10.5	10.2	5.7	8.9	22.6	4.1

RBM38	17.8	20.2	7.5	12.4	13.4	12.6	12.2	20.4	7.4
LOC100128531	21.1	35.6	5.4	6.6	4.5	11.7	8.1	27.3	3.6
C6orf106	17.5	26.0	5.3	10.9	10.2	10.3	11.2	27.7	4.6
CDC42SE1	19.8	21.3	4.5	9.5	8.1	12.0	12.6	30.4	5.5
ACTR3	18.5	28.8	4.8	10.5	9.1	12.7	9.3	25.2	4.8
PIP4K2B	22.3	26.7	6.4	9.4	8.8	6.1	11.1	26.5	6.1
SRP68	19.3	27.4	5.5	9.6	8.1	8.1	11.0	29.4	5.1
TMEM54	22.6	28.8	8.8	14.5	14.2	7.1	7.7	15.2	4.6
OVCA2	19.3	27.2	5.1	12.3	10.2	8.6	10.8	24.2	5.6
KLC2	21.7	25.3	6.8	10.1	9.4	6.7	11.8	25.5	6.0
SART1	21.7	19.1	8.2	10.1	10.1	6.6	15.7	24.5	7.0
GOT2	19.0	27.7	4.9	13.2	10.5	9.7	8.7	25.1	4.2
RNF213	21.2	28.4	6.4	10.4	9.4	9.7	8.4	25.2	3.8
PIP5K1C	20.6	24.4	7.5	11.2	10.9	6.0	11.6	24.0	6.8
LOC101928493	22.7	28.8	6.1	9.8	7.9	5.9	10.4	26.8	4.4
DCTD	19.0	28.2	5.6	10.5	8.9	8.8	9.4	28.0	4.4
ERCC1	21.0	26.8	6.4	10.1	11.3	5.4	11.6	23.8	6.3
URM1	18.3	28.7	5.7	10.7	8.6	7.0	12.6	25.8	5.4
SAMD4B	20.0	24.6	6.8	10.7	9.9	6.2	11.4	27.1	6.0
CNPPD1	20.3	28.6	6.6	8.8	9.4	9.7	9.3	24.3	5.6
TRNM	11.2	36.4	15.5	7.1	4.1	4.0	21.7	20.9	1.9
FAM84B	19.9	26.5	6.0	5.3	5.0	4.1	15.9	33.4	6.4
MME	26.7	45.0	6.0	4.1	3.4	3.8	6.6	23.0	3.9
C20orf24	18.1	19.2	6.6	13.6	12.6	10.1	13.7	23.6	5.0
HMGN2	22.8	22.2	5.7	12.9	11.6	12.5	8.0	22.2	4.5
C11orf68	20.5	29.9	7.5	11.1	10.4	7.3	10.4	21.5	3.9
EXTL3	18.4	26.2	5.8	9.0	8.5	6.9	11.6	30.4	5.7
EPS8L2	21.8	24.6	6.5	16.5	13.9	9.3	9.4	16.1	4.3
NSFL1C	20.9	26.2	6.3	10.3	10.9	9.3	10.0	23.0	5.2
ARHGAP18	22.3	30.7	5.9	8.7	6.6	6.7	10.3	25.5	5.7
PRDX4	16.8	30.9	5.0	9.9	6.8	6.2	12.2	29.0	5.5
TIMM17B	18.8	27.7	5.3	9.7	8.2	11.1	11.6	24.7	5.1
ATN1	22.9	21.3	6.9	11.8	11.6	5.0	12.1	23.5	7.1
GTF2IRD1	24.8	29.2	7.6	10.6	9.8	5.2	9.2	20.5	5.4
JUNB	12.2	13.5	4.4	5.3	3.8	45.8	10.7	20.8	5.7
SLCO4A1	10.6	13.1	3.3	13.7	13.6	8.0	16.4	34.6	8.9
CIAO1	19.5	28.8	5.3	10.5	9.4	7.3	10.4	25.2	5.7
SCAP	18.8	24.8	5.6	9.4	8.6	8.3	12.6	27.8	6.1
ARPC4	17.8	25.3	4.7	10.6	11.3	9.9	11.3	24.7	6.3
TOMM20	20.0	27.7	5.2	9.4	6.3	10.0	10.9	27.8	4.6
SHARPIN	20.2	24.4	7.1	11.7	11.1	6.3	11.2	24.1	5.7
FAM104A	21.5	16.9	7.0	11.9	13.8	7.4	13.0	22.9	7.2
WDR13	20.7	26.7	6.5	8.6	7.6	6.1	14.0	26.1	5.4
YWHAH	18.2	24.0	5.9	14.9	13.9	12.4	8.2	20.0	4.3
ATP8B2	19.8	24.8	5.0	11.0	9.2	9.1	10.2	27.7	4.6
FAM20C	16.6	18.8	5.7	7.5	6.2	3.7	17.6	37.0	8.5
LOC101928091	35.9	26.5	18.2	4.7	3.6	1.5	9.1	12.0	10.0

MTCH2	17.8	28.1	4.8	12.8	8.5	10.6	9.0	26.3	3.6
GALNT2	20.4	25.9	6.3	9.5	8.4	8.7	11.5	25.7	5.0
SIPA1L3	22.6	26.3	8.3	9.9	11.1	6.5	9.3	21.6	5.7
CTDSP2	19.7	25.6	5.7	7.5	6.4	7.7	11.2	32.2	5.4
ATF6B	21.2	23.4	5.9	13.7	11.9	9.9	9.2	20.9	5.2
EMC4	17.9	26.2	5.1	12.2	9.3	7.8	11.9	26.4	4.3
CNPY2	18.7	23.3	5.5	9.7	8.5	8.7	11.5	29.8	5.4
UBALD2	23.2	20.5	8.7	9.9	9.8	9.3	10.6	21.1	8.1
ACSS2	27.1	37.2	6.9	10.3	8.4	6.3	6.1	15.6	3.2
SCRN2	21.4	28.6	6.9	12.0	10.4	7.1	10.4	19.9	4.3
PTPN14	17.6	20.1	5.8	10.4	10.3	7.2	11.7	31.6	6.4
GALK1	19.6	23.9	6.7	13.1	11.0	6.8	11.7	23.2	5.1
AP2A2	20.7	24.7	6.7	11.5	10.8	7.8	9.8	23.5	5.5
DGUOK	19.8	28.7	6.1	9.4	8.2	6.4	11.0	26.4	5.0
ST5	27.5	33.5	7.4	8.0	7.3	4.9	7.5	20.8	4.0
LIX1L	21.4	27.4	6.3	8.4	7.8	9.2	10.8	24.6	4.9
NRSN2	20.7	27.5	5.9	10.3	9.2	8.3	10.9	23.9	4.1
UBE2E3	16.7	25.9	4.4	10.3	9.3	9.0	9.8	29.2	6.1
DNPEP	20.3	24.8	5.8	8.7	6.6	5.9	12.6	30.2	5.7
NDFIP1	19.3	30.2	4.8	8.8	7.3	7.6	8.7	28.2	5.7
SDCCAG3	23.5	27.0	6.3	9.3	9.5	5.5	10.6	23.1	5.9
ABHD12	24.9	32.3	6.9	11.7	11.5	8.1	6.7	15.3	3.2
LOC100144595	22.7	33.5	5.7	6.5	5.4	11.1	8.8	23.4	3.4
ARL8B	22.6	30.0	6.7	9.5	8.5	8.3	9.3	21.6	4.0
TMED7	17.4	26.5	4.8	10.4	7.3	6.9	10.9	30.1	6.1
MLX	21.8	25.4	6.0	10.2	8.5	6.4	11.7	24.4	5.9
SLC38A10	16.9	21.5	5.8	9.9	9.8	6.5	12.8	31.0	6.3
SLC2A6	22.3	30.2	6.7	11.8	11.0	5.9	9.0	19.8	3.8
RBM39	21.6	27.1	5.1	9.0	6.9	9.8	10.1	25.3	5.2
RPL26L1	20.7	25.3	7.7	14.8	11.9	7.2	8.8	19.3	4.7
TSC2	19.9	25.1	6.3	9.6	9.7	7.5	11.0	25.9	5.3
MZF1	18.4	25.7	5.7	7.0	5.4	3.2	14.9	32.7	7.2
RRAGA	18.3	24.2	5.2	11.3	9.8	7.0	10.9	28.3	5.1
RUVBL1	19.1	26.7	4.9	10.9	10.0	8.5	9.5	25.7	4.7
RAF1	21.2	27.8	5.2	12.4	10.6	10.0	7.9	20.8	4.2
DUT	18.5	29.1	5.8	10.6	8.9	9.4	11.0	22.1	4.8
RAB2A	20.3	26.0	5.1	9.8	8.1	7.7	9.3	28.5	5.2
FLYWCH2	20.8	22.9	9.2	9.5	10.3	5.0	11.8	22.0	8.4
USP5	19.4	25.9	5.5	9.1	8.2	7.7	11.2	27.8	5.0
UBXN4	19.0	27.6	4.5	10.1	8.0	10.2	9.2	27.1	4.2
SPNS1	24.5	30.5	7.5	7.9	7.8	5.4	9.8	21.7	4.7
LOC101928785	21.4	32.2	5.1	6.9	5.4	9.8	7.5	27.6	3.9
SPTSSA	16.7	24.8	5.8	8.2	6.6	5.6	15.4	31.6	5.0
TRUB2	18.7	25.4	6.0	10.7	10.3	6.1	11.1	26.7	4.8
NAPA	19.7	25.7	5.8	8.2	7.5	6.6	12.1	27.2	6.8
PAFAH1B3	19.8	27.7	6.8	14.1	14.2	7.0	8.6	17.6	3.9
NIPSNAP1	20.3	27.8	6.1	12.1	9.0	8.7	8.9	22.6	4.2

FLOT2	21.6	29.6	7.3	11.8	9.9	9.5	8.1	18.3	3.5
TAF7	20.3	23.5	6.1	9.4	7.7	6.9	11.2	28.7	5.7
LOC101929402	21.4	30.1	5.5	6.7	4.8	12.8	8.0	26.5	3.7
NPC1	25.8	37.9	6.4	9.5	8.1	7.1	5.2	16.8	2.6
TAF9	19.7	28.5	5.1	11.1	8.6	7.1	8.0	25.5	5.6
DUS1L	19.5	24.4	5.9	10.9	10.2	8.0	11.4	22.2	6.6
NUMBL	21.8	27.0	7.7	10.9	9.9	6.1	10.1	20.3	5.3
NACC2	15.7	20.4	5.9	11.9	13.5	6.5	11.7	26.4	7.1
TMEM154	23.8	35.8	6.3	4.4	3.1	4.9	7.9	28.7	4.1
UBAC1	19.0	25.1	6.2	12.0	10.4	7.5	9.7	23.6	5.3
PABPC4	17.7	23.0	5.6	11.0	9.9	8.1	10.2	28.2	5.2
C11orf24	17.5	18.7	5.7	8.1	9.0	2.9	13.8	34.3	8.8
VHL	21.4	26.7	6.3	8.3	5.4	7.3	12.8	25.3	5.3
CS	21.3	27.7	5.6	10.7	8.2	7.4	9.9	23.7	4.1
WIZ	19.4	24.5	5.6	9.1	8.7	5.4	11.4	29.0	5.5
STK24	17.3	22.7	5.9	10.6	9.3	10.7	10.3	26.2	5.6
OLA1	17.0	27.5	3.8	10.5	7.7	9.8	8.3	29.0	5.1
ERAL1	18.5	25.9	5.8	10.6	9.1	7.4	10.1	26.2	4.9
ARRDC1	22.1	27.5	7.5	11.1	10.3	5.8	9.2	20.3	4.6
PHF1	19.0	22.5	4.8	9.7	9.1	9.1	12.9	25.3	5.9
PRPF6	21.3	26.2	6.8	8.3	8.4	6.8	11.7	24.3	4.4
TOB1	16.4	20.6	4.8	9.4	9.0	8.1	11.1	31.0	7.8
FAM102A	17.1	20.2	5.8	13.4	12.6	10.8	9.6	23.1	5.5
ETF1	18.6	26.0	4.3	11.1	7.4	10.7	8.7	26.7	4.5
GTF3C1	20.3	25.0	6.4	8.5	8.2	6.6	10.6	27.3	5.1
SCRIB	19.5	22.8	6.5	10.8	10.3	8.0	12.1	23.0	5.2
TEX264	18.1	24.4	5.5	10.3	8.5	6.9	11.2	27.9	4.9
CD2BP2	20.7	25.9	5.8	8.9	8.2	9.6	10.4	24.1	4.3
PCYT1A	21.3	23.7	5.3	10.3	8.3	8.5	10.2	25.3	4.8
MLLT6	20.9	24.0	5.9	9.3	7.4	8.2	11.1	25.1	6.0
LRCH4	19.4	20.4	6.4	10.4	11.1	11.6	11.3	21.4	5.8
DAP3	20.9	27.2	4.5	12.3	8.8	8.1	8.0	23.1	4.8
IMP3	18.5	26.5	6.0	9.0	9.5	4.6	12.8	25.3	5.5
TMCO1	16.6	29.4	4.2	9.8	7.8	6.9	9.4	27.6	5.9
NPTN	18.0	23.6	5.1	11.0	7.0	9.3	10.1	28.3	5.1
ARCN1	16.8	26.7	5.1	8.1	6.8	7.7	10.1	30.9	5.2
HIST1H2BG	18.7	23.2	7.2	16.9	14.2	7.8	8.4	17.5	3.5
VGLL4	18.2	16.9	5.3	9.4	10.7	5.0	13.6	29.6	8.6
ASNS	18.2	27.5	3.7	5.3	4.3	4.4	10.6	37.2	6.0
APEH	18.6	26.3	5.7	11.9	10.8	7.7	9.0	22.6	4.4
RAB15	18.3	22.9	6.2	11.8	10.3	6.5	11.2	23.5	6.3
NAP1L4	18.4	29.2	4.6	9.3	7.8	9.8	9.3	23.4	5.2
RANBP3	20.7	24.6	5.6	10.7	9.1	6.9	10.1	23.0	6.2
SPTLC1	18.5	27.7	5.3	9.0	7.4	7.4	9.6	26.9	5.0
WLS	18.4	26.7	4.5	4.4	3.9	3.1	12.4	37.6	5.8
C11orf49	19.1	24.7	5.8	10.3	10.2	6.1	10.3	24.6	5.7
PLEKHH3	17.9	22.0	6.2	7.1	7.6	4.8	14.9	29.1	6.9

ABI2	22.8	26.9	7.7	10.3	9.4	6.3	7.1	21.6	4.5
NDUFV2	17.2	28.5	4.3	11.0	8.4	10.2	8.8	24.0	4.2
COA4	18.6	24.9	7.8	12.7	10.8	4.7	9.6	21.1	6.2
MOB3A	20.3	26.8	6.2	9.2	8.0	9.1	9.3	22.5	5.0
P2RX5-TAX1BP3	20.6	28.0	6.5	10.4	9.0	8.8	8.3	21.1	3.8
NID1	21.6	26.6	7.2	7.9	7.6	4.8	10.3	25.4	5.0
ASAH1	20.6	30.5	5.7	10.6	10.4	10.8	6.6	18.2	3.1
ACAT2	20.8	32.1	5.8	12.0	7.7	9.0	6.7	18.7	3.5
LOC100506792	19.0	29.0	6.2	9.1	5.8	9.3	9.7	24.1	4.2
APLP1	20.3	24.7	6.6	12.6	11.4	7.4	9.3	19.7	4.4
TBC1D2	26.4	30.9	7.9	15.8	16.2	9.4	2.7	5.5	1.5
CDC42EP2	20.3	23.6	7.1	7.5	8.1	5.0	10.4	28.8	5.2
XAB2	21.5	25.0	6.3	7.9	7.6	5.4	13.2	22.6	6.5
SLC6A9	13.5	15.0	4.3	8.2	7.8	4.8	15.3	39.2	8.0
AMZ2	18.7	28.2	4.3	10.9	8.4	8.2	8.1	24.9	4.4
MTDH	18.9	25.2	4.5	11.2	7.5	10.1	8.1	25.6	5.1
SUPT4H1	19.3	27.6	5.3	9.7	9.3	7.6	9.0	23.1	5.1
ZNF217	18.7	26.3	4.8	8.2	7.3	6.1	9.7	29.0	5.7
EIF3D	20.1	28.7	5.9	8.6	7.3	9.6	8.9	22.0	4.7
SERINC1	17.4	28.2	4.6	10.0	7.6	8.4	8.2	27.1	4.4
CEBPG	18.7	26.3	4.6	7.2	5.8	4.5	11.0	32.4	5.4
ZCCHC24	16.5	20.8	4.8	7.2	6.7	4.2	13.9	34.8	6.8
SAR1A	16.6	25.6	4.0	9.8	6.6	5.6	10.9	30.3	6.2
HNF1A-AS1	20.2	30.4	5.3	7.2	5.6	12.7	7.6	23.2	3.5
TSPAN17	18.0	22.9	6.1	13.0	10.7	7.8	10.3	22.8	4.0
MAGEF1	15.5	21.8	5.6	8.5	8.0	5.2	13.2	30.4	7.1
NKIRAS2	19.1	24.1	6.2	9.3	8.4	9.0	11.5	23.5	4.0
SLC29A4	20.2	22.5	7.0	12.0	12.6	5.3	9.6	20.9	5.2
RNF44	19.4	21.2	7.1	8.1	9.3	4.8	12.2	25.6	7.5
RUFY1	18.7	24.0	5.5	10.9	9.0	7.5	10.7	23.9	5.0
ZER1	18.8	24.3	6.0	9.0	8.9	6.1	10.6	26.0	5.6
FZD2	19.6	24.4	7.2	12.0	13.0	5.3	8.5	20.1	5.1
NDUFS3	15.9	23.6	5.3	12.0	8.8	8.5	11.6	24.5	5.1
VMP1	20.1	27.5	3.8	10.0	6.1	7.5	10.2	25.3	4.4
TTC3	18.8	23.5	2.8	5.6	5.0	5.3	10.5	37.9	5.5
EIF3H	20.5	22.3	5.9	11.1	7.5	11.4	9.4	22.0	4.7
TOMM40	16.9	23.9	6.3	9.3	9.0	6.6	11.6	26.1	5.1
INO80B-WBP1	22.2	25.2	5.9	8.9	7.3	4.7	11.4	23.5	5.8
MYO9B	18.9	23.4	6.3	9.0	8.8	9.7	10.0	23.4	5.4
MRPL9	16.9	23.7	6.0	12.4	9.4	10.9	10.3	20.7	4.6
MSMO1	19.4	32.4	3.9	10.6	7.6	8.8	5.3	23.1	3.7
ETV5	20.6	24.0	5.1	6.3	5.9	3.6	11.5	30.9	6.7
HMOX2	18.7	26.3	5.6	9.5	8.9	7.1	9.8	24.0	4.5
HDAC5	20.9	23.9	6.9	8.3	8.4	5.5	11.6	23.4	5.6
MBNL1	17.3	24.3	4.7	9.6	9.3	9.8	8.0	26.2	5.3
NT5DC2	15.2	18.8	5.3	9.8	10.0	4.4	12.7	30.3	8.0
FTSJ1	18.4	21.2	5.6	10.2	9.2	7.2	10.8	25.4	6.2

DVL2	22.3	23.3	6.4	11.5	10.2	7.3	9.2	19.7	4.0
TUSC1	19.5	23.8	8.6	11.4	11.0	7.9	10.4	16.4	5.0
UNC119	19.2	26.6	6.0	8.8	8.4	6.9	10.1	21.3	6.7
CD74	0.7	1.1	0.1	0.2	0.2	110.0	0.4	1.1	0.2
TRPC4AP	19.5	26.4	4.8	9.7	8.0	8.5	8.8	23.7	4.3
FAM134C	16.7	25.5	5.2	8.1	6.8	6.9	10.9	28.8	5.0
KLF6	19.1	23.6	6.0	8.1	7.2	24.2	6.5	15.3	3.8
ELOVL5	18.8	23.0	4.7	8.1	6.9	9.9	10.1	26.2	6.2
TOR1AIP2	16.2	23.5	4.7	9.4	7.1	7.7	9.8	30.5	4.9
CYFIP1	21.3	29.3	6.0	9.1	7.6	5.2	8.5	22.6	4.3
MSANTD3	15.6	29.8	3.2	24.5	13.7	8.6	3.9	12.8	1.8
PLCB3	20.5	23.6	7.1	10.4	10.4	6.1	9.9	20.2	5.6
ALKBH6	20.8	25.3	6.7	11.8	15.0	6.8	6.7	16.1	4.7
SRSF7	18.5	29.7	4.8	9.7	9.1	14.3	6.9	17.4	3.4
CALCOCO2	19.4	24.5	5.0	9.3	7.1	6.7	9.5	27.0	5.3
SULF2	10.3	10.8	3.2	7.8	10.6	4.2	15.6	42.8	8.5
BIN1	17.2	17.6	5.4	10.9	11.3	8.8	12.0	24.1	6.3
NUDT21	19.8	25.7	5.2	9.2	8.4	8.7	8.5	22.4	5.8
WDR45B	17.6	24.1	5.3	9.3	9.1	5.4	11.1	26.1	5.6
BLOC1S5-TXNDC5	16.5	23.7	4.4	7.3	5.6	4.6	11.9	33.6	6.1
ARFIP2	16.4	21.5	5.4	10.6	8.3	5.2	12.5	27.6	5.8
AP3S1	19.1	23.5	6.4	13.0	12.2	8.8	8.4	18.1	3.9
ATP2B4	20.8	29.1	6.3	10.1	8.0	8.2	7.0	20.2	3.6
CCDC50	17.8	25.5	5.7	11.6	10.2	9.5	7.5	21.4	4.1
HIST1H2BO	16.0	17.8	7.0	22.0	22.8	11.7	3.4	10.0	2.4
MXD4	16.0	18.0	5.6	7.4	7.7	7.1	14.4	29.3	7.8
IMPDH2	17.7	22.4	5.0	11.6	10.2	8.8	9.0	23.6	4.8
COX7A2	14.8	28.9	4.2	10.7	8.0	12.2	7.8	23.5	3.0
SYDE1	19.0	24.7	6.8	14.0	14.3	9.8	6.6	14.9	3.0
DEF8	21.0	26.5	6.2	9.8	8.7	6.4	10.4	19.5	4.5
LOC284023	19.6	28.4	5.3	7.0	4.7	12.3	8.1	23.9	3.7
SCARNA16	6.3	42.1	3.2	5.9	3.3	14.1	8.1	28.1	1.8
SERPINB6	17.1	26.5	5.2	10.4	9.9	7.7	8.7	22.6	4.8
GSTO1	16.9	31.2	4.5	11.8	7.8	11.0	7.5	19.5	2.5
SLC16A1	14.9	22.1	3.3	4.6	3.5	4.1	12.0	41.3	7.0
PSPH	17.7	24.1	4.7	8.8	9.1	5.8	9.9	27.5	5.2
FAM129A	17.3	23.1	5.1	6.4	5.8	5.4	12.3	32.2	5.2
LOC101927812	20.6	21.5	7.6	8.9	8.0	8.8	11.2	21.2	5.0
NDUFA8	18.0	21.4	5.5	12.4	10.4	8.9	9.6	22.5	4.0
PITX1	22.2	22.8	7.6	10.9	10.7	6.3	8.9	18.5	4.7
BAZ1B	16.1	20.6	4.4	11.1	9.4	9.2	9.7	27.1	5.1
GYPC	18.7	22.0	6.2	12.2	12.4	6.0	8.6	21.3	5.2
ECI1	19.4	25.3	6.0	10.3	9.8	7.3	9.5	20.2	4.7
TRIO	22.2	29.4	7.1	8.6	7.5	6.0	7.4	20.5	3.8
MRPL24	18.6	20.8	6.8	11.8	12.3	6.1	9.9	20.5	5.6
HEXA	20.7	28.6	6.0	12.9	10.2	6.8	7.1	17.0	3.1
LOC728392	22.1	29.3	7.2	11.8	10.5	7.3	7.0	13.2	3.8

PLEKHO1	22.5	26.8	7.3	10.5	10.2	7.5	7.3	16.0	4.1
NECAP2	18.7	27.0	6.0	8.5	9.1	8.5	9.0	20.5	4.7
GAS5	15.1	31.2	4.6	9.9	6.1	8.9	9.2	24.9	2.3
SLC39A14	17.9	23.2	4.4	10.3	8.2	6.5	10.0	27.4	4.0
ANKRD1	13.7	16.9	3.1	27.4	25.2	22.8	0.5	1.8	0.5
IDUA	20.2	23.5	6.8	8.8	9.0	5.7	11.0	20.1	6.9
NAA60	20.4	24.1	5.3	9.3	8.9	4.7	10.2	24.3	4.6
MRPL49	18.1	24.0	5.3	10.1	8.2	7.8	9.8	23.7	4.8
ZMIZ1	15.9	16.2	5.9	6.5	7.4	4.6	13.5	33.6	8.2
WDR6	19.0	23.9	5.2	8.6	7.8	6.0	11.2	25.0	5.0
XBP1	16.7	23.2	4.7	6.4	5.4	5.6	12.2	30.2	7.3
CACNG4	21.3	26.4	8.0	8.9	9.1	4.1	9.1	19.3	5.3
IMPDH1	18.0	24.1	5.7	9.1	9.5	6.6	10.0	23.6	5.0
SPCS2	16.0	22.2	4.3	9.0	6.8	9.0	11.0	28.3	5.0
MAN2B1	17.1	22.1	5.0	11.4	10.4	11.1	9.1	20.7	4.7
GET4	19.3	23.6	6.1	9.9	9.6	7.5	9.8	21.0	4.8
TMEM14C	14.8	28.4	4.1	11.8	7.4	14.1	6.7	20.7	3.6
PTPN11	18.4	25.0	4.6	9.9	7.8	10.3	8.1	23.2	4.3
MRPS7	19.7	22.8	6.4	10.8	9.7	7.4	8.5	21.8	4.5
GAS2L1	17.5	21.2	6.3	8.2	8.9	5.4	12.8	25.0	6.2
TBC1D10B	16.5	20.2	6.0	11.2	9.3	8.8	11.1	23.4	4.8
LOC284454	20.7	29.6	5.1	6.6	3.8	8.3	9.0	24.0	4.3
PRCC	16.7	22.0	5.6	9.4	10.0	7.5	9.8	24.5	5.8
CHMP2A	20.4	25.7	7.3	9.8	8.1	6.8	9.1	19.1	5.0
PDCD5	19.7	17.6	5.2	11.3	11.0	8.6	10.1	22.9	4.9
MRP63	16.7	23.7	5.1	11.9	9.2	6.0	10.0	22.6	6.0
EIF4E2	20.1	21.6	6.0	8.6	7.4	7.5	12.2	22.0	5.8
PRPF19	19.8	23.4	5.6	9.4	8.2	6.6	9.0	23.6	5.6
APBB1	17.9	23.2	5.9	10.3	9.8	7.1	9.8	22.5	4.5
NOL7	19.8	19.4	7.6	9.4	9.3	6.5	13.3	20.7	5.0
ALG3	15.9	17.9	4.5	9.0	8.8	4.7	12.0	31.0	7.3
CD70	17.9	24.5	6.2	16.5	15.6	7.3	6.3	12.8	3.9
PLBD2	19.6	26.1	6.3	10.6	10.7	7.0	8.3	18.5	3.7
C9orf172	18.8	23.1	7.3	8.5	7.8	3.8	12.0	22.4	7.0
UBE2D2	17.2	27.6	4.0	11.1	7.8	9.2	6.9	22.3	4.7
ABHD8	17.2	20.9	6.0	11.5	10.7	6.5	10.4	22.0	5.3
BAIAP2	22.9	25.1	8.5	10.8	11.4	6.8	6.6	15.1	3.5
PIH1D1	16.3	19.9	4.8	8.5	8.3	7.9	11.1	26.9	6.8
TRIM16	16.1	20.3	4.7	8.8	6.3	7.1	11.6	30.4	5.3
ELOVL6	12.6	19.5	2.8	5.1	2.9	2.3	14.1	44.8	6.4
MAEA	18.7	21.7	5.5	10.5	10.6	6.7	10.0	21.2	5.4
BRD2	16.9	21.4	5.1	8.2	8.0	11.4	9.5	24.4	5.4
GNPTG	18.8	22.3	5.7	11.3	11.3	9.4	8.6	18.8	4.0
NAV1	17.4	20.6	5.7	7.9	7.7	4.8	10.6	30.0	5.7
TMEM256	18.3	24.6	6.2	13.7	9.0	6.6	8.6	18.9	4.6
RCN2	16.5	27.2	4.8	10.5	8.6	7.6	8.0	21.8	5.2
SNF8	16.8	23.5	5.3	13.0	8.3	8.8	9.6	21.0	4.0

PREB	14.6	20.1	4.7	9.3	8.4	7.6	11.3	28.5	5.8
CYTH2	17.3	21.5	5.4	6.3	5.8	5.4	13.3	28.9	6.2
TMCO3	17.0	22.9	5.0	12.8	10.2	8.8	8.1	21.2	4.1
PSIP1	18.9	20.2	5.6	11.1	10.0	8.2	8.9	21.7	5.3
BAZ2A	18.7	24.0	5.9	7.6	6.5	6.2	9.1	26.8	5.1
PTRHD1	17.0	28.3	7.3	7.1	7.6	5.7	9.6	20.9	6.3
SRCAP	18.2	21.4	5.9	8.0	7.7	6.1	9.8	27.4	5.4
CYSTM1	17.9	21.3	4.3	14.1	12.2	8.5	9.2	18.7	3.6
MRPL51	16.2	23.9	4.0	10.3	7.8	11.4	8.4	23.9	3.8
SNHG16	14.2	21.6	4.4	8.4	6.8	6.6	10.9	31.2	5.6
SRSF6	18.0	24.3	5.3	9.1	8.5	9.0	9.0	22.2	4.2
UBE2E1	19.4	25.0	4.4	11.2	10.2	7.1	6.3	21.3	4.7
SEMA3C	19.9	28.0	5.0	7.5	6.6	6.2	8.7	22.8	4.9
SEC62	17.7	19.7	4.9	8.2	6.8	7.9	11.2	26.8	6.3
YY1AP1	17.8	25.2	4.3	6.7	7.6	4.5	9.3	29.0	5.0
MUC1	21.4	20.8	8.6	18.3	17.7	6.5	4.2	8.7	3.3
SDHC	17.7	29.2	4.4	11.0	7.9	9.5	6.8	19.3	3.6
EIF3L	17.6	24.0	4.7	9.1	8.3	9.5	9.1	21.8	5.0
SNORA8	6.2	29.1	5.0	4.3	4.0	10.2	18.6	28.3	3.4
EPRS	17.6	25.6	3.9	7.4	5.6	7.2	8.7	28.7	4.4
TAP1	19.5	24.5	5.1	7.8	6.7	10.8	8.9	21.7	4.2
ELL2	21.9	31.1	6.5	12.6	11.3	9.8	4.3	9.0	2.9
NANS	17.5	21.8	5.0	9.8	8.4	6.4	11.0	24.9	4.5
NR2F2	21.6	23.5	8.1	10.9	11.5	5.3	7.7	15.9	4.6
RAB14	18.2	25.5	3.9	8.5	6.9	9.1	7.8	25.1	3.9
SETD7	18.4	26.7	5.8	7.9	7.0	5.5	8.4	24.5	4.7
CYTH3	24.9	27.3	9.3	10.7	10.7	7.5	5.3	10.6	2.6
ZFPL1	17.3	19.3	6.2	7.9	7.2	4.6	11.9	27.0	7.5
NDRG3	18.6	22.5	4.2	9.7	8.4	7.4	8.6	25.6	3.9
SAFB	17.9	17.5	7.0	8.6	7.9	8.9	12.4	22.3	6.0
IFRD2	15.5	22.6	4.9	9.5	9.0	6.3	12.6	23.8	4.5
LOC101928783	19.6	25.9	6.8	7.5	7.9	4.8	8.8	23.3	3.8
GFPT1	15.9	26.7	4.0	7.1	4.9	5.9	8.8	30.5	4.8
CETN2	14.8	22.7	4.3	9.5	7.8	9.2	10.1	24.4	5.5
KCMF1	18.0	24.6	5.3	11.0	9.7	7.6	7.9	19.6	4.6
PBXIP1	18.9	20.2	5.8	8.9	7.4	9.2	11.4	22.6	4.0
STAG3L5P-PVRIG2P-PILRB	21.6	26.3	5.2	5.9	4.9	3.5	11.5	23.6	5.9
ZFR	19.0	23.4	4.9	9.6	7.7	6.9	8.3	23.6	4.8
ATP6V1E1	19.2	26.9	5.2	11.2	7.1	7.3	7.2	21.2	2.9
TFPT	18.1	20.8	6.3	10.0	10.8	6.3	11.3	19.4	5.0
PITPNA	15.8	20.2	4.8	7.6	7.6	10.7	10.2	26.2	5.0
PURB	18.5	21.9	6.4	8.7	7.2	7.9	9.9	21.8	5.7
MPST	17.1	22.4	5.9	10.5	10.3	7.1	9.0	19.4	6.3
HIST1H2AG	11.9	17.9	6.3	20.4	23.9	14.2	3.0	7.8	2.6
NOTCH2	23.6	31.7	5.4	7.0	5.8	5.6	5.9	19.4	3.5
MYEOV2	13.7	25.1	5.7	11.9	9.5	10.0	10.7	17.8	3.5
HNRNPAB	16.0	18.4	5.0	13.3	10.7	10.4	9.1	20.0	4.8

C16orf45	12.4	16.4	3.4	7.2	5.3	4.6	14.9	35.5	8.0
PSME3	17.2	23.9	4.5	9.4	8.3	8.2	8.5	23.7	4.0
TPBG	10.6	14.7	4.3	8.1	8.0	4.7	13.3	35.3	8.3
NEK6	22.3	25.3	7.7	9.9	10.0	5.0	6.6	16.6	4.0
LINC00493	19.0	23.2	5.5	9.0	8.3	4.7	8.5	21.9	7.3
ADH5	17.9	28.5	5.0	10.9	6.7	9.1	6.8	19.6	2.9
NFE2L2	19.9	27.6	4.7	8.5	6.8	6.4	6.7	22.2	4.6
HNRNPUL2	21.0	14.3	4.1	8.9	7.7	11.7	9.7	24.5	5.5
DOK1	17.3	20.7	6.8	7.8	8.2	7.4	11.8	22.4	5.0
NRP1	15.8	22.5	4.2	3.4	2.7	3.2	11.7	37.9	5.9
EDEM1	14.9	23.2	4.1	5.8	5.3	9.2	10.8	29.5	4.6
TMEM171	14.1	21.2	3.5	13.4	11.6	9.7	8.3	20.9	4.5
CASC3	17.9	22.0	5.3	8.6	6.9	6.9	10.4	24.0	5.3
MRPL14	15.6	23.2	5.0	10.0	8.3	8.7	9.3	23.5	3.7
CHPF2	16.3	21.1	4.9	8.3	6.7	5.6	11.9	27.1	5.3
HIST1H4D	13.1	23.9	6.2	10.1	9.6	9.8	8.3	21.7	4.4
TUBA4A	15.2	21.6	4.8	14.1	14.1	9.9	6.8	16.9	3.7
RFNG	18.4	20.1	6.0	11.8	10.0	8.5	8.8	19.3	4.2
SLC27A4	17.4	21.6	5.3	10.8	10.7	7.1	8.7	20.8	4.6
KRT81	8.9	9.7	3.6	13.8	14.2	7.9	17.0	23.9	8.0
PHF23	16.2	17.8	5.3	9.1	9.6	6.3	10.8	25.9	5.9
EMC10	14.0	19.4	4.4	7.8	8.7	8.1	13.9	25.1	5.4
RANBP1	15.3	18.4	5.7	13.6	12.2	6.2	8.9	20.8	5.8
NFKBIA	9.7	12.8	3.6	7.7	8.0	37.8	6.8	17.0	3.3
SAT1	17.0	22.1	4.2	5.0	4.0	7.5	11.2	28.6	6.9
RBM3	19.7	29.0	6.0	5.5	3.9	8.5	9.1	21.6	3.3
BCKDHA	17.8	23.8	5.6	7.3	7.1	5.5	11.0	22.7	5.8
TWSG1	20.2	31.0	4.5	9.1	7.1	7.4	7.0	17.4	2.8
GLIS2	16.6	21.2	5.7	10.1	10.0	6.0	10.1	21.0	5.8
CNPY3	19.0	20.1	6.0	8.6	8.2	7.8	10.0	21.6	5.1
ARL2-SNX15	18.3	22.0	6.8	9.7	9.5	7.1	9.2	19.1	4.6
EIF3M	15.3	28.5	4.1	8.6	6.2	7.3	7.3	24.0	5.0
USF1	18.3	19.7	4.8	6.7	6.0	5.6	12.4	27.6	5.3
PAIP1	16.9	23.7	4.5	9.4	7.4	7.6	8.7	23.4	4.6
MOV10	21.3	25.9	5.8	8.6	7.4	6.4	7.7	19.2	3.8
FXR2	16.8	23.3	5.0	8.1	7.3	6.9	9.4	24.6	4.8
CAMK2N1	17.7	18.9	5.6	5.7	6.0	3.6	12.0	28.7	7.8
DLG1	19.9	29.6	4.9	10.8	8.9	8.3	5.0	15.7	3.1
SSSCA1	16.7	18.8	6.2	9.4	8.6	5.3	12.3	23.1	5.7
GSK3B	19.9	27.9	5.4	9.1	7.2	7.0	6.7	19.0	3.7
ZMYND11	17.5	23.2	5.3	8.2	7.3	7.9	8.6	23.3	4.7
C15orf52	20.9	20.8	7.7	10.3	10.1	7.6	8.4	16.2	3.9
BACH1	19.4	30.8	4.5	7.0	5.2	5.4	7.4	22.4	3.8
SOCS3	20.9	21.4	6.7	7.4	8.1	9.9	10.2	16.3	4.7
RBM4	18.6	21.1	4.9	10.0	9.2	7.6	8.1	22.6	3.7
SRPR	15.2	21.3	4.1	7.2	6.2	6.8	10.7	29.3	4.9
NUFIP2	19.9	20.2	5.4	8.6	7.9	8.2	8.7	21.6	5.0

PAGR1	17.1	22.0	5.0	8.0	7.2	5.1	12.5	23.8	5.0
PRR14	16.0	18.5	5.9	8.7	9.4	4.9	11.3	24.0	7.0
C12orf75	22.2	26.2	7.5	12.1	10.6	9.9	5.3	9.8	2.2
IGSF8	21.2	28.0	7.1	8.7	7.0	6.0	7.3	16.9	3.3
TIMM50	18.3	25.0	5.5	11.6	9.1	8.2	8.0	15.4	4.7
ATG4D	17.6	23.4	5.8	8.0	8.6	4.8	10.6	22.4	4.4
LSM14A	18.9	21.6	6.1	8.4	8.3	6.9	8.8	22.7	4.0
CLN3	19.3	23.0	5.7	11.3	10.5	6.3	8.4	16.6	4.4
TIMP3	3.5	3.8	0.8	7.9	6.7	4.4	17.7	50.5	10.1
CHCHD5	19.4	22.8	8.2	9.6	11.1	4.2	8.9	16.1	5.0
RHOBTB3	18.5	29.3	4.3	11.8	8.6	8.8	5.2	16.2	2.6
DNAJC7	18.5	23.3	5.6	8.1	7.0	6.9	9.0	21.8	5.1
SNAR-A13	17.6	17.8	9.0	9.6	5.4	10.2	21.1	11.1	3.4
SNAR-A12	17.6	17.8	9.0	9.6	5.4	10.2	21.1	11.1	3.4
POMGNT1	18.1	23.7	5.1	9.3	8.5	5.9	9.2	21.4	4.0
SDC2	16.6	28.2	5.2	10.8	10.3	8.1	6.1	16.7	3.2
NPEPPS	17.1	25.9	4.4	9.7	6.7	7.7	7.4	21.7	4.5
TGFBR2	9.1	12.5	3.0	2.7	2.4	5.4	16.6	46.3	7.0
PPARD	22.1	26.7	5.9	6.1	5.8	5.1	9.4	20.9	3.2
DNASE1L1	20.8	30.3	5.9	9.8	10.0	4.7	6.3	14.6	2.8
USP39	17.9	24.7	5.0	8.9	7.7	6.3	8.7	21.4	4.4
RBM15B	16.8	17.4	6.1	8.8	9.2	5.7	11.6	23.5	5.9
HRAS	16.3	20.6	6.0	9.3	9.9	6.2	11.1	21.6	4.0
MMS19	17.8	23.6	5.1	8.1	7.0	5.5	8.6	23.6	5.5
STT3A	15.5	20.9	4.1	8.5	7.5	7.5	9.0	27.5	4.5
ACBD3	17.1	20.9	5.3	7.2	6.1	6.8	10.7	26.2	4.6
PLXNA3	20.4	24.4	5.6	8.0	7.8	4.5	9.0	20.7	4.4
HHLA3	18.3	24.9	6.1	14.2	12.3	7.6	4.7	13.4	3.2
FBXW4	18.1	19.5	5.8	9.7	8.5	7.1	9.9	21.7	4.6
C1orf86	18.2	23.8	5.6	7.3	8.2	7.4	8.8	21.4	4.1
DENR	16.6	23.8	4.4	7.7	6.2	10.4	8.8	22.3	4.5
C12orf10	18.5	21.1	6.1	7.8	7.5	4.2	10.7	23.3	5.5
TINAGL1	14.3	15.5	4.8	23.6	23.5	12.4	2.9	6.0	1.6
CDC42EP4	16.8	18.1	5.0	9.3	8.2	4.6	11.2	27.1	4.4
TMEM141	16.3	21.2	5.9	14.9	10.4	5.6	8.2	18.4	3.8
LDB1	18.7	20.1	5.4	7.5	8.2	6.1	10.9	21.8	6.1
SPR	13.2	22.0	4.9	9.9	9.0	8.6	10.4	22.7	4.0
LLGL1	16.1	21.8	5.6	10.4	9.7	6.4	9.1	21.3	4.2
CXXC5	16.8	21.8	6.3	6.1	6.8	4.8	11.7	24.0	6.2
RAB11FIP3	18.6	25.7	5.7	6.4	7.3	5.2	9.4	21.5	4.8
PLOD1	17.0	21.0	5.5	9.1	8.8	5.5	9.5	22.9	5.2
RBM34	18.5	24.3	4.9	7.3	5.5	9.6	7.9	22.4	4.0
PPP1R9B	15.6	17.6	6.0	7.9	7.1	10.6	12.4	22.2	4.8
ATP5F1	17.8	21.7	4.3	10.0	8.3	7.2	9.8	20.3	5.0
TRNAA10	12.1	22.9	7.7	5.7	3.9	2.4	19.3	27.2	3.1
HOXB3	23.9	26.9	7.6	4.9	5.1	2.8	8.6	19.3	5.2
RAP1B	18.4	27.9	3.8	9.0	7.1	10.3	5.8	17.7	4.1

PDCD6IP	18.5	25.7	5.0	8.5	7.6	7.8	7.1	20.0	4.0
MRPL54	17.1	22.5	6.3	6.1	5.4	6.4	11.0	23.2	6.2
PPP1R7	19.0	20.6	5.5	9.1	8.2	7.7	9.7	20.4	3.9
MYL9	22.1	25.8	7.1	12.0	11.2	7.4	5.0	10.4	3.1
C5orf24	14.4	19.5	4.5	8.7	8.5	6.6	9.7	26.7	5.3
TERF2IP	16.6	21.9	5.3	7.5	6.4	8.5	10.5	22.7	4.7
UNC45A	18.1	22.9	5.5	9.1	8.0	7.0	9.5	20.0	3.9
HIST1H1B	13.0	18.5	6.3	22.3	20.1	14.8	3.1	4.7	1.1
GSTK1	17.4	24.5	4.5	11.0	10.7	5.8	6.3	18.3	5.3
RFC1	8.5	12.2	2.0	13.0	11.3	12.3	8.7	30.1	5.7
MSI2	14.4	24.1	4.6	8.0	6.8	7.9	9.4	23.3	5.2
RNF126	14.2	17.4	5.5	9.0	11.1	7.3	12.2	21.7	5.4
INPP5K	18.2	24.2	5.0	6.4	5.9	4.5	11.0	23.6	4.8
PARVA	18.6	26.2	5.6	9.7	8.6	6.0	7.3	17.4	4.2
PTGES3	19.1	20.7	5.8	8.0	7.5	10.7	9.1	18.7	4.1
DYNLL2	15.6	19.1	5.4	8.8	8.8	4.6	11.1	23.7	6.6
TSG101	17.9	24.1	5.3	8.2	8.0	5.5	8.5	21.0	5.2
PPP2CB	17.3	25.9	5.1	9.0	8.6	7.4	7.1	19.9	3.4
RGMB	17.8	21.6	6.2	7.9	7.4	3.6	10.1	23.9	5.1
CCND3	13.5	19.2	4.2	11.9	10.8	11.8	8.3	18.4	5.5
RBMX	14.8	20.5	5.1	10.1	9.1	8.5	8.4	22.8	4.2
KHDRBS1	16.4	20.6	5.8	9.2	8.0	8.3	9.2	20.7	5.2
UBE2K	17.5	22.2	3.9	9.2	8.6	9.1	7.8	21.6	3.6
NCOR1	15.5	23.4	6.1	8.2	7.0	7.3	8.1	23.2	4.4
ABHD11	18.4	25.0	5.3	8.2	5.5	9.2	7.2	21.2	3.1
MBTPS1	16.5	22.6	4.1	9.3	8.0	7.6	8.0	22.7	4.4
HNRNPUL2-BSCL2	16.2	22.0	6.8	9.1	8.9	3.3	11.6	20.1	5.2
BRD4	19.3	17.0	6.7	9.0	9.6	7.3	9.5	19.1	5.8
BCKDK	17.2	21.3	5.6	12.0	12.7	5.9	7.3	16.9	4.2
NT5C2	18.2	25.1	4.5	13.0	11.5	11.0	4.0	13.1	2.7
MRPL20	16.2	23.6	5.4	7.8	7.6	7.0	9.6	21.2	4.6
VASP	16.4	19.0	5.5	10.2	9.8	12.1	8.8	17.6	3.7
ETFA	15.9	24.4	4.1	8.8	6.6	7.3	8.3	22.8	4.6
HADHA	15.6	24.4	4.2	8.0	7.4	7.2	7.8	23.8	4.6
ECSIT	14.4	18.5	4.2	5.6	4.9	4.9	14.9	30.0	5.6
TIAL1	15.5	25.2	3.8	8.0	6.5	7.8	7.3	24.0	4.6
FNDC3B	16.3	22.3	4.5	8.6	7.7	6.3	7.8	24.8	4.4
CELF1	17.4	23.6	5.5	8.4	7.6	7.6	7.3	21.0	4.4
SNORA38	4.5	27.4	1.6	4.6	2.3	6.0	18.7	36.2	1.2
POLD4	19.1	23.7	6.1	9.4	10.4	6.6	7.6	15.7	4.0
DCAF6	15.9	21.3	3.9	9.4	7.3	6.3	8.4	24.7	5.5
FAM101B	18.0	16.0	7.0	19.3	20.2	13.0	2.7	4.7	1.7
GATSL2	16.9	20.0	5.5	8.2	6.7	4.1	10.0	26.0	5.2
ECI2	17.8	27.0	5.2	11.3	8.1	9.0	5.9	15.6	2.8
TRNN	16.2	22.4	5.6	6.6	6.9	1.6	16.3	25.2	1.8
GJA1	10.8	17.6	3.1	3.4	2.9	2.6	13.4	41.1	7.6
LARS	16.2	25.6	3.7	7.4	5.5	6.9	7.3	26.2	3.5

MAN2A2	16.3	19.1	4.3	10.9	9.7	7.8	8.8	21.2	4.3
GPR124	20.9	21.8	6.3	3.9	4.2	2.1	12.4	24.9	5.9
NCOA4	13.6	23.4	3.7	9.9	8.1	9.1	7.5	22.7	4.2
GUSB	18.7	24.5	4.6	9.5	7.7	9.2	7.9	16.6	3.6
TIMMDC1	14.3	24.6	3.6	8.6	6.8	8.8	7.9	24.1	3.5
NES	15.4	19.5	5.7	5.4	4.6	3.2	13.6	29.3	5.6
UBE2R2	18.7	19.5	6.7	9.4	8.8	6.5	8.8	18.7	5.0
SMARCD2	17.3	22.0	5.7	8.2	8.1	5.5	8.9	21.5	5.0
TRIM41	18.6	21.0	5.8	6.6	6.2	4.2	11.6	22.6	5.5
RPL41	16.1	9.4	7.1	14.1	14.9	4.7	12.1	18.5	5.2
FAM173A	18.4	22.3	6.8	10.4	8.3	6.5	9.1	16.1	4.2
CC2D1A	18.0	19.3	5.8	9.0	9.5	5.3	9.8	20.4	5.0
BCL7C	15.9	19.9	8.1	11.0	9.8	6.0	8.2	18.1	5.1
TMEM123	14.7	22.4	4.7	13.6	11.9	9.4	4.8	16.5	4.0
STARD10	14.5	18.7	4.2	13.4	11.6	6.9	9.3	18.8	4.5
B3GAT3	16.5	22.1	5.0	9.0	8.1	5.7	9.4	21.4	4.6
ACSL3	19.7	30.3	4.1	9.4	6.8	9.0	4.5	15.7	2.5
TYK2	16.1	20.7	4.7	8.4	8.1	6.8	10.1	22.1	4.6
NAT14	15.4	28.8	5.9	10.0	7.0	6.5	9.0	17.0	2.2
SNRPA	20.8	22.2	5.9	10.0	9.0	6.3	7.4	15.0	5.2
DEDD	16.3	24.6	4.6	9.2	7.2	4.9	8.2	22.2	4.5
RNASEK-C17orf49	18.8	20.3	6.6	10.0	8.5	4.8	9.1	18.6	4.9
TRIM25	19.5	22.6	6.1	6.4	6.7	5.1	9.1	20.1	6.0
RASSF3	15.2	21.4	4.4	8.4	6.1	7.3	8.9	24.4	5.5
PVR	17.9	22.7	5.6	9.9	8.5	7.0	8.2	18.0	3.5
SYVN1	15.6	18.3	5.4	6.2	5.5	4.0	11.8	27.9	6.6
LAPTM4B	16.6	27.6	5.6	7.7	6.8	5.8	8.9	18.5	3.7
RDH11	16.6	23.0	4.5	7.9	5.4	4.1	10.1	26.2	3.3
GSS	18.2	23.6	5.3	10.1	8.2	5.4	7.4	19.2	3.8
HP1BP3	16.5	21.4	3.9	7.9	6.0	9.5	7.9	24.0	4.2
NDUFB2	10.0	21.7	2.9	9.5	7.2	8.4	10.4	27.0	4.0
ARL2BP	14.7	24.5	3.5	7.6	6.5	6.6	7.7	25.3	4.7
CSK	15.5	19.3	4.9	9.0	8.2	9.3	9.9	20.5	4.5
RNF135	18.2	21.8	5.7	8.9	7.6	4.0	8.9	20.8	5.2
HIST1H2AM	13.5	17.3	6.0	13.1	14.0	12.1	6.4	13.9	4.7
EHMT1	17.1	20.3	5.8	7.2	6.9	5.7	9.5	23.6	4.9
SS18	16.9	17.6	5.1	8.0	8.4	5.1	9.3	24.8	5.6
NABP2	15.0	17.9	3.9	8.4	8.3	6.7	11.1	23.4	6.2
ENTPD4	14.5	23.4	3.4	7.3	6.5	8.7	7.3	25.2	4.6
DNTTIP1	12.2	14.6	3.5	6.8	7.0	4.4	13.5	32.0	6.9
AHSA1	17.6	18.7	5.2	8.5	8.0	6.3	9.5	21.9	5.1
HNRNPR	16.2	20.3	4.9	10.0	8.4	7.1	8.1	22.3	3.6
PRRC2B	17.3	20.3	5.9	8.4	8.0	9.7	7.8	19.2	4.3
CCSER2	18.3	23.6	3.6	9.4	7.2	7.1	6.2	21.8	3.6
DTD1	17.4	24.1	4.4	8.5	6.3	5.9	8.1	23.0	3.2
SNRPE	11.5	28.5	3.7	8.7	5.1	13.7	6.6	19.6	3.2
HDAC7	18.6	21.6	5.2	7.2	7.1	6.1	9.4	20.4	5.0

POGZ	19.2	23.4	4.4	6.1	5.6	4.7	8.8	24.1	4.2
TBXA2R	19.5	26.5	5.1	7.0	5.5	6.4	7.7	19.4	3.4
C19orf66	15.0	19.9	5.3	10.1	9.2	6.3	9.0	20.1	5.5
TMEM189	17.7	22.1	5.7	7.7	7.7	4.7	9.0	21.0	4.8
FOXK2	16.6	20.4	5.7	8.7	8.5	6.1	8.9	20.2	5.4
E2F4	16.3	20.7	5.3	6.8	8.0	5.6	10.7	21.5	5.5
FAM192A	18.4	20.8	5.0	8.9	7.6	5.7	8.1	21.0	4.9
DLG5	16.0	19.2	5.4	8.1	7.9	5.5	10.1	23.0	5.1
CYB5R1	16.4	22.2	4.3	10.0	9.3	8.2	7.2	19.3	3.5
PAFAH1B2	14.2	21.5	4.9	10.0	8.6	7.0	9.8	20.2	4.1
MICA	19.0	24.9	6.8	8.0	7.3	5.1	6.9	18.5	3.6
ZNF687	16.2	19.8	4.8	8.6	8.5	5.5	9.5	22.6	4.6
POP7	15.2	19.8	4.8	10.6	10.8	7.1	9.0	17.5	5.4
TEAD1	15.0	20.3	4.7	10.5	9.5	8.9	6.9	20.6	3.6
RSU1	14.2	19.2	4.5	9.8	7.9	7.5	8.3	23.6	5.1
ELAC2	15.7	20.3	5.6	8.7	8.1	6.2	9.0	21.5	4.9
ARL1	12.6	23.0	3.5	7.4	5.6	5.4	8.7	28.2	5.5
HOXB5	21.3	24.1	8.6	7.1	6.2	3.1	7.9	16.7	5.1
SPSB3	16.5	21.5	5.2	7.9	8.0	7.0	9.9	19.3	4.8
PAK2	17.0	21.9	4.5	8.6	7.5	7.4	6.6	22.4	4.1
SMPD4	17.5	19.7	5.2	9.3	8.5	6.1	8.8	20.1	4.8
AGBL5	16.4	22.5	4.7	9.4	9.7	5.5	7.2	20.4	4.1
ARHGEF17	19.3	23.5	6.0	8.3	7.8	5.0	8.2	17.4	4.3
PPP1CC	13.1	21.2	3.0	9.0	5.9	9.8	8.1	26.3	3.4
XRCC1	15.3	18.1	5.1	7.6	7.3	6.4	10.9	23.3	5.7
RPA1	15.2	22.5	4.3	9.6	7.1	7.2	8.3	21.8	3.8
SYNGR3	18.8	25.3	6.9	8.0	7.1	4.0	9.7	16.4	3.4
TMEM179B	18.3	20.3	5.2	8.6	7.9	3.5	9.6	20.7	5.6
DEGS1	13.6	23.6	4.0	10.3	9.0	8.4	6.8	20.2	3.7
WHSC1	17.9	20.0	5.0	10.8	10.9	7.2	5.1	19.8	2.9
NSA2	15.0	20.6	3.8	7.7	7.3	7.1	9.0	23.7	5.4
DRAM1	18.1	23.5	3.9	14.7	10.6	11.6	4.3	10.6	2.1
MED24	17.3	21.3	5.4	7.5	6.8	5.4	9.6	21.7	4.6
GOLPH3	14.8	18.2	4.6	8.1	7.1	5.2	10.5	26.0	5.0
C2orf68	20.3	19.7	5.7	8.3	7.7	5.6	8.8	18.0	5.2
YBX3	19.6	22.8	6.3	7.3	7.5	4.2	8.2	18.3	5.3
RRAS2	14.3	20.3	4.3	15.6	13.4	13.8	4.1	11.2	2.4
LOC100190986	18.1	28.3	4.6	4.8	3.5	8.3	5.9	22.8	3.0
MRPL37	13.5	22.9	4.9	11.2	10.0	7.7	7.4	17.1	4.6
PPP1R35	16.2	19.2	5.7	10.7	9.3	10.1	8.9	15.5	3.7
SEL1L3	21.4	28.2	6.1	11.0	8.7	9.9	3.3	8.7	1.8
DEK	15.4	17.5	3.5	12.3	9.9	10.0	7.6	18.1	4.9
GRB2	15.9	23.6	4.1	7.9	6.0	10.3	7.6	19.6	4.1
CUX1	15.7	19.8	5.3	6.7	8.1	5.9	8.4	23.6	5.5
PLEKHJ1	15.8	21.0	6.1	12.0	8.8	9.1	8.2	14.4	3.8
SLX1A-SULT1A3	16.3	20.2	5.0	7.1	6.2	3.6	12.2	23.0	5.4
SNX8	19.2	20.5	5.6	10.8	12.1	6.3	7.1	14.1	3.4

PAIP2	15.7	22.5	3.8	8.5	6.4	7.5	7.0	23.1	4.6
SRRT	16.9	16.2	5.3	9.4	8.7	6.5	10.2	20.1	5.7
TMEM245	16.7	24.6	5.3	9.7	7.7	6.6	6.6	18.6	3.3
CPSF6	18.4	17.4	5.2	7.7	9.9	7.1	9.9	18.8	4.5
PEX11B	16.5	23.2	4.7	8.4	5.4	4.5	8.5	24.1	3.5
ATP6V0D1	17.0	22.5	5.1	9.4	8.3	5.3	8.1	19.3	3.9
ARFRP1	16.9	21.4	5.2	7.8	7.6	6.2	9.0	20.6	4.1
IQCE	16.1	19.2	5.4	8.1	7.7	4.9	10.1	22.3	5.0
WAC	15.5	20.8	4.8	7.7	7.0	8.5	8.7	21.2	4.6
EIF4A3	15.3	21.9	4.4	8.6	6.4	8.7	7.5	21.8	4.1
MPZL1	15.2	23.6	4.4	10.4	8.3	9.2	5.9	18.0	3.7
SRPRB	11.7	18.7	3.0	6.7	4.5	5.1	12.5	30.5	6.0
RPRD1A	17.0	22.3	3.8	8.5	6.4	5.5	8.1	22.6	4.4
NCBP2	17.7	18.7	5.4	9.6	8.8	6.2	9.1	18.4	4.7
RAB8A	15.7	18.3	6.2	9.1	9.8	6.5	8.8	18.6	5.6
NDUFB3	13.9	24.7	4.6	10.5	7.6	9.8	6.2	18.3	3.1
STRA13	10.2	11.7	2.9	18.3	14.2	11.6	9.0	16.2	4.3
COQ4	19.0	19.5	6.3	8.4	7.9	3.7	9.6	17.6	6.6
RAP2B	15.4	17.8	5.1	7.5	9.1	7.4	9.6	21.2	5.4
PVRL2	18.1	23.1	7.4	9.0	9.0	3.8	7.9	16.1	4.0
CCT2	15.8	23.2	3.6	8.9	6.9	10.5	6.5	19.9	3.2
NDUFS1	16.1	23.5	3.6	8.4	5.9	7.5	6.9	22.7	3.8
ANAPC16	14.8	23.7	3.5	7.8	5.0	8.3	7.5	25.0	2.7
NARS	15.4	24.5	3.8	7.6	6.2	7.3	7.6	22.6	3.3
NUDT5	14.6	24.8	4.0	9.9	7.6	6.6	6.6	20.3	3.8
IL4R	15.0	22.4	5.9	6.0	4.9	11.6	7.9	20.5	4.0
RAB32	17.1	29.2	6.9	9.8	9.1	8.3	4.9	10.9	1.9
C16orf58	15.7	19.9	4.4	6.9	6.6	4.7	11.2	24.2	4.4
THRAP3	15.4	20.6	5.0	8.7	7.7	7.1	8.1	21.2	4.2
PMVK	15.8	18.6	4.3	10.4	8.3	5.8	10.8	20.4	3.6
NEU1	16.7	26.6	5.2	7.3	6.7	6.1	7.4	17.8	4.0
SRGAP2C	17.7	20.5	5.2	9.1	7.5	6.3	7.3	20.5	3.6
HN1L	16.0	19.6	6.0	10.5	10.9	9.9	5.6	14.4	4.7
MAPK1IP1L	18.7	18.9	5.3	9.1	8.9	4.9	8.1	18.0	5.8
GSDMD	16.7	20.7	5.2	9.1	8.2	7.0	8.8	17.9	3.9
TPRA1	18.5	23.1	5.7	8.7	7.0	5.3	7.9	17.8	3.7
PSMA4	13.3	23.4	3.3	9.9	7.0	8.6	6.1	22.3	3.8
SNORD32A	9.9	15.9	7.7	3.6	3.7	1.2	23.2	29.8	2.7
BRD3	16.7	15.6	5.3	7.4	7.8	5.0	11.1	22.1	6.5
NCK2	14.5	15.5	5.9	9.0	9.1	9.2	9.9	20.1	4.3
DDX42	15.3	18.9	5.3	7.7	6.6	6.2	9.4	23.0	4.9
NOLC1	16.2	17.9	4.5	6.4	6.2	4.7	10.8	25.6	5.2
SCARA3	19.2	24.3	6.4	11.3	10.6	6.1	5.3	11.4	2.9
STAP2	17.9	25.3	5.8	7.7	6.9	6.7	7.0	17.0	3.2
RHOT2	18.4	21.1	4.8	8.4	8.1	7.0	8.5	17.1	3.8
MAPK8IP3	19.2	23.0	4.9	5.8	5.0	4.4	11.2	18.8	5.1
TP53I13	15.8	19.4	5.2	8.2	7.3	8.0	10.0	19.0	4.5

CHD4	17.0	21.1	4.6	7.2	6.0	8.0	7.6	22.0	3.7
PLXNB3	17.0	20.2	5.5	9.2	8.6	4.3	9.1	18.8	4.4
DYNC1LI2	16.4	21.8	4.6	6.8	5.9	6.1	8.8	22.5	4.1
UQCRB	18.4	20.1	5.8	9.9	9.5	6.5	5.6	14.8	6.5
UCKL1	16.8	19.4	6.0	8.4	7.7	6.5	8.9	18.6	4.7
ADRBK1	15.4	18.6	5.3	6.9	6.4	11.0	10.3	19.2	3.8
FN3K	20.0	22.2	6.4	10.2	7.8	6.5	7.6	13.4	2.9
TIGD1	16.0	28.7	3.6	4.2	3.2	10.0	5.1	23.6	2.6
C6orf62	14.8	21.1	3.3	6.6	6.7	7.0	7.4	26.6	3.4
ZNF460	16.1	21.7	4.2	5.3	5.0	5.2	8.5	26.9	3.9
CLINT1	14.4	17.7	3.9	7.6	6.6	6.4	9.0	26.6	4.7
MCM7	13.7	17.9	4.2	12.6	11.8	10.9	7.2	15.4	3.1
UBN1	15.0	17.2	5.1	7.4	7.2	6.3	8.2	26.1	4.3
EMC8	18.2	21.0	5.0	8.0	7.5	3.7	9.4	19.4	4.6
NUP62	17.6	18.6	5.8	9.6	10.4	5.8	7.6	16.7	4.7
SREK1	16.6	19.5	4.1	7.1	6.0	6.8	9.3	22.2	5.2
SMIM7	15.4	22.1	4.8	6.8	6.1	8.3	8.1	20.0	5.1
ARL3	15.2	21.1	3.4	8.0	6.7	7.2	8.5	22.7	3.7
UBAP1	15.3	19.7	5.0	7.3	6.4	5.2	9.1	23.9	4.8
TIMM23	16.6	19.1	4.9	9.4	8.3	6.7	7.6	19.3	4.5
CAPZB	14.9	20.0	4.3	8.2	6.5	8.0	8.4	22.5	3.7
COMMD3	15.1	23.8	4.1	8.5	6.8	4.6	7.8	20.8	5.0
FAM3C	18.4	25.6	4.5	6.0	4.6	5.6	7.7	20.0	3.9
OGT	14.2	21.9	3.3	8.1	6.7	11.2	6.1	21.5	3.3
PSMD6	16.0	21.1	3.9	7.6	6.4	5.9	9.0	22.1	4.4
ALDH18A1	13.7	18.7	3.3	5.8	4.9	5.1	10.1	29.6	4.9
ITGA6	17.9	26.9	4.9	6.7	5.8	6.2	6.2	17.9	3.9
APEX1	14.0	19.1	4.1	8.9	7.7	8.6	9.0	20.5	4.3
FH	16.5	23.3	4.8	8.6	7.6	6.8	6.9	18.2	3.5
CRK	15.0	18.6	4.4	8.5	6.9	7.8	9.6	20.7	4.7
UXT	14.9	21.1	5.6	7.7	8.6	4.9	9.9	19.2	4.3
MRPS21	18.4	19.5	5.4	11.6	9.2	7.8	6.2	14.8	3.3
PPP3CA	11.1	14.7	2.5	6.1	4.6	5.2	10.7	35.1	6.1
NAGK	16.3	20.1	3.9	8.5	7.2	6.4	8.7	21.5	3.5
CDC123	16.1	23.6	3.8	8.5	6.8	7.0	7.5	19.3	3.5
SLC39A6	14.7	20.3	3.8	7.3	6.0	4.8	8.8	26.0	4.4
ANKRD40	15.9	19.4	4.8	9.6	8.5	6.2	7.7	18.8	5.1
VEGFC	13.4	20.3	3.8	14.2	12.8	9.4	5.2	14.1	2.7
ORAI1	14.7	17.3	5.5	8.0	8.3	4.2	9.1	23.5	5.4
LANCL1	16.4	26.0	3.9	8.2	6.0	5.5	6.4	20.5	2.8
LOC101927279	16.7	23.8	4.5	5.8	4.0	12.5	6.1	19.4	3.1
TMEM115	15.6	18.8	4.5	8.3	7.6	5.5	9.9	20.8	4.8
CBX4	15.4	14.9	5.1	7.1	8.1	6.8	10.9	21.4	6.2
COX17	20.1	14.6	8.1	9.5	8.1	5.7	7.4	15.7	6.7
SPIN1	17.1	21.2	4.5	9.7	7.5	7.4	6.2	18.7	3.6
SUZ12	15.7	21.3	4.3	9.9	8.4	7.1	7.1	18.4	3.7
CALCOCO1	17.0	21.2	4.7	6.6	5.5	4.5	9.3	22.3	4.5

ULK1	17.7	17.5	5.8	7.6	8.2	5.6	9.3	18.9	5.1
SPG7	17.2	20.5	5.0	7.4	7.0	5.6	9.2	19.8	4.1
THAP4	15.0	15.9	4.6	9.7	9.8	4.4	9.3	20.8	5.9
SNORA34	7.0	28.3	3.0	5.1	2.6	17.4	11.9	17.8	2.6
LOC101928291	17.6	16.8	6.1	8.3	8.0	4.5	9.5	19.6	5.2
SOGA1	17.2	21.9	6.5	7.6	7.2	4.8	8.1	18.6	3.6
PPP2R5B	15.7	20.4	4.6	6.0	5.8	5.7	9.6	22.6	5.0
ACP1	12.5	20.6	3.5	9.0	7.4	7.9	7.7	22.8	3.9
SNORA64	6.3	35.2	1.8	2.8	1.8	16.6	10.8	19.1	0.9
ITPRIP	11.6	14.1	3.5	10.1	9.3	7.4	10.1	24.3	4.9
C9orf89	14.4	20.7	4.1	6.9	6.6	5.5	9.5	22.4	5.2
PJA2	14.9	20.3	4.1	6.8	5.7	6.2	8.6	24.3	4.3
VASN	11.1	12.2	3.7	5.3	5.2	3.2	14.0	32.6	7.9
NOB1	16.8	19.7	5.0	9.0	9.3	5.0	7.6	18.3	4.5
MRPL18	14.9	20.3	4.0	8.5	7.9	7.3	8.6	19.4	4.3
COG8	15.3	19.5	4.7	7.7	7.9	5.3	8.7	21.0	5.0
DDX39B	12.8	22.1	3.0	5.9	10.3	6.3	8.1	23.0	3.4
TBCD	16.1	20.9	5.6	9.8	8.8	7.4	7.0	16.0	3.5
SIRT2	17.3	21.2	4.5	7.3	6.0	4.9	9.0	20.5	4.1
FGD5-AS1	14.2	21.7	3.6	8.2	6.2	7.5	7.4	22.3	3.8
RFXANK	16.0	21.1	5.6	8.3	7.9	5.1	8.5	18.4	3.9
NARF	14.3	19.2	4.1	7.9	6.2	4.8	10.8	22.9	4.6
DCXR	13.9	20.4	4.3	10.2	9.6	9.7	7.4	16.3	3.0
ILF3-AS1	15.1	22.5	4.0	5.7	3.8	7.0	9.0	23.3	4.1
HMGCR	18.0	27.0	4.5	5.0	4.3	4.3	7.3	21.0	3.2
MRPS23	15.8	17.9	3.6	10.2	6.6	6.8	9.5	21.0	3.2
LEPROT	14.1	23.4	3.3	9.9	5.9	9.2	6.7	20.2	1.7
TAOK2	15.8	19.0	4.6	7.3	7.1	6.1	9.5	21.0	4.2
BCLAF1	17.0	20.3	3.2	8.3	6.2	7.9	6.1	22.3	3.3
SLC35C1	17.1	18.6	5.1	6.6	6.7	3.1	10.4	22.2	4.9
ZKSCAN1	16.1	21.2	4.0	5.7	5.1	4.4	8.0	26.3	3.7
BRSK1	15.7	19.7	4.8	5.9	6.1	4.3	10.1	22.2	5.7
RBMS1	15.6	18.9	4.6	7.9	7.6	5.4	8.2	21.0	5.4
MRFAP1L1	16.0	15.8	5.5	8.9	7.6	5.0	12.4	18.2	5.0
POLR3D	15.3	15.8	4.7	5.8	5.2	3.4	12.0	27.0	5.0
MAGEA6	14.0	20.4	4.8	8.3	5.8	4.7	9.7	22.7	3.9
SCPEP1	13.4	20.4	3.3	9.3	6.4	7.3	8.4	22.6	3.1
LEMD2	14.9	21.3	5.3	7.6	8.0	7.2	8.6	17.8	3.6
PRRC2C	17.2	20.1	4.7	7.2	6.3	6.5	6.6	21.6	4.0
SLC27A1	18.1	23.3	5.2	7.0	5.8	4.1	8.3	18.1	4.1
B4GALNT1	17.9	20.6	5.5	10.9	9.8	4.6	6.4	14.6	3.9
COMMD6	10.6	23.7	4.3	7.3	6.0	5.7	7.6	24.0	4.9
REEP4	13.0	17.1	4.1	7.8	8.8	4.7	10.6	22.9	5.0
YME1L1	14.3	24.0	3.8	7.0	4.9	9.0	6.3	21.6	3.2
KDM4B	15.5	18.2	5.1	6.6	6.2	6.0	9.9	21.6	4.9
EPB41L1	19.5	21.9	6.1	8.2	8.9	6.1	6.5	13.6	3.2
SLC22A18	19.3	26.9	5.9	7.7	8.1	4.3	6.5	12.2	3.1

MAPK8IP1	16.0	19.1	5.8	7.1	7.4	5.1	9.2	18.8	5.3
HEBP2	17.5	25.5	5.1	8.7	7.6	5.2	6.3	14.6	3.5
RHBDF2	21.9	27.3	5.0	5.5	5.0	2.4	8.3	15.1	3.4
C12orf44	13.9	16.9	3.9	7.2	6.0	5.2	11.2	25.3	4.2
C21orf59	15.4	19.4	4.8	7.4	7.1	6.8	9.0	19.4	4.5
ZNF787	16.1	16.2	5.8	8.6	9.0	4.5	9.8	18.2	5.5
GLUL	15.1	19.3	4.4	9.2	9.3	8.1	6.6	17.6	4.0
SNX1	15.0	19.6	3.7	8.2	6.5	6.6	8.2	22.0	3.7
EHMT2	16.5	19.0	4.1	8.6	8.3	6.0	9.1	18.2	3.9
VAMP3	18.9	20.0	5.8	9.1	7.4	6.9	6.7	15.1	3.8
SF3B14	13.7	23.1	3.6	8.9	6.2	8.3	6.5	19.7	3.6
EXT2	16.1	22.2	3.7	9.6	6.4	7.8	6.2	18.9	2.7
RNF166	16.2	19.7	6.2	7.9	7.1	4.1	9.2	18.0	5.2
SWI5	14.4	17.6	5.3	8.2	8.6	3.6	9.8	20.7	5.3
C2CD2	14.0	17.0	4.3	8.3	7.6	5.5	9.4	23.2	4.2
PALM2-AKAP2	17.2	20.3	5.3	12.3	11.2	7.4	4.8	12.3	2.7
LOC100506688	16.1	24.8	4.5	4.9	3.9	8.7	6.4	21.1	3.0
FAM3A	15.2	19.9	5.0	7.7	6.9	4.8	10.1	19.8	4.2
CD320	13.4	15.7	4.0	9.8	9.5	6.4	9.6	19.7	5.2
AKAP8L	18.4	17.0	6.2	8.0	7.1	5.2	9.1	17.6	4.8
DFNA5	18.0	25.7	5.6	9.2	8.2	5.9	5.0	13.5	2.3
TP53INP2	16.3	22.2	8.1	6.5	6.7	4.3	9.4	14.9	4.8
C14orf178	13.5	27.7	3.9	4.7	3.1	9.6	6.2	22.1	2.5
GLO1	16.9	27.1	4.1	9.6	7.5	7.2	4.6	13.4	2.8
DYRK1B	15.0	17.0	5.4	7.7	7.8	4.3	9.4	20.7	5.8
SAP30L	16.4	18.3	5.2	7.1	6.7	4.7	8.2	22.2	4.3
CRCP	15.1	20.1	3.4	9.3	7.0	8.8	7.1	19.2	3.1
GID8	15.5	21.7	4.0	7.1	4.9	5.8	8.8	21.4	4.0
SNORA3	6.5	21.8	4.7	5.5	4.5	14.3	12.7	19.8	3.2
C8orf33	15.6	17.0	5.9	9.0	8.3	7.0	8.8	16.7	4.7
DDX24	16.0	20.5	4.5	8.2	6.8	7.2	6.6	19.5	3.8
FLYWCH1	16.1	19.9	4.8	7.8	7.2	5.8	9.0	18.7	3.7
PRKAR2A-AS1	15.8	24.3	4.7	5.9	3.9	10.3	5.9	19.2	2.9
MDM2	15.5	22.9	3.4	6.7	4.8	5.6	6.9	23.0	4.1
ABCD1	14.7	18.3	4.1	6.7	6.2	3.8	10.9	23.3	5.0
MAP2K7	16.3	19.2	4.7	5.4	5.9	6.4	9.4	20.3	5.3
UBE2N	13.0	23.5	3.9	8.9	7.3	9.3	6.0	18.3	2.7
PSMD1	14.9	21.6	3.9	9.2	7.1	6.9	6.7	19.0	3.4
TNS3	16.6	18.9	4.9	11.8	11.0	6.6	5.4	14.5	2.9
DCTPP1	13.5	20.0	4.5	11.3	9.1	7.4	8.0	15.8	3.0
CHCHD1	16.4	14.0	4.6	10.4	8.7	5.0	12.2	16.7	4.6
PIAS3	14.3	18.7	4.2	5.7	5.3	4.0	9.6	25.0	5.6
POLRMT	15.4	18.6	4.3	6.7	6.2	4.4	10.8	20.7	5.3
GPT2	13.2	16.8	4.0	4.9	5.0	3.0	11.6	29.3	4.8
PNRC1	10.9	14.2	3.9	6.5	6.0	14.9	9.1	20.8	6.2
RUVBL2	17.1	18.1	5.2	8.5	9.0	5.4	8.2	16.9	4.0
ATG13	14.6	21.7	3.9	6.7	5.8	4.4	7.0	23.8	4.4

TMEM106C	13.3	21.5	3.4	13.1	10.3	8.6	5.5	15.3	1.5
CISD3	14.4	17.4	5.7	10.1	12.7	7.7	6.6	13.6	4.3
KLF2	8.5	9.4	3.3	8.2	9.5	39.7	3.7	7.8	2.4
SF3A1	15.7	16.5	4.9	6.6	5.5	5.7	9.4	23.2	5.0
ZSWIM8	16.3	18.9	4.1	6.6	5.7	4.9	9.8	21.4	4.8
COL4A2	15.4	17.5	5.3	5.6	5.1	4.7	10.2	23.9	4.6
RNF114	12.8	22.2	3.9	6.5	5.9	9.4	8.0	20.3	3.0
DNAJA3	15.3	18.9	4.7	7.6	7.1	5.4	8.8	19.8	4.6
SNRPN	12.7	16.1	3.7	9.4	8.7	7.6	8.5	20.9	4.5
TOMM22	15.0	18.5	7.2	9.0	7.0	6.9	9.6	14.6	4.2
MAP2K3	15.7	20.8	5.1	9.2	8.6	9.2	7.2	13.8	2.4
RAB4B-EGLN2	14.2	17.9	4.4	7.9	7.4	7.9	8.8	19.9	3.4
DENND1A	15.8	17.1	4.9	8.3	8.4	5.2	8.6	18.3	5.2
PPAPDC1B	9.4	16.5	3.2	5.4	4.2	7.5	10.3	30.1	5.3
KLHDC3	14.9	19.2	3.8	5.8	5.7	5.8	10.0	22.1	4.6
SOCS2-AS1	18.4	25.2	5.2	5.6	6.3	5.4	5.9	17.0	2.7
DEDD2	13.3	18.6	4.4	5.9	5.1	7.4	10.2	22.3	4.6
GLTP	15.8	17.4	5.0	10.1	9.4	5.7	7.0	16.1	5.0
TRNS1	10.3	19.3	6.3	5.4	3.4	0.6	13.6	29.9	2.8
NHP2L1	17.3	22.8	5.7	6.8	5.9	4.0	7.0	18.4	3.6
CHERP	17.4	15.7	6.3	6.7	7.2	4.7	10.3	17.9	5.3
SLC50A1	13.8	17.3	3.6	12.2	10.1	7.1	6.8	16.9	3.7
KLF16	14.5	15.5	6.3	8.6	9.5	4.1	9.2	16.6	7.1
UAP1	17.9	29.1	4.5	8.3	6.3	8.2	3.4	11.8	1.9
POLR2G	19.7	24.9	4.3	6.9	5.2	4.6	6.6	15.9	3.3
SLC38A1	14.7	23.4	3.4	7.0	5.9	6.6	5.4	21.4	3.6
GTF3A	14.5	20.7	5.0	9.9	8.4	6.7	6.6	15.6	3.9
ECHS1	13.9	19.8	3.3	9.4	7.1	6.9	8.2	19.4	3.4
PITRM1	15.1	19.5	4.1	8.0	7.4	6.1	7.6	19.2	4.3
C1orf85	16.3	19.5	5.0	12.1	11.2	6.4	5.7	11.8	3.1
RC3H2	15.9	22.0	4.0	6.9	5.6	5.4	6.8	21.2	3.6
ITGB1BP1	14.4	21.2	4.0	10.9	8.1	5.2	6.2	18.4	2.8
TRABD	17.1	19.1	5.4	6.8	6.2	6.1	9.2	16.1	5.2
PSMA6	12.6	22.2	3.8	8.0	6.5	11.3	5.6	17.6	3.3
HDDC2	15.3	19.5	4.3	10.7	8.5	6.9	6.3	16.0	3.5
SQLE	15.2	22.9	4.1	5.5	4.5	5.0	7.2	22.7	3.9
FLJ46363	17.2	20.6	6.1	6.5	6.1	4.3	7.3	19.4	3.5
SIN3B	18.3	22.1	5.3	6.9	6.8	4.6	7.6	15.3	3.9
TAOK1	15.3	21.1	4.2	5.7	5.1	5.7	6.9	22.6	4.1
OTUB1	14.8	18.0	4.4	6.1	6.5	6.1	9.7	20.5	4.8
NDUFA10	15.9	18.9	4.7	7.6	7.3	5.2	7.6	19.0	4.4
COX11	15.1	16.5	5.3	8.1	6.8	6.4	9.7	18.6	4.2
COPS3	13.4	22.7	3.7	7.8	5.9	5.7	6.7	21.1	3.7
ACO2	15.2	18.9	4.9	7.5	7.4	5.9	8.0	19.0	4.0
WASH1	17.5	20.9	5.2	6.9	5.9	3.5	8.7	17.1	4.9
PPM1B	17.1	23.0	4.3	7.1	4.8	7.2	6.2	18.2	2.8
PGRMC2	14.9	15.8	7.0	9.0	8.2	3.6	10.4	17.4	4.4

MAPK9	14.7	20.7	4.1	9.4	9.1	7.1	5.5	17.1	2.8
SLC35A2	12.9	16.9	3.6	6.6	5.8	4.7	10.2	24.5	5.5
OCIAD1	13.2	20.8	2.5	7.3	4.8	8.1	5.8	23.8	4.3
SIRT6	15.4	17.0	5.8	7.3	7.1	6.1	9.3	17.2	5.2
RHEB	15.4	24.2	3.6	9.7	7.3	9.6	4.6	12.4	3.6
KARS	14.9	19.2	4.1	7.6	6.5	6.8	7.1	20.2	4.0
PSMB8	13.6	19.5	4.1	9.5	6.2	12.6	6.8	16.1	1.9
FBXW11	13.9	20.6	3.8	8.3	6.5	5.2	7.5	21.0	3.5
MTX1	15.5	20.9	4.7	8.5	6.7	5.5	7.7	17.3	3.6
BCL2L13	15.1	20.2	3.5	7.1	6.5	6.2	7.3	20.4	4.0
PGAM5	13.7	16.0	4.6	7.6	7.4	5.6	9.8	20.4	5.0
CHTOP	17.3	20.9	5.6	6.5	5.5	4.2	8.7	17.8	3.7
FAF2	15.0	18.9	3.9	5.8	5.8	3.8	8.6	23.9	4.4
CNIH3	7.0	10.7	2.0	1.7	1.4	1.2	12.6	46.1	7.4
44815	15.2	20.8	4.3	8.9	7.7	7.5	5.9	16.6	3.4
TBC1D20	13.6	18.6	4.0	6.4	5.4	4.1	8.8	24.5	4.8
CKS1B	11.2	18.8	3.5	13.3	12.1	11.1	5.3	12.2	2.6
GTF3C5	15.6	16.9	4.6	8.0	7.3	5.7	8.8	19.0	4.2
NTSR1	11.2	15.1	4.6	2.2	2.8	1.0	13.2	32.0	7.9
IRF3	13.8	18.0	4.8	8.0	6.9	8.1	8.5	18.1	3.7
SYAP1	14.6	20.2	4.6	7.1	5.8	7.9	7.3	18.4	4.0
ATG4B	15.4	18.5	4.1	6.2	6.5	4.8	8.5	20.8	5.0
LPHN1	15.5	16.9	5.4	6.9	6.5	3.9	9.5	20.2	5.1
CMTM3	14.4	17.0	4.0	7.5	6.3	5.7	9.1	21.6	4.2
REXO4	14.2	16.4	4.7	8.8	7.4	6.0	9.1	19.2	4.1
MED15	15.5	16.3	5.3	9.2	10.3	5.1	7.6	15.7	4.7
GDE1	12.5	18.3	3.2	8.0	5.4	4.9	8.6	24.4	4.4
APBB2	13.0	17.8	4.6	5.6	6.0	3.5	8.8	25.6	4.9
HIST1H2AI	13.1	13.7	6.6	13.3	14.5	8.4	6.1	11.1	3.0
MAST2	16.0	19.9	5.1	8.1	7.8	4.6	7.7	16.5	3.7
GLTSCR2	14.9	16.6	5.0	7.0	6.8	9.4	9.3	15.9	4.5
SFXN1	14.0	20.8	3.6	8.2	6.2	5.4	7.0	21.2	2.9
STOML2	13.6	21.4	3.5	8.5	8.4	6.8	6.9	17.2	3.1
SETD1A	16.8	16.3	4.9	6.4	6.0	4.1	9.8	19.8	5.3
KCTD5	15.8	17.8	6.0	6.6	6.9	5.0	8.5	18.1	4.8
SUMO1	14.6	19.6	3.5	9.3	5.5	8.3	6.2	19.0	3.5
SLC10A3	12.7	14.5	4.3	7.3	6.6	5.4	9.8	24.1	4.6
LRRC41	15.1	20.0	4.3	8.1	6.6	5.0	7.7	18.6	3.9
LSM10	13.8	20.4	3.8	7.0	5.8	6.9	7.0	21.4	3.3
PCNXL3	14.7	17.9	4.6	6.9	6.7	4.5	8.9	20.4	4.6
VKORC1L1	13.4	20.7	3.9	8.1	7.6	6.0	7.9	18.0	3.6
TGIF1	16.8	22.2	3.7	6.0	6.4	6.2	7.1	17.4	3.3
LOC101928372	16.2	24.1	4.5	4.4	3.9	6.3	6.4	20.3	2.8
PRRX1	8.3	9.9	2.6	4.1	3.5	3.4	15.2	33.0	8.9
ARPC5L	14.9	18.0	4.8	8.1	6.5	7.0	8.4	18.2	3.0
CHST3	14.0	17.0	4.6	9.0	10.2	6.4	7.3	16.6	3.9
ATF5	18.2	20.7	5.6	6.9	6.4	3.3	6.8	15.7	5.2

HADHB	14.2	21.8	3.9	7.9	6.0	6.8	6.5	19.1	2.9
ANKLE2	15.3	19.6	3.8	9.8	7.9	8.0	5.8	15.6	2.9
NQO1	10.9	16.1	2.3	10.3	7.6	7.9	7.6	21.9	4.2
FOSL1	21.6	20.5	8.1	8.6	8.9	4.2	5.6	8.7	2.6
YAP1	14.6	18.2	4.3	8.2	7.9	5.5	6.9	19.4	3.8
GDPD5	20.2	23.3	6.2	11.0	10.1	5.3	3.1	7.7	1.6
INPP5E	19.0	22.2	5.8	8.4	8.1	4.3	6.0	11.7	3.4
ZNF579	15.9	18.4	5.8	8.2	8.5	5.0	8.2	14.9	3.9
CPSF7	15.7	17.0	4.0	6.9	6.1	5.8	8.5	19.0	5.6
SSR1	11.8	18.6	3.0	6.9	4.6	6.2	8.0	25.5	4.0
TTC7B	17.8	23.2	5.5	7.8	7.1	5.3	5.6	13.7	2.8
PSME2	13.1	18.9	2.9	8.6	6.8	7.6	7.3	20.4	3.2
TFAP2A	13.7	15.4	4.4	8.5	8.8	4.1	8.0	20.0	5.7
ACIN1	17.3	16.9	5.3	7.3	6.2	5.8	8.3	17.3	4.2
GLRX	14.7	21.4	4.9	6.9	4.2	6.9	7.2	18.8	3.5
PPFIBP1	16.2	26.6	4.5	4.7	3.8	4.2	6.0	19.3	3.2
CD8B	15.0	24.5	4.2	4.5	3.6	8.0	5.5	20.4	2.7
TMEM173	19.7	24.2	5.7	9.3	7.9	6.9	4.1	9.0	1.7
LPCAT1	15.0	19.9	4.0	7.8	7.4	6.1	7.6	16.6	4.0
SEC23B	15.5	21.7	4.1	8.6	7.0	6.2	5.5	16.4	3.2
SDHB	13.9	19.5	4.6	8.2	7.3	5.1	8.0	17.9	3.9
UBA2	14.3	20.8	3.9	8.5	6.7	6.8	6.5	17.4	3.4
C9orf37	16.0	21.2	4.0	5.6	5.0	4.3	9.2	19.5	3.4
MAFG	13.7	17.6	5.2	7.2	6.4	6.8	10.7	17.1	3.4
ZDHHC5	16.1	17.4	4.6	6.3	6.3	3.7	8.3	20.9	4.5
IPO5	13.4	21.9	3.4	9.7	7.5	7.4	5.4	16.5	2.8
FAM65A	17.1	20.2	5.4	8.2	7.5	5.8	6.4	14.1	3.3
ZIC1	15.3	17.6	5.2	4.2	5.3	2.1	10.0	22.3	6.0
KLHL5	13.4	20.3	3.5	7.7	5.7	5.6	7.1	20.8	3.9
DNAJB11	13.6	15.0	3.8	7.5	6.6	8.7	8.1	20.3	4.3
DDTL	17.4	12.8	2.5	5.9	9.3	6.5	11.9	19.3	2.4
NMT2	22.4	29.0	6.6	7.9	6.7	5.0	2.7	6.2	1.2
ANP32A	14.8	13.3	4.3	7.7	7.4	6.9	9.9	20.0	3.6
CDK10	15.6	18.6	4.2	7.1	6.2	4.8	9.2	18.1	4.1
HIBADH	15.1	25.0	4.5	8.8	6.8	7.2	5.1	12.7	2.7
ZNF444	13.3	17.5	4.8	7.2	6.9	4.9	9.6	18.4	5.1
NFKBIB	13.4	19.0	3.6	5.7	6.4	8.7	7.2	20.0	3.7
USP14	13.6	20.5	3.1	8.0	5.7	6.7	5.3	21.5	3.3
ACACA	16.2	25.0	4.2	6.6	4.8	5.2	5.3	17.8	2.6
SNORA24	4.4	23.9	0.8	4.5	2.2	19.8	12.5	17.5	2.2
ORC6	14.8	21.9	4.0	6.8	5.1	9.6	5.3	17.5	2.7
CDK5	14.0	18.2	3.6	8.1	8.3	5.5	7.5	17.8	4.7
RELL1	19.6	19.8	6.7	5.6	4.2	3.0	8.3	16.3	4.1
DPF2	13.7	18.0	4.1	6.4	6.1	5.7	8.6	20.7	4.4
GPRC5B	15.8	17.6	5.3	17.9	16.3	9.5	1.4	3.1	0.8
YY1	13.5	16.1	5.6	8.0	8.1	6.3	9.7	15.7	4.8
VDAC3	12.0	22.2	2.4	8.3	6.4	9.2	5.2	18.9	3.0

RPE	14.0	19.1	3.9	9.1	5.6	6.7	7.1	18.4	3.8
ZDHHC16	12.7	19.1	3.0	10.5	8.9	6.0	7.1	17.4	2.8
MRPL11	14.1	19.2	4.8	9.6	7.8	5.5	6.8	16.7	2.9
GMPPA	13.2	18.5	4.2	6.0	6.3	3.9	9.0	21.6	4.7
STK39	13.4	16.8	3.7	8.5	6.9	8.1	6.5	20.0	3.6
MRPS18B	13.5	19.2	4.2	7.9	7.7	5.5	6.4	18.5	4.4
SOD3	18.6	22.0	5.7	5.3	6.8	2.5	8.3	13.5	4.5
RBBP4	14.1	20.3	3.9	7.0	6.0	7.8	6.0	19.3	2.8
SAC3D1	14.3	17.4	5.3	9.8	9.9	5.9	7.9	13.0	3.6
PAFAH1B1	13.8	22.8	3.6	6.7	5.3	5.9	6.4	19.6	2.8
LOC101928229	19.1	20.2	5.8	6.6	6.8	3.9	6.3	15.7	2.4
TRPT1	14.2	21.3	4.2	7.1	6.8	3.8	8.5	16.6	4.3
PPP1R11	12.9	12.3	4.7	7.1	9.0	4.7	8.5	22.8	4.9
ODC1	13.9	20.9	3.4	6.5	5.5	7.7	6.9	18.6	3.4
MPDU1	15.1	18.0	4.0	7.3	7.1	4.3	7.6	19.7	3.7
PBX2	15.7	17.2	4.6	6.0	5.7	5.7	8.6	19.0	4.2
FBXL18	14.0	18.5	5.4	7.1	7.6	4.3	7.4	18.4	3.8
MAPRE3	16.7	19.4	4.7	6.9	7.8	4.0	6.0	17.2	4.0
ACOT8	12.6	18.2	4.0	8.5	6.2	4.5	8.5	19.6	4.5
RAB12	16.6	17.4	3.8	6.8	6.3	5.9	7.6	17.6	4.6
DENND5A	14.3	20.5	4.2	8.3	7.0	6.2	6.1	17.1	3.0
MVK	15.1	18.2	4.5	7.9	6.2	3.8	8.0	19.3	3.6
LOC101927011	15.4	27.3	3.5	3.3	2.9	8.4	4.1	19.6	2.2
SCARNA17	11.0	17.2	3.6	5.7	5.4	24.2	7.9	8.8	2.8
GOLGA7	15.4	19.0	4.5	8.1	6.4	8.3	6.5	14.9	3.2
NDUFA6	13.1	15.6	4.6	8.4	6.7	7.4	8.5	17.8	4.4
SHC2	19.4	22.8	5.9	6.6	8.0	3.1	5.8	11.8	3.0
MSL1	13.4	17.7	4.1	7.4	6.9	7.4	7.4	18.1	3.9
LOC90834	15.8	20.3	4.3	5.1	3.8	12.5	5.6	16.5	2.5
TPRN	13.1	15.1	5.0	9.7	9.1	5.4	8.0	16.6	4.4
EMC3	15.1	21.0	4.2	8.5	5.9	4.6	6.5	16.8	3.6
SSB	14.7	19.1	3.5	7.9	5.6	7.5	6.2	17.8	3.9
ABCF1	13.9	15.3	4.3	6.9	6.5	5.5	9.4	20.2	4.2
PPM1F	13.9	19.0	4.9	6.5	6.9	4.7	8.1	17.9	4.3
C7orf26	13.2	16.9	3.9	7.2	6.1	5.5	8.5	20.3	4.6
TSTA3	12.0	16.0	3.6	8.1	7.1	5.7	9.7	20.0	4.1
SFXN3	15.5	19.9	3.8	5.6	4.8	4.0	7.4	21.9	3.4
HSPH1	11.8	16.3	2.9	9.7	7.8	9.7	5.7	18.7	3.6
ZNF512B	14.9	15.4	4.7	7.9	7.3	3.7	9.0	18.6	4.7
LOC101929361	16.4	24.5	5.9	4.3	4.4	4.7	6.1	17.2	2.6
TMEM175	16.0	17.5	4.2	5.5	5.4	2.8	9.7	18.9	6.0
SRP72	14.7	17.0	3.7	7.3	5.7	5.9	7.5	19.7	4.4
TSKU	13.0	13.7	4.9	5.8	5.5	2.7	10.8	23.3	6.4
BCL9L	17.0	16.4	6.6	6.6	7.0	5.5	7.3	15.4	4.2
SRGAP2B	13.8	19.5	4.5	6.8	6.4	4.2	7.4	19.6	3.8
INE1	14.9	24.0	4.2	4.2	3.6	7.7	5.4	18.9	2.9
B3GNT1	13.8	19.4	4.5	7.7	7.9	4.9	7.6	16.6	3.4

UFD1L	13.6	17.7	3.7	8.2	6.5	6.5	6.9	17.9	4.8
LDOC1	13.8	17.4	5.7	8.7	8.4	4.8	7.6	14.8	4.5
PKP2	15.3	19.4	4.2	12.6	11.2	8.5	3.9	8.8	1.9
GCN1L1	13.8	17.4	3.9	5.8	5.1	5.0	8.3	23.0	3.5
EID2	14.2	15.5	5.5	9.2	9.4	7.7	7.4	12.7	4.2
DDX49	13.2	15.5	4.2	7.8	6.3	4.4	9.6	19.7	4.9
ABCF3	12.7	17.2	3.6	6.8	6.8	5.9	8.6	20.0	4.0
ENDOG	14.9	20.5	4.3	7.4	8.0	4.9	6.7	16.0	2.8
BET1L	10.1	17.6	2.7	4.9	3.6	4.2	11.4	27.1	4.0
TMPPE	19.9	24.8	5.4	7.8	3.8	4.9	7.2	9.1	2.7
LINC00294	14.7	20.3	4.0	5.8	4.3	9.2	5.9	18.3	3.2
PTPRA	15.9	21.7	4.0	5.7	5.0	4.8	6.7	18.4	3.4
RETSAT	13.5	18.9	3.7	7.4	5.9	5.3	7.8	20.1	3.1
APOBEC3C	12.7	14.1	3.3	8.0	6.6	5.1	9.0	22.4	4.5
RPS19BP1	13.5	14.4	5.7	10.4	7.7	4.9	10.5	14.9	3.7
LOC100268168	14.2	21.4	4.1	5.6	4.4	9.6	5.3	17.7	3.3
AAK1	16.1	19.4	5.3	6.3	6.3	5.1	6.3	16.9	3.9
MICAL1	17.0	21.7	4.6	6.1	5.0	4.7	7.2	15.3	3.8
CHAC1	14.4	16.7	4.3	5.4	5.1	2.6	10.3	22.4	4.4
SPRED2	13.0	17.3	4.1	4.9	5.0	3.4	9.7	22.2	5.9
ZDHHC4	14.6	20.5	3.8	7.5	6.6	5.2	6.7	17.0	3.4
DDX54	15.3	16.2	4.9	7.1	6.8	6.4	8.5	15.9	4.2
NSDHL	14.8	17.7	4.1	8.1	7.3	6.0	7.0	16.7	3.6
FAM214B	15.3	19.0	5.1	7.5	7.9	4.2	7.1	16.4	2.9
DNAJA2	14.3	18.4	3.7	8.4	6.9	6.1	7.1	17.2	3.1
FEZ2	14.1	21.7	4.3	9.5	7.6	8.1	4.3	12.9	2.8
R3HDM2	16.5	16.8	4.9	5.8	5.5	4.3	8.0	18.9	4.5
KCTD20	12.7	19.7	3.2	7.6	6.5	5.4	6.5	20.6	2.9
SYMPK	15.3	18.3	5.0	5.0	5.6	4.1	8.6	19.3	4.0
VIMP	13.5	15.1	3.2	5.2	3.8	3.7	11.4	23.9	5.4
VAC14	16.5	23.3	5.7	7.5	7.1	4.0	4.8	13.5	2.7
CYHR1	15.6	18.1	4.2	6.2	5.9	4.2	8.8	18.4	3.7
C7orf73	16.6	18.0	4.7	8.6	6.8	5.0	6.2	15.0	4.3
LOC101929264	13.9	16.5	5.7	5.7	5.5	5.7	9.6	17.0	5.3
STX1A	13.8	16.9	4.2	3.5	3.2	2.1	10.6	25.3	5.4
CBX6	14.3	13.4	4.6	6.9	6.7	5.3	8.7	18.8	6.2
MFSD6	14.6	21.0	3.9	5.7	4.3	4.9	6.2	20.8	3.6
REXO2	13.9	17.3	4.6	7.3	7.3	7.0	8.7	15.1	3.7
NOTCH1	15.2	16.8	5.8	5.7	5.7	4.1	8.1	19.4	4.3
UPK3B	19.2	19.2	5.6	11.6	11.3	6.4	3.3	7.3	0.9
ASAP2	16.1	20.3	5.0	7.5	6.7	5.2	6.0	14.6	3.2
ANO6	15.4	22.8	3.9	9.8	7.8	8.1	3.8	10.7	2.4
SLC12A4	15.9	20.5	5.0	7.4	6.0	6.0	6.7	14.0	3.1
FLJ30403	16.2	18.6	3.9	4.5	3.3	16.0	4.9	14.3	2.8
GALNT1	10.1	15.0	2.9	5.6	3.6	5.0	9.0	28.0	5.4
PES1	13.1	15.4	4.4	5.4	5.4	3.8	10.2	22.4	4.6
HS6ST1	12.2	15.5	4.2	7.2	7.3	4.4	9.1	18.6	5.8

PDHA1	13.5	16.4	3.4	9.1	9.1	6.7	5.9	17.3	3.1
DTYMK	12.2	17.7	3.1	10.4	8.2	9.9	5.8	14.2	2.9
SEMA3B	18.9	23.8	5.6	8.5	7.8	4.9	4.6	8.7	1.7
ZDHHC7	15.7	20.1	4.5	8.9	7.8	6.4	5.4	13.3	2.5
CCDC92	14.2	14.8	4.9	7.3	7.8	4.6	8.0	17.9	4.9
SMARCA5-AS1	14.9	14.0	5.9	6.5	7.0	6.7	9.2	15.5	4.7
TCF7	9.8	9.4	2.5	1.7	1.6	5.9	17.0	26.5	9.9
GLB1	10.3	24.6	3.0	5.7	8.3	4.0	3.6	21.5	3.3
APOBEC3F	12.7	12.9	4.6	6.0	6.2	4.5	8.8	22.4	6.2
EGLN1	13.6	17.2	5.1	7.5	7.4	6.3	7.5	15.8	4.0
SLBP	15.1	17.9	4.8	8.0	8.4	7.8	5.5	13.4	3.4
CHMP7	12.3	15.6	3.3	6.3	5.6	7.5	8.9	20.1	4.5
CAPN5	13.5	16.5	4.8	9.1	8.2	4.2	7.2	16.1	4.5
NUMB	14.3	16.9	4.0	6.9	7.0	4.1	6.9	20.1	4.0
SUCLG1	13.7	18.6	4.2	7.7	6.5	5.6	7.0	17.5	3.3
SUGP2	14.0	19.3	3.9	6.9	6.1	6.9	6.7	17.1	3.3
NTNG2	13.2	14.1	4.4	3.8	4.1	1.8	11.2	25.6	5.9
SNORA71C	8.5	13.3	3.2	7.2	6.4	10.7	15.2	15.3	4.2
PATL1	16.1	20.9	4.5	6.1	5.1	4.2	5.8	17.5	3.7
SPATC1L	16.1	17.2	5.5	8.1	9.6	4.0	7.4	12.4	3.7
TUSC3	14.7	23.3	3.3	7.8	7.1	5.7	4.5	14.9	2.6
LOC101928750	14.7	17.1	4.5	7.4	7.7	3.9	9.0	15.2	4.4
TERF2	12.8	16.1	3.9	5.2	4.7	4.5	8.7	23.3	4.7
PTPN1	14.5	19.2	4.3	7.3	6.4	7.7	5.9	15.1	3.5
PRAME	12.3	18.4	3.3	9.7	8.8	6.2	5.7	16.2	3.2
ZMYND19	12.6	16.0	4.2	7.2	6.4	6.1	8.7	18.8	3.9
RSL1D1	14.3	15.1	4.3	8.0	7.1	5.3	6.6	18.8	4.3
PTDSS2	15.6	17.8	4.6	7.1	6.8	3.4	8.4	16.5	3.7
TAF15	11.7	9.9	3.3	10.5	8.1	6.1	8.3	22.4	3.4
MECP2	13.1	12.4	4.9	7.0	8.6	5.1	8.5	19.3	4.8
TWIST1	13.1	14.3	6.8	6.5	6.1	2.4	11.7	16.9	6.0
44622	12.7	18.4	3.8	9.1	8.4	6.8	6.7	15.3	2.5
XPO6	13.1	18.9	4.0	8.6	6.7	9.2	4.9	15.2	3.0
CRTC2	12.7	14.6	4.6	7.2	7.1	4.6	8.7	19.1	5.1
WDR82	14.1	18.6	3.8	6.6	4.9	5.1	7.0	19.9	3.7
RALA	13.6	21.3	4.5	8.7	6.7	6.2	5.9	13.6	3.2
ERBB2	16.0	19.9	4.9	8.5	7.8	5.0	6.1	12.3	3.0
LIMD2	13.6	14.2	3.8	6.5	6.6	13.7	6.1	14.9	4.0
TRAPPC2L	16.1	15.5	4.4	7.6	7.4	3.8	8.7	15.6	4.4
B4GALT1	13.1	17.5	3.6	6.3	6.9	7.8	6.7	17.7	3.9
CSF1	15.1	21.8	4.4	11.8	10.1	6.9	3.2	8.7	1.4
LMAN1	11.9	16.4	3.7	5.9	5.7	5.1	8.1	21.9	4.6
LOC101927740	14.3	21.4	3.7	5.1	4.0	9.0	5.9	17.2	2.8
MRGBP	13.6	15.8	4.3	8.0	6.4	5.8	8.4	17.7	3.3
ZBTB38	12.3	16.3	3.2	8.1	6.9	6.6	6.4	19.3	4.1
RGL2	14.1	18.5	3.8	6.1	5.8	5.7	7.7	18.0	3.5
CPD	13.5	21.0	3.8	5.8	5.1	6.5	5.6	18.5	3.4

C1orf122	11.9	18.7	4.8	6.7	6.5	7.9	8.5	14.1	4.0
SYNCRIP	14.9	16.3	4.0	6.3	6.4	6.6	7.1	17.7	3.9
RPS6KB2	12.8	14.7	4.1	7.1	7.1	5.8	8.3	18.2	5.0
ADD2	18.4	20.2	6.2	8.7	7.6	4.1	4.8	10.4	2.7
MALSU1	12.5	18.4	4.2	8.0	8.1	6.0	7.9	14.4	3.6
IQSEC1	14.2	16.1	5.2	6.5	6.3	7.8	7.0	16.2	3.8
MLXIP	15.4	15.5	4.8	5.4	5.5	5.6	7.6	18.4	5.0
TTC7A	12.4	16.2	4.0	6.1	5.3	6.5	9.1	19.9	3.6
AKIRIN1	13.5	17.8	4.2	8.1	7.4	8.0	6.5	13.8	3.7
BASP1	13.0	11.0	5.2	12.6	13.1	9.5	6.2	8.9	3.4
CCDC85B	14.5	20.7	6.2	8.3	10.2	7.8	4.6	8.8	1.9
SCARB2	14.0	23.1	3.9	7.3	5.2	5.9	5.1	15.7	2.8
UBE2B	13.1	14.8	3.8	8.3	5.7	8.5	7.2	16.5	4.9
RING1	12.7	15.3	4.1	7.3	7.3	4.9	8.7	18.0	4.4
LOC550643	14.5	18.4	2.9	5.9	5.3	5.7	7.5	18.2	4.0
ABHD4	14.2	17.9	4.2	7.9	6.9	5.4	6.5	15.8	3.7
HDAC3	13.2	18.2	3.7	7.0	7.7	5.4	5.9	17.8	3.5
P4HA2	13.9	21.5	4.4	5.3	4.8	3.7	6.6	19.1	3.0
IDH2	14.8	19.6	5.3	6.9	6.8	7.2	5.8	13.3	2.7
ZDHHC24	14.2	16.7	4.9	5.4	5.8	4.1	8.0	19.1	4.2
SNX22	12.6	18.0	3.8	6.0	5.8	5.0	6.8	20.8	3.5
FEM1A	13.7	14.8	4.4	6.0	6.4	5.0	9.1	18.1	4.7
POMP	10.6	24.5	2.6	7.7	4.8	10.1	4.2	15.4	2.5
LSM14B	14.1	17.5	4.9	7.4	6.4	4.0	7.9	16.0	4.1
FAM219B	13.9	18.3	4.4	6.8	5.7	4.7	7.8	17.0	3.7
DEXI	13.3	17.0	3.9	7.3	6.6	5.6	6.3	18.2	4.0
TCF12	13.7	17.5	3.0	5.1	5.0	3.9	7.9	22.7	3.4
CREG1	14.2	14.8	4.5	7.8	7.3	5.2	8.0	15.5	4.9
RALB	15.4	16.6	4.2	7.2	6.3	6.1	7.4	15.7	3.2
WDR81	14.6	16.4	4.4	5.6	5.6	3.8	8.8	18.9	4.0
RNF41	14.3	20.0	3.8	5.3	4.4	8.0	6.1	17.2	2.9
ELK4	12.5	15.7	3.0	6.0	5.0	8.8	6.2	21.0	3.8
SERTAD3	11.6	14.5	3.8	6.1	4.7	5.1	8.4	22.5	5.2
ERLEC1	11.2	18.6	2.8	5.2	4.1	5.3	7.3	23.0	4.5
PRRC1	12.8	17.4	3.3	4.9	3.7	4.0	7.9	23.7	4.3
LOC100287166	10.3	15.8	3.4	14.7	13.1	9.8	3.7	9.5	1.7
MATR3	13.6	17.6	3.0	7.0	5.3	7.5	6.1	18.0	4.0
FAM115A	14.4	20.2	3.9	5.7	5.1	4.0	6.9	18.4	3.4
STARD7	12.5	18.5	2.8	6.7	5.7	6.3	7.1	19.0	3.3
SEC23A	12.3	19.0	3.2	6.0	5.1	5.6	6.5	20.6	3.6
FBXO44	12.5	15.0	3.8	9.4	8.9	6.6	8.0	13.9	3.7
SARNP	16.5	13.7	3.8	7.2	5.4	5.3	7.3	18.3	4.4
ANAPC13	12.7	18.2	3.3	7.8	7.0	3.4	5.9	20.0	3.5
SAT2	14.3	22.3	3.9	6.3	4.0	6.3	6.4	15.8	2.6
NPIPA2	17.2	21.0	2.6	3.7	3.6	2.6	7.5	20.5	3.1
TBC1D13	12.9	16.4	3.9	8.5	7.5	6.2	6.9	16.5	2.8
CLUH	13.0	14.1	4.4	6.4	6.8	4.8	8.9	18.7	4.5

MRPL2	13.0	16.0	4.7	6.6	6.9	3.9	8.8	17.4	4.3
STK10	16.8	18.2	5.0	6.1	5.8	7.3	5.9	13.5	3.0
B9D1	14.9	17.9	4.0	9.4	9.3	5.7	5.6	12.4	2.4
NTMT1	16.2	17.4	4.7	7.4	6.0	4.7	6.9	15.3	2.9
NPB	17.6	17.4	5.9	8.9	8.9	4.4	6.3	9.1	2.9
SSH1	12.3	14.4	4.3	8.5	7.7	4.9	7.1	19.0	3.2
RHOB	9.9	13.5	3.5	9.6	8.2	16.6	5.3	12.6	2.3
CHD6	10.7	16.7	3.3	5.2	4.1	4.4	8.7	23.4	4.8
SLC35B1	12.5	18.9	2.9	5.7	5.5	5.8	7.0	19.7	3.3
CDS2	12.3	20.8	3.1	7.1	5.9	5.4	6.2	17.2	3.2
USP10	12.1	16.9	3.4	6.4	5.3	5.3	7.7	19.9	4.3
VPS9D1	15.2	16.7	4.1	7.1	7.4	4.3	7.8	14.3	4.3
HIATL1	13.2	19.8	3.6	7.7	5.3	7.4	5.4	16.1	2.7
SNHG8	11.1	18.5	2.7	6.1	4.8	6.2	8.5	19.6	3.5
MRPL32	12.6	14.2	4.3	7.8	7.9	7.1	7.3	16.6	3.4
MRPL10	14.4	17.8	3.7	6.8	6.3	6.1	6.3	14.9	4.8
LOH12CR2	14.0	19.2	3.0	4.7	3.1	11.5	5.5	17.7	2.4
FOXF1	13.6	15.5	4.9	5.3	6.1	3.3	8.1	18.7	5.6
PEF1	14.5	18.7	3.4	7.5	7.0	5.2	6.6	15.2	2.9
C7orf55	10.8	21.5	3.4	5.3	3.8	5.9	7.6	19.9	2.8
DPY30	14.3	17.8	2.7	7.8	5.7	8.9	5.0	16.0	2.8
TARDBP	13.1	17.7	3.0	9.4	5.9	7.3	5.5	16.0	3.0
DAPK1	11.9	16.9	3.6	13.2	13.3	9.5	3.1	8.1	1.4
OAF	17.2	22.9	5.3	4.1	3.8	2.4	6.9	15.2	3.2
SLC25A4	10.6	16.9	3.6	9.5	8.9	7.8	5.9	14.5	3.2
LETM1	13.7	16.5	4.6	6.9	6.5	5.5	7.6	16.1	3.5
PARP3	15.2	18.0	4.1	7.1	6.0	4.5	6.6	15.5	3.8
MAP3K10	13.7	15.7	4.8	5.8	6.2	4.7	8.5	17.1	4.3
UFC1	11.0	16.9	3.1	7.9	5.9	6.1	8.5	17.1	4.2
MANBAL	17.7	14.8	5.9	7.5	9.0	4.7	6.5	10.1	4.6
NFYC	13.4	15.9	4.4	6.5	7.0	4.7	7.3	18.0	3.7
TNIP2	11.9	14.9	4.3	7.0	6.3	7.4	8.6	16.6	3.5
LINC00649	14.8	22.3	4.1	4.0	3.7	4.6	5.6	18.6	3.0
MAP2K1	11.2	18.3	3.5	7.4	6.2	6.0	6.6	18.1	3.3
GTPBP6	15.2	18.3	5.2	6.6	6.0	3.9	8.0	14.4	3.1
GTPBP6	15.2	18.3	5.2	6.6	6.0	3.9	8.0	14.4	3.1
GADD45B	16.1	15.6	4.2	11.6	11.6	12.3	2.9	5.5	0.9
MOSPD3	13.0	16.3	4.7	7.2	7.3	5.2	6.7	15.5	4.8
TRAPPC12	14.1	18.4	4.5	6.0	5.8	5.4	7.4	15.1	3.9
KIF3B	12.4	15.6	3.7	5.9	5.1	5.1	8.7	20.1	4.0
STX5	11.4	14.0	3.4	5.5	5.0	3.9	9.6	23.4	4.3
DCTN6	13.4	16.5	3.4	6.5	5.4	5.0	6.9	18.9	4.4
ANO8	14.1	17.9	4.7	4.9	4.8	4.3	8.0	17.8	3.9
CHST14	14.1	18.3	4.5	7.8	7.4	5.3	5.7	14.3	3.2
CCDC47	11.0	15.9	3.1	7.2	5.3	6.0	6.9	20.9	4.0
RBX1	9.4	25.7	2.8	5.6	3.3	6.3	6.5	17.8	2.8
WDR54	12.4	17.9	4.0	8.0	6.9	4.1	6.6	17.0	3.3

H2AFX	11.2	11.8	4.1	13.4	14.1	7.8	4.9	9.9	3.1
LIMS2	16.4	24.5	4.7	9.2	9.3	5.8	3.2	5.9	1.3
CHMP3	12.3	15.1	4.7	8.5	7.0	6.4	7.1	14.2	4.9
VEZF1	12.9	12.6	3.7	6.1	5.8	4.7	7.7	21.7	4.9
CNIH4	11.5	18.6	2.6	6.7	6.5	6.9	5.8	19.1	2.4
MYEF2	16.3	18.5	3.5	5.8	4.2	3.5	7.8	16.9	3.6
ZBTB47	14.2	15.2	5.3	6.2	6.4	2.6	8.2	17.4	4.7
BCAM	17.3	18.2	6.0	8.4	7.8	4.9	5.5	9.4	2.6
AP1S2	11.7	18.8	2.5	6.3	4.6	6.1	7.2	19.5	3.3
PPP6C	11.6	20.6	2.8	6.4	5.4	6.6	5.0	18.2	3.4
FBXO7	14.1	18.6	3.4	6.9	6.5	5.1	5.3	16.8	3.3
CCNY	12.9	17.3	3.4	6.9	6.3	7.0	5.4	17.3	3.5
DOK4	15.4	18.1	4.3	8.6	7.7	4.5	5.5	13.2	2.6
IPO7	12.6	19.1	3.1	7.8	6.3	5.8	4.8	17.2	3.2
FAM167B	15.0	15.3	4.6	12.1	10.9	7.1	4.4	8.8	1.5
ATP5SL	12.4	17.0	3.7	6.1	5.3	4.4	8.3	18.7	3.8
ATP6V1B2	14.7	21.2	3.5	5.9	5.0	5.5	6.0	15.1	2.8
PTPN23	14.7	15.5	4.5	5.7	5.5	4.9	7.8	17.1	4.1
RNASEH2C	8.8	15.8	3.5	9.3	7.5	8.4	8.3	14.8	3.3
RNPEP	12.4	17.0	4.2	8.0	7.8	4.7	6.9	15.3	3.5
ZC3H18	13.8	13.8	4.9	6.1	6.0	4.5	8.8	17.2	4.6
MED29	12.5	15.2	4.3	6.7	7.1	3.8	7.9	17.8	4.3
DST	13.0	19.6	3.6	6.6	5.2	6.5	4.4	18.2	2.6
OSTM1	12.6	19.5	3.4	6.9	5.8	6.0	6.3	15.7	3.5
PDZD11	13.2	20.4	3.0	7.6	7.6	6.4	5.6	12.8	2.9
C9orf78	14.0	12.1	4.5	6.5	5.6	4.5	8.7	18.9	4.9
JAGN1	9.9	16.1	3.2	7.5	6.4	3.1	7.9	22.1	3.4
QRICH1	13.6	13.7	4.0	6.1	6.5	4.8	8.1	18.0	4.6
OAZ2	11.7	18.5	3.2	7.7	6.3	4.9	6.1	18.0	3.1
LYRM4	13.2	19.4	3.6	5.5	4.0	7.8	5.1	17.4	3.5
SLC35C2	15.4	18.3	4.2	5.2	4.3	5.1	6.5	17.2	3.3
LOC284889	11.1	18.0	4.6	8.4	9.6	3.6	7.7	12.5	4.1
SNX12	14.5	19.0	4.2	6.1	6.0	6.5	5.4	14.8	3.1
HIPK3	12.3	21.0	3.1	7.7	5.3	6.2	4.9	16.3	2.8
CMIP	11.8	16.5	4.0	8.3	7.4	8.3	5.6	14.4	3.1
LOC101926982	13.4	19.9	4.2	4.4	3.2	7.6	6.2	17.8	2.7
GOLIM4	12.6	11.7	4.2	6.1	5.7	4.0	10.5	19.4	5.2
PDLIM2	11.4	15.8	4.9	9.4	10.8	7.4	5.7	10.8	3.3
EIF3CL	15.8	17.3	4.9	6.7	6.3	4.2	6.6	14.1	3.6
FLNC	14.4	16.7	4.8	9.3	9.7	6.0	4.8	11.1	2.4
EXOSC1	13.8	15.4	4.9	6.0	6.7	4.8	6.4	17.1	4.1
CLIP1	14.7	18.8	4.1	6.4	5.9	5.2	5.7	15.6	2.7
STRN4	13.9	15.0	4.8	6.1	6.6	5.3	7.8	16.1	3.6
TTC1	13.0	17.1	3.8	9.2	6.9	6.8	4.9	14.4	3.0
OSBPL2	13.0	14.7	4.0	5.9	4.5	5.1	8.8	18.9	4.2
CKLF	10.7	19.6	2.3	7.3	6.8	2.5	5.0	22.4	2.5
SHANK2-AS3	12.1	21.9	3.7	5.5	3.9	6.2	5.6	17.7	2.6

NDOR1	14.3	16.6	3.9	7.7	6.3	4.5	7.2	14.8	3.8
PDP1	19.4	27.3	4.7	4.0	2.9	2.8	4.0	11.8	2.2
FBXO18	12.3	16.1	3.4	6.6	5.0	4.8	7.3	19.8	3.7
GART	10.8	19.8	2.9	6.4	6.7	5.5	5.1	19.4	2.5
TRAK2	11.5	17.1	3.0	7.0	5.5	4.4	6.5	19.9	4.0
ZNF865	12.2	14.2	4.7	7.3	7.5	5.1	7.8	16.6	3.7
SORT1	12.8	19.0	3.6	7.5	6.2	6.5	5.6	14.8	2.9
RFX5	13.1	14.7	3.3	8.2	6.6	6.5	6.5	16.4	3.5
SNTA1	14.6	19.8	5.0	9.1	7.7	6.5	4.4	9.8	2.0
CES2	13.4	17.5	4.0	6.2	5.7	4.7	7.1	16.5	3.8
FSD1	13.0	17.0	3.7	8.6	8.5	5.4	6.2	13.5	2.8
NDUFA5	11.1	14.9	3.1	8.8	5.6	7.3	6.3	17.8	3.9
SEPHS1	11.6	14.3	2.6	7.5	6.5	5.4	7.5	19.7	3.7
METRN	16.0	20.5	5.6	9.4	8.7	4.2	4.7	7.2	2.3
VPS53	14.1	18.3	4.0	6.8	6.1	5.1	5.4	15.6	3.1
MAT2B	12.0	17.2	2.4	6.1	5.6	6.8	7.3	18.0	3.4
LUC7L2	12.3	14.5	3.5	6.5	5.5	5.4	7.8	18.3	4.9
LOC100652901	13.3	23.4	3.9	2.3	2.4	8.0	4.1	19.1	2.1
CPSF1	13.2	14.9	3.9	6.1	5.7	7.1	7.9	16.2	3.7
GPNMB	20.8	27.5	4.6	7.7	7.0	5.7	1.4	3.0	0.9
EIF1AX	11.4	18.0	2.9	8.0	6.6	9.1	4.6	15.9	2.2
LOC643406	12.6	24.2	3.3	3.1	2.7	8.1	3.6	18.7	2.2
TSN	10.9	17.5	2.3	6.6	4.3	7.3	6.0	20.0	3.6
LARP4B	13.5	18.6	4.1	6.2	5.7	4.8	6.2	16.0	3.3
PGM1	12.6	17.4	3.7	7.7	6.7	5.7	5.7	15.4	3.5
WASF2	14.9	18.2	4.8	5.6	4.8	5.0	5.8	15.5	3.7
LOC101927929	13.8	18.5	3.9	4.3	3.7	8.8	6.0	16.7	2.5
DYNLT3	12.3	17.0	3.5	4.8	3.9	3.1	8.1	22.3	3.4
ANKRD13A	13.6	20.1	3.9	10.2	8.7	9.4	2.7	8.0	1.7
MINPP1	14.3	16.9	4.3	7.1	6.3	3.2	7.1	14.1	4.9
EVI5L	13.3	15.9	4.5	7.3	6.9	4.0	7.7	15.7	3.0
RPS6KA4	12.9	16.1	4.3	8.7	8.6	7.1	6.5	11.6	2.4
KIAA0226	12.8	17.4	3.5	6.8	6.4	5.4	6.2	16.9	2.9
LINC-ROR	14.8	14.7	4.0	12.3	11.0	5.4	3.8	10.1	2.1
CCNDBP1	15.2	19.7	3.0	5.1	3.9	4.0	5.7	19.7	2.0
TSEN15	12.9	19.0	3.7	7.0	5.2	5.4	5.4	16.3	3.4
HAGHL	16.2	19.2	4.2	7.3	6.3	4.4	6.0	10.8	4.0
ABTB1	13.5	16.3	4.0	5.9	4.8	6.6	8.6	15.4	3.1
POFUT2	13.5	16.8	3.2	4.1	3.4	3.0	9.1	20.8	4.3
LONP2	12.6	18.0	3.9	6.2	5.1	4.7	6.3	18.4	2.9
HSPA13	11.1	17.2	2.4	5.9	4.2	6.0	6.5	21.3	3.4
MSANTD3-TMEFF1	15.0	16.3	4.5	7.7	7.9	8.4	4.2	11.4	2.7
LRP11	12.4	17.8	4.2	7.7	7.8	4.5	6.2	14.5	2.9
CFLAR	12.8	22.1	3.1	6.3	4.6	10.1	4.1	13.1	1.8
LOC101929698	13.6	20.2	4.2	4.8	3.3	8.1	4.9	16.4	2.5
ELK1	11.9	13.2	4.5	6.5	7.4	4.5	8.3	17.4	4.4
KLF13	14.7	13.9	5.7	4.3	5.4	9.8	6.7	13.7	3.8

MOB2	14.1	15.8	3.9	4.6	4.3	4.2	8.7	18.1	4.2
PACS2	14.3	16.4	4.5	6.9	7.0	4.0	6.6	14.7	3.5
RPL17-C18orf32	11.3	17.0	3.4	6.8	6.2	12.7	4.5	13.2	2.8
EMC7	10.7	17.9	3.6	7.4	5.2	5.1	7.1	17.5	3.3
HIST1H3C	9.9	14.3	3.6	15.8	16.1	11.1	1.6	4.3	1.1
PSMD14	11.7	20.2	2.8	7.0	4.7	7.4	5.2	16.8	2.1
TOMM34	13.6	15.2	4.1	9.3	8.3	5.3	5.6	13.9	2.3
IRGQ	11.5	15.3	4.9	5.0	4.7	4.4	9.1	19.6	3.2
ICT1	14.2	15.3	4.1	7.4	6.1	3.0	8.6	14.9	4.2
LOC101928303	14.1	20.4	3.0	4.1	3.3	8.7	4.9	16.9	2.3
CCDC130	15.1	16.2	3.7	5.1	5.3	3.5	8.3	15.5	4.9
ARAP1	13.8	16.8	4.7	7.7	6.9	8.3	5.6	11.6	2.3
LOC101929224	12.7	20.4	3.8	4.0	3.8	7.9	5.6	16.8	2.5
LRFN4	14.5	16.9	5.0	8.6	8.7	5.1	5.1	10.5	3.1
FAM136A	11.8	17.3	4.5	5.6	5.1	3.7	7.7	18.5	3.3
CD55	17.6	26.9	4.8	6.6	5.2	5.6	2.2	7.0	1.6
SEPHS2	11.5	15.6	3.0	6.9	5.3	7.3	7.1	18.2	2.5
THRA	11.4	15.5	3.9	5.0	4.9	3.6	8.3	20.0	4.7
NT5C	15.1	17.8	4.4	6.8	5.6	6.0	6.9	11.3	3.4
44812	12.3	14.7	4.0	8.0	6.5	5.6	7.7	15.1	3.4
CAPZA2	10.5	19.6	2.6	8.5	5.1	8.8	4.4	15.8	2.0
SOX4	7.6	7.5	3.0	1.9	2.3	2.8	15.3	27.8	9.2
EIF4E	12.5	16.4	3.8	7.4	5.1	6.9	5.5	16.6	3.0
NDUFS4	13.6	13.8	4.7	7.2	6.1	4.5	7.3	16.1	3.9
RUSC2	15.4	20.4	5.3	5.7	5.4	2.8	5.6	13.3	3.2
YIPF5	9.9	16.5	2.5	6.7	5.1	5.4	6.0	21.5	3.4
TMEM101	12.0	20.1	3.9	6.7	5.3	4.3	6.8	15.0	2.9
IMPAD1	10.3	19.5	3.3	4.9	4.1	5.1	7.2	19.9	2.7
POLR2A	12.7	14.9	3.7	6.3	5.8	6.5	6.2	17.5	3.5
HIST1H2BE	11.8	11.4	5.1	8.0	8.8	15.3	5.1	8.4	3.1
DDX1	11.6	16.1	2.7	6.4	4.7	5.8	6.7	19.8	3.2
SYPL1	13.2	20.1	3.2	7.2	4.6	6.3	5.5	14.7	2.0
RRP12	12.5	15.7	4.3	5.0	4.4	3.3	8.4	19.1	4.2
LINC00263	15.6	19.1	5.1	5.4	5.2	3.8	5.1	14.2	3.3
MAML1	12.6	14.5	3.9	4.4	5.1	3.4	7.5	20.3	5.0
SAFB2	11.7	11.1	4.3	5.3	5.5	4.9	11.2	17.3	5.4
ANKRD28	12.9	19.4	2.9	6.1	4.8	5.1	5.1	17.6	2.8
NBPF24	13.1	19.5	3.4	5.7	4.5	3.9	6.2	17.3	3.1
TBL2	12.1	15.3	3.7	4.9	4.1	3.0	8.9	21.0	3.7
MMP24-AS1	15.7	18.4	5.4	7.4	7.6	4.6	5.7	9.0	3.0
CHST12	11.3	13.5	3.5	6.6	7.3	4.2	8.4	17.1	4.6
AKR7A2	13.7	18.3	4.2	6.2	5.8	3.6	6.7	15.1	2.9
COMMD9	12.3	17.3	3.3	6.6	8.1	4.8	5.5	15.6	3.1
LOC101929567	13.3	24.1	3.2	3.0	2.5	7.0	3.5	17.6	2.3
LOC101929455	14.3	20.7	3.3	6.5	5.1	2.8	5.2	15.6	3.1
MRPL47	12.0	17.6	2.7	7.1	4.9	5.7	5.8	16.7	3.9
QSOX2	12.1	15.6	3.8	6.0	5.0	5.6	6.7	17.8	4.0

FAM210B	11.6	13.5	4.9	8.4	6.0	3.7	7.7	16.0	4.6
PLEKHG2	14.1	16.6	4.1	6.3	5.5	3.9	6.5	15.9	3.4
PIM3	10.3	14.1	3.3	5.7	5.8	15.4	6.4	12.4	3.0
FBRSL1	13.2	13.8	4.5	6.7	6.8	8.1	6.5	13.1	3.6
ADAT1	12.5	18.6	3.3	5.8	4.6	8.2	4.9	15.9	2.4
XYLT2	14.2	17.1	5.2	6.7	6.9	4.0	5.9	13.2	3.2
TMED1	11.6	17.5	3.5	6.6	5.6	3.9	7.5	17.3	2.7
CPNE2	14.4	15.5	4.6	8.1	7.5	4.3	5.6	13.1	3.1
MFF	11.5	17.4	2.5	7.4	5.1	5.7	5.7	17.7	3.4
UPF1	12.1	14.8	4.4	6.3	6.8	6.3	6.4	16.0	3.1
RAB24	12.8	14.1	3.4	5.9	4.8	5.6	9.3	16.3	4.0
COA1	11.6	19.2	3.4	5.8	4.3	7.6	5.0	16.8	2.4
ACAP3	11.3	14.2	3.7	5.7	4.8	9.1	8.3	15.4	3.3
KPNA4	12.0	18.3	2.7	7.9	6.2	7.6	4.5	14.3	2.5
ADAM17	12.7	18.0	3.3	5.9	4.2	5.1	5.6	17.6	3.6
FLJ13197	12.5	18.2	3.8	3.9	3.3	5.6	7.2	16.6	4.8
ETHE1	11.6	13.8	4.4	10.4	10.3	6.2	5.0	11.3	3.0
LOC101929078	11.4	14.8	5.7	7.5	6.9	4.1	8.3	14.2	3.1
TTC9C	13.3	17.6	3.4	5.4	4.8	7.5	5.5	15.1	3.3
SNAPC2	12.5	16.0	4.6	5.5	5.5	3.8	7.1	16.7	4.2
WWC3	12.0	13.5	3.4	7.1	6.1	5.4	7.3	17.8	3.2
USP7	12.3	15.8	3.4	5.5	4.6	6.4	6.4	17.9	3.6
IL17RC	13.2	18.1	4.2	5.8	5.4	3.2	7.5	14.6	3.8
HSPB8	16.4	19.3	5.5	11.7	9.2	5.9	1.7	4.6	1.4
ADAM10	13.3	18.8	3.1	6.2	4.7	6.2	5.4	15.7	2.5
FBXL19	12.1	13.4	3.6	6.3	5.6	4.4	8.8	17.5	4.0
ZNRF3-AS1	12.9	20.5	3.4	4.1	3.0	8.8	4.7	15.9	2.5
BDNF	12.7	16.3	3.9	10.2	9.7	6.9	3.7	9.6	2.7
ATXN7L3	11.9	14.8	3.0	6.0	4.9	5.6	7.5	18.6	3.5
CLDND1	13.4	21.1	2.9	5.6	3.7	5.6	4.9	16.7	1.8
PCIF1	14.6	18.2	4.5	5.7	5.2	4.8	6.1	13.3	3.3
GLIS3	20.6	21.6	6.5	5.3	5.1	2.5	3.6	8.3	2.2
PHF15	10.9	11.8	3.5	6.0	5.7	5.6	8.7	19.2	4.3
NSMCE1	12.1	19.4	4.3	6.3	4.9	4.8	7.0	14.6	2.3
RAP2A	12.2	16.8	4.9	7.1	6.0	5.4	5.9	14.6	2.7
RABGAP1	11.5	17.8	3.0	5.5	4.5	4.7	5.7	19.4	3.3
TEX261	10.5	13.0	3.1	9.1	7.4	5.4	5.4	18.4	3.3
HGSNAT	11.5	18.1	3.5	6.9	5.5	4.4	5.8	17.6	2.3
PLEKHM1	15.0	18.0	4.9	5.8	5.7	3.7	6.1	13.3	3.0
SGSH	13.2	15.6	4.8	8.3	8.2	4.2	5.8	12.2	3.2
RXRB	12.0	15.0	3.3	5.3	5.0	4.2	8.5	18.8	3.5
CARD11	13.9	14.3	4.5	7.5	7.6	7.1	5.7	12.1	2.7
NOTCH3	13.1	17.7	4.8	8.3	8.1	4.5	4.7	11.9	2.3
CXorf40A	10.7	17.7	3.2	5.0	4.2	5.3	7.4	18.9	3.0
UBL7	10.8	15.1	3.6	7.9	7.2	4.9	6.3	16.4	3.3
LOC101928137	13.8	26.7	2.9	2.3	2.5	6.6	3.2	15.1	2.2
ACBD6	13.4	16.9	3.1	6.0	4.5	7.0	5.8	15.9	2.7

GALNT10	12.8	17.7	4.7	5.6	5.2	4.6	6.4	15.2	3.1
RALGDS	11.5	14.6	3.5	5.3	4.5	4.0	8.5	19.3	4.2
FBXW2	11.4	18.1	3.0	7.0	5.5	5.8	5.6	16.2	2.8
RCC2	11.9	15.4	3.8	5.1	4.5	6.0	7.2	17.6	3.8
IDI1	14.1	19.2	3.8	6.4	4.6	5.6	4.3	14.4	2.9
GPRC5A	15.1	19.5	4.1	12.0	10.1	6.5	2.3	5.0	0.6
ZNF524	12.3	16.8	3.8	6.8	5.8	3.3	8.5	15.2	2.7
TESK1	11.5	13.9	3.7	6.9	7.3	5.6	6.8	16.1	3.5
TNFSF4	16.0	26.4	4.8	2.6	2.6	2.3	5.1	13.3	2.1
VPS35	11.4	18.4	2.9	7.1	5.4	6.6	5.0	15.6	2.7
LOC645553	15.6	17.6	4.3	11.1	9.2	5.2	3.0	7.2	2.0
RPAIN	13.7	15.5	4.3	5.9	5.5	3.4	7.8	16.5	2.6
CUL4A	13.1	16.6	3.7	7.3	6.1	5.3	5.4	14.6	2.9
MRPL27	11.1	15.6	3.6	7.1	5.9	6.0	7.1	15.3	3.3
LOC101929426	13.1	18.0	3.0	5.6	3.2	10.4	4.3	14.4	2.9
AXIN1	12.4	14.6	3.8	6.9	6.3	5.7	7.1	14.8	3.5
MCRS1	13.5	16.1	3.5	5.6	5.1	4.7	7.5	15.5	3.6
CAMK2G	13.5	16.4	4.2	6.1	5.7	4.5	6.9	14.4	3.3
GAK	12.9	16.0	4.2	5.1	5.2	5.1	6.9	15.9	3.7
IST1	12.6	18.7	3.1	5.0	4.0	5.4	5.3	18.1	2.8
ATP9A	11.9	15.8	3.8	7.2	5.9	4.2	6.6	16.6	3.1
PARP6	12.1	15.3	2.9	6.6	4.8	4.1	7.4	17.8	3.8
RRP36	12.4	16.3	4.1	7.1	6.8	5.8	6.4	13.1	3.0
ZBTB45	13.8	14.5	4.6	6.7	7.0	3.3	7.1	14.0	3.8
PPP1R26	12.9	14.6	4.7	7.4	6.9	4.8	6.8	13.4	3.3
CIAPIN1	13.5	19.2	3.0	6.3	5.1	5.0	5.8	14.6	2.3
YTHDF1	10.8	13.7	3.5	5.9	5.7	4.7	8.1	17.3	4.9
GIGYF1	13.9	15.8	3.8	5.4	4.5	4.8	8.0	15.3	3.3
CEP170	12.4	17.4	3.0	5.1	4.3	5.0	5.3	18.8	3.3
KIAA2013	11.6	15.5	4.4	5.5	5.7	3.8	7.8	16.9	3.5
ZNF496	12.3	18.2	3.0	7.7	6.2	5.1	4.9	14.6	2.7
SLC8A1	14.4	23.0	3.4	3.5	2.6	3.3	5.4	16.5	2.5
COQ9	10.8	15.6	3.2	6.3	5.7	5.1	6.8	17.5	3.7
COG1	12.0	15.5	3.3	5.5	4.8	3.8	7.4	18.6	3.7
QTRT1	13.6	15.6	3.8	5.3	6.2	4.0	7.7	15.1	3.4
SUPT7L	13.6	19.9	3.9	6.3	5.8	4.3	5.7	13.0	2.3
STX6	11.4	14.1	3.1	7.2	6.1	5.4	6.2	18.1	3.0
ETS1	10.5	12.9	3.1	7.2	6.6	15.4	4.7	11.5	2.8
TXN2	11.6	14.8	5.4	6.9	7.3	4.7	7.1	13.3	3.7
HOXB9	11.7	12.1	3.9	3.8	3.8	2.0	9.9	21.4	5.8
RAD21	10.7	17.5	3.0	6.7	5.2	7.1	5.5	16.1	2.7
LOC100289019	12.5	19.0	3.3	3.5	2.7	5.1	6.0	18.9	3.4
DHX15	11.4	17.3	2.8	6.2	5.0	5.9	5.8	16.8	3.2
RUSC1	11.4	15.8	3.2	7.1	5.6	4.7	7.1	16.7	2.9
44814	10.8	16.8	2.6	8.9	6.5	6.6	5.0	14.7	2.6
LSM1	11.6	18.3	3.6	7.5	6.2	5.8	5.7	12.4	3.4
SP1	11.9	14.8	3.8	6.8	5.6	5.0	6.1	17.0	3.5

NCRUPAR	12.3	18.0	3.1	5.1	3.4	10.4	5.2	14.5	2.3
PSMG4	10.0	14.8	4.3	8.6	7.8	3.5	7.2	14.7	3.4
SAP30BP	13.4	20.1	3.3	5.6	5.1	5.1	4.6	14.6	2.5
FAM45A	13.0	18.6	3.2	8.1	5.7	5.7	4.5	12.5	2.9
TRAPPC10	11.7	15.6	3.5	5.8	5.1	5.2	6.2	17.2	3.9
DALRD3	12.2	17.1	3.8	6.9	6.5	4.6	6.2	13.9	3.0
GLE1	11.8	15.9	3.4	6.1	5.7	4.1	6.1	17.9	3.2
NPIPA5	11.8	16.6	2.9	5.6	4.1	3.8	6.6	18.6	4.0
NDRG1	12.1	13.2	3.6	9.7	11.5	5.8	4.6	11.5	2.2
STRAP	11.4	18.2	2.6	5.8	4.0	5.9	5.5	17.6	3.0
SLC25A46	12.0	19.9	3.1	5.6	3.8	4.4	6.5	16.0	2.8
AKR1A1	10.7	14.9	2.4	7.7	7.5	5.5	5.9	17.1	2.2
HIF1AN	10.7	15.7	2.9	7.5	6.3	8.4	5.8	13.6	3.0
PEMT	12.4	17.5	4.7	6.3	6.3	3.3	6.3	13.8	3.4
TRIP12	11.5	16.2	2.9	6.1	5.0	5.8	5.4	18.0	3.1
FAM160B2	13.7	14.8	4.2	6.1	5.9	3.4	7.3	14.7	3.7
NDUFA12	11.5	16.1	2.6	6.1	6.7	6.1	5.8	15.0	3.8
SLC7A1	13.1	18.2	3.8	6.2	5.4	4.0	5.5	15.1	2.4
COL7A1	12.6	17.1	3.5	6.2	4.3	3.9	7.8	15.3	3.0
MFN2	11.9	15.8	3.5	5.8	5.5	4.0	6.7	17.7	3.0
LPCAT4	13.7	16.8	4.4	7.2	7.1	4.7	4.7	12.3	2.9
LIN7C	11.8	17.3	2.6	6.2	5.5	5.4	5.3	16.1	3.5
PDF	9.6	14.0	2.9	6.5	4.7	5.6	9.0	18.3	3.2
MYBBP1A	11.7	13.8	3.5	4.6	4.3	3.5	8.6	19.3	4.3
LRWD1	11.9	14.7	4.0	6.5	5.8	4.1	7.8	15.8	3.2
RGP1	11.7	15.5	3.3	4.8	5.4	3.7	7.3	18.4	3.6
IMMT	12.0	16.4	3.2	6.5	5.9	4.9	5.7	15.6	3.5
SCCPDH	10.2	15.5	2.5	9.6	8.3	7.9	4.6	12.6	2.3
CREBBP	12.0	12.4	4.6	5.4	5.9	5.6	6.7	17.0	4.0
HMGXB3	12.4	16.6	3.5	5.6	5.2	4.4	6.2	16.2	3.3
PFDN6	14.7	14.6	3.2	6.5	4.7	6.0	6.4	14.5	2.9
FAT1	11.4	16.8	3.3	7.1	6.2	5.6	4.3	16.4	2.3
ZBED6	12.6	18.5	2.8	4.4	3.1	4.3	5.6	18.6	3.6
LOC101929678	13.0	15.6	3.7	6.5	5.4	3.5	5.8	16.9	3.2
PCNA	9.3	14.3	2.7	9.8	7.6	8.8	4.0	14.7	2.2
LOC101928925	10.9	15.2	3.7	5.8	5.2	5.8	6.5	16.9	3.3
RBM14	13.1	14.0	3.7	6.8	5.3	6.3	5.4	15.4	3.4
RPL7L1	11.4	15.4	3.1	7.0	4.2	7.4	6.1	16.1	2.8
PGP	12.0	15.1	3.3	8.5	7.6	4.7	6.7	12.4	3.2
ERI3	10.1	15.0	2.8	5.9	5.7	4.4	7.3	19.1	3.0
BRMS1	10.9	11.9	3.9	6.1	6.3	4.7	9.3	16.1	4.1
DAXX	11.7	13.4	4.1	7.7	7.8	6.1	7.1	12.2	3.3
GPBP1L1	11.2	15.0	2.9	5.6	4.6	5.2	6.4	19.9	2.6
RIT1	13.2	18.7	3.2	6.8	5.6	6.2	5.1	12.4	2.1
PLEKHA2	12.0	15.2	3.4	6.7	5.6	5.6	6.1	15.1	3.6
UBP1	11.3	14.9	2.8	5.7	5.3	5.8	6.4	17.5	3.6
ATXN10	12.7	18.8	3.3	7.8	6.7	6.1	4.5	11.3	1.9

POMZP3	11.0	16.2	3.0	9.4	6.8	6.4	4.8	13.3	2.3
PDS5A	13.1	19.4	2.5	5.4	4.0	5.3	4.1	16.6	2.7
C12orf49	13.5	18.5	3.3	6.1	5.9	5.5	5.6	12.8	2.0
MRPS11	11.2	14.7	2.9	7.5	6.7	4.4	6.9	15.3	3.5
POGK	10.9	16.8	3.1	5.4	5.2	5.3	6.0	17.4	2.9
POM121	13.6	15.8	4.2	6.7	6.3	3.3	5.5	13.9	3.7
NACAD	17.6	18.0	6.6	6.9	7.0	3.0	3.7	8.0	2.1
NEDD4	10.6	14.4	2.6	9.5	6.8	7.0	4.5	14.9	2.7
BCAT2	16.6	21.1	5.1	4.0	3.0	3.2	4.8	12.8	2.4
PYGO2	11.2	13.3	3.7	6.4	5.8	4.8	7.0	16.8	3.9
TFRC	11.1	17.4	2.6	4.8	3.4	4.9	6.1	19.5	3.0
CYFIP2	12.8	15.6	3.2	5.7	5.5	7.7	5.8	13.9	2.7
TSIX	11.6	13.4	3.2	12.5	10.5	8.5	3.0	8.4	1.9
XPO7	12.3	16.0	2.8	5.4	4.6	4.0	6.2	18.0	3.5
GM2A	13.8	20.0	3.9	7.3	7.0	6.1	3.6	9.7	1.5
TUBB2B	16.5	15.9	5.5	5.4	8.4	2.8	4.5	11.3	2.5
CAPN15	12.4	15.7	4.2	5.7	5.8	5.0	6.5	14.3	3.2
CDC73	12.6	16.9	3.2	6.6	4.8	5.5	4.6	15.8	2.8
SNRNP25	9.9	11.6	2.9	6.9	7.8	6.8	8.1	15.1	3.5
BSDC1	11.9	16.0	3.7	5.2	4.6	4.6	6.8	16.8	3.2
KANSL3	10.8	17.4	3.3	5.5	5.4	5.2	5.9	15.9	3.3
RALBP1	12.4	15.2	3.4	6.5	5.5	5.4	6.2	15.4	2.8
KIAA1279	13.0	17.0	4.1	6.2	5.6	4.4	5.9	13.8	2.8
FAM58A	11.2	13.4	3.6	7.6	6.3	5.3	6.6	15.0	3.7
SCARNA6	8.4	13.6	2.3	4.3	4.4	10.5	8.5	19.6	1.2
NFKB2	12.3	14.7	3.4	5.3	5.0	7.0	6.6	15.4	2.9
EXOC4	12.4	17.1	3.3	6.2	5.0	6.0	5.1	14.4	3.0
REEP3	11.7	16.6	3.7	6.5	6.2	5.6	4.8	14.9	2.6
SNAP47	11.2	14.3	3.3	6.2	6.4	3.9	7.9	15.9	3.5
MPC2	11.6	16.9	3.7	6.0	5.2	7.2	6.0	13.6	2.4
SNRPD1	11.0	15.6	3.4	7.2	4.8	5.4	6.6	16.2	2.3
TPGS1	11.9	15.4	4.6	6.3	5.9	3.9	8.0	12.9	3.5
EIF5	10.8	16.3	3.0	5.5	3.9	7.5	6.4	16.2	2.9
IPO9	11.2	16.6	3.3	7.2	6.2	5.5	4.6	15.3	2.5
PUM2	11.5	16.4	2.5	5.6	4.2	5.4	5.6	18.1	3.0
PPP1R10	13.2	15.2	3.3	6.6	5.7	4.7	5.5	15.1	2.9
LOC100126447	4.4	4.2	1.2	23.6	22.4	12.6	0.9	2.6	0.5
SGCB	10.9	17.6	2.7	6.6	4.9	5.3	5.8	16.0	2.6
LOC101929511	12.8	19.0	3.2	4.3	3.3	7.9	4.5	14.8	2.4
LHX9	16.4	19.2	5.1	3.7	4.4	2.2	6.3	12.2	3.0
SLC39A11	13.6	20.6	3.4	5.4	3.9	4.2	5.6	13.5	2.1
SRRM1	15.3	13.9	3.7	6.3	6.1	4.1	5.5	13.4	3.9
POFUT1	13.7	15.6	3.4	6.7	5.6	4.1	5.5	15.2	2.5
GNAQ	11.9	14.8	3.3	6.9	5.7	5.9	5.6	14.8	3.5
LOC101927476	12.3	19.5	3.4	4.2	2.9	7.1	4.8	15.7	2.4
ADNP	11.8	16.3	2.7	5.9	4.6	5.2	5.1	17.6	3.0
BUB3	10.0	14.0	3.3	7.5	6.6	7.5	5.9	14.8	2.6

FAM131B	9.6	11.7	2.9	5.0	3.9	2.7	10.2	21.5	4.7
RNU5F-1	7.0	28.9	1.9	2.5	1.2	7.9	5.4	16.0	1.4
FBXL17	11.7	14.2	4.1	5.1	4.8	4.1	7.4	16.9	3.9
TMEM106B	10.9	17.6	2.9	6.6	4.2	4.4	5.1	17.3	3.2
PHC2	10.6	14.0	3.3	2.4	3.1	3.0	8.9	22.2	4.6
EIF3E	10.8	19.2	2.4	6.4	4.5	8.7	4.1	13.3	2.6
DCLK2	17.9	25.4	5.9	6.1	5.8	4.1	1.7	4.1	1.0
C10orf32-AS3MT	12.2	18.7	3.0	4.7	3.7	8.4	4.6	14.5	2.3
SRSF4	13.3	11.5	4.3	5.3	5.4	4.7	8.5	14.4	4.6
ZBTB7B	11.0	13.6	3.8	6.2	6.3	4.3	7.4	16.2	3.3
MAP3K3	12.1	13.9	3.4	5.7	5.7	6.0	6.9	15.1	3.2
VTI1B	11.2	14.3	3.2	7.3	6.3	6.6	5.2	14.9	3.1
DANCR	11.9	13.8	3.6	4.2	4.3	3.2	7.9	19.5	3.5
VTA1	13.9	15.4	3.7	6.3	5.3	4.6	6.3	12.6	3.8
STX16	11.5	15.9	2.9	7.0	5.8	7.9	4.6	13.8	2.4
ABLIM3	11.7	16.3	3.1	5.1	4.4	2.9	7.0	18.1	3.3
TRAPPC3	11.9	15.3	3.0	6.7	5.2	5.6	6.0	15.2	3.0
INO80C	9.4	14.5	2.5	4.1	3.6	4.6	7.5	22.0	3.5
CFD	10.9	14.5	3.4	5.9	5.9	15.7	4.5	8.2	2.8
ACD	10.5	12.8	2.9	5.3	5.8	4.3	8.4	18.1	3.8
MED10	10.8	16.2	2.9	5.9	5.5	5.4	5.8	16.4	3.0
NUP93	11.4	14.2	3.2	8.9	7.4	6.0	4.9	12.9	3.0
NUCB2	11.5	15.2	2.6	6.6	5.0	4.8	4.5	18.2	3.6
MAP1S	12.9	14.3	4.6	6.8	7.0	5.3	5.6	11.9	3.4
HOXA9	13.3	14.3	4.4	2.6	2.8	1.1	10.2	16.8	6.4
LIPA	12.4	20.7	2.8	5.1	4.5	5.3	4.9	14.0	1.9
MB21D2	18.1	27.4	5.9	3.8	3.7	2.6	2.5	6.4	1.5
LOC100996251	12.7	19.4	3.1	4.2	3.1	7.0	4.8	15.3	2.2
MAPK1	12.6	17.0	3.9	5.8	5.9	5.7	4.9	13.1	2.9
GLOD4	13.0	16.8	2.9	5.8	3.8	5.4	5.7	15.9	2.4
SLC25A44	10.6	13.9	3.6	6.1	4.6	4.4	6.6	18.6	3.4
TATDN2	10.7	13.7	3.2	5.6	5.3	4.1	6.8	19.0	3.4
DDHD2	12.3	18.3	3.5	5.2	4.3	5.3	5.2	14.8	2.7
SSBP1	11.2	16.1	2.7	6.7	4.5	6.6	5.6	15.5	2.9
CAMTA2	13.6	15.9	3.8	5.8	4.9	4.3	6.0	14.2	3.2
MOB1A	8.4	17.2	1.5	7.4	6.1	9.5	3.7	15.8	1.9
RNF5	10.8	15.1	2.6	7.0	5.8	5.2	6.4	15.1	3.4
NRD1	10.2	14.9	2.6	7.4	5.9	6.6	5.6	15.8	2.4
OAT	9.9	18.3	2.2	6.0	4.8	5.2	5.8	16.7	2.7
MEF2A	15.1	16.3	4.3	4.5	3.8	3.1	4.7	17.1	2.7
RPUSD1	11.3	13.1	3.8	7.1	7.5	3.9	7.2	13.7	4.0
PDK2	11.6	14.7	3.5	5.8	6.2	2.2	7.3	16.3	3.8
KLC4	12.9	14.1	3.5	4.7	4.0	3.4	7.7	17.6	3.5
DPP3	11.1	14.2	3.3	5.3	5.2	4.0	7.6	17.2	3.5
NPRL2	9.2	12.8	2.7	4.5	3.7	3.5	8.4	21.6	5.0
BOP1	10.2	13.1	3.2	5.9	6.0	4.8	8.0	16.5	3.6
RQCD1	11.4	15.6	3.2	5.2	5.4	4.3	5.9	17.1	3.2

TMEM11	9.9	14.9	3.7	4.8	5.5	3.5	8.1	17.0	3.8
SETD5	11.8	15.8	3.2	4.7	4.2	4.4	5.7	17.9	3.4
PPA1	8.8	14.9	1.7	7.2	5.1	6.0	6.3	17.6	3.6
PI4KA	11.8	14.0	3.3	4.7	4.6	5.7	6.9	16.6	3.6
NDE1	12.4	15.1	3.5	4.7	4.4	5.2	6.1	16.4	3.2
GNA13	12.4	15.2	3.0	5.0	4.8	8.3	5.4	13.5	3.5
IP6K1	13.0	13.5	4.3	5.0	5.6	4.3	7.6	14.2	3.7
DDX27	11.6	13.0	3.7	5.6	5.3	4.2	7.7	16.2	3.7
DBNDD1	11.0	12.1	4.2	9.0	9.9	4.9	6.8	9.6	3.4
WDR55	10.3	14.6	3.8	5.9	4.6	4.3	7.0	17.3	3.1
WIPF2	13.0	10.3	3.5	5.9	7.2	4.7	6.5	15.6	4.1
MEN1	12.3	13.9	3.9	4.6	4.9	5.6	7.4	15.0	3.2
FAM53C	12.1	14.4	4.4	6.1	6.1	3.9	6.3	14.0	3.5
XRN2	12.0	15.8	3.2	6.0	5.1	4.2	5.6	15.4	3.4
MBD6	13.5	11.3	4.1	5.4	5.3	3.1	7.9	15.7	4.5
BMPR2	11.3	15.9	3.2	6.0	4.7	4.6	5.1	17.1	3.0
IARS2	11.1	18.2	2.8	6.8	4.9	5.9	4.6	14.2	2.2
ANKRD13D	12.0	14.3	4.1	6.2	6.1	4.9	6.2	13.6	3.3
TLK1	11.7	17.5	3.1	5.8	4.8	4.7	4.7	15.6	2.8
CD82	7.9	10.8	2.4	4.4	4.2	5.2	9.1	22.7	3.8
ARIH2	11.1	15.1	2.7	6.0	5.2	4.6	5.7	16.8	3.3
LOC389641	12.6	18.6	3.6	3.8	2.9	7.5	4.7	14.7	2.2
ESYT2	10.4	13.8	2.7	6.4	5.9	6.6	5.6	16.5	2.7
PRPF40A	12.6	15.9	3.0	5.7	4.9	5.2	5.2	15.0	3.1
LINC00094	11.5	15.7	3.5	5.4	4.5	4.4	6.3	16.1	3.1
UCP2	13.0	17.5	4.4	7.6	6.4	10.9	2.7	6.7	1.3
FADD	12.5	13.9	3.3	7.6	6.0	5.6	6.3	12.5	2.8
POLR3K	9.7	16.2	3.4	8.4	5.9	5.1	6.3	13.7	1.8
ANAPC5	11.0	15.9	2.7	4.9	4.2	4.1	6.8	17.7	3.3
TRIAP1	9.4	12.8	3.9	7.6	6.3	4.0	8.9	13.5	3.9
GNL1	10.6	12.9	3.3	6.2	5.4	4.9	7.3	16.4	3.5
CXorf40B	11.6	16.2	3.4	5.1	5.1	3.3	7.2	16.1	2.5
DSCR3	12.3	14.8	2.9	6.0	5.5	4.2	6.0	16.0	2.8
C1orf198	14.8	17.7	5.2	8.2	7.1	4.4	4.2	7.1	1.7
WASL	13.3	11.7	4.1	5.7	5.9	4.0	7.0	15.2	3.4
PAPOLA	11.2	15.1	2.4	5.1	4.8	6.7	5.2	16.9	3.1
NFKBIL1	11.7	14.1	4.3	5.5	6.2	3.4	7.2	14.6	3.4
AKT3	11.6	18.1	3.0	6.2	4.4	6.9	3.9	13.6	2.6
PAPSS1	10.4	15.1	2.9	7.4	6.3	6.1	5.2	14.4	2.5
PSMB9	10.9	17.8	3.7	5.1	4.5	8.0	5.5	12.4	2.5
FTSJ2	11.5	14.3	3.3	6.1	5.2	6.2	6.5	14.7	2.5
ADCK4	13.9	17.4	3.5	5.3	4.7	3.5	6.2	12.8	3.1
GOSR2	9.2	13.5	2.4	6.1	4.9	4.3	7.2	19.7	3.0
THEM6	10.0	14.3	4.3	5.0	5.0	2.0	8.6	17.1	3.8
NTHL1	11.4	15.1	3.5	7.5	6.7	4.4	6.3	12.7	2.6
WRB	10.4	17.8	2.5	6.1	5.5	4.3	6.3	14.7	2.5
MRPS15	11.7	15.6	2.7	7.1	5.8	4.4	5.0	14.5	3.2

KIAA1551	6.9	10.5	1.3	1.7	1.6	2.6	7.5	32.7	5.3
SRSF11	13.1	14.9	3.4	5.4	4.8	5.0	6.1	14.1	3.2
IFNGR2	11.3	18.2	2.9	5.9	6.0	4.0	4.7	13.7	3.3
RNF4	9.5	18.6	2.4	4.8	4.6	4.8	5.1	18.0	2.3
TMOD3	11.4	16.1	2.8	9.5	7.2	8.1	3.2	9.5	2.2
PFDN1	12.8	10.1	5.0	9.3	9.0	6.4	5.1	9.6	2.7
YTHDF3	13.5	16.4	3.4	6.0	4.8	5.7	4.9	12.3	2.9
TYMS	8.1	10.7	2.7	15.5	12.6	8.5	3.0	6.8	2.0
DENND4B	13.7	15.7	3.4	5.1	4.7	5.1	6.1	13.1	3.0
HINT2	10.2	13.3	3.3	8.1	8.0	6.2	6.2	11.8	2.7
NFAT5	11.5	15.4	3.2	5.5	4.6	4.3	4.6	17.7	2.9
HMGB2	11.8	13.6	3.9	12.3	10.7	9.7	2.2	4.6	0.8
GBF1	11.0	13.4	3.2	4.7	4.4	3.7	6.4	19.9	3.2
LOC101928868	12.4	18.0	3.0	4.2	2.8	8.3	4.6	14.4	2.0
LOC101928328	11.8	19.8	3.1	3.5	2.7	7.5	4.5	14.6	2.3
DMWD	11.2	13.4	3.4	5.5	5.5	4.7	7.2	15.3	3.5
RAPH1	16.0	17.8	4.9	6.2	6.2	3.7	3.1	9.4	2.4
GALNS	11.0	15.8	3.4	5.9	5.9	4.4	6.1	14.2	3.0
CLASRP	14.7	14.8	4.6	4.6	4.5	4.2	7.1	11.1	4.0
CHCHD3	11.0	14.0	3.2	7.9	6.2	4.8	5.5	14.3	2.7
NOC2L	10.8	13.6	3.1	5.9	5.1	4.4	7.3	16.4	3.1
KRT15	1.9	1.8	0.6	0.7	0.7	0.3	19.4	34.5	9.6
DIDO1	10.8	13.2	3.2	5.6	5.3	5.2	6.4	16.9	3.1
POLDIP3	10.6	14.5	3.2	5.2	4.5	4.5	6.8	16.7	3.6
BTBD10	11.0	16.8	2.6	6.6	5.5	4.8	5.1	14.8	2.5
SMG9	13.8	15.2	4.2	5.5	5.4	3.6	5.5	13.6	2.9
MIER2	12.4	15.1	3.8	5.3	5.1	3.5	6.6	14.6	3.0
CORO6	16.1	17.6	3.9	6.1	4.7	2.8	5.6	9.8	2.9
AVL9	11.3	16.4	3.0	5.7	4.9	4.3	5.2	15.9	2.8
PDHB	11.0	16.3	3.0	6.8	5.1	6.0	5.2	13.8	2.2
LOC101928514	12.3	19.2	3.1	3.7	2.9	6.5	4.3	15.2	2.3
LOC101559451	12.6	9.5	6.1	8.9	8.3	3.6	8.4	7.3	4.9
FAM98A	11.7	13.4	3.2	5.5	5.2	4.5	6.6	16.2	3.2
CACUL1	11.0	14.7	3.5	5.8	4.9	5.5	6.1	14.8	3.2
ATIC	10.2	15.2	3.0	8.1	6.8	6.2	4.4	12.9	2.5
ALCAM	11.4	19.8	2.9	7.8	5.5	5.4	3.4	11.0	2.3
TRA2A	11.7	17.9	3.6	5.3	5.1	5.6	4.7	12.6	2.9
CBX5	10.1	14.8	3.6	6.2	5.3	8.0	4.5	14.7	2.1
CCNL1	13.4	14.7	2.2	4.8	2.7	11.9	5.4	12.0	2.4
SAR1B	8.7	15.5	2.1	5.7	3.7	4.2	6.5	20.0	3.0
NT5C3B	10.0	12.8	3.1	7.1	6.9	5.4	7.0	14.1	2.9
LRRC47	11.1	14.8	4.2	5.3	4.7	4.6	6.9	14.4	3.3
UBQLN2	11.1	15.2	2.7	6.2	6.1	4.5	4.9	15.5	3.0
RRP1	12.8	12.8	4.0	6.6	6.4	5.1	7.0	11.7	2.8
KIAA0907	11.7	15.5	2.6	5.1	4.0	4.3	6.5	16.1	3.4
DLG4	11.0	13.6	3.2	4.6	4.3	2.7	7.6	18.3	3.8
SLC41A1	12.4	14.9	2.9	5.2	4.5	3.7	6.5	16.1	3.0

WHSC1L1	11.9	16.1	2.9	6.6	5.8	6.2	4.2	12.8	2.6
SIVA1	10.6	15.4	3.4	8.4	6.4	4.0	6.1	12.7	2.1
CLK3	10.7	14.4	3.5	5.6	5.9	4.7	6.1	14.6	3.6
BLOC1S1	8.5	15.6	2.5	5.1	4.3	3.6	5.6	19.7	4.2
RNF14	11.3	17.0	2.6	7.0	5.1	5.1	4.8	14.1	2.2
FYN	12.1	15.7	3.9	5.7	4.3	6.0	4.9	13.6	3.0
CNOT7	11.5	16.8	2.1	6.7	3.0	6.2	4.3	16.1	2.4
MAU2	10.8	13.4	3.7	4.3	4.5	4.0	7.3	17.4	3.6
ATL3	10.6	18.5	2.8	5.9	4.5	6.7	4.3	13.6	2.0
PACS1	12.6	14.4	4.2	7.1	6.9	7.2	4.2	10.6	1.8
C21orf2	11.5	14.5	3.2	5.4	5.6	3.5	7.7	14.2	3.5
MRPS18A	12.4	13.9	3.4	6.9	7.5	5.9	4.9	10.9	3.1
TMEM161A	12.0	15.5	4.1	6.2	5.8	3.4	6.4	13.0	2.5
B4GALT7	9.9	12.7	3.6	6.0	6.2	4.8	6.6	15.4	3.8
ATG12	12.5	14.1	3.2	6.9	5.4	5.4	4.8	13.1	3.5
LOC101929770	12.0	18.4	2.7	3.5	2.9	7.8	4.4	15.4	1.9
LOC101929259	11.6	17.7	3.2	3.6	3.2	7.7	4.4	15.2	2.2
ENTPD6	12.8	15.2	4.4	6.1	4.3	3.9	5.8	13.3	3.0
FOXK1	12.9	12.5	4.5	5.2	5.8	4.3	6.6	13.0	3.9
RBM17	12.1	14.5	3.5	5.8	5.0	5.4	5.8	13.8	2.9
SMAD2	11.2	17.8	2.6	5.8	4.3	5.5	4.1	15.2	2.3
LOC101927178	11.8	17.9	3.3	3.9	3.0	7.4	4.5	14.9	2.2
R3HDM4	9.7	12.2	3.3	4.7	3.6	8.2	7.4	16.7	3.1
ZFP36	8.1	6.6	2.9	2.9	3.1	34.5	3.3	5.7	1.6
FLCN	13.3	18.8	4.1	5.1	4.9	3.6	5.0	11.6	2.3
TXNDC17	9.0	14.6	2.1	5.6	4.5	4.4	7.1	18.7	2.8
GATAD2B	10.2	13.7	3.4	5.3	5.1	4.7	5.7	17.5	3.0
DCAKD	12.7	18.0	4.4	5.0	5.2	3.0	5.3	12.8	2.3
CKAP5	11.9	16.3	3.0	6.4	5.3	5.1	4.3	13.8	2.4
FRG1	11.7	15.5	2.7	7.6	4.6	3.6	5.1	15.2	2.8
CBFB	12.8	13.1	3.9	5.7	5.2	5.7	6.2	13.0	2.9
C11orf83	10.2	12.4	4.5	6.7	5.9	5.1	8.5	12.0	3.3
TBL3	11.4	14.7	3.9	6.6	6.1	3.4	6.5	13.0	2.9
RERE	11.9	10.4	3.8	6.5	7.2	6.0	6.7	12.3	3.6
MRPL22	12.1	15.1	3.4	5.8	5.2	5.4	5.3	13.4	2.7
CYB561	9.9	13.6	3.0	5.8	5.3	4.0	6.6	16.9	3.3
FOXP4	10.7	11.3	3.2	6.0	6.2	4.2	7.3	16.0	3.6
DOLPP1	10.5	12.0	3.8	4.7	4.6	2.7	7.7	18.0	4.5
TULP4	9.0	13.0	2.7	3.6	3.9	2.8	8.0	21.7	3.8
LOC101929415	11.7	20.1	3.0	2.7	2.0	6.0	3.6	17.6	1.8
PRR12	12.1	12.8	4.6	4.8	5.0	4.2	6.5	14.4	3.8
ASXL1	10.0	14.9	3.3	6.6	5.4	5.2	4.9	15.4	2.5
HLCS	12.2	15.5	3.3	5.1	4.7	3.6	5.6	15.4	2.8
EIF4EBP2	10.9	10.1	3.9	6.4	4.4	3.9	7.7	15.6	5.3
GOSR1	11.3	13.6	3.1	4.5	4.3	4.2	6.4	16.7	4.2
SEH1L	11.3	16.4	3.0	6.2	5.3	5.5	4.9	13.5	2.1
FYTTD1	11.1	15.3	2.9	4.8	4.2	4.3	6.8	15.1	3.7

POP4	10.7	14.0	3.6	7.5	6.8	4.4	5.9	12.1	3.2
ERCC3	11.1	14.6	2.7	5.9	5.0	4.2	6.2	15.2	3.2
POLR2H	9.7	16.6	2.0	7.2	5.2	4.3	5.0	14.9	3.2
COMMD4	11.4	14.7	3.4	9.3	7.3	4.7	4.8	10.6	1.9
MIF4GD	11.6	17.9	3.1	6.8	5.2	4.8	4.6	12.0	2.0
MNT	13.2	11.6	5.2	3.3	4.0	2.6	8.4	14.7	5.0
NDUFB1	8.3	16.2	2.8	7.5	5.9	6.4	6.0	12.0	3.0
ABHD2	10.4	14.2	3.0	3.9	4.0	4.7	6.0	18.5	3.2
PNKP	12.7	15.0	3.9	5.9	5.7	5.4	5.5	10.9	3.0
YPEL3	11.4	12.1	4.2	5.5	5.1	7.6	6.8	11.2	4.0
DYNC1I2	9.8	14.7	2.6	7.0	5.6	4.0	5.3	16.5	2.6
SNTB2	8.6	12.5	2.6	4.6	3.9	4.1	8.0	19.6	4.0
HIST1H2BJ	8.4	10.8	3.0	13.1	13.5	9.5	2.8	5.4	1.5
DCUN1D2	11.7	17.8	3.0	4.1	3.2	7.1	4.4	14.6	2.1
ENC1	8.1	12.4	2.1	6.4	5.2	4.9	6.8	18.7	3.3
LRSAM1	13.4	16.8	3.5	5.0	4.3	3.4	5.5	13.0	3.1
ITPA	11.7	13.2	3.0	7.5	5.5	7.1	5.0	12.4	2.6
DUSP7	10.7	13.4	4.8	5.5	7.2	2.5	6.7	13.6	3.7
PGRMC1	12.4	12.4	4.0	6.4	6.2	4.6	7.7	11.6	2.6
C1QL1	13.2	14.8	6.1	8.2	8.8	5.6	3.2	5.9	2.2
USP9X	11.0	17.2	2.7	5.5	4.3	5.9	4.1	14.7	2.3
MED8	10.1	15.8	3.0	4.8	4.1	3.5	6.6	16.8	2.9
LUC7L	12.3	15.3	2.7	3.8	3.0	3.1	8.1	15.3	4.1
AGTRAP	11.7	15.0	3.8	6.6	7.2	3.1	5.8	11.9	2.5
PREPL	9.7	15.1	1.9	5.5	4.0	4.8	4.9	18.3	3.4
PRKAR1B	11.1	14.1	3.6	6.6	6.3	4.8	5.9	12.5	2.6
TARS2	11.4	17.6	3.0	5.8	4.4	6.3	4.3	12.8	2.1
RRP1B	11.3	13.4	3.5	5.5	5.6	5.2	5.6	14.6	2.9
SKI	10.4	12.9	3.8	4.7	4.7	8.0	6.7	13.0	3.4
LRP8	10.6	14.3	3.2	6.7	6.3	4.0	5.3	14.5	2.8
NUS1	10.3	15.7	2.8	5.2	5.5	4.1	5.9	14.9	3.2
RDX	7.2	12.3	1.9	6.2	4.5	3.8	5.8	22.1	3.7
C7orf50	11.4	13.6	4.6	6.5	5.4	4.0	6.5	12.7	2.9
UHRF1	10.1	12.4	4.1	10.7	10.7	7.2	3.7	7.0	1.6
PRMT5	9.8	14.0	3.2	6.1	5.6	4.4	6.3	15.5	2.5
GLYR1	9.2	13.5	2.9	6.0	6.7	4.9	5.1	15.9	3.3
RILPL1	13.4	13.4	5.6	5.0	5.5	2.1	7.3	11.3	3.7
DTX3	12.8	15.6	3.5	4.7	4.4	2.7	6.0	13.8	3.9
NME1	9.9	15.2	2.8	5.7	3.9	5.7	6.5	15.5	2.3
MGEA5	12.1	18.1	3.4	4.3	3.7	5.6	4.4	13.6	2.2
USP19	10.8	14.6	3.0	5.5	4.7	5.2	6.2	14.8	2.7
PTPN12	10.4	13.3	2.4	6.2	4.8	5.2	5.0	16.6	3.5
BBX	10.5	13.6	2.6	6.0	5.3	4.7	5.2	16.2	3.1
LOXL1-AS1	14.3	19.9	4.1	7.6	6.4	6.4	2.2	5.4	0.9
FAM219A	13.0	12.1	3.8	7.1	6.4	2.9	6.1	12.7	3.1
WBP1L	10.8	14.5	3.6	5.1	5.5	4.6	5.6	14.4	3.3
PLEKHM2	11.1	13.3	3.4	7.1	6.6	5.8	5.3	12.1	2.6

GFER	9.1	12.9	5.0	6.8	7.4	4.8	6.0	11.6	3.6
PPT1	13.0	15.2	3.7	7.2	5.7	5.7	4.1	10.6	1.9
CDC16	10.5	14.8	2.5	6.5	6.1	5.3	4.7	13.6	3.0
TIAF1	9.3	13.9	2.8	7.3	4.4	6.0	5.2	16.0	2.3
CACYBP	8.6	12.7	2.1	7.8	6.2	7.8	4.4	14.6	2.8
WIPI1	12.1	15.7	3.4	3.7	2.8	2.2	6.9	16.8	3.6
GLRX5	10.0	10.5	3.8	6.1	7.2	4.9	8.2	11.8	4.7
VPS29	10.3	17.5	2.5	7.7	5.6	6.6	3.9	10.6	2.4
PSMG3	10.7	14.6	3.9	6.4	5.5	5.6	5.2	12.7	2.4
STIM1	12.3	14.7	3.6	5.3	4.6	4.3	5.3	13.7	3.2
GSR	11.8	17.0	3.0	6.6	4.8	4.7	4.6	12.2	2.4
ABL2	12.5	17.8	3.5	7.4	8.4	5.5	2.6	7.9	1.3
GATM-AS1	12.0	15.5	3.2	4.5	2.6	9.3	4.6	12.8	2.7
FAM195B	12.1	14.6	4.3	4.0	5.0	2.5	7.0	14.0	3.5
CMPK1	10.3	13.5	2.7	6.3	4.1	5.3	6.7	14.7	3.5
PHC3	11.8	15.1	3.0	4.6	4.1	4.6	5.2	15.8	2.7
UBALD1	11.2	8.0	4.0	5.6	6.8	2.4	8.3	14.9	5.7
ANKIB1	10.6	16.4	2.5	5.6	4.4	4.6	4.4	15.8	2.4
AIDA	10.0	14.8	2.6	6.5	5.1	6.4	4.6	14.2	2.8
EXOSC4	10.9	14.4	3.6	5.4	5.5	3.6	6.2	13.6	3.7
NUDT3	10.6	13.9	3.9	5.6	4.8	4.7	5.9	14.7	2.7
ABCF2	11.2	13.1	2.7	6.8	5.3	4.5	5.6	14.7	2.8
SNORD97	4.4	21.2	1.0	2.5	0.4	9.0	5.9	21.9	0.5
GRK6	10.7	13.8	3.6	6.5	5.6	7.8	5.2	11.7	1.8
TMEM185B	10.6	17.5	3.4	5.6	4.8	4.4	5.0	12.4	3.0
TOR2A	11.7	14.9	4.5	5.3	5.7	2.9	6.6	12.3	2.8
STT3B	11.4	15.9	3.0	5.2	4.7	6.6	4.4	13.1	2.4
ARFGAP2	11.4	16.6	3.2	5.8	4.2	6.0	5.0	12.2	2.4
ACOT9	12.9	17.8	2.8	5.7	4.2	4.9	4.2	12.0	2.3
TMEM150A	12.3	16.3	3.5	4.0	3.9	1.9	6.8	15.2	2.8
GUCD1	9.9	14.7	3.7	7.2	5.4	5.6	5.2	12.4	2.6
MNF1	10.0	14.3	3.5	9.5	5.9	7.1	4.5	10.4	1.4
MIB1	11.5	17.5	3.0	5.0	4.0	5.7	4.6	13.4	2.0
NECAB3	11.0	14.5	3.3	7.7	5.8	4.0	5.6	12.3	2.4
TBL1XR1	9.2	13.6	2.4	5.9	4.4	6.0	5.2	17.1	2.7
METTL5	10.9	17.6	2.5	5.9	4.5	4.6	4.0	14.5	2.1
IQSEC2	10.7	11.8	3.1	5.2	4.5	2.8	7.0	18.2	3.4
COPS8	10.1	15.1	2.4	7.1	6.0	6.1	4.5	12.7	2.5
RABGEF1	11.8	16.5	2.8	5.3	4.3	4.1	4.9	13.8	3.0
HDAC2	10.7	15.0	2.6	4.8	4.1	6.6	4.9	15.1	2.7
DLD	9.2	17.6	2.4	6.3	4.6	5.9	4.0	14.1	2.3
MCOLN1	11.8	13.3	3.3	8.0	6.9	4.1	5.5	10.7	2.9
AP1B1	10.5	13.0	3.3	6.6	6.3	5.0	5.6	13.2	3.1
FAM193B	13.3	15.4	3.3	3.7	3.2	2.7	7.1	14.2	3.6
RPP21	11.0	11.3	3.0	8.5	7.0	3.6	6.1	12.0	3.8
C11orf80	10.7	16.9	2.9	6.0	4.6	4.1	5.0	13.9	2.3
FAM171A1	12.5	16.5	4.6	5.7	5.3	3.1	4.8	11.3	2.6

NAA50	8.9	14.3	2.0	7.1	4.1	7.8	4.7	15.7	1.8
LOC101927710	10.3	19.1	2.6	3.3	2.7	6.6	4.5	15.4	1.9
NF1	10.1	22.7	2.7	3.9	3.1	3.4	3.8	14.4	2.2
PTP4A1	11.2	16.7	2.5	4.6	3.2	5.6	5.2	14.2	3.1
PPAN	12.9	11.7	3.5	5.4	4.6	4.0	8.6	12.1	3.3
PDE4DIP	11.8	15.7	3.2	5.6	4.8	4.1	4.8	13.5	2.6
FAM220A	9.9	12.9	3.2	5.7	4.2	5.5	5.6	16.4	2.9
USP4	12.1	15.2	3.4	5.5	4.7	6.3	5.0	11.6	2.3
FIP1L1	12.4	14.1	3.7	5.0	4.0	3.2	5.7	14.6	3.5
CNOT11	10.4	14.3	2.7	6.1	6.0	4.6	5.2	13.6	3.4
GORASP1	11.6	13.3	2.7	5.5	5.7	3.2	6.0	14.7	3.5
TPRG1L	10.6	13.3	3.7	5.7	5.5	4.6	6.5	13.9	2.5
ARGLU1	11.2	11.9	2.9	5.8	4.5	7.8	5.9	13.3	2.9
ITFG1	10.4	16.3	3.1	5.9	5.1	5.3	4.6	12.6	2.6
CRKL	10.5	14.7	2.9	5.3	4.5	5.3	5.1	14.5	3.2
ELP4	11.4	17.1	3.1	4.4	3.3	7.3	4.3	12.9	2.4
PSMD9	11.7	13.8	3.6	5.4	5.8	4.8	6.1	12.2	2.6
C11orf48	12.4	9.6	5.1	7.0	5.8	5.2	7.2	10.6	3.2
LMF2	8.4	11.4	2.8	7.1	6.6	5.6	6.2	14.5	3.4
AKAP17A	10.6	11.7	3.4	4.5	4.3	4.9	8.4	14.2	4.2
AKAP17A	10.6	11.7	3.4	4.5	4.3	4.9	8.4	14.2	4.2
C14orf166	10.3	14.6	2.4	6.3	4.4	6.3	5.0	13.3	3.4
SGSM3	10.0	13.9	3.6	5.7	4.4	3.2	7.5	14.9	3.0
VPS26B	10.9	14.0	3.2	5.3	4.4	3.5	6.5	15.1	3.1
AP5S1	11.2	14.3	3.7	6.1	6.4	3.0	5.2	13.3	2.7
CLDN4	10.3	10.9	3.0	7.4	7.3	3.6	6.5	14.2	2.8
CLN8	10.1	16.0	3.2	5.0	4.3	4.3	5.9	14.6	2.6
EBNA1BP2	11.3	13.6	3.1	6.4	5.3	4.8	5.4	13.3	2.7
TTC19	13.6	18.2	3.6	4.5	4.4	3.4	4.6	10.9	2.7
KIFC2	11.8	14.6	3.4	6.3	5.5	4.0	6.3	11.5	2.6
PRRT2	13.6	15.3	4.1	3.7	3.2	1.6	6.4	14.3	3.7
TSC22D2	11.5	14.4	4.2	6.5	7.3	6.9	3.9	9.1	2.1
STRADA	11.0	15.5	2.7	5.9	5.4	3.7	5.2	14.0	2.4
HIST1H3F	10.1	12.7	3.1	11.6	11.8	6.5	2.8	5.6	1.7
CWC25	11.1	14.4	3.1	4.1	3.1	9.7	4.9	13.0	2.4
LOC101928597	10.6	16.9	2.9	3.9	2.8	6.6	4.8	15.0	2.2
MEIS3	12.2	14.5	3.5	5.9	5.5	3.0	5.6	12.4	3.2
LYPLA2	9.9	12.8	2.5	4.9	6.0	6.0	6.4	14.1	3.1
ZFAND2B	11.2	12.4	3.4	5.5	6.2	3.2	7.1	13.4	3.2
RBM10	11.2	13.2	3.3	4.9	4.8	3.8	6.6	14.7	3.3
BTBD19	15.7	19.6	3.9	3.2	3.1	3.5	4.4	10.4	1.9
CANT1	10.6	13.2	3.2	5.9	5.5	3.9	6.3	14.5	2.6
HSD17B1	11.3	13.4	3.3	5.2	4.2	3.0	7.1	14.2	3.9
GAPVD1	10.5	15.4	2.6	5.2	4.2	4.8	4.4	15.6	2.7
REXO1	11.1	12.5	3.8	5.4	5.7	4.9	6.6	12.6	3.1
TSPAN14	10.2	18.0	2.6	4.6	3.8	5.3	5.2	12.7	3.1
HIST1H2AL	8.6	8.3	2.9	13.3	12.6	13.5	1.5	3.3	1.3

ZGPAT	12.8	13.0	3.8	4.7	4.0	3.5	7.0	13.0	3.7
TRIM22	9.1	14.2	2.4	3.7	2.4	5.8	6.6	17.9	3.4
CCDC90B	9.2	13.7	2.6	6.3	5.2	5.4	5.6	14.8	2.8
PELP1	11.6	12.1	3.6	6.2	5.3	4.3	6.1	13.3	2.9
ZFAND6	11.0	13.6	2.6	4.9	5.3	4.5	4.5	15.6	3.5
ZBTB18	10.8	13.0	3.1	5.1	5.4	5.3	6.1	13.0	3.6
LOC101927340	10.1	15.3	2.2	3.9	2.7	10.0	3.8	15.6	2.0
VMA21	10.0	13.6	3.0	7.2	4.4	4.0	6.6	14.4	2.3
ANKZF1	11.1	14.9	2.9	3.7	2.7	4.1	7.3	15.7	3.1
LOC101929767	12.1	15.2	3.8	6.4	6.2	3.5	4.0	11.9	2.3
RABEP2	9.3	11.4	3.4	3.8	4.0	4.3	8.6	16.7	3.9
NPIPA1	11.8	14.7	2.7	4.7	3.8	2.0	5.6	16.5	3.7
SNAP23	9.0	12.5	2.8	5.4	4.6	5.6	6.3	16.2	3.0
SMO	10.8	13.9	3.5	6.4	6.4	3.5	5.2	12.7	2.9
BOLA3	9.1	16.2	2.6	8.4	7.1	4.6	4.0	11.6	1.6
TRIM65	10.4	13.7	3.2	4.6	4.6	3.6	6.5	15.4	3.3
MAD2L2	11.1	15.6	3.3	5.9	7.1	5.1	4.0	10.8	2.3
FLJ42102	11.4	18.0	2.7	3.7	2.9	6.9	4.2	13.2	2.2
XIAP	11.0	17.2	3.0	4.2	2.9	6.5	4.4	13.6	2.4
OSER1	10.4	14.3	2.2	5.2	4.4	3.8	6.4	15.8	2.7
ARHGAP17	12.3	14.0	3.6	6.0	5.4	5.4	4.6	11.1	2.8
DCUN1D3	8.1	12.0	1.7	3.4	3.0	2.6	9.4	21.8	3.2
IL32	14.3	19.8	4.8	4.7	4.0	5.5	3.4	7.4	1.3
RBFOX2	11.5	14.1	3.5	4.9	4.9	3.2	5.7	13.9	3.5
GFPT2	6.4	8.8	1.8	6.4	5.5	3.6	6.8	22.0	4.0
PPP5D1	11.8	18.4	2.9	3.4	2.4	4.4	4.4	15.0	2.4
TAB2	9.4	12.6	2.0	6.2	5.3	5.2	4.7	16.4	3.3
SDHAF2	7.7	14.1	2.5	6.1	4.8	6.7	4.6	16.3	2.3
SAMD1	8.3	10.4	3.9	6.2	6.5	5.0	7.3	13.9	3.5
C10orf10	14.1	15.8	3.9	3.4	2.6	1.9	6.8	12.8	3.8
SGK196	9.7	13.7	2.6	4.3	4.1	3.5	5.7	18.6	2.8
ANKRD13C	10.6	14.0	3.2	5.6	5.3	6.8	4.7	12.2	2.6
IGFN1	20.5	18.8	5.1	7.1	4.9	3.2	1.5	3.1	0.7
LOC101929165	11.3	17.9	3.0	3.8	2.4	6.9	4.0	13.5	2.0
MRAS	12.4	14.0	3.6	4.8	5.4	2.8	5.9	13.7	2.2
ATP6V1A	10.4	16.2	2.3	6.4	4.5	5.6	4.4	13.2	1.8
ARHGEF18	10.6	13.2	3.9	5.3	5.4	8.4	5.0	10.7	2.4
SATB2	10.7	13.2	3.1	4.7	4.0	2.6	6.1	16.7	3.7
TIMM8B	9.9	13.1	2.4	6.5	3.9	6.5	6.6	13.5	2.5
SNORA12	9.0	17.2	2.0	4.7	4.1	4.7	9.3	11.8	2.1
M6PR	9.2	12.9	2.7	6.3	4.8	6.8	5.3	14.4	2.4
GPX8	10.0	14.7	2.1	5.2	4.3	4.6	4.1	16.6	3.1
LAT	13.8	17.1	3.9	4.3	4.4	5.9	4.3	8.7	2.2
C14orf23	12.1	14.5	3.4	4.6	3.2	8.2	4.8	11.9	2.0
DIP2C	11.2	14.5	3.8	4.6	4.9	4.4	5.3	13.3	2.7
LOC441528	10.7	13.4	3.1	6.6	5.5	4.2	5.1	13.0	3.0
FKBP14	10.5	15.6	2.4	3.5	2.7	5.5	4.8	17.3	2.4

MRPL19	8.5	14.6	2.7	5.6	4.7	5.8	6.0	14.2	2.5
RFTN1	13.9	16.3	3.4	5.3	4.4	3.9	4.5	10.4	2.5
COPS7A	11.7	14.6	4.0	5.6	5.6	3.6	6.0	11.7	1.7
MCM2	10.9	13.2	3.7	10.5	9.5	5.6	3.0	6.7	1.4
THOC6	11.6	14.2	3.5	6.5	5.7	4.5	5.0	11.1	2.4
FNTA	8.8	12.2	3.0	5.3	4.5	4.2	7.1	16.7	2.8
CNIH	10.5	12.3	3.0	5.8	4.8	2.8	5.3	15.2	4.8
IDH3A	9.2	15.0	2.2	4.3	4.3	4.6	5.7	16.7	2.5
TIPRL	11.1	15.1	3.3	4.9	4.3	4.1	4.4	14.0	3.3
AGPAT5	8.3	13.7	2.7	5.3	4.9	4.5	5.9	15.9	3.4
DNAJC10	8.4	14.1	1.7	4.9	3.7	4.8	5.1	18.6	3.1
SNX33	13.2	15.7	4.4	5.2	4.6	2.8	5.0	11.5	2.0
AJUBA	11.1	15.0	3.4	9.2	9.1	6.9	2.7	5.5	1.4
DNAJC9	11.4	12.4	2.3	6.0	4.0	4.7	6.5	14.2	2.8
UBR4	11.2	16.0	3.7	4.5	3.8	4.3	3.8	14.8	2.1
NEURL4	11.2	14.6	2.7	4.0	3.2	3.2	7.5	14.7	3.3
SETD8	10.5	11.5	3.3	7.0	5.6	5.0	5.4	13.4	2.6
SNORA38B	1.7	13.7	2.6	1.8	2.7	10.0	9.2	21.0	1.7
RNF220	11.4	13.0	3.2	5.7	5.2	3.7	6.4	13.3	2.4
AAAS	9.3	13.7	2.7	4.4	3.8	3.1	6.6	16.8	3.9
ARHGAP21	9.7	11.9	2.6	4.4	4.0	3.3	6.3	18.5	3.6
GMPS	10.8	17.4	2.2	5.9	4.9	5.1	3.4	12.3	2.2
METTL23	8.9	14.2	2.0	6.0	4.5	5.2	5.6	16.0	1.9
PI4K2A	10.7	16.0	3.5	5.3	5.0	4.2	5.0	12.0	2.6
G3BP2	10.1	12.7	2.4	5.9	4.0	4.4	6.0	16.0	2.8
RBM14-RBM4	7.5	18.3	3.5	3.8	1.8	5.1	5.6	17.7	0.9
ALAS1	10.5	15.0	2.6	4.4	4.4	3.5	5.4	15.7	2.6
ETFB	11.0	13.4	3.6	6.0	5.0	4.2	6.6	12.0	2.3
CELSR2	12.3	14.7	4.3	5.9	5.2	3.2	4.7	11.5	2.3
SNRPB2	10.6	16.0	2.5	5.6	4.4	3.9	3.9	15.0	2.1
FLAD1	8.7	14.1	3.1	5.3	4.7	3.9	6.4	15.2	2.8
LOC101928287	10.2	13.5	3.1	3.9	2.8	12.8	4.2	11.6	2.0
LOC100507507	8.8	12.7	3.5	5.0	4.6	4.0	7.7	14.5	3.2
SNORA80B	2.4	18.1	2.7	3.7	2.2	9.3	10.6	11.7	3.2
MEGF8	10.4	13.9	3.4	5.1	4.7	3.3	6.0	14.6	2.7
XXYLT1	13.2	16.4	2.7	3.7	3.3	2.6	6.1	13.1	2.7
AGFG2	5.5	6.9	1.8	14.7	13.8	7.8	3.2	8.2	2.0
FBXL16	11.7	13.4	3.7	6.6	6.5	2.8	5.6	11.4	2.1
FBXL19-AS1	10.2	15.3	2.8	3.7	3.0	4.5	5.4	16.4	2.6
LEPROTL1	9.6	16.1	2.1	4.7	2.9	4.2	5.5	15.8	2.9
TGFBRAP1	10.8	13.3	3.5	6.3	5.5	4.5	5.1	12.2	2.7
CDC42BPB	11.3	14.4	3.6	5.0	4.1	3.0	5.9	13.9	2.7
SLC16A4	10.3	15.8	2.6	5.0	3.8	5.9	4.1	13.7	2.6
TRIM52-AS1	10.8	15.5	3.2	4.4	3.7	5.7	4.6	13.7	2.2
ZXDC	10.4	14.5	3.6	4.4	4.0	3.0	5.2	15.3	3.3
CAMLG	9.5	11.9	2.5	5.2	4.4	4.8	6.7	16.2	2.5
ZNHIT3	10.0	14.7	3.1	6.4	3.4	3.8	5.3	14.1	3.0

SMG6	9.1	12.2	2.9	5.0	5.1	5.1	5.9	15.4	3.0
PPAP2A	8.8	13.5	2.6	7.3	6.3	5.0	4.5	12.9	2.6
RNU5D-1	5.2	22.3	2.7	2.9	1.7	11.5	4.6	11.6	1.3
ADO	9.9	13.2	3.5	4.8	5.5	3.1	6.4	13.4	3.9
PARL	10.3	14.3	2.9	5.5	4.1	4.1	5.8	14.0	2.6
HSD17B14	14.5	18.1	4.0	5.6	6.0	3.2	4.2	6.4	1.7
TMEM120B	9.1	14.5	2.4	3.6	3.0	2.2	8.1	17.2	3.4
SRSF5	9.1	12.0	2.1	4.2	3.0	8.2	5.8	16.0	3.4
XPC	9.9	12.0	3.3	4.8	4.1	4.2	6.6	16.0	2.8
AEN	10.4	13.4	3.8	5.3	4.4	3.4	7.1	12.7	3.0
ATP13A3	11.1	18.0	2.6	4.8	3.2	4.5	3.7	13.4	2.2
TIMM44	11.2	12.6	3.3	4.7	4.4	4.4	6.6	13.1	3.3
FKBP1A-SDCBP2	11.6	11.8	4.2	5.3	6.3	1.8	7.2	11.7	3.6
RGS10	10.1	14.7	3.7	6.8	5.6	3.8	4.4	10.8	3.5
TRIM27	9.7	13.8	3.0	4.9	4.5	3.1	5.7	15.9	2.8
ELMSAN1	12.0	12.7	4.0	4.2	4.3	4.0	6.3	13.0	3.0
NELFA	11.9	12.3	4.3	4.2	5.3	4.8	6.1	11.4	3.0
HBP1	9.2	14.5	2.0	3.9	2.5	3.0	6.6	18.9	2.8
SEC24D	10.3	13.6	2.8	3.4	3.0	1.8	6.8	17.9	3.8
SHB	10.7	12.0	4.5	5.9	5.9	3.9	5.5	12.2	2.7
TRIM5	8.7	13.4	2.2	5.7	4.5	5.5	5.3	15.4	2.6
PMF1-BGLAP	11.2	10.3	2.9	4.7	6.0	3.0	8.2	11.9	5.0
USP36	11.2	12.3	3.3	3.6	3.5	4.5	6.8	14.9	3.2
AAGAB	11.1	16.9	2.5	4.1	4.3	4.5	5.3	12.9	1.7
TBC1D14	11.0	14.7	3.1	4.9	4.6	4.3	5.2	12.6	2.7
SLAIN2	10.4	12.9	2.9	5.5	4.9	3.7	5.5	14.2	3.2
LOC101929147	11.2	18.5	3.2	2.7	2.4	3.3	3.9	16.2	1.8
DPH1	10.3	13.6	2.7	5.1	4.6	3.6	6.8	13.2	3.3
MAPK12	12.9	15.4	3.5	6.4	4.8	2.8	5.3	10.0	2.1
JPH3	12.1	11.8	4.0	5.4	6.0	3.1	6.0	12.2	2.6
PHLDB2	14.1	19.6	3.8	4.1	3.6	3.2	3.6	9.0	2.0
PANK3	11.2	17.2	2.5	4.5	3.1	5.1	4.0	13.6	1.8
PRCP	10.3	13.3	3.1	6.5	4.9	5.3	5.1	11.8	2.7
HIST1H2AH	7.0	10.1	3.1	13.5	11.1	8.6	3.4	5.0	1.2
MATN2	11.4	16.1	2.9	9.6	7.8	6.9	2.1	5.1	1.1
ZNF24	9.9	15.9	2.2	3.7	3.2	4.5	5.7	15.1	2.7
TM9SF1	9.0	12.7	2.5	4.9	4.2	4.2	5.7	17.2	2.7
FKBP4	9.1	12.7	2.7	6.3	5.4	5.0	5.8	13.9	2.0
DPCD	8.8	11.3	3.6	5.6	6.2	4.9	6.6	13.0	3.1
FLJ22184	13.1	11.6	5.0	5.8	6.3	2.2	5.4	10.2	3.4
CLCN3	9.2	12.7	2.0	4.2	3.2	4.2	5.9	18.5	2.9
SNX5	10.8	15.1	2.2	6.3	4.5	6.2	3.7	11.5	2.7
PEX16	10.6	14.0	3.3	5.1	4.2	4.2	6.3	12.4	2.8
FOXD1	6.8	7.6	2.9	3.4	2.6	1.9	10.9	21.5	5.3
SEMA4C	10.2	13.5	3.0	3.9	3.4	2.7	6.8	16.3	3.2
KDM2A	9.0	12.1	2.6	4.1	3.8	5.0	5.9	17.3	3.0
ZDHHC8	11.2	12.2	3.7	5.4	5.3	3.6	5.9	12.5	3.2

GPN1	11.8	15.5	3.1	5.5	3.9	4.1	4.5	12.1	2.4
C6orf223	8.5	7.2	12.4	8.6	5.1	0.7	9.0	6.1	5.1
TRIM2	12.5	15.2	4.1	3.8	3.2	2.4	4.7	13.6	3.2
LTA4H	9.7	15.0	3.0	6.3	5.2	4.8	4.4	11.8	2.6
MRI1	11.3	15.1	3.2	4.3	3.6	5.8	5.2	12.4	2.0
KCTD7	10.2	14.8	3.9	4.5	4.3	3.6	5.6	13.6	2.3
EIF2B4	9.9	13.3	2.7	5.0	4.3	3.0	7.0	14.6	2.9
VPS16	12.1	14.3	2.9	4.8	3.9	4.7	5.6	12.4	2.1
PROSC	9.9	12.6	3.7	5.4	5.6	3.4	5.8	13.3	2.9
EHD4	11.9	15.1	4.1	5.3	5.6	4.3	4.4	9.6	2.2
NSD1	10.6	15.2	2.8	4.3	3.4	4.4	4.6	14.5	2.6
INE2	11.4	13.3	3.0	3.9	3.2	10.4	4.1	11.5	1.9
DYRK1A	11.3	14.2	2.5	5.0	4.1	4.8	4.6	13.7	2.4
SEC11A	9.1	15.3	2.2	6.3	3.7	5.3	5.2	12.8	2.6
FDX1L	10.8	13.2	3.5	6.9	5.3	3.6	5.7	11.4	2.1
LOC100129269	10.7	14.5	3.5	3.7	3.1	6.2	4.9	13.8	2.1
PEX19	10.3	19.2	3.1	5.7	4.3	3.8	3.6	10.0	2.5
BRD7	10.1	11.3	2.5	6.7	5.5	6.1	4.9	12.4	2.9
SLC1A4	9.9	13.9	3.0	3.0	2.9	2.2	6.6	17.8	3.3
CHTF8	10.6	13.4	2.5	6.1	5.7	4.8	4.4	12.6	2.3
GPKOW	11.4	13.5	3.5	4.9	4.9	4.7	5.2	11.8	2.6
UGCG	14.0	22.0	3.8	5.0	3.2	4.5	2.3	6.5	1.2
VPS41	10.7	16.5	2.8	6.0	4.0	5.7	3.8	10.9	2.0
ZFP36L2	7.0	6.5	3.6	2.6	3.2	28.0	3.2	6.6	1.8
LRFN3	10.8	11.7	3.5	5.5	5.8	2.5	6.8	12.3	3.5
HIST1H3H	8.9	11.3	2.6	8.3	8.0	6.8	4.9	9.7	1.9
ZFP90	11.4	15.1	2.3	4.7	4.0	4.6	4.4	13.4	2.6
DSG2	12.5	18.9	3.5	7.3	5.3	5.6	2.0	6.0	1.2
GBAS	11.6	16.0	3.1	3.7	3.2	3.3	5.5	12.6	3.3
CCDC12	10.2	11.2	3.7	4.0	4.8	4.9	7.6	13.2	2.7
ARL6IP6	9.0	13.0	2.4	5.9	4.6	5.0	6.6	13.3	2.5
GPR161	10.1	12.7	3.2	6.0	6.2	3.5	5.2	13.2	2.2
LOC100188947	11.1	11.2	3.9	8.8	8.3	6.6	2.5	8.6	1.4
B4GALT3	9.8	13.9	2.7	5.9	5.6	4.7	4.8	12.8	2.2
YES1	9.5	12.8	2.5	5.5	4.1	4.8	4.8	15.0	3.2
MAP4K2	9.4	12.3	3.4	6.1	6.3	5.5	5.1	11.7	2.5
MMP11	7.8	9.1	2.0	1.6	1.2	1.1	10.5	24.1	4.9
DIAPH1	9.1	12.5	2.7	7.0	6.4	7.0	4.2	11.5	1.8
CISD2	7.0	15.1	1.8	5.2	3.8	5.0	6.2	16.1	2.0
SNX18	9.0	11.3	3.7	6.0	5.3	5.7	6.1	12.0	3.1
GNPDA1	12.0	15.9	2.8	6.9	4.2	3.6	3.8	11.4	1.6
SNORA74B	12.4	17.4	4.3	4.1	4.1	2.9	5.1	10.3	1.6
FOXO3	9.5	12.9	2.6	4.2	4.0	4.6	5.1	15.6	3.8
GSTM3	10.7	15.5	3.1	6.2	4.4	5.9	3.6	10.9	1.8
LOC101929046	13.0	8.2	4.2	7.0	6.2	3.2	7.0	9.7	3.6
ITGB8	6.1	9.2	1.3	6.8	5.5	5.3	6.0	18.8	3.1
MRPL36	8.7	13.9	3.3	5.8	6.4	3.3	4.8	13.4	2.5

TP53BP1	8.6	12.7	2.8	4.6	3.8	4.0	5.6	17.2	2.8
RSRC1	10.4	11.7	2.6	5.1	4.4	4.2	5.4	15.5	2.8
SIRT3	9.9	14.4	3.4	5.3	4.3	3.4	6.0	12.6	2.7
PRKAG2	14.2	20.3	3.4	6.0	4.9	4.8	2.2	5.2	1.1
TMEM33	8.5	14.8	2.4	6.9	4.7	4.1	3.3	14.9	2.5
XPO1	8.9	14.9	1.9	6.9	4.6	6.6	3.5	12.3	2.3
TMEM87B	8.7	13.7	2.1	5.4	4.1	4.9	5.7	15.0	2.5
SAE1	10.1	13.4	2.8	6.8	5.7	5.3	4.3	11.2	2.3
NUDT1	10.9	13.5	3.7	7.4	6.3	3.7	4.4	9.9	2.1
KDM5B	10.5	14.2	2.8	4.2	3.6	3.1	4.8	16.1	2.6
OSTC	7.8	13.9	2.4	8.4	4.8	5.4	4.0	13.3	1.8
IL6ST	8.4	12.5	2.1	5.3	4.5	4.9	4.9	16.3	2.9
POLR1D	12.2	11.5	3.3	6.3	5.7	6.1	4.1	10.9	1.8
DERL1	9.0	12.2	2.4	5.0	3.4	3.2	7.4	15.6	3.5
HDGFRP3	10.9	13.2	3.0	6.9	5.3	6.5	3.4	9.8	2.8
HMGN4	10.8	17.0	3.3	4.9	3.6	2.7	4.8	13.1	1.6
TEAD2	8.9	11.7	2.9	5.9	5.9	2.9	5.7	14.4	3.4
LOC101928118	10.5	17.6	3.3	2.4	1.8	7.4	3.4	13.7	1.6
HLTF-AS1	11.1	11.5	3.8	6.6	5.1	1.4	7.3	12.2	2.8
44819	6.6	15.8	1.7	4.5	2.6	7.7	5.2	15.3	2.3
DPH2	9.3	12.5	2.2	3.6	3.1	2.5	7.4	18.2	2.8
RAB40C	10.9	13.3	3.7	3.4	3.1	2.7	6.5	14.2	3.9
EIF2AK4	10.0	13.5	3.1	5.9	5.0	4.2	4.9	12.4	2.5
SCARF2	11.2	12.7	4.2	6.9	7.9	3.5	4.5	8.3	2.4
PIAS4	10.9	13.2	3.5	4.7	4.7	3.8	5.2	12.7	2.9
DHX9	8.7	12.6	2.1	5.4	4.3	4.9	4.7	16.0	2.8
CCDC6	9.8	11.3	3.2	4.9	5.3	3.7	6.0	13.9	3.4
ALDH7A1	10.6	11.1	2.8	7.0	5.1	5.1	6.6	11.3	2.0
BNIP2	8.6	14.5	2.6	4.2	3.9	4.9	5.8	14.0	3.0
PIDD	13.2	14.8	3.4	4.2	3.7	1.8	6.3	10.9	3.1
PFKM	10.4	15.6	2.9	5.4	4.3	4.0	4.6	12.2	2.1
FAM207A	9.5	10.0	4.6	7.3	7.0	5.6	5.4	9.7	2.4
RNF25	10.6	13.5	3.1	4.8	4.9	3.5	5.8	12.2	3.0
UNK	11.9	13.3	4.0	4.8	4.7	3.8	4.8	11.1	3.2
ALG2	8.5	11.3	2.4	3.8	3.7	2.9	7.6	17.9	3.4
C11orf73	9.7	16.5	2.8	6.1	3.8	3.1	4.1	12.4	2.9
EMP1	15.4	20.8	5.6	3.2	2.7	1.4	3.0	8.2	1.0
CDK12	10.8	12.3	3.3	5.0	4.7	4.6	4.7	13.3	2.7
UGGT1	9.8	15.2	2.5	4.4	3.3	5.5	4.3	14.1	2.2
FAM106A	10.6	17.2	3.0	3.2	2.6	5.4	3.7	13.5	2.0
PSMD12	9.3	13.1	2.6	5.3	4.2	5.2	5.4	13.7	2.4
C4orf3	10.4	10.5	3.8	6.4	4.5	5.7	7.2	10.9	1.9
NUP188	10.1	14.0	3.1	5.2	4.7	3.8	4.5	13.8	2.1
ASB1	11.4	16.2	3.3	4.6	3.9	2.6	4.9	12.2	2.2
ACOX1	9.6	14.3	2.4	4.2	3.7	3.8	4.9	15.8	2.6
SLC41A3	12.3	15.1	3.3	5.1	5.4	3.1	4.6	10.3	2.1
DIABLO	10.1	13.7	2.2	4.7	3.3	3.1	6.1	15.0	3.0

C6orf226	13.2	9.7	3.0	4.0	5.1	2.0	6.6	13.8	3.9
CCDC167	9.6	10.8	2.8	7.3	6.4	5.1	4.6	12.7	2.1
PIP4K2A	11.2	12.7	3.5	5.9	5.0	6.1	4.3	10.7	1.9
UBE2G2	8.8	12.4	2.2	6.2	4.7	6.2	5.1	12.9	2.8
LHFPL2	12.8	19.5	3.2	4.2	4.2	3.0	3.0	10.0	1.3
PTP4A3	12.5	13.3	3.8	6.0	6.2	3.4	4.1	9.3	2.5
PKP4	9.4	11.9	2.6	7.7	6.5	5.0	3.9	11.5	2.5
DUSP5	13.3	14.0	4.8	4.5	3.9	8.9	3.2	7.2	1.5
MEAF6	9.9	14.8	3.4	4.1	4.3	5.5	4.6	11.4	2.9
RXRA	9.9	10.5	2.8	5.1	4.6	2.8	7.2	14.4	3.7
TP53I3	8.3	10.5	2.1	5.0	4.5	4.5	7.4	15.8	3.0
SIL1	11.0	13.8	3.1	5.3	4.9	3.4	5.4	12.3	1.9
PPP6R3	9.6	12.7	3.1	5.1	4.4	4.2	5.0	13.9	3.0
FAM162A	8.6	12.8	2.6	5.9	4.5	5.0	5.8	13.8	2.2
OLFM1	7.7	9.8	2.0	8.0	6.8	5.4	6.6	11.6	3.0
SOGA2	14.4	15.5	4.8	5.6	5.1	3.3	3.3	7.5	1.4
PCED1A	12.5	13.8	3.3	5.8	5.9	4.9	4.3	8.7	1.8
JAK1	9.0	13.2	2.5	4.4	3.6	7.6	4.8	13.3	2.4
DPH7	9.2	11.8	3.1	4.7	3.7	3.3	6.6	15.7	2.9
MAPKAPK3	12.1	14.9	3.8	6.2	5.8	3.6	3.7	9.2	1.6
LITAF	10.8	14.2	3.0	6.4	5.1	5.3	4.0	10.1	2.1
CAMK1	10.3	12.9	3.5	7.0	6.2	4.8	4.1	10.0	2.0
LSM3	8.1	9.6	2.7	6.7	4.3	7.1	6.1	14.5	1.9
TMEM14B	8.2	18.3	3.5	6.6	4.5	6.1	3.1	9.1	1.6
ARHGEF11	9.8	13.5	2.9	4.2	4.0	3.2	5.7	14.6	3.0
TNRC6A	10.0	11.7	2.8	4.9	4.0	4.1	5.2	15.4	2.7
MFSD3	9.5	13.9	2.6	6.1	5.4	4.8	5.1	11.5	1.9
CUL3	10.0	13.3	2.7	4.8	4.1	5.0	4.3	13.5	3.1
NSUN2	9.7	12.6	2.8	4.8	4.6	4.0	6.0	13.7	2.7
ARMC6	8.6	11.7	2.6	5.5	5.2	3.2	6.3	14.9	2.8
HNRNPDL	9.9	12.5	3.1	6.0	4.9	6.0	4.8	11.0	2.6
CDKN2D	9.9	12.5	4.2	6.1	6.0	4.8	5.2	9.3	2.7
MKRN1	9.7	13.0	2.5	4.9	4.1	4.8	5.6	13.8	2.4
SNX2	8.9	12.9	2.7	4.7	3.6	5.9	4.9	14.2	2.8
RER1	9.1	11.9	2.0	4.9	4.3	3.8	5.3	15.9	3.4
FAM222B	10.3	10.6	3.7	5.3	5.2	2.0	6.0	13.7	3.9
SERF2-C15ORF63	10.7	7.3	3.9	6.0	5.6	4.5	8.7	10.7	3.3
TMEM41B	10.1	14.6	3.6	4.1	3.8	2.4	5.8	13.2	3.1
PLK3	13.0	16.0	3.2	3.6	3.5	6.2	4.2	8.8	2.2
MIB2	9.8	11.7	2.6	3.9	3.8	3.1	7.8	13.9	4.0
LOC101929306	11.3	15.2	3.1	4.6	3.9	2.7	4.8	12.5	2.5
TSPAN9	11.7	12.6	3.3	8.8	7.2	4.0	3.7	7.9	1.4
PSEN1	9.8	14.0	2.7	4.3	3.8	3.5	5.0	14.6	2.9
MTHFD1L	9.2	14.3	2.2	6.2	5.4	3.8	4.1	13.6	1.8
44626	10.1	14.8	2.5	4.7	3.3	4.2	4.5	14.1	2.4
NGEF	12.1	15.0	4.4	6.4	5.7	3.3	3.4	8.1	2.2
NUDT19	11.0	12.9	4.2	4.6	5.2	2.3	5.1	11.4	3.8

PTRH2	8.4	11.9	2.0	5.8	4.3	2.8	5.8	16.8	2.6
C11orf84	10.3	12.3	3.9	5.5	5.9	3.4	5.6	10.9	2.7
ZMAT2	9.3	10.3	3.1	7.4	5.8	6.0	5.2	11.3	2.0
DNMT1	9.6	11.5	3.1	6.5	5.9	4.9	4.8	11.8	2.4
SEC24A	8.4	12.6	2.4	3.3	2.7	2.6	5.9	19.3	3.3
PPP2R5D	11.5	13.1	3.2	5.6	4.7	3.3	4.8	11.2	3.0
CDKN2AIPNL	6.7	11.4	2.4	5.6	5.5	7.5	6.4	12.6	2.3
TBC1D16	10.1	13.1	3.1	4.7	5.0	3.1	5.9	12.6	2.6
HTRA2	8.9	11.1	2.6	6.2	6.2	5.2	5.1	11.9	3.1
ABI1	9.8	14.0	2.7	4.5	3.8	6.2	4.4	12.7	2.4
STK11IP	11.3	13.4	2.8	3.3	2.7	3.3	7.1	13.4	3.1
CHCHD6	11.0	11.5	3.1	6.4	5.3	3.9	5.4	11.4	2.3
LOC101929360	9.3	15.6	3.2	3.2	2.5	5.9	4.4	13.9	2.1
CAMSAP1	9.1	10.9	3.2	5.8	5.2	4.1	5.4	13.4	3.1
DRG2	11.6	15.7	3.1	4.2	3.8	3.3	5.0	11.3	2.2
ISG15	7.6	13.1	3.3	6.0	5.3	4.5	5.3	11.8	3.3
ZNF655	10.1	16.1	2.3	5.0	3.5	6.1	3.1	11.9	2.0
ITGAV	9.1	14.9	2.3	5.4	3.7	4.2	4.3	13.8	2.4
PARD3	9.8	14.4	2.4	5.3	4.6	3.5	4.4	13.5	2.2
NUDT4	8.4	12.9	3.0	5.2	3.6	4.4	5.4	14.2	3.0
WWC2	9.7	13.6	2.7	8.1	6.8	5.5	3.3	8.7	1.7
NUP98	9.2	12.7	2.7	4.9	3.8	4.6	4.2	15.2	2.8
SNORA21	7.3	17.0	2.0	3.9	2.1	8.1	5.4	13.0	1.2
RARS	9.5	15.5	1.9	4.6	3.2	5.8	3.9	13.2	2.5
SSTR2	6.9	9.1	1.7	2.6	1.7	1.6	9.0	23.2	4.1
COX14	5.9	12.5	3.1	7.3	6.9	4.0	5.5	11.9	2.9
TEX2	7.4	10.2	2.0	6.6	5.2	4.8	5.6	14.7	3.4
THOC5	10.6	12.5	3.0	4.3	3.5	4.2	5.3	13.9	2.6
STAT5B	9.8	13.9	3.0	4.7	4.7	4.3	4.5	12.8	2.3
RABL5	8.0	13.3	2.5	5.9	4.4	4.6	5.1	14.0	2.0
BCL2L11	8.6	11.9	2.2	2.8	2.0	2.7	6.9	18.8	4.0
NAT9	9.1	12.6	2.4	4.5	3.7	3.1	7.5	14.7	2.3
PARP10	9.8	12.5	2.8	4.5	3.7	5.2	6.4	12.5	2.4
SMIM12	9.2	12.0	2.5	5.7	5.4	5.3	4.6	12.8	2.4
ZNF414	11.2	10.7	4.3	5.3	5.4	3.3	5.9	9.7	3.8
LOC286437	10.1	19.7	2.7	2.1	1.9	5.3	2.8	13.4	1.6
C16orf80	8.8	15.4	3.1	5.4	5.6	3.4	4.2	10.7	3.1
METTL9	8.2	12.4	3.0	4.8	4.8	5.9	5.3	12.4	2.9
E2F7	12.7	17.5	3.5	3.8	3.6	3.3	3.8	9.5	2.1
ZNF462	10.4	13.9	3.1	4.3	4.1	2.5	4.6	14.1	2.7
TMEM167A	7.6	14.5	2.1	3.4	3.0	5.7	5.9	15.4	2.1
CCDC104	8.1	12.0	2.4	5.7	4.0	2.9	5.5	15.5	3.4
SNAP29	10.3	11.5	3.7	5.3	4.9	4.1	5.7	10.8	3.3
EIF2A	9.4	15.1	1.9	3.6	3.6	4.0	4.6	14.3	3.0
CCNG1	9.6	16.5	1.8	5.6	4.0	5.9	3.3	11.0	1.8
ACSF3	10.1	15.1	3.0	4.9	5.3	3.1	4.2	11.3	2.7
RNASEH1	9.2	13.6	2.7	6.0	4.2	3.7	5.0	12.5	2.8

MAN2A1	10.4	16.8	2.9	5.3	3.7	5.5	3.3	9.8	1.9
DNAJC8	10.3	12.3	2.9	6.6	4.5	5.5	4.6	10.9	1.8
CTU2	8.5	11.8	2.7	3.5	3.3	2.9	6.9	16.4	3.5
TRMT2A	8.6	11.0	2.5	5.0	4.3	4.6	6.5	14.0	3.1
PDCL	8.4	9.4	2.8	6.4	5.0	5.1	6.1	13.5	2.8
FAM166A	9.8	11.9	3.2	7.2	7.1	2.2	4.8	11.2	2.1
C6orf89	9.4	13.0	2.8	5.8	4.9	4.2	4.3	12.7	2.4
RAB22A	8.7	14.6	2.7	4.7	4.0	5.6	3.9	12.4	2.8
NADK	8.8	11.8	2.6	6.1	5.7	4.9	5.2	11.8	2.6
OXA1L	10.2	12.2	3.1	5.3	5.0	4.6	5.0	11.6	2.4
FEN1	7.8	12.1	2.6	6.4	5.9	4.5	5.2	12.9	1.9
RARG	10.5	13.8	3.1	6.6	6.2	4.2	4.3	9.4	1.3
ZNF76	10.9	12.8	2.9	3.7	3.2	2.5	6.3	13.6	3.6
CLEC4C	10.2	15.4	2.7	3.2	2.5	6.9	4.3	12.4	1.8
ERCC2	12.3	13.5	3.4	6.6	7.2	5.5	3.1	6.1	1.6
PCM1	8.8	12.7	2.2	5.2	3.9	4.6	4.4	15.0	2.4
NDEL1	8.1	13.8	2.7	3.9	3.1	3.5	6.0	15.3	2.8
RGS4	14.1	20.4	3.1	5.2	4.3	2.6	1.9	6.1	1.6
KCTD10	9.0	10.1	3.0	6.8	5.9	5.6	5.0	11.3	2.8
ZMYM3	10.4	12.9	2.4	4.4	3.8	3.1	4.4	15.1	2.8
AATF	9.1	11.5	2.8	5.5	4.4	4.6	5.7	12.7	3.0
RARRES2	3.0	3.0	1.0	15.4	15.6	10.4	3.3	5.9	1.6
CYB5D2	10.5	14.1	2.5	5.4	4.5	4.1	4.6	11.3	2.2
OSBP	8.9	12.1	2.8	3.8	3.0	3.0	6.3	16.4	3.0
TXNL4A	9.3	12.8	3.1	5.2	4.9	4.6	5.2	11.7	2.4
SMARCD1	9.5	13.0	3.1	4.6	3.4	4.0	5.3	13.4	2.9
HERC4	11.1	16.6	2.7	3.9	2.8	5.6	3.5	11.3	1.6
LOC100506085	7.9	7.5	2.4	11.8	11.1	8.5	2.2	6.4	1.6
FBXL12	9.1	12.1	3.7	5.2	5.2	3.8	5.1	12.4	2.5
DARS	9.3	14.4	2.1	4.5	3.3	4.9	4.6	13.9	2.1
MAGOH	9.5	11.2	2.5	5.7	3.3	4.2	6.2	13.3	3.3
PLEKHA3	9.1	13.4	2.1	4.4	3.5	3.6	4.3	15.9	2.9
PDZD8	8.3	13.8	2.7	4.5	3.8	4.8	4.9	13.4	2.9
PIGY	8.1	14.6	2.5	6.6	3.3	3.1	3.0	16.0	1.7
RGS19	8.4	10.3	3.1	6.5	6.5	6.4	5.2	10.5	2.0
GPR176	11.6	14.7	3.2	8.5	7.7	4.3	2.5	5.3	1.1
ATAD1	11.0	14.9	2.0	4.7	3.0	2.8	4.1	13.9	2.5
PPP1R15B	9.0	12.5	2.3	3.9	3.2	4.2	5.5	14.8	3.6
LYRM1	7.9	10.2	2.2	5.5	5.3	2.6	6.4	15.6	3.2
SEC24B-AS1	9.1	15.3	3.0	3.7	2.4	4.8	4.5	14.0	2.2
PDCD2	8.7	12.4	3.3	5.9	4.8	3.6	5.2	11.2	3.7
PCGF3	9.3	11.9	3.2	4.1	3.7	3.7	4.9	15.1	3.0
MICALL2	11.8	13.3	3.3	4.9	4.4	2.5	5.8	10.0	2.9
DGCR6L	8.5	12.9	3.2	6.3	5.5	4.2	5.8	10.4	2.2
SNAR-C3	13.6	8.1	2.3	8.9	6.7	4.6	5.6	7.9	1.2
RTCB	8.5	12.6	2.1	4.6	4.0	3.3	6.0	15.3	2.4
LUZP6	8.4	15.7	1.5	5.4	3.7	7.0	3.4	11.5	2.2

ZNF593	9.6	13.2	3.9	5.9	4.7	3.9	4.7	10.1	2.9
ACSF2	12.3	14.4	3.5	6.0	5.4	3.5	3.7	8.2	1.8
RPTOR	10.6	12.6	3.2	4.3	4.6	2.7	5.0	13.1	2.7
STK17A	8.0	13.3	2.1	5.0	3.9	6.3	4.2	13.6	2.4
ZNF503	12.1	12.7	4.7	3.6	4.4	2.2	5.8	10.3	3.1
LOC101927447	10.4	16.4	2.4	3.0	2.0	7.0	3.6	12.5	1.6
MYO19	9.5	11.2	2.5	7.2	6.5	3.9	4.6	11.4	2.0
WDFY1	10.8	12.4	3.4	5.6	5.6	4.4	4.4	9.8	2.4
CMC2	5.9	11.2	2.1	6.6	4.2	5.8	5.6	14.5	2.9
RBM27	9.9	11.9	2.7	4.7	4.3	4.0	4.9	13.5	2.7
TCERG1	10.8	12.2	3.0	5.4	4.3	4.3	4.5	11.3	2.9
BFAR	10.1	15.0	2.4	5.1	4.1	3.6	3.6	12.0	2.6
AP1G1	9.0	14.0	2.4	4.9	3.9	3.5	3.9	14.3	2.6
AGPAT6	9.7	12.6	2.1	3.9	4.0	3.2	6.0	14.4	2.6
TACC3	9.5	10.4	2.7	10.6	9.7	6.6	2.6	5.5	1.1
ENDOV	10.0	13.3	3.3	3.7	3.2	2.8	5.7	13.6	3.0
FAHD1	9.4	11.6	2.3	6.2	5.0	3.6	5.6	12.8	2.0
ZBTB12	9.1	11.0	3.4	4.0	4.1	1.7	6.9	14.8	3.5
SMARCAL1	9.8	12.7	2.7	5.5	4.7	3.3	4.6	12.8	2.5
SLC19A1	10.2	12.1	3.4	5.8	6.0	4.1	4.4	10.2	2.3
IPO13	9.6	11.7	2.5	4.5	4.1	2.7	5.3	15.2	2.9
MPV17L2	9.0	9.9	2.7	6.2	5.8	4.5	5.3	12.2	2.9
GLRX3	8.7	16.1	2.2	5.0	4.2	4.2	4.1	12.0	2.0
MSRB2	9.4	14.9	3.0	4.8	4.3	3.6	4.4	11.6	2.5
HECTD3	8.8	11.9	2.6	5.2	4.4	4.7	5.6	12.9	2.4
DGCR2	9.7	12.9	3.2	5.0	4.7	4.6	4.9	10.8	2.6
SPATS2	9.7	14.1	2.6	3.7	3.2	5.0	4.2	13.6	2.3
GRB10	10.8	12.3	2.7	3.2	3.4	2.2	5.7	15.7	2.4
ZFAND2A	8.0	14.7	2.5	6.0	4.9	4.0	4.1	12.2	1.9
LOC100505817	6.4	6.7	1.8	15.1	11.8	11.1	1.4	3.4	0.6
FTSJ3	10.2	11.3	2.9	4.8	3.7	4.0	5.8	13.1	2.5
SRPX	4.4	6.6	1.2	2.7	2.1	1.8	9.4	25.7	4.5
SPG21	9.2	15.4	2.2	5.7	3.9	4.2	3.7	12.1	1.9
POLR2B	9.2	15.6	2.1	4.5	3.3	3.9	4.0	13.4	2.3
SMDT1	7.7	11.9	2.5	6.1	3.4	8.2	4.5	11.1	2.7
PTPRH	14.9	17.8	3.8	3.9	3.4	2.3	3.4	7.3	1.3
DDX21	9.7	12.0	2.6	4.5	3.8	5.6	4.6	12.8	2.5
STARD3NL	9.1	14.4	2.8	5.5	3.1	3.8	4.2	12.9	2.5
GON4L	10.3	13.3	2.5	3.7	3.5	3.4	4.4	14.6	2.5
RNF20	9.9	11.4	2.6	6.1	4.4	4.6	4.8	12.2	2.3
SMARCA5	9.5	12.7	2.4	4.6	3.3	5.0	4.3	13.6	2.8
EIF3J	9.6	10.1	3.7	5.7	4.4	4.6	5.4	11.2	3.4
TICAM1	10.5	11.7	3.5	5.4	5.9	3.4	4.4	10.5	2.9
LRPPRC	9.2	13.9	2.3	4.3	3.8	4.2	4.5	13.2	2.8
TMEFF2	11.1	17.3	2.9	2.4	2.0	2.0	5.1	13.3	2.2
DOHH	8.6	12.8	3.5	5.4	4.8	3.6	5.2	11.1	3.1
DHX38	9.6	10.7	2.6	5.4	4.7	4.8	5.6	12.2	2.3

HIP1	7.0	8.5	2.1	8.3	7.6	5.4	4.3	12.7	2.2
TSR2	8.0	10.7	2.8	5.9	4.7	5.5	6.3	12.2	2.0
SIRT7	12.3	13.4	3.2	4.4	4.8	4.7	4.7	8.5	2.0
MED1	9.3	12.9	2.5	4.7	4.2	3.6	4.1	14.2	2.4
LOC101928936	10.4	17.3	3.1	2.4	2.2	3.3	3.3	14.3	1.8
NCKIPSD	12.6	13.4	3.7	4.8	4.7	2.2	4.2	9.8	2.5
MUS81	9.4	11.2	2.9	5.5	5.3	3.8	5.6	12.1	2.3
SRSF10	9.5	13.8	2.3	4.8	4.9	6.0	4.4	10.0	2.3
IVD	9.4	13.0	2.8	4.1	3.6	3.4	5.7	13.7	2.4
SNAPC4	11.3	13.6	3.5	3.4	4.1	2.5	6.0	11.4	2.1
HIST1H2BF	9.0	10.2	3.9	6.7	6.1	4.4	5.4	9.1	3.0
CACNB3	9.7	12.6	2.8	6.1	5.8	3.4	5.1	10.2	2.1
NARFL	9.9	11.9	3.0	5.3	4.3	4.0	5.4	11.6	2.4
DDIT3	11.2	15.2	3.5	3.3	2.7	3.0	4.5	11.8	2.6
ARNT	10.5	13.1	2.8	3.7	3.4	3.3	4.8	13.7	2.6
CLPB	8.3	11.2	2.5	4.6	4.2	3.9	5.9	14.6	2.6
GTPBP4	10.5	11.4	2.9	5.0	4.0	3.5	5.2	12.6	2.7
TYSND1	9.5	14.6	3.1	4.4	4.9	4.3	4.5	10.4	2.1
ATF6	8.7	13.2	2.4	5.0	4.5	4.2	4.0	13.2	2.6
RANGRF	8.9	13.8	3.0	5.9	4.8	3.4	4.9	11.3	1.9
SMG7	8.2	9.9	2.3	5.7	5.4	4.6	4.5	14.6	2.6
ABCC1	11.4	14.6	3.6	4.5	4.1	3.7	3.8	10.1	1.8
TMEM208	6.7	9.4	2.3	4.5	3.4	4.5	7.5	17.6	1.8
ENO2	11.0	13.8	3.1	5.7	5.0	7.1	3.2	7.2	1.6
DDX3Y	9.5	13.3	2.0	4.4	3.2	3.4	5.1	14.4	2.4
GIT2	11.0	13.6	3.1	4.2	4.2	3.6	4.2	11.3	2.6
PIKFYVE	9.1	14.5	1.5	2.8	1.9	3.1	4.7	17.1	3.0
FAM20B	10.0	14.3	2.6	5.6	4.2	4.3	4.0	10.7	1.8
MCM3AP	9.5	12.3	2.6	4.1	3.6	4.1	5.4	13.7	2.4
FLJ31356	9.5	15.2	2.7	3.8	2.3	6.1	4.0	12.2	2.0
TM7SF3	8.3	13.4	2.1	6.0	4.4	6.5	3.8	11.3	1.9
MON1B	9.0	11.5	2.8	4.3	4.1	4.2	5.7	13.1	2.8
CYB561D2	6.3	9.7	1.9	4.6	3.2	2.4	7.7	18.9	3.0
RYK	9.8	14.4	2.7	4.9	3.9	3.6	4.1	11.6	2.6
XPNPEP1	8.2	11.4	2.2	3.8	3.4	3.6	5.8	15.7	3.2
RAMP2-AS1	9.9	14.7	2.7	3.6	2.7	6.0	3.7	12.0	2.1
CRLF1	11.3	14.3	4.7	5.9	6.9	3.2	3.4	6.3	1.6
MAP4	9.9	14.6	3.1	4.1	3.9	3.3	4.5	11.7	2.4
ATPAF1	9.7	13.0	2.4	4.8	4.8	3.8	4.7	11.7	2.5
ITCH	9.9	12.7	2.4	4.4	3.5	4.8	4.3	12.9	2.5
DHX8	9.1	12.8	2.5	4.5	3.9	4.0	5.0	13.4	2.4
LOC100506022	9.8	15.2	2.5	3.6	2.3	6.5	3.9	12.0	1.6
LOC100289283	10.6	16.6	1.8	3.8	2.3	1.9	5.0	13.8	1.6
TCEAL4	11.4	9.4	4.3	5.5	5.3	4.3	5.0	9.9	2.4
PPP1R13L	12.5	13.4	4.9	7.2	7.2	3.7	2.6	4.5	1.4
UNC5C	9.1	12.6	2.7	2.8	2.6	1.9	5.4	16.9	3.4
LOC100131564	9.7	15.7	2.8	3.1	2.6	5.8	3.8	12.0	1.9

ARL16	9.0	9.8	3.3	6.3	5.7	3.1	6.5	11.0	2.6
SLC4A3	11.4	14.0	2.9	5.3	4.8	2.4	4.7	9.6	2.2
EHBP1	8.3	14.4	2.2	4.1	2.6	2.8	4.9	15.6	2.5
NCOA5	8.5	10.4	2.5	4.6	3.6	3.8	6.2	14.5	3.3
PMF1	6.0	10.7	3.1	5.8	5.4	4.5	5.5	14.8	1.4
TMEM9B	8.7	14.0	3.0	5.9	4.4	3.6	4.5	10.7	2.6
PRKAR2A	10.4	11.3	2.6	4.8	3.6	3.7	5.1	13.2	2.6
WNK1	8.5	11.3	2.7	5.0	5.0	5.0	4.2	13.0	2.6
CXCR4	2.3	3.2	0.5	0.3	0.2	11.4	9.5	25.7	4.1
TMEM165	8.3	13.2	2.6	5.4	4.7	4.1	4.2	12.0	2.7
MEPCE	8.5	9.6	2.5	4.8	5.1	5.5	5.4	12.7	3.1
GGA1	10.7	10.0	3.0	3.5	3.8	2.9	5.8	14.2	3.2
LOC100507131	9.6	15.3	2.8	3.2	2.5	6.0	3.7	12.1	1.9
NOP56	8.7	10.9	2.2	5.4	4.3	5.0	5.1	13.0	2.6
VBP1	7.1	13.6	1.9	5.2	3.9	7.0	3.7	12.9	1.9
PQLC1	9.7	13.2	2.9	3.6	4.7	3.4	5.2	11.9	2.5
PHF12	9.2	10.2	2.6	4.2	4.2	3.9	5.5	14.3	3.1
TRIOBP	8.4	11.1	2.3	5.6	5.5	5.8	5.0	11.1	2.2
LTBP4	11.5	14.0	3.7	4.3	3.6	2.7	4.6	10.2	2.4
PAQR4	6.6	10.8	3.1	7.2	6.2	5.2	5.1	10.7	2.0
GCSH	10.1	10.8	3.6	6.2	5.2	3.1	5.8	9.7	2.7
KLF5	12.0	15.3	3.8	7.6	7.5	3.7	1.4	4.6	1.2
GSTM4	10.7	12.3	3.2	8.0	7.4	3.1	3.2	7.4	1.6
SMAD5	8.5	11.8	2.0	3.5	3.4	3.7	5.4	15.7	2.9
MAX	8.3	11.0	2.4	3.6	4.1	4.6	6.0	14.0	3.1
GSE1	8.1	8.7	2.6	3.8	4.2	3.2	6.8	16.1	3.4
RRM2B	8.3	13.7	2.0	4.2	2.9	4.7	5.9	13.3	2.0
SRPK2	10.1	11.5	2.5	5.0	3.5	4.2	4.5	13.0	2.6
SLC35E2	9.4	11.5	2.4	4.1	2.9	3.4	6.1	14.1	2.8
GAS8	10.3	10.7	3.4	5.4	4.9	3.1	5.4	10.8	2.8
ZHX3	9.3	12.0	2.9	5.0	5.1	2.6	4.5	12.4	3.0
SMC1A	9.4	10.0	3.0	4.8	4.2	4.6	5.4	12.9	2.6
AK2	8.8	13.4	2.3	4.7	4.0	4.9	4.4	11.8	2.7
DFFB	9.8	14.6	2.7	3.4	2.6	5.5	4.0	12.2	2.1
TNFSF12	10.8	12.6	3.3	5.6	5.4	4.5	3.9	8.1	2.5
HIAT1	8.9	15.4	2.2	4.9	3.8	4.4	3.7	11.4	2.1
SLC25A28	9.1	13.0	2.2	3.9	4.1	4.4	5.1	12.0	2.9
TBC1D25	9.5	12.4	2.8	4.9	4.7	2.9	4.7	12.3	2.8
EVA1A	6.2	10.7	2.8	2.6	3.0	1.8	8.1	18.0	3.5
ARHGEF10	8.5	11.0	2.8	5.0	5.1	2.9	5.0	13.7	2.7
BREA2	9.2	14.1	2.6	3.4	2.2	5.4	4.5	13.3	2.1
MAGEA3	8.9	12.9	3.0	3.9	3.6	2.4	5.9	13.7	2.4
ROGDI	8.9	10.8	3.1	5.5	6.5	4.2	5.2	10.0	2.6
TWIST2	8.0	7.8	3.6	5.1	7.8	2.6	6.1	12.6	3.1
CERS4	12.0	13.3	3.4	4.4	4.1	2.3	4.6	10.2	2.4
WSB1	8.7	12.0	2.0	4.0	2.8	6.3	4.4	13.9	2.6
DPM1	9.2	11.8	1.9	4.9	3.6	4.8	5.3	12.4	2.8

JMJD6	7.6	10.1	2.3	3.7	4.1	3.8	6.2	15.3	3.6
ILVBL	8.6	12.7	2.4	4.9	4.1	3.3	5.6	13.1	2.0
TNKS	10.4	12.5	2.8	3.4	3.0	3.0	4.9	13.9	2.7
DOLK	9.3	12.7	2.5	5.5	4.7	3.9	4.6	11.3	2.2
FLJ44255	10.2	15.3	2.6	3.4	2.4	5.1	3.4	12.5	1.7
CKS2	6.2	10.2	2.3	8.8	6.2	7.2	4.3	9.5	1.7
CAD	10.2	12.7	2.7	5.0	4.6	3.9	4.2	11.2	2.1
IFRD1	9.6	13.5	2.4	5.0	3.0	4.2	4.5	12.2	2.1
LRIG1	5.2	7.2	1.5	2.3	2.0	2.5	8.5	23.0	4.3
TM2D2	8.8	11.6	2.7	5.7	4.1	5.0	5.5	11.2	2.0
SKIV2L	9.3	12.1	2.4	4.6	3.9	3.4	5.6	12.7	2.4
CCDC80	14.6	17.6	5.6	3.3	3.0	2.1	2.8	6.2	1.4
ORMDL1	9.5	13.5	1.8	4.7	3.7	4.6	3.8	13.6	1.2
LOC101929450	9.3	13.9	2.7	3.2	2.7	6.3	4.0	12.6	1.9
ANKH	6.0	7.7	1.8	2.0	2.1	2.4	8.4	22.3	3.7
LOC101927034	10.1	17.1	2.4	2.3	2.0	5.6	2.4	12.9	1.7
ANAPC15	9.7	8.7	3.3	4.5	4.4	2.8	7.5	13.5	2.0
TRRAP	9.2	12.2	2.9	3.9	3.4	3.8	4.4	14.1	2.5
RNF115	8.8	12.4	2.4	5.5	4.9	5.0	3.5	11.7	2.4
BAIAP2-AS1	9.3	12.2	2.9	4.9	4.1	4.0	4.5	12.2	2.3
TXNDC5	8.6	11.1	4.0	4.9	3.8	2.0	6.5	11.5	3.9
LOC100506302	8.9	15.2	2.4	3.4	2.4	6.2	3.6	12.4	1.9
LRRC42	8.8	12.4	2.3	6.6	5.4	5.1	3.7	10.7	1.3
ABCB8	9.6	11.4	2.6	4.2	3.6	3.0	6.1	13.3	2.6
ATG3	8.8	11.3	1.9	4.5	4.4	3.9	4.5	14.1	2.9
MCMBP	9.0	13.1	2.1	5.3	4.1	4.4	4.2	11.9	2.2
TOR3A	9.5	12.7	3.5	5.1	3.9	3.7	4.8	10.8	2.3
TBC1D5	8.6	12.0	2.1	4.8	4.1	4.6	4.6	13.1	2.5
ERLIN2	9.1	13.1	3.0	6.3	4.2	4.7	3.8	10.0	2.0
CASP2	9.0	12.6	2.2	4.7	4.4	5.2	4.1	11.8	2.3
SEC63	8.0	10.3	2.1	3.7	3.2	3.9	5.4	16.6	3.0
POLG	11.1	14.2	3.3	3.2	2.6	3.6	5.6	10.4	2.4
ITGB4	13.6	14.4	4.5	5.3	5.2	2.6	3.1	6.0	1.7
LOC101927096	14.8	13.0	5.8	5.6	3.8	1.1	4.3	5.9	1.8
MAN2B2	9.8	11.4	3.3	7.0	6.4	4.6	3.7	8.1	1.9
HSD11B1L	7.6	11.8	3.9	5.7	5.4	2.5	5.3	10.9	3.3
VASH1	9.6	11.2	2.8	2.9	2.1	2.0	6.6	15.3	3.6
ZNF316	9.0	11.1	3.0	5.1	5.1	3.6	5.8	10.9	2.6
LOC101928805	9.0	11.7	2.9	3.4	3.0	2.0	5.8	15.0	3.3
DLST	8.2	13.0	2.7	4.5	3.7	6.1	4.3	11.5	2.0
APBA3	8.0	9.5	2.3	3.5	3.7	3.4	6.9	14.8	3.9
RPRD2	9.9	11.6	2.8	4.5	4.2	3.7	3.8	12.8	2.8
RBMS2	11.1	13.4	3.2	5.7	4.7	4.7	3.1	8.2	1.9
HSPG2	11.1	12.8	4.0	6.8	6.7	3.6	2.6	7.1	1.3
PPP5C	8.8	11.1	2.8	5.2	4.6	3.1	5.0	12.5	2.7
SLC25A51	9.1	13.4	2.3	4.5	3.3	5.4	4.0	12.0	1.9
NR2F1	4.0	6.0	1.1	8.4	8.3	3.5	5.9	15.7	3.0

KCTD9	8.4	13.1	2.2	5.7	3.9	6.4	3.6	11.0	1.8
TIMM10B	9.2	13.5	2.6	3.5	2.9	3.4	4.7	13.3	2.8
LOC101928669	9.6	12.9	2.5	3.4	3.3	3.0	4.5	14.3	2.5
FBXO11	10.3	11.4	2.9	4.4	4.0	4.4	4.4	11.1	2.9
TAGLN	12.9	17.5	4.3	7.6	5.1	5.5	1.2	1.5	0.4
GTPBP3	10.4	12.6	3.4	4.4	4.4	3.0	5.6	9.6	2.6
DCAF15	9.2	10.4	2.5	5.2	4.9	4.6	5.4	10.8	2.8
TOM1L2	8.6	12.2	2.5	5.2	4.1	3.3	4.6	13.3	2.1
PYGL	7.6	10.4	2.1	5.0	4.0	3.2	5.9	14.5	3.1
LOC101928055	13.4	13.4	3.6	1.9	1.6	1.5	4.6	13.7	2.3
NMNAT1	9.4	14.7	2.3	3.3	2.7	5.6	3.6	12.3	1.8
TAF1C	9.6	11.8	2.9	4.6	4.6	3.5	5.4	10.3	3.1
TUSC2	8.2	8.8	2.3	5.0	4.7	5.6	6.1	11.5	3.5
KIAA0895L	9.7	12.0	2.5	3.4	2.8	1.6	7.2	13.0	3.6
SP3	8.0	11.1	2.1	5.1	4.1	5.6	4.3	12.7	2.8
ATP6V1G2-DDX39B	10.4	12.3	2.5	5.7	0.4	5.0	5.8	10.8	2.9
DHX16	9.7	11.0	2.8	4.9	3.9	3.3	5.5	12.3	2.4
USB1	9.4	10.4	2.7	5.7	5.9	3.8	5.3	10.6	2.0
LOC375295	10.3	13.3	3.4	4.1	3.1	4.1	3.7	11.7	1.9
TOR1A	9.0	11.8	2.6	5.3	5.0	4.4	4.2	11.2	2.2
ZEB1	9.3	12.6	2.5	3.3	2.8	2.4	4.8	15.2	2.8
HDAC6	9.6	10.7	2.4	3.9	3.8	2.8	6.0	13.8	2.9
PDLIM5	9.7	15.8	3.5	4.5	3.6	6.0	3.0	8.5	1.1
UNQ9370	10.1	15.3	2.5	3.2	2.2	5.0	3.7	11.8	1.8
CAP2	9.7	14.3	2.9	5.7	5.4	4.1	3.1	8.7	1.9
WDR74	8.5	10.5	2.4	4.7	4.9	5.1	5.8	10.7	3.2
KMT2B	9.9	11.2	3.2	4.1	3.9	4.0	4.9	11.7	2.7
MRPL3	8.6	12.6	2.0	4.4	4.0	4.3	4.7	12.7	2.4
ASL	9.3	13.0	2.3	5.9	5.9	4.8	3.6	9.3	1.6
DESI2	7.0	11.8	1.9	5.3	3.9	5.5	4.3	13.9	2.2
SCP2	6.8	20.5	1.7	4.9	3.0	4.5	3.4	9.7	1.2
KDM6B	8.9	9.3	2.9	2.8	2.5	5.2	6.2	14.4	3.6
ANKRD13B	10.5	11.7	3.3	5.0	4.9	2.6	5.0	9.9	2.8
LOC101928144	8.7	13.6	2.5	3.2	2.3	6.7	4.0	12.9	1.8
BICD2	9.6	9.7	3.6	5.6	5.6	3.8	4.8	10.5	2.4
PGAM2	8.8	11.4	2.8	2.4	2.1	2.1	6.0	17.2	2.7
PLOD2	6.2	9.1	1.4	8.2	5.7	8.0	3.7	11.3	2.0
METTL2A	9.3	12.8	2.5	5.0	3.8	3.9	4.4	11.3	2.6
UBXN7	10.1	14.8	2.6	3.9	3.6	4.0	3.4	11.0	2.2
SCARNA5	7.7	16.0	1.3	2.4	2.6	8.4	4.5	12.3	0.5
MFHAS1	8.3	11.3	2.7	3.4	3.2	3.8	5.5	14.8	2.5
SLC31A1	7.2	10.4	1.7	3.0	2.4	3.1	6.0	19.3	2.4
SOAT1	9.6	14.4	2.5	4.9	3.5	4.6	3.8	10.1	1.9
THBS3	12.6	16.7	3.0	5.0	3.6	3.2	2.7	7.4	1.3
PPP1R16A	8.4	10.3	3.3	4.2	4.6	3.1	7.0	11.5	3.1
BCAR3	7.0	9.3	2.3	7.4	7.7	4.8	3.9	10.8	2.2
C8orf82	8.8	10.6	2.7	5.3	4.0	2.7	6.7	11.7	3.0

ALAD	7.3	10.7	2.3	4.7	4.3	3.4	6.4	13.7	2.6
RAB21	10.6	10.5	2.7	4.7	3.3	4.0	5.1	11.8	2.7
AIMP1	9.9	15.7	2.8	4.6	3.2	3.3	3.8	10.2	2.0
DHRS7	7.5	11.6	2.4	4.6	3.3	3.8	6.3	13.2	2.5
TTL	9.1	12.5	3.2	5.6	4.5	4.1	4.2	10.5	1.6
ARHGEF40	8.7	11.5	2.6	4.0	3.3	2.3	6.1	13.9	3.0
LOC100134868	9.1	13.9	2.5	4.1	3.0	5.9	3.7	11.3	1.8
NXF1	7.1	11.4	2.0	4.1	2.7	6.7	5.3	13.3	2.7
OSBPL8	9.3	15.1	2.2	4.3	3.3	4.1	3.3	11.7	2.0
MAN1B1-AS1	9.1	12.8	2.5	3.4	2.8	4.4	4.4	13.4	2.5
ZNF609	7.0	7.9	2.3	3.1	3.0	3.3	6.9	17.9	3.9
WBSCR16	7.7	12.5	2.5	6.4	4.3	5.7	4.2	9.6	2.3
ESRRA	8.0	12.8	2.7	3.9	3.7	4.0	5.6	12.3	2.3
VANGL2	9.5	11.2	2.7	5.0	4.5	3.2	4.9	11.8	2.4
AFAP1	7.7	10.6	2.3	7.1	6.4	4.5	4.1	10.7	1.6
CDH11	5.4	6.8	1.4	5.0	3.9	3.0	6.3	19.7	3.5
RSAD1	8.0	10.7	1.9	4.1	3.1	3.9	6.4	13.7	3.3
KAT8	8.8	12.3	2.4	3.7	3.0	2.5	6.2	13.5	2.7
FAM83H-AS1	9.4	14.9	2.4	3.1	2.1	6.5	3.7	11.5	1.6
NTPCR	7.1	13.9	1.8	5.7	4.7	4.1	4.3	11.9	1.7
PHAX	8.6	9.9	2.3	5.7	4.5	4.8	5.4	11.1	2.8
LOC729867	10.2	13.5	4.4	7.0	5.8	3.5	3.9	5.5	1.4
MTA1	9.1	9.3	3.0	5.5	5.8	4.7	5.3	9.7	2.6
LOC653375	11.5	11.3	2.5	3.0	5.9	4.5	4.1	11.4	1.0
AKAP10	8.6	12.3	2.7	4.3	3.9	4.3	4.3	12.6	2.2
PDHX	9.1	11.8	2.1	3.8	3.5	4.0	4.6	13.0	3.2
KIAA1430	9.2	12.2	2.0	4.8	4.3	4.4	3.7	12.2	2.3
PPFIA1	9.0	11.8	2.6	4.8	4.0	3.4	4.7	12.1	2.5
RRP7A	8.9	10.8	2.7	6.0	5.3	4.4	4.4	10.8	1.8
ANKLE1	9.2	13.9	3.0	3.7	2.8	4.5	4.0	11.6	2.3
MLLT10	10.0	13.1	1.7	3.9	3.6	4.1	3.8	12.9	1.7
DGKD	9.7	13.1	2.6	3.2	2.3	4.8	4.8	11.9	2.5
PRKAG1	8.5	13.2	2.5	4.5	3.9	3.4	4.2	12.7	2.0
DDX46	8.6	11.2	2.2	2.9	2.3	3.0	6.2	15.4	3.0
FMNL2	8.3	10.2	2.3	3.4	3.2	2.9	5.6	16.2	2.8
PDGFA	13.3	16.0	3.6	3.8	3.2	2.4	3.5	7.4	1.6
EI24	8.2	14.2	2.0	4.2	3.9	3.1	4.4	12.9	1.9
ZNF771	8.7	9.3	2.9	5.3	5.8	2.7	6.3	10.6	3.3
WNT5A	11.4	12.4	3.0	2.0	2.3	2.3	6.3	12.4	2.7
LIN37	8.2	11.3	2.7	3.9	4.1	2.5	6.1	12.9	3.1
HDAC1	9.0	11.9	2.0	5.2	4.2	6.4	4.0	10.2	1.9
SWAP70	9.3	9.9	2.8	5.9	4.7	6.0	4.3	9.7	2.1
FMR1	9.5	13.2	2.4	4.4	3.4	4.2	3.9	11.9	2.0
PON2	7.9	11.7	2.0	6.9	4.9	5.4	3.7	9.8	2.4
ENY2	7.8	9.3	3.0	4.6	3.3	4.4	5.8	12.7	3.8
UPF3A	8.7	9.1	3.4	5.9	5.0	3.8	5.2	10.8	2.7
CPSF4	9.6	12.1	2.9	4.3	4.4	3.5	4.6	10.6	2.6

LZTR1	8.8	11.8	2.9	4.9	4.9	3.7	5.0	10.3	2.4
RSL24D1	8.8	14.6	1.9	3.6	2.8	4.5	3.9	11.7	2.9
SLTM	8.8	8.6	2.9	5.5	4.9	4.0	5.3	11.7	2.9
COMMD5	7.8	12.1	2.5	4.4	3.9	4.3	5.3	12.2	2.1
GRSF1	8.6	11.3	2.9	5.2	4.7	4.7	5.1	9.6	2.4
RELL2	11.5	12.9	3.0	4.0	3.6	2.6	5.4	8.9	2.7
ADCY6	9.4	12.4	2.6	4.1	3.2	2.2	5.1	13.0	2.6
SH3GLB1	9.2	12.4	2.3	3.6	2.9	4.8	4.2	12.8	2.4
CAMSAP2	8.8	12.6	2.1	3.4	3.1	3.3	4.0	15.0	2.4
OBFC1	9.6	13.0	2.4	2.9	2.6	1.8	4.8	14.5	3.1
ACAA1	9.0	10.8	2.6	5.1	4.7	3.9	5.1	11.5	1.9
BROX	8.2	12.1	1.7	5.5	4.2	5.3	3.4	11.9	2.1
GAB2	9.7	11.5	2.9	6.9	6.1	3.3	3.3	8.2	2.4
ICMT	7.7	10.0	2.6	7.6	5.7	3.7	4.3	10.6	2.3
GNG10	7.1	15.8	3.3	4.4	2.9	2.8	4.4	12.4	1.4
LOC100996598	9.7	14.4	2.5	2.4	2.1	6.1	3.5	11.7	2.0
CDK11A	9.5	8.4	3.0	4.8	4.7	3.9	6.5	10.3	3.3
PTPLAD1	8.4	12.9	2.4	6.1	4.8	4.7	3.4	10.3	1.6
VPS37A	9.2	12.4	2.2	4.6	4.2	3.9	3.7	11.4	2.8
IER3IP1	8.8	9.8	2.9	4.8	5.4	3.3	4.9	10.9	3.6
NRBP2	10.7	12.7	2.9	5.0	4.0	2.8	5.1	9.1	2.2
OIP5-AS1	8.7	12.7	2.3	4.2	3.1	5.4	4.0	12.1	1.9
UGDH	6.6	11.3	1.6	4.1	3.9	4.9	4.7	14.7	2.5
YTHDF2	9.0	12.3	2.3	5.5	4.0	4.5	3.8	10.7	2.3
CENPT	9.3	12.4	2.9	4.2	4.0	2.8	5.1	10.6	3.0
ARHGEF26-AS1	9.0	17.5	2.1	2.1	1.7	5.2	2.3	12.7	1.6
FAM120AOS	7.3	11.8	2.2	6.0	4.5	2.4	5.2	12.2	2.5
CLASP1	9.6	13.5	2.8	3.9	2.8	3.1	4.2	12.0	2.3
HDAC10	11.4	12.9	3.4	3.6	3.3	2.2	5.4	9.9	2.2
BSN-AS2	9.5	15.1	2.4	2.8	2.1	5.3	3.3	11.9	1.8
PAPD4	10.2	14.3	2.4	3.9	3.2	3.7	4.0	10.3	2.3
HMHA1	7.1	11.0	1.9	2.9	2.2	15.2	3.3	9.4	1.3
ZNF205-AS1	9.0	14.1	2.8	3.7	2.2	4.8	3.7	12.0	1.9
TSR1	7.8	11.1	2.1	4.8	4.0	4.3	4.6	13.3	2.1
RHOT1	9.9	15.4	2.4	4.2	3.0	3.1	3.2	10.7	2.3
SURF2	8.0	9.5	2.8	5.9	6.5	4.0	5.1	9.6	2.6
TOP1	8.8	11.1	2.2	3.9	3.3	4.2	4.9	13.1	2.8
KIF5C	9.4	10.5	3.0	5.1	4.2	3.0	4.7	12.2	2.1
SCMH1	9.2	11.2	2.9	5.7	4.5	2.4	4.6	10.3	3.3
EXT1	10.3	12.8	2.9	5.3	4.1	4.0	3.7	9.2	1.8
SMCR7	10.2	13.9	2.7	4.9	4.2	4.3	4.2	7.6	2.1
CHMP6	10.1	9.6	2.8	4.8	5.0	2.9	5.5	11.0	2.3
AMBRA1	9.6	13.3	2.6	3.6	3.2	4.1	4.1	11.3	2.3
OXLD1	7.8	12.5	2.7	4.1	4.0	3.6	5.7	11.3	2.4
ARHGAP35	8.2	11.0	2.4	4.4	3.9	4.1	4.4	13.4	2.3
LOC101926996	9.1	14.7	2.4	3.2	2.4	5.1	3.7	11.7	1.9
GTF3C2	7.7	11.2	2.3	4.3	3.3	3.6	5.4	13.6	2.8

TANC1	9.0	11.5	2.6	3.7	3.2	2.5	5.1	14.0	2.5
AGAP1	7.8	8.5	2.5	3.9	3.3	2.3	4.9	17.2	3.6
ISCA1	6.6	9.2	2.1	3.8	3.6	6.4	6.4	14.2	1.5
VAMP7	8.6	12.8	1.8	5.2	3.6	4.2	3.8	11.7	2.2
VAMP7	8.6	12.8	1.8	5.2	3.6	4.2	3.8	11.7	2.2
PPP1R2	9.6	12.3	2.6	5.0	3.2	2.9	4.2	11.3	2.9
C7orf49	8.9	10.5	2.4	4.5	5.0	3.5	5.1	11.7	2.5
PPIC	7.7	11.9	2.1	4.1	3.9	3.5	4.9	13.7	1.9
UBE2C	6.5	9.7	2.5	10.7	10.6	5.6	1.9	5.2	1.0
CC2D1B	8.4	10.6	2.3	6.3	5.1	4.9	4.2	10.0	2.1
MIA3	8.9	10.8	2.4	2.9	3.0	2.2	5.2	14.9	3.5
ADAP1	11.1	14.7	3.3	4.2	3.8	5.0	3.4	7.4	1.0
C8orf44	8.8	14.1	2.7	3.7	2.6	6.3	3.4	10.6	1.6
NCOA6	9.4	10.9	2.7	4.1	4.0	2.8	4.5	12.8	2.5
MKRN2	8.1	11.2	2.0	4.5	3.6	3.5	4.9	13.5	2.6
MAPK7	8.1	9.2	2.9	4.2	4.9	2.8	5.7	13.0	3.0
ZNF692	10.8	13.3	2.3	3.6	3.1	3.1	4.8	10.3	2.4
BPTF	9.8	10.8	2.9	4.1	3.4	3.6	4.4	12.4	2.4
HSD17B4	8.5	12.0	1.7	5.7	4.2	4.7	3.7	11.3	1.8
BRWD1	16.0	12.2	1.7	1.6	1.1	3.2	5.4	11.6	0.9
TACO1	9.9	11.5	3.3	5.1	5.0	4.5	3.4	9.2	1.8
AK3	8.0	12.0	2.6	5.4	4.1	4.7	4.0	11.0	2.1
C6orf141	13.8	14.7	4.1	1.9	1.1	1.1	5.1	9.2	2.8
PARN	8.7	11.9	2.1	4.1	3.1	3.5	4.1	13.8	2.3
ERBB2IP	8.7	12.1	1.8	5.9	4.5	6.1	2.5	9.9	2.1
E4F1	9.2	10.8	2.8	3.3	3.6	3.8	6.3	11.0	2.8
LOC100506385	8.5	13.8	2.0	3.2	2.1	5.1	4.1	13.0	1.8
TXNDC15	9.0	12.1	2.7	4.0	3.6	2.9	5.0	12.0	2.4
SNX9	8.9	13.9	2.2	4.8	3.3	3.6	3.8	10.9	2.1
MED22	10.4	10.0	2.3	4.1	4.6	2.7	5.4	11.7	2.3
KLF11	9.6	11.4	2.8	4.6	5.0	4.2	3.8	9.8	2.4
BCAS4	11.2	12.1	3.7	4.2	3.9	2.8	5.4	8.2	2.0
LOC101927550	8.4	15.2	2.4	3.4	2.5	4.5	3.6	12.0	1.6
RPS6KA2	10.2	13.6	3.4	3.9	3.3	2.3	4.0	10.6	2.2
TGFB2	14.1	17.1	3.6	4.6	4.4	4.0	1.2	3.7	0.9
FAM213B	8.5	10.7	2.7	5.9	5.6	3.7	5.5	9.0	2.0
COG4	8.0	11.8	2.3	5.1	4.2	3.9	4.4	11.9	1.9
DMTN	9.5	11.0	3.3	6.0	5.1	3.6	3.7	9.1	2.2
GTF3C6	8.1	12.3	1.8	4.4	3.6	4.1	3.7	13.5	1.9
MTX2	9.0	11.3	2.6	4.8	4.0	4.1	4.2	11.1	2.4
NR1D2	9.0	12.0	1.8	3.9	3.1	4.4	4.0	13.3	2.0
SRC	9.4	10.7	3.4	4.3	5.2	3.0	4.6	10.3	2.6
GNB5	9.6	12.2	3.1	4.5	3.4	3.0	4.4	10.9	2.3
C15orf61	10.9	10.7	3.8	4.7	4.7	1.6	4.9	9.4	2.6
SOX11	10.4	11.6	4.3	4.6	4.5	2.7	4.7	8.7	1.9
SRP19	7.7	8.5	1.8	5.0	4.0	5.2	4.7	14.1	2.3
RTF1	9.6	9.8	3.2	5.2	4.3	3.4	4.4	10.6	3.0

FJX1	9.0	13.3	2.8	4.0	4.4	3.2	4.6	10.1	2.0
UIMC1	8.4	12.6	1.5	4.7	4.0	3.3	4.2	13.1	1.6
WBP5	9.4	8.7	1.8	6.5	3.7	4.9	5.1	10.5	2.8
LOC101927415	9.0	14.0	2.6	3.2	2.2	5.6	3.4	11.7	1.7
AP3M1	9.2	13.0	2.1	4.7	3.6	3.8	3.5	11.1	2.3
HOXD10	11.2	15.3	3.0	4.0	2.6	2.1	3.1	9.8	2.2
SLC4A7	10.8	14.9	3.6	3.5	2.8	3.6	3.3	8.8	2.0
PRPF4B	9.7	12.6	1.9	3.3	2.7	4.6	4.4	11.8	2.3
ZMPSTE24	8.3	13.7	2.0	4.3	3.3	4.4	3.6	11.5	2.2
LOC100130373	8.8	10.8	2.5	3.8	2.8	8.4	4.2	10.1	1.7
VPS45	8.3	13.0	1.8	4.0	3.4	3.9	4.0	12.5	2.2
ZCRB1	8.9	11.6	2.1	5.9	4.9	3.0	4.2	9.8	2.9
RAB5A	7.9	12.5	2.5	4.1	3.5	4.0	3.9	12.4	2.4
UBE2G1	9.0	14.0	1.9	3.4	3.5	4.8	3.3	11.2	2.1
PCGF1	9.9	11.2	2.5	4.0	4.8	3.7	4.3	10.2	2.4
GALT	10.3	12.8	2.4	3.5	4.1	4.2	4.1	10.0	1.8
KMT2D	8.8	11.0	3.2	3.6	3.6	4.2	4.0	12.6	2.2
ATP2B1	7.9	13.1	1.9	3.7	2.6	4.5	3.9	13.7	2.1
PPIF	8.6	10.1	2.8	7.0	6.1	3.0	4.7	8.8	2.2
E2F1	7.0	7.5	2.3	11.7	10.0	7.6	1.9	4.0	1.1
RECQL5	10.5	12.3	2.2	4.3	3.8	2.9	4.6	9.9	2.6
HIPK2	6.4	9.2	2.4	4.0	3.7	3.3	5.4	15.4	3.2
OPA1	9.0	15.1	1.9	4.7	3.3	4.3	3.2	9.8	1.8
RAB18	7.7	11.7	2.4	3.8	3.2	3.7	4.6	13.4	2.6
ENTPD1-AS1	9.0	13.9	2.5	3.2	2.4	5.5	3.3	11.5	1.8
KIF1A	9.6	11.6	3.4	3.4	3.5	1.9	5.1	12.0	2.5
UBE2V2	9.8	12.6	2.0	3.6	3.1	4.1	4.1	11.3	2.5
LOC100288911	8.2	12.0	2.8	9.5	7.6	4.8	2.5	4.6	0.9
LUC7L3	9.6	10.1	2.3	4.6	4.0	4.5	5.1	9.9	2.8
FNBP1	8.4	10.6	2.7	4.6	4.8	5.4	3.6	10.8	2.3
LOC101928809	8.4	13.7	2.3	3.6	2.4	6.5	3.6	10.7	1.8
ZNF385A	5.9	6.7	2.2	5.8	5.9	3.5	6.1	13.2	3.8
TEAD3	9.8	10.7	2.9	5.1	4.7	3.0	4.0	10.2	2.7
SMURF2	8.4	14.5	2.4	5.0	4.7	5.0	2.7	8.2	2.1
LZTS3	9.8	12.0	3.7	5.3	4.8	3.1	4.6	7.8	1.7
SPECC1	7.6	9.6	2.1	5.8	4.2	4.1	4.3	12.5	2.8
LOC100128239	9.3	11.7	2.8	4.0	2.9	6.9	3.6	9.9	1.8
ATXN7L3B	7.6	13.1	2.4	4.8	3.3	5.8	4.8	9.6	1.8
CLK2	11.1	13.3	2.8	3.1	2.5	2.5	4.4	10.1	2.9
SEL1L	7.5	11.4	2.3	3.4	3.2	3.0	5.4	14.1	2.6
API5	8.2	12.1	2.1	4.8	3.2	4.3	3.8	12.1	2.3
GBE1	9.3	16.5	2.4	3.6	2.8	3.7	2.8	10.1	1.6
ZAK	10.4	13.7	3.1	4.9	4.1	3.8	2.9	8.5	1.4
DCHS1	9.8	12.5	3.4	2.3	2.3	1.4	5.3	13.2	2.5
DAK	8.8	12.7	2.9	4.2	3.4	3.3	4.7	10.9	2.1
SPRED3	9.5	11.3	3.0	3.3	3.1	2.4	5.3	12.1	2.7
MAN2C1	9.3	12.8	2.4	4.7	3.5	3.6	4.8	9.8	1.8

RASA3	8.6	10.6	3.4	4.3	4.0	5.3	4.2	10.5	2.0
COPS7B	7.3	11.4	2.6	4.3	4.0	2.7	4.7	13.0	2.6
B3GALT6	8.9	10.6	4.9	3.9	4.5	2.3	5.3	10.0	2.4
NCDN	7.7	9.2	2.2	3.4	2.9	2.1	6.5	15.4	3.2
GLT8D1	8.6	12.2	1.9	4.1	3.7	3.5	4.2	12.1	2.5
NDNL2	8.6	9.7	2.2	5.4	4.6	4.4	4.4	10.7	2.6
TRIM14	11.1	13.1	3.6	4.3	4.0	3.4	3.9	7.5	1.7
C6orf120	9.9	11.6	3.3	5.0	5.1	2.6	4.5	7.6	3.1
ABCC3	7.2	8.8	1.6	4.2	4.3	1.9	5.8	15.4	3.6
SULT1A1	10.8	13.5	3.5	5.7	5.5	3.2	2.4	6.5	1.5
KDM3B	8.9	10.4	2.6	4.4	3.5	4.0	4.3	12.3	2.4
ADAMTSL5	11.0	13.5	3.7	5.7	6.0	2.9	2.6	5.6	1.6
LOC101929586	9.1	13.6	2.6	3.1	2.3	5.5	3.8	11.0	1.7
RNASEH2A	7.5	11.6	2.0	8.5	8.1	6.0	2.7	5.4	0.9
OGFOD1	8.2	12.1	1.9	4.3	3.1	3.4	4.4	13.0	2.2
LOC101929352	8.7	13.6	1.8	3.1	1.8	6.2	3.5	12.6	1.3
C16orf91	8.7	9.1	3.2	4.1	5.2	3.0	6.6	10.9	1.9
RAB3GAP1	8.6	12.7	2.3	4.7	3.5	3.8	3.8	11.5	1.8
RNF6	8.1	10.0	2.3	5.8	4.1	4.2	4.3	12.3	1.5
PPP1R15A	11.2	10.5	3.5	3.8	3.0	6.9	4.0	7.9	1.9
DNAJB12	9.0	10.6	2.6	3.7	3.2	2.8	4.9	13.0	2.7
IGF2BP2	10.9	12.5	3.1	4.6	4.3	3.5	3.2	8.4	2.0
GNL3L	8.1	11.3	1.9	4.2	3.9	4.6	4.4	12.3	1.9
KIF5C	8.1	11.5	2.7	4.5	4.1	3.7	4.3	11.8	2.0
CCDC71L	10.5	11.0	4.1	4.5	3.7	2.6	4.9	9.1	2.1
LOC101929202	11.1	11.6	3.6	2.9	2.7	1.3	5.5	11.5	2.4
ZSWIM4	8.2	11.5	2.9	3.1	2.8	2.1	5.3	13.7	3.0
PHF2	8.9	10.2	3.0	3.9	3.8	3.0	5.2	11.9	2.6
UNKL	8.3	10.7	2.7	5.6	4.0	4.4	5.1	9.4	2.2
UBA3	7.0	11.8	1.7	4.9	3.8	4.5	4.4	12.2	2.2
KCTD13	9.6	12.4	2.8	3.4	3.5	3.4	4.9	10.3	2.1
BTBD1	7.0	11.9	2.7	5.4	4.7	4.0	3.7	10.3	2.8
ETV1	8.5	9.0	2.2	1.3	1.4	0.6	7.5	17.7	4.1
IGFBP2	9.9	13.1	4.0	1.9	1.7	2.1	5.6	11.7	2.3
PTDSS1	7.2	11.8	1.9	5.2	4.0	4.4	4.3	12.0	1.6
EXOC3	9.1	11.1	2.5	4.6	4.9	3.8	4.4	10.0	1.9
LINC00987	7.2	8.5	2.7	5.7	5.8	1.8	5.0	11.8	3.8
HOXB7	9.2	12.9	2.3	6.4	5.6	3.2	3.5	7.0	2.0
DNER	7.6	11.4	2.0	8.5	8.6	4.1	2.2	6.5	1.3
UBE3C	7.6	10.9	2.2	4.7	4.0	4.8	4.1	11.8	2.2
APOL2	8.3	12.5	2.8	3.8	3.4	3.5	4.2	11.4	2.3
USP34	7.8	11.8	1.9	3.8	2.8	4.2	3.8	14.0	2.2
PC	7.3	8.7	2.3	5.3	4.7	3.1	5.5	12.9	2.4
ABHD14B	9.9	12.3	3.5	4.4	4.0	3.5	4.1	9.1	1.5
CDKN1C	10.8	12.5	3.6	4.4	3.8	3.2	4.1	7.3	2.5
RNF168	9.4	11.5	2.3	3.7	3.1	3.1	4.4	12.4	2.2
SCAF4	9.4	11.2	2.5	4.3	3.7	3.0	4.2	11.3	2.6

GTF2H5	8.0	13.5	2.1	4.9	2.9	7.0	3.6	9.4	0.7
PKD2	7.7	9.1	1.7	10.1	7.3	7.0	2.4	6.1	0.9
EPS15L1	10.2	11.6	3.2	3.6	4.2	3.1	4.2	9.8	2.3
RPS6KA3	10.1	15.6	2.3	4.4	3.8	4.8	2.3	7.4	1.5
SLC39A10	8.5	14.1	2.0	3.9	2.6	3.8	3.5	11.7	2.0
TMEM183A	8.7	12.9	2.0	4.2	3.3	3.4	4.6	10.9	2.2
ARHGEF25	9.7	11.2	2.4	4.5	4.3	2.7	5.0	10.1	2.3
SNHG4	9.7	13.4	2.1	3.1	2.1	5.4	4.0	11.1	1.3
IL10RB	8.2	11.7	2.3	4.6	4.5	3.5	3.6	11.3	2.4
MGAT5	8.3	11.5	2.5	3.5	2.9	2.7	4.2	14.3	2.2
CAND1	8.1	12.1	1.8	4.5	3.0	3.9	4.1	12.6	2.0
C12orf52	9.2	10.2	3.4	5.2	6.1	2.9	4.9	7.8	2.5
TNKS2	8.1	11.9	1.9	4.0	3.0	4.1	4.1	12.8	2.2
NUP88	7.9	11.5	2.0	5.1	3.8	3.7	3.8	12.0	2.2
LYPLAL1	9.3	15.5	2.7	4.8	3.0	3.9	3.1	8.1	1.5
ALKBH3	7.3	10.7	2.8	5.1	3.4	3.4	3.8	12.3	3.3
LINC00311	8.6	13.5	2.3	3.2	2.3	5.4	3.6	11.5	1.6
TAP2	8.1	10.5	2.3	3.7	3.5	5.1	4.8	11.8	2.3
SH2D4A	7.1	8.7	2.0	8.2	6.1	5.2	3.6	9.4	1.8
LENG1	10.3	7.3	3.2	4.6	4.9	3.4	6.4	9.3	2.6
FAM103A1	7.2	12.2	1.9	4.6	4.8	3.1	4.2	11.2	2.7
ARHGEF7	9.0	11.1	2.6	3.4	3.1	4.2	4.6	11.3	2.8
MTMR14	8.3	11.6	2.3	4.0	3.2	4.2	4.5	11.9	2.0
PRO2852	8.8	14.1	2.2	3.2	2.2	5.9	3.5	10.4	1.7
SPCS3	5.9	10.0	2.1	5.0	5.2	4.9	4.7	11.5	2.7
NPIPA8	9.4	12.1	2.3	3.4	3.1	1.7	4.1	12.8	3.0
NPIPA7	9.4	12.1	2.3	3.4	3.1	1.7	4.1	12.8	3.0
PRPF38B	9.3	10.0	3.1	4.2	3.3	3.6	5.7	9.8	2.8
SNRNP27	8.7	10.1	2.8	4.9	5.4	2.9	3.9	10.3	2.9
SPRY2	7.5	10.2	1.9	1.1	0.9	0.8	8.2	17.0	4.4
BCAS2	9.2	10.8	2.0	4.8	4.1	5.2	4.0	9.3	2.5
RBM22	9.4	9.7	2.7	3.7	3.7	2.7	5.0	11.8	3.1
STAT1	8.4	11.9	1.9	4.9	4.4	5.2	3.3	10.2	1.8
RNF121	7.7	10.0	1.9	4.8	3.7	2.9	4.8	14.1	2.0
GALE	11.1	14.2	2.9	5.2	4.5	3.2	2.8	6.6	1.3
ACTL6A	7.5	9.8	1.4	4.7	3.5	4.6	4.5	13.6	2.4
SMARCC1	8.9	9.6	2.6	3.8	3.3	3.8	4.9	12.0	2.9
PAQR5	6.6	10.2	1.7	9.1	9.4	4.3	2.2	6.9	1.4
DKC1	6.9	10.1	2.2	4.2	4.0	4.4	5.2	12.8	2.1
ZNF706	10.5	13.3	2.7	4.2	3.2	2.6	3.9	9.6	2.0
GRPEL1	7.8	10.3	2.4	5.1	4.2	3.1	4.5	11.8	2.6
LOC730268	10.4	14.4	2.7	4.3	3.8	3.1	3.5	9.1	0.4
NFU1	7.6	10.9	2.6	5.2	5.3	4.7	3.1	9.5	2.9
PIANP	9.8	9.8	3.8	4.5	4.3	2.1	5.0	9.0	3.5
PDGFC	8.6	11.5	2.1	5.3	4.2	4.4	3.5	10.0	2.1
IFT140	10.5	11.9	2.8	3.5	3.6	2.2	4.7	10.4	2.1
TXNL1	10.0	11.4	2.5	4.4	3.1	2.8	4.1	11.2	2.4

R3HCC1	8.2	11.2	2.9	3.7	3.9	2.5	5.1	11.4	2.7
UROS	7.4	13.0	2.2	5.4	4.9	3.8	4.0	9.0	2.0
DDX6	8.1	12.2	1.9	3.9	3.1	5.7	3.4	11.6	1.8
LMF1	8.7	10.3	2.8	4.5	4.4	1.8	5.7	10.7	2.9
FAM69B	8.1	11.2	3.4	7.4	6.5	3.5	3.5	6.6	1.3
UTP6	7.8	12.2	1.8	3.8	2.5	3.2	4.3	13.3	2.8
TPR	8.4	10.8	2.2	4.4	3.6	4.0	3.7	12.5	2.0
ABCB6	8.6	11.0	2.3	4.8	4.0	2.2	5.0	11.3	2.4
GHDC	9.4	11.6	3.0	4.2	3.5	3.5	4.6	9.8	2.0
NUDCD2	6.3	11.2	2.6	5.6	3.1	6.2	4.9	10.0	1.7
SDC4	5.3	7.9	1.9	6.1	6.5	3.4	4.3	13.4	2.7
CDKN1B	6.5	8.1	1.6	2.7	2.6	6.5	7.0	13.2	3.2
OSBPL3	7.1	10.1	1.9	4.7	3.8	3.6	4.6	13.3	2.6
ZFYVE21	9.0	12.3	2.9	5.6	4.7	3.7	4.1	7.5	1.8
SLC35F5	7.9	13.6	1.6	4.2	2.8	3.5	3.3	12.5	1.9
LOC100132077	8.7	13.5	2.5	3.4	2.4	4.2	3.4	11.7	1.8
SMU1	8.2	12.0	2.2	3.9	3.8	4.0	4.0	11.5	1.9
SNORD94	1.1	16.5	1.1	1.7	1.4	15.5	4.3	9.0	0.9
DNAJB14	8.8	12.3	2.4	4.1	3.2	5.1	4.4	9.4	1.8
MRPL50	7.2	13.4	1.6	4.5	3.7	4.7	3.4	11.1	1.9
LSM2	6.6	12.6	2.1	4.5	4.3	2.5	4.2	11.6	3.0
PPRC1	7.8	10.0	2.6	3.7	3.6	3.1	4.8	13.3	2.6
DOM3Z	9.9	10.9	2.2	3.3	2.8	2.4	5.3	11.9	2.8
DYX1C1-CCPG1	6.8	11.1	1.7	2.9	2.0	2.5	4.9	16.1	3.2
ZNF148	7.5	10.8	1.8	4.5	3.9	3.9	3.7	12.8	2.3
SNAR-I	7.9	3.7	4.8	7.5	2.8	1.6	15.7	7.1	0.3
BTG2	5.6	7.4	1.9	2.2	2.3	21.5	2.5	6.0	1.8
REST	7.2	10.7	1.8	3.9	3.2	4.5	4.4	13.2	2.3
TK1	7.2	9.7	2.6	11.3	9.7	6.3	1.3	2.9	0.3
SCO1	8.7	11.0	2.3	5.4	4.1	4.7	3.5	9.7	1.9
LOC101929333	9.6	14.3	2.8	2.7	2.7	3.4	3.4	10.7	1.7
SCARB1	10.8	12.1	3.2	5.8	5.4	2.6	3.1	7.0	1.3
SOCS5	6.7	11.3	1.8	4.6	4.3	4.4	3.8	12.1	2.2
ACAD9	7.2	10.6	2.2	5.7	5.0	4.1	4.1	10.3	1.9
ITPK1	9.0	9.0	2.6	5.9	5.7	4.4	3.9	9.0	1.8
POLR3GL	9.9	10.9	3.1	4.8	4.8	2.7	4.8	8.3	1.9
AP5Z1	9.3	11.4	2.6	4.0	3.5	5.2	4.3	8.7	2.1
CLEC16A	9.6	11.2	3.0	4.8	4.6	3.4	3.7	9.4	1.6
FUNDC2	7.9	11.4	2.2	4.7	3.1	3.3	4.5	11.7	2.5
COMMD7	7.6	13.1	2.1	4.8	4.6	3.9	4.1	9.7	1.3
JOSD1	9.6	12.1	2.5	5.0	3.8	4.2	3.4	9.4	1.2
MICU1	8.4	13.4	2.7	5.1	3.7	4.7	3.2	8.4	1.8
IFT27	6.9	11.4	2.4	4.3	3.1	2.4	5.0	13.3	2.5
HEXDC	8.8	11.0	2.6	3.1	2.6	2.9	6.6	11.1	2.5
LPGAT1	8.2	11.8	2.2	4.7	3.3	4.0	3.5	11.6	1.9
PHKG2	6.6	10.7	1.8	5.6	3.9	5.6	4.0	10.7	2.2
AAR2	8.9	11.2	2.6	3.8	3.0	2.7	4.5	12.3	2.1

EDA2R	8.8	9.4	1.8	3.6	3.0	1.9	6.2	14.1	2.3
DHRSX	7.7	11.6	2.7	4.4	4.4	2.9	4.4	10.4	2.5
DHRSX	7.7	11.6	2.7	4.4	4.4	2.9	4.4	10.4	2.5
LOC100630923	9.3	10.3	2.8	3.9	3.5	2.7	5.7	10.2	2.5
RFC2	5.9	7.7	2.0	8.6	7.3	5.6	3.8	8.4	1.6
BAG4	9.0	11.8	2.6	5.1	4.4	2.9	3.4	9.5	2.2
BBIP1	9.7	12.1	2.1	4.1	2.5	2.0	3.0	11.8	3.6
SPPL3	9.1	11.8	2.1	3.6	3.1	2.7	4.5	11.0	3.0
ZC3H15	8.2	10.2	2.1	4.0	2.8	4.6	4.5	12.5	2.0
VTRNA1-3	3.5	14.8	4.4	0.0	1.8	1.5	10.4	13.3	1.2
RAD50	8.6	11.4	1.9	4.8	3.5	3.7	3.1	12.0	2.1
CDK13	8.8	11.0	2.2	3.3	2.5	3.3	4.5	12.6	2.7
BTBD3	8.8	14.8	2.8	3.3	3.2	3.1	3.6	9.6	1.8
MIR22HG	7.5	10.6	2.0	3.3	2.3	2.4	5.7	14.4	2.8
RWDD1	6.7	9.6	1.7	5.3	4.2	5.4	3.9	11.9	2.2
TRADD	7.2	11.1	2.7	5.0	4.8	4.2	4.8	9.7	1.5
TRUB1	7.8	10.2	1.8	5.1	4.4	4.3	4.3	11.0	2.0
PDGFB	11.7	14.4	3.8	3.4	2.6	1.7	3.7	7.0	2.6
DGKA	8.2	11.4	2.0	4.9	3.9	7.8	3.0	8.2	1.5
ACO1	8.4	13.4	1.9	4.4	3.7	4.0	3.3	10.3	1.5
POLR3H	7.2	9.5	2.1	3.5	3.5	3.2	6.2	13.1	2.6
PTK2	8.4	12.3	2.6	4.6	3.8	3.5	3.1	10.8	1.9
ING1	8.2	7.7	3.3	3.9	4.8	2.2	6.7	11.2	3.0
EIF2B1	7.5	11.6	2.0	4.7	3.2	4.7	4.0	11.0	2.2
MAP4K4	6.5	9.3	2.1	2.8	3.0	2.1	5.1	17.0	3.1
SPIRE1	9.1	12.8	2.7	4.8	3.7	3.4	3.3	9.5	1.7
RHOD	8.4	11.4	2.5	6.7	5.8	3.6	3.8	6.6	2.1
TCP11L1	7.9	11.2	2.0	3.7	3.7	2.8	4.3	12.8	2.4
VPS39	8.4	11.6	2.6	4.8	3.5	4.3	3.6	9.8	2.3
WDR5	8.4	10.9	2.0	4.5	4.3	5.0	4.0	10.2	1.6
SGSM2	9.1	11.6	2.4	4.8	4.6	3.0	4.4	8.9	2.1
HIPK1	7.1	9.3	2.1	3.2	2.8	3.4	5.2	14.9	2.9
PHKB	8.2	12.7	1.9	4.7	3.8	4.1	3.2	10.2	2.0
TRMT1	8.7	10.4	2.5	3.8	3.9	3.0	5.2	10.7	2.6
NOMO2	8.5	10.4	2.2	3.8	3.1	2.4	4.8	13.0	2.6
B3GAT1	10.1	11.6	3.5	4.1	5.2	1.7	3.7	8.0	2.7
KANK1	9.1	11.2	2.8	6.4	5.3	3.8	3.1	7.7	1.4
AGO2	8.8	10.5	2.8	4.0	3.8	3.9	4.0	10.9	2.0
TXNRD2	9.1	13.4	2.6	5.1	3.8	4.4	3.7	7.6	1.1
C12orf23	7.7	9.8	2.7	3.7	3.1	3.1	5.3	13.0	2.4
SLK	8.7	10.2	2.1	2.8	2.5	2.9	4.4	14.6	2.4
SPRYD7	8.4	12.0	2.0	4.3	3.2	4.7	3.8	10.1	2.2
RNU6-2	10.6	4.5	4.0	3.7	0.7	5.5	5.8	12.2	3.5
HUS1	8.7	12.2	2.3	4.1	3.0	4.1	3.5	11.0	1.6
PBX3	6.3	8.8	2.2	4.4	4.2	3.8	4.3	13.1	3.4
ANKRD9	8.6	11.4	3.2	5.4	5.0	3.3	3.8	7.7	2.2
CD47	9.0	12.9	1.7	2.6	1.9	5.9	3.4	11.6	1.6

PWP2	8.7	11.8	2.5	3.9	3.7	3.0	4.5	10.7	1.8
HOXB4	11.6	14.2	4.3	3.7	2.3	1.6	3.4	6.6	2.8
SOCS7	8.4	11.2	2.7	2.9	2.7	2.2	5.6	12.3	2.7
CAAP1	8.1	9.6	2.5	3.3	3.2	2.9	5.3	13.3	2.5
SETDB1	9.9	12.1	2.7	2.9	3.5	2.9	3.5	11.2	1.8
LOC101927131	8.6	13.3	2.2	2.8	2.2	5.7	3.2	10.9	1.6
UBE2A	7.9	11.9	2.4	5.3	4.0	4.1	4.3	8.1	2.5
UQCC	8.2	12.5	1.6	4.3	3.7	3.7	4.0	10.5	2.0
C16orf62	9.2	11.6	2.5	4.8	4.2	3.6	3.6	9.2	1.9
SMIM14	6.1	10.9	1.6	4.2	2.5	3.5	3.7	16.0	2.1
TSC22D3	3.4	5.8	0.9	2.9	2.1	21.8	3.6	8.4	1.7
GATSL3	9.9	13.1	3.5	3.9	3.7	1.9	4.1	8.5	1.9
LINC00958	8.4	13.2	2.0	2.7	2.3	5.8	3.4	11.2	1.5
MOB4	7.3	11.4	2.1	3.5	2.4	4.8	4.1	12.9	1.9
ADAMTS7	10.5	13.2	3.2	3.4	3.4	2.1	3.8	8.7	2.0
TOP2B	8.1	11.1	1.8	3.8	3.1	4.7	3.4	12.2	2.2
FAM57A	8.5	11.5	3.0	5.3	5.7	2.6	3.0	8.5	2.2
HCCS	7.7	11.5	2.4	5.5	3.3	3.6	4.2	10.6	1.5
LOC100507236	8.4	13.2	2.2	3.2	2.1	5.4	3.4	10.5	1.9
GBA2	8.5	11.7	2.2	3.1	2.1	2.2	5.5	12.6	2.5
TOMM70A	7.6	13.0	2.3	3.8	2.9	2.7	4.1	11.6	2.3
FNDC4	8.9	12.2	3.4	5.0	4.4	3.2	3.5	8.0	1.7
TMEM50A	7.8	14.7	0.7	3.7	3.5	4.9	2.6	10.9	1.4
MRPL45	7.6	12.0	1.9	4.7	3.9	3.6	3.3	10.7	2.3
ICOSLG	6.8	8.5	1.8	1.4	1.5	2.3	7.3	17.7	3.0
PPP2R5C	7.6	11.6	2.0	3.8	4.1	5.2	3.2	10.4	2.3
PURA	6.9	10.6	2.8	4.1	3.5	2.9	5.5	11.6	2.2
GNPAT	7.6	13.1	1.7	4.5	2.9	4.2	3.1	10.9	2.0
ADSS	8.4	12.3	2.0	4.6	3.0	4.3	3.1	10.3	2.0
EML3	8.1	10.7	2.8	4.5	3.6	4.4	4.2	9.6	2.3
NME6	8.3	12.7	2.1	3.3	2.5	4.9	3.8	10.7	1.7
GTF2IRD2	9.7	9.1	2.1	3.5	2.8	2.5	6.1	11.9	2.4
EFHD2	7.8	9.2	2.9	5.8	5.0	7.1	3.6	6.8	1.7
NOP16	7.6	9.9	2.3	4.6	5.0	3.8	4.4	10.7	1.7
RANBP9	7.4	9.7	2.8	5.0	4.7	3.5	3.8	10.4	2.8
RMND5B	7.8	11.3	2.4	4.4	3.1	4.6	4.3	10.5	1.8
UNC119B	6.9	10.0	2.8	4.1	5.2	3.0	4.9	10.0	3.0
CLEC2D	8.1	10.4	2.3	4.9	3.8	5.1	3.8	9.3	2.4
BLCAP	7.5	10.9	2.4	6.4	3.8	3.2	3.5	9.9	2.5
INPP1	8.5	10.3	2.3	5.5	4.2	3.3	3.6	10.4	2.0
KCTD11	7.9	9.0	2.6	4.3	4.3	2.7	4.8	11.5	2.9
EDC4	7.7	10.3	2.7	4.1	3.7	3.2	4.7	11.3	2.2
44624	9.2	14.4	4.0	5.4	5.2	4.6	2.0	4.2	1.0
RABEPK	6.9	10.0	1.9	3.7	3.4	2.4	5.7	13.2	2.7
GRIPAP1	9.3	8.5	2.9	3.8	3.5	4.0	5.3	10.5	2.3
SNHG18	7.9	10.2	2.1	3.8	3.4	2.5	5.1	12.5	2.5
TRAF3IP2	8.3	13.2	2.3	3.1	2.3	5.7	3.2	10.4	1.5

FAM221A	8.6	13.4	2.0	3.0	2.2	5.3	3.3	10.6	1.6
SH3BGRL	5.9	12.3	1.5	4.2	3.4	5.4	3.0	11.5	2.7
MED13	7.8	12.3	2.0	3.4	2.7	3.4	3.5	12.9	1.8
HS1BP3	9.1	13.5	2.6	4.7	4.2	4.0	3.1	7.5	1.2
CNDP2	6.5	9.9	2.2	4.1	3.2	4.9	4.7	12.5	1.7
TBC1D10A	9.6	11.8	2.3	3.4	3.5	3.8	4.2	8.7	2.6
MPI	8.5	10.7	3.0	5.6	4.9	3.3	3.7	8.6	1.6
ALPK2	6.8	9.1	2.0	8.0	6.9	6.5	2.4	7.1	1.1
GOLGA4	7.8	9.5	2.0	3.9	2.8	3.8	4.1	13.4	2.4
OTUD4	8.2	11.7	2.1	4.0	3.3	3.3	3.6	10.9	2.6
NCBP2-AS2	7.7	11.2	2.1	3.7	3.0	2.8	5.6	11.2	2.4
PCCB	8.5	11.9	3.0	4.3	4.2	3.5	3.8	8.9	1.6
FHL3	8.3	10.4	2.6	5.4	4.9	3.7	4.1	8.8	1.7
LOC101929524	8.6	11.3	2.3	3.0	2.0	9.2	3.1	8.8	1.6
LPP	8.8	10.9	2.5	3.6	3.5	3.2	3.7	11.3	2.2
ABCA1	9.7	14.1	2.6	4.8	3.5	3.6	2.5	7.8	1.1
ARIH1	7.8	11.2	2.7	3.9	3.0	4.4	4.6	10.4	1.6
LINC00923	7.8	11.7	2.6	2.2	2.2	2.2	4.8	12.3	3.8
HIST1H1D	6.6	9.6	2.5	6.9	5.8	10.5	2.2	4.7	1.0
IFI6	8.2	14.0	3.0	4.8	3.7	5.7	2.6	6.7	1.1
ATMIN	7.5	10.7	2.2	4.0	3.7	3.2	4.0	12.0	2.4
LOC283335	7.8	13.2	1.9	3.3	2.7	4.4	3.1	11.4	1.8
D2HGDH	8.5	11.3	2.0	3.6	3.6	3.8	4.2	10.7	1.9
TRNH	11.2	10.9	4.4	1.2	1.2	0.6	10.1	9.4	0.7
KCTD2	7.1	10.6	2.3	4.1	4.2	4.0	4.3	10.6	2.5
PACSIN2	8.2	9.6	3.4	5.4	5.3	3.5	4.2	8.1	1.9
44629	7.0	8.8	2.6	4.2	3.7	3.1	5.4	12.2	2.6
S100A3	7.6	9.6	2.3	8.2	9.1	5.5	2.1	4.0	1.3
TSSC1	8.7	10.4	2.4	4.7	3.8	4.1	3.7	9.6	2.1
IFI35	9.1	13.6	2.7	3.9	4.1	4.0	2.7	7.9	1.6
ZEB2	8.6	11.4	2.7	4.2	3.6	4.1	3.3	9.7	1.9
NUP85	7.9	11.8	1.8	4.4	4.0	3.7	3.6	10.8	1.6
TRNAI11	0.8	2.3	3.1	3.8	0.4	0.2	19.7	19.0	0.3
ARRB1	7.3	7.6	2.3	6.5	6.1	5.7	3.2	8.7	2.0
NOP2	8.9	11.2	2.3	3.1	2.8	2.8	5.0	11.1	2.3
FBXO31	7.9	11.3	2.8	3.4	3.3	3.7	4.6	9.9	2.5
RMND5A	8.0	10.5	2.3	3.0	2.8	3.1	4.2	12.7	2.8
RAI14	9.4	12.0	2.6	3.2	3.0	2.1	4.1	10.8	2.3
ZNF384	8.9	8.3	2.7	3.7	3.9	2.4	5.5	11.0	3.0
MRPS30	7.2	11.6	2.4	4.2	4.0	3.1	4.2	10.2	2.4
STK16	8.1	10.6	2.4	4.5	3.7	2.5	4.8	11.0	1.9
TENC1	10.2	11.3	3.5	3.7	3.4	1.7	3.9	9.3	2.2
CCDC85C	8.5	10.6	3.6	6.0	6.4	2.2	3.8	6.5	1.8
TK2	8.6	10.6	2.9	3.5	2.6	2.1	5.0	12.0	1.9
TANGO2	9.4	10.0	2.7	4.7	4.8	3.0	3.7	8.8	2.3
DERL2	6.9	12.8	1.4	5.5	3.2	3.8	3.4	10.5	1.8
DNAJC14	7.7	9.7	1.9	4.8	3.6	3.8	4.7	10.8	2.4

LAMP2	8.3	12.8	1.7	5.0	3.4	3.8	3.4	9.2	1.7
ATHL1	10.2	12.5	2.7	5.3	4.0	3.9	3.4	5.4	1.8
NICN1	8.6	10.6	2.4	5.0	4.3	4.5	4.4	7.6	1.8
TM2D3	8.2	11.5	1.7	4.9	3.3	3.6	4.3	10.1	1.7
GGNBP2	8.0	12.2	2.1	3.9	2.9	3.7	3.6	10.9	2.0
TRAF2	7.9	10.7	2.5	4.9	4.8	3.4	3.7	9.3	2.1
ACP2	7.3	9.4	1.7	6.4	5.5	3.9	4.1	9.3	1.7
TRIM4	7.3	9.2	2.3	3.5	3.5	3.4	5.3	12.5	2.2
RAB2B	8.4	12.3	1.9	3.5	2.7	4.6	3.6	10.9	1.4
LOC101927875	7.9	13.3	2.4	2.9	2.2	4.3	3.2	11.6	1.5
BNIP3	7.6	11.6	1.9	4.8	3.6	2.4	2.9	11.8	2.6
ZDHHC9	7.2	8.0	1.8	3.2	3.0	1.9	5.5	15.1	3.3
SYNGR1	9.8	12.3	3.3	4.6	4.9	1.7	3.5	7.0	1.9
TTC37	7.7	12.5	1.8	4.4	3.3	3.7	3.3	10.7	1.8
LMBR1	8.0	12.6	1.6	4.7	3.0	4.4	2.6	10.8	1.3
HBE1	9.9	14.8	2.6	3.7	3.2	3.0	2.9	7.7	1.3
ZFYVE27	7.2	10.8	2.0	3.6	3.1	3.6	4.5	12.4	1.7
RIN2	5.8	7.4	2.0	3.4	3.4	2.4	6.0	15.0	3.4
LOC100506746	8.1	12.8	2.2	2.9	2.1	4.8	3.5	10.7	1.8
TRAPPC4	6.8	11.7	2.0	4.4	3.3	3.2	5.0	11.1	1.5
CPNE3	8.2	13.3	1.9	4.2	2.8	3.3	3.2	10.8	1.3
ZNF652	8.4	8.9	2.4	2.9	2.8	2.3	3.9	14.8	2.6
U2SURP	7.6	11.5	1.8	4.3	3.4	3.9	3.4	11.3	1.9
TECPR1	10.0	12.3	2.4	4.2	2.7	3.5	4.2	8.1	1.6
RAB7L1	8.5	11.1	2.0	5.2	4.8	4.6	2.6	8.9	1.4
ZNF622	7.7	9.9	2.2	4.2	3.8	4.7	4.0	10.1	2.3
AIG1	7.7	12.2	1.4	5.3	4.2	3.6	4.4	8.8	1.3
ZNF668	7.8	10.2	3.0	5.3	5.3	3.2	3.9	8.1	2.0
AKAP1	7.4	9.1	2.4	3.2	3.2	2.1	5.1	13.6	2.7
THOC7	8.0	12.7	2.2	4.5	4.3	3.8	2.6	8.9	1.9
UBTD2	6.3	8.5	2.1	3.6	3.4	2.4	5.9	13.8	2.8
NXT1	7.4	9.4	2.4	3.7	3.3	2.2	4.4	13.5	2.4
SRF	7.5	8.2	2.8	4.6	5.6	3.0	4.8	9.7	2.6
PRKDC	8.0	11.3	2.2	3.8	3.3	4.4	2.9	11.3	1.5
SNRPF	6.6	11.9	2.1	6.2	3.1	7.1	3.9	6.4	1.5
ME1	6.4	8.7	1.4	7.3	4.6	4.9	3.3	10.6	1.7
KLHL36	8.4	11.7	2.6	3.9	3.5	3.3	3.9	9.6	1.8
IFT57	7.6	11.0	2.3	4.4	3.4	3.4	3.9	10.8	2.1
GTF2H1	7.5	10.6	1.6	4.6	3.5	3.9	3.7	11.0	2.3
MIRLET7BHG	9.3	11.9	2.5	2.6	1.9	4.8	3.8	10.0	1.9
MAPK6	8.4	12.3	2.2	3.1	2.6	2.8	3.5	11.9	2.0
ZNFX1	7.7	9.9	2.4	4.2	3.6	3.8	4.0	11.1	2.1
RARA	7.6	9.7	1.7	3.7	3.1	7.5	3.8	9.2	2.4
PACSIN3	7.7	9.0	2.5	6.2	6.3	3.6	3.5	8.2	1.7
PSMG1	7.1	9.6	2.7	5.9	4.4	3.0	4.1	9.1	2.8
RDH14	9.2	12.9	2.3	4.7	3.4	3.4	3.4	8.3	1.2
IGBP1	6.9	11.4	2.2	4.9	4.1	3.8	3.5	10.0	1.8

MISP	7.8	9.2	3.2	8.8	9.0	5.4	1.8	3.0	0.6
CHRAC1	7.4	8.3	2.2	4.2	3.7	3.5	4.3	12.2	2.8
SDHD	6.3	12.0	2.1	4.8	3.9	3.7	3.6	11.1	1.2
ZDHHC3	8.0	11.6	2.0	5.3	3.9	3.4	3.6	9.4	1.5
FAM177A1	6.7	8.4	2.8	3.5	3.7	2.5	4.9	12.6	3.4
ERP44	8.6	12.3	2.2	3.6	3.1	4.2	3.0	9.5	2.2
WTIP	8.1	10.7	3.3	7.5	6.7	3.7	2.0	5.6	1.0
FUT11	7.7	10.5	3.2	3.6	4.3	2.9	4.5	9.2	2.7
UBAC2	8.2	9.4	2.3	4.1	3.5	3.0	4.7	11.4	2.0
WAC-AS1	7.7	11.9	1.9	3.9	3.0	4.2	3.5	10.8	1.5
SEMA4B	8.9	10.8	2.9	3.4	3.1	3.6	4.2	9.6	2.0
CASP8	8.3	11.3	2.1	3.1	2.4	7.5	3.0	9.3	1.5
MTFR1L	8.9	11.5	2.5	3.9	3.8	2.4	3.5	9.0	3.0
SKIL	7.3	10.1	1.6	3.5	2.8	3.1	4.2	13.3	2.7
TTC17	6.9	8.4	1.6	3.4	2.7	3.1	4.6	15.2	2.6
GNE	6.3	11.0	1.8	4.9	4.2	3.6	3.5	11.6	1.6
LOC101928501	9.3	13.3	2.2	2.7	1.8	1.5	4.1	11.8	1.4
POLB	7.2	9.7	1.5	5.4	4.3	4.6	3.7	10.5	1.3
SNORA76	3.1	25.2	1.8	2.3	2.3	4.9	3.6	4.5	0.5
GNAI3	7.9	11.8	1.8	3.3	1.9	4.1	4.1	12.0	1.3
WDR43	7.2	9.9	2.2	4.1	3.6	3.2	4.3	11.5	2.3
ZBTB22	8.0	10.5	3.0	3.4	3.0	1.8	5.2	11.3	2.1
LINC00338	8.0	12.0	2.4	3.1	2.4	3.6	3.8	10.9	2.3
RASSF8	6.0	8.2	1.6	3.0	1.8	2.0	5.4	17.4	2.8
VPS51	8.1	10.1	2.5	3.6	3.0	4.0	5.1	9.3	2.4
HIST1H4A	4.3	7.9	1.3	7.1	5.0	15.4	2.1	4.0	1.1
TCOF1	7.5	8.0	2.2	4.4	4.2	4.7	4.8	10.2	2.1
SLC23A2	9.0	11.0	2.4	4.5	3.4	3.6	3.3	9.5	1.4
ANP32E	7.6	8.0	2.5	6.9	5.0	6.3	3.7	7.1	1.2
STX8	7.5	10.4	2.3	4.5	3.7	3.3	3.8	10.1	2.7
CCNJL	7.9	8.5	2.7	3.4	3.5	2.0	5.1	12.3	2.8
NR2C2	8.5	10.2	2.2	3.4	3.5	3.4	3.7	11.3	2.1
ETNK1	8.8	9.9	2.6	3.6	3.5	2.7	5.0	9.8	2.3
GLTPD1	8.2	10.1	3.0	4.6	5.8	2.5	3.8	7.8	2.3
PCDH1	10.2	12.9	3.3	3.3	2.6	3.0	3.1	7.7	1.9
ZNF446	7.8	9.7	2.7	3.6	3.8	2.1	4.9	11.0	2.5
STK40	7.5	9.9	2.5	3.6	2.9	4.3	4.6	10.8	1.8
AGFG1	7.9	10.8	1.9	3.7	3.7	3.2	3.6	11.2	2.1
DSE	6.2	9.4	1.9	3.4	2.9	3.1	4.9	13.9	2.4
TOR1AIP1	7.1	9.9	1.8	3.8	2.8	3.1	4.5	12.3	2.6
C19orf25	7.8	9.0	2.7	4.7	4.5	3.1	4.9	9.5	1.7
SFT2D2	5.9	9.1	1.7	3.9	3.2	5.7	4.2	12.8	1.5
PHTF2	7.6	12.0	1.7	4.5	3.4	3.6	3.0	10.4	1.9
WDR83	8.1	10.5	2.5	3.8	3.4	2.9	4.9	9.9	2.0
C9orf123	8.6	10.8	2.2	5.5	4.4	2.7	3.9	8.5	1.3
IGF2BP1	8.7	9.9	2.6	4.1	3.6	2.5	4.7	9.9	1.8
SNX27	7.1	9.3	2.6	4.1	3.4	3.0	4.1	12.4	2.0

HIST1H2BH	8.0	7.7	3.7	6.5	7.4	3.7	3.7	5.8	1.5
POLM	9.3	10.3	2.7	3.8	3.2	2.3	4.4	9.8	2.2
SNPH	7.6	9.0	2.6	3.6	3.5	2.8	5.2	11.5	2.1
MTMR4	7.7	10.4	2.0	4.7	4.2	3.5	3.6	9.7	2.0
UNC93B1	9.0	11.7	3.0	3.5	2.8	3.7	3.7	8.7	1.9
PDCL3	7.9	10.7	1.8	3.8	3.3	4.5	3.7	10.1	2.0
EDEM2	8.0	10.5	2.4	5.4	4.7	4.0	3.4	7.7	1.7
TUBGCP2	7.8	10.2	2.5	3.3	2.8	2.8	4.8	11.0	2.7
PPIE	8.6	12.1	2.1	4.0	2.2	3.3	3.3	11.0	1.0
STAT2	9.0	11.1	2.2	3.9	3.0	3.4	3.4	10.3	1.6
ECHDC1	7.4	12.0	2.0	3.8	2.7	5.6	3.1	9.5	1.7
EIF2B2	7.7	10.2	2.1	4.5	4.7	2.9	3.5	9.9	2.2
ASIC1	6.5	7.4	2.0	3.3	3.1	1.5	6.6	14.2	3.2
PRPF40B	8.9	9.5	2.3	3.2	3.0	1.9	5.1	11.4	2.4
SF3A3	8.7	9.3	2.3	3.6	3.4	3.2	4.4	10.3	2.7
CYLD	6.6	10.4	1.4	5.2	3.8	5.8	3.0	9.9	1.6
DNAAF3	6.8	8.9	2.0	5.2	4.6	3.9	4.4	9.7	2.3
AZIN1	7.4	11.9	1.6	4.4	3.0	5.6	2.9	9.4	1.4
NIPA2	6.6	10.5	1.6	4.2	2.8	4.8	4.0	11.3	2.0
CTIF	10.9	10.9	3.7	4.7	4.5	3.0	3.0	5.6	1.4
FLJ31104	8.1	11.9	2.1	2.6	2.1	6.2	3.1	10.3	1.3
UBE2J2	8.2	9.8	2.1	3.8	4.0	3.4	4.4	9.9	2.1
NADSYN1	8.7	10.7	2.3	4.2	3.8	2.9	4.2	8.8	2.0
FRY-AS1	8.0	12.6	2.3	2.9	2.2	5.5	2.8	9.9	1.4
ARMC10	7.9	12.4	2.0	4.3	2.5	2.9	3.7	10.1	1.8
SLC30A9	8.1	11.4	2.0	4.1	3.4	3.7	3.2	10.2	1.5
SPAG9	7.6	10.9	1.9	3.6	2.8	3.7	3.6	11.7	1.9
ATXN1	8.9	8.6	3.5	2.4	2.4	2.1	5.3	11.3	3.1
C19orf71	8.3	11.1	2.8	4.0	4.2	2.0	4.0	9.4	1.8
NUPL1	8.3	11.8	2.3	4.2	4.0	3.5	2.9	8.8	1.7
44627	8.2	12.0	1.7	4.0	2.7	4.0	3.0	10.4	1.8
LOC100506922	7.7	6.7	3.4	3.9	4.1	4.0	5.1	9.2	3.4
HPS1	7.0	10.6	2.5	4.8	4.1	4.8	4.0	8.0	1.8
LOC101929017	7.7	12.1	2.1	2.8	2.0	6.1	3.0	10.1	1.6
COQ10B	6.4	10.8	2.5	3.6	3.2	3.3	4.8	11.1	1.9
LOC101929652	8.7	12.1	2.0	2.7	2.0	4.7	3.4	10.4	1.5
JMY	5.7	7.0	1.4	2.1	2.0	4.3	5.8	16.1	3.1
MSRB1	6.4	9.5	1.9	5.2	5.1	4.9	3.9	8.8	1.8
MRPS14	6.7	10.9	1.6	3.5	3.4	4.3	3.6	11.7	1.7
GABARAPL1	7.2	10.9	2.0	4.2	4.7	5.4	2.6	8.5	1.9
FILIP1L	8.5	8.8	2.2	5.1	3.9	3.8	3.5	9.5	2.0
USP32	8.1	12.1	2.0	3.8	3.0	3.8	3.1	9.7	1.8
SYBU	13.5	16.6	3.7	2.2	2.7	1.7	1.8	4.4	0.9
ZDHHC1	8.4	9.7	2.5	4.9	4.6	2.7	3.7	8.5	2.3
ZFYVE20	7.9	10.7	2.0	2.7	2.7	2.4	4.1	12.9	1.9
KDM5D	7.2	10.9	1.7	3.2	2.6	2.2	4.9	12.2	2.6
SLC4A11	7.9	10.1	2.6	4.3	3.9	2.8	4.3	9.4	2.3

KPNA6	7.9	10.4	2.1	4.5	3.4	3.5	3.6	10.3	1.7
FARSB	7.2	11.5	1.8	4.1	2.9	3.1	3.4	10.9	2.3
ACBD5	8.3	11.5	2.5	3.8	2.9	3.1	3.8	9.9	1.6
LOC101927884	8.8	12.8	2.4	1.9	1.3	4.0	2.7	12.3	1.2
RNF19A	4.2	6.1	1.1	4.0	3.5	4.6	4.6	16.7	2.7
PET117	8.7	8.7	2.8	4.6	3.5	2.9	4.4	10.0	1.7
CCDC86	7.2	8.0	2.5	5.0	5.3	4.2	4.3	8.7	2.1
LRTOMT	8.1	11.8	1.9	2.9	2.5	4.9	3.3	10.3	1.8
ANKRD39	7.9	9.9	2.7	6.0	5.6	3.4	3.2	7.4	1.3
TJP1	7.5	10.0	2.1	3.9	3.6	2.9	3.6	11.4	2.2
ATP6V0A1	8.8	12.9	2.3	2.5	2.2	2.3	3.4	10.7	2.2
UBTF	7.7	8.8	2.5	4.1	3.9	3.8	4.6	9.6	2.3
SARDH	9.7	13.1	2.9	6.6	5.7	3.7	1.6	3.2	0.8
SIAH2	6.9	8.9	2.3	2.9	2.9	2.8	5.1	12.0	3.4
ATP2C1	6.6	11.1	1.5	4.5	3.1	4.4	3.2	11.2	1.6
GGPS1	8.7	10.8	2.0	3.8	2.9	5.0	3.1	9.2	1.7
DDX19A	8.3	9.1	2.4	3.8	3.4	3.4	3.8	10.8	2.2
TM2D1	7.3	10.8	1.9	3.9	3.6	3.2	4.0	10.5	2.0
SYF2	8.7	8.5	2.5	4.4	3.9	4.2	4.8	7.5	2.8
WDTC1	7.5	10.3	2.5	3.6	3.5	3.2	4.1	10.4	2.0
TOR1B	5.9	11.5	1.6	3.9	2.9	4.9	3.9	10.3	2.2
FAM174A	7.6	10.2	2.9	4.5	4.6	2.7	4.2	8.2	2.3
FMN2	6.9	7.7	2.6	10.2	9.6	5.7	1.2	2.6	0.8
SHOX2	8.4	8.1	2.2	3.7	3.6	2.0	5.5	10.6	3.0
MOXD1	9.4	13.2	2.4	4.4	3.8	3.2	3.0	6.1	1.5
SLC8B1	7.0	9.4	1.9	7.5	7.0	5.5	2.4	5.2	1.2
TSNAX	7.1	10.8	1.9	3.8	2.8	3.3	3.7	11.7	2.0
HSPA14	7.6	11.0	1.8	3.7	2.7	4.2	3.4	10.5	2.1
ABHD16A	6.8	9.1	1.8	3.1	2.4	2.9	5.6	13.3	2.2
PMM1	7.2	11.6	2.6	4.0	3.3	2.6	4.2	9.6	2.0
SH3BP4	8.9	10.1	2.4	4.5	4.2	3.1	3.6	8.4	1.9
DGAT1	6.8	8.7	2.4	5.1	4.7	4.3	4.1	8.7	2.2
CUL7	7.5	9.7	2.2	4.1	3.5	3.2	4.4	10.6	1.9
NRM	6.8	8.6	1.7	6.0	4.3	3.3	4.0	9.9	2.3
INPP4B	8.3	11.9	2.1	2.6	2.2	3.2	3.6	11.0	2.0
WTAP	7.4	9.2	1.7	3.9	3.3	4.3	4.2	10.7	2.2
SEPW1	7.5	10.6	2.1	4.8	3.0	5.7	2.8	8.8	1.7
NOL3	9.5	11.8	2.9	3.6	2.7	2.0	3.9	8.3	2.2
CUL4B	7.6	10.7	1.3	3.3	2.0	3.0	3.8	12.9	2.5
VPS26A	7.4	12.0	1.6	3.7	3.1	5.3	3.0	9.1	1.8
KNSTRN	7.8	9.0	2.3	5.6	6.0	3.5	3.4	7.7	1.7
GOLGA1	7.7	10.1	1.9	2.6	2.9	2.2	4.9	12.0	2.7
SAP130	8.0	9.0	2.3	4.4	4.2	2.5	3.7	10.4	2.5
IRF2BP1	7.6	9.0	2.3	3.3	4.0	3.1	5.0	10.3	2.3
MAP3K12	8.0	13.1	2.3	3.2	3.0	2.3	3.9	9.3	1.8
CEP170B	7.5	8.7	2.4	4.6	4.8	3.0	4.3	9.3	2.3
SGPL1	6.3	11.3	1.8	3.0	2.6	2.9	3.5	13.6	1.9

SLC35D2	7.8	13.8	2.3	3.3	2.5	3.2	2.5	10.0	1.6
MYZAP	3.9	4.6	1.0	11.3	8.6	6.0	2.6	7.6	1.3
TTLL12	7.2	8.6	2.4	5.2	5.0	2.9	4.1	9.4	2.1
MESDC2	7.3	8.1	2.5	5.4	4.2	3.2	4.3	9.8	2.1
SURF6	7.8	9.2	2.9	3.8	3.8	2.7	4.9	9.8	2.1
TCEAL3	8.6	6.3	2.6	6.0	4.8	4.2	4.6	8.0	1.8
PNN	8.9	8.6	2.2	3.9	3.3	4.3	4.6	8.8	2.2
SPRY4	5.9	6.3	1.6	1.6	1.8	1.0	6.7	19.0	3.0
METAP2	7.5	8.5	2.3	4.3	4.0	3.8	4.8	9.3	2.4
LIME1	9.1	10.3	2.7	3.0	2.7	3.0	5.6	8.0	2.5
TMEM222	6.1	8.6	2.4	4.0	3.2	2.9	5.0	12.0	2.7
UCK2	8.2	10.0	3.0	4.6	4.1	3.3	3.5	8.5	1.6
RABIF	4.2	8.6	1.6	5.2	4.3	2.3	5.6	11.9	3.2
SLC35E2B	7.2	8.8	1.9	3.0	2.8	3.1	5.3	13.1	1.9
SECISBP2	7.3	9.7	2.0	3.3	2.9	3.2	4.2	11.9	2.3
WDR24	8.7	11.0	2.7	4.3	3.9	2.2	3.8	8.1	2.1
DNMT3A	6.4	10.5	3.6	2.3	1.7	3.9	5.3	11.8	1.2
CSNK2A3	3.6	18.0	0.9	1.6	1.5	6.0	2.0	11.7	1.4
IKBIP	6.2	8.4	1.4	4.1	3.0	3.4	5.2	12.1	2.9
SPRYD3	8.3	10.9	2.1	2.8	2.4	3.5	5.0	9.9	1.7
NDUFB6	6.0	9.4	2.0	4.8	3.6	4.6	4.2	10.3	1.8
TOX4	7.7	10.1	2.1	3.9	3.3	3.6	4.1	10.1	1.7
MCCC2	7.5	11.2	1.8	3.6	3.0	3.0	3.8	10.9	1.9
RBL2	7.3	10.8	1.8	3.7	2.9	3.4	3.7	11.2	1.7
LOC100506518	7.7	11.6	3.0	4.1	3.7	2.0	4.3	9.2	1.2
PIGO	8.1	11.4	2.1	3.6	2.6	3.4	3.5	10.2	1.7
COL27A1	8.8	9.1	2.4	3.9	3.2	2.1	4.8	9.8	2.4
SEC22C	6.6	12.3	2.0	4.3	2.4	2.9	4.1	10.9	1.3
FAM213A	7.6	13.7	1.7	5.2	4.6	3.8	2.4	6.6	1.1
NUAK1	7.8	9.9	2.7	4.8	4.3	3.1	3.2	8.9	1.8
C20orf96	8.8	11.2	2.5	2.9	2.8	1.2	4.4	10.5	2.3
DND1	7.0	8.5	2.0	4.0	4.1	2.7	5.1	10.7	2.4
MROH1	8.6	11.3	2.7	3.9	3.5	4.5	3.1	7.5	1.5
TRNQ	7.6	8.9	3.4	2.8	2.7	1.1	6.3	11.7	2.2
NQO2	8.1	10.7	1.9	3.8	4.3	3.0	3.7	9.5	1.5
CCDC94	8.9	7.7	3.4	4.1	4.3	3.3	4.2	7.8	2.8
NR1D1	8.8	9.0	2.9	3.1	3.0	1.6	5.3	10.2	2.6
TROVE2	7.4	10.6	1.9	3.1	2.3	3.8	3.5	11.9	2.2
GFOD2	6.4	8.8	2.3	4.1	3.8	2.1	5.2	11.1	2.5
PPP1R37	8.9	9.9	3.0	3.5	3.6	3.4	4.0	8.2	2.1
SAA1	4.9	5.9	1.1	12.2	10.8	9.6	0.6	1.1	0.3
SELO	8.6	11.3	2.8	3.0	3.3	2.9	4.2	8.2	2.2
COL4A1	7.2	9.2	2.5	2.7	1.9	2.0	5.3	13.7	2.0
INPP4A	7.8	10.0	2.2	3.4	3.3	4.6	3.7	9.5	1.9
AGPS	7.2	10.0	2.2	4.3	3.5	4.2	3.6	9.4	2.1
C17orf75	7.9	10.9	2.5	3.9	3.7	3.5	3.9	8.8	1.2
C2CD3	5.4	6.9	1.5	8.8	7.7	5.6	2.4	7.0	1.3

UVSSA	8.4	11.2	2.2	3.2	2.2	2.0	4.4	11.3	1.5
CDC42BPA	6.7	10.5	1.7	4.3	3.4	3.7	2.9	11.6	1.6
MAPK14	7.3	11.2	1.9	4.3	3.3	4.1	3.4	9.2	1.7
NAA38	8.0	7.8	1.9	4.3	4.3	3.0	4.8	9.0	3.2
VPS37B	7.2	8.0	3.0	3.0	4.4	2.6	4.7	10.6	2.7
MRPS5	7.0	9.2	1.9	5.5	4.7	3.1	3.2	9.7	2.0
CTNNBL1	8.1	10.0	2.1	4.2	3.7	3.5	3.4	9.6	1.7
FLJ42842	8.6	12.1	2.1	2.8	2.0	3.9	3.3	9.8	1.7
EFCAB14	6.9	10.7	1.6	3.8	2.7	3.9	3.4	10.8	2.4
ALG1	6.6	9.9	1.7	3.9	3.4	4.1	4.1	10.1	2.5
SCAMP1	7.6	12.1	2.0	3.9	3.5	3.0	2.6	9.4	2.0
TSFM	7.7	9.9	1.5	4.5	3.1	4.2	5.0	9.3	1.0
RILPL2	6.5	6.6	2.1	3.3	3.3	2.5	6.3	12.5	3.1
LINC00092	7.5	12.3	2.2	2.3	2.0	5.1	3.4	9.8	1.6
C14orf119	7.7	9.9	2.3	4.2	2.7	2.6	3.6	11.1	2.0
SNORA68	5.3	8.6	2.0	3.1	1.8	8.7	6.1	9.1	1.4
SECISBP2L	7.8	10.3	1.8	3.5	2.9	3.2	3.4	11.4	1.9
GOT1	7.1	9.8	2.0	4.9	4.5	3.4	2.7	10.0	1.7
NAB1	8.1	11.4	1.6	3.0	2.5	3.7	3.7	10.5	1.6
ING4	7.5	12.1	2.2	3.0	2.3	3.5	4.3	9.4	2.0
CELSR3	9.6	11.8	2.9	2.4	2.1	1.2	4.2	9.6	2.2
SIPA1	7.7	9.3	2.8	3.6	3.7	12.1	2.2	4.0	0.9
CSNK2A2	7.1	10.4	2.8	4.3	3.5	2.8	3.1	10.5	1.7
SLIRP	6.8	10.9	1.8	4.5	3.4	4.3	3.6	8.6	2.1
PRKD2	6.4	9.6	2.5	4.1	3.9	5.5	3.4	8.4	2.3
GYS1	8.9	10.5	3.1	4.4	4.6	3.1	3.1	6.7	1.7
THOC3	6.0	8.2	1.4	4.6	4.6	4.0	4.6	10.6	2.1
GYG1	7.8	11.3	2.3	3.7	3.1	2.5	3.1	10.2	2.0
CYB561A3	4.8	8.5	2.0	4.5	4.6	4.7	4.1	11.1	1.6
MMAB	8.2	10.6	2.6	3.6	2.9	2.4	4.1	9.3	2.3
ZC3H4	8.6	8.6	2.6	3.7	4.2	3.6	3.9	8.4	2.5
ADORA2B	8.7	13.6	3.0	3.3	2.9	2.2	3.4	7.2	1.7
ACOT13	8.2	12.2	2.0	4.3	4.2	1.9	2.5	9.2	1.5
SUV420H2	9.0	10.0	3.0	2.3	2.6	1.8	4.6	9.7	2.8
SMNDC1	7.9	11.5	1.2	3.3	2.6	3.5	3.9	10.3	1.6
PDPK1	7.1	11.3	1.7	2.9	2.5	2.9	4.0	11.2	2.3
OTUD7B	6.7	8.1	2.2	3.8	3.1	2.6	4.3	12.9	2.1
MEX3C	6.8	8.9	2.0	3.7	2.9	4.2	4.4	10.8	2.1
PMPCB	6.4	10.9	1.5	4.1	2.8	4.9	3.2	9.9	2.2
OGFOD3	8.2	10.8	2.7	3.7	3.8	2.8	4.3	7.8	1.9
LYPLA1	6.0	10.6	1.5	3.7	2.6	3.4	3.9	12.0	2.0
LOC101927063	7.7	12.2	2.0	2.7	2.2	4.8	2.9	10.1	1.4
LINC00941	7.5	12.2	2.1	3.3	2.6	4.5	2.9	9.5	1.3
MTA3	7.1	11.1	2.1	5.1	4.0	4.3	2.7	7.6	1.8
USP54	8.2	10.9	2.1	2.6	2.0	2.9	3.7	11.5	1.9
GGA3	7.7	9.5	2.2	3.5	3.3	3.0	4.2	9.9	2.5
NIP7	6.5	11.0	2.2	4.6	3.1	4.9	3.2	8.6	1.7

DOT1L	8.7	9.3	3.1	2.8	2.8	2.4	4.5	9.5	2.5
HIST2H2AB	6.2	9.2	2.1	5.9	7.1	6.6	2.7	5.2	0.7
DOCK5	7.8	10.4	2.1	4.0	3.4	3.4	3.0	9.9	1.7
NDFIP2	7.2	10.9	3.0	5.1	5.0	2.6	3.1	7.3	1.5
TUG1	7.7	11.0	1.8	2.7	2.3	2.5	3.6	11.9	2.2
PSMD10	7.0	14.3	1.6	3.4	1.9	3.6	3.2	9.8	1.1
MINOS1	7.2	9.9	1.3	3.5	2.0	6.9	4.5	8.8	1.5
TOX	8.7	9.2	2.8	4.5	4.5	3.4	3.3	7.4	2.0
ASH1L	7.6	11.1	1.6	3.1	2.1	3.4	3.4	11.6	1.6
TMEM185A	7.9	11.8	2.1	2.7	3.2	2.7	3.2	10.0	1.9
GPBP1	7.3	9.9	1.6	3.7	3.3	4.5	3.0	10.6	1.7
FCF1	7.5	11.6	2.0	2.9	2.3	5.0	2.8	9.9	1.7
RNF103	8.2	12.6	1.5	3.6	2.0	3.4	3.6	9.0	1.7
SNORD68	3.1	4.4	7.4	1.5	1.5	2.2	16.4	8.0	1.1
CASP4	9.8	15.5	2.6	2.6	2.4	3.2	2.1	6.3	1.0
TAX1BP3	7.6	9.7	3.1	4.0	3.5	3.1	5.0	8.5	0.9
FGD1	8.0	9.0	2.4	4.8	4.8	2.8	3.5	8.6	1.8
NUP210	8.2	9.9	2.7	4.4	3.8	5.3	3.0	6.8	1.3
FLJ31662	7.9	14.1	2.0	2.0	1.4	4.2	2.1	10.7	1.2
TRAK1	8.6	9.8	2.6	4.1	3.6	2.9	3.5	8.8	1.7
LOC101927796	7.6	10.2	2.1	3.0	2.3	7.1	3.3	8.6	1.5
SKA1	8.0	12.4	2.1	3.0	2.2	4.0	2.6	9.8	1.4
HERC2	7.5	10.4	2.1	3.3	2.6	2.9	3.6	11.4	1.8
UNC50	6.3	10.3	1.4	3.1	2.7	2.3	4.0	13.3	2.1
TMEM140	7.6	7.5	2.3	3.3	3.9	2.3	5.0	11.6	2.0
PUM1	7.5	10.0	2.1	3.6	3.1	3.6	3.6	10.1	1.8
LARP4	7.5	10.4	2.1	4.0	3.4	3.5	3.2	9.7	1.7
SH3PXD2A	8.2	9.4	2.7	2.8	2.8	1.8	4.2	11.6	2.0
PSPC1	6.6	9.2	2.1	3.8	3.4	4.8	3.5	9.7	2.3
AKTIP	7.6	8.3	1.8	3.6	2.8	2.9	4.1	12.1	2.2
ITSN1	9.5	11.3	3.0	4.4	3.8	3.1	2.4	6.5	1.4
LOC100287792	7.8	11.5	2.3	2.4	1.9	4.9	3.2	9.7	1.6
SLC26A6	8.2	12.0	2.1	2.2	1.7	1.7	4.6	11.0	2.0
LOC100505564	7.3	9.8	2.6	4.2	4.2	2.1	4.2	8.7	2.3
EIF4G3	8.5	8.4	2.2	4.4	4.2	3.2	2.6	10.3	1.6
EXOC6B	7.6	11.0	2.1	4.3	3.7	3.6	2.7	8.7	1.6
PTPRM	7.3	10.7	2.1	5.4	4.6	3.2	2.8	7.5	1.7
AP3B1	6.8	11.4	1.7	3.3	2.7	3.1	3.5	11.0	1.8
MOCS2	4.9	11.7	1.2	3.8	3.0	5.9	3.2	9.7	1.7
SMYD5	7.1	9.5	1.9	3.5	2.7	2.1	4.9	11.8	1.9
TSPAN15	10.4	12.2	2.7	4.4	4.4	3.3	1.9	4.8	1.2
NIPSNAP3A	7.1	12.3	1.8	4.6	3.2	3.3	2.9	8.2	1.8
HIPK2	6.5	6.0	2.4	3.3	3.3	2.1	5.6	12.9	3.2
RAI1	7.5	8.3	2.6	3.6	3.6	2.6	4.2	10.6	2.4
COPS2	6.8	10.4	1.6	3.9	3.1	3.7	3.5	10.2	2.2
KIF2A	7.3	11.0	1.7	4.3	3.0	4.2	2.8	9.1	1.8
ESD	7.4	12.7	1.7	4.0	2.4	3.7	3.1	9.3	1.0

C18orf8	7.7	10.1	2.0	3.6	3.3	3.2	3.5	10.0	1.9
TM4SF19-TCTEX1D2	13.2	15.5	3.4	3.8	3.7	1.6	0.9	2.7	0.5
OGG1	7.5	10.2	2.1	3.7	4.2	3.2	3.4	9.3	1.5
UQCRC2	6.4	10.2	1.7	5.0	2.9	4.7	3.5	9.0	1.7
KDSR	8.0	10.3	1.8	3.8	2.7	4.5	3.8	8.6	1.6
IGF2BP3	8.3	12.0	2.3	3.5	2.6	2.9	3.1	8.8	1.6
MRPL40	6.1	9.1	2.3	5.1	5.0	3.2	4.0	9.0	1.4
SRD5A1	7.3	10.2	2.4	4.0	4.1	3.0	4.1	8.1	1.8
CCDC122	7.6	12.4	1.9	2.5	2.1	4.8	2.5	10.0	1.3
PRPSAP1	8.2	10.7	2.0	4.5	3.9	3.2	3.7	7.6	1.2
PNPO	6.8	8.6	1.8	4.7	4.4	4.8	3.5	8.8	1.6
PAWR	8.5	8.4	3.0	5.8	5.4	3.5	2.6	6.0	1.9
TRIM11	8.0	10.0	2.9	3.4	3.8	2.5	3.9	8.4	2.1
LINC00973	8.4	13.3	1.6	2.4	1.4	1.5	3.4	10.9	1.9
NCKAP5L	7.3	8.2	2.4	3.6	3.3	2.4	4.8	10.5	2.4
LCMT1	6.5	9.9	2.0	4.1	2.7	3.9	3.8	10.3	1.8
PLXDC2	7.7	10.0	1.9	2.1	1.9	1.7	4.1	12.9	2.6
RPA3	5.4	7.9	1.6	5.9	5.7	4.1	4.5	8.9	1.1
GPATCH4	7.4	7.0	2.8	5.3	5.0	3.4	4.5	7.5	2.0
LYRM2	7.7	10.3	2.2	4.1	3.5	3.2	2.9	9.0	2.0
KPNA3	6.6	9.5	2.1	4.9	4.2	4.0	2.8	9.0	1.8
ALDH9A1	7.7	11.1	2.3	3.7	3.0	3.6	3.3	8.6	1.6
RBM25	7.7	8.2	2.3	4.4	3.3	4.0	4.2	8.7	2.1
RCOR3	8.4	10.2	1.8	3.7	3.3	2.2	3.8	9.4	2.1
LOC101928061	7.9	12.4	1.9	2.6	1.9	4.1	3.0	9.5	1.6
TOM1	7.2	7.2	2.4	3.4	3.5	3.7	4.5	10.9	2.0
OXSR1	8.1	11.2	2.1	4.1	3.2	3.4	2.6	8.7	1.6
BANP	7.0	8.0	2.8	4.2	3.6	3.0	4.8	8.9	2.6
MBTPS2	8.0	12.0	1.8	3.9	2.7	3.0	2.9	9.0	1.6
TRIM52	7.4	8.4	2.0	2.5	2.0	3.0	4.9	12.3	2.4
CCDC74A	9.0	9.7	3.5	6.0	5.1	3.1	2.4	4.9	1.2
TP53BP2	7.3	10.2	2.0	3.6	3.5	3.2	3.6	9.6	1.9
AEBP2	6.3	6.7	3.1	4.6	3.4	3.4	6.2	8.5	2.7
IKBKAP	6.9	10.7	1.8	2.9	2.5	2.8	3.9	11.4	1.8
RBM33	7.5	8.7	2.6	2.8	2.5	2.7	4.4	11.0	2.7
RBM5	7.3	9.0	1.7	3.4	3.2	3.6	3.9	10.7	1.9
C17orf70	6.9	9.5	2.3	4.5	4.1	3.1	4.1	8.3	1.9
FAM21C	7.8	10.9	1.7	3.0	3.5	1.7	4.4	10.5	1.2
ANXA8	8.2	8.9	2.4	8.9	9.0	5.9	0.4	0.6	0.3
LOC100505685	7.5	10.9	2.0	3.5	2.2	4.1	3.7	9.0	1.7
SNORA71B	4.6	5.6	2.5	1.8	2.2	18.2	3.9	5.2	0.5
BBC3	9.4	7.6	3.2	2.8	2.7	2.5	5.3	9.1	2.1
ADCY3	7.9	9.3	2.0	6.3	5.5	2.9	2.4	6.7	1.5
NPIPB5	7.3	14.0	1.5	2.2	1.5	2.2	2.6	11.7	1.5
CNOT1	7.0	10.6	1.7	3.1	2.7	3.5	3.2	10.8	1.8
KDM4A	7.3	10.4	2.0	2.7	2.4	2.5	3.8	11.6	2.0
MLXIP	7.6	8.3	3.2	3.7	3.0	4.4	3.3	7.9	3.2

CNOT8	6.8	11.1	1.5	3.3	2.5	3.2	3.8	11.0	1.2
ZNF219	7.0	8.4	2.3	5.0	5.0	2.5	4.3	7.9	2.1
LOC100505876	7.0	11.5	1.6	3.8	2.7	4.2	2.9	9.3	1.6
LIMS1	5.8	8.4	1.9	3.9	3.8	3.3	4.4	10.6	2.5
LOC101927084	7.9	12.0	2.3	2.4	1.7	4.2	2.8	10.0	1.3
LOC100133091	7.7	10.5	2.6	3.3	2.8	2.2	3.9	9.5	1.8
AACS	7.5	9.8	2.3	3.5	3.2	4.3	3.6	8.6	1.6
GINM1	7.5	10.0	1.9	4.6	4.3	4.0	3.3	7.3	1.7
FAM8A1	6.6	7.6	2.5	3.2	3.0	2.9	5.7	9.9	2.9
ANGEL2	7.1	10.8	1.9	3.4	2.5	3.8	3.1	10.0	1.8
ZNF324	7.6	8.6	2.3	3.4	2.8	1.9	5.3	10.2	2.4
LDOC1L	7.6	8.5	2.0	3.9	3.6	1.8	4.0	9.7	3.3
AZI2	6.6	9.7	1.7	3.2	3.2	3.3	3.5	10.9	2.3
NUP43	5.1	9.4	2.0	4.0	3.6	3.6	4.4	10.4	1.9
DUSP8	8.2	9.5	3.2	4.3	3.3	2.9	3.5	7.5	2.0
RRP9	7.8	9.2	2.2	3.3	2.8	2.3	4.8	10.0	2.0
NTN4	4.2	5.7	1.3	10.8	9.0	8.1	1.2	3.5	0.5
GTF2A2	7.5	8.8	2.3	5.2	3.7	3.4	2.7	9.2	1.6
EP400	7.9	8.9	2.8	2.8	2.7	2.8	3.8	10.5	2.2
NUDT16	7.4	9.1	2.5	4.0	3.8	1.9	4.2	9.3	2.2
CCNK	9.0	8.4	2.8	3.9	3.8	2.4	4.0	7.4	2.6
ASB6	6.8	9.1	2.2	3.5	3.9	3.4	4.5	9.4	1.5
PPP4R1	6.6	9.7	1.9	4.5	3.4	4.0	3.2	9.3	1.7
PDPR	6.6	9.8	1.7	3.4	2.9	2.6	3.7	11.6	1.9
ASPHD1	6.7	8.2	2.4	3.1	2.1	1.5	5.3	12.6	2.3
MRPS10	7.3	12.9	1.8	4.3	2.4	2.6	2.9	8.1	2.0
TUT1	8.2	9.7	2.0	2.9	2.7	2.1	4.3	10.2	2.1
LATS2	7.9	8.5	2.5	5.5	5.2	3.1	3.0	6.9	1.6
KAT5	7.8	8.7	1.9	3.3	2.8	2.8	4.3	10.3	2.3
ZFYVE1	8.2	10.2	2.1	2.9	2.5	2.7	3.6	9.8	2.2
SYNPO	7.1	7.3	2.5	8.5	7.9	3.3	2.1	4.2	1.3
CAT	6.5	7.9	1.9	4.0	3.2	4.1	4.0	10.5	2.1
TET3	7.4	8.0	2.4	1.9	1.7	2.2	4.9	13.1	2.5
CUEDC1	6.4	8.2	2.2	3.5	3.3	2.2	4.9	11.6	2.0
UCKL1-AS1	7.9	11.0	2.3	3.1	2.4	4.6	3.0	8.3	1.6
BRD8	7.5	9.6	1.7	2.9	2.4	2.3	4.5	11.4	1.9
NTAN1	6.9	9.8	2.0	5.9	4.8	4.0	2.5	6.6	1.6
EPS15	6.5	11.5	1.9	3.7	2.9	3.8	2.6	9.4	1.9
MAP3K13	7.1	10.3	1.9	3.4	2.4	4.2	3.2	10.1	1.5
HIST1H3E	7.2	10.1	2.0	3.7	2.7	6.1	3.7	7.3	1.4
PLA2G15	7.7	11.3	2.6	5.6	5.8	2.3	1.9	5.4	1.6
ADSL	6.6	9.8	1.7	3.7	3.6	3.9	3.5	9.0	2.2
CNTROB	7.3	9.9	2.1	3.8	3.4	3.0	3.9	8.7	1.9
LPHN2	6.7	9.9	1.6	3.7	3.1	3.1	3.4	10.3	2.2
ARHGEF10L	9.7	12.6	2.9	4.0	3.3	2.2	2.7	5.4	1.2
SIKE1	7.3	10.1	2.1	3.2	2.9	3.2	3.7	9.4	2.1
NXPE3	6.3	9.4	1.3	4.1	2.8	4.2	3.8	10.6	1.5

ZNF587	7.0	10.0	1.4	2.5	1.8	2.2	3.8	13.1	2.1
APOO	6.2	9.9	2.4	3.4	3.1	2.4	3.8	11.3	1.6
SH3PXD2B	6.5	7.7	2.4	3.6	3.8	3.0	4.3	10.2	2.3
CHCHD4	7.3	8.7	2.8	3.5	3.8	2.1	3.6	9.7	2.7
HLTF	6.3	10.9	1.6	4.0	2.8	3.5	3.0	10.0	1.8
TMEM131	7.3	9.4	2.3	3.5	3.0	3.5	3.2	9.7	2.1
EIF2S1	7.6	10.0	1.8	4.1	2.9	4.4	3.0	8.3	1.8
PROCR	9.5	11.7	3.6	4.8	4.3	2.1	2.4	4.5	1.1
PSKH1	7.6	8.5	1.9	4.1	3.8	2.8	4.0	9.0	2.3
PTBP3	7.8	9.6	1.6	4.2	3.6	4.4	2.4	8.8	1.5
USE1	8.0	8.0	2.4	3.7	3.3	3.2	4.9	8.3	2.2
TSG1	7.5	12.7	2.0	2.3	1.7	3.7	2.3	10.3	1.4
SARM1	8.2	10.2	2.7	3.7	3.0	2.0	3.7	8.4	1.9
PLD2	8.1	9.8	1.7	3.8	3.3	2.8	3.3	8.9	2.2
SUDS3	7.6	8.9	2.0	3.7	3.6	2.7	4.1	9.8	1.6
NRAS	6.3	12.1	1.5	4.2	2.7	4.7	2.6	8.5	1.2
METTL13	7.3	9.9	2.0	4.2	3.2	3.4	3.8	8.5	1.6
EIF2D	6.7	9.7	2.1	4.6	3.9	3.1	3.1	9.0	1.5
FKBP3	7.1	8.1	2.0	3.4	3.8	4.3	3.7	9.4	2.0
DNAJC13	7.1	11.2	1.6	3.8	3.0	3.6	2.8	9.2	1.6
SNAPC3	6.5	10.4	1.9	3.6	3.5	2.8	3.4	9.8	2.0
CACTIN	8.0	7.9	2.8	3.5	3.4	2.7	4.9	8.3	2.3
SIMC1	7.9	9.3	2.4	4.4	4.3	2.0	3.2	8.3	1.9
TMEM55A	8.5	10.5	2.1	3.7	2.6	2.3	3.5	8.9	1.8
CPT1A	6.4	8.3	1.9	2.9	2.4	3.0	4.6	11.9	2.5
SP2	7.9	8.7	2.4	3.5	3.1	2.2	4.1	9.7	2.1
SHOC2	7.1	9.3	1.5	3.6	2.9	3.0	3.4	10.9	2.0
PM20D2	7.4	10.9	2.2	4.1	4.1	3.0	2.7	7.9	1.4
CDKN2C	5.5	7.8	1.3	4.4	3.7	3.5	4.4	10.9	2.2
TNPO1	6.4	10.0	1.7	5.1	3.7	4.1	2.5	8.7	1.5
VRK3	7.5	9.8	1.9	3.2	3.0	2.9	3.7	10.0	1.7
ATRN	7.4	10.6	1.9	2.7	2.3	2.1	3.8	10.9	2.0
WDR46	6.5	8.4	2.0	4.1	3.6	3.8	4.3	9.1	1.9
SLC25A24	7.4	10.2	2.1	5.1	3.2	3.2	2.9	7.8	1.9
TARBP2	6.1	9.2	2.0	3.9	4.0	2.9	4.6	8.9	1.9
EMX2OS	7.3	11.5	2.0	2.5	2.0	4.5	2.9	9.5	1.5
BLOC1S4	6.6	7.9	3.1	4.2	3.5	2.4	4.6	9.6	1.7
AFF1	7.4	7.8	2.3	3.9	3.9	3.7	3.2	9.3	2.1
C2CD2L	7.7	7.8	2.5	2.8	3.0	2.8	4.4	9.8	2.8
BRE	6.2	10.6	1.6	4.2	4.3	3.1	2.7	9.4	1.2
LRRFIP2	7.8	10.2	2.4	5.0	4.2	4.1	2.3	6.1	1.4
ADRA1B	6.1	7.9	2.1	8.1	7.7	5.3	1.6	4.0	0.8
ATF2	7.5	8.2	1.3	4.4	3.6	2.9	4.0	8.5	3.0
NFATC2IP	7.4	8.9	2.2	5.3	4.4	3.5	3.0	7.5	1.3
CELSR1	9.6	11.6	3.5	3.5	3.5	2.2	2.5	5.9	1.2
LOC101929291	11.9	10.1	4.2	0.6	0.7	1.7	1.0	12.7	0.6
LOC101928738	7.3	11.6	2.0	2.7	2.0	5.3	2.5	8.9	1.3

SAPCD2	6.1	7.2	2.1	5.6	6.4	3.8	3.6	7.1	1.5
PPP3R1	6.1	9.2	1.9	3.9	2.7	4.9	2.9	10.1	1.7
RASSF7	7.0	10.4	2.4	5.5	5.3	4.3	2.2	5.5	0.8
LOC101928672	5.9	6.9	2.2	5.1	5.6	3.8	3.6	8.8	1.6
PCDH10	10.0	11.3	3.7	4.9	4.1	2.5	2.0	4.0	1.1
LINC00672	7.3	11.6	1.9	2.6	1.9	4.4	2.7	9.5	1.5
CERK	7.5	9.2	2.0	3.8	2.9	3.9	3.6	9.0	1.4
PPIL3	8.0	10.8	1.3	3.4	2.2	3.5	2.6	10.3	1.2
TBC1D9	6.9	10.0	2.2	3.3	2.7	3.7	3.5	9.2	1.9
C9orf114	6.7	8.8	2.4	3.5	3.3	3.4	4.4	8.8	2.0
TCTN1	5.9	10.1	2.1	3.7	4.5	2.8	3.5	9.3	1.6
CPZ	10.7	13.5	3.7	2.2	1.7	1.5	2.8	5.8	1.3
HERPUD2	6.8	10.0	1.6	4.0	3.4	4.1	2.6	8.8	1.9
SETD1B	6.9	7.8	2.4	3.0	2.9	3.0	4.9	9.8	2.6
NMNAT2	7.6	10.5	2.3	4.0	3.4	2.7	2.9	8.5	1.4
DHX37	7.8	8.7	2.1	3.3	3.3	2.8	4.2	8.9	2.1
MPC1	4.5	7.2	1.3	2.2	2.9	3.2	5.0	13.5	3.5
BBS2	6.9	10.5	1.7	4.3	3.8	3.3	3.1	8.4	1.4
HDAC11	6.2	7.6	2.3	5.3	6.0	4.0	3.2	7.3	1.3
CSRNP1	6.3	8.4	2.2	2.6	2.1	8.8	3.2	7.8	1.9
PDCD10	7.1	9.6	1.6	4.8	3.7	3.7	2.6	8.1	1.9
SLC39A9	6.8	9.5	2.0	3.1	2.1	2.6	4.1	11.1	2.0
PMAIP1	7.8	9.6	2.4	3.8	1.7	6.6	3.2	6.0	2.0
LIFR	10.1	16.1	2.1	2.3	2.0	1.8	1.7	6.1	0.9
ITGA2	4.9	7.3	1.1	2.5	1.9	2.5	4.1	16.3	2.5
FHOD1	7.2	9.3	2.2	3.0	2.9	2.6	4.5	9.3	2.0
SYNC	6.2	7.2	1.8	5.5	3.9	3.6	4.1	9.0	1.9
PROS1	7.9	13.3	1.9	4.0	2.4	3.2	2.4	7.1	1.1
LSG1	6.8	9.9	1.7	3.1	2.9	2.3	3.8	10.9	1.7
PDP2	7.1	9.4	2.2	4.0	3.4	1.9	3.9	9.3	1.9
TMEM200A	4.8	7.1	1.1	1.3	1.2	1.1	5.8	18.1	2.6
SLX1A	6.6	7.8	2.2	3.1	3.1	2.6	5.4	9.7	2.5
SLX1B	6.6	7.8	2.2	3.1	3.1	2.6	5.4	9.7	2.5
LINC00674	7.9	10.6	2.2	3.1	2.5	2.8	3.3	9.0	1.7
FAM208A	7.0	9.1	1.7	3.8	3.1	4.3	3.1	9.4	1.7
BAK1	7.6	10.1	2.3	3.5	3.2	2.3	3.8	8.5	1.8
LINC00476	6.1	9.3	2.2	2.8	2.4	3.9	4.0	10.9	1.6
OCEL1	7.5	8.7	2.7	4.5	3.7	2.7	3.8	7.5	2.1
ULK3	8.2	10.3	1.5	2.6	2.4	3.2	3.9	9.4	1.6
ZNF511	6.1	8.8	2.3	3.4	3.0	2.9	4.1	10.6	1.7
FUCA2	7.0	10.8	2.1	3.9	3.7	3.7	3.0	7.4	1.4
MTOR	7.0	9.2	2.0	3.2	2.9	2.5	3.4	10.9	1.8
ALS2	6.6	14.3	1.5	4.0	4.1	3.5	2.0	6.0	1.1
ERICH1	8.0	7.8	3.2	4.1	4.5	1.9	4.2	7.5	1.7
HINT3	6.4	8.9	2.8	4.5	3.5	2.9	3.6	8.3	2.1
GTF3C4	6.0	8.5	1.7	3.1	2.8	3.0	4.1	11.8	2.0
PRICKLE2-AS3	7.3	10.6	1.9	2.5	2.0	4.9	3.0	9.4	1.4

ACVR1	7.1	9.3	1.9	2.8	2.3	1.8	4.1	11.6	2.1
PRR24	6.1	7.4	1.8	2.6	2.7	1.8	5.0	12.9	2.6
LOC101928378	8.5	10.7	2.4	2.4	1.6	1.6	5.2	8.1	2.3
PNMA2	7.1	9.7	2.2	3.8	3.6	3.6	3.2	8.2	1.5
WDR11-AS1	7.6	11.8	1.9	2.6	1.9	4.6	2.7	8.4	1.4
SMIM15	6.7	9.2	1.4	4.7	3.7	4.1	3.5	8.0	1.6
ZZEF1	6.5	10.0	2.1	3.1	2.2	3.2	3.6	10.2	1.9
NUDT6	6.9	10.8	1.7	6.2	4.6	2.9	2.0	6.6	1.1
RNF13	5.8	10.0	1.0	3.0	2.3	2.5	4.0	12.2	2.2
SCOC	5.8	9.1	2.8	5.6	4.0	2.8	3.4	7.7	1.7
BTF3L4	5.0	9.3	1.4	4.5	2.1	3.5	3.2	11.8	2.1
CSE1L	6.4	9.9	1.5	5.1	4.0	4.6	2.4	7.7	1.3
ARMC9	7.4	10.1	2.2	4.6	3.6	2.9	2.7	7.7	1.7
MALT1	5.2	7.8	1.4	4.1	3.0	4.5	3.5	11.4	1.9
NSMAF	7.1	10.4	1.8	3.8	3.2	3.1	3.1	8.4	1.9
KIDINS220	7.0	9.1	1.8	3.1	3.0	3.2	3.2	10.5	1.9
PSMA3	5.7	12.2	1.6	4.1	2.2	3.7	2.9	8.8	1.5
THAP7	7.1	8.0	2.1	4.0	4.3	3.1	3.9	8.1	2.1
CCDC14	8.9	12.4	1.6	2.7	2.2	2.5	2.9	7.7	1.9
LOC101927714	8.5	8.0	2.6	2.7	2.8	1.3	5.0	9.6	2.2
C2orf74	5.9	7.7	2.1	4.5	2.7	3.1	3.7	10.1	2.9
FAM195A	6.3	7.4	2.3	3.4	3.3	3.5	5.6	8.7	2.2
ZDHHC18	6.7	8.1	2.3	3.9	3.8	3.3	3.4	9.0	2.1
MID1IP1	8.0	9.2	2.9	3.5	3.1	3.2	3.9	7.7	1.2
CDC26	8.2	6.3	1.5	4.0	2.1	4.6	5.2	8.5	2.3
TRAFD1	8.0	10.0	1.8	2.7	2.2	2.5	3.5	10.0	2.0
DPAGT1	6.4	7.7	1.9	3.2	2.7	2.1	4.0	12.3	2.3
MC1R	7.2	9.9	2.4	2.6	2.9	1.4	4.0	10.0	2.3
CDR2	6.6	7.6	2.1	3.5	3.1	2.0	4.5	11.2	2.2
PPM1M	6.2	9.1	1.8	3.6	3.0	5.5	3.4	8.6	1.4
DHX33	7.2	10.2	2.3	3.5	3.5	1.8	3.5	9.0	1.7
PDE4C	7.8	11.2	1.8	2.6	1.8	4.7	2.8	8.7	1.2
EIF1B	5.2	10.0	1.3	4.1	3.4	4.0	3.3	8.8	2.6
EFR3A	6.3	10.6	1.4	3.9	2.5	3.5	3.1	10.0	1.4
SLC44A1	6.2	8.6	1.4	5.0	3.9	3.3	3.0	9.5	1.8
PPP1R12A	7.9	9.1	1.6	3.8	3.3	3.7	2.9	8.9	1.5
STYXL1	7.3	10.2	1.7	4.0	3.5	2.5	2.8	9.1	1.4
BTN2A1	7.1	10.6	2.1	2.6	2.2	2.5	4.1	9.8	1.7
PARP4	7.3	10.0	1.8	3.2	2.6	2.8	3.2	9.9	1.6
ASH2L	6.9	9.4	1.8	4.1	3.5	3.8	3.2	8.5	1.4
CAPS	7.4	9.8	2.5	2.9	1.8	1.9	5.2	9.4	1.6
DNAJC30	7.4	7.6	2.1	4.7	5.5	2.7	3.5	7.1	2.0
NRG1	4.7	6.5	1.5	8.8	7.8	5.9	2.0	4.1	1.2
USP21	6.7	9.2	2.2	3.5	3.4	2.7	4.0	8.9	2.0
DHX40	6.5	9.5	1.5	3.6	2.3	2.9	3.5	11.0	1.8
CASK	5.3	7.7	1.4	3.5	2.7	2.8	3.7	13.1	2.2
LARS2-AS1	7.1	10.7	1.6	2.7	2.1	5.1	2.8	9.1	1.3

RNASET2	8.4	11.4	2.0	3.1	2.9	5.5	2.7	5.4	1.2
DYNC1LI1	6.3	9.3	2.0	4.1	3.2	3.8	3.2	9.0	1.6
SCARNA22	6.8	5.4	1.3	4.4	1.6	7.0	8.9	5.3	1.7
HIST1H2AD	6.8	9.6	2.8	4.7	4.8	4.4	2.7	5.6	1.2
ATP6V1D	6.8	10.6	1.6	5.0	3.0	4.0	2.1	7.9	1.5
CAMK2D	8.3	12.6	2.3	3.9	3.0	4.4	1.8	5.1	1.0
PWP1	6.7	8.6	1.8	3.8	3.4	3.3	3.9	9.1	1.8
HIST1H2BN	7.2	7.0	2.3	5.3	5.8	3.4	3.6	6.2	1.7
MRPS27	7.0	10.3	1.9	3.8	3.4	2.7	3.4	8.5	1.4
ARV1	5.2	7.7	1.6	3.7	3.2	2.1	5.0	12.0	2.1
SFSWAP	8.6	8.7	2.2	3.3	2.8	2.6	3.6	8.3	2.3
AHDC1	8.4	9.2	3.1	3.8	3.9	2.1	3.3	7.1	1.7
CRELD1	8.7	10.2	2.7	3.1	2.7	2.0	3.5	7.5	2.0
MAGT1	6.2	9.4	1.7	3.1	2.6	4.5	3.3	10.0	1.5
CCDC53	6.3	10.6	1.8	3.9	4.1	2.8	3.0	7.8	2.1
LINC00665	7.2	10.9	2.0	2.5	1.8	4.9	2.5	9.1	1.4
C19orf52	6.8	7.1	2.6	4.7	4.1	3.3	5.2	6.2	2.2
LOC101928032	10.6	10.6	2.3	1.2	1.8	0.8	2.7	10.6	1.8
NIPAL3	7.7	10.1	2.4	6.0	4.7	4.6	1.9	4.5	0.5
MAP3K7	5.8	10.1	1.6	3.8	3.1	3.1	3.0	10.3	1.5
LINC00547	7.6	12.8	2.1	2.1	1.6	3.2	2.3	9.6	1.1
CNNM3	5.6	8.2	1.9	3.0	2.3	2.4	4.6	12.0	2.3
CDIP1	8.2	8.3	2.9	3.3	4.0	2.8	3.3	7.6	1.9
TMEM97	7.4	10.8	2.3	5.3	4.7	2.7	1.8	5.8	1.5
ZNF282	7.7	8.1	2.4	3.6	3.9	2.4	4.0	8.0	2.0
GAS6-AS2	6.8	9.8	1.8	3.5	2.6	6.2	2.9	7.4	1.3
TRIM56	7.0	8.4	2.6	3.6	3.1	3.5	3.4	9.0	1.6
NAPG	6.8	10.1	1.7	4.4	2.8	4.1	2.5	8.2	1.5
TIMM10	5.8	9.2	2.3	3.6	2.8	3.0	4.7	7.9	2.9
TMEM55B	7.5	9.5	2.4	3.2	3.1	3.1	3.7	7.8	1.8
AAED1	7.0	10.4	2.4	4.2	3.7	3.0	2.3	7.4	1.8
CDYL	7.6	8.7	2.5	3.3	3.2	2.8	3.5	8.4	2.1
LINC00507	8.4	12.5	2.4	1.6	1.3	3.4	2.1	9.3	1.1
LOC101927903	7.7	10.2	3.3	2.2	2.1	1.6	4.0	8.9	2.1
ATP8B1	8.2	12.0	2.2	3.1	2.9	2.4	2.6	7.5	1.3
IPO4	6.1	7.5	1.8	4.0	3.6	2.8	4.3	10.1	2.0
MFAP3	6.6	9.8	1.7	3.8	2.6	2.3	3.1	10.9	1.4
SYNGAP1	8.0	8.8	2.0	3.3	2.4	2.0	4.0	9.5	2.1
MTUS1	8.8	12.5	1.9	2.6	2.2	1.8	2.8	7.9	1.7
RABEP1	7.8	9.0	1.9	4.0	3.0	3.2	3.3	8.4	1.5
ARVCF	8.6	10.9	3.0	3.1	3.2	2.5	3.3	6.2	1.4
PTPRJ	7.4	9.8	2.2	3.2	2.4	3.7	3.1	8.3	2.0
KBTBD2	6.0	10.6	1.5	3.5	2.0	3.5	2.8	10.7	1.6
PRR11	7.1	10.3	2.0	3.9	3.1	5.0	2.1	7.5	1.1
SBNO1	6.5	9.9	1.5	3.6	2.4	3.3	3.2	10.2	1.5
LOC101926943	6.3	9.3	1.4	2.1	1.8	3.4	3.7	12.3	1.8
ERI1	7.8	8.7	1.8	3.8	3.0	3.4	2.8	8.7	2.0

DCAF11	6.9	9.7	1.9	2.5	2.0	3.4	3.7	10.2	1.7
MRPL15	6.1	9.1	1.8	3.1	3.2	2.5	4.0	10.0	2.4
ZBTB46	6.8	9.2	2.3	3.8	3.2	1.7	3.8	9.3	1.9
RHOJ	5.0	7.4	1.9	3.5	3.3	2.2	4.5	12.6	1.8
ANAPC7	6.8	9.9	1.9	4.7	3.1	3.0	3.1	7.8	1.8
NUB1	7.1	8.8	1.8	2.9	2.6	2.6	3.6	10.1	2.4
TRIM37	6.8	10.0	1.5	4.3	3.0	3.8	2.7	8.6	1.5
LOC101927918	6.7	11.8	2.8	2.0	1.9	2.2	2.9	9.1	2.6
CALHM2	7.6	8.5	2.8	3.3	2.9	2.3	4.0	8.9	1.7
P4HA1	7.1	10.5	1.6	2.7	2.1	3.1	3.0	10.6	1.4
ZFP91-CNTF	6.6	7.8	2.0	3.7	3.3	3.0	4.1	9.2	2.2
LINC01012	7.4	10.8	1.8	1.9	1.6	4.8	2.7	9.5	1.4
FAR1	5.8	11.1	1.6	4.1	2.5	3.6	2.7	9.0	1.6
AFG3L2	6.4	10.2	1.8	3.2	2.8	3.3	3.3	9.5	1.4
DFFA	7.1	8.8	2.2	4.6	4.4	3.0	2.8	7.4	1.7
PIN4	6.1	7.7	2.4	4.1	3.6	3.1	4.1	8.7	2.0
PPM1D	6.6	9.3	1.9	4.0	3.3	3.7	3.1	8.4	1.5
FAM199X	6.9	9.3	2.2	3.8	3.2	2.3	3.2	9.1	2.0
VPS18	7.2	9.0	2.1	3.7	3.6	2.7	3.6	8.3	1.8
DR1	6.3	8.6	1.8	4.0	2.7	4.3	3.0	9.2	1.9
LOC648987	7.2	9.3	2.0	3.1	2.2	6.7	3.0	7.0	1.4
FAM127C	7.0	8.4	2.9	4.0	3.6	1.2	5.1	7.6	2.1
TDP2	6.2	9.2	1.7	3.5	3.6	2.8	4.3	9.1	1.6
OSBPL5	7.6	8.5	2.7	2.9	3.0	1.9	4.1	8.9	2.3
LOC100130451	7.8	13.0	2.3	1.9	1.2	3.0	1.9	9.5	1.3
SERGEF	6.5	10.3	1.4	3.9	2.4	3.8	3.0	9.1	1.4
TRIM46	8.5	9.6	2.1	4.4	3.2	2.9	2.8	6.6	1.6
URI1	6.0	9.0	1.9	3.6	2.9	3.3	3.4	9.6	2.1
CRELD2	6.6	8.6	2.0	3.9	4.2	2.5	4.5	7.8	1.6
LOC100128288	8.1	10.5	1.9	2.3	2.0	3.9	2.9	9.1	1.2
GMIP	6.7	9.1	2.0	3.1	3.0	6.0	3.3	7.1	1.5
SEMA4D	5.4	7.1	1.6	2.8	2.4	9.9	2.7	8.3	1.6
PLEKHO2	8.3	11.1	3.0	2.6	2.9	3.6	2.2	6.7	1.3
ZNF672	6.6	8.5	2.4	3.4	3.7	2.3	4.1	8.2	2.5
PPP6R2	7.1	8.2	2.1	3.6	3.3	3.1	3.9	8.5	1.9
NCOA3	6.6	9.6	1.9	3.0	2.5	4.4	3.0	9.1	1.6
KNOP1	8.8	7.3	2.6	4.5	4.6	3.4	3.1	6.1	1.3
FAM178A	8.0	7.5	2.4	4.1	3.3	1.8	4.9	7.3	2.2
KCTD3	7.6	10.3	1.7	3.9	2.9	2.9	2.6	8.2	1.5
MIIP	7.8	8.1	2.4	3.6	3.6	2.5	3.9	7.7	2.0
PLEKHG5	8.7	9.5	2.3	3.6	3.0	2.3	3.7	6.8	1.7
BOLA2B	6.8	7.1	2.2	5.0	5.1	1.7	4.3	7.0	2.5
IRF1	5.4	6.7	1.4	1.2	1.2	9.2	3.7	10.9	2.0
ITGBL1	6.8	9.3	1.8	6.3	5.1	4.9	2.2	4.5	0.8
RTN4R	7.9	9.0	2.5	2.5	2.6	1.4	4.2	9.4	2.1
SNX19	6.6	10.3	1.9	3.5	2.9	3.8	3.2	8.0	1.6
LOC339803	7.1	10.0	1.9	3.0	2.1	4.1	3.0	8.9	1.4

IGHM	0.2	0.4	0.1	0.2	0.1	40.0	0.1	0.4	0.1
SPTBN2	8.0	10.1	2.8	3.6	3.4	2.2	3.2	7.1	1.3
CPEB4	7.5	10.0	1.9	2.9	2.2	2.5	2.9	9.9	1.8
EXOSC5	5.9	9.9	1.6	2.6	2.4	2.3	4.8	10.3	1.7
SPDL1	9.6	14.8	2.6	3.0	2.6	2.5	1.4	3.9	1.0
GATA4	7.7	7.7	2.4	3.6	3.5	2.2	3.7	8.6	2.1
LOC101927752	6.7	10.8	2.3	4.1	3.1	2.6	3.2	7.0	1.7
SPHK2	6.5	7.3	2.2	3.9	3.6	2.5	4.2	8.4	2.9
LOC101928254	6.4	7.9	2.0	6.5	5.3	4.6	1.9	5.8	1.1
FLT3LG	6.5	6.6	2.6	2.7	3.7	5.6	3.2	8.4	2.1
NOC4L	6.3	7.2	2.0	3.2	3.5	3.0	4.2	9.8	2.3
IWS1	6.5	8.6	1.7	4.5	3.8	3.0	3.1	8.5	1.7
BLOC1S6	6.2	8.3	1.6	2.7	2.7	3.1	4.0	11.0	1.9
NIT2	6.7	11.4	1.5	3.8	3.2	4.4	2.4	6.8	1.4
CMTM4	6.8	7.7	1.7	3.0	2.6	1.7	5.3	10.7	2.1
CXXC1	5.3	7.0	1.5	2.9	3.1	3.8	4.9	10.7	2.3
KLF7	7.5	12.3	2.4	3.8	2.5	2.4	2.5	6.8	1.3
NUBP1	7.0	8.4	1.7	2.4	2.1	2.0	4.6	10.9	2.4
ELMO2	7.3	8.4	1.6	3.4	3.9	2.6	2.9	9.5	1.7
TMEM8B	8.1	10.1	2.6	3.6	2.9	2.0	3.2	7.6	1.4
LOC100287015	6.7	10.9	1.8	2.4	1.8	3.9	2.9	9.2	1.7
ABT1	6.2	8.2	2.7	4.3	3.5	3.1	3.8	8.1	1.7
SALL1	5.9	6.6	1.7	1.7	1.7	1.0	5.1	14.4	3.2
PTRH1	7.8	8.2	2.6	4.0	5.1	2.5	4.0	5.6	1.7
CFL2	6.5	10.2	1.5	4.0	2.7	3.0	3.2	8.8	1.6
LOC151475	6.9	10.7	2.0	2.2	1.7	4.2	2.7	9.6	1.4
CNPY4	6.3	8.7	2.1	5.3	4.4	2.7	3.5	6.8	1.6
COA6	6.8	8.5	2.7	3.3	2.7	5.0	4.2	6.2	1.9
CSGALNACT2	6.0	9.4	1.7	2.7	1.8	3.1	3.6	11.6	1.7
TP53I11	6.7	9.5	2.0	4.7	2.6	1.8	2.6	10.6	0.9
ZCCHC3	6.4	7.8	2.5	2.7	2.9	2.8	5.3	8.6	2.3
RGS14	6.5	7.4	1.8	2.8	2.5	5.6	4.1	9.0	1.7
TWF1	5.2	9.5	1.3	5.0	2.7	4.4	2.2	9.4	1.6
TRIM35	6.1	7.9	1.9	3.8	4.3	1.5	4.6	9.1	2.0
SCML1	7.2	10.9	1.7	3.0	2.5	1.5	3.5	9.1	1.9
FBXO21	6.6	8.8	2.4	3.5	3.5	3.7	3.0	8.4	1.4
DDT	2.1	7.9	4.8	10.4	5.6	1.1	1.2	3.3	4.8
REEP6	7.4	10.5	1.8	4.6	3.3	3.2	2.5	7.0	1.0
DCAF10	5.9	8.2	2.1	3.8	3.2	2.9	3.6	9.8	1.8
CWC15	7.0	10.6	2.1	3.3	2.8	2.3	2.7	9.2	1.3
PPIL1	5.2	8.7	1.5	4.7	4.0	4.4	3.3	8.0	1.5
CCBL1	7.7	10.9	2.3	3.1	2.9	2.3	3.0	7.9	1.2
HOXD11	7.9	10.6	2.4	4.0	3.0	2.7	2.6	6.8	1.2
HBS1L	6.9	9.4	1.5	4.3	3.2	4.4	2.4	7.5	1.6
SUMF1	5.5	9.4	1.5	5.8	3.7	4.2	2.8	6.8	1.6
SENP5	5.8	9.2	1.2	3.3	2.6	3.3	3.1	11.1	1.5
DAB2IP	5.7	6.2	1.9	3.5	3.9	2.1	4.8	10.5	2.7

PCGF5	5.5	9.5	2.0	3.8	3.6	3.8	2.7	8.5	1.7
SLC4A1AP	7.1	8.6	2.5	3.8	3.1	3.2	3.3	7.7	1.9
COMMD3-BMI1	5.7	9.0	1.4	3.0	2.4	2.7	3.4	11.5	2.1
MUM1	6.6	9.1	1.8	3.4	2.7	2.5	3.9	9.3	1.9
KAT6A	6.2	7.6	1.9	2.9	2.8	3.1	3.9	10.6	2.2
RMDN3	6.3	8.6	1.5	3.6	2.3	3.2	4.2	10.1	1.3
GULP1	5.9	7.4	0.7	5.4	4.5	4.3	3.3	8.2	1.5
POLR1A	7.3	9.0	2.3	3.1	2.9	2.6	3.2	8.9	1.7
CHN1	7.1	9.9	1.6	3.9	3.2	3.0	2.7	8.0	1.7
FTX	6.6	9.4	1.9	2.6	2.1	4.1	3.3	9.9	1.3
NCOA7	8.1	11.2	2.1	2.7	2.3	3.1	2.9	6.4	2.2
CTTNBP2NL	8.3	8.4	2.3	3.7	3.0	2.2	3.0	8.4	1.8
SNHG6	6.9	7.9	2.6	4.9	4.0	4.8	2.7	5.8	1.5
ZNF205	6.4	6.8	2.4	4.4	4.7	2.2	4.0	8.0	2.3
PSMD5	5.6	8.9	1.6	3.9	3.5	3.2	3.4	9.3	1.6
PHYH	7.0	8.8	2.6	3.7	2.5	2.7	3.4	8.7	1.6
SUGP1	6.6	7.5	2.4	3.3	3.4	2.5	4.2	8.5	2.5
C9orf9	7.7	8.6	2.5	4.7	4.3	1.4	3.4	6.5	1.7
TACC1	5.7	6.9	1.4	4.1	2.4	4.9	3.7	9.8	1.9
STARD4	6.6	12.5	1.6	2.7	2.3	3.1	2.9	8.3	1.0
CRTC1	7.7	7.6	3.0	2.4	2.7	1.5	4.6	8.8	2.5
MRPS36	6.9	7.3	2.5	4.1	3.5	2.4	4.1	8.5	1.6
KDM2B	7.8	8.4	2.5	3.0	2.4	3.9	3.3	8.0	1.7
DNAJC11	7.0	8.3	2.3	3.7	2.9	2.2	4.1	8.5	2.0
MORC3	5.4	8.1	1.2	3.7	2.9	3.3	3.5	10.8	2.0
CLDN12	8.2	10.7	2.2	3.0	3.3	2.2	3.0	6.9	1.4
SMOX	6.6	7.2	1.5	2.3	2.5	1.7	4.9	11.8	2.2
ASPSCR1	6.0	8.6	1.9	3.2	3.1	2.3	4.4	9.5	1.8
NRBF2	7.1	8.5	1.8	2.5	2.1	2.8	4.0	10.5	1.8
MBD4	5.7	9.4	1.1	3.3	2.8	3.0	3.2	10.2	2.3
TLK2	6.9	9.1	2.0	3.8	2.8	3.3	2.7	8.4	1.8
LOC646329	6.6	12.2	1.9	2.8	1.7	3.5	2.3	8.6	1.2
YIPF4	6.3	9.9	1.0	2.9	1.8	4.4	2.2	11.5	0.9
FAM129C	7.0	10.9	1.8	2.2	1.8	4.3	2.7	8.9	1.3
C19orf12	6.5	10.4	1.6	3.0	3.0	2.5	2.7	9.6	1.5
PELI3	6.7	8.0	2.3	3.8	3.9	2.6	3.5	8.4	1.8
KLF3	7.3	8.1	1.8	2.9	2.8	4.3	3.0	8.8	1.9
HPS6	6.5	9.3	2.0	3.2	3.3	3.4	3.5	7.9	1.6
ERAP2	4.9	9.1	1.2	3.1	2.5	2.9	3.3	11.6	2.0
SPICE1	7.5	10.9	1.9	2.6	1.9	2.5	2.7	9.4	1.5
EIF2B5	6.5	8.2	2.0	2.8	2.3	2.9	4.4	9.8	1.9
C1QTNF6	8.9	12.8	2.5	2.6	2.6	1.0	2.6	6.3	1.4
STX2	7.3	10.2	2.0	4.1	3.4	2.7	2.3	6.9	1.8
XPO5	7.1	9.9	1.8	3.1	2.4	2.6	2.8	9.7	1.4
SLC2A10	6.8	9.5	2.3	2.5	2.7	1.8	3.6	10.0	1.5
APIP	6.3	9.3	1.5	3.1	2.2	3.0	3.3	10.1	2.0
TADA2B	6.1	8.1	1.9	3.4	3.5	3.2	4.1	9.0	1.5

BCL2L12	6.1	7.8	1.6	4.8	4.8	3.4	3.3	7.5	1.5
CIRH1A	5.9	10.0	1.3	4.0	3.4	3.1	3.0	8.5	1.5
CUL1	6.4	9.2	1.9	3.9	2.7	3.3	3.1	8.3	1.8
RNF26	6.3	8.2	1.8	5.2	4.5	4.4	2.4	6.2	1.5
AKAP8	6.3	7.9	2.0	3.2	2.9	3.5	4.5	8.3	2.2
PP7080	6.9	8.7	2.6	3.8	3.3	2.0	3.1	8.6	1.5
FARP1	6.8	10.2	2.3	3.3	3.4	2.1	2.7	8.3	1.8
CRLS1	6.0	9.4	1.7	2.9	2.8	3.0	4.2	8.7	1.9
MCM3	5.1	6.8	1.4	5.8	4.8	3.9	3.2	8.0	1.5
UNG	6.1	8.8	1.5	4.7	3.7	3.3	3.4	7.6	1.5
ADCK2	7.3	9.6	2.1	4.7	3.2	3.6	2.9	6.2	1.1
RTKN	6.5	8.1	2.1	3.1	3.3	2.0	3.9	9.6	2.1
PSMB10	7.0	8.6	2.2	3.0	1.8	4.0	4.4	8.2	1.3
TCEAL8	6.3	7.6	2.3	4.1	3.8	2.9	3.6	8.0	2.1
ARMC5	6.9	8.6	2.3	2.2	2.4	2.0	4.5	9.3	2.3
MAD1L1	7.7	8.4	2.5	3.1	3.2	2.1	4.1	7.5	1.8
ISY1	0.7	11.5	2.7	4.8	1.6	5.1	0.9	10.8	2.5
SEC23IP	6.1	7.3	1.6	2.6	2.3	2.0	3.9	12.7	2.1
TPST1	5.6	8.0	1.1	3.1	2.4	2.9	3.8	11.5	2.0
FZD6	8.1	9.8	1.5	3.5	3.2	2.7	2.4	8.1	1.1
ZSWIM7	5.5	7.4	1.9	3.0	2.9	2.7	4.6	10.7	1.8
PIGG	4.9	8.1	1.4	3.3	2.4	2.6	4.5	11.5	1.7
PAM16	3.9	8.7	1.7	3.8	3.3	3.1	4.0	10.4	1.6
EME2	8.0	9.3	2.3	3.9	3.0	1.8	3.7	6.6	1.7
RNF123	7.5	8.8	2.0	3.5	2.7	2.7	3.5	8.3	1.4
LINC01000	7.2	9.7	2.0	2.6	2.2	2.1	3.2	9.7	1.6
ENOPH1	6.8	9.1	2.3	4.5	2.7	3.2	2.9	7.8	1.1
LOC100288152	8.1	9.3	2.1	3.4	2.3	2.1	3.8	7.8	1.5
PRKCD	7.4	8.6	2.3	3.6	3.3	3.0	3.3	7.0	1.8
ADCY9	6.8	9.0	2.2	4.1	3.9	2.7	2.9	7.3	1.6
MGAT2	6.5	9.3	2.1	3.7	3.4	3.1	3.5	7.2	1.5
S100A2	5.1	6.0	0.9	9.1	6.2	4.9	2.8	4.9	0.5
NFS1	6.9	9.4	1.7	3.3	2.8	2.9	2.7	8.9	1.8
MTG1	5.6	8.3	1.4	2.9	2.8	4.1	3.7	10.1	1.4
NOP14	6.5	8.2	2.1	3.6	3.2	2.4	4.0	8.3	2.0
SKA2	6.2	8.7	1.2	5.1	2.7	5.5	2.1	7.7	1.1
LOC100506990	7.3	11.7	1.7	2.8	2.3	2.4	2.5	8.5	1.2
SNW1	6.8	7.6	1.6	3.3	2.6	4.2	3.4	9.1	1.8
FIZ1	6.8	8.2	2.1	2.8	3.1	2.5	3.9	8.2	2.7
MOK	6.6	11.3	1.6	2.6	2.1	2.8	2.8	9.2	1.4
PCMTD2	7.0	9.2	1.6	3.3	2.6	3.2	3.2	8.5	1.7
CDK20	6.2	8.4	1.8	2.5	2.8	0.9	4.3	10.8	2.6
COG7	7.3	9.8	1.8	2.6	2.2	1.6	3.7	9.2	2.1
ZNF71	6.7	8.5	2.1	3.7	3.5	2.1	3.5	8.2	1.9
ETV4	7.7	9.7	2.9	3.2	2.9	2.3	2.9	6.7	1.9
KRR1	5.5	7.5	1.3	3.1	2.7	3.0	3.7	11.4	2.1
MARK4	7.1	7.7	2.7	3.6	3.4	2.5	3.5	7.6	2.1

FITM2	6.5	7.7	1.7	3.0	3.7	2.5	4.1	9.4	1.8
LOC101929130	6.8	10.8	1.7	2.3	1.8	4.9	2.4	8.3	1.3
TAF13	3.6	7.3	1.9	4.7	2.5	3.8	3.9	12.0	0.5
LTB4R	8.4	11.0	2.2	1.9	1.4	1.9	4.0	7.8	1.6
CACNB1	7.7	10.3	2.0	3.8	3.2	2.2	2.7	7.2	1.1
CORO2A	8.5	9.1	2.5	4.6	4.2	2.7	2.2	5.1	1.3
C20orf112	7.7	7.4	2.5	3.2	3.1	1.9	3.7	8.2	2.5
MAPK11	6.7	9.1	2.0	3.8	3.3	2.6	3.4	7.5	1.7
CNOT6	6.4	9.7	1.5	2.8	2.5	3.1	2.9	9.4	1.7
PEX5	6.6	8.7	1.7	3.3	2.8	2.9	3.8	8.8	1.7
PSEN2	7.5	10.8	2.2	3.3	2.3	1.7	3.5	7.8	1.2
CRHR1-IT1	6.4	10.9	1.8	2.3	1.8	3.4	2.8	9.3	1.3
CD3EAP	6.7	9.5	2.1	2.5	2.4	1.7	4.3	8.9	2.0
HES6	7.4	8.5	2.3	3.8	3.7	3.2	3.6	6.4	1.2
PRPS2	6.6	8.9	1.7	5.0	4.0	4.1	2.4	6.6	0.8
SLC29A1	5.7	7.9	1.7	4.4	4.2	2.3	3.6	8.1	2.0
PHF10	5.4	7.3	1.7	2.8	2.4	3.3	3.8	11.3	2.2
ZNF276	7.9	9.8	2.4	2.5	1.8	2.0	4.4	7.6	1.6
RAB3B	6.6	7.2	2.1	6.4	6.0	3.6	1.5	5.9	0.9
ABCC5	5.3	8.6	0.9	3.8	2.6	2.8	4.1	11.3	0.7
EML2	8.6	10.9	2.8	2.8	2.4	2.0	3.0	6.4	1.2
IKZF3	6.2	9.6	1.6	2.2	1.6	7.6	2.3	7.8	1.1
SIX5	7.2	8.1	2.4	2.3	2.8	1.2	4.6	8.9	2.4
COMMD2	7.4	10.7	1.4	3.3	2.1	2.8	3.3	7.9	1.1
PPA2	5.8	9.0	1.8	3.8	3.3	2.0	3.5	9.5	1.4
TANK	6.9	8.2	1.6	3.9	3.0	3.5	2.8	8.2	1.8
ECD	7.1	9.3	1.9	2.8	3.1	2.7	2.8	8.6	1.8
TRIM32	5.9	9.3	1.5	3.4	3.3	2.5	3.4	9.3	1.5
PTCD3	6.7	9.9	1.4	4.1	3.0	2.8	2.8	7.9	1.4
RANBP10	7.7	9.1	2.7	3.4	2.9	2.3	3.3	6.9	1.7
MTMR12	6.6	9.7	1.8	3.1	3.2	2.5	2.7	8.6	1.9
FBXO17	7.0	9.8	2.3	3.2	2.9	2.6	3.1	7.5	1.6
LOC101927512	7.7	11.1	1.8	2.3	1.5	4.3	2.2	7.6	1.4
LOC729652	7.3	10.6	1.8	2.0	1.7	3.6	2.7	8.9	1.3
C10orf54	9.5	8.4	2.8	4.6	4.3	5.5	1.5	2.6	0.7
HDAC9	10.8	13.5	2.9	2.5	3.0	1.9	1.4	3.4	0.5
CSTF1	6.2	9.4	1.7	3.8	3.5	3.0	3.0	7.8	1.5
GTDC2	6.5	7.5	1.9	4.7	4.7	2.2	3.3	7.2	1.8
BCL3	5.5	5.9	1.9	3.5	3.1	3.3	4.4	9.5	2.6
TAB3	6.9	9.1	1.9	3.7	3.0	2.3	2.8	8.3	1.9
MRPL16	6.0	8.2	1.9	4.7	3.6	3.1	3.0	7.4	1.9
SLC5A6	6.4	8.8	1.8	3.4	2.6	2.2	3.7	9.4	1.6
PHF19	4.9	7.0	1.5	6.7	6.7	4.3	1.9	6.1	0.6
PNO1	6.0	6.9	2.1	4.2	3.3	2.7	3.5	8.3	2.7
PIP4K2C	6.7	9.2	1.7	4.0	3.0	3.9	2.7	7.3	1.3
BLMH	6.7	9.3	1.9	3.1	2.2	3.1	2.9	9.0	1.4
OGFOD2	6.4	8.4	2.0	2.9	3.0	2.4	4.5	9.1	1.1

UMPS	6.9	8.6	1.8	4.1	3.3	3.8	2.7	7.2	1.3
ACAP2	6.2	10.3	1.6	2.6	2.0	3.3	2.8	9.0	2.0
DUS3L	6.6	8.0	1.8	3.1	2.7	3.1	4.3	8.0	2.1
KRI1	6.8	6.2	2.2	3.2	3.5	2.1	4.9	8.7	2.0
LOC101927469	6.5	10.3	1.6	2.2	0.9	1.2	4.3	11.6	1.1
PGM3	5.4	8.5	1.2	2.4	1.8	2.3	4.0	12.6	1.5
MRPS18C	6.8	8.4	1.3	4.0	2.2	3.5	4.0	8.2	1.3
LINC00114	7.6	11.1	1.6	2.2	1.4	3.1	2.4	8.7	1.5
PTPLA	7.2	9.0	3.5	5.1	4.3	2.5	2.3	4.4	1.4
LOC100129034	7.1	9.0	2.3	3.5	3.2	2.2	2.8	7.8	1.6
SART3	6.2	7.9	2.0	3.4	2.9	3.3	4.2	8.0	1.8
KCTD18	6.6	9.5	1.9	3.6	2.7	2.6	3.0	7.9	1.8
HLA-G	0.5	0.7	0.2	0.1	0.2	36.8	0.3	0.6	0.2
KIAA0319L	6.3	9.5	1.9	3.0	2.2	3.4	3.1	8.7	1.6
FDXR	5.8	7.9	1.8	4.0	3.7	2.8	3.9	8.1	1.7
PMS2	7.7	10.2	1.9	3.1	2.7	2.4	2.8	7.4	1.4
DEPTOR	2.7	3.5	0.6	0.9	0.7	0.4	6.9	20.2	3.6
PIM1	8.1	9.0	2.4	5.1	4.1	5.0	1.7	3.4	0.8
FAM63A	5.1	5.4	1.5	2.5	2.4	1.9	5.0	13.0	2.8
RABGGTA	5.6	8.4	2.3	3.0	3.1	2.3	3.5	9.4	1.9
ALDH3B1	8.9	10.5	3.0	3.5	4.4	2.3	1.7	4.3	1.0
TMEM223	6.3	8.3	2.3	3.3	3.3	2.2	4.0	8.1	2.0
GGA2	5.1	8.1	1.7	3.6	2.7	4.4	3.1	9.2	1.5
UGP2	6.1	9.6	1.5	4.3	2.8	4.3	2.2	7.6	1.2
RLIM	6.3	8.9	1.3	3.0	2.5	3.6	3.0	9.3	1.6
MED14	6.2	8.5	1.7	3.4	3.3	3.1	2.7	8.9	1.9
HOXC13	6.6	8.2	2.1	3.6	3.0	2.0	3.5	8.0	2.4
PCDHB5	5.9	8.2	1.5	2.5	2.5	1.9	4.0	10.5	2.5
EPN2	6.4	7.5	1.9	3.2	3.1	2.3	3.9	9.8	1.4
ARID1A	6.7	7.4	2.2	3.1	3.1	4.5	3.1	7.8	1.6
CCDC107	6.8	8.9	2.9	3.4	3.5	2.4	3.8	5.7	2.1
ZNF146	5.9	9.5	1.5	3.0	1.9	3.1	2.6	10.6	1.5
PAK1	7.5	9.7	1.8	3.5	3.4	3.4	2.4	6.4	1.4
STRIP1	6.0	8.2	1.8	3.3	2.8	2.9	3.7	9.0	1.8
FAF1	5.7	10.9	1.5	3.8	2.4	2.5	2.7	8.7	1.3
CNTNAP1	7.6	9.4	1.9	3.1	3.0	2.0	2.9	8.0	1.5
TCTN3	6.3	7.5	1.4	3.4	3.5	2.4	3.3	9.7	1.9
ATXN2	7.2	7.4	2.4	3.8	3.8	2.8	2.8	7.4	1.8
WNT9A	3.6	5.3	1.6	2.0	2.0	1.5	6.0	14.9	2.5
BRCC3	6.3	8.2	1.9	3.7	2.6	3.5	3.1	8.7	1.3
AKIRIN2	7.2	7.0	2.2	2.9	2.9	3.1	3.5	8.1	2.7
MFN1	6.0	9.0	1.4	3.0	2.4	3.0	2.8	9.9	1.8
ZNF629	7.4	7.4	2.4	3.3	3.6	2.1	3.1	7.8	2.3
PNISR	6.9	7.6	1.9	3.2	2.2	3.7	3.2	8.3	2.4
TMEM237	6.8	10.7	1.7	3.1	2.5	2.7	2.3	8.2	1.3
PPP1R3F	6.8	7.6	1.9	3.6	3.5	2.3	4.1	7.4	2.1
PODXL2	7.3	8.9	2.4	2.2	2.1	1.3	4.1	9.5	1.6

VAV2	6.6	7.9	2.2	3.2	2.8	2.7	3.6	9.0	1.5
RFX1	6.0	6.4	2.0	3.2	3.2	2.5	4.4	9.1	2.3
NPIPB3	8.3	9.0	2.4	2.1	2.2	0.9	3.0	9.9	1.6
HECTD1	6.3	9.6	1.4	3.4	2.4	3.4	2.7	8.8	1.3
CDC14B	7.2	9.9	1.9	3.1	2.5	3.6	2.3	7.5	1.3
ARMC4	7.1	9.8	1.9	3.8	2.9	3.4	2.2	7.3	1.1
ABCE1	6.3	9.3	1.2	3.2	2.2	2.6	2.7	10.0	1.7
GRWD1	6.7	7.8	1.7	3.4	3.1	3.1	3.8	8.0	1.8
MRRF	6.7	8.8	1.7	3.2	2.9	2.3	2.9	9.2	1.6
SDR39U1	6.6	8.0	1.3	3.2	3.0	2.9	4.0	8.4	1.9
PER1	6.1	6.3	1.8	2.0	1.8	9.2	3.1	7.1	1.8
HMGB3	7.1	7.5	1.4	4.2	3.6	4.0	2.6	6.6	2.0
TPD52	6.8	6.6	1.4	3.4	3.3	3.3	4.4	8.4	1.5
KIF13A	6.5	8.2	1.5	4.8	2.8	3.1	2.8	8.5	1.0
IDE	7.3	10.0	1.5	2.9	2.5	2.8	2.7	8.1	1.6
UBLCP1	5.5	9.7	1.5	3.5	2.8	2.7	2.5	9.2	1.8
FBXO25	6.8	8.1	1.7	3.7	2.9	3.1	2.7	8.1	2.1
FOXJ3	6.1	6.9	1.8	2.8	2.7	2.2	4.1	10.3	2.3
ELL	6.9	8.1	2.6	2.7	3.2	2.9	3.6	7.0	2.1
MFSD5	6.2	9.5	1.4	3.8	2.9	2.3	3.5	8.1	1.5
TXNDC9	5.1	10.3	1.4	3.9	2.4	3.5	2.6	8.4	1.7
ARMCX6	6.2	9.8	1.6	3.8	3.5	2.6	3.3	6.6	1.7
TAF8	6.4	8.0	2.0	3.1	2.7	1.7	2.6	10.0	2.6
RBPMS2	6.6	6.4	2.0	4.2	5.1	2.9	3.1	6.7	2.0
LDLRAD3	5.4	6.5	1.8	2.9	3.4	1.4	4.8	10.6	2.4
LOC101928099	7.1	9.2	1.8	2.4	2.1	4.3	2.8	7.9	1.5
DCUN1D1	6.6	9.8	1.4	3.2	2.5	2.7	2.3	9.0	1.8
FBXO3	6.0	10.4	1.6	2.8	2.2	2.3	3.0	9.3	1.5
PIGC	6.0	8.3	1.5	3.1	2.0	2.5	3.5	10.8	1.4
DNMBP	6.2	8.9	2.0	2.6	2.6	2.4	3.0	9.6	1.7
DONSON	6.0	8.4	1.5	5.0	4.8	3.9	1.9	6.4	1.2
CMTM6	4.5	8.9	1.2	3.1	2.0	3.3	3.7	10.9	1.5
DPH3	6.1	9.6	1.5	4.3	2.9	2.5	2.4	8.2	1.6
WDR77	6.3	8.6	1.2	4.3	3.2	2.8	2.9	8.5	1.4
LAS1L	5.9	9.0	2.1	3.3	3.1	2.7	3.6	7.9	1.5
NADK2	3.9	5.2	1.0	3.3	2.2	3.0	4.8	13.9	1.8
ARID1B	5.6	6.4	2.1	2.7	3.0	3.1	4.1	10.1	2.1
SNRPA1	5.3	6.7	1.5	3.4	3.0	5.4	3.8	8.5	1.4
NCS1	6.6	10.2	1.8	3.5	3.2	4.0	2.6	6.1	1.0
IFI16	7.6	8.8	1.9	2.6	2.5	4.3	2.3	7.1	1.8
CERS1	7.4	9.8	2.2	3.7	3.3	2.4	2.6	6.3	1.4
SARS2	7.1	7.7	2.0	2.7	3.0	2.4	3.8	8.2	2.1
ANTXR2	7.4	10.2	1.7	2.5	1.9	3.0	2.8	8.1	1.4
SRGAP2	7.4	8.1	2.2	3.0	3.0	2.7	3.1	7.8	1.6
KLHL21	6.1	7.8	2.1	2.9	3.2	2.5	4.1	8.6	1.7
C10orf35	6.1	10.0	1.4	3.5	3.6	2.4	3.4	7.5	1.0
ZNF264	6.6	9.8	1.8	2.4	1.9	3.4	2.7	9.1	1.3

SNX4	6.8	8.5	2.2	3.0	2.7	2.6	3.1	8.2	1.8
DDX18	5.7	8.3	1.4	3.1	1.8	2.9	3.3	10.4	2.0
MAP6	9.4	12.9	3.4	2.6	2.5	1.6	1.5	4.0	1.0
AQP1	7.1	9.5	2.5	2.9	2.9	1.6	3.7	7.1	1.5
ANKS6	6.7	7.2	1.8	3.9	3.6	2.6	3.1	8.7	1.4
SNORD3C	9.0	8.9	2.6	0.6	1.0	4.6	6.4	5.5	0.3
YRDC	5.3	8.4	2.2	2.7	3.3	2.1	4.5	8.3	2.0
PDZD4	8.0	9.4	2.7	3.1	3.0	2.2	3.2	5.7	1.6
ABRACL	6.8	9.6	1.8	4.5	3.1	5.2	2.2	5.0	0.6
SERPINB1	6.5	10.0	1.7	3.0	2.0	4.5	2.1	7.7	1.2
ACAT1	6.1	9.3	2.4	3.3	3.1	2.8	2.6	7.9	1.3
TMEM255B	4.6	5.4	1.1	4.4	4.5	3.3	4.6	9.1	1.9
PPOX	5.7	8.5	1.6	3.0	2.8	1.6	4.2	9.6	1.9
ACTA2	7.9	8.8	2.1	4.3	4.4	2.1	2.4	5.7	1.3
LOC101927257	6.8	9.7	1.9	2.4	1.6	4.6	2.3	8.3	1.3
CCDC25	6.6	8.3	1.8	5.3	3.2	3.2	2.9	6.4	1.2
EPDR1	6.8	9.9	1.8	4.1	4.4	3.2	1.9	5.3	1.5
FTO	5.4	7.7	1.6	3.3	2.7	2.6	3.3	10.6	1.5
LRRC14	7.4	9.8	2.0	3.2	2.8	1.5	3.6	7.1	1.5
DNAJC4	7.3	7.5	2.3	3.1	2.6	1.6	3.8	8.1	2.5
ANKRD54	5.1	7.4	1.6	3.5	3.0	2.0	4.3	9.8	2.0
NUTM2A-AS1	8.2	7.7	3.0	3.4	3.1	2.0	3.2	6.7	1.6
COPS4	6.2	10.6	1.6	3.2	2.3	3.7	2.7	7.6	0.9
SMN1	6.7	9.0	1.8	2.9	2.9	2.6	3.2	8.0	1.7
LOC101928684	6.8	9.6	2.0	2.7	1.7	5.0	2.7	7.0	1.2
FUZ	6.8	9.1	2.1	3.7	3.2	3.0	3.3	6.4	1.1
KIAA0368	6.1	9.1	1.5	3.5	2.7	3.0	2.7	8.5	1.6
KBTBD4	6.4	7.7	1.7	3.6	2.9	3.0	3.5	8.4	1.5
H6PD	6.4	8.3	2.2	3.1	3.4	2.5	3.1	8.1	1.8
CACFD1	5.4	9.3	1.8	2.3	2.1	3.2	4.7	8.4	1.5
MED19	6.7	7.6	2.2	2.4	2.2	1.3	3.9	9.7	2.5
PRKAA1	5.2	9.0	1.3	2.9	2.6	3.8	2.8	9.5	1.6
HCG18	6.0	9.5	1.7	2.6	1.8	2.7	2.9	10.3	1.2
LOC643770	6.7	10.3	1.7	2.1	1.2	3.7	2.7	9.1	1.2
C22orf23	7.5	10.0	1.9	3.7	3.0	2.0	3.1	6.5	0.9
PTPN9	7.4	9.0	1.7	3.3	2.6	2.6	2.5	8.1	1.5
TDRKH	6.0	8.8	1.3	3.1	3.1	2.4	2.9	9.4	1.7
FN3KRP	5.9	8.1	1.4	5.5	4.6	4.1	2.1	5.8	1.2
URGCP	5.9	8.4	1.9	3.9	3.6	4.0	2.6	6.9	1.4
TRIM16L	6.0	7.6	1.3	3.7	4.0	3.8	3.3	7.5	1.6
MGAT5B	6.8	9.0	2.2	2.4	2.6	1.4	3.8	8.5	1.9
RBM24	7.9	8.7	2.5	3.0	3.9	1.8	2.6	6.5	1.7
MRPS33	5.7	9.4	1.5	5.2	3.1	3.0	2.1	7.4	1.3
KCTD17	6.7	7.8	1.9	3.9	3.0	2.1	4.1	7.3	1.7
ADD3	4.6	7.2	1.2	2.2	2.1	3.9	3.4	12.0	2.0
R3HDM1	6.6	7.2	1.6	3.3	3.2	2.2	3.3	9.2	2.0
UXS1	4.9	7.5	1.9	4.9	3.7	3.5	2.9	7.6	1.5

GOLPH3L	5.3	7.3	1.4	3.2	2.6	3.0	3.9	10.3	1.6
HLA-DRA	0.2	0.4	0.1	0.1	0.1	37.3	0.0	0.3	0.0
USP20	6.4	8.1	1.8	2.3	2.2	2.3	4.3	9.2	2.0
GGT7	8.8	10.9	2.4	2.4	2.2	1.8	2.7	5.9	1.4
STX3	7.0	9.3	1.9	3.6	2.8	3.7	2.4	6.4	1.4
OSGEPL1-AS1	6.3	10.3	1.8	2.2	1.4	3.3	3.1	8.8	1.4
GLI4	7.7	9.1	2.6	2.0	2.3	2.1	3.5	7.5	1.8
LGALS3	8.1	10.5	2.3	3.2	3.0	2.7	2.6	5.0	1.2
FSTL5	5.5	9.0	1.3	3.6	2.4	2.5	3.1	9.4	1.6
F8A3	5.3	7.3	2.1	3.7	3.0	2.4	4.3	8.4	2.0
F8A2	5.3	7.3	2.1	3.7	3.0	2.4	4.3	8.4	2.0
F8A1	5.3	7.3	2.1	3.7	3.0	2.4	4.3	8.4	2.0
FAM160B1	6.5	10.0	1.3	3.0	2.4	2.3	2.7	8.7	1.5
TJAP1	6.8	8.5	2.0	2.2	2.2	2.3	3.9	8.5	2.2
EMC1	6.6	8.8	1.8	2.9	2.4	2.1	3.2	9.0	1.6
LOC101928451	6.8	9.6	1.2	2.6	1.7	2.5	3.7	8.8	1.7
CHKB-CPT1B	8.5	10.6	1.7	2.1	1.9	1.7	3.6	6.6	1.7
RPL22L1	5.8	11.0	2.0	4.7	3.5	5.5	1.0	3.9	1.0
BAGE2	7.7	9.9	1.7	2.6	2.4	1.6	2.5	8.5	1.5
SLC26A11	6.6	8.1	2.2	3.0	3.1	2.2	3.3	8.3	1.6
TBC1D3C	9.5	10.7	2.2	1.2	2.3	0.3	4.0	7.9	0.4
TXNDC12	5.1	9.3	1.8	2.5	2.2	3.0	3.7	9.0	1.9
MAMDC4	6.9	8.3	2.0	3.3	2.6	2.1	3.8	7.6	2.0
NIPA1	6.4	8.2	1.9	3.3	3.0	2.6	3.6	8.4	1.1
FBXL2	7.1	10.9	1.7	3.9	3.5	2.7	2.1	5.5	1.1
PPP2R2A	7.0	8.9	1.4	3.6	2.8	3.9	2.2	7.5	1.1
DDX47	5.9	8.8	1.6	4.0	2.6	4.3	2.5	7.2	1.4
BCAN	6.3	9.0	2.2	2.3	2.1	1.1	4.3	9.2	1.8
MGC32805	3.4	3.9	1.2	11.3	8.5	5.0	1.3	3.2	0.8
MAPRE2	6.6	8.6	1.4	3.0	2.5	3.6	2.8	7.9	1.8
POLR3C	6.4	8.3	1.6	3.6	2.7	3.0	3.4	7.9	1.5
NAA16	6.3	10.1	1.7	2.4	1.9	3.5	2.5	8.7	1.2
LMAN2L	5.1	7.8	1.4	4.1	3.3	2.9	3.2	9.0	1.4
SLC25A32	6.2	8.3	1.7	2.6	1.8	3.6	3.0	9.2	1.8
AMMECR1L	5.8	8.4	1.5	2.7	2.9	2.1	3.6	9.2	2.1
AKR1C3	1.9	3.7	0.5	10.1	7.9	8.2	1.4	3.6	0.9
KLHL7	6.7	8.8	1.9	3.6	2.2	2.0	3.4	8.4	1.3
CMBL	5.2	8.6	1.4	3.3	2.6	3.6	2.9	8.9	1.6
NFYA	6.3	6.4	1.6	2.8	2.7	1.9	4.1	10.2	2.2
MRPS35	5.7	8.1	2.0	4.2	3.3	2.5	3.1	7.4	2.0
SLC9A1	6.7	7.7	2.1	2.7	2.5	2.5	3.7	8.3	1.9
PLRG1	5.3	9.8	1.6	2.7	1.8	3.1	3.3	9.2	1.5
NAT10	6.7	8.4	1.7	3.0	3.1	2.4	2.9	8.3	1.6
GCC1	6.1	6.7	1.8	3.0	2.5	2.8	4.1	9.8	1.5
ENGASE	6.6	8.8	1.4	3.6	2.6	2.3	3.5	7.9	1.5
ADPGK	5.2	8.1	1.5	4.1	2.7	3.6	2.6	8.8	1.4
MFAP5	7.5	11.2	2.1	2.5	1.7	3.8	2.0	6.2	1.0

FAM98C	6.0	8.5	1.8	3.0	3.1	3.2	3.6	7.0	1.8
RSF1	6.4	7.5	1.9	2.9	2.3	3.0	3.1	9.1	1.8
DCAF12	6.1	8.8	1.6	2.9	2.2	2.0	3.3	9.7	1.4
ARHGAP12	6.8	10.4	1.8	2.4	1.9	2.4	2.3	8.4	1.6
CUL2	6.5	9.7	1.4	3.0	2.1	3.0	2.7	8.4	1.4
SLC38A7	7.2	9.1	2.0	3.5	2.4	2.3	3.2	7.0	1.2
MRPS22	5.4	8.8	1.7	3.2	2.8	2.6	2.9	8.6	2.0
TREX1	5.4	6.6	2.0	4.3	4.3	2.5	3.2	7.9	1.9
MAST3	6.9	7.7	2.2	3.0	2.8	3.5	3.3	6.8	1.7
PRKCI	6.6	9.0	1.3	3.6	2.6	3.7	2.2	7.6	1.2
GFM2	6.2	9.3	1.4	3.8	3.0	3.4	2.3	7.2	1.3
TBC1D1	5.2	7.9	1.6	5.1	4.3	5.1	2.1	5.6	1.1
44625	5.1	9.6	1.4	3.1	2.9	3.7	2.9	7.6	1.6
ZNF263	5.9	7.7	2.0	2.4	2.1	1.8	3.9	10.1	2.0
CTCF	6.1	7.0	1.4	3.4	3.0	3.2	3.6	8.3	2.0
C2orf47	7.1	8.5	1.6	3.3	3.0	2.0	3.2	7.1	1.9
UBE2L3	6.1	5.9	1.8	2.7	3.8	2.8	4.1	9.6	1.2
SMAD1	5.1	6.6	1.0	2.3	1.8	1.5	4.2	13.4	2.0
ZNF346	6.9	8.4	2.1	3.7	2.9	2.7	3.0	6.9	1.3
APOL1	8.1	9.4	2.2	3.3	2.8	2.9	2.3	5.6	1.2
SNX6	6.0	10.2	1.4	3.4	2.4	4.1	2.1	6.8	1.5
LINC00910	6.2	10.0	1.8	2.2	1.7	3.9	2.5	8.5	1.0
POLE3	5.4	6.6	1.7	3.3	3.2	2.9	3.9	9.0	1.9
LRRC58	5.2	8.8	1.7	2.9	2.6	3.6	3.1	8.6	1.4
POLH	5.6	8.5	1.4	4.1	3.0	3.4	2.4	7.9	1.7
BMPR1A	5.7	9.4	1.1	3.4	2.5	3.4	2.4	8.5	1.5
SYTL2	7.3	15.3	1.3	2.2	2.3	2.9	1.5	4.3	0.7
BIN3	5.5	7.6	2.3	3.1	3.4	2.4	4.1	7.6	2.0
DNAJC21	5.7	7.2	1.4	4.6	3.0	3.7	3.1	7.7	1.4
LOC100506248	5.4	3.9	0.9	3.9	4.2	2.4	6.6	8.3	2.1
TMX1	5.2	7.9	1.2	3.5	2.4	3.4	3.3	9.0	2.0
LIG3	6.5	8.5	1.8	2.8	2.8	2.1	3.2	8.6	1.4
CASKIN2	6.6	7.2	2.1	3.9	3.9	2.5	3.2	6.7	1.7
KIAA1462	3.2	4.3	1.0	1.8	1.7	1.0	5.6	16.2	3.0
GPN2	6.3	7.9	1.9	2.9	3.3	2.4	3.5	7.8	1.7
TINF2	5.1	8.1	1.6	3.8	3.7	2.9	2.8	8.2	1.7
SRRM2-AS1	6.5	10.5	1.8	2.4	1.9	3.5	2.2	7.8	1.1
SYS1-DBNDD2	5.8	6.4	1.7	4.6	4.6	2.6	3.2	7.5	1.2
MORC2	6.8	7.3	1.9	2.8	3.1	2.2	3.2	8.3	2.0
KIAA1143	6.8	7.4	1.8	3.3	2.5	2.7	3.4	8.0	1.7
TXNDC11	5.0	7.1	1.4	3.3	3.2	3.0	3.4	9.9	1.3
ARID5A	6.9	7.3	2.4	2.2	2.2	3.9	3.3	7.5	2.0
SLC7A6	5.2	8.8	1.8	2.5	2.5	2.6	3.1	9.7	1.4
LOC100507431	6.5	9.9	1.7	2.3	1.7	4.1	2.3	7.6	1.4
MSMP	5.6	7.0	1.8	3.1	2.0	1.7	4.1	10.7	1.6
NSF	5.1	7.2	1.3	3.5	2.6	2.8	3.5	9.8	1.6
PAPSS2	5.6	8.5	1.6	4.3	3.2	3.2	2.6	7.3	1.2

PSD3	3.9	7.8	1.2	3.6	2.7	2.4	3.5	11.0	1.5
SENP2	5.7	7.2	1.2	3.5	2.7	4.2	3.0	8.5	1.5
APPBP2	5.8	9.3	1.4	3.6	2.6	2.6	2.5	8.6	1.3
THBS1	5.3	6.1	1.8	8.5	7.3	6.1	0.6	1.4	0.4
GATC	6.7	8.7	1.6	3.0	2.5	3.8	3.1	6.7	1.4
MED13L	5.9	8.7	1.8	2.6	2.3	3.6	2.6	8.4	1.6
PLEKHB1	4.3	5.4	1.6	3.6	3.5	2.1	4.5	9.6	2.9
HIST3H2A	6.2	8.4	2.3	2.7	2.2	1.6	4.6	7.0	2.5
CAMKK2	6.1	8.6	2.0	2.9	2.9	3.2	3.3	7.0	1.5
FLJ41200	6.3	9.2	1.8	2.5	1.6	3.9	3.1	7.7	1.4
KLHDC10	6.2	7.9	1.5	2.4	2.2	2.5	2.9	10.4	1.6
CRIPAK	5.9	8.5	2.3	2.3	2.1	1.1	3.7	9.3	2.3
C22orf39	6.3	5.9	3.1	3.8	4.5	1.3	4.9	6.3	1.4
BTG3	5.8	9.4	1.4	3.6	2.7	3.1	2.1	8.0	1.4
SLC30A5	4.3	8.1	1.1	2.4	1.4	2.6	3.9	11.8	1.8
LOC100505794	6.3	10.0	1.9	2.1	1.9	3.7	3.1	7.1	1.5
PPP1R8	6.5	7.4	1.7	2.7	2.6	1.8	3.2	9.6	1.9
SLC22A17	7.0	8.1	2.5	2.9	3.0	1.8	3.4	7.1	1.6
NCBP1	5.6	7.6	1.3	3.6	2.8	3.4	2.7	8.8	1.7
BACE2	5.5	7.5	1.5	3.8	3.2	2.8	3.4	8.3	1.4
FAM160A2	6.9	8.6	2.0	2.2	2.2	1.8	3.8	8.3	1.7
SMEK2	5.6	8.7	1.3	3.0	2.6	3.1	2.8	8.9	1.6
KANSL1	5.7	8.1	1.9	2.4	2.6	3.6	3.1	8.3	1.6
SMN2	6.5	8.6	1.8	2.8	2.8	2.4	3.2	7.6	1.7
CFDP1	5.8	5.1	2.4	4.6	4.0	3.1	3.8	6.7	1.8
CREBZF	6.6	8.2	1.9	3.6	2.4	2.4	3.6	6.9	1.6
MBOAT2	6.2	7.5	2.2	4.5	4.2	2.8	2.5	6.1	1.3
ZBTB8OS	4.7	7.9	2.0	4.1	3.2	2.9	3.1	8.0	1.4
EAF1	6.7	8.5	2.1	2.6	2.9	1.8	3.3	7.5	2.0
C14orf132	5.8	6.0	1.5	3.6	3.6	1.2	4.7	8.3	2.5
PTPRK	6.0	7.6	1.5	2.3	1.8	1.7	3.4	11.1	1.8
SLMAP	5.9	8.0	1.3	3.5	2.9	3.6	2.2	8.5	1.3
DYRK2	5.5	8.0	1.8	1.8	1.6	3.7	3.4	9.7	1.8
GFM1	6.6	9.1	1.6	3.3	2.5	2.9	2.6	7.2	1.4
RASA4	7.0	8.8	2.5	4.0	3.7	2.2	2.7	5.3	1.1
FAM204A	4.8	9.1	1.4	3.7	2.3	3.2	2.3	8.9	1.5
AIFM2	5.5	8.1	2.0	4.1	3.7	2.3	3.0	7.3	1.2
THTPA	6.1	8.3	1.6	2.9	2.0	2.1	3.3	8.8	2.1
LINC00116	5.5	9.6	2.1	3.3	2.9	2.8	2.9	6.6	1.6
ATAD3B	6.8	7.9	2.1	2.4	2.2	2.8	3.8	7.5	1.7
NDST2	6.3	8.6	1.8	3.1	2.9	2.9	2.8	7.2	1.6
NEIL2	5.4	7.2	1.4	3.1	2.6	2.2	3.4	10.6	1.2
ATP6V1H	5.8	9.2	1.7	3.4	2.6	3.2	2.4	7.4	1.5
TRNAG28	3.3	4.1	1.4	1.6	1.0	0.5	13.7	9.9	1.6
TRNAG1	3.3	4.1	1.4	1.6	1.0	0.5	13.7	9.9	1.6
C7orf25	5.6	8.7	1.3	3.5	2.5	3.0	2.8	8.5	1.1
NBR2	6.3	8.2	2.1	3.1	2.9	1.9	3.4	7.9	1.5

FNBP4	6.3	8.2	1.7	2.4	2.1	3.0	3.1	8.6	1.8
CMC1	6.0	6.6	1.7	4.1	4.1	3.0	3.4	6.4	1.7
NFE2L3	5.9	8.7	1.4	3.5	3.4	3.0	2.5	7.3	1.4
LOC101929037	6.8	10.2	1.5	2.1	1.7	3.9	2.2	7.6	1.2
MEF2BNB	7.8	5.9	1.3	3.5	4.9	2.9	1.9	8.0	1.1
EPB41L2	6.1	6.0	1.5	3.5	3.2	3.2	3.3	8.3	1.9
ZC3H14	6.4	7.6	1.7	3.0	2.7	2.7	2.5	8.4	2.0
PRKRA	5.7	7.8	1.8	3.2	2.8	3.0	3.3	8.1	1.5
CCDC115	5.9	7.6	1.6	2.8	3.1	2.5	4.0	8.1	1.5
SLC6A6	5.2	6.4	1.6	4.6	4.3	3.5	2.6	7.6	1.3
DDX55	6.2	9.7	1.7	2.6	1.9	3.0	2.8	8.2	1.1
ATF1	6.5	7.5	1.6	3.3	2.8	2.0	3.0	8.5	1.8
OSER1-AS1	6.1	9.3	1.6	2.8	2.0	3.2	2.5	8.3	1.1
LYPD6	6.3	8.4	1.7	5.1	5.1	1.9	1.8	5.6	1.0
POLR2D	7.9	6.0	1.4	2.9	3.2	3.4	2.9	7.5	1.9
ID2	2.2	2.4	0.9	1.7	2.3	8.0	5.2	11.2	3.1
C14orf2	5.8	8.3	0.9	3.0	3.6	4.7	2.2	6.0	2.4
MAP1LC3B2	3.4	20.3	0.7	3.1	1.1	1.6	1.4	4.9	0.5
STAM	6.8	9.3	1.6	3.4	2.9	2.2	2.2	7.1	1.4
LOC101927319	6.3	10.4	1.1	2.3	1.6	3.3	2.4	8.1	1.5
DBH-AS1	8.3	10.1	2.7	4.3	4.2	2.4	1.4	2.8	0.7
LGALS8	4.9	12.6	1.6	2.5	2.5	3.9	2.4	5.6	0.9
DLC1	7.7	10.1	2.4	4.5	3.8	3.0	1.4	3.2	0.9
HMBS	5.0	7.5	1.6	3.4	3.7	2.7	3.2	8.7	1.0
TANC2	5.9	7.8	1.8	2.6	2.7	1.9	3.2	9.4	1.7
CRLF3	5.7	9.3	1.8	2.7	2.2	3.8	2.6	7.6	1.3
IFT20	4.7	7.0	1.1	2.3	1.7	2.0	4.4	11.9	1.9
MPP1	5.9	7.1	1.5	5.3	4.2	4.0	2.5	5.4	1.1
ULK2	6.6	8.9	2.1	3.5	3.2	2.9	2.4	6.3	1.1
PSMC6	6.1	9.1	1.4	3.1	2.0	3.2	2.3	8.1	1.4
JKAMP	5.5	9.5	1.2	3.5	2.5	2.7	2.1	8.0	1.8
MFAP1	5.9	6.9	2.1	4.1	3.1	2.7	3.1	7.3	1.8
WASIR2	6.2	9.3	1.5	2.3	1.4	4.6	2.7	7.5	1.4
RAB39B	5.9	7.9	1.7	1.7	1.7	0.9	4.6	9.8	2.7
DUSP11	5.7	8.2	1.5	3.3	2.5	3.0	2.7	8.3	1.6
PRUNE	6.8	8.1	1.7	2.4	2.4	2.2	3.3	8.2	1.9
WDR61	5.5	9.0	1.4	2.6	2.0	3.0	3.3	8.7	1.3
UBE2O	6.5	7.8	2.1	2.2	2.2	1.9	3.9	8.3	1.9
BRSK2	6.4	6.1	1.8	3.7	3.5	2.5	3.5	7.4	1.8
PTGFRN	6.8	8.7	2.4	3.7	3.3	2.3	2.9	5.6	1.1
PEX13	4.9	7.9	1.5	2.5	2.3	2.7	3.4	9.7	2.0
LOC100133286	7.0	6.3	2.5	3.4	4.6	1.7	4.7	4.7	2.0
DNM1L	5.7	8.4	1.6	3.5	2.6	3.4	2.5	7.5	1.6
POLD1	5.3	6.2	1.6	5.2	4.4	3.9	3.1	5.7	1.5
DLEU2	5.9	9.4	1.7	2.3	1.7	4.4	2.5	7.5	1.4
TMEM63A	6.1	8.6	1.7	3.2	2.8	3.1	2.6	7.4	1.3
SLC2A3	5.6	9.2	1.4	2.1	2.1	5.3	2.4	7.4	1.1

ZNF576	6.5	7.0	2.0	2.4	3.0	2.1	4.0	7.6	2.1
COL4A3BP	5.9	8.8	1.7	3.0	2.2	3.2	3.0	7.3	1.6
STX1B	9.5	9.2	2.3	3.1	3.2	1.6	2.0	4.4	1.3
CCDC101	6.4	7.6	1.5	2.9	2.1	2.1	4.1	8.1	1.8
F3	5.7	7.5	1.5	7.5	6.3	7.4	0.3	0.4	0.1
WDR41	5.4	9.2	1.2	3.8	2.9	3.6	2.3	6.9	1.3
DOCK9	7.1	10.0	1.8	2.8	2.4	2.2	2.3	6.8	1.2
AP4B1-AS1	6.1	10.1	1.2	2.2	1.7	4.1	2.3	7.6	1.3
HSD3B7	7.8	9.6	2.7	3.8	2.7	1.7	2.1	5.3	1.0
LOC101929491	5.8	6.0	2.2	3.5	3.4	1.6	3.0	9.8	1.4
PPP4R2	5.4	7.6	1.2	3.2	2.2	4.2	2.6	8.9	1.3
RNF103-CHMP3	6.8	9.6	0.9	1.2	1.2	0.9	2.4	12.7	0.8
PIGM	5.4	8.4	1.4	3.3	2.9	1.8	2.6	9.1	1.8
C4orf26	6.5	9.3	2.0	2.1	1.5	3.5	2.3	8.2	1.2
WBP11	6.1	6.8	1.9	3.0	2.9	3.0	3.1	7.6	2.2
CTBP2	5.8	8.1	2.1	2.6	2.7	1.7	3.2	8.4	1.8
AARSD1	6.0	7.5	2.2	3.4	2.6	3.2	3.5	6.4	1.7
COQ7	6.2	7.7	2.0	2.9	2.3	1.9	4.1	7.6	1.9
CHRNB1	5.6	7.0	1.7	3.9	3.4	2.2	3.0	7.9	1.9
RPUSD3	6.7	7.2	1.9	4.3	4.2	2.5	2.5	5.8	1.3
DECR2	7.4	9.0	2.3	3.2	2.7	1.9	3.0	5.4	1.7
RBM23	5.8	8.4	1.4	2.7	2.4	2.9	3.0	8.1	1.7
LRRC8E	6.7	9.8	2.2	3.2	3.2	1.6	2.4	6.2	1.1
TRIM3	7.1	8.9	2.0	2.9	3.1	2.0	3.3	5.9	1.2
CLEC11A	6.5	7.1	2.2	2.1	2.5	0.7	4.6	8.2	2.5
SPIDR	5.3	7.6	1.5	4.3	4.1	3.4	2.1	7.0	1.1
FAM19A5	7.4	8.9	3.0	3.5	2.8	2.0	2.9	5.1	0.8
IL34	5.6	6.3	1.6	3.4	2.5	1.7	4.2	9.4	1.7
CMTM7	5.6	6.8	2.2	3.1	3.4	2.8	3.0	7.4	2.2
SLC12A9	4.4	5.7	1.3	3.3	2.5	2.3	4.4	10.5	2.1
ZSWIM8-AS1	6.6	7.4	1.7	2.9	2.5	2.1	3.3	8.2	1.8
POLR2K	5.0	9.0	1.2	2.4	2.5	3.6	3.1	8.1	1.6
NIT1	5.9	8.5	1.3	4.1	3.1	3.1	2.7	6.4	1.1
AGAP2-AS1	5.9	7.1	2.3	3.1	3.7	1.9	3.1	7.9	1.3
CLCC1	5.6	7.9	1.6	2.8	2.9	2.6	2.5	8.8	1.6
HIRIP3	5.8	5.3	2.3	3.8	3.1	2.3	4.2	7.3	2.2
SMC4	4.6	6.9	1.2	4.0	3.0	3.8	2.3	8.8	1.8
GOLGA8B	6.9	8.8	1.3	2.7	2.0	2.0	3.7	7.6	1.3
GNB4	5.9	10.5	1.6	2.4	1.7	2.3	2.3	8.5	1.2
KALRN	7.8	11.3	2.5	1.4	1.2	1.3	2.6	7.2	1.0
LOC101928224	6.2	9.3	1.7	2.1	1.6	3.8	2.6	7.8	1.2
ST3GAL2	5.8	6.9	2.2	3.2	2.7	2.3	3.5	7.9	1.9
GPANK1	5.3	6.8	2.0	3.2	2.8	2.0	4.0	8.0	2.1
LOC101929694	6.7	9.6	1.8	2.0	1.4	3.3	2.2	7.9	1.4
LPIN3	7.5	7.3	1.6	3.1	2.6	1.8	3.6	7.2	1.6
LRCH3	6.0	7.7	1.5	3.2	2.8	2.3	3.1	8.0	1.6
AHSA2	6.9	11.9	1.1	2.3	1.4	1.4	1.7	8.2	1.4

KLHL42	5.6	8.2	2.1	3.3	3.3	2.4	3.1	6.8	1.4
FECH	6.3	7.3	1.4	3.2	2.8	2.4	3.1	7.7	1.9
HAT1	4.6	8.7	1.3	3.7	3.1	3.7	2.1	7.5	1.5
PCMTD1	5.7	7.3	1.2	2.8	2.2	4.6	2.4	8.8	1.3
CBWD2	5.5	7.2	1.5	3.1	2.3	2.7	3.8	8.1	1.9
ZNF581	5.6	7.3	1.9	2.9	3.7	2.6	3.2	6.8	2.2
ANKRD16	6.5	7.7	1.7	2.9	2.8	1.3	3.9	7.8	1.5
TIMM22	6.0	8.7	2.0	3.3	2.9	3.7	2.9	5.8	0.8
SCD5	4.3	6.8	1.2	2.6	2.6	2.1	3.7	11.1	1.7
MXI1	5.6	7.2	1.4	2.8	2.4	3.2	3.3	8.5	1.7
SNORA61	2.4	10.8	2.2	2.7	2.7	1.3	5.9	7.1	1.0
NDUFA9	7.3	8.8	1.4	3.7	2.2	2.7	2.6	6.6	0.9
MAP3K2	5.8	7.6	1.1	2.8	2.1	3.1	2.5	9.2	1.8
ARHGEF12	5.5	7.2	1.3	3.6	2.8	3.0	2.6	8.8	1.3
ZBTB17	5.5	6.3	1.7	3.3	3.5	2.5	3.8	7.5	2.1
LINC00963	5.7	8.3	1.4	3.1	2.1	3.2	2.5	8.3	1.6
SIK2	5.9	6.9	1.9	2.3	2.4	1.9	3.5	9.4	2.0
TOB2	4.5	6.3	2.0	2.3	2.3	4.2	4.3	8.3	1.9
C19orf54	7.2	9.9	1.6	3.1	2.6	2.6	2.4	6.0	0.9
IGKC	0.1	0.7	0.1	0.1	0.1	34.5	0.1	0.2	0.1
RALGAPB	5.8	9.1	1.4	3.0	2.6	2.9	2.2	7.5	1.4
TRAF3IP2-AS1	5.8	9.8	1.7	2.0	1.4	3.1	2.4	8.7	1.2
ST3GAL5	7.4	10.1	1.7	2.5	1.9	1.8	2.2	6.9	1.4
TRIM26	5.5	6.9	1.6	3.1	2.5	3.0	3.6	8.4	1.4
TIA1	5.9	9.1	1.5	2.1	1.5	2.0	3.3	8.7	1.8
IRF9	5.9	7.6	1.8	3.1	2.4	4.1	3.3	6.4	1.5
C19orf47	5.2	6.4	1.6	3.7	3.0	2.0	3.7	8.9	1.5
TMBIM4	5.2	7.8	1.5	3.6	3.2	3.8	3.1	6.5	1.4
QKI	6.6	8.3	1.5	2.6	2.4	3.0	2.5	7.8	1.4
SS18L1	7.0	7.1	1.7	1.6	2.1	1.9	3.7	9.1	1.8
PXN-AS1	6.0	10.0	2.3	3.4	2.3	1.3	2.6	6.1	2.0
YJEFN3	7.1	9.9	2.1	2.3	2.1	0.8	3.0	7.3	1.6
BAMBI	6.3	7.8	1.3	5.4	5.1	3.4	1.3	4.6	0.9
ZFX	5.4	8.5	1.1	2.9	2.2	2.7	2.1	9.6	1.4
LDLRAP1	5.9	6.6	1.6	3.5	3.0	3.2	3.5	7.0	1.8
LINC00663	6.5	9.5	1.6	2.3	1.6	3.9	2.3	7.4	1.1
TRMT10B	6.0	9.4	1.6	1.9	1.6	3.7	2.6	7.5	1.5
THAP8	7.0	6.3	2.5	3.8	3.8	1.4	3.4	6.0	1.7
PGBD2	5.9	10.2	1.6	1.5	1.1	3.8	2.1	8.4	1.2
LOC101926928	5.8	9.8	1.6	1.9	1.5	4.3	2.4	7.4	1.2
NOP9	7.0	9.0	1.7	2.5	1.9	1.4	3.4	7.4	1.6
MARK2	6.1	6.6	1.9	2.9	2.7	3.4	3.4	7.2	1.7
ANKRD50	6.6	9.9	1.5	2.1	1.6	1.8	2.4	8.8	1.2
TFDP2	4.9	7.1	1.7	4.1	3.2	2.2	2.6	8.6	1.4
CCS	6.0	7.7	2.1	3.8	3.6	2.3	3.1	6.2	1.1
CACNG6	8.4	10.5	2.7	2.7	2.4	1.9	1.6	4.6	1.0
CTDSPL	5.0	7.8	1.6	3.6	3.5	2.6	3.1	7.4	1.2

INTS10	5.3	7.9	1.3	3.1	2.3	2.3	3.0	8.9	1.7
BEX1	4.4	7.6	1.2	1.4	2.2	1.7	4.0	11.6	1.8
HIP1R	4.9	5.5	1.7	2.9	2.9	6.3	3.4	6.7	1.6
CHPT1	6.6	8.6	2.0	4.5	3.3	3.1	1.9	4.8	0.9
LINC00998	5.3	9.1	1.6	4.5	2.7	3.3	1.9	6.3	1.1
CHD2	6.4	7.9	1.9	1.7	1.5	3.2	3.2	8.5	1.5
SRR	5.9	9.2	1.3	2.5	1.8	2.5	2.3	8.7	1.6
EIF5B	5.5	6.4	1.7	3.4	2.7	2.8	3.4	8.1	1.7
BIRC6	5.2	8.8	1.4	2.1	1.7	2.7	2.5	10.2	1.2
SMCR7L	5.3	7.3	2.0	3.3	3.3	2.2	3.2	7.6	1.6
EXOSC10	6.6	8.7	1.8	2.4	2.2	2.3	2.9	7.6	1.3
LOC101927723	6.5	9.8	1.9	2.0	1.4	3.4	2.2	7.5	1.1
BAHD1	6.2	6.7	1.7	3.2	3.1	2.9	3.3	7.0	1.7
TCF19	3.9	5.4	1.2	6.6	5.7	4.4	1.8	5.4	1.2
PRKD3	4.9	7.9	1.2	3.6	2.1	3.2	2.6	9.0	1.3
TTC31	6.3	8.4	1.6	2.2	2.0	2.0	3.1	8.5	1.7
ASAP1	5.3	6.5	1.4	4.5	3.8	3.2	2.3	7.3	1.6
C16orf93	7.9	8.6	1.9	2.9	1.6	1.1	3.6	6.9	1.2
TMEM57	4.6	5.7	1.2	1.7	1.9	1.7	4.1	12.6	2.3
AMH	6.5	8.9	2.0	1.9	1.5	1.0	4.5	7.1	2.3
THYN1	5.2	8.3	1.4	4.0	2.7	2.4	2.1	8.0	1.6
PTCSC3	6.2	10.8	1.6	1.8	1.5	3.2	1.7	7.7	1.2
DPY19L1	5.5	9.1	1.2	3.3	2.6	2.5	2.2	8.0	1.3
LOC440157	5.6	8.4	1.8	2.8	2.1	1.8	2.9	8.7	1.5
HIST1H2AB	4.0	8.2	1.6	5.9	5.1	6.0	1.0	3.4	0.5
LOC728755	5.6	10.6	2.4	2.0	1.3	2.4	2.5	7.4	1.7
C19orf55	6.3	6.7	2.2	3.3	3.1	1.5	3.1	7.3	2.1
TMEM199	5.8	7.6	2.4	2.4	2.7	3.1	3.3	7.0	1.3
CD68	7.5	9.4	2.7	3.6	3.7	2.5	1.5	3.8	1.0
B3GALNT2	5.8	8.8	1.7	2.8	2.0	2.9	2.5	8.0	1.1
MPV17L	5.5	9.3	1.7	1.8	1.7	3.3	2.7	8.6	0.9
SPOP	5.5	8.6	1.0	2.6	2.0	3.5	3.1	7.8	1.6
YIPF6	5.3	8.7	1.2	3.1	2.4	4.0	2.2	7.3	1.2
MLH1	5.7	8.0	1.3	3.3	3.1	3.1	2.4	7.3	1.4
HOTAIRM1	7.1	9.6	2.0	1.9	1.4	1.0	3.0	7.8	1.8
LOC101928174	6.1	10.0	1.6	1.9	1.6	3.6	2.3	7.5	1.0
ARRB2	5.7	6.5	1.7	3.2	2.8	3.8	3.6	6.6	1.8
EPM2AIP1	6.3	8.7	1.4	2.7	1.8	2.6	3.3	7.6	1.1
LOC284865	5.7	10.1	1.8	1.9	1.4	4.5	2.2	6.8	1.2
UBE2F	6.0	8.4	1.4	2.6	2.5	3.2	2.1	7.8	1.5
HIRA	5.5	7.1	1.7	2.7	2.6	2.6	3.4	8.5	1.5
PKP3	6.3	8.2	2.2	4.4	4.7	2.7	2.1	4.1	0.9
CTBS	6.7	7.2	1.6	4.1	3.3	1.9	2.7	6.8	1.2
MYO5A	5.3	7.1	1.4	2.7	2.5	2.7	2.7	9.3	1.8
FAM193A	5.7	7.3	2.0	2.6	2.6	2.3	3.1	8.0	1.8
STK38	6.2	7.3	1.4	3.8	2.8	4.5	1.9	6.4	1.1
PHRF1	5.8	6.5	2.1	2.7	2.6	2.8	3.4	7.9	1.7

CCSAP	6.1	5.6	1.9	2.2	2.1	2.5	3.5	9.1	2.5
TLDC1	6.8	8.1	1.8	3.6	3.7	3.0	2.1	5.2	1.2
GIGYF2	6.6	7.4	1.8	2.6	2.1	2.5	3.1	7.5	1.8
CDK2	3.7	7.4	1.1	5.2	4.6	5.5	1.9	4.9	1.1
LOC101927559	6.0	9.5	1.7	2.0	1.5	3.7	2.3	7.6	1.0
TMEM187	5.4	7.5	1.5	3.2	2.5	2.4	3.6	8.0	1.4
THUMPD1	6.4	6.8	1.8	2.8	2.4	1.9	3.7	7.7	1.9
FMNL3	6.2	7.5	1.9	2.4	1.9	2.0	3.3	8.6	1.7
MAP3K14	6.3	7.7	2.1	3.4	3.4	3.4	2.4	5.5	1.2
ORMDL2	5.5	6.5	1.1	4.3	4.0	2.4	3.0	7.0	1.5
NIF3L1	5.3	8.8	1.2	3.2	2.4	1.4	2.6	9.0	1.5
POLR1C	5.1	8.8	1.4	3.0	2.3	2.5	2.8	8.0	1.4
COPS5	4.9	7.7	1.8	2.8	2.7	2.7	3.1	8.0	1.7
RAE1	5.3	8.3	1.7	3.2	2.3	2.5	3.1	7.8	1.3
KLHL12	5.4	8.1	1.0	3.6	3.3	3.4	2.5	7.0	1.2
CDC27	5.5	8.2	1.3	3.4	2.3	3.0	2.3	7.8	1.4
ARSA	6.1	7.7	1.6	3.6	3.3	2.6	3.1	6.1	1.5
RAB4A	6.0	6.6	1.8	3.7	2.8	3.3	2.3	7.5	1.4
GTPBP1	6.4	8.0	2.0	2.2	2.1	3.2	2.7	7.1	1.6
GNAI1	6.3	8.8	1.7	3.4	2.7	3.6	1.9	5.8	1.1
NSL1	4.0	7.8	1.4	3.3	3.1	3.2	3.1	8.3	1.2
IFITM1	2.6	4.5	0.7	0.7	0.5	23.5	0.5	1.9	0.5
EP300	6.0	6.6	1.8	2.3	2.2	2.9	2.7	9.0	1.7
ZMAT5	5.2	6.7	1.8	2.1	2.7	2.3	3.4	8.7	2.3
PSME4	4.8	8.0	1.1	2.5	1.9	2.8	2.6	9.9	1.7
CCDC109B	6.5	7.8	1.9	3.5	2.7	3.7	2.3	6.0	0.9
CORO7	5.2	7.1	1.9	3.2	3.1	4.3	2.9	6.1	1.6
C19orf48	4.0	6.3	1.5	4.1	4.0	2.0	4.2	7.4	1.9
LYST	6.1	10.9	1.6	1.9	1.4	3.5	2.0	7.0	1.0
RTN4RL2	5.9	6.1	2.4	3.0	3.2	1.0	3.4	8.1	2.1
ARFGEF2	5.9	8.2	1.4	2.5	2.2	2.6	2.7	8.4	1.5
CRTC3	6.9	7.5	1.6	3.1	2.4	2.8	2.5	6.9	1.5
GATAD1	5.9	8.6	1.5	2.3	1.6	2.3	2.9	8.7	1.5
ATRX	5.3	6.9	1.3	2.7	2.1	3.1	2.7	9.4	1.7
TPT1-AS1	6.1	9.4	1.9	2.5	2.2	1.9	3.2	6.8	1.3
TUFT1	6.3	7.4	2.0	3.8	3.7	3.8	2.2	4.7	1.2
ANAPC1	5.3	8.5	1.3	2.5	2.1	2.3	2.5	8.9	1.8
CLSTN3	6.7	8.5	1.9	3.6	3.2	2.3	2.3	5.6	1.1
LOXL4	2.2	2.6	0.5	11.8	9.2	7.7	0.2	0.9	0.1
TBC1D15	5.7	7.9	1.3	2.5	1.7	6.0	2.3	6.8	1.0
GRPEL2	5.1	8.1	1.2	3.0	2.0	1.7	3.4	9.3	1.3
ADORA1	5.7	7.0	1.8	2.7	2.3	1.9	3.7	8.4	1.6
LOC101927185	5.6	7.7	1.2	3.2	3.1	2.0	2.5	8.2	1.6
CASP10	5.9	5.9	1.7	5.9	4.5	4.4	1.7	4.5	0.7
ITGA7	6.3	7.8	1.6	2.9	2.0	1.9	3.2	7.8	1.6
BID	5.6	7.9	2.0	2.7	2.3	3.0	2.9	7.6	1.1
PDCD7	6.9	7.0	2.3	2.6	2.8	2.2	3.1	6.5	1.6

GRAMD4	6.5	7.2	1.8	2.2	2.2	3.0	3.3	7.5	1.5
ZNF275	4.8	7.1	1.6	2.5	3.0	1.9	3.8	9.0	1.3
ZNF592	5.6	6.9	1.8	2.3	2.5	2.3	3.5	8.4	1.7
SMG1	5.6	8.8	1.5	2.2	1.7	3.3	2.2	8.7	1.2
LRRC24	6.9	8.5	2.2	2.9	2.2	1.6	3.3	6.0	1.4
BACE1	5.7	7.6	1.9	3.2	2.4	2.1	3.0	7.7	1.5
ACSL1	6.2	8.6	2.1	2.3	2.1	1.8	2.8	7.8	1.4
IPO8	5.3	8.1	1.4	3.1	2.3	2.4	2.5	8.1	1.9
SLC25A38	4.4	7.6	1.2	3.2	2.8	4.0	3.0	7.6	1.1
PSMG2	5.2	9.4	1.0	3.3	1.6	4.0	2.2	7.0	1.2
RRNAD1	5.8	7.9	1.4	2.6	2.2	2.1	3.5	8.1	1.4
DUSP6	3.9	5.5	1.3	0.5	0.7	1.0	6.1	12.8	3.2
PRPSAP2	4.2	6.4	1.2	3.1	2.6	2.9	3.0	10.4	1.2
SMTN	6.5	8.4	2.6	4.9	4.8	2.2	1.6	3.1	0.9
AP3S2	5.6	8.7	1.5	2.7	2.1	2.6	2.6	7.9	1.3
FRMD8	4.9	6.8	1.5	2.9	2.7	3.1	3.7	8.1	1.3
LINC00883	4.4	6.6	1.2	3.9	2.9	2.9	2.9	8.9	1.4
RIN1	6.3	7.9	2.1	2.3	2.3	1.0	3.7	7.2	2.1
MAPKBP1	5.8	8.9	1.6	2.9	2.1	2.3	2.6	6.9	1.7
FAM91A1	4.8	8.7	1.2	3.0	2.1	2.7	2.5	8.6	1.5
LRRC45	5.4	5.9	1.8	4.6	3.8	3.5	3.0	5.1	1.7
STK19	6.7	8.2	1.6	2.1	1.5	1.5	3.8	8.0	1.3
RRM1	5.1	7.8	1.2	5.1	4.1	3.9	1.7	5.1	0.9
PTTG1	5.5	8.9	1.2	6.2	5.6	4.2	0.6	2.1	0.7
HECA	5.2	7.0	1.8	2.2	2.3	5.8	2.8	6.2	1.6
NUDT9	5.7	8.3	1.9	2.6	2.6	2.4	3.0	7.1	1.3
C1R	8.4	9.4	1.9	3.2	3.0	1.2	1.8	4.8	1.1
HFE	5.9	7.9	1.7	3.3	2.3	3.1	2.4	7.0	1.2
ATP11A	5.6	7.6	1.6	2.3	2.3	2.4	3.0	8.4	1.6
SYT1	5.8	8.4	1.1	2.0	2.2	1.5	3.0	9.1	1.8
DEAF1	5.9	7.6	2.0	2.7	3.2	2.3	2.8	6.5	1.8
CDK7	6.2	8.2	1.6	2.7	2.1	2.9	2.3	7.2	1.5
ASCC2	6.0	5.7	1.5	2.5	2.7	2.4	3.7	8.5	1.9
IRF2	6.9	7.3	1.8	2.3	2.2	2.6	3.0	6.8	1.8
LOC101929668	8.5	9.2	2.3	2.0	2.3	0.4	2.4	6.3	1.4
NCK1	5.2	8.7	1.4	2.4	1.7	1.5	2.5	10.0	1.3
FAM96A	4.2	9.9	1.1	3.7	2.4	3.3	2.4	6.3	1.5
PAICS	4.3	7.5	1.0	3.9	2.7	4.2	2.2	7.9	1.0
TUBGCP6	6.9	8.3	1.5	2.2	1.7	2.5	3.7	6.5	1.5
OSBPL11	5.1	7.9	1.4	3.2	2.5	3.4	2.3	7.5	1.4
ZNF689	5.9	6.9	2.0	3.1	3.1	2.0	3.1	7.1	1.5
EIF1AD	5.5	8.2	1.5	2.7	1.8	2.5	3.6	7.0	1.8
RAB40B	6.4	7.8	2.0	2.9	2.6	1.9	3.5	6.2	1.4
EIF1AY	4.2	8.2	0.9	4.0	2.7	2.8	2.4	8.2	1.3
GOLGB1	5.1	7.1	1.3	2.4	1.8	2.3	2.9	10.2	1.5
URB1	5.9	8.2	1.9	2.8	2.3	2.0	2.7	7.7	1.2
FAM74A1	4.5	10.4	1.1	3.0	1.6	4.5	2.0	6.5	1.1

EFNB3	5.5	6.6	2.2	3.0	2.7	1.8	3.5	7.9	1.5
CXCL16	7.2	8.1	1.9	3.1	2.9	1.8	2.2	6.3	1.1
KRCC1	4.6	6.1	1.3	3.9	3.1	2.9	2.6	8.0	2.2
DCAF5	6.3	7.0	1.5	2.3	2.1	2.5	3.1	8.1	1.9
NR3C1	4.9	7.3	1.1	3.1	2.3	3.8	2.5	8.5	1.2
PRC1	5.2	6.5	1.8	7.3	5.8	5.2	0.6	2.0	0.3
TST	5.6	7.9	1.6	2.7	2.6	1.8	3.1	8.1	1.2
KMT2E	5.7	7.0	1.5	2.6	2.2	3.0	2.7	8.2	1.7
TGIF2	5.2	6.3	2.0	3.0	3.6	2.3	3.6	7.1	1.6
C14orf1	5.3	9.1	1.4	3.6	3.3	2.4	2.2	6.2	1.2
BBS1	6.3	9.0	1.7	2.6	1.9	1.1	2.8	7.7	1.4
STRADB	4.7	9.5	1.3	3.3	2.0	2.7	2.4	7.7	1.1
MSH6	4.9	7.8	1.3	3.4	2.8	3.3	2.3	7.6	1.1
NAGPA	5.6	7.4	1.9	3.9	3.9	2.3	2.5	5.7	1.4
EXOSC2	5.3	8.7	1.8	2.8	1.8	3.5	2.3	7.1	1.3
NNT	5.7	9.1	1.3	3.0	2.4	3.1	2.4	6.6	1.0
CPSF3	5.1	7.8	1.3	3.4	2.7	3.4	2.5	7.2	1.3
APOBEC3G	3.7	5.3	1.1	2.2	2.4	1.9	4.0	11.5	2.4
FBXW9	6.0	8.2	2.1	2.3	2.4	1.3	3.4	7.0	1.9
GINS3	5.6	6.2	1.5	2.5	2.6	2.2	3.4	8.9	1.5
ZC3H3	6.2	6.7	2.1	3.0	3.0	2.5	3.5	5.9	1.7
SMAP1	6.0	7.1	1.7	3.1	2.2	2.6	2.5	7.7	1.5
FRMD6	6.2	7.7	1.6	3.4	3.4	3.1	2.4	5.6	1.1
LOC101927528	6.2	8.8	1.6	2.0	1.3	3.9	2.3	7.4	1.1
TP53RK	6.6	6.5	1.6	2.4	2.8	1.3	3.6	7.5	2.2
FLJ37453	5.9	9.2	1.5	2.3	1.5	2.8	3.0	7.2	1.1
CRNKL1	5.1	7.6	1.4	2.7	2.1	3.0	3.0	8.4	1.3
MED31	4.4	8.3	1.8	3.1	2.6	2.3	2.5	7.9	1.5
PHF5A	6.1	6.6	1.0	2.9	3.0	2.4	3.0	8.4	1.0
ZNF628	6.1	6.4	2.5	2.5	2.6	1.8	3.7	6.9	1.9
RRP8	5.9	8.1	2.0	2.3	2.2	1.7	3.0	7.9	1.3
PTAR1	5.7	8.4	1.5	1.8	1.6	1.6	2.6	10.0	1.2
YLPM1	6.4	7.2	1.9	2.5	2.4	2.4	2.6	7.4	1.6
MBOAT1	4.5	6.3	1.1	2.1	1.5	10.5	1.8	5.6	0.9
TNFAIP8L1	5.4	7.3	1.9	4.7	4.4	2.2	2.3	4.7	1.3
CDC42SE2	4.2	7.6	1.4	2.5	2.3	3.7	2.6	8.0	2.0
TAF5L	5.0	7.2	1.0	2.5	2.3	3.0	3.1	8.9	1.4
FAM21A	8.2	8.3	2.5	2.5	1.2	2.4	2.0	5.1	2.3
SEMA4F	6.2	7.8	1.6	3.3	2.7	2.8	2.5	6.4	1.1
STAG1	4.1	5.9	0.9	2.9	2.2	3.1	3.3	10.5	1.6
SLU7	6.2	7.4	1.5	2.6	2.1	2.9	2.8	7.3	1.6
MCM5	4.8	6.4	1.6	6.2	5.9	3.7	1.4	3.6	0.8
CMTR1	6.1	8.2	1.5	2.9	2.2	2.7	2.9	6.8	1.0
SCYL2	5.9	8.6	1.3	3.2	2.2	2.8	1.9	7.3	1.2
WDR4	5.3	7.0	1.5	3.1	2.3	2.8	3.0	8.4	1.0
CLDN15	6.2	7.4	1.2	1.7	1.7	1.3	4.3	7.9	2.4
CCDC137	4.9	5.4	1.6	3.0	3.3	3.5	3.6	7.2	1.8

VAPB	5.4	8.3	1.4	2.9	2.3	2.6	2.3	7.6	1.5
SNORA78	3.1	3.1	2.2	3.4	3.1	4.6	10.3	3.1	1.5
TGFBR1	4.2	7.1	1.1	2.4	1.7	2.0	2.7	11.5	1.6
MYH10	6.0	7.1	1.8	4.0	3.7	3.1	2.2	5.4	1.1
CPOX	6.3	7.2	2.1	3.5	3.2	2.6	2.9	5.3	1.2
SMIM11	5.2	7.5	0.6	3.6	3.3	2.5	2.3	7.2	2.2
RAB6B	7.9	7.9	1.9	3.2	2.1	1.6	2.8	5.8	1.1
CCNH	5.6	7.4	1.6	4.4	3.6	3.8	1.5	5.1	1.3
C1orf35	5.6	5.5	1.9	2.8	2.9	2.5	4.8	6.6	1.6
FAM171A2	5.9	6.6	2.1	4.0	3.6	2.2	2.7	5.7	1.4
SOWAHC	5.6	7.4	2.1	2.7	2.8	1.3	3.0	7.5	1.7
ADPRHL2	6.1	8.3	1.5	2.5	3.1	2.4	2.6	6.8	1.0
REPS1	5.7	6.8	1.9	3.2	2.4	2.4	3.0	7.4	1.5
MITF	7.4	7.5	2.2	4.1	4.2	3.0	1.4	3.3	1.0
ERCC5	5.7	6.8	1.6	2.9	2.4	2.1	2.9	8.2	1.6
CTNS	5.9	7.4	1.3	3.1	3.0	1.9	2.8	7.3	1.3
DLAT	5.8	6.6	1.7	3.0	2.6	2.8	2.9	7.1	1.6
EDC3	5.3	6.8	1.3	2.4	2.3	2.1	3.1	8.9	1.9
PTPRCAP	2.2	2.7	0.8	0.8	0.6	22.0	1.5	2.6	0.7
DUSP12	5.2	8.2	1.8	3.5	2.7	2.3	2.4	6.7	1.3
VPS52	6.7	8.5	2.0	2.9	2.7	2.8	2.4	5.0	1.1
ZNF584	5.1	7.4	1.3	3.1	2.4	2.0	3.0	8.5	1.2
DCTN5	5.2	8.0	1.5	2.7	2.3	3.4	3.0	7.0	1.1
CBFA2T2	5.7	6.8	1.6	2.4	2.2	1.4	3.4	9.0	1.6
SPG20	5.3	7.5	1.3	3.4	2.7	3.6	1.9	7.3	1.1
ZNF259	4.8	6.6	1.7	2.6	2.4	3.4	3.3	7.9	1.5
CRAMP1L	5.6	7.0	1.8	2.3	2.2	2.0	3.2	8.4	1.6
SCAF11	5.4	7.3	1.3	3.0	2.7	2.9	2.3	7.9	1.5
PJA1	5.7	7.3	1.7	3.0	2.1	1.7	3.1	7.7	1.8
LOC101927181	5.7	8.6	1.4	2.2	1.9	3.0	2.3	7.8	1.3
ZNF410	5.2	8.7	1.2	2.5	1.9	3.1	2.4	7.9	1.2
EDEM3	4.4	7.3	1.0	2.0	1.4	2.2	3.0	11.3	1.4
WDR59	6.5	8.7	1.4	2.2	1.7	1.9	2.9	7.2	1.4
USP40	5.9	7.8	1.4	3.0	2.2	2.4	2.2	7.7	1.4
NCEH1	4.8	7.1	1.4	4.6	3.9	2.4	2.4	6.1	1.2
UBE3B	6.2	8.3	1.5	3.2	2.3	2.3	2.3	6.5	1.4
SPPL2A	5.2	8.3	1.1	2.7	1.9	3.0	2.4	8.4	1.0
VARS2	6.1	8.4	1.3	2.8	2.3	2.4	2.7	6.8	1.4
SDC3	6.0	6.3	1.9	2.4	2.3	2.2	3.4	7.7	1.8
PHLDB3	5.3	6.7	1.8	3.0	2.9	1.8	3.7	7.1	1.8
LPPR3	6.3	7.9	2.2	4.5	4.0	2.2	1.9	3.9	1.1
LOC100287497	6.3	4.7	2.0	5.5	5.3	3.5	1.6	4.0	1.0
SH3RF1	6.6	8.8	2.3	2.9	2.8	1.7	2.4	5.3	1.2
TMEM87A	5.3	7.8	1.6	2.7	1.6	2.7	2.9	7.8	1.5
LMO4	5.1	6.9	1.1	2.5	1.8	3.0	3.5	8.9	1.2
FAM83G	6.1	7.8	2.1	2.7	2.7	1.7	2.9	6.6	1.4
HNRNPA1L2	5.0	6.8	1.3	2.4	2.1	5.0	2.6	7.6	1.1

LOC101926913	6.1	8.9	1.5	2.0	1.5	3.6	2.2	7.0	1.0
TXLNA	5.9	6.0	1.6	2.5	2.4	3.1	3.4	7.2	1.8
TP53INP1	4.6	6.8	0.9	2.0	1.6	2.2	3.2	10.7	1.9
RWDD4	5.2	8.3	0.9	2.4	2.6	2.4	2.2	8.2	1.6
IRAK2	4.1	6.0	1.2	1.6	1.2	2.9	3.7	11.2	2.0
UBA7	6.9	9.4	1.5	1.8	1.7	2.9	2.4	6.1	1.2
LOC101927126	5.1	9.1	1.5	1.9	1.5	3.8	2.2	7.6	1.2
GMCL1	4.9	7.2	1.6	2.8	2.1	2.7	2.9	8.1	1.5
PDCD4	5.4	6.8	1.3	2.2	1.4	3.6	3.3	8.4	1.6
KAT2B	5.2	8.0	1.4	3.4	3.2	3.0	2.1	6.4	1.2
C1orf123	6.4	8.5	2.0	3.1	2.3	2.9	2.3	5.3	1.1
SIN3A	5.3	6.1	1.6	2.6	2.3	2.5	3.4	8.2	1.8
TELO2	5.4	7.1	1.5	3.7	3.6	2.4	3.1	5.8	1.4
SMEK1	5.0	6.9	1.5	2.8	2.3	3.8	3.4	6.5	1.7
ABHD10	5.3	7.7	1.5	3.1	2.5	2.4	2.8	7.0	1.6
UTP18	5.8	7.9	1.5	2.3	1.9	3.3	3.2	6.4	1.4
MRPL44	6.0	8.7	1.7	2.9	2.5	2.3	2.6	5.8	1.2
PTGES	8.5	7.0	2.3	6.0	6.0	2.7	0.3	0.6	0.1
ZNF48	5.0	6.9	1.8	3.0	2.9	2.3	3.2	7.1	1.5
MRPL35	5.9	7.7	0.9	2.8	2.1	2.6	2.5	7.9	1.4
KDELC2	5.0	7.7	1.7	4.3	4.0	2.5	1.7	5.7	1.0
ZNF106	5.6	7.8	1.5	3.4	2.4	2.8	2.0	7.1	1.2
GOLGA5	4.8	6.7	1.0	1.8	2.0	1.6	3.3	10.5	2.1
TENM3	5.7	8.1	1.8	3.2	2.8	2.3	2.2	6.4	1.3
ABCC10	5.6	7.3	1.3	1.8	1.6	2.3	3.5	8.7	1.6
AMD1	3.6	7.3	1.1	3.7	2.8	4.1	2.1	7.7	1.3
LOC101929676	5.6	9.1	1.5	2.1	1.6	2.8	2.9	6.7	1.3
PFDN4	5.5	7.8	1.1	3.0	2.8	3.5	1.5	7.4	1.2
CDK5RAP1	5.6	7.4	1.4	2.6	2.1	3.1	2.7	7.0	1.7
PRR3	5.8	6.9	1.6	3.3	2.2	2.4	3.3	6.8	1.3
MRPL13	5.3	7.6	1.2	3.4	2.2	2.4	2.2	7.5	1.9
DHRS4	4.1	6.1	1.8	2.6	2.2	2.8	4.4	8.1	1.6
ADIPOR2	5.1	7.4	1.4	2.8	1.7	2.2	2.9	9.0	1.3
HSDL1	5.1	8.8	1.4	3.4	1.7	3.0	2.1	7.0	1.2
LOC101926911	5.9	9.2	1.5	2.3	1.4	3.0	2.1	7.4	0.9
NOL6	5.1	6.8	1.2	3.1	2.7	2.1	3.2	8.0	1.4
EPAS1	7.8	9.1	2.1	2.9	3.1	2.0	1.9	4.0	0.9
MGC57346	5.1	9.5	1.4	2.5	2.2	2.5	2.2	7.0	1.2
PCDHB9	5.3	6.8	1.7	2.0	2.1	1.2	3.7	9.2	1.8
SYNRG	5.2	7.9	1.4	2.4	1.8	2.9	2.5	7.9	1.6
CHSY1	5.6	7.6	1.6	2.0	1.6	2.5	2.8	8.0	1.9
ANKRD27	5.9	7.8	1.7	2.8	2.0	2.5	2.6	7.0	1.3
SH3D19	6.4	8.0	1.4	3.5	2.9	2.6	1.9	5.7	1.2
P4HTM	6.3	8.8	2.0	2.6	2.2	2.1	2.7	5.7	1.2
CINP	6.1	8.2	1.4	2.8	2.4	1.7	2.7	6.8	1.5
TMEM160	5.1	6.7	1.8	4.5	3.7	3.4	2.7	4.5	1.2
PRKX	5.0	6.2	1.6	2.4	2.4	4.1	2.9	7.5	1.5

RNFT2	6.2	7.7	1.9	3.5	2.9	1.9	2.5	6.1	0.9
RPS6KB1	4.9	7.2	1.1	2.8	1.9	2.4	2.7	8.8	1.7
ALDOC	8.7	11.7	3.0	2.5	2.3	1.5	1.0	2.4	0.5
FAM73B	4.3	7.4	1.4	2.4	2.5	2.0	3.9	8.3	1.4
LRP5	4.6	5.6	1.7	3.3	3.1	2.1	3.8	7.7	1.7
PUSL1	5.6	6.9	1.6	2.9	3.2	1.8	3.3	6.7	1.5
BMS1	5.4	6.6	1.5	2.3	2.0	2.7	3.3	8.2	1.6
RHOBTB1	5.6	8.8	2.0	3.5	2.9	3.1	2.2	4.4	1.1
RNU11	7.7	12.4	3.1	1.9	1.0	1.1	2.3	3.6	0.5
LOC101929479	6.3	7.0	1.6	2.3	2.3	1.9	2.6	7.9	1.5
CDC23	4.7	9.0	1.4	2.6	2.2	2.3	2.7	7.2	1.4
STX7	6.6	7.6	1.7	3.6	2.6	3.3	1.8	5.7	0.6
TSHZ3	5.0	6.0	1.5	1.6	1.6	0.7	4.1	10.6	2.3
COPZ2	5.9	8.5	1.8	3.6	3.2	1.9	2.1	5.5	0.8
WDR33	5.5	7.5	1.3	2.2	2.0	2.9	2.9	7.7	1.5
TRNC	4.9	9.2	2.6	3.0	0.4	1.8	2.6	8.0	1.0
NSRP1	5.9	5.3	1.7	2.9	2.6	2.0	3.5	7.6	1.9
PCYOX1L	7.0	9.2	2.4	2.9	2.8	1.8	1.8	4.6	0.9
PFKFB3	4.7	5.8	1.8	1.8	1.8	4.5	3.5	7.9	1.6
B4GALT6	5.7	10.2	1.5	3.1	2.0	2.7	1.7	5.9	0.7
SERTAD2	5.5	5.8	2.1	3.5	3.6	3.8	2.0	5.7	1.4
ETNK2	5.4	7.0	1.8	4.1	4.4	2.5	2.1	4.5	1.5
USP48	4.4	8.2	1.2	2.2	1.5	3.2	2.2	9.1	1.4
DUSP10	5.1	6.0	1.4	3.7	3.2	3.6	2.6	6.6	1.3
H1FX-AS1	5.4	7.2	1.7	3.0	2.5	3.0	2.8	6.2	1.5
PELO	4.6	7.0	1.4	3.3	2.6	2.4	3.1	7.9	1.1
SCN1B	5.4	7.8	1.4	3.1	2.3	3.2	2.9	5.7	1.6
LMCD1	6.4	7.9	1.6	4.1	3.9	1.7	2.1	4.6	1.0
TMX4	5.4	6.9	1.9	2.4	2.1	2.3	3.6	7.2	1.5
RBM26	6.3	8.0	1.3	2.2	2.3	2.8	2.4	6.6	1.4
MARK3	6.2	7.6	1.2	3.0	2.1	2.6	2.5	6.8	1.3
DRG1	4.3	7.5	1.3	2.2	1.6	3.1	2.9	9.1	1.3
ASAP3	6.0	7.3	1.8	2.8	2.8	1.9	2.8	6.6	1.4
DGCR14	5.2	6.7	2.1	2.8	2.8	1.9	2.9	7.0	2.0
MCUR1	5.3	8.0	1.3	2.6	1.9	3.0	2.4	7.3	1.4
CHMP2B	4.7	8.5	1.1	2.6	2.7	2.8	2.2	7.3	1.3
SESN2	4.8	6.1	1.6	2.1	1.9	1.7	3.6	9.4	2.0
PRPF4	5.5	8.1	1.7	2.6	2.1	2.4	2.7	6.8	1.4
ATG2A	5.9	7.1	1.9	2.9	3.1	3.0	2.4	5.8	1.3
BACE1-AS	6.6	8.5	1.7	1.6	1.7	3.3	2.1	6.6	1.2
LOC285033	4.4	7.1	1.6	2.2	2.6	1.9	3.4	8.5	1.5
MTHFD1	5.6	7.5	1.6	3.9	3.7	2.9	2.0	5.1	1.0
DGKQ	5.0	7.0	1.7	3.1	2.4	2.8	3.3	6.8	1.1
NPM3	4.4	8.5	1.6	2.9	2.8	2.4	2.6	6.7	1.2
FAM122A	5.5	6.1	1.4	3.0	2.5	2.4	3.4	7.4	1.5
FEM1B	5.2	7.5	1.3	2.3	2.1	2.4	2.9	8.0	1.5
CCDC59	6.4	7.7	1.7	2.7	2.8	2.4	2.0	6.3	1.3

ZNF500	5.2	7.3	1.6	2.2	2.6	1.3	3.3	7.8	1.9
NFATC1	5.3	5.0	1.7	2.7	3.4	5.6	2.6	5.4	1.4
IGFBP4	4.4	5.8	1.5	4.9	4.5	3.2	2.4	5.1	1.4
ZNF3	5.5	7.3	1.1	1.8	1.8	2.0	2.5	9.1	2.0
SDE2	5.5	7.8	1.4	2.2	1.7	2.9	2.8	7.5	1.3
LPIN2	5.4	6.8	1.3	2.8	3.1	3.1	2.1	7.2	1.4
MRPL46	4.6	6.4	1.7	3.3	3.1	2.2	2.9	7.6	1.3
DENND2A	6.9	9.8	2.2	2.6	2.3	1.9	2.1	4.6	0.8
MKI67IP	5.5	8.1	1.1	2.7	1.9	3.2	2.3	6.8	1.7
PTPLB	4.9	6.0	1.3	3.6	2.9	3.1	2.6	7.8	1.0
MED12	5.4	6.3	1.7	2.9	2.5	2.5	2.7	7.9	1.5
LOC100506465	5.2	7.2	1.5	2.8	2.8	2.4	2.5	7.3	1.3
RASA4B	6.3	7.9	2.2	3.6	3.3	1.9	2.3	4.7	0.9
CUL9	5.9	7.8	1.5	2.5	2.0	2.4	2.7	6.9	1.4
STK32C	6.4	8.4	1.9	2.5	1.9	1.1	2.8	6.7	1.3
RANBP2	4.6	7.0	1.0	2.1	1.7	3.1	2.5	9.5	1.5
TULP3	5.3	7.4	1.2	2.1	2.1	1.8	2.7	8.7	1.8
AAMDC	4.8	7.7	1.6	3.4	1.6	2.7	3.3	6.5	1.7
GTF2E2	5.7	8.5	1.3	2.7	2.1	2.4	2.1	6.9	1.5
CHURC1	3.9	7.8	1.0	2.8	2.5	4.1	2.8	7.5	0.7
LOC100132249	5.1	6.9	1.8	1.7	1.9	1.2	4.0	8.6	1.7
JAM3	5.1	8.1	1.3	3.2	2.0	2.8	2.7	6.9	1.0
PIGX	5.9	7.8	1.5	4.0	2.8	2.5	1.8	5.7	1.1
TRMT6	5.0	6.4	1.3	2.6	2.4	2.5	2.9	8.2	1.7
PLXNB1	5.6	7.2	1.6	2.8	2.2	1.4	3.3	7.3	1.5
LOC284801	0.2	0.6	0.1	0.1	0.1	31.5	0.1	0.4	0.1
SCARNA9	6.8	4.9	1.3	4.1	2.3	3.8	2.6	5.8	1.4
TMEM138	5.4	7.1	1.6	2.6	2.5	1.8	2.7	7.7	1.6
RHPN1-AS1	5.5	9.6	1.3	1.8	1.1	3.3	2.2	7.3	0.9
DPP8	5.5	8.0	1.4	2.4	1.7	2.4	2.6	7.7	1.2
INVS	4.6	7.0	1.4	3.1	2.6	2.3	2.6	7.9	1.6
FAM126A	5.0	9.1	1.0	2.9	1.8	3.0	1.9	7.2	1.1
MED21	4.6	5.8	1.8	2.3	3.3	2.6	2.2	8.8	1.5
SUPT16H	5.8	7.2	1.4	2.9	2.4	3.6	2.4	5.9	1.3
MAD2L1BP	5.6	7.9	1.2	3.1	3.2	1.9	2.5	5.9	1.4
SNN	5.0	7.6	1.2	3.8	3.0	4.8	2.1	4.7	0.6
RUNDC1	5.0	6.7	1.6	2.2	2.1	1.8	3.3	8.9	1.3
TMPO	5.1	7.1	1.1	4.2	3.9	4.2	1.8	4.4	0.9
PIAS1	6.2	7.7	1.3	3.7	3.0	2.8	1.9	5.2	1.1
PPIG	4.8	6.7	1.5	2.7	2.2	2.4	3.6	7.7	1.3
ACHE	8.5	9.6	2.9	3.0	2.3	1.9	1.2	2.7	0.7
SNORA16A	1.6	7.2	2.4	2.0	1.2	4.2	6.7	6.2	1.3
GNL3	4.9	6.0	1.4	2.4	2.0	2.1	3.0	9.3	1.6
KIAA0430	4.8	7.0	1.3	2.1	1.6	2.2	2.6	9.6	1.5
GPR126	7.0	10.2	1.7	3.1	2.4	2.7	1.2	3.9	0.7
STRN	5.2	7.5	1.4	2.6	2.1	2.3	2.7	7.4	1.5
HNRNPH2	5.7	7.9	1.3	2.6	2.2	2.7	2.7	6.4	1.3

LOC400958	5.5	9.1	1.4	1.6	1.3	3.5	2.4	6.8	1.0
MCM4	4.2	5.4	1.2	5.0	4.4	3.6	1.9	6.0	1.2
MAN1A2	5.4	6.4	1.1	2.8	2.2	3.0	2.5	7.9	1.5
ARSD	5.2	6.9	2.0	2.7	2.3	1.7	3.0	7.3	1.7
ZNF574	5.1	6.6	1.8	2.5	2.9	1.9	3.2	7.2	1.5
SLC25A25	5.8	6.8	1.7	3.1	2.6	2.3	2.8	6.4	1.3
CBL	4.8	7.2	1.9	2.8	2.6	2.7	2.3	7.0	1.3
SLC2A8	5.5	7.5	1.6	3.4	3.2	2.1	2.7	5.7	1.1
C8orf58	5.4	7.2	1.9	4.4	3.8	2.5	2.2	4.3	0.9
C17orf59	5.1	6.7	1.9	2.5	2.9	2.2	3.4	6.1	1.8
MSLN	6.4	7.2	2.5	4.9	5.0	2.6	1.4	2.0	0.7
LTB4R2	7.1	9.3	2.4	1.6	1.3	1.0	3.1	5.4	1.4
TMEM18	6.5	6.5	2.1	2.9	3.0	2.8	2.7	4.9	1.3
C4BPB	5.1	8.5	1.7	2.7	1.4	3.6	2.0	7.1	0.6
INCENP	5.2	5.5	2.2	5.0	4.7	2.6	2.0	4.3	1.1
SETD2	5.1	7.1	1.5	2.5	2.1	2.6	2.4	7.9	1.4
GABPA	5.5	8.4	1.3	2.1	2.0	2.4	2.3	7.3	1.3
FAM210A	7.1	10.0	1.6	2.5	2.1	2.2	1.1	4.9	1.1
CHIC2	5.4	9.3	1.3	2.7	2.2	1.6	2.1	6.3	1.6
MGC16142	5.7	9.2	1.5	1.7	1.5	3.2	2.2	6.6	0.9
LOC101927622	4.9	7.5	1.7	3.3	2.1	2.6	2.8	6.8	0.9
LOC654433	5.3	7.9	1.4	2.4	1.9	3.5	2.2	6.8	1.2
ZBTB21	5.2	7.2	1.6	2.1	1.9	1.7	2.7	8.5	1.6
C1orf174	4.4	5.9	1.3	2.5	2.8	1.6	3.6	8.9	1.5
TSC1	6.5	8.4	1.8	2.3	2.2	1.7	2.3	6.3	1.2
DFNB31	5.8	6.9	1.6	2.3	2.1	1.4	3.4	7.2	1.8
RPAP1	5.6	6.9	1.7	2.7	2.5	1.9	2.9	7.0	1.5
C6orf47	4.1	6.6	1.1	2.6	2.5	2.1	3.4	8.7	1.4
SVIP	6.2	4.4	1.2	4.6	4.4	2.7	2.5	4.4	2.1
CRBN	5.5	8.1	1.5	2.6	1.7	2.2	2.9	6.4	1.6
HEBP1	5.3	7.7	0.9	4.0	3.7	2.8	1.8	5.7	0.5
WASF1	5.3	6.2	1.7	3.1	2.7	1.4	2.1	7.9	2.1
HTT	5.1	7.0	1.5	2.9	2.6	3.3	2.2	6.8	1.1
CDK19	4.4	5.6	1.5	2.8	2.3	2.4	3.3	8.1	2.1
GNG7	6.5	8.5	1.9	2.4	2.3	2.5	2.3	5.2	0.9
ANKFY1	5.8	7.9	1.5	2.7	2.4	2.4	2.3	6.3	1.1
FAM53B	4.7	6.7	1.9	1.8	1.9	3.9	3.4	6.8	1.6
ATG16L1	5.4	7.5	1.3	2.3	1.5	2.8	2.8	7.4	1.5
VANGL1	5.2	5.9	1.5	3.6	3.3	2.7	2.6	6.1	1.4
COA5	4.0	6.0	2.1	4.9	1.8	1.2	3.1	7.6	1.6
SMC3	5.1	6.4	1.1	3.2	2.5	3.5	2.4	6.8	1.5
GPR137B	7.1	8.3	2.4	3.1	2.4	2.0	1.8	4.1	1.1
SCARNA20	1.6	11.0	0.9	2.1	1.1	5.1	3.8	6.2	0.7
AREL1	5.6	8.2	1.4	2.2	2.4	2.1	2.3	6.7	1.5
ISG20L2	5.3	5.5	1.5	2.0	2.0	2.0	2.8	9.4	2.0
HEMK1	6.0	8.4	1.3	2.3	1.7	2.0	2.8	6.0	1.8
DNAJC3	4.3	6.2	1.0	2.6	1.5	2.5	3.0	9.5	1.8

LOC100506083	5.6	8.3	1.4	1.9	1.6	4.0	2.3	6.1	1.1
KPNA1	5.2	7.9	1.4	2.4	2.1	2.2	2.4	7.3	1.5
LOC100126584	4.8	8.9	1.2	1.8	1.3	4.5	2.2	6.6	1.1
NBPF1	4.3	5.5	1.1	2.3	2.0	1.4	3.7	10.2	1.8
NKX2-5	5.6	5.6	2.1	3.1	3.5	1.5	4.3	5.5	1.1
SUCLG2	5.4	7.7	1.9	4.0	2.7	3.8	1.4	4.6	0.8
THUMPD3	6.1	8.4	1.2	2.5	1.9	2.5	2.0	6.7	0.9
TNPO3	5.1	6.8	1.4	3.2	2.7	2.5	2.5	7.0	1.2
BYSL	6.7	7.2	1.5	3.9	2.7	1.9	2.3	5.1	1.1
SNORD76	2.8	6.6	3.3	2.1	0.3	1.0	9.0	5.2	2.0
GMEB2	5.3	6.6	1.7	2.6	3.1	3.2	2.7	5.8	1.2
CA11	6.1	7.2	1.7	2.4	2.4	1.5	3.1	6.7	1.3
PMM2	5.0	6.5	1.2	1.7	1.7	1.6	2.7	9.5	2.3
RNF208	6.8	6.3	2.1	3.0	2.7	1.1	3.1	5.7	1.5
PAN2	6.2	8.4	1.3	1.6	1.2	2.1	3.0	7.1	1.5
COX15	5.3	8.1	1.3	2.9	2.4	1.7	2.5	7.3	0.8
RAB8B	6.0	7.5	2.1	3.1	2.2	3.4	2.1	4.5	1.4
GZF1	4.6	6.4	1.6	2.1	1.9	1.6	3.8	8.6	1.6
ENTHD2	5.6	6.8	2.0	2.5	2.5	2.5	3.3	5.5	1.6
EXOSC3	5.5	5.9	1.9	3.6	2.4	2.6	3.0	6.2	1.1
CEBPD	3.9	4.5	1.5	5.6	5.7	3.2	2.0	4.6	1.2
SLC17A5	4.6	8.1	1.0	2.9	2.1	2.7	2.2	7.1	1.7
AMDHD2	5.8	6.9	1.5	4.1	3.9	2.7	2.2	3.8	1.1
SMIM4	4.6	6.2	2.3	3.4	3.4	1.3	3.3	6.4	1.2
PPAN-P2RY11	4.2	6.7	2.4	1.8	2.1	0.6	3.1	8.6	2.6
LGMN	6.1	8.8	1.7	2.5	2.7	1.9	2.1	5.3	1.1
ADK	4.3	8.0	1.3	3.2	1.9	2.5	2.1	7.3	1.4
ELP2	4.9	7.7	1.0	2.7	1.6	2.5	2.3	8.5	0.9
DLGAP1-AS1	5.1	7.3	1.6	2.7	2.0	1.2	2.5	8.4	1.3
ANKHD1-EIF4EBP3	6.0	7.8	1.9	2.6	2.4	2.3	1.8	5.4	1.9
MRPS25	4.8	6.1	1.6	4.1	3.9	2.8	2.2	5.7	0.9
HELZ	4.9	6.6	1.4	2.2	2.2	2.4	2.5	8.3	1.6
ASXL2	5.8	6.2	1.6	2.1	1.9	2.1	2.6	8.0	1.7
ASMTL	5.1	6.5	1.4	2.4	2.0	1.7	3.4	8.0	1.6
ASMTL	5.1	6.5	1.4	2.4	2.0	1.7	3.4	8.0	1.6
NEXN-AS1	5.7	8.2	1.8	2.2	1.6	3.0	2.5	6.3	0.8
IFFO1	7.1	8.0	1.7	2.0	1.6	2.0	3.0	5.6	1.2
ZNF638	5.1	7.5	1.1	2.5	1.9	2.6	2.2	7.9	1.3
APEX2	4.9	6.3	1.5	3.9	3.3	2.1	2.4	6.2	1.4
LOC101929616	4.7	7.2	1.2	2.3	2.3	1.3	2.5	9.0	1.5
FAM117A	5.6	5.8	1.6	3.0	2.7	4.2	2.4	5.3	1.3
GOPC	5.7	7.0	1.4	2.0	1.7	2.5	2.7	7.6	1.5
SCO2	5.0	6.6	1.5	3.0	2.2	1.9	3.2	7.3	1.4
SPIRE2	5.3	6.9	1.6	2.6	2.5	1.8	3.0	6.8	1.6
PIK3C2A	4.2	7.3	1.0	2.7	2.0	2.6	2.1	8.9	1.2
SLC9A7	4.7	6.9	1.4	2.6	2.1	2.5	3.0	7.8	1.0
PPIH	5.2	6.5	1.3	2.2	1.8	3.6	3.3	6.8	1.2

TMCC2	4.8	5.7	1.6	4.3	4.9	2.9	2.4	4.2	1.1
QDPR	5.1	7.4	1.2	3.7	2.3	2.5	2.3	6.3	1.1
LOC100129637	5.7	8.2	1.6	2.0	1.6	2.4	2.5	6.9	0.9
ZNF621	5.9	8.0	1.3	2.2	2.0	1.2	2.2	7.5	1.7
TBC1D24	4.8	6.0	1.5	2.3	2.5	1.4	3.4	8.6	1.4
SLC25A29	4.8	6.6	1.6	3.8	4.0	2.3	2.5	5.3	1.1
C18orf21	4.6	6.7	1.3	3.4	2.7	2.4	2.7	6.7	1.4
COL13A1	2.4	3.2	0.8	0.4	0.3	0.4	7.5	13.9	3.1
LYNX1	5.5	6.1	1.9	2.4	2.5	0.9	3.0	7.2	2.4
KPRP	9.6	11.2	3.9	2.6	2.8	1.1	0.1	0.4	0.2
OSBPL9	5.1	7.6	1.2	2.9	2.5	2.9	2.0	6.7	1.2
SPTY2D1	4.7	5.6	1.0	2.0	1.7	1.9	3.4	9.6	1.9
SLC9A8	5.8	6.7	1.7	2.2	2.7	2.3	2.6	6.6	1.3
RPP38	5.4	6.4	1.0	2.4	2.1	2.0	2.2	8.5	1.8
LOC101928565	5.0	10.8	1.1	1.4	0.9	2.9	1.8	6.9	1.0
LINC00346	5.3	8.5	1.4	2.3	1.9	2.7	1.9	6.8	1.0
SNHG15	5.7	8.7	1.7	3.0	2.5	2.5	1.6	5.2	1.1
C17orf85	5.5	6.9	1.5	2.8	2.3	2.3	2.7	6.4	1.4
SPATA2L	4.9	7.3	2.1	2.3	1.9	2.8	3.0	6.0	1.5
GLIPR1	2.4	3.7	0.6	2.2	2.0	3.4	3.4	12.1	1.9
RBM12	4.6	7.5	1.5	2.7	2.1	3.2	2.1	7.0	1.0
MVB12B	5.4	6.1	1.8	3.3	2.5	2.6	2.6	6.2	1.4
MPP5	5.0	7.4	1.5	3.0	2.3	2.2	2.3	6.9	1.2
TSEN54	4.8	6.7	1.5	3.0	2.5	3.6	2.7	5.8	1.0
ZDHHC2	5.3	9.2	1.5	3.3	2.9	2.4	1.5	4.9	0.7
KCNQ1OT1	5.2	9.2	1.3	1.2	1.0	3.4	1.6	8.0	1.0
NCOA1	5.0	6.7	1.4	2.3	2.4	2.0	2.5	7.9	1.7
APPL2	5.1	8.2	1.5	3.3	2.2	2.5	2.0	6.0	1.0
RFWD2	4.7	6.9	1.3	2.5	1.9	2.5	2.5	7.9	1.4
LINC00875	5.5	6.8	1.5	2.2	1.6	3.3	2.6	7.0	1.4
DIP2A	4.4	6.6	1.3	2.3	2.3	2.6	3.0	7.6	1.6
WRAP73	5.0	7.1	1.8	2.7	2.4	1.7	3.1	6.6	1.3
C4B	7.3	8.2	1.8	1.2	1.2	0.8	2.8	6.9	1.4
TMEM231	6.1	8.6	1.9	2.1	1.4	1.4	2.5	6.3	1.3
CD37	2.5	3.8	0.9	0.7	0.6	13.5	2.3	6.5	0.9
FAM208B	4.7	6.7	1.1	2.2	1.8	2.1	2.6	9.1	1.5
SNUPN	5.2	7.7	1.9	2.5	1.8	2.2	2.5	6.6	1.3
FBXO9	4.1	7.3	0.9	2.7	1.9	2.8	2.6	7.9	1.7
FBXO38	5.2	8.3	1.5	2.3	1.9	2.0	2.4	7.0	1.3
MKL1	5.2	5.1	2.0	2.8	2.8	2.6	3.2	6.4	1.7
AMOTL1	5.9	6.2	2.1	2.2	2.2	1.2	2.9	7.5	1.4
ZNF532	5.1	7.6	1.4	2.8	1.9	2.1	2.2	7.1	1.3
PPP2R3B	5.6	6.0	1.5	3.5	3.2	1.8	2.9	5.9	1.2
PPP2R3B	5.6	6.0	1.5	3.5	3.2	1.8	2.9	5.9	1.2
TRNAG27	3.3	5.1	1.8	1.5	0.2	0.7	6.7	11.3	1.0
TRNAG15	3.3	5.1	1.8	1.5	0.2	0.7	6.7	11.3	1.0
TRNAG21	3.3	5.1	1.8	1.5	0.2	0.7	6.7	11.3	1.0

TRNAG23	3.3	5.1	1.8	1.5	0.2	0.7	6.7	11.3	1.0
TRNAG30	3.3	5.1	1.8	1.5	0.2	0.7	6.7	11.3	1.0
RNF34	5.1	6.5	1.7	2.4	2.3	3.0	2.5	6.4	1.7
FBXO28	5.5	7.4	1.3	2.4	2.6	2.3	2.4	6.8	0.9
SLC11A2	4.2	7.5	1.2	2.6	1.9	2.0	2.6	8.3	1.4
ZNF746	5.0	6.0	2.0	2.5	2.8	2.4	2.9	6.2	1.7
CHD9	4.9	6.8	1.0	2.4	1.9	2.1	2.4	8.8	1.3
CORO1A	0.7	1.2	0.4	0.8	1.2	26.1	0.4	0.7	0.1
AGO1	4.4	5.9	1.5	2.7	2.4	2.3	2.9	7.8	1.5
CSNK1G3	4.5	7.8	1.0	3.0	2.3	3.2	2.2	6.2	1.4
MPHOSPH8	5.2	7.3	1.4	2.3	1.4	3.1	2.6	7.1	1.1
ACAA2	4.4	7.9	1.0	2.5	2.3	2.0	2.9	7.1	1.4
SNAI3-AS1	5.3	9.0	1.8	2.9	2.2	1.7	2.3	5.3	1.1
SPON2	7.8	9.5	2.9	2.2	1.5	1.0	1.7	3.7	1.1
FCHSD2	5.4	7.0	1.4	3.0	2.6	3.3	2.0	5.6	1.2
MICAL3	6.6	9.4	2.9	2.0	1.8	2.2	1.5	4.2	0.8
TUBGCP3	4.7	6.5	1.6	2.8	2.8	2.6	2.6	6.7	1.3
AUH	6.2	7.5	1.0	2.2	1.3	2.6	2.4	6.6	1.7
HNRNPLL	5.0	7.7	0.9	2.8	1.8	2.4	2.4	7.3	1.1
ADRA1A	5.6	7.6	1.6	1.7	1.4	3.9	2.2	6.4	1.0
ABHD14A-ACY1	5.5	7.7	1.6	3.2	2.5	1.7	2.3	5.6	1.2
GSTT1	5.0	6.5	1.6	2.7	3.2	2.2	3.0	5.4	2.0
SPSB1	5.2	5.8	1.4	2.7	2.6	1.8	3.2	7.3	1.4
SEC24B	5.1	7.2	1.3	2.4	2.2	2.0	2.1	7.5	1.5
DESI1	5.5	6.9	1.0	2.8	2.1	2.2	2.7	7.1	1.1
MEMO1	4.9	6.9	1.0	2.8	2.8	2.9	1.9	6.8	1.4
TRAPPC6A	5.9	8.1	1.4	2.3	1.9	2.2	2.5	5.6	1.4
CCDC103	5.4	5.9	1.3	3.0	2.4	2.1	3.4	6.5	1.5
ZNF777	5.5	5.4	1.7	3.3	3.2	1.9	2.8	5.9	1.7
ARHGAP4	5.2	6.3	1.4	1.8	1.3	8.9	1.9	3.7	0.9
LOC100506469	6.2	8.4	1.5	1.9	1.0	1.8	2.8	6.6	1.3
PCNX	5.6	7.1	1.5	2.8	2.2	2.5	2.1	6.6	1.0
SSH2	5.0	6.6	1.4	2.0	1.9	3.8	2.3	7.1	1.4
PCDHB14	4.8	7.3	1.4	1.1	0.9	0.6	3.6	9.4	2.2
DMTF1	4.9	8.3	0.9	2.0	1.8	2.6	2.0	7.4	1.5
PHF20L1	4.4	6.9	1.0	2.1	2.3	1.7	3.0	8.2	1.6
WRAP53	4.2	6.3	1.3	2.9	2.5	2.7	3.1	6.9	1.5
ALG12	4.8	6.0	1.7	2.2	2.4	2.3	3.1	7.2	1.6
SLC20A2	6.4	6.7	1.7	2.0	2.3	1.2	2.9	6.6	1.6
ZFP64	5.4	5.8	1.6	2.6	2.3	2.0	3.1	6.8	1.7
TMX2-CTNND1	5.2	7.5	1.5	2.0	1.9	1.5	2.5	7.9	1.2
RBM7	5.9	8.0	1.1	2.2	1.6	1.7	2.7	6.6	1.6
LOC101929035	4.8	7.4	1.3	2.0	1.3	3.4	2.4	7.4	1.1
DMAP1	4.9	5.8	1.7	3.7	2.9	2.5	3.1	5.5	1.3
TLE2	4.2	5.1	1.5	4.7	4.0	2.1	2.7	5.5	1.5
FOXM1	5.0	5.3	1.7	6.2	5.9	3.6	0.7	2.2	0.7
AHCTF1	4.7	7.5	1.1	2.4	1.8	2.6	2.2	7.8	1.1

LOC101928255	5.2	8.1	1.4	1.7	1.3	3.5	2.0	6.9	1.1
RSPRY1	4.9	7.2	1.2	2.3	1.9	1.6	2.5	7.9	1.7
BAG1	4.3	5.8	1.3	3.3	2.6	4.4	3.0	5.6	1.0
SFT2D1	4.4	8.2	1.1	3.8	1.9	2.6	2.8	5.9	0.6
ATP11B	5.4	8.1	1.3	2.2	1.5	2.4	2.1	7.0	1.2
LOC646471	5.2	7.9	1.4	1.6	1.2	3.8	2.1	7.2	1.0
ZNRF1	4.0	7.4	1.3	2.7	2.2	2.1	2.7	7.6	1.4
TRIB2	3.8	5.9	1.0	1.1	0.6	1.9	3.9	10.6	2.3
GJC2	4.8	6.8	2.1	4.1	3.7	2.2	2.3	3.9	1.3
GEMIN8	5.4	6.4	1.3	2.0	2.3	1.9	3.0	7.2	1.7
FBXW7	4.9	5.5	0.8	2.3	2.1	1.7	3.4	9.4	1.2
TAPSAR1	4.9	7.2	1.0	2.2	1.6	2.9	2.6	7.8	1.0
PLAA	4.8	7.3	1.4	2.4	2.3	2.1	2.2	7.3	1.3
WAPAL	4.7	6.5	1.1	2.5	1.7	2.4	2.5	8.1	1.5
MLYCD	4.4	6.3	1.3	2.7	2.9	2.3	3.2	6.6	1.5
JDP2	5.3	5.6	2.0	2.5	3.4	2.2	3.3	4.6	2.0
SHROOM3	5.3	6.7	1.8	3.4	3.1	1.9	2.3	5.5	1.1
CASP9	4.2	6.1	1.7	2.3	1.6	2.5	3.1	8.0	1.6
PORCN	5.1	7.5	1.7	2.5	2.2	1.4	2.9	6.5	1.3
ALKBH4	4.6	6.5	1.6	2.9	2.2	1.9	3.2	6.7	1.7
LOC101927692	5.2	8.9	1.4	1.7	1.1	3.1	1.8	6.9	1.0
DCAF4	6.2	8.5	1.5	2.7	2.7	2.1	1.9	4.4	1.0
PKNOX1	5.8	7.0	1.5	2.2	1.8	1.7	3.1	6.8	1.3
NKX2-2	6.2	6.2	1.8	3.0	3.0	1.5	2.7	5.4	1.3
LBR	4.1	7.4	1.1	2.5	1.8	3.6	2.1	7.1	1.3
TPX2	4.9	6.2	1.1	6.4	4.7	4.2	0.9	2.2	0.5
PGM2L1	2.7	4.2	0.4	2.1	1.7	2.2	3.3	12.5	1.8
SYNJ2	5.4	7.1	1.5	2.8	2.2	2.0	2.3	6.7	1.1
DZIP1	5.3	6.5	1.6	3.1	2.9	2.0	2.4	5.9	1.2
PCK2	4.8	6.6	1.4	2.1	2.1	1.6	3.1	7.9	1.3
LINC01010	4.8	8.3	1.6	2.0	1.3	2.5	2.2	7.0	1.3
UEVLD	4.9	6.8	0.8	2.8	2.2	2.4	1.7	8.1	1.3
YAE1D1	4.7	5.3	1.4	3.1	2.4	1.8	3.6	7.5	1.2
NMD3	4.7	7.5	1.1	2.1	1.8	2.3	2.0	7.9	1.6
GMPR2	4.8	7.3	1.1	2.9	2.5	3.5	1.9	6.1	0.9
HELZ2	5.0	6.6	1.8	1.7	1.9	5.2	2.4	5.6	1.0
CCDC43	5.4	6.2	1.8	3.2	2.4	2.1	2.9	5.6	1.3
GPER	2.5	2.9	0.7	5.3	5.1	2.5	2.9	7.3	1.8
L3HYPDH	5.2	8.5	1.6	2.1	1.7	1.7	2.9	6.0	1.3
C19orf81	4.1	5.1	1.2	1.7	1.2	0.8	5.6	9.3	1.9
EPHA4	3.9	4.9	1.0	1.2	0.8	1.2	3.9	12.1	2.0
IFI30	3.3	3.1	0.7	1.6	1.3	16.4	1.3	2.7	0.6
THAP11	5.1	5.7	2.1	2.9	3.5	2.2	2.3	5.8	1.4
C12orf57	5.8	6.7	1.7	1.2	0.8	3.7	3.3	5.9	1.9
F2R	5.2	7.2	1.2	2.3	1.8	1.7	2.6	7.7	1.4
SEC11C	3.4	6.0	1.4	3.1	2.3	3.0	2.9	7.7	1.2
KLHL2	4.1	5.8	1.0	1.3	1.3	1.4	3.5	10.7	1.8

FKBP15	5.5	6.9	1.5	2.5	2.4	2.6	2.1	6.4	1.2
ANO10	4.8	9.0	1.2	3.0	2.3	2.3	1.8	5.7	0.9
LINC00864	5.1	9.0	1.3	1.4	1.2	3.2	1.5	7.2	1.0
CERS5	4.8	7.3	1.6	2.8	2.0	3.1	2.1	6.2	1.1
YTHDC1	4.9	6.7	1.2	2.7	2.3	3.0	2.5	6.4	1.3
RHPN2	4.7	6.6	1.6	2.6	2.6	1.8	2.8	6.7	1.4
DUSP22	5.4	7.2	1.4	3.0	2.0	3.0	2.3	5.4	1.2
POLR3E	4.8	5.6	1.5	2.1	2.1	1.7	3.6	7.6	1.8
PXK	4.8	6.9	1.0	2.0	1.7	3.3	2.5	7.3	1.5
EVL	5.9	5.3	2.0	2.2	1.5	6.3	2.0	4.8	0.9
MBD2	5.0	6.4	1.6	3.1	2.6	2.4	1.8	6.4	1.5
WDR37	4.7	7.3	1.5	2.1	2.1	1.8	2.9	7.2	1.2
AP4M1	5.0	7.4	1.2	2.3	1.7	2.0	2.9	7.0	1.4
LOC100131691	5.3	6.4	1.6	2.6	2.0	1.6	3.3	6.5	1.4
SEPT5-GP1BB	6.1	7.3	2.0	2.8	3.0	1.6	2.2	4.6	1.3
STAU2-AS1	4.9	8.3	1.4	1.9	1.6	3.3	2.0	6.5	1.0
STAT6	6.1	7.2	1.6	2.2	2.0	4.6	1.9	4.2	1.1
CDC5L	4.7	7.0	1.2	2.8	2.7	2.5	2.2	6.4	1.3
METTL10	4.8	8.2	1.1	2.1	1.9	2.7	2.2	6.8	0.9
SPAST	4.5	7.2	1.0	2.9	2.2	2.7	2.0	6.8	1.4
ZIC2	5.0	5.4	1.9	2.3	2.5	1.3	3.2	7.0	2.1
RSRC2	5.3	6.2	2.2	3.0	2.9	3.2	1.7	4.4	1.7
DNAJC27-AS1	5.3	8.2	1.3	1.6	1.3	3.1	2.0	6.8	1.1
MANEAL	5.3	5.9	2.5	2.8	3.5	1.2	2.7	5.5	1.3
POMT1	4.9	6.9	1.8	2.3	1.9	2.0	3.2	6.4	1.4
IVNS1ABP	5.0	7.0	0.9	2.6	1.8	3.5	2.3	6.6	1.0
PDCD4-AS1	5.5	8.8	1.4	1.8	1.2	2.8	2.0	6.5	0.8
NAA35	4.8	7.0	1.0	2.5	1.8	2.3	2.3	7.7	1.5
MAPK13	5.2	8.5	1.3	1.8	1.2	2.9	1.8	7.0	1.0
SVIL	2.2	2.9	0.7	0.9	0.7	0.9	5.2	14.4	2.9
SCFD2	4.0	6.3	1.2	1.6	1.5	1.9	3.3	9.2	1.7
ZNHIT2	4.7	6.8	1.7	2.3	2.5	2.1	3.3	5.8	1.5
HECTD4	5.0	7.2	1.6	1.8	1.6	1.7	2.4	8.1	1.3
DDAH1	4.2	6.9	2.0	5.6	4.8	3.9	0.8	2.2	0.3
QSER1	5.2	6.9	1.2	2.6	2.1	2.2	1.9	7.2	1.4
WDR72	6.7	7.3	2.2	3.2	2.0	0.6	2.9	4.3	1.5
TMEM14A	2.7	7.8	0.9	3.5	3.3	3.4	1.6	6.0	1.4
GPATCH8	5.4	6.1	1.6	2.1	2.0	1.9	2.8	7.4	1.5
44809	6.6	3.0	2.0	4.1	4.2	2.2	2.6	4.8	1.2
ATF7IP	4.1	5.9	1.3	2.1	1.8	2.7	2.7	8.3	1.9
ARMC7	5.4	5.6	2.0	4.1	3.6	1.1	2.5	5.2	1.1
LOC101928527	4.7	7.6	1.3	2.3	1.6	2.9	2.7	6.5	1.0
C9orf169	6.0	8.1	1.7	2.7	3.1	0.9	2.1	4.7	1.3
GSTM2	6.6	8.0	1.7	3.4	2.8	2.8	1.3	3.3	0.8
PRPF38A	5.1	5.9	1.8	2.0	2.2	2.5	2.9	6.6	1.6
AKAP13	5.0	6.0	1.6	1.7	1.7	4.1	2.3	7.3	1.1
FBXW8	4.7	6.6	1.3	2.5	2.0	2.2	2.7	7.8	1.0

DERL3	2.6	2.8	0.6	0.4	0.4	0.8	6.7	13.1	3.2
CDC20	4.6	5.9	1.8	6.6	6.1	3.9	0.3	1.4	0.1
ROCK1	5.0	6.0	1.0	2.8	2.1	3.2	1.9	7.4	1.3
SMYD3	4.5	7.0	1.2	3.4	2.7	2.8	2.2	5.6	1.2
PALLD	4.6	5.0	1.8	3.8	4.5	2.3	1.9	5.2	1.4
FYCO1	5.7	6.7	1.9	3.0	3.0	2.5	1.9	4.8	0.9
LOC729887	4.9	5.5	1.9	2.9	2.1	1.6	3.6	6.3	1.8
SMARCA2	4.5	4.6	1.4	2.6	2.1	2.8	3.0	7.8	1.7
NLRP1	6.8	9.0	1.8	1.3	1.3	1.5	2.1	5.5	1.2
USP47	4.3	6.9	1.1	2.2	1.4	2.9	2.3	8.2	1.2
EXOC2	5.6	7.3	1.4	2.3	1.9	2.3	2.1	6.2	1.4
RAD1	5.1	7.3	1.3	2.0	1.5	3.0	2.1	7.2	1.1
FBXL8	5.2	8.0	1.9	1.8	1.7	0.9	3.4	6.2	1.5
DGCR6	5.4	7.4	1.8	3.2	2.1	1.6	2.3	5.4	1.2
AMN1	5.2	7.7	1.1	2.0	1.6	1.4	2.2	8.1	1.1
LOC101928423	5.1	8.2	1.4	1.7	1.0	3.2	2.5	6.8	0.8
DNAJC17	5.6	4.6	2.4	2.4	2.1	1.7	3.7	6.2	1.7
VWA9	4.8	7.4	1.4	2.6	2.1	1.6	2.0	7.3	1.2
TBC1D23	4.7	6.7	1.0	2.3	1.6	2.3	2.4	8.0	1.4
SKAP2	5.2	5.7	0.8	3.0	1.9	3.2	2.3	7.0	1.4
LNPEP	4.5	8.7	1.1	2.3	1.7	3.3	1.7	6.3	1.0
LAMA4	5.8	7.9	1.9	1.3	1.0	0.8	2.9	7.3	1.5
NCAPD2	5.0	6.8	1.4	3.2	3.1	2.8	1.8	5.5	0.8
CLPX	5.0	6.9	1.3	2.4	1.9	2.5	2.2	6.9	1.2
LOC101928151	5.0	7.6	1.3	1.8	1.3	3.8	2.1	6.8	0.8
FBXL20	4.4	7.1	0.9	2.1	1.2	1.8	2.9	8.5	1.4
PSMC3IP	5.0	6.3	1.2	4.1	2.9	2.9	1.9	5.6	0.7
USP16	4.9	7.0	1.0	2.3	1.3	2.8	2.4	7.4	1.3
TMF1	4.6	6.2	1.2	2.2	1.4	2.1	2.7	8.7	1.4
FBXO22	4.0	6.9	1.1	3.1	2.4	2.3	2.9	6.5	1.3
IRS1	5.7	6.1	1.9	3.4	3.6	2.1	1.6	5.0	1.0
RNF169	5.5	7.0	1.5	2.4	2.1	1.8	2.4	6.5	1.3
LOC101927969	4.7	8.3	1.4	1.7	1.5	3.1	1.9	7.0	0.9
CCDC57	6.0	7.5	1.3	1.3	1.4	1.1	3.3	7.0	1.4
TMCC1	5.3	6.7	1.2	2.8	2.1	2.2	2.4	6.4	1.4
MPPE1	5.5	7.7	1.3	1.9	1.2	2.4	2.3	7.1	1.0
KLHL17	5.8	6.7	1.4	2.3	2.3	1.5	3.3	5.7	1.5
RFX2	6.9	7.7	2.7	1.6	2.0	1.2	2.1	5.0	1.3
C12orf73	6.3	6.0	2.0	1.6	2.6	1.3	3.1	6.2	1.4
GTF2H4	6.1	7.3	1.3	2.4	1.9	1.6	2.7	5.9	1.2
ZCCHC14	4.9	6.3	1.5	2.2	1.9	1.9	2.8	7.4	1.6
GPR89A	5.2	7.3	1.2	2.9	2.1	2.3	2.2	6.1	1.1
ZNF639	4.2	7.5	1.0	2.1	1.7	2.0	2.3	7.8	1.7
CEP63	5.8	5.6	1.6	3.2	2.0	2.2	2.4	6.2	1.4
CCDC97	5.5	6.6	1.7	2.0	2.0	2.6	2.5	6.4	1.1
VSTM4	5.5	7.3	1.5	2.1	1.4	3.8	2.1	5.8	0.9
ASCC3	5.1	6.2	1.2	1.9	1.5	2.1	2.2	8.7	1.4

RNMT	6.5	9.0	1.4	1.9	1.4	2.0	1.6	5.8	0.9
IQCH-AS1	4.9	8.1	1.5	1.5	1.5	2.9	2.4	6.8	0.8
HSD17B11	3.5	8.8	0.7	2.5	2.2	3.6	1.9	6.2	1.0
UBE2Q2	4.8	6.4	1.3	2.8	2.3	1.9	2.5	7.0	1.4
LOC101928496	5.3	7.8	1.4	1.8	1.4	3.4	1.9	6.4	1.0
AGGF1	5.0	5.7	1.3	2.8	2.4	2.5	2.5	6.6	1.5
OSR1	7.3	7.1	1.9	1.4	1.7	0.7	2.7	5.4	2.0
FOPNL	6.0	6.2	1.3	3.3	2.1	2.9	1.8	6.0	0.8
POLL	5.6	6.8	1.7	2.9	2.3	2.2	2.2	5.6	1.0
PAQR6	7.1	8.9	1.5	1.6	1.2	0.5	2.6	5.9	1.0
NEDD9	7.2	9.1	2.1	3.0	2.2	2.2	1.2	2.6	0.7
KIF1B	5.1	7.1	1.4	2.2	1.9	1.9	2.1	7.4	1.2
BPHL	4.9	8.2	1.6	3.1	1.9	2.0	2.1	5.5	1.0
SETD3	4.4	7.0	1.0	2.8	2.7	2.7	2.2	6.4	1.2
SETMAR	6.1	8.2	1.5	2.1	1.9	2.3	1.9	5.5	0.8
SMARCA1	4.6	6.8	1.1	2.9	2.4	2.4	2.0	6.9	1.2
BIRC5	4.0	6.2	1.0	6.7	5.5	4.6	0.4	1.5	0.2
LOC100506476	4.9	7.6	1.3	2.0	1.4	2.4	2.3	7.6	0.8
TRNAS21	4.7	8.2	1.8	0.7	1.4	0.9	4.8	6.5	1.3
BRD9	5.0	7.1	1.5	2.1	1.9	2.2	3.1	5.9	1.4
PEX10	5.0	6.4	1.4	3.9	3.5	1.4	2.4	5.2	1.1
RBPJ	4.6	6.7	1.1	2.0	1.8	2.0	2.7	7.8	1.6
PDCD11	5.6	7.3	1.5	2.0	1.7	1.7	2.9	6.4	1.1
KHNYN	4.7	5.8	1.4	2.0	1.2	2.7	3.3	7.5	1.7
LOC100505711	4.4	8.0	1.5	1.8	1.2	2.7	2.4	6.8	1.3
LOC100128233	5.3	7.9	1.3	1.7	1.3	3.9	1.9	5.9	1.1
LOC101928104	5.8	7.8	1.3	1.6	1.2	3.6	2.0	6.1	0.8
TBCC	5.2	6.2	1.4	2.4	1.6	2.2	2.8	6.4	1.9
SNRNP40	4.6	6.2	1.4	2.4	2.2	2.9	2.9	6.3	1.3
PPID	4.9	6.6	1.1	2.7	2.0	2.9	2.3	6.4	1.4
SS18L2	3.3	8.3	1.2	2.3	1.4	4.9	2.0	6.2	0.5
PEX14	5.4	5.6	1.7	2.4	2.4	2.0	3.4	6.0	1.3
WIBG	5.3	5.2	1.6	2.7	2.7	1.4	3.6	6.8	0.9
MRS2	5.0	8.9	1.3	2.4	1.9	2.1	2.0	5.4	1.1
PAPD7	4.7	5.6	1.5	2.7	2.5	2.6	2.3	6.7	1.6
NPAS2	3.4	3.3	1.0	2.7	3.1	1.8	3.5	9.0	2.3
PITPNA-AS1	5.0	5.9	1.5	3.0	2.9	1.7	2.8	5.9	1.2
LCLAT1	5.4	8.2	1.3	2.1	1.8	2.2	1.9	6.0	1.3
SCHIP1	4.7	7.2	1.5	2.6	2.4	1.6	2.7	6.0	1.4
CCNB1IP1	4.8	8.7	1.2	2.8	1.8	1.8	1.7	6.4	0.9
LLPH	5.9	5.0	1.6	3.7	1.8	3.1	2.3	5.6	1.2
ZNF764	4.6	6.2	2.0	2.1	1.7	1.9	3.1	6.8	1.7
RPIA	5.0	7.9	1.4	2.1	1.6	3.4	2.5	5.6	0.6
RNF2	4.6	6.2	1.5	2.2	2.1	1.8	2.3	8.0	1.4
ZNF8	4.9	5.9	1.3	2.5	2.3	2.3	2.7	6.9	1.3
ROCK2	4.7	6.6	1.0	3.2	2.3	3.0	1.8	6.4	1.0
ZFP41	4.7	4.7	1.7	2.7	2.1	2.6	2.7	7.1	1.6

LOC441124	6.2	6.3	1.9	2.1	1.9	0.6	2.7	6.4	1.9
BPNT1	4.5	7.4	1.4	3.0	2.3	2.0	2.0	6.4	1.1
ZNF142	4.6	5.9	1.5	2.6	2.5	1.7	2.8	6.9	1.5
NUDT18	5.0	6.6	1.1	3.4	2.0	2.5	2.5	6.3	0.7
BCL2L2	4.6	6.3	1.1	2.3	2.7	1.8	3.5	6.7	0.9
FOXC1	4.4	4.8	2.1	1.6	1.1	1.1	4.1	8.6	2.4
FBXL5	5.4	7.6	1.3	3.2	2.1	2.3	2.1	5.2	0.9
C5orf51	4.5	8.1	0.9	2.7	1.8	3.0	1.6	6.2	1.2
GLRX2	4.2	5.7	1.2	2.3	1.7	2.2	2.5	7.9	2.2
C7orf43	5.9	6.1	1.5	2.9	2.6	2.2	2.7	4.9	1.3
KAT7	4.8	6.7	1.4	2.4	2.5	3.3	2.0	5.7	1.2
LOC101928101	5.1	8.0	1.3	1.6	1.3	3.5	1.7	6.3	1.1
KIAA1244	5.1	7.2	1.6	2.9	2.8	1.7	1.9	5.9	0.9
STAT5A	5.3	7.2	1.4	2.1	2.0	5.4	1.8	3.9	0.9
MRPL21	4.1	7.4	1.4	2.1	2.2	1.8	3.3	6.6	1.2
SMARCD3	5.3	7.4	1.8	2.3	2.0	1.6	2.8	5.3	1.5
CSNK1G1	4.7	7.1	1.4	1.8	1.7	2.5	2.4	7.3	1.0
CAB39	5.0	7.1	1.3	2.7	1.9	2.8	2.2	6.0	1.0
TTC14	5.5	7.7	0.9	2.1	1.3	2.6	2.5	6.1	1.3
C1orf63	5.6	4.9	0.8	2.5	1.4	7.4	1.9	4.2	1.2
PALM2	4.8	7.1	2.0	1.2	0.8	0.9	2.9	8.5	1.8
COMTD1	5.8	6.5	2.2	3.4	3.5	2.7	1.8	3.4	0.7
XPR1	4.5	6.6	1.0	3.0	2.6	2.9	1.8	6.2	1.3
RAB9A	5.4	7.4	1.0	3.1	1.7	2.0	1.8	6.5	1.0
EIF2AK3	5.1	7.3	1.5	1.7	1.3	2.7	2.3	6.6	1.4
ABCA7	5.9	7.1	1.5	2.5	2.1	2.3	2.6	4.8	1.1
GNG11	4.4	6.7	1.2	2.9	1.8	3.5	2.2	5.0	2.3
ACSL4	4.8	8.6	1.2	2.6	1.7	2.7	1.8	5.3	1.1
LAP3	4.5	7.0	1.2	2.3	1.8	3.1	2.7	6.1	1.1
LOC338799	5.6	7.6	1.3	1.6	1.1	2.7	2.8	5.9	1.4
FZD7	5.0	6.4	2.0	3.3	3.5	1.7	1.9	4.9	1.2
MAMLD1	5.7	6.6	2.9	2.7	3.3	1.6	1.8	4.3	1.1
PGS1	5.4	6.9	1.4	2.2	1.9	2.4	2.6	6.3	1.0
TSPYL4	4.5	6.3	1.4	2.3	1.8	1.8	2.9	7.1	1.7
RAD18	5.3	9.7	1.4	3.3	2.7	3.7	0.7	2.5	0.6
LURAP1L	3.3	5.1	1.1	3.2	2.8	1.4	3.0	8.1	1.9
LINC00514	4.7	8.5	1.3	1.7	1.4	3.3	2.2	5.9	0.9
INTS5	4.8	6.8	1.3	2.3	2.2	2.0	2.6	6.6	1.3
CABIN1	4.9	5.9	1.5	2.2	2.1	2.6	2.6	6.9	1.2
FBXL6	5.3	6.0	1.4	2.9	2.8	1.8	2.7	5.2	1.7
CAPN10	5.3	7.0	1.5	1.9	1.9	1.9	3.1	6.0	1.2
LOC101928050	6.8	8.7	1.8	1.7	1.4	0.8	1.8	5.8	1.0
UBR5	4.9	6.7	1.3	1.9	1.7	2.6	2.2	7.2	1.2
SNORA37	2.0	8.0	2.1	1.1	1.2	1.9	3.7	9.0	0.7
STX18	4.2	7.1	1.2	2.9	2.1	2.2	2.5	6.4	1.1
CCAR1	5.4	6.2	1.4	2.4	2.1	2.2	2.2	6.7	1.2
CSTF3	4.8	6.4	1.0	1.4	1.6	1.7	3.2	8.7	1.1

PFAS	5.3	7.4	1.5	2.6	2.1	1.8	2.7	5.3	1.1
ECE2	1.7	2.8	0.6	1.6	1.1	1.2	5.2	12.6	2.9
C2orf49	4.9	5.6	1.2	3.1	2.9	2.6	1.9	6.7	0.9
ROBO4	5.9	8.6	2.1	1.9	1.9	1.0	2.1	5.1	1.1
CCNB1	3.9	7.2	1.0	6.1	4.3	3.7	0.6	2.3	0.5
PHYHD1	4.8	7.6	1.5	1.7	1.2	4.7	2.0	5.5	0.8
CSDC2	7.3	7.0	2.3	4.0	3.4	2.2	1.0	2.0	0.6
BAI2	5.2	6.2	1.5	2.7	2.3	1.7	2.8	5.8	1.5
LTBP1	5.0	6.0	1.3	1.6	1.4	1.2	2.7	8.9	1.5
ARSB	3.9	6.2	1.6	2.6	2.1	2.1	2.6	7.0	1.7
MSL3	4.7	6.0	1.2	2.3	2.5	2.8	2.3	6.1	1.8
SGK223	3.5	4.8	1.1	0.7	0.7	1.8	4.6	10.4	2.1
SERINC5	4.3	6.4	1.2	1.8	1.2	1.1	3.3	8.6	1.7
PANK2	3.7	5.9	1.4	2.7	2.5	4.0	2.8	5.8	1.0
SNORD46	2.0	5.1	2.0	1.1	1.3	2.4	6.8	8.1	1.0
RFK	5.0	7.9	1.1	2.9	1.7	3.2	1.9	4.9	1.1
RABL3	4.3	6.0	1.4	1.9	1.7	2.3	2.9	7.7	1.4
LPCAT3	5.8	6.4	1.3	2.9	2.6	2.6	2.0	5.1	1.0
CTNNAL1	4.0	6.9	1.2	4.3	2.9	3.5	1.5	4.9	0.6
HIF1A-AS2	4.1	6.0	0.8	4.7	3.0	5.2	0.8	4.5	0.6
WDFY3	4.1	6.8	1.2	2.2	1.6	1.8	2.4	8.5	1.2
TNFRSF21	5.4	6.7	1.6	2.6	2.2	1.4	2.6	6.1	1.0
KMT2C	4.7	7.0	1.3	1.4	1.3	2.6	2.4	7.7	1.2
ZDHHC20	4.1	6.5	1.0	2.7	2.1	3.2	2.2	6.5	1.3
HOXC10	4.7	5.2	2.0	2.8	2.9	2.0	2.6	6.0	1.5
ZNF335	5.1	5.5	1.7	2.2	2.3	2.4	2.7	6.2	1.6
NUP153	4.4	6.4	1.2	2.6	2.3	3.4	1.9	6.2	1.2
NNT-AS1	5.2	7.8	1.3	1.6	1.4	2.8	1.9	6.5	1.0
RAD54L2	5.0	6.7	1.6	2.2	1.8	1.7	2.6	6.8	1.2
MYPOP	5.1	5.0	1.9	2.8	3.4	1.5	2.8	5.3	1.8
KATNB1	4.8	6.4	1.5	3.0	2.7	2.3	2.5	4.8	1.5
NAV3	6.8	8.2	1.7	1.9	1.9	1.4	1.6	5.1	1.0
SOS1	4.6	6.2	1.0	2.4	1.9	2.1	2.2	7.8	1.4
EIF2AK2	4.7	5.6	0.9	3.3	2.1	3.3	2.3	6.4	1.0
CLOCK	4.7	6.4	1.2	2.7	2.4	2.4	1.8	6.8	1.3
ADAMTS10	5.7	6.8	1.5	3.0	2.4	2.1	2.5	4.3	1.2
PSD4	3.6	4.1	1.2	3.6	3.4	5.6	2.2	4.7	1.2
KLF10	4.6	6.1	1.1	1.6	1.5	2.9	2.8	7.2	1.6
LINC00269	5.1	8.0	1.3	1.5	1.1	3.0	2.1	6.6	0.9
RPRD1B	5.1	7.1	1.3	2.0	1.6	3.1	2.0	6.1	1.3
SETX	4.4	7.1	1.1	2.1	1.5	2.4	1.9	7.8	1.2
AP5B1	4.2	6.3	1.4	2.6	2.4	2.8	2.5	6.1	1.1
CCDC93	4.4	7.9	1.2	2.5	2.3	2.4	1.9	6.0	0.8
PBX1	4.4	5.1	1.3	1.9	1.9	1.1	3.6	8.3	2.0
MTG2	4.7	6.2	1.5	2.0	2.3	2.0	3.0	6.2	1.6
SMIM19	5.9	7.1	1.4	2.5	2.0	2.2	1.1	5.4	1.8
AP1M2	6.1	5.9	2.2	5.2	4.6	4.2	0.4	0.6	0.3

VMAC	4.6	5.3	1.2	1.3	1.6	1.7	4.7	7.3	1.8
DIRC2	4.9	7.7	1.3	2.8	2.2	3.1	1.8	4.8	0.8
PVALB	10.1	11.7	1.8	0.4	0.2	0.5	1.3	3.1	0.4
STK35	5.0	6.1	1.4	2.3	2.1	1.9	2.8	6.4	1.4
RRN3	4.8	7.6	1.1	1.9	1.6	1.5	2.5	7.2	1.3
TGM2	4.2	5.1	1.1	3.4	3.3	2.1	2.6	6.3	1.2
TMEM44	5.0	6.5	1.5	2.1	1.9	1.0	3.0	6.9	1.5
LOC101927655	4.0	5.9	1.1	2.2	2.5	4.4	3.0	5.0	1.3
RBM15	4.3	6.0	1.5	2.4	2.1	2.7	2.8	6.3	1.2
C1R	7.7	7.8	1.7	2.2	2.4	1.3	2.0	3.8	0.6
PITHD1	2.9	6.5	1.0	2.8	2.6	2.5	2.9	6.4	1.7
LOC439994	4.8	5.7	1.6	2.6	2.5	1.8	2.7	5.7	2.0
CYTH1	4.3	5.1	1.6	2.8	2.2	3.2	2.7	6.2	1.2
FAM120B	4.5	6.7	1.4	2.1	1.7	1.9	2.4	7.2	1.3
PSMG3-AS1	4.6	5.6	1.5	3.1	2.6	1.8	2.4	6.4	1.1
KRTAP2-3	9.9	11.6	3.0	1.8	1.9	0.2	0.5	0.5	0.1
TMLHE	4.6	7.6	1.3	2.1	2.1	1.8	2.2	6.3	1.3
BLOC1S2	4.3	7.1	1.3	2.4	1.7	3.1	2.2	6.3	1.1
IFT81	5.7	6.4	1.0	2.4	1.7	1.9	1.9	6.9	1.5
MYL5	6.4	7.8	1.6	2.5	1.7	1.3	2.4	5.0	0.5
SGPP1	5.0	7.1	1.4	2.2	2.0	2.7	2.0	6.0	0.9
DHTKD1	4.7	6.5	1.3	2.9	2.5	1.8	2.3	6.1	1.3
STK36	4.6	7.1	1.0	2.3	1.9	1.2	2.5	7.2	1.4
ELP3	5.4	6.8	1.2	2.5	2.1	2.2	2.0	6.2	0.9
NAMPT	4.7	5.8	1.3	2.7	2.0	2.5	2.0	7.3	1.0
CNRIP1	5.4	6.7	1.8	2.5	1.7	1.7	2.8	5.8	0.8
PCDHB3	4.4	6.0	1.5	2.3	2.1	1.6	2.7	7.3	1.3
FNIP1	5.3	7.2	1.2	2.2	1.7	2.2	1.9	6.7	1.0
PITPNB	4.9	6.1	1.2	2.3	1.6	3.2	2.3	6.2	1.7
TMEM167B	4.8	9.5	0.5	2.7	1.5	1.7	1.5	6.0	1.0
GEMIN6	3.9	5.4	1.3	3.7	2.8	2.4	2.3	6.6	0.9
PIGU	5.0	7.4	1.2	2.7	2.0	1.7	1.9	6.1	1.1
ANP32A-IT1	5.0	7.4	1.3	1.8	1.2	3.1	2.1	6.5	0.9
SNHG17	5.2	6.1	1.5	1.9	2.1	1.8	3.2	6.4	0.9
KCNMA1	6.3	9.7	2.0	2.1	1.7	2.2	1.2	3.1	1.0
CPSF2	4.7	7.1	1.1	2.2	1.9	2.5	2.3	6.4	1.1
DDX50	4.2	6.4	1.2	2.0	1.9	2.6	2.6	7.2	1.1
C16orf95	5.0	6.1	1.3	3.2	2.9	1.3	2.8	5.8	0.9
TNXB	7.0	8.1	2.4	2.3	2.4	1.2	1.6	3.6	0.8
SLCO3A1	4.9	6.1	1.8	2.9	3.0	2.5	1.9	4.9	1.1
TP53TG1	5.5	4.9	1.2	2.8	2.7	2.5	2.6	5.6	1.6
PCNT	4.8	6.1	1.4	2.3	1.8	2.0	2.7	6.8	1.3
RHBG	4.5	8.0	1.3	1.6	1.1	3.2	1.9	6.5	1.0
SRP54	4.2	6.4	1.1	2.0	1.7	2.2	2.6	7.8	1.1
TSPAN31	4.9	6.2	1.2	2.2	1.6	2.6	2.4	7.0	1.1
RHOBTB2	4.9	6.4	1.6	2.2	2.0	1.3	2.5	6.7	1.4
NASP	3.9	5.8	1.5	3.4	2.9	3.1	2.3	4.9	1.3

DNMBP-AS1	4.7	7.7	1.3	1.7	1.2	3.2	1.9	6.5	0.8
GPSM3	5.1	5.3	1.9	2.5	2.5	6.6	1.5	2.7	0.9
METTL16	5.1	7.1	1.3	1.7	1.6	1.7	2.5	6.9	1.1
MEGF9	4.8	5.5	1.6	2.6	2.6	2.7	2.5	5.1	1.5
UBAP2	5.0	5.9	1.6	2.4	2.3	1.5	2.3	6.3	1.6
LOC101929380	5.0	7.6	1.2	1.8	1.2	3.6	1.6	6.2	0.8
PPP2R2D	5.3	6.3	1.0	2.6	2.3	2.5	2.4	5.2	1.3
KATNAL1	4.5	7.8	1.4	3.3	2.4	2.5	1.4	4.8	0.8
BRPF3	3.5	4.2	1.0	2.5	2.5	1.8	3.1	8.7	1.7
TOM1L1	5.1	7.0	1.0	2.4	2.0	2.3	1.9	6.2	1.1
MAFG-AS1	4.9	5.2	1.9	2.5	2.1	1.3	2.9	6.7	1.4
SH3BP5-AS1	5.6	7.5	1.2	1.5	1.3	1.7	1.9	6.8	1.3
SEMA6C	5.8	6.4	1.4	2.2	1.9	1.4	2.5	5.9	1.2
LOC101927419	4.3	8.0	1.3	1.7	1.4	2.8	1.8	6.8	0.8
POP5	4.2	5.4	1.2	3.7	3.2	2.5	2.4	5.4	0.9
CCDC9	4.4	3.9	1.3	1.8	2.0	2.9	3.6	7.3	1.7
ACTR8	4.1	5.9	1.0	2.3	2.0	2.0	2.7	7.4	1.4
DOCK6	5.9	7.0	1.7	2.2	1.9	1.1	2.3	5.4	1.2
FERMT2	5.2	7.6	1.2	2.4	1.9	2.3	1.8	5.4	1.1
CBX7	4.9	5.5	1.8	1.8	1.9	2.8	3.2	5.5	1.6
SMUG1	5.0	6.5	1.5	3.2	2.2	2.1	2.0	5.5	0.9
MAK16	4.2	5.7	1.3	2.5	2.6	2.0	2.4	6.8	1.4
APBA2	5.2	5.0	1.7	2.9	3.0	2.8	2.2	4.7	1.2
C12orf76	5.0	6.4	1.9	2.0	0.6	1.5	3.5	6.2	1.7
MZT1	2.3	6.7	1.7	2.8	1.8	4.0	3.3	5.2	0.9
USP53	5.7	8.3	1.1	1.7	1.1	1.6	1.8	6.3	1.1
SRPK1	4.4	6.2	1.0	3.1	2.3	2.8	2.0	6.0	1.0
TBP	4.8	6.8	1.5	2.4	1.9	1.9	2.6	5.5	1.2
SLC7A6OS	5.0	7.0	1.9	3.1	2.1	1.8	2.1	4.5	1.1
RCBTB1	4.4	7.1	1.1	2.1	2.5	2.8	1.8	6.3	0.6
PIK3C3	5.0	7.3	1.1	2.2	1.4	2.0	2.2	6.5	1.1
LONRF2	4.3	6.4	1.2	1.1	0.9	1.1	2.9	9.5	1.4
ANKRD17	4.6	5.8	1.3	2.1	1.9	2.4	2.4	6.9	1.2
DDX51	5.4	6.1	2.0	2.1	1.8	1.9	3.2	5.2	1.1
ZNF83	5.9	8.3	1.0	2.1	1.5	1.7	1.5	5.7	0.9
TRAF3IP1	4.5	4.0	1.6	2.9	2.7	2.1	3.2	6.1	1.7
LMTK2	4.9	5.9	1.5	2.7	2.6	2.3	2.1	5.6	1.0
NAB2	4.9	6.6	1.5	2.6	2.2	1.6	2.3	5.7	1.2
TAF1	4.8	6.5	1.2	1.6	1.3	2.0	2.4	8.0	1.0
RNF215	5.2	6.8	1.4	2.7	1.8	1.2	2.5	5.9	1.1
BEAN1	5.3	5.1	2.1	1.5	1.9	1.2	3.1	7.2	1.2
IFNAR2	4.9	6.4	0.9	2.2	1.7	1.9	1.8	7.5	1.3
LOC100505989	4.6	7.3	1.2	1.8	1.2	3.4	1.9	6.2	1.1
ATP5J2-PTCD1	5.0	5.7	1.4	2.3	2.6	2.0	2.5	5.7	1.3
VTI1A	4.5	5.4	1.4	2.1	2.5	3.2	2.2	6.2	1.1
ALG8	4.1	6.8	1.0	3.3	2.1	2.3	1.7	6.1	1.2
STAM2	4.1	6.6	0.8	2.2	2.1	2.0	2.2	7.3	1.2

IREB2	4.8	7.6	1.1	2.2	1.4	2.1	1.9	6.4	1.0
DLG3	5.1	6.3	1.5	2.0	2.0	1.3	2.7	6.8	0.9
DPYSL4	4.2	5.8	1.9	2.2	2.3	1.3	2.9	6.5	1.4
PXMP4	4.5	5.2	1.2	3.4	2.6	1.8	2.2	6.0	1.6
HIVEP1	4.9	7.3	1.4	2.2	2.0	2.4	1.6	5.7	1.1
WWP2	4.9	6.6	1.2	2.1	2.0	2.6	2.5	5.4	1.2
FGFR4	5.4	7.1	1.8	4.1	3.7	2.3	1.1	2.4	0.6
TXLNG	5.1	5.1	1.3	2.5	1.7	1.9	2.5	6.9	1.6
DAGLB	5.0	6.6	1.3	2.5	2.4	2.3	1.9	5.5	1.1
GRK5	5.0	5.9	1.4	1.9	1.7	3.3	2.2	6.0	1.2
NDUFAF2	4.7	4.9	1.2	3.5	2.5	2.9	2.3	5.1	1.5
RAD51C	4.8	6.2	1.2	3.5	2.6	3.0	1.5	4.9	0.8
HOOK3	4.2	6.3	1.1	1.9	1.6	2.2	2.4	7.7	1.2
HAUS6	4.4	5.9	0.9	1.6	1.4	1.8	2.5	8.3	1.6
DOCK7	5.1	7.6	1.2	1.9	1.6	1.7	1.6	7.1	0.8
RELT	5.3	5.7	1.4	3.7	3.0	3.4	1.7	3.4	0.8
C2CD4C	5.7	6.9	1.8	2.6	1.9	1.5	2.5	4.6	1.1
LOC100507642	5.0	7.1	1.5	1.8	1.3	1.6	2.2	7.0	1.1
LINC00485	5.0	7.9	1.3	1.4	1.3	2.3	1.7	6.9	0.8
SNX25	3.6	5.7	1.2	3.8	2.9	3.9	1.5	5.0	0.9
UBA5	3.6	6.0	0.6	2.5	2.1	2.4	2.5	7.5	1.3
ISCA2	4.6	6.4	1.8	2.7	2.9	2.5	2.1	4.5	1.1
GFRA1	5.3	6.8	2.0	4.2	3.6	2.1	1.2	2.3	0.9
SLC22A23	3.1	3.5	0.8	3.1	2.8	2.5	3.2	7.6	1.9
SLC48A1	5.0	4.5	1.6	3.5	2.6	1.5	2.3	6.2	1.3
IFT122	5.0	6.8	1.3	2.4	1.8	1.9	2.1	6.0	1.1
XPNPEP3	4.3	6.5	1.0	1.7	1.7	1.0	2.8	7.7	1.7
HIST1H4L	4.0	4.3	1.9	4.0	5.1	2.5	2.0	3.3	1.4
TPRKB	4.5	6.3	0.7	3.0	1.4	3.2	2.2	6.4	0.7
DCP1A	4.8	6.0	1.8	1.4	1.3	1.4	2.9	7.2	1.5
LOC100507175	4.5	7.4	1.3	1.8	1.1	3.2	1.8	6.4	0.9
TYW1	4.5	5.7	1.2	2.1	1.6	2.3	2.7	6.9	1.4
MEX3A	3.6	4.4	1.4	2.5	2.6	1.4	3.3	7.2	2.0
MPDZ	4.4	6.6	1.1	2.7	2.0	1.8	2.1	6.7	0.9
SCAF8	4.7	6.6	1.3	2.3	2.0	2.7	1.8	5.8	1.2
ZSCAN25	4.6	5.9	1.6	2.5	1.9	1.4	2.6	6.6	1.2
RFWD3	4.1	5.5	1.1	3.4	2.6	2.3	2.3	6.1	1.0
CTDP1	4.3	5.3	1.6	2.2	2.1	3.6	2.7	5.5	1.2
CHD8	4.8	6.4	1.3	2.1	2.0	2.5	2.1	6.3	1.0
LOC101929431	4.6	7.4	1.3	1.8	1.4	3.3	1.7	5.9	1.0
LOC101929680	4.6	7.6	1.3	2.0	1.4	2.5	2.0	6.0	0.9
JMJD8	5.8	6.3	1.9	2.5	1.7	2.0	2.1	4.8	1.2
TMEM79	5.8	6.6	1.3	1.9	1.8	1.1	2.9	5.7	1.4
SHPK	4.2	6.7	1.5	2.5	2.6	1.8	2.2	5.5	1.3
LOC100506325	4.3	8.0	1.3	1.7	1.6	2.8	1.5	6.2	1.0
ATP6V1C1	4.6	6.9	1.2	2.6	1.7	2.0	1.9	6.4	0.9
ASB16-AS1	4.6	6.0	1.3	2.4	1.8	1.5	2.4	7.0	1.5

NOXA1	5.1	6.7	1.5	3.1	2.7	2.1	2.3	3.9	0.9
KIAA1715	3.9	4.7	1.0	2.8	2.0	2.6	1.8	8.0	1.5
TYRO3	5.4	7.0	1.5	2.9	2.7	1.7	2.0	4.3	0.7
ZNF830	4.2	5.2	1.5	2.5	2.2	2.5	2.6	5.9	1.7
CARS2	4.5	5.6	1.3	4.0	3.3	2.4	1.9	4.4	0.9
ZNF319	5.2	5.0	1.6	2.8	3.0	1.5	2.4	5.7	1.2
ANKHD1	5.0	7.7	0.1	0.3	0.7	1.0	3.5	9.8	0.3
EVC	5.0	6.5	1.6	2.0	1.7	1.2	2.4	6.6	1.2
JMJD1C	5.0	7.1	1.1	1.8	1.5	2.5	1.8	6.4	1.1
FARS2	3.9	7.1	0.9	2.4	1.7	2.1	2.1	7.0	1.1
TMEM206	4.9	7.3	1.8	2.4	1.9	2.1	1.8	5.2	0.9
MMD2	5.4	7.2	1.1	1.4	1.0	3.6	2.0	5.4	1.1
DGCR8	4.5	5.0	1.3	2.2	1.6	2.9	2.6	6.7	1.4
FAM161A	4.9	6.4	1.2	2.0	1.4	3.9	1.8	5.8	0.9
HSF4	5.5	7.0	1.2	1.2	1.4	0.7	3.2	6.6	1.5
LOC101928846	4.0	7.8	1.3	1.5	1.2	3.2	1.8	6.5	1.0
LOC101927924	4.6	8.1	1.1	1.8	1.0	2.7	1.8	6.3	0.9
GRID1	4.9	5.7	1.7	2.1	2.0	1.2	2.9	6.6	1.1
IFT52	4.0	6.4	1.1	2.5	2.1	2.2	1.9	7.2	0.9
MSH2	4.1	6.4	0.9	3.1	2.3	2.9	1.9	5.6	0.9
ZNF213	5.0	5.2	1.5	2.3	1.9	1.4	2.9	6.6	1.3
STX16-NPEPL1	4.9	5.6	1.3	2.6	2.3	2.1	2.7	5.6	1.0
CDK5RAP2	4.7	5.8	1.3	2.8	2.1	2.5	2.1	5.9	1.0
ZC3H7A	3.7	6.2	0.8	2.7	2.4	2.9	2.0	5.9	1.5
NFYB	4.0	4.8	1.4	3.8	2.7	2.1	2.3	6.1	1.0
CASP7	3.9	5.4	1.2	2.2	1.5	1.4	2.8	8.6	1.3
DTX3L	4.3	5.4	1.2	2.5	1.9	2.3	2.1	6.9	1.5
LINC00996	4.9	7.7	1.3	1.5	1.2	3.0	1.9	5.9	0.8
RBPMS	5.9	7.0	1.4	3.4	3.7	1.1	1.7	3.2	0.8
LOC101060691	4.9	7.4	1.5	2.0	2.1	1.6	1.8	6.1	0.7
SNORD89	2.1	4.0	1.3	2.3	1.2	3.2	6.4	6.8	0.8
SUPV3L1	5.6	6.3	1.3	2.4	1.8	1.7	2.2	5.5	1.3
BHLHE40-AS1	5.1	7.4	1.2	1.7	1.2	2.9	1.8	5.9	1.0
CAMK2N2	5.9	5.4	2.0	2.8	2.5	0.6	3.4	4.1	1.4
PPP3CB	4.1	6.1	0.9	2.3	1.7	2.8	2.1	6.8	1.3
HMBOX1	5.4	6.4	1.4	1.7	1.1	1.7	2.2	7.0	1.2
IFIT5	4.6	8.3	1.1	2.4	1.7	2.2	1.6	5.4	0.8
MTMR11	5.5	7.6	1.2	2.9	2.0	2.2	1.8	4.2	0.9
TRNAS16	0.5	1.3	0.8	0.4	0.7	0.5	11.4	12.2	0.4
PLS3	3.4	6.1	0.8	3.6	2.6	2.9	2.1	5.8	0.8
HHAT	5.1	7.3	1.1	3.7	2.5	2.3	1.2	4.1	0.8
CABLES2	3.6	4.6	1.4	3.1	3.7	1.9	2.3	5.6	1.8
CIR1	4.9	5.1	1.2	2.5	2.0	1.7	2.6	6.8	1.2
CDCA7L	4.2	6.0	1.0	2.8	2.1	4.1	2.2	4.9	0.8
ADAMTSL4	6.8	8.9	2.2	3.2	3.0	1.9	0.4	1.4	0.2
ARL5A	4.1	6.0	0.9	2.4	1.8	2.8	2.0	7.0	1.1
SOCS6	4.0	6.2	1.3	1.8	1.6	1.5	2.6	7.7	1.4

EEFSEC	4.5	5.1	1.7	2.0	2.3	2.1	2.7	6.4	1.2
LOC731424	4.6	7.5	1.2	1.8	1.5	2.8	1.9	6.0	0.8
KIAA0513	4.8	5.1	1.7	2.0	2.2	2.3	2.9	6.0	1.0
FEM1C	4.9	7.7	1.1	2.2	1.7	2.1	1.9	5.5	0.9
CCDC51	5.4	6.2	1.9	3.1	2.6	1.8	1.9	4.4	0.7
LOC100507437	4.7	6.6	1.3	2.0	1.9	3.8	1.7	5.1	1.0
METAP1	4.4	7.2	1.2	3.2	2.3	2.3	1.7	4.5	1.2
UCK1	3.8	5.2	1.3	2.8	3.0	2.1	2.7	5.7	1.4
NBL1	5.2	5.3	1.7	1.4	2.0	1.0	3.1	6.7	1.5
LINC00342	5.2	8.2	1.2	1.1	0.9	1.6	1.6	7.4	0.8
ARFGAP3	4.3	6.5	0.9	2.0	1.7	1.7	2.2	7.5	1.3
HADH	5.3	6.6	1.5	2.8	2.4	2.9	1.7	4.0	0.8
CMAS	3.7	6.5	1.2	2.8	2.9	2.1	1.9	5.9	1.0
AGPAT4	4.9	7.0	1.1	1.6	1.5	1.8	2.6	6.1	1.5
DSTYK	4.4	6.8	1.3	2.4	1.8	2.0	2.1	6.1	0.9
RASSF1	3.3	4.1	1.2	1.4	1.8	7.6	2.1	5.5	0.9
NOP58	4.0	5.7	1.1	2.5	1.7	3.1	2.2	6.3	1.3
LOC101060060	5.6	7.9	1.4	1.4	1.5	1.2	1.5	6.8	0.7
JPX	4.5	6.5	1.2	1.8	1.3	4.3	1.7	6.0	0.7
SPOPL	4.5	8.4	1.4	2.1	1.4	2.1	1.5	5.1	1.5
KLHL22	4.4	5.6	1.4	2.3	2.5	1.8	2.5	6.4	1.0
TNNT2	8.0	5.3	3.4	4.2	3.2	3.1	0.5	0.1	0.1
COX20	3.8	5.2	0.9	2.0	2.7	4.8	1.7	5.4	1.6
NEK4	4.3	6.7	1.2	2.1	1.5	2.8	2.2	6.0	1.1
MKKS	4.1	5.2	1.1	2.6	2.3	1.6	2.3	7.5	1.4
LPAR2	5.3	7.4	1.5	1.6	1.6	2.6	2.1	4.7	1.2
PREP	3.1	4.4	0.7	1.7	1.6	2.1	3.5	9.3	1.6
SPATA2	4.9	5.3	1.4	2.0	2.1	1.8	2.7	6.2	1.4
HPS3	4.8	7.7	1.2	2.2	2.0	2.4	1.6	5.1	1.0
SUGT1	4.3	5.0	1.0	2.1	1.5	2.5	2.6	7.5	1.4
HOXD-AS2	5.3	7.7	1.4	2.4	2.3	1.4	2.2	4.1	1.1
RNF31	4.4	6.2	1.3	2.2	2.1	2.5	2.4	5.6	1.2
FBXL15	4.1	6.7	1.0	2.5	2.5	1.7	2.8	5.4	1.1
MDM4	4.8	7.0	1.2	2.0	1.6	2.8	1.7	5.9	0.8
MR1	3.3	5.5	1.4	3.7	3.2	3.3	1.6	5.1	0.7
SLC25A10	3.9	4.5	1.2	4.1	4.0	2.7	2.4	4.1	0.9
RECQL	4.1	6.1	0.9	2.5	2.1	2.7	1.6	6.7	1.2
RBBP9	4.9	6.1	1.7	3.2	2.1	2.5	2.2	4.2	1.0
SNORD118	1.6	11.0	1.1	1.0	1.4	4.8	2.6	3.8	0.5
RBMS3	3.9	4.4	1.4	4.1	4.1	2.4	1.7	4.6	1.2
CRADD	5.0	7.1	1.2	2.0	1.3	1.9	2.2	5.8	1.3
KTN1	4.2	5.3	1.1	2.6	1.9	2.4	2.2	6.9	1.3
TWISTNB	4.1	5.7	1.6	2.7	3.1	2.3	1.9	4.9	1.4
PVRL3	3.9	6.5	1.2	3.1	2.3	2.3	1.6	6.0	1.0
MAPKAPK5	5.0	6.7	1.5	2.4	2.0	2.0	1.5	5.9	0.8
MUL1	4.3	6.3	1.1	1.7	2.4	1.4	2.8	6.5	1.1
ZNF506	4.9	7.4	1.4	1.4	1.0	3.3	1.7	5.8	0.8

SLC36A1	5.9	7.5	2.3	2.6	2.2	1.7	1.3	3.5	0.7
CRYM-AS1	4.5	7.4	1.1	1.8	1.6	2.6	1.7	6.2	0.9
ELP6	4.5	5.5	0.8	3.0	2.2	2.6	2.1	6.2	0.8
DNAJB4	4.8	6.9	0.9	3.7	2.9	4.0	1.0	3.1	0.5
RREB1	4.8	5.7	1.8	1.8	1.9	1.5	2.7	6.2	1.3
SP100	5.1	6.2	1.2	2.5	2.0	3.1	1.7	5.0	0.9
LOC101927838	4.3	6.4	1.4	1.6	1.6	2.0	2.7	6.5	1.2
METTL21A	5.3	5.7	1.2	2.8	1.9	2.4	2.0	5.6	0.9
ACTR10	4.2	7.1	1.1	2.7	1.7	2.7	1.6	5.6	0.9
PRPS1	4.5	7.0	1.1	3.0	2.1	3.0	1.7	4.6	0.8
RNF38	4.5	5.2	1.6	2.0	2.4	2.0	2.4	6.4	1.4
CNOT2	4.0	5.7	1.0	2.3	1.8	2.3	2.3	7.0	1.3
TCFL5	4.1	6.0	1.4	2.4	2.5	2.2	2.7	5.2	1.3
KAT6B	4.0	4.5	1.0	1.7	1.7	1.7	2.9	8.3	1.8
CBLB	4.7	6.6	1.1	1.2	1.1	2.0	2.2	7.4	1.4
PLCXD1	5.4	6.9	1.5	2.2	1.8	2.4	1.8	4.8	0.8
PLCXD1	5.4	6.9	1.5	2.2	1.8	2.4	1.8	4.8	0.8
CD27-AS1	4.8	7.4	1.3	1.5	1.2	2.3	1.8	6.4	0.9
IGSF3	4.1	5.0	1.3	2.3	2.2	1.2	3.0	7.0	1.5
UBE2F-SCLY	4.4	5.2	1.3	2.7	2.3	1.4	2.7	5.9	1.7
PP13	4.5	7.6	1.6	1.8	1.4	2.3	2.0	5.6	0.8
DHRS7B	3.8	5.6	1.5	2.2	2.0	2.1	2.9	6.4	1.2
ZNF497	3.7	6.0	1.5	1.6	2.0	1.1	3.3	7.0	1.4
SACM1L	4.7	6.8	0.8	2.2	2.1	2.4	1.9	5.7	1.1
MRPL48	3.8	7.1	0.8	2.0	1.7	1.2	2.5	6.9	1.6
NOL11	4.2	6.2	1.2	2.2	1.8	1.7	2.3	6.5	1.4
SNHG3	4.3	7.2	1.3	1.5	0.9	1.4	2.4	6.9	1.6
ADCY7	3.4	5.3	1.4	2.6	2.3	3.5	2.6	5.5	0.9
VPS4B	4.9	6.5	1.2	2.3	1.6	2.1	1.9	6.0	1.1
BIVM	4.5	5.9	1.1	3.3	2.1	3.0	1.4	5.2	1.0
LOC101927707	5.3	6.1	1.7	2.1	2.0	1.3	2.2	5.3	1.6
USP42	4.6	5.6	1.4	1.9	1.8	2.0	2.7	6.2	1.3
SLC25A20	3.4	5.3	1.2	2.0	2.2	1.6	3.0	7.6	1.1
HEXIM2	4.4	6.0	1.7	2.0	2.2	1.8	2.9	5.1	1.5
SYNE1	4.9	7.6	1.6	1.2	1.0	1.7	1.8	6.9	0.9
RMDN1	4.6	6.2	1.3	2.7	2.0	1.9	2.0	5.4	1.4
RTEL1-TNFRSF6B	3.8	6.0	1.4	2.7	2.5	2.3	2.5	5.0	1.2
ETV3	4.7	4.9	1.4	1.5	1.6	2.5	2.5	7.2	1.3
DIMT1	3.7	6.2	0.8	2.6	1.9	2.5	2.1	6.1	1.6
GJD3	5.2	4.3	2.0	2.1	2.3	0.9	3.6	5.6	1.4
LOC101928336	5.5	8.8	1.6	1.2	1.2	1.5	1.6	5.4	0.8
SMURF1	3.6	6.0	1.1	2.2	1.9	1.7	2.2	7.2	1.4
KDELC1	5.0	5.9	1.3	2.1	2.2	1.9	2.3	5.6	1.4
C8orf59	3.8	6.6	0.7	2.3	1.4	3.7	1.9	6.2	0.8
FUK	5.1	7.1	1.3	1.6	1.4	1.5	2.6	5.7	1.2
RAB11FIP2	4.8	6.2	1.0	2.2	1.6	2.2	2.0	6.2	1.2
ZNF12	3.7	6.4	1.0	2.1	1.8	1.9	2.7	6.9	1.1

IFT43	4.5	6.1	1.9	2.4	1.7	1.6	2.0	6.3	0.9
TNRC6B	4.0	4.9	1.3	1.6	1.6	2.9	2.6	7.1	1.4
IL13RA1	4.8	6.6	1.4	2.2	1.9	2.5	1.8	4.9	1.3
CEP68	4.8	6.2	1.4	2.4	1.9	2.1	1.9	5.8	0.9
TAF6L	4.4	6.3	1.5	1.7	1.8	1.7	2.6	5.9	1.4
ID3	1.8	1.8	0.5	5.2	6.1	6.0	1.9	3.2	1.0
TNFRSF25	5.4	6.1	1.4	1.5	1.6	4.2	2.5	4.0	0.8
KLHL18	4.1	5.8	1.5	1.8	2.0	2.1	2.8	5.9	1.4
MTR	4.0	5.9	1.1	2.7	2.2	2.7	1.7	6.1	1.0
ZRANB1	4.2	6.5	1.2	2.0	1.4	2.6	2.1	6.4	1.0
ZNF408	4.9	5.2	1.4	2.3	2.3	1.9	2.6	5.3	1.5
ASIC3	5.9	6.4	1.7	1.2	1.1	0.4	2.6	6.1	1.8
ARRDC2	4.1	5.8	1.2	1.7	1.5	3.5	2.9	5.9	0.7
HSPB11	3.3	5.8	0.9	3.4	2.4	4.6	1.0	4.8	1.0
MED28	4.4	6.5	1.1	2.0	1.6	3.8	1.8	4.9	1.1
ZNF674-AS1	4.5	7.1	1.2	1.4	0.9	2.0	2.2	6.9	1.0
ZKSCAN5	4.3	5.4	1.3	2.0	2.4	1.5	2.3	6.7	1.4
ASAP1-IT2	4.5	8.1	1.2	1.5	1.3	2.5	1.6	5.9	0.7
LOC101929295	4.7	7.7	1.1	1.5	1.0	2.6	1.8	6.0	0.9
SPTLC2	4.4	6.1	1.4	2.4	2.2	2.4	2.0	5.5	0.9
TBKBP1	5.0	5.5	1.5	2.2	2.1	1.7	2.8	5.1	1.5
ARID5B	5.7	5.8	1.7	1.3	1.2	1.9	2.1	6.3	1.4
PUS1	4.3	5.2	1.0	2.0	1.8	1.8	3.1	6.7	1.2
TAF11	4.3	6.4	1.5	2.1	1.6	2.4	2.5	5.6	0.9
RB1CC1	4.5	6.1	0.9	2.3	1.5	2.2	1.8	6.8	1.1
GLCCI1	4.5	6.1	1.5	1.6	1.4	1.6	2.1	6.9	1.4
TGIF2-C20orf24	5.2	7.8	0.3	0.5	0.6	2.4	0.9	8.2	1.4
GGH	4.0	6.3	1.1	4.5	3.5	3.8	1.0	2.5	0.4
DDX52	4.4	6.2	1.0	2.6	1.8	2.7	1.9	5.7	0.9
ZNF317	4.9	5.6	1.4	1.9	2.1	2.5	2.2	5.5	1.0
SOX2	3.0	2.7	0.6	0.1	0.5	0.2	5.6	11.2	3.1
LTBP2	4.5	6.4	1.5	2.0	2.0	1.4	2.5	5.8	1.1
WWP1	4.3	6.8	0.9	2.5	2.1	2.4	1.5	5.8	1.0
MYC	6.3	6.8	1.8	3.7	3.2	3.8	0.5	0.8	0.1
TSTD2	4.2	5.7	0.9	2.0	1.6	1.1	2.1	7.8	1.7
CCDC78	5.8	7.5	1.6	1.4	1.1	0.7	3.1	4.7	1.2
C14orf142	3.4	3.5	1.3	2.8	2.3	1.9	3.6	6.6	1.7
NGLY1	4.2	7.0	1.2	1.7	1.5	2.7	1.9	5.9	1.0
IL15RA	5.4	6.0	1.5	1.4	2.0	1.4	2.4	5.9	1.1
BAIAP2L1	5.0	6.2	1.5	3.7	3.1	2.1	1.3	3.5	0.9
PRRT3	4.4	5.5	1.5	2.6	2.7	1.3	2.5	5.5	1.1
MTRF1L	3.9	5.4	1.0	2.1	1.6	2.4	2.5	6.3	1.8
SAMHD1	3.7	6.2	1.0	2.3	1.6	5.0	1.7	4.7	0.9
SZT2	4.6	7.0	1.4	1.6	1.1	1.9	2.1	6.3	1.0
PALM3	4.6	3.8	1.8	4.5	4.1	2.2	2.2	3.3	0.7
NUP160	4.0	6.4	1.1	1.5	1.2	1.6	2.3	8.0	1.0
DSP	4.7	5.9	1.3	2.7	2.6	2.1	1.7	5.1	1.0

MRTO4	4.4	5.7	1.2	2.5	2.4	2.2	1.8	5.7	1.2
CPQ	4.9	7.4	1.2	2.7	1.8	1.9	1.6	4.8	0.8
ZNF805	3.7	5.8	1.3	1.5	1.2	1.5	2.6	8.2	1.1
STOML1	4.0	6.4	1.4	2.7	2.7	1.9	2.2	4.8	1.0
LOC101927060	4.1	5.3	1.5	2.9	2.7	2.4	2.3	4.7	1.2
LOC101927462	4.0	4.9	1.1	3.6	2.6	1.9	2.5	5.4	1.0
BRIX1	3.9	5.7	1.1	2.7	1.9	2.6	2.5	5.6	0.9
AP3M2	4.8	6.9	1.1	2.1	1.7	1.9	2.1	5.5	0.9
STAU2	4.5	6.5	1.0	2.2	1.8	2.6	1.7	5.8	0.9
LMNB1	4.7	5.8	1.4	3.6	3.1	3.1	1.2	3.4	0.6
ITSN2	5.5	6.1	1.0	2.1	1.6	2.5	1.7	5.3	1.2
MRPS9	4.7	5.6	1.1	3.3	2.5	1.9	2.1	5.1	0.7
GNB1L	5.1	5.7	1.5	1.5	1.6	1.8	2.6	5.7	1.4
CKB	5.9	6.1	2.5	3.8	3.5	1.9	0.8	1.7	0.7
CREB3L2	4.1	5.5	1.6	2.0	1.6	1.5	2.3	6.9	1.3
SENP6	4.6	6.1	1.0	2.2	1.7	2.2	2.0	6.0	1.0
ZNF646	4.9	5.5	1.6	2.3	2.2	1.7	2.1	5.5	1.1
KIAA0355	4.8	6.3	1.4	1.6	1.8	1.4	2.3	5.9	1.4
MCM6	4.1	5.8	1.0	3.8	2.8	3.4	1.4	4.0	0.5
FLJ42627	4.3	6.9	1.3	1.5	1.4	2.1	1.9	6.6	0.9
HOXD8	6.0	7.0	1.6	1.5	1.6	1.4	2.0	4.6	1.2
JMJD4	4.2	5.6	0.9	2.5	2.1	2.7	2.7	5.0	1.3
MTMR1	4.2	5.7	1.1	2.7	2.0	2.3	2.3	5.6	0.9
SLFN5	4.6	6.8	1.0	1.0	1.0	2.3	1.8	7.1	1.2
IRF5	5.2	5.6	1.7	2.2	2.3	2.3	2.0	4.2	1.2
BCAT1	4.1	6.4	1.1	1.4	1.1	1.7	2.3	7.9	0.9
MYBL2	3.8	4.3	1.4	6.0	5.8	3.6	0.5	1.3	0.2
RUNX2	6.9	6.7	2.3	1.7	1.4	1.2	1.8	3.8	1.0
LOC284577	4.5	7.6	1.3	1.4	1.0	2.7	1.8	5.8	0.8
ABCD3	3.9	5.0	1.5	2.4	2.1	1.6	2.2	7.3	0.9
LOC101929390	4.6	7.2	1.1	1.5	1.0	3.2	1.7	5.5	1.0
F8	4.4	6.4	0.8	2.5	2.1	3.1	1.5	5.0	0.9
NOMO3	4.6	5.8	1.1	2.0	1.6	1.2	2.3	6.9	1.4
LOC400548	4.6	6.9	1.3	1.3	1.2	2.8	1.8	5.6	1.2
LTV1	4.0	5.6	0.9	2.3	1.9	2.5	2.2	5.9	1.4
RTN1	4.0	5.9	1.2	0.6	0.5	0.4	3.4	8.8	1.9
ELMOD3	3.8	6.4	1.3	1.8	1.8	1.6	2.3	6.5	1.2
RRM2	2.6	2.8	1.3	7.0	7.1	4.2	0.4	1.0	0.3
TOMM40L	3.7	5.8	1.7	3.0	2.5	2.1	2.3	4.8	0.8
RASA1	3.5	6.0	0.9	2.1	1.6	2.4	1.9	7.0	1.2
44810	4.0	4.8	1.3	2.1	2.0	4.6	1.9	5.0	1.0
ATF7	4.9	5.0	1.3	1.9	1.6	1.6	2.4	6.7	1.5
HKDC1	7.7	8.2	2.3	1.8	1.7	1.2	1.3	2.1	0.4
FAM105B	4.1	5.1	1.3	2.1	1.4	2.9	2.3	5.9	1.7
SRSF8	4.4	7.3	1.1	1.4	1.1	3.3	1.8	5.6	0.8
YAF2	3.5	6.7	1.2	1.9	1.7	2.5	2.0	6.5	0.8
TMEM50B	4.1	6.4	1.0	2.3	1.5	2.0	2.2	6.1	1.0

SBF2	4.8	6.5	1.2	1.8	1.7	1.7	1.9	6.2	1.0
ARHGAP27	3.9	5.2	1.5	1.4	1.7	5.8	1.7	4.7	0.8
CREM	3.9	5.8	1.2	2.5	2.2	2.2	2.4	5.5	1.2
LOC101927134	5.0	6.5	1.2	1.8	1.1	2.7	2.0	5.6	0.8
MYLK2	4.4	7.0	1.2	1.6	1.0	3.3	1.7	5.6	0.8
WNT3	4.1	4.4	1.4	2.8	2.8	5.3	1.8	3.1	0.9
ZCCHC10	4.5	6.3	1.7	2.5	2.2	1.8	1.6	5.1	1.0
TMEM180	4.5	5.9	1.7	2.5	2.5	1.7	2.1	4.9	0.8
VPS37D	4.1	5.2	1.7	2.6	2.6	1.7	2.7	4.7	1.4
C21orf119	4.1	6.0	1.7	2.6	1.9	1.5	2.2	5.6	0.9
TFEB	4.0	4.2	1.3	1.8	1.9	2.5	3.2	5.7	1.9
ATXN7	4.2	5.3	1.2	1.7	1.6	2.4	2.4	6.6	1.2
NME7	3.6	6.0	0.7	2.8	1.8	2.8	1.6	6.3	1.0
COG5	4.1	5.9	0.8	2.0	1.8	1.9	2.1	6.9	1.1
PKN3	4.7	5.6	1.5	4.2	4.2	2.5	1.3	2.0	0.5
MTMR10	3.9	5.1	1.0	2.5	2.1	2.8	2.1	5.9	1.1
ZFC3H1	4.6	6.2	0.9	1.8	1.4	3.0	1.9	5.5	1.1
METTL22	4.7	6.2	1.3	2.1	2.5	1.3	2.1	5.3	1.2
SNX14	4.0	6.0	0.9	2.3	1.8	1.9	2.1	6.5	1.1
RNF214	4.8	6.0	1.5	2.1	1.8	1.3	2.2	5.7	1.2
SUCO	2.9	4.7	0.7	1.3	1.3	2.0	2.9	9.1	1.6
CYB5D1	4.0	5.1	1.1	2.2	2.0	1.4	2.4	6.8	1.5
DROSHA	5.1	6.4	1.3	2.1	1.9	1.9	1.8	5.0	1.0
C18orf32	3.7	8.3	0.7	1.6	2.0	1.8	1.3	6.2	0.9
NAT8L	4.6	6.4	1.4	2.6	2.5	1.3	2.3	4.3	1.1
ADARB1	4.6	5.9	1.6	3.2	3.0	1.7	1.4	4.1	0.9
PQLC2	5.1	6.2	1.7	2.0	2.0	1.5	1.9	5.1	0.9
CAMKK1	5.4	6.7	1.9	1.7	1.8	1.1	2.1	4.5	1.3
RRAGC	4.7	5.6	0.9	2.4	2.3	2.9	1.5	5.0	1.0
EVA1B	3.4	4.6	1.3	4.2	3.4	2.3	2.2	4.0	1.0
WDR73	4.5	6.4	1.1	1.9	1.4	1.6	2.4	6.0	1.1
ERLIN1	3.7	5.9	1.0	3.2	2.1	3.1	1.8	5.1	0.6
LINC00913	4.8	5.5	1.4	1.3	1.1	1.5	2.7	6.2	1.8
ABHD17C	5.5	6.1	1.6	2.0	1.8	1.7	1.9	4.9	0.9
LOC101927797	4.3	8.4	1.0	2.2	0.9	1.2	1.6	6.0	0.9
CLCN4	5.2	7.2	1.6	1.8	1.7	2.0	1.6	4.6	0.8
LOC100128593	4.4	7.4	1.5	1.4	1.1	2.2	1.6	6.0	0.8
ARHGAP29	3.7	5.8	0.8	2.5	1.9	1.8	1.7	7.1	1.1
CTPS1	3.8	5.5	1.1	3.3	2.3	3.2	1.7	4.6	0.8
PGAP2	4.2	5.6	1.2	2.4	1.8	1.4	2.5	5.9	1.3
EHD3	5.9	7.3	1.7	1.2	1.4	0.9	2.0	5.1	1.0
DNTTIP2	4.0	5.6	1.0	2.5	1.9	2.9	1.9	5.4	1.2
RNF19B	4.6	6.3	1.5	2.1	1.8	2.9	1.7	4.3	1.1
EEA1	5.1	6.1	1.2	2.5	2.0	2.3	1.6	4.5	1.1
NCAPH2	3.7	5.4	1.0	2.5	1.9	2.8	2.3	5.8	0.9
KIAA0232	5.0	6.6	1.2	2.0	1.5	1.9	1.6	5.7	0.8
KLHDC4	4.4	6.6	1.8	2.1	1.3	2.1	2.2	4.9	1.0

PPIL4	4.8	5.3	0.9	2.0	1.7	2.6	2.1	5.6	1.1
RIPK1	4.3	5.4	1.3	2.2	1.4	1.7	2.4	6.1	1.5
SHANK2-AS1	4.1	7.0	1.4	1.7	1.4	2.2	1.8	5.9	0.9
HDAC4	3.8	4.6	1.5	1.5	1.6	2.1	2.5	7.0	1.8
CERS6	4.0	5.7	0.7	2.8	2.0	2.7	1.9	5.4	1.1
CECR5	4.6	5.6	1.3	2.0	2.3	1.4	2.8	5.2	1.2
GGCT	3.8	5.6	1.1	2.9	2.4	3.7	1.4	4.4	0.8
DOCK4	2.3	3.3	0.6	1.0	1.0	1.0	3.2	12.2	1.8
COMMD10	4.4	6.7	0.8	1.9	2.3	2.3	1.7	5.4	0.9
UTP11L	3.9	6.6	0.9	2.6	2.4	2.2	1.7	4.5	1.5
LSM5	3.3	5.1	1.2	3.6	2.3	2.6	1.8	5.9	0.5
C17orf103	5.1	5.6	1.6	2.4	1.8	1.8	2.0	5.2	0.8
BRPF1	4.2	5.3	1.5	1.7	1.7	2.0	2.6	5.9	1.3
NKTR	4.9	8.4	1.0	1.2	1.1	1.6	1.5	5.7	0.9
FAM109A	4.4	5.2	1.5	2.5	3.0	1.6	2.3	4.6	1.2
ST3GAL3	5.1	6.4	1.5	2.4	1.9	1.8	1.6	4.7	0.8
PISD	4.0	5.2	1.3	1.6	1.7	4.0	2.4	5.1	0.8
PWWP2A	5.4	6.7	1.2	1.9	1.2	1.5	1.7	5.5	1.2
GGCX	3.0	5.2	0.7	3.0	2.4	2.6	2.0	6.2	1.0
CASC10	3.0	3.8	0.9	5.0	4.2	2.4	1.7	4.0	1.1
ABCD4	4.1	5.4	1.2	1.8	1.3	1.7	2.9	6.8	0.9
LOC90784	4.8	6.3	1.3	1.5	1.3	1.3	2.0	6.6	1.3
CCDC24	4.9	5.7	1.7	2.8	3.1	1.3	1.9	3.9	0.9
ABHD6	4.2	5.6	1.0	2.2	1.5	1.3	2.2	6.7	1.4
TFIP11	4.2	5.3	1.1	2.2	1.9	2.4	2.3	5.5	1.2
RHNO1	4.5	6.0	1.5	2.8	2.8	1.8	1.6	4.4	0.7
ZNF667-AS1	4.5	5.1	0.8	2.0	1.9	1.4	2.5	6.8	1.0
IFITM10	5.9	7.3	2.3	1.8	2.1	0.4	1.5	3.9	0.9
STK4	2.9	4.1	0.6	2.4	1.8	3.8	2.3	7.1	1.1
LOC100996702	3.3	4.9	1.0	4.3	4.2	2.8	1.2	3.6	0.8
KDM4A-AS1	4.9	6.5	1.1	1.5	1.3	3.3	1.6	5.1	0.8
ZNF688	4.6	5.1	1.5	2.6	1.8	1.7	2.3	5.1	1.5
DIP2B	4.6	6.7	1.2	1.7	1.6	2.1	1.8	5.5	0.9
ARMC1	3.8	6.1	1.1	2.6	2.1	2.4	2.1	5.2	0.8
ZNRD1	4.7	5.5	1.4	2.4	1.5	2.7	1.9	5.4	0.6
RB1	4.2	5.7	1.1	2.1	1.8	2.2	1.6	6.2	1.3
PREX1	2.8	3.6	0.9	1.7	1.6	8.2	1.8	4.7	0.8
PPP3CC	4.4	6.2	0.9	2.2	1.7	2.5	1.9	5.4	1.0
ECT2	4.1	5.9	0.9	3.4	2.7	3.5	1.0	3.9	0.7
TBC1D3F	5.6	5.5	1.4	1.9	1.5	1.1	2.5	4.9	1.7
LOC101929374	5.2	6.2	2.0	1.7	1.7	0.5	2.1	5.1	1.5
FCHSD1	4.8	5.5	1.4	2.3	2.3	3.5	1.9	3.7	0.7
PVT1	4.7	7.3	1.1	1.8	1.4	2.3	1.7	5.0	0.8
GDF11	4.0	4.4	1.3	2.1	2.4	1.4	2.6	6.4	1.5
LOC401098	4.2	6.8	0.9	1.8	1.2	2.6	1.8	5.8	0.9
MICU2	4.1	5.9	1.2	2.4	1.8	2.3	1.9	5.1	1.2
RRP15	4.4	4.7	1.2	3.1	2.6	2.5	1.9	5.0	0.7

PLXNA2	5.5	6.7	1.7	2.3	2.1	1.3	1.4	4.2	0.8
TBCE	5.1	7.0	1.2	2.3	1.8	2.1	1.4	4.2	0.9
BRD1	4.4	5.6	1.2	1.6	1.8	3.0	2.3	4.9	1.2
VPS37C	5.0	5.4	1.8	2.1	1.7	1.8	2.4	4.8	1.0
UBE4B	4.2	5.7	1.2	2.8	2.1	2.1	1.7	5.0	1.1
PBRM1	4.4	5.5	1.0	1.9	1.6	2.0	1.9	6.7	1.1
PWWP2B	4.6	5.5	1.7	2.2	2.4	2.3	2.4	4.0	1.0
COQ5	4.5	5.9	1.3	2.3	1.8	1.7	2.2	5.4	1.1
CHORDC1	3.7	5.0	1.0	2.2	1.8	3.9	1.7	5.6	1.0
SNX13	4.7	6.8	0.9	1.6	1.5	2.0	1.6	6.0	1.0
TMTC3	3.5	6.7	0.9	2.6	1.8	2.5	1.4	5.8	0.8
CDT1	3.1	4.0	1.2	5.3	5.7	2.4	1.3	2.5	0.6
ZDHHC6	4.0	6.9	0.8	1.8	1.0	1.5	2.2	6.5	1.3
EML4	3.7	5.1	0.9	2.3	2.1	3.2	1.8	5.9	0.9
PPP2R2C	3.7	4.0	1.2	4.1	3.7	3.0	1.8	3.4	1.0
CEP104	3.8	5.8	1.0	2.1	1.9	1.8	2.5	6.0	1.1
UBE4A	4.2	6.4	0.9	2.1	1.7	2.1	1.9	5.6	1.2
KIAA1033	4.4	6.8	1.1	1.8	1.4	2.3	1.7	5.4	1.0
SAMD8	3.5	7.1	1.3	2.3	1.5	1.7	1.8	5.6	1.0
BCCIP	3.1	5.2	1.4	2.7	2.2	2.5	2.6	5.2	1.0
RCC1	4.4	4.9	1.0	2.4	1.6	2.1	2.5	6.3	0.9
ETS2	3.5	3.8	1.1	2.7	2.8	2.8	2.2	5.5	1.4
DHX36	4.3	6.2	1.0	1.9	1.4	1.9	1.9	6.3	1.2
STX17	4.3	5.6	1.1	2.0	1.8	2.3	1.6	6.4	0.8
UPP1	4.9	7.8	0.9	2.7	1.8	2.2	1.5	3.5	0.5
ZNF395	4.1	4.4	1.0	1.5	1.9	2.5	2.5	6.5	1.5
CNOT10	4.1	5.7	1.1	2.1	1.6	1.9	2.3	6.0	1.2
GK5	4.3	6.9	1.2	1.5	1.0	2.7	1.8	5.7	0.9
C1orf21	4.2	5.1	1.4	4.4	4.4	2.3	1.0	2.9	0.3
CTR9	4.3	5.2	1.3	2.0	1.8	2.1	2.3	5.7	1.2
CCDC58	3.5	6.1	1.4	2.4	1.7	2.7	1.9	5.0	1.4
MSL2	3.8	5.7	0.7	2.0	1.6	3.7	1.6	5.6	1.2
EARS2	4.3	5.9	1.3	1.8	1.6	1.1	2.3	6.4	1.2
RC3H1	3.7	5.4	1.2	1.9	1.6	2.4	2.1	6.4	1.1
LOC158960	5.3	5.2	1.4	3.0	2.1	1.6	2.1	4.2	1.0
PCSK7	3.8	4.9	1.1	2.7	2.1	2.7	2.1	5.5	0.9
HTATSF1	3.8	4.6	1.0	2.3	2.0	1.8	2.6	6.4	1.2
ANKRD34A	5.1	6.0	1.8	3.1	3.0	1.7	1.6	2.8	0.8
AASDHPPT	3.9	6.2	1.3	1.9	2.1	2.0	2.6	5.2	0.8
SMCR8	4.1	5.2	1.2	2.1	2.0	1.9	2.0	6.0	1.2
LOC100128398	4.7	6.8	1.2	1.6	1.0	2.3	1.8	5.8	0.7
CREB5	7.2	5.8	1.8	0.5	0.5	0.2	2.4	5.6	1.8
RFFL	4.7	6.6	1.1	1.8	1.6	1.9	1.8	5.2	1.0
LINC00907	4.3	7.0	1.0	1.4	1.0	3.0	1.7	5.7	0.7
NRN1	3.1	4.8	1.1	3.0	2.2	1.0	2.7	6.8	1.1
SLFNL1-AS1	4.3	7.0	1.1	1.3	1.1	3.0	1.5	5.6	0.9
MOSPD2	4.7	7.4	1.0	1.9	1.7	1.9	1.6	4.8	1.0

ANLN	3.1	4.8	1.0	6.0	4.8	4.6	0.3	1.0	0.2
SLC30A1	3.5	5.2	1.2	2.7	2.5	2.7	2.2	4.7	1.0
UBXN2A	4.6	6.7	0.5	2.0	1.2	2.5	1.5	6.1	0.6
NUP133	4.2	6.6	0.9	2.2	1.7	2.4	1.6	5.2	1.0
RHBDD3	4.3	5.7	1.0	2.2	1.8	1.3	2.7	5.7	1.0
FBXL7	3.9	4.9	1.7	2.4	2.7	1.0	2.3	5.7	1.1
FGFR1OP2	4.4	5.1	0.7	1.9	2.2	2.0	2.1	6.2	1.2
KIF13B	4.2	6.0	1.5	1.3	1.2	1.5	2.1	6.6	1.1
EXOC8	3.9	5.4	1.4	2.2	1.7	2.0	2.1	5.7	1.2
MIER1	3.7	4.7	1.2	2.3	2.4	2.5	1.9	5.8	1.3
LOC101928454	3.1	4.6	1.6	2.4	2.1	1.3	2.9	6.8	0.8
VPS13C	3.8	6.1	0.8	1.5	1.2	2.3	1.6	7.2	1.0
FOXO4	4.9	4.5	1.3	1.7	1.8	1.2	2.6	6.1	1.6
LINC00889	4.7	7.3	1.3	1.2	1.1	2.1	1.4	6.0	0.6
ZNF526	3.5	4.5	1.2	1.9	2.3	1.4	2.6	7.0	1.4
NUDT2	3.8	6.2	1.6	3.1	2.0	1.7	1.4	5.4	0.4
QPCTL	3.7	4.6	1.0	2.8	2.3	1.6	2.6	5.9	1.2
HOXA-AS4	4.7	5.0	1.7	1.4	1.5	0.7	2.9	5.8	1.9
SPN	4.9	5.1	1.6	2.5	2.6	2.9	1.8	3.7	0.6
GPR125	3.9	4.9	0.9	2.1	1.6	1.9	2.2	6.8	1.4
STXBP3	3.9	6.0	0.9	2.1	1.3	1.9	1.9	6.3	1.2
HS2ST1	3.6	6.2	1.2	1.7	1.3	1.4	1.8	7.1	1.3
TTPAL	4.1	5.6	1.1	3.0	2.9	2.0	1.4	4.3	1.2
LOC100507501	4.4	6.4	1.2	1.5	1.2	2.7	1.6	5.7	1.0
OLFML2A	4.6	3.9	1.3	3.5	4.0	2.0	1.9	3.4	0.9
PRPF18	4.3	6.0	0.9	1.8	1.5	2.1	1.8	6.2	1.1
DRAM2	4.2	7.5	1.0	2.2	1.5	1.6	1.5	4.9	1.2
PPM1A	3.7	5.5	0.9	1.6	1.5	2.6	2.1	6.4	1.1
ZNF618	4.6	5.4	1.4	1.7	1.9	1.1	2.4	6.0	1.2
LOC101928228	4.0	7.5	1.3	0.7	0.8	2.1	1.5	7.0	0.7
RIOK3	4.1	6.5	1.0	2.2	1.5	2.5	1.5	5.3	1.0
PLEKHA8	4.0	6.6	0.8	1.7	1.5	1.6	2.2	6.1	0.9
UBE2D4	4.8	5.4	0.8	3.1	2.4	1.5	1.3	5.0	1.2
MRPL42	3.6	5.8	1.0	2.7	1.5	2.5	1.3	5.9	1.3
RGS20	2.4	3.5	0.9	1.3	1.4	1.2	3.5	10.0	1.4
LOC101926939	3.1	4.2	1.1	1.1	0.7	0.5	4.2	8.4	2.3
TOP1MT	4.8	4.8	1.6	1.9	2.0	2.0	2.4	4.7	1.3
ZRSR2	4.6	4.8	1.4	1.8	1.5	1.3	2.9	6.1	1.1
LOC101928555	4.4	7.0	1.1	1.8	1.4	2.5	1.6	4.9	0.8
BZW2	3.7	6.2	1.1	2.4	2.0	1.8	1.9	5.8	0.8
NXPH4	4.2	5.2	1.1	2.3	1.9	1.5	2.3	5.8	1.2
STAMBPL1	5.5	9.0	1.2	1.1	0.9	1.1	1.1	5.0	0.7
ZNF827	4.3	6.1	1.5	1.9	2.0	2.0	1.8	5.0	1.0
ZRANB2	3.2	4.8	0.5	1.7	1.6	1.9	2.3	8.0	1.5
CDK11B	4.5	4.5	1.2	1.9	1.9	2.3	2.8	5.1	1.3
CXorf38	4.3	6.4	1.3	2.0	1.6	3.4	1.8	3.7	1.0
FRA10AC1	5.3	5.5	1.0	1.8	1.3	2.0	1.8	5.9	0.9

OTUD1	3.9	3.7	1.5	2.4	2.0	3.6	2.6	4.5	1.3
METTL6	4.1	6.1	1.5	2.2	2.0	1.5	1.9	5.3	0.8
ASNSD1	3.9	6.1	0.7	2.3	1.5	2.4	1.8	5.8	1.0
RBM4B	3.9	4.6	1.0	1.3	1.3	1.5	3.4	7.0	1.5
IFNAR1	4.0	5.4	0.6	2.5	1.6	2.2	1.7	6.3	1.3
MED4	3.4	5.7	1.0	2.3	1.9	1.7	2.0	6.2	1.3
HIST1H3A	3.1	5.3	1.2	3.6	4.0	3.6	1.4	2.7	0.6
PABPC1L	4.9	5.3	1.2	2.2	1.9	1.4	2.2	5.0	1.2
YEATS2	4.3	5.4	1.3	1.9	2.0	1.8	1.8	5.9	1.1
CCDC106	5.0	5.4	1.8	2.4	1.9	1.2	2.5	4.1	1.3
BIRC2	3.1	6.1	0.7	2.2	1.7	2.4	1.7	6.6	1.0
CKMT2-AS1	4.2	6.2	1.1	1.6	1.2	3.6	1.5	5.2	0.9
NGDN	4.5	5.8	1.1	2.1	1.6	1.6	2.1	5.3	1.3
CBLL1	5.3	5.5	1.2	2.6	1.7	1.3	2.0	4.7	1.1
LINC00243	4.3	7.4	1.3	1.3	0.8	2.5	1.5	5.6	0.7
KDELR3	3.9	5.4	1.1	1.3	1.4	1.9	2.2	7.0	1.1
RAB30	4.5	6.2	0.9	1.9	1.0	1.2	2.2	6.0	1.6
UBE3A	3.9	5.8	0.9	2.0	1.4	2.6	1.8	6.0	1.0
LOC100129361	4.4	4.2	2.1	2.1	1.4	1.6	2.3	5.5	1.9
VEZT	3.7	6.4	0.8	2.8	2.3	2.7	1.3	4.6	0.8
MKL2	4.2	4.8	1.1	2.2	1.6	1.3	2.1	6.6	1.3
STC1	1.7	3.4	0.6	1.3	1.4	1.0	3.7	10.1	2.3
MRPS17	3.8	5.3	1.0	2.2	1.7	2.2	1.8	6.0	1.4
MKNK1	4.5	5.7	1.2	1.9	2.0	1.8	2.2	5.1	0.9
EMG1	3.5	5.5	1.3	2.8	2.3	2.7	1.8	4.6	0.8
RACGAP1	3.3	5.2	1.0	4.0	3.8	3.2	1.3	3.1	0.6
DNAJC1	3.7	3.9	1.4	1.9	1.7	1.3	3.0	7.3	1.2
C1orf216	3.7	4.0	1.2	2.4	2.8	2.6	2.6	5.0	1.2
ZFYVE19	4.0	4.4	1.0	2.8	2.5	1.5	2.2	5.8	1.0
COG2	3.8	6.2	1.0	2.0	1.4	1.8	2.2	5.9	0.9
UBR3	4.0	6.4	0.9	1.9	1.5	1.8	1.9	6.0	1.0
SERHL2	4.2	5.3	1.3	1.9	1.6	2.3	2.0	5.7	0.9
NLK	4.2	5.4	1.2	2.0	1.6	1.5	2.1	5.8	1.3
ALDH1B1	3.4	4.7	1.0	4.3	3.7	2.2	1.8	3.5	0.8
HAUS7	4.3	4.8	1.3	2.0	1.9	1.4	2.6	5.8	1.3
ZCCHC6	4.2	5.8	1.0	1.4	1.1	2.4	1.9	6.4	1.1
MTIF3	3.9	5.8	0.9	2.6	2.1	1.7	1.7	5.4	1.2
ANKRA2	4.8	5.6	1.0	1.9	1.2	1.6	2.2	5.5	1.4
GTF2A1	4.5	4.9	1.3	2.0	1.8	1.0	2.6	5.9	1.3
LOC101928150	4.2	6.2	1.1	1.6	1.0	3.6	1.7	5.1	0.7
TAB1	3.7	6.0	1.5	1.7	2.1	2.1	2.1	5.1	1.0
MDC1	4.2	5.2	1.4	1.9	2.0	1.2	2.2	5.9	1.2
PAQR8	2.2	2.0	0.6	2.0	1.6	0.7	4.2	9.7	2.4
BLOC1S1-RDH5	4.7	3.9	1.6	2.0	1.8	1.0	2.8	5.8	1.7
SASH1	3.9	5.2	1.1	2.7	2.6	1.7	1.9	5.0	1.1
SAP30	4.8	5.3	1.7	2.3	2.8	3.0	1.4	2.9	1.1
GEMIN7	3.8	5.8	0.8	2.2	1.6	1.6	2.0	6.1	1.3

DNAL4	4.3	6.4	1.2	1.3	1.7	1.1	1.9	6.2	1.1
FAM35A	5.1	7.1	0.9	2.1	1.4	1.8	1.5	4.4	1.0
KIAA0196	4.5	6.5	1.1	2.5	2.1	2.2	1.3	4.1	0.9
LRP12	3.9	6.4	0.7	2.3	1.8	1.7	1.8	5.8	0.8
TOX2	4.1	3.9	1.1	1.4	1.7	1.2	3.0	7.2	1.4
LOC101926954	4.2	6.0	0.8	1.7	1.3	1.7	1.8	6.5	1.3
CEP250	5.6	5.5	1.8	2.0	1.8	2.0	1.8	3.8	0.8
SLC25A42	3.2	5.2	1.0	1.8	1.9	1.9	2.5	6.0	1.6
BTD	3.3	4.9	1.1	2.4	2.0	1.7	2.2	6.5	1.0
ISY1-RAB43	11.1	1.6	1.9	0.9	4.1	0.2	3.7	0.8	0.9
HYKK	4.4	7.0	1.2	1.5	1.0	2.4	1.5	5.4	0.7
CNEP1R1	3.8	5.9	1.1	2.0	1.6	1.3	2.2	6.5	0.8
LOC255177	4.2	7.2	1.0	1.3	1.0	2.2	1.6	5.8	0.8
SLC30A6	2.8	5.2	0.9	2.3	1.9	1.8	2.0	6.8	1.3
CTF1	5.1	5.9	1.8	2.3	1.8	1.5	2.0	3.1	1.6
MAPK8IP2	4.9	5.2	1.5	3.6	3.2	2.1	1.3	2.7	0.5
NUP155	4.0	6.3	1.0	2.6	2.1	2.6	1.4	4.4	0.6
PHF3	4.0	5.5	1.0	2.0	1.6	2.4	1.7	5.7	1.1
CEPT1	4.7	6.8	0.9	1.7	1.3	1.7	1.7	5.2	1.0
DNAJC19	3.3	6.0	1.3	2.6	1.5	1.8	2.0	5.2	1.4
CMTM8	4.3	4.4	1.5	1.7	1.2	1.0	2.5	6.2	2.2
MPND	4.3	4.5	1.7	2.3	2.5	1.7	2.3	4.7	1.2
C17orf51	2.4	3.1	1.0	1.9	1.6	0.8	3.3	8.4	2.7
NBPF9	4.3	5.4	1.0	2.4	1.7	1.6	2.0	5.7	1.0
44628	3.7	5.0	1.2	2.2	1.9	1.3	2.4	6.0	1.3
TMEM38A	4.1	5.0	1.3	3.1	2.3	1.5	1.8	5.0	1.0
NPTXR	4.0	6.0	1.7	1.5	1.4	0.5	2.6	5.8	1.5
PRKRIR	4.1	6.3	0.7	1.6	1.2	1.9	1.9	6.1	1.2
CBR4	4.5	6.2	0.8	1.7	1.6	1.8	2.0	5.3	1.0
CREBL2	3.0	5.1	0.7	3.5	1.4	2.8	2.4	5.2	0.9
ACOX3	4.3	5.7	1.2	2.6	1.9	1.9	1.7	5.0	0.7
FZD8	2.2	3.4	1.1	1.5	1.7	0.5	3.6	9.0	2.0
SLC25A36	3.8	7.0	0.7	2.3	1.4	2.5	1.8	4.5	0.9
NUDT14	3.8	5.1	1.0	2.6	3.0	2.5	2.1	3.8	1.2
ANKRD42	3.1	5.0	1.1	1.9	1.2	1.2	2.5	7.9	1.2
TPCN1	4.7	5.7	1.4	1.9	1.8	1.8	2.0	4.4	1.2
SNORA44	1.3	5.2	2.3	1.4	1.5	0.7	6.2	5.9	0.5
TVP23C	4.1	5.8	1.0	1.7	0.8	1.3	2.4	6.9	0.8
FUNDC1	5.3	5.9	1.7	2.3	2.3	1.7	1.6	3.7	0.6
PLAGL2	3.7	4.5	1.1	2.0	2.5	1.6	2.4	5.5	1.5
LOC284950	4.3	6.1	1.1	1.5	1.0	2.6	1.8	5.6	0.9
NUP50	3.5	4.8	0.9	3.1	2.4	2.8	1.5	5.1	1.0
SMYD2	3.4	4.9	1.0	2.8	2.7	2.7	1.6	5.0	0.8
HYAL3	3.5	4.4	1.0	3.7	2.2	1.4	1.9	5.7	1.1
SMC6	3.7	5.5	0.9	2.2	1.7	2.8	1.8	5.3	1.0
VPS11	3.7	5.4	1.1	2.4	2.2	2.0	1.9	5.2	1.0
RPP30	3.7	6.3	0.9	2.2	1.9	2.5	1.7	4.4	1.3

COX16	3.7	5.5	0.6	2.2	1.8	3.6	1.6	5.2	0.6
CEBPZ	4.0	5.6	0.7	1.8	1.4	2.0	2.1	6.0	1.3
DHDDS	3.9	4.9	1.0	2.3	2.1	1.9	2.3	5.7	0.8
GPC6	2.2	2.6	0.5	0.4	0.3	0.2	3.9	12.7	2.0
LOC100506119	5.1	7.4	1.5	1.3	1.0	1.7	1.4	4.9	0.6
DECR1	3.2	5.5	1.1	2.6	1.9	3.3	1.5	5.0	0.7
ACYP2	3.3	6.2	1.4	1.3	1.8	1.0	2.8	5.9	1.1
THAP3	3.7	6.4	1.6	2.5	2.1	1.0	1.9	4.5	1.2
ARFIP1	3.0	7.7	0.7	1.4	1.4	1.7	1.8	5.8	1.5
RPS6KC1	3.6	5.9	1.2	1.7	1.3	1.8	2.0	6.4	1.0
CCDC22	4.2	5.0	1.3	1.7	1.5	1.7	2.5	5.9	0.9
FOXRED2	4.5	5.1	1.4	2.9	2.7	1.4	1.9	4.0	1.0
SNX7	4.0	5.7	1.0	2.7	1.6	2.2	1.8	5.3	0.6
RAB3GAP2	3.6	5.6	0.9	1.9	1.4	2.1	2.0	6.4	1.0
COX10-AS1	4.3	6.1	1.3	1.4	1.0	1.7	1.8	6.3	1.0
KCTD1	4.8	5.6	1.8	2.0	2.5	1.2	1.9	3.9	1.1
ASF1B	2.9	3.9	1.0	5.3	5.2	4.7	0.4	1.3	0.1
CDCA4	4.3	5.9	1.1	2.1	1.6	1.9	1.9	5.2	0.7
RAB3A	4.0	4.1	1.2	2.1	1.6	0.8	3.0	6.6	1.4
SMIM20	4.8	4.6	1.4	2.8	2.7	1.9	2.6	3.3	0.8
PCNXL4	4.3	7.0	0.9	2.0	1.6	2.0	1.3	4.9	0.9
CHTF18	4.0	4.7	1.1	3.3	3.2	2.3	1.8	3.5	0.9
PARD6A	3.8	4.1	1.5	2.1	2.2	2.0	2.7	5.1	1.4
MAPT	4.7	5.2	1.9	2.3	2.1	1.5	1.8	4.3	1.1
LOC101927033	4.8	6.0	0.9	2.0	0.7	2.2	1.4	5.9	1.0
GEMIN5	3.9	5.8	1.1	2.1	1.9	1.6	1.9	5.7	0.9
PRADC1	2.6	4.3	1.0	2.9	2.7	1.9	2.4	6.0	1.1
UTP3	4.4	4.8	1.2	2.1	1.9	1.5	2.5	5.5	0.8
GTF2H3	4.0	7.3	0.8	2.0	1.7	1.8	1.4	4.8	0.9
FAM229B	4.3	5.4	1.3	2.9	1.1	0.6	2.4	5.7	1.2
BRF1	3.8	5.8	1.3	1.6	1.5	2.4	2.2	5.3	0.9
SSBP3	4.0	4.0	1.3	1.8	2.1	1.9	2.4	5.6	1.5
SYS1	3.5	3.8	1.0	2.5	2.6	1.0	2.4	6.3	1.7
PPAP2B	2.7	3.6	0.8	2.4	2.0	1.6	2.9	7.2	1.7
LOC101928053	4.1	6.4	1.3	1.4	1.0	2.1	1.8	5.8	0.8
C8orf76	3.2	5.4	1.0	2.2	1.8	2.9	2.6	4.0	1.7
ABCC4	4.0	6.4	0.8	1.9	1.4	1.9	1.6	5.9	0.9
PANX1	5.1	6.2	1.1	1.6	1.2	1.4	1.7	5.4	1.1
TAF12	3.9	5.8	1.0	1.4	2.1	1.7	2.3	5.5	0.9
FAM92A1	3.8	5.6	1.3	3.2	2.0	2.1	1.4	4.1	1.1
MMP17	4.9	5.6	1.6	1.7	1.8	1.2	2.2	4.6	1.2
PDE12	3.5	5.0	1.2	2.4	2.0	2.4	2.1	5.1	1.1
SELK	3.0	5.0	0.5	1.8	1.4	4.1	2.4	5.9	0.7
ZSWIM1	4.2	4.9	1.4	2.0	2.1	0.8	2.0	6.1	1.2
DIS3	3.7	5.8	0.9	1.8	1.3	1.5	2.1	6.4	1.1
BCS1L	4.4	5.6	1.2	2.3	1.8	1.7	1.9	4.9	0.9
C11orf95	2.8	3.8	0.9	1.7	1.6	1.1	3.6	7.5	1.6

ZNF770	3.9	5.6	0.7	2.1	1.7	2.4	1.4	5.8	1.0
SFT2D3	3.9	5.7	1.8	1.4	1.5	2.1	2.8	4.4	1.0
MIR143HG	4.3	6.9	1.0	1.4	1.0	2.7	1.5	5.1	0.8
RFX7	3.8	5.5	1.1	1.8	1.7	1.4	1.9	6.4	1.1
HPN-AS1	3.9	6.6	1.2	1.3	1.0	2.1	1.7	5.8	0.9
MLLT4	3.9	5.6	1.1	2.0	1.7	1.8	2.0	5.6	1.0
LOC728769	5.1	6.6	1.2	1.2	1.2	1.9	1.7	5.1	0.6
HOOK2	5.1	5.0	1.3	2.1	2.0	1.5	2.1	4.4	1.1
KRIT1	4.6	6.5	0.7	1.4	1.0	1.4	1.8	6.5	0.8
MMP1	3.2	8.2	1.3	0.1	0.0	0.3	2.1	8.0	1.3
MOB3C	4.3	4.9	1.5	1.7	1.7	2.1	1.9	4.8	1.5
TAMM41	4.7	6.8	1.8	1.3	1.3	1.0	1.8	4.3	1.4
MACROD1	5.5	6.3	1.6	2.1	1.8	1.1	1.8	3.3	1.2
ATPAF2	4.9	5.6	1.1	2.0	1.8	1.5	2.1	4.4	1.0
OPA3	4.6	4.7	1.7	1.9	1.9	1.7	2.2	4.4	1.4
RTCA	3.6	5.7	0.7	2.2	1.9	2.2	1.8	5.4	1.0
SPATA33	3.8	4.3	1.3	2.5	2.3	1.9	1.7	5.3	1.3
FHOD3	4.1	5.2	1.2	1.9	1.6	1.4	2.3	5.7	1.1
LOC100862671	4.8	6.9	1.3	2.1	1.4	1.1	1.5	4.4	1.0
LINC00671	4.2	6.9	1.2	1.2	1.0	2.0	1.4	5.5	0.9
ZSCAN12	4.4	6.1	1.2	1.9	1.3	2.2	1.5	5.0	0.9
APBB3	4.4	6.3	1.0	1.1	0.9	1.4	2.4	5.6	1.4
NFKBIE	3.9	5.3	1.4	2.1	2.0	3.3	1.6	3.9	0.9
FRMD6-AS1	3.2	4.9	0.9	1.4	1.0	6.7	1.4	4.0	0.9
CCRN4L	4.3	5.6	1.4	2.5	2.2	1.3	1.6	4.6	1.2
LIMD1	3.6	4.5	0.8	2.0	2.2	3.0	2.0	5.4	0.9
LOC101929047	3.6	6.5	0.9	2.1	1.1	1.5	1.9	6.0	0.8
KIAA1432	3.7	6.0	0.8	1.8	1.4	1.7	2.0	6.2	0.9
PCAT1	4.2	5.9	1.2	1.6	1.2	2.7	1.7	5.1	0.8
GLI3	3.2	3.7	1.1	1.8	1.9	0.9	2.8	7.4	1.6
MRPL39	3.2	6.1	0.9	2.2	2.1	1.7	1.9	5.3	1.1
IL18	5.0	8.2	0.9	3.2	2.3	2.1	0.6	1.7	0.4
HDHD1	3.1	5.1	1.4	2.1	2.3	2.1	1.6	5.6	1.0
ZNF337	4.3	6.7	1.1	1.4	1.0	1.6	1.8	5.6	1.0
PHF11	3.9	5.5	1.2	2.5	1.5	3.2	1.7	4.2	0.9
TEN1-CDK3	4.7	5.4	1.3	1.5	1.3	0.9	2.8	5.4	1.2
CSRNP2	3.8	5.5	1.3	1.9	1.6	1.8	2.1	5.4	1.1
PRMT7	3.7	5.3	1.1	1.9	1.7	1.5	2.5	5.5	1.2
SAMD4A	4.5	4.9	1.2	3.3	3.2	2.1	1.3	3.2	0.7
PHF21A	3.9	4.5	1.2	1.7	1.6	1.4	2.4	6.2	1.4
KCNH2	3.1	3.6	1.1	2.6	2.8	1.8	2.5	5.1	1.8
C15orf39	3.4	3.5	1.1	1.4	1.5	2.9	2.5	6.7	1.5
POLR2M	3.6	5.1	0.9	2.3	2.5	2.0	1.8	5.0	1.2
CMSS1	3.8	5.0	1.3	2.4	2.1	2.1	1.7	4.7	1.1
SCRN3	4.4	5.7	0.8	2.3	1.4	1.8	1.7	5.2	1.0
LOC101927842	3.7	6.6	1.1	1.3	0.9	2.7	1.7	5.7	0.7
LMO7	5.3	6.8	1.4	3.3	2.5	2.4	0.7	1.7	0.3

BOD1L1	3.8	4.5	1.1	1.5	1.2	1.4	2.3	7.3	1.2
CRIPT	2.4	4.9	0.3	2.0	1.2	3.0	2.1	6.9	1.6
SMAD4	3.7	5.4	1.0	1.5	1.4	1.6	2.1	6.5	1.0
MKLN1	3.6	5.0	0.9	2.3	1.9	2.3	1.9	5.3	1.1
SC5D	4.2	6.0	1.1	1.8	1.8	2.0	1.7	5.1	0.7
LOC101929451	3.1	5.5	1.4	2.5	2.3	0.8	2.2	5.6	0.8
MED27	4.0	5.5	1.3	2.2	1.6	1.9	2.4	4.7	0.9
DOPEY2	3.8	5.3	1.1	2.4	2.2	2.1	1.6	4.9	0.8
USP46	4.8	6.5	1.1	2.1	1.4	1.1	1.4	4.6	1.3
DHX29	4.0	5.7	0.9	1.8	1.3	1.7	1.8	6.0	1.1
LSP1	2.0	2.1	0.6	1.1	1.0	13.8	1.0	2.2	0.5
CLUAP1	4.5	4.2	1.2	2.2	2.5	1.9	1.8	4.7	1.3
MBD1	3.3	4.4	1.3	1.9	1.8	2.3	2.4	5.4	1.4
RNF170	3.8	5.5	0.8	1.7	1.2	2.4	1.8	6.2	0.9
ITPKC	4.3	5.1	1.3	2.0	1.7	1.6	2.2	5.1	1.0
ADSSL1	5.5	7.7	1.7	1.2	0.9	0.8	1.7	4.1	0.7
SNORD3D	3.8	4.1	3.8	2.5	1.2	3.1	2.8	2.3	0.6
SKIV2L2	3.8	6.3	0.9	1.9	1.2	2.1	1.7	5.5	0.9
ZC3HAV1	3.3	4.7	1.1	1.4	1.3	4.8	1.6	5.1	1.0
TRAPPC9	3.8	5.4	1.1	1.9	1.7	2.2	2.1	5.0	1.1
RAP1GDS1	3.8	6.4	0.7	2.3	1.5	2.1	1.5	4.9	1.1
MET	3.3	5.0	1.0	4.1	3.3	3.8	0.7	2.5	0.5
OSBP2	5.6	7.7	2.1	1.5	1.7	0.9	1.3	2.8	0.6
CCDC85A	6.0	5.9	2.4	2.8	2.4	1.6	1.0	1.7	0.3
SPEN	3.9	4.6	1.3	1.7	1.7	2.5	1.9	5.6	1.0
LOC729732	4.3	6.7	1.3	1.4	1.2	1.8	1.7	5.3	0.7
HDDC3	4.6	5.1	1.7	2.4	1.8	1.8	1.9	4.2	0.7
NOM1	4.7	4.7	1.5	1.8	1.5	1.8	2.5	4.9	1.0
PPHLN1	3.5	5.7	1.3	2.5	1.8	2.3	1.6	4.7	0.8
VAT1L	3.0	5.1	1.1	0.3	0.3	0.3	3.4	9.3	1.3
NAT6	3.9	4.7	1.6	2.2	2.4	1.5	2.2	4.2	1.3
PEX6	4.2	5.6	1.1	1.9	1.7	2.9	1.7	4.1	1.0
SMIM13	3.8	4.0	1.9	2.2	1.7	2.0	2.6	5.4	0.5
DNAJC2	3.8	4.2	1.2	1.8	1.6	2.3	2.2	5.8	1.2
GCDH	3.6	5.5	1.4	2.5	2.0	2.1	1.9	4.4	0.8
DOCK11	5.4	7.9	1.1	1.4	1.1	2.4	0.9	3.3	0.6
LOC101928464	3.8	5.6	1.5	1.4	1.3	2.6	1.5	5.6	0.9
PHF20	4.1	5.6	1.2	2.0	1.6	2.0	1.6	5.0	1.0
ZFYVE9	3.7	5.6	1.0	2.3	1.8	2.0	1.5	5.4	0.9
SIDT2	4.1	5.6	1.3	1.5	1.5	1.7	2.0	5.4	1.2
N4BP1	3.3	4.9	0.7	2.3	1.7	2.6	1.8	5.6	1.2
ZNF543	3.7	5.2	1.4	1.6	1.1	0.8	2.1	7.0	1.2
RBM6	3.9	5.0	1.2	1.6	1.2	1.7	2.2	6.2	1.1
NFKB1	3.5	4.5	0.9	2.0	1.7	3.6	1.8	5.4	0.8
WDR12	3.9	6.5	1.0	2.4	1.5	1.4	1.6	5.0	0.7
PBDC1	2.8	5.2	0.6	2.1	1.4	1.5	2.8	6.7	1.0
LOC100507419	5.3	5.7	1.7	1.9	1.5	1.8	1.6	3.2	1.3

GTF2IRD2B	3.9	5.9	0.8	1.5	1.2	0.8	2.0	6.9	1.1
LOC100506596	5.0	6.8	1.9	1.4	1.3	1.1	1.7	4.2	0.7
LOC101927274	4.5	6.9	1.3	1.0	1.3	1.9	1.5	5.0	0.8
LOC101928401	4.2	6.5	1.0	1.4	0.9	2.8	1.5	5.3	0.6
C3orf38	3.5	5.0	1.0	2.4	1.8	2.1	1.8	5.5	1.1
A1BG	3.8	6.8	1.3	1.4	1.3	1.0	2.4	5.0	1.0
CDK17	4.6	5.5	1.0	1.8	1.5	2.1	1.4	5.1	1.1
CAMTA1	2.7	6.0	0.9	2.4	1.8	1.7	2.3	5.4	1.0
HOXD9	5.0	6.6	2.1	1.6	1.3	0.7	1.8	4.0	1.0
FAM118A	3.8	6.0	0.9	2.0	1.3	1.9	1.8	5.8	0.7
CUTC	4.7	6.0	1.3	1.9	1.3	1.3	2.2	4.5	0.9
CAPN7	4.1	5.8	1.0	1.7	1.4	1.8	1.9	5.3	1.1
DCAF13	3.3	4.6	0.8	1.8	1.7	1.6	2.5	6.7	1.1
ZSWIM6	4.9	6.2	1.7	1.7	1.5	1.3	1.6	4.3	0.9
ZNF783	5.4	6.1	1.2	1.3	1.2	0.7	2.4	4.9	0.9
RPP25L	3.1	5.4	0.9	2.1	2.5	2.3	2.0	4.9	0.8
SNORA14B	1.8	8.8	1.0	1.7	0.9	3.7	1.6	4.1	0.5
ILKAP	3.8	5.2	1.1	1.5	1.7	3.0	2.1	4.8	0.8
RNF111	4.0	5.5	1.1	1.9	1.7	1.6	1.8	5.5	0.9
ACP6	3.7	5.1	0.7	2.3	2.2	1.9	2.0	5.3	0.9
LOC101929007	4.1	6.3	1.1	1.4	0.9	2.7	1.4	5.4	0.7
BEND7	4.8	5.8	1.2	2.5	2.2	1.4	1.4	3.8	0.7
FBXO34	3.9	5.7	1.0	1.4	0.9	3.2	1.9	5.3	0.7
DES	6.7	7.6	2.0	2.5	2.6	1.5	0.4	0.5	0.1
CARD8	3.9	6.5	1.0	1.5	1.2	2.0	1.8	5.2	0.8
AK4	5.0	7.1	1.1	2.6	1.3	2.5	0.7	2.7	0.9
TRAF3	3.8	4.7	1.2	2.3	2.0	2.1	1.7	5.2	1.0
RASAL2	4.0	5.2	1.1	2.9	2.5	2.1	1.3	4.0	0.8
ZKSCAN8	4.1	5.0	1.1	1.7	1.2	1.8	2.3	5.7	1.0
CSTF3-AS1	3.6	5.8	1.4	1.8	1.6	1.7	2.2	5.1	0.9
TMEM254	4.7	4.4	0.9	2.1	1.4	1.7	2.3	5.5	0.9
NLRX1	4.2	5.4	1.4	2.6	2.3	1.9	1.7	3.6	0.7
EPC2	3.9	4.7	0.9	1.5	1.4	1.8	2.0	6.4	1.4
TSPAN5	3.5	6.3	1.1	2.0	1.5	2.1	1.8	4.9	0.7
PPP2R1B	3.4	6.3	1.0	2.1	1.5	2.3	1.5	5.0	0.8
LOC729083	5.5	7.7	1.0	1.5	1.5	1.2	1.1	3.6	0.8
MFSD1	3.4	5.5	0.9	2.1	1.5	1.6	2.1	5.9	1.0
SNRNP35	4.6	3.7	1.2	2.1	2.8	1.8	2.3	4.4	1.1
SEMA5A	3.1	4.3	0.9	1.4	1.2	0.6	2.6	8.5	1.4
ATP2A3	1.4	1.8	0.5	0.8	0.8	16.7	0.5	1.1	0.2
C6orf136	4.3	4.6	1.4	2.1	1.9	1.7	2.0	4.2	1.6
STK38L	4.4	5.3	0.8	2.1	1.4	2.5	1.6	4.8	1.0
TOP3A	3.4	5.3	0.8	1.8	1.8	1.9	2.3	5.5	1.1
CEP89	4.5	4.5	1.1	2.2	1.9	1.6	2.0	4.8	1.2
RUFY2	4.4	5.9	1.0	1.8	1.4	1.4	1.4	5.5	1.0
SPC24	3.2	2.6	1.1	6.8	5.6	3.4	0.2	0.8	0.1
TDRD7	3.3	5.3	0.8	2.3	2.0	2.4	1.5	5.3	0.9

AFTPH	3.4	4.5	0.8	2.0	1.8	2.3	1.7	6.1	1.2
UFM1	2.9	4.5	0.7	1.5	0.4	2.2	3.2	7.5	0.8
KAZALD1	4.5	5.3	1.2	1.8	1.7	1.0	2.2	5.0	1.0
LINC00632	3.8	6.7	1.0	1.1	1.0	3.1	1.5	4.7	0.9
ZCCHC7	3.9	4.9	0.7	1.5	1.2	1.4	2.2	6.9	1.1
S100PBP	4.1	5.7	0.8	1.6	1.4	1.3	1.9	6.2	0.8
LEPREL2	4.7	5.8	1.3	3.0	2.0	1.8	1.4	3.3	0.5
RP9	3.2	3.8	1.2	1.4	2.3	1.7	3.3	5.5	1.4
CNST	3.7	5.0	1.0	2.0	1.6	2.6	1.9	5.0	1.0
LYSMD2	4.2	4.3	1.6	2.4	2.6	1.9	2.3	3.2	1.1
LOC100506100	4.0	6.1	1.1	1.9	1.8	1.1	1.7	4.6	1.5
DENND5B	4.2	5.9	1.1	1.8	1.3	2.1	1.6	5.0	0.8
LUZP1	4.2	5.3	1.1	2.2	2.1	1.6	1.6	4.9	0.8
SRPK3	7.5	8.8	2.0	0.7	0.6	0.4	1.1	1.9	0.7
ZMYM4	3.9	4.9	0.7	2.1	1.8	2.1	1.6	5.4	1.0
VPRBP	3.8	5.1	0.9	1.6	1.4	1.3	2.2	6.1	1.2
PROSER2	5.8	6.4	1.8	2.2	2.3	1.5	1.1	1.9	0.6
TRIM33	3.7	5.3	1.0	1.9	1.6	2.0	1.9	5.2	0.9
TMC6	3.3	4.6	1.2	2.7	2.4	6.3	0.8	1.6	0.7
ACBD4	4.6	5.2	1.4	2.7	1.6	1.7	1.6	4.0	0.8
LGALSL	5.5	6.3	0.8	1.5	1.8	1.5	1.5	4.1	0.6
ABHD15	3.4	4.3	1.2	2.7	2.2	2.8	1.9	4.2	1.0
BCAS3	4.3	5.0	1.2	1.9	1.7	1.5	1.9	5.3	0.9
MOCS3	3.2	5.1	1.1	2.2	1.8	1.9	2.2	4.9	1.1
TIMP4	2.5	3.6	0.6	3.6	2.8	1.6	2.0	5.9	1.1
UHRF1BP1	4.0	5.5	1.2	1.9	1.6	1.6	1.6	5.3	0.8
RAP1A	3.2	5.7	1.0	2.3	1.3	3.5	1.5	4.6	0.6
TTC13	3.4	5.7	0.9	1.8	1.1	1.9	2.1	5.9	0.9
RGS12	4.2	5.8	0.9	1.5	1.7	1.3	1.9	5.1	1.2
DENND1B	4.0	6.3	1.2	2.0	1.5	1.7	1.4	5.0	0.6
ELFN1	5.4	4.8	2.0	1.4	2.1	0.7	2.2	3.8	1.2
FOS	0.2	0.4	0.0	0.1	0.1	22.1	0.2	0.5	0.1
CLASP2	3.6	5.4	0.9	1.7	1.6	1.9	1.7	5.7	1.1
PRDM4	4.2	5.0	0.8	2.2	1.9	1.7	1.7	5.2	1.0
SGTB	4.5	6.2	0.8	2.3	1.7	1.1	1.6	4.8	0.5
TRPC1	4.0	6.6	1.1	2.1	1.8	1.6	1.3	4.3	0.8
PQLC3	3.3	6.3	1.2	2.7	2.7	2.5	1.0	3.3	0.8
MSRA	3.1	4.5	0.8	2.1	2.2	1.8	2.0	5.4	1.7
C9orf116	2.7	3.6	0.8	1.8	2.4	1.6	2.8	6.1	1.8
VPS33A	3.9	5.2	0.8	2.1	1.2	1.9	2.1	5.3	1.0
SLC15A3	4.8	4.6	1.2	1.9	1.9	2.7	1.8	3.8	0.8
FAM122B	4.3	5.3	1.2	2.3	2.6	2.1	1.5	3.6	0.5
HMG20A	3.7	4.7	0.9	1.8	1.5	1.4	2.3	5.8	1.3
TMEM80	3.3	5.0	0.9	1.7	1.6	1.7	2.5	5.7	1.1
HPS5	3.4	4.8	0.6	3.6	2.1	2.8	1.3	4.2	0.7
HIST1H3J	3.3	3.7	1.0	2.7	2.8	4.9	1.2	3.4	0.6
NDRG4	3.7	4.5	1.0	1.7	1.6	1.3	2.2	6.2	1.2

TMEM60	3.9	5.5	0.7	2.2	2.3	2.4	1.1	4.7	0.8
GTF3C3	4.2	6.7	0.9	1.7	1.3	2.0	1.3	4.5	0.9
FAM60A	3.6	5.5	0.8	2.0	1.3	2.6	1.9	5.3	0.5
RNMTL1	3.7	5.3	1.1	2.1	2.2	2.3	2.1	4.0	0.7
U2AF1L4	3.9	4.8	1.1	1.7	1.6	1.6	2.3	5.3	1.2
BOLA1	3.9	5.3	1.1	2.4	2.2	1.9	1.8	4.3	0.7
SUFU	3.9	4.7	1.2	1.9	1.8	1.5	1.9	5.2	1.4
EBF4	4.4	4.8	1.4	2.5	2.4	2.0	1.5	3.5	1.0
RASSF2	2.8	4.2	0.9	2.1	2.4	4.0	1.6	4.4	1.0
GSN-AS1	3.8	5.5	1.1	1.8	1.6	1.3	2.1	5.4	0.9
UPK3BL	4.0	6.0	1.3	1.6	1.3	0.9	2.2	5.3	0.9
RABL2A	4.6	5.6	1.2	1.8	1.2	1.0	2.0	5.1	0.9
CHAF1A	3.3	3.9	1.1	4.0	3.9	2.9	1.1	2.7	0.6
TMEM64	3.2	5.3	1.1	1.5	1.3	1.6	2.5	6.1	0.8
MDFIC	3.4	4.5	1.1	3.1	2.8	2.0	1.4	4.3	0.8
EBPL	3.0	5.5	1.0	3.0	3.2	2.1	1.8	3.2	0.6
SNX29	3.2	3.8	1.3	1.7	2.1	2.8	2.0	5.4	1.2
KRAS	3.5	5.7	1.0	2.2	2.1	1.7	1.8	4.3	1.0
NAE1	3.5	5.3	0.8	2.5	1.5	2.5	1.3	5.1	1.0
HR	3.3	4.0	0.9	2.6	2.1	1.4	2.4	5.8	1.0
BCR	3.2	3.5	0.9	3.3	2.9	2.4	1.9	4.4	0.9
A4GALT	3.7	5.1	1.6	1.8	1.9	1.4	2.1	4.6	1.0
PAXBP1-AS1	4.2	5.7	1.2	1.6	1.2	3.0	1.6	4.1	0.7
C11orf71	3.7	6.2	1.5	1.0	1.1	1.7	2.4	4.3	1.6
ARL14EP	3.3	6.1	1.0	2.0	1.5	1.7	1.4	5.4	0.8
TMEM209	4.3	5.9	1.1	1.6	1.5	1.1	1.5	5.4	1.0
FBXL14	3.4	4.2	1.1	1.8	1.9	1.6	2.4	5.9	1.0
FBN2	4.8	6.9	1.4	1.2	1.1	0.9	1.4	5.1	0.7
ZMYM2	3.3	5.6	0.7	1.6	1.2	2.2	1.6	6.0	1.1
TMEM41A	4.1	6.4	1.2	2.3	1.2	1.3	1.7	4.1	0.9
HES4	4.6	6.2	1.7	2.4	2.6	2.8	0.9	1.7	0.5
MON1A	3.4	3.9	1.3	2.2	2.0	0.9	2.6	5.6	1.5
PDCD2L	4.2	5.7	1.3	2.1	2.0	1.3	1.7	4.2	0.8
SGCE	3.9	4.5	1.1	2.2	1.5	1.7	1.8	5.4	1.2
A1BG-AS1	4.1	4.7	1.0	1.3	0.9	1.0	2.7	6.5	1.1
SLC35B4	4.0	5.4	1.0	2.4	2.3	1.7	1.4	4.4	0.7
RBBP5	3.6	5.4	1.0	2.1	1.3	2.2	1.5	5.2	0.9
TRMT61A	3.3	4.2	1.6	2.1	2.2	1.1	2.5	5.2	1.1
SRRM3	4.4	3.9	2.1	2.9	3.4	1.5	1.8	2.2	1.1
BTAF1	3.6	5.0	0.8	1.5	1.1	3.0	1.6	5.7	0.9
MIR210HG	4.2	6.2	0.9	1.6	1.1	2.4	1.4	4.8	0.8
PRR7-AS1	4.2	5.3	1.1	1.4	1.0	2.0	1.9	5.3	1.0
MAGEH1	3.5	2.9	1.0	0.6	1.1	1.2	3.5	7.0	2.5
RPA2	3.0	4.4	1.0	2.3	2.3	1.8	1.9	5.4	1.1
GID4	3.9	4.7	2.0	2.1	1.7	1.0	2.3	4.5	1.0
LOC648691	4.2	6.0	1.0	1.2	0.9	2.4	1.4	5.3	0.7
SLC34A3	5.7	6.3	1.8	1.4	0.9	0.4	2.1	3.7	0.9

LYSMD3	2.7	4.9	0.5	1.3	1.2	2.6	2.3	6.7	1.1
EAPP	3.0	5.3	1.3	2.1	1.4	1.6	1.9	5.6	0.8
HIBCH	3.4	5.4	0.5	2.0	1.7	2.2	1.9	5.1	1.1
ZYG11B	3.5	5.3	0.9	1.9	1.5	1.7	1.7	5.6	1.0
LOC285696	4.2	6.1	1.3	1.0	0.9	1.9	2.1	4.8	0.8
MAP4K5	3.3	4.9	0.8	2.4	1.9	1.9	1.4	5.3	1.1
RAB31	4.3	3.8	1.3	2.5	2.2	2.9	1.5	3.7	1.0
RUFY3	4.3	6.5	1.2	1.4	1.1	2.2	1.6	4.2	0.9
LOC642852	3.7	4.7	1.1	2.2	1.7	1.4	2.1	5.3	1.0
LOC100287042	3.7	5.9	1.1	1.6	1.1	2.5	1.6	5.0	0.7
RECQL4	3.2	3.8	0.8	2.8	2.9	1.6	2.3	4.7	1.1
ASCC1	3.5	5.7	1.0	1.8	1.4	2.0	1.8	5.0	0.8
MED11	3.1	3.8	0.6	2.8	2.5	2.0	1.8	5.5	1.0
SFN	2.8	5.0	1.4	3.9	4.5	1.8	1.0	2.3	0.5
FERMT3	4.4	6.4	1.8	0.8	0.8	4.4	1.2	2.8	0.5
P2RY11	4.9	5.7	1.7	1.8	1.4	1.0	0.0	6.6	0.0
PHLPP2	3.6	4.9	0.9	2.2	1.9	1.8	1.7	5.3	0.9
LOC728975	3.6	5.7	1.4	1.1	1.0	1.6	2.1	5.7	0.9
UBXN11	3.4	5.2	1.2	2.1	1.8	1.2	1.9	4.9	1.2
TRNAG3	2.2	5.2	1.3	1.5	0.0	0.5	5.7	4.4	2.2
IGHMBP2	4.3	4.8	1.1	1.7	1.5	1.9	2.0	4.8	1.0
CCDC127	2.7	5.1	1.2	2.5	1.9	1.9	1.9	4.8	1.0
NINL	4.3	5.4	1.4	1.9	1.4	1.2	2.0	4.5	1.0
LOC100996291	4.2	5.2	1.0	1.3	1.1	4.1	1.6	3.9	0.8
PI4K2B	3.1	5.2	0.9	2.3	2.0	3.0	1.7	3.9	0.9
ZNF653	3.9	4.5	1.3	1.8	1.7	1.4	2.3	5.0	1.2
GMFB	2.6	5.4	1.1	2.6	1.0	2.3	1.3	5.7	0.9
LOC148413	3.7	4.6	1.1	2.1	1.5	1.4	2.7	5.0	1.0
USO1	2.8	5.0	0.5	2.1	1.5	2.0	1.8	6.2	1.2
ZCCHC17	2.7	4.0	0.5	2.1	1.2	1.9	2.5	6.7	1.3
FAHD2A	3.9	5.0	1.1	2.7	2.3	1.6	1.8	3.7	0.8
CCDC91	3.5	5.4	0.9	1.9	1.5	1.6	1.7	5.5	0.9
NHSL1	3.2	4.3	1.2	0.6	0.6	0.4	2.7	8.6	1.5
WEE1	3.3	5.0	1.1	3.1	2.3	2.7	1.1	3.4	0.8
ITGA10	2.8	4.3	0.6	1.3	0.9	1.0	2.8	8.2	1.2
GALNT11	3.9	5.1	1.2	2.0	1.9	2.2	1.6	4.3	0.8
RAB27A	2.5	3.4	0.6	1.4	0.8	1.6	2.7	8.6	1.3
KIAA0947	3.5	5.0	1.0	1.8	1.5	1.6	1.8	5.8	1.1
LOC101927954	3.7	6.0	1.0	1.2	0.9	2.7	1.6	5.2	0.7
LOC101928093	3.1	3.9	1.4	2.4	1.8	1.8	3.0	4.4	1.2
ZBED4	3.2	4.3	1.1	1.9	1.6	1.5	2.3	5.8	1.2
DNAAF1	4.0	6.0	1.1	1.2	0.9	2.3	1.5	5.3	0.7
KIAA1598	3.9	5.3	1.0	2.1	1.7	1.4	1.7	4.8	1.1
CASP3	3.4	6.8	0.8	1.9	1.1	2.8	1.4	4.2	0.6
PPIP5K1	3.7	5.6	0.9	2.0	1.7	1.7	1.6	4.9	0.7
SMAD3	4.3	3.7	1.3	2.5	2.9	3.6	1.1	2.8	0.8
MCCC1	4.0	4.8	1.1	2.4	2.0	1.9	1.5	4.3	0.8

KIF22	3.7	4.7	1.1	3.6	2.9	2.4	1.3	2.5	0.7
TRNP1	3.4	5.7	1.6	3.6	2.6	2.5	1.1	1.9	0.7
ZNF251	4.4	4.6	1.0	1.4	1.4	0.9	2.7	5.7	0.7
HRH1	2.7	4.2	1.0	2.2	1.9	1.6	1.9	6.1	1.2
EPHB2	3.8	4.0	1.1	2.8	2.6	1.8	1.7	4.2	1.0
TBX1	4.4	4.5	1.5	1.6	1.7	1.0	2.1	4.7	1.4
LCAT	5.2	5.8	1.2	1.3	1.3	1.4	1.9	3.5	1.3
SNHG10	2.8	4.7	0.6	1.2	0.9	3.2	3.4	5.4	0.8
LOC100506548	3.6	6.3	1.1	1.6	1.0	1.7	1.6	5.3	0.8
KHK	2.7	4.5	0.6	2.8	2.2	2.3	1.8	5.1	0.8
ARL17A	3.6	4.9	0.9	2.0	1.2	2.0	2.1	5.3	0.9
LOC101929220	3.1	3.7	0.5	1.5	1.1	2.8	3.1	6.0	1.2
SLC27A3	4.9	6.5	1.2	1.1	0.9	0.8	1.8	4.4	1.1
PCNXL2	3.9	5.4	1.2	2.1	1.9	1.4	1.6	4.7	0.7
LOC101929368	3.3	6.3	0.2	1.4	0.6	2.3	2.1	6.7	0.0
SLC35A5	3.1	6.0	0.9	2.2	1.3	1.7	1.8	4.8	1.1
ARID4B	3.6	4.5	0.8	1.6	1.3	2.1	1.8	6.2	1.0
SH3RF3	3.5	4.0	1.5	1.4	1.4	1.1	2.3	6.3	1.3
SMC5	3.6	5.3	1.0	1.5	1.2	1.5	1.8	5.7	1.3
RIF1	3.3	5.5	0.8	1.7	1.4	2.2	1.3	5.7	0.9
DHRS13	3.6	4.5	0.9	2.3	2.0	1.8	2.4	4.5	0.8
SLC12A7	3.7	4.3	1.2	2.1	2.1	2.1	1.9	4.6	0.8
COX10	3.9	5.4	0.8	1.8	1.5	1.6	1.9	5.0	0.9
LEO1	3.9	4.3	1.0	2.1	1.4	2.1	1.8	5.1	1.1
PRRG1	3.2	5.4	1.1	2.3	1.7	1.8	1.3	5.0	0.9
USP25	3.5	6.0	1.0	2.3	1.8	2.0	1.1	4.4	0.7
TVP23B	3.0	5.1	0.7	2.0	1.4	2.3	2.1	5.5	0.6
LOC101929010	3.9	5.9	0.9	1.1	0.9	2.5	1.6	5.1	0.8
ZNF318	3.1	4.4	1.0	2.0	1.7	1.8	2.0	5.8	1.1
LOC286186	3.9	5.9	1.3	1.3	0.9	2.1	1.5	5.0	0.8
CYP4V2	3.0	5.0	1.0	1.3	1.3	1.7	2.1	6.4	1.0
LETM2	4.8	6.2	1.8	2.7	2.8	1.4	0.6	2.1	0.4
PFKFB2	4.9	6.9	1.2	2.6	2.1	2.1	0.8	1.8	0.4
LOC101927312	3.9	8.4	0.9	1.0	0.5	0.7	0.9	5.6	0.9
PAPD5	4.4	4.6	1.3	1.7	1.6	1.6	1.9	4.7	1.0
NHS	3.3	4.1	1.0	0.9	1.0	0.6	2.6	7.5	1.8
PRPF39	4.2	6.2	0.8	1.4	1.2	1.6	1.5	4.9	0.8
CHAMP1	3.8	4.6	0.8	2.4	2.1	1.2	1.8	5.0	1.1
E2F6	4.1	5.2	1.0	1.9	1.7	1.5	1.7	5.0	0.6
PCSK4	4.9	5.5	1.7	1.9	1.6	0.8	2.0	3.1	1.2
PTPRE	3.2	4.3	0.8	1.6	1.5	1.3	2.6	6.4	1.1
SNORD80	2.3	2.4	1.7	0.4	1.6	1.1	5.6	6.4	1.3
BFSP1	2.5	2.8	0.6	3.9	3.4	2.0	1.7	5.0	0.9
CBX8	3.9	3.0	0.9	1.5	1.9	1.3	3.1	5.9	1.2
PHF8	3.7	4.9	0.9	1.3	1.3	1.4	2.2	5.9	1.1
LOC100506844	3.1	5.7	0.7	2.1	1.9	2.1	2.0	4.2	0.9
AP1AR	4.4	6.2	0.8	1.9	1.3	1.4	1.5	4.3	0.9

TRIM38	3.5	5.8	0.9	1.3	1.2	2.1	1.7	5.4	0.8
PPIL2	4.6	4.8	1.3	2.0	1.4	1.5	2.0	4.2	0.9
MESDC1	3.2	5.0	1.4	1.8	1.8	2.7	2.1	3.8	0.8
PHACTR2	3.7	4.8	1.0	2.5	1.7	1.4	1.7	5.0	0.8
OLFML3	5.3	6.4	1.7	1.3	1.3	0.6	1.9	3.3	0.8
MITD1	4.2	3.9	1.4	2.3	1.4	1.9	1.9	4.0	1.6
AKAP11	3.2	5.3	0.7	1.5	1.1	1.4	1.8	6.5	1.0
EXOSC7	4.1	5.1	1.2	2.2	1.3	1.8	2.4	4.1	0.7
ANKMY1	4.5	5.2	1.0	1.3	1.2	0.7	2.4	5.5	1.0
FBXO41	4.1	5.2	1.5	1.7	1.8	1.1	2.0	4.1	1.1
PSMD5-AS1	3.6	5.7	1.0	1.2	1.0	2.2	1.8	5.3	0.8
XRN1	3.9	5.6	1.0	1.4	1.1	1.9	1.4	5.4	1.0
MAP2K4	3.6	5.0	1.0	1.9	1.7	1.9	1.7	4.6	1.2
PHF13	3.6	4.9	1.2	1.9	1.7	1.3	2.0	5.3	0.8
RPA3-AS1	3.8	5.3	1.0	2.1	1.7	1.3	1.8	4.6	1.1
METTL17	3.9	4.9	1.1	1.8	1.5	1.5	2.2	4.7	1.1
USP6NL	2.5	3.3	0.9	1.4	1.3	1.2	3.2	7.3	1.5
PCDHGA10	3.8	4.9	1.1	1.4	0.9	0.9	2.3	5.9	1.4
ERC1	3.6	4.9	1.1	1.9	1.3	1.7	1.8	5.5	0.9
FAM171B	4.1	5.5	1.2	1.6	1.6	1.2	1.6	5.1	0.8
ARMC8	3.5	5.3	0.8	1.8	1.7	1.8	1.8	5.1	0.8
MAPKAPK5-AS1	3.6	5.3	1.1	2.2	1.7	1.6	2.1	4.5	0.6
ARAP3	4.0	4.7	1.2	1.8	1.7	1.5	2.2	4.5	1.0
DBP	4.3	5.8	1.3	1.6	1.3	1.5	2.1	3.7	1.0
ATG7	3.3	5.3	0.7	2.0	1.8	1.6	1.8	5.4	0.7
NSMCE4A	3.8	5.0	0.8	1.4	1.6	2.1	1.9	4.8	1.1
HIST1H2BL	3.6	2.7	1.6	2.7	2.9	5.3	1.0	2.2	0.7
ZBTB11	3.6	5.7	1.0	1.5	1.6	1.8	1.4	4.8	1.1
NFX1	3.5	5.0	0.9	1.7	1.4	1.9	1.9	5.4	1.0
LONRF1	4.4	6.4	1.5	1.3	1.1	1.7	1.3	3.7	1.2
SNORD14C	2.9	3.1	1.4	0.6	0.9	6.8	1.9	3.0	1.8
RHBDD1	3.5	6.0	0.9	1.6	1.0	2.3	1.5	4.8	0.8
CEP350	3.6	5.1	0.8	1.3	1.1	1.6	1.8	6.3	1.0
KMT2A	3.9	5.3	1.1	1.3	1.2	2.3	1.4	5.2	0.8
LOC101929493	2.0	4.0	0.6	4.8	3.7	3.0	0.7	3.1	0.6
CHMP4A	4.5	3.8	1.1	2.2	1.5	1.9	1.9	4.4	1.3
ULBP2	3.6	5.2	1.4	2.4	2.5	1.0	1.8	3.8	0.8
CCDC74B-AS1	4.0	5.4	1.2	1.2	1.0	3.1	1.4	4.5	0.6
IQGAP3	3.7	4.6	1.2	5.1	4.0	2.7	0.3	0.8	0.2
TMEM2	2.0	2.8	0.5	1.5	1.2	3.0	2.2	8.0	1.3
CCNC	3.3	4.7	0.8	2.8	1.7	2.3	1.4	4.8	0.7
C8orf49	3.5	5.5	0.8	1.4	1.1	2.2	1.6	5.5	0.8
C12orf65	3.7	3.9	0.8	2.2	1.5	2.4	1.8	4.9	1.2
IL21R-AS1	3.7	5.9	1.1	1.2	1.0	2.5	1.5	4.8	0.8
ASF1A	2.9	4.4	0.6	2.1	1.7	2.2	1.6	5.7	1.2
SKP2	3.7	5.9	1.0	3.1	2.3	2.9	0.7	2.3	0.5
RRAGB	4.6	5.4	1.2	2.0	1.6	2.3	1.3	3.1	0.8

IDNK	3.7	6.2	0.7	1.8	1.2	2.4	1.2	4.6	0.6
GAGE1	3.9	4.4	1.4	4.2	1.5	1.5	1.7	3.1	0.7
B3GNT2	3.1	4.6	0.7	2.1	1.7	3.9	1.3	4.2	0.7
LOC100506365	3.4	4.6	1.3	1.6	2.0	1.2	1.9	5.2	1.1
LOC101927919	4.6	5.4	1.4	1.4	1.2	2.6	1.2	3.7	0.8
ZSCAN16-AS1	3.8	4.7	1.3	3.2	1.8	1.5	1.7	3.8	0.6
ZMYND8	4.4	4.9	1.7	1.8	1.7	1.4	1.5	4.2	0.9
MIOS	4.4	5.4	0.5	1.5	1.1	1.4	2.1	5.2	0.9
ARNT2	4.6	5.6	1.2	2.1	1.6	1.2	1.6	3.7	0.8
APC	3.2	4.8	0.7	1.9	1.5	1.7	1.7	5.8	1.0
LINC00839	4.1	4.9	1.1	1.9	2.0	1.4	1.9	4.4	0.8
LSM6	3.6	3.9	0.8	2.5	2.6	3.7	1.5	2.7	1.2
ZNF394	3.6	4.4	1.3	1.3	1.2	4.0	2.0	4.0	0.6
SLC33A1	3.3	4.8	0.7	1.9	1.3	1.5	1.8	6.1	0.9
CRYZL1	4.5	5.8	0.9	2.1	1.1	1.4	1.6	4.4	0.6
RBM18	3.6	4.2	0.9	1.8	1.8	1.6	2.5	4.9	1.1
LOC101929372	3.9	6.0	1.1	1.2	0.9	2.3	1.3	4.7	0.7
PICK1	3.8	4.8	0.9	2.1	2.2	1.3	2.0	4.3	0.9
MAFF	4.9	8.2	1.2	0.8	1.5	1.7	1.0	2.6	0.4
LOC101928231	4.0	6.0	1.2	1.1	0.9	2.0	1.4	5.0	0.7
SLC7A11	3.4	4.6	0.9	1.7	1.3	1.6	1.9	6.3	0.8
ALDH16A1	2.6	3.0	0.8	2.1	1.9	2.8	2.7	5.2	1.3
ZWINT	2.7	3.4	0.6	4.9	4.7	4.4	0.3	1.1	0.2
USP8	3.6	4.8	0.9	1.9	1.5	2.0	1.6	4.9	1.1
KDM5A	3.3	5.0	0.9	1.7	1.2	2.5	1.7	5.3	0.9
ALG14	3.0	5.4	0.8	2.5	1.2	1.7	1.6	5.4	0.7
MRPS28	3.5	4.8	0.9	2.4	2.1	1.8	1.9	4.4	0.6
ZNF740	4.2	4.7	1.0	1.4	1.3	1.4	2.3	5.2	0.9
UBOX5	4.2	5.0	1.2	1.9	1.5	1.6	1.9	4.0	1.1
DYSF	4.0	4.6	1.1	3.8	3.7	2.6	0.7	1.4	0.4
TAF4	3.9	4.2	1.5	1.8	1.8	1.4	2.0	4.6	1.1
PHKA2	4.3	5.6	1.0	1.6	1.3	1.3	1.8	4.4	0.9
PARP16	3.6	5.4	1.1	1.6	1.3	1.2	2.1	4.8	1.1
GMPPB	3.1	3.8	0.9	2.2	2.1	2.1	2.2	4.8	1.0
KIAA1324	3.7	5.6	1.1	1.5	1.0	2.6	1.5	4.5	0.8
UVRAG	3.2	4.5	0.7	1.6	1.4	2.7	1.5	5.8	0.8
FAM120C	3.3	4.6	1.2	1.8	1.4	0.8	2.4	5.7	1.0
LOC440173	4.1	6.4	1.4	1.1	1.2	1.0	1.6	4.6	0.8
SNAPC5	3.7	3.5	1.6	2.3	2.1	1.9	1.5	4.3	1.2
CTBP1-AS2	3.3	4.8	1.0	2.0	1.6	1.5	1.8	5.3	0.9
LOC101929132	4.1	5.5	1.2	1.3	0.9	2.2	1.7	4.6	0.7
PRKRIP1	4.5	5.5	1.5	1.8	3.0	0.5	1.3	2.9	1.3
TBC1D7	4.1	5.5	0.9	1.6	1.5	1.0	1.6	5.0	1.1
CREB1	4.4	3.4	1.2	1.6	2.2	2.7	1.9	3.8	1.0
ANXA9	4.0	4.7	0.8	2.7	2.3	1.7	1.3	4.0	0.8
ZNF286A	3.3	5.4	0.8	1.7	1.2	1.3	1.9	5.6	0.9
BTN2A2	4.0	6.3	1.1	1.6	1.2	1.8	1.8	3.7	0.7

LOC100507156	3.8	6.2	1.0	1.7	1.3	2.1	1.2	4.3	0.6
LOC101927562	4.0	4.9	0.9	1.8	2.1	1.6	2.0	3.9	0.9
OPN3	3.5	4.1	0.9	1.8	1.7	1.1	2.7	5.1	1.1
HIST1H2AJ	2.5	4.0	0.8	3.0	2.3	5.9	1.1	2.5	0.0
MTFMT	3.6	5.2	1.0	2.2	1.8	2.1	1.6	4.1	0.6
ORC4	2.9	5.5	0.7	1.9	1.6	2.1	1.4	5.1	0.9
BCHE	4.0	5.9	0.7	1.3	0.7	1.2	1.9	5.5	0.9
KLHL9	3.4	6.3	1.0	1.9	1.1	2.3	1.1	4.3	0.8
RAP1GAP2	4.5	5.1	1.6	1.9	2.0	2.0	1.4	2.8	0.8
AHCYL2	3.3	5.0	1.0	1.5	1.4	1.6	2.1	5.3	1.0
PLA2G6	4.6	5.4	1.6	1.3	1.2	1.1	2.3	3.6	0.9
ZCCHC11	3.5	5.0	0.7	1.6	1.3	1.7	1.6	5.4	1.2
N4BP2L2	3.4	5.0	0.9	1.6	0.9	1.6	1.7	6.1	0.9
USP38	3.4	4.8	0.8	1.7	1.7	1.8	1.9	5.1	0.9
LOC100506054	3.4	4.3	0.9	2.5	2.2	1.2	2.2	4.7	0.8
DNAJB9	2.5	3.8	0.7	1.7	1.4	2.3	1.6	6.7	1.4
RBM41	3.8	5.7	0.9	1.4	1.1	2.2	1.3	5.1	0.5
ARHGAP5	2.5	3.5	0.4	1.3	1.0	1.6	2.0	8.4	1.4
SLIT2	5.4	8.1	1.6	1.1	1.2	1.0	0.9	2.3	0.4
CDH4	3.8	4.4	1.6	3.3	3.3	1.5	1.2	2.3	0.7
SFXN4	4.0	5.2	1.0	1.7	1.8	1.2	1.7	4.8	0.7
XAGE1E	4.6	2.1	1.8	2.4	2.4	1.4	2.6	3.5	1.4
XAGE1C	4.6	2.1	1.8	2.4	2.4	1.4	2.6	3.5	1.4
XAGE1A	4.6	2.1	1.8	2.4	2.4	1.4	2.6	3.5	1.4
XAGE1B	4.6	2.1	1.8	2.4	2.4	1.4	2.6	3.5	1.4
TMEM192	3.8	5.1	0.8	2.7	1.5	1.9	1.7	3.7	0.9
FOCAD	3.3	5.3	0.9	2.2	1.4	1.6	1.6	5.0	0.8
CCDC102A	3.7	3.8	1.2	2.7	2.5	1.6	1.9	3.7	0.9
PHC1	3.7	4.3	0.9	2.1	2.0	1.8	1.8	4.5	1.0
METTL3	3.6	4.5	1.0	1.7	1.4	2.0	2.1	4.9	0.8
LINC00662	4.1	5.5	1.0	1.5	1.1	1.7	1.7	4.6	0.8
LMBR1L	3.7	5.3	1.0	1.4	1.3	1.7	1.9	4.8	0.9
ARL5B	3.6	4.5	0.6	1.2	0.6	2.9	1.5	5.7	1.5
LOC100507459	3.9	5.6	0.9	1.4	1.3	2.1	1.6	4.6	0.6
IL7R	4.0	8.2	1.4	1.2	0.9	4.8	0.3	1.0	0.2
NS3BP	5.0	5.9	1.3	1.7	1.1	0.9	1.7	3.8	0.6
KIAA0101	1.9	4.4	1.0	4.6	4.1	4.9	0.1	0.6	0.2
FAM64A	3.4	3.8	1.1	4.8	4.4	3.5	0.3	0.5	0.2
TTBK2	4.1	5.0	1.1	1.4	1.3	1.4	1.7	5.3	0.8
TIGD5	3.1	4.4	1.2	1.8	2.0	1.7	2.3	4.7	0.9
ATL2	2.4	3.8	0.5	1.3	0.9	1.9	2.2	8.1	1.0
ATAT1	4.1	3.9	1.2	1.6	1.1	0.4	2.5	5.4	1.7
FLJ37448	3.9	5.7	1.0	1.2	1.0	2.3	1.5	4.6	0.8
TCP11L2	2.7	4.6	0.5	0.9	0.9	1.3	2.2	7.8	1.1
INTS6	3.5	4.3	0.8	1.9	1.5	2.9	1.6	4.6	0.9
LFNG	1.7	1.8	0.5	0.7	0.6	6.7	2.8	5.7	1.5
LINC00965	4.1	6.1	1.0	1.1	1.1	1.1	1.5	5.0	0.9

GEMIN4	3.1	4.2	0.9	2.1	1.9	1.4	2.1	5.5	0.8
LOC101927588	4.1	6.0	1.1	1.4	1.0	2.4	1.2	4.2	0.6
MTMR3	3.5	4.6	1.2	1.4	1.3	1.6	1.9	5.6	0.9
CBWD5	3.6	4.8	1.2	1.7	1.5	1.6	1.9	4.6	1.1
FAM172A	3.2	5.6	0.8	2.1	1.6	2.1	1.5	4.0	0.9
SAMM50	3.6	5.3	1.2	2.0	1.4	2.0	1.8	4.0	0.7
LOC101929301	4.8	6.4	1.6	1.4	1.0	1.1	1.5	3.6	0.4
ZNF451	3.1	5.1	0.7	1.6	1.3	1.7	1.7	6.0	0.9
HARS2	3.7	4.5	1.0	2.0	2.0	1.4	1.8	4.4	1.1
NREP	3.0	3.7	1.1	2.2	1.2	0.8	2.2	6.2	1.6
KLF4	4.1	4.5	1.4	1.5	1.6	1.1	1.7	4.6	1.4
SDHAF1	3.3	5.2	1.0	1.7	1.1	2.3	2.2	4.2	0.8
FOXJ2	4.1	3.8	1.1	1.6	1.5	1.4	2.2	4.9	1.2
NXT2	4.0	5.6	0.9	2.9	1.9	2.0	1.0	3.3	0.4
ARID3A	2.3	2.9	1.0	1.4	1.4	1.6	3.4	6.3	1.5
DENND6B	4.3	5.4	1.4	1.9	1.7	1.3	1.7	3.3	0.8
GYG2	3.3	4.9	1.2	2.6	2.5	2.0	1.1	3.7	0.6
CREBRF	3.3	5.0	0.9	1.4	1.2	2.2	1.4	5.5	0.9
ARL4D	4.5	5.7	1.4	1.9	2.5	1.4	1.3	2.5	0.7
RAPGEF1	3.2	4.2	0.8	1.4	1.3	5.5	1.2	3.5	0.7
TMEM51	3.9	5.9	1.4	1.1	1.4	1.0	1.9	4.0	1.3
PHF14	3.9	4.9	1.0	1.5	1.1	1.3	1.7	5.6	0.8
KIF3A	4.2	4.7	1.0	1.6	1.5	1.6	1.6	4.8	0.8
LOC100287225	3.8	5.4	1.0	1.5	1.0	2.1	1.4	5.1	0.6
HLA-F	3.0	3.7	1.0	0.8	0.5	10.0	0.7	1.7	0.3
ARL17B	3.3	4.8	0.9	1.9	1.2	1.7	2.0	5.1	0.9
PARG	3.8	5.2	1.2	1.7	1.5	1.6	1.6	4.3	0.9
ZNF274	3.7	5.0	1.1	1.7	1.6	1.6	1.8	4.3	0.9
ABHD3	4.8	5.9	1.4	1.7	1.1	1.8	1.4	3.1	0.6
ZNF174	3.5	4.5	1.0	1.5	1.8	0.8	1.8	5.5	1.3
RRS1	3.4	3.8	1.4	1.9	2.4	1.4	2.2	4.4	0.9
RPUSD2	3.4	4.6	1.0	2.1	2.1	1.1	2.2	4.4	0.8
MSX1	3.3	4.0	1.0	2.3	3.1	1.9	1.9	3.1	1.1
PDRG1	2.6	5.6	1.5	1.3	2.3	2.3	1.9	3.7	0.5
LOC101929144	3.4	5.4	0.9	1.3	1.0	2.9	1.5	4.6	0.7
ATXN1L	3.2	4.3	1.1	1.6	1.6	2.6	1.7	4.8	1.0
MCU	3.6	5.2	1.3	2.1	1.9	1.8	1.5	3.8	0.6
COBLL1	3.2	4.1	0.8	2.4	2.1	1.7	1.7	4.6	1.2
ASB8	2.9	4.9	0.8	2.2	1.4	2.0	1.9	4.6	1.0
PIK3CA	3.4	5.4	0.5	1.8	1.3	1.8	1.4	5.3	0.8
ZMYM6NB	3.0	6.4	0.6	3.1	1.2	1.5	1.6	3.7	0.7
FLJ37644	4.1	6.7	1.2	1.0	1.0	0.9	1.1	5.2	0.5
RYBP	3.0	3.3	1.1	2.2	2.1	2.1	2.2	4.5	1.2
LOC339988	3.4	5.3	0.7	2.0	1.7	2.1	1.3	4.2	0.9
ZHX1	3.3	5.2	0.8	1.6	1.1	1.4	1.7	5.5	0.9
TPP2	3.6	5.0	0.8	1.7	1.2	1.5	1.8	4.9	1.0
CCNT1	3.1	4.7	0.9	1.5	1.0	1.8	2.0	5.7	1.0

LOC100130954	3.6	5.2	1.0	1.1	0.8	2.9	1.5	4.8	0.7
NAGA	3.9	4.7	1.2	1.9	1.7	3.0	1.2	3.3	0.7
CCNO	3.8	4.7	1.3	1.7	1.9	0.9	1.9	4.4	1.0
GPR173	3.1	3.9	0.9	1.0	0.7	0.4	2.6	7.8	1.2
LOC101928495	3.3	5.7	1.2	1.2	0.5	3.7	1.0	4.3	0.7
ACAP1	3.0	3.5	0.8	0.9	0.6	10.1	0.8	1.6	0.4
MELK	3.6	5.2	0.9	2.8	2.1	2.8	0.9	2.8	0.4
GDF1	5.0	5.4	1.6	2.0	1.9	1.2	1.5	2.3	0.6
C18orf25	3.2	5.1	0.8	1.8	1.1	2.3	1.7	5.1	0.6
NHLRC2	3.6	5.8	0.7	1.7	1.4	1.6	1.3	4.8	0.7
CD109	3.4	6.1	0.8	2.0	1.4	1.8	1.2	4.1	0.6
TTLL3	4.3	4.9	1.2	1.2	1.0	1.7	1.8	4.4	0.9
SNX11	2.8	6.0	1.1	1.8	1.0	1.6	1.8	5.1	0.3
ZBTB43	3.5	4.3	1.1	1.5	1.6	1.7	2.5	4.5	0.9
NEDD1	3.3	5.5	0.8	2.5	2.2	2.3	0.9	3.6	0.5
TEX10	2.7	4.3	0.6	2.3	1.6	2.4	1.5	5.0	1.0
MED20	3.2	5.1	0.8	2.1	1.3	2.4	1.6	4.3	0.7
HEATR6	4.0	5.3	1.1	1.7	1.4	1.3	1.5	4.4	0.9
MFSD11	3.3	6.2	0.8	1.4	0.9	1.9	1.3	4.7	0.8
LOC100129917	3.6	5.6	1.0	1.3	1.0	1.5	1.5	5.2	0.8
ZBTB40	3.7	4.4	0.9	1.5	1.2	1.6	2.0	5.2	1.0
PLK1	2.9	4.1	1.2	4.6	4.9	2.7	0.3	0.6	0.2
LINC01021	4.2	5.5	1.2	1.7	0.8	2.0	1.4	4.2	0.6
STX12	3.0	5.4	0.7	1.9	1.2	2.0	1.7	4.8	0.7
FANCG	3.3	4.8	1.1	2.2	2.4	2.0	1.3	3.7	0.6
PIGH	2.5	4.0	0.5	1.9	1.5	1.4	2.0	6.2	1.3
SPNS2	4.1	5.2	1.4	2.1	1.8	0.9	1.6	3.5	0.7
SDCCAG8	3.2	4.5	1.0	1.5	1.6	1.3	1.8	5.3	1.1
APTX	3.5	5.4	1.0	1.9	1.4	1.6	1.6	4.5	0.5
LINC00670	3.7	6.5	0.7	1.0	0.8	2.0	0.9	5.2	0.6
LOC728730	3.6	5.2	1.2	1.5	1.1	2.6	1.5	4.0	0.7
SPSB2	3.5	3.9	1.1	2.4	2.2	1.1	1.9	4.3	0.9
LINC00578	4.0	5.4	0.8	1.2	0.7	2.4	1.1	5.1	0.6
METTL1	3.9	3.7	0.9	1.7	1.3	1.7	2.2	4.4	1.7
PDE8A	3.5	4.6	0.8	1.9	1.5	1.7	1.5	5.0	0.9
TMEM92	4.2	4.0	1.4	3.0	2.9	1.3	1.3	2.6	0.8
LOC101928029	3.6	5.2	1.0	1.2	1.0	1.9	1.6	5.2	0.6
C1orf50	3.1	4.0	1.2	2.1	1.9	1.1	2.1	4.7	1.1
BAZ2B	3.7	4.7	0.9	1.2	0.9	0.9	1.9	6.1	1.2
TIMM23B	1.4	6.0	1.2	0.4	0.1	0.2	3.1	7.7	1.2
FLVCR1	3.6	5.2	1.1	1.8	1.6	1.6	1.8	3.9	0.7
PROSER1	3.5	4.3	0.8	2.0	1.7	1.4	1.7	4.9	1.1
TTI2	3.5	4.7	0.8	1.8	1.8	1.3	1.6	4.7	1.0
ZNF79	3.2	4.5	0.9	1.5	2.1	1.2	2.1	4.8	1.1
UBE2D1	3.7	5.4	0.4	2.4	1.4	1.4	1.0	4.7	0.9
C19orf26	4.4	4.1	1.3	2.0	2.1	0.9	2.1	3.3	1.1
ING5	3.5	4.7	1.0	1.3	1.4	1.1	2.5	4.8	0.9

ADNP2	3.2	4.4	0.9	1.4	1.3	1.8	1.9	5.5	0.9
DARS2	3.4	5.2	0.9	2.4	2.1	1.6	0.9	4.0	0.6
C12orf43	3.7	5.2	1.2	1.7	1.9	2.0	1.8	3.3	0.6
GTF2E1	3.6	4.9	1.1	1.7	1.4	1.4	1.5	4.3	1.2
LOC101927860	3.3	5.9	0.9	1.2	0.9	2.6	1.5	4.3	0.6
LRRFIP1	3.0	3.8	1.0	2.5	2.0	2.4	1.5	4.5	0.7
CDC42EP5	5.2	5.9	2.2	1.5	1.4	0.6	1.3	2.3	0.8
ZBTB1	2.9	4.5	0.5	1.7	1.1	2.9	1.6	5.4	0.8
ZNF670-ZNF695	3.3	4.5	1.0	1.6	0.7	4.5	1.3	3.6	0.7
EXOC1	3.5	5.3	0.8	1.6	1.5	1.7	1.4	4.6	0.8
TMEM251	3.4	5.0	0.8	1.9	1.2	1.0	2.0	4.9	1.1
SPAG16	3.0	5.0	0.7	1.8	1.5	1.2	1.9	4.9	1.2
INTS9	3.7	4.7	1.1	1.7	1.3	1.4	1.7	4.6	0.9
RSBN1L	3.3	5.0	0.9	1.5	1.2	1.8	1.7	4.9	0.9
RUNDC3A	3.8	5.2	1.1	1.2	1.0	2.2	1.3	4.5	0.8
NFKBIZ	2.9	3.6	0.7	0.7	0.3	8.0	1.4	3.1	0.7
APPL1	3.5	5.0	0.9	1.4	1.1	1.9	1.6	4.9	0.8
HTRA1	2.7	3.6	0.8	1.6	1.5	1.2	2.4	6.0	1.4
PLCB4	3.4	5.0	1.0	2.3	2.0	1.8	1.2	3.7	0.7
LEPREL1	4.8	6.8	1.5	2.4	2.1	1.4	0.6	1.3	0.2
PIK3CB	3.4	5.6	0.6	1.7	1.4	1.8	1.2	4.5	0.8
GPATCH2	3.5	4.5	0.6	1.7	1.6	1.5	1.6	5.4	0.7
MPHOSPH10	3.3	4.1	0.8	1.4	1.1	1.4	2.1	5.7	1.4
SMCHD1	2.8	4.6	0.7	1.6	1.2	3.1	1.3	5.0	0.8
SUCLA2	3.1	5.3	0.6	2.6	1.6	1.7	1.5	4.3	0.7
LILRB3	3.4	5.6	0.9	1.2	0.9	3.4	1.2	4.0	0.6
RAPGEF2	3.9	5.0	1.0	1.6	1.6	1.9	1.4	3.9	0.8
NLGN4Y	3.4	5.0	1.2	1.7	1.3	1.4	1.6	4.7	0.9
AIFM1	3.3	4.4	1.0	1.6	1.3	1.1	2.3	5.2	1.1
NIPBL	3.3	4.8	0.9	1.4	1.1	1.8	1.5	5.3	0.9
LIMCH1	4.7	6.0	1.3	2.0	1.7	1.2	1.0	2.5	0.6
KCNAB2	2.8	3.4	0.8	2.2	1.7	4.0	1.7	3.4	1.0
KIAA1199	5.5	6.8	1.4	1.5	1.4	1.4	0.8	1.8	0.5
C14orf80	3.0	3.6	1.0	2.2	1.9	4.2	1.5	3.2	0.5
LEMD1-AS1	3.7	5.2	1.0	1.2	0.9	2.6	1.4	4.3	0.7
UHRF2	3.5	4.6	0.9	1.8	1.2	2.4	1.4	4.5	0.8
TPST2	2.8	3.8	0.9	1.4	1.7	3.5	1.8	4.5	0.6
FAM203A	2.4	4.3	0.7	2.3	2.6	1.8	1.9	4.3	0.7
RABGGTB	3.3	6.1	0.7	1.7	1.3	3.1	1.0	3.2	0.6
ABCB10	3.0	5.2	1.0	2.3	1.8	2.2	1.2	3.7	0.6
MAP2K5	3.2	5.4	0.9	1.3	1.6	1.9	1.6	4.4	0.7
PTPN4	3.0	5.1	0.6	1.4	1.3	1.5	1.6	5.5	1.1
TRMT61B	3.0	4.5	0.8	2.1	1.2	1.6	1.6	5.3	0.9
NT5DC3	4.3	4.8	1.2	1.7	1.6	1.4	1.5	3.6	0.8
ZNF398	3.1	3.7	1.1	1.6	1.3	1.4	2.4	5.4	1.1
ZCCHC8	4.2	5.1	1.1	1.3	1.5	1.3	1.5	4.0	1.0
LOC101929672	4.1	5.0	1.3	1.6	1.6	0.2	1.2	5.2	0.8

FLJ35934	5.5	5.3	1.2	1.3	1.0	0.7	1.7	3.4	0.8
KDM1B	2.9	4.3	0.9	2.8	1.7	2.1	1.5	4.3	0.6
RNF182	3.0	3.2	0.4	2.1	2.6	1.9	1.8	5.3	0.8
IL11	2.9	5.0	1.1	1.7	1.8	1.1	2.3	4.0	0.9
FAM86A	3.9	5.1	1.1	2.0	1.3	1.1	1.7	4.1	0.7
C9orf91	3.3	4.8	1.0	1.3	1.2	1.6	2.0	5.0	0.7
RAB3D	2.6	4.5	0.9	1.2	1.5	1.4	2.1	5.5	1.3
COL1A1	3.7	4.9	1.3	0.9	0.7	0.6	2.5	4.9	1.3
PCDH7	4.7	6.4	1.4	2.0	2.0	1.6	0.7	1.7	0.4
PEX26	3.4	4.7	0.9	2.1	2.3	2.0	1.2	3.8	0.4
LINC00921	3.5	5.4	1.1	1.1	0.7	2.2	1.5	4.6	0.7
ARID2	3.2	4.7	0.7	1.4	1.3	1.7	1.7	5.3	1.0
RAPGEF5	1.4	2.1	0.3	0.3	0.4	0.4	3.0	11.4	1.6
SNAI3	3.4	6.0	1.3	2.1	1.7	1.1	1.5	3.3	0.5
SMAGP	3.8	5.2	0.9	2.7	2.5	0.7	1.8	2.7	0.6
TMEM70	3.1	4.8	0.6	1.8	1.4	2.0	1.8	4.2	1.0
IGFBP3	4.3	4.3	1.7	1.2	0.8	0.4	2.0	5.3	0.9
MXD3	3.0	3.8	1.6	4.3	3.7	2.3	0.6	1.4	0.4
L3MBTL2	3.4	4.3	1.1	1.5	1.5	1.4	2.0	4.9	0.9
C2orf69	3.8	5.8	1.0	1.8	1.2	1.5	1.9	3.3	0.6
LOC101927915	1.8	2.2	0.7	6.4	4.3	2.6	0.7	1.6	0.6
INO80D	3.2	4.4	1.1	1.5	1.2	1.6	1.7	5.2	0.9
ATP6V0A2	3.5	4.4	0.7	1.6	1.3	1.3	1.8	5.4	1.0
ZNF121	3.3	4.9	0.6	1.7	1.0	2.2	1.3	4.9	1.0
LOC100507217	2.5	3.7	1.1	1.8	1.2	4.4	1.6	3.4	1.2
TOPBP1	3.0	4.3	0.6	2.0	1.6	1.9	1.5	4.9	0.9
RPP14	2.9	5.1	0.9	1.6	1.1	1.8	1.8	5.0	0.6
PIGF	4.3	4.3	0.8	2.3	2.0	1.5	1.3	3.9	0.4
USP30	3.8	4.6	1.1	1.7	1.7	1.3	1.7	3.8	1.0
KISS1	3.3	6.8	2.7	3.1	3.1	1.2	0.4	0.3	0.2
LOC729683	3.5	4.9	1.0	1.6	1.2	2.7	1.5	3.7	0.7
P2RX7	4.0	5.3	1.0	1.4	1.1	2.0	1.4	4.2	0.5
ZBTB9	3.9	4.2	0.8	1.2	1.3	1.0	2.1	5.5	0.9
LOC643623	3.6	5.1	1.0	1.5	0.8	2.4	1.5	4.4	0.6
MINA	3.2	4.3	0.8	1.4	1.1	1.6	1.9	5.5	1.0
ANKMY2	3.4	5.3	0.8	1.6	1.5	1.5	1.2	4.7	0.8
COMMD1	2.3	5.4	1.0	2.4	2.0	1.7	1.4	3.8	0.9
KIAA1704	3.8	4.9	1.0	1.9	1.1	1.6	1.5	4.2	0.8
SNORA65	4.6	8.1	1.7	1.1	0.3	0.9	1.2	2.5	0.5
DHX32	2.5	4.5	0.8	1.8	1.5	1.3	1.9	5.7	0.8
YARS2	3.6	5.1	0.9	1.4	1.2	1.5	1.4	5.1	0.7
PEAK1	2.7	3.6	0.7	1.4	1.1	0.9	1.9	7.3	1.1
ZNF362	3.9	3.4	1.4	1.9	1.6	1.9	2.1	3.7	0.8
MED26	2.8	3.8	1.2	1.5	1.8	1.9	2.1	4.6	1.3
UCN	4.7	4.9	1.6	1.4	1.3	0.8	1.3	4.1	0.6
ZNF778	3.8	5.1	1.2	1.2	1.2	0.9	1.6	5.0	0.8
RPAP3	3.2	4.3	0.8	2.1	1.3	1.9	1.5	4.9	0.7

ISG20	3.0	4.6	1.3	0.7	1.3	6.8	0.6	2.0	0.6
MCAT	3.0	4.8	1.2	1.6	1.5	1.6	2.0	4.3	0.7
LOC100130285	4.0	5.8	0.7	1.2	0.8	2.2	1.4	4.2	0.6
EFNA3	3.6	3.4	1.4	0.8	1.3	1.2	2.4	5.1	1.6
LOC101929096	3.4	4.3	1.4	1.6	1.4	1.4	2.0	4.3	0.9
LOC101927582	3.5	6.0	0.7	1.1	0.7	2.3	1.4	4.6	0.6
BBS10	3.5	5.2	1.0	2.3	1.7	2.1	1.1	3.2	0.7
C14orf159	3.7	5.4	1.0	1.5	1.4	1.1	1.6	4.3	0.8
FAM89A	3.8	4.9	1.1	1.8	1.2	1.2	2.0	4.2	0.6
LOC101928209	4.6	5.5	2.3	1.4	1.5	1.3	1.2	2.3	0.7
KLHL11	3.0	4.4	0.9	1.4	1.2	1.7	1.8	5.1	1.1
KLHL24	3.0	5.0	0.6	1.2	0.8	1.7	1.7	5.7	0.9
NUDT8	2.7	3.8	0.9	1.6	1.4	1.3	2.7	4.9	1.5
LRBA	3.7	4.4	0.8	1.4	1.1	2.0	1.5	5.0	0.8
LYSMD1	2.7	5.7	0.7	2.0	1.6	0.9	1.6	4.7	0.8
SLC35G2	2.4	5.8	0.8	1.8	1.2	1.4	1.3	5.3	0.7
C16orf70	3.7	4.5	0.7	2.0	1.6	1.1	1.5	4.5	1.1
DCPS	3.6	4.1	1.1	1.9	2.3	1.2	1.7	3.9	0.9
HIVEP3	3.6	4.4	1.2	1.1	1.1	1.0	1.8	5.5	1.1
LOC101927663	3.7	5.2	1.0	2.2	1.4	0.8	2.2	3.4	0.9
SLC16A13	3.8	4.1	1.2	3.0	2.5	2.1	0.9	2.8	0.4
TAOK3	4.0	4.0	1.2	1.8	1.7	1.9	1.6	3.9	0.6
RPF1	3.6	4.7	0.9	1.6	1.4	2.1	1.4	4.1	1.0
TRAPPC8	3.2	5.2	0.9	1.7	1.2	1.6	1.4	4.7	0.9
HMGN3	3.4	4.3	0.7	2.4	3.0	1.8	1.6	2.8	0.7
LOC101928120	3.5	3.5	0.9	1.9	1.4	1.2	2.7	4.5	1.0
GRAMD3	3.2	4.1	0.8	3.4	2.8	2.2	1.1	2.5	0.5
OSGIN1	4.5	4.7	1.2	2.0	2.1	1.4	1.3	2.8	0.7
SNHG11	3.6	4.0	1.0	1.8	1.2	1.4	2.3	4.3	1.0
LRRC20	2.9	2.5	0.7	3.6	3.7	1.8	1.1	3.5	0.7
ARMCX3	2.5	4.5	0.8	1.9	1.2	1.1	2.1	5.5	1.0
CIB2	4.1	3.4	0.7	2.6	2.2	2.4	1.6	2.8	0.7
LINC00673	2.6	4.6	0.8	0.9	0.7	1.1	2.4	6.4	1.0
ADAMTS12	2.7	3.8	0.9	4.0	3.3	2.6	0.8	2.0	0.5
GABRA3	3.8	4.4	0.9	1.4	1.3	0.7	2.0	5.1	1.0
BRICD5	4.0	3.9	0.9	1.5	1.5	0.8	2.5	3.6	1.8
PGAP3	3.2	4.0	1.1	1.1	1.0	1.1	2.2	5.8	1.1
BTRC	2.8	4.3	0.8	1.7	1.6	1.6	1.6	5.3	0.9
LCOR	2.8	4.5	0.8	1.2	1.3	1.6	1.7	6.0	0.6
TSPYL2	3.2	3.0	1.0	1.8	1.7	2.8	1.9	4.0	1.2
SERTAD4-AS1	2.1	2.1	0.2	2.9	2.7	1.4	2.6	5.5	1.1
NAIF1	3.7	4.1	1.0	1.6	1.6	1.1	2.1	4.5	1.1
TEF	3.1	3.0	1.0	1.7	1.6	1.1	2.3	5.5	1.3
GPATCH2L	3.5	4.6	1.1	1.8	1.2	1.4	1.8	4.4	0.9
FAM63B	3.3	4.2	1.0	1.7	1.3	1.9	1.7	4.8	0.8
C11orf30	3.2	3.9	0.8	1.4	1.4	1.2	1.8	5.8	1.1
PCF11	3.5	5.0	0.9	1.2	1.2	1.7	1.6	4.6	0.8

PAOX	3.7	4.8	1.2	1.4	1.2	2.2	1.8	3.7	0.7
HSD17B7	3.7	5.6	0.8	1.6	1.4	1.2	1.3	4.5	0.6
GEM	4.7	5.6	1.1	1.8	1.3	1.2	1.5	2.7	0.7
CNNM2	3.3	4.3	1.4	1.4	1.6	1.1	1.7	4.6	1.1
PCED1B-AS1	3.5	5.5	1.0	1.0	0.9	2.3	1.3	4.4	0.6
AMMECR1	3.8	5.5	1.1	1.1	1.0	1.2	1.8	4.4	0.7
AVPI1	3.6	4.9	1.6	2.2	1.8	1.3	1.1	3.4	0.7
FOXP1	3.0	2.3	0.8	1.2	1.1	2.6	2.2	6.0	1.3
LOC100505767	4.3	5.8	1.4	1.0	1.2	0.7	1.2	4.3	0.5
BOC	2.3	3.1	0.8	0.5	0.4	0.2	3.1	8.2	1.8
FBXO42	3.5	4.5	1.0	1.4	1.3	1.7	1.3	4.8	1.0
FDX1	3.2	4.5	0.8	1.7	1.2	3.0	1.5	3.6	0.9
TCHP	3.8	3.7	1.5	1.8	2.1	1.5	2.2	3.0	0.9
MMACHC	4.2	3.9	1.2	2.7	2.0	1.0	1.2	3.5	0.8
UTP14C	2.8	3.6	0.8	1.7	1.2	1.1	2.1	6.0	1.2
C17orf80	3.2	4.1	0.8	1.8	1.6	1.6	1.6	4.9	0.9
NHEJ1	3.6	4.0	0.5	1.6	1.5	1.2	1.8	5.6	0.7
RHBDL1	5.1	5.6	1.3	0.8	0.8	0.4	2.2	3.4	0.9
UFSP2	2.9	4.9	0.7	1.7	1.3	1.5	1.7	5.0	0.9
NBPF15	4.7	6.3	0.7	1.8	0.8	0.6	2.0	3.2	0.4
DTNB	2.6	4.7	0.9	2.8	2.4	1.8	1.2	3.5	0.6
CLN5	3.7	4.2	0.8	1.6	1.6	1.3	1.9	4.4	0.9
SUV39H2	2.9	4.8	0.9	1.4	1.1	2.7	1.3	4.6	0.7
LOC101928649	3.5	5.2	1.0	1.2	1.1	1.1	1.6	4.7	1.0
NAA15	3.1	5.1	0.7	1.9	1.5	2.2	1.2	4.1	0.7
NOL10	3.1	4.3	0.7	2.0	1.5	1.8	1.7	4.5	0.7
SERTAD4	0.9	1.7	0.3	3.9	2.7	3.0	1.5	5.4	1.1
CIDEB	4.0	4.6	1.0	1.5	1.4	0.6	1.6	5.0	0.7
ZNF517	3.3	4.8	1.2	1.3	1.3	1.6	1.8	4.5	0.6
DHFR	3.1	3.8	0.8	3.1	2.3	2.7	1.0	3.2	0.5
IMPACT	3.9	5.4	0.8	1.8	1.5	1.8	0.9	3.6	0.7
USP33	3.5	5.0	0.5	1.8	1.1	1.9	1.3	4.4	0.9
SLC30A4	2.9	4.4	0.7	1.3	1.2	1.2	1.7	6.0	1.1
SPRTN	3.4	4.5	0.8	1.8	1.4	1.7	1.9	4.1	0.9
METTL15	4.1	5.4	0.9	1.6	1.3	2.1	1.1	3.3	0.6
AARS2	3.1	4.1	0.6	2.2	1.7	1.6	2.0	4.3	0.7
ZC3H13	3.5	3.5	0.9	1.8	1.5	1.6	1.9	4.7	1.0
DHX58	4.3	5.4	1.1	1.2	1.0	1.8	1.5	3.3	0.8
UBL3	1.9	3.9	0.5	1.6	1.2	3.1	2.0	5.6	0.6
PTPN21	3.7	4.5	1.5	2.0	2.1	1.0	1.4	3.5	0.7
BCL6	3.3	3.9	1.1	2.4	2.1	2.2	1.3	3.2	0.9
C1D	2.6	5.3	0.8	1.6	1.0	2.8	1.2	4.5	0.5
MYBPH	5.8	6.9	2.1	1.1	0.9	0.2	1.0	2.1	0.4
AQP4-AS1	3.5	4.2	0.8	1.2	0.8	4.1	1.4	3.5	0.8
RNF157	3.8	4.3	1.2	2.4	2.5	1.7	1.1	2.7	0.7
ITPR2	3.4	4.9	0.9	1.3	1.0	1.7	1.5	4.9	0.8
GCC2	3.4	4.7	0.7	1.6	1.1	1.5	1.6	4.8	0.9

TBC1D8	3.4	4.9	0.8	2.0	1.6	1.6	1.4	4.0	0.6
CENPM	3.4	4.8	0.9	2.4	2.2	2.1	1.1	3.1	0.4
OCLN	3.4	5.7	1.0	1.6	1.5	1.0	1.3	3.9	0.8
FLJ20021	3.0	2.8	1.2	1.8	1.8	0.6	2.6	5.5	1.0
HCG11	3.5	5.2	0.9	1.1	0.8	2.6	1.3	4.2	0.7
VPS8	3.3	4.7	0.7	2.0	1.5	2.3	1.1	4.0	0.6
OSBPL7	4.3	4.6	1.1	1.5	1.3	1.4	1.8	3.5	0.9
IQCB1	3.2	4.3	0.9	2.1	1.6	1.4	1.6	4.2	1.0
TJP2	3.7	4.2	1.1	3.5	2.8	2.3	0.7	1.6	0.3
KIAA1211	4.2	4.2	1.4	2.0	1.8	1.5	1.6	3.0	0.6
SUV420H1	3.2	4.9	0.8	1.1	0.9	1.4	1.8	5.2	0.9
MDK	4.7	4.7	1.6	1.7	1.8	1.5	1.1	2.7	0.6
ANKS3	3.4	4.2	0.8	1.2	1.2	1.2	2.2	5.1	0.9
ZNF154	3.6	5.3	1.0	1.2	0.8	2.3	1.3	4.2	0.5
NLRC5	3.0	3.8	0.7	1.2	1.2	6.3	1.1	2.5	0.5
BLZF1	2.5	4.3	0.6	1.5	1.2	1.6	1.6	5.9	1.0
KIAA0247	2.4	3.3	0.9	0.8	0.7	2.9	2.2	5.8	1.2
UTRN	3.3	4.7	0.9	1.5	1.2	3.1	1.1	4.0	0.5
CPT2	3.4	4.9	0.8	1.6	1.2	1.1	1.7	4.8	0.7
MAP9	3.1	4.1	0.6	2.0	0.8	1.7	1.7	5.4	0.9
PRKAB1	3.8	4.5	1.3	1.4	1.1	1.2	1.8	4.2	0.9
PANK4	3.9	4.5	0.9	1.4	1.4	1.7	1.6	3.8	1.0
UBIAD1	2.6	3.1	0.8	1.6	1.8	1.2	2.1	5.7	1.2
GOLGA8A	4.0	4.6	1.0	1.5	1.1	1.2	1.5	4.4	0.9
IQCK	3.6	4.3	0.9	2.7	2.5	2.4	1.0	2.2	0.5
TBRG1	3.1	4.6	0.8	1.8	1.0	1.3	1.8	5.0	0.8
HACL1	2.7	4.6	0.8	1.4	1.1	1.4	1.8	5.6	0.8
HS3ST3B1	3.7	4.3	1.1	1.4	1.3	1.7	1.7	3.8	1.1
NAA30	3.2	3.7	1.0	1.7	1.7	2.2	2.0	3.9	1.0
IFNGR1	3.1	4.5	0.7	1.2	1.2	1.8	1.6	4.8	1.1
TMEM126B	2.6	4.9	0.5	2.8	1.6	2.1	1.1	4.1	0.6
ATAD3A	2.6	3.5	1.2	1.9	1.7	1.1	2.7	4.2	1.3
LOC102723630	3.1	5.8	0.9	1.3	0.8	1.3	1.4	5.1	0.5
LOC101927187	2.6	4.3	0.9	1.3	1.0	0.9	2.3	5.7	1.0
PCGF6	3.0	4.6	0.7	2.2	2.0	1.6	1.5	3.8	0.7
REV1	2.9	4.1	0.9	1.6	1.2	1.7	1.6	5.2	0.9
CLMP	3.2	3.9	0.7	1.1	1.1	0.8	2.2	5.7	1.3
CLCN6	3.8	5.1	1.1	1.0	1.0	0.9	1.7	4.5	0.9
MOAP1	3.0	5.2	0.9	1.4	1.1	2.5	1.3	4.2	0.5
PAX2	2.9	2.5	0.9	1.8	1.3	1.0	2.2	6.1	1.5
RBM19	4.0	4.1	1.1	1.9	1.7	1.7	1.6	3.3	0.8
WDR48	3.2	5.0	0.8	1.1	1.0	1.4	1.6	5.0	1.0
PTPRG-AS1	3.7	4.7	0.9	1.0	0.8	3.0	1.4	4.0	0.6
LARP7	3.3	4.4	1.0	1.5	1.4	1.6	1.3	4.6	1.0
BTN3A1	3.1	4.3	0.8	1.1	1.1	3.9	1.4	3.7	0.6
DEPDC4	3.3	4.3	1.0	1.3	0.8	3.6	1.3	4.0	0.5
MPP4	3.3	5.4	1.0	1.1	0.7	1.8	1.2	4.8	0.7

TRMU	3.4	3.8	0.9	1.3	1.2	1.9	2.0	4.5	1.1
C4orf46	2.6	3.5	1.5	3.2	2.2	1.4	1.9	3.1	0.7
ENTPD3-AS1	3.1	5.5	0.9	1.3	0.9	1.6	1.6	4.5	0.6
ZP3	3.1	5.5	0.7	2.2	1.5	0.8	1.6	3.9	0.6
CBX2	2.5	3.4	1.2	2.5	1.8	1.4	2.0	4.4	0.9
LOC100128573	3.9	6.2	0.5	1.2	0.7	1.7	1.3	4.2	0.5
OLIG2	5.1	4.9	1.7	0.9	1.1	0.4	1.7	3.6	0.7
DIEXF	3.3	4.7	0.9	1.8	1.5	2.0	1.3	3.6	0.8
DHRS11	3.1	4.6	0.9	3.0	2.6	1.7	1.2	2.5	0.5
SDAD1	3.6	5.5	0.9	1.4	1.0	1.3	1.3	4.2	0.7
NPIPB11	4.0	5.4	1.2	0.9	0.8	0.4	1.6	4.7	0.9
ZNF330	3.8	5.3	1.1	2.4	1.1	1.6	1.1	3.0	0.7
POGLUT1	2.5	4.7	0.6	1.7	1.2	1.5	1.9	5.2	0.8
TMEM86B	3.9	4.9	0.9	1.1	0.9	0.7	2.2	4.4	1.0
RASSF4	3.3	3.1	0.6	2.0	1.6	1.3	2.3	4.4	1.4
ARHGEF28	3.7	5.9	1.1	2.3	1.9	1.7	0.7	2.2	0.4
MGC27345	3.5	5.2	1.0	1.1	0.9	1.4	1.3	4.8	0.7
ANK2	3.9	4.3	1.0	1.7	1.6	1.3	1.4	4.2	0.6
PPP2R5A	3.7	4.2	0.8	1.8	1.2	1.8	1.3	4.0	1.2
OXNAD1	2.5	4.6	0.9	1.4	1.4	1.1	1.5	5.6	0.9
CLIP4	3.0	4.8	0.6	1.8	1.3	1.6	1.5	4.6	0.8
FBXO45	3.5	4.4	1.2	1.1	1.4	1.5	1.7	4.5	0.7
MRM1	3.7	4.5	0.7	1.9	1.6	1.6	1.2	3.9	0.7
CARD10	3.5	4.0	1.1	1.8	1.8	1.1	2.2	3.6	0.9
PTPN2	3.2	4.5	0.7	2.1	1.3	1.7	1.3	4.8	0.6
PIGP	3.0	4.5	0.8	1.6	1.8	1.6	1.2	4.9	0.5
LOC101929086	3.4	4.7	1.1	0.8	0.9	0.8	2.1	5.0	1.2
LOC100996255	3.8	5.2	1.1	1.2	1.2	0.9	1.7	4.2	0.7
RELB	3.1	4.0	1.3	1.1	1.3	1.9	1.9	4.2	1.1
LOC101929186	3.3	3.4	0.6	1.4	1.3	1.4	2.0	4.6	1.8
MTHFR	3.9	4.2	1.1	1.3	1.1	1.2	1.8	4.4	0.9
ZBTB11-AS1	3.4	4.3	1.0	1.6	1.2	1.8	1.6	4.2	0.8
LOC101929097	3.9	4.2	0.9	1.3	1.7	1.1	1.7	4.0	1.1
YIPF1	2.9	4.3	1.0	2.2	1.9	1.6	1.3	4.1	0.8
PPT2-EGFL8	3.1	4.1	1.2	1.5	1.4	1.4	1.9	4.3	1.0
TBC1D4	1.9	2.7	0.6	2.0	1.8	1.5	2.5	5.8	1.2
ORC2	3.1	4.9	0.7	1.4	1.2	1.3	1.6	4.7	0.9
CTPS2	3.9	5.6	0.8	1.9	1.5	1.3	1.3	3.1	0.6
MPP6	2.9	4.5	0.6	1.8	1.4	2.0	1.1	4.9	0.7
AP5M1	3.1	5.0	0.7	1.6	1.3	1.7	1.2	4.4	1.0
OGFRL1	3.3	4.8	1.2	2.1	1.6	2.0	1.2	3.0	0.8
RARRES3	3.6	4.7	0.7	0.7	0.9	2.9	1.7	4.2	0.5
TTLL4	3.4	4.4	0.9	1.9	1.3	1.6	1.4	4.4	0.7
IFT80	2.9	4.3	0.7	2.1	1.1	1.4	1.6	5.3	0.7
SPAG5	3.0	3.9	0.9	3.9	3.1	2.9	0.5	1.4	0.3
ANKRD23	3.2	4.2	0.9	1.5	1.3	1.7	1.8	4.5	0.8
MTHFSD	3.5	4.2	1.3	1.2	1.8	1.0	1.4	4.5	1.1

FRMD5	4.6	5.9	1.3	1.0	1.3	0.7	1.2	3.2	0.6
NCAM1	3.2	3.8	1.0	1.6	1.4	1.1	2.1	4.5	1.1
LOC101926960	3.5	5.7	0.8	1.0	0.9	2.3	1.2	4.0	0.6
FAM220BP	3.5	3.1	1.6	1.8	1.8	0.9	2.1	4.1	1.1
DHRS4-AS1	3.3	4.7	0.9	1.3	1.0	2.1	1.5	4.3	0.8
CASP6	3.0	5.0	0.5	2.0	1.5	1.5	1.3	4.4	0.7
PLA2G12A	3.4	4.2	1.0	2.4	2.1	1.4	1.2	3.8	0.5
ITPKA	3.1	4.3	1.1	1.9	1.4	0.9	2.2	4.2	0.7
ZCCHC9	2.8	4.7	0.7	2.3	1.6	2.4	1.5	3.1	0.8
LOC101929159	3.3	5.6	0.8	1.1	0.8	2.1	1.1	4.6	0.5
TMTC1	2.7	3.4	0.8	2.2	1.7	1.4	1.7	5.1	0.9
UBE2T	2.7	3.6	0.9	2.9	3.7	2.2	0.7	2.5	0.7
C1orf52	2.8	4.0	0.9	1.4	1.3	1.8	2.3	4.3	1.1
TCF20	2.6	3.7	0.8	1.7	1.6	2.3	1.6	4.7	0.9
NOA1	3.1	4.6	1.0	1.8	1.8	1.6	1.7	3.6	0.7
SOX13	3.5	3.9	1.3	1.9	2.4	1.1	1.6	3.6	0.6
GABBR1	4.1	5.5	1.1	1.3	0.9	0.7	1.5	4.0	0.6
CCNYL1	3.3	3.8	0.8	1.6	1.7	1.5	1.9	3.8	1.3
POLR1B	2.8	4.2	0.7	1.4	1.2	1.4	1.7	5.7	0.8
APC2	3.5	4.1	1.3	1.9	1.8	1.1	1.7	3.6	0.8
PTPN3	3.1	4.5	0.9	1.9	1.7	1.8	1.2	4.0	0.7
ZNF134	3.2	4.4	0.7	1.3	1.5	1.1	1.5	4.9	1.3
SDF2L1	2.2	3.8	0.6	2.2	1.8	2.2	2.4	3.9	0.8
DENND3	3.6	4.4	1.0	1.5	1.8	2.9	1.1	2.8	0.6
NUPL2	2.9	4.0	1.4	1.7	1.4	2.0	1.4	4.1	0.8
SBF2-AS1	3.2	5.3	0.8	2.1	1.4	1.6	0.9	3.8	0.6
WDR90	3.5	4.4	0.9	1.7	1.3	1.1	2.0	4.0	0.8
ALG13	2.9	4.6	1.1	1.6	1.0	0.9	1.5	5.1	1.2
C16orf87	2.8	3.5	1.0	2.0	1.3	1.8	1.4	5.0	0.9
PLSCR4	3.5	4.9	0.9	2.6	2.6	1.9	0.7	2.0	0.5
FAM134B	3.9	5.8	1.2	1.9	1.7	2.0	0.9	1.9	0.4
TCTEX1D4	3.8	6.4	1.1	0.7	0.7	2.2	1.1	2.8	0.9
INO80	3.1	4.6	0.8	1.6	1.3	1.6	1.4	4.4	0.9
ZFYVE28	3.7	3.9	1.3	1.2	1.0	1.1	1.6	5.1	0.9
LOC101928103	3.3	6.0	0.8	0.9	0.6	2.0	0.8	4.8	0.5
TADA2A	3.9	4.7	0.9	1.5	1.2	1.0	1.4	4.6	0.7
SLC26A10	4.0	4.8	0.9	1.7	1.4	0.6	1.6	3.9	0.7
LOC101928953	3.4	4.1	1.0	1.3	1.4	1.3	2.0	4.4	0.9
LY6G5B	2.9	5.4	0.6	1.0	1.1	0.9	1.7	4.9	1.1
NBEAL2	3.3	4.2	0.9	1.7	1.4	3.5	1.3	2.8	0.6
LOC728485	3.6	5.2	0.9	1.1	0.8	1.6	1.4	4.4	0.7
RSPH3	3.2	3.4	1.0	1.7	1.5	1.1	2.0	4.8	1.1
KIAA1429	3.2	4.6	0.7	1.3	1.1	1.4	1.6	4.7	0.9
GPR180	2.8	3.9	0.8	1.8	1.0	1.1	1.9	5.5	1.1
PPP2R5E	3.1	4.3	0.6	2.0	1.6	1.6	1.4	4.2	0.8
RABL2B	3.6	4.0	0.9	1.7	1.1	0.6	2.0	4.7	1.2
EIF4ENIF1	3.7	4.6	0.8	1.3	1.6	1.0	1.7	4.0	1.0

ZNF784	3.2	4.5	1.3	1.4	1.4	0.7	2.1	4.3	0.9
C14orf169	3.1	4.0	1.1	1.9	1.9	1.6	1.4	3.7	1.0
NKAP	2.6	4.5	0.8	1.2	1.4	1.2	1.7	5.3	0.9
INSR	3.9	4.8	1.0	1.5	1.4	1.1	1.3	4.0	0.7
ANXA2R	5.1	5.9	1.6	1.2	1.0	0.9	1.1	2.5	0.3
INTS4	3.1	4.7	0.8	1.4	0.8	1.4	1.5	5.1	0.9
EPS8	3.7	4.9	1.0	1.8	1.8	1.3	1.2	3.3	0.6
ZFHX3	3.4	4.0	1.3	1.8	2.0	1.0	1.5	3.9	0.8
DENND4C	3.0	4.8	0.7	1.4	1.0	1.5	1.4	5.1	0.9
TNIK	2.6	2.9	0.8	1.5	1.3	2.9	2.0	4.7	1.1
TMEM110	3.3	1.1	0.2	2.1	1.9	2.0	1.7	6.7	0.7
NLRC3	3.3	5.2	0.8	1.2	0.9	2.5	1.2	4.0	0.6
UCHL5	3.0	3.8	0.9	1.9	1.9	1.5	1.9	3.9	0.8
PYCRL	2.0	3.3	0.6	1.8	1.4	1.0	2.3	6.0	1.2
ACOT2	3.5	4.8	0.7	1.9	1.5	1.6	1.3	3.7	0.7
TM7SF2	3.7	4.8	0.8	0.7	0.5	0.9	2.2	5.1	1.0
ZBED5-AS1	3.6	5.8	0.8	1.2	1.0	1.4	1.0	3.9	0.9
DNAJC15	3.0	6.0	0.9	1.2	1.4	1.4	1.6	3.6	0.5
CLK1	2.9	4.0	0.5	1.3	0.7	2.5	1.7	4.8	1.2
GMDS	2.9	4.8	1.0	1.5	1.7	2.1	1.3	3.6	0.6
SMPD2	3.2	4.1	0.7	1.7	1.4	1.6	1.8	4.0	1.0
NBAS	3.1	4.8	0.8	1.4	1.1	1.3	1.5	4.9	0.7
SEMA3A	3.8	5.5	1.0	0.6	0.8	0.6	1.6	4.9	0.8
RNF141	2.9	4.9	0.9	1.3	0.8	2.8	1.2	4.4	0.3
NETO1	3.7	4.1	1.2	2.2	1.5	0.8	1.7	3.4	0.9
TMA16	3.4	5.0	1.1	1.9	1.3	1.4	1.3	3.5	0.7
KANSL2	3.2	4.9	0.8	1.5	1.4	1.4	1.7	4.1	0.8
ENO3	3.6	4.5	0.5	2.5	1.5	1.8	1.3	3.1	0.7
ZNF641	3.5	4.6	1.2	2.1	2.0	0.8	1.4	3.5	0.5
BRAP	3.0	4.3	0.8	1.8	1.6	1.6	1.6	4.1	0.8
IBTK	3.2	4.8	0.5	1.5	1.1	1.5	1.4	4.7	0.8
KRTAP5-AS1	4.2	4.4	1.5	1.3	1.2	0.7	1.4	4.0	1.0
STXBP5	3.4	4.5	0.9	1.5	1.3	1.5	1.4	4.4	0.7
FNBP1L	3.0	3.2	0.8	1.7	1.4	1.4	1.9	5.1	1.1
MED6	3.6	4.9	0.8	1.5	1.4	1.3	1.4	4.0	0.6
FKBP11	2.6	4.9	1.3	1.1	0.8	1.1	1.5	5.1	1.1
FAM43A	3.8	3.9	1.0	1.0	0.9	1.6	2.3	4.1	1.0
EVI5	3.0	4.3	0.8	1.3	1.3	1.1	1.8	5.0	0.9
TFB2M	2.9	4.9	0.6	1.4	1.0	1.2	1.8	4.8	0.8
LOC101927093	3.8	4.3	0.9	2.1	1.6	1.3	1.4	3.5	0.7
CCDC74B	4.0	4.2	1.0	2.9	2.8	2.0	1.0	1.2	0.4
PARP14	3.1	4.7	0.7	1.4	1.1	1.8	1.3	4.7	0.6
FGFR3	4.3	5.1	1.8	1.7	2.2	1.0	1.0	1.8	0.5
ZNF365	3.0	3.9	0.9	1.2	1.0	1.0	2.1	5.3	1.1
SAAL1	3.7	3.9	0.8	2.0	1.6	1.8	1.4	3.4	0.8
RGL1	2.5	3.8	0.7	1.8	1.5	1.4	1.7	5.2	0.9
ZNF710	3.3	3.9	1.1	1.5	1.7	1.8	1.8	3.5	0.9

KCNG1	2.7	3.8	1.1	0.9	0.9	0.3	2.5	5.9	1.4
PKMYT1	2.4	3.5	0.7	3.8	3.3	2.3	0.9	2.2	0.4
LOC101928645	4.1	5.6	1.0	1.2	0.8	1.0	1.3	3.8	0.7
ABHD12B	3.6	4.6	1.0	0.9	0.8	3.3	1.4	3.4	0.5
ASMTL-AS1	3.2	5.2	1.0	1.4	0.9	1.5	1.5	4.1	0.7
ASMTL-AS1	3.2	5.2	1.0	1.4	0.9	1.5	1.5	4.1	0.7
TTC33	3.0	4.1	0.9	2.4	1.2	1.5	1.4	4.5	0.5
ZNF37A	2.8	3.4	0.5	1.3	1.0	1.1	1.7	6.5	1.1
DPYSL5	3.3	3.9	1.2	3.1	2.5	2.0	0.9	2.2	0.3
CCDC71	3.5	4.1	0.8	1.9	2.0	1.8	1.3	3.4	0.8
GPD1L	3.0	4.0	0.7	1.7	1.2	1.5	1.9	4.4	1.0
CHRM3	3.0	4.2	1.0	0.4	0.3	0.2	2.5	6.6	1.3
BCL9	2.9	3.2	0.7	1.4	1.5	1.5	1.9	5.1	1.1
PDXP	2.5	4.1	1.2	1.8	1.6	1.3	2.0	4.1	0.9
MTIF2	3.4	4.6	0.6	1.6	1.0	1.8	1.4	4.4	0.6
FAM104B	3.4	3.9	1.0	1.6	1.2	1.3	1.7	4.7	0.6
TNFAIP3	2.3	3.2	0.7	1.3	1.1	10.0	0.3	0.5	0.1
PYROXD2	3.8	4.4	1.2	1.7	1.1	1.3	1.7	3.5	0.7
STAG2	3.2	4.8	0.9	1.5	1.2	1.8	1.2	4.1	0.7
LOC100505812	3.7	5.2	0.7	1.0	0.8	1.9	1.3	4.0	0.8
RHPN1	3.3	4.1	0.9	1.9	1.9	1.2	2.3	3.2	0.7
ERCC6L2	3.0	4.2	0.5	1.5	1.1	1.7	1.4	5.0	0.9
AGT	6.0	7.2	1.5	1.2	0.9	0.6	0.5	1.0	0.3
BBS7	3.4	5.7	0.7	1.4	1.0	1.6	1.2	3.8	0.6
BAG5	2.8	3.7	0.6	1.7	1.1	2.2	1.8	4.7	0.8
KIF4A	3.1	5.0	0.9	2.2	1.5	2.4	0.9	3.0	0.4
TMEM69	3.1	4.1	0.6	1.7	1.3	1.1	1.8	4.5	1.2
C18orf61	3.4	4.9	0.9	1.2	0.7	2.2	1.3	4.3	0.6
GPRIN1	2.7	3.1	0.7	1.6	1.3	1.0	2.1	5.9	1.0
DIS3L	3.2	4.6	0.8	1.5	1.5	1.8	1.2	4.0	0.7
HIVEP2	3.0	3.8	0.9	1.3	1.3	1.9	1.5	4.9	0.8
C9orf40	3.0	4.2	0.9	3.0	2.2	1.2	1.6	2.3	1.0
CACTIN-AS1	4.1	4.7	1.1	1.4	1.3	1.0	1.7	3.6	0.4
TBC1D2B	2.5	3.7	0.7	2.1	1.8	2.2	1.5	4.1	0.8
MON2	2.8	4.9	0.6	0.8	1.0	1.2	1.4	5.8	0.8
RBM5-AS1	3.1	4.5	0.8	1.3	0.7	2.8	1.4	4.3	0.4
VPS13B	3.0	4.6	0.8	1.2	0.9	2.3	1.4	4.5	0.8
LOC101927054	3.3	5.9	0.7	1.1	0.9	1.2	1.5	4.2	0.5
TCTEX1D2	3.8	4.3	1.1	1.8	0.7	1.4	1.8	3.8	0.7
TBC1D22A	3.0	4.1	1.1	1.6	1.5	2.0	1.4	3.7	0.8
BTBD7	3.1	4.4	0.7	1.5	1.2	1.1	1.5	4.7	0.9
KCNK1	3.4	4.7	1.2	2.0	1.9	1.4	1.5	2.6	0.4
SLC26A2	2.8	4.9	0.6	1.5	1.2	1.3	1.4	4.7	0.9
TMEM184C	2.9	4.9	0.8	1.4	1.0	1.6	1.6	4.3	0.8
LOC100507033	3.1	5.1	0.9	1.0	0.8	2.2	1.2	4.3	0.7
THEMIS	3.2	4.5	0.9	1.3	1.1	1.9	1.5	4.1	0.7
APOPT1	3.5	3.2	1.1	1.8	1.0	1.3	1.7	4.1	1.5

EZH1	3.5	5.0	0.8	1.2	1.2	1.2	1.5	4.1	0.8
INHBE	1.8	1.9	0.5	0.3	0.3	0.3	3.2	9.3	1.7
NAA25	3.1	5.5	0.9	1.6	1.2	1.6	1.1	3.8	0.5
LOC101929059	3.1	6.3	0.9	0.6	0.7	1.7	0.8	4.5	0.6
LINC00337	3.1	5.4	0.9	1.4	0.8	1.5	1.4	4.3	0.5
MICB	3.3	4.1	0.8	2.6	2.6	1.2	1.3	2.8	0.4
GLTSCR1L	3.1	4.1	0.8	1.2	1.1	1.3	1.7	5.1	0.7
HEATR1	3.3	5.1	0.8	1.6	1.3	1.7	1.1	3.7	0.7
THAP5	3.0	4.1	0.6	1.5	1.3	2.0	1.2	4.8	0.7
LOC101928218	3.4	5.7	0.7	1.5	1.2	0.9	1.4	3.7	0.7
KIAA0556	3.4	4.9	1.1	1.3	1.2	1.0	1.5	3.9	0.9
TTLL11	2.6	3.3	0.8	1.4	1.0	0.7	2.4	5.6	1.4
DPH5	3.3	3.6	0.6	1.8	1.6	2.0	1.4	4.0	0.8
EXOC5	3.0	5.2	0.5	1.6	1.1	1.8	1.1	4.1	0.7
TTC9	3.9	4.2	1.4	2.4	1.9	1.6	1.2	1.6	0.9
ZC3HC1	3.4	4.3	1.1	2.0	1.4	1.3	1.3	3.6	0.7
ACAD8	2.3	4.4	0.9	1.4	0.9	1.5	2.1	4.6	1.0
LRRN4CL	3.8	5.0	1.5	1.1	1.1	0.8	1.6	3.6	0.8
KLC1	2.7	4.1	1.0	1.7	1.3	1.4	1.7	4.5	0.7
EFTUD1	3.3	4.7	0.7	1.7	1.3	1.4	1.2	4.1	0.9
CSTF2T	3.1	3.7	0.7	1.7	1.4	1.5	1.8	4.2	0.9
RNF217	3.4	4.0	1.0	1.7	1.9	1.0	1.6	3.8	0.7
C6orf211	3.9	4.0	0.7	1.9	1.3	1.6	1.2	3.9	0.7
LOC100130872	3.4	4.9	0.9	1.1	0.8	1.7	1.3	4.3	0.7
WDR92	3.0	3.9	0.9	2.0	1.5	1.3	1.4	4.1	1.1
TDP1	2.9	4.2	1.0	1.8	1.6	1.4	1.4	4.0	0.8
DYM	2.6	4.5	0.7	1.4	1.6	2.0	1.4	4.2	0.7
CHUK	2.7	4.7	0.6	1.7	1.3	1.6	1.3	4.7	0.5
PTGES2-AS1	3.6	5.5	1.0	1.2	0.9	0.9	1.2	4.0	0.7
DNAJC16	2.8	4.1	0.6	1.2	0.8	1.1	1.6	5.8	1.2
FBF1	3.0	3.4	1.0	1.9	1.7	1.2	1.9	4.2	0.8
LOC101929403	2.6	4.5	0.8	1.1	0.7	3.4	1.2	4.0	0.7
LOC100506472	3.3	5.0	0.9	0.9	0.9	2.2	1.3	4.0	0.6
MCTS1	2.4	4.6	0.6	1.9	1.1	1.2	1.6	4.6	1.2
TINCR	3.1	4.7	0.9	1.1	1.0	2.5	1.2	3.9	0.6
ENTPD7	3.6	4.7	0.7	1.3	0.8	0.9	1.5	4.9	0.8
FER	3.0	4.5	0.7	1.7	1.2	1.5	1.1	4.7	0.6
C1orf51	4.0	4.3	1.1	1.4	1.4	1.1	1.3	3.4	1.0
RPF2	3.0	4.3	0.7	1.5	1.2	2.4	1.2	4.3	0.5
SYNJ1	2.8	3.7	0.8	1.4	1.2	1.3	1.7	5.2	1.0
C9orf156	3.2	4.0	0.9	1.5	1.7	1.3	1.4	4.1	1.0
PNPLA8	2.8	3.9	0.7	1.4	1.1	1.5	1.6	5.2	0.8
TCTN2	3.2	4.5	0.8	2.5	1.9	1.5	0.9	3.1	0.7
TMEM53	2.9	4.1	1.3	1.6	1.3	0.5	1.7	4.3	1.3
GNL2	3.1	3.2	0.7	1.5	1.2	1.4	2.0	5.0	1.0
MAPK8	2.9	4.3	0.6	1.4	0.9	1.6	1.3	5.0	1.0
LIG4	3.0	4.9	0.8	1.7	1.0	1.5	1.1	4.3	0.8

AUTS2	3.3	3.7	1.0	1.8	1.8	1.3	1.6	3.8	0.6
LOC101927357	3.1	5.1	0.9	1.1	0.7	2.2	1.2	4.3	0.5
RNF24	2.1	3.9	0.4	1.8	1.6	1.7	1.9	5.1	0.5
SLC4A8	3.8	4.7	1.1	1.4	1.3	2.2	1.0	3.0	0.6
RNF138	4.1	3.2	0.8	1.5	1.1	2.0	1.2	4.4	0.6
SLC45A4	2.8	3.3	0.8	1.3	1.2	1.4	2.1	5.0	1.0
BOLA3-AS1	2.9	5.8	0.8	1.4	1.1	1.5	1.1	3.7	0.5
DUSP18	2.9	3.7	0.7	1.3	1.2	0.7	1.7	5.7	0.9
FOSB	0.2	0.3	0.0	0.1	0.1	17.3	0.2	0.6	0.2
TANGO6	3.2	4.4	0.7	2.0	1.6	2.3	1.1	3.2	0.6
PHACTR4	3.1	3.7	0.8	1.9	1.8	1.2	1.4	4.2	0.8
PPM1H	3.4	4.1	1.0	1.3	1.5	1.0	1.5	4.1	1.0
COQ6	2.7	4.0	0.8	1.7	0.9	1.9	1.8	4.2	1.0
NDUFB2-AS1	3.0	4.2	1.0	1.4	1.2	1.4	1.6	4.4	0.7
UCHL3	2.2	4.1	0.6	1.5	1.2	2.0	1.5	4.8	0.9
C6orf123	3.5	5.0	0.9	1.1	0.9	2.0	1.4	3.7	0.5
PNP	3.0	5.0	1.0	1.6	1.4	1.7	1.3	3.6	0.3
FAM173B	2.1	5.0	0.7	1.5	1.8	1.4	0.9	4.9	0.6
FGF12	3.3	3.6	1.0	2.9	2.8	1.7	1.0	2.2	0.3
EMC2	3.4	4.0	0.8	1.6	1.3	1.7	1.1	3.8	1.0
KIAA0141	3.5	4.0	1.1	1.3	1.1	0.8	1.7	4.5	0.7
KLF9	2.7	3.6	1.2	0.6	0.6	2.6	1.6	4.3	1.6
TRMT1L	2.9	4.3	0.7	2.1	1.5	2.2	1.2	3.6	0.5
NUP205	2.9	4.6	0.7	1.6	1.3	1.8	1.3	4.0	0.7
DIS3L2	2.9	4.6	0.7	1.3	1.2	1.8	1.4	4.3	0.8
ERGIC2	2.0	4.6	0.5	1.9	1.4	1.9	1.2	4.6	0.8
VPS36	2.6	4.0	0.9	2.2	1.1	1.8	1.2	4.5	0.6
KCNH3	3.6	4.2	1.2	1.6	1.6	1.7	1.5	2.7	0.7
CD2AP	3.4	4.5	0.7	1.5	1.2	2.3	1.0	3.5	0.7
SNORD105B	0.4	1.8	3.2	0.3	1.5	0.0	5.9	5.1	0.7
MTX3	3.5	5.6	0.7	1.2	1.2	1.0	1.2	3.7	0.7
KLHDC2	2.3	3.9	0.6	1.6	1.5	1.7	1.4	5.2	0.7
TMEM181	3.0	4.3	0.8	0.9	0.9	0.8	1.5	5.6	0.8
NIN	2.9	3.5	0.8	1.4	1.1	2.2	1.6	4.4	0.9
HID1	2.9	2.9	0.8	3.2	3.2	2.0	1.2	2.1	0.6
ZBED5	2.7	5.1	0.5	1.1	1.0	2.1	1.1	4.3	0.8
LOC101927809	3.4	5.7	1.1	2.3	1.6	1.2	0.9	2.4	0.3
LINC00967	4.6	6.5	1.0	1.3	0.9	1.4	0.4	2.4	0.3
BET1	2.5	4.7	0.1	1.3	0.8	2.0	1.8	4.4	1.2
COIL	2.8	4.3	0.6	1.4	0.9	1.6	1.3	5.1	0.7
ZNF281	3.2	4.0	0.8	1.8	1.5	1.7	1.4	3.6	0.8
KIF18B	3.1	3.5	1.1	4.1	4.1	1.7	0.2	0.7	0.3
TMEM39A	2.8	3.9	0.6	1.0	1.0	1.2	1.8	5.7	0.8
KIAA1683	4.5	5.3	1.3	1.0	0.9	1.0	1.1	3.2	0.5
FLJ45256	3.3	4.9	0.8	1.0	0.8	2.2	1.2	4.0	0.7
RBAK-RBAKDN	3.4	4.6	1.1	1.2	0.7	0.6	1.4	5.4	0.5
BCORL1	3.4	3.3	1.1	1.5	1.4	1.0	1.7	4.4	1.1

USP3	2.8	4.6	0.8	1.1	1.1	1.9	1.2	4.4	1.1
XAF1	3.2	5.6	0.7	0.8	0.6	2.0	0.9	4.4	0.6
EXD3	4.1	5.3	1.2	1.0	0.7	0.8	1.7	3.4	0.6
POLK	3.1	4.5	0.7	1.9	1.2	1.8	1.1	3.9	0.6
LOC101928887	3.2	4.9	1.0	0.9	0.8	1.9	1.3	4.0	0.7
LOC101928893	3.3	4.7	0.8	1.4	0.8	2.2	1.4	3.5	0.6
TMEM126A	2.9	4.3	0.7	2.2	1.4	1.7	1.0	4.0	0.6
EIF2B3	2.8	4.7	0.6	1.5	1.4	1.8	1.6	3.9	0.5
TRAPPC11	3.2	4.7	0.7	1.2	1.0	1.3	1.3	4.6	0.7
PPP1R3C	3.7	5.5	0.8	1.6	0.8	0.8	1.2	3.7	0.8
TNFRSF1B	2.6	3.7	0.6	0.8	0.6	6.2	0.8	3.0	0.4
FRS2	3.3	4.7	0.8	1.9	1.1	1.1	1.4	3.6	0.9
ERAP1	2.2	3.1	0.6	1.7	1.2	1.6	1.7	5.6	0.9
LTN1	3.3	4.6	0.6	1.3	0.9	1.5	1.3	4.5	0.7
PWARSN	3.0	4.8	0.8	1.2	0.7	2.1	1.3	4.2	0.6
PDE6D	2.2	4.1	0.8	2.1	1.7	1.3	1.3	4.1	1.0
MTO1	3.0	4.8	0.8	1.6	1.1	1.5	1.3	4.0	0.7
SIPA1L1	2.6	3.0	0.7	1.5	1.5	1.5	1.9	5.0	0.9
HRSP12	3.4	3.9	0.5	2.1	0.9	2.0	1.5	3.6	0.8
UFL1	2.7	4.2	0.7	1.1	0.9	1.3	1.6	5.4	0.9
FLT4	5.0	5.9	1.9	1.4	1.6	0.9	0.6	1.1	0.3
APOBEC3B	2.6	3.7	0.9	2.2	1.7	1.5	1.5	3.5	1.1
CACNG8	4.1	4.5	1.5	1.4	0.8	0.5	1.6	3.6	0.7
WWC1	3.8	4.2	1.1	3.1	3.0	1.9	0.4	0.8	0.3
C14orf105	3.1	4.8	0.8	1.1	0.9	2.1	1.3	4.2	0.4
TMEM177	3.9	4.2	1.0	1.8	1.5	1.0	1.2	3.4	0.8
APH1B	2.8	5.2	1.2	1.9	1.2	1.2	1.0	3.5	0.8
DYNC2LI1	2.4	6.0	0.5	1.4	0.9	1.3	1.1	4.4	0.6
RNF149	2.2	4.3	0.9	1.7	1.3	2.9	1.5	3.2	0.7
C9orf41	3.8	3.4	0.9	1.8	1.4	1.1	1.9	3.7	0.6
ATG5	2.2	4.1	0.4	1.1	1.2	2.1	1.5	5.1	1.0
MYO6	3.4	4.6	0.9	1.7	1.2	1.3	1.3	3.7	0.6
AP1G2	3.6	4.0	0.8	1.1	1.0	1.9	1.6	3.8	0.8
KIAA1147	2.4	4.2	0.6	1.0	1.0	1.2	2.4	4.9	0.9
KIAA1737	2.8	3.4	0.9	1.3	1.3	1.1	1.9	5.0	1.1
LOC100289561	3.7	4.0	1.0	1.4	0.8	0.5	2.2	4.2	0.8
LOC101928703	3.1	5.5	0.8	0.9	0.7	2.0	1.1	3.8	0.6
DPYD	3.0	6.8	0.7	1.7	1.2	1.5	0.8	2.4	0.5
NOVA2	3.4	3.5	1.4	1.5	1.3	0.5	2.2	3.6	1.3
SMG8	3.1	4.5	0.8	1.8	1.5	1.3	1.3	3.7	0.7
ZNF324B	2.7	3.7	1.2	1.5	1.7	0.8	1.6	4.4	1.0
GCLM	2.4	3.8	0.5	2.4	1.7	2.1	0.9	4.0	0.8
RAB11B-AS1	3.3	3.0	1.0	1.2	1.4	1.2	2.6	3.9	1.1
PFKFB4	3.4	4.4	1.2	1.5	1.1	0.7	1.6	4.3	0.5
ATXN3	3.0	4.8	0.8	1.1	0.7	1.8	1.2	4.6	0.7
TMEM19	2.8	3.8	0.6	1.7	1.4	1.6	1.2	4.9	0.6
PANX2	3.6	3.7	1.2	2.0	2.2	1.1	1.3	2.7	0.9

WDR60	3.4	3.8	0.8	1.8	1.1	1.1	1.8	3.9	0.9
SAMD11	1.8	1.7	0.5	1.9	2.2	1.4	2.9	5.2	1.0
PNRC2	2.8	2.7	0.5	2.3	1.7	3.8	1.4	2.7	0.8
GNPTAB	3.2	4.4	0.8	2.3	1.7	1.9	0.9	2.9	0.5
HECW1	4.0	4.8	1.2	1.2	1.0	0.8	1.3	3.7	0.7
AGK	2.9	4.5	1.0	1.8	1.5	1.3	1.1	3.8	0.5
LRRC8D	3.5	5.5	0.9	1.8	1.4	1.4	0.8	2.8	0.6
APOM	3.7	4.9	1.2	1.1	1.3	0.9	1.4	3.4	0.6
NTM	1.0	1.5	0.3	0.5	0.4	0.2	3.3	8.9	2.4
USP6NL-IT1	3.2	4.5	0.8	0.9	0.9	2.9	1.2	3.7	0.6
AURKB	2.9	3.6	0.7	4.7	3.5	2.2	0.2	0.6	0.1
BRI3BP	2.6	4.1	1.0	1.6	1.7	1.8	1.3	3.7	0.7
SSBP2	2.7	3.3	0.6	0.8	1.0	1.3	2.1	5.5	1.3
FAM76A	2.8	5.4	0.9	1.5	0.9	1.5	1.1	3.5	0.9
LOC101927977	6.2	8.2	1.5	0.6	0.8	0.9	0.2	0.1	0.1
CCBE1	4.3	5.6	1.3	2.0	1.5	1.7	0.5	1.4	0.3
KIAA0753	2.8	4.3	0.7	1.7	1.3	1.5	1.4	4.1	0.8
MUT	2.9	4.5	0.7	1.7	1.0	1.5	1.4	4.2	0.7
ICK	2.6	3.0	0.7	1.6	1.5	1.0	1.9	5.2	1.1
SIGLEC8	3.1	4.5	0.9	1.1	0.8	1.9	1.3	4.3	0.7
PDGFD	3.6	4.8	1.0	0.5	0.5	0.3	1.7	5.2	1.0
GDAP2	2.9	4.1	0.7	1.4	0.8	1.3	1.4	5.3	0.7
LOC100996473	3.0	4.2	1.0	1.5	1.0	1.2	2.1	3.6	1.0
HDAC8	3.0	3.9	0.8	1.7	1.5	1.5	1.5	4.0	0.7
CCDC117	2.5	4.2	0.6	1.5	1.3	0.9	1.7	4.8	1.0
NR4A1	2.3	2.5	0.6	0.7	0.8	9.2	0.9	1.2	0.3
NABP1	3.7	4.8	0.9	2.1	2.0	2.8	0.6	1.3	0.2
ZNF623	2.7	4.4	0.7	1.7	1.0	1.5	1.5	4.3	0.7
LOC101928196	2.4	3.1	0.4	0.6	0.2	8.1	0.8	2.4	0.5
LOC101929591	3.3	4.5	0.8	1.0	0.9	2.3	1.2	3.8	0.6
BST2	2.2	4.4	0.7	0.5	0.9	1.9	2.2	5.2	0.6
CCDC142	3.2	4.2	0.9	1.4	1.2	0.9	1.8	4.1	0.9
CRX	2.9	5.0	1.0	0.9	0.7	1.5	1.4	4.3	0.7
CETN3	2.4	4.1	0.7	1.7	1.1	2.7	1.4	3.9	0.4
BICD1	2.8	3.6	1.0	0.8	0.9	0.6	1.8	5.8	1.0
PIK3CD	2.4	3.0	0.8	1.4	1.2	6.4	0.8	2.0	0.4
TONSL	2.9	3.5	0.8	2.9	2.9	2.2	1.0	1.9	0.4
ERVK13-1	3.2	4.9	0.9	1.1	0.8	0.9	1.4	4.5	0.8
HOXA10	2.8	4.1	0.7	2.1	1.3	0.9	1.7	3.9	1.0
GRAMD1B	3.1	3.8	0.9	2.6	2.1	1.4	1.3	2.9	0.4
BATF3	3.1	3.3	1.1	1.5	1.6	0.8	2.5	3.4	1.0
NCOA2	3.1	3.9	0.9	1.3	1.2	1.7	1.4	4.1	0.8
LOC100129516	3.2	4.6	0.9	1.0	0.9	1.3	1.0	4.7	0.9
IL18BP	2.9	4.6	0.7	1.5	1.5	1.1	1.2	4.5	0.6
PTPN13	2.4	3.8	0.6	1.7	1.3	1.4	1.2	5.4	0.7
PPARA	3.1	4.0	0.9	1.5	1.2	1.0	1.6	4.2	0.8
ZBTB41	2.6	4.3	0.6	1.3	1.0	1.1	1.4	5.4	0.7

PCDHB10	3.3	4.2	0.9	1.2	1.0	0.8	1.7	4.4	0.8
TAF1B	3.0	4.2	0.7	1.6	1.0	1.6	1.4	4.4	0.6
GATS	2.8	4.0	0.8	1.0	0.9	0.6	1.7	5.4	1.2
SPG11	3.3	4.5	0.8	1.3	1.0	1.6	1.1	4.0	0.7
LRRC23	2.3	4.5	1.0	1.5	1.2	0.8	1.9	4.3	0.9
OSGIN2	2.7	4.9	0.9	1.6	1.2	1.2	1.2	4.1	0.5
DNAJC18	3.0	4.6	1.0	1.6	1.2	1.9	0.9	3.7	0.6
ZNF561	1.9	3.8	0.4	1.8	1.3	1.6	1.4	5.4	0.7
TMEM198	2.4	3.1	0.7	0.9	0.8	0.5	2.6	5.9	1.4
STK3	3.4	4.2	0.9	1.8	0.9	1.4	1.1	4.0	0.6
POLR1E	2.9	4.0	0.9	2.1	1.8	2.0	1.0	2.9	0.7
LUCAT1	2.8	3.9	0.8	1.5	0.8	1.9	1.5	4.6	0.6
LOC101928847	3.4	4.8	0.8	1.1	0.9	2.2	1.1	3.4	0.6
CDK14	3.1	3.9	0.7	1.9	1.6	1.5	1.5	3.6	0.5
SLC5A2	2.6	3.4	0.8	1.4	1.1	0.6	2.4	5.2	0.9
HAUS4	3.4	4.6	0.9	2.0	1.6	2.3	1.0	2.2	0.4
LOC100288748	3.5	4.9	0.9	1.2	0.6	1.8	1.1	3.7	0.7
PARD3B	3.6	4.6	1.1	1.6	1.3	1.3	0.9	3.2	0.7
ANGEL1	3.1	3.7	0.8	1.4	1.0	1.1	1.7	4.8	0.9
ZNF236	3.2	3.7	0.9	1.6	1.5	1.4	1.3	3.8	0.8
TMEM9B-AS1	2.7	4.2	0.6	1.6	0.9	1.0	1.9	4.8	0.5
LOC100507462	4.1	4.5	0.9	0.9	0.9	2.0	1.1	3.2	0.6
MAP4K3	2.8	4.9	0.5	1.6	1.5	1.4	1.2	3.6	0.7
CEP55	2.4	2.7	0.6	4.0	3.2	4.3	0.1	0.7	0.1
ZNF273	2.9	4.6	0.9	1.2	0.9	1.7	1.2	4.3	0.5
SYT17	2.8	3.0	0.8	1.7	1.7	1.0	1.6	4.6	0.9
FBXL3	2.4	3.6	0.5	1.6	1.5	1.9	1.4	4.5	0.9
TNRC6C-AS1	3.0	4.7	0.8	1.1	0.9	2.6	1.1	3.6	0.6
TEAD4	3.4	3.7	1.4	2.0	2.2	1.4	1.1	2.3	0.5
ADCK5	3.1	4.8	0.9	1.5	1.6	1.6	1.5	2.4	0.8
TTC38	2.8	3.8	0.9	1.7	1.4	1.5	1.7	3.7	0.7
RHOF	2.1	2.7	0.7	0.5	0.5	5.4	1.5	4.3	0.4
AQR	3.1	4.3	0.7	1.3	1.1	1.5	1.3	4.1	0.7
NBPF10	3.0	4.1	0.9	1.4	1.2	1.1	1.4	4.4	0.8
TVP23C-CDRT4	2.8	3.7	0.9	1.2	0.8	0.6	2.0	5.5	0.7
HTATIP2	2.2	3.7	0.7	2.6	1.7	2.9	1.0	2.9	0.5
MIS18A	2.5	4.2	1.2	2.6	1.6	1.6	1.4	2.8	0.5
AVEN	2.7	3.7	0.9	1.5	1.7	1.4	1.8	3.9	0.7
PTPDC1	3.6	4.8	0.8	1.0	0.8	0.6	1.5	4.2	0.9
HNMT	4.1	5.1	1.1	0.5	0.9	0.4	1.5	3.9	0.8
PPTC7	2.5	3.7	0.8	1.5	1.5	1.9	1.7	3.8	0.7
CNOT4	3.1	3.8	0.7	1.8	1.4	1.4	1.3	4.0	0.8
LMBRD1	2.9	5.1	0.7	1.8	1.1	1.5	0.9	3.6	0.6
POMT2	2.8	3.9	0.6	1.5	1.2	1.1	1.8	4.3	1.0
TCEB3	2.7	3.2	0.7	1.3	1.1	1.6	1.9	4.7	0.9
CRY1	2.7	4.0	0.6	2.3	1.1	1.5	1.4	3.9	0.6
THAP1	3.0	3.9	0.6	1.5	1.2	1.1	1.5	4.4	0.9

WDR75	3.2	5.2	0.8	1.4	1.3	1.3	1.0	3.3	0.7
GLIPR2	1.7	3.7	0.6	1.6	1.0	5.0	1.0	2.6	0.8
DTWD2	3.4	3.7	1.0	1.1	1.4	1.1	1.6	3.7	1.1
ZNF367	2.0	2.7	0.7	4.3	4.1	3.0	0.4	0.9	0.2
GNAZ	3.5	3.9	1.2	2.2	2.3	0.7	0.9	2.5	0.9
CAPRIN2	3.4	5.1	0.8	1.1	0.7	1.4	1.5	3.5	0.8
SERTAD1	2.4	3.2	0.9	0.9	1.0	2.8	1.7	4.4	0.8
ADRA1D	3.5	4.2	1.2	2.0	2.3	0.9	1.2	2.1	0.7
NAA40	2.1	3.3	1.0	2.1	1.5	1.2	1.6	4.6	0.6
TMEM201	3.0	3.6	0.9	1.9	1.6	1.1	1.4	4.0	0.6
TMEM38B	2.5	4.9	0.4	1.7	1.1	2.1	0.9	3.6	0.9
CKAP2	2.7	4.3	0.7	2.4	2.1	1.9	0.8	2.4	0.6
LINC00330	3.1	4.3	0.9	1.2	0.8	2.6	1.1	3.6	0.5
TFCP2	2.7	3.8	0.6	1.1	0.9	1.4	1.4	5.1	0.9
MXD1	2.6	3.1	0.6	1.0	0.4	2.1	1.6	5.5	1.2
PAK1IP1	2.7	4.3	1.0	1.9	1.3	1.4	1.4	3.6	0.5
FICD	2.8	3.2	1.0	1.8	1.6	0.8	1.5	4.5	0.9
LOC100379224	3.1	5.3	0.7	0.9	0.6	0.9	1.2	4.5	0.8
MPHOSPH6	2.4	3.4	0.7	2.1	2.0	2.3	1.7	2.7	0.8
FXN	2.9	3.9	1.1	1.2	1.4	1.7	1.6	3.4	0.8
TERF1	3.0	3.2	0.9	1.8	1.5	1.0	1.9	3.6	1.0
AIM1	2.6	4.0	0.8	1.2	1.2	1.7	1.4	4.4	0.8
DNASE1	3.5	3.9	1.1	1.6	1.5	1.0	1.3	3.4	0.7
LOC646762	3.1	4.4	0.9	1.6	1.2	1.3	1.2	3.7	0.7
RAB3IL1	3.4	4.1	1.3	1.7	1.8	1.1	1.1	2.8	0.7
LHFP	3.6	5.8	1.1	1.9	1.6	1.0	0.9	1.8	0.3
IL17RA	2.5	3.0	0.7	1.2	1.3	3.1	1.7	3.8	0.7
BAHCC1	3.1	3.1	1.0	1.7	1.6	1.0	1.8	3.8	0.9
INPP5B	2.9	4.8	1.0	1.4	1.1	1.3	1.6	3.4	0.5
SNORD60	2.3	2.4	3.6	0.6	2.0	0.7	2.5	3.0	0.8
ARL15	3.7	5.0	0.8	0.7	0.6	0.7	1.3	3.9	1.3
[truncated: 1,202,822 more chars]
